# Uncovering the transcriptional landscape of *Fomes fomentarius* during fungal-based material production through gene co-expression network analysis

**DOI:** 10.1186/s40694-024-00192-3

**Published:** 2025-02-13

**Authors:** Timothy Cairns, Carsten Freidank-Pohl, Anna Sofia Birke, Carmen Regner, Sascha Jung, Vera Meyer

**Affiliations:** Chair of Applied and Molecular Microbiology, Institute of Biotechnology, Technische Universität Berlin, Berlin, Germany

**Keywords:** Biomaterial, Fungal-based composite, *Fomes fomentarius*, Co-expression, Network, Clusters, Cell wall, CAZyme, Lignin, White-rot

## Abstract

**Background:**

Fungal-based composites have emerged as renewable, high-performance biomaterials that are produced on lignocellulosic residual streams from forestry and agriculture. Production at an industrial scale promises to revolutionize the world humans inhabit by generating sustainable, low emission, non-toxic and biodegradable construction, packaging, textile, and other materials. The polypore *Fomes fomentarius* is one of the basidiomycete species used for biomaterial production, yet nothing is known about the transcriptional basis of substrate decomposition, nutrient uptake, or fungal growth during composite formation. Co-expression network analysis based on RNA-Seq profiling has enabled remarkable insights into a range of fungi, and we thus aimed to develop such resources for *F. fomentarius*.

**Results:**

We analysed gene expression from a wide range of laboratory cultures (*n* = 9) or biomaterial formation (*n* = 18) to determine the transcriptional landscape of *F. fomentarius* during substrate decomposition and to identify genes important for (i) the enzymatic degradation of lignocellulose and other plant-based substrates, (ii) the uptake of their carbon monomers, and (iii) genes guiding mycelium formation through hyphal growth and cell wall biosynthesis. Simple scripts for co-expression network construction were generated and tested, and harnessed to identify a fungal-specific transcription factor named CacA strongly co-expressed with multiple chitin and glucan biosynthetic genes or Rho GTPase encoding genes, suggesting this protein is a high-priority target for engineering adhesion and branching during composite growth. We then updated carbohydrate activated enzymes (CAZymes) encoding gene annotation, used phylogenetics to assign putative uptake systems, and applied network analysis to predict repressing/activating transcription factors for lignocellulose degradation. Finally, we identified entirely new types of co-expressed contiguous clusters not previously described in fungi, including genes predicted to encode CAZymes, hydrophobins, kinases, lipases, F-box domains, chitin synthases, amongst others.

**Conclusion:**

The systems biology data generated in this study will enable us to understand the genetic basis of *F. fomentarius* biomaterial formation in unprecedented detail. We provided proof-of-principle for accurate network-derived predictions of gene function in *F. fomentarius* and generated the necessary data and scripts for analysis by any end user. Entirely new classes of contiguous co-expressed gene clusters were discovered, and multiple transcription factor encoding genes which are high-priority targets for genetic engineering were identified.

**Supplementary Information:**

The online version contains supplementary material available at 10.1186/s40694-024-00192-3.

## Background

Fungi are used as cell factories for the bulk manufacture of proteins, enzymes, acids, and platform chemicals [1, 2]. Many molecules produced by fungi (e.g., citric acid, penicillin, pectinases) are valued at over a billion dollars annually [3]. Recent estimates of the full contribution of fungi to the global economy (therapeutics, food/beverages, colorants/pigments, cosmetics, crop protection, biofuels, bioremediation, carbon sinks and other applications) value fungi at over 54 trillion dollars [4]. Such diverse utilities of the fungal kingdom have been extensively used by large biotech companies and small/medium enterprises for many decades [5]. Thus, fungi are a major source of novel patents, innovative applied science, and technological breakthroughs [5–8]. This productivity is exemplified by the multipurpose cell factory *Aspergillus niger*, which has been used for industrial production of organic acids (1920s), enzymes (1960s), and secondary metabolites (2010s [9]).

A new frontier in sustainable biotechnology is the bulk generation of high-performance fungal biomaterials (reviewed in [10–14]). These consist exclusively of fungal biomass, or, alternatively, are composites of a fungus with a growth substrate (normally plant waste [15] or less commonly plant-plastic mix [16]). Applications include clothing, packaging, and construction materials for use in the fashion, textile, automotive, electrical and other industries [6, 13]. Recent quantitative comparisons between fungal-plant composites and conventional concrete demonstrate biomaterials perform favourably with regards to water usage, air pollution, and the emission of greenhouse gases [17]. Moreover, fungal biomaterials effectively sequester carbon, which is in contrast to significant emissions from extracting and producing conventional construction materials [18]. Thus, fungal biomaterials have recently emerged as an important yet underexploited driver for the transition from a petroleum to bio-based civilisation [1].

**Fig. 1:**
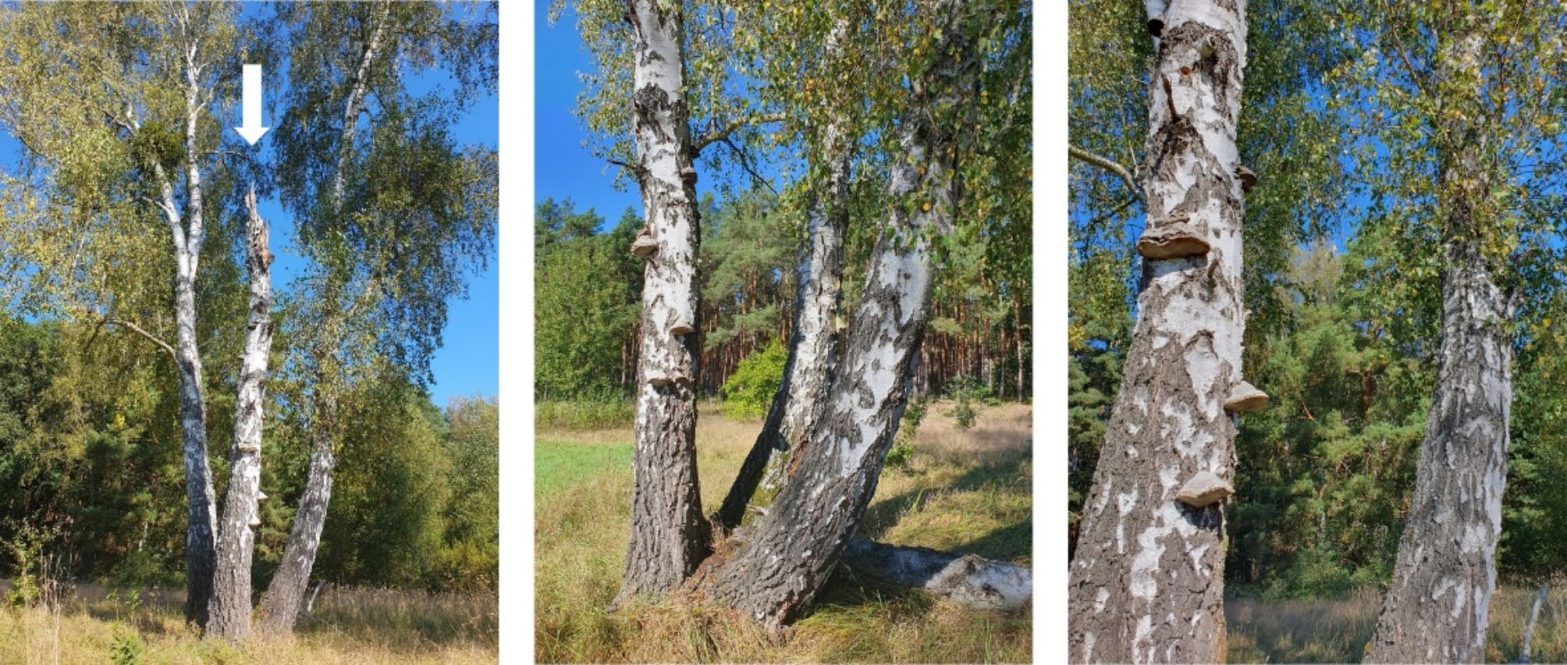
*F. fomentarius* fruiting bodies are observed on dead but not healthy tree trunks. A birch consisting of three tree trunks, one of which is dead (marked with an arrow). Only the dead one bears perennial fruiting bodies of *F. fomentarius*. Picture taken in Brandenburg, Germany, September 2024, © Vera Meyer

Approximately 30 filamentous species have been named in biomaterial patent applications, which include either ascomycetes or basidiomycetes [6]. Fascinatingly, the first documented use of fungi as a biomaterial came from archaeological analysis of the European ‘Iceman’, who was carrying dried biomass from the basidiomycete *Fomes fomentarius* for use as kindling when he died over 5000 years ago [19]. Considerably more recently, our group has conducted a catalogue of potential biomaterial-forming fungi in the woodlands surrounding Berlin, which shortlisted *F. fomentarius* as a leading candidate for further study due to the notable mechanical strength, light weight, and hydrophobic perennial fruiting bodies [20]. *F. fomentarius* decomposes a wide variety of physically damaged or dead trees, including hardwoods (e.g., birches, beeches) and softwoods (e.g., conifers). Little is known about the molecular or cellular basis of tree decomposition, yet hyphae are assumed to penetrate damaged bark, ultimately producing large perennial fruiting bodies (Fig. 1) [21]. Importantly, fungal culture from healthy European beech immediately after felling revealed no *F. fomentarius*, but extensive colonisation 24 weeks later [22]. These data demonstrate that *F. fomentarius* primarily exists as an endophyte or mutualist, which begins plant decomposition following increase in oxygen/nutrients following physical damage of the tree [22, 23]. Indeed, we have documented instances where *F. fomentarius* fruiting bodies form on a dead birch, yet are absent from healthy trees formed from the same root (Fig. 1).

Previously, we have generated various proof-of-principle studies regarding *F. fomentarius* biomaterials, including a three-stage solid-state fermentation process at lab scale [15], and achieved composites with promising physical properties that perform comparably or better than conventional materials (e.g., compression strength, thermal conductivity, flammability [24]). Additionally, we have conducted 3D printing of biomaterials using a fungal, alginate, and water paste [25]. Thus, *F. fomentarius* has been confirmed as a suitable species to transform plant biomass into renewable, high performance composite materials.

In order to increase understanding of complex fungal systems, mycologists studying medical, plant infecting, industrial, and model species have developed sophisticated genomic, transcriptomic, proteomic, and metabolomic tools/datasets [26–28]. Such systems-level approaches have enabled the understanding of fungal biology in unprecedented detail, with a strong focus on predicting (rather than simply describing) protein functions, phenotypes, and metabolic capabilities. The need to develop these methods/datasets for biomaterials is urgent. The *F. fomentarius* genome is publicly available [29] and comparative transcriptional analyses during angiosperm and gymnosperm deadwood decomposition with a focus on substrate specific carbohydrate-activated enzymes (CAZYmes) have recently been published [30], which confirmed expression of substrate specific CAZymes.

As transcriptional co-expression networks have not yet been generated for any biomaterial-forming fungus we set out to address this shortcoming using *F. fomentarius*. In general, correlation coefficients (e.g., Spearman) are used in this approach to define genes with similar expression patterns from a respective dataset, thus generating a network [31–33]. These data can be used to predict the biological process or pathway in which a gene and encoded protein are active, for example by defining enriched gene ontology (GO) terms of a given network [31–33]. In *A. niger*, a well-established fungal cell factory with 14,165 predicted genes, we have generated a co-expression network which enabled us to update functional predictions for ~ 9500 genes (> 65% of the genome [31]). This has led to accurate functional predictions for genes/proteins involved in regulating secondary metabolism [31, 33], morphology [34, 35], and organic acid production [36]. In the human-infecting yeast *Candida albicans*, co-expression networks were used to ‘deORFanize’ the genome, thus enabling the identification of a novel cell cycle regulator [32] and sophisticated functional predictions of a novel drug target [37]. More recently, a co-expression network for clinically important *Cryptococcus neoformans* enabled the prediction of 13 new DNA damage response genes with 100% accuracy [38], thus highlighting the remarkable predictive power of co-expression network approaches. Such accurate functional predictions are especially important for fungi as the experimental time to generate and confirm mutants are drastically increased when compared to model organisms.

The aim of this study was thus to generate datasets and tools for co-expression network construction for *F. fomentarius*. In order to enable comparisons of gene expression between laboratory and endophytic/decomposition growth, we captured transcriptional data from various solid state and submerged cultures and a wide range of plant substrates during composite production. Network analysis ultimately enabled us to predict transcription factors controlling cell wall synthesis and lignocellulose modification during biomaterial formation. Remarkably, we demonstrate that predicted secondary metabolite gene clusters and putative effector proteins suggest that *F. fomentarius* is not a pathogen. We furthermore show here clear co-expression of novel classes of contiguous gene clusters that have not been described in fungi. Taken together, this study gives a detailed analysis of the *F. fomentarius* transcriptional landscape, identifies new types of co-regulated gene clusters, and delivers the necessary dataset and tools for rapid construction of user desired co-expression networks.

## Results

### Genomic analysis predicts *F. fomentarius* is a mycotoxin-free decomposer

Most general and fungal specific sequence analysis software or databases have recently undergone exciting advances and updates [39–42]. We therefore reanalysed the predicted 14,757 genes of *F. fomentarius* [29] using publicly available software to both profile its natural product repertoire and glean important clues as to the endophytic/saprophytic niche of this fungus.

Firstly, AntiSMASH was used to identify secondary metabolite biosynthetic gene clusters and predict cognate products, which resulted in 530 genes residing in 39 clusters (Additional file 1 [41]). Clusters ranged in gene number from 4 to 82, with a median occupancy of 11 genes. A total of 56 core biosynthetic enzymes were predicted to be encoded, with putative products including terpenes (*n* = 28), polyketides (*n* = 4), non-ribosomal peptides (*n* = 14) and ribosomally synthesized and post-translationally modified peptides (RiPPs, *n* = 10 [43]). Importantly, cluster BLASTs against the recently updated Minimum Information about a Biosynthetic Gene cluster (MIBiG) database [42] did not reveal similarity to any major mycotoxin cluster (e.g., aflatoxin, ochratoxin, dothistromin, preterpestacin, etc.) or virulence factor for plant infection (botcinic acid, AM-toxin, fumonisin, etc., Additional File 1 [44]). This analysis did predict various clusters which possibly synthesise basidoferrin (which may synthesise a siderophore), antifungal α-cadinol, beta-lactamase inhibitor clavulanic acid, and antifungal/antibacterial armillyl orsellinate. These data for the first time genetically exclude the possibility that *F. fomentarius* composite materials could contain any so far known mycotoxin, which is consistent with the safe use of this fungus throughout history [45]. Additionally, the genetic absence of bona fide secondary metabolite virulence factors further support the hypothesis that *F. fomentarius* is not a plant pathogen [22, 23].

Indeed, with regards to producing biomaterials at scale, these composite products could conceivably be more challenging to market if derived from a plant pathogen as opposed to a general decomposer [46]. Interestingly, recent machine learning models of secreted effectors have been able to robustly distinguish apoplastic (space between plant cells) or cytoplasmic targeting [40]. In saprobes/mutualists, apoplastic effectors are common, yet cytoplasmic targeted proteins are significantly less when compared to most pathogens [40]. In order to identify *F. fomentarius* secreted and putative effector proteins, amino acid sequences were searched for signal peptide (*n* = 1114) and putative effector functions (*n* = 282), respectively [39, 40]. We refined these data by also screening for glycosylphosphatidylinositol (GPI) anchors (*n* = 297, predicted effectors with anchor = 35, Additional File 2 [47]). We did not remove effectors with GPI anchors from the analysis due recent identification of membrane attached effector complexes in fungi [48]. 232 and 50 *F. fomentarius* effectors were predicted to localize to the plant apoplast or cytoplasm, respectively (representing 20.8% and 4.5% of the predicted secretome) which is comparable to many other saprophytic but not generally pathogenic fungi [40]. Taken together these genomic analyses predict that *F. fomentarius* is a mycotoxin-free, non-pathogenic fungus thriving in healthy hardwoods, such as birches and beeches.

### Generating a diverse gene expression dataset from biomaterial, in planta and in vitro *F. fomentarius* growth

**Fig. 2:**
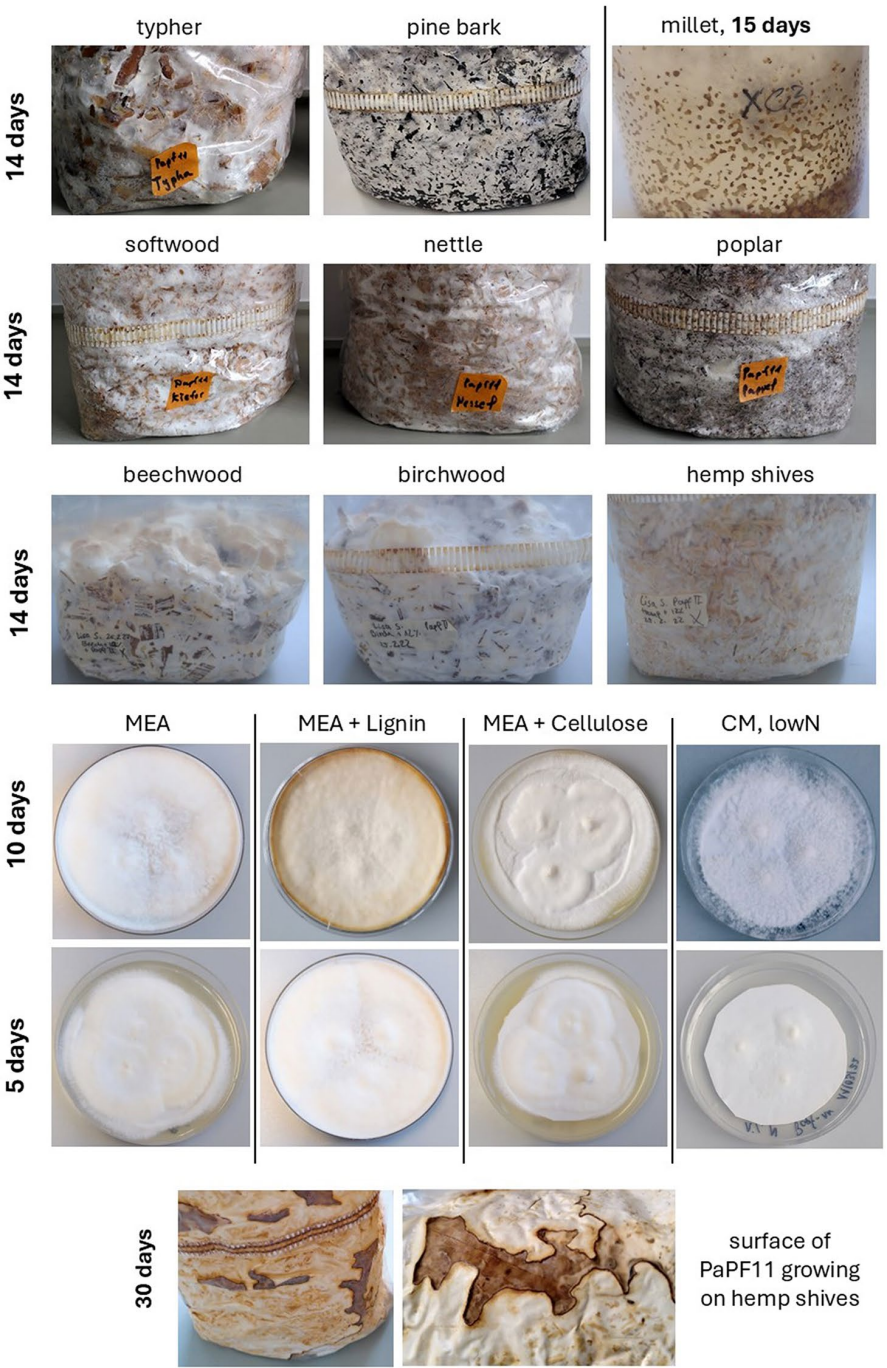
Composite and standard agar cultivations of *F. fomentarius* used in this study. Compact (5 days) and fluffy (10 days) colony phenotypes were observed. A lack of pigmentation was consistent across all cultivations with the exception of 30-day cultivation on hemp shives, where brownish fungal growth was observed. Note, liquid cultures are not depicted

**Fig. 3:**
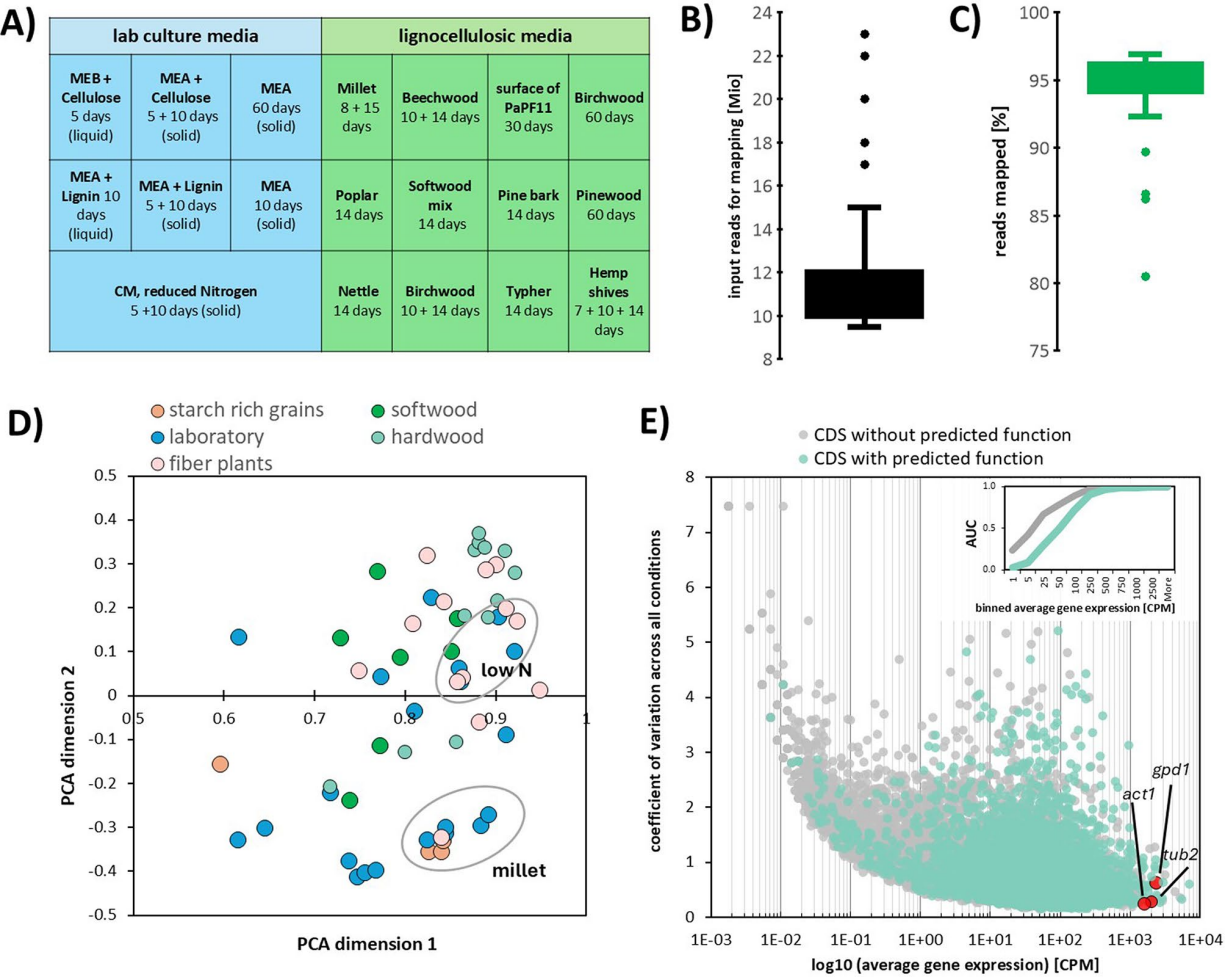
Cultivation experiments and RNA-Seq analysis output of this study. (**A**) Overview of *F. fomentarius* biomass samples taken in this study. Conditions are grouped by laboratory culture media (emerse or submerged growth) and plant-based, lignocellulosic media used for biomaterial production. (**B**) Box-Whisker-Plot of amount of input reads used for mapping, average = 11.8 Mio 150 bp PE reads. (**C**) Box-Whisker-Plot of percentage of input reads successfully mapped to the *F. fomentarius* genome, average = 94.6%. (**D**) Principal component analysis (PCA) of CPM-normalized gene expression values for all samples analysed in this study. Samples are grouped and colour-coded into 5 categories: starch rich grains, softwood, hardwood, fibre plants and laboratory for simplicity. Per sample data for the PCA are included in Additional File 3. (**E**) Scatter plot of coefficient of variation over the average of expression for each gene under all 27 conditions. For clarity, coding sequences (CDS) with a hypothetical protein are labelled differently. Inset graphs shows the different distribution of average expression for CDS encoding identified or hypothetical proteins, respectively

The quality of co-expression networks and accuracy of subsequent gene functional predictions require transcriptional signatures across a diverse range of conditions [31, 49, 50]. For this reason, and in order to reflect the extensive capabilities of *F. fomentarius* to form composites from a variety of plant substrates, we isolated fungal RNA from hemp, beech, birch, nettle, typha, poplar, millet, and pine from a range of composite and in planta assays, including bags, jars and bark growth (Fig. 2). In order to enable the delineation of genes expressed during composite vs. standard laboratory growth, we also included maltose or glucose agar/liquid culture with a variety of macronutrient compositions (Fig. 3A).

With regards to the number of RNA samples required to generate high-quality co-expression networks, this study included a total of 27 cultivation conditions (24 biological duplicates generated in this study and 3 retrieved from [30]). This is above the recommended minimal threshold (*n* = 20) published in 2015 [49]. It should be noted that we recently calculated that the advised minimum sample number is 32 [50]. However, this limitation can be compensated for by using stringent thresholds for defining co-expressed gene pairs (i.e. high Spearman correlation coefficients [50]) and applying multiple hypotheses corrected p-value filtering (see Materials and Methods). We also reasoned that average values from biological duplicates are important for alternative ways to probe the dataset (e.g., differential gene expression values between conditions) and thus rejected the strategy of non-replicated (yet more diverse) RNA sampling.

Raw counts were DESeq2 processed to give normalized values of gene expression across the 27 conditions (Fig. 3A-C, Additional File 2). Principal component analysis (Fig. 3D) showed no clear differentiation between a priori and arbitrary grouping but identified the cultivation on culture medium with reduced nitrogen availability to cluster more with plant-based media. Likewise, starch-rich millet grains do seem to elicit a distinct overall transcriptional response as they do form an outgroup from all lignocellulosic conditions. In order to estimate the number of genes transcribed in the dataset, we calculated their expression values relative to the mean of three highly expressed genes (actin 1265750 (*act1/actA*), beta tubulin 1188384 (*tub2/tubB*), and glyceradehyde-3-phosphate dehydrogenase 1372934 (*gpd1/gpdA*). This revealed 539 genes were highly expressed (> 50%), 6130 were moderately expressed (> 5%) and 10,233 were detectably expressed (> 1%) in at least one of the 27 conditions (Additional File 2). Thus, about 69.3% of the predicted 14,757 *F. fomentarius* genes are transcribed in the 27 cultivation experiments analysed in this study. To our knowledge, these data are the most extensive gene expression profiling of any biomaterial-forming fungus to date.

### Co-expression networks enable predictions of *F. fomentarius* gene and protein functions

**Fig. 4:**
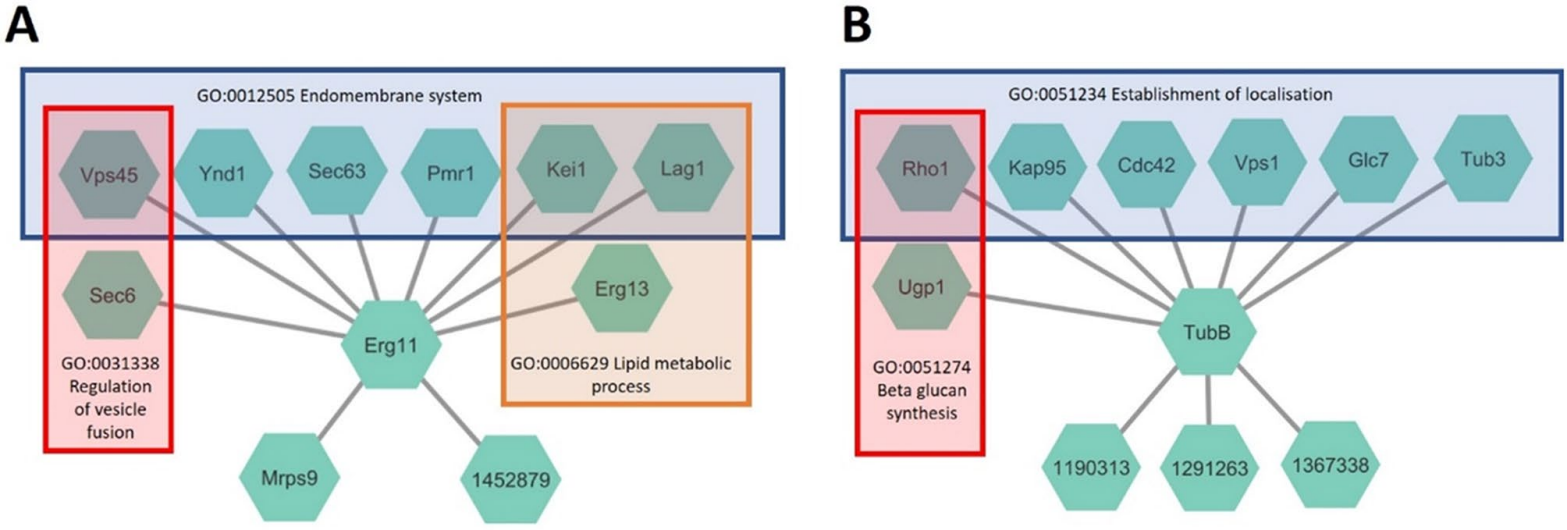
Co-expression modules for *erg11* (**A**) and *tubB* (**B**) are consistent with the function of well-conserved proteins in fungi and additionally assign new functional predictions to several *F. fomentarius* genes. Top ten Spearman correlations (> 0.86, FDR p value < 0.000001) are depicted for *F. fomentarius* *erg11* or *tubB* modules. Genes names follow *S. cerevisiae* nomenclature and were identified by amino acid BLASTs. *F. fomentarius* protein IDs are given where no yeast orthologue was found. Enriched GO terms (FDR *p* < 0.05) are highlighted for various genes in the module. Note, Eg11 or TubB belong to each indicated enriched GO term

In order to enable facile generation and interrogation of *F. fomentarius* co-expression data, we generated simple R scripts for calculating Spearman correlation coefficients between user defined gene pairs (Additional File 4). We then built modules for positive control genes, i.e., where the function of the encoded protein is comprehensively understood in unicellular and filamentous fungi, e.g., lanosterol demethylase Erg11 (*F. fomentarius* protein ID 1348887) important for ergosterol and membrane lipid biosynthesis and β-tubulin TubB (*F. fomentarius* protein ID 1188384), important for unicellular and hyphal growth. Pairwise co-expression relationships between *erg11/tubB* and all other *F. fomentarius* genes were generated (Additional Files 5 and 6). The top ten most highly co-expressed genes were visualized as connections (edges) with either *erg11* or *tubB*. Gene names in each module were assigned based on amino acid BLASTs against the so far best-annotated fungal genome, the unicellular yeast *Saccharomyces cerevisiae*. As demonstrated in Fig. 4, the modules predicted for *F. fomentarius* were enriched with genes that were obviously consistent with the function of Erg11 (ergosterol synthesis Erg13, endomembrane proteins Ynd1, Sec63, Lag1, Pmr1, Kei1) or β-tubulin (α-tubulin Tub3, cytoskeletal organizing proteins Rho1, Cdc42, Glc7, Vps1, Fig. 4) in fungi. Additionally, several *F. fomentarius* genes lacking orthologues in *S. cerevisiae* were identified, which suggests for example that gene 1452879 (predicted cyclopropane-fatty-acyl-phospholipid synthase) or gene 1190313 (predicted GGL domain containing protein) likely function in ergosterol synthesis or cytoskeletal processes, respectively (Fig. 4). We have previously used similar control analyses to confirm networks are sufficiently interconnected for robust predictions of gene/protein function in the filamentous fungus *A. niger* [31], and thus conclude the data and scripts generated in this study can be harnessed to considerably improve *F. fomentarius* genome annotation.

### Co-expression network mining couples cell wall-associated genes with an uncharacterized fungal-specific transcription factor

Hyphal shape, growth, and adhesion to plant or any other solid surfaces is largely dependent on the fungal cell wall. The coordinated biosynthesis of this dynamic structure is orchestrated at the transcriptional level, yet nothing is known about putative transcription factors that might control this process in *F. fomentarius*. We thus analysed transcriptional profiles for a selected set of 13 highly conserved genes in yeast and filamentous fungi known to be fundamental for cell wall biosynthesis (note, we use filamentous fungal nomenclature for gene names). These genes included one predicted α-1,3-glucan synthase *agsA* (1363000), two β-1,3*-*glucan synthases *fksA*/*fksB* (1313161, 1370413), all nine putative *F. fomentarius* chitin synthases (1364985, 1367937, 1258680, 1397629, 1316987, 1374350, 1303258, 1370108, and 682709) and the major Rho family GTPase *rhoA* and essential regulator ensuring polar growth and cell wall integrity (1396228 [51], Table 1). With the notable exception of putative chitin synthase gene 1258680, expression was high for all genes across the dataset. Conditions which reduced (malt extract plus cellulose, day 10) and increased expression (complete medium with reduced nitrogen day 10, pine park) were observed (Table 1), demonstrating culture-specific transcription of cell wall biosynthetic genes.

**Table 1:**
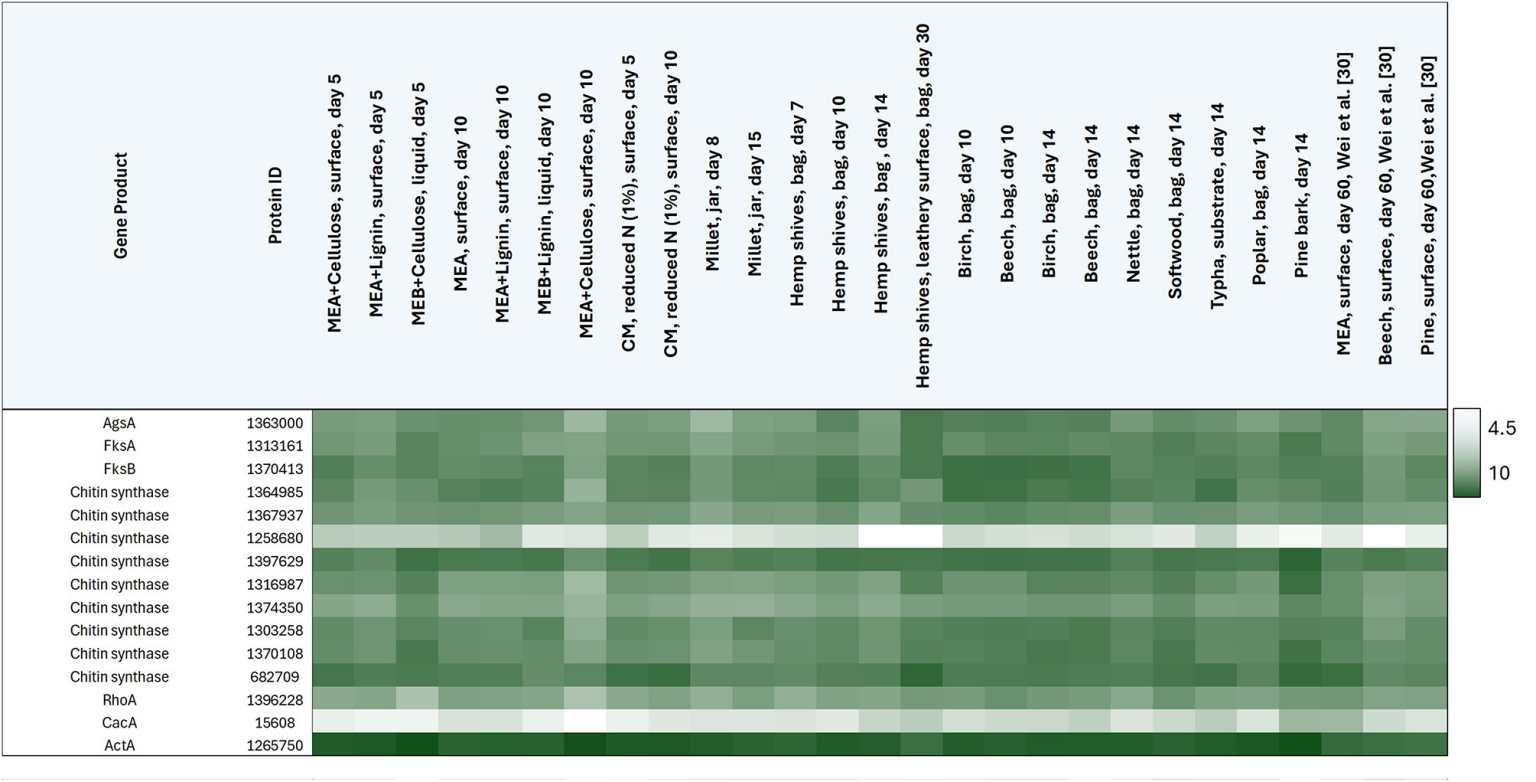
Heat map depicting normalized transcriptional values of genes predicted to encode *F. fomentarius* cell wall biosynthetic genes. Actin (ActA) is given as a reference. Values for predicted transcription factor CacA are given. The heat map shows expression levels as log10 values of DeSeq normalised raw counts with white = 4.5, dark green = 10

**Fig. 5:**
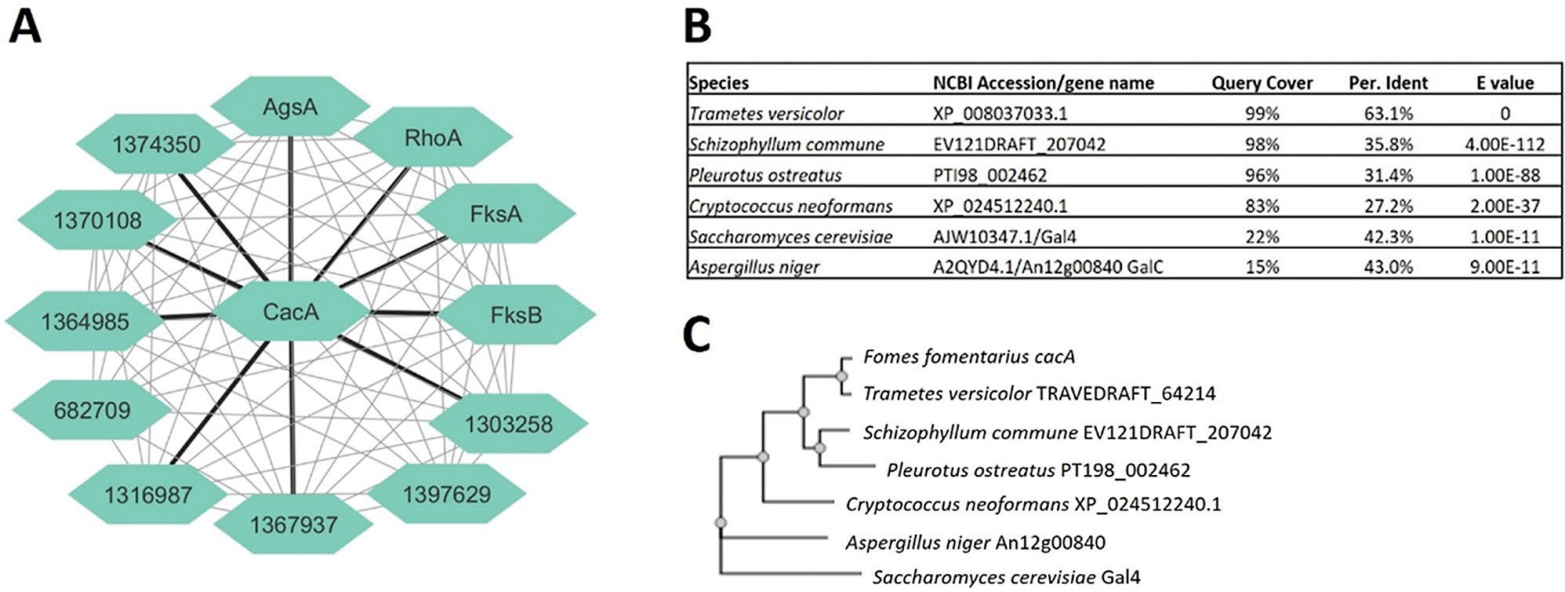
Putative fungal-specific transcription factor encoding gene *cacA* is embedded in a *F. fomentarius* cell wall co-expression network and is conserved in biomaterial-forming fungi. (**A**) Schematic diagram depicting co-expression pairs (Spearman > 0.4, FDR < 0.05) represented in light grey, with edges connecting to *cacA* highlighted in black. Only chitin synthase encoding genes 682709 and 1397629 are not co-expressed with *cacA*. Note remarkable frequency of interconnections to all genes in the network. (**B**) Putative *cacA* orthologues from NCBI protein BLASTS in selected fungi. (**C**) Simple phylogenetic reconstruction of *cacA* and predicted orthologues generated using [52]

Next, we built a cell wall co-expression network by generating pairwise Spearman correlation coefficients between all 13 genes. This network was remarkably interconnected (Fig. 5), with 63 from a possible 78 gene pair comparisons positively co-expressed (Spearman > 0.4, FDR *p* < 0.05). Only a single gene was not expressed with any other candidate (predicted chitin synthase encoding gene 1258680), which was likely due to its very low expression in the dataset (Table 1). The robust co-expression relationships between cell wall-associated genes are further evidence that our dataset will enable accurate predictions of gene function for *F. fomentarius* [31].

Next, from a list of 280 predicted *F. fomentarius* regulatory genes (Additional File 7), we identified a candidate which was highly embedded. This revealed a predicted fungal-specific Zn(2)-Cys(6) transcription factor, 15608, that was connected with all genes in the network except two predicted chitin synthase encoding genes (Fig. 5). Thus, we hypothesize *F. fomentarius* 15608 is a high priority candidate for manipulating cell wall synthesis during biomaterial formation. Indeed, we have previously used such genome mining network approaches to predict in silico global and pathway-specific transcription factors controlling secondary metabolism in *A. niger*, which were ultimately proven through wet-lab experiments [31, 33]. We thus renamed gene 15608 *c*o-expression *a*ssociated *c*ell wall transcription factor A (*cacA*). Interestingly, *cacA* was well conserved in biomaterial forming fungi (e.g., *Schizophyllum commune*, *Trametes versicolor*, *Pleurotus ostreatus*), moderately conserved in other basidiomycete (*C. neoformans*) and poorly in model fungi (*S. cerevisiae* or *A. niger*) (Fig. 5B and C). Thus, *cacA* might be a genetic target to control hyphal branching and surface adhesion in biomaterials formed by other fungal species. These data highlight how novel predictions of gene function can be gleaned from co-expression datasets.

### Profiling CAZyme and sugar transporter expression on culture media and plant-based substrates

Engineering substrate utilisation is an important step to optimise, scale and industrialise *F. fomentarius*-based biomaterials production. White-rot fungi are known to degrade lignin, cellulose and hemicellulose (for example reviewed in [53]). However, there are differences in the mode of degradation – simultaneous or sequential – depending on the fungus and lignocellulosic substrate it is growing on [54]. Further, genomic analyses of lignocellulolytic enzymes in white- and brown-rot fungi have put this strict distinction between white-rot and brown-rot fungi into question [55]. This highlights the necessity of specifically studying *F. fomentarius* lignocellulose decomposition, especially in the context of composite production.

While *F. fomentarius* has been reported to produce cellulolytic enzymes [56, 57], it is currently unknown which proteins (i) are mainly responsible for decomposing lignocellulosic material, (ii) subsequently transport liberated monomers into the fungus, and (iii) regulate these processes (e.g., transcription factors). We used the Conserved Unique Peptide Patterns (CUPP) online platform to predict CAZyme-encoding genes, which revealed 504 candidates among which were 104 putative lignocellulosic enzymes, namely laccases/(multi)-copper oxidases/phenoloxidases (*n* = 24), manganese peroxidases/versatile peroxidases (*n* = 16), cellulases (*n* = 3), xylanases (*n* = 2), neuraminidases (*n* = 1), glucosidases (*n* = 8), trehalases (*n* = 1), glucoamylases (*n* = 1), mannosidases (*n* = 2), pectinases (*n* = 13), glucanases (*n* = 31), and carbohydrate esterases (*n* = 2) (Table 2 [58, 59]). CAZyme-encoding genes were well represented in the transcriptional dataset according to the thresholds defined previously. Both lignin-modifying and hydrolytic enzymes were co-expressed in our data set, suggesting simultaneous degradation of lignin, cellulose and hemicellulose at the point of sampling. However, since end point samples were analysed in this study, further studies are required to be able to draw definite conclusions regarding the mode of degradation – simultaneous or sequential - of lignin, cellulose and hemicellulose by *F. fomentarius*.

Amongst the lignin-modifying enzymes (Table 2A), the gene encoding copper radical oxidase 1316053 reached the highest expression levels out of all lignocellulolytic CAZymes. Notably, these were measured in samples taken from hemp (43043), birch (77579) and beech (63702) substrates. Growth of *F. fomentarius* on hemp shives in general appeared to have a positive effect on expression of lignin-modifying enzymes, especially on genes encoding oxidases and peroxidases. This trend was not observed for hydrolytic enzymes (Table 2B), which points to different induction mechanisms for lignin-modification than for cellulose breakdown in *F. fomentarius*. The highest expression levels for any hydrolytic enzyme-encoding gene were observed for the gene encoding concanavalin A-like lectin/glucanase 1204939 on birch (44801) and beech (45452) substrate.

Interestingly, some of the genes coding for functionally highly similar proteins, e.g., MnP or pectin lyase-encoding genes, are not expressed under the tested conditions while others are constitutively expressed. This could potentially point to functional and/or transcriptional divergence having occurred following gene family expansion events to enhance niche specificity and metabolic robustness. These data will serve as the basis for further studies aiming to decipher the complex lignocellulolytic system in *F. fomentarius*.

**Table 2:**
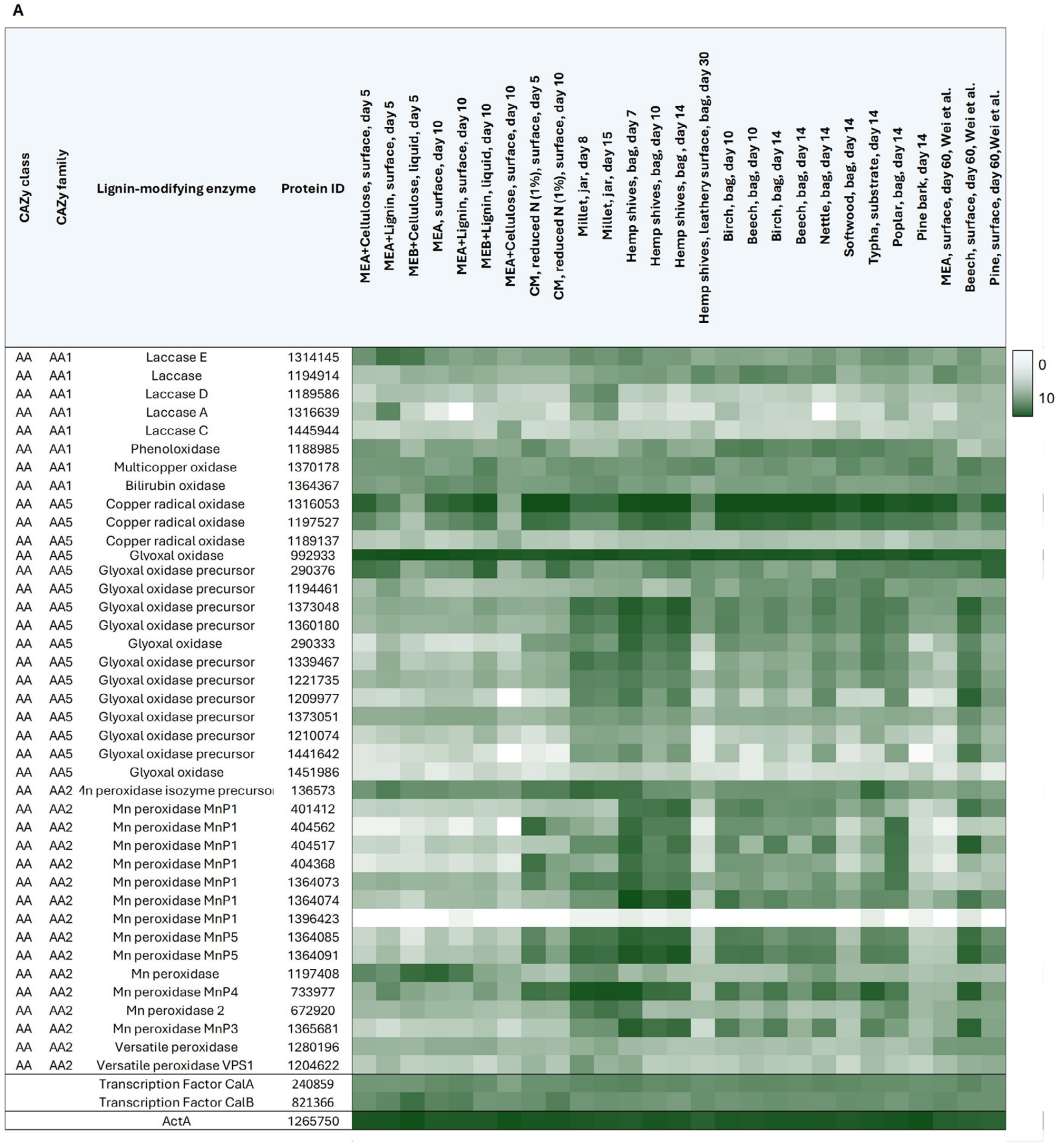

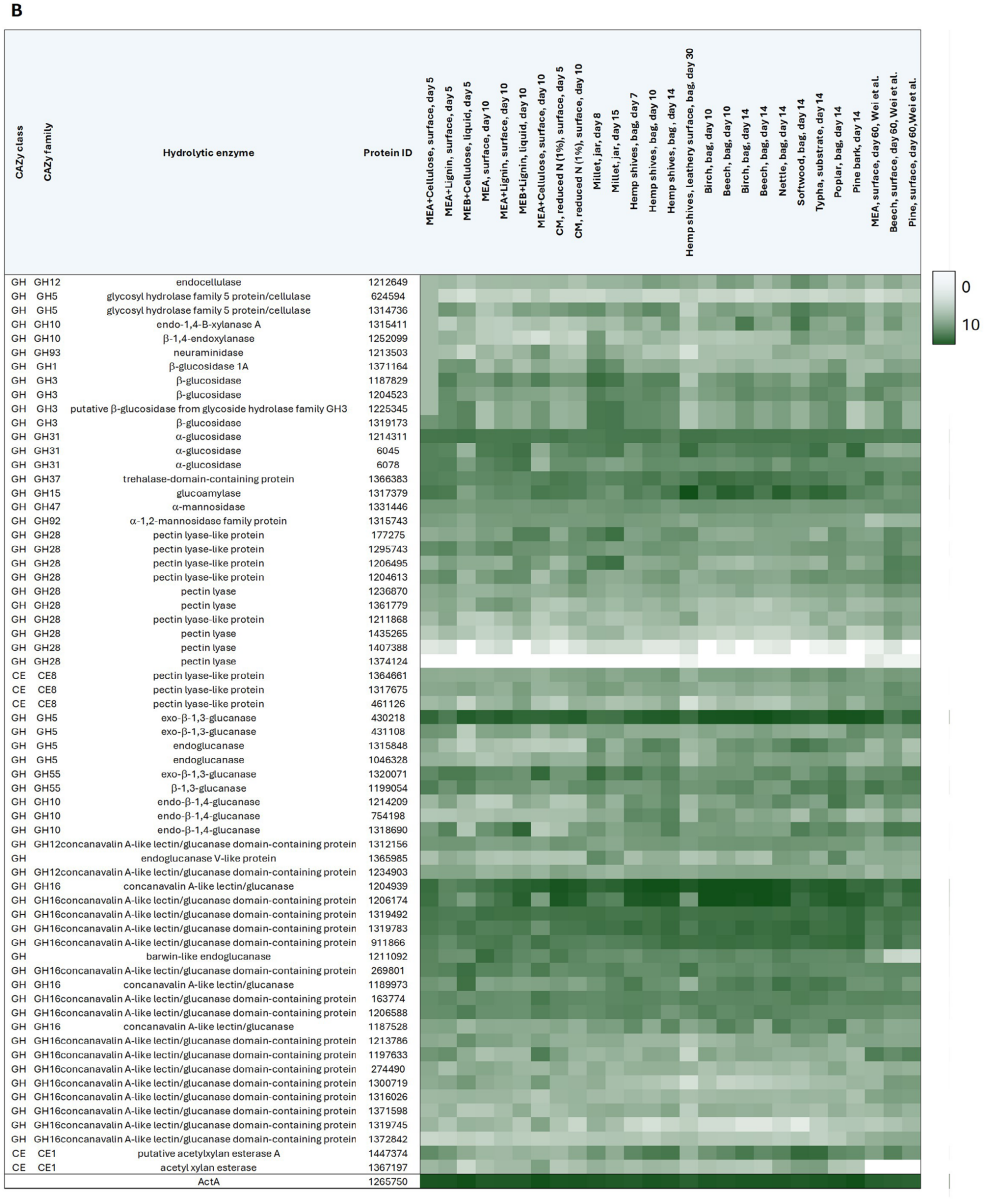
Overview of 104 putative lignin-modifying and hydrolytic lignocellulosic CAZymes and their gene expression profiles under 27 different cultivation conditions. The heat map visualises the transcriptional profiles of selected CAZyme-encoding genes involved in degradation of plant substrates. CAZY classes and families are shown in the first and second column, respectively (AA: Auxiliary Activities, GH: Glycosyl Hydrolase; CE: Carbohydrate Esterase). Predicted protein function and JGI IDs are listed in columns three and four, respectively. The heat map shows expression levels as log10 values of DESeq2 normalised raw counts with white = 0 and dark green = 10. (**A**) Gene expression heat map of 40 putative lignin-modifying enzymes. Expression levels of the newly identified transcription factors, *calA* and *calB*, as well as of the house keeping gene actin as a reference are depicted in the last three rows. (**B**) Gene expression heat map of 64 putative hydrolytic lignocellulosic enzymes and actin (bottom row)

**Fig. 6:**
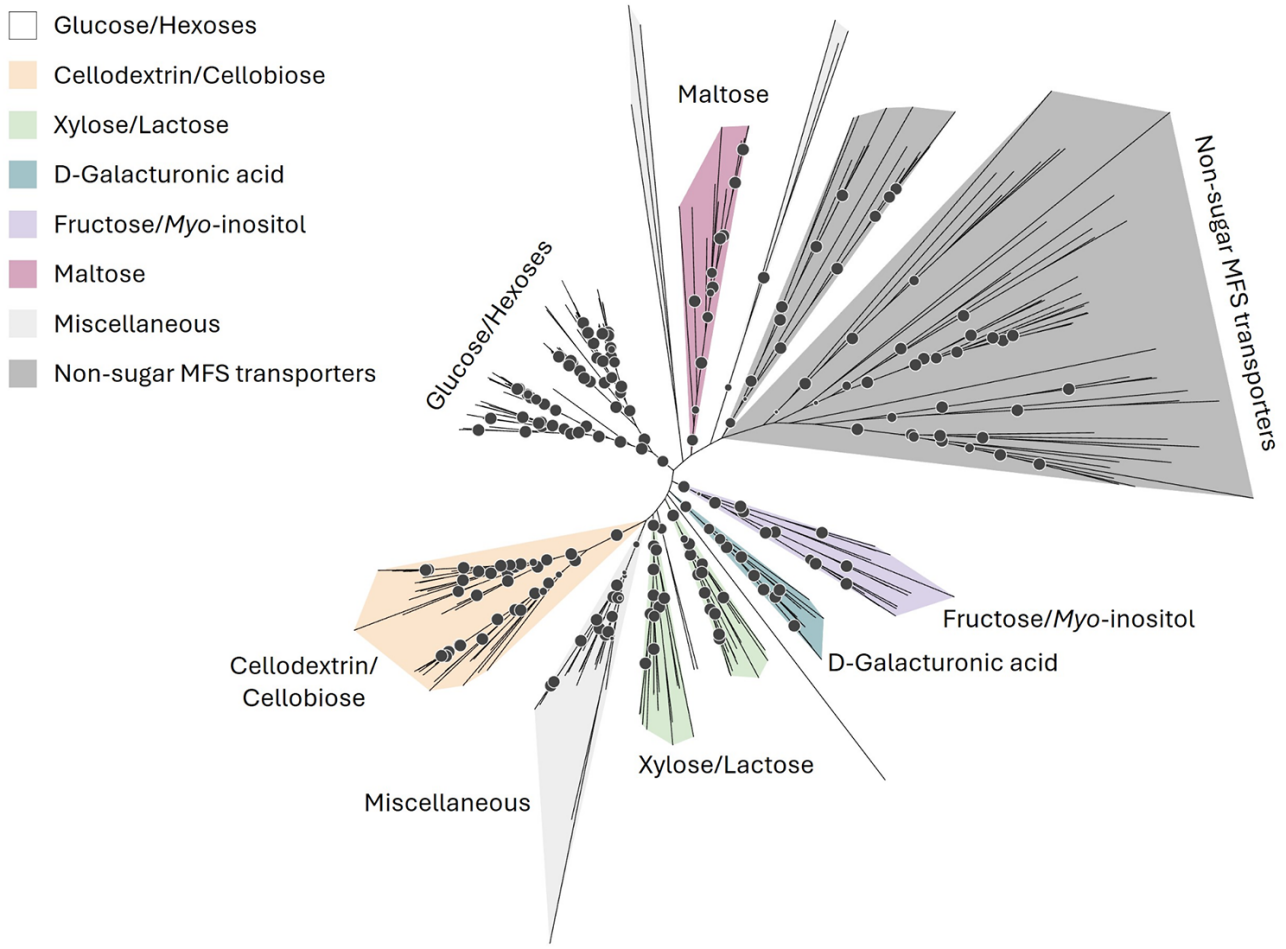
Evolutionary analysis of 92 in silico identified MFS carbon (sugar and non-sugar) transporters in *F. fomentarius* with transporters forming stable clades based on their substrates. Unrooted phylogenetic tree of 263 fungal MFS transporters, including 92 carbon MFS transporters from *F. fomentarius* [60, 61]. Clades are colour-coded according to substrates as shown in the legend. Circles shown at mid-branch points indicate bootstrap 1000 values, with the smallest and largest circles representing bootstrap values of 0.8 and 1.0, respectively

Carbon utilisation, does not solely rely on extracellular breakdown of available lignocellulose and carbohydrates. Carbon uptake is a crucial bottleneck for biomass formation. Therefore, we searched for genes encoding putative sugar transporters that contain the sugar transporter PFAM (PF00083) domain in the genome of *F. fomentarius*. Subsequently, we constructed a phylogenetic tree that contained 263 experimentally validated fungal sugar transporters of the Major Facilitator Superfamily (MFS, 263 in total) from [60, 61] and the 92 retrieved sequences from *F. fomentarius* (Fig. 6). We also used a recent machine learning model (SPOT) to predict the likelihood of substrate-transporter pairings (0: unlikely, 1: most likely [62]), and searched experimentally validated orthologues using the Transporter Classification DataBase (TCDB, Table 3 [63]). These tools and analyses enabled the compilation of a list of 36 putative sugar transporters in *F. fomentarius*, along with their predicted substrates.

To identify a confident prediction for transporter-substrate pairings, the non-sugar transporters were included in the analysis. The average likelihood of any of the non-sugar transporters pairing with any of the tested substrates yielded a “baseline” likelihood score of 0.142 ± 0.111. Pairings of putative sugar transport proteins with water averaged at 0.117 ± 0.0796. Scores for transporter-substrate pairings ranged from 0.002 to 0.826 (Table 3). D-galacturonic acid was not included in the analysis due to it not being among the substrates in the training set.

While the SPOT analysis results aligned with phylogeny-based predictions of putative substrates for some of the transporters, such as the sequences in the top five rows with glucose which had scores of > 0.500, this was not always the case. The putative cellodextrin/cellobiose transporters in the bottom five rows, for instance, did not produce scores of > 0.500 (0.292 ± 0.072) when paired with cellobiose. SPOT performs weaker for eukaryotic transporters and was not specifically trained for fungal transporters [62], which is why the overall accuracy of the prediction might have been lower than it would have been for prokaryotic transporters, for example. Nonetheless, this additional prediction tool further aided in compiling the list of sugar transporters from the initial 92 carbon MFS transporters.

Interestingly, and in contrast to CAZyme expression, the majority of putative transporters can be divided in constitutively expressed genes and weakly expressed/silent genes independently from the cultivation condition (Table 4). The highest expression was observed for the gene encoding the putative glucose transporter 1194748 (7285 ± 4607), followed by expression of genes encoding putative D-galacturonic acid (protein 1314663), glucose (protein 1367140) and cellobiose (protein 1208171) transporters with normalised raw counts of 2367 ± 767, 2399 ± 1630, and 2308 ± 1308, respectively. *F. fomentarius* transporter 1194748 is one of the candidates that have the potential of acting as a carbon sensor based on its sequence identity with known carbon sensors, such as MstC from *Aspergillus spp.* [64]. In contrast, the gene encoding another putative glucose transporter (protein 1211317), along with three other transporter-encoding genes, were not expressed at all under the tested conditions. However, it remains to be elucidated if this is the result of the chosen cultivation conditions or whether these genes are truly silent.

One potential candidate for an inducible transport system is protein 267012, a putative cellobiose transporter. Expression of the corresponding gene was increased in millet, hemp shives, and on the wood substrates compared to cultivation on laboratory media. This protein shares 39% sequence identify with Cdt-2 from *Neurospora crassa*, an essential transporter for cellulose and hemicellulose utilisation [65]. Another candidate for an inducible systems is protein 1232421 that shares 46% sequence identity with MstE from *A. nidulans* [66]. Yet, from the expression data, it appears that the corresponding gene is expressed constitutively. However, this might well be the result of the experimental design as end point samples were analysed, as opposed to changes in gene expression over the course of multiple days.

Nevertheless, these predictions are indispensable for targeted further studies aiming to optimise carbon utilisation and speeding up biomass formation during biomaterial production, a crucial step to ensuring industrial and economic feasibility. More inducible transport systems are likely to exist and should be identified as they may act as the vital link between extracellular substrate degradation and biomass production.

**Table 3:**
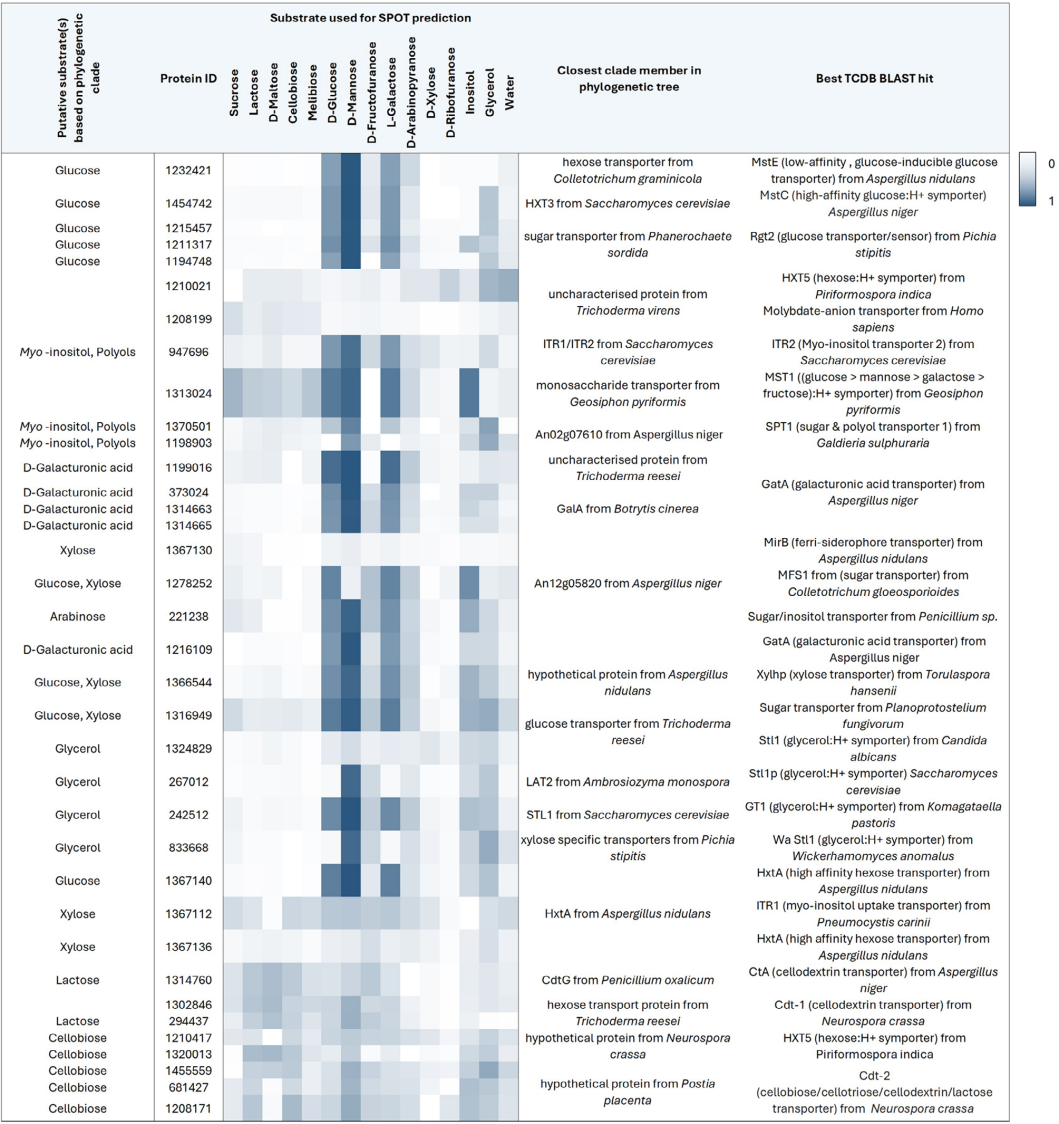
Curated list of 36 predicted MFS sugar transporters in *F. fomentarius* based on phylogeny and sequence similarity to validated transporters from TCDB. Putative substrates based on the respective protein’s position in the phylogenetic tree (Figure 6) are listed in the first column, JGI protein IDs in the second. SPOT-predicted likelihoods for transporter-substrate pairing are depicted by the heat map (white = 0 (not likely); dark blue = 1 (most likely)). The selection of substrates only contains compounds included over 20 times in the SPOT training set (as of July 2024). Closest clade members from the phylogenetic tree and best BLAST hits against the TCDB are shown in the last two columns, respectively

**Table 4:**
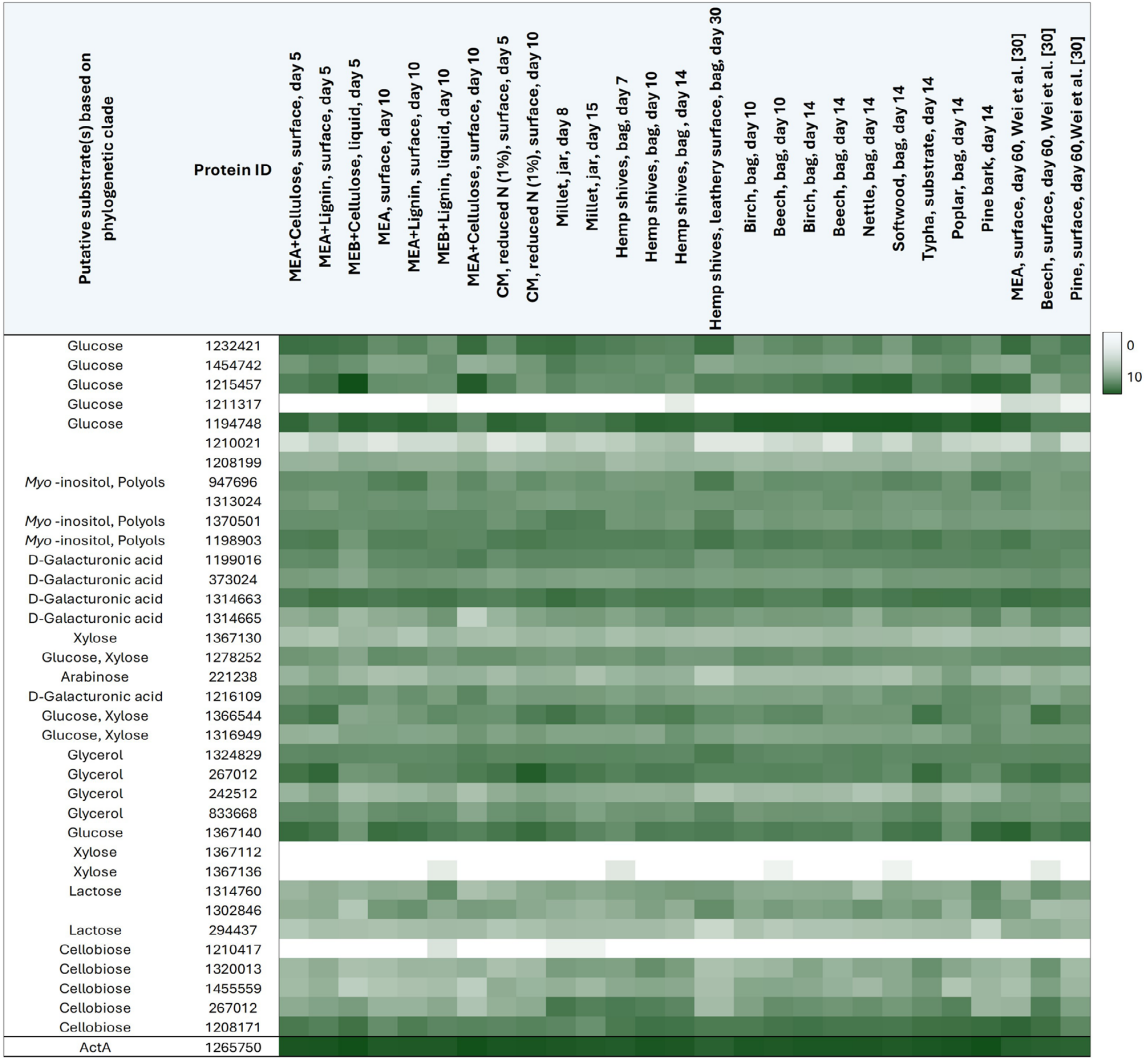
Gene expression heat map of 36 putative MFS sugar transporters in *F. fomentarius* under 27 different cultivation conditions highlighting the difference between predicted transporters that are constitutively expressed and weakly expressed/silent transporter-encoding genes. Phylogeny-based substrate predictions are shown in the first column as before (Table 2), JGI protein IDs in the second. Gene expression levels are depicted as log10 DeSeq normalised raw counts in the heat map with white = 0 and dark green = 10. Expression of *actA* is shown in the last row of the table

### Predicting *F. fomentarius* regulators for controlling substrate utilization during biomaterial formation

**Fig. 7:**
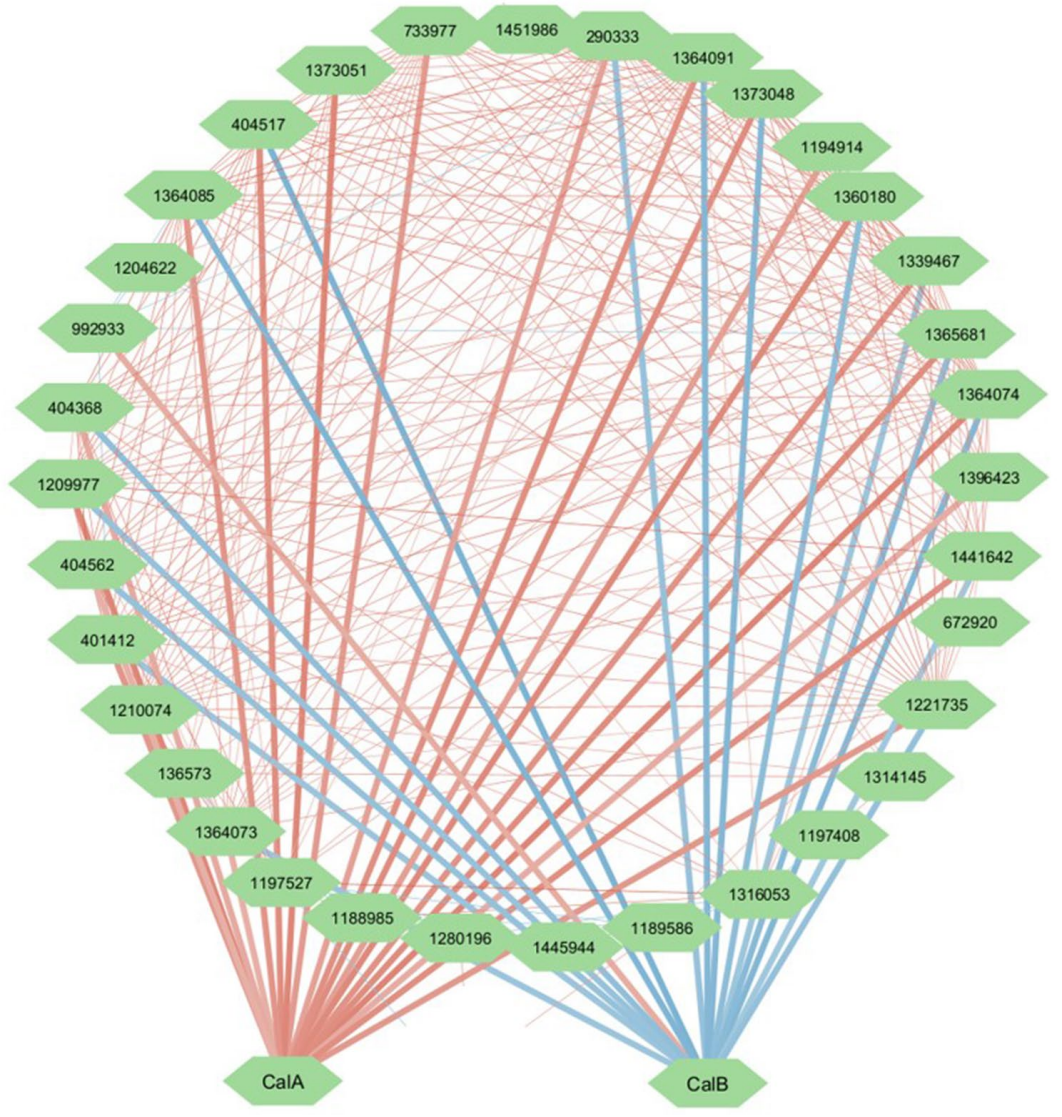
Predicted transcription factor encoding genes *calA* and *calB* are embedded in a *F. fomentarius* lignin-modifying network. Schematic diagram depicting co-expression pairs (FDR < 0.05) with Spearman values represented in red (positive) or blue (negative). Edges connecting to CalA*/*CalB are highlighted in bold. Note multiple lignin-modifying genes are positively and negatively co-expressed with CalA or CalB respectively

To provide further high-priority genetic leads to enable control of specific CAZyme cohorts, we generated a co-expression network from lignin-modifying enzymes (*n* = 40, Table 2A) and probed for highly interconnected transcription factors embedded in the network. Interestingly, this identified two transcription factors with high numbers of positive (protein ID 240859, 21 out of 40) or negative (protein ID 821366, 16 out of 40) correlations with this network (Fig. 7). Such connections are indicative of activating (positive connection) and repressing (negative connection) transcription factors, respectively. We hypothesize that the protein encoded by 821366 could potentially act as negative regulator in carbon catabolite repression because of its relatively high expression in glucose-containing media which may repress transcription of genes encoding lignin-modifying enzymes. It should be noted that a major carbon catabolite repressor was recently functionally analysed in *Coprinopsis cinerea* [67], and that its *F. fomentarius* orthologue (1201901) displayed only two connections with the lignin-modifying network (data not shown). We thus named 240859 and 821366 *c*o-expression *a*ssociated *l*ignin modifiers CalA and CalB, respectively. These data show the potential of employing co-expression networks for identifying global transcriptional regulators or engineering carbon degradation during biomaterial formation.

### The *F. fomentarius* genome encodes an astounding variety of contiguous co-expressed gene clusters

We hypothesized that *F. fomentarius* genes encoding secondary metabolite biosynthetic/tailoring enzymes or endophytic effector molecules (Additional File 2) could be located in co-expressed clusters on the genome. By analysing transcript values across the dataset as a function of chromosomal location [68], we found indeed evidence of contiguous co-expression of 926 genes in 246 clusters, with occupancy ranging from 3 to 12 genes (Additional File 2). These data confirmed co-expression of two or more genes at nine putative secondary metabolite clusters, including those predicted to synthesize terpenes (SM cluster numbers 4, 13, 17, 19, 30, 31), NRPSs (SM cluster numbers 5, 27), and RiPPs (SM cluster number 33, Table 5, Additional File 2). We also found clear evidence of six co-expressed effector clusters in *F. fomentarius*, (*n* = 26 genes), several of which displayed elevated expression when the fungus was growing on specific plant substrates during composite production (Additional File 2, Table 5).

It is worth noting that genes found in several of the contiguous effector clusters (numbers 13, 19, 38, and 243) encode putative hydrophobin domains. Such ‘hydrophobin effectors’ have been reported in other fungi [69], and it is unclear if the *F. fomentarius* proteins primarily function to modulate plant immunity, control cell wall hydrophobicity, or a combination of both. These clusters were obviously activated under specific conditions. For example, cluster 13 was highest expressed when grown on MEA + cellulose, or nettle/poplar substrates. Fascinatingly, growth on a leathery-like surface of a fruiting body isolated from hemp shives caused cluster 243 to be expressed at very high levels, whereas cluster 19 was turned off transcriptionally (Additional Table 1). Taken together, these data strongly suggest that these proteins are important to establish growth on plant substrates, and thus are high priority targets for genetic engineering of biomaterials produced with *F. fomentarius*.

**Fig. 8:**
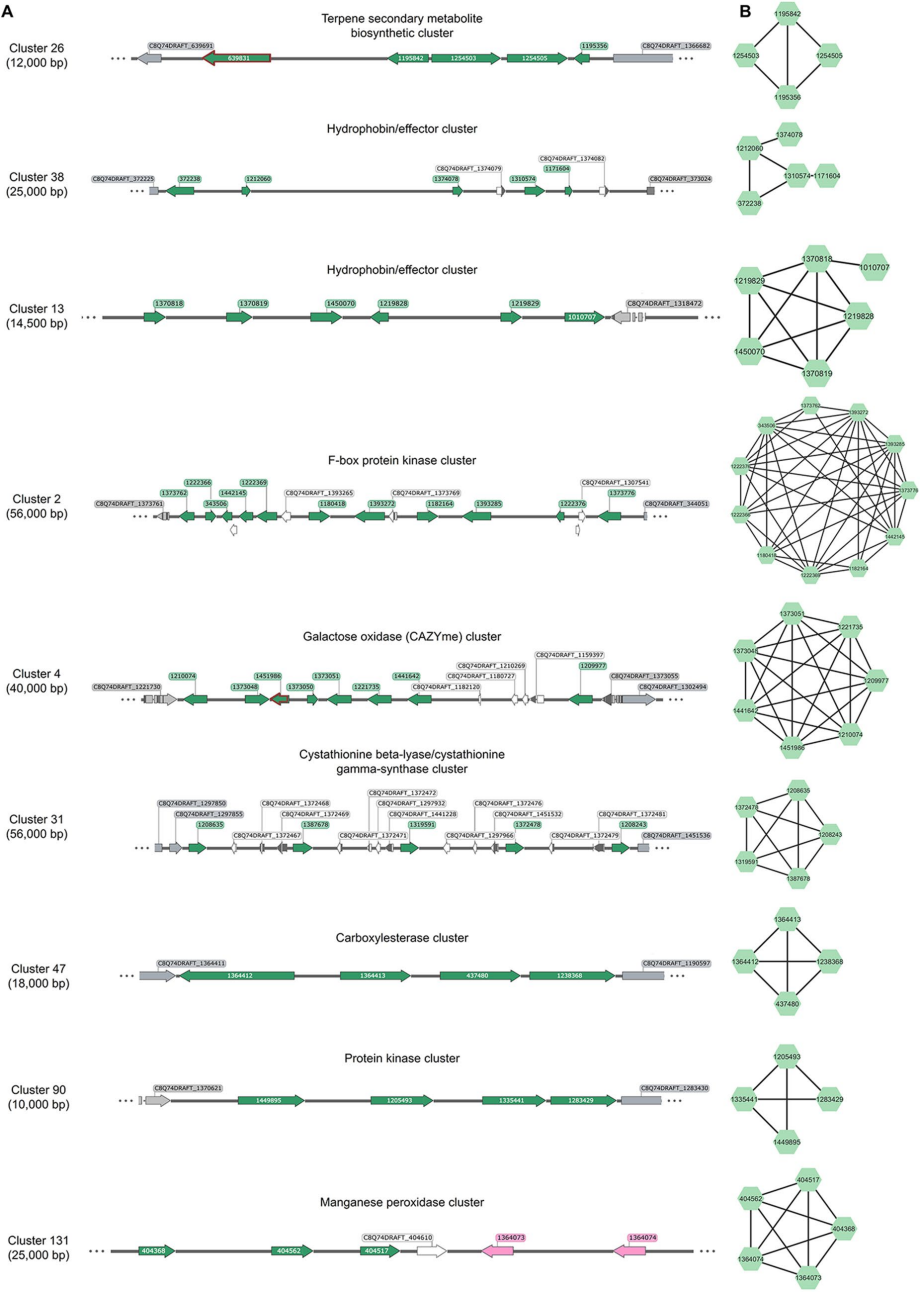
*F. fomentarius* encodes new classes of co-expressed gene clusters. Loci of selected contiguous co-expression gene clusters and their corresponding co-expression networks using only significant Spearman correlations (adjusted p-value < 0.5) are depicted. (**A**) The cluster numbers are shown on the left with the total number of base pairs (bp) shown in brackets. Clusters were defined as significantly co-expressed based on the FunGeneClusterS algorithm. Cluster titles are given above each cluster. Genes are represented by arrows: green = genes co-expressed within a cluster, white = silent/not co-expressed within a cluster, grey = outside predicted cluster boundary, flanking genes. Numbers shown in each arrow are the corresponding JGI protein IDs. See Table 5 for information of predicted gene products. Green arrows highlighted with orange outlines did not report significant Spearman correlations in a second network analysis reported in panel (**B**). The pink arrows in cluster 131 represent MnP-encoding genes that are part of the co-expression network but that were not predicted to be part of the cluster. (**B**) Co-expression networks of the corresponding clusters based on significant Spearman correlations using the R script developed in this study (Additional File 3). Corresponding protein IDs are shown in the nodes. The length of each edge and the resulting relative distance of two nodes to one another are based on the calculated Spearman correlation coefficient for each pairwise interaction. Note remarkable concordance between FunGeneClusterS algorithm and Spearman calculations from our standard R script (Additional File 3)

One striking yet unexpected discovery when analysing these data was the clear co-expression of physically clustered genes with identical predicted functions (Fig. 8). These included genes encoding CAZymes/carbohydrate metabolism (cellulases, chitinases, peroxidases, galactose oxidases, lectins), primary metabolism associated proteins (methionine biosynthesis, NADPH-/FADH-dependent reductases, carbonic anhydrases), transcription (RNA polymerases), protein-protein interactions (F-box proteins, kinases, BTB/POZ domains), membrane transporters, hydrophobins, and others (Table 5, Fig. 8, Additional File 2, Additional Table 1).

We could decipher clear co-ordinated expression of various clusters during specific growth conditions. For example, the manganese oxidase locus (cluster #131, 5 genes) was lowly expressed in most culture media, yet transcriptionally activated during growth on most plant substrates, an observation obviously suggesting the degradation of lignin (Table 5). Interestingly, such co-ordinated gene expression suggests manganese peroxidases encoded by the cluster may not be functionally redundant. Cluster 131 was lowly expressed in the leathery-like surface isolated from hemp shive composites but accompanied by activation of a putative terpene secondary metabolite locus (cluster #26, Table 5). It is also clear that other CAZyme gene clusters have substrate specific activation, e.g., the galactose oxidase locus (cluster #4, 8 genes) demonstrated highest gene expression when *F. fomentarius* was grown on millet or hemp, but showed lower activity on nettle, poplar or pine. It is interesting to speculate that the *F. fomentarius* galactose oxidase cluster may thus be active due to a faster degradation of pectins and hemicelluloses in millet or hemp. Expression of this cluster also seemed to be temporal, with high expression on beech at day 60 yet low expression at only day 14 (Table 5). Taken together, we report the discovery of entirely new types of fungal clusters. These loci are co-expressed during biomaterial growth and thus provide additional leads for engineering and understanding composites. They are also new frontiers for the study of fungal evolution and genome organisation, as delineating coordinated transcriptional deployment at the cluster level in other fungi has enabled key insights into the function, evolutionary history, epigenetic regulation, and biosynthetic pathways of the encoded genes [33, 44, 70–73].

**Table 5:**
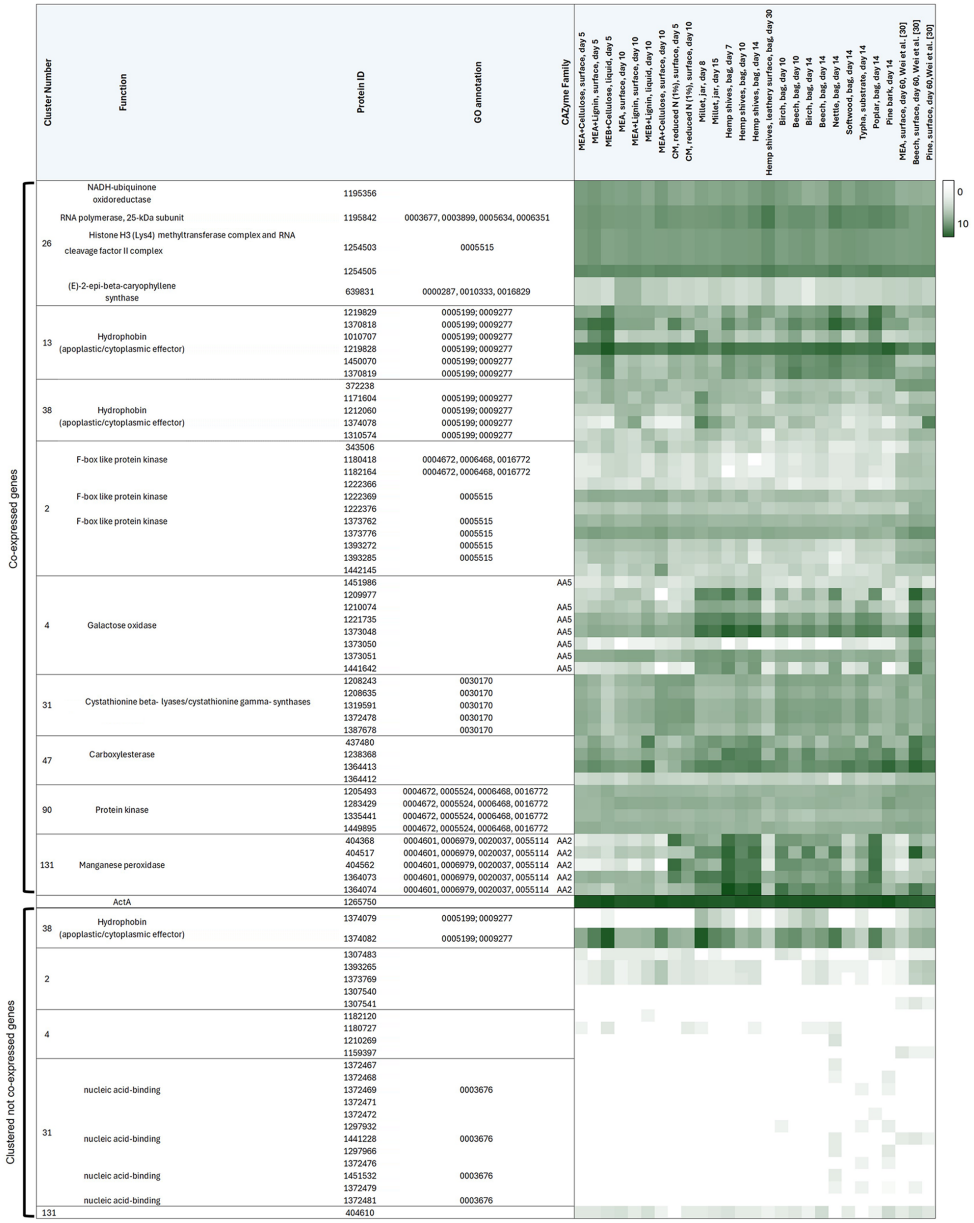
Gene expression heat map for selected contiguous co-expressed gene clusters (top section) and genes within clusters that are silent/not co-expressed (lower section) under the 27 cultivation conditions. Cluster numbers, predicted gene product function, JGI protein Ids, GO annotations and CAZY families (where applicable) are shown in the first five columns. Gene expression levels are shown as log10 DESeq2 normalised raw counts in the heat map with white=0 and dark green=10. Expression of *actA* is shown in the middle

## Discussion

Fungal-based materials promise an entirely new class of biologically produced and biodegradable materials from residual agricultural and forestry resources [10]. The benefits of producing renewable, CO_2_ emission negative, non-toxic, biodegradable, high-performance composites at scale can barely be over-stated [1, 4]. We predict that seismic changes in material production can mirror that of acid fermentation, where a single fungal species, *A. niger*, founded an entire renewable industry over a century ago [74]. We consider *F. fomentarius* will be one of the leading candidates to be that revolutionary fungus for this century [15, 24, 25]. To fully harness its potential, however, fundamental aspects of *F. fomentarius* biology need to be understood trough systems biology and optimized through genetic and metabolic engineering approaches. The plant pathologist Nick Talbot has likened working with genetically untouched fungal species as ‘taming a wild beast’ [75], yet encouragingly, advances in genomics and transcriptomics have advanced from their more descriptive origins to fully predictive disciplines. We have thus employed such approaches to predict various aspects of *F. fomentarius* biology to prioritize genetic leads for the bioengineering of biomaterial formation.

This study used updated online platforms [40, 76] to predict secondary metabolite biosynthetic gene clusters and effector proteins. These data demonstrate that *F. fomentarius* lacks known carcinogenic mycotoxins, conventional plant secondary metabolite virulence factors, and is enriched in apoplastic effectors, which in combination suggest this fungus is non-toxic and not a pathogen. Growth on or in a healthy/damaged tree will occur by a wide-range of non-pathogenic species, with fungal endophytes and mutualists being very common and likely underestimated in number [22, 23]. Experimental evidence from felled and then stored healthy European beech demonstrated extensive *F. fomentarius* colonization after 24 weeks, which certainly shows this fungus can exist without causing disease [22]. We thus support the conclusion of other authors [22, 23] that *F. fomentarius* is not a plant pathogen. From the perspective of gene functional predictions, the transcriptional resource and associated scripts developed in this study will enable easy and rapid generation of co-expression networks. Interrogation of networks where function of an encoded protein is virtually certain (Erg11, TubB), clearly showed these resources will be useful for functional prediction of the pathways or processes into which *F. fomentarius* genes are involved that currently have only dubious or absent annotations. Thus, like *A. niger* [31, 50], *C. albicans* [77], and *C. neoformans* [38], co-expression network technology can now be applied to update genome annotation in a biomaterial forming fungus.

We used such network analyses to predict transcription factor encoding genes that may orchestrate chitin and glucan cell wall biosynthesis. Indeed, the fungal cell wall defines cell shape and provides protection from the environment while mediating contact and adhesion with solid surfaces. We have recently used X-ray microcomputed tomography to demonstrate clear contact between *F. fomentarius* and hemp substrate particles during composite formation [24]. This analysis also confirmed hyphal branching increases with proximity to the plant, indicating cell wall directed changes to *F. fomentarius* filamentation [24]. Fungal cell walls are a complex yet organized mesh of chitin, β-1,3 D-glucan β-1,6 -glucans, β-1,4 -glucan, α-1,3-glucan, mannans, galactomannan, melanins, and a wide variety of proteins including hydrophobins [78–80]. Because of the remarkable co-expression of *cacA* with chitin and glucan synthases and *rhoA* orthologues we predict that the putative zinc finger transcription factor CacA regulates *F. fomentarius* cell wall synthesis. This gene is thus a high-priority candidate for future genetic engineering of hyphal adhesion and hyphal branching during biomaterial formation. Similarly, we found highly embedded transcription factors in a co-expression network with genes that encode lignin-modifying enzymes. These data suggest transcriptional activation and repression by CalA and CalB respectively, and their encoding genes *calA* and *calB* are therefore high-priority targets for engineering substrate utilisation of *F. fomentarius*. Moreover, this work also uncovered several candidate genes encoding highly expressed sugar transporters, which expression can be optimized in further studies going forward, to accelerate carbon uptake and metabolism by *F. fomentarius* and thus to shorten the time needed for biomaterial production.

Finally, we demonstrate here co-expression of contiguously clustered secondary metabolite and numerous apoplastic effector genes. These data were expected, as fungal secondary metabolite or effector clusters have been known since at least 1989 [81] and 2006 [72], respectively. However, our very remarkable yet unexpected discovery was the physical proximity and co-expression of a wide variety of genes predicted to encode proteins with identical function(s). To our knowledge, many of these types of clusters have not been previously reported e.g., kinases, F-box domains, CAZymes, RNA polymerases, NADPH/FADH reductases, lipases, ascorbic acid binding, carbonic anhydrases, and many others (Fig. 8, Additional File 2). The astonishing variation in gene function and highly comparable expression profiles are fascinating and might have various explanations. Firstly, the gene duplication, diversification and differential gene loss (DDL) hypothesis [82] proposes certain chromosomal loci may have high genetic and genomic variability (e.g., repeat-rich regions causing DNA polymerase slippage, or physical entanglement of subtelomeres causing mitotic cross-overs during anaphase). These loci might act as gene ‘factories’ where duplication events occur with high frequency [82]. An alternative explanation is horizontal gene transfer (HGT), where a selective advantage is more likely to occur to a recipient organism when entire biosynthetic pathways or many functionally related genes are introduced [83]. Finally, clustering could enable epigenetic regulation, as transfer between active or inactive chromatin states would enable coordinated activation/repression of entire functionally related gene cohorts [71]. Establishing which explanation(s) best explain novel *F. fomentarius* clusters is beyond the scope of this study, but something we will establish in future work. Excitingly, we have developed polycistronic expression technology in fungi [84], which could enable transcriptional control of entire clusters in a single heterologous system for simple functional analysis, thus enabling rapid understanding of biomaterial formation in future.

## Conclusions

The ability to understand and engineer the genetic basis of composite formation is of paramount importance if the full potential of fungal biomaterials is to be rapidly realised. We have used a variety of genomic, phylogenetic, and gene co-expression approaches to predict and analyse various aspects of *F. fomentarius* growth on renewable plant biomass. We showed here that the genome of this fungus does not encode known plant toxin clusters nor is enriched in putative apoplastic effectors, thus supporting field data of others reporting that *F. fomentarius* is not a plant pathogen. This study has also provided proof-of-principle that accurate predictions of gene function will be possible from our *F. fomentarius* transcriptional dataset, and we have provided diverse cultivation conditions and necessary scripts for such analyses by any mycologist. Additionally, we have for the first-time predicted carbon uptake systems and putative transcription factor encoding genes *cacA*/*calA*/*calB* which are high-priority targets for control of hyphal adhesion, hyphal branching, and lignocellulose degradation of *F. fomentarius* during composite formation. Entirely new classes of contiguous co-expressed gene clusters were also found, and their evolutionary history and the function of encoded gene products represents a new and exciting frontier in biomaterial research. Taken together, this study will enable the research community to further understand and engineer *F. fomentarius* growth for high-performance biomaterials at an industrial scale.

## Methods

### Strains used in this study

*F. fomentarius* PaPF11, a dikaryotic strain, was isolated in Brandenburg, Germany [15] and used for all cultivations performed in this study. PaPF11 was maintained on malt extract agar (Roth, Germany) and incubated at 25 °C in the dark for 8–10 days until plates where fully covered. Plates were kept not longer than 4 weeks at 2–8 °C for long-term storage before performing experiments.

### Cultivation of *F. fomentarius*

Samples for RNA sequencing were collected from PaPF11 cultures growing in 24 different conditions in biological duplicate (see Fig. 2). These cultivation conditions represent the variety of growth conditions established in our lab and cover a wide range of substrates. Mycelium was obtained from surface cultures on agar plates with different media and supplements, from liquid cultures in shake flasks, from millet cultures in jars and from bag cultures growing on various different substrates as described in [15].

### Preparation of surface culture samples

Surface cultures were grown on malt extract agar (MEA) or Aspergillus complete medium with reduced nitrogen content (55 mM glucose, 11 mM KH_2_PO_4_, 7 mM KCl, 178 nM H_3_BO_3_, 2 mM MgSO_4_, 76 nM ZnSO_4_, 0.07 mM NaNO_3_, 6.2 nM Na_2_MoO_4_, 18 nM FeSO_4_, 7.1 nM CoCl_2_, 6.4 nM CuSO_4_, 25 nM MnCl_2_, 174 nM EDTA, 0.005% (w/v) yeast extract and 0.001% (w/v) casamino acids). MEA plates or malt extract broth (MEB) were optionally supplemented with 10 g/L cellulose (Avicel, 20–160 μm particle size; Merck, Germany) or 10 g/L soluble lignin (ligninsulphonic acid calcium salt; Carl Roth, Germany). All plates were supplemented with 50 µg/mL ampicillin and 50 µg/mL streptomycin (AppliChem, Darmstadt, Germany). For inoculation of surface cultures, PaPF11 biomass was transferred onto a 0.45 μm nylon membrane (K058.1, Carl Roth, Karlsruhe, Germany) that had been placed on an agar plate. After 10 days, biomass was transferred from the membrane onto a new nylon membrane on identical plates using sterile toothpicks. The mycelium for analysis was scraped from the nylon membrane after five or 10 days and used for RNA extraction.

### Preparation of samples from liquid culture

For liquid cultures, mycelium from a fully overgrown ME plate was shredded in 500 mL ME broth with supplements using an immersion blender (Mixino 260; Siemens, Germany) for 30 s. From the culture, 100 ml were transferred to two 500 ml erlenmeyer flasks and incubated on an axial shaker at 150 rpm until sampling at 5 or 10 days. The mycelium was harvested by filtering the culture through filter paper (3 hw, Sartorius, Goettingen, Germany).

### Preparation of millet jar and bag culture samples

Millet jar cultures and bag cultures were prepared according to [15]. Millet was purchased from Mühle Schlingemann (Waltrop, Germany). Samples were taken from one to two cm below the surface of the substrate in the jar. The mixture of mycelium and millet was used for RNA extraction. Hemp shives (Hemparade) were purchased from Futtermittel Louven e.K (Mönchengladbach, Germany), birch and beech were supplied by Axtschlag GmbH (Heidesee, Germany). Softwood substrate (mix of spruce and pine) was purchased from Allspan (Wilburgstetten Germany). Pine bark was supplied by Floragard (Oldenburg, Germany). Nettle (Bornim I, harvest 2021), typha (Neukalen GMC, harvest 2012) and poplar (Bornim I, harvest 2019) were provided by the Leibniz Institute for Agricultural Engineering and Bioeconomy (Potsdam, Germany). For sampling at the designated time points, mycelium was scraped off from wood chips taken from one to two cm below the surface with the exception of the brown leather-like biomass found on the surface of hemp shive bags cultivated for 30 days.

### RNA extraction

For millet cultures, RNA was extracted using the RNeasy Plant Mini Kit for RNA Extraction (Quiagen, Hamburg, Germany) directly after sampling. For all other samples, biomass was submerged in 1 ml TriReagent (Zymo Research, Germany) with glass beads (0.75–1 mm diameter, Carl Roth, Germany) in screw-cap tubes and stored at -80 °C until further processing. The Direct-zol RNA Miniprep kit (Zymo Research, Freiburg im Breisgau, Germany) was used according to the manufacturer’s instructions but omitting the on-column DNAse treatment. Instead, gDNA was removed using the DNA-free DNA Removal Kit and samples were supplemented with SUPERaseIn (both from Life Technologies, Darmstadt, Germany) prior to storage at -80 °C until shipping on dry ice for library construction and sequencing.

### RNA sequencing and data processing

RNA sequencing was performed by GenomeScan B.V. (Leiden, The Netherlands) using the NEBNext Ultra II Directional RNA Library Prep Kit for Illumina (NEB) for library construction. Clustering and 150 bp paired-end sequencing was performed on a NovaSeq6000 (Illumina). NovaSeq control software NCS v1.7.5 was used. Image analysis, base calling, and quality check was performed with the Illumina data analysis pipeline RTA3.4.4 and Bcl2fastq v2.20. FASTQ files were subjected to quality control using MultiQC v1.13 (https://multiqc.info/). For generation of an indexed genome and mapping, STAR [85] version 2.7.10a was used (https://github.com/alexdobin/STAR). The genome index was generated with the reference genome (Fomfom_GCA_022606135.1) and annotation files for *F. fomentarius* CIRM-BRFM 1821 [29] submitted by the DOE Joint Genome Institute to the National Center for Biotechnology Information (NCBI). Files were downloaded on December 8th, 2023. The flag genomeSAindexNbases in STAR was set to 11. Mapping was performed with adjustments to the outFilterScoreMinOverLread and outFilterMatchNminOverLread parameters, both set to 0.33. The quality of mapping was assessed using MultiQC. All bam files were then further processed in Rstudio Server. With the list of bam files and the genome annotation file used for mapping, a count matrix of the raw counts was generated using the package GenomicFeatures to extract the gene positions from the GTF file of *F. fomentarius* CIRM-BRFM 1821 and GenomicAlignments to quantify mapped reads per gene. The count matrix was normalized using DESeq2 [86]. Subsequently, counts for biological duplicates were averaged and the final count matrix was rounded to the next full digit and saved as CSV file. The raw sequencing data are publicly available under the SRA accession numbers provided in Additional Table 2. Additionally, a dataset generated with *F. fomentarius* strain Wei 8498, isolated in Changbaishan, China [30] was reanalyzed during the bioinformatic analyses (BioProject PRJNA1029195).

### Analysis of RNA sequencing data and generation of expression heat maps

RNA sequencing raw counts were normalised using DESeq2 [86]. Heat maps were generated in Microsoft Excel but log10 transforming the raw counts. Conditional formatting was applied using a 2-colour scale with white and dark green representing 0 or 4.5 and 10, respectively.

### Compilation of list of lignocellulolytic CAZymes

The full list of putative *F. fomentarius* protein sequences was downloaded from JGI (at https://genome.jgi.doe.gov/portal/pages/dynamicOrganismDownload.jsf?organism=Fomfom1, July 2024 [87–89]), and submitted to the Conserved Unique Peptide Pattern (CUPP) tool [58, 59]. The list of putative lignocellulolytic, starch-acting and pectin-acting CAZymes was compiled by selecting laccases/(multi)-copper oxicases/phenoloxidases, manganese oxidases, versatile peroxidase, cellulases, xylanases, neuraminidases, glucosidases, trehalases, glucoamylases, mannosidases, pectinases, glucanases, and carbohydrate esterases [90].

### In silico identification of sugar transporters in *F. fomentarius*

Major Facilitator Superfamily (MFS) transporters containing the sugar transporter domain PF00083 were identified following the workflow described by [60]. This yielded sugar and non-sugar transporters, which were further distinguished by evolutionary analysis that included experimentally validated sugar transporters from [60, 61]. Sequences were aligned in JalView version 2.11.5.0 using MUSCLE version 3.8.31 and subsequently used to construct a maximum-likelihood tree containing all 236 protein sequences was constructed using IQ-TREE multicore version 1.6.12 [91–94]. A bootstrap of 1000 was applied using UFBOOT integrated into IQ-TREE [95]. The consensus tree was visualised and edited in iTOL (Letunic and Bork, 2007, available from https://itol.embl.de/itol.cgi).

### SPOT analysis of putative sugar transporters

Substrate predictions for in silico identified sugar transporters were carried out using the SPOT tool [62]. Only substrates that were included more than 20 times in the model training set were included in the analysis. The corresponding heat map showing likelihood values of between 0 and 1 for each pairwise transporter-substrate were generated using Microsoft Excel with white representing 0 and dark blue representing 1.

### BLAST against the transporter classification database (TCDB)

The built-in BLAST tool [97] was used to find similar proteins stored in the TCDB, many of which have been validated and characterised experimentally [63]. The blast tool was used with default setting and a more stringent e-value cut off of 0.001.

### Predicting *F. fomentarius* secondary metabolite loci and secreted effector encoding genes

Secondary metabolite loci were predicted using AntiSMASH version 7.0 and default parameters [98]. Genes encoding signal peptides were identified by screening predicted amino acid sequences with signalP version 6.0 [39], and additionally NetGPI version 1.1 [47]. Effectors were predicted from amino acid sequences using EffectorP version 3.0 [40].

### Identification of contiguous co-expressed gene clusters

Contiguous co-expressed loci were identified from normalized gene expression data (Additional File 2) using FunGeneCluster and default parameters [68], with a minimum of 3 co-expressed genes considered clustered. Genes predicted to be co-expressed were subsequently re-analysed using our in-house script (Additional File 4) to confirm co-expression across the dataset. Gene clusters were visualised using SnapGene (available from https://www.snapgene.com/).

### Generation and visualisation of co-expression networks

Co-expression networks were generated using R version 4.3.1. Scripts were debugged using ChatGPT. Spearman correlation coefficients were calculated pairwise between specified gene cohorts and comparisons that yielded a Benjamini–Hochberg False Discovery Rate adjusted p-value of < 0.5 were included in subsequent networks (Additional File 4). These were visualised using Cytoscape version 3.10.2 [99] using the default layout algorithm with spring coefficient of 1E^− 4^, a spring length of 50 and a default node mass of 3.

## Electronic supplementary material

Below is the link to the electronic supplementary material.

Supplementary Material 1

Supplementary Material 2

Supplementary Material 3

Supplementary Material 4

Supplementary Material 5

Supplementary Material 6

Supplementary Material 7

Supplementary Table 1

Supplementary Table 2

## Data Availability

All data generated or analysed during this study are included in this published manuscript and its Additional information files. All raw sequencing data are available at NCBI Bioproject number PRJNA957595. Full details for raw sequencing data are given in Additional Table 2.
