# Supplementary material for: Uncovering the transcriptional landscape of Fomes fomentarius during fungal-based material production through gene co-expression network analysis
Source: Fungal Biol Biotechnol. 2025 Feb 13;12:1. doi: 10.1186/s40694-024-00192-3 (PMC11827164; doi:10.1186/s40694-024-00192-3)
Supplement: Supplementary file 1 — Supplementary Material 1 [file 40694_2024_192_MOESM1_ESM.zip › index.html]

Fomfom1\_AssemblyScaffolds - 39 region(s) - antiSMASH results


antiSMASH version 7.1.0

Download

- Download all results
- Download GenBank summary file
- Download JSON results file
- Download log file

About

Help

Contact

Select genomic region:

Overview

1.1

1.2

1.3

1.4

1.5

1.6

2.1

3.1

3.2

3.3

4.1

4.2

4.3

4.4

6.1

7.1

7.2

7.3

7.4

8.1

9.1

9.2

10.1

11.1

11.2

11.3

12.1

12.2

12.3

13.1

13.2

14.1

14.2

14.3

14.4

15.1

16.1

16.2

19.1

### Identified secondary metabolite regions using strictness 'relaxed'

**scaffold\_1**

| Region | Type | From | To | Most similar known cluster | | Similarity |
| --- | --- | --- | --- | --- | --- | --- |
| Region&nbsp1.1 | terpene | 611,883 | 633,221 | armillyl orsellinate/8α-hydroxy-6-protoilludene | Terpene | 60% |
| Region&nbsp1.2 | NRPS | 2,535,681 | 2,583,811 | basidioferrin | Other | 100% |
| Region&nbsp1.3 | terpene | 2,699,638 | 2,720,679 |  | | |
| Region&nbsp1.4 | terpene | 3,475,416 | 3,496,977 | (+)-δ-cadinol | Terpene | 100% |
| Region&nbsp1.5 | NRPS-like | 3,769,170 | 3,813,302 |  | | |
| Region&nbsp1.6 | terpene | 4,715,014 | 4,736,157 |  | | |

**scaffold\_2**

| Region | Type | From | To | Most similar known cluster | | Similarity |
| --- | --- | --- | --- | --- | --- | --- |
| Region&nbsp2.1 | NRPS-like | 2,085,108 | 2,129,960 |  | | |

**scaffold\_3**

| Region | Type | From | To | Most similar known cluster | | Similarity |
| --- | --- | --- | --- | --- | --- | --- |
| Region&nbsp3.1 | terpene | 566,520 | 588,565 |  | | |
| Region&nbsp3.2 | terpene | 2,291,633 | 2,314,747 | clavaric acid | Terpene | 100% |
| Region&nbsp3.3 | terpene | 2,401,713 | 2,424,823 | clavaric acid | Terpene | 100% |

**scaffold\_4**

| Region | Type | From | To | Most similar known cluster | | Similarity |
| --- | --- | --- | --- | --- | --- | --- |
| Region&nbsp4.1 | fungal-RiPP-like | 750,987 | 812,290 |  | | |
| Region&nbsp4.2 | terpene | 1,313,548 | 1,349,437 | (+)-δ-cadinol | Terpene | 40% |
| Region&nbsp4.3 | terpene | 1,636,898 | 1,663,675 |  | | |
| Region&nbsp4.4 | T1PKS | 2,445,951 | 2,494,240 |  | | |

**scaffold\_6**

| Region | Type | From | To | Most similar known cluster | | Similarity |
| --- | --- | --- | --- | --- | --- | --- |
| Region&nbsp6.1 | NRPS-like | 2,225,160 | 2,269,419 |  | | |

**scaffold\_7**

| Region | Type | From | To | Most similar known cluster | | Similarity |
| --- | --- | --- | --- | --- | --- | --- |
| Region&nbsp7.1 | T1PKS | 109,106 | 154,660 |  | | |
| Region&nbsp7.2 | terpene | 1,383,557 | 1,405,009 |  | | |
| Region&nbsp7.3 | terpene | 1,939,765 | 1,961,203 |  | | |
| Region&nbsp7.4 | terpene | 1,979,400 | 2,007,923 |  | | |

**scaffold\_8**

| Region | Type | From | To | Most similar known cluster | | Similarity |
| --- | --- | --- | --- | --- | --- | --- |
| Region&nbsp8.1 | terpene | 470,272 | 503,064 |  | | |

**scaffold\_9**

| Region | Type | From | To | Most similar known cluster | | Similarity |
| --- | --- | --- | --- | --- | --- | --- |
| Region&nbsp9.1 | T1PKS | 339,306 | 385,036 |  | | |
| Region&nbsp9.2 | NRPS-like | 1,972,441 | 2,016,999 |  | | |

**scaffold\_10**

| Region | Type | From | To | Most similar known cluster | | Similarity |
| --- | --- | --- | --- | --- | --- | --- |
| Region&nbsp10.1 | NRPS-like | 221,909 | 266,107 |  | | |

**scaffold\_11**

| Region | Type | From | To | Most similar known cluster | | Similarity |
| --- | --- | --- | --- | --- | --- | --- |
| Region&nbsp11.1 | T1PKS | 1 | 40,651 |  | | |
| Region&nbsp11.2 | terpene | 925,561 | 947,053 | (+)-δ-cadinol | Terpene | 100% |
| Region&nbsp11.3 | fungal-RiPP-like | 1,033,338 | 1,094,299 |  | | |

**scaffold\_12**

| Region | Type | From | To | Most similar known cluster | | Similarity |
| --- | --- | --- | --- | --- | --- | --- |
| Region&nbsp12.1 | NRPS-like | 256,606 | 300,912 |  | | |
| Region&nbsp12.2 | NRPS-like | 316,272 | 389,811 |  | | |
| Region&nbsp12.3 | NRPS-like | 405,136 | 449,531 |  | | |

**scaffold\_13**

| Region | Type | From | To | Most similar known cluster | | Similarity |
| --- | --- | --- | --- | --- | --- | --- |
| Region&nbsp13.1 | terpene | 93,968 | 115,242 | armillyl orsellinate/8α-hydroxy-6-protoilludene | Terpene | 60% |
| Region&nbsp13.2 | terpene | 137,996 | 159,259 |  | | |

**scaffold\_14**

| Region | Type | From | To | Most similar known cluster | | Similarity |
| --- | --- | --- | --- | --- | --- | --- |
| Region&nbsp14.1 | terpene | 296,700 | 317,904 |  | | |
| Region&nbsp14.2 | fungal-RiPP-like | 324,080 | 515,825 |  | | |
| Region&nbsp14.3 | NRPS-like | 829,881 | 895,923 |  | | |
| Region&nbsp14.4 | NRPS-like | 914,477 | 958,700 |  | | |

**scaffold\_15**

| Region | Type | From | To | Most similar known cluster | | Similarity |
| --- | --- | --- | --- | --- | --- | --- |
| Region&nbsp15.1 | terpene | 167,779 | 189,620 |  | | |

**scaffold\_16**

| Region | Type | From | To | Most similar known cluster | | Similarity |
| --- | --- | --- | --- | --- | --- | --- |
| Region&nbsp16.1 | terpene | 1 | 11,816 |  | | |
| Region&nbsp16.2 | terpene | 34,800 | 79,187 |  | | |

**scaffold\_19**

| Region | Type | From | To | Most similar known cluster | | Similarity |
| --- | --- | --- | --- | --- | --- | --- |
| Region&nbsp19.1 | terpene | 126,382 | 147,637 |  | | |

| Region | Type | From | To | Most similar known cluster | | Similarity |
| --- | --- | --- | --- | --- | --- | --- |
| Region&nbsp1.1 | terpene | 611,883 | 633,221 | armillyl orsellinate/8α-hydroxy-6-protoilludene | Terpene | 60% |
| Region&nbsp1.2 | NRPS | 2,535,681 | 2,583,811 | basidioferrin | Other | 100% |
| Region&nbsp1.3 | terpene | 2,699,638 | 2,720,679 |  | | |
| Region&nbsp1.4 | terpene | 3,475,416 | 3,496,977 | (+)-δ-cadinol | Terpene | 100% |
| Region&nbsp1.5 | NRPS-like | 3,769,170 | 3,813,302 |  | | |
| Region&nbsp1.6 | terpene | 4,715,014 | 4,736,157 |  | | |
| Region&nbsp2.1 | NRPS-like | 2,085,108 | 2,129,960 |  | | |
| Region&nbsp3.1 | terpene | 566,520 | 588,565 |  | | |
| Region&nbsp3.2 | terpene | 2,291,633 | 2,314,747 | clavaric acid | Terpene | 100% |
| Region&nbsp3.3 | terpene | 2,401,713 | 2,424,823 | clavaric acid | Terpene | 100% |
| Region&nbsp4.1 | fungal-RiPP-like | 750,987 | 812,290 |  | | |
| Region&nbsp4.2 | terpene | 1,313,548 | 1,349,437 | (+)-δ-cadinol | Terpene | 40% |
| Region&nbsp4.3 | terpene | 1,636,898 | 1,663,675 |  | | |
| Region&nbsp4.4 | T1PKS | 2,445,951 | 2,494,240 |  | | |
| Region&nbsp6.1 | NRPS-like | 2,225,160 | 2,269,419 |  | | |
| Region&nbsp7.1 | T1PKS | 109,106 | 154,660 |  | | |
| Region&nbsp7.2 | terpene | 1,383,557 | 1,405,009 |  | | |
| Region&nbsp7.3 | terpene | 1,939,765 | 1,961,203 |  | | |
| Region&nbsp7.4 | terpene | 1,979,400 | 2,007,923 |  | | |
| Region&nbsp8.1 | terpene | 470,272 | 503,064 |  | | |
| Region&nbsp9.1 | T1PKS | 339,306 | 385,036 |  | | |
| Region&nbsp9.2 | NRPS-like | 1,972,441 | 2,016,999 |  | | |
| Region&nbsp10.1 | NRPS-like | 221,909 | 266,107 |  | | |
| Region&nbsp11.1 | T1PKS | 1 | 40,651 |  | | |
| Region&nbsp11.2 | terpene | 925,561 | 947,053 | (+)-δ-cadinol | Terpene | 100% |
| Region&nbsp11.3 | fungal-RiPP-like | 1,033,338 | 1,094,299 |  | | |
| Region&nbsp12.1 | NRPS-like | 256,606 | 300,912 |  | | |
| Region&nbsp12.2 | NRPS-like | 316,272 | 389,811 |  | | |
| Region&nbsp12.3 | NRPS-like | 405,136 | 449,531 |  | | |
| Region&nbsp13.1 | terpene | 93,968 | 115,242 | armillyl orsellinate/8α-hydroxy-6-protoilludene | Terpene | 60% |
| Region&nbsp13.2 | terpene | 137,996 | 159,259 |  | | |
| Region&nbsp14.1 | terpene | 296,700 | 317,904 |  | | |
| Region&nbsp14.2 | fungal-RiPP-like | 324,080 | 515,825 |  | | |
| Region&nbsp14.3 | NRPS-like | 829,881 | 895,923 |  | | |
| Region&nbsp14.4 | NRPS-like | 914,477 | 958,700 |  | | |
| Region&nbsp15.1 | terpene | 167,779 | 189,620 |  | | |
| Region&nbsp16.1 | terpene | 1 | 11,816 |  | | |
| Region&nbsp16.2 | terpene | 34,800 | 79,187 |  | | |
| Region&nbsp19.1 | terpene | 126,382 | 147,637 |  | | |

No secondary metabolite regions were found in these records:
:   **scaffold\_5**
:   **scaffold\_17**
:   **scaffold\_18**
:   **scaffold\_20**
:   **scaffold\_21**
:   **scaffold\_22**
:   **scaffold\_23**
:   **scaffold\_24**
:   **scaffold\_25**
:   **scaffold\_26**

Compact view

scaffold\_1 - Region 1 - terpene

Shows the layout of the region, marking coding sequences and areas of interest. Clicking a gene will select it and show any relevant details. Clicking an area feature (e.g. a candidate cluster) will select all coding sequences within that area. Double clicking an area feature will zoom to that area. Multiple genes and area features can be selected by clicking them while holding the Ctrl key.  
More detailed help is available here.

Download region GenBank file

Download region SVG

Location: 611,883 - 633,221 nt. (total: 21,339 nt)
Show pHMM detection rules used

terpene: (Terpene\_synth or Terpene\_synth\_C or phytoene\_synt or Lycopene\_cycl or terpene\_cyclase or NapT7 or fung\_ggpps or fung\_ggpps2 or trichodiene\_synth or TRI5)

#### Legend:

core biosynthetic genes

additional biosynthetic genes

transport-related genes

regulatory genes

other genes

resistance

reset view

zoom to selection

Gene details

Shows details of the most recently selected gene, including names, products, location, and other annotations.

Select a gene to view the details available for it

Gene overview

KnownClusterBlast

SubClusterBlast

Gene/CDS overview

A brief tabular summary of genes/CDS features within the region.  
Filtering the table will also search biosynthetic profiles and gene function data. If enabled, the overview will then zoom to show the area covered by the filtered selection.  
Genes selected in the region drawing above will be marked in the table with an indicator to the left of the gene name.

Filter:

Automatically zoom to filtered/selected features

| Identifier | Product | Length | | Function | Sequence | | NCBI Blast | Filter details |
| --- | --- | --- | --- | --- | --- | --- | --- | --- |
|  |  | NT | AA |  | NT | AA |  |  |

No genes match the given filter

Similar known gene clusters from MIBiG 3.1

Shows clusters from the MiBIG database that are similar to the current region. Genes marked with the same colour are interrelated. White genes have no relationship.  
Click on reference genes to show details of similarities to genes within the current region.  
Click on an accession to open that entry in the MiBIG database.

All hits

armillyl orsellinate/8α-hydroxy-6-protoilludene
Download graphic

Similar subclusters

Shows sub-cluster units that are similar to the current region. Genes marked with the same colour are interrelated. White genes have no relationship.  
Click on reference genes to show details of similarities to genes within the current region.

No matches found.

scaffold\_1 - Region 2 - NRPS

Shows the layout of the region, marking coding sequences and areas of interest. Clicking a gene will select it and show any relevant details. Clicking an area feature (e.g. a candidate cluster) will select all coding sequences within that area. Double clicking an area feature will zoom to that area. Multiple genes and area features can be selected by clicking them while holding the Ctrl key.  
More detailed help is available here.

Download region GenBank file

Download region SVG

Location: 2,535,681 - 2,583,811 nt. (total: 48,131 nt)
Show pHMM detection rules used

NRPS: cds(Condensation and (AMP-binding or A-OX))

#### Legend:

core biosynthetic genes

additional biosynthetic genes

transport-related genes

regulatory genes

other genes

resistance

reset view

zoom to selection

Gene details

Shows details of the most recently selected gene, including names, products, location, and other annotations.

Select a gene to view the details available for it

Gene overview

NRPS/PKS domains

KnownClusterBlast

SubClusterBlast

NRPS/PKS modules

Gene/CDS overview

A brief tabular summary of genes/CDS features within the region.  
Filtering the table will also search biosynthetic profiles and gene function data. If enabled, the overview will then zoom to show the area covered by the filtered selection.  
Genes selected in the region drawing above will be marked in the table with an indicator to the left of the gene name.

Filter:

Automatically zoom to filtered/selected features

| Identifier | Product | Length | | Function | Sequence | | NCBI Blast | Filter details |
| --- | --- | --- | --- | --- | --- | --- | --- | --- |
|  |  | NT | AA |  | NT | AA |  |  |

No genes match the given filter

Detailed domain annotation

Shows NRPS- and PKS-related domains for each feature that contains them. Click on each domain for more information about the domain's location, consensus monomer prediction, and other details.  
A domain glossary is available here, and an explanation of the visualisation is available here.

Selected features only

Show module domains

Similar known gene clusters from MIBiG 3.1

Shows clusters from the MiBIG database that are similar to the current region. Genes marked with the same colour are interrelated. White genes have no relationship.  
Click on reference genes to show details of similarities to genes within the current region.  
Click on an accession to open that entry in the MiBIG database.

All hits

basidioferrin
Download graphic

Similar subclusters

Shows sub-cluster units that are similar to the current region. Genes marked with the same colour are interrelated. White genes have no relationship.  
Click on reference genes to show details of similarities to genes within the current region.

No matches found.

Module view

Shows module structures for each candidate cluster in NRPS and PKS regions.   
Genes are shown in predicted order, and are only present when containing at least one complete module.   
A domain glossary is available here, and an explanation of the visualisation is available here.

Candidate 2 (2535680 - 2583811): single NRPS

Legend

NRPS/PKS products

NRPS/PKS substrates

Predicted core structure(s)

Shows estimated product structure and polymer for each candidate cluster in the region. To show the product, click on the expander or the candidate cluster feature drawn in the overview.

For candidate cluster 2, location 2535680 - 2583811:

Rough prediction of core scaffold based on assumed PKS/NRPS colinearity; tailoring reactions not taken into account

**Polymer prediction:**
:   (X)

  
Direct lookup in NORINE database:
strict
or
relaxed

Link to NORINE database query form

NRPS/PKS substrate predictions

Shows the predicted substrates for each adenylation domain and acyltransferase within genes. Each gene prediction can be expanded to view detailed predictions of each domain. Each prediction can be expanded to view the predictions by tool (and, for some tools, further expanded for extra details).

**jgi.p\_Fomfom1\_1187419**: X

:   **AMP-binding (260..670)**: X

    nrpys: (unknown)

    SVM prediction details:
    :   Predicted physicochemical class:
        :   N/A

        Large clusters prediction:
        :   N/A

        Small clusters prediction:
        :   N/A

        Single AA prediction:
        :   N/A

    Stachelhaus prediction details:
    :   Stachelhaus sequence:
        :   DVAGAGFIGK

        Nearest Stachelhaus code(s):
        :   E-ahmohOrn DVGGGGVIGK (71% 8Å match)

        Stachelhaus code match:
        :   70% (weak)

scaffold\_1 - Region 3 - terpene

Shows the layout of the region, marking coding sequences and areas of interest. Clicking a gene will select it and show any relevant details. Clicking an area feature (e.g. a candidate cluster) will select all coding sequences within that area. Double clicking an area feature will zoom to that area. Multiple genes and area features can be selected by clicking them while holding the Ctrl key.  
More detailed help is available here.

Download region GenBank file

Download region SVG

Location: 2,699,638 - 2,720,679 nt. (total: 21,042 nt)
Show pHMM detection rules used

terpene: (Terpene\_synth or Terpene\_synth\_C or phytoene\_synt or Lycopene\_cycl or terpene\_cyclase or NapT7 or fung\_ggpps or fung\_ggpps2 or trichodiene\_synth or TRI5)

#### Legend:

core biosynthetic genes

additional biosynthetic genes

transport-related genes

regulatory genes

other genes

resistance

reset view

zoom to selection

Gene details

Shows details of the most recently selected gene, including names, products, location, and other annotations.

Select a gene to view the details available for it

Gene overview

KnownClusterBlast

SubClusterBlast

Gene/CDS overview

A brief tabular summary of genes/CDS features within the region.  
Filtering the table will also search biosynthetic profiles and gene function data. If enabled, the overview will then zoom to show the area covered by the filtered selection.  
Genes selected in the region drawing above will be marked in the table with an indicator to the left of the gene name.

Filter:

Automatically zoom to filtered/selected features

| Identifier | Product | Length | | Function | Sequence | | NCBI Blast | Filter details |
| --- | --- | --- | --- | --- | --- | --- | --- | --- |
|  |  | NT | AA |  | NT | AA |  |  |

No genes match the given filter

Similar known gene clusters from MIBiG 3.1

Shows clusters from the MiBIG database that are similar to the current region. Genes marked with the same colour are interrelated. White genes have no relationship.  
Click on reference genes to show details of similarities to genes within the current region.  
Click on an accession to open that entry in the MiBIG database.

No matches found.

Similar subclusters

Shows sub-cluster units that are similar to the current region. Genes marked with the same colour are interrelated. White genes have no relationship.  
Click on reference genes to show details of similarities to genes within the current region.

No matches found.

scaffold\_1 - Region 4 - terpene

Shows the layout of the region, marking coding sequences and areas of interest. Clicking a gene will select it and show any relevant details. Clicking an area feature (e.g. a candidate cluster) will select all coding sequences within that area. Double clicking an area feature will zoom to that area. Multiple genes and area features can be selected by clicking them while holding the Ctrl key.  
More detailed help is available here.

Download region GenBank file

Download region SVG

Location: 3,475,416 - 3,496,977 nt. (total: 21,562 nt)
Show pHMM detection rules used

terpene: (Terpene\_synth or Terpene\_synth\_C or phytoene\_synt or Lycopene\_cycl or terpene\_cyclase or NapT7 or fung\_ggpps or fung\_ggpps2 or trichodiene\_synth or TRI5)

#### Legend:

core biosynthetic genes

additional biosynthetic genes

transport-related genes

regulatory genes

other genes

resistance

reset view

zoom to selection

Gene details

Shows details of the most recently selected gene, including names, products, location, and other annotations.

Select a gene to view the details available for it

Gene overview

KnownClusterBlast

SubClusterBlast

Gene/CDS overview

A brief tabular summary of genes/CDS features within the region.  
Filtering the table will also search biosynthetic profiles and gene function data. If enabled, the overview will then zoom to show the area covered by the filtered selection.  
Genes selected in the region drawing above will be marked in the table with an indicator to the left of the gene name.

Filter:

Automatically zoom to filtered/selected features

| Identifier | Product | Length | | Function | Sequence | | NCBI Blast | Filter details |
| --- | --- | --- | --- | --- | --- | --- | --- | --- |
|  |  | NT | AA |  | NT | AA |  |  |

No genes match the given filter

Similar known gene clusters from MIBiG 3.1

Shows clusters from the MiBIG database that are similar to the current region. Genes marked with the same colour are interrelated. White genes have no relationship.  
Click on reference genes to show details of similarities to genes within the current region.  
Click on an accession to open that entry in the MiBIG database.

All hits

(+)-δ-cadinol
Download graphic

Similar subclusters

Shows sub-cluster units that are similar to the current region. Genes marked with the same colour are interrelated. White genes have no relationship.  
Click on reference genes to show details of similarities to genes within the current region.

No matches found.

scaffold\_1 - Region 5 - NRPS-like

Shows the layout of the region, marking coding sequences and areas of interest. Clicking a gene will select it and show any relevant details. Clicking an area feature (e.g. a candidate cluster) will select all coding sequences within that area. Double clicking an area feature will zoom to that area. Multiple genes and area features can be selected by clicking them while holding the Ctrl key.  
More detailed help is available here.

Download region GenBank file

Download region SVG

Location: 3,769,170 - 3,813,302 nt. (total: 44,133 nt)
Show pHMM detection rules used

NRPS-like: cds((PP-binding or NAD\_binding\_4) and (AMP-binding or A-OX))

#### Legend:

core biosynthetic genes

additional biosynthetic genes

transport-related genes

regulatory genes

other genes

resistance

reset view

zoom to selection

Gene details

Shows details of the most recently selected gene, including names, products, location, and other annotations.

Select a gene to view the details available for it

Gene overview

NRPS/PKS domains

KnownClusterBlast

SubClusterBlast

Gene/CDS overview

A brief tabular summary of genes/CDS features within the region.  
Filtering the table will also search biosynthetic profiles and gene function data. If enabled, the overview will then zoom to show the area covered by the filtered selection.  
Genes selected in the region drawing above will be marked in the table with an indicator to the left of the gene name.

Filter:

Automatically zoom to filtered/selected features

| Identifier | Product | Length | | Function | Sequence | | NCBI Blast | Filter details |
| --- | --- | --- | --- | --- | --- | --- | --- | --- |
|  |  | NT | AA |  | NT | AA |  |  |

No genes match the given filter

Detailed domain annotation

Shows NRPS- and PKS-related domains for each feature that contains them. Click on each domain for more information about the domain's location, consensus monomer prediction, and other details.  
A domain glossary is available here, and an explanation of the visualisation is available here.

Selected features only

Show module domains

Similar known gene clusters from MIBiG 3.1

Shows clusters from the MiBIG database that are similar to the current region. Genes marked with the same colour are interrelated. White genes have no relationship.  
Click on reference genes to show details of similarities to genes within the current region.  
Click on an accession to open that entry in the MiBIG database.

No matches found.

Similar subclusters

Shows sub-cluster units that are similar to the current region. Genes marked with the same colour are interrelated. White genes have no relationship.  
Click on reference genes to show details of similarities to genes within the current region.

No matches found.

NRPS/PKS substrates

NRPS/PKS substrate predictions

Shows the predicted substrates for each adenylation domain and acyltransferase within genes. Each gene prediction can be expanded to view detailed predictions of each domain. Each prediction can be expanded to view the predictions by tool (and, for some tools, further expanded for extra details).

**jgi.p\_Fomfom1\_1188506**: X

:   **AMP-binding (24..426)**: X

    nrpys: Ala, Gly, Val, Leu, Ile, Abu, Ival, Ser, Thr, Hpg, Dhpg, Cys, Pro, Hpr

    SVM prediction details:
    :   Predicted physicochemical class:
        :   hydrophobic-aliphatic (Ala, Gly, Val, Leu, Ile, Abu, Ival, Ser, Thr, Hpg, Dhpg, Cys, Pro, Hpr)

        Large clusters prediction:
        :   N/A

        Small clusters prediction:
        :   N/A

        Single AA prediction:
        :   N/A

    Stachelhaus prediction details:
    :   Stachelhaus sequence:
        :   ALFVWGVAVK

        Nearest Stachelhaus code(s):

        Stachelhaus code match:
        :   0% (weak)

scaffold\_1 - Region 6 - terpene

Shows the layout of the region, marking coding sequences and areas of interest. Clicking a gene will select it and show any relevant details. Clicking an area feature (e.g. a candidate cluster) will select all coding sequences within that area. Double clicking an area feature will zoom to that area. Multiple genes and area features can be selected by clicking them while holding the Ctrl key.  
More detailed help is available here.

Download region GenBank file

Download region SVG

Location: 4,715,014 - 4,736,157 nt. (total: 21,144 nt)
Show pHMM detection rules used

terpene: (Terpene\_synth or Terpene\_synth\_C or phytoene\_synt or Lycopene\_cycl or terpene\_cyclase or NapT7 or fung\_ggpps or fung\_ggpps2 or trichodiene\_synth or TRI5)

#### Legend:

core biosynthetic genes

additional biosynthetic genes

transport-related genes

regulatory genes

other genes

resistance

reset view

zoom to selection

Gene details

Shows details of the most recently selected gene, including names, products, location, and other annotations.

Select a gene to view the details available for it

Gene overview

KnownClusterBlast

SubClusterBlast

Gene/CDS overview

A brief tabular summary of genes/CDS features within the region.  
Filtering the table will also search biosynthetic profiles and gene function data. If enabled, the overview will then zoom to show the area covered by the filtered selection.  
Genes selected in the region drawing above will be marked in the table with an indicator to the left of the gene name.

Filter:

Automatically zoom to filtered/selected features

| Identifier | Product | Length | | Function | Sequence | | NCBI Blast | Filter details |
| --- | --- | --- | --- | --- | --- | --- | --- | --- |
|  |  | NT | AA |  | NT | AA |  |  |

No genes match the given filter

Similar known gene clusters from MIBiG 3.1

Shows clusters from the MiBIG database that are similar to the current region. Genes marked with the same colour are interrelated. White genes have no relationship.  
Click on reference genes to show details of similarities to genes within the current region.  
Click on an accession to open that entry in the MiBIG database.

No matches found.

Similar subclusters

Shows sub-cluster units that are similar to the current region. Genes marked with the same colour are interrelated. White genes have no relationship.  
Click on reference genes to show details of similarities to genes within the current region.

No matches found.

scaffold\_2 - Region 1 - NRPS-like

Shows the layout of the region, marking coding sequences and areas of interest. Clicking a gene will select it and show any relevant details. Clicking an area feature (e.g. a candidate cluster) will select all coding sequences within that area. Double clicking an area feature will zoom to that area. Multiple genes and area features can be selected by clicking them while holding the Ctrl key.  
More detailed help is available here.

Download region GenBank file

Download region SVG

Location: 2,085,108 - 2,129,960 nt. (total: 44,853 nt)
Show pHMM detection rules used

NRPS-like: cds((PP-binding or NAD\_binding\_4) and (AMP-binding or A-OX))

#### Legend:

core biosynthetic genes

additional biosynthetic genes

transport-related genes

regulatory genes

other genes

resistance

reset view

zoom to selection

Gene details

Shows details of the most recently selected gene, including names, products, location, and other annotations.

Select a gene to view the details available for it

Gene overview

NRPS/PKS domains

KnownClusterBlast

SubClusterBlast

NRPS/PKS modules

Gene/CDS overview

A brief tabular summary of genes/CDS features within the region.  
Filtering the table will also search biosynthetic profiles and gene function data. If enabled, the overview will then zoom to show the area covered by the filtered selection.  
Genes selected in the region drawing above will be marked in the table with an indicator to the left of the gene name.

Filter:

Automatically zoom to filtered/selected features

| Identifier | Product | Length | | Function | Sequence | | NCBI Blast | Filter details |
| --- | --- | --- | --- | --- | --- | --- | --- | --- |
|  |  | NT | AA |  | NT | AA |  |  |

No genes match the given filter

Detailed domain annotation

Shows NRPS- and PKS-related domains for each feature that contains them. Click on each domain for more information about the domain's location, consensus monomer prediction, and other details.  
A domain glossary is available here, and an explanation of the visualisation is available here.

Selected features only

Show module domains

Similar known gene clusters from MIBiG 3.1

Shows clusters from the MiBIG database that are similar to the current region. Genes marked with the same colour are interrelated. White genes have no relationship.  
Click on reference genes to show details of similarities to genes within the current region.  
Click on an accession to open that entry in the MiBIG database.

No matches found.

Similar subclusters

Shows sub-cluster units that are similar to the current region. Genes marked with the same colour are interrelated. White genes have no relationship.  
Click on reference genes to show details of similarities to genes within the current region.

No matches found.

Module view

Shows module structures for each candidate cluster in NRPS and PKS regions.   
Genes are shown in predicted order, and are only present when containing at least one complete module.   
A domain glossary is available here, and an explanation of the visualisation is available here.

Candidate 1 (2085107 - 2129960): single NRPS-like

Legend

NRPS/PKS products

NRPS/PKS substrates

Predicted core structure(s)

Shows estimated product structure and polymer for each candidate cluster in the region. To show the product, click on the expander or the candidate cluster feature drawn in the overview.

For candidate cluster 1, location 2085107 - 2129960:

Rough prediction of core scaffold based on assumed PKS/NRPS colinearity; tailoring reactions not taken into account

**Polymer prediction:**
:   (X)

  
Direct lookup in NORINE database:
strict
or
relaxed

Link to NORINE database query form

NRPS/PKS substrate predictions

Shows the predicted substrates for each adenylation domain and acyltransferase within genes. Each gene prediction can be expanded to view detailed predictions of each domain. Each prediction can be expanded to view the predictions by tool (and, for some tools, further expanded for extra details).

**jgi.p\_Fomfom1\_1364407**: X

:   **AMP-binding (1..370)**: X

    nrpys: Ala, Gly, Val, Leu, Ile, Abu, Ival, Ser, Thr, Hpg, Dhpg, Cys, Pro, Hpr

    SVM prediction details:
    :   Predicted physicochemical class:
        :   hydrophobic-aliphatic (Ala, Gly, Val, Leu, Ile, Abu, Ival, Ser, Thr, Hpg, Dhpg, Cys, Pro, Hpr)

        Large clusters prediction:
        :   N/A

        Small clusters prediction:
        :   N/A

        Single AA prediction:
        :   N/A

    Stachelhaus prediction details:
    :   Stachelhaus sequence:
        :   GGRYAASPIK

        Nearest Stachelhaus code(s):

        Stachelhaus code match:
        :   0% (weak)

scaffold\_3 - Region 1 - terpene

Shows the layout of the region, marking coding sequences and areas of interest. Clicking a gene will select it and show any relevant details. Clicking an area feature (e.g. a candidate cluster) will select all coding sequences within that area. Double clicking an area feature will zoom to that area. Multiple genes and area features can be selected by clicking them while holding the Ctrl key.  
More detailed help is available here.

Download region GenBank file

Download region SVG

Location: 566,520 - 588,565 nt. (total: 22,046 nt)
Show pHMM detection rules used

terpene: (Terpene\_synth or Terpene\_synth\_C or phytoene\_synt or Lycopene\_cycl or terpene\_cyclase or NapT7 or fung\_ggpps or fung\_ggpps2 or trichodiene\_synth or TRI5)

#### Legend:

core biosynthetic genes

additional biosynthetic genes

transport-related genes

regulatory genes

other genes

resistance

reset view

zoom to selection

Gene details

Shows details of the most recently selected gene, including names, products, location, and other annotations.

Select a gene to view the details available for it

Gene overview

KnownClusterBlast

SubClusterBlast

Gene/CDS overview

A brief tabular summary of genes/CDS features within the region.  
Filtering the table will also search biosynthetic profiles and gene function data. If enabled, the overview will then zoom to show the area covered by the filtered selection.  
Genes selected in the region drawing above will be marked in the table with an indicator to the left of the gene name.

Filter:

Automatically zoom to filtered/selected features

| Identifier | Product | Length | | Function | Sequence | | NCBI Blast | Filter details |
| --- | --- | --- | --- | --- | --- | --- | --- | --- |
|  |  | NT | AA |  | NT | AA |  |  |

No genes match the given filter

Similar known gene clusters from MIBiG 3.1

Shows clusters from the MiBIG database that are similar to the current region. Genes marked with the same colour are interrelated. White genes have no relationship.  
Click on reference genes to show details of similarities to genes within the current region.  
Click on an accession to open that entry in the MiBIG database.

No matches found.

Similar subclusters

Shows sub-cluster units that are similar to the current region. Genes marked with the same colour are interrelated. White genes have no relationship.  
Click on reference genes to show details of similarities to genes within the current region.

No matches found.

scaffold\_3 - Region 2 - terpene

Shows the layout of the region, marking coding sequences and areas of interest. Clicking a gene will select it and show any relevant details. Clicking an area feature (e.g. a candidate cluster) will select all coding sequences within that area. Double clicking an area feature will zoom to that area. Multiple genes and area features can be selected by clicking them while holding the Ctrl key.  
More detailed help is available here.

Download region GenBank file

Download region SVG

Location: 2,291,633 - 2,314,747 nt. (total: 23,115 nt)
Show pHMM detection rules used

terpene: (Terpene\_synth or Terpene\_synth\_C or phytoene\_synt or Lycopene\_cycl or terpene\_cyclase or NapT7 or fung\_ggpps or fung\_ggpps2 or trichodiene\_synth or TRI5)

#### Legend:

core biosynthetic genes

additional biosynthetic genes

transport-related genes

regulatory genes

other genes

resistance

reset view

zoom to selection

Gene details

Shows details of the most recently selected gene, including names, products, location, and other annotations.

Select a gene to view the details available for it

Gene overview

KnownClusterBlast

SubClusterBlast

Gene/CDS overview

A brief tabular summary of genes/CDS features within the region.  
Filtering the table will also search biosynthetic profiles and gene function data. If enabled, the overview will then zoom to show the area covered by the filtered selection.  
Genes selected in the region drawing above will be marked in the table with an indicator to the left of the gene name.

Filter:

Automatically zoom to filtered/selected features

| Identifier | Product | Length | | Function | Sequence | | NCBI Blast | Filter details |
| --- | --- | --- | --- | --- | --- | --- | --- | --- |
|  |  | NT | AA |  | NT | AA |  |  |

No genes match the given filter

Similar known gene clusters from MIBiG 3.1

Shows clusters from the MiBIG database that are similar to the current region. Genes marked with the same colour are interrelated. White genes have no relationship.  
Click on reference genes to show details of similarities to genes within the current region.  
Click on an accession to open that entry in the MiBIG database.

All hits

clavaric acid
Download graphic

Similar subclusters

Shows sub-cluster units that are similar to the current region. Genes marked with the same colour are interrelated. White genes have no relationship.  
Click on reference genes to show details of similarities to genes within the current region.

No matches found.

scaffold\_3 - Region 3 - terpene

Shows the layout of the region, marking coding sequences and areas of interest. Clicking a gene will select it and show any relevant details. Clicking an area feature (e.g. a candidate cluster) will select all coding sequences within that area. Double clicking an area feature will zoom to that area. Multiple genes and area features can be selected by clicking them while holding the Ctrl key.  
More detailed help is available here.

Download region GenBank file

Download region SVG

Location: 2,401,713 - 2,424,823 nt. (total: 23,111 nt)
Show pHMM detection rules used

terpene: (Terpene\_synth or Terpene\_synth\_C or phytoene\_synt or Lycopene\_cycl or terpene\_cyclase or NapT7 or fung\_ggpps or fung\_ggpps2 or trichodiene\_synth or TRI5)

#### Legend:

core biosynthetic genes

additional biosynthetic genes

transport-related genes

regulatory genes

other genes

resistance

reset view

zoom to selection

Gene details

Shows details of the most recently selected gene, including names, products, location, and other annotations.

Select a gene to view the details available for it

Gene overview

KnownClusterBlast

SubClusterBlast

Gene/CDS overview

A brief tabular summary of genes/CDS features within the region.  
Filtering the table will also search biosynthetic profiles and gene function data. If enabled, the overview will then zoom to show the area covered by the filtered selection.  
Genes selected in the region drawing above will be marked in the table with an indicator to the left of the gene name.

Filter:

Automatically zoom to filtered/selected features

| Identifier | Product | Length | | Function | Sequence | | NCBI Blast | Filter details |
| --- | --- | --- | --- | --- | --- | --- | --- | --- |
|  |  | NT | AA |  | NT | AA |  |  |

No genes match the given filter

Similar known gene clusters from MIBiG 3.1

Shows clusters from the MiBIG database that are similar to the current region. Genes marked with the same colour are interrelated. White genes have no relationship.  
Click on reference genes to show details of similarities to genes within the current region.  
Click on an accession to open that entry in the MiBIG database.

All hits

clavaric acid
Download graphic

Similar subclusters

Shows sub-cluster units that are similar to the current region. Genes marked with the same colour are interrelated. White genes have no relationship.  
Click on reference genes to show details of similarities to genes within the current region.

No matches found.

scaffold\_4 - Region 1 - fungal-RiPP-like

Shows the layout of the region, marking coding sequences and areas of interest. Clicking a gene will select it and show any relevant details. Clicking an area feature (e.g. a candidate cluster) will select all coding sequences within that area. Double clicking an area feature will zoom to that area. Multiple genes and area features can be selected by clicking them while holding the Ctrl key.  
More detailed help is available here.

Download region GenBank file

Download region SVG

Location: 750,987 - 812,290 nt. (total: 61,304 nt)
Show pHMM detection rules used

fungal-RiPP-like: DUF3328

#### Legend:

core biosynthetic genes

additional biosynthetic genes

transport-related genes

regulatory genes

other genes

resistance

reset view

zoom to selection

Gene details

Shows details of the most recently selected gene, including names, products, location, and other annotations.

Select a gene to view the details available for it

Gene overview

KnownClusterBlast

SubClusterBlast

Gene/CDS overview

A brief tabular summary of genes/CDS features within the region.  
Filtering the table will also search biosynthetic profiles and gene function data. If enabled, the overview will then zoom to show the area covered by the filtered selection.  
Genes selected in the region drawing above will be marked in the table with an indicator to the left of the gene name.

Filter:

Automatically zoom to filtered/selected features

| Identifier | Product | Length | | Function | Sequence | | NCBI Blast | Filter details |
| --- | --- | --- | --- | --- | --- | --- | --- | --- |
|  |  | NT | AA |  | NT | AA |  |  |

No genes match the given filter

Similar known gene clusters from MIBiG 3.1

Shows clusters from the MiBIG database that are similar to the current region. Genes marked with the same colour are interrelated. White genes have no relationship.  
Click on reference genes to show details of similarities to genes within the current region.  
Click on an accession to open that entry in the MiBIG database.

No matches found.

Similar subclusters

Shows sub-cluster units that are similar to the current region. Genes marked with the same colour are interrelated. White genes have no relationship.  
Click on reference genes to show details of similarities to genes within the current region.

No matches found.

scaffold\_4 - Region 2 - terpene

Shows the layout of the region, marking coding sequences and areas of interest. Clicking a gene will select it and show any relevant details. Clicking an area feature (e.g. a candidate cluster) will select all coding sequences within that area. Double clicking an area feature will zoom to that area. Multiple genes and area features can be selected by clicking them while holding the Ctrl key.  
More detailed help is available here.

Download region GenBank file

Download region SVG

Location: 1,313,548 - 1,349,437 nt. (total: 35,890 nt)
Show pHMM detection rules used

terpene: (Terpene\_synth or Terpene\_synth\_C or phytoene\_synt or Lycopene\_cycl or terpene\_cyclase or NapT7 or fung\_ggpps or fung\_ggpps2 or trichodiene\_synth or TRI5)

#### Legend:

core biosynthetic genes

additional biosynthetic genes

transport-related genes

regulatory genes

other genes

resistance

reset view

zoom to selection

Gene details

Shows details of the most recently selected gene, including names, products, location, and other annotations.

Select a gene to view the details available for it

Gene overview

KnownClusterBlast

SubClusterBlast

Gene/CDS overview

A brief tabular summary of genes/CDS features within the region.  
Filtering the table will also search biosynthetic profiles and gene function data. If enabled, the overview will then zoom to show the area covered by the filtered selection.  
Genes selected in the region drawing above will be marked in the table with an indicator to the left of the gene name.

Filter:

Automatically zoom to filtered/selected features

| Identifier | Product | Length | | Function | Sequence | | NCBI Blast | Filter details |
| --- | --- | --- | --- | --- | --- | --- | --- | --- |
|  |  | NT | AA |  | NT | AA |  |  |

No genes match the given filter

Similar known gene clusters from MIBiG 3.1

Shows clusters from the MiBIG database that are similar to the current region. Genes marked with the same colour are interrelated. White genes have no relationship.  
Click on reference genes to show details of similarities to genes within the current region.  
Click on an accession to open that entry in the MiBIG database.

All hits

(+)-δ-cadinol
Download graphic

Similar subclusters

Shows sub-cluster units that are similar to the current region. Genes marked with the same colour are interrelated. White genes have no relationship.  
Click on reference genes to show details of similarities to genes within the current region.

No matches found.

scaffold\_4 - Region 3 - terpene

Shows the layout of the region, marking coding sequences and areas of interest. Clicking a gene will select it and show any relevant details. Clicking an area feature (e.g. a candidate cluster) will select all coding sequences within that area. Double clicking an area feature will zoom to that area. Multiple genes and area features can be selected by clicking them while holding the Ctrl key.  
More detailed help is available here.

Download region GenBank file

Download region SVG

Location: 1,636,898 - 1,663,675 nt. (total: 26,778 nt)
Show pHMM detection rules used

terpene: (Terpene\_synth or Terpene\_synth\_C or phytoene\_synt or Lycopene\_cycl or terpene\_cyclase or NapT7 or fung\_ggpps or fung\_ggpps2 or trichodiene\_synth or TRI5)

#### Legend:

core biosynthetic genes

additional biosynthetic genes

transport-related genes

regulatory genes

other genes

resistance

reset view

zoom to selection

Gene details

Shows details of the most recently selected gene, including names, products, location, and other annotations.

Select a gene to view the details available for it

Gene overview

KnownClusterBlast

SubClusterBlast

Gene/CDS overview

A brief tabular summary of genes/CDS features within the region.  
Filtering the table will also search biosynthetic profiles and gene function data. If enabled, the overview will then zoom to show the area covered by the filtered selection.  
Genes selected in the region drawing above will be marked in the table with an indicator to the left of the gene name.

Filter:

Automatically zoom to filtered/selected features

| Identifier | Product | Length | | Function | Sequence | | NCBI Blast | Filter details |
| --- | --- | --- | --- | --- | --- | --- | --- | --- |
|  |  | NT | AA |  | NT | AA |  |  |

No genes match the given filter

Similar known gene clusters from MIBiG 3.1

Shows clusters from the MiBIG database that are similar to the current region. Genes marked with the same colour are interrelated. White genes have no relationship.  
Click on reference genes to show details of similarities to genes within the current region.  
Click on an accession to open that entry in the MiBIG database.

No matches found.

Similar subclusters

Shows sub-cluster units that are similar to the current region. Genes marked with the same colour are interrelated. White genes have no relationship.  
Click on reference genes to show details of similarities to genes within the current region.

No matches found.

scaffold\_4 - Region 4 - T1PKS

Shows the layout of the region, marking coding sequences and areas of interest. Clicking a gene will select it and show any relevant details. Clicking an area feature (e.g. a candidate cluster) will select all coding sequences within that area. Double clicking an area feature will zoom to that area. Multiple genes and area features can be selected by clicking them while holding the Ctrl key.  
More detailed help is available here.

Download region GenBank file

Download region SVG

Location: 2,445,951 - 2,494,240 nt. (total: 48,290 nt)
Show pHMM detection rules used

T1PKS: cds(PKS\_AT and (PKS\_KS or ene\_KS or mod\_KS or hyb\_KS or itr\_KS or tra\_KS))

#### Legend:

core biosynthetic genes

additional biosynthetic genes

transport-related genes

regulatory genes

other genes

resistance

reset view

zoom to selection

Gene details

Shows details of the most recently selected gene, including names, products, location, and other annotations.

Select a gene to view the details available for it

Gene overview

NRPS/PKS domains

KnownClusterBlast

SubClusterBlast

NRPS/PKS modules

Gene/CDS overview

A brief tabular summary of genes/CDS features within the region.  
Filtering the table will also search biosynthetic profiles and gene function data. If enabled, the overview will then zoom to show the area covered by the filtered selection.  
Genes selected in the region drawing above will be marked in the table with an indicator to the left of the gene name.

Filter:

Automatically zoom to filtered/selected features

| Identifier | Product | Length | | Function | Sequence | | NCBI Blast | Filter details |
| --- | --- | --- | --- | --- | --- | --- | --- | --- |
|  |  | NT | AA |  | NT | AA |  |  |

No genes match the given filter

Detailed domain annotation

Shows NRPS- and PKS-related domains for each feature that contains them. Click on each domain for more information about the domain's location, consensus monomer prediction, and other details.  
A domain glossary is available here, and an explanation of the visualisation is available here.

Selected features only

Show module domains

Similar known gene clusters from MIBiG 3.1

Shows clusters from the MiBIG database that are similar to the current region. Genes marked with the same colour are interrelated. White genes have no relationship.  
Click on reference genes to show details of similarities to genes within the current region.  
Click on an accession to open that entry in the MiBIG database.

No matches found.

Similar subclusters

Shows sub-cluster units that are similar to the current region. Genes marked with the same colour are interrelated. White genes have no relationship.  
Click on reference genes to show details of similarities to genes within the current region.

No matches found.

Module view

Shows module structures for each candidate cluster in NRPS and PKS regions.   
Genes are shown in predicted order, and are only present when containing at least one complete module.   
A domain glossary is available here, and an explanation of the visualisation is available here.

Candidate 4 (2445950 - 2494240): single T1PKS

Legend

NRPS/PKS products

NRPS/PKS substrates

Predicted core structure(s)

Shows estimated product structure and polymer for each candidate cluster in the region. To show the product, click on the expander or the candidate cluster feature drawn in the overview.

For candidate cluster 4, location 2445950 - 2494240:

Rough prediction of core scaffold based on assumed PKS/NRPS colinearity; tailoring reactions not taken into account

**Polymer prediction:**
:   (pk)

  
Direct lookup in NORINE database:
strict
or
relaxed

Link to NORINE database query form

NRPS/PKS substrate predictions

Shows the predicted substrates for each adenylation domain and acyltransferase within genes. Each gene prediction can be expanded to view detailed predictions of each domain. Each prediction can be expanded to view the predictions by tool (and, for some tools, further expanded for extra details).

**jgi.p\_Fomfom1\_1315837**: pk

:   **PKS\_AT (526..839)**: pk

    ATSignature: Malonyl-CoA

    Top 3 matches:
    :   Malonyl-CoA: 62.5%
    :   Methylmalonyl-CoA: 54.2%

      
    minowa: Methylmalonyl-CoA

    Prediction, score:
    :   Methylmalonyl-CoA: 43.3


        Malonyl-CoA: 40.7


        Methoxymalonyl-CoA: 37.4


        Ethylmalonyl-CoA: 26.8


        Propionyl-CoA: 25.5


        Isobutyryl-CoA: 23.5


        fatty\_acid: 15.4


        2-Methylbutyryl-CoA: 15.1


        inactive: 12.2


        trans-1,2-CPDA: 10.4


        Benzoyl-CoA: 8.0


        CHC-CoA: 3.2


        Acetyl-CoA: 0.0


        3-Methylbutyryl-CoA: 0.0

  
**jgi.p\_Fomfom1\_1256193**: X

:   **AMP-binding (47..457)**: X

    nrpys: Ala, Gly, Val, Leu, Ile, Abu, Ival, Ser, Thr, Hpg, Dhpg, Cys, Pro, Hpr

    SVM prediction details:
    :   Predicted physicochemical class:
        :   hydrophobic-aliphatic (Ala, Gly, Val, Leu, Ile, Abu, Ival, Ser, Thr, Hpg, Dhpg, Cys, Pro, Hpr)

        Large clusters prediction:
        :   N/A

        Small clusters prediction:
        :   N/A

        Single AA prediction:
        :   N/A

    Stachelhaus prediction details:
    :   Stachelhaus sequence:
        :   GLVFGGGSIK

        Nearest Stachelhaus code(s):

        Stachelhaus code match:
        :   0% (weak)

scaffold\_6 - Region 1 - NRPS-like

Shows the layout of the region, marking coding sequences and areas of interest. Clicking a gene will select it and show any relevant details. Clicking an area feature (e.g. a candidate cluster) will select all coding sequences within that area. Double clicking an area feature will zoom to that area. Multiple genes and area features can be selected by clicking them while holding the Ctrl key.  
More detailed help is available here.

Download region GenBank file

Download region SVG

Location: 2,225,160 - 2,269,419 nt. (total: 44,260 nt)
Show pHMM detection rules used

NRPS-like: cds((PP-binding or NAD\_binding\_4) and (AMP-binding or A-OX))

#### Legend:

core biosynthetic genes

additional biosynthetic genes

transport-related genes

regulatory genes

other genes

resistance

reset view

zoom to selection

Gene details

Shows details of the most recently selected gene, including names, products, location, and other annotations.

Select a gene to view the details available for it

Gene overview

NRPS/PKS domains

KnownClusterBlast

SubClusterBlast

Gene/CDS overview

A brief tabular summary of genes/CDS features within the region.  
Filtering the table will also search biosynthetic profiles and gene function data. If enabled, the overview will then zoom to show the area covered by the filtered selection.  
Genes selected in the region drawing above will be marked in the table with an indicator to the left of the gene name.

Filter:

Automatically zoom to filtered/selected features

| Identifier | Product | Length | | Function | Sequence | | NCBI Blast | Filter details |
| --- | --- | --- | --- | --- | --- | --- | --- | --- |
|  |  | NT | AA |  | NT | AA |  |  |

No genes match the given filter

Detailed domain annotation

Shows NRPS- and PKS-related domains for each feature that contains them. Click on each domain for more information about the domain's location, consensus monomer prediction, and other details.  
A domain glossary is available here, and an explanation of the visualisation is available here.

Selected features only

Show module domains

Similar known gene clusters from MIBiG 3.1

Shows clusters from the MiBIG database that are similar to the current region. Genes marked with the same colour are interrelated. White genes have no relationship.  
Click on reference genes to show details of similarities to genes within the current region.  
Click on an accession to open that entry in the MiBIG database.

No matches found.

Similar subclusters

Shows sub-cluster units that are similar to the current region. Genes marked with the same colour are interrelated. White genes have no relationship.  
Click on reference genes to show details of similarities to genes within the current region.

No matches found.

NRPS/PKS substrates

NRPS/PKS substrate predictions

Shows the predicted substrates for each adenylation domain and acyltransferase within genes. Each gene prediction can be expanded to view detailed predictions of each domain. Each prediction can be expanded to view the predictions by tool (and, for some tools, further expanded for extra details).

**jgi.p\_Fomfom1\_1269532**: X

:   **AMP-binding (29..430)**: X

    nrpys: Gly, Ala, Val, Leu, Ile, Abu, Ival

    SVM prediction details:
    :   Predicted physicochemical class:
        :   hydrophobic-aliphatic (Ala, Gly, Val, Leu, Ile, Abu, Ival, Ser, Thr, Hpg, Dhpg, Cys, Pro, Hpr)

        Large clusters prediction:
        :   Apolar, aliphatic (Gly, Ala, Val, Leu, Ile, Abu, Ival)

        Small clusters prediction:
        :   N/A

        Single AA prediction:
        :   N/A

    Stachelhaus prediction details:
    :   Stachelhaus sequence:
        :   AIIMIGLAIK

        Nearest Stachelhaus code(s):
        :   Gly NIIMISGGIK (29% 8Å match)

        Stachelhaus code match:
        :   60% (weak)

scaffold\_7 - Region 1 - T1PKS

Shows the layout of the region, marking coding sequences and areas of interest. Clicking a gene will select it and show any relevant details. Clicking an area feature (e.g. a candidate cluster) will select all coding sequences within that area. Double clicking an area feature will zoom to that area. Multiple genes and area features can be selected by clicking them while holding the Ctrl key.  
More detailed help is available here.

Download region GenBank file

Download region SVG

Location: 109,106 - 154,660 nt. (total: 45,555 nt)
Show pHMM detection rules used

T1PKS: cds(PKS\_AT and (PKS\_KS or ene\_KS or mod\_KS or hyb\_KS or itr\_KS or tra\_KS))

#### Legend:

core biosynthetic genes

additional biosynthetic genes

transport-related genes

regulatory genes

other genes

resistance

reset view

zoom to selection

Gene details

Shows details of the most recently selected gene, including names, products, location, and other annotations.

Select a gene to view the details available for it

Gene overview

NRPS/PKS domains

KnownClusterBlast

SubClusterBlast

NRPS/PKS modules

Gene/CDS overview

A brief tabular summary of genes/CDS features within the region.  
Filtering the table will also search biosynthetic profiles and gene function data. If enabled, the overview will then zoom to show the area covered by the filtered selection.  
Genes selected in the region drawing above will be marked in the table with an indicator to the left of the gene name.

Filter:

Automatically zoom to filtered/selected features

| Identifier | Product | Length | | Function | Sequence | | NCBI Blast | Filter details |
| --- | --- | --- | --- | --- | --- | --- | --- | --- |
|  |  | NT | AA |  | NT | AA |  |  |

No genes match the given filter

Detailed domain annotation

Shows NRPS- and PKS-related domains for each feature that contains them. Click on each domain for more information about the domain's location, consensus monomer prediction, and other details.  
A domain glossary is available here, and an explanation of the visualisation is available here.

Selected features only

Show module domains

Similar known gene clusters from MIBiG 3.1

Shows clusters from the MiBIG database that are similar to the current region. Genes marked with the same colour are interrelated. White genes have no relationship.  
Click on reference genes to show details of similarities to genes within the current region.  
Click on an accession to open that entry in the MiBIG database.

No matches found.

Similar subclusters

Shows sub-cluster units that are similar to the current region. Genes marked with the same colour are interrelated. White genes have no relationship.  
Click on reference genes to show details of similarities to genes within the current region.

No matches found.

Module view

Shows module structures for each candidate cluster in NRPS and PKS regions.   
Genes are shown in predicted order, and are only present when containing at least one complete module.   
A domain glossary is available here, and an explanation of the visualisation is available here.

Candidate 1 (109105 - 154660): single T1PKS

Legend

NRPS/PKS products

NRPS/PKS substrates

Predicted core structure(s)

Shows estimated product structure and polymer for each candidate cluster in the region. To show the product, click on the expander or the candidate cluster feature drawn in the overview.

For candidate cluster 1, location 109105 - 154660:

Rough prediction of core scaffold based on assumed PKS/NRPS colinearity; tailoring reactions not taken into account

**Polymer prediction:**
:   (mal)

  
Direct lookup in NORINE database:
strict
or
relaxed

Link to NORINE database query form

NRPS/PKS substrate predictions

Shows the predicted substrates for each adenylation domain and acyltransferase within genes. Each gene prediction can be expanded to view detailed predictions of each domain. Each prediction can be expanded to view the predictions by tool (and, for some tools, further expanded for extra details).

**jgi.p\_Fomfom1\_1203106**: mal

:   **PKS\_AT (525..818)**: mal

    ATSignature: Malonyl-CoA

    Top 3 matches:
    :   Malonyl-CoA: 66.7%
    :   inactive: 62.5%
    :   2-Rhyd-Malonyl-CoA: 58.3%

      
    minowa: Malonyl-CoA

    Prediction, score:
    :   Malonyl-CoA: 118.0


        inactive: 87.9


        Methoxymalonyl-CoA: 67.1


        Methylmalonyl-CoA: 65.9


        Propionyl-CoA: 32.3


        Ethylmalonyl-CoA: 30.2


        fatty\_acid: 28.4


        Isobutyryl-CoA: 27.8


        CHC-CoA: 26.1


        Benzoyl-CoA: 24.2


        2-Methylbutyryl-CoA: 20.8


        trans-1,2-CPDA: 13.8


        Acetyl-CoA: 13.7


        3-Methylbutyryl-CoA: 9.7

  
**jgi.p\_Fomfom1\_1369104**: X

:   **AMP-binding (848..1299)**: X

    nrpys: (unknown)

    SVM prediction details:
    :   **NOTE: uncertain match**  

        Predicted physicochemical class:
        :   N/A

        Large clusters prediction:
        :   N/A

        Small clusters prediction:
        :   N/A

        Single AA prediction:
        :   N/A

    Stachelhaus prediction details:
    :   Stachelhaus sequence:
        :   ERES-----K

        Nearest Stachelhaus code(s):

        Stachelhaus code match:
        :   0% (weak)

scaffold\_7 - Region 2 - terpene

Shows the layout of the region, marking coding sequences and areas of interest. Clicking a gene will select it and show any relevant details. Clicking an area feature (e.g. a candidate cluster) will select all coding sequences within that area. Double clicking an area feature will zoom to that area. Multiple genes and area features can be selected by clicking them while holding the Ctrl key.  
More detailed help is available here.

Download region GenBank file

Download region SVG

Location: 1,383,557 - 1,405,009 nt. (total: 21,453 nt)
Show pHMM detection rules used

terpene: (Terpene\_synth or Terpene\_synth\_C or phytoene\_synt or Lycopene\_cycl or terpene\_cyclase or NapT7 or fung\_ggpps or fung\_ggpps2 or trichodiene\_synth or TRI5)

#### Legend:

core biosynthetic genes

additional biosynthetic genes

transport-related genes

regulatory genes

other genes

resistance

reset view

zoom to selection

Gene details

Shows details of the most recently selected gene, including names, products, location, and other annotations.

Select a gene to view the details available for it

Gene overview

KnownClusterBlast

SubClusterBlast

Gene/CDS overview

A brief tabular summary of genes/CDS features within the region.  
Filtering the table will also search biosynthetic profiles and gene function data. If enabled, the overview will then zoom to show the area covered by the filtered selection.  
Genes selected in the region drawing above will be marked in the table with an indicator to the left of the gene name.

Filter:

Automatically zoom to filtered/selected features

| Identifier | Product | Length | | Function | Sequence | | NCBI Blast | Filter details |
| --- | --- | --- | --- | --- | --- | --- | --- | --- |
|  |  | NT | AA |  | NT | AA |  |  |

No genes match the given filter

Similar known gene clusters from MIBiG 3.1

Shows clusters from the MiBIG database that are similar to the current region. Genes marked with the same colour are interrelated. White genes have no relationship.  
Click on reference genes to show details of similarities to genes within the current region.  
Click on an accession to open that entry in the MiBIG database.

No matches found.

Similar subclusters

Shows sub-cluster units that are similar to the current region. Genes marked with the same colour are interrelated. White genes have no relationship.  
Click on reference genes to show details of similarities to genes within the current region.

No matches found.

scaffold\_7 - Region 3 - terpene

Shows the layout of the region, marking coding sequences and areas of interest. Clicking a gene will select it and show any relevant details. Clicking an area feature (e.g. a candidate cluster) will select all coding sequences within that area. Double clicking an area feature will zoom to that area. Multiple genes and area features can be selected by clicking them while holding the Ctrl key.  
More detailed help is available here.

Download region GenBank file

Download region SVG

Location: 1,939,765 - 1,961,203 nt. (total: 21,439 nt)
Show pHMM detection rules used

terpene: (Terpene\_synth or Terpene\_synth\_C or phytoene\_synt or Lycopene\_cycl or terpene\_cyclase or NapT7 or fung\_ggpps or fung\_ggpps2 or trichodiene\_synth or TRI5)

#### Legend:

core biosynthetic genes

additional biosynthetic genes

transport-related genes

regulatory genes

other genes

resistance

reset view

zoom to selection

Gene details

Shows details of the most recently selected gene, including names, products, location, and other annotations.

Select a gene to view the details available for it

Gene overview

KnownClusterBlast

SubClusterBlast

Gene/CDS overview

A brief tabular summary of genes/CDS features within the region.  
Filtering the table will also search biosynthetic profiles and gene function data. If enabled, the overview will then zoom to show the area covered by the filtered selection.  
Genes selected in the region drawing above will be marked in the table with an indicator to the left of the gene name.

Filter:

Automatically zoom to filtered/selected features

| Identifier | Product | Length | | Function | Sequence | | NCBI Blast | Filter details |
| --- | --- | --- | --- | --- | --- | --- | --- | --- |
|  |  | NT | AA |  | NT | AA |  |  |

No genes match the given filter

Similar known gene clusters from MIBiG 3.1

Shows clusters from the MiBIG database that are similar to the current region. Genes marked with the same colour are interrelated. White genes have no relationship.  
Click on reference genes to show details of similarities to genes within the current region.  
Click on an accession to open that entry in the MiBIG database.

No matches found.

Similar subclusters

Shows sub-cluster units that are similar to the current region. Genes marked with the same colour are interrelated. White genes have no relationship.  
Click on reference genes to show details of similarities to genes within the current region.

No matches found.

scaffold\_7 - Region 4 - terpene

Shows the layout of the region, marking coding sequences and areas of interest. Clicking a gene will select it and show any relevant details. Clicking an area feature (e.g. a candidate cluster) will select all coding sequences within that area. Double clicking an area feature will zoom to that area. Multiple genes and area features can be selected by clicking them while holding the Ctrl key.  
More detailed help is available here.

Download region GenBank file

Download region SVG

Location: 1,979,400 - 2,007,923 nt. (total: 28,524 nt)
Show pHMM detection rules used

terpene: (Terpene\_synth or Terpene\_synth\_C or phytoene\_synt or Lycopene\_cycl or terpene\_cyclase or NapT7 or fung\_ggpps or fung\_ggpps2 or trichodiene\_synth or TRI5)

#### Legend:

core biosynthetic genes

additional biosynthetic genes

transport-related genes

regulatory genes

other genes

resistance

reset view

zoom to selection

Gene details

Shows details of the most recently selected gene, including names, products, location, and other annotations.

Select a gene to view the details available for it

Gene overview

KnownClusterBlast

SubClusterBlast

Gene/CDS overview

A brief tabular summary of genes/CDS features within the region.  
Filtering the table will also search biosynthetic profiles and gene function data. If enabled, the overview will then zoom to show the area covered by the filtered selection.  
Genes selected in the region drawing above will be marked in the table with an indicator to the left of the gene name.

Filter:

Automatically zoom to filtered/selected features

| Identifier | Product | Length | | Function | Sequence | | NCBI Blast | Filter details |
| --- | --- | --- | --- | --- | --- | --- | --- | --- |
|  |  | NT | AA |  | NT | AA |  |  |

No genes match the given filter

Similar known gene clusters from MIBiG 3.1

Shows clusters from the MiBIG database that are similar to the current region. Genes marked with the same colour are interrelated. White genes have no relationship.  
Click on reference genes to show details of similarities to genes within the current region.  
Click on an accession to open that entry in the MiBIG database.

No matches found.

Similar subclusters

Shows sub-cluster units that are similar to the current region. Genes marked with the same colour are interrelated. White genes have no relationship.  
Click on reference genes to show details of similarities to genes within the current region.

No matches found.

scaffold\_8 - Region 1 - terpene

Shows the layout of the region, marking coding sequences and areas of interest. Clicking a gene will select it and show any relevant details. Clicking an area feature (e.g. a candidate cluster) will select all coding sequences within that area. Double clicking an area feature will zoom to that area. Multiple genes and area features can be selected by clicking them while holding the Ctrl key.  
More detailed help is available here.

Download region GenBank file

Download region SVG

Location: 470,272 - 503,064 nt. (total: 32,793 nt)
Show pHMM detection rules used

terpene: (Terpene\_synth or Terpene\_synth\_C or phytoene\_synt or Lycopene\_cycl or terpene\_cyclase or NapT7 or fung\_ggpps or fung\_ggpps2 or trichodiene\_synth or TRI5)

#### Legend:

core biosynthetic genes

additional biosynthetic genes

transport-related genes

regulatory genes

other genes

resistance

reset view

zoom to selection

Gene details

Shows details of the most recently selected gene, including names, products, location, and other annotations.

Select a gene to view the details available for it

Gene overview

KnownClusterBlast

SubClusterBlast

Gene/CDS overview

A brief tabular summary of genes/CDS features within the region.  
Filtering the table will also search biosynthetic profiles and gene function data. If enabled, the overview will then zoom to show the area covered by the filtered selection.  
Genes selected in the region drawing above will be marked in the table with an indicator to the left of the gene name.

Filter:

Automatically zoom to filtered/selected features

| Identifier | Product | Length | | Function | Sequence | | NCBI Blast | Filter details |
| --- | --- | --- | --- | --- | --- | --- | --- | --- |
|  |  | NT | AA |  | NT | AA |  |  |

No genes match the given filter

Similar known gene clusters from MIBiG 3.1

Shows clusters from the MiBIG database that are similar to the current region. Genes marked with the same colour are interrelated. White genes have no relationship.  
Click on reference genes to show details of similarities to genes within the current region.  
Click on an accession to open that entry in the MiBIG database.

No matches found.

Similar subclusters

Shows sub-cluster units that are similar to the current region. Genes marked with the same colour are interrelated. White genes have no relationship.  
Click on reference genes to show details of similarities to genes within the current region.

No matches found.

scaffold\_9 - Region 1 - T1PKS

Shows the layout of the region, marking coding sequences and areas of interest. Clicking a gene will select it and show any relevant details. Clicking an area feature (e.g. a candidate cluster) will select all coding sequences within that area. Double clicking an area feature will zoom to that area. Multiple genes and area features can be selected by clicking them while holding the Ctrl key.  
More detailed help is available here.

Download region GenBank file

Download region SVG

Location: 339,306 - 385,036 nt. (total: 45,731 nt)
Show pHMM detection rules used

T1PKS: cds(PKS\_AT and (PKS\_KS or ene\_KS or mod\_KS or hyb\_KS or itr\_KS or tra\_KS))

#### Legend:

core biosynthetic genes

additional biosynthetic genes

transport-related genes

regulatory genes

other genes

resistance

reset view

zoom to selection

Gene details

Shows details of the most recently selected gene, including names, products, location, and other annotations.

Select a gene to view the details available for it

Gene overview

NRPS/PKS domains

KnownClusterBlast

SubClusterBlast

NRPS/PKS modules

Gene/CDS overview

A brief tabular summary of genes/CDS features within the region.  
Filtering the table will also search biosynthetic profiles and gene function data. If enabled, the overview will then zoom to show the area covered by the filtered selection.  
Genes selected in the region drawing above will be marked in the table with an indicator to the left of the gene name.

Filter:

Automatically zoom to filtered/selected features

| Identifier | Product | Length | | Function | Sequence | | NCBI Blast | Filter details |
| --- | --- | --- | --- | --- | --- | --- | --- | --- |
|  |  | NT | AA |  | NT | AA |  |  |

No genes match the given filter

Detailed domain annotation

Shows NRPS- and PKS-related domains for each feature that contains them. Click on each domain for more information about the domain's location, consensus monomer prediction, and other details.  
A domain glossary is available here, and an explanation of the visualisation is available here.

Selected features only

Show module domains

Similar known gene clusters from MIBiG 3.1

Shows clusters from the MiBIG database that are similar to the current region. Genes marked with the same colour are interrelated. White genes have no relationship.  
Click on reference genes to show details of similarities to genes within the current region.  
Click on an accession to open that entry in the MiBIG database.

No matches found.

Similar subclusters

Shows sub-cluster units that are similar to the current region. Genes marked with the same colour are interrelated. White genes have no relationship.  
Click on reference genes to show details of similarities to genes within the current region.

No matches found.

Module view

Shows module structures for each candidate cluster in NRPS and PKS regions.   
Genes are shown in predicted order, and are only present when containing at least one complete module.   
A domain glossary is available here, and an explanation of the visualisation is available here.

Candidate 1 (339305 - 385036): single T1PKS

Legend

NRPS/PKS products

NRPS/PKS substrates

Predicted core structure(s)

Shows estimated product structure and polymer for each candidate cluster in the region. To show the product, click on the expander or the candidate cluster feature drawn in the overview.

For candidate cluster 1, location 339305 - 385036:

Rough prediction of core scaffold based on assumed PKS/NRPS colinearity; tailoring reactions not taken into account

**Polymer prediction:**
:   (mal)

  
Direct lookup in NORINE database:
strict
or
relaxed

Link to NORINE database query form

NRPS/PKS substrate predictions

Shows the predicted substrates for each adenylation domain and acyltransferase within genes. Each gene prediction can be expanded to view detailed predictions of each domain. Each prediction can be expanded to view the predictions by tool (and, for some tools, further expanded for extra details).

**jgi.p\_Fomfom1\_1205222**: mal

:   **PKS\_AT (556..860)**: mal

    ATSignature: Malonyl-CoA

    Top 3 matches:
    :   Malonyl-CoA: 58.3%

      
    minowa: Malonyl-CoA

    Prediction, score:
    :   Malonyl-CoA: 106.3


        Methoxymalonyl-CoA: 72.8


        inactive: 72.3


        Methylmalonyl-CoA: 71.2


        Isobutyryl-CoA: 45.9


        Ethylmalonyl-CoA: 39.7


        Propionyl-CoA: 35.8


        Benzoyl-CoA: 34.5


        2-Methylbutyryl-CoA: 24.2


        CHC-CoA: 23.8


        Acetyl-CoA: 19.6


        trans-1,2-CPDA: 18.2


        fatty\_acid: 16.8


        3-Methylbutyryl-CoA: 0.0

scaffold\_9 - Region 2 - NRPS-like

Shows the layout of the region, marking coding sequences and areas of interest. Clicking a gene will select it and show any relevant details. Clicking an area feature (e.g. a candidate cluster) will select all coding sequences within that area. Double clicking an area feature will zoom to that area. Multiple genes and area features can be selected by clicking them while holding the Ctrl key.  
More detailed help is available here.

Download region GenBank file

Download region SVG

Location: 1,972,441 - 2,016,999 nt. (total: 44,559 nt)
Show pHMM detection rules used

NRPS-like: cds((PP-binding or NAD\_binding\_4) and (AMP-binding or A-OX))

#### Legend:

core biosynthetic genes

additional biosynthetic genes

transport-related genes

regulatory genes

other genes

resistance

reset view

zoom to selection

Gene details

Shows details of the most recently selected gene, including names, products, location, and other annotations.

Select a gene to view the details available for it

Gene overview

NRPS/PKS domains

KnownClusterBlast

SubClusterBlast

NRPS/PKS modules

Gene/CDS overview

A brief tabular summary of genes/CDS features within the region.  
Filtering the table will also search biosynthetic profiles and gene function data. If enabled, the overview will then zoom to show the area covered by the filtered selection.  
Genes selected in the region drawing above will be marked in the table with an indicator to the left of the gene name.

Filter:

Automatically zoom to filtered/selected features

| Identifier | Product | Length | | Function | Sequence | | NCBI Blast | Filter details |
| --- | --- | --- | --- | --- | --- | --- | --- | --- |
|  |  | NT | AA |  | NT | AA |  |  |

No genes match the given filter

Detailed domain annotation

Shows NRPS- and PKS-related domains for each feature that contains them. Click on each domain for more information about the domain's location, consensus monomer prediction, and other details.  
A domain glossary is available here, and an explanation of the visualisation is available here.

Selected features only

Show module domains

Similar known gene clusters from MIBiG 3.1

Shows clusters from the MiBIG database that are similar to the current region. Genes marked with the same colour are interrelated. White genes have no relationship.  
Click on reference genes to show details of similarities to genes within the current region.  
Click on an accession to open that entry in the MiBIG database.

No matches found.

Similar subclusters

Shows sub-cluster units that are similar to the current region. Genes marked with the same colour are interrelated. White genes have no relationship.  
Click on reference genes to show details of similarities to genes within the current region.

No matches found.

Module view

Shows module structures for each candidate cluster in NRPS and PKS regions.   
Genes are shown in predicted order, and are only present when containing at least one complete module.   
A domain glossary is available here, and an explanation of the visualisation is available here.

Candidate 2 (1972440 - 2016999): single NRPS-like

Legend

NRPS/PKS products

NRPS/PKS substrates

Predicted core structure(s)

Shows estimated product structure and polymer for each candidate cluster in the region. To show the product, click on the expander or the candidate cluster feature drawn in the overview.

For candidate cluster 2, location 1972440 - 2016999:

Rough prediction of core scaffold based on assumed PKS/NRPS colinearity; tailoring reactions not taken into account

**Polymer prediction:**
:   (Aad)

  
Direct lookup in NORINE database:
strict
or
relaxed

Link to NORINE database query form

NRPS/PKS substrate predictions

Shows the predicted substrates for each adenylation domain and acyltransferase within genes. Each gene prediction can be expanded to view detailed predictions of each domain. Each prediction can be expanded to view the predictions by tool (and, for some tools, further expanded for extra details).

**jgi.p\_Fomfom1\_1287664**: X

:   **AMP-binding (96..528)**: X

    nrpys: Ala, Gly, Val, Leu, Ile, Abu, Ival, Ser, Thr, Hpg, Dhpg, Cys, Pro, Hpr

    SVM prediction details:
    :   Predicted physicochemical class:
        :   hydrophobic-aliphatic (Ala, Gly, Val, Leu, Ile, Abu, Ival, Ser, Thr, Hpg, Dhpg, Cys, Pro, Hpr)

        Large clusters prediction:
        :   N/A

        Small clusters prediction:
        :   N/A

        Single AA prediction:
        :   N/A

    Stachelhaus prediction details:
    :   Stachelhaus sequence:
        :   GLGHIAAPVK

        Nearest Stachelhaus code(s):

        Stachelhaus code match:
        :   0% (weak)

  
**jgi.p\_Fomfom1\_1205933**: Aad

:   Search NORINE for peptide:
    strict
    or
    relaxed
  
:   **AMP-binding (282..755)**: Aad

    nrpys: Aad

    SVM prediction details:
    :   Predicted physicochemical class:
        :   hydrophobic-aliphatic (Ala, Gly, Val, Leu, Ile, Abu, Ival, Ser, Thr, Hpg, Dhpg, Cys, Pro, Hpr)

        Large clusters prediction:
        :   Aliphatic chain with H-bond donor (Asp, Asn, Glu, Gln, Aad)

        Small clusters prediction:
        :   N/A

        Single AA prediction:
        :   N/A

    Stachelhaus prediction details:
    :   Stachelhaus sequence:
        :   DPRHFVMRAK

        Nearest Stachelhaus code(s):
        :   Aad DPRHFVMRAK (97% 8Å match)

        Stachelhaus code match:
        :   100% (strong)

scaffold\_10 - Region 1 - NRPS-like

Shows the layout of the region, marking coding sequences and areas of interest. Clicking a gene will select it and show any relevant details. Clicking an area feature (e.g. a candidate cluster) will select all coding sequences within that area. Double clicking an area feature will zoom to that area. Multiple genes and area features can be selected by clicking them while holding the Ctrl key.  
More detailed help is available here.

Download region GenBank file

Download region SVG

Location: 221,909 - 266,107 nt. (total: 44,199 nt)
Show pHMM detection rules used

NRPS-like: cds((PP-binding or NAD\_binding\_4) and (AMP-binding or A-OX))

#### Legend:

core biosynthetic genes

additional biosynthetic genes

transport-related genes

regulatory genes

other genes

resistance

reset view

zoom to selection

Gene details

Shows details of the most recently selected gene, including names, products, location, and other annotations.

Select a gene to view the details available for it

Gene overview

NRPS/PKS domains

KnownClusterBlast

SubClusterBlast

NRPS/PKS modules

Gene/CDS overview

A brief tabular summary of genes/CDS features within the region.  
Filtering the table will also search biosynthetic profiles and gene function data. If enabled, the overview will then zoom to show the area covered by the filtered selection.  
Genes selected in the region drawing above will be marked in the table with an indicator to the left of the gene name.

Filter:

Automatically zoom to filtered/selected features

| Identifier | Product | Length | | Function | Sequence | | NCBI Blast | Filter details |
| --- | --- | --- | --- | --- | --- | --- | --- | --- |
|  |  | NT | AA |  | NT | AA |  |  |

No genes match the given filter

Detailed domain annotation

Shows NRPS- and PKS-related domains for each feature that contains them. Click on each domain for more information about the domain's location, consensus monomer prediction, and other details.  
A domain glossary is available here, and an explanation of the visualisation is available here.

Selected features only

Show module domains

Similar known gene clusters from MIBiG 3.1

Shows clusters from the MiBIG database that are similar to the current region. Genes marked with the same colour are interrelated. White genes have no relationship.  
Click on reference genes to show details of similarities to genes within the current region.  
Click on an accession to open that entry in the MiBIG database.

No matches found.

Similar subclusters

Shows sub-cluster units that are similar to the current region. Genes marked with the same colour are interrelated. White genes have no relationship.  
Click on reference genes to show details of similarities to genes within the current region.

No matches found.

Module view

Shows module structures for each candidate cluster in NRPS and PKS regions.   
Genes are shown in predicted order, and are only present when containing at least one complete module.   
A domain glossary is available here, and an explanation of the visualisation is available here.

Candidate 1 (221908 - 266107): single NRPS-like

Legend

NRPS/PKS products

NRPS/PKS substrates

Predicted core structure(s)

Shows estimated product structure and polymer for each candidate cluster in the region. To show the product, click on the expander or the candidate cluster feature drawn in the overview.

For candidate cluster 1, location 221908 - 266107:

Rough prediction of core scaffold based on assumed PKS/NRPS colinearity; tailoring reactions not taken into account

**Polymer prediction:**
:   (X)

  
Direct lookup in NORINE database:
strict
or
relaxed

Link to NORINE database query form

NRPS/PKS substrate predictions

Shows the predicted substrates for each adenylation domain and acyltransferase within genes. Each gene prediction can be expanded to view detailed predictions of each domain. Each prediction can be expanded to view the predictions by tool (and, for some tools, further expanded for extra details).

**jgi.p\_Fomfom1\_1289136**: X

:   **AMP-binding (23..362)**: X

    nrpys: Ala, Gly, Val, Leu, Ile, Abu, Ival, Ser, Thr, Hpg, Dhpg, Cys, Pro, Hpr

    SVM prediction details:
    :   Predicted physicochemical class:
        :   hydrophobic-aliphatic (Ala, Gly, Val, Leu, Ile, Abu, Ival, Ser, Thr, Hpg, Dhpg, Cys, Pro, Hpr)

        Large clusters prediction:
        :   N/A

        Small clusters prediction:
        :   N/A

        Single AA prediction:
        :   N/A

    Stachelhaus prediction details:
    :   Stachelhaus sequence:
        :   HAMLFGWAIK

        Nearest Stachelhaus code(s):
        :   Leu DAMLVGAACK (32% 8Å match)

        Stachelhaus code match:
        :   60% (weak)

scaffold\_11 - Region 1 - T1PKS

Shows the layout of the region, marking coding sequences and areas of interest. Clicking a gene will select it and show any relevant details. Clicking an area feature (e.g. a candidate cluster) will select all coding sequences within that area. Double clicking an area feature will zoom to that area. Multiple genes and area features can be selected by clicking them while holding the Ctrl key.  
More detailed help is available here.

Download region GenBank file

Download region SVG

Location: 1 - 40,651 nt. (total: 40,651 nt)
Show pHMM detection rules used

Region on contig edge.

T1PKS: cds(PKS\_AT and (PKS\_KS or ene\_KS or mod\_KS or hyb\_KS or itr\_KS or tra\_KS))

#### Legend:

core biosynthetic genes

additional biosynthetic genes

transport-related genes

regulatory genes

other genes

resistance

reset view

zoom to selection

Gene details

Shows details of the most recently selected gene, including names, products, location, and other annotations.

Select a gene to view the details available for it

Gene overview

NRPS/PKS domains

KnownClusterBlast

SubClusterBlast

NRPS/PKS modules

Gene/CDS overview

A brief tabular summary of genes/CDS features within the region.  
Filtering the table will also search biosynthetic profiles and gene function data. If enabled, the overview will then zoom to show the area covered by the filtered selection.  
Genes selected in the region drawing above will be marked in the table with an indicator to the left of the gene name.

Filter:

Automatically zoom to filtered/selected features

| Identifier | Product | Length | | Function | Sequence | | NCBI Blast | Filter details |
| --- | --- | --- | --- | --- | --- | --- | --- | --- |
|  |  | NT | AA |  | NT | AA |  |  |

No genes match the given filter

Detailed domain annotation

Shows NRPS- and PKS-related domains for each feature that contains them. Click on each domain for more information about the domain's location, consensus monomer prediction, and other details.  
A domain glossary is available here, and an explanation of the visualisation is available here.

Selected features only

Show module domains

Similar known gene clusters from MIBiG 3.1

Shows clusters from the MiBIG database that are similar to the current region. Genes marked with the same colour are interrelated. White genes have no relationship.  
Click on reference genes to show details of similarities to genes within the current region.  
Click on an accession to open that entry in the MiBIG database.

No matches found.

Similar subclusters

Shows sub-cluster units that are similar to the current region. Genes marked with the same colour are interrelated. White genes have no relationship.  
Click on reference genes to show details of similarities to genes within the current region.

No matches found.

Module view

Shows module structures for each candidate cluster in NRPS and PKS regions.   
Genes are shown in predicted order, and are only present when containing at least one complete module.   
A domain glossary is available here, and an explanation of the visualisation is available here.

Candidate 1 (0 - 40651): single T1PKS

Legend

NRPS/PKS products

NRPS/PKS substrates

Predicted core structure(s)

Shows estimated product structure and polymer for each candidate cluster in the region. To show the product, click on the expander or the candidate cluster feature drawn in the overview.

For candidate cluster 1, location 0 - 40651:

Rough prediction of core scaffold based on assumed PKS/NRPS colinearity; tailoring reactions not taken into account

**Polymer prediction:**
:   (pk)

  
Direct lookup in NORINE database:
strict
or
relaxed

Link to NORINE database query form

NRPS/PKS substrate predictions

Shows the predicted substrates for each adenylation domain and acyltransferase within genes. Each gene prediction can be expanded to view detailed predictions of each domain. Each prediction can be expanded to view the predictions by tool (and, for some tools, further expanded for extra details).

**jgi.p\_Fomfom1\_1371971**: pk

:   **PKS\_AT (516..831)**: pk

    ATSignature: Malonyl-CoA

    Top 3 matches:
    :   Malonyl-CoA: 62.5%
    :   Methylmalonyl-CoA: 54.2%

      
    minowa: Methylmalonyl-CoA

    Prediction, score:
    :   Methylmalonyl-CoA: 50.9


        Methoxymalonyl-CoA: 48.4


        Malonyl-CoA: 40.7


        Ethylmalonyl-CoA: 34.4


        Propionyl-CoA: 30.3


        fatty\_acid: 29.4


        CHC-CoA: 21.7


        Isobutyryl-CoA: 21.0


        2-Methylbutyryl-CoA: 15.6


        trans-1,2-CPDA: 14.5


        inactive: 10.2


        3-Methylbutyryl-CoA: 1.6


        Benzoyl-CoA: 0.0


        Acetyl-CoA: 0.0

scaffold\_11 - Region 2 - terpene

Shows the layout of the region, marking coding sequences and areas of interest. Clicking a gene will select it and show any relevant details. Clicking an area feature (e.g. a candidate cluster) will select all coding sequences within that area. Double clicking an area feature will zoom to that area. Multiple genes and area features can be selected by clicking them while holding the Ctrl key.  
More detailed help is available here.

Download region GenBank file

Download region SVG

Location: 925,561 - 947,053 nt. (total: 21,493 nt)
Show pHMM detection rules used

terpene: (Terpene\_synth or Terpene\_synth\_C or phytoene\_synt or Lycopene\_cycl or terpene\_cyclase or NapT7 or fung\_ggpps or fung\_ggpps2 or trichodiene\_synth or TRI5)

#### Legend:

core biosynthetic genes

additional biosynthetic genes

transport-related genes

regulatory genes

other genes

resistance

reset view

zoom to selection

Gene details

Shows details of the most recently selected gene, including names, products, location, and other annotations.

Select a gene to view the details available for it

Gene overview

KnownClusterBlast

SubClusterBlast

Gene/CDS overview

A brief tabular summary of genes/CDS features within the region.  
Filtering the table will also search biosynthetic profiles and gene function data. If enabled, the overview will then zoom to show the area covered by the filtered selection.  
Genes selected in the region drawing above will be marked in the table with an indicator to the left of the gene name.

Filter:

Automatically zoom to filtered/selected features

| Identifier | Product | Length | | Function | Sequence | | NCBI Blast | Filter details |
| --- | --- | --- | --- | --- | --- | --- | --- | --- |
|  |  | NT | AA |  | NT | AA |  |  |

No genes match the given filter

Similar known gene clusters from MIBiG 3.1

Shows clusters from the MiBIG database that are similar to the current region. Genes marked with the same colour are interrelated. White genes have no relationship.  
Click on reference genes to show details of similarities to genes within the current region.  
Click on an accession to open that entry in the MiBIG database.

All hits

(+)-δ-cadinol
Download graphic

Similar subclusters

Shows sub-cluster units that are similar to the current region. Genes marked with the same colour are interrelated. White genes have no relationship.  
Click on reference genes to show details of similarities to genes within the current region.

No matches found.

scaffold\_11 - Region 3 - fungal-RiPP-like

Shows the layout of the region, marking coding sequences and areas of interest. Clicking a gene will select it and show any relevant details. Clicking an area feature (e.g. a candidate cluster) will select all coding sequences within that area. Double clicking an area feature will zoom to that area. Multiple genes and area features can be selected by clicking them while holding the Ctrl key.  
More detailed help is available here.

Download region GenBank file

Download region SVG

Location: 1,033,338 - 1,094,299 nt. (total: 60,962 nt)
Show pHMM detection rules used

fungal-RiPP-like: DUF3328

#### Legend:

core biosynthetic genes

additional biosynthetic genes

transport-related genes

regulatory genes

other genes

resistance

reset view

zoom to selection

Gene details

Shows details of the most recently selected gene, including names, products, location, and other annotations.

Select a gene to view the details available for it

Gene overview

KnownClusterBlast

SubClusterBlast

Gene/CDS overview

A brief tabular summary of genes/CDS features within the region.  
Filtering the table will also search biosynthetic profiles and gene function data. If enabled, the overview will then zoom to show the area covered by the filtered selection.  
Genes selected in the region drawing above will be marked in the table with an indicator to the left of the gene name.

Filter:

Automatically zoom to filtered/selected features

| Identifier | Product | Length | | Function | Sequence | | NCBI Blast | Filter details |
| --- | --- | --- | --- | --- | --- | --- | --- | --- |
|  |  | NT | AA |  | NT | AA |  |  |

No genes match the given filter

Similar known gene clusters from MIBiG 3.1

Shows clusters from the MiBIG database that are similar to the current region. Genes marked with the same colour are interrelated. White genes have no relationship.  
Click on reference genes to show details of similarities to genes within the current region.  
Click on an accession to open that entry in the MiBIG database.

No matches found.

Similar subclusters

Shows sub-cluster units that are similar to the current region. Genes marked with the same colour are interrelated. White genes have no relationship.  
Click on reference genes to show details of similarities to genes within the current region.

No matches found.

scaffold\_12 - Region 1 - NRPS-like

Shows the layout of the region, marking coding sequences and areas of interest. Clicking a gene will select it and show any relevant details. Clicking an area feature (e.g. a candidate cluster) will select all coding sequences within that area. Double clicking an area feature will zoom to that area. Multiple genes and area features can be selected by clicking them while holding the Ctrl key.  
More detailed help is available here.

Download region GenBank file

Download region SVG

Location: 256,606 - 300,912 nt. (total: 44,307 nt)
Show pHMM detection rules used

NRPS-like: cds((PP-binding or NAD\_binding\_4) and (AMP-binding or A-OX))

#### Legend:

core biosynthetic genes

additional biosynthetic genes

transport-related genes

regulatory genes

other genes

resistance

reset view

zoom to selection

Gene details

Shows details of the most recently selected gene, including names, products, location, and other annotations.

Select a gene to view the details available for it

Gene overview

NRPS/PKS domains

KnownClusterBlast

SubClusterBlast

NRPS/PKS modules

Gene/CDS overview

A brief tabular summary of genes/CDS features within the region.  
Filtering the table will also search biosynthetic profiles and gene function data. If enabled, the overview will then zoom to show the area covered by the filtered selection.  
Genes selected in the region drawing above will be marked in the table with an indicator to the left of the gene name.

Filter:

Automatically zoom to filtered/selected features

| Identifier | Product | Length | | Function | Sequence | | NCBI Blast | Filter details |
| --- | --- | --- | --- | --- | --- | --- | --- | --- |
|  |  | NT | AA |  | NT | AA |  |  |

No genes match the given filter

Detailed domain annotation

Shows NRPS- and PKS-related domains for each feature that contains them. Click on each domain for more information about the domain's location, consensus monomer prediction, and other details.  
A domain glossary is available here, and an explanation of the visualisation is available here.

Selected features only

Show module domains

Similar known gene clusters from MIBiG 3.1

Shows clusters from the MiBIG database that are similar to the current region. Genes marked with the same colour are interrelated. White genes have no relationship.  
Click on reference genes to show details of similarities to genes within the current region.  
Click on an accession to open that entry in the MiBIG database.

No matches found.

Similar subclusters

Shows sub-cluster units that are similar to the current region. Genes marked with the same colour are interrelated. White genes have no relationship.  
Click on reference genes to show details of similarities to genes within the current region.

No matches found.

Module view

Shows module structures for each candidate cluster in NRPS and PKS regions.   
Genes are shown in predicted order, and are only present when containing at least one complete module.   
A domain glossary is available here, and an explanation of the visualisation is available here.

Candidate 1 (256605 - 300912): single NRPS-like

Legend

NRPS/PKS products

NRPS/PKS substrates

Predicted core structure(s)

Shows estimated product structure and polymer for each candidate cluster in the region. To show the product, click on the expander or the candidate cluster feature drawn in the overview.

For candidate cluster 1, location 256605 - 300912:

Rough prediction of core scaffold based on assumed PKS/NRPS colinearity; tailoring reactions not taken into account

**Polymer prediction:**
:   (X)

  
Direct lookup in NORINE database:
strict
or
relaxed

Link to NORINE database query form

NRPS/PKS substrate predictions

Shows the predicted substrates for each adenylation domain and acyltransferase within genes. Each gene prediction can be expanded to view detailed predictions of each domain. Each prediction can be expanded to view the predictions by tool (and, for some tools, further expanded for extra details).

**jgi.p\_Fomfom1\_1319667**: X

:   **AMP-binding (74..461)**: X

    nrpys: Ala, Gly, Val, Leu, Ile, Abu, Ival, Ser, Thr, Hpg, Dhpg, Cys, Pro, Hpr

    SVM prediction details:
    :   Predicted physicochemical class:
        :   hydrophobic-aliphatic (Ala, Gly, Val, Leu, Ile, Abu, Ival, Ser, Thr, Hpg, Dhpg, Cys, Pro, Hpr)

        Large clusters prediction:
        :   N/A

        Small clusters prediction:
        :   N/A

        Single AA prediction:
        :   N/A

    Stachelhaus prediction details:
    :   Stachelhaus sequence:
        :   HVMYFAFAVK

        Nearest Stachelhaus code(s):

        Stachelhaus code match:
        :   0% (weak)

scaffold\_12 - Region 2 - NRPS-like

Shows the layout of the region, marking coding sequences and areas of interest. Clicking a gene will select it and show any relevant details. Clicking an area feature (e.g. a candidate cluster) will select all coding sequences within that area. Double clicking an area feature will zoom to that area. Multiple genes and area features can be selected by clicking them while holding the Ctrl key.  
More detailed help is available here.

Download region GenBank file

Download region SVG

Location: 316,272 - 389,811 nt. (total: 73,540 nt)
Show pHMM detection rules used

NRPS-like: cds((PP-binding or NAD\_binding\_4) and (AMP-binding or A-OX))

#### Legend:

core biosynthetic genes

additional biosynthetic genes

transport-related genes

regulatory genes

other genes

resistance

reset view

zoom to selection

Gene details

Shows details of the most recently selected gene, including names, products, location, and other annotations.

Select a gene to view the details available for it

Gene overview

NRPS/PKS domains

KnownClusterBlast

SubClusterBlast

NRPS/PKS modules

Gene/CDS overview

A brief tabular summary of genes/CDS features within the region.  
Filtering the table will also search biosynthetic profiles and gene function data. If enabled, the overview will then zoom to show the area covered by the filtered selection.  
Genes selected in the region drawing above will be marked in the table with an indicator to the left of the gene name.

Filter:

Automatically zoom to filtered/selected features

| Identifier | Product | Length | | Function | Sequence | | NCBI Blast | Filter details |
| --- | --- | --- | --- | --- | --- | --- | --- | --- |
|  |  | NT | AA |  | NT | AA |  |  |

No genes match the given filter

Detailed domain annotation

Shows NRPS- and PKS-related domains for each feature that contains them. Click on each domain for more information about the domain's location, consensus monomer prediction, and other details.  
A domain glossary is available here, and an explanation of the visualisation is available here.

Selected features only

Show module domains

Similar known gene clusters from MIBiG 3.1

Shows clusters from the MiBIG database that are similar to the current region. Genes marked with the same colour are interrelated. White genes have no relationship.  
Click on reference genes to show details of similarities to genes within the current region.  
Click on an accession to open that entry in the MiBIG database.

No matches found.

Similar subclusters

Shows sub-cluster units that are similar to the current region. Genes marked with the same colour are interrelated. White genes have no relationship.  
Click on reference genes to show details of similarities to genes within the current region.

No matches found.

Module view

Shows module structures for each candidate cluster in NRPS and PKS regions.   
Genes are shown in predicted order, and are only present when containing at least one complete module.   
A domain glossary is available here, and an explanation of the visualisation is available here.

Candidate 2 (316271 - 389811): neighbouring NRPS-like

Candidate 4 (345407 - 389811): single NRPS-like

Legend

NRPS/PKS products

NRPS/PKS substrates

Predicted core structure(s)

Shows estimated product structure and polymer for each candidate cluster in the region. To show the product, click on the expander or the candidate cluster feature drawn in the overview.

For candidate cluster 2, location 316271 - 389811:

Rough prediction of core scaffold based on assumed PKS/NRPS colinearity; tailoring reactions not taken into account

**Polymer prediction:**
:   (X)

  
Direct lookup in NORINE database:
strict
or
relaxed

---

For candidate cluster 4, location 345407 - 389811:

Rough prediction of core scaffold based on assumed PKS/NRPS colinearity; tailoring reactions not taken into account

**Polymer prediction:**
:   (X)

  
Direct lookup in NORINE database:
strict
or
relaxed

Link to NORINE database query form

NRPS/PKS substrate predictions

Shows the predicted substrates for each adenylation domain and acyltransferase within genes. Each gene prediction can be expanded to view detailed predictions of each domain. Each prediction can be expanded to view the predictions by tool (and, for some tools, further expanded for extra details).

**jgi.p\_Fomfom1\_1299880**: X

:   **AMP-binding (109..463)**: X

    nrpys: Gly, Ala, Val, Leu, Ile, Abu, Ival

    SVM prediction details:
    :   Predicted physicochemical class:
        :   hydrophobic-aliphatic (Ala, Gly, Val, Leu, Ile, Abu, Ival, Ser, Thr, Hpg, Dhpg, Cys, Pro, Hpr)

        Large clusters prediction:
        :   Apolar, aliphatic (Gly, Ala, Val, Leu, Ile, Abu, Ival)

        Small clusters prediction:
        :   N/A

        Single AA prediction:
        :   N/A

    Stachelhaus prediction details:
    :   Stachelhaus sequence:
        :   IMLYIAFAVK

        Nearest Stachelhaus code(s):

        Stachelhaus code match:
        :   0% (weak)

  
**jgi.p\_Fomfom1\_1319683**: X

:   **AMP-binding (79..460)**: X

    nrpys: Ala, Gly, Val, Leu, Ile, Abu, Ival, Ser, Thr, Hpg, Dhpg, Cys, Pro, Hpr

    SVM prediction details:
    :   Predicted physicochemical class:
        :   hydrophobic-aliphatic (Ala, Gly, Val, Leu, Ile, Abu, Ival, Ser, Thr, Hpg, Dhpg, Cys, Pro, Hpr)

        Large clusters prediction:
        :   N/A

        Small clusters prediction:
        :   N/A

        Single AA prediction:
        :   N/A

    Stachelhaus prediction details:
    :   Stachelhaus sequence:
        :   HVAYIAFAAK

        Nearest Stachelhaus code(s):

        Stachelhaus code match:
        :   0% (weak)

scaffold\_12 - Region 3 - NRPS-like

Shows the layout of the region, marking coding sequences and areas of interest. Clicking a gene will select it and show any relevant details. Clicking an area feature (e.g. a candidate cluster) will select all coding sequences within that area. Double clicking an area feature will zoom to that area. Multiple genes and area features can be selected by clicking them while holding the Ctrl key.  
More detailed help is available here.

Download region GenBank file

Download region SVG

Location: 405,136 - 449,531 nt. (total: 44,396 nt)
Show pHMM detection rules used

NRPS-like: cds((PP-binding or NAD\_binding\_4) and (AMP-binding or A-OX))

#### Legend:

core biosynthetic genes

additional biosynthetic genes

transport-related genes

regulatory genes

other genes

resistance

reset view

zoom to selection

Gene details

Shows details of the most recently selected gene, including names, products, location, and other annotations.

Select a gene to view the details available for it

Gene overview

NRPS/PKS domains

KnownClusterBlast

SubClusterBlast

NRPS/PKS modules

Gene/CDS overview

A brief tabular summary of genes/CDS features within the region.  
Filtering the table will also search biosynthetic profiles and gene function data. If enabled, the overview will then zoom to show the area covered by the filtered selection.  
Genes selected in the region drawing above will be marked in the table with an indicator to the left of the gene name.

Filter:

Automatically zoom to filtered/selected features

| Identifier | Product | Length | | Function | Sequence | | NCBI Blast | Filter details |
| --- | --- | --- | --- | --- | --- | --- | --- | --- |
|  |  | NT | AA |  | NT | AA |  |  |

No genes match the given filter

Detailed domain annotation

Shows NRPS- and PKS-related domains for each feature that contains them. Click on each domain for more information about the domain's location, consensus monomer prediction, and other details.  
A domain glossary is available here, and an explanation of the visualisation is available here.

Selected features only

Show module domains

Similar known gene clusters from MIBiG 3.1

Shows clusters from the MiBIG database that are similar to the current region. Genes marked with the same colour are interrelated. White genes have no relationship.  
Click on reference genes to show details of similarities to genes within the current region.  
Click on an accession to open that entry in the MiBIG database.

No matches found.

Similar subclusters

Shows sub-cluster units that are similar to the current region. Genes marked with the same colour are interrelated. White genes have no relationship.  
Click on reference genes to show details of similarities to genes within the current region.

No matches found.

Module view

Shows module structures for each candidate cluster in NRPS and PKS regions.   
Genes are shown in predicted order, and are only present when containing at least one complete module.   
A domain glossary is available here, and an explanation of the visualisation is available here.

Candidate 5 (405135 - 449531): single NRPS-like

Legend

NRPS/PKS products

NRPS/PKS substrates

Predicted core structure(s)

Shows estimated product structure and polymer for each candidate cluster in the region. To show the product, click on the expander or the candidate cluster feature drawn in the overview.

For candidate cluster 5, location 405135 - 449531:

Rough prediction of core scaffold based on assumed PKS/NRPS colinearity; tailoring reactions not taken into account

**Polymer prediction:**
:   (X)

  
Direct lookup in NORINE database:
strict
or
relaxed

Link to NORINE database query form

NRPS/PKS substrate predictions

Shows the predicted substrates for each adenylation domain and acyltransferase within genes. Each gene prediction can be expanded to view detailed predictions of each domain. Each prediction can be expanded to view the predictions by tool (and, for some tools, further expanded for extra details).

**jgi.p\_Fomfom1\_1388504**: X

:   **AMP-binding (54..460)**: X

    nrpys: Ala, Gly, Val, Leu, Ile, Abu, Ival, Ser, Thr, Hpg, Dhpg, Cys, Pro, Hpr

    SVM prediction details:
    :   Predicted physicochemical class:
        :   hydrophobic-aliphatic (Ala, Gly, Val, Leu, Ile, Abu, Ival, Ser, Thr, Hpg, Dhpg, Cys, Pro, Hpr)

        Large clusters prediction:
        :   N/A

        Small clusters prediction:
        :   N/A

        Single AA prediction:
        :   N/A

    Stachelhaus prediction details:
    :   Stachelhaus sequence:
        :   --LYIAFAAK

        Nearest Stachelhaus code(s):

        Stachelhaus code match:
        :   0% (weak)

scaffold\_13 - Region 1 - terpene

Shows the layout of the region, marking coding sequences and areas of interest. Clicking a gene will select it and show any relevant details. Clicking an area feature (e.g. a candidate cluster) will select all coding sequences within that area. Double clicking an area feature will zoom to that area. Multiple genes and area features can be selected by clicking them while holding the Ctrl key.  
More detailed help is available here.

Download region GenBank file

Download region SVG

Location: 93,968 - 115,242 nt. (total: 21,275 nt)
Show pHMM detection rules used

terpene: (Terpene\_synth or Terpene\_synth\_C or phytoene\_synt or Lycopene\_cycl or terpene\_cyclase or NapT7 or fung\_ggpps or fung\_ggpps2 or trichodiene\_synth or TRI5)

#### Legend:

core biosynthetic genes

additional biosynthetic genes

transport-related genes

regulatory genes

other genes

resistance

reset view

zoom to selection

Gene details

Shows details of the most recently selected gene, including names, products, location, and other annotations.

Select a gene to view the details available for it

Gene overview

KnownClusterBlast

SubClusterBlast

Gene/CDS overview

A brief tabular summary of genes/CDS features within the region.  
Filtering the table will also search biosynthetic profiles and gene function data. If enabled, the overview will then zoom to show the area covered by the filtered selection.  
Genes selected in the region drawing above will be marked in the table with an indicator to the left of the gene name.

Filter:

Automatically zoom to filtered/selected features

| Identifier | Product | Length | | Function | Sequence | | NCBI Blast | Filter details |
| --- | --- | --- | --- | --- | --- | --- | --- | --- |
|  |  | NT | AA |  | NT | AA |  |  |

No genes match the given filter

Similar known gene clusters from MIBiG 3.1

Shows clusters from the MiBIG database that are similar to the current region. Genes marked with the same colour are interrelated. White genes have no relationship.  
Click on reference genes to show details of similarities to genes within the current region.  
Click on an accession to open that entry in the MiBIG database.

All hits

armillyl orsellinate/8α-hydroxy-6-protoilludene

hirsutene
Download graphic

Similar subclusters

Shows sub-cluster units that are similar to the current region. Genes marked with the same colour are interrelated. White genes have no relationship.  
Click on reference genes to show details of similarities to genes within the current region.

No matches found.

scaffold\_13 - Region 2 - terpene

Shows the layout of the region, marking coding sequences and areas of interest. Clicking a gene will select it and show any relevant details. Clicking an area feature (e.g. a candidate cluster) will select all coding sequences within that area. Double clicking an area feature will zoom to that area. Multiple genes and area features can be selected by clicking them while holding the Ctrl key.  
More detailed help is available here.

Download region GenBank file

Download region SVG

Location: 137,996 - 159,259 nt. (total: 21,264 nt)
Show pHMM detection rules used

terpene: (Terpene\_synth or Terpene\_synth\_C or phytoene\_synt or Lycopene\_cycl or terpene\_cyclase or NapT7 or fung\_ggpps or fung\_ggpps2 or trichodiene\_synth or TRI5)

#### Legend:

core biosynthetic genes

additional biosynthetic genes

transport-related genes

regulatory genes

other genes

resistance

reset view

zoom to selection

Gene details

Shows details of the most recently selected gene, including names, products, location, and other annotations.

Select a gene to view the details available for it

Gene overview

KnownClusterBlast

SubClusterBlast

Gene/CDS overview

A brief tabular summary of genes/CDS features within the region.  
Filtering the table will also search biosynthetic profiles and gene function data. If enabled, the overview will then zoom to show the area covered by the filtered selection.  
Genes selected in the region drawing above will be marked in the table with an indicator to the left of the gene name.

Filter:

Automatically zoom to filtered/selected features

| Identifier | Product | Length | | Function | Sequence | | NCBI Blast | Filter details |
| --- | --- | --- | --- | --- | --- | --- | --- | --- |
|  |  | NT | AA |  | NT | AA |  |  |

No genes match the given filter

Similar known gene clusters from MIBiG 3.1

Shows clusters from the MiBIG database that are similar to the current region. Genes marked with the same colour are interrelated. White genes have no relationship.  
Click on reference genes to show details of similarities to genes within the current region.  
Click on an accession to open that entry in the MiBIG database.

No matches found.

Similar subclusters

Shows sub-cluster units that are similar to the current region. Genes marked with the same colour are interrelated. White genes have no relationship.  
Click on reference genes to show details of similarities to genes within the current region.

No matches found.

scaffold\_14 - Region 1 - terpene

Shows the layout of the region, marking coding sequences and areas of interest. Clicking a gene will select it and show any relevant details. Clicking an area feature (e.g. a candidate cluster) will select all coding sequences within that area. Double clicking an area feature will zoom to that area. Multiple genes and area features can be selected by clicking them while holding the Ctrl key.  
More detailed help is available here.

Download region GenBank file

Download region SVG

Location: 296,700 - 317,904 nt. (total: 21,205 nt)
Show pHMM detection rules used

terpene: (Terpene\_synth or Terpene\_synth\_C or phytoene\_synt or Lycopene\_cycl or terpene\_cyclase or NapT7 or fung\_ggpps or fung\_ggpps2 or trichodiene\_synth or TRI5)

#### Legend:

core biosynthetic genes

additional biosynthetic genes

transport-related genes

regulatory genes

other genes

resistance

reset view

zoom to selection

Gene details

Shows details of the most recently selected gene, including names, products, location, and other annotations.

Select a gene to view the details available for it

Gene overview

KnownClusterBlast

SubClusterBlast

Gene/CDS overview

A brief tabular summary of genes/CDS features within the region.  
Filtering the table will also search biosynthetic profiles and gene function data. If enabled, the overview will then zoom to show the area covered by the filtered selection.  
Genes selected in the region drawing above will be marked in the table with an indicator to the left of the gene name.

Filter:

Automatically zoom to filtered/selected features

| Identifier | Product | Length | | Function | Sequence | | NCBI Blast | Filter details |
| --- | --- | --- | --- | --- | --- | --- | --- | --- |
|  |  | NT | AA |  | NT | AA |  |  |

No genes match the given filter

Similar known gene clusters from MIBiG 3.1

Shows clusters from the MiBIG database that are similar to the current region. Genes marked with the same colour are interrelated. White genes have no relationship.  
Click on reference genes to show details of similarities to genes within the current region.  
Click on an accession to open that entry in the MiBIG database.

No matches found.

Similar subclusters

Shows sub-cluster units that are similar to the current region. Genes marked with the same colour are interrelated. White genes have no relationship.  
Click on reference genes to show details of similarities to genes within the current region.

No matches found.

scaffold\_14 - Region 2 - fungal-RiPP-like

Shows the layout of the region, marking coding sequences and areas of interest. Clicking a gene will select it and show any relevant details. Clicking an area feature (e.g. a candidate cluster) will select all coding sequences within that area. Double clicking an area feature will zoom to that area. Multiple genes and area features can be selected by clicking them while holding the Ctrl key.  
More detailed help is available here.

Download region GenBank file

Download region SVG

Location: 324,080 - 515,825 nt. (total: 191,746 nt)
Show pHMM detection rules used

fungal-RiPP-like: DUF3328

#### Legend:

core biosynthetic genes

additional biosynthetic genes

transport-related genes

regulatory genes

other genes

resistance

reset view

zoom to selection

Gene details

Shows details of the most recently selected gene, including names, products, location, and other annotations.

Select a gene to view the details available for it

Gene overview

KnownClusterBlast

SubClusterBlast

Gene/CDS overview

A brief tabular summary of genes/CDS features within the region.  
Filtering the table will also search biosynthetic profiles and gene function data. If enabled, the overview will then zoom to show the area covered by the filtered selection.  
Genes selected in the region drawing above will be marked in the table with an indicator to the left of the gene name.

Filter:

Automatically zoom to filtered/selected features

| Identifier | Product | Length | | Function | Sequence | | NCBI Blast | Filter details |
| --- | --- | --- | --- | --- | --- | --- | --- | --- |
|  |  | NT | AA |  | NT | AA |  |  |

No genes match the given filter

Similar known gene clusters from MIBiG 3.1

Shows clusters from the MiBIG database that are similar to the current region. Genes marked with the same colour are interrelated. White genes have no relationship.  
Click on reference genes to show details of similarities to genes within the current region.  
Click on an accession to open that entry in the MiBIG database.

No matches found.

Similar subclusters

Shows sub-cluster units that are similar to the current region. Genes marked with the same colour are interrelated. White genes have no relationship.  
Click on reference genes to show details of similarities to genes within the current region.

No matches found.

scaffold\_14 - Region 3 - NRPS-like

Shows the layout of the region, marking coding sequences and areas of interest. Clicking a gene will select it and show any relevant details. Clicking an area feature (e.g. a candidate cluster) will select all coding sequences within that area. Double clicking an area feature will zoom to that area. Multiple genes and area features can be selected by clicking them while holding the Ctrl key.  
More detailed help is available here.

Download region GenBank file

Download region SVG

Location: 829,881 - 895,923 nt. (total: 66,043 nt)
Show pHMM detection rules used

NRPS-like: cds((PP-binding or NAD\_binding\_4) and (AMP-binding or A-OX))

#### Legend:

core biosynthetic genes

additional biosynthetic genes

transport-related genes

regulatory genes

other genes

resistance

reset view

zoom to selection

Gene details

Shows details of the most recently selected gene, including names, products, location, and other annotations.

Select a gene to view the details available for it

Gene overview

NRPS/PKS domains

KnownClusterBlast

SubClusterBlast

NRPS/PKS modules

Gene/CDS overview

A brief tabular summary of genes/CDS features within the region.  
Filtering the table will also search biosynthetic profiles and gene function data. If enabled, the overview will then zoom to show the area covered by the filtered selection.  
Genes selected in the region drawing above will be marked in the table with an indicator to the left of the gene name.

Filter:

Automatically zoom to filtered/selected features

| Identifier | Product | Length | | Function | Sequence | | NCBI Blast | Filter details |
| --- | --- | --- | --- | --- | --- | --- | --- | --- |
|  |  | NT | AA |  | NT | AA |  |  |

No genes match the given filter

Detailed domain annotation

Shows NRPS- and PKS-related domains for each feature that contains them. Click on each domain for more information about the domain's location, consensus monomer prediction, and other details.  
A domain glossary is available here, and an explanation of the visualisation is available here.

Selected features only

Show module domains

Similar known gene clusters from MIBiG 3.1

Shows clusters from the MiBIG database that are similar to the current region. Genes marked with the same colour are interrelated. White genes have no relationship.  
Click on reference genes to show details of similarities to genes within the current region.  
Click on an accession to open that entry in the MiBIG database.

No matches found.

Similar subclusters

Shows sub-cluster units that are similar to the current region. Genes marked with the same colour are interrelated. White genes have no relationship.  
Click on reference genes to show details of similarities to genes within the current region.

No matches found.

Module view

Shows module structures for each candidate cluster in NRPS and PKS regions.   
Genes are shown in predicted order, and are only present when containing at least one complete module.   
A domain glossary is available here, and an explanation of the visualisation is available here.

Candidate 7 (829880 - 895923): neighbouring NRPS-like

Candidate 8 (829880 - 874133): single NRPS-like

Candidate 9 (836451 - 880676): single NRPS-like

Candidate 10 (851763 - 895923): single NRPS-like

Legend

NRPS/PKS products

NRPS/PKS substrates

Predicted core structure(s)

Shows estimated product structure and polymer for each candidate cluster in the region. To show the product, click on the expander or the candidate cluster feature drawn in the overview.

For candidate cluster 7, location 829880 - 895923:

Rough prediction of core scaffold based on assumed PKS/NRPS colinearity; tailoring reactions not taken into account

**Polymer prediction:**
:   (X) + (X)

  
Direct lookup in NORINE database:
strict
or
relaxed

---

For candidate cluster 8, location 829880 - 874133:

Rough prediction of core scaffold based on assumed PKS/NRPS colinearity; tailoring reactions not taken into account

**Polymer prediction:**
:   (X) + (X)

  
Direct lookup in NORINE database:
strict
or
relaxed

---

For candidate cluster 9, location 836451 - 880676:

Rough prediction of core scaffold based on assumed PKS/NRPS colinearity; tailoring reactions not taken into account

**Polymer prediction:**
:   (X) + (X)

  
Direct lookup in NORINE database:
strict
or
relaxed

---

For candidate cluster 10, location 851763 - 895923:

Rough prediction of core scaffold based on assumed PKS/NRPS colinearity; tailoring reactions not taken into account

**Polymer prediction:**
:   (X) + (X)

  
Direct lookup in NORINE database:
strict
or
relaxed

Link to NORINE database query form

NRPS/PKS substrate predictions

Shows the predicted substrates for each adenylation domain and acyltransferase within genes. Each gene prediction can be expanded to view detailed predictions of each domain. Each prediction can be expanded to view the predictions by tool (and, for some tools, further expanded for extra details).

**jgi.p\_Fomfom1\_1373552**: X

:   **AMP-binding (35..448)**: X

    nrpys: Val, Leu, Ile, Abu, Ival

    SVM prediction details:
    :   Predicted physicochemical class:
        :   hydrophobic-aliphatic (Ala, Gly, Val, Leu, Ile, Abu, Ival, Ser, Thr, Hpg, Dhpg, Cys, Pro, Hpr)

        Large clusters prediction:
        :   Apolar, aliphatic (Gly, Ala, Val, Leu, Ile, Abu, Ival)

        Small clusters prediction:
        :   Aliphatic, branched hydrophobic (Val, Leu, Ile, Abu, Ival)

        Single AA prediction:
        :   N/A

    Stachelhaus prediction details:
    :   Stachelhaus sequence:
        :   GMYWLGILIK

        Nearest Stachelhaus code(s):
        :   Kiv GLYWLGSSGK (29% 8Å match)

        Stachelhaus code match:
        :   60% (weak)

  
**jgi.p\_Fomfom1\_324375**: X

:   **AMP-binding (32..353)**: X

    nrpys: Ala, Gly, Val, Leu, Ile, Abu, Ival, Ser, Thr, Hpg, Dhpg, Cys, Pro, Hpr

    SVM prediction details:
    :   Predicted physicochemical class:
        :   hydrophobic-aliphatic (Ala, Gly, Val, Leu, Ile, Abu, Ival, Ser, Thr, Hpg, Dhpg, Cys, Pro, Hpr)

        Large clusters prediction:
        :   N/A

        Small clusters prediction:
        :   N/A

        Single AA prediction:
        :   N/A

    Stachelhaus prediction details:
    :   Stachelhaus sequence:
        :   ALFWMGE--K

        Nearest Stachelhaus code(s):

        Stachelhaus code match:
        :   0% (weak)

  
**jgi.p\_Fomfom1\_324542**: X

:   **AMP-binding (33..448)**: X

    nrpys: Val, Leu, Ile, Abu, Ival

    SVM prediction details:
    :   Predicted physicochemical class:
        :   hydrophobic-aliphatic (Ala, Gly, Val, Leu, Ile, Abu, Ival, Ser, Thr, Hpg, Dhpg, Cys, Pro, Hpr)

        Large clusters prediction:
        :   Apolar, aliphatic (Gly, Ala, Val, Leu, Ile, Abu, Ival)

        Small clusters prediction:
        :   Aliphatic, branched hydrophobic (Val, Leu, Ile, Abu, Ival)

        Single AA prediction:
        :   N/A

    Stachelhaus prediction details:
    :   Stachelhaus sequence:
        :   -HGWIGGTVK

        Nearest Stachelhaus code(s):
        :   Ala DVGWITGIVK (38% 8Å match)
        :   Val DAFWIGGTFK (35% 8Å match)

        Stachelhaus code match:
        :   60% (weak)

scaffold\_14 - Region 4 - NRPS-like

Shows the layout of the region, marking coding sequences and areas of interest. Clicking a gene will select it and show any relevant details. Clicking an area feature (e.g. a candidate cluster) will select all coding sequences within that area. Double clicking an area feature will zoom to that area. Multiple genes and area features can be selected by clicking them while holding the Ctrl key.  
More detailed help is available here.

Download region GenBank file

Download region SVG

Location: 914,477 - 958,700 nt. (total: 44,224 nt)
Show pHMM detection rules used

NRPS-like: cds((PP-binding or NAD\_binding\_4) and (AMP-binding or A-OX))

#### Legend:

core biosynthetic genes

additional biosynthetic genes

transport-related genes

regulatory genes

other genes

resistance

reset view

zoom to selection

Gene details

Shows details of the most recently selected gene, including names, products, location, and other annotations.

Select a gene to view the details available for it

Gene overview

NRPS/PKS domains

KnownClusterBlast

SubClusterBlast

NRPS/PKS modules

Gene/CDS overview

A brief tabular summary of genes/CDS features within the region.  
Filtering the table will also search biosynthetic profiles and gene function data. If enabled, the overview will then zoom to show the area covered by the filtered selection.  
Genes selected in the region drawing above will be marked in the table with an indicator to the left of the gene name.

Filter:

Automatically zoom to filtered/selected features

| Identifier | Product | Length | | Function | Sequence | | NCBI Blast | Filter details |
| --- | --- | --- | --- | --- | --- | --- | --- | --- |
|  |  | NT | AA |  | NT | AA |  |  |

No genes match the given filter

Detailed domain annotation

Shows NRPS- and PKS-related domains for each feature that contains them. Click on each domain for more information about the domain's location, consensus monomer prediction, and other details.  
A domain glossary is available here, and an explanation of the visualisation is available here.

Selected features only

Show module domains

Similar known gene clusters from MIBiG 3.1

Shows clusters from the MiBIG database that are similar to the current region. Genes marked with the same colour are interrelated. White genes have no relationship.  
Click on reference genes to show details of similarities to genes within the current region.  
Click on an accession to open that entry in the MiBIG database.

No matches found.

Similar subclusters

Shows sub-cluster units that are similar to the current region. Genes marked with the same colour are interrelated. White genes have no relationship.  
Click on reference genes to show details of similarities to genes within the current region.

No matches found.

Module view

Shows module structures for each candidate cluster in NRPS and PKS regions.   
Genes are shown in predicted order, and are only present when containing at least one complete module.   
A domain glossary is available here, and an explanation of the visualisation is available here.

Candidate 11 (914476 - 958700): single NRPS-like

Legend

NRPS/PKS products

NRPS/PKS substrates

Predicted core structure(s)

Shows estimated product structure and polymer for each candidate cluster in the region. To show the product, click on the expander or the candidate cluster feature drawn in the overview.

For candidate cluster 11, location 914476 - 958700:

Rough prediction of core scaffold based on assumed PKS/NRPS colinearity; tailoring reactions not taken into account

**Polymer prediction:**
:   (X)

  
Direct lookup in NORINE database:
strict
or
relaxed

Link to NORINE database query form

NRPS/PKS substrate predictions

Shows the predicted substrates for each adenylation domain and acyltransferase within genes. Each gene prediction can be expanded to view detailed predictions of each domain. Each prediction can be expanded to view the predictions by tool (and, for some tools, further expanded for extra details).

**jgi.p\_Fomfom1\_1210865**: X

:   **AMP-binding (37..446)**: X

    nrpys: Gly, Ala, Val, Leu, Ile, Abu, Ival

    SVM prediction details:
    :   Predicted physicochemical class:
        :   hydrophobic-aliphatic (Ala, Gly, Val, Leu, Ile, Abu, Ival, Ser, Thr, Hpg, Dhpg, Cys, Pro, Hpr)

        Large clusters prediction:
        :   Apolar, aliphatic (Gly, Ala, Val, Leu, Ile, Abu, Ival)

        Small clusters prediction:
        :   N/A

        Single AA prediction:
        :   N/A

    Stachelhaus prediction details:
    :   Stachelhaus sequence:
        :   GLFWMGMMIK

        Nearest Stachelhaus code(s):

        Stachelhaus code match:
        :   0% (weak)

scaffold\_15 - Region 1 - terpene

Shows the layout of the region, marking coding sequences and areas of interest. Clicking a gene will select it and show any relevant details. Clicking an area feature (e.g. a candidate cluster) will select all coding sequences within that area. Double clicking an area feature will zoom to that area. Multiple genes and area features can be selected by clicking them while holding the Ctrl key.  
More detailed help is available here.

Download region GenBank file

Download region SVG

Location: 167,779 - 189,620 nt. (total: 21,842 nt)
Show pHMM detection rules used

terpene: (Terpene\_synth or Terpene\_synth\_C or phytoene\_synt or Lycopene\_cycl or terpene\_cyclase or NapT7 or fung\_ggpps or fung\_ggpps2 or trichodiene\_synth or TRI5)

#### Legend:

core biosynthetic genes

additional biosynthetic genes

transport-related genes

regulatory genes

other genes

resistance

reset view

zoom to selection

Gene details

Shows details of the most recently selected gene, including names, products, location, and other annotations.

Select a gene to view the details available for it

Gene overview

KnownClusterBlast

SubClusterBlast

Gene/CDS overview

A brief tabular summary of genes/CDS features within the region.  
Filtering the table will also search biosynthetic profiles and gene function data. If enabled, the overview will then zoom to show the area covered by the filtered selection.  
Genes selected in the region drawing above will be marked in the table with an indicator to the left of the gene name.

Filter:

Automatically zoom to filtered/selected features

| Identifier | Product | Length | | Function | Sequence | | NCBI Blast | Filter details |
| --- | --- | --- | --- | --- | --- | --- | --- | --- |
|  |  | NT | AA |  | NT | AA |  |  |

No genes match the given filter

Similar known gene clusters from MIBiG 3.1

Shows clusters from the MiBIG database that are similar to the current region. Genes marked with the same colour are interrelated. White genes have no relationship.  
Click on reference genes to show details of similarities to genes within the current region.  
Click on an accession to open that entry in the MiBIG database.

No matches found.

Similar subclusters

Shows sub-cluster units that are similar to the current region. Genes marked with the same colour are interrelated. White genes have no relationship.  
Click on reference genes to show details of similarities to genes within the current region.

No matches found.

scaffold\_16 - Region 1 - terpene

Shows the layout of the region, marking coding sequences and areas of interest. Clicking a gene will select it and show any relevant details. Clicking an area feature (e.g. a candidate cluster) will select all coding sequences within that area. Double clicking an area feature will zoom to that area. Multiple genes and area features can be selected by clicking them while holding the Ctrl key.  
More detailed help is available here.

Download region GenBank file

Download region SVG

Location: 1 - 11,816 nt. (total: 11,816 nt)
Show pHMM detection rules used

Region on contig edge.

terpene: (Terpene\_synth or Terpene\_synth\_C or phytoene\_synt or Lycopene\_cycl or terpene\_cyclase or NapT7 or fung\_ggpps or fung\_ggpps2 or trichodiene\_synth or TRI5)

#### Legend:

core biosynthetic genes

additional biosynthetic genes

transport-related genes

regulatory genes

other genes

resistance

reset view

zoom to selection

Gene details

Shows details of the most recently selected gene, including names, products, location, and other annotations.

Select a gene to view the details available for it

Gene overview

KnownClusterBlast

SubClusterBlast

Gene/CDS overview

A brief tabular summary of genes/CDS features within the region.  
Filtering the table will also search biosynthetic profiles and gene function data. If enabled, the overview will then zoom to show the area covered by the filtered selection.  
Genes selected in the region drawing above will be marked in the table with an indicator to the left of the gene name.

Filter:

Automatically zoom to filtered/selected features

| Identifier | Product | Length | | Function | Sequence | | NCBI Blast | Filter details |
| --- | --- | --- | --- | --- | --- | --- | --- | --- |
|  |  | NT | AA |  | NT | AA |  |  |

No genes match the given filter

Similar known gene clusters from MIBiG 3.1

Shows clusters from the MiBIG database that are similar to the current region. Genes marked with the same colour are interrelated. White genes have no relationship.  
Click on reference genes to show details of similarities to genes within the current region.  
Click on an accession to open that entry in the MiBIG database.

No matches found.

Similar subclusters

Shows sub-cluster units that are similar to the current region. Genes marked with the same colour are interrelated. White genes have no relationship.  
Click on reference genes to show details of similarities to genes within the current region.

No matches found.

scaffold\_16 - Region 2 - terpene

Shows the layout of the region, marking coding sequences and areas of interest. Clicking a gene will select it and show any relevant details. Clicking an area feature (e.g. a candidate cluster) will select all coding sequences within that area. Double clicking an area feature will zoom to that area. Multiple genes and area features can be selected by clicking them while holding the Ctrl key.  
More detailed help is available here.

Download region GenBank file

Download region SVG

Location: 34,800 - 79,187 nt. (total: 44,388 nt)
Show pHMM detection rules used

terpene: (Terpene\_synth or Terpene\_synth\_C or phytoene\_synt or Lycopene\_cycl or terpene\_cyclase or NapT7 or fung\_ggpps or fung\_ggpps2 or trichodiene\_synth or TRI5)

#### Legend:

core biosynthetic genes

additional biosynthetic genes

transport-related genes

regulatory genes

other genes

resistance

reset view

zoom to selection

Gene details

Shows details of the most recently selected gene, including names, products, location, and other annotations.

Select a gene to view the details available for it

Gene overview

KnownClusterBlast

SubClusterBlast

Gene/CDS overview

A brief tabular summary of genes/CDS features within the region.  
Filtering the table will also search biosynthetic profiles and gene function data. If enabled, the overview will then zoom to show the area covered by the filtered selection.  
Genes selected in the region drawing above will be marked in the table with an indicator to the left of the gene name.

Filter:

Automatically zoom to filtered/selected features

| Identifier | Product | Length | | Function | Sequence | | NCBI Blast | Filter details |
| --- | --- | --- | --- | --- | --- | --- | --- | --- |
|  |  | NT | AA |  | NT | AA |  |  |

No genes match the given filter

Similar known gene clusters from MIBiG 3.1

Shows clusters from the MiBIG database that are similar to the current region. Genes marked with the same colour are interrelated. White genes have no relationship.  
Click on reference genes to show details of similarities to genes within the current region.  
Click on an accession to open that entry in the MiBIG database.

No matches found.

Similar subclusters

Shows sub-cluster units that are similar to the current region. Genes marked with the same colour are interrelated. White genes have no relationship.  
Click on reference genes to show details of similarities to genes within the current region.

No matches found.

scaffold\_19 - Region 1 - terpene

Shows the layout of the region, marking coding sequences and areas of interest. Clicking a gene will select it and show any relevant details. Clicking an area feature (e.g. a candidate cluster) will select all coding sequences within that area. Double clicking an area feature will zoom to that area. Multiple genes and area features can be selected by clicking them while holding the Ctrl key.  
More detailed help is available here.

Download region GenBank file

Download region SVG

Location: 126,382 - 147,637 nt. (total: 21,256 nt)
Show pHMM detection rules used

terpene: (Terpene\_synth or Terpene\_synth\_C or phytoene\_synt or Lycopene\_cycl or terpene\_cyclase or NapT7 or fung\_ggpps or fung\_ggpps2 or trichodiene\_synth or TRI5)

#### Legend:

core biosynthetic genes

additional biosynthetic genes

transport-related genes

regulatory genes

other genes

resistance

reset view

zoom to selection

Gene details

Shows details of the most recently selected gene, including names, products, location, and other annotations.

Select a gene to view the details available for it

Gene overview

KnownClusterBlast

SubClusterBlast

Gene/CDS overview

A brief tabular summary of genes/CDS features within the region.  
Filtering the table will also search biosynthetic profiles and gene function data. If enabled, the overview will then zoom to show the area covered by the filtered selection.  
Genes selected in the region drawing above will be marked in the table with an indicator to the left of the gene name.

Filter:

Automatically zoom to filtered/selected features

| Identifier | Product | Length | | Function | Sequence | | NCBI Blast | Filter details |
| --- | --- | --- | --- | --- | --- | --- | --- | --- |
|  |  | NT | AA |  | NT | AA |  |  |

No genes match the given filter

Similar known gene clusters from MIBiG 3.1

Shows clusters from the MiBIG database that are similar to the current region. Genes marked with the same colour are interrelated. White genes have no relationship.  
Click on reference genes to show details of similarities to genes within the current region.  
Click on an accession to open that entry in the MiBIG database.

No matches found.

Similar subclusters

Shows sub-cluster units that are similar to the current region. Genes marked with the same colour are interrelated. White genes have no relationship.  
Click on reference genes to show details of similarities to genes within the current region.

No matches found.

If you have found antiSMASH useful, please cite us.
