# Supplementary material for: Uncovering the transcriptional landscape of Fomes fomentarius during fungal-based material production through gene co-expression network analysis
Source: Fungal Biol Biotechnol. 2025 Feb 13;12:1. doi: 10.1186/s40694-024-00192-3 (PMC11827164; doi:10.1186/s40694-024-00192-3)
Supplement: Supplementary file 1 — Supplementary Material 1 [file 40694_2024_192_MOESM1_ESM.zip › knownclusterblast/region1/jgi.p_Fomfom1_1212942_mibig_hits.html]

| MIBiG Protein | Description | MIBiG Cluster | MiBiG Product | % ID | % Coverage | BLAST Score | E-value |
| --- | --- | --- | --- | --- | --- | --- | --- |
| QJQ03972.1 | CYP-Arm3 | BGC0002445 | Terpene | 49.0 | 90.0 | 494.0 | 5.87e-171 |
| KAA1470686.1 | cytochrome\_P450 | BGC0002218 | Terpene | 46.0 | 95.6 | 482.0 | 4.46e-166 |
| XP\_007301850.1 | cytochrome\_P450 | BGC0001617 | Terpene | 48.0 | 93.5 | 480.0 | 3.1e-165 |
| XP\_007301602.1 | cytochrome\_P450 | BGC0001617 | Terpene | 46.0 | 96.1 | 457.0 | 3.59e-156 |
| QJQ03971.1 | CYP-Arm2 | BGC0002445 | Terpene | 47.0 | 90.4 | 456.0 | 4.06e-156 |
| XP\_007301852.1 | cytochrome\_P450 | BGC0001617 | Terpene | 38.0 | 93.0 | 366.0 | 7.56e-121 |
| XP\_007301851.1 | cytochrome\_P450 | BGC0001617 | Terpene | 36.0 | 87.6 | 308.0 | 3.33e-98 |
| QGW49097.1 | putative\_cytochrome\_P450 | BGC0002731 | Polyketide | 35.0 | 91.1 | 271.0 | 2.91e-84 |
| KJA16708.1 | hypothetical\_protein | BGC0002246 | Terpene | 31.0 | 96.1 | 265.0 | 1.52e-81 |
| FAC38\_04 |  | BGC0002198 | NRP | 33.0 | 94.3 | 262.0 | 1.14e-80 |
| DAB41655.1 | cytochrome\_P450\_monooxygenase | BGC0001585 | Alkaloid | 31.0 | 94.3 | 253.0 | 4.72e-77 |
| CBF82795.1 | cytochrome\_P450,\_putative\_(Eurofung) | BGC0001668 | NRP | 34.0 | 96.9 | 248.0 | 2.89e-75 |
| QQO98481.1 | FrzL | BGC0002146 | NRP | 33.0 | 88.0 | 247.0 | 3.73e-75 |
| KIJ60843.1 | hypothetical\_protein | BGC0002214 | Polyketide | 35.0 | 80.1 | 245.0 | 5.32e-75 |
| BCI98769.1 | putative\_cytochrome\_P450 | BGC0002181 | Terpene | 30.0 | 97.2 | 246.0 | 9.61e-75 |
| AQZ42158.1 | putative\_cytochrome\_P450 | BGC0001820 | NRP | 30.0 | 92.3 | 246.0 | 2.23e-74 |
| EHK18434.1 | hypothetical\_protein | BGC0002233 | Polyketide | 32.0 | 91.7 | 243.0 | 2.77e-73 |
| QBC75451.1 | MacC | BGC0002615 | Terpene | 31.0 | 95.4 | 236.0 | 1.6e-70 |
| EAL85116.1 | cytochrome\_P450\_oxidoreductase | BGC0001067 | Terpene+Polyketide:Iterative type I polyketide | 30.0 | 95.8 | 234.0 | 8.57e-70 |
| KIJ60837.1 | hypothetical\_protein | BGC0002214 | Polyketide | 33.0 | 84.1 | 229.0 | 3.73e-68 |
| BAE60012.1 |  | BGC0001518 | Terpene | 30.0 | 90.8 | 228.0 | 5.35e-68 |
| KIJ60846.1 | hypothetical\_protein | BGC0002214 | Polyketide | 31.0 | 90.0 | 228.0 | 1.12e-67 |
| KIJ60841.1 | hypothetical\_protein | BGC0002214 | Polyketide | 33.0 | 89.1 | 226.0 | 4.84e-67 |
| QJQ03970.1 | CYP-Arm1 | BGC0002445 | Terpene | 29.0 | 98.2 | 218.0 | 7.87e-64 |
| QDO73503.1 | PeniB | BGC0002557 | Terpene | 29.0 | 90.8 | 213.0 | 7.08e-62 |
| EIN09540.1 | cytochrome\_P450 | BGC0002213 | Polyketide | 31.0 | 88.2 | 208.0 | 2.47e-60 |
| EIM84826.1 | cytochrome\_P450 | BGC0002219 | Terpene | 30.0 | 85.2 | 207.0 | 3.75e-60 |
| OQD69071.1 | hypothetical\_protein | BGC0002745 | Polyketide | 31.0 | 84.1 | 205.0 | 4.4e-59 |
| CBF82292.1 | cytochrome\_P450,\_putative\_(Eurofung) | BGC0002180 | Polyketide | 30.0 | 91.3 | 205.0 | 5.17e-59 |
| BBB04330.1 | cytochrome\_P450 | BGC0001717 | NRP | 30.0 | 81.2 | 201.0 | 1.38e-57 |
| QJQ82463.1 | BisI | BGC0002290 | Other | 30.0 | 85.6 | 196.0 | 1.13e-55 |
| AAS90061.1 | OrdA | BGC0000009 | Polyketide | 29.0 | 93.4 | 195.0 | 1.88e-55 |
| AAS90081.1 | OrdA | BGC0000010 | Polyketide | 28.0 | 92.3 | 195.0 | 1.88e-55 |
| AAS90013.1 | OrdA | BGC0000007 | Polyketide | 29.0 | 97.2 | 195.0 | 2.61e-55 |
| AAS90035.1 | OrdA | BGC0000008 | Polyketide | 28.0 | 92.3 | 194.0 | 6.99e-55 |
| AAS90105.1 | OrdA | BGC0000006 | Polyketide | 28.0 | 92.3 | 193.0 | 1.35e-54 |
| XP\_020057670.1 | uncharacterized\_protein | BGC0001718 | NRP | 28.0 | 87.5 | 191.0 | 4.99e-54 |
| BAE71330.1 | oxidoreductase\_A;oxidoreductase/cytochrome\_P450\_monooxygenase | BGC0000004 | Polyketide | 28.0 | 93.9 | 190.0 | 1.85e-53 |
| BCI98774.1 | putative\_cytochrome\_P450 | BGC0002181 | Terpene | 27.0 | 96.3 | 177.0 | 5.82e-49 |
| EAL85111.2 | cytochrome\_P450\_oxidoreductase | BGC0001037 | NRP+Polyketide:Iterative type I polyketide | 28.0 | 90.0 | 176.0 | 1.36e-48 |
| QMS79071.1 | fumitremorgin\_C\_synthase | BGC0002198 | NRP | 29.0 | 98.7 | 170.0 | 5.37e-46 |
| AGK82824.1 | cytochrome\_P450-2 | BGC0001323 | Terpene | 26.0 | 89.5 | 164.0 | 4.58e-44 |
| chr3.CM0241.850.r2.m |  | BGC0001316 | Other | 27.0 | 91.5 | 163.0 | 6.63e-44 |
| AGK82817.1 | cytochrome\_P450-1 | BGC0001324 | Terpene | 26.0 | 91.1 | 163.0 | 7.07e-44 |
| BCA42574.1 | cytochrome\_P450\_monooxygenase\_GrgG | BGC0002185 | Polyketide | 29.0 | 97.2 | 163.0 | 1.91e-43 |
| KGN46390.1 | hypothetical\_protein | BGC0001315 | Terpene | 27.0 | 91.1 | 161.0 | 4.37e-43 |
| AGK82831.1 | cytochrome\_P450-2 | BGC0001321 | Terpene | 26.0 | 91.7 | 159.0 | 1.62e-42 |
| AGK82807.1 | cytochrome\_P450-1 | BGC0001322 | Terpene | 26.0 | 91.5 | 158.0 | 4.01e-42 |
| AGK82815.1 | cytochrome\_P450-2 | BGC0001324 | Terpene | 26.0 | 91.1 | 158.0 | 4.01e-42 |
| CCT72382.1 | related\_to\_O-methylsterigmatocystin\_oxidoreductase | BGC0001305 | Polyketide | 27.0 | 98.9 | 156.0 | 5.59e-41 |
| BAF09097.1 |  | BGC0000672 | Terpene | 27.0 | 85.2 | 149.0 | 7.44e-39 |
| XP\_044963914.1 | cytochrome\_P450\_99A2-like | BGC0002395 | Terpene | 26.0 | 91.1 | 148.0 | 1.94e-38 |
| pseudo106205\_112773 |  | BGC0001322 | Terpene | 26.0 | 88.6 | 147.0 | 2.85e-38 |
| EEF48740.1 | cytochrome\_P450,\_putative | BGC0002393 | Terpene | 27.0 | 93.0 | 143.0 | 1.09e-36 |
| NP\_199073.1 | cytochrome\_P450\_71A16 | BGC0000669 | Terpene | 25.0 | 87.1 | 140.0 | 1.29e-35 |
| chr3.CM0292.110.r2.m |  | BGC0001317 | Terpene | 26.0 | 89.5 | 139.0 | 3.28e-35 |
| BAF14091.1 |  | BGC0000671 | Terpene | 27.0 | 93.4 | 137.0 | 1.28e-34 |
| CYP82X1 |  | BGC0001325 | Alkaloid | 26.0 | 101.1 | 137.0 | 2.54e-34 |
| BAF14086.1 |  | BGC0000671 | Terpene | 26.0 | 89.5 | 133.0 | 2.63e-33 |
| EER93097.1 | hypothetical\_protein | BGC0000798 | Saccharide | 25.0 | 89.7 | 125.0 | 1.93e-30 |
| XP\_037497843.1 | premnaspirodiene\_oxygenase | BGC0002724 | Terpene | 26.0 | 88.2 | 124.0 | 3.58e-30 |
| chr3.CM0241.310.r2.m |  | BGC0001316 | Other | 24.0 | 91.1 | 115.0 | 3.79e-27 |
| chr3.CM0241.700.r2.m |  | BGC0001316 | Other | 22.0 | 98.0 | 112.0 | 3.99e-26 |
| CYP82Y1 |  | BGC0001325 | Alkaloid | 24.0 | 90.6 | 109.0 | 6.27e-25 |
| AAG27132.1 | Fum6p | BGC0000062 | Polyketide | 24.0 | 96.1 | 104.0 | 5.75e-23 |
| Manes.12G133500 |  | BGC0001318 | Other | 34.0 | 34.9 | 101.0 | 2.57e-22 |
| BBF25317.1 | bifunctional\_P-450:NADPH-P450\_reductase | BGC0001923 | Terpene+Polyketide | 24.0 | 92.3 | 101.0 | 5.42e-22 |
| EED17999.1 | benzoate\_4-monooxygenase\_cytochrome\_P450,\_putative | BGC0000154 | Polyketide:Iterative type I polyketide | 33.0 | 30.3 | 85.0 | 2.49e-17 |
| EGO28830.1 | hypothetical\_protein | BGC0002221 | Terpene | 22.0 | 103.9 | 86.0 | 3.52e-17 |
| ESU09203.1 | hypothetical\_protein | BGC0002594 | Polyketide | 23.0 | 93.2 | 85.0 | 4.55e-17 |
| EED17998.1 | P450 | BGC0000154 | Polyketide:Iterative type I polyketide | 33.0 | 30.3 | 85.0 | 5.13e-17 |
| Manes.12G132900 |  | BGC0001318 | Other | 32.0 | 24.5 | 78.0 | 8.36e-15 |
| QTC09989.1 | cytochrome\_P450 | BGC0002372 | Polyketide+Terpene+Alkaloid | 22.0 | 81.4 | 76.0 | 2.85e-14 |
| Manes.12G132800 |  | BGC0001318 | Other | 30.0 | 26.8 | 76.0 | 3.48e-14 |
| CBF83149.1 | cytochrome\_P450,\_putative\_(Eurofung) | BGC0001722 | Polyketide | 26.0 | 30.1 | 72.0 | 7.57e-13 |
| AWM95791.1 | cytochrome\_P450\_oxygenase | BGC0001827 | Polyketide | 25.0 | 36.2 | 69.0 | 7.29e-12 |
| CDM36724.1 | Cytochrome\_P450 | BGC0001360 | Polyketide | 27.0 | 34.1 | 67.0 | 2.45e-11 |
| AJG44382.1 | MpaDE' | BGC0002619 | Polyketide | 28.0 | 30.6 | 64.0 | 3.76e-10 |
| BBU37365.1 | P450\_monooxygenase | BGC0002525 | Polyketide | 27.0 | 36.2 | 62.0 | 6.97e-10 |
| CAQ18837.1 | putative\_cytochrome\_P450 | BGC0000954 | NRP+Polyketide:Modular type I polyketide | 26.0 | 41.7 | 61.0 | 1.88e-09 |
| AGO65988.1 | putative\_cytochrome\_p450 | BGC0000992 | NRP+Polyketide | 25.0 | 35.8 | 59.0 | 8.47e-09 |
| QNH68019.1 | PfpB | BGC0002268 | Polyketide+NRP | 32.0 | 28.0 | 58.0 | 1.37e-08 |
| QPC57088.1 | cytochrome\_P450 | BGC0002230 | Polyketide+NRP | 32.0 | 20.1 | 58.0 | 1.41e-08 |
| PKY07888.1 | cytochrome\_P450 | BGC0001544 | NRP+Polyketide | 27.0 | 34.9 | 58.0 | 1.85e-08 |
| AZZ09610.1 | PvhE | BGC0002304 | Polyketide+NRP | 25.0 | 30.8 | 57.0 | 4.36e-08 |
| iliC |  | BGC0002035 | NRP+Polyketide | 31.0 | 20.8 | 51.0 | 1.91e-06 |
