# Supplementary material for: Uncovering the transcriptional landscape of Fomes fomentarius during fungal-based material production through gene co-expression network analysis
Source: Fungal Biol Biotechnol. 2025 Feb 13;12:1. doi: 10.1186/s40694-024-00192-3 (PMC11827164; doi:10.1186/s40694-024-00192-3)
Supplement: Supplementary file 1 — Supplementary Material 1 [file 40694_2024_192_MOESM1_ESM.zip › knownclusterblast/region1/jgi.p_Fomfom1_1190804_mibig_hits.html]

| MIBiG Protein | Description | MIBiG Cluster | MiBiG Product | % ID | % Coverage | BLAST Score | E-value |
| --- | --- | --- | --- | --- | --- | --- | --- |
| QUJ09159.1 | Lon12 | BGC0002440 | NRP | 29.0 | 73.0 | 124.0 | 4.77e-31 |
| PluTT01m\_09605 | hypothetical\_protein | BGC0000383 | NRP+Polyketide:Modular type I polyketide | 31.0 | 47.0 | 116.0 | 9.77e-30 |
| AKT74269.1 | TxnP2 | BGC0002141 | Polyketide | 26.0 | 80.8 | 103.0 | 3.59e-23 |
