# Supplementary material for: Uncovering the transcriptional landscape of Fomes fomentarius during fungal-based material production through gene co-expression network analysis
Source: Fungal Biol Biotechnol. 2025 Feb 13;12:1. doi: 10.1186/s40694-024-00192-3 (PMC11827164; doi:10.1186/s40694-024-00192-3)
Supplement: Supplementary file 1 — Supplementary Material 1 [file 40694_2024_192_MOESM1_ESM.zip › knownclusterblast/region1/jgi.p_Fomfom1_1364407_mibig_hits.html]

| MIBiG Protein | Description | MIBiG Cluster | MiBiG Product | % ID | % Coverage | BLAST Score | E-value |
| --- | --- | --- | --- | --- | --- | --- | --- |
| AFV52199.1 | acyl-ACP\_synthetase | BGC0000081 | NRP+Polyketide:Iterative type I polyketide+Polyketide:Enediyne type I polyketide | 33.0 | 30.3 | 223.0 | 4.13e-61 |
| AEA35021.1 | hypothetical\_protein | BGC0002502 | Polyketide | 33.0 | 24.6 | 166.0 | 8.74e-43 |
| ACB12556.1 | Fum10 | BGC0000063 | Polyketide | 32.0 | 24.6 | 161.0 | 4.06e-41 |
| AGM05529.1 | long-chain\_acyl-CoA\_synthetase | BGC0002098 | Polyketide | 32.0 | 24.0 | 150.0 | 9.92e-38 |
| AAN74813.2 | Fum10p | BGC0000062 | Polyketide | 31.0 | 25.6 | 149.0 | 4.37e-37 |
| NP\_626687.1 | acyl-CoA\_synthetase | BGC0000595 | RiPP | 32.0 | 23.1 | 137.0 | 1.54e-33 |
| WP\_003976362.1 | acyl-CoA\_synthetase | BGC0000596 | RiPP | 32.0 | 23.1 | 137.0 | 1.54e-33 |
| SSTG\_01180 | acyl-CoA\_synthetase | BGC0000597 | RiPP:Proteusin | 32.0 | 24.1 | 134.0 | 1.15e-32 |
| BAQ25518.1 | acyl-CoA\_synthetase | BGC0001288 | Polyketide | 30.0 | 23.1 | 129.0 | 7.37e-31 |
| BCK51658.1 | putative\_acyl-CoA\_synthetase | BGC0002520 | Polyketide | 31.0 | 24.4 | 125.0 | 8.58e-30 |
| BBD17761.1 | non-ribosomal\_peptide\_synthetase | BGC0001919 | NRP+Polyketide | 27.0 | 32.5 | 127.0 | 1.03e-28 |
| AAQ84158.1 | PlmJK | BGC0000123 | Polyketide | 30.0 | 23.3 | 122.0 | 2.04e-27 |
| AHH25585.1 | AMP-dependent\_synthetase\_and\_ligase | BGC0000957 | NRP+Polyketide | 30.0 | 22.8 | 118.0 | 2.98e-26 |
| AKD43499.1 | Acyl-CoA\_ligase | BGC0001409 | Polyketide | 28.0 | 22.8 | 114.0 | 5.49e-26 |
| BBD17741.1 | non-ribosomal\_peptide\_synthetase | BGC0001918 | NRP+Polyketide | 27.0 | 27.6 | 117.0 | 9.89e-26 |
| UHY14129.1 | PKS\_I | BGC0002671 | Polyketide | 28.0 | 35.6 | 114.0 | 6.5e-25 |
| AGM05527.1 | AMP-dependent\_synthetase\_and\_ligase | BGC0002098 | Polyketide | 27.0 | 23.9 | 109.0 | 3.93e-24 |
| ABU70356.1 | hypothetical\_protein | BGC0001890 | NRP | 26.0 | 23.7 | 108.0 | 5.88e-24 |
| KZM73517.1 | hypothetical\_protein | BGC0000632 | Terpene+Saccharide | 26.0 | 23.7 | 107.0 | 1.47e-23 |
| KDQ70107.1 | Triostin\_synthetase\_I | BGC0001444 | Other:shikimate derived | 27.0 | 22.4 | 103.0 | 2.71e-22 |
| AWX24482.1 | non-ribosomal\_peptide\_synthetase | BGC0001695 | NRP | 26.0 | 24.4 | 103.0 | 2.1e-21 |
| BBD17759.1 | non-ribosomal\_peptide\_synthetase | BGC0001919 | NRP+Polyketide | 26.0 | 24.1 | 101.0 | 8.07e-21 |
| AFB35629.1 | 6-MSA\_adenylase | BGC0000935 | Polyketide+Other:Aminocoumarin | 27.0 | 25.4 | 98.0 | 2.57e-20 |
| ADI58632.1 | 5-Enolpyruvylshikimate-3-phosphate\_synthase/CHC-CoA\_ligase | BGC0000187 | Polyketide:Type II polyketide | 28.0 | 23.1 | 95.0 | 3.82e-19 |
| CAG69514.1 | 2,3-dihydroxybenzoate-AMP\_ligase | BGC0000352 | NRP:NRP siderophore | 24.0 | 22.4 | 92.0 | 1.14e-18 |
| ADE34495.1 | ssfL1 | BGC0000269 | Polyketide:Type II polyketide+Saccharide:Hybrid/tailoring saccharide | 26.0 | 23.0 | 92.0 | 1.54e-18 |
| ACL77541.1 | AMP-dependent\_synthetase\_and\_ligase | BGC0001891 | Other | 25.0 | 22.6 | 91.0 | 2.46e-18 |
| CCC55917.1 | putative\_acyl\_CoA\_ligase | BGC0000973 | NRP+Polyketide:Modular type I polyketide | 27.0 | 25.6 | 91.0 | 3.86e-18 |
| MBE3202941.1 | AMP-binding\_protein | BGC0002410 | NRP | 27.0 | 23.5 | 89.0 | 1.34e-17 |
| AFV52187.1 | acyl-CoA\_synthetase | BGC0000081 | NRP+Polyketide:Iterative type I polyketide+Polyketide:Enediyne type I polyketide | 26.0 | 24.1 | 89.0 | 1.98e-17 |
| ADB92576.1 | Ccb2 | BGC0001225 | NRP | 24.0 | 22.6 | 87.0 | 4.51e-17 |
| BAI63284.1 | putative\_peptide\_arylation\_enzyme | BGC0000434 | NRP | 27.0 | 22.6 | 87.0 | 5.51e-17 |
| ctg4\_1 |  | BGC0002017 | NRP | 24.0 | 22.1 | 85.0 | 2.5e-16 |
| AQZ26587.1 | obafluorin\_dimodular\_nonribosomal\_peptide\_synthetase | BGC0001437 | NRP | 25.0 | 23.4 | 86.0 | 2.56e-16 |
| AAY42398.1 | Nonribosomal\_peptide\_synthetase | BGC0001000 | NRP:Lipopeptide+Polyketide:Modular type I polyketide | 26.0 | 25.0 | 86.0 | 2.88e-16 |
| ABW70812.1 | PchD | BGC0002475 | NRP | 25.0 | 22.9 | 85.0 | 3.25e-16 |
| AAY42397.1 | Nonribosomal\_peptide\_synthetase | BGC0001000 | NRP:Lipopeptide+Polyketide:Modular type I polyketide | 25.0 | 25.0 | 86.0 | 4.84e-16 |
| AQH32483.1 | hybrid\_peptide\_synthetase/polyketide\_synthase | BGC0001667 | NRP+Polyketide | 26.0 | 26.5 | 83.0 | 2.36e-15 |
| AAZ55904.1 | 2,3-dihydroxybenzoate-AMP\_ligase | BGC0000359 | NRP | 24.0 | 24.3 | 80.0 | 1.16e-14 |
| AAM77987.1 | adenylate\_ligase | BGC0000112 | Polyketide:Iterative type I polyketide+Polyketide:Enediyne type I polyketide | 26.0 | 28.6 | 80.0 | 1.21e-14 |
| ABS75234.1 | DhbE | BGC0001185 | NRP:NRP siderophore | 24.0 | 23.5 | 79.0 | 2.64e-14 |
| ATV95617.1 | CoA\_ligase | BGC0001503 | Polyketide | 24.0 | 22.7 | 78.0 | 4.74e-14 |
| ACU36660.1 | AMP-dependent\_synthetase\_and\_ligase | BGC0000392 | NRP | 23.0 | 24.6 | 77.0 | 8.37e-14 |
| ABU70375.1 | hypothetical\_protein | BGC0001890 | NRP | 26.0 | 22.8 | 77.0 | 1.05e-13 |
| QYA95658.1 | AMP-binding\_protein | BGC0002676 | NRP | 24.0 | 23.6 | 77.0 | 1.06e-13 |
| AAQ59159.1 | 2,3-dihydroxybenzoate-AMP\_ligase | BGC0002679 | NRP | 23.0 | 23.0 | 76.0 | 1.81e-13 |
| AAY93445.1 | non-ribosomal\_peptide\_synthetase\_PvdL | BGC0000413 | NRP | 25.0 | 25.6 | 77.0 | 1.85e-13 |
| ABL70475.1 | 2,3-dihydroxybenzoate-AMP\_ligase | BGC0002493 | NRP | 24.0 | 32.2 | 75.0 | 3.19e-13 |
| CAJ34366.1 | putative\_3-hydroxy-quinaldate-AMP-Ligase | BGC0000445 | NRP:Cyclic depsipeptide | 23.0 | 23.1 | 75.0 | 4.02e-13 |
| CAB15188.1 | 2,3-dihydroxybenzoate-AMP\_ligase | BGC0000309 | NRP | 22.0 | 23.9 | 75.0 | 4.2e-13 |
| BAC16758.1 | probable\_2,3-dihydroxybenzoate-AMP\_ligase\_protein | BGC0002474 | NRP | 25.0 | 24.6 | 74.0 | 7.33e-13 |
| CAC17498.1 | putative\_AMP-binding\_ligase | BGC0000324 | NRP | 24.0 | 23.6 | 74.0 | 7.54e-13 |
| CAD29795.1 | peptide\_synthetase | BGC0001015 | NRP+Polyketide | 26.0 | 24.5 | 75.0 | 8.83e-13 |
| AEM06018.2 | hypothetical\_protein | BGC0000966 | NRP+Polyketide | 26.0 | 24.2 | 74.0 | 9.64e-13 |
| ACN64830.1 | PokM3 | BGC0001061 | Polyketide:Iterative type I polyketide+Polyketide:Type II polyketide+Saccharide:Hybrid/tailoring saccharide | 24.0 | 28.2 | 74.0 | 9.9e-13 |
| AFD30957.1 | CrmE | BGC0000966 | NRP+Polyketide | 26.0 | 24.2 | 74.0 | 1.18e-12 |
| ABD14712.1 | cesB | BGC0000320 | NRP:Cyclic depsipeptide | 23.0 | 28.5 | 74.0 | 1.52e-12 |
| WP\_005009579.1 | AMP-binding\_protein | BGC0002473 | NRP | 22.0 | 23.5 | 72.0 | 3.97e-12 |
| AAF00957.1 | mcyG | BGC0001017 | NRP+Polyketide:Modular type I polyketide | 26.0 | 24.8 | 72.0 | 4.44e-12 |
| ABD14711.1 | cesA | BGC0000320 | NRP:Cyclic depsipeptide | 25.0 | 22.6 | 72.0 | 5.99e-12 |
| QBC75017.1 | acyl-CoA\_synthase | BGC0001968 | NRP | 28.0 | 20.4 | 71.0 | 7.26e-12 |
| QRK05499.1 | (2,3-dihydroxybenzoyl)adenylate\_synthase | BGC0002324 | NRP+Polyketide | 23.0 | 23.3 | 71.0 | 8.98e-12 |
| CCA53797.1 | Long-chain-fatty-acid--CoA\_ligase | BGC0001801 | NRP | 23.0 | 23.0 | 70.0 | 1.14e-11 |
| NPC94428.1 | (2,3-dihydroxybenzoyl)adenylate\_synthase | BGC0002695 | NRP | 23.0 | 23.7 | 70.0 | 1.16e-11 |
| ALK21567.1 | 2,3-dihydroxybenzoate-AMP\_ligase | BGC0002678 | NRP | 25.0 | 22.6 | 70.0 | 1.18e-11 |
| CBA63660.1 | 2,3-dihydroxybenzoate-AMP\_ligase | BGC0000368 | NRP | 22.0 | 23.1 | 70.0 | 1.54e-11 |
| KJY85277.1 | enterobactin\_synthase\_subunit\_E | BGC0002491 | NRP | 22.0 | 23.0 | 69.0 | 2.71e-11 |
| WP\_026723805.1 | AMP-dependent\_synthetase | BGC0001467 | NRP:Cyclic depsipeptide+Polyketide:Modular type I polyketide | 25.0 | 25.3 | 67.0 | 1.31e-10 |
| RGP42811.1 | 2,3-dihydroxybenzoate-AMP\_ligase | BGC0002696 | NRP | 25.0 | 23.5 | 66.0 | 2.47e-10 |
| ABC42549.1 | putative\_Co-A\_ligase | BGC0000698 | Saccharide | 27.0 | 23.3 | 66.0 | 2.55e-10 |
| BAH33406.1 | 2,3-dihydroxybenzoate-AMP\_ligase | BGC0000371 | NRP | 25.0 | 23.5 | 65.0 | 4.27e-10 |
| MBE8994627.1 | fatty\_acyl-AMP\_ligase | BGC0002623 | NRP+Polyketide | 22.0 | 25.2 | 65.0 | 4.45e-10 |
| ADD82966.1 | BatY | BGC0001099 | NRP+Polyketide:Modular type I polyketide+Polyketide:Trans-AT type I polyketide | 23.0 | 26.1 | 64.0 | 7.69e-10 |
| CAP20363.1 | equibactin\_siderophore\_biosynthetic\_protein | BGC0000347 | NRP | 21.0 | 22.8 | 64.0 | 1.23e-09 |
| WP\_068925906.1 | AMP-binding\_protein | BGC0002688 | NRP | 22.0 | 23.8 | 63.0 | 2.07e-09 |
| AFV52195.1 | acyl-CoA\_synthetase | BGC0000081 | NRP+Polyketide:Iterative type I polyketide+Polyketide:Enediyne type I polyketide | 22.0 | 22.6 | 62.0 | 2.58e-09 |
| QIE08740.1 | salicylate-AMP\_ligase | BGC0002544 | NRP | 21.0 | 24.3 | 62.0 | 2.82e-09 |
| EWM62997.1 | non-ribosomal\_peptide\_synthetase | BGC0001328 | NRP:Cyclic depsipeptide+Polyketide:Modular type I polyketide | 26.0 | 21.5 | 63.0 | 3.35e-09 |
| WP\_036342114.1 | type\_I\_polyketide\_synthase | BGC0001327 | NRP:Cyclic depsipeptide+Polyketide:Modular type I polyketide | 25.0 | 20.9 | 63.0 | 3.77e-09 |
| EJK79842.1 | amino\_acid\_adenylation\_enzyme/thioester\_reductase\_family\_protein | BGC0000436 | NRP | 25.0 | 40.7 | 62.0 | 7.64e-09 |
| CAJ45636.1 | 2,3-dihydroxybenzoate-AMP\_ligase | BGC0000454 | NRP | 23.0 | 22.6 | 61.0 | 1.13e-08 |
| ACZ65474.1 | palmitoyl-CoA\_synthetase | BGC0000140 | Polyketide | 23.0 | 22.2 | 61.0 | 1.22e-08 |
| BAO84862.1 | putative\_long-chain-fatty-acid-CoA\_ligase | BGC0000414 | NRP | 24.0 | 24.3 | 60.0 | 1.81e-08 |
| KYC42745.1 | AMP-dependent\_synthetase | BGC0002484 | NRP+Polyketide | 23.0 | 25.2 | 59.0 | 3.08e-08 |
| EDT06082.1 | AMP-dependent\_synthetase\_and\_ligase | BGC0001897 | Polyketide | 24.0 | 25.7 | 57.0 | 1.43e-07 |
| QDA77058.1 | polyketide\_synthase | BGC0002026 | NRP+Polyketide | 24.0 | 27.2 | 57.0 | 2.79e-07 |
| CAJ76286.1 | putative\_non-ribosomal\_peptide\_synthetase | BGC0000972 | NRP+Polyketide:Modular type I polyketide+Polyketide:Trans-AT type I polyketide | 22.0 | 23.3 | 56.0 | 4.39e-07 |
| AKU20507.1 | polyketide\_synthase | BGC0002687 | Polyketide+NRP | 24.0 | 20.7 | 54.0 | 1.44e-06 |
| EFL06871.1 | 2,3-dihydroxybenzoate-AMP\_ligase | BGC0000300 | NRP | 22.0 | 23.3 | 52.0 | 6.04e-06 |
