# Supplementary material for: Uncovering the transcriptional landscape of Fomes fomentarius during fungal-based material production through gene co-expression network analysis
Source: Fungal Biol Biotechnol. 2025 Feb 13;12:1. doi: 10.1186/s40694-024-00192-3 (PMC11827164; doi:10.1186/s40694-024-00192-3)
Supplement: Supplementary file 1 — Supplementary Material 1 [file 40694_2024_192_MOESM1_ESM.zip › knownclusterblast/region1/jgi.p_Fomfom1_1191794_mibig_hits.html]

| MIBiG Protein | Description | MIBiG Cluster | MiBiG Product | % ID | % Coverage | BLAST Score | E-value |
| --- | --- | --- | --- | --- | --- | --- | --- |
| KFH44392.1 | Salicylate\_hydroxylase-like\_protein | BGC0002190 | Polyketide | 31.0 | 107.8 | 194.0 | 9.85e-57 |
| OPB37949.1 | salicylate\_hydroxylase | BGC0002206 | Polyketide | 31.0 | 105.4 | 184.0 | 2.32e-53 |
| AGN71623.1 | hydroxylase | BGC0000027 | Polyketide:Iterative type I polyketide | 30.0 | 101.7 | 182.0 | 1.06e-52 |
| CAP95403.1 |  | BGC0001404 | Polyketide | 31.0 | 104.2 | 180.0 | 1.34e-51 |
| AWM95796.1 | salicylate\_hydroxylase | BGC0001827 | Polyketide | 30.0 | 109.9 | 173.0 | 1.07e-48 |
| EED18000.1 | FAD\_oxygenase | BGC0000154 | Polyketide:Iterative type I polyketide | 30.0 | 104.0 | 171.0 | 2.12e-48 |
| AWF83809.1 | Tropone\_2-monooxygenase | BGC0001487 | Other | 32.0 | 95.5 | 169.0 | 4.12e-48 |
| QCO93109.1 | monooxygenase | BGC0001976 | Terpene | 29.0 | 107.5 | 166.0 | 1.54e-46 |
| EAQ86391.1 | hypothetical\_protein | BGC0001405 | Polyketide | 31.0 | 104.0 | 166.0 | 3.55e-46 |
| EAU31924.1 | conserved\_hypothetical\_protein | BGC0002267 | Polyketide | 29.0 | 104.7 | 164.0 | 6.48e-46 |
| EAU32816.1 | conserved\_hypothetical\_protein | BGC0000160 | Polyketide | 34.0 | 91.0 | 164.0 | 7.44e-46 |
| QCF41210.1 | CcxS | BGC0002726 | Polyketide | 32.0 | 105.9 | 164.0 | 1.2e-45 |
| EAA65601.1 | hypothetical\_protein | BGC0000022 | Polyketide | 29.0 | 104.7 | 156.0 | 7.26e-43 |
| EHA28235.1 | hypothetical\_protein | BGC0001143 | Polyketide | 31.0 | 92.9 | 154.0 | 3.05e-42 |
| EWM63055.1 | monooxygenase | BGC0000679 | Terpene | 31.0 | 94.1 | 153.0 | 4.3e-42 |
| ALA99209.1 | CreL | BGC0001295 | Other | 30.0 | 100.5 | 153.0 | 5.74e-42 |
| ADD82995.1 | PtnB3 | BGC0001156 | Terpene | 31.0 | 91.7 | 149.0 | 1.16e-40 |
| ALV82350.1 | salicylate\_hydroxylase | BGC0001370 | NRP | 34.0 | 91.7 | 149.0 | 1.92e-40 |
| CAB38889.1 | Hexenoyl-S-ACP\_Monooxygenase\_(hcmO) | BGC0000315 | NRP:Lipopeptide:Ca+-dependent lipopeptide | 31.0 | 104.2 | 149.0 | 3.54e-40 |
| CBF83145.1 | conserved\_hypothetical\_protein | BGC0001722 | Polyketide | 29.0 | 102.4 | 149.0 | 1e-39 |
| QBL56164.1 | monooxygenase | BGC0002376 | Polyketide | 32.0 | 100.2 | 146.0 | 3.01e-39 |
| ACO31289.1 | PtmB3 | BGC0001140 | Terpene | 32.0 | 92.9 | 144.0 | 8.27e-39 |
| ATV82119.1 | hydroxylase | BGC0001909 | Polyketide | 29.0 | 110.4 | 142.0 | 1.82e-37 |
| AXO35181.1 | putative\_n-hydroxybenzoate\_hydroxylase | BGC0001848 | Other | 31.0 | 88.4 | 139.0 | 5.07e-37 |
| AMJ52083.1 | lijD | BGC0002255 | Polyketide | 26.0 | 105.9 | 137.0 | 1.09e-35 |
| AME18017.1 | epoxidase | BGC0001378 | Polyketide:Enediyne type I polyketide | 32.0 | 90.6 | 133.0 | 8.88e-35 |
| AMK92570.1 | FAD-dependent\_oxidoreductase | BGC0001377 | Polyketide | 32.0 | 88.4 | 131.0 | 3.96e-34 |
| WP\_038234862.1 | FAD-dependent\_monooxygenase | BGC0001873 | NRP:Lipopeptide | 30.0 | 89.4 | 124.0 | 3.02e-31 |
| QJQ82458.1 | BisD | BGC0002290 | Other | 28.0 | 100.5 | 124.0 | 4.51e-31 |
| CDF96615.1 | FAD-dependent\_Baeyer—Villiger\_monooxygenase | BGC0001149 | NRP:Lipopeptide+Saccharide:Hybrid/tailoring saccharide | 28.0 | 86.8 | 117.0 | 4.55e-29 |
| AXO35220.1 | FAD-dependent\_oxidoreductase | BGC0001848 | Other | 30.0 | 89.2 | 115.0 | 2.73e-28 |
| AYV61417.1 | salicylate\_hydroxylase | BGC0001965 | Other | 29.0 | 87.5 | 112.0 | 1e-26 |
| RZB16712.1 | FAD-binding\_protein | BGC0001850 | Other:Shikimate-derived | 30.0 | 96.7 | 108.0 | 7.09e-26 |
| AAG06716.1 | probable\_FAD-dependent\_monooxygenase | BGC0002037 | NRP | 28.0 | 89.4 | 102.0 | 1.25e-23 |
| CAM34356.1 | putative\_FAD-depending\_monooxygenase | BGC0000242 | Polyketide | 30.0 | 90.3 | 101.0 | 3.47e-23 |
| AIZ66878.1 | FAD-dependent\_monooxygenase | BGC0002666 | NRP+Alkaloid | 27.0 | 87.7 | 100.0 | 4.47e-23 |
| CAQ52625.1 | FAD-dependent\_mono\_oxygenase | BGC0001066 | Polyketide:Modular type I polyketide | 27.0 | 86.6 | 99.0 | 1.57e-22 |
| ALJ99865.1 | FlsP | BGC0001904 | Polyketide | 30.0 | 90.8 | 99.0 | 1.82e-22 |
| OKJ62000.1 | FAD-dependent\_monooxygenase | BGC0002147 | NRP | 28.0 | 88.7 | 98.0 | 3.85e-22 |
| AIE54237.1 | PauY17 | BGC0001732 | Other | 28.0 | 100.7 | 98.0 | 5.01e-22 |
| AIE54184.1 | Pau17 | BGC0001731 | Other | 28.0 | 100.7 | 97.0 | 6.79e-22 |
| FAA01293.1 | flavin-dependent\_monooxygenase\_PyvC | BGC0002210 | Polyketide+NRP | 26.0 | 99.3 | 96.0 | 4.31e-21 |
| EHA19293.1 | hypothetical\_protein | BGC0001124 | Polyketide | 25.0 | 98.6 | 95.0 | 5.49e-21 |
| AGP37409.1 | hypothetical\_protein | BGC0002386 | NRP+Polyketide | 29.0 | 90.8 | 95.0 | 7.11e-21 |
| AFW04593.1 | FAD-dependent\_oxidoreductase | BGC0001783 | Other | 29.0 | 98.8 | 94.0 | 1.19e-20 |
| AFW04561.1 | oxidoreductase | BGC0001783 | Other | 28.0 | 88.0 | 93.0 | 2.92e-20 |
| QJS40191.1 | PhzS | BGC0002439 | Other | 29.0 | 95.5 | 92.0 | 4.44e-20 |
| ACZ87042.1 | monooxygenase,\_FAD-binding\_protein | BGC0002732 | Polyketide | 29.0 | 90.1 | 91.0 | 1.36e-19 |
| AEE65479.1 | oxidoreductase | BGC0000223 | Polyketide:Type II polyketide | 29.0 | 87.3 | 90.0 | 2.46e-19 |
| UHY14128.1 | FAD-dependent\_monooxygenase | BGC0002671 | Polyketide | 27.0 | 86.8 | 89.0 | 3.91e-19 |
| PKY07891.1 | FAD\_binding\_domain-containing\_protein | BGC0001544 | NRP+Polyketide | 27.0 | 94.3 | 89.0 | 7.17e-19 |
| AAP85357.1 | putative\_monooxygenase | BGC0000233 | Polyketide | 26.0 | 87.0 | 87.0 | 2.73e-18 |
| AEW95635.1 | FAD-dependent\_monooxygenase | BGC0002697 | NRP+Polyketide | 26.0 | 87.0 | 85.0 | 1.51e-17 |
| QJS40200.1 | Salicylate\_hydroxylase | BGC0002439 | Other | 25.0 | 98.6 | 80.0 | 5.31e-16 |
| AAQ82549.1 | FscO | BGC0000034 | NRP+Polyketide | 26.0 | 82.3 | 79.0 | 1.6e-15 |
| ARE67860.1 | AbsH3 | BGC0001492 | Polyketide | 27.0 | 84.7 | 78.0 | 2.06e-15 |
| QDJ94215.1 | SpzS | BGC0002561 | Alkaloid | 27.0 | 93.9 | 78.0 | 2.22e-15 |
| OQD69144.1 | hypothetical\_protein | BGC0002745 | Polyketide | 26.0 | 86.8 | 79.0 | 3.51e-15 |
| ADE22290.1 | FAD-binding\_monooxygenase | BGC0000279 | Polyketide | 23.0 | 96.7 | 75.0 | 2.19e-14 |
| AIT42124.1 | putative\_monooxygenase | BGC0001221 | Other:Phenazine | 27.0 | 93.6 | 74.0 | 4.23e-14 |
| AGA37278.1 | FAD\_monooxygenase | BGC0000819 | NRP+Alkaloid | 23.0 | 95.8 | 72.0 | 3.81e-13 |
| AKU20511.1 | hypothetical\_protein | BGC0002687 | Polyketide+NRP | 25.0 | 91.3 | 69.0 | 1.71e-12 |
| AFD30958.1 | CrmF | BGC0000966 | NRP+Polyketide | 25.0 | 95.0 | 69.0 | 2.04e-12 |
| CCT67992.1 | bikaverin\_cluster-monooxygenase | BGC0000030 | Polyketide | 24.0 | 84.7 | 69.0 | 3.02e-12 |
| ARP51720.1 | NAD/FAD\_monooxygenase | BGC0001741 | NRP+Polyketide | 25.0 | 88.4 | 67.0 | 1.36e-11 |
| ABF92878.1 | monooxygenase,\_FAD-binding | BGC0001831 | Polyketide | 25.0 | 82.5 | 66.0 | 1.5e-11 |
| AAD51810.1 | monooxygebase | BGC0000829 | Alkaloid | 24.0 | 90.1 | 65.0 | 6.02e-11 |
| QKG86310.1 | FAD\_binding\_domain\_protein | BGC0002254 | Polyketide | 25.0 | 89.6 | 65.0 | 6.12e-11 |
| QKG86303.1 | flavin\_monooxygenase | BGC0002254 | Polyketide | 23.0 | 93.2 | 62.0 | 3.53e-10 |
| BBI47419.1 | FAD\_monooxygenase | BGC0002258 | Polyketide | 24.0 | 84.4 | 62.0 | 4.83e-10 |
| ADM34142.1 | FAD\_binding\_domain\_protein | BGC0001084 | NRP+Terpene+Alkaloid | 26.0 | 82.3 | 60.0 | 2.46e-09 |
| ACZ87068.1 | putative\_monoxygenase | BGC0002732 | Polyketide | 27.0 | 89.9 | 59.0 | 3.13e-09 |
| AMK51267.1 | Hex10 | BGC0001376 | Polyketide | 27.0 | 90.1 | 57.0 | 1.72e-08 |
| BAZ95821.1 | monooxygenase,\_FAD\_binding\_domain\_cpaO | BGC0001563 | NRP+Polyketide | 24.0 | 84.9 | 57.0 | 2.26e-08 |
| AJO72705.1 | Monooxygenase | BGC0001381 | Polyketide | 26.0 | 81.4 | 57.0 | 2.92e-08 |
| EDU47080.1 | conserved\_hypothetical\_protein | BGC0002250 | Polyketide+NRP | 25.0 | 86.3 | 56.0 | 3e-08 |
| AAL33754.1 | putative\_monooxygenase/hydroxylase | BGC0000421 | NRP | 24.0 | 82.1 | 56.0 | 3.57e-08 |
| QSV12647.1 | AvmO1 | BGC0002456 | Polyketide+NRP | 26.0 | 85.1 | 56.0 | 3.64e-08 |
| AXI91553.1 | FunA12 | BGC0001944 | Polyketide | 25.0 | 92.7 | 55.0 | 7.46e-08 |
| AFW04598.1 | monooxygenase | BGC0001783 | Other | 26.0 | 84.2 | 55.0 | 9.29e-08 |
| QIQ51367.1 | hypothetical\_protein | BGC0002199 | Alkaloid | 22.0 | 102.4 | 54.0 | 2.38e-07 |
| AWW87414.1 | FAD-binding\_monooxygenase | BGC0001755 | Polyketide | 25.0 | 90.3 | 53.0 | 3.89e-07 |
| AWM95786.1 | FAD-dependent\_monooxygenase | BGC0001827 | Polyketide | 22.0 | 81.1 | 53.0 | 3.99e-07 |
| ADY16696.1 | TqaH | BGC0001142 | NRP | 23.0 | 91.5 | 53.0 | 4.21e-07 |
| AGC83580.1 | FAD\_monooxygenase | BGC0000818 | NRP | 25.0 | 84.0 | 52.0 | 5.31e-07 |
| XP\_020057668.1 | uncharacterized\_protein | BGC0001718 | NRP | 24.0 | 90.1 | 52.0 | 9.6e-07 |
| CCE67072.1 | monooxygenase | BGC0001242 | Polyketide | 28.0 | 48.3 | 52.0 | 1.05e-06 |
| ATL73025.1 | oxidoreductase | BGC0001807 | NRP+Polyketide | 26.0 | 90.3 | 51.0 | 1.11e-06 |
| AHE14649.1 | FAD-binding\_monooxygenase | BGC0001336 | Alkaloid | 26.0 | 82.1 | 50.0 | 4.38e-06 |
| AGO65986.1 | putative\_monooxygenase | BGC0000992 | NRP+Polyketide | 23.0 | 101.2 | 49.0 | 6.51e-06 |
| ACT67684.1 | VioC | BGC0000831 | Alkaloid | 23.0 | 93.9 | 49.0 | 6.66e-06 |
| BBM95966.1 | monooxygenase | BGC0002558 | Polyketide | 26.0 | 90.1 | 49.0 | 7.32e-06 |
