# Supplementary material for: Uncovering the transcriptional landscape of Fomes fomentarius during fungal-based material production through gene co-expression network analysis
Source: Fungal Biol Biotechnol. 2025 Feb 13;12:1. doi: 10.1186/s40694-024-00192-3 (PMC11827164; doi:10.1186/s40694-024-00192-3)
Supplement: Supplementary file 1 — Supplementary Material 1 [file 40694_2024_192_MOESM1_ESM.zip › knownclusterblast/region1/jgi.p_Fomfom1_1238327_mibig_hits.html]

| MIBiG Protein | Description | MIBiG Cluster | MiBiG Product | % ID | % Coverage | BLAST Score | E-value |
| --- | --- | --- | --- | --- | --- | --- | --- |
| AGN71623.1 | hydroxylase | BGC0000027 | Polyketide:Iterative type I polyketide | 31.0 | 100.7 | 176.0 | 5.13e-50 |
| KFH44392.1 | Salicylate\_hydroxylase-like\_protein | BGC0002190 | Polyketide | 32.0 | 107.0 | 171.0 | 4e-48 |
| AWF83809.1 | Tropone\_2-monooxygenase | BGC0001487 | Other | 32.0 | 96.1 | 167.0 | 5e-47 |
| CAP95403.1 |  | BGC0001404 | Polyketide | 30.0 | 102.3 | 150.0 | 1.73e-40 |
| EED18000.1 | FAD\_oxygenase | BGC0000154 | Polyketide:Iterative type I polyketide | 29.0 | 105.1 | 148.0 | 1.26e-39 |
| EAQ86391.1 | hypothetical\_protein | BGC0001405 | Polyketide | 30.0 | 99.8 | 146.0 | 5.27e-39 |
| OPB37949.1 | salicylate\_hydroxylase | BGC0002206 | Polyketide | 29.0 | 102.1 | 142.0 | 1.1e-37 |
| ALV82350.1 | salicylate\_hydroxylase | BGC0001370 | NRP | 31.0 | 91.6 | 137.0 | 3.92e-36 |
| QCF41210.1 | CcxS | BGC0002726 | Polyketide | 30.0 | 104.6 | 139.0 | 3.98e-36 |
| EAU31924.1 | conserved\_hypothetical\_protein | BGC0002267 | Polyketide | 30.0 | 100.0 | 138.0 | 4.67e-36 |
| EAU32816.1 | conserved\_hypothetical\_protein | BGC0000160 | Polyketide | 29.0 | 100.9 | 136.0 | 2.6e-35 |
| ADD82995.1 | PtnB3 | BGC0001156 | Terpene | 30.0 | 94.2 | 134.0 | 4.46e-35 |
| QBL56164.1 | monooxygenase | BGC0002376 | Polyketide | 30.0 | 90.7 | 134.0 | 1.12e-34 |
| ALA99209.1 | CreL | BGC0001295 | Other | 30.0 | 90.7 | 131.0 | 7.87e-34 |
| EHA28235.1 | hypothetical\_protein | BGC0001143 | Polyketide | 27.0 | 93.5 | 131.0 | 1.38e-33 |
| ACO31289.1 | PtmB3 | BGC0001140 | Terpene | 29.0 | 94.2 | 129.0 | 4.07e-33 |
| CAB38889.1 | Hexenoyl-S-ACP\_Monooxygenase\_(hcmO) | BGC0000315 | NRP:Lipopeptide:Ca+-dependent lipopeptide | 29.0 | 94.0 | 127.0 | 2.93e-32 |
| QCO93109.1 | monooxygenase | BGC0001976 | Terpene | 27.0 | 103.0 | 127.0 | 5.78e-32 |
| AWM95796.1 | salicylate\_hydroxylase | BGC0001827 | Polyketide | 26.0 | 104.9 | 122.0 | 3.73e-30 |
| CBF83145.1 | conserved\_hypothetical\_protein | BGC0001722 | Polyketide | 27.0 | 101.4 | 121.0 | 8.74e-30 |
| CDF96615.1 | FAD-dependent\_Baeyer—Villiger\_monooxygenase | BGC0001149 | NRP:Lipopeptide+Saccharide:Hybrid/tailoring saccharide | 29.0 | 88.4 | 110.0 | 2.71e-26 |
| QJQ82458.1 | BisD | BGC0002290 | Other | 30.0 | 87.2 | 109.0 | 5.46e-26 |
| ATV82119.1 | hydroxylase | BGC0001909 | Polyketide | 25.0 | 108.1 | 108.0 | 2.86e-25 |
| AME18017.1 | epoxidase | BGC0001378 | Polyketide:Enediyne type I polyketide | 31.0 | 89.8 | 106.0 | 4.47e-25 |
| ALJ99865.1 | FlsP | BGC0001904 | Polyketide | 30.0 | 90.7 | 105.0 | 1.08e-24 |
| AIZ66878.1 | FAD-dependent\_monooxygenase | BGC0002666 | NRP+Alkaloid | 28.0 | 87.0 | 105.0 | 1.2e-24 |
| WP\_038234862.1 | FAD-dependent\_monooxygenase | BGC0001873 | NRP:Lipopeptide | 28.0 | 88.9 | 103.0 | 5.62e-24 |
| AAG06716.1 | probable\_FAD-dependent\_monooxygenase | BGC0002037 | NRP | 27.0 | 90.5 | 100.0 | 6.27e-23 |
| CAM34356.1 | putative\_FAD-depending\_monooxygenase | BGC0000242 | Polyketide | 31.0 | 90.0 | 97.0 | 7.9e-22 |
| AEW95635.1 | FAD-dependent\_monooxygenase | BGC0002697 | NRP+Polyketide | 28.0 | 86.8 | 97.0 | 1.11e-21 |
| AMK92570.1 | FAD-dependent\_oxidoreductase | BGC0001377 | Polyketide | 29.0 | 90.0 | 97.0 | 1.22e-21 |
| PKY07891.1 | FAD\_binding\_domain-containing\_protein | BGC0001544 | NRP+Polyketide | 25.0 | 102.6 | 94.0 | 1.61e-20 |
| ACZ87042.1 | monooxygenase,\_FAD-binding\_protein | BGC0002732 | Polyketide | 28.0 | 89.8 | 94.0 | 1.78e-20 |
| EHA19293.1 | hypothetical\_protein | BGC0001124 | Polyketide | 26.0 | 88.2 | 93.0 | 1.97e-20 |
| AXO35220.1 | FAD-dependent\_oxidoreductase | BGC0001848 | Other | 28.0 | 90.7 | 92.0 | 5.63e-20 |
| QJS40191.1 | PhzS | BGC0002439 | Other | 28.0 | 94.7 | 92.0 | 8.59e-20 |
| FAA01293.1 | flavin-dependent\_monooxygenase\_PyvC | BGC0002210 | Polyketide+NRP | 26.0 | 90.5 | 87.0 | 4.29e-18 |
| AEE65479.1 | oxidoreductase | BGC0000223 | Polyketide:Type II polyketide | 27.0 | 91.2 | 86.0 | 5.08e-18 |
| AGP37409.1 | hypothetical\_protein | BGC0002386 | NRP+Polyketide | 27.0 | 89.8 | 86.0 | 5.32e-18 |
| UHY14128.1 | FAD-dependent\_monooxygenase | BGC0002671 | Polyketide | 26.0 | 87.5 | 85.0 | 1.1e-17 |
| OKJ62000.1 | FAD-dependent\_monooxygenase | BGC0002147 | NRP | 28.0 | 88.6 | 85.0 | 1.12e-17 |
| AYV61417.1 | salicylate\_hydroxylase | BGC0001965 | Other | 27.0 | 96.8 | 84.0 | 5.55e-17 |
| CAQ52625.1 | FAD-dependent\_mono\_oxygenase | BGC0001066 | Polyketide:Modular type I polyketide | 25.0 | 88.4 | 83.0 | 6.68e-17 |
| AAP85357.1 | putative\_monooxygenase | BGC0000233 | Polyketide | 26.0 | 87.0 | 82.0 | 1.33e-16 |
| QDJ94215.1 | SpzS | BGC0002561 | Alkaloid | 28.0 | 90.3 | 81.0 | 3e-16 |
| AFW04593.1 | FAD-dependent\_oxidoreductase | BGC0001783 | Other | 29.0 | 88.4 | 81.0 | 3.05e-16 |
| RZB16712.1 | FAD-binding\_protein | BGC0001850 | Other:Shikimate-derived | 27.0 | 96.1 | 79.0 | 9.44e-16 |
| AIT42124.1 | putative\_monooxygenase | BGC0001221 | Other:Phenazine | 28.0 | 91.2 | 77.0 | 5.8e-15 |
| ARE67860.1 | AbsH3 | BGC0001492 | Polyketide | 25.0 | 90.5 | 77.0 | 6.92e-15 |
| QJS40200.1 | Salicylate\_hydroxylase | BGC0002439 | Other | 25.0 | 88.6 | 75.0 | 2.45e-14 |
| AFW04561.1 | oxidoreductase | BGC0001783 | Other | 28.0 | 89.6 | 75.0 | 3.23e-14 |
| AIE54237.1 | PauY17 | BGC0001732 | Other | 26.0 | 87.0 | 73.0 | 9.61e-14 |
| AIE54184.1 | Pau17 | BGC0001731 | Other | 26.0 | 87.0 | 72.0 | 1.72e-13 |
| OQD69144.1 | hypothetical\_protein | BGC0002745 | Polyketide | 25.0 | 95.4 | 72.0 | 3.48e-13 |
| AKU20511.1 | hypothetical\_protein | BGC0002687 | Polyketide+NRP | 26.0 | 91.0 | 71.0 | 5.59e-13 |
| CCT67992.1 | bikaverin\_cluster-monooxygenase | BGC0000030 | Polyketide | 22.0 | 92.8 | 69.0 | 3.15e-12 |
| AWW87414.1 | FAD-binding\_monooxygenase | BGC0001755 | Polyketide | 26.0 | 92.6 | 65.0 | 4.53e-11 |
| BBI47419.1 | FAD\_monooxygenase | BGC0002258 | Polyketide | 26.0 | 86.8 | 65.0 | 6.88e-11 |
| ARP51720.1 | NAD/FAD\_monooxygenase | BGC0001741 | NRP+Polyketide | 25.0 | 89.1 | 60.0 | 1.81e-09 |
| WP\_063842922.1 | FAD-dependent\_monooxygenase | BGC0001653 | Polyketide | 27.0 | 91.9 | 58.0 | 8.75e-09 |
| AAQ82549.1 | FscO | BGC0000034 | NRP+Polyketide | 25.0 | 91.6 | 58.0 | 1.1e-08 |
| AJO72705.1 | Monooxygenase | BGC0001381 | Polyketide | 25.0 | 90.7 | 57.0 | 3.01e-08 |
| ADM34142.1 | FAD\_binding\_domain\_protein | BGC0001084 | NRP+Terpene+Alkaloid | 25.0 | 91.6 | 56.0 | 3.25e-08 |
| AAL33754.1 | putative\_monooxygenase/hydroxylase | BGC0000421 | NRP | 25.0 | 90.3 | 55.0 | 1.13e-07 |
| BAZ95821.1 | monooxygenase,\_FAD\_binding\_domain\_cpaO | BGC0001563 | NRP+Polyketide | 22.0 | 88.9 | 54.0 | 1.27e-07 |
| AAD51810.1 | monooxygebase | BGC0000829 | Alkaloid | 23.0 | 88.4 | 54.0 | 1.76e-07 |
| ACZ87068.1 | putative\_monoxygenase | BGC0002732 | Polyketide | 26.0 | 87.0 | 54.0 | 2.27e-07 |
| AXI91553.1 | FunA12 | BGC0001944 | Polyketide | 24.0 | 99.1 | 54.0 | 2.38e-07 |
| QTC09985.1 | FAD-dependent\_monooxygenase | BGC0002372 | Polyketide+Terpene+Alkaloid | 25.0 | 91.9 | 52.0 | 6.74e-07 |
| AGC83580.1 | FAD\_monooxygenase | BGC0000818 | NRP | 25.0 | 91.2 | 52.0 | 7.25e-07 |
| AMK51267.1 | Hex10 | BGC0001376 | Polyketide | 27.0 | 86.3 | 52.0 | 9.3e-07 |
| AGO65986.1 | putative\_monooxygenase | BGC0000992 | NRP+Polyketide | 24.0 | 90.5 | 51.0 | 1.67e-06 |
| AFW04598.1 | monooxygenase | BGC0001783 | Other | 24.0 | 98.6 | 50.0 | 3.77e-06 |
