# Supplementary material for: Uncovering the transcriptional landscape of Fomes fomentarius during fungal-based material production through gene co-expression network analysis
Source: Fungal Biol Biotechnol. 2025 Feb 13;12:1. doi: 10.1186/s40694-024-00192-3 (PMC11827164; doi:10.1186/s40694-024-00192-3)
Supplement: Supplementary file 1 — Supplementary Material 1 [file 40694_2024_192_MOESM1_ESM.zip › knownclusterblast/region1/jgi.p_Fomfom1_1195752_mibig_hits.html]

| MIBiG Protein | Description | MIBiG Cluster | MiBiG Product | % ID | % Coverage | BLAST Score | E-value |
| --- | --- | --- | --- | --- | --- | --- | --- |
| AKD43762.1 | HerP | BGC0001349 | NRP+Polyketide | 52.0 | 93.4 | 306.0 | 7.19e-104 |
| ANH11401.1 | SceF | BGC0001770 | Polyketide | 52.0 | 93.4 | 304.0 | 2.67e-103 |
| QGA70087.1 | peptidase | BGC0002517 | Polyketide | 51.0 | 93.4 | 303.0 | 1.08e-102 |
| UHH90019.1 | VicJ | BGC0002634 | Polyketide+NRP+Other | 51.0 | 93.4 | 298.0 | 7.35e-101 |
| BAD08367.1 | proline\_iminopeptidase | BGC0000167 | Polyketide | 51.0 | 93.4 | 296.0 | 5.96e-100 |
| ACO94497.1 | putative\_L-amino\_acid\_amidase/proline\_iminopeptidase | BGC0000097 | Polyketide:Modular type I polyketide | 51.0 | 93.7 | 296.0 | 8.84e-100 |
| SAI82909.1 | HrnP;\_Putative\_L-amino\_acid\_amidase/\_proline\_iminopeptidase;\_alpha/beta\_hydrolase\_fold;\_Pfam00561 | BGC0002101 | Polyketide | 50.0 | 93.7 | 294.0 | 5.05e-99 |
| BAR73018.1 | putative\_amidohydrolase | BGC0001194 | Polyketide | 50.0 | 93.4 | 293.0 | 7.55e-99 |
| BAO66540.1 | putative\_proline\_iminopeptidase | BGC0000042 | Polyketide | 51.0 | 93.4 | 290.0 | 7.36e-98 |
| ABP55219.1 | proline-specific\_peptidase | BGC0000142 | Polyketide | 49.0 | 93.4 | 285.0 | 1.84e-95 |
| ACO94469.1 | putative\_L-amino\_acid\_amidase/proline\_iminopeptidase | BGC0000029 | Polyketide:Modular type I polyketide | 48.0 | 93.4 | 283.0 | 1.32e-94 |
| ALA09360.1 | peptidase | BGC0001303 | Polyketide | 48.0 | 93.4 | 279.0 | 2.92e-93 |
| OAP25801.1 | L-amino\_acid\_amidase | BGC0001658 | Polyketide | 46.0 | 94.1 | 271.0 | 2.92e-90 |
| BAV56008.1 | amidohydrolase | BGC0001597 | Polyketide | 49.0 | 93.7 | 271.0 | 3.65e-90 |
| sipL5 | L-proline\_amide\_hydrolase | BGC0001452 | Polyketide | 46.0 | 93.4 | 259.0 | 1.7e-85 |
| AWR88413.1 | putative\_haloalkane\_dehalogenase | BGC0001522 | Polyketide | 46.0 | 93.4 | 259.0 | 1.7e-85 |
| CCA29204.1 | putative\_type-II\_thioesterase | BGC0000955 | NRP+Polyketide:Modular type I polyketide | 31.0 | 94.4 | 141.0 | 2.06e-39 |
