# Supplementary material for: Uncovering the transcriptional landscape of Fomes fomentarius during fungal-based material production through gene co-expression network analysis
Source: Fungal Biol Biotechnol. 2025 Feb 13;12:1. doi: 10.1186/s40694-024-00192-3 (PMC11827164; doi:10.1186/s40694-024-00192-3)
Supplement: Supplementary file 1 — Supplementary Material 1 [file 40694_2024_192_MOESM1_ESM.zip › knownclusterblast/region1/jgi.p_Fomfom1_1315426_mibig_hits.html]

| MIBiG Protein | Description | MIBiG Cluster | MiBiG Product | % ID | % Coverage | BLAST Score | E-value |
| --- | --- | --- | --- | --- | --- | --- | --- |
| QGA70087.1 | peptidase | BGC0002517 | Polyketide | 40.0 | 103.1 | 221.0 | 8.58e-71 |
| UHH90019.1 | VicJ | BGC0002634 | Polyketide+NRP+Other | 40.0 | 103.1 | 221.0 | 1.25e-70 |
| AKD43762.1 | HerP | BGC0001349 | NRP+Polyketide | 40.0 | 103.7 | 221.0 | 1.27e-70 |
| QBL56180.1 | L-amino\_acid\_amidase | BGC0002376 | Polyketide | 42.0 | 100.0 | 220.0 | 2.46e-70 |
| BAD08367.1 | proline\_iminopeptidase | BGC0000167 | Polyketide | 39.0 | 103.1 | 218.0 | 2e-69 |
| BAO66540.1 | putative\_proline\_iminopeptidase | BGC0000042 | Polyketide | 40.0 | 101.0 | 212.0 | 3.4e-67 |
| BAP34711.1 | proline\_iminopeptidase | BGC0000078 | Polyketide | 41.0 | 98.6 | 212.0 | 4.84e-67 |
| sipL5 | L-proline\_amide\_hydrolase | BGC0001452 | Polyketide | 39.0 | 101.0 | 207.0 | 1.93e-65 |
| ACO94497.1 | putative\_L-amino\_acid\_amidase/proline\_iminopeptidase | BGC0000097 | Polyketide:Modular type I polyketide | 39.0 | 103.1 | 206.0 | 6.5e-65 |
| AWR88413.1 | putative\_haloalkane\_dehalogenase | BGC0001522 | Polyketide | 40.0 | 101.0 | 205.0 | 1.54e-64 |
| ABP55219.1 | proline-specific\_peptidase | BGC0000142 | Polyketide | 38.0 | 95.9 | 205.0 | 2.24e-64 |
| ACO94469.1 | putative\_L-amino\_acid\_amidase/proline\_iminopeptidase | BGC0000029 | Polyketide:Modular type I polyketide | 38.0 | 103.7 | 205.0 | 2.74e-64 |
| SAI82909.1 | HrnP;\_Putative\_L-amino\_acid\_amidase/\_proline\_iminopeptidase;\_alpha/beta\_hydrolase\_fold;\_Pfam00561 | BGC0002101 | Polyketide | 38.0 | 103.1 | 204.0 | 3.66e-64 |
| ALA09360.1 | peptidase | BGC0001303 | Polyketide | 38.0 | 95.9 | 199.0 | 3.45e-62 |
| BAV56008.1 | amidohydrolase | BGC0001597 | Polyketide | 39.0 | 95.9 | 199.0 | 5.45e-62 |
| OAP25801.1 | L-amino\_acid\_amidase | BGC0001658 | Polyketide | 38.0 | 103.1 | 196.0 | 5e-61 |
