# Supplementary material for: Uncovering the transcriptional landscape of Fomes fomentarius during fungal-based material production through gene co-expression network analysis
Source: Fungal Biol Biotechnol. 2025 Feb 13;12:1. doi: 10.1186/s40694-024-00192-3 (PMC11827164; doi:10.1186/s40694-024-00192-3)
Supplement: Supplementary file 1 — Supplementary Material 1 [file 40694_2024_192_MOESM1_ESM.zip › knownclusterblast/region1/jgi.p_Fomfom1_1315433_mibig_hits.html]

| MIBiG Protein | Description | MIBiG Cluster | MiBiG Product | % ID | % Coverage | BLAST Score | E-value |
| --- | --- | --- | --- | --- | --- | --- | --- |
| KGO40485.1 | Taurine\_catabolism\_dioxygenase\_TauD/TfdA | BGC0001205 | Polyketide | 42.0 | 80.8 | 224.0 | 1.31e-69 |
| KGO40482.1 | Taurine\_catabolism\_dioxygenase\_TauD/TfdA | BGC0001205 | Polyketide | 39.0 | 84.8 | 210.0 | 3.05e-64 |
| BAV32142.1 | putative\_alpha-ketoglutarate-dependent\_taurine\_dioxygenase\_protein | BGC0001373 | Polyketide | 39.0 | 74.1 | 199.0 | 3.6e-60 |
| ABB69741.1 | PlaO1 | BGC0000654 | Terpene+Saccharide:Hybrid/tailoring saccharide | 36.0 | 70.8 | 177.0 | 1.19e-52 |
| ATL73039.1 | taurine\_dioxygenase | BGC0001807 | NRP+Polyketide | 37.0 | 70.1 | 170.0 | 1.59e-49 |
| KZM73522.1 | taurine\_dioxygenase | BGC0000632 | Terpene+Saccharide | 36.0 | 69.6 | 169.0 | 1.87e-49 |
| AGC09525.1 | dioxygenase | BGC0001183 | Polyketide | 37.0 | 69.3 | 169.0 | 2.96e-49 |
| DAC74131.1 | alpha-ketoglutarate-dependent\_dioxygenase | BGC0002019 | Terpene | 35.0 | 69.6 | 164.0 | 2.39e-47 |
| XP\_001827203.1 |  | BGC0001996 | Other | 37.0 | 71.1 | 155.0 | 2.07e-43 |
| QOV03412.1 | SpoG | BGC0002262 | Polyketide | 35.0 | 79.1 | 154.0 | 1.15e-42 |
| AQX14443.1 | monobactam\_CAS\_homolog | BGC0001671 | NRP | 34.0 | 70.3 | 144.0 | 5.58e-40 |
| AOZ21322.1 | SulO | BGC0001790 | NRP | 32.0 | 71.3 | 138.0 | 1.72e-37 |
| AWH12930.1 | StmO4 | BGC0001784 | Polyketide | 34.0 | 74.1 | 135.0 | 5.23e-36 |
