# Supplementary material for: Uncovering the transcriptional landscape of Fomes fomentarius during fungal-based material production through gene co-expression network analysis
Source: Fungal Biol Biotechnol. 2025 Feb 13;12:1. doi: 10.1186/s40694-024-00192-3 (PMC11827164; doi:10.1186/s40694-024-00192-3)
Supplement: Supplementary file 1 — Supplementary Material 1 [file 40694_2024_192_MOESM1_ESM.zip › knownclusterblast/region1/jgi.p_Fomfom1_827688_mibig_hits.html]

| MIBiG Protein | Description | MIBiG Cluster | MiBiG Product | % ID | % Coverage | BLAST Score | E-value |
| --- | --- | --- | --- | --- | --- | --- | --- |
| QYA95705.1 | GNAT\_family\_N-acetyltransferase | BGC0002676 | NRP | 36.0 | 88.2 | 108.0 | 4.51e-29 |
| AEA30249.1 | putative\_N-acetyltransferase | BGC0000429 | Polyketide+NRP:Cyclic depsipeptide | 34.0 | 88.2 | 101.0 | 2.08e-26 |
