# Supplementary material for: Uncovering the transcriptional landscape of Fomes fomentarius during fungal-based material production through gene co-expression network analysis
Source: Fungal Biol Biotechnol. 2025 Feb 13;12:1. doi: 10.1186/s40694-024-00192-3 (PMC11827164; doi:10.1186/s40694-024-00192-3)
Supplement: Supplementary file 1 — Supplementary Material 1 [file 40694_2024_192_MOESM1_ESM.zip › knownclusterblast/region1/jgi.p_Fomfom1_1269532_mibig_hits.html]

| MIBiG Protein | Description | MIBiG Cluster | MiBiG Product | % ID | % Coverage | BLAST Score | E-value |
| --- | --- | --- | --- | --- | --- | --- | --- |
| ESK96610.1 | hypothetical\_protein | BGC0002212 | Polyketide | 28.0 | 105.7 | 321.0 | 5.1e-93 |
| ASK38699.1 | putative\_nonribosomal\_peptide\_synthetase-like\_protein | BGC0001436 | Polyketide:Iterative type I polyketide | 27.0 | 96.1 | 303.0 | 1.14e-86 |
| EAU35432.1 | predicted\_protein | BGC0002734 | Polyketide | 27.0 | 94.8 | 289.0 | 7.31e-82 |
| CEF75881.1 |  | BGC0001600 | Polyketide | 27.0 | 99.5 | 279.0 | 2.79e-78 |
| EWG54274.1 | hypothetical\_protein | BGC0001190 | Polyketide | 26.0 | 102.1 | 261.0 | 3.01e-72 |
| KIA75587.1 | NRPS-like\_enzyme | BGC0002209 | Polyketide | 26.0 | 88.3 | 256.0 | 3.26e-70 |
| BAV19380.1 | NRPS-like\_enzyme | BGC0001390 | NRP+Polyketide | 25.0 | 102.4 | 248.0 | 1.04e-67 |
| AMJ52084.1 | lijE | BGC0002255 | Polyketide | 32.0 | 38.5 | 178.0 | 8.89e-45 |
| AGN71604.1 | conidial\_yellow\_pigment\_biosynthesis\_polyketide\_synthase | BGC0000027 | Polyketide:Iterative type I polyketide | 28.0 | 36.3 | 152.0 | 6.4e-37 |
| EAU31923.1 | hypothetical\_protein | BGC0002267 | Polyketide | 31.0 | 36.7 | 148.0 | 1.3e-35 |
| AUW31047.1 | PKS-like\_protein | BGC0002483 | Polyketide | 30.0 | 32.5 | 133.0 | 1.01e-33 |
| CAP95404.1 |  | BGC0001404 | Polyketide | 26.0 | 40.7 | 126.0 | 9.24e-29 |
| ABW71853.1 | nonribosomal\_peptide\_synthetase | BGC0000303 | NRP | 28.0 | 40.3 | 101.0 | 2.68e-21 |
| AAO23334.1 | NcpB | BGC0000397 | NRP | 21.0 | 97.6 | 94.0 | 7.78e-19 |
| AEA29644.1 | putative\_nonribosomal\_peptide\_synthetase\_and\_kinurenine\_monooxygenase | BGC0000409 | NRP | 28.0 | 31.5 | 90.0 | 1.33e-17 |
| AOC89001.1 | putative\_nonribosomal\_peptide\_synthetase | BGC0001652 | NRP | 26.0 | 44.9 | 88.0 | 3.69e-17 |
| ATD51280.1 | nonribosomal\_peptide\_synthase | BGC0001650 | NRP | 27.0 | 41.0 | 86.0 | 2.47e-16 |
| QRK05501.1 | myxochelin\_non-ribosomal\_peptide\_synthetase\_MxcG | BGC0002324 | NRP+Polyketide | 24.0 | 51.2 | 84.0 | 9.36e-16 |
| AAK57184.1 | MxaA | BGC0001022 | NRP+Polyketide | 27.0 | 28.5 | 80.0 | 1.09e-14 |
| ABF87167.1 | non-ribosomal\_peptide\_synthase\_MxcG | BGC0002492 | NRP | 26.0 | 46.9 | 79.0 | 3.19e-14 |
| KJY85279.1 | long-chain\_fatty\_acid--CoA\_ligase | BGC0002491 | NRP | 22.0 | 73.2 | 78.0 | 5.48e-14 |
| AWS21279.1 | type\_I\_polyketide\_synthase | BGC0001934 | Polyketide | 27.0 | 31.1 | 74.0 | 1.08e-12 |
| AZY91989.1 | polyketide\_synthase | BGC0002022 | Polyketide | 27.0 | 31.1 | 74.0 | 1.08e-12 |
| ACN39727.1 | SibD | BGC0000428 | NRP | 26.0 | 32.5 | 70.0 | 1.25e-11 |
| AEC14349.1 | nonribosomal\_peptide\_synthetase | BGC0000377 | NRP | 24.0 | 31.6 | 70.0 | 1.43e-11 |
| AGC45618.1 | non-ribosomal\_peptide\_synthetase | BGC0001394 | NRP+Polyketide | 25.0 | 30.4 | 70.0 | 1.65e-11 |
| ACN39015.1 | putative\_nonribosomal\_peptide\_synthetase\_TomB | BGC0000448 | NRP | 26.0 | 29.3 | 69.0 | 2.17e-11 |
| CDG76959.1 | non-ribosomal\_peptide\_synthetase,\_terminal\_component | BGC0000446 | NRP:Pyrrolobenzodiazepine | 23.0 | 37.4 | 69.0 | 2.81e-11 |
| ATJ04411.1 | NRPS,\_TomB\_binding | BGC0001637 | NRP | 24.0 | 37.4 | 69.0 | 2.81e-11 |
| EOY45602.1 | Adenylation\_and\_reductase\_domains\_containing\_protein | BGC0001168 | NRP | 24.0 | 39.5 | 64.0 | 1.12e-09 |
| AAT12283.1 | LtxA | BGC0000384 | NRP | 24.0 | 31.1 | 64.0 | 1.36e-09 |
| ATD51278.1 | nonribosomal\_peptide\_synthase | BGC0001650 | NRP | 25.0 | 29.5 | 61.0 | 9.24e-09 |
| BAT51067.1 | type\_I\_polyketide\_synthase | BGC0001296 | Polyketide | 24.0 | 47.2 | 61.0 | 1.16e-08 |
