# Supplementary material for: Uncovering the transcriptional landscape of Fomes fomentarius during fungal-based material production through gene co-expression network analysis
Source: Fungal Biol Biotechnol. 2025 Feb 13;12:1. doi: 10.1186/s40694-024-00192-3 (PMC11827164; doi:10.1186/s40694-024-00192-3)
Supplement: Supplementary file 1 — Supplementary Material 1 [file 40694_2024_192_MOESM1_ESM.zip › knownclusterblast/region1/jgi.p_Fomfom1_852943_mibig_hits.html]

| MIBiG Protein | Description | MIBiG Cluster | MiBiG Product | % ID | % Coverage | BLAST Score | E-value |
| --- | --- | --- | --- | --- | --- | --- | --- |
| CBF85175.1 | conserved\_hypothetical\_protein | BGC0000673 | Terpene | 36.0 | 46.8 | 174.0 | 4.6e-45 |
| EFJ68972.1 | ATPase,\_AAA\_family | BGC0000628 | RiPP:Thiopeptide | 30.0 | 31.6 | 64.0 | 8.59e-11 |
