# Supplementary material for: Uncovering the transcriptional landscape of Fomes fomentarius during fungal-based material production through gene co-expression network analysis
Source: Fungal Biol Biotechnol. 2025 Feb 13;12:1. doi: 10.1186/s40694-024-00192-3 (PMC11827164; doi:10.1186/s40694-024-00192-3)
Supplement: Supplementary file 1 — Supplementary Material 1 [file 40694_2024_192_MOESM1_ESM.zip › knownclusterblast/region1/jgi.p_Fomfom1_1203106_mibig_hits.html]

| MIBiG Protein | Description | MIBiG Cluster | MiBiG Product | % ID | % Coverage | BLAST Score | E-value |
| --- | --- | --- | --- | --- | --- | --- | --- |
| ESK96613.1 | polyketide\_synthase | BGC0002212 | Polyketide | 40.0 | 107.2 | 1186.0 | 0.0 |
| EJP62792.1 | polyketide\_synthase | BGC0001720 | Polyketide | 36.0 | 107.4 | 937.0 | 9.72e-306 |
| ESU09199.1 | hypothetical\_protein | BGC0002594 | Polyketide | 34.0 | 102.7 | 875.0 | 4.69e-284 |
| ADM79459.1 | PKS16\_protein | BGC0001266 | Polyketide | 29.0 | 103.1 | 732.0 | 9.62e-230 |
| EAU38791.1 | hypothetical\_protein | BGC0000161 | Polyketide:Iterative type I polyketide | 30.0 | 105.2 | 699.0 | 7.5e-218 |
| BAE61567.1 |  | BGC0002175 | Polyketide | 30.0 | 96.0 | 690.0 | 7.22e-214 |
| ETS82099.1 | hypothetical\_protein | BGC0002161 | Polyketide | 28.0 | 105.1 | 675.0 | 2.82e-208 |
| gene6 |  | BGC0001906 | Polyketide | 28.0 | 105.3 | 671.0 | 2.46e-206 |
| AAD38786.1 | polyketide\_synthase | BGC0001257 | Polyketide | 28.0 | 105.0 | 661.0 | 3.38e-203 |
| CBF74114.1 | Conidial\_yellow\_pigment\_biosynthesis\_polyketide\_synthase\_(PKS)(EC\_2.3.1.-)\_[Source:UniProtKB/Swiss-Prot;Acc:Q03149] | BGC0000107 | Polyketide | 30.0 | 97.9 | 659.0 | 1.19e-202 |
| QKG86295.1 | non-reducing\_polyketide\_synthase | BGC0002253 | Polyketide | 30.0 | 99.3 | 657.0 | 3.24e-202 |
| EHA55627.1 | conidial\_yellow\_pigment\_biosynthesis\_polyketide\_synthase | BGC0002154 | Polyketide | 28.0 | 99.0 | 656.0 | 3.08e-201 |
| AAN59953.1 | polyketide\_synthase\_1 | BGC0001258 | Polyketide | 28.0 | 103.7 | 652.0 | 4.55e-200 |
| QDK64760.1 | AshP | BGC0002301 | Polyketide | 30.0 | 97.6 | 650.0 | 1.84e-199 |
| BAE65965.1 |  | BGC0002236 | Polyketide | 29.0 | 103.0 | 647.0 | 2.08e-198 |
| EED21099.1 | polyketide\_synthase,\_putative | BGC0001578 | Polyketide | 28.0 | 104.8 | 647.0 | 2.56e-198 |
| ERF77221.1 | hypothetical\_protein | BGC0002215 | Polyketide | 29.0 | 97.3 | 635.0 | 8.09e-194 |
| KDB16994.1 | polyketide\_synthetase\_PksP | BGC0002177 | Polyketide | 30.0 | 97.4 | 631.0 | 1.05e-190 |
| XP\_028481820.1 | non-reducing\_polyketide\_synthase | BGC0001866 | Polyketide | 31.0 | 87.6 | 628.0 | 1.48e-190 |
| EGD99348.1 | polyketide\_synthase | BGC0001144 | Polyketide | 34.0 | 74.9 | 619.0 | 1.86e-190 |
| XP\_001798923.1 | polyketide\_synthase | BGC0001865 | Polyketide:Iterative type I polyketide | 29.0 | 95.9 | 618.0 | 2.18e-189 |
| CCE67070.1 | polyketide\_synthase | BGC0001242 | Polyketide | 31.0 | 86.5 | 623.0 | 7.46e-189 |
| AEN83889.1 | AdaA | BGC0000156 | Polyketide:Iterative type I polyketide | 33.0 | 74.1 | 613.0 | 2.83e-188 |
| EED57518.1 | polyketide\_synthase,\_putative | BGC0001446 | Polyketide:Iterative type I polyketide | 29.0 | 103.4 | 613.0 | 1.71e-186 |
| EAL84397.1 | polyketide\_synthase | BGC0001118 | Polyketide:Iterative type I polyketide | 32.0 | 71.4 | 606.0 | 5.05e-186 |
| ARU80380.1 | polyketide\_synthase | BGC0001542 | Polyketide | 28.0 | 99.1 | 611.0 | 4.59e-185 |
| KAF7597159.1 | hypothetical\_protein | BGC0002646 | Polyketide | 31.0 | 86.6 | 600.0 | 1.39e-183 |
| AUW31184.1 | putative\_type\_I\_PKS | BGC0001489 | Polyketide | 31.0 | 86.1 | 605.0 | 5.88e-183 |
| CCE31584.1 | polyketide\_synthase\_that\_catalyse\_the\_condensation\_of\_one\_acetyl-CoA\_and\_six\_malonyl-CoA\_resulting\_in\_formation\_of\_nor-rubrofusarin | BGC0001886 | Polyketide | 32.0 | 74.7 | 597.0 | 1.53e-182 |
| CBF70387.1 | polyketide\_synthase,\_putative\_(JCVI) | BGC0000684 | Polyketide | 32.0 | 74.5 | 594.0 | 2.12e-181 |
| EAU31624.1 | hypothetical\_protein | BGC0002592 | Polyketide | 30.0 | 82.4 | 589.0 | 9.72e-180 |
| EAA59563.1 | polyketide\_synthase | BGC0000057 | Polyketide:Iterative type I polyketide | 29.0 | 104.7 | 594.0 | 1.64e-179 |
| ESU07748.1 | hypothetical\_protein | BGC0002709 | Polyketide | 29.0 | 105.0 | 593.0 | 2.66e-179 |
| AKN45693.1 | polyketide\_synthase | BGC0001284 | Polyketide | 32.0 | 72.2 | 586.0 | 1.11e-178 |
| AAS90047.1 | PksA | BGC0000009 | Polyketide | 27.0 | 107.0 | 586.0 | 1.67e-176 |
| QBG38888.1 | nr-PKS | BGC0002062 | Polyketide | 32.0 | 71.9 | 580.0 | 2.91e-176 |
| AGC95321.1 | CurS2 | BGC0000045 | Polyketide | 29.0 | 104.1 | 583.0 | 8.99e-176 |
| AAS89999.1 | PksA | BGC0000007 | Polyketide | 27.0 | 107.0 | 583.0 | 1.24e-175 |
| QCL09091.1 | dmx-nrPKS | BGC0002063 | Polyketide:Iterative type I polyketide | 30.0 | 82.3 | 577.0 | 2.47e-175 |
| ADI24926.1 | VrtA | BGC0000168 | Polyketide:Iterative type I polyketide | 30.0 | 86.8 | 578.0 | 2.63e-175 |
| AGO59040.1 | PtaA | BGC0000121 | Polyketide | 30.0 | 80.7 | 576.0 | 3.58e-175 |
| AAS90093.1 | PksA | BGC0000006 | Polyketide | 27.0 | 107.1 | 582.0 | 4.38e-175 |
| KAF7526531.1 | hypothetical\_protein | BGC0002244 | Polyketide | 31.0 | 74.6 | 575.0 | 1.65e-174 |
| ACD39753.1 | non-reducing\_polyketide\_synthase | BGC0000076 | Polyketide | 29.0 | 96.0 | 579.0 | 1.89e-174 |
| ACD39762.1 | non-reducing\_polyketide\_synthase | BGC0000077 | Polyketide | 29.0 | 96.0 | 579.0 | 1.89e-174 |
| ACH72912.1 | AflC | BGC0000011 | Polyketide | 33.0 | 74.0 | 581.0 | 2.3e-174 |
| AAS90022.1 | PksA | BGC0000008 | Polyketide | 27.0 | 107.1 | 580.0 | 2.9e-174 |
| EAL89339.1 | polyketide\_synthase,\_putative | BGC0001403 | Polyketide | 30.0 | 81.5 | 572.0 | 1.62e-173 |
| BAE71314.1 | polyketide\_synthase | BGC0000004 | Polyketide | 28.0 | 99.4 | 575.0 | 2.66e-173 |
| QNT61260.1 | polyketide\_synthase | BGC0002507 | Polyketide | 31.0 | 71.4 | 570.0 | 4.2e-173 |
| CCT67991.1 | bikaverin\_cluster-polyketide\_synthase | BGC0000030 | Polyketide | 28.0 | 103.8 | 574.0 | 1.34e-172 |
| QLM00044.1 | polyketide\_synthase | BGC0002257 | Polyketide | 33.0 | 73.9 | 563.0 | 4.24e-170 |
| OAQ63055.1 | polyketide\_synthase | BGC0002187 | Polyketide | 28.0 | 100.1 | 565.0 | 4.73e-169 |
| AAC49191.1 | putative\_polyketide\_synthase | BGC0000152 | Polyketide | 32.0 | 70.1 | 564.0 | 1.79e-168 |
| CBD77748.1 | polyketide\_synthase | BGC0000974 | NRP+Polyketide | 33.0 | 67.9 | 560.0 | 2.41e-168 |
| PKX92308.1 | putative\_polyketide\_synthase | BGC0001988 | Polyketide | 30.0 | 81.2 | 558.0 | 2.6e-168 |
| ABB90282.1 | polyketide\_synthase | BGC0001057 | NRP+Polyketide | 29.0 | 80.6 | 561.0 | 5.99e-168 |
| AQA28563.1 | type\_I\_polyketide\_synthase | BGC0001663 | Polyketide | 37.0 | 53.1 | 546.0 | 2.77e-167 |
| ADI24953.1 | GsfA | BGC0000070 | Polyketide:Iterative type I polyketide | 29.0 | 85.9 | 554.0 | 4.42e-167 |
| BAP81867.1 | AndM | BGC0002612 | Terpene | 31.0 | 80.1 | 563.0 | 4.89e-167 |
| ACD39770.1 | non-reducing\_polyketide\_synthase | BGC0000134 | Polyketide | 28.0 | 100.3 | 559.0 | 5.37e-167 |
| CCE33500.1 | polyketide\_synthase\_that\_catalyse\_the\_condensation\_of\_one\_acetyl-CoA\_and\_six\_malonyl-CoA\_resulting\_in\_formation\_of\_nor-rubrofusarin | BGC0002596 | Polyketide | 28.0 | 103.7 | 560.0 | 6.3e-167 |
| CDM36726.1 | Beta-ketoacyl\_synthase | BGC0001360 | Polyketide | 31.0 | 78.3 | 561.0 | 1.64e-166 |
| AQW44888.1 | polyketide\_synthase | BGC0001737 | NRP+Polyketide | 36.0 | 60.2 | 563.0 | 3.19e-166 |
| QCF41201.1 | CcxJ | BGC0002726 | Polyketide | 32.0 | 68.5 | 550.0 | 1.75e-165 |
| AAK57187.1 | MxaC | BGC0001022 | NRP+Polyketide | 35.0 | 60.0 | 560.0 | 6.11e-165 |
| BCD52390.1 | polyketide\_synthase\_SptM | BGC0002537 | Polyketide+Terpene | 30.0 | 80.1 | 556.0 | 1.2e-164 |
| CAD19091.1 | StiG\_protein | BGC0000153 | NRP+Polyketide:Modular type I polyketide | 34.0 | 63.1 | 538.0 | 2.6e-164 |
| AHV78253.1 | ResS2 | BGC0001246 | Polyketide | 27.0 | 106.6 | 552.0 | 3.61e-164 |
| ADF88280.1 | polyketide\_synthase | BGC0000981 | NRP+Polyketide | 35.0 | 59.1 | 548.0 | 4.01e-164 |
| CAD19087.1 | StiC\_protein | BGC0000153 | NRP+Polyketide:Modular type I polyketide | 34.0 | 62.6 | 547.0 | 7.24e-164 |
| AGC45620.1 | polyketide\_synthase | BGC0001394 | NRP+Polyketide | 36.0 | 60.3 | 555.0 | 1.56e-163 |
| ABX60162.1 | polyketide\_synthase | BGC0000978 | NRP+Alkaloid+Polyketide:Modular type I polyketide | 35.0 | 59.1 | 546.0 | 1.94e-163 |
| AIW82279.1 | PuwB | BGC0001125 | NRP+Polyketide | 32.0 | 67.1 | 552.0 | 4.41e-163 |
| KKP00966.1 | RADS2\_nonreducing\_polyketide\_synthase | BGC0001854 | Polyketide:Iterative type I polyketide | 30.0 | 78.3 | 542.0 | 4.58e-163 |
| DAB41653.1 | polyketide\_synthase | BGC0001583 | Polyketide | 32.0 | 71.3 | 538.0 | 7.5e-163 |
| EED53479.1 | polyketide\_synthase,\_putative | BGC0001304 | Polyketide | 29.0 | 79.9 | 541.0 | 8.72e-163 |
| ACC80699.1 | beta-ketoacyl\_synthase | BGC0002677 | Other | 35.0 | 62.3 | 544.0 | 1.06e-162 |
| AZH23788.1 | MgcR | BGC0001970 | NRP+Polyketide | 34.0 | 61.5 | 551.0 | 1.32e-162 |
| BAE62229.1 |  | BGC0002237 | Polyketide | 36.0 | 54.7 | 541.0 | 6.47e-162 |
| AGC45619.1 | polyketide\_synthase | BGC0001394 | NRP+Polyketide | 32.0 | 73.0 | 545.0 | 6.86e-162 |
| ACC80700.1 | beta-ketoacyl\_synthase | BGC0002677 | Other | 36.0 | 56.3 | 540.0 | 1.04e-160 |
| AFV96138.1 | polyketide\_synthase | BGC0001064 | Polyketide:Modular type I polyketide+Polyketide:Type III polyketide | 35.0 | 53.1 | 523.0 | 1.39e-160 |
| ARU81118.1 | CylD | BGC0001566 | Polyketide | 35.0 | 53.1 | 523.0 | 1.39e-160 |
| WP\_051206795.1 | type\_I\_polyketide\_synthase | BGC0002624 | NRP+Polyketide | 35.0 | 54.5 | 518.0 | 5.67e-160 |
| QCP68966.1 | VatW | BGC0002296 | NRP+Polyketide | 36.0 | 55.9 | 543.0 | 1.13e-159 |
| ACC80701.1 | beta-ketoacyl\_synthase | BGC0002677 | Other | 34.0 | 59.5 | 534.0 | 1.48e-159 |
| QCP68974.1 | VatV | BGC0002296 | NRP+Polyketide | 35.0 | 56.0 | 528.0 | 4.43e-159 |
| CAD19092.1 | StiH\_protein | BGC0000153 | NRP+Polyketide:Modular type I polyketide | 37.0 | 52.5 | 528.0 | 5.5e-159 |
| ALI92655.1 | CitS\_citrinin\_polyketide\_synthase | BGC0001338 | Polyketide:Iterative type I polyketide | 30.0 | 81.7 | 540.0 | 6.32e-159 |
| WP\_018540604.1 | type\_I\_polyketide\_synthase | BGC0001332 | NRP+Polyketide | 37.0 | 52.5 | 514.0 | 1.3e-158 |
| ABX60153.1 | polyketide\_synthase | BGC0000978 | NRP+Alkaloid+Polyketide:Modular type I polyketide | 37.0 | 52.7 | 521.0 | 1.64e-158 |
| ADF88275.1 | polyketide\_synthase | BGC0000981 | NRP+Polyketide | 37.0 | 52.7 | 521.0 | 2.26e-158 |
| AGC45622.1 | polyketide\_synthase | BGC0001394 | NRP+Polyketide | 32.0 | 72.0 | 530.0 | 3.76e-158 |
| AQW44890.1 | polyketide\_synthase | BGC0001737 | NRP+Polyketide | 32.0 | 67.3 | 533.0 | 7.82e-158 |
| QBM78307.1 | polyketide\_synthase | BGC0002542 | Polyketide+NRP | 36.0 | 52.9 | 537.0 | 9.96e-158 |
| AHB82053.1 | polyketide\_synthase | BGC0001019 | NRP+Polyketide:Modular type I polyketide | 36.0 | 52.5 | 523.0 | 1e-157 |
| AEE88289.1 | CurA | BGC0000976 | NRP+Polyketide:Modular type I polyketide | 35.0 | 54.2 | 534.0 | 1.97e-157 |
| AAT70096.1 | CurA | BGC0001165 | NRP+Polyketide:Modular type I polyketide | 35.0 | 54.2 | 534.0 | 1.97e-157 |
| CAD19090.1 | StiF\_protein | BGC0000153 | NRP+Polyketide:Modular type I polyketide | 32.0 | 68.9 | 533.0 | 2.52e-157 |
| AMB48442.1 | polyketide\_synthase | BGC0001357 | Polyketide | 34.0 | 52.9 | 518.0 | 2.77e-157 |
| ABM21570.1 | crpB | BGC0000975 | NRP+Polyketide | 35.0 | 53.3 | 536.0 | 2.99e-157 |
| ADF88276.1 | polyketide\_synthase | BGC0000981 | NRP+Polyketide | 35.0 | 58.7 | 527.0 | 4.28e-157 |
| AAZ95017.1 | polyketide\_synthase | BGC0000048 | Polyketide | 36.0 | 52.5 | 533.0 | 4.5e-157 |
| BBF25315.1 | polyketide\_synthase | BGC0001923 | Terpene+Polyketide | 30.0 | 82.7 | 531.0 | 4.89e-157 |
| AZH23819.1 | MgiR | BGC0001971 | NRP+Polyketide | 35.0 | 53.0 | 529.0 | 7.92e-157 |
| AQW44893.1 | polyketide\_synthase | BGC0001737 | NRP+Polyketide | 32.0 | 71.6 | 526.0 | 8.44e-157 |
| AHB82064.1 | polyketide\_synthase | BGC0001231 | NRP+Polyketide:Modular type I polyketide | 36.0 | 52.8 | 521.0 | 9.04e-157 |
| AXN93597.1 | PuwB | BGC0001952 | NRP | 32.0 | 66.9 | 533.0 | 1.14e-156 |
| EWM63000.1 | non-ribosomal\_peptide\_synthetase | BGC0001328 | NRP:Cyclic depsipeptide+Polyketide:Modular type I polyketide | 37.0 | 52.5 | 514.0 | 1.21e-156 |
| AZH23789.1 | MgcI | BGC0001970 | NRP+Polyketide | 34.0 | 61.3 | 530.0 | 1.21e-156 |
| AHV78247.1 | LasS2 | BGC0001245 | Polyketide | 28.0 | 95.4 | 529.0 | 1.69e-156 |
| AEU11006.1 | NpnB | BGC0001029 | NRP+Polyketide | 36.0 | 53.8 | 533.0 | 3.85e-156 |
| ACR33078.1 | polyketide\_synthase | BGC0000017 | Alkaloid+Polyketide:Modular type I polyketide | 33.0 | 59.5 | 525.0 | 4.59e-156 |
| ADY00130.1 | polyketide\_synthase | BGC0000104 | Terpene+Polyketide:Iterative type I polyketide | 30.0 | 77.8 | 531.0 | 4.76e-156 |
| AEE88280.1 | CurJ | BGC0000976 | NRP+Polyketide:Modular type I polyketide | 33.0 | 62.9 | 530.0 | 5.13e-156 |
| AAT70105.1 | CurJ | BGC0001165 | NRP+Polyketide:Modular type I polyketide | 33.0 | 62.9 | 530.0 | 5.13e-156 |
| BBG67008.1 | polyketide\_synthase\_Sre6 | BGC0002604 | Polyketide | 30.0 | 88.7 | 524.0 | 6.23e-156 |
| BAV19379.1 | polyketide\_synthase | BGC0001390 | NRP+Polyketide | 29.0 | 86.4 | 528.0 | 7.41e-156 |
| AFY58526.1 | polyketide\_synthase\_family\_protein | BGC0002411 | NRP+Polyketide | 35.0 | 52.4 | 506.0 | 8.32e-156 |
| QCP68968.1 | VatM | BGC0002296 | NRP+Polyketide | 33.0 | 64.0 | 526.0 | 2.65e-155 |
| ABX60152.1 | polyketide\_synthase | BGC0000978 | NRP+Alkaloid+Polyketide:Modular type I polyketide | 35.0 | 58.7 | 521.0 | 6.43e-155 |
| MBV7329455.1 | amino\_acid\_adenylation\_domain-containing\_protein | BGC0002131 | Polyketide+NRP:Glycopeptide+Saccharide:Hybrid/tailoring saccharide | 35.0 | 58.8 | 528.0 | 6.71e-155 |
| AGC45624.1 | polyketide\_synthase | BGC0001394 | NRP+Polyketide | 32.0 | 71.9 | 525.0 | 1.12e-154 |
| QCP68970.1 | VatU | BGC0002296 | NRP+Polyketide | 35.0 | 53.0 | 521.0 | 1.53e-154 |
| WP\_052165465.1 | type\_I\_polyketide\_synthase | BGC0001327 | NRP:Cyclic depsipeptide+Polyketide:Modular type I polyketide | 37.0 | 52.5 | 515.0 | 2.05e-154 |
| ATX68115.1 | malonyl\_CoA-acyl\_carrier\_protein\_transacylase | BGC0001772 | Polyketide | 36.0 | 53.2 | 521.0 | 2.36e-154 |
| CAD19086.1 | StiB\_protein | BGC0000153 | NRP+Polyketide:Modular type I polyketide | 34.0 | 52.5 | 514.0 | 2.73e-154 |
| ctg1\_orf3 |  | BGC0001329 | Polyketide+NRP:Cyclic depsipeptide | 36.0 | 53.0 | 514.0 | 2.87e-154 |
| AZH23818.1 | MgiI | BGC0001971 | NRP+Polyketide | 33.0 | 61.3 | 523.0 | 2.99e-154 |
| CAQ18829.1 | polyketide\_synthase | BGC0000954 | NRP+Polyketide:Modular type I polyketide | 36.0 | 57.7 | 527.0 | 3.37e-154 |
| AAK57189.1 | MxaE | BGC0001022 | NRP+Polyketide | 31.0 | 72.2 | 519.0 | 3.52e-154 |
| AXN93610.1 | PuwB | BGC0001953 | NRP | 32.0 | 68.3 | 525.0 | 4.68e-154 |
| QCP68973.1 | VatL | BGC0002296 | NRP+Polyketide | 35.0 | 53.7 | 515.0 | 4.77e-154 |
| KIA75596.1 | polyketide\_synthase | BGC0002209 | Polyketide | 29.0 | 81.4 | 524.0 | 5.5e-154 |
| DAB41916.1 | ArzN\_-\_PKS\_(KS,\_AT,\_OMT,\_KR,\_ACP) | BGC0001884 | NRP+Polyketide | 36.0 | 54.1 | 519.0 | 6.81e-154 |
| MCF2150415.1 | Polyketide\_synthase | BGC0002625 | NRP+Polyketide | 35.0 | 52.5 | 520.0 | 8.71e-154 |
| ACB46195.1 | polyketide\_synthase | BGC0000989 | NRP+Polyketide | 34.0 | 59.2 | 526.0 | 8.83e-154 |
| EAU35431.1 | hypothetical\_protein | BGC0002734 | Polyketide | 29.0 | 86.0 | 525.0 | 9.61e-154 |
| QDA77058.1 | polyketide\_synthase | BGC0002026 | NRP+Polyketide | 33.0 | 66.9 | 524.0 | 2.1e-153 |
| QBK15044.1 | clavatol\_synthase\_ClaF | BGC0002196 | Polyketide | 30.0 | 79.8 | 523.0 | 2.32e-153 |
| AEE88278.1 | CurL | BGC0000976 | NRP+Polyketide:Modular type I polyketide | 34.0 | 53.9 | 518.0 | 3.65e-153 |
| AAT70107.1 | CurL | BGC0001165 | NRP+Polyketide:Modular type I polyketide | 34.0 | 53.9 | 518.0 | 3.65e-153 |
| AAF62883.1 | epoD | BGC0000991 | NRP+Polyketide | 34.0 | 59.3 | 524.0 | 3.82e-153 |
| AQW44889.1 | polyketide\_synthase | BGC0001737 | NRP+Polyketide | 32.0 | 67.1 | 520.0 | 4.2e-153 |
| AFU82616.1 | polyketide\_synthase | BGC0000998 | NRP+Polyketide | 34.0 | 60.9 | 519.0 | 4.32e-153 |
| ATX68116.1 | malonyl\_CoA-acyl\_carrier\_protein\_transacylase | BGC0001772 | Polyketide | 37.0 | 53.1 | 520.0 | 6.23e-153 |
| QCQ67874.1 | type\_I\_polyketide\_synthase | BGC0002297 | NRP+Polyketide | 33.0 | 67.2 | 523.0 | 7.01e-153 |
| AJG44381.1 | MpaC' | BGC0002619 | Polyketide | 30.0 | 77.0 | 519.0 | 9e-153 |
| AAF26921.1 | polyketide\_synthase | BGC0000988 | NRP+Polyketide | 34.0 | 59.2 | 523.0 | 9.2e-153 |
| AKL71649.1 | NocP | BGC0001703 | Other | 30.0 | 70.9 | 504.0 | 9.53e-153 |
| AQA28562.1 | type\_I\_polyketide\_synthase | BGC0001663 | Polyketide | 31.0 | 68.4 | 521.0 | 9.78e-153 |
| AVI26388.1 | polyketide\_synthase | BGC0001800 | NRP+Polyketide | 35.0 | 58.1 | 522.0 | 1.03e-152 |
| BAV69313.1 | PrhL | BGC0001729 | Polyketide+Terpene | 30.0 | 79.9 | 521.0 | 1.3e-152 |
| ADB12491.1 | EpoD | BGC0000990 | NRP+Polyketide | 34.0 | 59.2 | 522.0 | 1.65e-152 |
| AAF19814.1 | MtaF | BGC0001024 | NRP+Polyketide:Modular type I polyketide | 35.0 | 52.7 | 503.0 | 4.58e-152 |
| AXN93577.1 | PuwB | BGC0001950 | NRP | 30.0 | 71.8 | 519.0 | 6.18e-152 |
| AVI26389.1 | polyketide\_synthase | BGC0001800 | NRP+Polyketide | 34.0 | 61.2 | 512.0 | 8.53e-152 |
| AXN93586.1 | PuwB | BGC0001951 | NRP | 30.0 | 71.8 | 518.0 | 1.12e-151 |
| AAS98783.1 | polyketide\_synthase/nonribosomal\_peptide\_synthase\_hybrid | BGC0001001 | NRP+Polyketide | 34.0 | 60.3 | 519.0 | 1.32e-151 |
| CAQ34920.1 | polyketide\_synthase | BGC0000986 | NRP+Polyketide | 33.0 | 67.2 | 511.0 | 1.32e-151 |
| DAB41915.1 | ArzM\_-\_PKS\_(KS,\_AT,\_DH,\_MT,\_ER,\_KR,\_ACP) | BGC0001884 | NRP+Polyketide | 34.0 | 61.0 | 517.0 | 2.87e-151 |
| ACR33079.1 | polyketide\_synthase | BGC0000017 | Alkaloid+Polyketide:Modular type I polyketide | 35.0 | 54.2 | 507.0 | 3.31e-151 |
| CBD77736.1 | polyketide\_synthase | BGC0000974 | NRP+Polyketide | 32.0 | 69.0 | 511.0 | 4.04e-151 |
| ADZ24997.1 | polyketide\_synthase | BGC0000380 | NRP+Polyketide:Modular type I polyketide | 31.0 | 72.8 | 510.0 | 4.79e-151 |
| AAU04878.1 | polyketide\_synthase | BGC0000365 | NRP | 33.0 | 61.5 | 517.0 | 5.4e-151 |
| CAQ18828.1 | polyketide\_synthase | BGC0000954 | NRP+Polyketide:Modular type I polyketide | 35.0 | 60.8 | 514.0 | 5.61e-151 |
| AQW44892.1 | polyketide\_synthase | BGC0001737 | NRP+Polyketide | 32.0 | 66.8 | 509.0 | 7.82e-151 |
| QCP68972.1 | VatE | BGC0002296 | NRP+Polyketide | 33.0 | 55.4 | 508.0 | 8.12e-151 |
| BAD38874.1 | polyketide\_synthase | BGC0000111 | Polyketide | 33.0 | 61.5 | 515.0 | 2e-150 |
| AAS98777.1 | polyketide\_synthetase | BGC0001001 | NRP+Polyketide | 34.0 | 52.8 | 506.0 | 3.53e-150 |
| AEE88282.1 | CurH | BGC0000976 | NRP+Polyketide:Modular type I polyketide | 35.0 | 57.3 | 511.0 | 4.21e-150 |
| AAT70103.1 | CurH | BGC0001165 | NRP+Polyketide:Modular type I polyketide | 35.0 | 57.3 | 511.0 | 4.21e-150 |
| CBF79143.1 | polyketide\_synthase,\_putative\_(JCVI) | BGC0000013 | Polyketide | 35.0 | 54.7 | 508.0 | 4.42e-150 |
| AAS98782.1 | polyketide\_synthase | BGC0001001 | NRP+Polyketide | 34.0 | 56.1 | 504.0 | 5.69e-150 |
| AXN93601.1 | PuwE | BGC0001952 | NRP | 35.0 | 56.0 | 514.0 | 6.5e-150 |
| QKW94285.1 | short-chain\_dehydrogenase/reductase\_SDR | BGC0002342 | NRP+Polyketide | 34.0 | 61.5 | 512.0 | 8.75e-150 |
| AAY42396.1 | Polyketide\_synthase | BGC0001000 | NRP:Lipopeptide+Polyketide:Modular type I polyketide | 35.0 | 52.6 | 507.0 | 1.1e-149 |
| AEU11005.1 | NpnA | BGC0001029 | NRP+Polyketide | 35.0 | 52.1 | 513.0 | 1.22e-149 |
| WP\_051206794.1 | type\_I\_polyketide\_synthase | BGC0002624 | NRP+Polyketide | 35.0 | 52.3 | 483.0 | 1.84e-149 |
| AXN93613.1 | PuwE | BGC0001953 | NRP | 34.0 | 55.5 | 511.0 | 3.74e-149 |
| AWS21279.1 | type\_I\_polyketide\_synthase | BGC0001934 | Polyketide | 36.0 | 52.9 | 497.0 | 4.19e-149 |
| AZY91989.1 | polyketide\_synthase | BGC0002022 | Polyketide | 36.0 | 52.9 | 497.0 | 4.19e-149 |
| KFA69335.1 | hypothetical\_protein | BGC0001626 | Polyketide | 28.0 | 86.4 | 509.0 | 4.4e-149 |
| AIW82282.1 | PuwE | BGC0001125 | NRP+Polyketide | 36.0 | 53.4 | 511.0 | 6.66e-149 |
| WP\_035121546.1 | type\_I\_polyketide\_synthase | BGC0001467 | NRP:Cyclic depsipeptide+Polyketide:Modular type I polyketide | 35.0 | 55.5 | 506.0 | 6.79e-149 |
| CCE88378.1 | polyketide\_synthase | BGC0001034 | NRP+Polyketide:Modular type I polyketide | 33.0 | 67.4 | 508.0 | 2.26e-148 |
| ADZ24998.1 | polyketide\_synthase | BGC0000380 | NRP+Polyketide:Modular type I polyketide | 31.0 | 67.1 | 506.0 | 2.63e-148 |
| QBM78312.1 | polyketide\_synthase | BGC0002542 | Polyketide+NRP | 30.0 | 66.5 | 508.0 | 6.5e-148 |
| MCF2150414.1 | Polyketide\_synthase | BGC0002625 | NRP+Polyketide | 32.0 | 61.3 | 507.0 | 7.9e-148 |
| AEE88281.1 | CurI | BGC0000976 | NRP+Polyketide:Modular type I polyketide | 34.0 | 54.2 | 498.0 | 8.9e-148 |
| AAT70104.1 | CurI | BGC0001165 | NRP+Polyketide:Modular type I polyketide | 34.0 | 54.2 | 498.0 | 8.9e-148 |
| CAD89777.1 | MelF\_protein | BGC0001010 | NRP+Polyketide:Modular type I polyketide | 35.0 | 52.8 | 491.0 | 1.26e-147 |
| AAF62884.1 | EpoE | BGC0000991 | NRP+Polyketide | 31.0 | 66.9 | 507.0 | 1.49e-147 |
| ADH01663.1 | putative\_polyketide\_synthase\_PKS3 | BGC0000099 | Polyketide | 29.0 | 87.9 | 505.0 | 4.05e-147 |
| ADB12492.1 | EpoE | BGC0000990 | NRP+Polyketide | 30.0 | 66.9 | 505.0 | 4.77e-147 |
| PKX88487.1 | polyketide\_synthase | BGC0001708 | Polyketide+Terpene | 30.0 | 80.3 | 503.0 | 6.19e-147 |
| AZH23791.1 | MgcH | BGC0001970 | NRP+Polyketide | 34.0 | 54.9 | 495.0 | 1.14e-146 |
| CAD19093.1 | StiJ\_protein | BGC0000153 | NRP+Polyketide:Modular type I polyketide | 34.0 | 54.9 | 486.0 | 1.44e-146 |
| ACB46196.1 | polyketide\_synthase | BGC0000989 | NRP+Polyketide | 30.0 | 66.9 | 504.0 | 1.53e-146 |
| AAF00959.1 | mcyD | BGC0001017 | NRP+Polyketide:Modular type I polyketide | 32.0 | 67.3 | 504.0 | 1.56e-146 |
| AQW44891.1 | polyketide\_synthase | BGC0001737 | NRP+Polyketide | 32.0 | 66.8 | 497.0 | 1.63e-146 |
| AAT70108.1 | CurM | BGC0001165 | NRP+Polyketide:Modular type I polyketide | 35.0 | 53.2 | 500.0 | 2.62e-146 |
| ACV42478.1 | polyketide\_synthase | BGC0000043 | Polyketide | 35.0 | 53.2 | 500.0 | 3.95e-146 |
| AEE88277.1 | CurM | BGC0000976 | NRP+Polyketide:Modular type I polyketide | 35.0 | 53.2 | 500.0 | 3.95e-146 |
| AAF19813.1 | MtaE | BGC0001024 | NRP+Polyketide:Modular type I polyketide | 35.0 | 55.4 | 496.0 | 7.76e-146 |
| CAJ46689.1 | polyketide\_synthase | BGC0000969 | NRP:Cyclic depsipeptide+Polyketide:Modular type I polyketide | 31.0 | 67.8 | 501.0 | 9.4e-146 |
| AAF26922.1 | polyketide\_synthase | BGC0000988 | NRP+Polyketide | 30.0 | 66.9 | 501.0 | 1.58e-145 |
| AXM42950.1 | polyketide\_synthase | BGC0001941 | NRP+Polyketide | 35.0 | 54.1 | 495.0 | 2.29e-145 |
| CCE88379.1 | polyketide\_synthase | BGC0001034 | NRP+Polyketide:Modular type I polyketide | 32.0 | 67.4 | 494.0 | 2.53e-145 |
| AUW31052.1 | putative\_type\_I\_PKS | BGC0002483 | Polyketide | 29.0 | 82.6 | 499.0 | 3.53e-145 |
| MBA0053739.1 | acyltransferase\_domain-containing\_protein | BGC0002096 | Polyketide | 35.0 | 54.2 | 487.0 | 4.84e-145 |
| AAW03329.1 | CtaF | BGC0000982 | NRP+Polyketide | 35.0 | 52.7 | 484.0 | 5.34e-145 |
| AFY58525.1 | polyketide\_synthase\_family\_protein | BGC0002411 | NRP+Polyketide | 35.0 | 52.4 | 488.0 | 7.05e-145 |
| AZH23821.1 | MgiH | BGC0001971 | NRP+Polyketide | 34.0 | 54.9 | 489.0 | 1.24e-144 |
| XP\_011392701.1 | uncharacterized\_protein | BGC0001281 | Polyketide | 29.0 | 76.7 | 493.0 | 1.53e-144 |
| WP\_102918845.1 | type\_I\_polyketide\_synthase | BGC0002104 | NRP+Polyketide | 33.0 | 66.8 | 498.0 | 2.26e-144 |
| AAW03328.1 | CtaE | BGC0000982 | NRP+Polyketide | 36.0 | 52.7 | 491.0 | 2.82e-144 |
| CAD29793.1 | polyketide\_synthase\_type\_I | BGC0001015 | NRP+Polyketide | 32.0 | 66.6 | 497.0 | 2.97e-144 |
| AIT55260.1 | polyketide\_synthase | BGC0000072 | Polyketide:Modular type I polyketide | 32.0 | 61.3 | 491.0 | 3.06e-144 |
| AHA12078.1 | polyketide\_synthase\_type\_1 | BGC0001172 | NRP+Polyketide:Modular type I polyketide | 36.0 | 54.1 | 494.0 | 1.79e-143 |
| CBF69451.1 | polyketide\_synthase,\_putative\_(JCVI) | BGC0000037 | Polyketide | 28.0 | 86.0 | 492.0 | 4.95e-143 |
| AAK57188.1 | MxaD | BGC0001022 | NRP+Polyketide | 31.0 | 67.3 | 487.0 | 5.59e-143 |
| AZH23790.1 | MgcG | BGC0001970 | NRP+Polyketide | 33.0 | 54.1 | 488.0 | 5.82e-143 |
| ABO15860.1 | polyketide\_synthase | BGC0000130 | Polyketide | 35.0 | 53.6 | 491.0 | 5.95e-143 |
| CBD77738.1 | polyketide\_synthase | BGC0000974 | NRP+Polyketide | 35.0 | 52.8 | 466.0 | 6.19e-143 |
| CCE88376.1 | polyketide\_synthase | BGC0001034 | NRP+Polyketide:Modular type I polyketide | 31.0 | 67.2 | 493.0 | 7.45e-143 |
| CAQ43077.1 | polyketide\_synthase | BGC0000970 | NRP+Polyketide:Modular type I polyketide | 30.0 | 72.4 | 479.0 | 8.43e-143 |
| ACR33077.1 | polyketide\_synthase | BGC0000017 | Alkaloid+Polyketide:Modular type I polyketide | 31.0 | 67.5 | 489.0 | 9.01e-143 |
| EHA28237.1 | hypothetical\_protein | BGC0001143 | Polyketide | 29.0 | 82.3 | 491.0 | 1.03e-142 |
| CAQ18830.1 | polyketide\_synthase | BGC0000954 | NRP+Polyketide:Modular type I polyketide | 33.0 | 61.6 | 489.0 | 1.39e-142 |
| AZH23820.1 | MgiG | BGC0001971 | NRP+Polyketide | 33.0 | 54.1 | 486.0 | 1.45e-142 |
| AEE88279.1 | CurK | BGC0000976 | NRP+Polyketide:Modular type I polyketide | 29.0 | 73.9 | 489.0 | 1.98e-142 |
| AAT70106.1 | CurK | BGC0001165 | NRP+Polyketide:Modular type I polyketide | 29.0 | 73.9 | 489.0 | 1.98e-142 |
| CAQ18833.1 | polyketide\_synthase | BGC0000954 | NRP+Polyketide:Modular type I polyketide | 36.0 | 52.9 | 481.0 | 2.05e-142 |
| AAF26923.1 | polyketide\_synthase | BGC0000988 | NRP+Polyketide | 32.0 | 67.1 | 490.0 | 2.41e-142 |
| AGC45623.1 | polyketide\_synthase | BGC0001394 | NRP+Polyketide | 31.0 | 67.0 | 485.0 | 3.95e-142 |
| CAD19088.1 | StiD\_protein | BGC0000153 | NRP+Polyketide:Modular type I polyketide | 34.0 | 55.0 | 485.0 | 4.78e-142 |
| CAD19089.1 | StiE\_protein | BGC0000153 | NRP+Polyketide:Modular type I polyketide | 35.0 | 52.7 | 485.0 | 5.05e-142 |
| ATY12793.1 | type\_I\_polyketide\_synthase | BGC0001504 | Polyketide | 32.0 | 67.2 | 486.0 | 8.43e-142 |
| CAQ18834.1 | polyketide\_synthase | BGC0000954 | NRP+Polyketide:Modular type I polyketide | 37.0 | 52.1 | 488.0 | 1.4e-141 |
| AAF62885.1 | EpoF | BGC0000991 | NRP+Polyketide | 31.0 | 67.5 | 488.0 | 1.41e-141 |
| QVV57686.1 | hypothetical\_protein | BGC0002338 | Polyketide | 32.0 | 60.2 | 488.0 | 2.14e-141 |
| EAU29529.1 | hypothetical\_protein | BGC0000682 | Terpene | 31.0 | 80.5 | 486.0 | 3.22e-141 |
| ADB12493.1 | EpoF | BGC0000990 | NRP+Polyketide | 31.0 | 67.7 | 486.0 | 3.41e-141 |
| AXN93580.1 | PuwE | BGC0001950 | NRP | 34.0 | 57.4 | 486.0 | 6.38e-141 |
| AXN93589.1 | PuwE | BGC0001951 | NRP | 34.0 | 57.4 | 486.0 | 6.38e-141 |
| CAO98879.1 | polyketide\_synthase\_AufD | BGC0000023 | Polyketide:Modular type I polyketide | 34.0 | 55.5 | 486.0 | 6.89e-141 |
| AHH34189.1 | polyketide\_synthase | BGC0001162 | Polyketide:Modular type I polyketide | 33.0 | 55.2 | 484.0 | 9.34e-141 |
| AVV61984.1 | type\_I\_modular\_polyketide\_synthase | BGC0001477 | NRP+Polyketide:Modular type I polyketide | 32.0 | 67.4 | 486.0 | 1.31e-140 |
| ACB46197.1 | polyketide\_synthase | BGC0000989 | NRP+Polyketide | 31.0 | 67.1 | 484.0 | 1.99e-140 |
| MBE8994630.1 | amino\_acid\_adenylation\_domain-containing\_protein | BGC0002623 | NRP+Polyketide | 33.0 | 52.8 | 484.0 | 2.27e-140 |
| CAQ34928.1 | polyketide\_synthase | BGC0000986 | NRP+Polyketide | 31.0 | 69.0 | 479.0 | 2.45e-140 |
| ctg1\_orf16 |  | BGC0001457 | NRP | 34.0 | 59.5 | 479.0 | 2.98e-140 |
| ART41209.1 | AdrD | BGC0001508 | Polyketide | 28.0 | 80.4 | 483.0 | 7.28e-140 |
| TGZ15166.1 | hypothetical\_protein | BGC0002032 | Polyketide | 34.0 | 61.9 | 477.0 | 1.01e-139 |
| QVV57685.1 | malonyl\_CoA-acyl\_carrier\_protein\_transacylase | BGC0002338 | Polyketide | 32.0 | 61.1 | 478.0 | 1.26e-139 |
| CAQ43078.1 | polyketide\_synthase | BGC0000970 | NRP+Polyketide:Modular type I polyketide | 31.0 | 72.2 | 477.0 | 2.27e-139 |
| AAK57186.1 | MxaB2 | BGC0001022 | NRP+Polyketide | 34.0 | 52.5 | 457.0 | 2.4e-139 |
| BCP96883.1 | non-reducing\_polyketide\_synthase | BGC0002614 | NRP+Polyketide | 28.0 | 85.6 | 479.0 | 2.83e-139 |
| CAD89776.1 | MelE\_protein | BGC0001010 | NRP+Polyketide:Modular type I polyketide | 35.0 | 53.3 | 477.0 | 3.28e-139 |
| ACR50791.1 | putative\_polyketide\_synthase | BGC0000163 | Polyketide | 33.0 | 59.9 | 478.0 | 4.04e-139 |
| AHH34186.1 | polyketide\_synthase | BGC0001161 | Polyketide:Modular type I polyketide | 33.0 | 54.3 | 479.0 | 6.15e-139 |
| AVV61980.1 | type\_I\_modular\_PKS | BGC0001477 | NRP+Polyketide:Modular type I polyketide | 31.0 | 66.8 | 481.0 | 7.77e-139 |
| EGJ35088.1 | Polyketide\_synthase | BGC0001163 | Polyketide:Modular type I polyketide | 33.0 | 55.6 | 478.0 | 1e-138 |
| CAC20931.1 | PimS1\_protein | BGC0000125 | Polyketide | 32.0 | 66.8 | 480.0 | 1.37e-138 |
| AQT01382.1 | SgnS1 | BGC0001690 | Polyketide | 32.0 | 66.8 | 480.0 | 1.37e-138 |
| AIT55263.1 | polyketide\_synthase | BGC0000072 | Polyketide:Modular type I polyketide | 31.0 | 66.9 | 478.0 | 1.55e-138 |
| ACN69988.1 | polyketide\_synthase | BGC0000079 | Polyketide | 36.0 | 53.8 | 479.0 | 2.14e-138 |
| AAF19812.1 | MtaD | BGC0001024 | NRP+Polyketide:Modular type I polyketide | 30.0 | 67.3 | 479.0 | 2.23e-138 |
| AGN71604.1 | conidial\_yellow\_pigment\_biosynthesis\_polyketide\_synthase | BGC0000027 | Polyketide:Iterative type I polyketide | 29.0 | 86.9 | 478.0 | 4.13e-138 |
| ARM20282.1 | polyketide\_synthase | BGC0001523 | Polyketide | 31.0 | 67.7 | 478.0 | 4.16e-138 |
| AHA12079.1 | polyketide\_synthase\_type\_1 | BGC0001172 | NRP+Polyketide:Modular type I polyketide | 35.0 | 52.7 | 466.0 | 5.42e-138 |
| AQW44873.1 | polyketide\_synthase | BGC0001761 | Polyketide | 31.0 | 66.6 | 472.0 | 5.63e-138 |
| AZH23817.1 | MgiQ | BGC0001971 | NRP+Polyketide | 30.0 | 61.0 | 477.0 | 7.81e-138 |
| QQZ01586.1 | PKS | BGC0002498 | Other | 31.0 | 67.0 | 478.0 | 7.99e-138 |
| CAP95404.1 |  | BGC0001404 | Polyketide | 29.0 | 80.7 | 476.0 | 9.68e-138 |
| ADN13832.1 | Polyketide\_Synthase | BGC0001164 | Polyketide:Modular type I polyketide | 34.0 | 52.9 | 476.0 | 1.4e-137 |
| AAS98784.1 | polyketide\_synthase | BGC0001001 | NRP+Polyketide | 33.0 | 53.2 | 453.0 | 2.71e-137 |
| BAW35615.1 | modular\_polyketide\_synthase | BGC0002357 | Polyketide+Other | 31.0 | 68.0 | 476.0 | 3.14e-137 |
| QQZ01588.1 | PKS | BGC0002498 | Other | 31.0 | 68.3 | 476.0 | 3.15e-137 |
| AGY30676.1 | Ann4 | BGC0001298 | Polyketide | 32.0 | 66.3 | 476.0 | 3.24e-137 |
| ABB05104.1 | LipPks3 | BGC0001003 | NRP:Lipopeptide+Polyketide:Modular type I polyketide+Saccharide:Hybrid/tailoring saccharide | 30.0 | 71.3 | 475.0 | 3.28e-137 |
| AXM42951.1 | polyketide\_synthase | BGC0001941 | NRP+Polyketide | 33.0 | 52.6 | 462.0 | 3.36e-137 |
| AJY78092.1 | polyketide\_synthase | BGC0001902 | NRP+Polyketide | 34.0 | 53.4 | 466.0 | 4.42e-137 |
| ACA99172.1 | polyketide\_synthase | BGC0001160 | Polyketide:Modular type I polyketide | 35.0 | 52.1 | 474.0 | 5.77e-137 |
| AGC45621.1 | polyketide\_synthase | BGC0001394 | NRP+Polyketide | 32.0 | 61.2 | 469.0 | 6.1e-137 |
| CAO98849.1 | polyketide\_synthase\_AufF | BGC0000023 | Polyketide:Modular type I polyketide | 34.0 | 54.1 | 474.0 | 7.37e-137 |
| ADX66472.1 | ScnS1 | BGC0000108 | Polyketide | 32.0 | 67.5 | 474.0 | 1.06e-136 |
| QCP68967.1 | VatT | BGC0002296 | NRP+Polyketide | 30.0 | 68.0 | 472.0 | 1.07e-136 |
| QVV57687.1 | polyketide\_synthase | BGC0002338 | Polyketide | 30.0 | 67.1 | 473.0 | 1.43e-136 |
| AKL64829.1 | polyketide\_synthase | BGC0002072 | Polyketide:Modular type I polyketide | 31.0 | 67.6 | 473.0 | 2.6e-136 |
| BAC68127.1 | modular\_polyketide\_synthase | BGC0000059 | Polyketide | 31.0 | 67.6 | 467.0 | 3.22e-136 |
| AZF85941.1 | type\_I\_polyketide\_synthase | BGC0001963 | NRP+Polyketide | 32.0 | 68.0 | 472.0 | 4.9e-136 |
| BAW35653.1 | modular\_polyketide\_synthase | BGC0002355 | Polyketide+Other | 33.0 | 61.0 | 472.0 | 6.02e-136 |
| BCB17026.1 | modular\_polyketide\_synthase | BGC0002523 | NRP | 33.0 | 60.8 | 471.0 | 9.81e-136 |
| AAS98781.1 | polyketide\_synthase | BGC0001001 | NRP+Polyketide | 28.0 | 68.5 | 471.0 | 9.84e-136 |
| CAQ34919.1 | polyketide\_synthase | BGC0000986 | NRP+Polyketide | 33.0 | 60.3 | 465.0 | 1.78e-135 |
| ALP32045.1 | CycE | BGC0001293 | Polyketide | 31.0 | 67.1 | 470.0 | 1.88e-135 |
| AEZ53946.1 | polyketide\_synthase | BGC0000144 | Polyketide:Modular type I polyketide | 31.0 | 72.2 | 470.0 | 2e-135 |
| AMB48441.1 | polyketide\_synthase | BGC0001357 | Polyketide | 32.0 | 53.3 | 467.0 | 2.11e-135 |
| QCP68965.1 | VatK | BGC0002296 | NRP+Polyketide | 28.0 | 68.5 | 469.0 | 3.11e-135 |
| AAQ90174.1 | polyketide\_synthase\_type\_I | BGC0000128 | Polyketide | 34.0 | 52.7 | 463.0 | 3.4e-135 |
| AFU82617.1 | polyketide\_synthase | BGC0000998 | NRP+Polyketide | 34.0 | 56.4 | 466.0 | 3.49e-135 |
| MBV7329454.1 | type\_I\_polyketide\_synthase | BGC0002131 | Polyketide+NRP:Glycopeptide+Saccharide:Hybrid/tailoring saccharide | 29.0 | 69.3 | 465.0 | 3.59e-135 |
| CAD17792.1 | probable\_non\_ribosomal\_peptide\_synthetase\_protein | BGC0001363 | NRP+Polyketide | 35.0 | 52.4 | 469.0 | 4.59e-135 |
| ALP32043.1 | CycC | BGC0001293 | Polyketide | 32.0 | 61.2 | 469.0 | 4.6e-135 |
| AMJ52084.1 | lijE | BGC0002255 | Polyketide | 29.0 | 83.6 | 468.0 | 5.83e-135 |
| AAA79984.2 | soraphen\_polyketide\_synthase\_B | BGC0000147 | Polyketide:Modular type I polyketide | 31.0 | 68.0 | 468.0 | 8.72e-135 |
| KFH44362.1 | Conidial\_yellow\_pigment\_biosynthesis\_polyketide\_synthase-like\_protein | BGC0002190 | Polyketide | 28.0 | 84.9 | 467.0 | 1.01e-134 |
| AEP40939.1 | polyketide\_synthase\_type\_I | BGC0000021 | Polyketide | 31.0 | 67.0 | 462.0 | 1.05e-134 |
| BAQ21946.1 | putative\_type\_I\_polyketide\_synthase | BGC0001204 | Polyketide | 32.0 | 67.6 | 468.0 | 1.07e-134 |
| AZH23787.1 | MgcQ | BGC0001970 | NRP+Polyketide | 30.0 | 61.6 | 467.0 | 1.11e-134 |
| ABO15861.1 | polyketide\_synthase | BGC0000130 | Polyketide | 34.0 | 53.5 | 466.0 | 1.11e-134 |
| CAQ43075.1 | polyketide\_synthase | BGC0000970 | NRP+Polyketide:Modular type I polyketide | 31.0 | 68.2 | 467.0 | 1.17e-134 |
| AID65222.1 | putative\_aspartate\_racemase | BGC0000335 | NRP+Polyketide | 34.0 | 52.2 | 467.0 | 1.58e-134 |
| BAO66528.1 | type\_I\_polyketide\_synthase | BGC0000042 | Polyketide | 32.0 | 60.6 | 446.0 | 1.79e-134 |
| ACB37741.1 | putative\_type\_I\_polyketide\_synthase | BGC0000162 | Polyketide | 33.0 | 60.3 | 466.0 | 6.23e-134 |
| BBG28471.1 | polyketide\_synthase\_Cle1 | BGC0002603 | Polyketide | 28.0 | 87.5 | 460.0 | 7.93e-134 |
| AUO16398.1 | polyketide\_synthase | BGC0001700 | Polyketide | 31.0 | 68.8 | 464.0 | 1.05e-133 |
| BAW35613.1 | modular\_polyketide\_synthase | BGC0002357 | Polyketide+Other | 31.0 | 67.0 | 464.0 | 1.35e-133 |
| EED18001.1 | NR-PKS | BGC0000154 | Polyketide:Iterative type I polyketide | 28.0 | 88.0 | 464.0 | 1.38e-133 |
| ABK32290.1 | JerD | BGC0000080 | Polyketide | 31.0 | 67.2 | 460.0 | 1.54e-133 |
| QQZ01582.1 | PKS | BGC0002498 | Other | 34.0 | 53.4 | 464.0 | 1.87e-133 |
| AVI57434.1 | AbmB2 | BGC0001694 | Polyketide | 31.0 | 68.5 | 463.0 | 2.79e-133 |
| BCB17027.1 | modular\_polyketide\_synthase | BGC0002523 | NRP | 34.0 | 54.3 | 463.0 | 3.19e-133 |
| ABP55210.1 | beta-ketoacyl\_synthase | BGC0000142 | Polyketide | 30.0 | 72.7 | 463.0 | 3.56e-133 |
| AAQ82564.1 | FscC | BGC0000034 | NRP+Polyketide | 31.0 | 68.3 | 463.0 | 3.87e-133 |
| EPH46608.1 | putative\_Erythronolide\_synthase,\_modules\_3\_and\_4 | BGC0001519 | NRP+Polyketide | 32.0 | 52.8 | 442.0 | 3.95e-133 |
| AAF71766.1 | nysI | BGC0000115 | Polyketide:Modular type I polyketide+Saccharide:Hybrid/tailoring saccharide | 35.0 | 54.3 | 463.0 | 5.06e-133 |
| CBF83139.1 | polyketide\_synthase,\_putative\_(JCVI) | BGC0001722 | Polyketide | 28.0 | 81.5 | 461.0 | 5.76e-133 |
| AVI57435.1 | AbmB3 | BGC0001694 | Polyketide | 33.0 | 52.9 | 442.0 | 7.45e-133 |
| AGC24270.1 | prlP | BGC0001038 | NRP+Polyketide:Modular type I polyketide | 34.0 | 56.2 | 456.0 | 9.4e-133 |
| QCO93110.1 | polyketide\_synthase | BGC0001976 | Terpene | 27.0 | 87.9 | 461.0 | 1.06e-132 |
| BAO66519.1 | type\_I\_polyketide\_synthase | BGC0000042 | Polyketide | 32.0 | 67.7 | 461.0 | 1.09e-132 |
| AVI57433.1 | AbmB1 | BGC0001694 | Polyketide | 34.0 | 56.4 | 462.0 | 1.09e-132 |
| BAW35612.1 | modular\_polyketide\_synthase | BGC0002357 | Polyketide+Other | 35.0 | 52.7 | 461.0 | 1.34e-132 |
| QWF78544.1 | 3-ketoacyl-CoA\_thiolase | BGC0002142 | Polyketide | 30.0 | 67.2 | 461.0 | 1.42e-132 |
| QIZ24098.1 | type\_I\_polyketide\_synthase | BGC0002540 | Polyketide | 35.0 | 52.5 | 461.0 | 2.14e-132 |
| AFV96142.1 | polyketide\_synthase | BGC0001064 | Polyketide:Modular type I polyketide+Polyketide:Type III polyketide | 33.0 | 52.8 | 459.0 | 2.81e-132 |
| ARU81122.1 | CylH | BGC0001566 | Polyketide | 33.0 | 52.8 | 459.0 | 2.81e-132 |
| ADX66461.1 | ScnS2 | BGC0000108 | Polyketide | 34.0 | 53.1 | 461.0 | 2.86e-132 |
| BAR73019.1 | putative\_PKS\_(KS-AT-DH-KR-ACP-TE) | BGC0001194 | Polyketide | 31.0 | 66.5 | 457.0 | 3.16e-132 |
| CAJ46690.1 | polyketide\_synthase | BGC0000969 | NRP:Cyclic depsipeptide+Polyketide:Modular type I polyketide | 31.0 | 67.7 | 458.0 | 3.44e-132 |
| CBD77746.1 | non-ribosomal\_peptide\_synthetase/polyketide\_synthase | BGC0000974 | NRP+Polyketide | 34.0 | 57.4 | 459.0 | 3.86e-132 |
| AAG23262.1 | polyketide\_synthase\_extender\_modules\_8-10 | BGC0000148 | Polyketide | 33.0 | 57.8 | 460.0 | 4.4e-132 |
| ABK32258.1 | AmbD | BGC0000014 | Polyketide | 30.0 | 68.1 | 456.0 | 4.69e-132 |
| ADX66459.1 | ScnS4 | BGC0000108 | Polyketide | 33.0 | 59.7 | 457.0 | 4.74e-132 |
| CBD77734.1 | polyketide\_synthase | BGC0000974 | NRP+Polyketide | 34.0 | 53.0 | 456.0 | 5.08e-132 |
| AAZ94388.1 | nodular\_polyketide\_synthase | BGC0000040 | Polyketide | 31.0 | 67.9 | 459.0 | 5.59e-132 |
| CAL58685.1 | polyketide\_synthase | BGC0000149 | Polyketide:Modular type I polyketide | 34.0 | 52.4 | 459.0 | 5.61e-132 |
| AAQ82566.1 | FscF | BGC0000034 | NRP+Polyketide | 33.0 | 56.3 | 456.0 | 7.52e-132 |
| ATG32075.1 | polyketide\_synthase | BGC0001750 | NRP+Polyketide | 29.0 | 74.2 | 454.0 | 7.89e-132 |
| CAC20919.1 | PimS4\_protein | BGC0000125 | Polyketide | 31.0 | 65.9 | 456.0 | 1.18e-131 |
| AQT01395.1 | SgnS4 | BGC0001690 | Polyketide | 31.0 | 65.9 | 456.0 | 1.18e-131 |
| AKL64831.1 | polyketide\_synthase | BGC0002072 | Polyketide:Modular type I polyketide | 31.0 | 67.2 | 458.0 | 1.38e-131 |
| BCB17031.1 | modular\_polyketide\_synthase | BGC0002523 | NRP | 31.0 | 68.6 | 458.0 | 1.41e-131 |
| ABX60161.1 | mixed\_NRPS/PKS | BGC0000978 | NRP+Alkaloid+Polyketide:Modular type I polyketide | 31.0 | 62.2 | 457.0 | 1.6e-131 |
| AIT55261.1 | polyketide\_synthase | BGC0000072 | Polyketide:Modular type I polyketide | 34.0 | 52.5 | 449.0 | 1.79e-131 |
| QQZ01585.1 | PKS | BGC0002498 | Other | 34.0 | 53.0 | 458.0 | 1.81e-131 |
| QWF78551.1 | hypothetical\_protein | BGC0002142 | Polyketide | 32.0 | 61.0 | 458.0 | 1.84e-131 |
| AAW03327.1 | CtaD | BGC0000982 | NRP+Polyketide | 30.0 | 73.6 | 457.0 | 2.42e-131 |
| BAD08360.1 | polyketide\_synthase\_modules\_7-8 | BGC0000167 | Polyketide | 30.0 | 67.3 | 457.0 | 2.72e-131 |
| ACY13415.1 | KR\_domain\_protein | BGC0001367 | NRP+Polyketide | 33.0 | 54.9 | 457.0 | 2.99e-131 |
| CAQ43079.1 | polyketide\_synthase | BGC0000970 | NRP+Polyketide:Modular type I polyketide | 33.0 | 54.3 | 453.0 | 3.18e-131 |
| QGA70079.1 | type\_I\_polyketide\_synthase | BGC0002517 | Polyketide | 31.0 | 67.8 | 457.0 | 3.46e-131 |
| WP\_032929422.1 | type\_I\_polyketide\_synthase | BGC0002106 | Polyketide | 31.0 | 67.3 | 457.0 | 3.53e-131 |
| AAX98186.1 | polyketide\_synthase\_type\_I | BGC0000052 | Polyketide | 32.0 | 59.1 | 457.0 | 3.53e-131 |
| QIQ28636.1 | Nbc40 | BGC0002541 | Other | 33.0 | 58.9 | 457.0 | 3.73e-131 |
| ADF88279.1 | mixed\_NRPS/PKS | BGC0000981 | NRP+Polyketide | 31.0 | 62.2 | 456.0 | 3.81e-131 |
| UHY14127.1 | PKS\_I | BGC0002671 | Polyketide | 34.0 | 53.1 | 456.0 | 4.37e-131 |
| AUO16422.1 | polyketide\_synthase | BGC0001700 | Polyketide | 30.0 | 67.9 | 456.0 | 4.49e-131 |
| AQH32481.1 | hybrid\_polyketide\_synthase/peptide\_synthetase | BGC0001667 | NRP+Polyketide | 34.0 | 52.4 | 456.0 | 4.55e-131 |
| AAZ77698.1 | ChlA5 | BGC0000036 | Polyketide:Modular type I polyketide+Polyketide:Iterative type I polyketide+Saccharide:Oligosaccharide | 31.0 | 67.3 | 456.0 | 4.94e-131 |
| BAY02135.1 | putative\_beta-ketoacyl\_synthase | BGC0002532 | NRP+Polyketide | 33.0 | 52.8 | 447.0 | 4.96e-131 |
| ABK32291.1 | JerE | BGC0000080 | Polyketide | 34.0 | 53.7 | 456.0 | 4.99e-131 |
| CAL58682.1 | polyketide\_synthase | BGC0000149 | Polyketide:Modular type I polyketide | 31.0 | 67.2 | 456.0 | 5.95e-131 |
| AHA38203.1 | GphJ | BGC0000069 | Polyketide | 35.0 | 53.4 | 451.0 | 6.36e-131 |
| CAO98850.1 | polyketide\_synthase\_AufG | BGC0000023 | Polyketide:Modular type I polyketide | 32.0 | 57.4 | 454.0 | 6.83e-131 |
| AVX51098.1 | nysI | BGC0001709 | Polyketide | 35.0 | 52.5 | 456.0 | 6.84e-131 |
| ANH11415.1 | SceT | BGC0001770 | Polyketide | 34.0 | 54.4 | 442.0 | 7.23e-131 |
| BAD08359.1 | polyketide\_synthase\_modules\_5-6 | BGC0000167 | Polyketide | 31.0 | 67.3 | 456.0 | 7.82e-131 |
| sipP3 | Type\_I\_Modular\_PKS | BGC0001452 | Polyketide | 31.0 | 67.5 | 456.0 | 8.32e-131 |
| PAU45552.1 | Iterative\_polyketide\_synthase | BGC0002138 | Polyketide | 29.0 | 79.9 | 443.0 | 8.7e-131 |
| ABV91286.1 | type\_I\_modular\_polyketide\_synthase | BGC0000158 | Polyketide:Modular type I polyketide | 31.0 | 60.6 | 456.0 | 9.13e-131 |
| QQZ01581.1 | PKS | BGC0002498 | Other | 33.0 | 58.0 | 456.0 | 1.18e-130 |
| ADZ24996.1 | polyketide\_synthase | BGC0000380 | NRP+Polyketide:Modular type I polyketide | 32.0 | 61.0 | 454.0 | 1.42e-130 |
| AEP40934.1 | polyketide\_synthase\_type\_I | BGC0000021 | Polyketide | 30.0 | 66.6 | 455.0 | 1.44e-130 |
| AAW03325.1 | CtaB | BGC0000982 | NRP+Polyketide | 31.0 | 53.0 | 436.0 | 1.54e-130 |
| ABI94379.1 | tautomycetin\_biosynthetic\_PKS | BGC0000157 | Polyketide | 31.0 | 60.6 | 455.0 | 1.63e-130 |
| AVI26390.1 | polyketide\_synthase\_/\_nonribosomal\_peptide\_synthase\_hybrid | BGC0001800 | NRP+Polyketide | 33.0 | 56.4 | 455.0 | 1.75e-130 |
| AEZ53952.1 | polyketide\_synthase | BGC0000144 | Polyketide:Modular type I polyketide | 29.0 | 77.1 | 454.0 | 2.01e-130 |
| ALA09371.1 | type\_I\_modular\_PKS | BGC0001303 | Polyketide | 33.0 | 57.2 | 455.0 | 2.02e-130 |
| BAW35641.1 | modular\_polyketide\_synthase | BGC0002356 | Polyketide+Other | 32.0 | 55.1 | 455.0 | 2.09e-130 |
| QKG20146.1 | Type\_I\_polyketide\_synthase | BGC0002124 | Polyketide | 33.0 | 57.6 | 454.0 | 2.51e-130 |
| CAD29794.1 | peptide\_synthetase | BGC0001015 | NRP+Polyketide | 34.0 | 52.5 | 454.0 | 2.55e-130 |
| OPB37950.1 | hypothetical\_protein | BGC0002206 | Polyketide | 29.0 | 81.8 | 454.0 | 2.61e-130 |
| WP\_030180235.1 | type\_I\_polyketide\_synthase | BGC0002106 | Polyketide | 35.0 | 52.6 | 454.0 | 2.89e-130 |
| AZH23823.1 | MgiK | BGC0001971 | NRP+Polyketide | 32.0 | 53.0 | 439.0 | 3.15e-130 |
| ABB05105.1 | LipPks4 | BGC0001003 | NRP:Lipopeptide+Polyketide:Modular type I polyketide+Saccharide:Hybrid/tailoring saccharide | 29.0 | 66.9 | 454.0 | 3.23e-130 |
| AFL48520.1 | laidlomycin\_polyketide\_synthase\_(module\_9) | BGC0000084 | Polyketide | 34.0 | 52.7 | 447.0 | 3.48e-130 |
| AWR88399.1 | putative\_beta-ketoacyl\_synthase | BGC0001522 | Polyketide | 31.0 | 67.5 | 454.0 | 3.51e-130 |
| BAR73020.1 | putative\_PKS\_(KS-AT-DH-KR-ACP-KS-AT-DH-KR-ACP-KS-AT-DH-KR-ACP) | BGC0001194 | Polyketide | 30.0 | 71.7 | 454.0 | 4.3e-130 |
| MBA0053740.1 | acyltransferase\_domain-containing\_protein | BGC0002096 | Polyketide | 34.0 | 54.1 | 453.0 | 5.33e-130 |
| AAO62582.1 | polyketide\_synthase\_peptide\_sythetase\_fusion\_protein | BGC0001016 | NRP+Polyketide | 34.0 | 52.4 | 452.0 | 8.07e-130 |
| AWC08655.1 | polyketide\_synthase\_type\_I | BGC0001662 | Polyketide | 32.0 | 55.3 | 453.0 | 8.84e-130 |
| CAC20921.1 | PimS2\_protein | BGC0000125 | Polyketide | 34.0 | 52.7 | 453.0 | 9.15e-130 |
| AQT01393.1 | SgnS2 | BGC0001690 | Polyketide | 34.0 | 52.7 | 453.0 | 9.15e-130 |
| AAZ77696.1 | ChlA3 | BGC0000036 | Polyketide:Modular type I polyketide+Polyketide:Iterative type I polyketide+Saccharide:Oligosaccharide | 32.0 | 59.2 | 452.0 | 1.06e-129 |
| BAJ16467.1 | polyketide\_synthase | BGC0000058 | Polyketide | 30.0 | 67.6 | 452.0 | 1.16e-129 |
| BAW35658.1 | modular\_polyketide\_synthase | BGC0002355 | Polyketide+Other | 30.0 | 67.5 | 452.0 | 1.16e-129 |
| AFL48527.1 | laidlomycin\_polyketide\_synthase\_(module\_3\_and\_module\_4) | BGC0000084 | Polyketide | 30.0 | 68.3 | 452.0 | 1.18e-129 |
| ABC84470.1 | NIGAVIII | BGC0000114 | Polyketide:Modular type I polyketide | 30.0 | 68.1 | 451.0 | 1.25e-129 |
| BAW35655.1 | modular\_polyketide\_synthase | BGC0002355 | Polyketide+Other | 33.0 | 53.5 | 452.0 | 1.37e-129 |
| AHN85651.1 | Phn2 | BGC0000122 | Polyketide:Modular type I polyketide | 32.0 | 61.0 | 452.0 | 1.53e-129 |
| AAF00958.1 | mcyE | BGC0001017 | NRP+Polyketide:Modular type I polyketide | 34.0 | 52.9 | 451.0 | 2.56e-129 |
| WP\_083502114.1 | type\_I\_polyketide\_synthase | BGC0001653 | Polyketide | 32.0 | 67.5 | 451.0 | 3.05e-129 |
| AAS98787.1 | polyketide\_synthase/thioesterase | BGC0001001 | NRP+Polyketide | 34.0 | 53.1 | 446.0 | 3.18e-129 |
| AMB20394.1 | polyketide\_synthase | BGC0002072 | Polyketide:Modular type I polyketide | 30.0 | 68.2 | 451.0 | 3.24e-129 |
| AIT55262.1 | polyketide\_synthase | BGC0000072 | Polyketide:Modular type I polyketide | 34.0 | 53.7 | 433.0 | 3.27e-129 |
| AUO16399.1 | polyketide\_synthase | BGC0001700 | Polyketide | 28.0 | 71.4 | 451.0 | 3.32e-129 |
| ctg1\_orf10 |  | BGC0000053 | Polyketide | 30.0 | 67.3 | 451.0 | 3.33e-129 |
| ATP76239.1 | NdaF | BGC0001705 | NRP+Polyketide | 34.0 | 52.4 | 451.0 | 3.41e-129 |
| AAX98184.1 | polyketide\_synthase\_type\_I | BGC0000052 | Polyketide | 33.0 | 55.3 | 451.0 | 4.98e-129 |
| QKV49769.1 | PKS | BGC0002526 | Polyketide | 32.0 | 61.0 | 450.0 | 5.84e-129 |
| AFP87523.1 | type\_I\_polyketide\_synthase | BGC0001159 | NRP+Polyketide:Modular type I polyketide | 34.0 | 53.7 | 449.0 | 6.23e-129 |
| AAQ82567.1 | FscE | BGC0000034 | NRP+Polyketide | 31.0 | 66.6 | 450.0 | 6.56e-129 |
| TXD00026.1 | SDR\_family\_NAD(P)-dependent\_oxidoreductase | BGC0001877 | Polyketide | 33.0 | 55.2 | 450.0 | 7.7e-129 |
| QQZ01629.1 | PKS | BGC0002497 | Other | 31.0 | 67.1 | 450.0 | 8.01e-129 |
| CAL58683.1 | polyketide\_synthase | BGC0000149 | Polyketide:Modular type I polyketide | 34.0 | 52.9 | 443.0 | 9.9e-129 |
| APZ78807.1 | polyketide\_synthase | BGC0001428 | NRP:Cyclic depsipeptide+Polyketide:Iterative type I polyketide | 30.0 | 67.8 | 447.0 | 1.08e-128 |
| AAG23266.1 | polyketide\_synthase\_extender\_modules\_3-4 | BGC0000148 | Polyketide | 33.0 | 53.2 | 449.0 | 1.32e-128 |
| APZ78727.1 | polyketide\_synthase | BGC0001421 | NRP:Cyclic depsipeptide+Polyketide:Iterative type I polyketide | 30.0 | 68.0 | 447.0 | 1.44e-128 |
| APZ78754.1 | polyketide\_synthase | BGC0001423 | NRP:Cyclic depsipeptide+Polyketide:Iterative type I polyketide | 30.0 | 67.7 | 447.0 | 1.44e-128 |
| BAG23199.1 | putative\_type-I\_PKS | BGC0002673 | Polyketide+Alkaloid | 32.0 | 61.5 | 447.0 | 1.58e-128 |
| QKG20147.1 | type\_I\_polyketide\_synthase | BGC0002124 | Polyketide | 30.0 | 66.8 | 449.0 | 1.88e-128 |
| BAC57029.1 | protomycinolide\_IV\_synthase\_2 | BGC0000102 | Polyketide | 32.0 | 60.2 | 444.0 | 1.93e-128 |
| AZH23793.1 | MgcK | BGC0001970 | NRP+Polyketide | 32.0 | 52.9 | 434.0 | 1.96e-128 |
| AAY28227.1 | HbmAIII | BGC0000074 | Polyketide | 30.0 | 73.2 | 448.0 | 2.07e-128 |
| QEA08889.1 | JenA3 | BGC0002559 | Polyketide | 29.0 | 72.7 | 448.0 | 2.12e-128 |
| BAQ21939.1 | putative\_type\_I\_polyketide\_synthase | BGC0001204 | Polyketide | 31.0 | 68.0 | 449.0 | 2.14e-128 |
| AUA09464.1 | Erythronolide\_synthase,\_modules\_3\_and\_4 | BGC0002291 | Polyketide | 31.0 | 62.3 | 447.0 | 3.34e-128 |
| ACR50774.1 | polyketide\_synthase | BGC0000163 | Polyketide | 29.0 | 67.5 | 448.0 | 3.34e-128 |
| APZ78780.1 | polyketide\_synthase | BGC0001426 | NRP:Cyclic depsipeptide+Polyketide:Iterative type I polyketide | 30.0 | 67.8 | 446.0 | 3.49e-128 |
| BCK51640.1 | modular\_polyketide\_synthase | BGC0002520 | Polyketide | 30.0 | 67.5 | 448.0 | 3.66e-128 |
| BAP34739.1 | type\_I\_polyketide\_synthase | BGC0000078 | Polyketide | 30.0 | 72.6 | 447.0 | 3.67e-128 |
| AKU20507.1 | polyketide\_synthase | BGC0002687 | Polyketide+NRP | 33.0 | 53.2 | 447.0 | 3.93e-128 |
| ADC45534.1 | modular\_polyketide\_synthase | BGC0000093 | Polyketide | 29.0 | 68.3 | 447.0 | 4.55e-128 |
| BAO66541.1 | type\_I\_polyketide\_synthase | BGC0000042 | Polyketide | 31.0 | 66.7 | 444.0 | 5.72e-128 |
| WP\_042799407.1 | SDR\_family\_NAD(P)-dependent\_oxidoreductase | BGC0001283 | Polyketide | 32.0 | 63.0 | 446.0 | 5.94e-128 |
| ABI93779.1 | GdmPKS | BGC0000068 | Polyketide | 28.0 | 72.9 | 446.0 | 7.16e-128 |
| QNS30807.1 | hybrid\_non-ribosomal\_peptide\_synthetase/type\_I\_polyketide\_syn-thase | BGC0002509 | NRP | 32.0 | 61.5 | 446.0 | 7.42e-128 |
| CAD89775.1 | MelD\_protein | BGC0001010 | NRP+Polyketide:Modular type I polyketide | 30.0 | 68.3 | 446.0 | 7.68e-128 |
| AAO06918.1 | GdmAIII | BGC0000066 | Polyketide | 30.0 | 73.1 | 446.0 | 8.76e-128 |
| ABB86410.1 | GelC | BGC0000067 | Polyketide | 30.0 | 72.9 | 446.0 | 1.17e-127 |
| BAR73021.1 | putative\_PKS\_(KS-AT-KR-ACP) | BGC0001194 | Polyketide | 32.0 | 54.8 | 439.0 | 1.34e-127 |
| BAG85027.1 | putative\_polyketide\_synthase | BGC0000086 | Polyketide | 31.0 | 60.8 | 446.0 | 1.39e-127 |
| CAQ64687.1 | lasalocid\_modular\_polyketide\_synthase | BGC0000087 | Polyketide | 31.0 | 60.8 | 446.0 | 1.39e-127 |
| ARM20278.1 | polyketide\_synthase | BGC0001523 | Polyketide | 29.0 | 67.2 | 446.0 | 1.39e-127 |
| AGM05531.1 | type\_I\_polyketide\_synthase | BGC0002098 | Polyketide | 34.0 | 56.2 | 446.0 | 1.54e-127 |
| AAC38076.1 | polyketide\_synthase\_type\_I | BGC0000127 | Polyketide | 32.0 | 55.0 | 441.0 | 1.59e-127 |
| DAB41918.1 | ArzP\_-\_PKS\_(KS,\_AT,\_OMT,\_ACP,\_TE) | BGC0001884 | NRP+Polyketide | 34.0 | 52.8 | 440.0 | 1.68e-127 |
| BAO66529.1 | type\_I\_polyketide\_synthase | BGC0000042 | Polyketide | 30.0 | 72.5 | 446.0 | 1.82e-127 |
| ABV83230.1 | CppC | BGC0000116 | Polyketide | 30.0 | 66.6 | 446.0 | 2.22e-127 |
| EFY96950.1 | BcPKS18,\_polyketide\_synthase | BGC0002427 | Terpene+Polyketide | 33.0 | 58.4 | 444.0 | 2.23e-127 |
| AWM95789.1 | non-reduciing\_polyketide\_synthase\_methylorcinaldehyde\_synthase | BGC0001827 | Polyketide | 26.0 | 89.1 | 444.0 | 2.64e-127 |
| ABB52545.1 | putative\_type\_I\_polyketide\_synthase | BGC0000047 | Polyketide | 33.0 | 56.9 | 434.0 | 2.77e-127 |
| AFI57006.1 | QmnA2 | BGC0000133 | Polyketide | 30.0 | 68.6 | 440.0 | 2.83e-127 |
| QDA77059.1 | polyketide\_synthase/nonribosomal\_peptide\_synthetase | BGC0002026 | NRP+Polyketide | 34.0 | 52.5 | 445.0 | 2.89e-127 |
| AEW95639.1 | type\_I\_polyketide\_synthase | BGC0002697 | NRP+Polyketide | 33.0 | 57.1 | 445.0 | 3.4e-127 |
| ABK32259.1 | AmbE | BGC0000014 | Polyketide | 31.0 | 61.2 | 444.0 | 3.42e-127 |
| QQZ01627.1 | PKS | BGC0002497 | Other | 32.0 | 57.1 | 445.0 | 3.51e-127 |
| ctg1\_orf11 |  | BGC0000053 | Polyketide | 31.0 | 61.9 | 442.0 | 3.79e-127 |
| QFU19826.1 | PKS | BGC0002431 | Polyketide+Saccharide | 31.0 | 62.2 | 442.0 | 3.84e-127 |
| ABI91466.1 | beta-ketoacyl\_synthase | BGC0001094 | NRP+Polyketide | 33.0 | 53.6 | 444.0 | 3.92e-127 |
| AVX51108.1 | nysC | BGC0001709 | Polyketide | 33.0 | 59.2 | 445.0 | 3.95e-127 |
| QGU18619.1 | polyketide\_synthase/non-ribosomal\_peptide\_synthetase | BGC0002365 | Other+Polyketide | 31.0 | 67.3 | 444.0 | 4.12e-127 |
| AAG23263.1 | polyketide\_synthase\_extender\_modules\_5-7 | BGC0000148 | Polyketide | 31.0 | 61.5 | 444.0 | 4.2e-127 |
| BAC68126.1 | modular\_polyketide\_synthase | BGC0000059 | Polyketide | 34.0 | 53.0 | 444.0 | 4.58e-127 |
| ABK32289.1 | JerC | BGC0000080 | Polyketide | 31.0 | 57.4 | 444.0 | 4.7e-127 |
| APZ78793.1 | polyketide\_synthase | BGC0001427 | NRP:Cyclic depsipeptide+Polyketide:Iterative type I polyketide | 30.0 | 68.0 | 442.0 | 4.97e-127 |
| AJD77023.1 | IkaA | BGC0001435 | NRP+Polyketide:Iterative type I polyketide | 31.0 | 69.3 | 444.0 | 5.48e-127 |
| CAD89773.1 | MelB\_protein | BGC0001010 | NRP+Polyketide:Modular type I polyketide | 31.0 | 53.2 | 426.0 | 5.59e-127 |
| AEU17897.1 | putative\_type\_I\_PKS | BGC0001072 | Saccharide+Polyketide:Modular type I polyketide+Polyketide:Type II polyketide+Other:Aminocoumarin | 30.0 | 69.0 | 442.0 | 5.63e-127 |
| AEC13079.1 | fosA | BGC0000060 | Polyketide | 32.0 | 57.2 | 444.0 | 5.78e-127 |
| AEK75502.1 | type\_1\_polyketide\_synthase | BGC0000001 | Polyketide:Modular type I polyketide | 33.0 | 57.4 | 444.0 | 5.97e-127 |
| CAQ34918.1 | nonribosomal\_peptide\_synthetase/\_polyketide\_synthase | BGC0000986 | NRP+Polyketide | 29.0 | 71.9 | 443.0 | 6.93e-127 |
| AKL64834.1 | polyketide\_synthase | BGC0002072 | Polyketide:Modular type I polyketide | 34.0 | 53.8 | 444.0 | 7.06e-127 |
| QKG20144.1 | type\_I\_polyketide\_synthase | BGC0002124 | Polyketide | 30.0 | 67.4 | 439.0 | 7.81e-127 |
| CAJ88186.1 | Type\_I\_modular\_polyketide\_synthase | BGC0000151 | Polyketide:Modular type I polyketide+Saccharide:Hybrid/tailoring saccharide | 30.0 | 66.8 | 443.0 | 1.09e-126 |
| BAQ21940.1 | putative\_Type\_I\_polyketide\_synthase | BGC0001204 | Polyketide | 31.0 | 57.1 | 443.0 | 1.15e-126 |
| AAO65801.1 | monensin\_polyketide\_synthase\_module\_9 | BGC0000100 | Polyketide | 33.0 | 53.8 | 437.0 | 1.19e-126 |
| ANZ52464.1 | MonAVI | BGC0001670 | Polyketide | 33.0 | 53.8 | 437.0 | 1.19e-126 |
| BAW35608.1 | modular\_polyketide\_synthase | BGC0002357 | Polyketide+Other | 32.0 | 52.9 | 443.0 | 1.28e-126 |
| ABK32257.1 | AmbC | BGC0000014 | Polyketide | 31.0 | 57.3 | 442.0 | 1.48e-126 |
| ABK32256.1 | AmbB | BGC0000014 | Polyketide | 32.0 | 53.8 | 442.0 | 1.49e-126 |
| AUO16397.1 | polyketide\_synthase | BGC0001700 | Polyketide | 29.0 | 68.1 | 442.0 | 1.56e-126 |
| QKV49767.1 | PKS | BGC0002526 | Polyketide | 32.0 | 60.7 | 442.0 | 1.58e-126 |
| QFU80901.1 | PKS | BGC0002550 | Polyketide | 31.0 | 60.8 | 442.0 | 1.59e-126 |
| BAO66542.1 | type\_I\_polyketide\_synthase | BGC0000042 | Polyketide | 29.0 | 71.8 | 442.0 | 1.81e-126 |
| AKD43763.1 | HerG | BGC0001349 | NRP+Polyketide | 30.0 | 66.9 | 440.0 | 1.92e-126 |
| BAJ16471.1 | polyketide\_synthase | BGC0000058 | Polyketide | 33.0 | 57.3 | 442.0 | 2.03e-126 |
| AQM37582.1 | polyketide\_synthase | BGC0001424 | NRP:Cyclic depsipeptide+Polyketide:Iterative type I polyketide | 30.0 | 67.6 | 441.0 | 2.08e-126 |
| TXD00265.1 | SDR\_family\_NAD(P)-dependent\_oxidoreductase | BGC0001877 | Polyketide | 29.0 | 74.2 | 442.0 | 2.12e-126 |
| AAS79463.1 | polyketide\_synthase\_subunit | BGC0000035 | Polyketide | 33.0 | 56.9 | 431.0 | 2.17e-126 |
| WP\_244927023.1 | type\_I\_polyketide\_synthase | BGC0002104 | NRP+Polyketide | 31.0 | 55.3 | 426.0 | 2.23e-126 |
| ARM20279.1 | polyketide\_synthase | BGC0001523 | Polyketide | 33.0 | 53.7 | 442.0 | 2.32e-126 |
| ADM46359.1 | polyketide\_synthase | BGC0000106 | Polyketide | 29.0 | 72.0 | 442.0 | 2.62e-126 |
| AHF22854.1 | MarL | BGC0000091 | Polyketide | 32.0 | 54.3 | 441.0 | 2.78e-126 |
| BAB69199.1 | modular\_polyketide\_synthase | BGC0000117 | Polyketide | 31.0 | 59.4 | 442.0 | 2.79e-126 |
| QIZ24099.1 | type\_I\_polyketide\_synthase | BGC0002540 | Polyketide | 33.0 | 56.5 | 437.0 | 2.82e-126 |
| AJW65410.1 | type\_I\_modular\_polyketide\_synthase | BGC0001195 | NRP+Polyketide | 33.0 | 56.7 | 437.0 | 3.01e-126 |
| BCB17028.1 | modular\_polyketide\_synthase | BGC0002523 | NRP | 34.0 | 52.9 | 442.0 | 3.18e-126 |
| QSV12661.1 | AvmC | BGC0002456 | Polyketide+NRP | 31.0 | 59.3 | 441.0 | 3.56e-126 |
| APZ78742.1 | polyketide\_synthase | BGC0001422 | NRP:Cyclic depsipeptide+Polyketide:Iterative type I polyketide | 30.0 | 68.0 | 440.0 | 3.74e-126 |
| TGZ15165.1 | hypothetical\_protein | BGC0002032 | Polyketide | 34.0 | 52.7 | 441.0 | 4.56e-126 |
| QGA70098.1 | type\_I\_polyketide\_synthase | BGC0002517 | Polyketide | 33.0 | 54.9 | 429.0 | 4.58e-126 |
| BBA84067.1 | type\_I\_polyketide\_synthase | BGC0001649 | Polyketide | 34.0 | 55.2 | 437.0 | 5.92e-126 |
| ANC94962.1 | AlmHV | BGC0001396 | Polyketide | 33.0 | 56.9 | 430.0 | 6.14e-126 |
| QWF78552.1 | 3-ketoacyl-CoA\_thiolase | BGC0002142 | Polyketide | 33.0 | 52.1 | 441.0 | 6.83e-126 |
| QHZ99323.1 | nargenicin\_PKS | BGC0001875 | Polyketide | 34.0 | 52.9 | 441.0 | 8e-126 |
| ASZ00148.1 | polyketide\_synthase | BGC0001785 | Polyketide | 30.0 | 72.4 | 440.0 | 8.19e-126 |
| ALP32042.1 | CycB | BGC0001293 | Polyketide | 30.0 | 62.3 | 439.0 | 8.58e-126 |
| APZ78714.1 | polyketide\_synthase | BGC0001420 | NRP:Cyclic depsipeptide+Polyketide:Iterative type I polyketide | 31.0 | 57.7 | 439.0 | 9.41e-126 |
| QKV49790.1 | PKS | BGC0002526 | Polyketide | 30.0 | 69.9 | 438.0 | 9.43e-126 |
| TMU97089.1 | SDR\_family\_NAD(P)-dependent\_oxidoreductase | BGC0002038 | Polyketide | 29.0 | 68.6 | 439.0 | 1.06e-125 |
| CQR60493.1 | Polyketide\_synthase,\_type\_I,\_modules:\_9\_and\_10 | BGC0001287 | Polyketide | 31.0 | 67.3 | 440.0 | 1.07e-125 |
| QGA70078.1 | type\_I\_polyketide\_synthase | BGC0002517 | Polyketide | 32.0 | 67.8 | 440.0 | 1.07e-125 |
| SAI82900.1 | HrnB;\_Macrolactam\_polyketide\_synthase\_type\_I;\_modules\_loading,\_1-2 | BGC0002101 | Polyketide | 32.0 | 61.2 | 440.0 | 1.08e-125 |
| ANH11409.1 | SceN | BGC0001770 | Polyketide | 33.0 | 56.7 | 440.0 | 1.1e-125 |
| ABK32288.1 | JerB | BGC0000080 | Polyketide | 32.0 | 53.2 | 440.0 | 1.11e-125 |
| ARM20280.1 | polyketide\_synthase | BGC0001523 | Polyketide | 31.0 | 61.0 | 440.0 | 1.17e-125 |
| QFU19840.1 | PKS | BGC0002431 | Polyketide+Saccharide | 31.0 | 67.3 | 440.0 | 1.18e-125 |
| APZ78690.1 | polyketide\_synthase | BGC0001418 | NRP:Cyclic depsipeptide+Polyketide:Iterative type I polyketide | 31.0 | 57.7 | 438.0 | 1.26e-125 |
| SCO70308.1 | Type\_I\_polyketide\_synthase | BGC0001433 | Polyketide:Modular type I polyketide | 30.0 | 67.9 | 440.0 | 1.28e-125 |
| QBL56181.1 | PKS | BGC0002376 | Polyketide | 29.0 | 66.8 | 439.0 | 1.5e-125 |
| TMV00153.1 | acyltransferase\_domain-containing\_protein | BGC0002038 | Polyketide | 29.0 | 70.6 | 439.0 | 1.58e-125 |
| AHB82070.1 | polyketide\_synthase | BGC0001231 | NRP+Polyketide:Modular type I polyketide | 33.0 | 52.4 | 431.0 | 1.71e-125 |
| BCB17032.1 | modular\_polyketide\_synthase | BGC0002523 | NRP | 29.0 | 69.1 | 439.0 | 1.82e-125 |
| ABK32263.1 | AmbH | BGC0000014 | Polyketide | 33.0 | 55.2 | 435.0 | 1.94e-125 |
| BAH02270.1 | polyketide\_synthase | BGC0000126 | Polyketide | 30.0 | 68.2 | 439.0 | 1.99e-125 |
| QOD94999.1 | PldAIV | BGC0002102 | Polyketide | 30.0 | 68.2 | 439.0 | 1.99e-125 |
| AAX98191.1 | polyketide\_synthase\_type\_I | BGC0000052 | Polyketide | 29.0 | 69.7 | 439.0 | 2.03e-125 |
| AFL48525.1 | laidlomycin\_polyketide\_synthase\_(loading\_module\_and\_module\_1) | BGC0000084 | Polyketide | 29.0 | 67.6 | 438.0 | 2.11e-125 |
| ADB12490.1 | EpoC | BGC0000990 | NRP+Polyketide | 29.0 | 68.2 | 435.0 | 3.13e-125 |
| AAF71767.1 | nysJ | BGC0000115 | Polyketide:Modular type I polyketide+Saccharide:Hybrid/tailoring saccharide | 32.0 | 60.6 | 439.0 | 3.25e-125 |
| ABK32255.1 | AmbA | BGC0000014 | Polyketide | 32.0 | 52.7 | 421.0 | 3.25e-125 |
| MBA5221219.1 | aminotransferase\_class\_I/II-fold\_pyridoxal\_phosphate-dependent\_enzyme | BGC0002090 | NRP+Polyketide:Modular type I polyketide | 34.0 | 53.8 | 438.0 | 3.41e-125 |
| AHH99921.1 | PKS\_I | BGC0000002 | Polyketide | 35.0 | 52.4 | 439.0 | 3.64e-125 |
| AAF26920.1 | polyketide\_synthase | BGC0000988 | NRP+Polyketide | 29.0 | 68.2 | 434.0 | 4.22e-125 |
| AEH42490.1 | polyketide\_synthase | BGC0000032 | Polyketide | 33.0 | 52.9 | 432.0 | 4.29e-125 |
| AAF71774.1 | nysA | BGC0000115 | Polyketide:Modular type I polyketide+Saccharide:Hybrid/tailoring saccharide | 33.0 | 56.8 | 427.0 | 4.53e-125 |
| SCN11949.1 | ebeA-type\_I\_polyketide\_synthase\_KSQ-ATa-ACP | BGC0001580 | Polyketide | 33.0 | 56.4 | 420.0 | 5.13e-125 |
| BCK51643.1 | modular\_polyketide\_synthase | BGC0002520 | Polyketide | 33.0 | 54.8 | 438.0 | 5.5e-125 |
| AHH99925.1 | PKS\_I | BGC0000002 | Polyketide | 32.0 | 56.2 | 438.0 | 5.56e-125 |
| ABK32287.1 | JerA | BGC0000080 | Polyketide | 31.0 | 53.3 | 421.0 | 5.74e-125 |
| QCF28927.1 | type\_I\_polyketide\_synthase | BGC0002308 | Alkaloid+Polyketide | 32.0 | 56.6 | 437.0 | 6.12e-125 |
| ABC87510.1 | polyketide\_synthase | BGC0001011 | NRP+Polyketide | 31.0 | 59.4 | 438.0 | 6.3e-125 |
| ctg1\_orf21 |  | BGC0001013 | NRP+Polyketide | 31.0 | 59.4 | 438.0 | 6.3e-125 |
| BAG17643.1 | putative\_NRPS-type-I\_PKS\_fusion\_protein | BGC0001043 | NRP+Polyketide | 30.0 | 67.2 | 437.0 | 7.28e-125 |
| QBF51757.1 | type\_I\_polyketide\_synthase | BGC0001856 | Polyketide:Modular type I polyketide | 34.0 | 53.6 | 437.0 | 7.59e-125 |
| ADM46360.1 | polyketide\_synthase | BGC0000106 | Polyketide | 33.0 | 60.5 | 437.0 | 8.03e-125 |
| BAJ16470.1 | polyketide\_synthase | BGC0000058 | Polyketide | 30.0 | 68.4 | 437.0 | 8.21e-125 |
| QQZ01583.1 | PKS | BGC0002498 | Other | 33.0 | 52.6 | 437.0 | 8.8e-125 |
| ctg1\_orf7 |  | BGC0000053 | Polyketide | 34.0 | 52.8 | 437.0 | 9.23e-125 |
| AUA09463.1 | Phenolphthiocerol\_synthesis\_polyketide\_synthase\_type\_I\_Pks15/1 | BGC0002291 | Polyketide | 32.0 | 59.5 | 435.0 | 9.72e-125 |
| CAE46850.1 | Type\_I\_modular\_polyketide\_synthase | BGC0000103 | Polyketide | 32.0 | 58.1 | 436.0 | 1.16e-124 |
| CAC22144.1 | CpkC;\_Polyketide\_synthase\_module\_5 | BGC0000038 | Polyketide:Modular type I polyketide | 30.0 | 67.2 | 435.0 | 1.24e-124 |
| AQH32483.1 | hybrid\_peptide\_synthetase/polyketide\_synthase | BGC0001667 | NRP+Polyketide | 32.0 | 54.8 | 436.0 | 1.39e-124 |
| AUO16400.1 | polyketide\_synthase | BGC0001700 | Polyketide | 32.0 | 55.1 | 436.0 | 1.71e-124 |
| APZ78854.1 | polyketide\_synthase | BGC0001432 | NRP:Cyclic depsipeptide+Polyketide:Iterative type I polyketide | 32.0 | 57.8 | 435.0 | 1.72e-124 |
| APZ78702.1 | polyketide\_synthase | BGC0001419 | NRP:Cyclic depsipeptide+Polyketide:Iterative type I polyketide | 31.0 | 57.7 | 435.0 | 1.73e-124 |
| EAQ86392.1 | hypothetical\_protein | BGC0001405 | Polyketide | 35.0 | 46.0 | 422.0 | 2.01e-124 |
| QEA08892.1 | JenA6 | BGC0002559 | Polyketide | 33.0 | 53.4 | 430.0 | 2.17e-124 |
| AAF71776.1 | nysC | BGC0000115 | Polyketide:Modular type I polyketide+Saccharide:Hybrid/tailoring saccharide | 30.0 | 66.9 | 436.0 | 2.18e-124 |
| AHB82051.1 | polyketide\_synthase | BGC0001019 | NRP+Polyketide:Modular type I polyketide | 29.0 | 73.5 | 436.0 | 2.37e-124 |
| AAF15892.2 | nosB | BGC0001028 | Polyketide+NRP:Cyclic depsipeptide | 33.0 | 53.1 | 423.0 | 2.4e-124 |
| AGZ15473.1 | putative\_type\_I\_polyketide\_synthase | BGC0001036 | NRP+Polyketide | 31.0 | 61.3 | 432.0 | 2.49e-124 |
| AEC13069.1 | fosC | BGC0000060 | Polyketide | 32.0 | 58.5 | 436.0 | 2.53e-124 |
| AHB82059.1 | non\_ribosomal\_peptide\_synthetase/polyketide\_synthase | BGC0001019 | NRP+Polyketide:Modular type I polyketide | 33.0 | 56.0 | 435.0 | 2.8e-124 |
| ARM20277.1 | polyketide\_synthase | BGC0001523 | Polyketide | 28.0 | 67.6 | 436.0 | 3.16e-124 |
| AJW65407.1 | type\_I\_modular\_polyketide\_synthase | BGC0001195 | NRP+Polyketide | 29.0 | 67.5 | 436.0 | 3.41e-124 |
| QKG20163.1 | type\_I\_polyketide\_synthase | BGC0002124 | Polyketide | 30.0 | 67.7 | 435.0 | 4.09e-124 |
| CAE46851.1 | Type\_I\_modular\_polyketide\_synthase | BGC0000103 | Polyketide | 32.0 | 58.1 | 436.0 | 4.09e-124 |
| CAA16183.1 | polyketide\_synthase | BGC0001063 | NRP+Polyketide | 33.0 | 53.5 | 434.0 | 4.17e-124 |
| ACN69991.1 | polyketide\_synthase | BGC0000079 | Polyketide | 30.0 | 68.2 | 434.0 | 4.24e-124 |
| BAR73017.1 | putative\_PKS\_(KS-AT-KR-ACP-KS-AT-DH-KR-ACP) | BGC0001194 | Polyketide | 32.0 | 67.2 | 435.0 | 4.27e-124 |
| WP\_019032756.1 | type\_I\_polyketide\_synthase | BGC0001331 | NRP:Cyclic depsipeptide+Polyketide:Modular type I polyketide | 39.0 | 38.3 | 416.0 | 4.34e-124 |
| EAU31923.1 | hypothetical\_protein | BGC0002267 | Polyketide | 29.0 | 79.5 | 434.0 | 4.62e-124 |
| AUA09467.1 | Erythronolide\_synthase,\_modules\_1\_and\_2 | BGC0002291 | Polyketide | 34.0 | 52.4 | 435.0 | 5.21e-124 |
| BCK51638.1 | modular\_polyketide\_synthase | BGC0002520 | Polyketide | 32.0 | 57.7 | 435.0 | 6.05e-124 |
| AKL64833.1 | polyketide\_synthase | BGC0002072 | Polyketide:Modular type I polyketide | 32.0 | 61.1 | 435.0 | 6.13e-124 |
| WP\_053065268.1 | type\_I\_polyketide\_synthase | BGC0001330 | NRP:Cyclic depsipeptide+Polyketide:Modular type I polyketide | 39.0 | 38.2 | 416.0 | 6.23e-124 |
| AAP42857.1 | NanA3 | BGC0000105 | Polyketide | 29.0 | 70.7 | 434.0 | 6.55e-124 |
| AEZ64503.1 | Herd | BGC0001065 | Polyketide | 30.0 | 66.8 | 432.0 | 8.1e-124 |
| AWR88393.1 | putative\_beta-ketoacyl\_synthase | BGC0001522 | Polyketide | 30.0 | 74.3 | 434.0 | 8.42e-124 |
| BAT51065.1 | type\_I\_polyketide\_synthase | BGC0001296 | Polyketide | 30.0 | 67.5 | 434.0 | 9.34e-124 |
| EPH46606.1 | putative\_Phenolphthiocerol\_synthesis\_polyketide\_synthase\_type\_I\_Pks15/1 | BGC0001519 | NRP+Polyketide | 31.0 | 61.3 | 431.0 | 9.74e-124 |
| ACR50785.1 | polyketide\_synthase | BGC0000163 | Polyketide | 29.0 | 70.3 | 434.0 | 9.8e-124 |
| ACB37740.1 | putative\_type\_I\_polyketide\_synthase | BGC0000162 | Polyketide | 29.0 | 70.6 | 434.0 | 1.07e-123 |
| QFU19842.1 | PKS | BGC0002431 | Polyketide+Saccharide | 31.0 | 59.1 | 432.0 | 1.13e-123 |
| AAP42860.1 | NanA6 | BGC0000105 | Polyketide | 33.0 | 53.9 | 428.0 | 1.17e-123 |
| QKV49766.1 | PKS | BGC0002526 | Polyketide | 31.0 | 59.9 | 432.0 | 1.19e-123 |
| AFD30954.1 | CrmA | BGC0000966 | NRP+Polyketide | 33.0 | 53.4 | 433.0 | 1.24e-123 |
| QIQ28635.1 | Nbc39 | BGC0002541 | Other | 32.0 | 54.0 | 433.0 | 1.29e-123 |
| WP\_035122279.1 | type\_I\_polyketide\_synthase | BGC0001467 | NRP:Cyclic depsipeptide+Polyketide:Modular type I polyketide | 33.0 | 53.1 | 430.0 | 1.36e-123 |
| AEZ64504.1 | Herc | BGC0001065 | Polyketide | 32.0 | 57.4 | 434.0 | 1.48e-123 |
| AVV61989.1 | beta-ketoacyl\_synthase | BGC0001477 | NRP+Polyketide:Modular type I polyketide | 30.0 | 66.9 | 431.0 | 1.51e-123 |
| BAJ16469.1 | polyketide\_synthase | BGC0000058 | Polyketide | 31.0 | 60.6 | 432.0 | 1.54e-123 |
| AAO65796.1 | monensin\_polyketide\_synthase\_loading\_module\_and\_module\_1 | BGC0000100 | Polyketide | 31.0 | 61.0 | 433.0 | 1.63e-123 |
| ANZ52459.1 | MonAI | BGC0001670 | Polyketide | 31.0 | 61.0 | 433.0 | 1.63e-123 |
| AAZ94387.1 | modular\_polyketide\_synthase | BGC0000040 | Polyketide | 32.0 | 56.2 | 433.0 | 1.7e-123 |
| BAJ16468.1 | polyketide\_synthase | BGC0000058 | Polyketide | 32.0 | 57.7 | 433.0 | 1.81e-123 |
| QCQ67877.1 | hybrid\_peptide\_synthetase/polyketide\_synthase | BGC0002297 | NRP+Polyketide | 32.0 | 53.6 | 432.0 | 1.84e-123 |
| AIT55259.1 | polyketide\_synthase | BGC0000072 | Polyketide:Modular type I polyketide | 32.0 | 55.4 | 426.0 | 1.88e-123 |
| AHB82062.1 | polyketide\_synthase | BGC0001231 | NRP+Polyketide:Modular type I polyketide | 31.0 | 61.0 | 433.0 | 1.88e-123 |
| CAM00064.1 | EryAII\_Erythromycin\_polyketide\_synthase\_modules\_3\_and\_4 | BGC0000055 | Polyketide:Modular type I polyketide+Saccharide:Hybrid/tailoring saccharide | 32.0 | 52.6 | 433.0 | 1.89e-123 |
| AAK83194.1 | polyketide\_synthase | BGC0000026 | Saccharide:Oligosaccharide | 33.0 | 52.8 | 421.0 | 1.94e-123 |
| TGZ15168.1 | hypothetical\_protein | BGC0002032 | Polyketide | 32.0 | 56.3 | 433.0 | 2.01e-123 |
| AAF62882.1 | EpoC | BGC0000991 | NRP+Polyketide | 29.0 | 68.2 | 429.0 | 2.03e-123 |
| OBR09781.1 | Polyketide\_synthase | BGC0002429 | Terpene+Polyketide | 32.0 | 58.4 | 432.0 | 2.04e-123 |
| ANR02554.1 | LodM | BGC0001648 | Polyketide | 32.0 | 56.7 | 432.0 | 2.09e-123 |
| QHZ99322.1 | nargenicin\_biosynthesis\_PKS | BGC0001875 | Polyketide | 30.0 | 67.4 | 433.0 | 2.25e-123 |
| ctg1\_orf15 |  | BGC0001457 | NRP | 34.0 | 52.6 | 431.0 | 2.5e-123 |
| sipP5 | Type\_I\_Modular\_PKS | BGC0001452 | Polyketide | 30.0 | 74.3 | 432.0 | 2.64e-123 |
| BAQ25512.1 | type\_I\_polyketide\_synthase | BGC0001288 | Polyketide | 29.0 | 68.9 | 433.0 | 2.64e-123 |
| BAQ25511.1 | type\_I\_polyketide\_synthase | BGC0001288 | Polyketide | 32.0 | 56.6 | 433.0 | 2.82e-123 |
| QPP46760.1 | polyketide\_synthase | BGC0002500 | Polyketide | 32.0 | 57.0 | 432.0 | 3.39e-123 |
| QLJ99331.2 | acyltransferase\_domain-containing\_protein | BGC0002088 | Polyketide+Saccharide:Oligosaccharide | 31.0 | 57.0 | 420.0 | 3.43e-123 |
| APZ78858.1 | polyketide\_synthase | BGC0001432 | NRP:Cyclic depsipeptide+Polyketide:Iterative type I polyketide | 33.0 | 51.0 | 431.0 | 3.47e-123 |
| ctg1\_orf254 |  | BGC0001200 | Polyketide | 31.0 | 60.5 | 432.0 | 3.57e-123 |
| AUO16401.1 | polyketide\_synthase | BGC0001700 | Polyketide | 31.0 | 61.5 | 432.0 | 3.61e-123 |
| ADZ24995.1 | non-ribosomal\_peptide\_synthase/polyketide\_synthase | BGC0000380 | NRP+Polyketide:Modular type I polyketide | 32.0 | 61.8 | 432.0 | 3.83e-123 |
| AAG23265.1 | polyketide\_synthase\_extender\_module\_2 | BGC0000148 | Polyketide | 32.0 | 57.1 | 431.0 | 4.05e-123 |
| AFV30249.1 | polyketide\_synthase | BGC0000075 | Polyketide | 30.0 | 67.7 | 429.0 | 4.17e-123 |
| BAE93729.1 | type\_I\_polyketide\_synthase | BGC0000164 | Polyketide | 32.0 | 53.5 | 432.0 | 4.31e-123 |
| QQZ01626.1 | PKS | BGC0002497 | Other | 33.0 | 52.7 | 432.0 | 4.41e-123 |
| ALV82345.1 | borrelidin\_type\_I\_polyketide\_synthase | BGC0001533 | Polyketide | 31.0 | 56.2 | 430.0 | 5.06e-123 |
| AEZ54375.1 | PieA2 | BGC0000124 | Polyketide | 32.0 | 56.3 | 431.0 | 5.7e-123 |
| CAL58687.1 | polyketide\_synthase | BGC0000149 | Polyketide:Modular type I polyketide | 33.0 | 54.5 | 431.0 | 5.96e-123 |
| AAM70355.1 | CalO5 | BGC0000033 | Polyketide | 31.0 | 57.0 | 419.0 | 6.29e-123 |
| QFU19827.1 | PKS | BGC0002431 | Polyketide+Saccharide | 31.0 | 59.7 | 428.0 | 6.33e-123 |
| QWF78549.1 | 3-ketoacyl-CoA\_thiolase | BGC0002142 | Polyketide | 32.0 | 53.7 | 432.0 | 6.37e-123 |
| ACY06286.1 | polyketide\_synthase | BGC0001042 | NRP+Polyketide | 30.0 | 61.0 | 430.0 | 6.38e-123 |
| AEH42491.1 | polyketide\_synthase | BGC0000032 | Polyketide | 31.0 | 59.9 | 431.0 | 6.4e-123 |
| AAP42856.1 | NanA2 | BGC0000105 | Polyketide | 31.0 | 57.4 | 430.0 | 6.47e-123 |
| ACC80698.1 | beta-ketoacyl\_synthase | BGC0002677 | Other | 32.0 | 53.6 | 415.0 | 7.05e-123 |
| ANR02555.1 | LodN | BGC0001648 | Polyketide | 30.0 | 67.5 | 431.0 | 7.51e-123 |
| APZ78844.1 | polyketide\_synthase | BGC0001431 | NRP:Cyclic depsipeptide+Polyketide:Iterative type I polyketide | 30.0 | 67.9 | 430.0 | 7.51e-123 |
| BAG23202.1 | putative\_type-I\_PKS | BGC0002673 | Polyketide+Alkaloid | 29.0 | 69.2 | 431.0 | 8.23e-123 |
| QPP46749.1 | polyketide\_synthase | BGC0002500 | Polyketide | 31.0 | 63.3 | 427.0 | 9.33e-123 |
| APZ78767.1 | polyketide\_synthase | BGC0001425 | NRP:Cyclic depsipeptide+Polyketide:Iterative type I polyketide | 30.0 | 67.8 | 429.0 | 9.66e-123 |
| AHH99926.1 | PKS\_I | BGC0000002 | Polyketide | 29.0 | 67.5 | 431.0 | 1.01e-122 |
| ACB46488.1 | polyketide\_synthase | BGC0000082 | Polyketide | 30.0 | 68.3 | 431.0 | 1.05e-122 |
| ACB46487.1 | polyketide\_synthase | BGC0000082 | Polyketide | 33.0 | 56.6 | 431.0 | 1.08e-122 |
| AAB66505.1 | tylactone\_synthase\_module\_3 | BGC0000166 | Polyketide | 31.0 | 69.8 | 427.0 | 1.11e-122 |
| BAP34740.1 | type\_I\_polyketide\_synthase | BGC0000078 | Polyketide | 32.0 | 55.2 | 426.0 | 1.23e-122 |
| QFU19839.1 | PKS | BGC0002431 | Polyketide+Saccharide | 32.0 | 56.9 | 430.0 | 1.53e-122 |
| AFU82614.1 | mixed\_NRPS\_PKS | BGC0000998 | NRP+Polyketide | 31.0 | 61.0 | 429.0 | 1.6e-122 |
| QSE03603.1 | LcmE | BGC0002333 | Polyketide | 31.0 | 66.4 | 428.0 | 1.69e-122 |
| ATY46594.1 | polyketide\_synthase | BGC0001666 | Polyketide | 31.0 | 55.7 | 412.0 | 1.71e-122 |
| AVV61985.1 | beta-ketoacyl\_synthase | BGC0001477 | NRP+Polyketide:Modular type I polyketide | 28.0 | 67.5 | 430.0 | 1.74e-122 |
| QBF51758.1 | type\_I\_polyketide\_synthase | BGC0001856 | Polyketide:Modular type I polyketide | 32.0 | 53.1 | 430.0 | 2.02e-122 |
| CAD29795.1 | peptide\_synthetase | BGC0001015 | NRP+Polyketide | 32.0 | 54.2 | 429.0 | 2.42e-122 |
| CBZ41585.1 | Type\_I\_modular\_polyketide\_synthase | BGC0000151 | Polyketide:Modular type I polyketide+Saccharide:Hybrid/tailoring saccharide | 29.0 | 67.3 | 429.0 | 2.48e-122 |
| ATY46595.1 | polyketide\_synthase | BGC0001666 | Polyketide | 32.0 | 57.6 | 426.0 | 2.53e-122 |
| QGA70099.1 | type\_I\_polyketide\_synthase | BGC0002517 | Polyketide | 31.0 | 67.0 | 429.0 | 3.07e-122 |
| CAE45671.1 | borrelidin\_polyketide\_synthase,\_type\_I | BGC0000031 | Polyketide:Modular type I polyketide | 31.0 | 56.2 | 428.0 | 3.11e-122 |
| QOV09193.1 | ClyE/NocP | BGC0002597 | NRP+Polyketide | 37.0 | 38.7 | 409.0 | 3.2e-122 |
| ABV83221.1 | CppI | BGC0000116 | Polyketide | 33.0 | 53.2 | 429.0 | 3.67e-122 |
| AEP40936.1 | polyketide\_synthase\_type\_I | BGC0000021 | Polyketide | 30.0 | 60.8 | 429.0 | 4.13e-122 |
| AHA38201.1 | GphH | BGC0000069 | Polyketide | 33.0 | 54.8 | 423.0 | 4.41e-122 |
| ADH04641.1 | TgaC | BGC0001051 | NRP+Polyketide:Modular type I polyketide | 33.0 | 52.7 | 429.0 | 4.48e-122 |
| QNN81297.1 | IonAI | BGC0002446 | Polyketide | 30.0 | 57.1 | 429.0 | 5.08e-122 |
| AXI91552.1 | FunP1 | BGC0001944 | Polyketide | 33.0 | 53.7 | 429.0 | 5.19e-122 |
| AAZ77693.1 | ChlA1 | BGC0000036 | Polyketide:Modular type I polyketide+Polyketide:Iterative type I polyketide+Saccharide:Oligosaccharide | 30.0 | 66.8 | 429.0 | 5.22e-122 |
| APZ78832.1 | polyketide\_synthase | BGC0001430 | NRP:Cyclic depsipeptide+Polyketide:Iterative type I polyketide | 30.0 | 65.4 | 427.0 | 5.67e-122 |
| AAO62585.1 | peptide\_sythetase\_polyketide\_synthase\_fusion\_protein | BGC0001016 | NRP+Polyketide | 32.0 | 53.9 | 428.0 | 5.7e-122 |
| APZ78678.1 | polyketide\_synthase | BGC0001417 | NRP:Cyclic depsipeptide+Polyketide:Iterative type I polyketide | 31.0 | 57.6 | 427.0 | 5.7e-122 |
| CAL58686.1 | polyketide\_synthase | BGC0000149 | Polyketide:Modular type I polyketide | 33.0 | 53.2 | 428.0 | 5.8e-122 |
| CAJ88176.1 | Type\_I\_modular\_polyketide\_synthase | BGC0000151 | Polyketide:Modular type I polyketide+Saccharide:Hybrid/tailoring saccharide | 29.0 | 67.5 | 428.0 | 5.98e-122 |
| AGY30677.1 | Ann5 | BGC0001298 | Polyketide | 31.0 | 60.2 | 428.0 | 6.18e-122 |
| ctg1\_orf524 |  | BGC0001199 | Polyketide | 33.0 | 53.8 | 417.0 | 6.87e-122 |
| SAI82911.1 | HrnF;\_Macrolactam\_polyketide\_synthase\_type\_I;\_modules\_6-7 | BGC0002101 | Polyketide | 32.0 | 56.4 | 428.0 | 7.49e-122 |
| ABI91470.1 | beta-ketoacyl\_synthase | BGC0001094 | NRP+Polyketide | 32.0 | 56.7 | 427.0 | 7.63e-122 |
| ACO94483.1 | polyketide\_synthase\_type\_I | BGC0000097 | Polyketide:Modular type I polyketide | 31.0 | 61.6 | 428.0 | 7.84e-122 |
| CAO85896.1 | protein\_modular\_polyketide\_synthase\_NorA' | BGC0000110 | Polyketide:Modular type I polyketide | 30.0 | 60.5 | 428.0 | 7.91e-122 |
| AKJ15836.1 | Type\_I\_polyketide\_synthase | BGC0002735 | Polyketide+NRP | 29.0 | 72.3 | 427.0 | 8.15e-122 |
| APD26279.1 | PtmA | BGC0001726 | NRP+Polyketide | 30.0 | 70.6 | 427.0 | 9.25e-122 |
| SAI82895.1 | HrnA1;\_Starter\_unit\_polyketide\_synthase\_type\_I;\_modules\_loading,\_1-3 | BGC0002101 | Polyketide | 32.0 | 57.0 | 428.0 | 1.04e-121 |
| WP\_012408784.1 | acyltransferase\_domain-containing\_protein | BGC0002061 | NRP:Cyclic depsipeptide+Polyketide:Modular type I polyketide | 32.0 | 53.9 | 416.0 | 1.11e-121 |
| QNN81301.1 | IonAV | BGC0002446 | Polyketide | 29.0 | 69.5 | 427.0 | 1.19e-121 |
| AGM05535.1 | modular\_polyketide\_synthase | BGC0002098 | Polyketide | 29.0 | 67.1 | 427.0 | 1.28e-121 |
| ATP76242.1 | NdaC | BGC0001705 | NRP+Polyketide | 32.0 | 54.2 | 427.0 | 1.36e-121 |
| CAG28678.1 | polyketide\_synthase | BGC0001023 | NRP+Polyketide:Modular type I polyketide | 32.0 | 57.7 | 426.0 | 1.36e-121 |
| APZ78820.1 | polyketide\_synthase | BGC0001429 | NRP:Cyclic depsipeptide+Polyketide:Iterative type I polyketide | 32.0 | 57.7 | 426.0 | 1.36e-121 |
| AFL48526.1 | laidlomycin\_polyketide\_synthase\_(module\_2) | BGC0000084 | Polyketide | 28.0 | 67.0 | 426.0 | 1.38e-121 |
| QES95478.1 | type\_I\_polyketide\_synthase | BGC0002453 | Polyketide | 29.0 | 74.8 | 424.0 | 1.44e-121 |
| WP\_018540593.1 | type\_I\_polyketide\_synthase | BGC0001332 | NRP+Polyketide | 31.0 | 67.7 | 427.0 | 1.49e-121 |
| QEA08888.1 | JenA2 | BGC0002559 | Polyketide | 31.0 | 58.1 | 426.0 | 1.59e-121 |
| ADU85988.1 | putative\_iterative\_type\_I\_polyketide\_synthase | BGC0000165 | Polyketide:Modular type I polyketide | 31.0 | 57.6 | 422.0 | 1.61e-121 |
| QHZ99321.1 | polyketide\_synthaase | BGC0001875 | Polyketide | 32.0 | 56.3 | 427.0 | 1.87e-121 |
| EAA65602.1 | hypothetical\_protein | BGC0000022 | Polyketide | 29.0 | 72.7 | 426.0 | 1.93e-121 |
| ABL86391.1 | hybrid\_polyketide\_synthase\_and\_nonribosomal\_peptide\_synthetase | BGC0000999 | NRP+Polyketide | 30.0 | 65.4 | 426.0 | 2.19e-121 |
| ACN69990.1 | polyketide\_synthase | BGC0000079 | Polyketide | 29.0 | 67.1 | 427.0 | 2.28e-121 |
| ACB46194.1 | polyketide\_synthase | BGC0000989 | NRP+Polyketide | 28.0 | 68.1 | 423.0 | 2.33e-121 |
| AAQ82565.1 | FscB | BGC0000034 | NRP+Polyketide | 30.0 | 60.9 | 427.0 | 2.34e-121 |
| AAX98185.1 | polyketide\_synthase\_type\_I | BGC0000052 | Polyketide | 32.0 | 53.5 | 426.0 | 2.38e-121 |
| AHE80994.1 | PieA4 | BGC0001169 | Polyketide:Modular type I polyketide | 29.0 | 66.9 | 425.0 | 2.48e-121 |
| ANI24099.1 | polyketide\_synthase | BGC0001235 | NRP+Polyketide | 31.0 | 59.5 | 427.0 | 2.49e-121 |
| CAD19085.1 | StiA\_protein | BGC0000153 | NRP+Polyketide:Modular type I polyketide | 32.0 | 52.3 | 426.0 | 2.59e-121 |
| QEA08890.1 | JenA4 | BGC0002559 | Polyketide | 29.0 | 67.7 | 426.0 | 2.61e-121 |
| AAQ82568.1 | FscD | BGC0000034 | NRP+Polyketide | 33.0 | 52.8 | 427.0 | 2.72e-121 |
| EHK80167.1 | modular\_polyketide\_synthase | BGC0001447 | Polyketide | 33.0 | 53.1 | 426.0 | 3.18e-121 |
| QKV49789.1 | PKS | BGC0002526 | Polyketide | 31.0 | 59.5 | 419.0 | 3.57e-121 |
| AQX77694.1 | NocP | BGC0001704 | Other | 37.0 | 38.7 | 406.0 | 3.97e-121 |
| WP\_081238290.1 | type\_I\_polyketide\_synthase | BGC0002105 | Polyketide | 29.0 | 66.9 | 422.0 | 4.17e-121 |
| ASZ00151.1 | polyketide\_synthase | BGC0001785 | Polyketide | 30.0 | 61.1 | 426.0 | 4.34e-121 |
| AAD03047.1 | type\_I\_polyketide\_synthase | BGC0000041 | Polyketide | 29.0 | 66.8 | 425.0 | 4.43e-121 |
| AAZ94386.1 | modular\_polyketide\_synthase | BGC0000040 | Polyketide | 32.0 | 55.7 | 425.0 | 4.66e-121 |
| AAQ90173.1 | polyketide\_synthase\_type\_I | BGC0000128 | Polyketide | 33.0 | 53.1 | 425.0 | 4.83e-121 |
| QEA08906.1 | JenA11 | BGC0002559 | Polyketide | 29.0 | 69.7 | 424.0 | 5.77e-121 |
| WP\_081238289.1 | type\_I\_polyketide\_synthase | BGC0002105 | Polyketide | 31.0 | 66.9 | 423.0 | 5.79e-121 |
| UMP03508.1 | NmvAIII | BGC0002649 | NRP+Polyketide | 33.0 | 51.9 | 419.0 | 6.23e-121 |
| TMU97099.1 | SDR\_family\_NAD(P)-dependent\_oxidoreductase | BGC0002038 | Polyketide | 31.0 | 62.3 | 425.0 | 6.3e-121 |
| QNN81303.1 | IonAVII | BGC0002446 | Polyketide | 28.0 | 68.6 | 424.0 | 6.45e-121 |
| AJW65409.1 | type\_I\_modular\_polyketide\_synthase | BGC0001195 | NRP+Polyketide | 29.0 | 67.6 | 425.0 | 6.99e-121 |
| QPP46758.1 | polyketide\_synthase | BGC0002500 | Polyketide | 30.0 | 68.0 | 425.0 | 7.71e-121 |
| ABC87511.1 | polyketide\_synthase | BGC0001011 | NRP+Polyketide | 33.0 | 52.8 | 425.0 | 8.2e-121 |
| ctg1\_orf22 |  | BGC0001013 | NRP+Polyketide | 33.0 | 52.8 | 425.0 | 8.2e-121 |
| WP\_081238291.1 | type\_I\_polyketide\_synthase | BGC0002105 | Polyketide | 34.0 | 52.1 | 425.0 | 8.47e-121 |
| ABC84471.1 | NigAVII | BGC0000114 | Polyketide:Modular type I polyketide | 32.0 | 53.7 | 425.0 | 9.17e-121 |
| ACO94456.1 | polyketide\_synthase\_type\_I | BGC0000029 | Polyketide:Modular type I polyketide | 31.0 | 61.5 | 425.0 | 9.79e-121 |
| AWH12936.1 | StmA | BGC0001784 | Polyketide | 33.0 | 53.6 | 425.0 | 1e-120 |
| BAF85839.1 | modular\_polyketide\_synthase | BGC0000109 | Polyketide | 28.0 | 72.8 | 425.0 | 1.01e-120 |
| CAE02605.1 | polyketide\_synthase\_type\_I | BGC0000024 | Polyketide:Modular type I polyketide | 31.0 | 60.1 | 423.0 | 1.02e-120 |
| QCF28926.1 | type\_I\_polyketide\_synthase | BGC0002308 | Alkaloid+Polyketide | 30.0 | 59.9 | 423.0 | 1.09e-120 |
| AAC01710.1 | RifA | BGC0000136 | Polyketide | 33.0 | 52.6 | 424.0 | 1.21e-120 |
| CBD77732.1 | polyketide\_synthase | BGC0000974 | NRP+Polyketide | 32.0 | 54.4 | 424.0 | 1.29e-120 |
| QRI43530.1 | type\_I\_polyketide\_synthase | BGC0002454 | Polyketide | 32.0 | 53.2 | 424.0 | 1.41e-120 |
| BAC68129.1 | modular\_polyketide\_synthase | BGC0000059 | Polyketide | 32.0 | 55.7 | 424.0 | 1.44e-120 |
| AGY62754.1 | EbeB | BGC0000051 | Polyketide | 29.0 | 67.6 | 423.0 | 1.49e-120 |
| SCN11950.1 | EbeB-type\_I\_polyketide\_synthase | BGC0001580 | Polyketide | 29.0 | 67.6 | 423.0 | 1.49e-120 |
| UMP03509.1 | NmvAII | BGC0002649 | NRP+Polyketide | 31.0 | 55.8 | 419.0 | 1.78e-120 |
| ANY10588.1 | polyketide\_synthase | BGC0001773 | Polyketide | 31.0 | 60.5 | 423.0 | 1.82e-120 |
| ABJ97438.1 | MerB | BGC0001012 | NRP+Polyketide | 31.0 | 59.3 | 424.0 | 1.88e-120 |
| ACB46471.1 | polyketide\_synthase | BGC0000082 | Polyketide | 30.0 | 67.9 | 424.0 | 1.93e-120 |
| AAM54076.1 | polyketide\_synthase | BGC0000020 | Polyketide | 32.0 | 58.6 | 423.0 | 2.12e-120 |
| TMU97102.1 | SDR\_family\_NAD(P)-dependent\_oxidoreductase | BGC0002038 | Polyketide | 32.0 | 60.6 | 422.0 | 2.15e-120 |
| ARE67853.1 | AbsB1 | BGC0001492 | Polyketide | 31.0 | 60.5 | 424.0 | 2.42e-120 |
| ABC84456.1 | NigAI | BGC0000114 | Polyketide:Modular type I polyketide | 29.0 | 68.8 | 422.0 | 2.67e-120 |
| QNN81300.1 | IonAIV | BGC0002446 | Polyketide | 30.0 | 61.5 | 423.0 | 2.72e-120 |
| ADM46357.1 | polyketide\_synthase | BGC0000106 | Polyketide | 30.0 | 67.4 | 423.0 | 3.02e-120 |
| AVX51099.1 | NysJ | BGC0001709 | Polyketide | 31.0 | 60.9 | 423.0 | 3.03e-120 |
| ADH04657.1 | TugA | BGC0001342 | NRP+Polyketide | 29.0 | 68.1 | 423.0 | 3.19e-120 |
| CAQ18838.1 | polyketide\_synthase | BGC0000954 | NRP+Polyketide:Modular type I polyketide | 34.0 | 50.4 | 420.0 | 3.45e-120 |
| QNN81302.1 | IonAVI | BGC0002446 | Polyketide | 28.0 | 73.5 | 423.0 | 3.62e-120 |
| ABW96542.1 | type\_I\_modular\_polyketide\_synthase | BGC0000159 | Polyketide:Modular type I polyketide | 28.0 | 67.5 | 423.0 | 3.63e-120 |
| ADC45535.1 | modular\_polyketide\_synthase | BGC0000093 | Polyketide | 32.0 | 55.0 | 423.0 | 3.99e-120 |
| ADH04659.1 | TugC | BGC0001342 | NRP+Polyketide | 32.0 | 54.8 | 422.0 | 4.17e-120 |
| ASZ00149.1 | polyketide\_synthase | BGC0001785 | Polyketide | 30.0 | 60.0 | 422.0 | 4.27e-120 |
| WP\_036342114.1 | type\_I\_polyketide\_synthase | BGC0001327 | NRP:Cyclic depsipeptide+Polyketide:Modular type I polyketide | 30.0 | 61.5 | 422.0 | 4.7e-120 |
| AKD43768.1 | HerA1 | BGC0001349 | NRP+Polyketide | 31.0 | 57.8 | 422.0 | 5e-120 |
| BAG23201.1 | putative\_type-I\_PKS | BGC0002673 | Polyketide+Alkaloid | 32.0 | 55.9 | 422.0 | 5.12e-120 |
| ACO94496.1 | polyketide\_synthase\_type\_I | BGC0000097 | Polyketide:Modular type I polyketide | 32.0 | 56.6 | 422.0 | 5.41e-120 |
| ACO94499.1 | polyketide\_synthase\_type\_I | BGC0000097 | Polyketide:Modular type I polyketide | 31.0 | 58.4 | 422.0 | 5.43e-120 |
| AGM05536.1 | type\_I\_polyketide\_synthase | BGC0002098 | Polyketide | 29.0 | 66.7 | 420.0 | 5.65e-120 |
| WP\_245661588.1 | hypothetical\_protein | BGC0001348 | Polyketide:Modular type I polyketide | 32.0 | 53.5 | 422.0 | 6.02e-120 |
| AAP42873.1 | NanA11 | BGC0000105 | Polyketide | 29.0 | 67.3 | 421.0 | 6.07e-120 |
| AHB82063.1 | polyketide\_synthase | BGC0001231 | NRP+Polyketide:Modular type I polyketide | 37.0 | 38.0 | 400.0 | 8.27e-120 |
| SCO70310.1 | Type\_I\_polyketide\_synthase | BGC0001433 | Polyketide:Modular type I polyketide | 33.0 | 53.9 | 421.0 | 8.37e-120 |
| WP\_039806854.1 | type\_I\_polyketide\_synthase | BGC0002001 | NRP+Polyketide | 33.0 | 54.4 | 415.0 | 9.23e-120 |
| CAE02602.1 | polyketide\_synthase\_type\_I | BGC0000024 | Polyketide:Modular type I polyketide | 28.0 | 73.1 | 419.0 | 9.37e-120 |
| AXI91548.1 | FunP5 | BGC0001944 | Polyketide | 31.0 | 61.5 | 422.0 | 1.03e-119 |
| BAF85838.1 | modular\_polyketide\_synthase | BGC0000109 | Polyketide | 30.0 | 62.6 | 421.0 | 1.04e-119 |
| UMP03506.1 | NmvAV | BGC0002649 | NRP+Polyketide | 31.0 | 60.8 | 421.0 | 1.26e-119 |
| AZF85932.1 | type\_I\_polyketide\_synthase | BGC0001963 | NRP+Polyketide | 31.0 | 56.2 | 414.0 | 1.32e-119 |
| ABC84457.1 | NigAII | BGC0000114 | Polyketide:Modular type I polyketide | 31.0 | 61.2 | 420.0 | 1.57e-119 |
| WP\_003060229.1 | type\_I\_polyketide\_synthase | BGC0002009 | Polyketide | 33.0 | 52.9 | 421.0 | 1.59e-119 |
| CAQ52626.1 | type\_I\_polyketide\_synthase,\_loading\_module\_and\_modules\_1-3 | BGC0001066 | Polyketide:Modular type I polyketide | 29.0 | 71.7 | 421.0 | 1.76e-119 |
| AVX51100.1 | nysK | BGC0001709 | Polyketide | 30.0 | 59.5 | 419.0 | 1.8e-119 |
| ACY06289.1 | type\_I\_polyketide\_synthase | BGC0001042 | NRP+Polyketide | 34.0 | 54.3 | 421.0 | 1.91e-119 |
| AHA38202.1 | GphI | BGC0000069 | Polyketide | 31.0 | 66.4 | 419.0 | 2.02e-119 |
| AWH12670.1 | RmpA2 | BGC0001759 | Polyketide | 32.0 | 53.1 | 414.0 | 2.23e-119 |
| AAG23264.1 | polyketide\_synthase\_loading\_and\_extender\_module\_1 | BGC0000148 | Polyketide | 33.0 | 53.4 | 420.0 | 2.25e-119 |
| AKD43753.1 | HerB | BGC0001349 | NRP+Polyketide | 29.0 | 67.1 | 420.0 | 2.36e-119 |
| AAK19883.1 | soraphen\_polyketide\_synthase\_A | BGC0000147 | Polyketide:Modular type I polyketide | 30.0 | 67.5 | 421.0 | 2.36e-119 |
| AGC24271.1 | prlQ | BGC0001038 | NRP+Polyketide:Modular type I polyketide | 32.0 | 53.1 | 409.0 | 2.78e-119 |
| CAQ52624.1 | type\_I\_polyketide\_synthase,\_modules\_7-8 | BGC0001066 | Polyketide:Modular type I polyketide | 30.0 | 60.8 | 420.0 | 3.06e-119 |
| AEC13071.1 | fosE | BGC0000060 | Polyketide | 29.0 | 68.9 | 420.0 | 3.12e-119 |
| QDA77044.1 | polyketide\_synthase | BGC0002025 | NRP+Polyketide | 33.0 | 51.5 | 420.0 | 3.14e-119 |
| BAW35651.1 | modular\_polyketide\_synthase | BGC0002355 | Polyketide+Other | 28.0 | 73.7 | 420.0 | 3.51e-119 |
| ADC79638.1 | TamAII | BGC0001052 | NRP+Polyketide:Modular type I polyketide | 29.0 | 66.9 | 419.0 | 3.99e-119 |
| AVV61983.1 | type\_I\_modular\_polyketide\_synthase | BGC0001477 | NRP+Polyketide:Modular type I polyketide | 29.0 | 68.9 | 420.0 | 4.03e-119 |
| ATL73033.1 | type\_I\_modular\_polyketide\_synthase | BGC0001807 | NRP+Polyketide | 32.0 | 52.4 | 420.0 | 4.22e-119 |
| AAO65806.1 | monensin\_polyketide\_synthase\_modules\_11\_and\_12 | BGC0000100 | Polyketide | 30.0 | 61.7 | 419.0 | 4.34e-119 |
| ANZ52469.1 | MonAVIII | BGC0001670 | Polyketide | 30.0 | 61.7 | 419.0 | 4.34e-119 |
| RLV64599.1 | Erythronolide\_synthase,\_modules\_1\_and\_2 | BGC0001845 | Polyketide+NRP+Other:Aminocoumarin | 32.0 | 53.3 | 419.0 | 4.41e-119 |
| AAK57190.1 | MxaF | BGC0001022 | NRP+Polyketide | 33.0 | 51.2 | 419.0 | 4.46e-119 |
| ABM21569.1 | crpA | BGC0000975 | NRP+Polyketide | 31.0 | 58.5 | 419.0 | 4.66e-119 |
| CAQ18839.1 | hybrid\_polyketide\_synthase/nonribosomal\_polypetide\_synthetase | BGC0000954 | NRP+Polyketide:Modular type I polyketide | 33.0 | 53.1 | 419.0 | 4.97e-119 |
| QBG82529.1 | Polyketide\_synthase | BGC0002587 | Polyketide | 29.0 | 68.5 | 419.0 | 5.12e-119 |
| ACO94468.1 | polyketide\_synthase\_type\_I | BGC0000029 | Polyketide:Modular type I polyketide | 32.0 | 56.7 | 419.0 | 5.32e-119 |
| AVX51106.1 | nysA | BGC0001709 | Polyketide | 32.0 | 56.2 | 410.0 | 5.32e-119 |
| AAU93806.2 | polyketide\_synthase\_modules\_3\_and\_4 | BGC0000054 | Polyketide | 30.0 | 67.2 | 419.0 | 5.57e-119 |
| AAZ77694.1 | ChlA2 | BGC0000036 | Polyketide:Modular type I polyketide+Polyketide:Iterative type I polyketide+Saccharide:Oligosaccharide | 31.0 | 59.1 | 419.0 | 5.97e-119 |
| AAO65798.1 | monensin\_polyketide\_synthase\_modules\_3\_and\_4 | BGC0000100 | Polyketide | 31.0 | 59.9 | 419.0 | 6.13e-119 |
| ANZ52461.1 | MonAIII | BGC0001670 | Polyketide | 31.0 | 59.9 | 419.0 | 6.13e-119 |
| WP\_030498975.1 | type\_I\_polyketide\_synthase | BGC0001327 | NRP:Cyclic depsipeptide+Polyketide:Modular type I polyketide | 33.0 | 54.1 | 416.0 | 6.58e-119 |
| QKV49791.1 | PKS | BGC0002526 | Polyketide | 32.0 | 54.5 | 419.0 | 6.65e-119 |
| UHY14126.1 | PKS\_I | BGC0002671 | Polyketide | 29.0 | 66.2 | 417.0 | 7.34e-119 |
| AWW87423.1 | type\_I\_polyketide\_synthase | BGC0001755 | Polyketide | 32.0 | 54.0 | 419.0 | 7.43e-119 |
| ABB88523.1 | polyketide\_synthase\_type\_I | BGC0000050 | Polyketide | 32.0 | 54.3 | 416.0 | 7.71e-119 |
| QSE03591.1 | LcmC | BGC0002333 | Polyketide | 34.0 | 52.2 | 419.0 | 8.13e-119 |
| AOE23578.1 | FoxBII | BGC0001598 | NRP+Polyketide | 31.0 | 61.5 | 419.0 | 8.83e-119 |
| BAO66543.1 | type\_I\_polyketide\_synthase | BGC0000042 | Polyketide | 32.0 | 52.7 | 413.0 | 9.62e-119 |
| AAS79461.1 | polyketide\_synthase\_subunit | BGC0000035 | Polyketide | 28.0 | 68.3 | 418.0 | 1.03e-118 |
| CAF05651.1 | TubF\_protein | BGC0001053 | NRP+Polyketide | 33.0 | 54.0 | 418.0 | 1.05e-118 |
| ADH04639.1 | TgaA | BGC0001051 | NRP+Polyketide:Modular type I polyketide | 33.0 | 51.1 | 419.0 | 1.06e-118 |
| CAL58684.1 | polyketide\_synthase | BGC0000149 | Polyketide:Modular type I polyketide | 29.0 | 67.5 | 418.0 | 1.23e-118 |
| QLD28380.2 | SDR\_family\_NAD(P)-dependent\_oxidoreductase | BGC0002086 | Polyketide | 32.0 | 60.1 | 418.0 | 1.35e-118 |
| BAW35654.1 | modular\_polyketide\_synthase | BGC0002355 | Polyketide+Other | 32.0 | 53.7 | 413.0 | 1.48e-118 |
| QUQ72344.1 | 3-ketoacyl-CoA\_thiolase | BGC0002349 | Polyketide+Saccharide | 34.0 | 51.8 | 417.0 | 1.56e-118 |
| WP\_226048588.1 | AMP-binding\_protein | BGC0002106 | Polyketide | 31.0 | 56.4 | 414.0 | 1.78e-118 |
| QEA08891.1 | JenA5 | BGC0002559 | Polyketide | 30.0 | 60.5 | 417.0 | 1.87e-118 |
| ctg1\_orf255 |  | BGC0001200 | Polyketide | 30.0 | 62.1 | 416.0 | 1.87e-118 |
| BAQ25507.1 | type\_I\_polyketide\_synthase | BGC0001288 | Polyketide | 30.0 | 57.1 | 414.0 | 2.27e-118 |
| ibo19 |  | BGC0001619 | Polyketide | 29.0 | 73.8 | 417.0 | 2.33e-118 |
| AFL48532.1 | laidlomycin\_polyketide\_synthase\_(module\_11\_and\_module\_12) | BGC0000084 | Polyketide | 30.0 | 59.4 | 417.0 | 2.35e-118 |
| TXD00034.1 | SDR\_family\_NAD(P)-dependent\_oxidoreductase | BGC0001877 | Polyketide | 32.0 | 56.3 | 417.0 | 2.59e-118 |
| AVX51107.1 | nysB | BGC0001709 | Polyketide | 31.0 | 54.0 | 417.0 | 2.8e-118 |
| ADU86004.1 | putative\_modular\_polyketide\_synthase | BGC0000165 | Polyketide:Modular type I polyketide | 33.0 | 54.2 | 417.0 | 3e-118 |
| AAR16521.1 | RimA | BGC0000138 | Polyketide | 31.0 | 56.4 | 413.0 | 3.46e-118 |
| UHY14129.1 | PKS\_I | BGC0002671 | Polyketide | 29.0 | 67.1 | 417.0 | 3.55e-118 |
| CAE46843.1 | Type\_I\_modular\_polyketide\_synthase | BGC0000103 | Polyketide | 32.0 | 53.1 | 417.0 | 3.61e-118 |
| WP\_048832936.1 | polyketide\_synthase | BGC0001348 | Polyketide:Modular type I polyketide | 32.0 | 58.3 | 417.0 | 3.77e-118 |
| ADH04660.1 | TugD | BGC0001342 | NRP+Polyketide | 32.0 | 53.4 | 417.0 | 4.14e-118 |
| ASZ00150.1 | polyketide\_synthase | BGC0001785 | Polyketide | 29.0 | 67.3 | 414.0 | 4.25e-118 |
| QFU19841.1 | PKS | BGC0002431 | Polyketide+Saccharide | 29.0 | 75.5 | 416.0 | 4.45e-118 |
| AWC08661.1 | polyketide\_synthase\_type\_I | BGC0001662 | Polyketide | 31.0 | 53.2 | 416.0 | 4.98e-118 |
| BAW35657.1 | modular\_polyketide\_synthase | BGC0002355 | Polyketide+Other | 32.0 | 53.1 | 416.0 | 5e-118 |
| ABB88522.1 | polyketide\_synthase\_type\_I | BGC0000050 | Polyketide | 33.0 | 58.4 | 416.0 | 5.1e-118 |
| ASZ00147.1 | polyketide\_synthase | BGC0001785 | Polyketide | 32.0 | 53.2 | 416.0 | 5.19e-118 |
| AAC01714.1 | RifE | BGC0000136 | Polyketide | 29.0 | 71.6 | 416.0 | 5.25e-118 |
| AFI57005.1 | QmnA1 | BGC0000133 | Polyketide | 34.0 | 52.2 | 416.0 | 5.31e-118 |
| AEK75504.1 | type\_1\_polyketide\_synthase | BGC0000001 | Polyketide:Modular type I polyketide | 32.0 | 53.2 | 399.0 | 5.48e-118 |
| UHY14125.1 | PKS\_I | BGC0002671 | Polyketide | 28.0 | 66.4 | 416.0 | 5.74e-118 |
| sipP4 | Type\_I\_Modular\_PKS | BGC0001452 | Polyketide | 32.0 | 53.8 | 411.0 | 5.85e-118 |
| AAP42859.1 | NanA5 | BGC0000105 | Polyketide | 29.0 | 61.6 | 416.0 | 5.86e-118 |
| QBF51754.1 | type\_I\_polyketide\_synthase | BGC0001856 | Polyketide:Modular type I polyketide | 31.0 | 56.8 | 416.0 | 6.06e-118 |
| CAD15508.1 | polyketide\_synthase/non-ribosomal\_peptide\_synthetase | BGC0001014 | NRP:NRP siderophore+Polyketide:Modular type I polyketide+Polyketide:Iterative type I polyketide | 33.0 | 53.5 | 416.0 | 6.11e-118 |
| AAO65797.1 | monensin\_polyketide\_synthase\_module\_2 | BGC0000100 | Polyketide | 28.0 | 73.0 | 415.0 | 6.6e-118 |
| ANZ52460.1 | MonAII | BGC0001670 | Polyketide | 28.0 | 73.0 | 415.0 | 6.6e-118 |
| QLD23491.1 | Polyketide\_synthase | BGC0002085 | Saccharide:Oligosaccharide | 31.0 | 56.5 | 405.0 | 7.23e-118 |
| BAT51066.1 | type\_I\_polyketide\_synthase | BGC0001296 | Polyketide | 29.0 | 67.7 | 416.0 | 7.27e-118 |
| ARW71485.1 | type\_I\_PKS\_module\_4,\_module\_5 | BGC0001812 | Polyketide | 31.0 | 60.2 | 416.0 | 7.48e-118 |
| QKG20136.1 | type\_I\_polyketide\_synthase | BGC0002124 | Polyketide | 29.0 | 68.3 | 416.0 | 7.79e-118 |
| CAN89636.1 | putative\_polyketide\_synthase | BGC0001070 | NRP+Polyketide:Modular type I polyketide+Polyketide:Trans-AT type I polyketide | 31.0 | 58.8 | 415.0 | 7.93e-118 |
| AWS21278.1 | type\_I\_polyketide\_synthase | BGC0001934 | Polyketide | 30.0 | 62.0 | 415.0 | 8.08e-118 |
| AZY91987.1 | polyketide\_synthase | BGC0002022 | Polyketide | 30.0 | 62.0 | 415.0 | 8.08e-118 |
| ACO94471.1 | polyketide\_synthase\_type\_I | BGC0000029 | Polyketide:Modular type I polyketide | 31.0 | 56.4 | 415.0 | 9.2e-118 |
| OAP25819.1 | Phenolphthiocerol\_synthesis\_polyketide\_synthase\_type\_I\_Pks15/1 | BGC0001658 | Polyketide | 31.0 | 56.5 | 412.0 | 9.42e-118 |
| AEP40940.1 | polyketide\_synthase\_type\_I | BGC0000021 | Polyketide | 33.0 | 52.1 | 416.0 | 9.57e-118 |
| AAZ77673.1 | ChlB1 | BGC0000036 | Polyketide:Modular type I polyketide+Polyketide:Iterative type I polyketide+Saccharide:Oligosaccharide | 30.0 | 59.9 | 412.0 | 9.7e-118 |
| AKL64832.1 | polyketide\_synthase | BGC0002072 | Polyketide:Modular type I polyketide | 31.0 | 52.5 | 410.0 | 1.13e-117 |
| AGC09484.1 | LobS1 | BGC0001183 | Polyketide | 32.0 | 54.6 | 415.0 | 1.28e-117 |
| UHH90012.1 | VicP4 | BGC0002634 | Polyketide+NRP+Other | 29.0 | 68.0 | 415.0 | 1.36e-117 |
| ACY13414.1 | amino\_acid\_adenylation\_domain\_protein | BGC0001367 | NRP+Polyketide | 31.0 | 57.0 | 415.0 | 1.42e-117 |
| AAR87760.2 | ZmaK | BGC0001059 | NRP+Polyketide | 32.0 | 54.1 | 414.0 | 1.59e-117 |
| ABJ97437.1 | MerA | BGC0001012 | NRP+Polyketide | 33.0 | 55.9 | 415.0 | 1.64e-117 |
| AAP85335.1 | type\_I\_PKS | BGC0000233 | Polyketide | 31.0 | 56.2 | 413.0 | 1.67e-117 |
| ADH04680.1 | hybrid\_polyketide\_synthase/non-ribosomal\_peptide\_synthetase | BGC0001344 | NRP+Polyketide | 33.0 | 53.0 | 414.0 | 1.68e-117 |
| CQR60496.1 | Polyketide\_synthase,\_type\_I,\_modules:\_4,\_5\_and\_6 | BGC0001287 | Polyketide | 31.0 | 61.1 | 414.0 | 2.13e-117 |
| ABV83222.1 | CppJ | BGC0000116 | Polyketide | 32.0 | 53.6 | 414.0 | 2.13e-117 |
| ABY21541.1 | AngAIV | BGC0000018 | Polyketide | 32.0 | 54.1 | 409.0 | 2.14e-117 |
| AAQ82561.1 | FscA | BGC0000034 | NRP+Polyketide | 32.0 | 52.9 | 410.0 | 2.14e-117 |
| OAP25821.1 | Phenolphthiocerol\_synthesis\_polyketide\_synthase\_type\_I\_Pks15/1 | BGC0001658 | Polyketide | 30.0 | 67.6 | 414.0 | 2.2e-117 |
| AGI99497.1 | type\_I\_polyketide\_synthase | BGC0001004 | Polyketide:Modular type I polyketide | 31.0 | 57.6 | 414.0 | 2.26e-117 |
| QFU80902.1 | PKS | BGC0002550 | Polyketide | 31.0 | 57.6 | 414.0 | 2.26e-117 |
| AXG22405.1 | type\_I\_polyketide\_synthase | BGC0002024 | Polyketide | 31.0 | 56.6 | 414.0 | 2.3e-117 |
| AHH25595.1 | PKS | BGC0000957 | NRP+Polyketide | 30.0 | 67.2 | 414.0 | 2.42e-117 |
| OJF16266.1 | AceP4 | BGC0001491 | Polyketide | 31.0 | 60.8 | 414.0 | 2.47e-117 |
| ANY10600.1 | polyketide\_synthase | BGC0001773 | Polyketide | 32.0 | 51.8 | 414.0 | 2.49e-117 |
| QUQ72345.1 | 3-ketoacyl-CoA\_thiolase | BGC0002349 | Polyketide+Saccharide | 34.0 | 51.8 | 414.0 | 3.15e-117 |
| AHB82057.1 | polyketide\_synthase | BGC0001019 | NRP+Polyketide:Modular type I polyketide | 33.0 | 52.4 | 407.0 | 3.9e-117 |
| QKV49770.1 | PKS | BGC0002526 | Polyketide | 31.0 | 56.4 | 413.0 | 4.38e-117 |
| ADH04658.1 | TugB | BGC0001342 | NRP+Polyketide | 32.0 | 54.5 | 413.0 | 4.7e-117 |
| BAW35635.1 | modular\_polyketide\_synthase | BGC0002356 | Polyketide+Other | 30.0 | 53.4 | 413.0 | 4.84e-117 |
| ANC94966.1 | AlmHI | BGC0001396 | Polyketide | 31.0 | 54.1 | 413.0 | 5.17e-117 |
| AFY58524.1 | beta-ketoacyl\_synthase\_family\_protein,acyltransferase\_family\_protein,phosphopantetheine-containing\_protein | BGC0002411 | NRP+Polyketide | 36.0 | 38.2 | 402.0 | 5.44e-117 |
| QPP46757.1 | polyketide\_synthase | BGC0002500 | Polyketide | 31.0 | 62.0 | 412.0 | 5.68e-117 |
| ACB37755.1 | putative\_type\_I\_polyketide\_synthase | BGC0000162 | Polyketide | 30.0 | 67.8 | 413.0 | 5.69e-117 |
| AHB82072.1 | non\_ribosomal\_peptide\_synthetase/polyketide\_synthase | BGC0001231 | NRP+Polyketide:Modular type I polyketide | 33.0 | 54.2 | 412.0 | 5.96e-117 |
| CQR60494.1 | Polyketide\_synthase,\_type\_I,\_module\_8 | BGC0001287 | Polyketide | 31.0 | 60.8 | 409.0 | 6.43e-117 |
| ctg1\_orf20 |  | BGC0001013 | NRP+Polyketide | 32.0 | 56.2 | 413.0 | 6.79e-117 |
| ABC87509.1 | polyketide\_synthase | BGC0001011 | NRP+Polyketide | 32.0 | 56.2 | 413.0 | 6.79e-117 |
| QIE07127.1 | OvmL1 | BGC0001719 | Polyketide | 28.0 | 70.0 | 411.0 | 8.27e-117 |
| CAJ88185.2 | Type\_I\_modular\_polyketide\_synthase | BGC0000151 | Polyketide:Modular type I polyketide+Saccharide:Hybrid/tailoring saccharide | 32.0 | 52.0 | 412.0 | 8.45e-117 |
| AZF85917.1 | type\_I\_polyketide\_synthase | BGC0001963 | NRP+Polyketide | 32.0 | 54.8 | 412.0 | 8.78e-117 |
| AHB82052.1 | polyketide\_synthase | BGC0001019 | NRP+Polyketide:Modular type I polyketide | 36.0 | 37.9 | 392.0 | 9.36e-117 |
| QSE03601.1 | LcmB | BGC0002333 | Polyketide | 32.0 | 56.4 | 412.0 | 9.41e-117 |
| AAB66508.1 | tylactone\_synthase\_module\_7 | BGC0000166 | Polyketide | 32.0 | 53.6 | 409.0 | 9.89e-117 |
| BBA66513.1 | type\_I\_polyketide\_synthase | BGC0001495 | Polyketide | 32.0 | 53.1 | 412.0 | 1.06e-116 |
| CCM44338.1 | Polyketide\_synthase | BGC0001056 | NRP+Polyketide:Modular type I polyketide+Polyketide:PUFA synthase or related polyketide | 32.0 | 53.8 | 406.0 | 1.08e-116 |
| AKA59089.1 | type-I\_PKS | BGC0001619 | Polyketide | 32.0 | 52.3 | 412.0 | 1.14e-116 |
| BAF85844.1 | modular\_polyketide\_synthase | BGC0000109 | Polyketide | 32.0 | 54.6 | 412.0 | 1.15e-116 |
| EFL02193.1 | amino\_acid\_adenylation\_domain-containing\_protein | BGC0000996 | NRP+Polyketide:Iterative type I polyketide | 30.0 | 67.0 | 411.0 | 1.32e-116 |
| AAM81584.2 | putative\_type\_I\_polyketide\_synthase | BGC0000047 | Polyketide | 31.0 | 54.1 | 412.0 | 1.42e-116 |
| BAW35638.1 | modular\_polyketide\_synthase | BGC0002356 | Polyketide+Other | 31.0 | 53.5 | 407.0 | 1.45e-116 |
| ACO94460.1 | polyketide\_synthase\_type\_I | BGC0000029 | Polyketide:Modular type I polyketide | 29.0 | 67.3 | 411.0 | 1.64e-116 |
| AFV30251.1 | polyketide\_synthase | BGC0000075 | Polyketide | 31.0 | 53.4 | 411.0 | 1.78e-116 |
| ABP55493.1 | thioester\_reductase\_domain | BGC0001006 | NRP+Polyketide | 32.0 | 53.8 | 410.0 | 1.8e-116 |
| AXI91545.1 | FunP8 | BGC0001944 | Polyketide | 29.0 | 66.7 | 409.0 | 2.53e-116 |
| ANR02552.1 | LodK | BGC0001648 | Polyketide | 31.0 | 53.6 | 406.0 | 2.61e-116 |
| ANH11410.1 | SceO | BGC0001770 | Polyketide | 31.0 | 68.6 | 410.0 | 2.88e-116 |
| AAM81586.2 | putative\_type\_I\_polyketide\_synthase | BGC0000047 | Polyketide | 31.0 | 54.5 | 410.0 | 3.02e-116 |
| BAB69195.1 | modular\_polyketide\_synthase | BGC0000117 | Polyketide | 31.0 | 53.5 | 410.0 | 3.44e-116 |
| AGY62755.1 | EbeC | BGC0000051 | Polyketide | 31.0 | 52.7 | 405.0 | 3.49e-116 |
| ADM46358.1 | polyketide\_synthase | BGC0000106 | Polyketide | 32.0 | 56.7 | 410.0 | 3.64e-116 |
| BAF85843.1 | modular\_polyketide\_synthase | BGC0000109 | Polyketide | 28.0 | 67.1 | 410.0 | 3.73e-116 |
| ABV83223.1 | CppK | BGC0000116 | Polyketide | 32.0 | 57.0 | 409.0 | 4.07e-116 |
| AWH12664.1 | RmpE2 | BGC0001759 | Polyketide | 29.0 | 71.5 | 409.0 | 4.27e-116 |
| AWW87424.1 | type\_I\_polyketide\_synthase | BGC0001755 | Polyketide | 32.0 | 53.1 | 410.0 | 4.29e-116 |
| BCK51648.1 | modular\_polyketide\_synthase | BGC0002520 | Polyketide | 32.0 | 52.7 | 410.0 | 4.67e-116 |
| ANC94963.1 | AlmHIV | BGC0001396 | Polyketide | 31.0 | 53.6 | 405.0 | 4.76e-116 |
| BAE93728.1 | type\_I\_polyketide\_synthase | BGC0000164 | Polyketide | 32.0 | 53.0 | 405.0 | 5.07e-116 |
| AEZ53947.1 | polyketide\_synthase | BGC0000144 | Polyketide:Modular type I polyketide | 33.0 | 52.9 | 409.0 | 5.18e-116 |
| CAE02606.1 | polyketide\_synthase\_type\_I | BGC0000024 | Polyketide:Modular type I polyketide | 31.0 | 53.1 | 409.0 | 6.05e-116 |
| AXM42949.1 | hybrid\_type\_1\_PKS/NRPS | BGC0001941 | NRP+Polyketide | 28.0 | 72.5 | 409.0 | 6.3e-116 |
| ABV97151.1 | AMP-dependent\_synthetase\_and\_ligase | BGC0000137 | Polyketide | 33.0 | 52.4 | 410.0 | 6.35e-116 |
| ACN69992.1 | polyketide\_synthase | BGC0000079 | Polyketide | 30.0 | 53.4 | 409.0 | 6.37e-116 |
| CQR60495.1 | Polyketide\_synthase,\_type\_I,\_module\_7 | BGC0001287 | Polyketide | 30.0 | 67.0 | 407.0 | 6.79e-116 |
| AEU17899.1 | putative\_type\_I\_PKS | BGC0001072 | Saccharide+Polyketide:Modular type I polyketide+Polyketide:Type II polyketide+Other:Aminocoumarin | 31.0 | 58.3 | 409.0 | 7.8e-116 |
| QGJ79675.1 | Polyketide\_synthase | BGC0002552 | Polyketide | 30.0 | 60.2 | 409.0 | 7.81e-116 |
| BAT51067.1 | type\_I\_polyketide\_synthase | BGC0001296 | Polyketide | 31.0 | 56.6 | 408.0 | 8.38e-116 |
| AKD43761.1 | HerD | BGC0001349 | NRP+Polyketide | 31.0 | 56.3 | 409.0 | 8.72e-116 |
| BAG23200.1 | putative\_type-I\_PKS | BGC0002673 | Polyketide+Alkaloid | 31.0 | 56.2 | 409.0 | 9.02e-116 |
| ACO94488.1 | polyketide\_synthase\_type\_I | BGC0000097 | Polyketide:Modular type I polyketide | 31.0 | 57.1 | 409.0 | 9.03e-116 |
| AVV61981.1 | type\_I\_modular\_PKS | BGC0001477 | NRP+Polyketide:Modular type I polyketide | 28.0 | 68.3 | 409.0 | 9.1e-116 |
| QBG82527.1 | Polyketide\_synthase | BGC0002587 | Polyketide | 32.0 | 53.1 | 409.0 | 9.33e-116 |
| ABV99085.1 | thioester\_reductase\_domain | BGC0001007 | Polyketide+NRP | 32.0 | 53.2 | 408.0 | 9.92e-116 |
| ANZ22991.1 | ZinG | BGC0001828 | Polyketide | 31.0 | 52.9 | 408.0 | 1.02e-115 |
| QRI43526.1 | type\_I\_polyketide\_synthase | BGC0002454 | Polyketide | 30.0 | 61.9 | 409.0 | 1.15e-115 |
| ACB37742.1 | putative\_type\_I\_polyketide\_synthase | BGC0000162 | Polyketide | 30.0 | 60.7 | 406.0 | 1.21e-115 |
| WP\_102919232.1 | type\_I\_polyketide\_synthase | BGC0002104 | NRP+Polyketide | 32.0 | 54.0 | 409.0 | 1.34e-115 |
| AWC08658.1 | polyketide\_synthase\_type\_I | BGC0001662 | Polyketide | 31.0 | 53.4 | 404.0 | 1.56e-115 |
| ADH04682.1 | polyketide\_synthase | BGC0001344 | NRP+Polyketide | 33.0 | 53.1 | 408.0 | 1.73e-115 |
| AAF71768.1 | nysK | BGC0000115 | Polyketide:Modular type I polyketide+Saccharide:Hybrid/tailoring saccharide | 30.0 | 59.3 | 406.0 | 2.54e-115 |
| CAJ88184.1 | Type\_I\_modular\_polyketide\_synthase | BGC0000151 | Polyketide:Modular type I polyketide+Saccharide:Hybrid/tailoring saccharide | 32.0 | 52.8 | 408.0 | 2.83e-115 |
| BBA66512.1 | type\_I\_polyketide\_synthase | BGC0001495 | Polyketide | 33.0 | 53.5 | 408.0 | 2.85e-115 |
| AFP87524.1 | type\_I\_polyketide\_synthase | BGC0001159 | NRP+Polyketide:Modular type I polyketide | 32.0 | 54.0 | 406.0 | 3.59e-115 |
| ABB05102.1 | LipPks1 | BGC0001003 | NRP:Lipopeptide+Polyketide:Modular type I polyketide+Saccharide:Hybrid/tailoring saccharide | 30.0 | 55.7 | 406.0 | 3.65e-115 |
| ctg1\_12 |  | BGC0001931 | Polyketide | 31.0 | 52.5 | 402.0 | 3.66e-115 |
| WP\_033261452.1 | type\_I\_polyketide\_synthase | BGC0002009 | Polyketide | 30.0 | 61.8 | 407.0 | 3.67e-115 |
| QGJ79676.1 | Polyketide\_synthase | BGC0002552 | Polyketide | 30.0 | 59.1 | 407.0 | 3.84e-115 |
| ctg1\_orf30 |  | BGC0000096 | Polyketide | 32.0 | 53.4 | 402.0 | 4.29e-115 |
| ADU86002.1 | putative\_modular\_polyketide\_synthase | BGC0000165 | Polyketide:Modular type I polyketide | 31.0 | 60.0 | 407.0 | 4.66e-115 |
| AEK75503.1 | type\_1\_polyketide\_synthase | BGC0000001 | Polyketide:Modular type I polyketide | 31.0 | 56.7 | 407.0 | 5.09e-115 |
| WP\_051137606.1 | type\_I\_polyketide\_synthase | BGC0002011 | Polyketide | 30.0 | 59.1 | 406.0 | 5.1e-115 |
| TMU97100.1 | SDR\_family\_NAD(P)-dependent\_oxidoreductase | BGC0002038 | Polyketide | 31.0 | 61.5 | 407.0 | 5.51e-115 |
| AKA59090.1 | type-I\_PKS | BGC0001619 | Polyketide | 31.0 | 56.5 | 407.0 | 5.96e-115 |
| CAQ52622.1 | type\_I\_polyketide\_synthase,\_modules\_4-5 | BGC0001066 | Polyketide:Modular type I polyketide | 28.0 | 67.7 | 406.0 | 7.16e-115 |
| AKA59088.1 | type-I\_PKS | BGC0001619 | Polyketide | 31.0 | 56.6 | 406.0 | 7.24e-115 |
| WP\_157358234.1 | SDR\_family\_NAD(P)-dependent\_oxidoreductase | BGC0002011 | Polyketide | 30.0 | 60.6 | 406.0 | 7.52e-115 |
| QBF51759.1 | type\_I\_polyketide\_synthase | BGC0001856 | Polyketide:Modular type I polyketide | 31.0 | 54.2 | 406.0 | 8.23e-115 |
| BCK51637.1 | modular\_modular\_polyketide\_synthase | BGC0002520 | Polyketide | 29.0 | 68.4 | 404.0 | 8.49e-115 |
| WP\_033261453.1 | type\_I\_polyketide\_synthase | BGC0002009 | Polyketide | 31.0 | 61.0 | 403.0 | 8.75e-115 |
| CAD70195.1 | non-ribosomal\_peptide\_synthetase | BGC0001047 | NRP+Polyketide | 29.0 | 67.3 | 406.0 | 9.81e-115 |
| AP234\_RS37235 | type\_I\_polyketide\_synthase | BGC0001653 | Polyketide | 31.0 | 52.3 | 385.0 | 1.05e-114 |
| CAI94682.1 | putative\_polyketide\_synthase | BGC0000141 | Polyketide | 31.0 | 54.3 | 406.0 | 1.1e-114 |
| QRI43528.1 | type\_I\_polyketide\_synthase | BGC0002454 | Polyketide | 30.0 | 62.3 | 404.0 | 1.36e-114 |
| SCN11951.1 | ebeC-type\_I\_polyketide\_synthase | BGC0001580 | Polyketide | 31.0 | 52.7 | 405.0 | 1.37e-114 |
| AAF86396.1 | FkbA | BGC0000994 | NRP+Polyketide | 29.0 | 66.3 | 405.0 | 1.56e-114 |
| AXG22407.1 | type\_I\_polyketide\_synthase | BGC0002024 | Polyketide | 32.0 | 54.1 | 405.0 | 2.02e-114 |
| QRI43531.1 | type\_I\_polyketide\_synthase | BGC0002454 | Polyketide | 29.0 | 56.3 | 405.0 | 2.11e-114 |
| BAG85029.1 | putative\_polyketide\_synthase | BGC0000086 | Polyketide | 31.0 | 52.8 | 400.0 | 2.18e-114 |
| ACY06287.1 | type\_I\_polyketide\_synthase | BGC0001042 | NRP+Polyketide | 33.0 | 52.4 | 405.0 | 2.3e-114 |
| CAD55506.1 | CpkA;\_Polyketide\_synthase\_loading\_module,\_and\_modules\_1\_and\_2 | BGC0000038 | Polyketide:Modular type I polyketide | 33.0 | 52.4 | 405.0 | 2.4e-114 |
| QWM97862.1 | hybrid\_non-ribosomal\_peptide\_synthetase/type\_I\_polyketide\_synthase | BGC0002434 | Polyketide+NRP | 28.0 | 69.0 | 404.0 | 2.5e-114 |
| ATX68114.1 | malonyl\_CoA-acyl\_carrier\_protein\_transacylase | BGC0001772 | Polyketide | 35.0 | 39.2 | 390.0 | 2.56e-114 |
| SAI82908.1 | HrnD;\_Macrolactam\_polyketidesynthase\_type\_I;\_modules\_3-4 | BGC0002101 | Polyketide | 31.0 | 56.4 | 404.0 | 2.63e-114 |
| BAB69194.1 | modular\_polyketide\_synthase | BGC0000117 | Polyketide | 28.0 | 67.5 | 404.0 | 2.94e-114 |
| AXI91551.1 | FunP2 | BGC0001944 | Polyketide | 32.0 | 54.9 | 399.0 | 3.23e-114 |
| CCC55921.1 | non-ribosomal\_peptide\_synthetase/polyketide\_synthase\_hybrid\_protein | BGC0000973 | NRP+Polyketide:Modular type I polyketide | 32.0 | 53.2 | 404.0 | 3.41e-114 |
| CAJ88187.2 | Type\_I\_modular\_polyketide\_synthase | BGC0000151 | Polyketide:Modular type I polyketide+Saccharide:Hybrid/tailoring saccharide | 30.0 | 60.6 | 404.0 | 3.46e-114 |
| EHA27898.1 | hypothetical\_protein | BGC0002171 | NRP+Polyketide | 31.0 | 58.0 | 402.0 | 3.64e-114 |
| ALG65339.1 | Var4 | BGC0002416 | NRP+Polyketide | 32.0 | 56.0 | 403.0 | 3.9e-114 |
| AEZ53945.1 | polyketide\_synthase | BGC0000144 | Polyketide:Modular type I polyketide | 31.0 | 57.5 | 404.0 | 4.4e-114 |
| CAO98852.1 | polyketide\_synthase\_AufI | BGC0000023 | Polyketide:Modular type I polyketide | 32.0 | 51.6 | 403.0 | 4.92e-114 |
| ANZ22985.1 | ZinB | BGC0001828 | Polyketide | 31.0 | 52.5 | 404.0 | 5.95e-114 |
| BCB17029.1 | modular\_polyketide\_synthase | BGC0002523 | NRP | 30.0 | 55.2 | 403.0 | 7.67e-114 |
| WP\_234353270.1 | SDR\_family\_NAD(P)-dependent\_oxidoreductase | BGC0001537 | Polyketide | 30.0 | 59.7 | 403.0 | 8.41e-114 |
| QIZ24102.1 | type\_I\_polyketide\_synthase | BGC0002540 | Polyketide | 32.0 | 53.9 | 403.0 | 8.71e-114 |
| AAB66506.1 | tylactone\_synthase\_modules\_4\_&\_5 | BGC0000166 | Polyketide | 30.0 | 57.6 | 403.0 | 8.82e-114 |
| WP\_015031691.1 | type\_I\_polyketide\_synthase | BGC0001819 | Polyketide | 31.0 | 54.8 | 394.0 | 9.11e-114 |
| AJW65408.1 | type\_I\_modular\_polyketide\_synthase | BGC0001195 | NRP+Polyketide | 32.0 | 53.3 | 403.0 | 1.05e-113 |
| ABX37384.1 | Beta-ketoacyl\_synthase | BGC0000984 | NRP+Polyketide | 32.0 | 54.1 | 397.0 | 1.05e-113 |
| AAO06917.1 | GdmAII | BGC0000066 | Polyketide | 31.0 | 54.8 | 402.0 | 1.1e-113 |
| ACF35447.1 | mbcAIII | BGC0000090 | Polyketide | 28.0 | 69.9 | 402.0 | 1.2e-113 |
| AGI99482.1 | Type\_I\_polyketide\_synthase | BGC0001004 | Polyketide:Modular type I polyketide | 28.0 | 67.6 | 402.0 | 1.21e-113 |
| QFU80887.1 | PKS | BGC0002550 | Polyketide | 28.0 | 67.6 | 402.0 | 1.21e-113 |
| QIE07128.1 | OvmL2 | BGC0001719 | Polyketide | 29.0 | 66.8 | 399.0 | 1.41e-113 |
| AAC01712.2 | RifC | BGC0000136 | Polyketide | 29.0 | 72.7 | 399.0 | 1.67e-113 |
| AAX98187.1 | polyketide\_synthase\_type\_I | BGC0000052 | Polyketide | 31.0 | 53.0 | 398.0 | 1.87e-113 |
| AEP40935.1 | polyketide\_synthase\_type\_I | BGC0000021 | Polyketide | 31.0 | 52.7 | 402.0 | 1.89e-113 |
| QRI43529.1 | type\_I\_polyketide\_synthase | BGC0002454 | Polyketide | 33.0 | 53.5 | 402.0 | 1.9e-113 |
| PYH50506.1 | hypothetical\_protein | BGC0002275 | NRP+Polyketide | 31.0 | 58.0 | 401.0 | 1.95e-113 |
| BAK64649.1 | polyketide\_synthase | BGC0000135 | Polyketide | 32.0 | 58.2 | 402.0 | 1.99e-113 |
| ctg1\_orf29 |  | BGC0000096 | Polyketide | 32.0 | 53.9 | 402.0 | 2.05e-113 |
| AAP42858.1 | NanA4 | BGC0000105 | Polyketide | 31.0 | 54.5 | 402.0 | 2.15e-113 |
| IF55\_RS36525 | polyketide\_synthase | BGC0001348 | Polyketide:Modular type I polyketide | 32.0 | 54.6 | 400.0 | 2.22e-113 |
| CAQ64689.1 | lasalocid\_modular\_polyketide\_synthase | BGC0000087 | Polyketide | 31.0 | 52.8 | 397.0 | 2.32e-113 |
| AAY28226.1 | HbmAII | BGC0000074 | Polyketide | 31.0 | 54.8 | 401.0 | 2.58e-113 |
| ABB86409.1 | GelB | BGC0000067 | Polyketide | 31.0 | 54.8 | 401.0 | 2.58e-113 |
| QBL56184.1 | PKS | BGC0002376 | Polyketide | 31.0 | 57.8 | 401.0 | 2.64e-113 |
| CCP20049.1 | divL2\_protein | BGC0001119 | Polyketide:Modular type I polyketide | 29.0 | 66.8 | 399.0 | 2.71e-113 |
| OJF16270.1 | AceP3 | BGC0001491 | Polyketide | 31.0 | 59.2 | 402.0 | 2.94e-113 |
| WP\_240490790.1 | type\_I\_polyketide\_synthase | BGC0002009 | Polyketide | 28.0 | 66.5 | 398.0 | 3.4e-113 |
| CAC22145.1 | CpkB;\_Polyketide\_synthase\_modules\_3\_and\_4 | BGC0000038 | Polyketide:Modular type I polyketide | 27.0 | 73.2 | 401.0 | 3.53e-113 |
| BAW32323.1 | hybrid\_cis-AT\_polyketide\_synthase\_-\_nonribosomal\_peptide\_synthetase | BGC0001630 | NRP+Polyketide | 32.0 | 52.5 | 401.0 | 3.62e-113 |
| CQR60497.1 | Polyketide\_synthase,\_type\_I,\_modules:\_loading,\_1,\_2\_and\_3 | BGC0001287 | Polyketide | 32.0 | 53.1 | 401.0 | 4.48e-113 |
| ABV97155.1 | Acyl\_transferase | BGC0000137 | Polyketide | 29.0 | 67.6 | 400.0 | 4.61e-113 |
| BCK51633.1 | modular\_polyketide\_synthase | BGC0002520 | Polyketide | 28.0 | 75.0 | 400.0 | 5.66e-113 |
| BBA66511.1 | type\_I\_polyketide\_synthase | BGC0001495 | Polyketide | 31.0 | 53.2 | 400.0 | 5.86e-113 |
| WP\_245661582.1 | polyketide\_synthase | BGC0001348 | Polyketide:Modular type I polyketide | 32.0 | 54.6 | 400.0 | 6.1e-113 |
| QVV57684.1 | beta-ketoacyl\_synthase | BGC0002338 | Polyketide | 34.0 | 48.2 | 400.0 | 6.34e-113 |
| AWW87425.1 | polyketide\_synthase | BGC0001755 | Polyketide | 29.0 | 67.4 | 400.0 | 6.45e-113 |
| QUQ72347.1 | 3-ketoacyl-CoA\_thiolase | BGC0002349 | Polyketide+Saccharide | 32.0 | 53.2 | 400.0 | 6.5e-113 |
| AXG22406.1 | type\_I\_polyketide\_synthase | BGC0002024 | Polyketide | 32.0 | 53.6 | 400.0 | 7.37e-113 |
| AJD47482.1 | erythronolide\_synthase | BGC0002418 | NRP+Polyketide | 32.0 | 53.7 | 399.0 | 7.68e-113 |
| ABV83228.1 | CppA | BGC0000116 | Polyketide | 33.0 | 52.1 | 386.0 | 9.39e-113 |
| OJF16269.1 | AceP2 | BGC0001491 | Polyketide | 30.0 | 57.0 | 400.0 | 1e-112 |
| CBA11582.1 | polyketide\_synthase\_type\_I | BGC0001046 | NRP+Polyketide:Modular type I polyketide+Saccharide:Hybrid/tailoring saccharide | 30.0 | 61.6 | 399.0 | 1.07e-112 |
| QKG20159.1 | type\_I\_polyketide\_synthase | BGC0002124 | Polyketide | 31.0 | 54.0 | 395.0 | 1.1e-112 |
| API82671.1 | putative\_polyketide\_synthase | BGC0002616 | Polyketide | 27.0 | 78.3 | 399.0 | 1.13e-112 |
| QFU80900.1 | PKS | BGC0002550 | Polyketide | 31.0 | 57.8 | 399.0 | 1.25e-112 |
| QBF51755.1 | type\_I\_polyketide\_synthase | BGC0001856 | Polyketide:Modular type I polyketide | 30.0 | 53.9 | 399.0 | 1.32e-112 |
| AWW87422.1 | type\_I\_polyketide\_synthase | BGC0001755 | Polyketide | 31.0 | 57.2 | 399.0 | 1.33e-112 |
| AAG13917.1 | megalomicin\_6-deoxyerythronolide\_B\_synthase\_1 | BGC0000092 | Polyketide | 31.0 | 53.1 | 399.0 | 1.45e-112 |
| AKG06377.1 | polyketide\_synthase\_type\_1 | BGC0001830 | Polyketide | 31.0 | 60.2 | 399.0 | 1.5e-112 |
| AGI99496.1 | Type\_I\_polyketide\_synthase | BGC0001004 | Polyketide:Modular type I polyketide | 31.0 | 57.8 | 399.0 | 1.5e-112 |
| WP\_019032757.1 | type\_I\_polyketide\_synthase | BGC0001331 | NRP:Cyclic depsipeptide+Polyketide:Modular type I polyketide | 31.0 | 53.5 | 398.0 | 1.88e-112 |
| ACR50773.1 | polyketide\_synthase | BGC0000163 | Polyketide | 29.0 | 60.2 | 399.0 | 2.37e-112 |
| ctg1\_orf523 |  | BGC0001199 | Polyketide | 32.0 | 52.6 | 398.0 | 2.82e-112 |
| ADC79616.1 | BafAI | BGC0000028 | Polyketide:Modular type I polyketide | 33.0 | 51.7 | 398.0 | 3.07e-112 |
| AXI91549.1 | FunP4 | BGC0001944 | Polyketide | 28.0 | 67.0 | 398.0 | 3.07e-112 |
| API82664.1 | putative\_polyketide\_synthase | BGC0001677 | Polyketide | 27.0 | 78.3 | 397.0 | 3.52e-112 |
| BCB17033.1 | modular\_polyketide\_synthase | BGC0002523 | NRP | 28.0 | 72.2 | 398.0 | 3.81e-112 |
| AWH12937.1 | StmB | BGC0001784 | Polyketide | 30.0 | 61.5 | 398.0 | 4.04e-112 |
| TXD00033.1 | SDR\_family\_NAD(P)-dependent\_oxidoreductase | BGC0001877 | Polyketide | 30.0 | 53.1 | 398.0 | 4.1e-112 |
| AAC01713.1 | RifD | BGC0000136 | Polyketide | 28.0 | 61.1 | 394.0 | 4.45e-112 |
| BBD17742.1 | polyketide\_synthase | BGC0001918 | NRP+Polyketide | 33.0 | 53.2 | 394.0 | 4.54e-112 |
| EJK79843.1 | amino\_acid\_adenylation\_enzyme/thioester\_reductase\_family\_protein | BGC0000436 | NRP | 28.0 | 71.5 | 397.0 | 4.99e-112 |
| ABB88519.1 | polyketide\_synthase\_type\_I | BGC0000050 | Polyketide | 32.0 | 54.4 | 397.0 | 5.09e-112 |
| ARV85763.1 | PieA4\_type\_I\_PKS | BGC0001742 | Polyketide | 28.0 | 61.3 | 396.0 | 5.14e-112 |
| ANY10590.1 | polyketide\_synthase | BGC0001773 | Polyketide | 30.0 | 55.7 | 397.0 | 5.48e-112 |
| ALA09355.1 | type\_I\_modular\_PKS | BGC0001303 | Polyketide | 32.0 | 53.5 | 397.0 | 6.87e-112 |
| TXD00025.1 | SDR\_family\_NAD(P)-dependent\_oxidoreductase | BGC0001877 | Polyketide | 31.0 | 53.2 | 397.0 | 7.41e-112 |
| ABO15888.1 | polyketide\_synthase | BGC0000132 | Polyketide | 33.0 | 45.1 | 395.0 | 7.72e-112 |
| CAM00062.1 | EryAI\_Erythromycin\_polyketide\_synthase\_modules\_1\_and\_2 | BGC0000055 | Polyketide:Modular type I polyketide+Saccharide:Hybrid/tailoring saccharide | 32.0 | 53.0 | 397.0 | 7.91e-112 |
| ADC79618.1 | BafAIII | BGC0000028 | Polyketide:Modular type I polyketide | 30.0 | 57.0 | 396.0 | 1.13e-111 |
| QFU19825.1 | PKS | BGC0002431 | Polyketide+Saccharide | 31.0 | 53.2 | 396.0 | 1.25e-111 |
| BAD08373.1 | polyketide\_synthase\_modules\_1-3 | BGC0000167 | Polyketide | 29.0 | 62.4 | 396.0 | 1.36e-111 |
| ABY83164.1 | Azi26 | BGC0000960 | NRP+Polyketide | 29.0 | 60.1 | 393.0 | 1.46e-111 |
| CAQ18832.1 | polyketide\_synthase | BGC0000954 | NRP+Polyketide:Modular type I polyketide | 28.0 | 70.4 | 395.0 | 1.67e-111 |
| AGU50952.1 | putative\_polyketide\_synthase | BGC0002417 | NRP+Polyketide | 31.0 | 55.0 | 390.0 | 2.01e-111 |
| QEA08887.1 | JenA1 | BGC0002559 | Polyketide | 32.0 | 54.1 | 395.0 | 2.03e-111 |
| AWH12668.1 | RmpC | BGC0001759 | Polyketide | 30.0 | 60.6 | 392.0 | 2.15e-111 |
| WP\_234353271.1 | SDR\_family\_NAD(P)-dependent\_oxidoreductase | BGC0001537 | Polyketide | 30.0 | 66.8 | 394.0 | 2.52e-111 |
| ANY10589.1 | polyketide\_synthase | BGC0001773 | Polyketide | 30.0 | 59.2 | 395.0 | 2.53e-111 |
| AAP42855.1 | NanA1 | BGC0000105 | Polyketide | 31.0 | 57.3 | 395.0 | 2.72e-111 |
| QFU19843.1 | PKS | BGC0002431 | Polyketide+Saccharide | 33.0 | 51.0 | 394.0 | 2.84e-111 |
| QFU80899.1 | PKS | BGC0002550 | Polyketide | 31.0 | 59.4 | 392.0 | 2.9e-111 |
| AKL64830.1 | polyketide\_synthase | BGC0002072 | Polyketide:Modular type I polyketide | 30.0 | 53.8 | 395.0 | 3e-111 |
| AAX98190.1 | polyketide\_synthase\_type\_I | BGC0000052 | Polyketide | 30.0 | 54.8 | 395.0 | 3.02e-111 |
| AGM05534.1 | modular\_polyketide\_synthase | BGC0002098 | Polyketide | 32.0 | 52.7 | 395.0 | 3.05e-111 |
| QKG20145.1 | type\_I\_polyketide\_synthase | BGC0002124 | Polyketide | 31.0 | 53.0 | 395.0 | 3.11e-111 |
| AAP42867.1 | NanA7 | BGC0000105 | Polyketide | 31.0 | 53.0 | 391.0 | 3.45e-111 |
| BBM96639.1 | modular\_polyketide\_synthase | BGC0002452 | Polyketide | 31.0 | 57.3 | 395.0 | 3.56e-111 |
| AGI99495.1 | Type\_I\_polyketide\_synthase | BGC0001004 | Polyketide:Modular type I polyketide | 31.0 | 59.4 | 392.0 | 3.88e-111 |
| ADC79637.1 | TamAI | BGC0001052 | NRP+Polyketide:Modular type I polyketide | 31.0 | 53.3 | 395.0 | 4.21e-111 |
| WP\_159041997.1 | SDR\_family\_NAD(P)-dependent\_oxidoreductase | BGC0002033 | Polyketide | 32.0 | 54.2 | 394.0 | 4.47e-111 |
| BAW35611.1 | modular\_polyketide\_synthase | BGC0002357 | Polyketide+Other | 31.0 | 52.6 | 390.0 | 4.92e-111 |
| AHD05615.1 | putative\_non-ribosomal\_peptide\_ligase/\_polyketide\_synthase\_hybrid | BGC0001033 | NRP+Polyketide | 33.0 | 52.9 | 394.0 | 4.98e-111 |
| ADU85981.1 | putative\_modular\_polyketide\_synthase | BGC0000165 | Polyketide:Modular type I polyketide | 32.0 | 52.9 | 389.0 | 5.49e-111 |
| AEH42473.1 | polyketide\_synthase | BGC0000032 | Polyketide | 32.0 | 53.8 | 394.0 | 6.44e-111 |
| QBG82528.1 | Polyketide\_synthase | BGC0002587 | Polyketide | 32.0 | 54.1 | 394.0 | 6.48e-111 |
| ADM46356.1 | polyketide\_synthase | BGC0000106 | Polyketide | 30.0 | 58.9 | 394.0 | 7.34e-111 |
| QKV49765.1 | PKS | BGC0002526 | Polyketide | 32.0 | 54.9 | 393.0 | 8.28e-111 |
| ANY10599.1 | polyketide\_synthase | BGC0001773 | Polyketide | 30.0 | 61.2 | 392.0 | 8.71e-111 |
| ARW71483.1 | type\_I\_PKS\_loading\_module,\_module\_1,\_module\_2 | BGC0001812 | Polyketide | 30.0 | 58.2 | 394.0 | 8.75e-111 |
| AQW44871.1 | polyketide\_synthase | BGC0001761 | Polyketide | 38.0 | 38.5 | 393.0 | 8.93e-111 |
| ANH11414.1 | SceS | BGC0001770 | Polyketide | 30.0 | 56.5 | 394.0 | 9.37e-111 |
| ADC79619.1 | BafAIV | BGC0000028 | Polyketide:Modular type I polyketide | 30.0 | 57.7 | 393.0 | 1e-110 |
| ABL74938.1 | PKS | BGC0001048 | NRP:Glycopeptide+Polyketide:Modular type I polyketide+Saccharide:Hybrid/tailoring saccharide | 31.0 | 52.5 | 388.0 | 1.01e-110 |
| BAP34733.1 | type\_I\_polyketide\_synthase | BGC0000078 | Polyketide | 31.0 | 56.2 | 393.0 | 1.03e-110 |
| CAA60462.1 | polyketide\_synthase | BGC0001040 | NRP+Polyketide | 29.0 | 59.1 | 393.0 | 1.33e-110 |
| QRI43527.1 | type\_I\_polyketide\_synthase | BGC0002454 | Polyketide | 29.0 | 67.9 | 393.0 | 1.36e-110 |
| ARW71486.1 | type\_I\_PKS\_module\_6 | BGC0001812 | Polyketide | 31.0 | 53.5 | 388.0 | 1.48e-110 |
| AWX24483.1 | type\_I\_polyketide\_synthase | BGC0001695 | NRP | 31.0 | 52.5 | 386.0 | 1.55e-110 |
| QCF28928.1 | type\_I\_polyketide\_synthase | BGC0002308 | Alkaloid+Polyketide | 33.0 | 49.7 | 393.0 | 1.56e-110 |
| QWF78545.1 | 3-ketoacyl-CoA\_thiolase | BGC0002142 | Polyketide | 27.0 | 73.4 | 393.0 | 1.71e-110 |
| BAD08358.1 | polyketide\_synthase\_modules\_4 | BGC0000167 | Polyketide | 28.0 | 63.7 | 392.0 | 1.78e-110 |
| AAC68815.1 | FK506\_polyketide\_synthase | BGC0000353 | NRP | 30.0 | 57.1 | 393.0 | 1.87e-110 |
| AXI91546.1 | FunP7 | BGC0001944 | Polyketide | 29.0 | 67.4 | 392.0 | 2.44e-110 |
| ACZ65476.1 | type\_I\_modular\_polyketide\_synthase | BGC0000140 | Polyketide | 32.0 | 55.1 | 377.0 | 2.58e-110 |
| KFG78606.1 | polyketide\_synthase | BGC0002240 | Polyketide | 28.0 | 71.4 | 392.0 | 2.76e-110 |
| ANZ22987.1 | ZinD | BGC0001828 | Polyketide | 30.0 | 52.5 | 392.0 | 2.86e-110 |
| OSS48297.1 | hypothetical\_protein | BGC0002194 | Polyketide | 28.0 | 70.3 | 391.0 | 2.99e-110 |
| AWH12669.1 | RmpB | BGC0001759 | Polyketide | 32.0 | 53.8 | 392.0 | 3.81e-110 |
| ACS20361.1 | KR\_domain\_protein | BGC0002420 | NRP+Polyketide | 31.0 | 55.0 | 386.0 | 3.91e-110 |
| ADH04640.1 | TgaB | BGC0001051 | NRP+Polyketide:Modular type I polyketide | 31.0 | 55.5 | 391.0 | 4.04e-110 |
| QQZ01584.1 | PKS | BGC0002498 | Other | 30.0 | 52.7 | 388.0 | 4.13e-110 |
| ACY06288.1 | type\_I\_polyketide\_synthase | BGC0001042 | NRP+Polyketide | 31.0 | 52.2 | 391.0 | 4.21e-110 |
| QIQ28617.1 | Nbc21 | BGC0002541 | Other | 33.0 | 50.3 | 391.0 | 5.07e-110 |
| BAB69196.1 | modular\_polyketide\_synthase | BGC0000117 | Polyketide | 30.0 | 53.1 | 391.0 | 5.57e-110 |
| AAC69331.1 | type\_I\_polyketide\_synthase\_PikAIII | BGC0000094 | Polyketide:Modular type I polyketide+Saccharide:Hybrid/tailoring saccharide | 30.0 | 52.8 | 386.0 | 5.82e-110 |
| BAP34763.1 | type\_I\_polyketide\_synthase | BGC0000078 | Polyketide | 29.0 | 69.5 | 391.0 | 5.86e-110 |
| BAQ25482.1 | type\_I\_polyketide\_synthase | BGC0001288 | Polyketide | 29.0 | 70.7 | 391.0 | 5.92e-110 |
| AEH42474.1 | polyketide\_synthase | BGC0000032 | Polyketide | 30.0 | 54.7 | 389.0 | 9.71e-110 |
| QBG82532.1 | Polyketide\_synthase | BGC0002587 | Polyketide | 30.0 | 58.9 | 390.0 | 9.76e-110 |
| ctg1\_orf9 |  | BGC0000053 | Polyketide | 30.0 | 53.1 | 386.0 | 1.13e-109 |
| CAM00065.1 | EryAIII\_Erythromycin\_polyketide\_synthase\_modules\_5\_and\_6 | BGC0000055 | Polyketide:Modular type I polyketide+Saccharide:Hybrid/tailoring saccharide | 30.0 | 54.5 | 390.0 | 1.17e-109 |
| ADU86003.1 | putative\_modular\_polyketide\_synthase | BGC0000165 | Polyketide:Modular type I polyketide | 30.0 | 56.6 | 390.0 | 1.18e-109 |
| CCA29203.1 | non-ribosomal\_peptide\_synthetase/polyketide\_synthase | BGC0000955 | NRP+Polyketide:Modular type I polyketide | 32.0 | 54.1 | 390.0 | 1.21e-109 |
| PHM26614.1 | Phthiocerol\_synthesis\_polyketide\_synthase\_type\_I\_PpsE | BGC0001130 | NRP+Polyketide | 31.0 | 55.0 | 386.0 | 1.38e-109 |
| QLD23837.1 | SDR\_family\_NAD(P)-dependent\_oxidoreductase | BGC0002086 | Polyketide | 30.0 | 53.5 | 385.0 | 1.57e-109 |
| AHB82065.1 | polyketide\_synthase | BGC0001231 | NRP+Polyketide:Modular type I polyketide | 36.0 | 38.2 | 370.0 | 1.57e-109 |
| AAF86392.1 | FkbC | BGC0000994 | NRP+Polyketide | 30.0 | 60.2 | 389.0 | 1.72e-109 |
| AAC69329.1 | type\_I\_polyketide\_synthase\_PikAI | BGC0000094 | Polyketide:Modular type I polyketide+Saccharide:Hybrid/tailoring saccharide | 30.0 | 53.8 | 389.0 | 1.99e-109 |
| QUQ72353.1 | type\_I\_polyketide\_synthase | BGC0002349 | Polyketide+Saccharide | 31.0 | 52.1 | 389.0 | 2.44e-109 |
| ABF87031.1 | non-ribosomal\_peptide\_synthetase/polyketide\_synthase | BGC0000393 | NRP+Polyketide:Modular type I polyketide | 31.0 | 54.4 | 389.0 | 2.65e-109 |
| CAQ34929.1 | putative\_polyketide\_synthase | BGC0000986 | NRP+Polyketide | 29.0 | 54.0 | 372.0 | 4.35e-109 |
| QQZ01587.1 | PKS | BGC0002498 | Other | 30.0 | 52.4 | 388.0 | 4.84e-109 |
| QWF78550.1 | 3-ketoacyl-CoA\_thiolase | BGC0002142 | Polyketide | 30.0 | 52.5 | 388.0 | 5.21e-109 |
| WP\_063764078.1 | polyketide\_synthase | BGC0001348 | Polyketide:Modular type I polyketide | 29.0 | 67.9 | 387.0 | 6.17e-109 |
| ARE67851.1 | AbsB3 | BGC0001492 | Polyketide | 29.0 | 55.6 | 374.0 | 7.02e-109 |
| UHH90010.1 | VicP2 | BGC0002634 | Polyketide+NRP+Other | 28.0 | 70.4 | 387.0 | 7.08e-109 |
| ABG02264.1 | SalB | BGC0000143 | Polyketide | 31.0 | 54.3 | 387.0 | 8.45e-109 |
| AAC01711.1 | RifB | BGC0000136 | Polyketide | 33.0 | 53.8 | 387.0 | 8.51e-109 |
| AAG13918.1 | megalomicin\_6-deoxyerythronolide\_B\_synthase\_2 | BGC0000092 | Polyketide | 28.0 | 71.5 | 387.0 | 9.27e-109 |
| CAA60460.1 | polyketide\_synthase | BGC0001040 | NRP+Polyketide | 29.0 | 59.0 | 387.0 | 9.98e-109 |
| BAC57028.1 | protomycinolide\_IV\_synthase\_1 | BGC0000102 | Polyketide | 31.0 | 52.6 | 387.0 | 1.04e-108 |
| QLD23835.2 | SDR\_family\_NAD(P)-dependent\_oxidoreductase | BGC0002086 | Polyketide | 30.0 | 58.6 | 387.0 | 1.06e-108 |
| AAF71775.1 | nysB | BGC0000115 | Polyketide:Modular type I polyketide+Saccharide:Hybrid/tailoring saccharide | 30.0 | 52.9 | 387.0 | 1.12e-108 |
| QTT72099.1 | type\_I\_polyketide\_synthase | BGC0002350 | NRP+Polyketide+Saccharide | 32.0 | 53.3 | 385.0 | 1.15e-108 |
| BAC76492.1 | lankamycin\_synthase\_LkmAII | BGC0000085 | Polyketide | 29.0 | 61.9 | 387.0 | 1.25e-108 |
| BAK64637.1 | polyketide\_synthase | BGC0000135 | Polyketide | 32.0 | 52.9 | 386.0 | 2.01e-108 |
| ARS01475.1 | NcmAIII | BGC0001702 | NRP+Polyketide | 31.0 | 52.4 | 381.0 | 2.5e-108 |
| ABV97153.1 | Beta-ketoacyl\_synthase | BGC0000137 | Polyketide | 29.0 | 67.3 | 384.0 | 2.61e-108 |
| UHH90025.1 | VicP1 | BGC0002634 | Polyketide+NRP+Other | 29.0 | 62.0 | 385.0 | 3.69e-108 |
| AAQ84146.1 | Plm6 | BGC0000123 | Polyketide | 31.0 | 53.0 | 381.0 | 4.27e-108 |
| AAC69332.1 | type\_I\_polyketide\_synthase\_PikAIV | BGC0000094 | Polyketide:Modular type I polyketide+Saccharide:Hybrid/tailoring saccharide | 30.0 | 52.4 | 377.0 | 5.18e-108 |
| AKG06378.1 | polyketide\_synthase\_type\_1 | BGC0001830 | Polyketide | 30.0 | 53.5 | 380.0 | 5.35e-108 |
| AFV52200.1 | polyketide\_synthase\_module | BGC0000081 | NRP+Polyketide:Iterative type I polyketide+Polyketide:Enediyne type I polyketide | 32.0 | 55.9 | 382.0 | 5.44e-108 |
| CAA60459.1 | polyketide\_synthase | BGC0001040 | NRP+Polyketide | 31.0 | 58.1 | 385.0 | 5.6e-108 |
| QNN81298.1 | IonAII | BGC0002446 | Polyketide | 29.0 | 54.1 | 381.0 | 6.03e-108 |
| AEC13072.1 | fosF | BGC0000060 | Polyketide | 31.0 | 55.4 | 382.0 | 8.87e-108 |
| ADC45515.1 | modular\_polyketide\_synthase | BGC0000093 | Polyketide | 30.0 | 55.7 | 382.0 | 9.47e-108 |
| QUQ72348.1 | type\_I\_polyketide\_synthase | BGC0002349 | Polyketide+Saccharide | 31.0 | 52.0 | 384.0 | 1.03e-107 |
| ACN64831.1 | PokM1 | BGC0001061 | Polyketide:Iterative type I polyketide+Polyketide:Type II polyketide+Saccharide:Hybrid/tailoring saccharide | 29.0 | 60.2 | 381.0 | 1.25e-107 |
| AJO72735.1 | Type\_I\_modular\_polyketide\_synthase | BGC0001381 | Polyketide | 31.0 | 53.0 | 384.0 | 1.4e-107 |
| AAZ94390.1 | modular\_polyketide\_synthase | BGC0000040 | Polyketide | 30.0 | 56.9 | 384.0 | 1.42e-107 |
| CAJ88177.1 | Type\_I\_modular\_polyketide\_synthase | BGC0000151 | Polyketide:Modular type I polyketide+Saccharide:Hybrid/tailoring saccharide | 31.0 | 55.0 | 384.0 | 1.5e-107 |
| AZF85945.1 | type\_I\_polyketide\_synthase | BGC0001963 | NRP+Polyketide | 30.0 | 54.3 | 383.0 | 1.88e-107 |
| BAW35614.1 | modular\_polyketide\_synthase | BGC0002357 | Polyketide+Other | 29.0 | 52.4 | 383.0 | 1.89e-107 |
| CAQ52623.1 | type\_I\_polyketide\_synthase,\_module\_6 | BGC0001066 | Polyketide:Modular type I polyketide | 28.0 | 61.2 | 382.0 | 1.9e-107 |
| AAG02357.1 | polyketide\_synthase | BGC0000963 | NRP:Glycopeptide+Polyketide:Modular type I polyketide+Saccharide:Hybrid/tailoring saccharide | 31.0 | 53.3 | 381.0 | 2.03e-107 |
| AQH32482.1 | type\_1\_polyketide\_synthase | BGC0001667 | NRP+Polyketide | 36.0 | 40.1 | 382.0 | 3.82e-107 |
| QPP46750.1 | polyketide\_synthase | BGC0002500 | Polyketide | 30.0 | 57.3 | 382.0 | 4.23e-107 |
| AEZ53953.1 | polyketide\_synthase | BGC0000144 | Polyketide:Modular type I polyketide | 29.0 | 57.8 | 381.0 | 5.09e-107 |
| WP\_053138519.1 | type\_I\_polyketide\_synthase | BGC0002033 | Polyketide | 31.0 | 54.2 | 377.0 | 7.87e-107 |
| AEW98134.1 | polyketide\_synthase | BGC0002642 | Alkaloid | 29.0 | 68.5 | 380.0 | 1.11e-106 |
| AAO62584.1 | polyketide\_synthase\_type\_1 | BGC0001016 | NRP+Polyketide | 37.0 | 38.8 | 380.0 | 1.18e-106 |
| ATV95616.1 | 6-methylsalicylic\_acid\_synthase | BGC0001503 | Polyketide | 29.0 | 60.6 | 378.0 | 1.27e-106 |
| CAL58681.1 | polyketide\_synthase | BGC0000149 | Polyketide:Modular type I polyketide | 32.0 | 50.8 | 380.0 | 1.29e-106 |
| AUA09465.1 | Erythronolide\_synthase,\_modules\_5\_and\_6 | BGC0002291 | Polyketide | 30.0 | 53.8 | 377.0 | 1.75e-106 |
| CBA11584.1 | polyketide\_synthase\_type\_I | BGC0001046 | NRP+Polyketide:Modular type I polyketide+Saccharide:Hybrid/tailoring saccharide | 32.0 | 53.5 | 380.0 | 1.93e-106 |
| AWH12938.1 | StmC | BGC0001784 | Polyketide | 29.0 | 67.9 | 380.0 | 1.99e-106 |
| CCE88377.1 | non-ribosomal\_peptide\_synthetase/polyketide\_synthase | BGC0001034 | NRP+Polyketide:Modular type I polyketide | 31.0 | 51.9 | 380.0 | 2.11e-106 |
| CAI94713.1 | putative\_polyketide\_synthase | BGC0000141 | Polyketide | 31.0 | 59.5 | 380.0 | 2.37e-106 |
| AAQ84144.1 | Plm4 | BGC0000123 | Polyketide | 32.0 | 49.5 | 375.0 | 2.94e-106 |
| CAJ76298.1 | putative\_hybrid\_polyketide-non-ribosomal\_peptide\_synthetase | BGC0000972 | NRP+Polyketide:Modular type I polyketide+Polyketide:Trans-AT type I polyketide | 32.0 | 52.6 | 379.0 | 3.15e-106 |
| QBG82531.1 | cytochrome\_P450 | BGC0002587 | Polyketide | 32.0 | 52.6 | 379.0 | 3.95e-106 |
| BAB69193.1 |  | BGC0000117 | Polyketide | 31.0 | 53.4 | 379.0 | 4.09e-106 |
| AAP85336.1 | type\_I\_PKS | BGC0000233 | Polyketide | 30.0 | 56.2 | 364.0 | 4.27e-106 |
| EHK80171.1 | modular\_polyketide\_synthase | BGC0001447 | Polyketide | 29.0 | 55.5 | 377.0 | 4.51e-106 |
| AJO72737.1 | Type\_I\_modular\_polyketide\_synthase | BGC0001381 | Polyketide | 32.0 | 52.9 | 379.0 | 4.6e-106 |
| ARV85760.1 | PieA1\_type\_I\_PKS | BGC0001742 | Polyketide | 27.0 | 67.2 | 378.0 | 6.05e-106 |
| AAU93805.2 | polyketide\_synthase\_modules\_5\_and\_6 | BGC0000054 | Polyketide | 30.0 | 54.6 | 378.0 | 7.31e-106 |
| ADC79617.1 | BafAII | BGC0000028 | Polyketide:Modular type I polyketide | 30.0 | 54.3 | 378.0 | 7.36e-106 |
| ATP76241.1 | NdaD | BGC0001705 | NRP+Polyketide | 37.0 | 39.2 | 378.0 | 8.47e-106 |
| AXM42948.1 | type\_1\_polyketide\_synthase | BGC0001941 | NRP+Polyketide | 36.0 | 38.8 | 377.0 | 9.5e-106 |
| CAO85893.1 | modular\_polyketide\_synthase\_NorA | BGC0000110 | Polyketide:Modular type I polyketide | 29.0 | 60.4 | 376.0 | 1.02e-105 |
| QBG82518.1 | Polyketide\_synthase | BGC0002587 | Polyketide | 29.0 | 57.1 | 377.0 | 1.03e-105 |
| QQZ01628.1 | PKS | BGC0002497 | Other | 28.0 | 57.1 | 377.0 | 1.07e-105 |
| ABY21540.1 | AngAIII | BGC0000018 | Polyketide | 30.0 | 58.4 | 377.0 | 1.95e-105 |
| AZF85934.1 | type\_I\_polyketide\_synthase | BGC0001963 | NRP+Polyketide | 35.0 | 38.5 | 362.0 | 2.22e-105 |
| WP\_033261454.1 | type\_I\_polyketide\_synthase | BGC0002009 | Polyketide | 31.0 | 53.9 | 377.0 | 2.25e-105 |
| AQZ37114.1 | polyketide\_synthase | BGC0001511 | Polyketide | 31.0 | 58.1 | 376.0 | 2.94e-105 |
| ABV97152.1 | Beta-ketoacyl\_synthase | BGC0000137 | Polyketide | 28.0 | 67.0 | 376.0 | 3.02e-105 |
| BAC76491.1 | lankamycin\_synthase\_LkmAIII | BGC0000085 | Polyketide | 32.0 | 52.6 | 376.0 | 3.06e-105 |
| IF55\_RS32375 | beta-ketoacyl\_synthase | BGC0001348 | Polyketide:Modular type I polyketide | 29.0 | 57.5 | 376.0 | 3.47e-105 |
| ABY66019.1 | 6-methylsalicylic\_acid\_synthase | BGC0001008 | Polyketide:Iterative type I polyketide+Polyketide:Enediyne type I polyketide | 30.0 | 58.3 | 373.0 | 4.17e-105 |
| AKQ52532.1 | nonribosomal\_peptide\_synthetase | BGC0002533 | NRP+Polyketide | 31.0 | 55.7 | 375.0 | 6.14e-105 |
| MBN3579113.1 | amino\_acid\_adenylation\_domain-containing\_protein | BGC0002613 | NRP+Polyketide | 31.0 | 52.5 | 375.0 | 8.13e-105 |
| AKA54627.1 | PKS | BGC0001216 | NRP+Polyketide | 32.0 | 53.1 | 367.0 | 8.63e-105 |
| WP\_047890614.1 | type\_I\_polyketide\_synthase | BGC0001330 | NRP:Cyclic depsipeptide+Polyketide:Modular type I polyketide | 30.0 | 56.0 | 372.0 | 1.36e-104 |
| AAC69330.1 | type\_I\_polyketide\_synthase\_PikAII | BGC0000094 | Polyketide:Modular type I polyketide+Saccharide:Hybrid/tailoring saccharide | 29.0 | 53.9 | 374.0 | 1.37e-104 |
| WP\_019032754.1 | type\_I\_polyketide\_synthase | BGC0001331 | NRP:Cyclic depsipeptide+Polyketide:Modular type I polyketide | 31.0 | 52.8 | 372.0 | 1.78e-104 |
| AAG13919.1 | megalomicin\_6-deoxyerythronolide\_B\_synthase\_3 | BGC0000092 | Polyketide | 30.0 | 56.4 | 373.0 | 2.82e-104 |
| AMJ52080.1 | lijA | BGC0002255 | Polyketide | 29.0 | 70.7 | 372.0 | 5.25e-104 |
| AAK89729.1 | polyketide\_synthase,\_siderophore\_biosynthesis\_protein | BGC0002107 | NRP+Polyketide | 32.0 | 52.9 | 368.0 | 5.36e-104 |
| ACY06290.1 | type\_I\_polyketide\_synthase | BGC0001042 | NRP+Polyketide | 30.0 | 56.2 | 372.0 | 6.88e-104 |
| OAP25820.1 | Erythronolide\_synthase,\_modules\_1\_and\_2 | BGC0001658 | Polyketide | 30.0 | 53.5 | 368.0 | 9.07e-104 |
| BBD17760.1 | polyketide\_synthase | BGC0001919 | NRP+Polyketide | 31.0 | 52.5 | 365.0 | 1.11e-103 |
| KKP00963.1 | fatty\_acid\_synthase\_S-acetyltransferase | BGC0001854 | Polyketide:Iterative type I polyketide | 30.0 | 59.9 | 370.0 | 1.41e-103 |
| ABY21542.1 | AngAV | BGC0000018 | Polyketide | 30.0 | 58.8 | 369.0 | 1.63e-103 |
| AGC65513.1 | TtcA | BGC0001876 | NRP | 30.0 | 53.4 | 369.0 | 1.96e-103 |
| ANZ22995.1 | ZinA | BGC0001828 | Polyketide | 31.0 | 56.0 | 370.0 | 2.61e-103 |
| AHB82054.1 | polyketide\_synthase | BGC0001019 | NRP+Polyketide:Modular type I polyketide | 36.0 | 38.3 | 354.0 | 2.89e-103 |
| QSE03602.1 | LcmA | BGC0002333 | Polyketide | 30.0 | 52.2 | 369.0 | 3.36e-103 |
| ACM79805.1 | ZmaA | BGC0001059 | NRP+Polyketide | 30.0 | 53.8 | 369.0 | 3.64e-103 |
| AHD05619.1 | putative\_polyketide\_synthase\_subunit | BGC0001033 | NRP+Polyketide | 30.0 | 53.1 | 365.0 | 4.38e-103 |
| KFL51883.1 | amino\_acid\_adenylation\_protein | BGC0001711 | NRP+Polyketide | 36.0 | 38.6 | 369.0 | 5.79e-103 |
| ABP55222.1 | beta-ketoacyl\_synthase | BGC0000142 | Polyketide | 32.0 | 52.8 | 369.0 | 5.87e-103 |
| AHH99922.1 | PKS\_I | BGC0000002 | Polyketide | 31.0 | 52.7 | 368.0 | 1.01e-102 |
| ARE67852.1 | AbsB2 | BGC0001492 | Polyketide | 26.0 | 74.2 | 368.0 | 1.19e-102 |
| AFA26384.1 | polyketide\_synthase\_A | BGC0001874 | NRP+Polyketide | 30.0 | 54.3 | 368.0 | 1.25e-102 |
| AAB66504.1 | tylactone\_synthase\_starter\_module\_and\_modules\_1\_&\_2 | BGC0000166 | Polyketide | 28.0 | 59.4 | 368.0 | 1.34e-102 |
| AJO72736.1 | Type\_I\_modular\_polyketide\_synthase | BGC0001381 | Polyketide | 31.0 | 54.1 | 368.0 | 1.64e-102 |
| ASK38717.1 | polyketide\_synthase | BGC0001436 | Polyketide:Iterative type I polyketide | 29.0 | 63.1 | 367.0 | 1.84e-102 |
| AGC09485.1 | LobS2 | BGC0001183 | Polyketide | 30.0 | 57.9 | 367.0 | 2.13e-102 |
| AAV66110.2 | fusaridione\_A\_synthetase | BGC0000992 | NRP+Polyketide | 30.0 | 59.5 | 367.0 | 2.22e-102 |
| ABP73645.1 | SalA | BGC0000145 | NRP+Polyketide | 32.0 | 50.7 | 365.0 | 3.9e-102 |
| AAB66507.1 | tylactone\_synthase\_module\_6 | BGC0000166 | Polyketide | 29.0 | 54.1 | 362.0 | 6.15e-102 |
| QIQ28639.1 | Nbc43 | BGC0002541 | Other | 29.0 | 56.4 | 365.0 | 6.56e-102 |
| AJO72743.1 | Type\_I\_modular\_polyketide\_synthase | BGC0001381 | Polyketide | 32.0 | 52.2 | 365.0 | 7.02e-102 |
| AAO56104.1 | yersiniabactin\_polyketide/non-ribosomal\_peptide\_synthetase | BGC0002570 | NRP+Polyketide | 31.0 | 52.1 | 365.0 | 7.6e-102 |
| antaD | Type\_I\_PKS | BGC0001455 | NRP+Polyketide | 31.0 | 54.0 | 357.0 | 1.08e-101 |
| AAF86393.1 | FkbB | BGC0000994 | NRP+Polyketide | 30.0 | 56.7 | 365.0 | 1.14e-101 |
| WP\_019634550.1 | type\_I\_polyketide\_synthase | BGC0001443 | NRP+Polyketide | 31.0 | 52.2 | 361.0 | 1.26e-101 |
| ABP53498.1 | PKS\_(ACP-AT-AT-KS-ACP-C) | BGC0001041 | NRP+Polyketide | 31.0 | 50.6 | 363.0 | 1.59e-101 |
| CAO91861.1 | PKS-NRPS\_hybrid | BGC0000968 | NRP+Polyketide:Iterative type I polyketide | 29.0 | 59.0 | 364.0 | 2.08e-101 |
| AAS79459.1 | polyketide\_synthase\_subunit | BGC0000035 | Polyketide | 31.0 | 52.8 | 364.0 | 2.2e-101 |
| CCP20048.1 | divL1\_protein | BGC0001119 | Polyketide:Modular type I polyketide | 26.0 | 70.3 | 362.0 | 4.44e-101 |
| AXI91550.1 | FunP3 | BGC0001944 | Polyketide | 30.0 | 52.7 | 362.0 | 5.19e-101 |
| CDG12864.1 | non-ribosomal\_peptide\_synthetase | BGC0001415 | NRP+Polyketide | 31.0 | 53.5 | 362.0 | 7.28e-101 |
| BAJ14522.1 | polyketide\_synthase | BGC0001254 | Polyketide | 29.0 | 59.1 | 361.0 | 1.23e-100 |
| ABY21538.1 | AngAI | BGC0000018 | Polyketide | 30.0 | 52.7 | 361.0 | 1.55e-100 |
| AKG06375.1 | polyketide\_synthase\_type\_1 | BGC0001830 | Polyketide | 30.0 | 53.7 | 360.0 | 3.58e-100 |
| OJJ98486.1 | hypothetical\_protein | BGC0002169 | Polyketide+NRP | 28.0 | 68.8 | 359.0 | 3.71e-100 |
| QOJ72663.1 | XenE | BGC0002505 | Polyketide+NRP | 28.0 | 69.6 | 360.0 | 4.48e-100 |
| QBK15047.1 | polyketide\_synthase\_ClaI | BGC0002196 | Polyketide | 29.0 | 63.9 | 359.0 | 4.63e-100 |
| ACC40923.1 | polyketide\_synthase\_Pks9 | BGC0001665 | Polyketide | 30.0 | 52.8 | 347.0 | 6.37e-100 |
| AZZ09613.1 | PvhA | BGC0002304 | Polyketide+NRP | 29.0 | 59.5 | 358.0 | 1.04e-99 |
| OAP25811.1 | Phenolphthiocerol\_synthesis\_polyketide\_synthase\_type\_I\_Pks15/1 | BGC0001658 | Polyketide | 34.0 | 39.1 | 337.0 | 1.2e-99 |
| KAF5858310.1 | HR-PKS | BGC0002139 | Polyketide | 28.0 | 67.7 | 356.0 | 5e-99 |
| KKP04599.1 | Non-ribosomal\_peptide\_synthetase\_-\_Polyketide\_synthase | BGC0002066 | NRP+Polyketide:Iterative type I polyketide | 30.0 | 60.4 | 356.0 | 7.25e-99 |
| AHV78252.1 | ResS1 | BGC0001246 | Polyketide | 29.0 | 67.5 | 355.0 | 7.85e-99 |
| ADF88262.1 | mixed\_nonribosomal\_peptide\_synthetase/\_polyketide\_synthase | BGC0000979 | NRP+Polyketide | 32.0 | 43.2 | 350.0 | 1.17e-98 |
| ADF88265.1 | mixed\_nonribosomal\_peptide\_synthetase/\_polyketide\_synthase | BGC0000980 | NRP+Polyketide | 32.0 | 43.2 | 350.0 | 1.17e-98 |
| AAS98200.1 | MSAS-type\_polyketide\_synthase | BGC0001273 | Polyketide | 27.0 | 57.1 | 353.0 | 1.24e-98 |
| CEN60541.1 | hypothetical\_protein | BGC0002266 | Terpene+Polyketide | 30.0 | 58.7 | 353.0 | 2.33e-98 |
| EPS29069.1 | hypothetical\_protein | BGC0001724 | NRP+Polyketide | 28.0 | 71.3 | 354.0 | 2.97e-98 |
| AHB38498.1 | polyketide\_synthase | BGC0000346 | NRP+Polyketide:Modular type I polyketide | 31.0 | 51.5 | 352.0 | 6.6e-98 |
| BAK26562.1 | PKS-NRPS\_hybrid | BGC0000977 | NRP+Polyketide | 29.0 | 54.6 | 352.0 | 8.83e-98 |
| WP\_010639241.1 | type\_I\_polyketide\_synthase | BGC0000958 | NRP:Cyclic depsipeptide+Polyketide:Modular type I polyketide | 31.0 | 52.8 | 345.0 | 1.92e-97 |
| BAF92601.1 | iterative\_type\_I\_PKS | BGC0000118 | Polyketide | 28.0 | 59.9 | 349.0 | 3.61e-97 |
| ACJ24875.1 | 6-methylsalicylic\_acid\_synthase | BGC0000119 | Polyketide:Iterative type I polyketide+Saccharide:Hybrid/tailoring saccharide | 28.0 | 59.9 | 349.0 | 3.61e-97 |
| OAG05545.1 | PKSKA1\_protein | BGC0002211 | Polyketide | 28.0 | 64.4 | 349.0 | 5.19e-97 |
| ARS01473.1 | NcmAI | BGC0001702 | NRP+Polyketide | 33.0 | 47.5 | 348.0 | 1.6e-96 |
| EAL85129.1 | polyketide\_synthase | BGC0001067 | Terpene+Polyketide:Iterative type I polyketide | 27.0 | 72.0 | 347.0 | 2.15e-96 |
| AEP40932.1 | polyketide\_synthase\_type\_I | BGC0000021 | Polyketide | 28.0 | 57.3 | 346.0 | 2.66e-96 |
| OAQ63050.2 | polyketide\_synthase | BGC0002187 | Polyketide | 28.0 | 67.5 | 347.0 | 2.71e-96 |
| EHA22196.1 | polyketide\_synthase | BGC0000170 | Polyketide | 28.0 | 60.6 | 346.0 | 2.75e-96 |
| ACD39774.1 | reducing\_polyketide\_synthase | BGC0000134 | Polyketide | 28.0 | 67.5 | 346.0 | 6.24e-96 |
| EAL89230.2 | LovB-like\_polyketide\_synthase,\_putative | BGC0000129 | Polyketide | 30.0 | 54.2 | 345.0 | 8.56e-96 |
| BBG28498.1 | putative\_polyketide\_synthase | BGC0001913 | Polyketide | 29.0 | 52.5 | 345.0 | 1.21e-95 |
| ATZ45182.1 | Bcboa6 | BGC0001892 | Polyketide | 29.0 | 58.7 | 344.0 | 1.99e-95 |
| OJF16267.1 | AceP5 | BGC0001491 | Polyketide | 33.0 | 47.2 | 343.0 | 4.58e-95 |
| CCT75967.1 | polyketide\_synthase | BGC0001606 | Polyketide | 26.0 | 71.8 | 343.0 | 4.79e-95 |
| BBQ09587.1 | PKS-NRPS\_hybrid | BGC0002261 | Polyketide | 29.0 | 59.6 | 343.0 | 5.29e-95 |
| BBG28484.1 | polyketide\_synthase\_CdmE | BGC0001926 | Polyketide | 27.0 | 70.8 | 342.0 | 1.49e-94 |
| AEO57481.1 | PKS-NRPSs | BGC0001449 | NRP+Alkaloid+Polyketide:Iterative type I polyketide | 29.0 | 59.2 | 342.0 | 2.12e-94 |
| QOG08944.1 | FfsA | BGC0002204 | Polyketide+NRP | 27.0 | 71.1 | 340.0 | 4.95e-94 |
| AAM54078.1 | polyketide\_synthase | BGC0000020 | Polyketide | 28.0 | 64.7 | 340.0 | 5.75e-94 |
| BAZ95823.1 | PKS-NRPS\_hybrid\_cpaA | BGC0001563 | NRP+Polyketide | 29.0 | 61.0 | 340.0 | 8.4e-94 |
| EDU47082.1 | lovastatin\_nonaketide\_synthase | BGC0002250 | Polyketide+NRP | 29.0 | 58.9 | 338.0 | 1.93e-93 |
| AGO86662.1 | equisetin\_synthetase | BGC0001255 | NRP+Polyketide | 28.0 | 60.9 | 337.0 | 5.91e-93 |
| EYT83439.1 | beta-ketoacyl\_synthase | BGC0001213 | Polyketide | 30.0 | 48.9 | 335.0 | 6.79e-93 |
| BAC20566.1 | polyketide\_synthase | BGC0000039 | Polyketide | 26.0 | 72.2 | 335.0 | 1.24e-92 |
| QGW49095.1 | putative\_polyketide\_synthase | BGC0002731 | Polyketide | 27.0 | 69.7 | 335.0 | 1.49e-92 |
| EHA52508.1 | mycocerosic\_acid\_synthase | BGC0001749 | Polyketide | 31.0 | 50.6 | 335.0 | 1.65e-92 |
| QBK15049.1 | PKS-NRPS\_hybrid\_TraA | BGC0002197 | Polyketide+NRP | 29.0 | 59.9 | 335.0 | 1.76e-92 |
| ACS68554.1 | hybrid\_PKS-NRPS\_protein | BGC0001026 | NRP+Polyketide | 29.0 | 59.1 | 335.0 | 1.78e-92 |
| ARP51711.1 | PKS-NRPS\_hybrid\_protein | BGC0001741 | NRP+Polyketide | 29.0 | 63.4 | 335.0 | 2.38e-92 |
| CAQ34917.1 | polyketide\_synthase | BGC0000986 | NRP+Polyketide | 34.0 | 38.7 | 331.0 | 2.88e-92 |
| ESU09893.1 | hypothetical\_protein | BGC0002191 | Polyketide | 26.0 | 71.9 | 334.0 | 3.77e-92 |
| AKA59437.1 | polyketide\_synthase | BGC0001202 | NRP+Polyketide | 31.0 | 47.6 | 318.0 | 5.69e-92 |
| ACZ57548.1 | polyketide\_synthase | BGC0000046 | Polyketide:Iterative type I polyketide | 29.0 | 59.9 | 333.0 | 5.92e-92 |
| QSJ20136.1 | polyketide\_synthase | BGC0002572 | NRP+Polyketide | 40.0 | 24.9 | 307.0 | 1.16e-91 |
| CBF80487.1 | hybrid\_PKS-NRPS\_(Eurofung) | BGC0000959 | NRP+Polyketide:Iterative type I polyketide | 30.0 | 59.7 | 333.0 | 1.24e-91 |
| EGX96624.1 | polyketide\_synthase,\_putative | BGC0002259 | Polyketide+NRP | 28.0 | 63.2 | 332.0 | 1.48e-91 |
| EHA28244.1 | hypothetical\_protein | BGC0001143 | Polyketide | 28.0 | 64.2 | 332.0 | 1.48e-91 |
| AGC09499.1 | LobS4 | BGC0001183 | Polyketide | 26.0 | 67.8 | 332.0 | 1.67e-91 |
| BAY02136.1 | beta-ketoacyl\_synthase | BGC0002532 | NRP+Polyketide | 34.0 | 39.0 | 328.0 | 2.03e-91 |
| CBF87072.1 | polyketide\_synthase,\_putative\_(Eurofung) | BGC0001290 | NRP | 29.0 | 61.3 | 331.0 | 2.58e-91 |
| QBC75448.1 | MacA | BGC0002615 | Terpene | 29.0 | 48.6 | 330.0 | 2.71e-91 |
| EKJ70677.1 | PKS6 | BGC0002188 | NRP+Polyketide | 29.0 | 55.3 | 331.0 | 3.45e-91 |
| EAW09117.1 | hybrid\_NRPS/PKS\_enzyme,\_putative | BGC0000983 | NRP+Polyketide:Iterative type I polyketide | 27.0 | 69.3 | 330.0 | 1.14e-90 |
| QXF14600.1 | PydA | BGC0002239 | Polyketide+NRP | 28.0 | 69.2 | 330.0 | 1.17e-90 |
| BAQ25466.1 | polyketide\_synthase | BGC0001264 | Polyketide | 29.0 | 59.2 | 329.0 | 1.19e-90 |
| AKQ22699.1 | malonyl\_CoA-acyl\_carrier\_protein\_transacylase | BGC0001186 | Polyketide | 33.0 | 38.5 | 329.0 | 1.77e-90 |
| ESU15174.1 | hypothetical\_protein | BGC0002186 | NRP+Polyketide | 28.0 | 71.1 | 328.0 | 2.38e-90 |
| AFP73394.1 | FusA | BGC0001268 | NRP+Polyketide | 29.0 | 62.2 | 328.0 | 2.51e-90 |
| QPC57090.1 | polyketide\_synthase-nonribosomal\_peptide\_synthetase | BGC0002230 | Polyketide+NRP | 26.0 | 71.0 | 328.0 | 4.54e-90 |
| BCA42568.1 | polyketide\_synthase\_GrgA | BGC0002185 | Polyketide | 29.0 | 61.0 | 327.0 | 5.24e-90 |
| AAM77986.1 | iterative\_type\_I\_polyketide\_synthase | BGC0000112 | Polyketide:Iterative type I polyketide+Polyketide:Enediyne type I polyketide | 28.0 | 59.7 | 326.0 | 5.42e-90 |
| BBC43184.1 | PKS-NRPS\_hybrid | BGC0001738 | NRP+Polyketide | 29.0 | 58.8 | 327.0 | 7.81e-90 |
| AIG62146.1 | 6-methylsalicylic\_acid\_synthase | BGC0000120 | Polyketide:Iterative type I polyketide | 27.0 | 56.5 | 325.0 | 1.32e-89 |
| QHD43130.1 | NRPS/PKS\_hybrid\_protein | BGC0002546 | NRP+Polyketide | 28.0 | 71.2 | 326.0 | 1.37e-89 |
| EPS34234.1 | nonribosomal\_peptide\_synthatase-polyketide\_synthase | BGC0002067 | NRP+Polyketide:Iterative type I polyketide | 29.0 | 59.3 | 326.0 | 1.79e-89 |
| CAO85898.1 | modular\_polyketide\_synthase\_NorC | BGC0000110 | Polyketide:Modular type I polyketide | 29.0 | 53.9 | 325.0 | 1.84e-89 |
| EAQ84779.1 | hypothetical\_protein | BGC0001219 | Polyketide | 46.0 | 22.4 | 325.0 | 2.61e-89 |
| KZL86691.1 | polyketide\_synthase | BGC0002228 | NRP | 26.0 | 69.0 | 325.0 | 2.67e-89 |
| AAT28740.1 | FUSS | BGC0000064 | NRP+Polyketide | 30.0 | 58.3 | 325.0 | 3e-89 |
| EHK18438.1 | putative\_polyketide\_synthase | BGC0002233 | Polyketide | 27.0 | 59.0 | 324.0 | 3.44e-89 |
| EAL85113.2 | hybrid\_PKS-NRPS\_enzyme | BGC0001037 | NRP+Polyketide:Iterative type I polyketide | 27.0 | 70.3 | 325.0 | 4.11e-89 |
| ADA82585.1 | hybrid\_trans-AT\_polyketide\_synthase\_-\_nonribosomal\_peptide\_synthetase | BGC0001110 | NRP+Polyketide:Trans-AT type I polyketide | 34.0 | 38.1 | 325.0 | 4.26e-89 |
| CEF75886.1 |  | BGC0001600 | Polyketide | 28.0 | 65.2 | 324.0 | 5.17e-89 |
| AHB38509.1 | polyketide\_synthase | BGC0000345 | NRP+Polyketide:Modular type I polyketide | 30.0 | 52.6 | 323.0 | 5.89e-89 |
| ADN43685.1 | PKS-NRPS | BGC0001136 | NRP+Polyketide:Iterative type I polyketide | 29.0 | 54.7 | 323.0 | 9.6e-89 |
| EJP62832.1 | polyketide\_synthase,\_putative | BGC0002203 | NRP+Polyketide+Other | 28.0 | 58.5 | 323.0 | 1.12e-88 |
| CCT72377.1 | probable\_polyketide\_synthase | BGC0001305 | Polyketide | 28.0 | 60.1 | 322.0 | 1.45e-88 |
| ARV85762.1 | PieA3\_type\_I\_PKS | BGC0001742 | Polyketide | 33.0 | 38.7 | 321.0 | 1.74e-88 |
| BCK51642.1 | modular\_polyketide\_synthase | BGC0002520 | Polyketide | 32.0 | 39.4 | 320.0 | 7.62e-88 |
| BBU42026.1 | putative\_polyketide\_synthase | BGC0002222 | Polyketide | 30.0 | 46.7 | 320.0 | 1.06e-87 |
| QTE75992.1 | ZopPKS | BGC0002224 | Polyketide | 30.0 | 46.7 | 320.0 | 1.06e-87 |
| AQZ37095.1 | polyketide\_synthase | BGC0001511 | Polyketide | 29.0 | 58.4 | 320.0 | 1.32e-87 |
| KYC42746.1 | beta-ketoacyl\_synthase | BGC0002484 | NRP+Polyketide | 40.0 | 25.0 | 295.0 | 1.62e-87 |
| ABA02239.1 | polyketide\_synthase | BGC0000098 | Polyketide | 28.0 | 60.9 | 319.0 | 1.63e-87 |
| ATZ45185.1 | Bcboa9 | BGC0001892 | Polyketide | 26.0 | 70.4 | 318.0 | 2.1e-87 |
| CAL69597.1 | PKS-NRPS | BGC0001049 | NRP+Polyketide:Iterative type I polyketide | 30.0 | 51.5 | 319.0 | 2.63e-87 |
| EHA55875.1 | polyketide\_synthase | BGC0002235 | Polyketide+NRP | 27.0 | 58.8 | 318.0 | 3.42e-87 |
| ATX68124.1 | malonyl\_CoA-acyl\_carrier\_protein\_transacylase | BGC0001795 | Polyketide | 31.0 | 38.8 | 318.0 | 3.98e-87 |
| AGC09486.1 | LobS3 | BGC0001183 | Polyketide | 27.0 | 62.6 | 317.0 | 4.89e-87 |
| AHV78245.1 | LasS1 | BGC0001245 | Polyketide | 28.0 | 60.1 | 317.0 | 8.62e-87 |
| QKV49768.1 | PKS | BGC0002526 | Polyketide | 33.0 | 36.5 | 299.0 | 1.07e-86 |
| KFL51881.1 | beta-ketoacyl\_synthase | BGC0001711 | NRP+Polyketide | 34.0 | 37.4 | 313.0 | 1.9e-86 |
| QBC19710.1 | TwmB | BGC0001954 | NRP+Polyketide | 29.0 | 60.8 | 315.0 | 2.5e-86 |
| ACR12418.1 | modular\_polyketide\_synthase,\_type\_I\_PKS | BGC0000185 | Polyketide | 39.0 | 25.0 | 316.0 | 2.54e-86 |
| BAE61265.1 |  | BGC0002238 | Polyketide | 29.0 | 55.6 | 315.0 | 2.65e-86 |
| OQD69647.1 | hypothetical\_protein | BGC0002745 | Polyketide | 29.0 | 59.6 | 315.0 | 3.73e-86 |
| ASA76643.1 | polyketide\_synthase | BGC0001751 | NRP+Polyketide | 37.0 | 29.9 | 314.0 | 3.82e-86 |
| AHA38199.1 | GphF | BGC0000069 | Polyketide | 40.0 | 25.0 | 315.0 | 4.39e-86 |
| EYE95336.1 | polyketide\_synthase | BGC0002234 | Polyketide | 28.0 | 58.3 | 313.0 | 1.04e-85 |
| EHA55860.1 | polyketide\_synthase/peptide\_synthetase | BGC0002235 | Polyketide+NRP | 27.0 | 63.7 | 313.0 | 1.59e-85 |
| CUX96955.1 | TmcH | BGC0001829 | NRP+Polyketide | 29.0 | 55.6 | 311.0 | 3.19e-85 |
| QQW45467.1 | polyketide\_synthase\_CalA' | BGC0002168 | Polyketide | 30.0 | 53.9 | 311.0 | 3.81e-85 |
| BBM05082.1 | polyketide\_synthase | BGC0002170 | Polyketide | 30.0 | 53.9 | 311.0 | 3.81e-85 |
| ARR97037.1 | SphD | BGC0001780 | NRP | 36.0 | 31.1 | 311.0 | 4.35e-85 |
| EAU38971.1 | PKS-NRPS\_hybrid | BGC0001122 | NRP+Polyketide:Iterative type I polyketide | 28.0 | 61.1 | 310.0 | 1.06e-84 |
| AAY32964.1 | DszA | BGC0001093 | NRP+Polyketide | 36.0 | 29.9 | 310.0 | 1.27e-84 |
| AEZ54376.1 | PieA3 | BGC0000124 | Polyketide | 32.0 | 39.5 | 307.0 | 1.92e-84 |
| GAW21479.1 | hypothetical\_protein | BGC0002192 | Polyketide | 26.0 | 62.8 | 309.0 | 2.05e-84 |
| AFR69333.1 | polyketide\_synthase\_SpiC1 | BGC0001045 | NRP:Cyclic depsipeptide+Polyketide:Modular type I polyketide | 35.0 | 31.9 | 303.0 | 2.31e-84 |
| ADI59531.1 | CorI | BGC0001091 | NRP+Polyketide | 36.0 | 30.4 | 308.0 | 7.26e-84 |
| EED49862.1 | hybrid\_PKS/NRPS\_enzyme,\_putative | BGC0001445 | NRP+Polyketide:Iterative type I polyketide | 27.0 | 69.6 | 308.0 | 7.32e-84 |
| ADN68476.1 | sorA | BGC0000184 | Polyketide:Trans-AT type I polyketide | 36.0 | 31.6 | 308.0 | 9.26e-84 |
| DAC80062.1 | PKS | BGC0001836 | Polyketide:Trans-AT type I polyketide | 39.0 | 25.1 | 307.0 | 1.39e-83 |
| QNH68024.1 | PfpA | BGC0002268 | Polyketide+NRP | 28.0 | 59.4 | 306.0 | 1.68e-83 |
| QBE85649.1 | BuaA | BGC0001857 | Alkaloid+NRP+Polyketide:Iterative type I polyketide | 28.0 | 58.6 | 306.0 | 2.9e-83 |
| AZY91988.1 | polyketide\_synthase | BGC0002022 | Polyketide | 33.0 | 39.2 | 304.0 | 3.02e-83 |
| AWS21290.1 | type\_I\_polyketide\_synthase | BGC0001934 | Polyketide | 33.0 | 39.2 | 304.0 | 3.52e-83 |
| ALD82522.1 | polyketide\_synthase | BGC0001212 | NRP+Polyketide | 42.0 | 25.0 | 305.0 | 3.78e-83 |
| OAP25815.1 | Phenolphthiocerol\_synthesis\_polyketide\_synthase\_type\_I\_Pks15/1 | BGC0001658 | Polyketide | 32.0 | 38.4 | 305.0 | 3.95e-83 |
| QVV57688.1 | KR\_domain-containing\_protein | BGC0002338 | Polyketide | 31.0 | 38.3 | 304.0 | 4.15e-83 |
| EAT85332.2 | hypothetical\_protein | BGC0002165 | Polyketide | 28.0 | 58.8 | 305.0 | 5.27e-83 |
| AIW00670.1 | mellein\_synthase | BGC0001244 | Polyketide | 27.0 | 57.6 | 303.0 | 5.65e-83 |
| AAF19810.1 | MtaB | BGC0001024 | NRP+Polyketide:Modular type I polyketide | 41.0 | 25.1 | 305.0 | 6.61e-83 |
| ARR97036.1 | SphC | BGC0001780 | NRP | 36.0 | 29.0 | 305.0 | 7.49e-83 |
| QCX41916.1 | Mhr10 | BGC0001956 | Polyketide | 29.0 | 54.4 | 296.0 | 9.52e-83 |
| QHD26313.1 | polyketide\_synthase | BGC0002479 | Polyketide+NRP+Saccharide | 29.0 | 54.4 | 296.0 | 9.52e-83 |
| WP\_055469550.1 | type\_I\_polyketide\_synthase | BGC0001537 | Polyketide | 30.0 | 55.0 | 303.0 | 1.29e-82 |
| ADI59533.1 | CorK | BGC0001091 | NRP+Polyketide | 36.0 | 29.7 | 303.0 | 1.52e-82 |
| DAC76733.1 | type\_I\_polyketide\_synthase | BGC0001885 | NRP+Polyketide | 34.0 | 38.5 | 293.0 | 1.67e-82 |
| QFU19831.1 | PKS | BGC0002431 | Polyketide+Saccharide | 32.0 | 37.9 | 291.0 | 1.74e-82 |
| ctg1\_orf5 |  | BGC0001329 | Polyketide+NRP:Cyclic depsipeptide | 33.0 | 36.0 | 301.0 | 3.37e-82 |
| AAY89050.1 | polyketide\_synthase | BGC0001069 | NRP+Polyketide:Trans-AT type I polyketide | 36.0 | 30.6 | 302.0 | 5.67e-82 |
| EAQ86385.1 | hypothetical\_protein | BGC0001405 | Polyketide | 30.0 | 45.7 | 301.0 | 6.77e-82 |
| EWM62997.1 | non-ribosomal\_peptide\_synthetase | BGC0001328 | NRP:Cyclic depsipeptide+Polyketide:Modular type I polyketide | 33.0 | 33.2 | 297.0 | 1.06e-81 |
| BBA21072.1 | putative\_modular\_polyketide\_synthase | BGC0001740 | NRP+Polyketide | 37.0 | 29.8 | 300.0 | 1.46e-81 |
| ABI91464.1 | beta-ketoacyl\_synthase | BGC0001094 | NRP+Polyketide | 32.0 | 38.7 | 299.0 | 2.78e-81 |
| QWF78553.1 | 3-ketoacyl-CoA\_thiolase | BGC0002142 | Polyketide | 32.0 | 38.4 | 299.0 | 3.24e-81 |
| OEI73461.1 | hypothetical\_protein | BGC0001520 | Polyketide | 36.0 | 30.1 | 289.0 | 6.82e-81 |
| ALD82521.1 | polyketide\_synthase | BGC0001212 | NRP+Polyketide | 39.0 | 25.9 | 297.0 | 1.01e-80 |
| RAT98529.1 | trans-acyltransferase\_polyketide\_synthase | BGC0001470 | Polyketide:Trans-AT type I polyketide | 35.0 | 30.6 | 297.0 | 1.06e-80 |
| QLG04866.1 | PulE | BGC0002374 | Polyketide | 35.0 | 30.7 | 298.0 | 1.17e-80 |
| ABC35522.1 | thiotemplate\_mechanism\_natural\_product\_synthetase | BGC0000186 | NRP+Polyketide:Modular type I polyketide | 35.0 | 30.1 | 297.0 | 1.19e-80 |
| AAY32965.1 | DszB | BGC0001093 | NRP+Polyketide | 37.0 | 29.6 | 297.0 | 1.41e-80 |
| QKV49783.1 | beta-ketoacyl\_synthase | BGC0002526 | Polyketide | 32.0 | 39.0 | 285.0 | 1.87e-80 |
| AFO59866.1 | ChxE | BGC0000175 | Polyketide:Trans-AT type I polyketide | 35.0 | 30.7 | 297.0 | 1.9e-80 |
| ADH01487.1 | polyketide\_synthase | BGC0000995 | NRP+Polyketide | 36.0 | 29.3 | 296.0 | 2.26e-80 |
| AIC32693.1 | FR9DEF | BGC0001113 | NRP+Polyketide | 36.0 | 29.3 | 296.0 | 2.57e-80 |
| AFX60332.1 | polyketide\_synthase | BGC0001032 | NRP+Polyketide | 35.0 | 30.0 | 296.0 | 2.6e-80 |
| AAY39344.1 | Beta-ketoacyl\_synthase:Beta-ketoacyl\_synthase:Phosphopantetheine-binding\_protein | BGC0002060 | Polyketide:Trans-AT type I polyketide | 35.0 | 32.4 | 296.0 | 3.16e-80 |
| WP\_012753526.1 | polyketide\_synthase | BGC0001991 | Polyketide | 36.0 | 25.5 | 288.0 | 3.69e-80 |
| BBI47418.1 | polyketide\_synthase | BGC0002258 | Polyketide | 28.0 | 51.6 | 295.0 | 4.46e-80 |
| AFB35635.1 | KS-AT-ACP | BGC0000935 | Polyketide+Other:Aminocoumarin | 32.0 | 38.3 | 286.0 | 5.71e-80 |
| QGY73449.1 | Itm17 | BGC0002451 | Polyketide | 38.0 | 25.9 | 295.0 | 8.84e-80 |
| ctg3\_19 |  | BGC0001853 | NRP+Polyketide:Modular type I polyketide | 39.0 | 26.4 | 295.0 | 9.92e-80 |
| CAL69889.1 | RhiB\_protein | BGC0001112 | NRP+Polyketide:Trans-AT type I polyketide | 33.0 | 31.1 | 294.0 | 1.28e-79 |
| ASA76631.1 | polyketide\_synthase | BGC0001751 | NRP+Polyketide | 33.0 | 30.3 | 294.0 | 1.37e-79 |
| ATG32074.1 | putative\_nonfunctional\_polyketide\_synthase\_module | BGC0001750 | NRP+Polyketide | 36.0 | 31.0 | 280.0 | 1.44e-79 |
| ACY01391.1 | AT-less\_polyketide\_synthase | BGC0000177 | Polyketide:Modular type I polyketide+Polyketide:Trans-AT type I polyketide | 36.0 | 31.4 | 294.0 | 1.74e-79 |
| ADN68477.1 | SorB | BGC0000184 | Polyketide:Trans-AT type I polyketide | 34.0 | 32.2 | 293.0 | 2.02e-79 |
| ATG32077.1 | polyketide\_synthase | BGC0001750 | NRP+Polyketide | 27.0 | 66.3 | 292.0 | 2.07e-79 |
| ABC33986.1 | polyketide\_synthase,\_putative | BGC0000186 | NRP+Polyketide:Modular type I polyketide | 38.0 | 25.3 | 292.0 | 4.47e-79 |
| AJQ95678.1 | polyketide\_synthase\_modules-related\_protein | BGC0002046 | NRP+Polyketide:Trans-AT type I polyketide | 33.0 | 29.4 | 292.0 | 4.52e-79 |
| AFX60313.1 | polyketide\_synthase | BGC0001031 | NRP+Polyketide | 33.0 | 32.5 | 292.0 | 4.97e-79 |
| CAN93348.1 | polyketide\_synthase | BGC0000179 | Polyketide:Trans-AT type I polyketide | 35.0 | 30.8 | 292.0 | 5.38e-79 |
| MCC5026024.1 | SDR\_family\_NAD(P)-dependent\_oxidoreductase | BGC0002118 | NRP+Polyketide | 39.0 | 25.6 | 292.0 | 6.44e-79 |
| MCC5025980.1 | SDR\_family\_NAD(P)-dependent\_oxidoreductase | BGC0002119 | NRP+Polyketide | 39.0 | 25.6 | 292.0 | 6.44e-79 |
| ATG32078.1 | polyketide\_synthase | BGC0001750 | NRP+Polyketide | 38.0 | 25.7 | 290.0 | 6.71e-79 |
| ALD83703.1 | tAT\_polyketide\_synthase | BGC0001299 | Polyketide | 38.0 | 26.1 | 291.0 | 8.87e-79 |
| mycI | polyketide\_synthase | BGC0002055 | NRP+Polyketide:Trans-AT type I polyketide | 32.0 | 34.5 | 291.0 | 1.09e-78 |
| CAG23966.1 | polyketide\_synthase\_type\_I | BGC0000181 | Polyketide | 38.0 | 25.5 | 290.0 | 1.35e-78 |
| CBK62733.1 |  | BGC0001115 | NRP+Polyketide | 34.0 | 30.4 | 291.0 | 1.49e-78 |
| CCA89328.1 | mixed\_trans-AT\_type\_I\_polyketide\_synthase/nonribosomal\_peptide\_synthetase | BGC0001111 | NRP+Polyketide:Trans-AT type I polyketide | 34.0 | 32.6 | 291.0 | 1.54e-78 |
| AMH40443.1 | PKS | BGC0001350 | Polyketide | 35.0 | 30.0 | 290.0 | 1.93e-78 |
| ACY01400.1 | AT-less\_polyketide\_synthase | BGC0000083 | Polyketide:Modular type I polyketide+Polyketide:Trans-AT type I polyketide | 39.0 | 25.2 | 290.0 | 1.99e-78 |
| ABI91467.1 | beta-ketoacyl\_synthase | BGC0001094 | NRP+Polyketide | 35.0 | 31.7 | 289.0 | 1.99e-78 |
| CAG23977.1 | polyketide\_synthase\_type\_I | BGC0000176 | Polyketide | 35.0 | 30.5 | 290.0 | 2.2e-78 |
| AQW44872.1 | polyketide\_synthase | BGC0001761 | Polyketide | 35.0 | 30.1 | 289.0 | 2.51e-78 |
| QTX15955.1 | polyketide\_synthase | BGC0002598 | Polyketide | 25.0 | 70.7 | 289.0 | 2.72e-78 |
| AIU36103.1 | LglD | BGC0000180 | Polyketide:Trans-AT type I polyketide | 36.0 | 29.7 | 290.0 | 3.06e-78 |
| DAC76730.1 | type\_I\_polyketide\_synthase | BGC0001885 | NRP+Polyketide | 37.0 | 27.3 | 289.0 | 4.52e-78 |
| AJQ95708.1 | polyketide\_synthase\_modules-related\_protein | BGC0001644 | Polyketide | 33.0 | 31.8 | 289.0 | 4.98e-78 |
| SKB24633.1 |  | BGC0002455 | Polyketide | 35.0 | 31.5 | 289.0 | 5.51e-78 |
| MCC5026025.1 | SDR\_family\_NAD(P)-dependent\_oxidoreductase | BGC0002118 | NRP+Polyketide | 38.0 | 25.7 | 289.0 | 5.94e-78 |
| MCC5025981.1 | SDR\_family\_NAD(P)-dependent\_oxidoreductase | BGC0002119 | NRP+Polyketide | 38.0 | 25.7 | 289.0 | 5.94e-78 |
| BAP05593.1 | calE | BGC0000967 | NRP+Polyketide:Trans-AT type I polyketide | 33.0 | 30.2 | 289.0 | 6.04e-78 |
| CAN89632.1 | putative\_polyketide\_synthase | BGC0001070 | NRP+Polyketide:Modular type I polyketide+Polyketide:Trans-AT type I polyketide | 38.0 | 25.7 | 288.0 | 8.78e-78 |
| ABI91469.1 | beta-ketoacyl\_synthase | BGC0001094 | NRP+Polyketide | 35.0 | 29.7 | 287.0 | 1.18e-77 |
| AIJ04683.1 | polyketide\_synthase | BGC0001383 | Polyketide | 38.0 | 25.5 | 287.0 | 1.2e-77 |
| AUS29500.1 | polyketide\_synthase | BGC0002607 | NRP+Polyketide | 25.0 | 68.7 | 287.0 | 1.39e-77 |
| AEC04356.1 | polyketide\_synthase | BGC0000178 | Polyketide:Trans-AT type I polyketide | 33.0 | 29.8 | 286.0 | 1.96e-77 |
| CBK62724.1 |  | BGC0001115 | NRP+Polyketide | 38.0 | 25.4 | 286.0 | 2.79e-77 |
| CAG23964.1 | polyketide\_synthase\_type\_I | BGC0000181 | Polyketide | 29.0 | 37.4 | 286.0 | 3.33e-77 |
| ERM18799.1 | polyketide\_synthase | BGC0000172 | Polyketide | 34.0 | 30.0 | 285.0 | 3.6e-77 |
| AAY89049.1 | polyketide\_synthase | BGC0001069 | NRP+Polyketide:Trans-AT type I polyketide | 35.0 | 29.9 | 286.0 | 3.79e-77 |
| AMH40422.1 | PKS | BGC0001350 | Polyketide | 34.0 | 30.3 | 286.0 | 4.76e-77 |
| DAC80076.1 | PKS | BGC0001835 | Polyketide | 39.0 | 26.0 | 285.0 | 5.86e-77 |
| ARR97039.1 | SphF | BGC0001780 | NRP | 35.0 | 30.1 | 284.0 | 7.24e-77 |
| AIJ04681.1 | polyketide\_synthase | BGC0001383 | Polyketide | 29.0 | 37.3 | 285.0 | 7.55e-77 |
| AEC04361.1 | polyketide\_synthase | BGC0000178 | Polyketide:Trans-AT type I polyketide | 37.0 | 29.3 | 283.0 | 8.18e-77 |
| ACY01390.1 | AT-less\_polyketide\_synthase | BGC0000177 | Polyketide:Modular type I polyketide+Polyketide:Trans-AT type I polyketide | 38.0 | 25.2 | 285.0 | 8.66e-77 |
| AAC38075.1 | polyketide\_synthase\_type\_I | BGC0000127 | Polyketide | 35.0 | 29.7 | 284.0 | 9.13e-77 |
| ALD83687.1 | tAT\_polyketide\_synthase | BGC0001300 | Polyketide | 35.0 | 32.9 | 285.0 | 1.03e-76 |
| AAM12913.2 | MmpD | BGC0000182 | Polyketide:Iterative type I polyketide+Polyketide:Trans-AT type I polyketide | 37.0 | 24.9 | 285.0 | 1.16e-76 |
| AUS29490.1 | polyketide\_synthase | BGC0002606 | NRP+Polyketide | 29.0 | 51.4 | 283.0 | 1.62e-76 |
| DAC80097.1 | PKS\_(DH-FkbM-FkbH-ACP-KS-ECH-ECH-ACP-ACP-KS-KR-ACP-KS) | BGC0001837 | Polyketide | 33.0 | 30.3 | 284.0 | 1.86e-76 |
| BAP05595.1 | calG | BGC0000967 | NRP+Polyketide:Trans-AT type I polyketide | 37.0 | 25.2 | 284.0 | 1.94e-76 |
| AXA20092.1 | trans-AT\_PKS\_LgaC | BGC0001646 | NRP+Polyketide | 34.0 | 32.5 | 283.0 | 2.65e-76 |
| RAT98530.1 | trans-acyltransferase\_polyketide\_synthase | BGC0001470 | Polyketide:Trans-AT type I polyketide | 34.0 | 30.3 | 281.0 | 2.86e-76 |
| ASA76642.1 | polyketide\_synthase | BGC0001751 | NRP+Polyketide | 37.0 | 28.2 | 283.0 | 2.94e-76 |
| AFN27483.1 | pks\_BonD | BGC0000173 | Polyketide:Modular type I polyketide | 35.0 | 30.0 | 283.0 | 2.96e-76 |
| CBK62731.1 |  | BGC0001115 | NRP+Polyketide | 34.0 | 29.8 | 283.0 | 3.03e-76 |
| DAC80063.1 | PKS | BGC0001836 | Polyketide:Trans-AT type I polyketide | 34.0 | 31.4 | 283.0 | 3.08e-76 |
| AWO77084.1 | hybrid\_non-ribosomal\_peptide\_synthetase/type\_I\_polyketide\_synthase | BGC0001556 | NRP+Polyketide | 39.0 | 25.1 | 283.0 | 3.48e-76 |
| SKB24634.1 |  | BGC0002455 | Polyketide | 37.0 | 27.8 | 283.0 | 3.74e-76 |
| AZF85947.1 | type\_I\_polyketide\_synthase | BGC0001963 | NRP+Polyketide | 38.0 | 25.2 | 282.0 | 5.93e-76 |
| ALD83704.1 | tAT\_polyketide\_synthase | BGC0001299 | Polyketide | 38.0 | 25.8 | 281.0 | 1.13e-75 |
| CCC21123.1 | type-I\_polyketide\_synthases | BGC0000171 | Polyketide:Modular type I polyketide | 34.0 | 31.5 | 281.0 | 1.34e-75 |
| BAF50727.1 | hybrid\_polyketide\_synthase-non\_ribosomal\_peptide\_synthetase | BGC0001116 | NRP+Polyketide | 35.0 | 29.7 | 281.0 | 1.37e-75 |
| AFX60309.1 | polyketide\_synthase | BGC0001031 | NRP+Polyketide | 33.0 | 30.1 | 281.0 | 1.43e-75 |
| QDG75024.1 | trans-\_AT\_polyketide\_synthase\_type\_I | BGC0002068 | NRP+Polyketide | 35.0 | 29.2 | 281.0 | 1.58e-75 |
| CAJ57409.1 | polyketide\_synthase\_type\_I | BGC0000176 | Polyketide | 32.0 | 29.7 | 281.0 | 1.66e-75 |
| ABC34832.1 | polyketide\_synthase | BGC0000186 | NRP+Polyketide:Modular type I polyketide | 37.0 | 25.9 | 281.0 | 1.7e-75 |
| WP\_126241644.1 | SDR\_family\_oxidoreductase | BGC0002087 | NRP+Polyketide | 36.0 | 25.4 | 280.0 | 1.76e-75 |
| QGZ36672.1 | amino\_acid\_adenylation\_domain-containing\_protein | BGC0002082 | NRP+Polyketide | 38.0 | 25.9 | 281.0 | 1.78e-75 |
| ADN68483.1 | sorH | BGC0000184 | Polyketide:Trans-AT type I polyketide | 34.0 | 30.4 | 280.0 | 1.94e-75 |
| CTQ34882.1 | AtcE;\_polyketide\_synthase,\_modules\_5-7 | BGC0001301 | Polyketide | 38.0 | 25.4 | 280.0 | 2.06e-75 |
| AGN74892.1 | nonribosomal\_peptide\_synthetase/polyketide\_synthase\_hybrid\_protein | BGC0000459 | NRP:Cyclic depsipeptide+Polyketide:Trans-AT type I polyketide | 36.0 | 31.3 | 280.0 | 2.08e-75 |
| ABS90475.1 | PKS | BGC0001106 | NRP+Polyketide | 36.0 | 26.0 | 280.0 | 2.15e-75 |
| bin5\_1\_edit\_las\_12 |  | BGC0002153 | Polyketide | 38.0 | 25.2 | 280.0 | 2.17e-75 |
| AJQ95705.1 | polyketide\_synthase\_modules-related\_protein | BGC0001644 | Polyketide | 34.0 | 31.3 | 280.0 | 2.29e-75 |
| KAF1058475.1 | Polyketide\_synthase\_PksL | BGC0002083 | Polyketide:Trans-AT type I polyketide | 36.0 | 25.4 | 280.0 | 2.31e-75 |
| ADA82581.1 | trans-AT\_polyketide\_synthase | BGC0001110 | NRP+Polyketide:Trans-AT type I polyketide | 35.0 | 29.6 | 280.0 | 2.33e-75 |
| AAN85523.1 | polyketide\_synthase | BGC0001101 | NRP+Polyketide:Modular type I polyketide+Polyketide:Trans-AT type I polyketide | 35.0 | 31.0 | 280.0 | 2.38e-75 |
| EAU29808.1 | hypothetical\_protein | BGC0001400 | Polyketide | 28.0 | 54.4 | 280.0 | 2.4e-75 |
| ADI59534.1 | CorL | BGC0001091 | NRP+Polyketide | 37.0 | 27.4 | 280.0 | 3.7e-75 |
| AQW44870.1 | polyketide\_synthase | BGC0001761 | Polyketide | 35.0 | 28.7 | 279.0 | 4.02e-75 |
| mycH | polyketide\_synthase | BGC0002055 | NRP+Polyketide:Trans-AT type I polyketide | 34.0 | 28.0 | 280.0 | 4.05e-75 |
| ABP57747.1 | DepC | BGC0000993 | NRP:Cyclic depsipeptide+Polyketide:Modular type I polyketide | 35.0 | 31.0 | 275.0 | 4.3e-75 |
| AEH42487.1 | polyketide\_synthase | BGC0000032 | Polyketide | 28.0 | 53.7 | 278.0 | 4.41e-75 |
| ABF92489.1 | mixed\_type\_I\_polyketide\_synthase\_-\_peptide\_synthetase | BGC0001025 | NRP+Polyketide:Trans-AT type I polyketide | 33.0 | 29.8 | 279.0 | 4.91e-75 |
| CAN89635.1 | putative\_polyketide\_synthase | BGC0001070 | NRP+Polyketide:Modular type I polyketide+Polyketide:Trans-AT type I polyketide | 34.0 | 28.6 | 278.0 | 5.07e-75 |
| ABF85931.1 | non-ribosomal\_peptide\_synthase/polyketide\_synthase\_Ta1 | BGC0001025 | NRP+Polyketide:Trans-AT type I polyketide | 34.0 | 27.9 | 279.0 | 5.59e-75 |
| AKQ22681.1 | malonyl\_CoA-acyl\_carrier\_protein\_transacylase | BGC0001656 | Polyketide | 33.0 | 30.5 | 278.0 | 8.4e-75 |
| CAG23960.2 | hybrid\_NRPS/PKS\_protein | BGC0001089 | Polyketide+NRP | 34.0 | 30.1 | 278.0 | 8.61e-75 |
| AGP37410.1 | peptide\_synthetase | BGC0002386 | NRP+Polyketide | 32.0 | 38.7 | 278.0 | 9.74e-75 |
| ABC36687.1 | polyketide\_synthase | BGC0000964 | NRP:Cyclic depsipeptide+Polyketide:Trans-AT type I polyketide | 35.0 | 30.0 | 273.0 | 1.36e-74 |
| QIE07365.1 | polyketide\_synthase\_NecF | BGC0002050 | NRP+Polyketide:Trans-AT type I polyketide | 36.0 | 25.1 | 278.0 | 1.54e-74 |
| AFN27482.1 | pks\_BonC | BGC0000173 | Polyketide:Modular type I polyketide | 35.0 | 30.6 | 276.0 | 1.55e-74 |
| OEI73463.1 | hypothetical\_protein | BGC0001520 | Polyketide | 33.0 | 30.0 | 277.0 | 1.9e-74 |
| DAC76734.1 | type\_I\_polyketide\_synthase/non-ribosomal\_peptide\_synthetase | BGC0001885 | NRP+Polyketide | 33.0 | 30.3 | 277.0 | 2.15e-74 |
| bin5\_1\_edit\_las\_15 |  | BGC0002153 | Polyketide | 34.0 | 30.4 | 277.0 | 2.92e-74 |
| ATX68111.1 | malonyl\_CoA-acyl\_carrier\_protein\_transacylase | BGC0001772 | Polyketide | 33.0 | 30.5 | 276.0 | 3.28e-74 |
| AUD08663.1 | iPKS-NRPS | BGC0001553 | NRP+Polyketide | 38.0 | 25.4 | 276.0 | 3.55e-74 |
| AFN27480.1 | pks\_BonA | BGC0000173 | Polyketide:Modular type I polyketide | 37.0 | 25.6 | 276.0 | 3.67e-74 |
| BAD38875.1 | polyketide\_synthase | BGC0000111 | Polyketide | 35.0 | 30.0 | 266.0 | 4.29e-74 |
| AEC04357.1 | polyketide\_synthase | BGC0000178 | Polyketide:Trans-AT type I polyketide | 34.0 | 31.3 | 276.0 | 4.49e-74 |
| ALD83702.1 | tAT\_polyketide\_synthase | BGC0001299 | Polyketide | 36.0 | 26.8 | 276.0 | 4.58e-74 |
| CAE52339.1 | Polyketide\_non-ribosomal\_peptide\_synthase | BGC0001088 | NRP+Polyketide | 35.0 | 29.7 | 276.0 | 4.68e-74 |
| DAC80102.1 | PKS\_(ACP-KS-KR-ACP-KS-ACP-ACP-ACP-KS-ACP-C) | BGC0001837 | Polyketide | 34.0 | 30.7 | 276.0 | 5.34e-74 |
| ABI91465.1 | beta-ketoacyl\_synthase | BGC0001094 | NRP+Polyketide | 35.0 | 25.1 | 275.0 | 5.74e-74 |
| ADD82939.1 | Bat1 | BGC0001099 | NRP+Polyketide:Modular type I polyketide+Polyketide:Trans-AT type I polyketide | 34.0 | 30.9 | 275.0 | 5.78e-74 |
| AJQ95706.1 | polyketide\_synthase\_modules-related\_protein | BGC0001644 | Polyketide | 32.0 | 32.5 | 276.0 | 6.4e-74 |
| BAP05591.1 | calC | BGC0000967 | NRP+Polyketide:Trans-AT type I polyketide | 35.0 | 30.0 | 275.0 | 7.2e-74 |
| AXA20091.1 | hybrid\_trans-AT\_PKS/NRPS\_LgaB | BGC0001646 | NRP+Polyketide | 36.0 | 25.8 | 275.0 | 7.93e-74 |
| AAM12909.2 | MmpA | BGC0000182 | Polyketide:Iterative type I polyketide+Polyketide:Trans-AT type I polyketide | 34.0 | 30.6 | 275.0 | 9.58e-74 |
| WP\_003598535.1 | SDR\_family\_NAD(P)-dependent\_oxidoreductase | BGC0001991 | Polyketide | 34.0 | 30.5 | 274.0 | 1.02e-73 |
| ADN68479.1 | SorD | BGC0000184 | Polyketide:Trans-AT type I polyketide | 34.0 | 29.3 | 275.0 | 1.02e-73 |
| AUS29495.1 | polyketide\_synthase | BGC0001030 | NRP+Polyketide | 27.0 | 54.6 | 274.0 | 1.14e-73 |
| ADD82941.1 | Bat3 | BGC0001099 | NRP+Polyketide:Modular type I polyketide+Polyketide:Trans-AT type I polyketide | 34.0 | 30.1 | 275.0 | 1.29e-73 |
| AAY39343.1 | Beta-ketoacyl\_synthase:Beta-ketoacyl\_synthase:Phosphopantetheine-binding\_protein | BGC0002060 | Polyketide:Trans-AT type I polyketide | 35.0 | 25.4 | 274.0 | 1.41e-73 |
| BAV56006.1 | PKS\_(ACP-KS-AT-DH-ER-KR-ACP-KS-AT-KR-ACP) | BGC0001597 | Polyketide | 36.0 | 25.5 | 274.0 | 1.52e-73 |
| AJY78091.1 | polyketide\_synthase | BGC0001902 | NRP+Polyketide | 37.0 | 25.3 | 273.0 | 1.55e-73 |
| AGN74894.1 | nonribosomal\_peptide\_synthetase/polyketide\_synthase\_hybrid\_protein | BGC0000459 | NRP:Cyclic depsipeptide+Polyketide:Trans-AT type I polyketide | 35.0 | 30.1 | 273.0 | 1.65e-73 |
| ADN68480.1 | SorE | BGC0000184 | Polyketide:Trans-AT type I polyketide | 34.0 | 30.5 | 274.0 | 1.69e-73 |
| DAC80073.1 | PKS | BGC0001835 | Polyketide | 33.0 | 30.0 | 274.0 | 1.82e-73 |
| ATX68112.1 | malonyl\_CoA-acyl\_carrier\_protein\_transacylase | BGC0001772 | Polyketide | 34.0 | 30.4 | 274.0 | 2.19e-73 |
| CAL69890.1 | RhiC\_protein | BGC0001112 | NRP+Polyketide:Trans-AT type I polyketide | 35.0 | 29.3 | 273.0 | 2.92e-73 |
| AFX60311.1 | polyketide\_synthase | BGC0001031 | NRP+Polyketide | 33.0 | 32.3 | 273.0 | 3.17e-73 |
| RAT98525.1 | trans-acyltransferase\_polyketide\_synthase | BGC0001470 | Polyketide:Trans-AT type I polyketide | 33.0 | 30.3 | 273.0 | 3.64e-73 |
| AVR48533.1 | CusA | BGC0001564 | NRP+Polyketide | 34.0 | 30.3 | 273.0 | 3.79e-73 |
| ADR01054.1 | Noc-11 | BGC0000609 | RiPP:Thiopeptide | 36.0 | 24.5 | 252.0 | 5.77e-73 |
| AEC04363.1 | polyketide\_synthase | BGC0000178 | Polyketide:Trans-AT type I polyketide | 33.0 | 31.0 | 272.0 | 6.24e-73 |
| CCG06113.1 | type\_I\_polyketide\_synthase | BGC0001543 | Polyketide | 34.0 | 29.0 | 271.0 | 7.03e-73 |
| ALD83688.1 | tAT\_polyketide\_synthase | BGC0001300 | Polyketide | 37.0 | 25.9 | 272.0 | 7.7e-73 |
| ACY01392.1 | AT-less\_polyketide\_synthase | BGC0000177 | Polyketide:Modular type I polyketide+Polyketide:Trans-AT type I polyketide | 36.0 | 25.9 | 271.0 | 9.6e-73 |
| AJY78093.1 | polyketide\_synthase | BGC0001902 | NRP+Polyketide | 32.0 | 38.8 | 270.0 | 9.86e-73 |
| AJQ95707.1 | polyketide\_synthase\_modules-related\_protein | BGC0001644 | Polyketide | 33.0 | 29.6 | 271.0 | 1.05e-72 |
| QZA73308.1 | type\_I\_polyketide\_synthase | BGC0002385 | Polyketide | 34.0 | 28.9 | 271.0 | 1.15e-72 |
| simG |  | BGC0000334 | NRP | 27.0 | 60.5 | 271.0 | 1.32e-72 |
| AAY89052.1 | polyketide\_synthase | BGC0001069 | NRP+Polyketide:Trans-AT type I polyketide | 36.0 | 27.6 | 271.0 | 1.46e-72 |
| SKB24638.1 |  | BGC0002455 | Polyketide | 34.0 | 30.0 | 271.0 | 1.54e-72 |
| QGZ36670.1 | SDR\_family\_NAD(P)-dependent\_oxidoreductase | BGC0002082 | NRP+Polyketide | 36.0 | 27.6 | 271.0 | 1.6e-72 |
| AGN74893.1 | nonribosomal\_peptide\_synthetase/polyketide\_synthase\_hybrid\_protein | BGC0000459 | NRP:Cyclic depsipeptide+Polyketide:Trans-AT type I polyketide | 34.0 | 31.2 | 270.0 | 2.02e-72 |
| CAG23959.2 | polyketide\_synthase\_of\_type\_I | BGC0001089 | Polyketide+NRP | 34.0 | 29.1 | 270.0 | 2.19e-72 |
| CAG23958.2 | polyketide\_synthase\_of\_type\_I | BGC0001089 | Polyketide+NRP | 33.0 | 30.0 | 270.0 | 2.44e-72 |
| BAP05594.1 | calF | BGC0000967 | NRP+Polyketide:Trans-AT type I polyketide | 38.0 | 25.2 | 270.0 | 2.66e-72 |
| BAP05589.1 | calA | BGC0000967 | NRP+Polyketide:Trans-AT type I polyketide | 33.0 | 30.2 | 270.0 | 2.99e-72 |
| ABM63529.1 | BryX | BGC0000174 | Polyketide | 34.0 | 31.0 | 270.0 | 3.18e-72 |
| AKQ22680.1 | malonyl\_CoA-acyl\_carrier\_protein\_transacylase | BGC0001656 | Polyketide | 34.0 | 30.4 | 270.0 | 3.3e-72 |
| ASA76644.1 | polyketide\_synthase | BGC0001751 | NRP+Polyketide | 36.0 | 25.1 | 269.0 | 3.7e-72 |
| AEP40938.1 | polyketide\_synthase\_type\_I | BGC0000021 | Polyketide | 35.0 | 25.1 | 249.0 | 4.76e-72 |
| mycF | polyketide\_synthase | BGC0002055 | NRP+Polyketide:Trans-AT type I polyketide | 36.0 | 25.3 | 270.0 | 5.01e-72 |
| AAY89053.1 | polyketide\_synthase | BGC0001069 | NRP+Polyketide:Trans-AT type I polyketide | 37.0 | 25.6 | 269.0 | 6e-72 |
| AFX60336.1 | polyketide\_synthase | BGC0001032 | NRP+Polyketide | 34.0 | 27.6 | 269.0 | 6.18e-72 |
| ATY69589.1 | type\_I\_polyketide\_synthase | BGC0001823 | NRP+Polyketide | 35.0 | 30.2 | 269.0 | 6.34e-72 |
| SKB24635.1 |  | BGC0002455 | Polyketide | 33.0 | 29.7 | 269.0 | 6.6e-72 |
| BBA21074.1 | putative\_modular\_polyketide\_synthase | BGC0001740 | NRP+Polyketide | 33.0 | 30.0 | 268.0 | 9.86e-72 |
| ATX68127.1 | malonyl\_CoA-acyl\_carrier\_protein\_transacylase | BGC0001795 | Polyketide | 35.0 | 29.9 | 268.0 | 1.11e-71 |
| QLG04867.1 | PulF | BGC0002374 | Polyketide | 33.0 | 30.8 | 268.0 | 1.26e-71 |
| QLG04868.1 | PulG | BGC0002374 | Polyketide | 33.0 | 30.8 | 268.0 | 1.36e-71 |
| pelC | polyketide\_synthase | BGC0002056 | NRP+Polyketide:Trans-AT type I polyketide | 33.0 | 28.9 | 268.0 | 1.45e-71 |
| AXA20096.1 | trans-AT\_PKS\_LgaG | BGC0001646 | NRP+Polyketide | 32.0 | 33.6 | 268.0 | 1.48e-71 |
| ADN68482.1 | sorG | BGC0000184 | Polyketide:Trans-AT type I polyketide | 39.0 | 25.0 | 267.0 | 1.62e-71 |
| ADD82940.1 | Bat2 | BGC0001099 | NRP+Polyketide:Modular type I polyketide+Polyketide:Trans-AT type I polyketide | 33.0 | 31.3 | 268.0 | 1.9e-71 |
| CAG23978.1 | polyketide\_synthase\_type\_I | BGC0000176 | Polyketide | 32.0 | 30.4 | 266.0 | 3.72e-71 |
| RAT98517.1 | trans-acyltransferase\_polyketide\_synthase | BGC0001470 | Polyketide:Trans-AT type I polyketide | 32.0 | 32.9 | 266.0 | 3.78e-71 |
| AJQ95676.1 | polyketide\_synthase\_modules-related\_protein | BGC0002046 | NRP+Polyketide:Trans-AT type I polyketide | 37.0 | 25.3 | 266.0 | 3.97e-71 |
| RAT98526.1 | trans-acyltransferase\_polyketide\_synthase | BGC0001470 | Polyketide:Trans-AT type I polyketide | 32.0 | 30.6 | 265.0 | 3.99e-71 |
| CAL69891.1 | RhiD\_protein | BGC0001112 | NRP+Polyketide:Trans-AT type I polyketide | 33.0 | 28.9 | 266.0 | 4.72e-71 |
| CAN89633.1 | putative\_hybrid\_non-ribosomal\_peptide\_synthetase/polyketide\_synthase | BGC0001070 | NRP+Polyketide:Modular type I polyketide+Polyketide:Trans-AT type I polyketide | 38.0 | 25.3 | 264.0 | 5.91e-71 |
| ctg3\_18 |  | BGC0001853 | NRP+Polyketide:Modular type I polyketide | 35.0 | 29.9 | 266.0 | 6.26e-71 |
| EHK80169.1 | acyl\_transferase | BGC0001447 | Polyketide | 38.0 | 24.8 | 266.0 | 7.06e-71 |
| CAL69894.1 | RhiF\_protein | BGC0001112 | NRP+Polyketide:Trans-AT type I polyketide | 31.0 | 35.5 | 265.0 | 1.07e-70 |
| ABM63528.1 | BryC | BGC0000174 | Polyketide | 35.0 | 25.4 | 265.0 | 1.16e-70 |
| AAS47562.1 | mixed\_type\_I\_polyketide\_synthase\_-\_peptide\_synthetase | BGC0001108 | NRP+Polyketide:Trans-AT type I polyketide | 35.0 | 25.7 | 265.0 | 1.21e-70 |
| ctg1\_orf8 |  | BGC0001109 | NRP+Polyketide | 35.0 | 25.7 | 265.0 | 1.21e-70 |
| ATY69569.1 | type\_I\_polyketide\_synthase | BGC0001611 | NRP+Polyketide | 37.0 | 25.8 | 265.0 | 1.25e-70 |
| ABC34176.1 | polyketide\_synthase,\_putative | BGC0000186 | NRP+Polyketide:Modular type I polyketide | 36.0 | 24.9 | 264.0 | 1.26e-70 |
| pelD | polyketide\_synthase | BGC0002056 | NRP+Polyketide:Trans-AT type I polyketide | 32.0 | 30.6 | 265.0 | 1.28e-70 |
| CAJ57411.1 | polyketide\_synthase\_type\_I | BGC0000176 | Polyketide | 34.0 | 30.4 | 264.0 | 1.41e-70 |
| ABC34675.1 | polyketide\_synthase,\_putative | BGC0000186 | NRP+Polyketide:Modular type I polyketide | 33.0 | 30.5 | 265.0 | 1.54e-70 |
| QZA73306.1 | type\_I\_polyketide\_synthase | BGC0002385 | Polyketide | 34.0 | 30.5 | 264.0 | 1.9e-70 |
| ATV82110.1 | PKS | BGC0001909 | Polyketide | 27.0 | 57.7 | 264.0 | 1.95e-70 |
| QGY73454.1 | Itm22 | BGC0002451 | Polyketide | 34.0 | 31.2 | 264.0 | 1.99e-70 |
| ALD83686.1 | tAT\_polyketide\_synthase | BGC0001300 | Polyketide | 35.0 | 30.4 | 264.0 | 2.09e-70 |
| CAG23957.2 | hybrid\_NRPS/PKS\_protein | BGC0001089 | Polyketide+NRP | 32.0 | 30.3 | 264.0 | 2.57e-70 |
| BBA21068.1 | putative\_non-ribosomal\_peptide\_synthetase | BGC0001740 | NRP+Polyketide | 34.0 | 30.1 | 264.0 | 2.7e-70 |
| AAS47564.1 | mixed\_type\_I\_polyketide\_synthase/nonribosomal\_peptide\_synthetase | BGC0001108 | NRP+Polyketide:Trans-AT type I polyketide | 34.0 | 31.3 | 264.0 | 2.91e-70 |
| ctg1\_orf6 |  | BGC0001109 | NRP+Polyketide | 34.0 | 31.3 | 264.0 | 2.91e-70 |
| ADA69239.2 | trans-AT\_hybrid\_polyketide\_synthase-NRPS | BGC0001071 | NRP+Polyketide:Modular type I polyketide+Polyketide:Trans-AT type I polyketide | 34.0 | 31.5 | 263.0 | 3.79e-70 |
| CAN89634.1 | putative\_polyketide\_synthase | BGC0001070 | NRP+Polyketide:Modular type I polyketide+Polyketide:Trans-AT type I polyketide | 38.0 | 25.5 | 263.0 | 4.72e-70 |
| QDG75035.1 | mixed\_type\_I\_polyketide\_synthase\_-\_peptide\_synthetase | BGC0002068 | NRP+Polyketide | 33.0 | 30.5 | 263.0 | 4.96e-70 |
| ABS90478.1 | PKS | BGC0001106 | NRP+Polyketide | 31.0 | 36.2 | 254.0 | 5.01e-70 |
| ATX68125.1 | malonyl\_CoA-acyl\_carrier\_protein\_transacylase | BGC0001795 | Polyketide | 34.0 | 28.9 | 263.0 | 5.05e-70 |
| AAV97870.1 | OnnB | BGC0001105 | NRP+Polyketide:Trans-AT type I polyketide | 33.0 | 30.3 | 263.0 | 5.54e-70 |
| AGJ76601.1 | HglE | BGC0000869 | Other | 26.0 | 59.5 | 261.0 | 5.97e-70 |
| BBA84070.1 | type\_I\_polyketide\_synthase | BGC0001649 | Polyketide | 37.0 | 25.2 | 263.0 | 6.01e-70 |
| CTQ34881.1 | AtcD;\_polyketide\_synthase,\_modules\_1-4 | BGC0001301 | Polyketide | 33.0 | 32.0 | 263.0 | 6.23e-70 |
| DAC80074.1 | PKS | BGC0001835 | Polyketide | 34.0 | 29.7 | 262.0 | 7.82e-70 |
| BAE93740.1 | type\_I\_polyketide\_synthase-related\_protein | BGC0000164 | Polyketide | 37.0 | 27.3 | 261.0 | 9e-70 |
| AMH40421.1 | PKS | BGC0001350 | Polyketide | 34.0 | 30.4 | 262.0 | 1.09e-69 |
| AIJ04684.1 | polyketide\_synthase | BGC0001383 | Polyketide | 35.0 | 25.0 | 261.0 | 1.11e-69 |
| AAM12911.1 | MmpB | BGC0000182 | Polyketide:Iterative type I polyketide+Polyketide:Trans-AT type I polyketide | 36.0 | 25.2 | 261.0 | 1.27e-69 |
| CCG06109.1 | type\_I\_polyketide\_synthase | BGC0001543 | Polyketide | 33.0 | 29.8 | 261.0 | 1.47e-69 |
| CAG23967.1 | polyketide\_synthase\_type\_I | BGC0000181 | Polyketide | 35.0 | 25.0 | 260.0 | 1.92e-69 |
| CAN93351.1 | polyketide\_synthase | BGC0000179 | Polyketide:Trans-AT type I polyketide | 34.0 | 30.1 | 261.0 | 1.98e-69 |
| BBA21069.1 | putative\_modular\_polyketide\_synthase | BGC0001740 | NRP+Polyketide | 30.0 | 37.6 | 253.0 | 2.04e-69 |
| DAD54487.1 | trans-acyltransferase\_polyketide\_synthase | BGC0002059 | NRP+Polyketide:Trans-AT type I polyketide | 37.0 | 25.4 | 261.0 | 2.23e-69 |
| CAN93347.1 | Polyketide\_synthase | BGC0000179 | Polyketide:Trans-AT type I polyketide | 36.0 | 25.9 | 261.0 | 2.27e-69 |
| QDG75033.1 | mixed\_type\_I\_polyketide\_synthase\_-\_peptide\_synthetase | BGC0002068 | NRP+Polyketide | 33.0 | 30.6 | 260.0 | 3.1e-69 |
| ABC35027.1 | JamP | BGC0000961 | NRP+Polyketide | 33.0 | 32.0 | 256.0 | 9.05e-69 |
| DAC80098.1 | PKS\_(MT-ACP-KS-KR-ACP-KS-KR-MT-ACP-KS-KR-ACP-KS-ACP-ACP-KS-MT-\_KR-ACP) | BGC0001837 | Polyketide | 34.0 | 30.3 | 259.0 | 9.56e-69 |
| ADI59532.1 | CorJ | BGC0001091 | NRP+Polyketide | 34.0 | 32.0 | 258.0 | 1.04e-68 |
| ACY01401.1 | AT-less\_polyketide\_synthase | BGC0000083 | Polyketide:Modular type I polyketide+Polyketide:Trans-AT type I polyketide | 34.0 | 25.7 | 258.0 | 1.29e-68 |
| QCC63000.1 | BII-rafflesfungin\_polyketide\_synthase | BGC0001966 | NRP+Polyketide | 38.0 | 26.1 | 258.0 | 1.43e-68 |
| QRN75753.1 | Polyketide\_synthase | BGC0002114 | NRP+Polyketide | 31.0 | 38.0 | 257.0 | 2.28e-68 |
| QGY73445.1 | Itm13 | BGC0002451 | Polyketide | 35.0 | 26.5 | 257.0 | 3.57e-68 |
| AIC32694.1 | FR9GH | BGC0001113 | NRP+Polyketide | 36.0 | 25.7 | 256.0 | 4.44e-68 |
| AVR48535.1 | CusC | BGC0001564 | NRP+Polyketide | 33.0 | 29.2 | 256.0 | 5.1e-68 |
| QCL09089.1 | dmxL2 | BGC0002063 | Polyketide:Iterative type I polyketide | 38.0 | 22.1 | 256.0 | 5.17e-68 |
| BAQ25481.1 | type\_I\_polyketide\_synthase | BGC0001288 | Polyketide | 37.0 | 25.5 | 256.0 | 6.39e-68 |
| CTQ34883.1 | AtcF;\_polyketide\_synthase,\_modules\_8-10 | BGC0001301 | Polyketide | 35.0 | 27.6 | 256.0 | 6.99e-68 |
| ASA76633.1 | polyketide\_synthase | BGC0001751 | NRP+Polyketide | 33.0 | 30.3 | 253.0 | 7.09e-68 |
| ACR13997.1 | modular\_polyketide\_synthase,\_type\_I\_PKS | BGC0000185 | Polyketide | 34.0 | 25.1 | 256.0 | 7.43e-68 |
| bin5\_1\_edit\_las\_08 |  | BGC0002153 | Polyketide | 33.0 | 31.0 | 255.0 | 8.18e-68 |
| QBG82526.1 | Polyketide\_synthase | BGC0002587 | Polyketide | 36.0 | 25.0 | 255.0 | 8.34e-68 |
| ABS90470.1 | NRPS/PKS | BGC0001106 | NRP+Polyketide | 34.0 | 25.0 | 256.0 | 8.49e-68 |
| AJQ95675.1 | polyketide\_synthase\_modules-related\_protein | BGC0002046 | NRP+Polyketide:Trans-AT type I polyketide | 35.0 | 29.2 | 255.0 | 8.93e-68 |
| DAC80061.1 | PKS | BGC0001836 | Polyketide:Trans-AT type I polyketide | 32.0 | 30.3 | 255.0 | 9.64e-68 |
| ABM63527.1 | BryB | BGC0000174 | Polyketide | 32.0 | 29.6 | 255.0 | 1.03e-67 |
| bin5\_1\_edit\_las\_13 |  | BGC0002153 | Polyketide | 36.0 | 24.9 | 254.0 | 1.91e-67 |
| XP\_001220460.1 | uncharacterized\_protein | BGC0001182 | NRP+Polyketide:Iterative type I polyketide | 26.0 | 62.9 | 254.0 | 2.23e-67 |
| bin5\_1\_edit\_las\_14 |  | BGC0002153 | Polyketide | 33.0 | 30.2 | 252.0 | 2.32e-67 |
| CAL69893.1 | RhiE\_protein | BGC0001112 | NRP+Polyketide:Trans-AT type I polyketide | 32.0 | 29.7 | 254.0 | 2.78e-67 |
| AKQ22696.1 | malonyl\_CoA-acyl\_carrier\_protein\_transacylase | BGC0001186 | Polyketide | 35.0 | 25.4 | 254.0 | 3.31e-67 |
| AKQ22698.1 | malonyl\_CoA-acyl\_carrier\_protein\_transacylase | BGC0001186 | Polyketide | 34.0 | 28.6 | 254.0 | 3.37e-67 |
| CCA89326.1 | mixed\_trans-AT\_type\_I\_polyketide\_synthase/nonribosomal\_peptide\_synthetase | BGC0001111 | NRP+Polyketide:Trans-AT type I polyketide | 34.0 | 31.2 | 254.0 | 3.5e-67 |
| ACR50796.1 | putative\_polyketide\_synthase | BGC0000163 | Polyketide | 36.0 | 25.7 | 253.0 | 3.56e-67 |
| QZA73305.1 | type\_I\_polyketide\_synthase | BGC0002385 | Polyketide | 32.0 | 30.0 | 253.0 | 4.32e-67 |
| ACY06292.1 | modular\_polyketide\_synthase | BGC0001042 | NRP+Polyketide | 28.0 | 52.0 | 251.0 | 6.37e-67 |
| AKQ22669.1 | malonyl\_CoA-acyl\_carrier\_protein\_transacylase | BGC0001656 | Polyketide | 32.0 | 30.4 | 252.0 | 9.12e-67 |
| ADH01490.1 | type\_I\_polyketide\_synthase | BGC0000995 | NRP+Polyketide | 33.0 | 29.4 | 252.0 | 9.36e-67 |
| AIC32695.1 | FR9I | BGC0001113 | NRP+Polyketide | 33.0 | 29.4 | 252.0 | 9.36e-67 |
| ATX68109.1 | malonyl\_CoA-acyl\_carrier\_protein\_transacylase | BGC0001772 | Polyketide | 32.0 | 29.9 | 252.0 | 9.77e-67 |
| ADA69237.1 | trans-AT\_polyketide\_synthase | BGC0001071 | NRP+Polyketide:Modular type I polyketide+Polyketide:Trans-AT type I polyketide | 32.0 | 30.9 | 252.0 | 1.17e-66 |
| BAC76474.1 | type\_I\_polyketide\_synthase\_LkcC | BGC0001100 | NRP+Polyketide | 32.0 | 30.4 | 251.0 | 1.31e-66 |
| AJQ95704.1 | polyketide\_synthase\_modules-related\_protein | BGC0001644 | Polyketide | 31.0 | 30.7 | 251.0 | 1.54e-66 |
| AQW44874.1 | polyketide\_synthase | BGC0001761 | Polyketide | 34.0 | 29.8 | 250.0 | 1.6e-66 |
| ATY69557.1 | type\_I\_polyketide\_synthase | BGC0001611 | NRP+Polyketide | 33.0 | 31.8 | 251.0 | 1.61e-66 |
| DAC80077.1 | PKS | BGC0001835 | Polyketide | 33.0 | 29.6 | 251.0 | 2.11e-66 |
| CAN93352.1 | polyketide\_synthase | BGC0000179 | Polyketide:Trans-AT type I polyketide | 32.0 | 30.3 | 251.0 | 2.73e-66 |
| RAT98518.1 | trans-acyltransferase\_polyketide\_synthase | BGC0001470 | Polyketide:Trans-AT type I polyketide | 34.0 | 25.8 | 250.0 | 2.96e-66 |
| BAC76471.1 | type\_I\_polyketide\_synthase\_LkcF | BGC0001100 | NRP+Polyketide | 34.0 | 28.9 | 250.0 | 2.99e-66 |
| AGN11882.1 | tstGH | BGC0001114 | NRP+Polyketide | 37.0 | 25.4 | 250.0 | 3.38e-66 |
| AJO72742.1 | Type\_I\_modular\_polyketide\_synthase | BGC0001381 | Polyketide | 35.0 | 26.7 | 250.0 | 3.98e-66 |
| CAN93349.1 | polyketide\_synthase | BGC0000179 | Polyketide:Trans-AT type I polyketide | 32.0 | 31.4 | 249.0 | 5.22e-66 |
| CAJ57408.1 | polyketide\_synthase\_type\_I | BGC0000176 | Polyketide | 34.0 | 28.2 | 248.0 | 7.03e-66 |
| AKQ22697.1 | malonyl\_CoA-acyl\_carrier\_protein\_transacylase | BGC0001186 | Polyketide | 32.0 | 30.0 | 249.0 | 7.45e-66 |
| AGN11883.1 | tstI | BGC0001114 | NRP+Polyketide | 32.0 | 29.6 | 248.0 | 1.42e-65 |
| AKQ22682.1 | malonyl\_CoA-acyl\_carrier\_protein\_transacylase | BGC0001656 | Polyketide | 35.0 | 25.2 | 248.0 | 1.8e-65 |
| bin5\_1\_edit\_las\_10 |  | BGC0002153 | Polyketide | 33.0 | 31.0 | 245.0 | 1.92e-65 |
| ABS90472.1 | PKS | BGC0001106 | NRP+Polyketide | 32.0 | 29.6 | 244.0 | 2.47e-65 |
| ABM63537.1 | BryA | BGC0000174 | Polyketide | 33.0 | 29.0 | 247.0 | 2.96e-65 |
| ACR13065.1 | modular\_polyketide\_synthase,\_type\_I\_PKS | BGC0000185 | Polyketide | 35.0 | 25.9 | 246.0 | 5.02e-65 |
| ATX68110.1 | malonyl\_CoA-acyl\_carrier\_protein\_transacylase | BGC0001772 | Polyketide | 31.0 | 30.0 | 246.0 | 5.89e-65 |
| OEI73466.1 | hypothetical\_protein | BGC0001520 | Polyketide | 32.0 | 30.3 | 246.0 | 6.58e-65 |
| CAJ57410.1 | polyketide\_synthase\_type\_I | BGC0000176 | Polyketide | 32.0 | 29.2 | 245.0 | 1.11e-64 |
| ATX68126.1 | malonyl\_CoA-acyl\_carrier\_protein\_transacylase | BGC0001795 | Polyketide | 31.0 | 29.6 | 245.0 | 1.12e-64 |
| MCC5026026.1 | polyketide\_synthase\_dehydratase\_domain-containing\_protein | BGC0002118 | NRP+Polyketide | 34.0 | 25.7 | 245.0 | 1.13e-64 |
| MCC5025982.1 | polyketide\_synthase\_dehydratase\_domain-containing\_protein | BGC0002119 | NRP+Polyketide | 34.0 | 25.7 | 245.0 | 1.13e-64 |
| QZA73307.1 | type\_I\_polyketide\_synthase | BGC0002385 | Polyketide | 32.0 | 30.0 | 244.0 | 2.66e-64 |
| DAD54488.1 | trans-acyltransferase\_polyketide\_synthase | BGC0002059 | NRP+Polyketide:Trans-AT type I polyketide | 33.0 | 29.6 | 243.0 | 3.29e-64 |
| AAY89051.1 | hybrid\_nonribosomal\_peptide\_synthetase/polyketide\_synthase | BGC0001069 | NRP+Polyketide:Trans-AT type I polyketide | 34.0 | 25.0 | 244.0 | 3.34e-64 |
| OPB37944.1 | putative\_polyketide\_synthase | BGC0002206 | Polyketide | 36.0 | 25.7 | 243.0 | 3.36e-64 |
| AKQ22670.1 | malonyl\_CoA-acyl\_carrier\_protein\_transacylase | BGC0001656 | Polyketide | 32.0 | 29.2 | 243.0 | 4.34e-64 |
| QIE07364.1 | polyketide\_synthase\_NecE | BGC0002050 | NRP+Polyketide:Trans-AT type I polyketide | 35.0 | 25.3 | 243.0 | 4.59e-64 |
| AEC04362.1 | polyketide\_synthase | BGC0000178 | Polyketide:Trans-AT type I polyketide | 33.0 | 29.0 | 243.0 | 7.5e-64 |
| BAP05596.1 | calH | BGC0000967 | NRP+Polyketide:Trans-AT type I polyketide | 31.0 | 32.7 | 243.0 | 7.61e-64 |
| SKB24636.1 |  | BGC0002455 | Polyketide | 31.0 | 29.7 | 242.0 | 1.01e-63 |
| AAY32966.1 | DszC | BGC0001093 | NRP+Polyketide | 31.0 | 37.4 | 241.0 | 1.58e-63 |
| PHM26606.1 | malonyl\_CoA-acyl\_carrier\_protein\_transacylase | BGC0001130 | NRP+Polyketide | 25.0 | 59.8 | 240.0 | 3.02e-63 |
| ATY69600.1 | type\_I\_polyketide\_synthase | BGC0001823 | NRP+Polyketide | 33.0 | 25.2 | 240.0 | 3.22e-63 |
| SKB24637.1 |  | BGC0002455 | Polyketide | 33.0 | 29.8 | 240.0 | 3.49e-63 |
| KAF1058474.1 | Polyketide\_synthase\_PksN | BGC0002083 | Polyketide:Trans-AT type I polyketide | 34.0 | 25.9 | 240.0 | 5.85e-63 |
| CAL69888.1 | RhiA\_protein | BGC0001112 | NRP+Polyketide:Trans-AT type I polyketide | 34.0 | 28.8 | 238.0 | 1.8e-62 |
| QRN75756.1 | Short-chain\_dehydrogenase/reductase\_SDR | BGC0002114 | NRP+Polyketide | 32.0 | 33.4 | 236.0 | 1.97e-62 |
| BAP05590.1 | calB | BGC0000967 | NRP+Polyketide:Trans-AT type I polyketide | 31.0 | 29.3 | 238.0 | 2.12e-62 |
| AAN85522.1 | hybrid\_nonribosomal\_peptide\_synthetase\_/\_polyketide\_synthase | BGC0001101 | NRP+Polyketide:Modular type I polyketide+Polyketide:Trans-AT type I polyketide | 33.0 | 25.0 | 237.0 | 3.29e-62 |
| ELY54\_RS14405 | SDR\_family\_NAD(P)-dependent\_oxidoreductase | BGC0002087 | NRP+Polyketide | 33.0 | 25.9 | 237.0 | 3.71e-62 |
| AHI59107.1 | locillomycin\_synthase\_D | BGC0001005 | NRP+Polyketide | 32.0 | 29.1 | 234.0 | 5.1e-62 |
| CAD15512.1 | polyketide\_synthase | BGC0001014 | NRP:NRP siderophore+Polyketide:Modular type I polyketide+Polyketide:Iterative type I polyketide | 33.0 | 29.7 | 236.0 | 5.36e-62 |
| ABM63530.1 | BryD | BGC0000174 | Polyketide | 32.0 | 29.0 | 236.0 | 6.75e-62 |
| BAF50721.1 | hybrid\_non\_ribosomal\_peptide\_synthetase-polyketide\_synthase | BGC0001116 | NRP+Polyketide | 33.0 | 26.3 | 233.0 | 1.12e-61 |
| QJY30853.1 | PKS-NRPS\_hybrid\_protein | BGC0002539 | Alkaloid | 29.0 | 46.0 | 235.0 | 1.19e-61 |
| AFX60341.1 | polyketide\_synthase | BGC0001032 | NRP+Polyketide | 31.0 | 31.4 | 234.0 | 3.96e-61 |
| AJO72734.1 | Type\_I\_modular\_polyketide\_synthase | BGC0001381 | Polyketide | 35.0 | 25.3 | 233.0 | 6.36e-61 |
| WP\_055469551.1 | SDR\_family\_NAD(P)-dependent\_oxidoreductase | BGC0001537 | Polyketide | 33.0 | 29.3 | 230.0 | 8.75e-61 |
| AJQ95674.1 | polyketide\_synthase\_modules-related\_protein | BGC0002046 | NRP+Polyketide:Trans-AT type I polyketide | 34.0 | 24.8 | 229.0 | 1.07e-60 |
| CAG23961.2 | polyketide\_synthase\_of\_type\_I | BGC0001089 | Polyketide+NRP | 28.0 | 33.5 | 232.0 | 1.1e-60 |
| QIE07367.1 | polyketide\_synthase\_NecH | BGC0002050 | NRP+Polyketide:Trans-AT type I polyketide | 34.0 | 25.5 | 231.0 | 2.39e-60 |
| AFX60318.1 | polyketide\_synthase | BGC0001031 | NRP+Polyketide | 33.0 | 24.9 | 231.0 | 2.62e-60 |
| ADH01484.1 | putative\_type-I\_PKS | BGC0000995 | NRP+Polyketide | 31.0 | 27.3 | 231.0 | 3.4e-60 |
| AIC32692.1 | FR9C | BGC0001113 | NRP+Polyketide | 31.0 | 27.3 | 231.0 | 3.4e-60 |
| AIJ04685.1 | polyketide\_synthase | BGC0001383 | Polyketide | 33.0 | 24.7 | 228.0 | 1.64e-59 |
| CAG23969.1 | polyketide\_synthase\_type\_I | BGC0000181 | Polyketide | 32.0 | 24.7 | 227.0 | 3.7e-59 |
| AGN11880.1 | tstC | BGC0001114 | NRP+Polyketide | 29.0 | 31.1 | 226.0 | 6.63e-59 |
| XP\_011392698.1 | uncharacterized\_protein | BGC0001281 | Polyketide | 29.0 | 32.1 | 224.0 | 1.67e-58 |
| ATQ39432.1 | PKS | BGC0001565 | NRP | 34.0 | 25.2 | 224.0 | 2.54e-58 |
| BAL90255.1 | putative\_beta-ketoacyl\_synthase | BGC0002021 | Polyketide | 34.0 | 25.2 | 213.0 | 2.98e-58 |
| ABS90471.1 | PKS\_type\_I | BGC0001106 | NRP+Polyketide | 34.0 | 25.2 | 224.0 | 3.6e-58 |
| ABS75102.2 | MBL\_fold\_metallo-hydrolase | BGC0002641 | NRP | 30.0 | 30.6 | 218.0 | 7.07e-58 |
| AFR69335.1 | polyketide\_synthase\_SpiC2 | BGC0001045 | NRP:Cyclic depsipeptide+Polyketide:Modular type I polyketide | 30.0 | 30.6 | 222.0 | 7.15e-58 |
| DAD54485.1 | trans-acyltransferase\_polyketide\_synthase | BGC0002059 | NRP+Polyketide:Trans-AT type I polyketide | 31.0 | 30.1 | 223.0 | 7.56e-58 |
| ERM18797.1 | polyketide\_synthase | BGC0000172 | Polyketide | 33.0 | 24.9 | 223.0 | 8.76e-58 |
| ANC94964.1 | AlmHIII | BGC0001396 | Polyketide | 28.0 | 37.5 | 222.0 | 1.18e-57 |
| CBJ82077.1 | hypothetical\_protein | BGC0001872 | Polyketide | 25.0 | 58.1 | 221.0 | 2.67e-57 |
| CAN89631.1 | putative\_polyketide\_synthase | BGC0001070 | NRP+Polyketide:Modular type I polyketide+Polyketide:Trans-AT type I polyketide | 33.0 | 24.6 | 220.0 | 3.11e-57 |
| AAM12934.1 | MmpF | BGC0000182 | Polyketide:Iterative type I polyketide+Polyketide:Trans-AT type I polyketide | 27.0 | 37.7 | 213.0 | 7.28e-57 |
| AAM12925.2 | MmpE | BGC0000182 | Polyketide:Iterative type I polyketide+Polyketide:Trans-AT type I polyketide | 30.0 | 26.1 | 216.0 | 1.64e-56 |
| AEW98133.1 | putative\_type-I\_PKS | BGC0002642 | Alkaloid | 32.0 | 24.7 | 209.0 | 3.57e-56 |
| ADH01489.1 | type\_I\_polyketide\_synthase | BGC0000995 | NRP+Polyketide | 38.0 | 20.8 | 215.0 | 1.32e-55 |
| CCG06108.1 | type\_I\_polyketide\_synthase | BGC0001543 | Polyketide | 32.0 | 25.1 | 212.0 | 2.69e-55 |
| CAJ76285.1 | putative\_polyketide\_synthase | BGC0000972 | NRP+Polyketide:Modular type I polyketide+Polyketide:Trans-AT type I polyketide | 35.0 | 26.6 | 208.0 | 4.68e-55 |
| CBK62718.1 |  | BGC0001115 | NRP+Polyketide | 30.0 | 29.4 | 202.0 | 1.57e-53 |
| ABC38737.1 | polyketide\_synthase | BGC0000964 | NRP:Cyclic depsipeptide+Polyketide:Trans-AT type I polyketide | 29.0 | 30.2 | 206.0 | 5.37e-53 |
| CBJ89766.1 | Polyketide\_synthase\_involved\_in\_xenocoumacin\_synthesis | BGC0001054 | NRP+Polyketide:Modular type I polyketide | 33.0 | 25.6 | 206.0 | 9.8e-53 |
| ATY12792.1 | polyketide\_synthase | BGC0001504 | Polyketide | 30.0 | 24.9 | 194.0 | 1.77e-52 |
| ABP57746.1 | DepB | BGC0000993 | NRP:Cyclic depsipeptide+Polyketide:Modular type I polyketide | 29.0 | 34.1 | 204.0 | 2.73e-52 |
| AFR69332.1 | polyketide\_synthase\_SpiB | BGC0001045 | NRP:Cyclic depsipeptide+Polyketide:Modular type I polyketide | 29.0 | 30.1 | 203.0 | 3.62e-52 |
| DAC80529.1 | malonyl\_CoA-acyl\_carrier\_protein\_transacylase | BGC0001878 | NRP+Polyketide | 30.0 | 30.7 | 203.0 | 8.12e-52 |
| BAC76470.1 | type\_I\_polyketide\_synthase\_LkcG | BGC0001100 | NRP+Polyketide | 31.0 | 25.7 | 197.0 | 5.07e-51 |
| AGC65514.1 | TtcB | BGC0001876 | NRP | 29.0 | 32.0 | 199.0 | 1.42e-50 |
| KYC42614.1 | hypothetical\_protein | BGC0002484 | NRP+Polyketide | 30.0 | 28.3 | 197.0 | 1.51e-50 |
| QQZ01636.1 | PKS | BGC0002497 | Other | 25.0 | 48.7 | 196.0 | 8.15e-50 |
| OEI73460.1 | hypothetical\_protein | BGC0001520 | Polyketide | 33.0 | 22.5 | 196.0 | 9.06e-50 |
| ADN68484.1 | sorI | BGC0000184 | Polyketide:Trans-AT type I polyketide | 28.0 | 30.0 | 194.0 | 3.79e-49 |
| WP\_004571777.1 | non-ribosomal\_peptide\_synthetase | BGC0001760 | NRP | 31.0 | 28.9 | 191.0 | 3.56e-48 |
| DAC80540.1 | nrps | BGC0001840 | NRP+Polyketide | 32.0 | 25.4 | 191.0 | 3.58e-48 |
| AAO39107.1 | AdmM | BGC0000956 | NRP:Beta-lactam+Polyketide:Type II polyketide | 29.0 | 28.9 | 187.0 | 7.51e-48 |
| DAC80525.1 | malonyl\_CoA-acyl\_carrier\_protein\_transacylase | BGC0001841 | NRP+Polyketide | 30.0 | 30.3 | 188.0 | 2.25e-47 |
| QUS58936.1 | non-ribosomal\_peptide\_synthetase | BGC0002123 | NRP+Polyketide | 29.0 | 26.2 | 185.0 | 2.02e-46 |
| RLV64601.1 | polyketide\_synthase\_of\_type\_I | BGC0001845 | Polyketide+NRP+Other:Aminocoumarin | 32.0 | 25.9 | 184.0 | 3.69e-46 |
| ATY69551.1 | hybrid\_nonribosomal\_peptide\_synthetase/type\_I\_polyketide\_synthase | BGC0001611 | NRP+Polyketide | 31.0 | 25.0 | 182.0 | 1.19e-45 |
| AAM94794.1 | CalE8 | BGC0000033 | Polyketide | 25.0 | 56.0 | 178.0 | 2.06e-44 |
| QPB41099.1 | non-ribosomal\_peptide\_synthetase | BGC0002503 | NRP+Polyketide | 29.0 | 29.9 | 178.0 | 3.45e-44 |
| SCL57535.1 | enediyne\_polyketide\_synthase | BGC0002387 | Polyketide | 26.0 | 51.0 | 175.0 | 1.79e-43 |
| AAO39109.1 | AdmO | BGC0000956 | NRP:Beta-lactam+Polyketide:Type II polyketide | 29.0 | 25.8 | 170.0 | 2.08e-42 |
| MBA5221205.1 | polyketide\_synthase | BGC0002090 | NRP+Polyketide:Modular type I polyketide | 30.0 | 27.6 | 169.0 | 6.19e-42 |
| ctg1\_13 |  | BGC0001931 | Polyketide | 31.0 | 22.7 | 164.0 | 6.05e-40 |
| ABB69082.1 | putative\_pyrrolyl-deta-ketoacyl\_ACP\_synthase | BGC0000260 | Polyketide | 29.0 | 26.8 | 158.0 | 1.07e-38 |
| EWM63004.1 | polyketide\_synthase\_type\_I | BGC0001328 | NRP:Cyclic depsipeptide+Polyketide:Modular type I polyketide | 31.0 | 22.0 | 158.0 | 1.98e-38 |
| EHA53213.1 | D-alanine-poly(phosphoribitol)\_ligase\_subunit\_1 | BGC0002158 | NRP+Polyketide | 30.0 | 24.1 | 154.0 | 6.41e-37 |
| APZ78852.1 | polyketide\_synthase | BGC0001432 | NRP:Cyclic depsipeptide+Polyketide:Iterative type I polyketide | 26.0 | 27.2 | 147.0 | 7.08e-35 |
| CCE88382.1 | polyketide\_synthase | BGC0001034 | NRP+Polyketide:Modular type I polyketide | 31.0 | 21.3 | 145.0 | 7.34e-35 |
| AAB81125.1 | unknown | BGC0000862 | Other | 27.0 | 27.8 | 143.0 | 1.35e-33 |
| AAQ84156.1 | Plm1 | BGC0000123 | Polyketide | 27.0 | 24.4 | 134.0 | 9.5e-31 |
| ABA23460.1 | Amino\_acid\_adenylation | BGC0000427 | NRP | 24.0 | 22.4 | 56.0 | 3.36e-07 |
