# Supplementary material for: Uncovering the transcriptional landscape of Fomes fomentarius during fungal-based material production through gene co-expression network analysis
Source: Fungal Biol Biotechnol. 2025 Feb 13;12:1. doi: 10.1186/s40694-024-00192-3 (PMC11827164; doi:10.1186/s40694-024-00192-3)
Supplement: Supplementary file 1 — Supplementary Material 1 [file 40694_2024_192_MOESM1_ESM.zip › knownclusterblast/region1/jgi.p_Fomfom1_1369104_mibig_hits.html]

| MIBiG Protein | Description | MIBiG Cluster | MiBiG Product | % ID | % Coverage | BLAST Score | E-value |
| --- | --- | --- | --- | --- | --- | --- | --- |
| ACB12561.1 | Fum16 | BGC0000063 | Polyketide | 26.0 | 47.1 | 225.0 | 1.05e-61 |
| AEA35027.1 | hypothetical\_protein | BGC0002502 | Polyketide | 26.0 | 47.3 | 224.0 | 2.11e-61 |
| AAN74819.2 | Fum16p | BGC0000062 | Polyketide | 26.0 | 48.2 | 218.0 | 4.01e-59 |
| QCT05736.1 | Tri3 | BGC0001983 | Other | 30.0 | 39.0 | 206.0 | 1.62e-56 |
| AJO72702.1 | Acyl-CoA\_ligase/oxidoreductase | BGC0001381 | Polyketide | 29.0 | 38.5 | 192.0 | 4.95e-49 |
| AFO59871.1 | three-domain\_carboxylic\_acid\_reductase | BGC0000175 | Polyketide:Trans-AT type I polyketide | 31.0 | 30.4 | 179.0 | 3.28e-45 |
| AXM43064.1 | AMP-dependent\_synthetase\_and\_ligase | BGC0001945 | NRP | 27.0 | 42.7 | 167.0 | 6.97e-43 |
| ABF88003.1 | putative\_long-chain-fatty-acid\_CoA\_ligase | BGC0000871 | Other:Nucleoside | 27.0 | 29.2 | 120.0 | 7.51e-27 |
| AEW31026.1 | short-chain\_acyl-CoA\_synthetase | BGC0000407 | NRP | 26.0 | 35.3 | 117.0 | 1.11e-26 |
| ACC80687.1 | AMP-dependent\_synthetase\_and\_ligase | BGC0002711 | Polyketide | 26.0 | 34.5 | 116.0 | 1.43e-26 |
| AAF08801.1 | YngI | BGC0001103 | NRP+Polyketide | 26.0 | 33.0 | 110.0 | 1.91e-24 |
| ABS74201.1 | YngI | BGC0001095 | NRP | 26.0 | 35.5 | 109.0 | 3.3e-24 |
| AKD43499.1 | Acyl-CoA\_ligase | BGC0001409 | Polyketide | 28.0 | 25.2 | 107.0 | 1.62e-23 |
| AGM05529.1 | long-chain\_acyl-CoA\_synthetase | BGC0002098 | Polyketide | 25.0 | 33.5 | 103.0 | 1.83e-22 |
| ABU70356.1 | hypothetical\_protein | BGC0001890 | NRP | 27.0 | 26.0 | 101.0 | 1.59e-21 |
| AAX98210.1 | acyl\_CoA\_ligase | BGC0000052 | Polyketide | 26.0 | 23.0 | 100.0 | 4.07e-21 |
| AYU66239.1 | TjhA5 | BGC0002461 | Polyketide | 29.0 | 22.5 | 99.0 | 6.13e-21 |
| ACN64850.1 | PokL | BGC0001061 | Polyketide:Iterative type I polyketide+Polyketide:Type II polyketide+Saccharide:Hybrid/tailoring saccharide | 27.0 | 26.9 | 97.0 | 3.67e-20 |
| QVQ68786.1 | mmyL | BGC0002129 | Polyketide | 29.0 | 22.2 | 97.0 | 4.11e-20 |
| AXM42922.1 | AMP-dependent\_synthetase\_and\_ligase/heterocyclase | BGC0001940 | Polyketide | 25.0 | 34.1 | 97.0 | 4.73e-20 |
| antaF | putative\_Acyl-CoA\_ligase | BGC0001455 | NRP+Polyketide | 26.0 | 23.7 | 96.0 | 6.44e-20 |
| CAH10130.1 | feruloyl-CoA\_synthetase | BGC0000268 | Polyketide | 29.0 | 26.1 | 95.0 | 1.29e-19 |
| ADC79613.1 | BafX | BGC0000028 | Polyketide:Modular type I polyketide | 27.0 | 32.0 | 95.0 | 1.85e-19 |
| ADI58647.1 | 5-Aminolevulinate\_CoA\_ligase | BGC0000187 | Polyketide:Type II polyketide | 24.0 | 32.6 | 92.0 | 9.27e-19 |
| AQT01384.1 | SgnS0 | BGC0001690 | Polyketide | 24.0 | 33.8 | 91.0 | 6.9e-18 |
| QQZ01621.1 | long-chain\_fatty\_acid--CoA\_ligase | BGC0002497 | Other | 25.0 | 33.3 | 89.0 | 8.82e-18 |
| WP\_240809958.1 | long-chain\_fatty\_acid--CoA\_ligase | BGC0002686 | NRP | 25.0 | 31.0 | 87.0 | 4.09e-17 |
| DAC74137.1 | AMP-dependent\_synthetase | BGC0002019 | Terpene | 25.0 | 23.0 | 87.0 | 4.45e-17 |
| WP\_051729283.1 | AMP-binding\_protein | BGC0002137 | Polyketide | 29.0 | 20.4 | 87.0 | 5.14e-17 |
| EFY99277.1 | long-chain-fatty-acid--CoA\_ligase | BGC0002710 | NRP | 23.0 | 33.1 | 87.0 | 6.05e-17 |
| CAC20930.1 | PimS0\_protein | BGC0000125 | Polyketide | 24.0 | 33.0 | 87.0 | 1.79e-16 |
| BBA21084.1 | putative\_5-aminolevulinate\_CoA-ligase | BGC0001740 | NRP+Polyketide | 26.0 | 32.6 | 85.0 | 2.52e-16 |
| MBD2892711.1 | 3-methylmercaptopropionyl-CoA\_ligase | BGC0002718 | NRP | 24.0 | 29.8 | 84.0 | 5.11e-16 |
| AIL50189.1 | putative\_5-aminolevulinate\_CoA-ligase | BGC0000213 | Polyketide:Type II polyketide | 27.0 | 23.3 | 81.0 | 4.2e-15 |
| MBE3200457.1 | AMP-binding\_protein | BGC0002409 | NRP | 24.0 | 23.4 | 81.0 | 5.79e-15 |
| ABC34346.1 | acyl-CoA\_ligase | BGC0001102 | NRP+Polyketide:Modular type I polyketide+Polyketide:Trans-AT type I polyketide | 25.0 | 31.5 | 80.0 | 9.8e-15 |
| AGY30675.1 | Ann3 | BGC0001298 | Polyketide | 24.0 | 24.1 | 78.0 | 2.96e-14 |
| AGM05527.1 | AMP-dependent\_synthetase\_and\_ligase | BGC0002098 | Polyketide | 26.0 | 25.9 | 77.0 | 9.24e-14 |
| AAG29784.2 | amide\_synthetase | BGC0000833 | Saccharide:Hybrid/tailoring saccharide+Other:Aminocoumarin | 26.0 | 23.0 | 73.0 | 1.51e-12 |
| AAQ82561.1 | FscA | BGC0000034 | NRP+Polyketide | 24.0 | 25.4 | 72.0 | 3.84e-12 |
| AQH32483.1 | hybrid\_peptide\_synthetase/polyketide\_synthase | BGC0001667 | NRP+Polyketide | 23.0 | 22.2 | 72.0 | 4.11e-12 |
| ABS74181.1 | bacillomycin\_D\_synthetase\_A\_ | BGC0001090 | Polyketide+NRP:Lipopeptide | 24.0 | 22.4 | 72.0 | 4.29e-12 |
| ADH04681.1 | non-ribosomal\_peptide\_synthetase | BGC0001344 | NRP+Polyketide | 25.0 | 22.8 | 71.0 | 1.33e-11 |
| AAF00957.1 | mcyG | BGC0001017 | NRP+Polyketide:Modular type I polyketide | 24.0 | 22.4 | 71.0 | 1.58e-11 |
| BAB69698.1 | iturin\_A\_synthetase\_A | BGC0001098 | NRP+Polyketide | 23.0 | 22.4 | 69.0 | 3.7e-11 |
| ACU71632.1 | amide\_synthetase | BGC0001154 | Other | 26.0 | 23.9 | 65.0 | 3.9e-10 |
| AAN32979.1 | BarE | BGC0000962 | NRP+Polyketide:Modular type I polyketide | 24.0 | 22.3 | 65.0 | 6.46e-10 |
| QIC03949.1 | CyaA | BGC0002347 | Alkaloid | 26.0 | 23.2 | 64.0 | 8.48e-10 |
| ABS75100.1 | AMP-binding\_protein | BGC0002641 | NRP | 22.0 | 24.7 | 64.0 | 1.18e-09 |
| ABD14712.1 | cesB | BGC0000320 | NRP:Cyclic depsipeptide | 24.0 | 33.7 | 64.0 | 2.02e-09 |
| QYA95682.1 | amino\_acid\_adenylation\_domain-containing\_protein | BGC0002676 | NRP | 24.0 | 26.1 | 63.0 | 2.77e-09 |
| QSV12656.1 | AvmN | BGC0002456 | Polyketide+NRP | 29.0 | 24.8 | 62.0 | 3.88e-09 |
| AEA30272.1 | peptide\_synthetase | BGC0000429 | Polyketide+NRP:Cyclic depsipeptide | 24.0 | 26.1 | 62.0 | 8.13e-09 |
| BAE61264.1 |  | BGC0002238 | Polyketide | 24.0 | 31.1 | 61.0 | 8.24e-09 |
| CAD29795.1 | peptide\_synthetase | BGC0001015 | NRP+Polyketide | 23.0 | 22.8 | 61.0 | 1.33e-08 |
| QXJ21811.1 | amino\_acid\_adenylation\_domain-containing\_protein | BGC0002370 | NRP | 26.0 | 25.5 | 59.0 | 3.05e-08 |
| AHJ31215.1 | Long-chain-fatty-acid--CoA\_ligase | BGC0000430 | NRP+Polyketide:Modular type I polyketide | 24.0 | 25.7 | 59.0 | 4.05e-08 |
| QNH67550.1 | Cip22 | BGC0002108 | NRP | 24.0 | 27.2 | 59.0 | 4.16e-08 |
| CAG15025.1 | Acyl-CoA\_synthase | BGC0000441 | NRP | 26.0 | 21.6 | 58.0 | 5.85e-08 |
| CAE53367.1 | acyl-CoA\_ligase | BGC0000440 | NRP:Glycopeptide | 26.0 | 21.6 | 58.0 | 5.87e-08 |
| AHH53507.1 | non-ribosomal\_peptide\_synthetase | BGC0000439 | NRP:Lipopeptide:Ca+-dependent lipopeptide | 26.0 | 26.8 | 58.0 | 9.32e-08 |
| BCK51658.1 | putative\_acyl-CoA\_synthetase | BGC0002520 | Polyketide | 23.0 | 25.2 | 57.0 | 1.46e-07 |
| AAF08795.1 | MycA | BGC0001103 | NRP+Polyketide | 23.0 | 23.9 | 57.0 | 2.66e-07 |
| AEH59100.1 | amino\_acid\_adenylation\_domain-containing\_protein/NRPS | BGC0000385 | NRP | 26.0 | 28.6 | 57.0 | 2.74e-07 |
| AID65222.1 | putative\_aspartate\_racemase | BGC0000335 | NRP+Polyketide | 23.0 | 21.6 | 56.0 | 3.49e-07 |
| AEU11006.1 | NpnB | BGC0001029 | NRP+Polyketide | 24.0 | 33.7 | 56.0 | 6.02e-07 |
| QRI43520.1 | NRPS/PKS\_hybrid | BGC0002454 | Polyketide | 28.0 | 23.7 | 55.0 | 1.01e-06 |
| EFL06865.1 | hypothetical\_protein | BGC0000300 | NRP | 24.0 | 27.4 | 54.0 | 1.28e-06 |
| QNL14925.1 | AptD | BGC0002512 | NRP | 22.0 | 27.1 | 54.0 | 1.57e-06 |
| AGI89790.1 | ATP-dependent\_valine\_adenylase | BGC0001792 | NRP | 25.0 | 23.2 | 54.0 | 1.75e-06 |
| AEF16021.1 | acyl-CoA\_synthase | BGC0000379 | NRP | 24.0 | 23.7 | 53.0 | 2.07e-06 |
| AIG79241.1 | Hypothetical\_protein | BGC0000419 | Saccharide+NRP:Glycopeptide | 25.0 | 33.0 | 54.0 | 2.29e-06 |
| QYA95662.1 | amino\_acid\_adenylation\_domain-containing\_protein | BGC0002676 | NRP | 24.0 | 25.8 | 53.0 | 2.59e-06 |
| QBG38782.1 | Atr21 | BGC0001975 | NRP | 23.0 | 24.6 | 53.0 | 2.99e-06 |
| QSJ20135.1 | non-ribosomal\_peptide\_synthase/polyketide\_synthase | BGC0002572 | NRP+Polyketide | 24.0 | 23.7 | 53.0 | 3.11e-06 |
| ctg3\_28 |  | BGC0001853 | NRP+Polyketide:Modular type I polyketide | 24.0 | 27.1 | 52.0 | 4.12e-06 |
| ACO94461.1 | putative\_AMP-dependent\_acyl-CoA\_synthetase/ligase | BGC0000029 | Polyketide:Modular type I polyketide | 26.0 | 30.7 | 52.0 | 4.37e-06 |
| QEO75071.1 | AMP-dependent\_synthetase\_and\_ligase | BGC0002079 | NRP:Cyclic depsipeptide | 23.0 | 25.7 | 52.0 | 4.5e-06 |
| AQX36215.1 | siderophore\_synthetase | BGC0001527 | Other | 22.0 | 22.5 | 52.0 | 4.96e-06 |
| AAY37647.1 | Amino\_acid\_adenylation | BGC0000437 | NRP | 24.0 | 25.4 | 52.0 | 5.29e-06 |
| QRN75755.1 | Amino\_acid\_adenylation\_domain\_protein | BGC0002114 | NRP+Polyketide | 25.0 | 23.2 | 52.0 | 6.39e-06 |
| ORC16618.1 | hypothetical\_protein | BGC0001341 | NRP | 24.0 | 25.6 | 52.0 | 6.64e-06 |
| FIS9431\_RS32925 | non-ribosomal\_peptide\_synthetase | BGC0001467 | NRP:Cyclic depsipeptide+Polyketide:Modular type I polyketide | 24.0 | 23.4 | 52.0 | 8e-06 |
| AHD05679.1 | putative\_non-ribosomal\_peptide\_ligase/\_polyketide\_synthase\_hybrid | BGC0000402 | NRP | 21.0 | 24.0 | 52.0 | 8.78e-06 |
