# Supplementary material for: Uncovering the transcriptional landscape of Fomes fomentarius during fungal-based material production through gene co-expression network analysis
Source: Fungal Biol Biotechnol. 2025 Feb 13;12:1. doi: 10.1186/s40694-024-00192-3 (PMC11827164; doi:10.1186/s40694-024-00192-3)
Supplement: Supplementary file 1 — Supplementary Material 1 [file 40694_2024_192_MOESM1_ESM.zip › knownclusterblast/region1/jgi.p_Fomfom1_1204244_mibig_hits.html]

| MIBiG Protein | Description | MIBiG Cluster | MiBiG Product | % ID | % Coverage | BLAST Score | E-value |
| --- | --- | --- | --- | --- | --- | --- | --- |
| ACR78139.1 | hypothetical\_protein | BGC0000312 | NRP | 27.0 | 70.6 | 84.0 | 1.59e-17 |
| AFR69339.1 | esterase/lipase\_SpiI | BGC0001045 | NRP:Cyclic depsipeptide+Polyketide:Modular type I polyketide | 30.0 | 65.4 | 78.0 | 1.17e-15 |
| ABA41522.1 | esterase | BGC0000722 | Saccharide+Other:Cyclitol | 28.0 | 55.6 | 66.0 | 1.28e-11 |
| CAQ71836.1 | putative\_ESTERASE/LIPASE\_PROTEIN | BGC0001189 | NRP | 33.0 | 28.1 | 52.0 | 4.02e-07 |
