# Supplementary material for: Uncovering the transcriptional landscape of Fomes fomentarius during fungal-based material production through gene co-expression network analysis
Source: Fungal Biol Biotechnol. 2025 Feb 13;12:1. doi: 10.1186/s40694-024-00192-3 (PMC11827164; doi:10.1186/s40694-024-00192-3)
Supplement: Supplementary file 1 — Supplementary Material 1 [file 40694_2024_192_MOESM1_ESM.zip › knownclusterblast/region1/jgi.p_Fomfom1_1205360_mibig_hits.html]

| MIBiG Protein | Description | MIBiG Cluster | MiBiG Product | % ID | % Coverage | BLAST Score | E-value |
| --- | --- | --- | --- | --- | --- | --- | --- |
| ALI92656.1 | MRR1\_Major\_Facilitator\_Superfamily\_(MFS)\_protein | BGC0001338 | Polyketide:Iterative type I polyketide | 44.0 | 87.3 | 365.0 | 1.22e-120 |
| PLB34719.1 | major\_facilitator\_superfamily\_domain-containing\_protein | BGC0002749 | NRP+Polyketide | 41.0 | 88.7 | 363.0 | 6.23e-120 |
| QBK15054.1 | major\_facilitator\_TraF | BGC0002197 | Polyketide+NRP | 39.0 | 92.8 | 338.0 | 2.23e-110 |
| QOV03401.1 | SpoC | BGC0002262 | Polyketide | 42.0 | 88.4 | 338.0 | 3.46e-110 |
| XP\_001827195.1 |  | BGC0001996 | Other | 40.0 | 89.7 | 331.0 | 1.55e-107 |
| EWG54279.1 | hypothetical\_protein | BGC0001190 | Polyketide | 41.0 | 85.1 | 323.0 | 2.67e-102 |
| CAP95407.1 |  | BGC0001404 | Polyketide | 38.0 | 85.4 | 312.0 | 5.12e-100 |
| EED18004.1 | conserved\_hypothetical\_protein | BGC0000154 | Polyketide:Iterative type I polyketide | 39.0 | 87.1 | 308.0 | 2.63e-98 |
| KFH44385.1 | putative\_transporter-like\_protein | BGC0002190 | Polyketide | 39.0 | 84.9 | 304.0 | 8.78e-97 |
| CBF87867.1 | MFS\_multidrug\_transporter,\_putative\_(AFU\_orthologue;\_AFUA\_1G10370) | BGC0001699 | NRP | 38.0 | 85.2 | 290.0 | 3.25e-91 |
| XP\_023093494.1 |  | BGC0001995 | Terpene | 35.0 | 88.2 | 286.0 | 2.17e-90 |
| BAE63187.1 |  | BGC0002195 | Other | 37.0 | 86.5 | 284.0 | 1.48e-88 |
| CCT67996.1 | bikaverin\_cluster-efflux\_pump | BGC0000030 | Polyketide | 39.0 | 85.8 | 281.0 | 1.94e-88 |
| PIB02403.1 | putative\_transporter | BGC0001541 | Polyketide | 36.0 | 85.8 | 276.0 | 3.4e-86 |
| ARU80382.1 | MFS\_transporter | BGC0001542 | Polyketide | 36.0 | 86.2 | 267.0 | 1.25e-82 |
| EAU32817.1 | conserved\_hypothetical\_protein | BGC0000160 | Polyketide | 35.0 | 86.5 | 263.0 | 1.36e-81 |
| ASK38705.1 | major\_facilitator\_superfamily\_transporter | BGC0001436 | Polyketide:Iterative type I polyketide | 34.0 | 89.5 | 261.0 | 2.08e-80 |
| ADI24938.1 | VrtL | BGC0000168 | Polyketide:Iterative type I polyketide | 33.0 | 87.5 | 253.0 | 2.62e-77 |
| AGO86666.1 | putative\_MFS\_transporter | BGC0001255 | NRP+Polyketide | 34.0 | 90.6 | 246.0 | 3.34e-75 |
| OAQ63053.2 | major\_facilitator\_superfamily\_protein | BGC0002187 | Polyketide | 33.0 | 87.3 | 248.0 | 3.42e-75 |
| ACD39772.1 | major\_facilitator\_superfamily\_transporter | BGC0000134 | Polyketide | 32.0 | 88.2 | 242.0 | 5.72e-73 |
| AGO65989.1 | putative\_MFS\_transporter | BGC0000992 | NRP+Polyketide | 32.0 | 88.2 | 228.0 | 6.96e-68 |
| EED57520.1 | efflux\_pump\_antibiotic\_resistance\_protein,\_putative | BGC0001446 | Polyketide:Iterative type I polyketide | 29.0 | 76.8 | 170.0 | 5.42e-47 |
| XP\_023093496.1 |  | BGC0001995 | Terpene | 25.0 | 76.2 | 95.0 | 3.77e-20 |
| ADM34144.1 | efflux\_pump | BGC0001084 | NRP+Terpene+Alkaloid | 29.0 | 29.0 | 66.0 | 7.41e-11 |
| AMY15073.1 | MFS\_transporter | BGC0001339 | Polyketide:Iterative type I polyketide | 26.0 | 29.3 | 58.0 | 1.4e-08 |
| CAG44659.1 | conserved\_membrane\_protein | BGC0002681 | Other | 28.0 | 29.3 | 57.0 | 1.89e-08 |
| CAP12594.1 | elloramycin\_permease | BGC0000219 | Polyketide:Type II polyketide+Saccharide:Hybrid/tailoring saccharide | 26.0 | 29.5 | 55.0 | 1.71e-07 |
| AQP25575.1 | MFS\_family\_transporter | BGC0001590 | Polyketide | 27.0 | 27.5 | 54.0 | 3.91e-07 |
