# Supplementary material for: Uncovering the transcriptional landscape of Fomes fomentarius during fungal-based material production through gene co-expression network analysis
Source: Fungal Biol Biotechnol. 2025 Feb 13;12:1. doi: 10.1186/s40694-024-00192-3 (PMC11827164; doi:10.1186/s40694-024-00192-3)
Supplement: Supplementary file 1 — Supplementary Material 1 [file 40694_2024_192_MOESM1_ESM.zip › knownclusterblast/region1/jgi.p_Fomfom1_1205222_mibig_hits.html]

| MIBiG Protein | Description | MIBiG Cluster | MiBiG Product | % ID | % Coverage | BLAST Score | E-value |
| --- | --- | --- | --- | --- | --- | --- | --- |
| ESK96613.1 | polyketide\_synthase | BGC0002212 | Polyketide | 39.0 | 105.4 | 1135.0 | 0.0 |
| EJP62792.1 | polyketide\_synthase | BGC0001720 | Polyketide | 36.0 | 106.0 | 894.0 | 9.13e-289 |
| ESU09199.1 | hypothetical\_protein | BGC0002594 | Polyketide | 37.0 | 79.2 | 814.0 | 2.44e-260 |
| ETS82099.1 | hypothetical\_protein | BGC0002161 | Polyketide | 29.0 | 104.2 | 653.0 | 7.9e-200 |
| ADM79459.1 | PKS16\_protein | BGC0001266 | Polyketide | 29.0 | 97.6 | 642.0 | 3.23e-196 |
| EAU38791.1 | hypothetical\_protein | BGC0000161 | Polyketide:Iterative type I polyketide | 29.0 | 102.9 | 637.0 | 1.85e-194 |
| EHA55627.1 | conidial\_yellow\_pigment\_biosynthesis\_polyketide\_synthase | BGC0002154 | Polyketide | 28.0 | 104.6 | 637.0 | 7.85e-194 |
| BAE61567.1 |  | BGC0002175 | Polyketide | 30.0 | 95.5 | 635.0 | 3.05e-193 |
| gene6 |  | BGC0001906 | Polyketide | 28.0 | 103.9 | 620.0 | 1.45e-187 |
| CBF74114.1 | Conidial\_yellow\_pigment\_biosynthesis\_polyketide\_synthase\_(PKS)(EC\_2.3.1.-)\_[Source:UniProtKB/Swiss-Prot;Acc:Q03149] | BGC0000107 | Polyketide | 29.0 | 96.3 | 612.0 | 7.36e-185 |
| AAD38786.1 | polyketide\_synthase | BGC0001257 | Polyketide | 28.0 | 104.2 | 608.0 | 1.3e-183 |
| AAN59953.1 | polyketide\_synthase\_1 | BGC0001258 | Polyketide | 28.0 | 96.0 | 600.0 | 8.75e-181 |
| XP\_028481820.1 | non-reducing\_polyketide\_synthase | BGC0001866 | Polyketide | 30.0 | 86.3 | 599.0 | 9.72e-180 |
| ERF77221.1 | hypothetical\_protein | BGC0002215 | Polyketide | 28.0 | 97.6 | 584.0 | 4.16e-175 |
| EAL84397.1 | polyketide\_synthase | BGC0001118 | Polyketide:Iterative type I polyketide | 32.0 | 71.4 | 574.0 | 6.74e-174 |
| EED21099.1 | polyketide\_synthase,\_putative | BGC0001578 | Polyketide | 28.0 | 95.6 | 579.0 | 3.08e-173 |
| QDK64760.1 | AshP | BGC0002301 | Polyketide | 30.0 | 95.9 | 576.0 | 2.2e-172 |
| AUW31184.1 | putative\_type\_I\_PKS | BGC0001489 | Polyketide | 28.0 | 105.3 | 573.0 | 2.32e-171 |
| CCE67070.1 | polyketide\_synthase | BGC0001242 | Polyketide | 30.0 | 85.9 | 573.0 | 1.66e-170 |
| BAE65965.1 |  | BGC0002236 | Polyketide | 29.0 | 94.7 | 570.0 | 3.73e-170 |
| QKG86295.1 | non-reducing\_polyketide\_synthase | BGC0002253 | Polyketide | 28.0 | 97.0 | 568.0 | 5.66e-170 |
| CCE33500.1 | polyketide\_synthase\_that\_catalyse\_the\_condensation\_of\_one\_acetyl-CoA\_and\_six\_malonyl-CoA\_resulting\_in\_formation\_of\_nor-rubrofusarin | BGC0002596 | Polyketide | 29.0 | 101.8 | 560.0 | 1.93e-166 |
| EAA59563.1 | polyketide\_synthase | BGC0000057 | Polyketide:Iterative type I polyketide | 28.0 | 104.1 | 551.0 | 6.95e-164 |
| EGD99348.1 | polyketide\_synthase | BGC0001144 | Polyketide | 29.0 | 85.7 | 544.0 | 8.14e-163 |
| ESU07748.1 | hypothetical\_protein | BGC0002709 | Polyketide | 31.0 | 71.4 | 546.0 | 3.65e-162 |
| XP\_001798923.1 | polyketide\_synthase | BGC0001865 | Polyketide:Iterative type I polyketide | 28.0 | 94.6 | 543.0 | 4.74e-162 |
| KAF7597159.1 | hypothetical\_protein | BGC0002646 | Polyketide | 29.0 | 86.4 | 540.0 | 1.35e-161 |
| EAU31624.1 | hypothetical\_protein | BGC0002592 | Polyketide | 31.0 | 75.1 | 539.0 | 3.11e-161 |
| KAF7526531.1 | hypothetical\_protein | BGC0002244 | Polyketide | 30.0 | 74.7 | 539.0 | 6.26e-161 |
| KDB16994.1 | polyketide\_synthetase\_PksP | BGC0002177 | Polyketide | 29.0 | 95.9 | 545.0 | 2.27e-160 |
| AGO59040.1 | PtaA | BGC0000121 | Polyketide | 31.0 | 76.5 | 535.0 | 5e-160 |
| ACH72912.1 | AflC | BGC0000011 | Polyketide | 28.0 | 100.7 | 539.0 | 3.35e-159 |
| CCE31584.1 | polyketide\_synthase\_that\_catalyse\_the\_condensation\_of\_one\_acetyl-CoA\_and\_six\_malonyl-CoA\_resulting\_in\_formation\_of\_nor-rubrofusarin | BGC0001886 | Polyketide | 30.0 | 75.3 | 528.0 | 3.52e-157 |
| AKN45693.1 | polyketide\_synthase | BGC0001284 | Polyketide | 29.0 | 72.5 | 525.0 | 3.15e-156 |
| ADY00130.1 | polyketide\_synthase | BGC0000104 | Terpene+Polyketide:Iterative type I polyketide | 31.0 | 77.9 | 532.0 | 4e-156 |
| EAL89339.1 | polyketide\_synthase,\_putative | BGC0001403 | Polyketide | 29.0 | 79.2 | 523.0 | 1.97e-155 |
| ADI24926.1 | VrtA | BGC0000168 | Polyketide:Iterative type I polyketide | 28.0 | 87.3 | 523.0 | 3.49e-155 |
| BAE62229.1 |  | BGC0002237 | Polyketide | 31.0 | 71.8 | 523.0 | 8.18e-155 |
| AAS90093.1 | PksA | BGC0000006 | Polyketide | 27.0 | 98.7 | 525.0 | 9.43e-155 |
| CBF70387.1 | polyketide\_synthase,\_putative\_(JCVI) | BGC0000684 | Polyketide | 30.0 | 73.4 | 520.0 | 2.76e-154 |
| CAD19091.1 | StiG\_protein | BGC0000153 | NRP+Polyketide:Modular type I polyketide | 34.0 | 63.6 | 511.0 | 5.7e-154 |
| BAE71314.1 | polyketide\_synthase | BGC0000004 | Polyketide | 27.0 | 98.7 | 522.0 | 5.71e-154 |
| AAS90022.1 | PksA | BGC0000008 | Polyketide | 27.0 | 98.7 | 522.0 | 1.48e-153 |
| CCT67991.1 | bikaverin\_cluster-polyketide\_synthase | BGC0000030 | Polyketide | 30.0 | 73.2 | 520.0 | 3.49e-153 |
| QNT61260.1 | polyketide\_synthase | BGC0002507 | Polyketide | 30.0 | 72.2 | 516.0 | 4.17e-153 |
| AEN83889.1 | AdaA | BGC0000156 | Polyketide:Iterative type I polyketide | 31.0 | 73.6 | 515.0 | 1.19e-152 |
| BAP81867.1 | AndM | BGC0002612 | Terpene | 29.0 | 77.7 | 522.0 | 1.19e-152 |
| CAD19087.1 | StiC\_protein | BGC0000153 | NRP+Polyketide:Modular type I polyketide | 34.0 | 58.9 | 516.0 | 2.03e-152 |
| ABX60163.1 | polyketide\_synthase | BGC0000978 | NRP+Alkaloid+Polyketide:Modular type I polyketide | 35.0 | 54.4 | 511.0 | 4.5e-152 |
| AAS89999.1 | PksA | BGC0000007 | Polyketide | 27.0 | 105.9 | 516.0 | 1.96e-151 |
| QBG38888.1 | nr-PKS | BGC0002062 | Polyketide | 31.0 | 74.6 | 511.0 | 3.2e-151 |
| QCL09091.1 | dmx-nrPKS | BGC0002063 | Polyketide:Iterative type I polyketide | 29.0 | 81.0 | 510.0 | 5.8e-151 |
| ADF88277.1 | polyketide\_synthase | BGC0000981 | NRP+Polyketide | 34.0 | 53.3 | 508.0 | 7.57e-151 |
| EED57518.1 | polyketide\_synthase,\_putative | BGC0001446 | Polyketide:Iterative type I polyketide | 29.0 | 77.3 | 513.0 | 9.45e-151 |
| CDM36726.1 | Beta-ketoacyl\_synthase | BGC0001360 | Polyketide | 30.0 | 77.5 | 516.0 | 1.68e-150 |
| AGC95321.1 | CurS2 | BGC0000045 | Polyketide | 28.0 | 95.4 | 512.0 | 3.31e-150 |
| BCD52390.1 | polyketide\_synthase\_SptM | BGC0002537 | Polyketide+Terpene | 29.0 | 77.7 | 514.0 | 3.44e-150 |
| QLM00044.1 | polyketide\_synthase | BGC0002257 | Polyketide | 30.0 | 74.3 | 505.0 | 2.86e-149 |
| AAZ95017.1 | polyketide\_synthase | BGC0000048 | Polyketide | 28.0 | 83.0 | 511.0 | 3.28e-149 |
| AAS90047.1 | PksA | BGC0000009 | Polyketide | 27.0 | 98.5 | 508.0 | 5.9e-149 |
| AVI26389.1 | polyketide\_synthase | BGC0001800 | NRP+Polyketide | 33.0 | 61.7 | 504.0 | 1.47e-148 |
| ACC80700.1 | beta-ketoacyl\_synthase | BGC0002677 | Other | 34.0 | 53.9 | 505.0 | 3.26e-148 |
| ADF88280.1 | polyketide\_synthase | BGC0000981 | NRP+Polyketide | 34.0 | 57.0 | 503.0 | 7.63e-148 |
| QCF41201.1 | CcxJ | BGC0002726 | Polyketide | 29.0 | 80.4 | 501.0 | 9.57e-148 |
| CBF79143.1 | polyketide\_synthase,\_putative\_(JCVI) | BGC0000013 | Polyketide | 34.0 | 58.8 | 501.0 | 2.14e-147 |
| AEE88289.1 | CurA | BGC0000976 | NRP+Polyketide:Modular type I polyketide | 33.0 | 55.9 | 505.0 | 2.58e-147 |
| AAT70096.1 | CurA | BGC0001165 | NRP+Polyketide:Modular type I polyketide | 33.0 | 55.9 | 505.0 | 2.58e-147 |
| ABX60162.1 | polyketide\_synthase | BGC0000978 | NRP+Alkaloid+Polyketide:Modular type I polyketide | 34.0 | 56.9 | 500.0 | 6.57e-147 |
| EED53479.1 | polyketide\_synthase,\_putative | BGC0001304 | Polyketide | 31.0 | 69.1 | 497.0 | 1.11e-146 |
| AMB48442.1 | polyketide\_synthase | BGC0001357 | Polyketide | 33.0 | 53.9 | 488.0 | 4.48e-146 |
| AHB82053.1 | polyketide\_synthase | BGC0001019 | NRP+Polyketide:Modular type I polyketide | 36.0 | 52.9 | 491.0 | 1.22e-145 |
| EAU35431.1 | hypothetical\_protein | BGC0002734 | Polyketide | 28.0 | 85.9 | 501.0 | 1.66e-145 |
| AQA28563.1 | type\_I\_polyketide\_synthase | BGC0001663 | Polyketide | 32.0 | 53.2 | 486.0 | 5.03e-145 |
| ADF88276.1 | polyketide\_synthase | BGC0000981 | NRP+Polyketide | 33.0 | 57.4 | 494.0 | 5.96e-145 |
| BAV69313.1 | PrhL | BGC0001729 | Polyketide+Terpene | 30.0 | 77.9 | 498.0 | 1.34e-144 |
| AZH23788.1 | MgcR | BGC0001970 | NRP+Polyketide | 31.0 | 60.8 | 498.0 | 1.69e-144 |
| WP\_018540604.1 | type\_I\_polyketide\_synthase | BGC0001332 | NRP+Polyketide | 35.0 | 52.5 | 476.0 | 1.77e-144 |
| AQW44893.1 | polyketide\_synthase | BGC0001737 | NRP+Polyketide | 32.0 | 63.7 | 492.0 | 2.89e-144 |
| EWM63000.1 | non-ribosomal\_peptide\_synthetase | BGC0001328 | NRP:Cyclic depsipeptide+Polyketide:Modular type I polyketide | 35.0 | 54.0 | 481.0 | 3.61e-144 |
| ACC80699.1 | beta-ketoacyl\_synthase | BGC0002677 | Other | 32.0 | 64.7 | 492.0 | 4.52e-144 |
| CBD77748.1 | polyketide\_synthase | BGC0000974 | NRP+Polyketide | 33.0 | 61.0 | 491.0 | 8.07e-144 |
| CAQ18835.1 | polyketide\_synthase | BGC0000954 | NRP+Polyketide:Modular type I polyketide | 33.0 | 62.8 | 486.0 | 1.09e-143 |
| AHB82064.1 | polyketide\_synthase | BGC0001231 | NRP+Polyketide:Modular type I polyketide | 34.0 | 62.8 | 485.0 | 1.29e-143 |
| AFV96138.1 | polyketide\_synthase | BGC0001064 | Polyketide:Modular type I polyketide+Polyketide:Type III polyketide | 33.0 | 52.9 | 477.0 | 1.93e-143 |
| ARU81118.1 | CylD | BGC0001566 | Polyketide | 33.0 | 52.9 | 477.0 | 1.93e-143 |
| ADI24953.1 | GsfA | BGC0000070 | Polyketide:Iterative type I polyketide | 30.0 | 68.1 | 488.0 | 4.06e-143 |
| ABX60152.1 | polyketide\_synthase | BGC0000978 | NRP+Alkaloid+Polyketide:Modular type I polyketide | 33.0 | 57.4 | 489.0 | 4.2e-143 |
| ALI92655.1 | CitS\_citrinin\_polyketide\_synthase | BGC0001338 | Polyketide:Iterative type I polyketide | 29.0 | 78.9 | 493.0 | 5.79e-143 |
| ACD39753.1 | non-reducing\_polyketide\_synthase | BGC0000076 | Polyketide | 29.0 | 81.5 | 490.0 | 5.98e-143 |
| ACD39762.1 | non-reducing\_polyketide\_synthase | BGC0000077 | Polyketide | 29.0 | 81.5 | 490.0 | 5.98e-143 |
| BBF25315.1 | polyketide\_synthase | BGC0001923 | Terpene+Polyketide | 30.0 | 73.3 | 491.0 | 7.16e-143 |
| ABB90282.1 | polyketide\_synthase | BGC0001057 | NRP+Polyketide | 27.0 | 95.3 | 490.0 | 7.31e-143 |
| ART41209.1 | AdrD | BGC0001508 | Polyketide | 28.0 | 80.6 | 490.0 | 4.97e-142 |
| KKP00966.1 | RADS2\_nonreducing\_polyketide\_synthase | BGC0001854 | Polyketide:Iterative type I polyketide | 29.0 | 79.1 | 483.0 | 5.79e-142 |
| WP\_052165465.1 | type\_I\_polyketide\_synthase | BGC0001327 | NRP:Cyclic depsipeptide+Polyketide:Modular type I polyketide | 35.0 | 54.1 | 481.0 | 9.45e-142 |
| ADF88275.1 | polyketide\_synthase | BGC0000981 | NRP+Polyketide | 33.0 | 53.3 | 475.0 | 1.22e-141 |
| AEU11006.1 | NpnB | BGC0001029 | NRP+Polyketide | 33.0 | 52.3 | 490.0 | 1.58e-141 |
| AAC49191.1 | putative\_polyketide\_synthase | BGC0000152 | Polyketide | 27.0 | 101.1 | 487.0 | 1.6e-141 |
| AAF19814.1 | MtaF | BGC0001024 | NRP+Polyketide:Modular type I polyketide | 34.0 | 52.7 | 475.0 | 1.77e-141 |
| AWS21279.1 | type\_I\_polyketide\_synthase | BGC0001934 | Polyketide | 36.0 | 52.9 | 476.0 | 2.5e-141 |
| AZY91989.1 | polyketide\_synthase | BGC0002022 | Polyketide | 36.0 | 52.9 | 476.0 | 2.5e-141 |
| CAD19090.1 | StiF\_protein | BGC0000153 | NRP+Polyketide:Modular type I polyketide | 34.0 | 57.6 | 487.0 | 2.63e-141 |
| CAD19086.1 | StiB\_protein | BGC0000153 | NRP+Polyketide:Modular type I polyketide | 33.0 | 53.5 | 479.0 | 3.28e-141 |
| BBG67008.1 | polyketide\_synthase\_Sre6 | BGC0002604 | Polyketide | 29.0 | 84.5 | 483.0 | 3.82e-141 |
| AGC45624.1 | polyketide\_synthase | BGC0001394 | NRP+Polyketide | 33.0 | 61.6 | 486.0 | 5.12e-141 |
| CBD77736.1 | polyketide\_synthase | BGC0000974 | NRP+Polyketide | 32.0 | 61.3 | 481.0 | 1.41e-140 |
| DAB41916.1 | ArzN\_-\_PKS\_(KS,\_AT,\_OMT,\_KR,\_ACP) | BGC0001884 | NRP+Polyketide | 34.0 | 52.5 | 481.0 | 2.05e-140 |
| AGC45622.1 | polyketide\_synthase | BGC0001394 | NRP+Polyketide | 32.0 | 64.0 | 481.0 | 2.1e-140 |
| QCP68974.1 | VatV | BGC0002296 | NRP+Polyketide | 32.0 | 53.9 | 477.0 | 2.23e-140 |
| AVI26388.1 | polyketide\_synthase | BGC0001800 | NRP+Polyketide | 33.0 | 61.4 | 485.0 | 3.03e-140 |
| QCP68966.1 | VatW | BGC0002296 | NRP+Polyketide | 34.0 | 54.2 | 485.0 | 3.36e-140 |
| ATP76241.1 | NdaD | BGC0001705 | NRP+Polyketide | 32.0 | 64.3 | 486.0 | 3.37e-140 |
| AEE88280.1 | CurJ | BGC0000976 | NRP+Polyketide:Modular type I polyketide | 31.0 | 64.0 | 484.0 | 4.03e-140 |
| AAT70105.1 | CurJ | BGC0001165 | NRP+Polyketide:Modular type I polyketide | 31.0 | 64.0 | 484.0 | 4.03e-140 |
| ACR33078.1 | polyketide\_synthase | BGC0000017 | Alkaloid+Polyketide:Modular type I polyketide | 32.0 | 57.6 | 480.0 | 4.19e-140 |
| AAF00959.1 | mcyD | BGC0001017 | NRP+Polyketide:Modular type I polyketide | 32.0 | 64.0 | 485.0 | 6.01e-140 |
| AQW44888.1 | polyketide\_synthase | BGC0001737 | NRP+Polyketide | 36.0 | 56.5 | 485.0 | 7.07e-140 |
| AIW82279.1 | PuwB | BGC0001125 | NRP+Polyketide | 31.0 | 60.1 | 483.0 | 1.03e-139 |
| MCF2150415.1 | Polyketide\_synthase | BGC0002625 | NRP+Polyketide | 33.0 | 53.5 | 480.0 | 1.36e-139 |
| AFU82616.1 | polyketide\_synthase | BGC0000998 | NRP+Polyketide | 33.0 | 62.5 | 481.0 | 1.56e-139 |
| ABX60153.1 | polyketide\_synthase | BGC0000978 | NRP+Alkaloid+Polyketide:Modular type I polyketide | 34.0 | 53.1 | 469.0 | 1.87e-139 |
| QBM78307.1 | polyketide\_synthase | BGC0002542 | Polyketide+NRP | 34.0 | 52.9 | 483.0 | 1.95e-139 |
| DAB41653.1 | polyketide\_synthase | BGC0001583 | Polyketide | 35.0 | 48.8 | 473.0 | 2.16e-139 |
| CAQ18828.1 | polyketide\_synthase | BGC0000954 | NRP+Polyketide:Modular type I polyketide | 33.0 | 62.1 | 481.0 | 2.4e-139 |
| AGC45619.1 | polyketide\_synthase | BGC0001394 | NRP+Polyketide | 32.0 | 61.7 | 480.0 | 3.7e-139 |
| AQW44890.1 | polyketide\_synthase | BGC0001737 | NRP+Polyketide | 32.0 | 61.7 | 480.0 | 3.88e-139 |
| ctg1\_orf3 |  | BGC0001329 | Polyketide+NRP:Cyclic depsipeptide | 35.0 | 52.3 | 471.0 | 1.7e-138 |
| WP\_051206795.1 | type\_I\_polyketide\_synthase | BGC0002624 | NRP+Polyketide | 33.0 | 53.6 | 459.0 | 1.83e-138 |
| AAO62584.1 | polyketide\_synthase\_type\_1 | BGC0001016 | NRP+Polyketide | 33.0 | 60.5 | 480.0 | 1.91e-138 |
| AAK57187.1 | MxaC | BGC0001022 | NRP+Polyketide | 32.0 | 60.2 | 480.0 | 3.02e-138 |
| QDA77058.1 | polyketide\_synthase | BGC0002026 | NRP+Polyketide | 32.0 | 64.5 | 479.0 | 4.55e-138 |
| ATX68115.1 | malonyl\_CoA-acyl\_carrier\_protein\_transacylase | BGC0001772 | Polyketide | 34.0 | 54.0 | 474.0 | 5.5e-138 |
| ADZ24998.1 | polyketide\_synthase | BGC0000380 | NRP+Polyketide:Modular type I polyketide | 33.0 | 60.2 | 477.0 | 6.17e-138 |
| QCP68972.1 | VatE | BGC0002296 | NRP+Polyketide | 32.0 | 55.9 | 472.0 | 6.34e-138 |
| XP\_011392701.1 | uncharacterized\_protein | BGC0001281 | Polyketide | 29.0 | 76.8 | 474.0 | 7.54e-138 |
| QKW94285.1 | short-chain\_dehydrogenase/reductase\_SDR | BGC0002342 | NRP+Polyketide | 33.0 | 61.4 | 477.0 | 9.44e-138 |
| QCP68968.1 | VatM | BGC0002296 | NRP+Polyketide | 30.0 | 64.0 | 475.0 | 2.22e-137 |
| AFU82615.1 | polyketide\_synthase | BGC0000998 | NRP+Polyketide | 35.0 | 53.5 | 465.0 | 2.98e-137 |
| AEE88278.1 | CurL | BGC0000976 | NRP+Polyketide:Modular type I polyketide | 32.0 | 54.7 | 473.0 | 3.01e-137 |
| AAT70107.1 | CurL | BGC0001165 | NRP+Polyketide:Modular type I polyketide | 32.0 | 54.7 | 473.0 | 3.01e-137 |
| ATX68116.1 | malonyl\_CoA-acyl\_carrier\_protein\_transacylase | BGC0001772 | Polyketide | 32.0 | 56.0 | 474.0 | 4.24e-137 |
| AEU11005.1 | NpnA | BGC0001029 | NRP+Polyketide | 32.0 | 52.0 | 476.0 | 7.18e-137 |
| AAS98783.1 | polyketide\_synthase/nonribosomal\_peptide\_synthase\_hybrid | BGC0001001 | NRP+Polyketide | 34.0 | 53.1 | 475.0 | 8.24e-137 |
| AAK57188.1 | MxaD | BGC0001022 | NRP+Polyketide | 32.0 | 61.3 | 470.0 | 8.82e-137 |
| PKX88487.1 | polyketide\_synthase | BGC0001708 | Polyketide+Terpene | 29.0 | 71.8 | 473.0 | 9.71e-137 |
| AZH23819.1 | MgiR | BGC0001971 | NRP+Polyketide | 32.0 | 53.8 | 471.0 | 1.1e-136 |
| QBK15044.1 | clavatol\_synthase\_ClaF | BGC0002196 | Polyketide | 28.0 | 78.2 | 474.0 | 1.57e-136 |
| CAQ18829.1 | polyketide\_synthase | BGC0000954 | NRP+Polyketide:Modular type I polyketide | 35.0 | 53.1 | 474.0 | 2.32e-136 |
| CAD89777.1 | MelF\_protein | BGC0001010 | NRP+Polyketide:Modular type I polyketide | 34.0 | 52.8 | 460.0 | 2.68e-136 |
| ABM21570.1 | crpB | BGC0000975 | NRP+Polyketide | 31.0 | 62.0 | 473.0 | 4.21e-136 |
| AQW44891.1 | polyketide\_synthase | BGC0001737 | NRP+Polyketide | 33.0 | 59.7 | 467.0 | 8.65e-136 |
| AAS98777.1 | polyketide\_synthetase | BGC0001001 | NRP+Polyketide | 32.0 | 55.4 | 465.0 | 1.43e-135 |
| QEA08890.1 | JenA4 | BGC0002559 | Polyketide | 34.0 | 56.7 | 471.0 | 1.95e-135 |
| AXN93597.1 | PuwB | BGC0001952 | NRP | 30.0 | 64.5 | 469.0 | 3.55e-135 |
| MCF2150414.1 | Polyketide\_synthase | BGC0002625 | NRP+Polyketide | 31.0 | 60.6 | 469.0 | 3.77e-135 |
| AFY58526.1 | polyketide\_synthase\_family\_protein | BGC0002411 | NRP+Polyketide | 32.0 | 52.6 | 449.0 | 4.45e-135 |
| CAD19092.1 | StiH\_protein | BGC0000153 | NRP+Polyketide:Modular type I polyketide | 34.0 | 52.9 | 461.0 | 4.74e-135 |
| DAB41915.1 | ArzM\_-\_PKS\_(KS,\_AT,\_DH,\_MT,\_ER,\_KR,\_ACP) | BGC0001884 | NRP+Polyketide | 31.0 | 61.1 | 469.0 | 5.17e-135 |
| ADB12492.1 | EpoE | BGC0000990 | NRP+Polyketide | 31.0 | 64.1 | 469.0 | 8.07e-135 |
| ACD39770.1 | non-reducing\_polyketide\_synthase | BGC0000134 | Polyketide | 28.0 | 77.3 | 466.0 | 9e-135 |
| AAF62884.1 | EpoE | BGC0000991 | NRP+Polyketide | 31.0 | 64.1 | 469.0 | 1.08e-134 |
| AQW44892.1 | polyketide\_synthase | BGC0001737 | NRP+Polyketide | 33.0 | 57.7 | 463.0 | 1.74e-134 |
| MBV7329455.1 | amino\_acid\_adenylation\_domain-containing\_protein | BGC0002131 | Polyketide+NRP:Glycopeptide+Saccharide:Hybrid/tailoring saccharide | 34.0 | 53.3 | 467.0 | 2.15e-134 |
| KIA75596.1 | polyketide\_synthase | BGC0002209 | Polyketide | 29.0 | 81.1 | 466.0 | 2.34e-134 |
| KFA69335.1 | hypothetical\_protein | BGC0001626 | Polyketide | 27.0 | 86.1 | 466.0 | 2.38e-134 |
| CAD19093.1 | StiJ\_protein | BGC0000153 | NRP+Polyketide:Modular type I polyketide | 33.0 | 54.9 | 452.0 | 4.05e-134 |
| AIT55260.1 | polyketide\_synthase | BGC0000072 | Polyketide:Modular type I polyketide | 32.0 | 61.0 | 461.0 | 7.93e-134 |
| ADZ24997.1 | polyketide\_synthase | BGC0000380 | NRP+Polyketide:Modular type I polyketide | 32.0 | 58.0 | 461.0 | 1.17e-133 |
| WP\_035121546.1 | type\_I\_polyketide\_synthase | BGC0001467 | NRP:Cyclic depsipeptide+Polyketide:Modular type I polyketide | 32.0 | 54.6 | 462.0 | 1.19e-133 |
| QCP68970.1 | VatU | BGC0002296 | NRP+Polyketide | 31.0 | 53.8 | 462.0 | 1.28e-133 |
| BAV19379.1 | polyketide\_synthase | BGC0001390 | NRP+Polyketide | 28.0 | 86.2 | 464.0 | 1.31e-133 |
| PKX92308.1 | putative\_polyketide\_synthase | BGC0001988 | Polyketide | 28.0 | 80.9 | 459.0 | 1.96e-133 |
| AZH23791.1 | MgcH | BGC0001970 | NRP+Polyketide | 32.0 | 55.0 | 458.0 | 1.97e-133 |
| AXN93601.1 | PuwE | BGC0001952 | NRP | 32.0 | 56.8 | 464.0 | 2.13e-133 |
| CAQ43077.1 | polyketide\_synthase | BGC0000970 | NRP+Polyketide:Modular type I polyketide | 32.0 | 62.8 | 453.0 | 2.39e-133 |
| AAF26922.1 | polyketide\_synthase | BGC0000988 | NRP+Polyketide | 31.0 | 64.2 | 464.0 | 3.4e-133 |
| AXN93613.1 | PuwE | BGC0001953 | NRP | 32.0 | 56.8 | 464.0 | 3.77e-133 |
| AGC45623.1 | polyketide\_synthase | BGC0001394 | NRP+Polyketide | 32.0 | 60.8 | 459.0 | 5.95e-133 |
| AKL71649.1 | NocP | BGC0001703 | Other | 29.0 | 68.3 | 450.0 | 7.21e-133 |
| AAF62883.1 | epoD | BGC0000991 | NRP+Polyketide | 33.0 | 53.0 | 463.0 | 8.17e-133 |
| AAY42396.1 | Polyketide\_synthase | BGC0001000 | NRP:Lipopeptide+Polyketide:Modular type I polyketide | 32.0 | 52.4 | 459.0 | 8.5e-133 |
| AZH23789.1 | MgcI | BGC0001970 | NRP+Polyketide | 30.0 | 64.2 | 461.0 | 8.85e-133 |
| AAF26921.1 | polyketide\_synthase | BGC0000988 | NRP+Polyketide | 33.0 | 53.0 | 462.0 | 1.45e-132 |
| AAP42858.1 | NanA4 | BGC0000105 | Polyketide | 32.0 | 60.0 | 462.0 | 1.47e-132 |
| AQA28562.1 | type\_I\_polyketide\_synthase | BGC0001663 | Polyketide | 30.0 | 61.3 | 461.0 | 1.58e-132 |
| AEE88281.1 | CurI | BGC0000976 | NRP+Polyketide:Modular type I polyketide | 32.0 | 53.2 | 455.0 | 1.68e-132 |
| AAT70104.1 | CurI | BGC0001165 | NRP+Polyketide:Modular type I polyketide | 32.0 | 53.2 | 455.0 | 1.68e-132 |
| AEE88282.1 | CurH | BGC0000976 | NRP+Polyketide:Modular type I polyketide | 33.0 | 53.9 | 460.0 | 1.69e-132 |
| AAT70103.1 | CurH | BGC0001165 | NRP+Polyketide:Modular type I polyketide | 33.0 | 53.9 | 460.0 | 1.69e-132 |
| QCP68973.1 | VatL | BGC0002296 | NRP+Polyketide | 31.0 | 55.2 | 455.0 | 1.74e-132 |
| CAQ34928.1 | polyketide\_synthase | BGC0000986 | NRP+Polyketide | 32.0 | 60.1 | 457.0 | 2.33e-132 |
| AAF19810.1 | MtaB | BGC0001024 | NRP+Polyketide:Modular type I polyketide | 32.0 | 60.5 | 461.0 | 2.64e-132 |
| ADB12491.1 | EpoD | BGC0000990 | NRP+Polyketide | 34.0 | 52.4 | 461.0 | 3.43e-132 |
| AZH23821.1 | MgiH | BGC0001971 | NRP+Polyketide | 31.0 | 55.2 | 454.0 | 4.14e-132 |
| AXN93610.1 | PuwB | BGC0001953 | NRP | 30.0 | 65.2 | 460.0 | 4.91e-132 |
| AZH23818.1 | MgiI | BGC0001971 | NRP+Polyketide | 30.0 | 64.5 | 459.0 | 5.14e-132 |
| AAT70108.1 | CurM | BGC0001165 | NRP+Polyketide:Modular type I polyketide | 33.0 | 53.1 | 458.0 | 5.9e-132 |
| AXM42950.1 | polyketide\_synthase | BGC0001941 | NRP+Polyketide | 34.0 | 54.3 | 456.0 | 7.31e-132 |
| ACV42478.1 | polyketide\_synthase | BGC0000043 | Polyketide | 33.0 | 53.1 | 458.0 | 7.64e-132 |
| AEE88277.1 | CurM | BGC0000976 | NRP+Polyketide:Modular type I polyketide | 33.0 | 53.1 | 458.0 | 7.64e-132 |
| ACB37741.1 | putative\_type\_I\_polyketide\_synthase | BGC0000162 | Polyketide | 34.0 | 57.3 | 459.0 | 1.43e-131 |
| AIW82282.1 | PuwE | BGC0001125 | NRP+Polyketide | 32.0 | 55.5 | 459.0 | 1.57e-131 |
| CAQ34920.1 | polyketide\_synthase | BGC0000986 | NRP+Polyketide | 31.0 | 60.6 | 454.0 | 2.03e-131 |
| CAQ18834.1 | polyketide\_synthase | BGC0000954 | NRP+Polyketide:Modular type I polyketide | 35.0 | 53.3 | 458.0 | 2.64e-131 |
| AAW03329.1 | CtaF | BGC0000982 | NRP+Polyketide | 33.0 | 52.6 | 446.0 | 2.73e-131 |
| ACB46196.1 | polyketide\_synthase | BGC0000989 | NRP+Polyketide | 30.0 | 64.1 | 458.0 | 3.36e-131 |
| CAD29793.1 | polyketide\_synthase\_type\_I | BGC0001015 | NRP+Polyketide | 31.0 | 64.6 | 458.0 | 3.43e-131 |
| CAQ43076.1 | polyketide\_synthase | BGC0000970 | NRP+Polyketide:Modular type I polyketide | 33.0 | 57.5 | 454.0 | 4.07e-131 |
| ACB46195.1 | polyketide\_synthase | BGC0000989 | NRP+Polyketide | 33.0 | 53.0 | 458.0 | 4.55e-131 |
| AAS98782.1 | polyketide\_synthase | BGC0001001 | NRP+Polyketide | 30.0 | 57.6 | 451.0 | 5.65e-131 |
| AXN93586.1 | PuwB | BGC0001951 | NRP | 30.0 | 62.4 | 456.0 | 6.82e-131 |
| AXN93577.1 | PuwB | BGC0001950 | NRP | 30.0 | 62.4 | 456.0 | 9.09e-131 |
| QES95474.1 | type\_I\_polyketide\_synthase | BGC0002453 | Polyketide | 34.0 | 53.2 | 456.0 | 1.72e-130 |
| OAQ63055.1 | polyketide\_synthase | BGC0002187 | Polyketide | 28.0 | 77.1 | 453.0 | 2.03e-130 |
| CAQ43078.1 | polyketide\_synthase | BGC0000970 | NRP+Polyketide:Modular type I polyketide | 31.0 | 64.9 | 451.0 | 2.25e-130 |
| MBE8994630.1 | amino\_acid\_adenylation\_domain-containing\_protein | BGC0002623 | NRP+Polyketide | 31.0 | 54.9 | 455.0 | 2.37e-130 |
| AVI26390.1 | polyketide\_synthase\_/\_nonribosomal\_peptide\_synthase\_hybrid | BGC0001800 | NRP+Polyketide | 33.0 | 54.8 | 454.0 | 6.85e-130 |
| AHA12079.1 | polyketide\_synthase\_type\_1 | BGC0001172 | NRP+Polyketide:Modular type I polyketide | 34.0 | 52.6 | 442.0 | 1.42e-129 |
| ABO15860.1 | polyketide\_synthase | BGC0000130 | Polyketide | 33.0 | 54.8 | 451.0 | 2.08e-129 |
| AFU82617.1 | polyketide\_synthase | BGC0000998 | NRP+Polyketide | 34.0 | 55.1 | 449.0 | 4.76e-129 |
| CAO98849.1 | polyketide\_synthase\_AufF | BGC0000023 | Polyketide:Modular type I polyketide | 33.0 | 57.6 | 451.0 | 7.06e-129 |
| CCE88378.1 | polyketide\_synthase | BGC0001034 | NRP+Polyketide:Modular type I polyketide | 31.0 | 64.4 | 450.0 | 7.81e-129 |
| AUW31052.1 | putative\_type\_I\_PKS | BGC0002483 | Polyketide | 32.0 | 57.9 | 450.0 | 8.63e-129 |
| AAU04878.1 | polyketide\_synthase | BGC0000365 | NRP | 32.0 | 56.2 | 449.0 | 1.79e-128 |
| AAF26923.1 | polyketide\_synthase | BGC0000988 | NRP+Polyketide | 31.0 | 62.3 | 447.0 | 7.64e-128 |
| ACB46197.1 | polyketide\_synthase | BGC0000989 | NRP+Polyketide | 31.0 | 61.0 | 447.0 | 7.64e-128 |
| CBF69451.1 | polyketide\_synthase,\_putative\_(JCVI) | BGC0000037 | Polyketide | 27.0 | 86.2 | 447.0 | 8.34e-128 |
| AFP87523.1 | type\_I\_polyketide\_synthase | BGC0001159 | NRP+Polyketide:Modular type I polyketide | 33.0 | 53.7 | 446.0 | 1.29e-127 |
| MBA0053739.1 | acyltransferase\_domain-containing\_protein | BGC0002096 | Polyketide | 33.0 | 53.7 | 438.0 | 1.38e-127 |
| CAD19089.1 | StiE\_protein | BGC0000153 | NRP+Polyketide:Modular type I polyketide | 34.0 | 52.8 | 443.0 | 1.85e-127 |
| ACO94484.1 | polyketide\_synthase\_type\_I | BGC0000097 | Polyketide:Modular type I polyketide | 31.0 | 59.8 | 427.0 | 3.36e-127 |
| CAD89776.1 | MelE\_protein | BGC0001010 | NRP+Polyketide:Modular type I polyketide | 34.0 | 54.8 | 442.0 | 3.8e-127 |
| CAQ18833.1 | polyketide\_synthase | BGC0000954 | NRP+Polyketide:Modular type I polyketide | 34.0 | 53.2 | 438.0 | 4.36e-127 |
| ACR33079.1 | polyketide\_synthase | BGC0000017 | Alkaloid+Polyketide:Modular type I polyketide | 31.0 | 53.4 | 438.0 | 6.04e-127 |
| AHV78247.1 | LasS2 | BGC0001245 | Polyketide | 28.0 | 78.2 | 442.0 | 7.33e-127 |
| AAF62885.1 | EpoF | BGC0000991 | NRP+Polyketide | 31.0 | 61.0 | 444.0 | 7.6e-127 |
| AVV61980.1 | type\_I\_modular\_PKS | BGC0001477 | NRP+Polyketide:Modular type I polyketide | 34.0 | 57.5 | 444.0 | 1.02e-126 |
| WP\_102918845.1 | type\_I\_polyketide\_synthase | BGC0002104 | NRP+Polyketide | 33.0 | 56.7 | 444.0 | 1.07e-126 |
| SAI82896.1 | HrnA2;\_Starter\_unit\_polyketide\_synthase\_type\_I;\_module\_4\_(partial) | BGC0002101 | Polyketide | 30.0 | 59.9 | 425.0 | 1.61e-126 |
| ARM20280.1 | polyketide\_synthase | BGC0001523 | Polyketide | 32.0 | 58.5 | 444.0 | 1.88e-126 |
| WP\_051206794.1 | type\_I\_polyketide\_synthase | BGC0002624 | NRP+Polyketide | 31.0 | 52.9 | 420.0 | 2.54e-126 |
| ADN13832.1 | Polyketide\_Synthase | BGC0001164 | Polyketide:Modular type I polyketide | 32.0 | 53.2 | 442.0 | 3.08e-126 |
| EHA28237.1 | hypothetical\_protein | BGC0001143 | Polyketide | 28.0 | 79.8 | 442.0 | 3.69e-126 |
| ADH01663.1 | putative\_polyketide\_synthase\_PKS3 | BGC0000099 | Polyketide | 26.0 | 86.7 | 442.0 | 3.89e-126 |
| AGC24270.1 | prlP | BGC0001038 | NRP+Polyketide:Modular type I polyketide | 33.0 | 53.4 | 437.0 | 5.15e-126 |
| AVI57435.1 | AbmB3 | BGC0001694 | Polyketide | 33.0 | 52.7 | 423.0 | 5.29e-126 |
| ADB12493.1 | EpoF | BGC0000990 | NRP+Polyketide | 31.0 | 61.0 | 441.0 | 5.66e-126 |
| QEA08891.1 | JenA5 | BGC0002559 | Polyketide | 32.0 | 60.4 | 442.0 | 5.77e-126 |
| ABP55210.1 | beta-ketoacyl\_synthase | BGC0000142 | Polyketide | 33.0 | 57.0 | 441.0 | 1e-125 |
| QVV57685.1 | malonyl\_CoA-acyl\_carrier\_protein\_transacylase | BGC0002338 | Polyketide | 31.0 | 59.8 | 437.0 | 1.38e-125 |
| AGN71604.1 | conidial\_yellow\_pigment\_biosynthesis\_polyketide\_synthase | BGC0000027 | Polyketide:Iterative type I polyketide | 27.0 | 87.3 | 440.0 | 1.63e-125 |
| CAL58685.1 | polyketide\_synthase | BGC0000149 | Polyketide:Modular type I polyketide | 34.0 | 54.2 | 440.0 | 2.07e-125 |
| AXN93580.1 | PuwE | BGC0001950 | NRP | 31.0 | 56.8 | 439.0 | 2.62e-125 |
| AXN93589.1 | PuwE | BGC0001951 | NRP | 31.0 | 56.8 | 439.0 | 2.62e-125 |
| BAH02271.1 | polyketide\_synthase | BGC0000126 | Polyketide | 33.0 | 53.5 | 436.0 | 2.71e-125 |
| QOD95000.1 | PldAV | BGC0002102 | Polyketide | 33.0 | 53.5 | 436.0 | 2.71e-125 |
| QQZ01588.1 | PKS | BGC0002498 | Other | 32.0 | 59.9 | 439.0 | 3.88e-125 |
| BAC68128.1 | modular\_polyketide\_synthase | BGC0000059 | Polyketide | 30.0 | 68.4 | 439.0 | 3.94e-125 |
| CAO98847.1 | polyketide\_synthase\_AufC | BGC0000023 | Polyketide:Modular type I polyketide | 30.0 | 60.2 | 436.0 | 4.25e-125 |
| EAU31923.1 | hypothetical\_protein | BGC0002267 | Polyketide | 28.0 | 81.6 | 438.0 | 5.25e-125 |
| ABK32257.1 | AmbC | BGC0000014 | Polyketide | 32.0 | 62.0 | 439.0 | 5.35e-125 |
| CRI73799.1 | CongC\_protein | BGC0001215 | NRP | 33.0 | 58.6 | 439.0 | 6.09e-125 |
| AGC45620.1 | polyketide\_synthase | BGC0001394 | NRP+Polyketide | 30.0 | 63.7 | 439.0 | 6.58e-125 |
| AFV30247.1 | polyketide\_synthase | BGC0000075 | Polyketide | 34.0 | 52.8 | 439.0 | 6.75e-125 |
| ACR33077.1 | polyketide\_synthase | BGC0000017 | Alkaloid+Polyketide:Modular type I polyketide | 31.0 | 57.8 | 437.0 | 7.32e-125 |
| AFL48527.1 | laidlomycin\_polyketide\_synthase\_(module\_3\_and\_module\_4) | BGC0000084 | Polyketide | 32.0 | 58.8 | 437.0 | 1.33e-124 |
| AGC45621.1 | polyketide\_synthase | BGC0001394 | NRP+Polyketide | 31.0 | 61.8 | 434.0 | 1.68e-124 |
| ALP32043.1 | CycC | BGC0001293 | Polyketide | 32.0 | 62.0 | 437.0 | 2.25e-124 |
| TMU97101.1 | SDR\_family\_NAD(P)-dependent\_oxidoreductase | BGC0002038 | Polyketide | 32.0 | 58.4 | 437.0 | 2.39e-124 |
| QDA77059.1 | polyketide\_synthase/nonribosomal\_peptide\_synthetase | BGC0002026 | NRP+Polyketide | 34.0 | 53.1 | 437.0 | 2.48e-124 |
| AAW03325.1 | CtaB | BGC0000982 | NRP+Polyketide | 32.0 | 55.2 | 419.0 | 2.88e-124 |
| BAW35610.1 | modular\_polyketide\_synthase | BGC0002357 | Polyketide+Other | 33.0 | 53.5 | 437.0 | 3.02e-124 |
| ALA09371.1 | type\_I\_modular\_PKS | BGC0001303 | Polyketide | 32.0 | 57.0 | 437.0 | 3.07e-124 |
| AAO65801.1 | monensin\_polyketide\_synthase\_module\_9 | BGC0000100 | Polyketide | 33.0 | 55.1 | 431.0 | 3.72e-124 |
| ANZ52464.1 | MonAVI | BGC0001670 | Polyketide | 33.0 | 55.1 | 431.0 | 3.72e-124 |
| FS847\_01985 | type\_I\_polyketide\_synthase | BGC0001877 | Polyketide | 32.0 | 53.2 | 414.0 | 3.84e-124 |
| AAX98191.1 | polyketide\_synthase\_type\_I | BGC0000052 | Polyketide | 33.0 | 61.3 | 436.0 | 4.14e-124 |
| AZH23790.1 | MgcG | BGC0001970 | NRP+Polyketide | 31.0 | 54.8 | 433.0 | 4.28e-124 |
| QKG20144.1 | type\_I\_polyketide\_synthase | BGC0002124 | Polyketide | 31.0 | 60.9 | 432.0 | 4.28e-124 |
| TGZ15165.1 | hypothetical\_protein | BGC0002032 | Polyketide | 34.0 | 53.5 | 436.0 | 5.18e-124 |
| ADZ24996.1 | polyketide\_synthase | BGC0000380 | NRP+Polyketide:Modular type I polyketide | 32.0 | 61.1 | 434.0 | 5.59e-124 |
| AAF71766.1 | nysI | BGC0000115 | Polyketide:Modular type I polyketide+Saccharide:Hybrid/tailoring saccharide | 34.0 | 53.0 | 436.0 | 5.82e-124 |
| AWH12936.1 | StmA | BGC0001784 | Polyketide | 32.0 | 60.5 | 436.0 | 6.81e-124 |
| EED18001.1 | NR-PKS | BGC0000154 | Polyketide:Iterative type I polyketide | 28.0 | 79.7 | 434.0 | 8.72e-124 |
| ABK32259.1 | AmbE | BGC0000014 | Polyketide | 32.0 | 56.7 | 435.0 | 8.95e-124 |
| EPH46608.1 | putative\_Erythronolide\_synthase,\_modules\_3\_and\_4 | BGC0001519 | NRP+Polyketide | 31.0 | 55.8 | 417.0 | 1.08e-123 |
| AAP42859.1 | NanA5 | BGC0000105 | Polyketide | 31.0 | 60.5 | 434.0 | 1.31e-123 |
| AVI57434.1 | AbmB2 | BGC0001694 | Polyketide | 32.0 | 61.0 | 434.0 | 1.32e-123 |
| ARM20279.1 | polyketide\_synthase | BGC0001523 | Polyketide | 33.0 | 54.0 | 434.0 | 1.46e-123 |
| AFL48520.1 | laidlomycin\_polyketide\_synthase\_(module\_9) | BGC0000084 | Polyketide | 33.0 | 53.3 | 428.0 | 1.9e-123 |
| BAC68127.1 | modular\_polyketide\_synthase | BGC0000059 | Polyketide | 32.0 | 58.0 | 430.0 | 2.12e-123 |
| AZH23820.1 | MgiG | BGC0001971 | NRP+Polyketide | 31.0 | 54.9 | 431.0 | 2.49e-123 |
| QKG20163.1 | type\_I\_polyketide\_synthase | BGC0002124 | Polyketide | 32.0 | 62.1 | 434.0 | 2.6e-123 |
| AAK57186.1 | MxaB2 | BGC0001022 | NRP+Polyketide | 33.0 | 51.0 | 412.0 | 3.36e-123 |
| ACY13415.1 | KR\_domain\_protein | BGC0001367 | NRP+Polyketide | 34.0 | 54.1 | 432.0 | 4.32e-123 |
| ABI91466.1 | beta-ketoacyl\_synthase | BGC0001094 | NRP+Polyketide | 32.0 | 55.0 | 432.0 | 7.46e-123 |
| TXD00026.1 | SDR\_family\_NAD(P)-dependent\_oxidoreductase | BGC0001877 | Polyketide | 34.0 | 52.5 | 432.0 | 8.4e-123 |
| TXD00265.1 | SDR\_family\_NAD(P)-dependent\_oxidoreductase | BGC0001877 | Polyketide | 32.0 | 59.7 | 432.0 | 9.71e-123 |
| QNN81301.1 | IonAV | BGC0002446 | Polyketide | 33.0 | 57.8 | 431.0 | 1e-122 |
| ACN69988.1 | polyketide\_synthase | BGC0000079 | Polyketide | 34.0 | 54.0 | 432.0 | 1.07e-122 |
| CAL58687.1 | polyketide\_synthase | BGC0000149 | Polyketide:Modular type I polyketide | 31.0 | 60.9 | 431.0 | 1.18e-122 |
| QQZ01626.1 | PKS | BGC0002497 | Other | 34.0 | 53.0 | 432.0 | 1.18e-122 |
| AHH34186.1 | polyketide\_synthase | BGC0001161 | Polyketide:Modular type I polyketide | 32.0 | 53.3 | 430.0 | 1.24e-122 |
| CCE88376.1 | polyketide\_synthase | BGC0001034 | NRP+Polyketide:Modular type I polyketide | 33.0 | 53.7 | 431.0 | 1.28e-122 |
| ANR02551.1 | LodJ | BGC0001648 | Polyketide | 31.0 | 59.5 | 416.0 | 1.32e-122 |
| ABB05105.1 | LipPks4 | BGC0001003 | NRP:Lipopeptide+Polyketide:Modular type I polyketide+Saccharide:Hybrid/tailoring saccharide | 32.0 | 55.5 | 431.0 | 1.93e-122 |
| AVV61983.1 | type\_I\_modular\_polyketide\_synthase | BGC0001477 | NRP+Polyketide:Modular type I polyketide | 32.0 | 58.4 | 431.0 | 2.04e-122 |
| QVV57687.1 | polyketide\_synthase | BGC0002338 | Polyketide | 33.0 | 53.2 | 431.0 | 2.15e-122 |
| AUO16422.1 | polyketide\_synthase | BGC0001700 | Polyketide | 32.0 | 58.7 | 430.0 | 3.58e-122 |
| TXD00024.1 | SDR\_family\_NAD(P)-dependent\_oxidoreductase | BGC0001877 | Polyketide | 32.0 | 57.2 | 429.0 | 6.61e-122 |
| EAQ86392.1 | hypothetical\_protein | BGC0001405 | Polyketide | 34.0 | 45.9 | 415.0 | 6.88e-122 |
| BCK51640.1 | modular\_polyketide\_synthase | BGC0002520 | Polyketide | 33.0 | 57.3 | 429.0 | 6.97e-122 |
| EFY96950.1 | BcPKS18,\_polyketide\_synthase | BGC0002427 | Terpene+Polyketide | 33.0 | 55.5 | 427.0 | 8.25e-122 |
| OBR09781.1 | Polyketide\_synthase | BGC0002429 | Terpene+Polyketide | 33.0 | 55.2 | 427.0 | 9.11e-122 |
| AIT55261.1 | polyketide\_synthase | BGC0000072 | Polyketide:Modular type I polyketide | 33.0 | 52.9 | 422.0 | 1.14e-121 |
| ACY06286.1 | polyketide\_synthase | BGC0001042 | NRP+Polyketide | 33.0 | 57.0 | 427.0 | 1.2e-121 |
| AZH23817.1 | MgiQ | BGC0001971 | NRP+Polyketide | 28.0 | 61.3 | 427.0 | 1.56e-121 |
| QBM78312.1 | polyketide\_synthase | BGC0002542 | Polyketide+NRP | 29.0 | 61.4 | 427.0 | 1.68e-121 |
| AAZ94388.1 | nodular\_polyketide\_synthase | BGC0000040 | Polyketide | 32.0 | 58.2 | 428.0 | 1.87e-121 |
| BAW35615.1 | modular\_polyketide\_synthase | BGC0002357 | Polyketide+Other | 32.0 | 58.1 | 428.0 | 1.99e-121 |
| AIT55263.1 | polyketide\_synthase | BGC0000072 | Polyketide:Modular type I polyketide | 30.0 | 63.4 | 427.0 | 2.16e-121 |
| AVX51098.1 | nysI | BGC0001709 | Polyketide | 35.0 | 52.9 | 428.0 | 2.29e-121 |
| QFU19841.1 | PKS | BGC0002431 | Polyketide+Saccharide | 32.0 | 59.9 | 427.0 | 2.95e-121 |
| AFI57007.1 | QmnA3 | BGC0000133 | Polyketide | 33.0 | 53.7 | 416.0 | 3.38e-121 |
| KFH44362.1 | Conidial\_yellow\_pigment\_biosynthesis\_polyketide\_synthase-like\_protein | BGC0002190 | Polyketide | 30.0 | 58.7 | 426.0 | 3.42e-121 |
| QCO93110.1 | polyketide\_synthase | BGC0001976 | Terpene | 27.0 | 82.5 | 426.0 | 3.49e-121 |
| BAQ25483.1 | type\_I\_polyketide\_synthase | BGC0001288 | Polyketide | 32.0 | 53.0 | 411.0 | 3.79e-121 |
| AAA79984.2 | soraphen\_polyketide\_synthase\_B | BGC0000147 | Polyketide:Modular type I polyketide | 32.0 | 60.5 | 427.0 | 3.99e-121 |
| AFL48529.1 | laidlomycin\_polyketide\_synthase\_(module\_5\_and\_module\_6) | BGC0000084 | Polyketide | 33.0 | 53.3 | 426.0 | 5.07e-121 |
| TGZ15166.1 | hypothetical\_protein | BGC0002032 | Polyketide | 34.0 | 52.1 | 423.0 | 5.29e-121 |
| QCF28928.1 | type\_I\_polyketide\_synthase | BGC0002308 | Alkaloid+Polyketide | 33.0 | 57.4 | 426.0 | 5.6e-121 |
| BAR73017.1 | putative\_PKS\_(KS-AT-KR-ACP-KS-AT-DH-KR-ACP) | BGC0001194 | Polyketide | 33.0 | 56.8 | 426.0 | 5.95e-121 |
| BAR73019.1 | putative\_PKS\_(KS-AT-DH-KR-ACP-TE) | BGC0001194 | Polyketide | 33.0 | 57.0 | 424.0 | 6.1e-121 |
| AGY30676.1 | Ann4 | BGC0001298 | Polyketide | 32.0 | 60.4 | 426.0 | 6.38e-121 |
| BAT51065.1 | type\_I\_polyketide\_synthase | BGC0001296 | Polyketide | 32.0 | 57.8 | 426.0 | 7.42e-121 |
| BAW35613.1 | modular\_polyketide\_synthase | BGC0002357 | Polyketide+Other | 32.0 | 57.4 | 426.0 | 8.03e-121 |
| BAC76493.1 | lankamycin\_synthase\_LkmAI | BGC0000085 | Polyketide | 34.0 | 56.0 | 426.0 | 8.55e-121 |
| AAO65798.1 | monensin\_polyketide\_synthase\_modules\_3\_and\_4 | BGC0000100 | Polyketide | 33.0 | 60.2 | 426.0 | 9.32e-121 |
| ANZ52461.1 | MonAIII | BGC0001670 | Polyketide | 33.0 | 60.2 | 426.0 | 9.32e-121 |
| QVV57686.1 | hypothetical\_protein | BGC0002338 | Polyketide | 33.0 | 54.0 | 425.0 | 1.06e-120 |
| BAW35609.1 | modular\_polyketide\_synthase | BGC0002357 | Polyketide+Other | 33.0 | 52.8 | 425.0 | 1.15e-120 |
| TGZ15168.1 | hypothetical\_protein | BGC0002032 | Polyketide | 32.0 | 57.0 | 426.0 | 1.21e-120 |
| AUO16401.1 | polyketide\_synthase | BGC0001700 | Polyketide | 31.0 | 58.5 | 426.0 | 1.22e-120 |
| ARM20282.1 | polyketide\_synthase | BGC0001523 | Polyketide | 32.0 | 58.6 | 425.0 | 1.44e-120 |
| AZF85941.1 | type\_I\_polyketide\_synthase | BGC0001963 | NRP+Polyketide | 32.0 | 57.6 | 425.0 | 1.66e-120 |
| AKU20507.1 | polyketide\_synthase | BGC0002687 | Polyketide+NRP | 33.0 | 54.0 | 424.0 | 2.24e-120 |
| AKD43769.1 | HerA2 | BGC0001349 | NRP+Polyketide | 30.0 | 61.5 | 407.0 | 2.27e-120 |
| AAO65800.1 | monensin\_polyketide\_synthase\_modules\_7\_and\_8 | BGC0000100 | Polyketide | 31.0 | 64.2 | 424.0 | 2.89e-120 |
| ANZ52463.1 | MonAV | BGC0001670 | Polyketide | 31.0 | 64.2 | 424.0 | 2.89e-120 |
| AMJ52084.1 | lijE | BGC0002255 | Polyketide | 28.0 | 80.2 | 423.0 | 3.4e-120 |
| AQT01382.1 | SgnS1 | BGC0001690 | Polyketide | 31.0 | 61.9 | 424.0 | 3.62e-120 |
| ADX66472.1 | ScnS1 | BGC0000108 | Polyketide | 31.0 | 62.3 | 424.0 | 3.62e-120 |
| QFU80900.1 | PKS | BGC0002550 | Polyketide | 33.0 | 57.8 | 424.0 | 3.99e-120 |
| AZF85945.1 | type\_I\_polyketide\_synthase | BGC0001963 | NRP+Polyketide | 31.0 | 61.2 | 424.0 | 4.27e-120 |
| ACN69991.1 | polyketide\_synthase | BGC0000079 | Polyketide | 32.0 | 58.3 | 422.0 | 4.53e-120 |
| AXG22407.1 | type\_I\_polyketide\_synthase | BGC0002024 | Polyketide | 32.0 | 59.3 | 424.0 | 4.59e-120 |
| ADC45534.1 | modular\_polyketide\_synthase | BGC0000093 | Polyketide | 31.0 | 57.6 | 424.0 | 4.6e-120 |
| QBF51758.1 | type\_I\_polyketide\_synthase | BGC0001856 | Polyketide:Modular type I polyketide | 33.0 | 54.3 | 424.0 | 5.09e-120 |
| AAQ84145.1 | Plm5 | BGC0000123 | Polyketide | 32.0 | 56.9 | 420.0 | 5.25e-120 |
| EGJ35088.1 | Polyketide\_synthase | BGC0001163 | Polyketide:Modular type I polyketide | 31.0 | 54.9 | 422.0 | 5.52e-120 |
| CAQ64688.1 | lasalocid\_modular\_polyketide\_synthase | BGC0000087 | Polyketide | 31.0 | 61.3 | 408.0 | 5.68e-120 |
| ABK32289.1 | JerC | BGC0000080 | Polyketide | 31.0 | 62.1 | 423.0 | 6.24e-120 |
| BAG85028.1 | putative\_polyketide\_synthase | BGC0000086 | Polyketide | 31.0 | 61.3 | 408.0 | 6.27e-120 |
| BAW35641.1 | modular\_polyketide\_synthase | BGC0002356 | Polyketide+Other | 33.0 | 53.2 | 423.0 | 6.69e-120 |
| AID65222.1 | putative\_aspartate\_racemase | BGC0000335 | NRP+Polyketide | 32.0 | 53.2 | 423.0 | 6.85e-120 |
| OPB37950.1 | hypothetical\_protein | BGC0002206 | Polyketide | 28.0 | 80.1 | 422.0 | 8.09e-120 |
| AEZ64504.1 | Herc | BGC0001065 | Polyketide | 32.0 | 60.5 | 423.0 | 8.59e-120 |
| EAU29529.1 | hypothetical\_protein | BGC0000682 | Terpene | 28.0 | 78.0 | 422.0 | 8.74e-120 |
| AVV61989.1 | beta-ketoacyl\_synthase | BGC0001477 | NRP+Polyketide:Modular type I polyketide | 31.0 | 59.2 | 420.0 | 1.01e-119 |
| BAW35636.1 | modular\_polyketide\_synthase | BGC0002356 | Polyketide+Other | 31.0 | 60.6 | 422.0 | 1.14e-119 |
| ACR50791.1 | putative\_polyketide\_synthase | BGC0000163 | Polyketide | 31.0 | 56.5 | 421.0 | 1.16e-119 |
| ADC79617.1 | BafAII | BGC0000028 | Polyketide:Modular type I polyketide | 30.0 | 66.3 | 422.0 | 1.35e-119 |
| BAR73020.1 | putative\_PKS\_(KS-AT-DH-KR-ACP-KS-AT-DH-KR-ACP-KS-AT-DH-KR-ACP) | BGC0001194 | Polyketide | 31.0 | 60.8 | 422.0 | 1.35e-119 |
| QKG20145.1 | type\_I\_polyketide\_synthase | BGC0002124 | Polyketide | 33.0 | 58.1 | 421.0 | 1.8e-119 |
| ARE67853.1 | AbsB1 | BGC0001492 | Polyketide | 32.0 | 58.6 | 422.0 | 1.95e-119 |
| QBF51757.1 | type\_I\_polyketide\_synthase | BGC0001856 | Polyketide:Modular type I polyketide | 33.0 | 53.5 | 421.0 | 2.41e-119 |
| BAC68126.1 | modular\_polyketide\_synthase | BGC0000059 | Polyketide | 33.0 | 53.7 | 421.0 | 2.55e-119 |
| ABK32288.1 | JerB | BGC0000080 | Polyketide | 31.0 | 61.9 | 421.0 | 2.59e-119 |
| AEZ53946.1 | polyketide\_synthase | BGC0000144 | Polyketide:Modular type I polyketide | 32.0 | 60.1 | 421.0 | 2.71e-119 |
| CAQ34919.1 | polyketide\_synthase | BGC0000986 | NRP+Polyketide | 31.0 | 60.4 | 418.0 | 2.96e-119 |
| ALP32046.1 | CycF | BGC0001293 | Polyketide | 32.0 | 57.7 | 420.0 | 3.12e-119 |
| AAZ77696.1 | ChlA3 | BGC0000036 | Polyketide:Modular type I polyketide+Polyketide:Iterative type I polyketide+Saccharide:Oligosaccharide | 31.0 | 60.0 | 421.0 | 3.3e-119 |
| ABK32256.1 | AmbB | BGC0000014 | Polyketide | 31.0 | 61.5 | 421.0 | 3.45e-119 |
| ABC84459.1 | NigAIV | BGC0000114 | Polyketide:Modular type I polyketide | 33.0 | 54.5 | 421.0 | 3.69e-119 |
| BBG28471.1 | polyketide\_synthase\_Cle1 | BGC0002603 | Polyketide | 27.0 | 85.3 | 417.0 | 3.9e-119 |
| AHA38203.1 | GphJ | BGC0000069 | Polyketide | 33.0 | 54.2 | 417.0 | 3.95e-119 |
| AHH34189.1 | polyketide\_synthase | BGC0001162 | Polyketide:Modular type I polyketide | 31.0 | 54.2 | 419.0 | 4.17e-119 |
| QQZ01628.1 | PKS | BGC0002497 | Other | 32.0 | 57.5 | 420.0 | 4.54e-119 |
| AJG44381.1 | MpaC' | BGC0002619 | Polyketide | 38.0 | 40.5 | 419.0 | 4.62e-119 |
| QEA08887.1 | JenA1 | BGC0002559 | Polyketide | 32.0 | 57.2 | 420.0 | 4.79e-119 |
| BAC68129.1 | modular\_polyketide\_synthase | BGC0000059 | Polyketide | 32.0 | 51.9 | 421.0 | 4.82e-119 |
| ABC84458.1 | NigAIII | BGC0000114 | Polyketide:Modular type I polyketide | 32.0 | 59.6 | 420.0 | 4.92e-119 |
| QHZ99322.1 | nargenicin\_biosynthesis\_PKS | BGC0001875 | Polyketide | 31.0 | 60.8 | 420.0 | 5.35e-119 |
| BCB17031.1 | modular\_polyketide\_synthase | BGC0002523 | NRP | 32.0 | 57.5 | 420.0 | 5.76e-119 |
| ACB37740.1 | putative\_type\_I\_polyketide\_synthase | BGC0000162 | Polyketide | 33.0 | 54.1 | 420.0 | 6.02e-119 |
| QQZ01627.1 | PKS | BGC0002497 | Other | 33.0 | 57.8 | 420.0 | 6.11e-119 |
| BCK51648.1 | modular\_polyketide\_synthase | BGC0002520 | Polyketide | 33.0 | 53.6 | 419.0 | 7.07e-119 |
| WP\_032929422.1 | type\_I\_polyketide\_synthase | BGC0002106 | Polyketide | 32.0 | 57.6 | 420.0 | 8.17e-119 |
| AAG23264.1 | polyketide\_synthase\_loading\_and\_extender\_module\_1 | BGC0000148 | Polyketide | 33.0 | 54.2 | 419.0 | 9.71e-119 |
| AAX98189.1 | polyketide\_synthase\_type\_I | BGC0000052 | Polyketide | 32.0 | 57.4 | 419.0 | 1e-118 |
| BAW35656.1 | modular\_polyketide\_synthase | BGC0002355 | Polyketide+Other | 31.0 | 57.4 | 419.0 | 1.01e-118 |
| CAJ46690.1 | polyketide\_synthase | BGC0000969 | NRP:Cyclic depsipeptide+Polyketide:Modular type I polyketide | 31.0 | 58.8 | 418.0 | 1.11e-118 |
| AAY28227.1 | HbmAIII | BGC0000074 | Polyketide | 31.0 | 62.5 | 419.0 | 1.11e-118 |
| WP\_102919232.1 | type\_I\_polyketide\_synthase | BGC0002104 | NRP+Polyketide | 32.0 | 55.0 | 419.0 | 1.18e-118 |
| BBA66513.1 | type\_I\_polyketide\_synthase | BGC0001495 | Polyketide | 33.0 | 52.9 | 419.0 | 1.19e-118 |
| BBM96641.1 | modular\_polyketide\_synthase | BGC0002452 | Polyketide | 33.0 | 52.7 | 419.0 | 1.2e-118 |
| AVI57433.1 | AbmB1 | BGC0001694 | Polyketide | 31.0 | 58.6 | 419.0 | 1.42e-118 |
| CAQ43075.1 | polyketide\_synthase | BGC0000970 | NRP+Polyketide:Modular type I polyketide | 32.0 | 57.7 | 418.0 | 1.52e-118 |
| AUO16400.1 | polyketide\_synthase | BGC0001700 | Polyketide | 32.0 | 54.0 | 419.0 | 1.66e-118 |
| QBF51760.1 | type\_I\_polyketide\_synthase | BGC0001856 | Polyketide:Modular type I polyketide | 32.0 | 57.2 | 418.0 | 1.87e-118 |
| BAG23202.1 | putative\_type-I\_PKS | BGC0002673 | Polyketide+Alkaloid | 31.0 | 57.6 | 418.0 | 1.88e-118 |
| AZH23787.1 | MgcQ | BGC0001970 | NRP+Polyketide | 28.0 | 61.9 | 418.0 | 1.9e-118 |
| BAW35659.1 | modular\_polyketide\_synthase | BGC0002355 | Polyketide+Other | 31.0 | 58.2 | 418.0 | 1.97e-118 |
| AWC08655.1 | polyketide\_synthase\_type\_I | BGC0001662 | Polyketide | 33.0 | 53.4 | 419.0 | 2.01e-118 |
| ABV91286.1 | type\_I\_modular\_polyketide\_synthase | BGC0000158 | Polyketide:Modular type I polyketide | 31.0 | 57.3 | 419.0 | 2.08e-118 |
| SCO70308.1 | Type\_I\_polyketide\_synthase | BGC0001433 | Polyketide:Modular type I polyketide | 32.0 | 58.2 | 418.0 | 2.18e-118 |
| QCF28927.1 | type\_I\_polyketide\_synthase | BGC0002308 | Alkaloid+Polyketide | 33.0 | 52.8 | 418.0 | 2.47e-118 |
| AAQ90174.1 | polyketide\_synthase\_type\_I | BGC0000128 | Polyketide | 33.0 | 53.2 | 414.0 | 2.49e-118 |
| QQZ01586.1 | PKS | BGC0002498 | Other | 32.0 | 57.3 | 418.0 | 2.6e-118 |
| QFU19840.1 | PKS | BGC0002431 | Polyketide+Saccharide | 31.0 | 62.8 | 418.0 | 2.67e-118 |
| AEZ54374.1 | PieA1 | BGC0000124 | Polyketide | 31.0 | 61.3 | 417.0 | 2.95e-118 |
| BAW35655.1 | modular\_polyketide\_synthase | BGC0002355 | Polyketide+Other | 32.0 | 53.1 | 418.0 | 3.08e-118 |
| QQZ01582.1 | PKS | BGC0002498 | Other | 33.0 | 53.5 | 417.0 | 3.17e-118 |
| CAL58686.1 | polyketide\_synthase | BGC0000149 | Polyketide:Modular type I polyketide | 31.0 | 62.0 | 417.0 | 3.21e-118 |
| ASZ00148.1 | polyketide\_synthase | BGC0001785 | Polyketide | 32.0 | 57.7 | 417.0 | 3.26e-118 |
| AAC46024.1 | polyketide\_synthase\_modules\_1\_and\_2 | BGC0000113 | Polyketide | 31.0 | 52.7 | 417.0 | 3.71e-118 |
| AAG23266.1 | polyketide\_synthase\_extender\_modules\_3-4 | BGC0000148 | Polyketide | 32.0 | 52.7 | 417.0 | 3.88e-118 |
| QIQ28635.1 | Nbc39 | BGC0002541 | Other | 33.0 | 52.7 | 417.0 | 3.9e-118 |
| CAO98850.1 | polyketide\_synthase\_AufG | BGC0000023 | Polyketide:Modular type I polyketide | 31.0 | 57.4 | 416.0 | 5.37e-118 |
| BAO66543.1 | type\_I\_polyketide\_synthase | BGC0000042 | Polyketide | 32.0 | 53.7 | 412.0 | 5.41e-118 |
| AUO16398.1 | polyketide\_synthase | BGC0001700 | Polyketide | 32.0 | 58.7 | 417.0 | 5.58e-118 |
| ADC79639.1 | TamAIII | BGC0001052 | NRP+Polyketide:Modular type I polyketide | 31.0 | 57.6 | 416.0 | 5.94e-118 |
| BAD08360.1 | polyketide\_synthase\_modules\_7-8 | BGC0000167 | Polyketide | 31.0 | 57.8 | 417.0 | 6.02e-118 |
| ABI94379.1 | tautomycetin\_biosynthetic\_PKS | BGC0000157 | Polyketide | 31.0 | 57.3 | 417.0 | 6.49e-118 |
| AAF19812.1 | MtaD | BGC0001024 | NRP+Polyketide:Modular type I polyketide | 31.0 | 57.5 | 416.0 | 7.09e-118 |
| BAG85027.1 | putative\_polyketide\_synthase | BGC0000086 | Polyketide | 30.0 | 61.1 | 417.0 | 7.29e-118 |
| CAQ64687.1 | lasalocid\_modular\_polyketide\_synthase | BGC0000087 | Polyketide | 30.0 | 61.1 | 417.0 | 7.32e-118 |
| ADM46360.1 | polyketide\_synthase | BGC0000106 | Polyketide | 32.0 | 57.7 | 416.0 | 7.54e-118 |
| ANH11412.1 | SceQ | BGC0001770 | Polyketide | 31.0 | 58.3 | 413.0 | 8.16e-118 |
| CAD89773.1 | MelB\_protein | BGC0001010 | NRP+Polyketide:Modular type I polyketide | 32.0 | 53.8 | 400.0 | 8.56e-118 |
| QGA70099.1 | type\_I\_polyketide\_synthase | BGC0002517 | Polyketide | 31.0 | 57.7 | 416.0 | 9.45e-118 |
| QBL56210.1 | PKS | BGC0002376 | Polyketide | 32.0 | 58.0 | 416.0 | 9.69e-118 |
| AAZ77697.1 | ChlA4 | BGC0000036 | Polyketide:Modular type I polyketide+Polyketide:Iterative type I polyketide+Saccharide:Oligosaccharide | 31.0 | 60.3 | 413.0 | 9.75e-118 |
| CBZ41585.1 | Type\_I\_modular\_polyketide\_synthase | BGC0000151 | Polyketide:Modular type I polyketide+Saccharide:Hybrid/tailoring saccharide | 31.0 | 60.5 | 416.0 | 1.01e-117 |
| CAC20931.1 | PimS1\_protein | BGC0000125 | Polyketide | 31.0 | 61.9 | 416.0 | 1.05e-117 |
| AFL48528.1 | laidlomycin\_polyketide\_synthase\_(module\_7\_and\_module\_8) | BGC0000084 | Polyketide | 32.0 | 53.5 | 416.0 | 1.08e-117 |
| AAO06918.1 | GdmAIII | BGC0000066 | Polyketide | 31.0 | 62.2 | 416.0 | 1.08e-117 |
| AWC08657.1 | polyketide\_synthase\_type\_I | BGC0001662 | Polyketide | 31.0 | 57.3 | 416.0 | 1.11e-117 |
| BAG85026.1 | putative\_polyketide\_synthase | BGC0000086 | Polyketide | 32.0 | 57.7 | 416.0 | 1.23e-117 |
| CAQ64686.1 | lasalocid\_modular\_polyketide\_synthase | BGC0000087 | Polyketide | 32.0 | 57.7 | 416.0 | 1.24e-117 |
| CAL58683.1 | polyketide\_synthase | BGC0000149 | Polyketide:Modular type I polyketide | 32.0 | 53.5 | 411.0 | 1.24e-117 |
| TGZ15164.1 | hypothetical\_protein | BGC0002032 | Polyketide | 33.0 | 51.6 | 416.0 | 1.27e-117 |
| AKL64829.1 | polyketide\_synthase | BGC0002072 | Polyketide:Modular type I polyketide | 30.0 | 61.1 | 416.0 | 1.43e-117 |
| CBF83139.1 | polyketide\_synthase,\_putative\_(JCVI) | BGC0001722 | Polyketide | 27.0 | 82.7 | 414.0 | 1.43e-117 |
| QQZ01583.1 | PKS | BGC0002498 | Other | 32.0 | 52.8 | 416.0 | 1.48e-117 |
| CAP95404.1 |  | BGC0001404 | Polyketide | 28.0 | 79.9 | 415.0 | 1.72e-117 |
| ANH11415.1 | SceT | BGC0001770 | Polyketide | 33.0 | 53.1 | 405.0 | 1.82e-117 |
| AZF85946.1 | type\_I\_polyketide\_synthase | BGC0001963 | NRP+Polyketide | 33.0 | 51.9 | 416.0 | 1.82e-117 |
| AAC38076.1 | polyketide\_synthase\_type\_I | BGC0000127 | Polyketide | 33.0 | 53.1 | 412.0 | 1.88e-117 |
| ABB86410.1 | GelC | BGC0000067 | Polyketide | 31.0 | 62.0 | 415.0 | 1.89e-117 |
| BAW35658.1 | modular\_polyketide\_synthase | BGC0002355 | Polyketide+Other | 31.0 | 57.9 | 416.0 | 1.9e-117 |
| CAE45672.1 | Borrelidin\_polyketide\_synthase,\_type\_I | BGC0000031 | Polyketide:Modular type I polyketide | 33.0 | 53.4 | 411.0 | 2e-117 |
| BAG23199.1 | putative\_type-I\_PKS | BGC0002673 | Polyketide+Alkaloid | 31.0 | 57.3 | 414.0 | 2.05e-117 |
| ctg1\_15 |  | BGC0001931 | Polyketide | 33.0 | 54.4 | 415.0 | 2.08e-117 |
| ARM20277.1 | polyketide\_synthase | BGC0001523 | Polyketide | 31.0 | 58.8 | 415.0 | 2.22e-117 |
| AAX98188.1 | polyketide\_synthase\_type\_I | BGC0000052 | Polyketide | 33.0 | 53.8 | 415.0 | 2.22e-117 |
| ADM46357.1 | polyketide\_synthase | BGC0000106 | Polyketide | 32.0 | 57.3 | 415.0 | 2.25e-117 |
| WP\_244927023.1 | type\_I\_polyketide\_synthase | BGC0002104 | NRP+Polyketide | 33.0 | 53.4 | 400.0 | 2.3e-117 |
| AAG23265.1 | polyketide\_synthase\_extender\_module\_2 | BGC0000148 | Polyketide | 33.0 | 53.4 | 414.0 | 2.33e-117 |
| AAS98781.1 | polyketide\_synthase | BGC0001001 | NRP+Polyketide | 28.0 | 62.7 | 414.0 | 2.93e-117 |
| QKG20146.1 | Type\_I\_polyketide\_synthase | BGC0002124 | Polyketide | 31.0 | 61.5 | 415.0 | 3.06e-117 |
| EHK80167.1 | modular\_polyketide\_synthase | BGC0001447 | Polyketide | 32.0 | 54.9 | 415.0 | 3.1e-117 |
| TGZ15167.1 | polyketide\_synthase | BGC0002032 | Polyketide | 32.0 | 58.1 | 414.0 | 3.16e-117 |
| BCK51638.1 | modular\_polyketide\_synthase | BGC0002520 | Polyketide | 30.0 | 61.7 | 415.0 | 3.19e-117 |
| ADH04640.1 | TgaB | BGC0001051 | NRP+Polyketide:Modular type I polyketide | 30.0 | 63.4 | 414.0 | 4.02e-117 |
| AKD43753.1 | HerB | BGC0001349 | NRP+Polyketide | 32.0 | 57.2 | 414.0 | 4.15e-117 |
| BCP96883.1 | non-reducing\_polyketide\_synthase | BGC0002614 | NRP+Polyketide | 27.0 | 84.7 | 413.0 | 4.48e-117 |
| AMB48441.1 | polyketide\_synthase | BGC0001357 | Polyketide | 30.0 | 53.2 | 412.0 | 4.54e-117 |
| AQW44873.1 | polyketide\_synthase | BGC0001761 | Polyketide | 32.0 | 58.1 | 411.0 | 4.8e-117 |
| ACO94488.1 | polyketide\_synthase\_type\_I | BGC0000097 | Polyketide:Modular type I polyketide | 32.0 | 57.5 | 414.0 | 5.47e-117 |
| EAA65602.1 | hypothetical\_protein | BGC0000022 | Polyketide | 26.0 | 81.5 | 413.0 | 5.71e-117 |
| AEU17897.1 | putative\_type\_I\_PKS | BGC0001072 | Saccharide+Polyketide:Modular type I polyketide+Polyketide:Type II polyketide+Other:Aminocoumarin | 31.0 | 61.7 | 412.0 | 6.01e-117 |
| AEH42490.1 | polyketide\_synthase | BGC0000032 | Polyketide | 33.0 | 53.2 | 409.0 | 6.57e-117 |
| QQZ01629.1 | PKS | BGC0002497 | Other | 31.0 | 61.7 | 414.0 | 7.2e-117 |
| AEK75504.1 | type\_1\_polyketide\_synthase | BGC0000001 | Polyketide:Modular type I polyketide | 32.0 | 52.8 | 396.0 | 7.58e-117 |
| QIQ28636.1 | Nbc40 | BGC0002541 | Other | 32.0 | 59.6 | 414.0 | 8.02e-117 |
| QFU19826.1 | PKS | BGC0002431 | Polyketide+Saccharide | 29.0 | 61.6 | 412.0 | 8.36e-117 |
| ABB05102.1 | LipPks1 | BGC0001003 | NRP:Lipopeptide+Polyketide:Modular type I polyketide+Saccharide:Hybrid/tailoring saccharide | 31.0 | 53.6 | 412.0 | 8.83e-117 |
| BAW35612.1 | modular\_polyketide\_synthase | BGC0002357 | Polyketide+Other | 32.0 | 53.2 | 413.0 | 9.13e-117 |
| QWF78544.1 | 3-ketoacyl-CoA\_thiolase | BGC0002142 | Polyketide | 30.0 | 57.4 | 413.0 | 9.63e-117 |
| AAQ82564.1 | FscC | BGC0000034 | NRP+Polyketide | 30.0 | 62.0 | 413.0 | 1.12e-116 |
| AEZ54378.1 | PieA5 | BGC0000124 | Polyketide | 32.0 | 57.9 | 410.0 | 1.12e-116 |
| ALV82335.1 | borrelidin\_type\_I\_polyketide\_synthase | BGC0001533 | Polyketide | 32.0 | 53.4 | 409.0 | 1.16e-116 |
| AHA38201.1 | GphH | BGC0000069 | Polyketide | 32.0 | 55.0 | 408.0 | 1.18e-116 |
| BAR73021.1 | putative\_PKS\_(KS-AT-KR-ACP) | BGC0001194 | Polyketide | 31.0 | 53.2 | 407.0 | 1.25e-116 |
| ABC84471.1 | NigAVII | BGC0000114 | Polyketide:Modular type I polyketide | 32.0 | 54.8 | 412.0 | 1.55e-116 |
| AGZ15475.1 | putative\_type\_1\_modular\_polyketide\_synthase | BGC0001036 | NRP+Polyketide | 30.0 | 53.8 | 396.0 | 1.57e-116 |
| BAW35639.1 | modular\_polyketide\_synthase | BGC0002356 | Polyketide+Other | 31.0 | 57.6 | 412.0 | 1.88e-116 |
| ACO94456.1 | polyketide\_synthase\_type\_I | BGC0000029 | Polyketide:Modular type I polyketide | 31.0 | 58.6 | 412.0 | 2.2e-116 |
| QKG20147.1 | type\_I\_polyketide\_synthase | BGC0002124 | Polyketide | 32.0 | 57.7 | 412.0 | 2.21e-116 |
| QDA77044.1 | polyketide\_synthase | BGC0002025 | NRP+Polyketide | 30.0 | 61.5 | 412.0 | 2.3e-116 |
| AFI57006.1 | QmnA2 | BGC0000133 | Polyketide | 30.0 | 57.5 | 408.0 | 2.58e-116 |
| IF55\_RS36525 | polyketide\_synthase | BGC0001348 | Polyketide:Modular type I polyketide | 32.0 | 54.5 | 410.0 | 2.85e-116 |
| QFU19843.1 | PKS | BGC0002431 | Polyketide+Saccharide | 32.0 | 57.0 | 410.0 | 3.31e-116 |
| ABK32255.1 | AmbA | BGC0000014 | Polyketide | 31.0 | 54.7 | 396.0 | 3.34e-116 |
| AUO16403.1 | polyketide\_synthase | BGC0001700 | Polyketide | 30.0 | 58.3 | 410.0 | 3.7e-116 |
| AEZ64503.1 | Herd | BGC0001065 | Polyketide | 31.0 | 61.5 | 409.0 | 3.89e-116 |
| ADH04659.1 | TugC | BGC0001342 | NRP+Polyketide | 32.0 | 54.5 | 411.0 | 3.9e-116 |
| AEZ54377.1 | PieA4 | BGC0000124 | Polyketide | 32.0 | 57.1 | 410.0 | 3.97e-116 |
| ASZ00149.1 | polyketide\_synthase | BGC0001785 | Polyketide | 31.0 | 57.4 | 411.0 | 4e-116 |
| ADH04682.1 | polyketide\_synthase | BGC0001344 | NRP+Polyketide | 33.0 | 54.0 | 410.0 | 4.23e-116 |
| ATY46587.1 | polyketide\_synthase | BGC0001666 | Polyketide | 30.0 | 61.1 | 411.0 | 4.87e-116 |
| QCP68965.1 | VatK | BGC0002296 | NRP+Polyketide | 28.0 | 62.9 | 410.0 | 4.93e-116 |
| ASZ00151.1 | polyketide\_synthase | BGC0001785 | Polyketide | 31.0 | 57.4 | 410.0 | 5.3e-116 |
| ADH04657.1 | TugA | BGC0001342 | NRP+Polyketide | 32.0 | 59.1 | 411.0 | 5.36e-116 |
| BAQ21940.1 | putative\_Type\_I\_polyketide\_synthase | BGC0001204 | Polyketide | 31.0 | 57.9 | 411.0 | 5.66e-116 |
| ALV82346.1 | borrelidin\_type\_I\_polyketide\_synthase | BGC0001533 | Polyketide | 33.0 | 53.0 | 405.0 | 6.79e-116 |
| AJW65410.1 | type\_I\_modular\_polyketide\_synthase | BGC0001195 | NRP+Polyketide | 31.0 | 57.4 | 407.0 | 7.54e-116 |
| CAE46850.1 | Type\_I\_modular\_polyketide\_synthase | BGC0000103 | Polyketide | 31.0 | 58.1 | 409.0 | 7.61e-116 |
| QIZ24098.1 | type\_I\_polyketide\_synthase | BGC0002540 | Polyketide | 33.0 | 53.2 | 410.0 | 7.93e-116 |
| AAZ94387.1 | modular\_polyketide\_synthase | BGC0000040 | Polyketide | 32.0 | 54.9 | 410.0 | 8.49e-116 |
| WP\_245661582.1 | polyketide\_synthase | BGC0001348 | Polyketide:Modular type I polyketide | 32.0 | 54.5 | 410.0 | 9.14e-116 |
| AVV61979.1 | beta-ketoacyl\_synthase | BGC0001477 | NRP+Polyketide:Modular type I polyketide | 31.0 | 61.7 | 410.0 | 9.39e-116 |
| AAQ82568.1 | FscD | BGC0000034 | NRP+Polyketide | 32.0 | 53.8 | 410.0 | 1.05e-115 |
| CAE45668.1 | borrelidin\_polyketide\_synthase,\_type\_I | BGC0000031 | Polyketide:Modular type I polyketide | 33.0 | 52.9 | 404.0 | 1.2e-115 |
| QDA77045.1 | polyketide\_synthase/nonribosomal\_peptide\_synthetase | BGC0002025 | NRP+Polyketide | 31.0 | 54.2 | 410.0 | 1.31e-115 |
| BAW35651.1 | modular\_polyketide\_synthase | BGC0002355 | Polyketide+Other | 31.0 | 62.2 | 410.0 | 1.41e-115 |
| BCB17026.1 | modular\_polyketide\_synthase | BGC0002523 | NRP | 31.0 | 57.7 | 409.0 | 1.55e-115 |
| CAL58684.1 | polyketide\_synthase | BGC0000149 | Polyketide:Modular type I polyketide | 33.0 | 54.3 | 409.0 | 1.56e-115 |
| AKJ15837.1 | acyl\_transferase | BGC0002735 | Polyketide+NRP | 31.0 | 55.1 | 393.0 | 1.67e-115 |
| BCB17027.1 | modular\_polyketide\_synthase | BGC0002523 | NRP | 31.0 | 53.4 | 409.0 | 2.04e-115 |
| QWF78551.1 | hypothetical\_protein | BGC0002142 | Polyketide | 31.0 | 62.5 | 409.0 | 2.09e-115 |
| BAO66539.1 | type\_I\_polyketide\_synthase | BGC0000042 | Polyketide | 33.0 | 52.3 | 408.0 | 2.1e-115 |
| QPP46760.1 | polyketide\_synthase | BGC0002500 | Polyketide | 31.0 | 58.3 | 408.0 | 2.17e-115 |
| sipP3 | Type\_I\_Modular\_PKS | BGC0001452 | Polyketide | 31.0 | 62.9 | 409.0 | 2.21e-115 |
| QLD28380.2 | SDR\_family\_NAD(P)-dependent\_oxidoreductase | BGC0002086 | Polyketide | 32.0 | 59.9 | 409.0 | 2.26e-115 |
| UHY14125.1 | PKS\_I | BGC0002671 | Polyketide | 31.0 | 57.8 | 409.0 | 2.31e-115 |
| QKV49767.1 | PKS | BGC0002526 | Polyketide | 31.0 | 57.5 | 409.0 | 2.36e-115 |
| AAO65799.1 | monensin\_polyketide\_synthase\_modules\_5\_and\_6 | BGC0000100 | Polyketide | 32.0 | 59.1 | 409.0 | 2.38e-115 |
| ANZ52462.1 | MonAIV | BGC0001670 | Polyketide | 32.0 | 59.1 | 409.0 | 2.38e-115 |
| AUA09463.1 | Phenolphthiocerol\_synthesis\_polyketide\_synthase\_type\_I\_Pks15/1 | BGC0002291 | Polyketide | 32.0 | 57.7 | 407.0 | 2.53e-115 |
| AIT55259.1 | polyketide\_synthase | BGC0000072 | Polyketide:Modular type I polyketide | 32.0 | 53.3 | 403.0 | 2.91e-115 |
| SAI82895.1 | HrnA1;\_Starter\_unit\_polyketide\_synthase\_type\_I;\_modules\_loading,\_1-3 | BGC0002101 | Polyketide | 31.0 | 58.1 | 409.0 | 2.93e-115 |
| ANI24099.1 | polyketide\_synthase | BGC0001235 | NRP+Polyketide | 31.0 | 63.0 | 409.0 | 2.98e-115 |
| QSV12661.1 | AvmC | BGC0002456 | Polyketide+NRP | 30.0 | 62.1 | 408.0 | 2.99e-115 |
| QQZ01581.1 | PKS | BGC0002498 | Other | 31.0 | 56.2 | 409.0 | 3.15e-115 |
| AHA12078.1 | polyketide\_synthase\_type\_1 | BGC0001172 | NRP+Polyketide:Modular type I polyketide | 32.0 | 58.1 | 408.0 | 3.22e-115 |
| CQR60495.1 | Polyketide\_synthase,\_type\_I,\_module\_7 | BGC0001287 | Polyketide | 30.0 | 61.5 | 405.0 | 3.63e-115 |
| BAH02268.1 | polyketide\_synthase | BGC0000126 | Polyketide | 31.0 | 62.2 | 408.0 | 3.93e-115 |
| QOD94996.1 | PldAI | BGC0002102 | Polyketide | 31.0 | 62.2 | 408.0 | 3.93e-115 |
| AWC08660.1 | polyketide\_synthase\_type\_I | BGC0001662 | Polyketide | 31.0 | 60.2 | 408.0 | 4.03e-115 |
| CAQ64692.1 | lasalocid\_modular\_polyketide\_synthase | BGC0000087 | Polyketide | 32.0 | 52.6 | 398.0 | 4.14e-115 |
| AVX51108.1 | nysC | BGC0001709 | Polyketide | 33.0 | 52.9 | 408.0 | 4.44e-115 |
| AKL64834.1 | polyketide\_synthase | BGC0002072 | Polyketide:Modular type I polyketide | 33.0 | 54.9 | 408.0 | 4.46e-115 |
| AKD43768.1 | HerA1 | BGC0001349 | NRP+Polyketide | 33.0 | 57.7 | 408.0 | 4.52e-115 |
| AAO62582.1 | polyketide\_synthase\_peptide\_sythetase\_fusion\_protein | BGC0001016 | NRP+Polyketide | 32.0 | 52.9 | 407.0 | 4.98e-115 |
| ABW96540.1 | type\_I\_modular\_polyketide\_synthase | BGC0000159 | Polyketide:Modular type I polyketide | 31.0 | 60.8 | 408.0 | 4.99e-115 |
| QHZ99323.1 | nargenicin\_PKS | BGC0001875 | Polyketide | 33.0 | 53.3 | 408.0 | 5.04e-115 |
| AAZ77698.1 | ChlA5 | BGC0000036 | Polyketide:Modular type I polyketide+Polyketide:Iterative type I polyketide+Saccharide:Oligosaccharide | 31.0 | 62.0 | 407.0 | 5.46e-115 |
| BAO66541.1 | type\_I\_polyketide\_synthase | BGC0000042 | Polyketide | 32.0 | 57.6 | 405.0 | 6.27e-115 |
| BAO66529.1 | type\_I\_polyketide\_synthase | BGC0000042 | Polyketide | 34.0 | 51.9 | 407.0 | 6.36e-115 |
| AAX98185.1 | polyketide\_synthase\_type\_I | BGC0000052 | Polyketide | 32.0 | 56.0 | 407.0 | 6.52e-115 |
| BAG85030.1 | putative\_polyketide\_synthase | BGC0000086 | Polyketide | 32.0 | 57.9 | 407.0 | 6.9e-115 |
| CAQ64690.1 | lasalocid\_modular\_polyketide\_synthase | BGC0000087 | Polyketide | 32.0 | 57.9 | 407.0 | 6.9e-115 |
| AEZ53952.1 | polyketide\_synthase | BGC0000144 | Polyketide:Modular type I polyketide | 31.0 | 57.6 | 407.0 | 6.97e-115 |
| ABO15861.1 | polyketide\_synthase | BGC0000130 | Polyketide | 32.0 | 55.0 | 406.0 | 7.16e-115 |
| UHY14130.1 | PKS\_I | BGC0002671 | Polyketide | 32.0 | 52.8 | 402.0 | 7.58e-115 |
| TMU97102.1 | SDR\_family\_NAD(P)-dependent\_oxidoreductase | BGC0002038 | Polyketide | 31.0 | 60.6 | 406.0 | 8.13e-115 |
| ARM20283.1 | polyketide\_synthase | BGC0001523 | Polyketide | 31.0 | 57.8 | 405.0 | 8.49e-115 |
| AAF71767.1 | nysJ | BGC0000115 | Polyketide:Modular type I polyketide+Saccharide:Hybrid/tailoring saccharide | 32.0 | 53.1 | 407.0 | 8.57e-115 |
| AWW87423.1 | type\_I\_polyketide\_synthase | BGC0001755 | Polyketide | 33.0 | 54.1 | 407.0 | 9.12e-115 |
| BAQ21939.1 | putative\_type\_I\_polyketide\_synthase | BGC0001204 | Polyketide | 31.0 | 57.8 | 407.0 | 9.93e-115 |
| AAP42856.1 | NanA2 | BGC0000105 | Polyketide | 31.0 | 57.9 | 405.0 | 1.04e-114 |
| QNN81297.1 | IonAI | BGC0002446 | Polyketide | 30.0 | 61.7 | 407.0 | 1.05e-114 |
| QWF78553.1 | 3-ketoacyl-CoA\_thiolase | BGC0002142 | Polyketide | 30.0 | 57.6 | 407.0 | 1.05e-114 |
| BAW35608.1 | modular\_polyketide\_synthase | BGC0002357 | Polyketide+Other | 31.0 | 54.6 | 407.0 | 1.06e-114 |
| BAW35640.1 | modular\_polyketide\_synthase | BGC0002356 | Polyketide+Other | 31.0 | 57.1 | 406.0 | 1.13e-114 |
| AEC13069.1 | fosC | BGC0000060 | Polyketide | 31.0 | 61.0 | 406.0 | 1.18e-114 |
| QIZ24102.1 | type\_I\_polyketide\_synthase | BGC0002540 | Polyketide | 32.0 | 57.4 | 407.0 | 1.23e-114 |
| ABV83221.1 | CppI | BGC0000116 | Polyketide | 32.0 | 52.4 | 407.0 | 1.33e-114 |
| ABC84461.1 | NigAVI | BGC0000114 | Polyketide:Modular type I polyketide | 31.0 | 56.4 | 402.0 | 1.68e-114 |
| TMV00153.1 | acyltransferase\_domain-containing\_protein | BGC0002038 | Polyketide | 31.0 | 61.4 | 405.0 | 1.74e-114 |
| CAD15508.1 | polyketide\_synthase/non-ribosomal\_peptide\_synthetase | BGC0001014 | NRP:NRP siderophore+Polyketide:Modular type I polyketide+Polyketide:Iterative type I polyketide | 33.0 | 54.2 | 406.0 | 1.78e-114 |
| BAW35652.1 | modular\_polyketide\_synthase | BGC0002355 | Polyketide+Other | 32.0 | 53.9 | 405.0 | 2.05e-114 |
| ACO94483.1 | polyketide\_synthase\_type\_I | BGC0000097 | Polyketide:Modular type I polyketide | 31.0 | 58.0 | 406.0 | 2.12e-114 |
| AAQ82566.1 | FscF | BGC0000034 | NRP+Polyketide | 34.0 | 51.4 | 404.0 | 2.27e-114 |
| QNN81302.1 | IonAVI | BGC0002446 | Polyketide | 29.0 | 72.4 | 405.0 | 2.36e-114 |
| BAC57032.1 | protomycinolide\_IV\_synthase\_5 | BGC0000102 | Polyketide | 32.0 | 61.2 | 404.0 | 2.4e-114 |
| BAG85032.1 | putative\_polyketide\_synthase | BGC0000086 | Polyketide | 32.0 | 52.6 | 395.0 | 2.49e-114 |
| AAB66505.1 | tylactone\_synthase\_module\_3 | BGC0000166 | Polyketide | 33.0 | 59.3 | 403.0 | 2.63e-114 |
| QBG82527.1 | Polyketide\_synthase | BGC0002587 | Polyketide | 33.0 | 53.1 | 405.0 | 2.84e-114 |
| AAQ82567.1 | FscE | BGC0000034 | NRP+Polyketide | 31.0 | 57.2 | 405.0 | 2.97e-114 |
| ABX60161.1 | mixed\_NRPS/PKS | BGC0000978 | NRP+Alkaloid+Polyketide:Modular type I polyketide | 28.0 | 62.0 | 405.0 | 3.02e-114 |
| QIQ28616.1 | Nbc20 | BGC0002541 | Other | 31.0 | 53.7 | 405.0 | 3.12e-114 |
| AAC69329.1 | type\_I\_polyketide\_synthase\_PikAI | BGC0000094 | Polyketide:Modular type I polyketide+Saccharide:Hybrid/tailoring saccharide | 29.0 | 65.0 | 405.0 | 3.26e-114 |
| QKV49790.1 | PKS | BGC0002526 | Polyketide | 30.0 | 59.9 | 404.0 | 3.31e-114 |
| ANR02555.1 | LodN | BGC0001648 | Polyketide | 31.0 | 57.9 | 405.0 | 3.51e-114 |
| SAI82900.1 | HrnB;\_Macrolactam\_polyketide\_synthase\_type\_I;\_modules\_loading,\_1-2 | BGC0002101 | Polyketide | 31.0 | 57.9 | 405.0 | 3.63e-114 |
| BAJ16470.1 | polyketide\_synthase | BGC0000058 | Polyketide | 30.0 | 66.2 | 405.0 | 3.72e-114 |
| AAX98186.1 | polyketide\_synthase\_type\_I | BGC0000052 | Polyketide | 31.0 | 57.7 | 405.0 | 3.79e-114 |
| AGY30677.1 | Ann5 | BGC0001298 | Polyketide | 31.0 | 59.1 | 405.0 | 3.87e-114 |
| AAC46025.1 | polyketide\_synthase\_module\_3 | BGC0000113 | Polyketide | 30.0 | 53.3 | 402.0 | 4.12e-114 |
| ABI93779.1 | GdmPKS | BGC0000068 | Polyketide | 29.0 | 61.9 | 404.0 | 4.22e-114 |
| ctg1\_orf27 |  | BGC0000096 | Polyketide | 32.0 | 52.9 | 405.0 | 4.24e-114 |
| AAF71776.1 | nysC | BGC0000115 | Polyketide:Modular type I polyketide+Saccharide:Hybrid/tailoring saccharide | 32.0 | 58.2 | 405.0 | 4.24e-114 |
| AVV61985.1 | beta-ketoacyl\_synthase | BGC0001477 | NRP+Polyketide:Modular type I polyketide | 30.0 | 58.1 | 404.0 | 4.51e-114 |
| CAE45669.1 | borrelidin\_polyketide\_synthase,\_type\_I | BGC0000031 | Polyketide:Modular type I polyketide | 32.0 | 57.2 | 404.0 | 4.79e-114 |
| ABG02263.1 | SalA | BGC0000143 | Polyketide | 32.0 | 54.5 | 401.0 | 4.86e-114 |
| ABK32263.1 | AmbH | BGC0000014 | Polyketide | 32.0 | 54.2 | 401.0 | 4.99e-114 |
| QFU80901.1 | PKS | BGC0002550 | Polyketide | 31.0 | 57.4 | 404.0 | 5.24e-114 |
| QPP46749.1 | polyketide\_synthase | BGC0002500 | Polyketide | 31.0 | 57.7 | 402.0 | 5.47e-114 |
| ADX66461.1 | ScnS2 | BGC0000108 | Polyketide | 33.0 | 53.5 | 405.0 | 5.47e-114 |
| ctg1\_orf7 |  | BGC0000053 | Polyketide | 32.0 | 53.7 | 404.0 | 5.56e-114 |
| QQZ01585.1 | PKS | BGC0002498 | Other | 31.0 | 52.9 | 404.0 | 6.07e-114 |
| ATY46594.1 | polyketide\_synthase | BGC0001666 | Polyketide | 31.0 | 55.7 | 389.0 | 6.34e-114 |
| AWM95789.1 | non-reduciing\_polyketide\_synthase\_methylorcinaldehyde\_synthase | BGC0001827 | Polyketide | 27.0 | 81.0 | 404.0 | 6.48e-114 |
| ALP32042.1 | CycB | BGC0001293 | Polyketide | 30.0 | 62.8 | 403.0 | 6.57e-114 |
| QLD23838.1 | SDR\_family\_NAD(P)-dependent\_oxidoreductase | BGC0002086 | Polyketide | 34.0 | 53.3 | 401.0 | 7.15e-114 |
| WP\_081238291.1 | type\_I\_polyketide\_synthase | BGC0002105 | Polyketide | 33.0 | 53.1 | 404.0 | 7.21e-114 |
| AEZ53950.1 | polyketide\_synthase | BGC0000144 | Polyketide:Modular type I polyketide | 31.0 | 58.5 | 397.0 | 7.52e-114 |
| AKG06379.1 | polyketide\_synthase\_type\_1 | BGC0001830 | Polyketide | 33.0 | 53.7 | 401.0 | 7.69e-114 |
| AAS98787.1 | polyketide\_synthase/thioesterase | BGC0001001 | NRP+Polyketide | 30.0 | 62.6 | 401.0 | 8.26e-114 |
| CAC22145.1 | CpkB;\_Polyketide\_synthase\_modules\_3\_and\_4 | BGC0000038 | Polyketide:Modular type I polyketide | 29.0 | 57.7 | 404.0 | 8.56e-114 |
| AFL48526.1 | laidlomycin\_polyketide\_synthase\_(module\_2) | BGC0000084 | Polyketide | 30.0 | 57.7 | 402.0 | 9.28e-114 |
| ADF88279.1 | mixed\_NRPS/PKS | BGC0000981 | NRP+Polyketide | 29.0 | 61.8 | 403.0 | 9.34e-114 |
| TMU97089.1 | SDR\_family\_NAD(P)-dependent\_oxidoreductase | BGC0002038 | Polyketide | 31.0 | 58.0 | 402.0 | 9.84e-114 |
| ATP76239.1 | NdaF | BGC0001705 | NRP+Polyketide | 32.0 | 52.7 | 403.0 | 1.11e-113 |
| ALP32045.1 | CycE | BGC0001293 | Polyketide | 31.0 | 62.7 | 403.0 | 1.14e-113 |
| BAH02270.1 | polyketide\_synthase | BGC0000126 | Polyketide | 31.0 | 57.4 | 403.0 | 1.17e-113 |
| QOD94999.1 | PldAIV | BGC0002102 | Polyketide | 31.0 | 57.4 | 403.0 | 1.17e-113 |
| AAZ94386.1 | modular\_polyketide\_synthase | BGC0000040 | Polyketide | 31.0 | 58.6 | 403.0 | 1.2e-113 |
| TMU97100.1 | SDR\_family\_NAD(P)-dependent\_oxidoreductase | BGC0002038 | Polyketide | 31.0 | 58.2 | 403.0 | 1.25e-113 |
| AKG06376.1 | polyketide\_synthase\_type\_1 | BGC0001830 | Polyketide | 32.0 | 53.3 | 401.0 | 1.26e-113 |
| ABC84470.1 | NIGAVIII | BGC0000114 | Polyketide:Modular type I polyketide | 31.0 | 58.2 | 402.0 | 1.3e-113 |
| ATG32075.1 | polyketide\_synthase | BGC0001750 | NRP+Polyketide | 31.0 | 58.7 | 400.0 | 1.36e-113 |
| BAD08359.1 | polyketide\_synthase\_modules\_5-6 | BGC0000167 | Polyketide | 32.0 | 58.1 | 403.0 | 1.43e-113 |
| ACB46488.1 | polyketide\_synthase | BGC0000082 | Polyketide | 33.0 | 53.2 | 403.0 | 1.51e-113 |
| BAQ25513.1 | type\_I\_polyketide\_synthase | BGC0001288 | Polyketide | 31.0 | 61.2 | 403.0 | 1.6e-113 |
| ACC80698.1 | beta-ketoacyl\_synthase | BGC0002677 | Other | 30.0 | 53.3 | 389.0 | 1.61e-113 |
| BAR73007.1 | putative\_PKS\_(ACP-KS-AT-DH-KR-ACP-KS-AT-DH-ER-KR-ACP) | BGC0001194 | Polyketide | 31.0 | 57.7 | 403.0 | 1.63e-113 |
| QFU19839.1 | PKS | BGC0002431 | Polyketide+Saccharide | 30.0 | 58.3 | 403.0 | 1.64e-113 |
| BCK51641.1 | modular\_polyketide\_synthase | BGC0002520 | Polyketide | 33.0 | 53.3 | 403.0 | 1.7e-113 |
| AAB66508.1 | tylactone\_synthase\_module\_7 | BGC0000166 | Polyketide | 33.0 | 53.9 | 400.0 | 1.77e-113 |
| CAE46843.1 | Type\_I\_modular\_polyketide\_synthase | BGC0000103 | Polyketide | 31.0 | 57.8 | 403.0 | 1.8e-113 |
| SCN11949.1 | ebeA-type\_I\_polyketide\_synthase\_KSQ-ATa-ACP | BGC0001580 | Polyketide | 32.0 | 53.4 | 388.0 | 1.82e-113 |
| CAE46851.1 | Type\_I\_modular\_polyketide\_synthase | BGC0000103 | Polyketide | 31.0 | 57.8 | 403.0 | 1.84e-113 |
| ARS01477.1 | NcmAV | BGC0001702 | NRP+Polyketide | 33.0 | 52.7 | 402.0 | 1.95e-113 |
| ACB46471.1 | polyketide\_synthase | BGC0000082 | Polyketide | 32.0 | 60.3 | 402.0 | 2.13e-113 |
| BBA84067.1 | type\_I\_polyketide\_synthase | BGC0001649 | Polyketide | 32.0 | 53.4 | 400.0 | 2.24e-113 |
| AFV30248.1 | polyketide\_synthase | BGC0000075 | Polyketide | 29.0 | 64.2 | 401.0 | 2.34e-113 |
| ACO94460.1 | polyketide\_synthase\_type\_I | BGC0000029 | Polyketide:Modular type I polyketide | 32.0 | 57.4 | 402.0 | 2.62e-113 |
| AFV96142.1 | polyketide\_synthase | BGC0001064 | Polyketide:Modular type I polyketide+Polyketide:Type III polyketide | 29.0 | 52.8 | 401.0 | 3.13e-113 |
| ARU81122.1 | CylH | BGC0001566 | Polyketide | 29.0 | 52.8 | 401.0 | 3.13e-113 |
| AAG23262.1 | polyketide\_synthase\_extender\_modules\_8-10 | BGC0000148 | Polyketide | 32.0 | 58.2 | 402.0 | 3.39e-113 |
| ACC40922.1 | polyketide\_synthase,\_Pks8 | BGC0001665 | Polyketide | 31.0 | 57.2 | 400.0 | 3.44e-113 |
| ACR50775.1 | polyketide\_synthase | BGC0000163 | Polyketide | 31.0 | 57.3 | 402.0 | 3.49e-113 |
| BAQ21946.1 | putative\_type\_I\_polyketide\_synthase | BGC0001204 | Polyketide | 31.0 | 57.7 | 402.0 | 3.52e-113 |
| QWF78549.1 | 3-ketoacyl-CoA\_thiolase | BGC0002142 | Polyketide | 32.0 | 53.3 | 402.0 | 3.8e-113 |
| ABC84456.1 | NigAI | BGC0000114 | Polyketide:Modular type I polyketide | 32.0 | 58.5 | 401.0 | 3.83e-113 |
| WP\_030180235.1 | type\_I\_polyketide\_synthase | BGC0002106 | Polyketide | 31.0 | 53.5 | 402.0 | 3.93e-113 |
| QBG82531.1 | cytochrome\_P450 | BGC0002587 | Polyketide | 32.0 | 52.6 | 401.0 | 3.97e-113 |
| ADH04658.1 | TugB | BGC0001342 | NRP+Polyketide | 32.0 | 53.8 | 401.0 | 4.09e-113 |
| AUO16423.1 | polyketide\_synthase | BGC0001700 | Polyketide | 31.0 | 58.5 | 400.0 | 4.6e-113 |
| ACY06288.1 | type\_I\_polyketide\_synthase | BGC0001042 | NRP+Polyketide | 31.0 | 57.2 | 401.0 | 4.7e-113 |
| AGI99496.1 | Type\_I\_polyketide\_synthase | BGC0001004 | Polyketide:Modular type I polyketide | 31.0 | 57.4 | 402.0 | 4.88e-113 |
| SCN11952.1 | ebeD-type\_I\_polyketide\_synthase | BGC0001580 | Polyketide | 32.0 | 57.8 | 401.0 | 4.98e-113 |
| AEH42491.1 | polyketide\_synthase | BGC0000032 | Polyketide | 31.0 | 60.5 | 401.0 | 5e-113 |
| QGA70100.1 | type\_I\_polyketide\_synthase | BGC0002517 | Polyketide | 33.0 | 52.7 | 396.0 | 5.01e-113 |
| AAX98184.1 | polyketide\_synthase\_type\_I | BGC0000052 | Polyketide | 32.0 | 56.0 | 402.0 | 5.04e-113 |
| AEZ54379.1 | PieA6 | BGC0000124 | Polyketide | 31.0 | 57.8 | 400.0 | 5.07e-113 |
| AAF71768.1 | nysK | BGC0000115 | Polyketide:Modular type I polyketide+Saccharide:Hybrid/tailoring saccharide | 31.0 | 60.1 | 400.0 | 5.45e-113 |
| QIQ28617.1 | Nbc21 | BGC0002541 | Other | 31.0 | 59.7 | 401.0 | 5.72e-113 |
| ATL73034.1 | type\_I\_modular\_polyketide\_synthase | BGC0001807 | NRP+Polyketide | 31.0 | 60.6 | 401.0 | 6.37e-113 |
| AAP42857.1 | NanA3 | BGC0000105 | Polyketide | 31.0 | 58.7 | 401.0 | 6.7e-113 |
| AKA59088.1 | type-I\_PKS | BGC0001619 | Polyketide | 34.0 | 53.1 | 401.0 | 6.72e-113 |
| AAZ77699.1 | ChlA6 | BGC0000036 | Polyketide:Modular type I polyketide+Polyketide:Iterative type I polyketide+Saccharide:Oligosaccharide | 32.0 | 52.9 | 396.0 | 6.79e-113 |
| AAZ77693.1 | ChlA1 | BGC0000036 | Polyketide:Modular type I polyketide+Polyketide:Iterative type I polyketide+Saccharide:Oligosaccharide | 31.0 | 61.0 | 401.0 | 7.31e-113 |
| AQH32481.1 | hybrid\_polyketide\_synthase/peptide\_synthetase | BGC0001667 | NRP+Polyketide | 31.0 | 52.6 | 400.0 | 8.05e-113 |
| BAW35653.1 | modular\_polyketide\_synthase | BGC0002355 | Polyketide+Other | 32.0 | 52.8 | 401.0 | 8.42e-113 |
| AEZ53948.1 | polyketide\_synthase | BGC0000144 | Polyketide:Modular type I polyketide | 31.0 | 53.0 | 396.0 | 8.53e-113 |
| CAE02605.1 | polyketide\_synthase\_type\_I | BGC0000024 | Polyketide:Modular type I polyketide | 31.0 | 60.4 | 399.0 | 8.86e-113 |
| QFU19842.1 | PKS | BGC0002431 | Polyketide+Saccharide | 30.0 | 60.6 | 399.0 | 9.62e-113 |
| WP\_003060229.1 | type\_I\_polyketide\_synthase | BGC0002009 | Polyketide | 32.0 | 53.8 | 400.0 | 9.74e-113 |
| ANZ22985.1 | ZinB | BGC0001828 | Polyketide | 30.0 | 60.9 | 400.0 | 1.01e-112 |
| WP\_055480219.1 | type\_I\_polyketide\_synthase | BGC0001653 | Polyketide | 33.0 | 52.9 | 400.0 | 1.03e-112 |
| AVX51099.1 | NysJ | BGC0001709 | Polyketide | 31.0 | 60.9 | 400.0 | 1.03e-112 |
| ctg1\_orf10 |  | BGC0000053 | Polyketide | 31.0 | 57.5 | 400.0 | 1.03e-112 |
| QKV49771.1 | PKS | BGC0002526 | Polyketide | 31.0 | 53.8 | 396.0 | 1.06e-112 |
| ANH11410.1 | SceO | BGC0001770 | Polyketide | 31.0 | 57.6 | 400.0 | 1.07e-112 |
| ABB05103.1 | LipPks2 | BGC0001003 | NRP:Lipopeptide+Polyketide:Modular type I polyketide+Saccharide:Hybrid/tailoring saccharide | 30.0 | 58.0 | 400.0 | 1.08e-112 |
| AHH25595.1 | PKS | BGC0000957 | NRP+Polyketide | 31.0 | 60.6 | 400.0 | 1.14e-112 |
| ANZ22991.1 | ZinG | BGC0001828 | Polyketide | 33.0 | 53.5 | 399.0 | 1.18e-112 |
| BCK51649.1 | modular\_polyketide\_synthase | BGC0002520 | Polyketide | 31.0 | 61.4 | 397.0 | 1.19e-112 |
| ANZ22986.1 | ZinC | BGC0001828 | Polyketide | 31.0 | 61.9 | 399.0 | 1.21e-112 |
| ATY46595.1 | polyketide\_synthase | BGC0001666 | Polyketide | 31.0 | 58.0 | 397.0 | 1.22e-112 |
| ANR02552.1 | LodK | BGC0001648 | Polyketide | 32.0 | 53.5 | 396.0 | 1.22e-112 |
| QKV49769.1 | PKS | BGC0002526 | Polyketide | 31.0 | 58.2 | 400.0 | 1.35e-112 |
| QGA70078.1 | type\_I\_polyketide\_synthase | BGC0002517 | Polyketide | 32.0 | 57.0 | 400.0 | 1.41e-112 |
| CRI73798.1 | CongD\_protein | BGC0001215 | NRP | 31.0 | 57.9 | 399.0 | 1.45e-112 |
| QOD94997.1 | PldAII | BGC0002102 | Polyketide | 31.0 | 59.5 | 399.0 | 1.52e-112 |
| ACR50782.1 | polyketide\_synthase | BGC0000163 | Polyketide | 32.0 | 53.2 | 394.0 | 1.66e-112 |
| AAC01710.1 | RifA | BGC0000136 | Polyketide | 32.0 | 53.8 | 400.0 | 1.71e-112 |
| BCK51643.1 | modular\_polyketide\_synthase | BGC0002520 | Polyketide | 32.0 | 54.5 | 400.0 | 1.73e-112 |
| AWC08656.1 | polyketide\_synthase\_type\_I | BGC0001662 | Polyketide | 31.0 | 57.7 | 399.0 | 1.81e-112 |
| ACR50774.1 | polyketide\_synthase | BGC0000163 | Polyketide | 30.0 | 57.8 | 400.0 | 1.84e-112 |
| ALV82320.1 | borrelidin\_type\_I\_polyketide\_synthase | BGC0001533 | Polyketide | 32.0 | 57.3 | 399.0 | 1.87e-112 |
| AAP42855.1 | NanA1 | BGC0000105 | Polyketide | 31.0 | 57.2 | 399.0 | 2.06e-112 |
| TMU97099.1 | SDR\_family\_NAD(P)-dependent\_oxidoreductase | BGC0002038 | Polyketide | 30.0 | 61.9 | 399.0 | 2.07e-112 |
| CAC20921.1 | PimS2\_protein | BGC0000125 | Polyketide | 33.0 | 53.1 | 400.0 | 2.13e-112 |
| AQT01393.1 | SgnS2 | BGC0001690 | Polyketide | 33.0 | 53.1 | 400.0 | 2.13e-112 |
| BAE93722.1 | type\_I\_polyketide\_synthase | BGC0000164 | Polyketide | 31.0 | 58.2 | 399.0 | 2.29e-112 |
| AAS79460.1 | polyketide\_synthase\_subunit | BGC0000035 | Polyketide | 29.0 | 69.2 | 397.0 | 2.32e-112 |
| AMB20394.1 | polyketide\_synthase | BGC0002072 | Polyketide:Modular type I polyketide | 32.0 | 53.2 | 399.0 | 2.37e-112 |
| ABJ97437.1 | MerA | BGC0001012 | NRP+Polyketide | 33.0 | 52.8 | 399.0 | 2.46e-112 |
| AAP42860.1 | NanA6 | BGC0000105 | Polyketide | 31.0 | 55.4 | 395.0 | 2.47e-112 |
| AAK19883.1 | soraphen\_polyketide\_synthase\_A | BGC0000147 | Polyketide:Modular type I polyketide | 31.0 | 57.8 | 399.0 | 2.54e-112 |
| AGI99497.1 | type\_I\_polyketide\_synthase | BGC0001004 | Polyketide:Modular type I polyketide | 32.0 | 54.0 | 399.0 | 2.55e-112 |
| QFU80902.1 | PKS | BGC0002550 | Polyketide | 32.0 | 54.0 | 399.0 | 2.55e-112 |
| UHY14126.1 | PKS\_I | BGC0002671 | Polyketide | 31.0 | 60.1 | 398.0 | 2.65e-112 |
| ABC84460.1 | NigAV | BGC0000114 | Polyketide:Modular type I polyketide | 31.0 | 57.9 | 399.0 | 2.75e-112 |
| AFL48525.1 | laidlomycin\_polyketide\_synthase\_(loading\_module\_and\_module\_1) | BGC0000084 | Polyketide | 30.0 | 60.5 | 398.0 | 2.79e-112 |
| AUA09464.1 | Erythronolide\_synthase,\_modules\_3\_and\_4 | BGC0002291 | Polyketide | 31.0 | 57.8 | 399.0 | 3.2e-112 |
| ADX66470.1 | ScnS0 | BGC0000108 | Polyketide | 32.0 | 53.8 | 395.0 | 3.49e-112 |
| ABW96542.1 | type\_I\_modular\_polyketide\_synthase | BGC0000159 | Polyketide:Modular type I polyketide | 29.0 | 61.7 | 399.0 | 3.83e-112 |
| BAF85839.1 | modular\_polyketide\_synthase | BGC0000109 | Polyketide | 31.0 | 57.7 | 399.0 | 4.4e-112 |
| SCO70309.1 | Type\_I\_polyketide\_synthase | BGC0001433 | Polyketide:Modular type I polyketide | 30.0 | 57.0 | 398.0 | 4.42e-112 |
| ARW71485.1 | type\_I\_PKS\_module\_4,\_module\_5 | BGC0001812 | Polyketide | 32.0 | 60.1 | 398.0 | 4.57e-112 |
| QNN81300.1 | IonAIV | BGC0002446 | Polyketide | 30.0 | 57.9 | 398.0 | 4.99e-112 |
| AHE80995.1 | PieA5 | BGC0001169 | Polyketide:Modular type I polyketide | 31.0 | 61.4 | 396.0 | 5.1e-112 |
| ANH11413.1 | SceR | BGC0001770 | Polyketide | 32.0 | 54.9 | 393.0 | 5.3e-112 |
| QEA08888.1 | JenA2 | BGC0002559 | Polyketide | 31.0 | 62.1 | 397.0 | 5.38e-112 |
| ACR50785.1 | polyketide\_synthase | BGC0000163 | Polyketide | 30.0 | 58.3 | 398.0 | 5.42e-112 |
| ADM46358.1 | polyketide\_synthase | BGC0000106 | Polyketide | 32.0 | 57.4 | 398.0 | 5.55e-112 |
| BAC68125.1 | modular\_polyketide\_synthase | BGC0000059 | Polyketide | 33.0 | 51.7 | 398.0 | 5.56e-112 |
| AKL64831.1 | polyketide\_synthase | BGC0002072 | Polyketide:Modular type I polyketide | 31.0 | 57.6 | 398.0 | 5.58e-112 |
| AQH32483.1 | hybrid\_peptide\_synthetase/polyketide\_synthase | BGC0001667 | NRP+Polyketide | 31.0 | 55.0 | 397.0 | 5.62e-112 |
| AKJ15895.1 | modular\_polyketide\_synthase | BGC0002735 | Polyketide+NRP | 32.0 | 53.3 | 383.0 | 5.64e-112 |
| ARM20284.1 | polyketide\_synthase | BGC0001523 | Polyketide | 31.0 | 57.8 | 397.0 | 5.79e-112 |
| AWR88399.1 | putative\_beta-ketoacyl\_synthase | BGC0001522 | Polyketide | 31.0 | 59.4 | 398.0 | 5.93e-112 |
| QBF51756.1 | type\_I\_polyketide\_synthase | BGC0001856 | Polyketide:Modular type I polyketide | 32.0 | 54.1 | 398.0 | 6.27e-112 |
| QBF51754.1 | type\_I\_polyketide\_synthase | BGC0001856 | Polyketide:Modular type I polyketide | 32.0 | 54.6 | 398.0 | 6.55e-112 |
| AAP42873.1 | NanA11 | BGC0000105 | Polyketide | 32.0 | 57.4 | 397.0 | 6.66e-112 |
| ANR02553.1 | LodL | BGC0001648 | Polyketide | 32.0 | 53.4 | 397.0 | 6.77e-112 |
| AGM05534.1 | modular\_polyketide\_synthase | BGC0002098 | Polyketide | 30.0 | 56.7 | 397.0 | 7.19e-112 |
| ADM46356.1 | polyketide\_synthase | BGC0000106 | Polyketide | 31.0 | 54.2 | 398.0 | 7.56e-112 |
| ADM46359.1 | polyketide\_synthase | BGC0000106 | Polyketide | 32.0 | 57.6 | 397.0 | 7.84e-112 |
| AFV30249.1 | polyketide\_synthase | BGC0000075 | Polyketide | 31.0 | 61.6 | 395.0 | 7.87e-112 |
| ABI94380.1 | tautomycetin\_biosynthetic\_PKS | BGC0000157 | Polyketide | 34.0 | 49.1 | 398.0 | 8.28e-112 |
| WP\_033261453.1 | type\_I\_polyketide\_synthase | BGC0002009 | Polyketide | 32.0 | 57.6 | 395.0 | 8.43e-112 |
| QNN81303.1 | IonAVII | BGC0002446 | Polyketide | 29.0 | 61.9 | 397.0 | 8.59e-112 |
| TMU97090.1 | SDR\_family\_NAD(P)-dependent\_oxidoreductase | BGC0002038 | Polyketide | 31.0 | 58.6 | 395.0 | 8.6e-112 |
| AWW87424.1 | type\_I\_polyketide\_synthase | BGC0001755 | Polyketide | 32.0 | 55.9 | 398.0 | 8.71e-112 |
| QOV09193.1 | ClyE/NocP | BGC0002597 | NRP+Polyketide | 35.0 | 40.5 | 380.0 | 9.49e-112 |
| AGC09484.1 | LobS1 | BGC0001183 | Polyketide | 32.0 | 54.1 | 397.0 | 1.04e-111 |
| AHN85651.1 | Phn2 | BGC0000122 | Polyketide:Modular type I polyketide | 31.0 | 52.8 | 397.0 | 1.09e-111 |
| AHB82070.1 | polyketide\_synthase | BGC0001231 | NRP+Polyketide:Modular type I polyketide | 31.0 | 55.1 | 391.0 | 1.15e-111 |
| ABO15888.1 | polyketide\_synthase | BGC0000132 | Polyketide | 33.0 | 52.7 | 395.0 | 1.25e-111 |
| QES95476.1 | type\_I\_polyketide\_synthase | BGC0002453 | Polyketide | 30.0 | 57.9 | 394.0 | 1.3e-111 |
| ARW71487.1 | type\_I\_PKS\_module\_7 | BGC0001812 | Polyketide | 33.0 | 53.3 | 394.0 | 1.32e-111 |
| QGA70079.1 | type\_I\_polyketide\_synthase | BGC0002517 | Polyketide | 32.0 | 57.8 | 397.0 | 1.37e-111 |
| CAO85896.1 | protein\_modular\_polyketide\_synthase\_NorA' | BGC0000110 | Polyketide:Modular type I polyketide | 31.0 | 60.0 | 397.0 | 1.38e-111 |
| AXG22405.1 | type\_I\_polyketide\_synthase | BGC0002024 | Polyketide | 33.0 | 53.6 | 397.0 | 1.39e-111 |
| AKA59091.1 | type-I\_PKS | BGC0001619 | Polyketide | 31.0 | 57.2 | 397.0 | 1.39e-111 |
| QKV49765.1 | PKS | BGC0002526 | Polyketide | 32.0 | 54.7 | 396.0 | 1.45e-111 |
| ANZ22995.1 | ZinA | BGC0001828 | Polyketide | 33.0 | 53.8 | 396.0 | 1.61e-111 |
| AZH23793.1 | MgcK | BGC0001970 | NRP+Polyketide | 30.0 | 53.7 | 386.0 | 1.68e-111 |
| AAQ82565.1 | FscB | BGC0000034 | NRP+Polyketide | 32.0 | 54.1 | 397.0 | 1.74e-111 |
| AAF00958.1 | mcyE | BGC0001017 | NRP+Polyketide:Modular type I polyketide | 30.0 | 53.1 | 396.0 | 1.77e-111 |
| ABV97155.1 | Acyl\_transferase | BGC0000137 | Polyketide | 31.0 | 56.5 | 396.0 | 1.77e-111 |
| QSV12662.1 | AvmD | BGC0002456 | Polyketide+NRP | 30.0 | 65.4 | 397.0 | 1.93e-111 |
| AAM70355.1 | CalO5 | BGC0000033 | Polyketide | 31.0 | 56.8 | 387.0 | 1.96e-111 |
| APZ78844.1 | polyketide\_synthase | BGC0001431 | NRP:Cyclic depsipeptide+Polyketide:Iterative type I polyketide | 31.0 | 57.1 | 395.0 | 2e-111 |
| WP\_035122279.1 | type\_I\_polyketide\_synthase | BGC0001467 | NRP:Cyclic depsipeptide+Polyketide:Modular type I polyketide | 31.0 | 54.9 | 394.0 | 2.12e-111 |
| QES95477.1 | type\_I\_polyketide\_synthase | BGC0002453 | Polyketide | 31.0 | 53.6 | 383.0 | 2.21e-111 |
| BBA66511.1 | type\_I\_polyketide\_synthase | BGC0001495 | Polyketide | 33.0 | 52.3 | 396.0 | 2.28e-111 |
| BAE93729.1 | type\_I\_polyketide\_synthase | BGC0000164 | Polyketide | 31.0 | 54.6 | 396.0 | 2.3e-111 |
| CQR60497.1 | Polyketide\_synthase,\_type\_I,\_modules:\_loading,\_1,\_2\_and\_3 | BGC0001287 | Polyketide | 32.0 | 54.2 | 396.0 | 2.32e-111 |
| BBA66512.1 | type\_I\_polyketide\_synthase | BGC0001495 | Polyketide | 33.0 | 53.1 | 396.0 | 2.43e-111 |
| AHH99919.1 | PKS\_I | BGC0000002 | Polyketide | 29.0 | 60.3 | 396.0 | 2.46e-111 |
| WP\_245661588.1 | hypothetical\_protein | BGC0001348 | Polyketide:Modular type I polyketide | 32.0 | 58.2 | 396.0 | 2.54e-111 |
| CAD19085.1 | StiA\_protein | BGC0000153 | NRP+Polyketide:Modular type I polyketide | 32.0 | 55.3 | 395.0 | 2.54e-111 |
| APZ78854.1 | polyketide\_synthase | BGC0001432 | NRP:Cyclic depsipeptide+Polyketide:Iterative type I polyketide | 31.0 | 57.7 | 395.0 | 2.65e-111 |
| APZ78714.1 | polyketide\_synthase | BGC0001420 | NRP:Cyclic depsipeptide+Polyketide:Iterative type I polyketide | 31.0 | 57.0 | 395.0 | 2.65e-111 |
| AAK83194.1 | polyketide\_synthase | BGC0000026 | Saccharide:Oligosaccharide | 31.0 | 58.6 | 387.0 | 2.72e-111 |
| WP\_083502114.1 | type\_I\_polyketide\_synthase | BGC0001653 | Polyketide | 33.0 | 53.2 | 395.0 | 2.78e-111 |
| QNS30807.1 | hybrid\_non-ribosomal\_peptide\_synthetase/type\_I\_polyketide\_syn-thase | BGC0002509 | NRP | 31.0 | 61.5 | 395.0 | 2.85e-111 |
| CAE02602.1 | polyketide\_synthase\_type\_I | BGC0000024 | Polyketide:Modular type I polyketide | 30.0 | 56.7 | 394.0 | 3.16e-111 |
| CAJ88175.1 | Type\_I\_modular\_polyketide\_synthase | BGC0000151 | Polyketide:Modular type I polyketide+Saccharide:Hybrid/tailoring saccharide | 32.0 | 53.4 | 396.0 | 3.43e-111 |
| CAQ18839.1 | hybrid\_polyketide\_synthase/nonribosomal\_polypetide\_synthetase | BGC0000954 | NRP+Polyketide:Modular type I polyketide | 31.0 | 54.7 | 395.0 | 3.73e-111 |
| AAG23263.1 | polyketide\_synthase\_extender\_modules\_5-7 | BGC0000148 | Polyketide | 31.0 | 57.8 | 395.0 | 3.84e-111 |
| ACN69990.1 | polyketide\_synthase | BGC0000079 | Polyketide | 30.0 | 57.5 | 395.0 | 3.94e-111 |
| ACO94496.1 | polyketide\_synthase\_type\_I | BGC0000097 | Polyketide:Modular type I polyketide | 31.0 | 57.4 | 395.0 | 4e-111 |
| AUO16399.1 | polyketide\_synthase | BGC0001700 | Polyketide | 29.0 | 60.8 | 395.0 | 4e-111 |
| ABB05104.1 | LipPks3 | BGC0001003 | NRP:Lipopeptide+Polyketide:Modular type I polyketide+Saccharide:Hybrid/tailoring saccharide | 30.0 | 59.5 | 395.0 | 4.23e-111 |
| CAO85897.1 | modular\_polyketide\_synthase\_NorB | BGC0000110 | Polyketide:Modular type I polyketide | 30.0 | 59.8 | 394.0 | 4.72e-111 |
| QBG82517.1 | Polyketide\_synthase | BGC0002587 | Polyketide | 30.0 | 60.3 | 394.0 | 4.84e-111 |
| ABK32287.1 | JerA | BGC0000080 | Polyketide | 30.0 | 54.9 | 381.0 | 4.91e-111 |
| AVX51100.1 | nysK | BGC0001709 | Polyketide | 32.0 | 54.4 | 394.0 | 5.02e-111 |
| WP\_053065267.1 | type\_I\_polyketide\_synthase | BGC0001330 | NRP:Cyclic depsipeptide+Polyketide:Modular type I polyketide | 32.0 | 52.8 | 394.0 | 5.2e-111 |
| AEP40936.1 | polyketide\_synthase\_type\_I | BGC0000021 | Polyketide | 31.0 | 57.2 | 395.0 | 5.24e-111 |
| AAP42874.1 | NanA8 | BGC0000105 | Polyketide | 30.0 | 59.1 | 395.0 | 5.41e-111 |
| AAO65806.1 | monensin\_polyketide\_synthase\_modules\_11\_and\_12 | BGC0000100 | Polyketide | 30.0 | 60.2 | 395.0 | 5.77e-111 |
| ANZ52469.1 | MonAVIII | BGC0001670 | Polyketide | 30.0 | 60.2 | 395.0 | 5.77e-111 |
| ASZ00147.1 | polyketide\_synthase | BGC0001785 | Polyketide | 33.0 | 53.0 | 395.0 | 7.1e-111 |
| EPH46606.1 | putative\_Phenolphthiocerol\_synthesis\_polyketide\_synthase\_type\_I\_Pks15/1 | BGC0001519 | NRP+Polyketide | 30.0 | 61.8 | 392.0 | 7.27e-111 |
| AFI57005.1 | QmnA1 | BGC0000133 | Polyketide | 31.0 | 56.8 | 395.0 | 7.27e-111 |
| AAD03047.1 | type\_I\_polyketide\_synthase | BGC0000041 | Polyketide | 30.0 | 56.8 | 394.0 | 7.43e-111 |
| ACF35445.1 | mbcAI | BGC0000090 | Polyketide | 32.0 | 57.6 | 395.0 | 7.6e-111 |
| AAP85335.1 | type\_I\_PKS | BGC0000233 | Polyketide | 31.0 | 56.8 | 393.0 | 7.67e-111 |
| QEA08889.1 | JenA3 | BGC0002559 | Polyketide | 29.0 | 61.9 | 394.0 | 7.97e-111 |
| APZ78690.1 | polyketide\_synthase | BGC0001418 | NRP:Cyclic depsipeptide+Polyketide:Iterative type I polyketide | 31.0 | 57.0 | 393.0 | 8.21e-111 |
| AAO65796.1 | monensin\_polyketide\_synthase\_loading\_module\_and\_module\_1 | BGC0000100 | Polyketide | 31.0 | 57.6 | 394.0 | 8.39e-111 |
| ANZ52459.1 | MonAI | BGC0001670 | Polyketide | 31.0 | 57.6 | 394.0 | 8.39e-111 |
| QFU19838.1 | PKS | BGC0002431 | Polyketide+Saccharide | 32.0 | 54.7 | 390.0 | 8.49e-111 |
| BCK51645.1 | modular\_polyketide\_synthase | BGC0002520 | Polyketide | 31.0 | 53.3 | 394.0 | 8.74e-111 |
| ABC84457.1 | NigAII | BGC0000114 | Polyketide:Modular type I polyketide | 30.0 | 58.2 | 393.0 | 9e-111 |
| WP\_048832936.1 | polyketide\_synthase | BGC0001348 | Polyketide:Modular type I polyketide | 31.0 | 58.7 | 394.0 | 9.08e-111 |
| BAK64638.1 | polyketide\_synthase | BGC0000135 | Polyketide | 30.0 | 62.8 | 394.0 | 1.01e-110 |
| AAQ82561.1 | FscA | BGC0000034 | NRP+Polyketide | 31.0 | 60.8 | 391.0 | 1.05e-110 |
| QBF51769.1 | type\_I\_polyketide\_synthase | BGC0001856 | Polyketide:Modular type I polyketide | 31.0 | 61.7 | 393.0 | 1.05e-110 |
| QGA70098.1 | type\_I\_polyketide\_synthase | BGC0002517 | Polyketide | 33.0 | 52.9 | 385.0 | 1.07e-110 |
| CAG28678.1 | polyketide\_synthase | BGC0001023 | NRP+Polyketide:Modular type I polyketide | 31.0 | 57.6 | 393.0 | 1.09e-110 |
| APZ78820.1 | polyketide\_synthase | BGC0001429 | NRP:Cyclic depsipeptide+Polyketide:Iterative type I polyketide | 31.0 | 57.6 | 393.0 | 1.09e-110 |
| TXD00034.1 | SDR\_family\_NAD(P)-dependent\_oxidoreductase | BGC0001877 | Polyketide | 31.0 | 58.1 | 394.0 | 1.09e-110 |
| ctg1\_orf30 |  | BGC0000096 | Polyketide | 33.0 | 53.9 | 389.0 | 1.19e-110 |
| WP\_019032757.1 | type\_I\_polyketide\_synthase | BGC0001331 | NRP:Cyclic depsipeptide+Polyketide:Modular type I polyketide | 31.0 | 52.9 | 393.0 | 1.22e-110 |
| ATP76242.1 | NdaC | BGC0001705 | NRP+Polyketide | 31.0 | 54.4 | 393.0 | 1.25e-110 |
| CAN89636.1 | putative\_polyketide\_synthase | BGC0001070 | NRP+Polyketide:Modular type I polyketide+Polyketide:Trans-AT type I polyketide | 32.0 | 53.8 | 393.0 | 1.4e-110 |
| QSE03591.1 | LcmC | BGC0002333 | Polyketide | 33.0 | 53.2 | 394.0 | 1.44e-110 |
| QBG82528.1 | Polyketide\_synthase | BGC0002587 | Polyketide | 32.0 | 53.6 | 394.0 | 1.46e-110 |
| QIQ28638.1 | Nbc42 | BGC0002541 | Other | 32.0 | 54.4 | 393.0 | 1.5e-110 |
| QRI43526.1 | type\_I\_polyketide\_synthase | BGC0002454 | Polyketide | 31.0 | 61.3 | 393.0 | 1.62e-110 |
| CAL58682.1 | polyketide\_synthase | BGC0000149 | Polyketide:Modular type I polyketide | 30.0 | 57.2 | 393.0 | 1.65e-110 |
| ctg1\_orf15 |  | BGC0001457 | NRP | 33.0 | 50.3 | 392.0 | 1.69e-110 |
| AEW95639.1 | type\_I\_polyketide\_synthase | BGC0002697 | NRP+Polyketide | 30.0 | 61.7 | 394.0 | 1.69e-110 |
| ALV82345.1 | borrelidin\_type\_I\_polyketide\_synthase | BGC0001533 | Polyketide | 31.0 | 56.5 | 392.0 | 1.81e-110 |
| UHY14129.1 | PKS\_I | BGC0002671 | Polyketide | 31.0 | 57.6 | 393.0 | 1.98e-110 |
| BAF02926.1 | type\_I\_polyketide\_synthase | BGC0000073 | Polyketide | 30.0 | 58.7 | 393.0 | 2.13e-110 |
| QHZ99321.1 | polyketide\_synthaase | BGC0001875 | Polyketide | 32.0 | 57.2 | 393.0 | 2.3e-110 |
| QBL56181.1 | PKS | BGC0002376 | Polyketide | 30.0 | 61.0 | 393.0 | 2.34e-110 |
| ABC87510.1 | polyketide\_synthase | BGC0001011 | NRP+Polyketide | 30.0 | 58.4 | 393.0 | 2.37e-110 |
| ctg1\_orf21 |  | BGC0001013 | NRP+Polyketide | 30.0 | 58.4 | 393.0 | 2.37e-110 |
| WP\_240490790.1 | type\_I\_polyketide\_synthase | BGC0002009 | Polyketide | 30.0 | 62.0 | 390.0 | 2.37e-110 |
| QRI43529.1 | type\_I\_polyketide\_synthase | BGC0002454 | Polyketide | 32.0 | 54.3 | 393.0 | 2.88e-110 |
| AHH99926.1 | PKS\_I | BGC0000002 | Polyketide | 30.0 | 62.0 | 393.0 | 2.88e-110 |
| QTT72113.1 | type\_I\_polyketide\_synthase | BGC0002350 | NRP+Polyketide+Saccharide | 30.0 | 59.5 | 392.0 | 2.92e-110 |
| AWH12670.1 | RmpA2 | BGC0001759 | Polyketide | 31.0 | 53.1 | 388.0 | 2.94e-110 |
| ADC45538.1 | modular\_polyketide\_synthase | BGC0000093 | Polyketide | 30.0 | 57.8 | 393.0 | 2.94e-110 |
| BAB69192.1 | modular\_polyketide\_synthase | BGC0000117 | Polyketide | 32.0 | 52.4 | 393.0 | 3e-110 |
| ctg1\_orf29 |  | BGC0000096 | Polyketide | 30.0 | 61.7 | 392.0 | 3.08e-110 |
| PAU45552.1 | Iterative\_polyketide\_synthase | BGC0002138 | Polyketide | 29.0 | 60.8 | 384.0 | 3.19e-110 |
| CAQ34918.1 | nonribosomal\_peptide\_synthetase/\_polyketide\_synthase | BGC0000986 | NRP+Polyketide | 28.0 | 73.1 | 392.0 | 3.35e-110 |
| APZ78702.1 | polyketide\_synthase | BGC0001419 | NRP:Cyclic depsipeptide+Polyketide:Iterative type I polyketide | 31.0 | 57.0 | 391.0 | 3.37e-110 |
| AFV30250.1 | polyketide\_synthase | BGC0000075 | Polyketide | 30.0 | 58.6 | 391.0 | 3.5e-110 |
| ABV83229.1 | CppB | BGC0000116 | Polyketide | 32.0 | 53.7 | 392.0 | 3.59e-110 |
| CCP20048.1 | divL1\_protein | BGC0001119 | Polyketide:Modular type I polyketide | 30.0 | 60.1 | 391.0 | 3.59e-110 |
| AHB82051.1 | polyketide\_synthase | BGC0001019 | NRP+Polyketide:Modular type I polyketide | 30.0 | 61.4 | 392.0 | 3.7e-110 |
| CBD77732.1 | polyketide\_synthase | BGC0000974 | NRP+Polyketide | 31.0 | 53.6 | 392.0 | 3.72e-110 |
| QKV49766.1 | PKS | BGC0002526 | Polyketide | 30.0 | 54.0 | 391.0 | 3.72e-110 |
| QCQ67875.1 | hybrid\_peptide\_synthetase/polyketide\_synthase | BGC0002297 | NRP+Polyketide | 29.0 | 54.1 | 392.0 | 3.9e-110 |
| ACB46487.1 | polyketide\_synthase | BGC0000082 | Polyketide | 32.0 | 57.5 | 392.0 | 4.12e-110 |
| BAF85837.1 | modular\_polyketide\_synthase | BGC0000109 | Polyketide | 32.0 | 57.2 | 391.0 | 4.12e-110 |
| AFV30251.1 | polyketide\_synthase | BGC0000075 | Polyketide | 31.0 | 60.9 | 392.0 | 4.22e-110 |
| AKA59093.1 | type-I\_PKS | BGC0001619 | Polyketide | 31.0 | 59.0 | 392.0 | 4.28e-110 |
| QKG20136.1 | type\_I\_polyketide\_synthase | BGC0002124 | Polyketide | 30.0 | 61.1 | 392.0 | 4.29e-110 |
| ANR02554.1 | LodM | BGC0001648 | Polyketide | 31.0 | 57.3 | 392.0 | 4.34e-110 |
| ALV82341.1 | borrelidin\_type\_I\_polyketide\_synthase | BGC0001533 | Polyketide | 32.0 | 55.5 | 388.0 | 4.48e-110 |
| AKL64832.1 | polyketide\_synthase | BGC0002072 | Polyketide:Modular type I polyketide | 31.0 | 53.5 | 388.0 | 4.93e-110 |
| BAF02923.1 | type\_I\_polyketide\_synthase | BGC0000073 | Polyketide | 30.0 | 67.9 | 392.0 | 4.94e-110 |
| ctg1\_orf20 |  | BGC0001013 | NRP+Polyketide | 32.0 | 53.1 | 392.0 | 5.12e-110 |
| ABC87509.1 | polyketide\_synthase | BGC0001011 | NRP+Polyketide | 32.0 | 53.1 | 392.0 | 5.13e-110 |
| CAE45671.1 | borrelidin\_polyketide\_synthase,\_type\_I | BGC0000031 | Polyketide:Modular type I polyketide | 31.0 | 56.5 | 390.0 | 5.83e-110 |
| AUA09467.1 | Erythronolide\_synthase,\_modules\_1\_and\_2 | BGC0002291 | Polyketide | 32.0 | 53.1 | 392.0 | 6.07e-110 |
| ABB88521.1 | polyketide\_synthase\_type\_I | BGC0000050 | Polyketide | 32.0 | 53.8 | 390.0 | 6.08e-110 |
| ctg1\_orf524 |  | BGC0001199 | Polyketide | 32.0 | 53.0 | 383.0 | 6.13e-110 |
| ARM20281.1 | polyketide\_synthase | BGC0001523 | Polyketide | 31.0 | 53.1 | 391.0 | 6.35e-110 |
| WP\_055480220.1 | type\_I\_polyketide\_synthase | BGC0001653 | Polyketide | 31.0 | 57.3 | 391.0 | 6.9e-110 |
| AIT55262.1 | polyketide\_synthase | BGC0000072 | Polyketide:Modular type I polyketide | 32.0 | 53.5 | 379.0 | 7.11e-110 |
| QRI43530.1 | type\_I\_polyketide\_synthase | BGC0002454 | Polyketide | 31.0 | 54.1 | 391.0 | 7.22e-110 |
| AGM05531.1 | type\_I\_polyketide\_synthase | BGC0002098 | Polyketide | 31.0 | 57.4 | 391.0 | 7.34e-110 |
| APZ78678.1 | polyketide\_synthase | BGC0001417 | NRP:Cyclic depsipeptide+Polyketide:Iterative type I polyketide | 31.0 | 57.1 | 390.0 | 7.85e-110 |
| SCO70310.1 | Type\_I\_polyketide\_synthase | BGC0001433 | Polyketide:Modular type I polyketide | 31.0 | 52.9 | 390.0 | 8.26e-110 |
| BCB17028.1 | modular\_polyketide\_synthase | BGC0002523 | NRP | 32.0 | 53.1 | 391.0 | 8.57e-110 |
| RLV64599.1 | Erythronolide\_synthase,\_modules\_1\_and\_2 | BGC0001845 | Polyketide+NRP+Other:Aminocoumarin | 32.0 | 54.9 | 390.0 | 9.63e-110 |
| EHK80163.1 | acyl\_transferase | BGC0001447 | Polyketide | 31.0 | 53.2 | 391.0 | 1.14e-109 |
| ABV97151.1 | AMP-dependent\_synthetase\_and\_ligase | BGC0000137 | Polyketide | 32.0 | 52.9 | 391.0 | 1.14e-109 |
| ANH11409.1 | SceN | BGC0001770 | Polyketide | 31.0 | 56.8 | 390.0 | 1.24e-109 |
| BAJ16467.1 | polyketide\_synthase | BGC0000058 | Polyketide | 30.0 | 57.7 | 391.0 | 1.29e-109 |
| CAE45670.1 | borrelidin\_polyketide\_synthase,\_type\_I | BGC0000031 | Polyketide:Modular type I polyketide | 32.0 | 55.5 | 386.0 | 1.3e-109 |
| AKD43763.1 | HerG | BGC0001349 | NRP+Polyketide | 31.0 | 59.8 | 389.0 | 1.3e-109 |
| QES95478.1 | type\_I\_polyketide\_synthase | BGC0002453 | Polyketide | 32.0 | 58.1 | 388.0 | 1.37e-109 |
| QSV12663.1 | AvmE | BGC0002456 | Polyketide+NRP | 32.0 | 57.4 | 390.0 | 1.39e-109 |
| ANY10590.1 | polyketide\_synthase | BGC0001773 | Polyketide | 32.0 | 54.5 | 390.0 | 1.47e-109 |
| AKD43761.1 | HerD | BGC0001349 | NRP+Polyketide | 30.0 | 57.5 | 390.0 | 1.55e-109 |
| BAJ16468.1 | polyketide\_synthase | BGC0000058 | Polyketide | 31.0 | 57.9 | 390.0 | 1.56e-109 |
| DAB41918.1 | ArzP\_-\_PKS\_(KS,\_AT,\_OMT,\_ACP,\_TE) | BGC0001884 | NRP+Polyketide | 31.0 | 54.3 | 387.0 | 1.6e-109 |
| AEP40940.1 | polyketide\_synthase\_type\_I | BGC0000021 | Polyketide | 31.0 | 52.8 | 390.0 | 1.63e-109 |
| AAZ77694.1 | ChlA2 | BGC0000036 | Polyketide:Modular type I polyketide+Polyketide:Iterative type I polyketide+Saccharide:Oligosaccharide | 32.0 | 57.8 | 390.0 | 1.74e-109 |
| QEA08892.1 | JenA6 | BGC0002559 | Polyketide | 31.0 | 55.4 | 386.0 | 1.79e-109 |
| TXD00033.1 | SDR\_family\_NAD(P)-dependent\_oxidoreductase | BGC0001877 | Polyketide | 31.0 | 58.1 | 390.0 | 1.96e-109 |
| ACF35446.1 | mbcAII | BGC0000090 | Polyketide | 32.0 | 57.6 | 390.0 | 2.06e-109 |
| BAK64649.1 | polyketide\_synthase | BGC0000135 | Polyketide | 31.0 | 58.1 | 390.0 | 2.16e-109 |
| ABC87511.1 | polyketide\_synthase | BGC0001011 | NRP+Polyketide | 32.0 | 54.3 | 390.0 | 2.31e-109 |
| ctg1\_orf22 |  | BGC0001013 | NRP+Polyketide | 32.0 | 54.3 | 390.0 | 2.31e-109 |
| QFU19825.1 | PKS | BGC0002431 | Polyketide+Saccharide | 31.0 | 56.8 | 390.0 | 2.56e-109 |
| BAP34734.1 | type\_I\_polyketide\_synthase | BGC0000078 | Polyketide | 30.0 | 62.1 | 390.0 | 2.69e-109 |
| ANC94964.1 | AlmHIII | BGC0001396 | Polyketide | 30.0 | 59.6 | 389.0 | 2.91e-109 |
| ADH04641.1 | TgaC | BGC0001051 | NRP+Polyketide:Modular type I polyketide | 30.0 | 59.6 | 390.0 | 2.91e-109 |
| ACB37755.1 | putative\_type\_I\_polyketide\_synthase | BGC0000162 | Polyketide | 31.0 | 58.1 | 389.0 | 3.05e-109 |
| BAB69198.1 | modular\_polyketide\_synthase | BGC0000117 | Polyketide | 30.0 | 60.0 | 387.0 | 3.07e-109 |
| QBF51759.1 | type\_I\_polyketide\_synthase | BGC0001856 | Polyketide:Modular type I polyketide | 31.0 | 55.8 | 389.0 | 3.53e-109 |
| QBL56183.1 | PKS | BGC0002376 | Polyketide | 31.0 | 58.1 | 385.0 | 3.54e-109 |
| BAF85843.1 | modular\_polyketide\_synthase | BGC0000109 | Polyketide | 30.0 | 57.7 | 389.0 | 3.65e-109 |
| ANC94966.1 | AlmHI | BGC0001396 | Polyketide | 31.0 | 54.0 | 389.0 | 3.69e-109 |
| AUO16397.1 | polyketide\_synthase | BGC0001700 | Polyketide | 30.0 | 58.1 | 389.0 | 4.03e-109 |
| CAL58681.1 | polyketide\_synthase | BGC0000149 | Polyketide:Modular type I polyketide | 31.0 | 58.1 | 389.0 | 4.37e-109 |
| BAO66542.1 | type\_I\_polyketide\_synthase | BGC0000042 | Polyketide | 31.0 | 57.5 | 389.0 | 4.66e-109 |
| ARS01475.1 | NcmAIII | BGC0001702 | NRP+Polyketide | 31.0 | 52.4 | 384.0 | 4.74e-109 |
| AAO62585.1 | peptide\_sythetase\_polyketide\_synthase\_fusion\_protein | BGC0001016 | NRP+Polyketide | 31.0 | 54.1 | 388.0 | 4.78e-109 |
| ABV91287.1 | type\_I\_modular\_polyketide\_synthase | BGC0000158 | Polyketide:Modular type I polyketide | 34.0 | 49.0 | 389.0 | 5.28e-109 |
| ABP55493.1 | thioester\_reductase\_domain | BGC0001006 | NRP+Polyketide | 31.0 | 52.9 | 388.0 | 5.33e-109 |
| WP\_036342114.1 | type\_I\_polyketide\_synthase | BGC0001327 | NRP:Cyclic depsipeptide+Polyketide:Modular type I polyketide | 31.0 | 60.9 | 388.0 | 5.42e-109 |
| QCF28926.1 | type\_I\_polyketide\_synthase | BGC0002308 | Alkaloid+Polyketide | 31.0 | 56.9 | 387.0 | 5.84e-109 |
| WP\_033261452.1 | type\_I\_polyketide\_synthase | BGC0002009 | Polyketide | 30.0 | 61.4 | 388.0 | 6.4e-109 |
| ABW96541.1 | type\_I\_modular\_polyketide\_synthase | BGC0000159 | Polyketide:Modular type I polyketide | 31.0 | 54.0 | 389.0 | 6.4e-109 |
| CAD29794.1 | peptide\_synthetase | BGC0001015 | NRP+Polyketide | 30.0 | 52.3 | 388.0 | 6.46e-109 |
| BAO66519.1 | type\_I\_polyketide\_synthase | BGC0000042 | Polyketide | 31.0 | 61.9 | 388.0 | 6.6e-109 |
| BAH02269.1 | polyketide\_synthase | BGC0000126 | Polyketide | 30.0 | 62.2 | 389.0 | 6.85e-109 |
| ABV99085.1 | thioester\_reductase\_domain | BGC0001007 | Polyketide+NRP | 32.0 | 54.5 | 387.0 | 7.05e-109 |
| ABV97153.1 | Beta-ketoacyl\_synthase | BGC0000137 | Polyketide | 30.0 | 61.1 | 386.0 | 7.08e-109 |
| AWS21278.1 | type\_I\_polyketide\_synthase | BGC0001934 | Polyketide | 29.0 | 60.8 | 388.0 | 7.29e-109 |
| AZY91987.1 | polyketide\_synthase | BGC0002022 | Polyketide | 29.0 | 60.8 | 388.0 | 7.29e-109 |
| BAC57029.1 | protomycinolide\_IV\_synthase\_2 | BGC0000102 | Polyketide | 32.0 | 57.9 | 386.0 | 7.47e-109 |
| AGI99494.1 | Type\_I\_polyketide\_synthase | BGC0001004 | Polyketide:Modular type I polyketide | 30.0 | 55.4 | 384.0 | 7.52e-109 |
| QFU80898.1 | PKS | BGC0002550 | Polyketide | 30.0 | 55.4 | 384.0 | 7.52e-109 |
| CAE02606.1 | polyketide\_synthase\_type\_I | BGC0000024 | Polyketide:Modular type I polyketide | 31.0 | 52.8 | 387.0 | 7.84e-109 |
| AVX51107.1 | nysB | BGC0001709 | Polyketide | 31.0 | 53.8 | 388.0 | 7.92e-109 |
| AGM05535.1 | modular\_polyketide\_synthase | BGC0002098 | Polyketide | 30.0 | 57.6 | 388.0 | 8.1e-109 |
| BCB17030.1 | modular\_polyketide\_synthase | BGC0002523 | NRP | 30.0 | 58.8 | 388.0 | 8.22e-109 |
| OAP25821.1 | Phenolphthiocerol\_synthesis\_polyketide\_synthase\_type\_I\_Pks15/1 | BGC0001658 | Polyketide | 30.0 | 60.8 | 388.0 | 8.49e-109 |
| AEP40934.1 | polyketide\_synthase\_type\_I | BGC0000021 | Polyketide | 30.0 | 57.7 | 388.0 | 8.58e-109 |
| BAB69196.1 | modular\_polyketide\_synthase | BGC0000117 | Polyketide | 30.0 | 57.6 | 388.0 | 8.79e-109 |
| AAC01712.2 | RifC | BGC0000136 | Polyketide | 31.0 | 57.5 | 385.0 | 8.96e-109 |
| BAJ16471.1 | polyketide\_synthase | BGC0000058 | Polyketide | 31.0 | 58.5 | 388.0 | 9.11e-109 |
| BAB69194.1 | modular\_polyketide\_synthase | BGC0000117 | Polyketide | 31.0 | 61.3 | 388.0 | 9.34e-109 |
| OJF16266.1 | AceP4 | BGC0001491 | Polyketide | 32.0 | 53.8 | 388.0 | 9.63e-109 |
| AXG22406.1 | type\_I\_polyketide\_synthase | BGC0002024 | Polyketide | 30.0 | 54.1 | 388.0 | 1.04e-108 |
| AAW03327.1 | CtaD | BGC0000982 | NRP+Polyketide | 30.0 | 57.7 | 387.0 | 1.08e-108 |
| BAF85844.1 | modular\_polyketide\_synthase | BGC0000109 | Polyketide | 31.0 | 55.9 | 388.0 | 1.08e-108 |
| BBM96640.1 | modular\_polyketide\_synthase | BGC0002452 | Polyketide | 30.0 | 61.6 | 386.0 | 1.13e-108 |
| AVV61984.1 | type\_I\_modular\_polyketide\_synthase | BGC0001477 | NRP+Polyketide:Modular type I polyketide | 32.0 | 53.6 | 387.0 | 1.17e-108 |
| AFL48532.1 | laidlomycin\_polyketide\_synthase\_(module\_11\_and\_module\_12) | BGC0000084 | Polyketide | 30.0 | 60.2 | 387.0 | 1.17e-108 |
| AKG06377.1 | polyketide\_synthase\_type\_1 | BGC0001830 | Polyketide | 30.0 | 60.6 | 387.0 | 1.19e-108 |
| QIQ28634.1 | Nbc38 | BGC0002541 | Other | 31.0 | 54.4 | 387.0 | 1.19e-108 |
| AFY58524.1 | beta-ketoacyl\_synthase\_family\_protein,acyltransferase\_family\_protein,phosphopantetheine-containing\_protein | BGC0002411 | NRP+Polyketide | 34.0 | 39.2 | 378.0 | 1.66e-108 |
| ASZ00150.1 | polyketide\_synthase | BGC0001785 | Polyketide | 31.0 | 57.3 | 385.0 | 1.84e-108 |
| CQR60493.1 | Polyketide\_synthase,\_type\_I,\_modules:\_9\_and\_10 | BGC0001287 | Polyketide | 31.0 | 57.7 | 387.0 | 1.98e-108 |
| ARV85760.1 | PieA1\_type\_I\_PKS | BGC0001742 | Polyketide | 29.0 | 60.6 | 386.0 | 1.99e-108 |
| AGY62755.1 | EbeC | BGC0000051 | Polyketide | 32.0 | 53.2 | 382.0 | 2.02e-108 |
| ADC79620.1 | BafAV | BGC0000028 | Polyketide:Modular type I polyketide | 31.0 | 57.9 | 385.0 | 2.09e-108 |
| ACB46485.1 | polyketide\_synthase | BGC0000082 | Polyketide | 31.0 | 56.2 | 381.0 | 2.19e-108 |
| APZ78780.1 | polyketide\_synthase | BGC0001426 | NRP:Cyclic depsipeptide+Polyketide:Iterative type I polyketide | 30.0 | 57.0 | 385.0 | 2.26e-108 |
| AAF71775.1 | nysB | BGC0000115 | Polyketide:Modular type I polyketide+Saccharide:Hybrid/tailoring saccharide | 31.0 | 53.5 | 386.0 | 2.43e-108 |
| AAO65797.1 | monensin\_polyketide\_synthase\_module\_2 | BGC0000100 | Polyketide | 30.0 | 57.6 | 385.0 | 2.52e-108 |
| ANZ52460.1 | MonAII | BGC0001670 | Polyketide | 30.0 | 57.6 | 385.0 | 2.52e-108 |
| UHY14127.1 | PKS\_I | BGC0002671 | Polyketide | 30.0 | 61.0 | 386.0 | 2.54e-108 |
| AAC46026.1 | polyketide\_synthase\_modules\_4\_and\_5 | BGC0000113 | Polyketide | 30.0 | 62.3 | 386.0 | 2.72e-108 |
| ABK32291.1 | JerE | BGC0000080 | Polyketide | 31.0 | 52.8 | 386.0 | 2.88e-108 |
| QGU18619.1 | polyketide\_synthase/non-ribosomal\_peptide\_synthetase | BGC0002365 | Other+Polyketide | 31.0 | 57.3 | 386.0 | 3.16e-108 |
| QIZ24104.1 | type\_I\_polyketide\_synthase | BGC0002540 | Polyketide | 30.0 | 61.8 | 383.0 | 3.23e-108 |
| BAK64637.1 | polyketide\_synthase | BGC0000135 | Polyketide | 32.0 | 54.4 | 386.0 | 3.32e-108 |
| BAF02924.1 | type\_I\_polyketide\_synthase | BGC0000073 | Polyketide | 31.0 | 53.5 | 386.0 | 3.35e-108 |
| WP\_234353270.1 | SDR\_family\_NAD(P)-dependent\_oxidoreductase | BGC0001537 | Polyketide | 31.0 | 60.9 | 386.0 | 3.52e-108 |
| QPP46758.1 | polyketide\_synthase | BGC0002500 | Polyketide | 30.0 | 59.6 | 386.0 | 3.55e-108 |
| AGC09487.1 | LobS5 | BGC0001183 | Polyketide | 30.0 | 55.2 | 382.0 | 3.74e-108 |
| TXD00261.1 | AMP-binding\_protein | BGC0001877 | Polyketide | 30.0 | 61.4 | 385.0 | 3.83e-108 |
| EHK80171.1 | modular\_polyketide\_synthase | BGC0001447 | Polyketide | 31.0 | 56.0 | 384.0 | 3.89e-108 |
| AGC24271.1 | prlQ | BGC0001038 | NRP+Polyketide:Modular type I polyketide | 31.0 | 53.0 | 377.0 | 3.92e-108 |
| AWH12937.1 | StmB | BGC0001784 | Polyketide | 30.0 | 61.4 | 386.0 | 4.22e-108 |
| AEC13070.1 | fosD | BGC0000060 | Polyketide | 32.0 | 52.9 | 383.0 | 4.38e-108 |
| AKJ15835.1 | type\_I\_polyketide\_synthase | BGC0002735 | Polyketide+NRP | 31.0 | 57.0 | 384.0 | 4.42e-108 |
| QPP46750.1 | polyketide\_synthase | BGC0002500 | Polyketide | 31.0 | 57.7 | 385.0 | 5.42e-108 |
| WP\_226048588.1 | AMP-binding\_protein | BGC0002106 | Polyketide | 31.0 | 53.7 | 382.0 | 5.52e-108 |
| AKL64830.1 | polyketide\_synthase | BGC0002072 | Polyketide:Modular type I polyketide | 31.0 | 54.1 | 385.0 | 5.65e-108 |
| AWW87422.1 | type\_I\_polyketide\_synthase | BGC0001755 | Polyketide | 30.0 | 62.0 | 385.0 | 5.71e-108 |
| AWH12669.1 | RmpB | BGC0001759 | Polyketide | 30.0 | 61.0 | 385.0 | 5.72e-108 |
| CQR60496.1 | Polyketide\_synthase,\_type\_I,\_modules:\_4,\_5\_and\_6 | BGC0001287 | Polyketide | 30.0 | 57.4 | 385.0 | 5.88e-108 |
| AFP87524.1 | type\_I\_polyketide\_synthase | BGC0001159 | NRP+Polyketide:Modular type I polyketide | 32.0 | 53.7 | 384.0 | 5.92e-108 |
| ACO94471.1 | polyketide\_synthase\_type\_I | BGC0000029 | Polyketide:Modular type I polyketide | 30.0 | 57.7 | 385.0 | 5.93e-108 |
| ABI91470.1 | beta-ketoacyl\_synthase | BGC0001094 | NRP+Polyketide | 31.0 | 57.6 | 385.0 | 6e-108 |
| CBA11583.1 | polyketide\_synthase\_type\_I | BGC0001046 | NRP+Polyketide:Modular type I polyketide+Saccharide:Hybrid/tailoring saccharide | 31.0 | 53.5 | 385.0 | 6.07e-108 |
| AAX98192.1 | polyketide\_synthase\_type\_I | BGC0000052 | Polyketide | 29.0 | 59.0 | 385.0 | 6.57e-108 |
| sipP4 | Type\_I\_Modular\_PKS | BGC0001452 | Polyketide | 32.0 | 53.5 | 381.0 | 6.65e-108 |
| ACY06289.1 | type\_I\_polyketide\_synthase | BGC0001042 | NRP+Polyketide | 32.0 | 53.0 | 385.0 | 6.73e-108 |
| BAP34740.1 | type\_I\_polyketide\_synthase | BGC0000078 | Polyketide | 31.0 | 59.3 | 382.0 | 7.15e-108 |
| BAG17643.1 | putative\_NRPS-type-I\_PKS\_fusion\_protein | BGC0001043 | NRP+Polyketide | 32.0 | 57.7 | 385.0 | 7.38e-108 |
| ANZ22987.1 | ZinD | BGC0001828 | Polyketide | 32.0 | 53.5 | 385.0 | 7.45e-108 |
| AWR88398.1 | putative\_beta-ketoacyl\_synthase | BGC0001522 | Polyketide | 32.0 | 54.0 | 381.0 | 7.54e-108 |
| UMP03508.1 | NmvAIII | BGC0002649 | NRP+Polyketide | 31.0 | 52.7 | 380.0 | 8.4e-108 |
| ACY06287.1 | type\_I\_polyketide\_synthase | BGC0001042 | NRP+Polyketide | 33.0 | 53.5 | 385.0 | 9.15e-108 |
| ALA09354.1 | type\_I\_modular\_PKS | BGC0001303 | Polyketide | 30.0 | 58.8 | 382.0 | 9.28e-108 |
| AEP40939.1 | polyketide\_synthase\_type\_I | BGC0000021 | Polyketide | 33.0 | 47.2 | 382.0 | 1.01e-107 |
| AAR16521.1 | RimA | BGC0000138 | Polyketide | 31.0 | 53.7 | 382.0 | 1.03e-107 |
| AEK75503.1 | type\_1\_polyketide\_synthase | BGC0000001 | Polyketide:Modular type I polyketide | 32.0 | 53.2 | 384.0 | 1.1e-107 |
| ATL73033.1 | type\_I\_modular\_polyketide\_synthase | BGC0001807 | NRP+Polyketide | 29.0 | 63.4 | 385.0 | 1.1e-107 |
| ctg1\_orf11 |  | BGC0000053 | Polyketide | 30.0 | 58.3 | 383.0 | 1.1e-107 |
| AWH12664.1 | RmpE2 | BGC0001759 | Polyketide | 30.0 | 56.6 | 384.0 | 1.18e-107 |
| APZ78793.1 | polyketide\_synthase | BGC0001427 | NRP:Cyclic depsipeptide+Polyketide:Iterative type I polyketide | 30.0 | 57.2 | 383.0 | 1.23e-107 |
| QBF51755.1 | type\_I\_polyketide\_synthase | BGC0001856 | Polyketide:Modular type I polyketide | 31.0 | 53.4 | 384.0 | 1.32e-107 |
| ABY21541.1 | AngAIV | BGC0000018 | Polyketide | 32.0 | 54.4 | 380.0 | 1.43e-107 |
| ALD82524.1 | polyketide\_synthase | BGC0001212 | NRP+Polyketide | 31.0 | 62.8 | 382.0 | 1.51e-107 |
| AAM54076.1 | polyketide\_synthase | BGC0000020 | Polyketide | 33.0 | 53.5 | 384.0 | 1.67e-107 |
| QKV49791.1 | PKS | BGC0002526 | Polyketide | 32.0 | 54.7 | 384.0 | 1.72e-107 |
| QIE07127.1 | OvmL1 | BGC0001719 | Polyketide | 30.0 | 59.9 | 383.0 | 1.74e-107 |
| AQX77694.1 | NocP | BGC0001704 | Other | 33.0 | 40.5 | 368.0 | 1.75e-107 |
| QES95475.1 | type\_I\_polyketide\_synthase | BGC0002453 | Polyketide | 32.0 | 58.5 | 382.0 | 1.77e-107 |
| ADU86002.1 | putative\_modular\_polyketide\_synthase | BGC0000165 | Polyketide:Modular type I polyketide | 31.0 | 57.2 | 384.0 | 1.78e-107 |
| AEZ54375.1 | PieA2 | BGC0000124 | Polyketide | 31.0 | 53.6 | 384.0 | 1.82e-107 |
| QCQ67877.1 | hybrid\_peptide\_synthetase/polyketide\_synthase | BGC0002297 | NRP+Polyketide | 30.0 | 55.0 | 383.0 | 1.84e-107 |
| ADH04639.1 | TgaA | BGC0001051 | NRP+Polyketide:Modular type I polyketide | 33.0 | 51.3 | 384.0 | 2.07e-107 |
| AVV61981.1 | type\_I\_modular\_PKS | BGC0001477 | NRP+Polyketide:Modular type I polyketide | 30.0 | 57.4 | 383.0 | 2.51e-107 |
| QBG82532.1 | Polyketide\_synthase | BGC0002587 | Polyketide | 31.0 | 55.1 | 383.0 | 2.53e-107 |
| AAM81586.2 | putative\_type\_I\_polyketide\_synthase | BGC0000047 | Polyketide | 31.0 | 54.6 | 383.0 | 2.6e-107 |
| WP\_063764078.1 | polyketide\_synthase | BGC0001348 | Polyketide:Modular type I polyketide | 31.0 | 57.7 | 382.0 | 2.83e-107 |
| BAB69193.1 |  | BGC0000117 | Polyketide | 32.0 | 54.1 | 383.0 | 3.02e-107 |
| AJW65409.1 | type\_I\_modular\_polyketide\_synthase | BGC0001195 | NRP+Polyketide | 31.0 | 52.9 | 383.0 | 3.05e-107 |
| AEC13079.1 | fosA | BGC0000060 | Polyketide | 31.0 | 57.8 | 383.0 | 3.12e-107 |
| QSV12664.1 | AvmF | BGC0002456 | Polyketide+NRP | 31.0 | 56.3 | 383.0 | 3.37e-107 |
| AEZ53949.1 | polyketide\_synthase | BGC0000144 | Polyketide:Modular type I polyketide | 30.0 | 58.3 | 383.0 | 3.45e-107 |
| BAO66528.1 | type\_I\_polyketide\_synthase | BGC0000042 | Polyketide | 34.0 | 48.9 | 369.0 | 3.46e-107 |
| CAQ18838.1 | polyketide\_synthase | BGC0000954 | NRP+Polyketide:Modular type I polyketide | 32.0 | 51.7 | 381.0 | 3.59e-107 |
| ARV85763.1 | PieA4\_type\_I\_PKS | BGC0001742 | Polyketide | 29.0 | 57.8 | 382.0 | 3.77e-107 |
| SCN11951.1 | ebeC-type\_I\_polyketide\_synthase | BGC0001580 | Polyketide | 32.0 | 53.2 | 382.0 | 3.85e-107 |
| AZF85917.1 | type\_I\_polyketide\_synthase | BGC0001963 | NRP+Polyketide | 30.0 | 61.0 | 382.0 | 4.12e-107 |
| ABB88522.1 | polyketide\_synthase\_type\_I | BGC0000050 | Polyketide | 32.0 | 58.1 | 382.0 | 4.12e-107 |
| ctg1\_orf28 |  | BGC0000096 | Polyketide | 30.0 | 62.4 | 381.0 | 4.33e-107 |
| AHB82062.1 | polyketide\_synthase | BGC0001231 | NRP+Polyketide:Modular type I polyketide | 30.0 | 61.1 | 382.0 | 4.37e-107 |
| AWH12668.1 | RmpC | BGC0001759 | Polyketide | 31.0 | 57.3 | 380.0 | 4.57e-107 |
| QKV49770.1 | PKS | BGC0002526 | Polyketide | 31.0 | 53.7 | 382.0 | 4.84e-107 |
| ADC45586.1 | modular\_polyketide\_synthase | BGC0000093 | Polyketide | 30.0 | 55.6 | 382.0 | 4.99e-107 |
| QSV12655.1 | AvmA | BGC0002456 | Polyketide+NRP | 32.0 | 56.7 | 382.0 | 5.16e-107 |
| CAD89775.1 | MelD\_protein | BGC0001010 | NRP+Polyketide:Modular type I polyketide | 30.0 | 62.3 | 382.0 | 5.44e-107 |
| CAI94682.1 | putative\_polyketide\_synthase | BGC0000141 | Polyketide | 31.0 | 53.2 | 382.0 | 5.52e-107 |
| AXI91551.1 | FunP2 | BGC0001944 | Polyketide | 31.0 | 54.2 | 377.0 | 5.64e-107 |
| ADC79619.1 | BafAIV | BGC0000028 | Polyketide:Modular type I polyketide | 31.0 | 57.6 | 382.0 | 5.75e-107 |
| BAT51066.1 | type\_I\_polyketide\_synthase | BGC0001296 | Polyketide | 30.0 | 58.7 | 382.0 | 5.85e-107 |
| ANZ22989.1 | ZinF | BGC0001828 | Polyketide | 31.0 | 62.2 | 382.0 | 6.02e-107 |
| APZ78832.1 | polyketide\_synthase | BGC0001430 | NRP:Cyclic depsipeptide+Polyketide:Iterative type I polyketide | 29.0 | 67.0 | 381.0 | 6.71e-107 |
| CAD55506.1 | CpkA;\_Polyketide\_synthase\_loading\_module,\_and\_modules\_1\_and\_2 | BGC0000038 | Polyketide:Modular type I polyketide | 30.0 | 62.3 | 382.0 | 6.78e-107 |
| AXI91552.1 | FunP1 | BGC0001944 | Polyketide | 32.0 | 54.1 | 382.0 | 6.9e-107 |
| AKJ15836.1 | Type\_I\_polyketide\_synthase | BGC0002735 | Polyketide+NRP | 31.0 | 57.4 | 381.0 | 6.97e-107 |
| CAD29795.1 | peptide\_synthetase | BGC0001015 | NRP+Polyketide | 30.0 | 55.2 | 381.0 | 7.46e-107 |
| BAG85029.1 | putative\_polyketide\_synthase | BGC0000086 | Polyketide | 31.0 | 53.1 | 378.0 | 7.88e-107 |
| AAX98187.1 | polyketide\_synthase\_type\_I | BGC0000052 | Polyketide | 31.0 | 53.2 | 378.0 | 8.44e-107 |
| AUO16402.1 | polyketide\_synthase | BGC0001700 | Polyketide | 30.0 | 53.1 | 381.0 | 9.31e-107 |
| ARV85764.1 | PieA5\_type\_I\_PKS | BGC0001742 | Polyketide | 31.0 | 59.1 | 380.0 | 9.5e-107 |
| CAJ88187.2 | Type\_I\_modular\_polyketide\_synthase | BGC0000151 | Polyketide:Modular type I polyketide+Saccharide:Hybrid/tailoring saccharide | 30.0 | 53.3 | 382.0 | 9.72e-107 |
| ATG32078.1 | polyketide\_synthase | BGC0001750 | NRP+Polyketide | 30.0 | 63.6 | 377.0 | 1.05e-106 |
| BAF85838.1 | modular\_polyketide\_synthase | BGC0000109 | Polyketide | 31.0 | 54.0 | 381.0 | 1.05e-106 |
| AAB66506.1 | tylactone\_synthase\_modules\_4\_&\_5 | BGC0000166 | Polyketide | 32.0 | 53.9 | 381.0 | 1.05e-106 |
| AGI99482.1 | Type\_I\_polyketide\_synthase | BGC0001004 | Polyketide:Modular type I polyketide | 30.0 | 62.9 | 381.0 | 1.09e-106 |
| QFU80887.1 | PKS | BGC0002550 | Polyketide | 30.0 | 62.9 | 381.0 | 1.09e-106 |
| ACC40921.1 | polyketide\_synthase\_Pks7 | BGC0001665 | Polyketide | 30.0 | 57.9 | 380.0 | 1.11e-106 |
| WP\_053138504.1 | type\_I\_polyketide\_synthase | BGC0002033 | Polyketide | 31.0 | 53.8 | 381.0 | 1.19e-106 |
| QBG82518.1 | Polyketide\_synthase | BGC0002587 | Polyketide | 30.0 | 57.4 | 380.0 | 1.25e-106 |
| ABV97152.1 | Beta-ketoacyl\_synthase | BGC0000137 | Polyketide | 30.0 | 61.3 | 381.0 | 1.27e-106 |
| QBL56182.1 | PKS | BGC0002376 | Polyketide | 30.0 | 61.7 | 381.0 | 1.35e-106 |
| AAP42867.1 | NanA7 | BGC0000105 | Polyketide | 32.0 | 53.1 | 377.0 | 1.4e-106 |
| BAK64650.1 | polyketide\_synthase | BGC0000135 | Polyketide | 31.0 | 58.5 | 381.0 | 1.4e-106 |
| ABJ97438.1 | MerB | BGC0001012 | NRP+Polyketide | 30.0 | 57.1 | 381.0 | 1.41e-106 |
| ABM21569.1 | crpA | BGC0000975 | NRP+Polyketide | 29.0 | 54.4 | 380.0 | 1.49e-106 |
| APZ78727.1 | polyketide\_synthase | BGC0001421 | NRP:Cyclic depsipeptide+Polyketide:Iterative type I polyketide | 30.0 | 57.1 | 380.0 | 1.53e-106 |
| AAZ94390.1 | modular\_polyketide\_synthase | BGC0000040 | Polyketide | 31.0 | 54.4 | 381.0 | 1.64e-106 |
| ARM20278.1 | polyketide\_synthase | BGC0001523 | Polyketide | 29.0 | 57.5 | 380.0 | 1.7e-106 |
| CBA11584.1 | polyketide\_synthase\_type\_I | BGC0001046 | NRP+Polyketide:Modular type I polyketide+Saccharide:Hybrid/tailoring saccharide | 29.0 | 62.6 | 381.0 | 1.78e-106 |
| AJW65407.1 | type\_I\_modular\_polyketide\_synthase | BGC0001195 | NRP+Polyketide | 30.0 | 56.8 | 381.0 | 1.8e-106 |
| TXD00266.1 | SDR\_family\_NAD(P)-dependent\_oxidoreductase | BGC0001877 | Polyketide | 29.0 | 58.3 | 380.0 | 1.91e-106 |
| APZ78807.1 | polyketide\_synthase | BGC0001428 | NRP:Cyclic depsipeptide+Polyketide:Iterative type I polyketide | 30.0 | 57.3 | 379.0 | 2.03e-106 |
| AWH12671.1 | RmpA1 | BGC0001759 | Polyketide | 31.0 | 52.7 | 380.0 | 2.11e-106 |
| ADC45535.1 | modular\_polyketide\_synthase | BGC0000093 | Polyketide | 30.0 | 60.0 | 380.0 | 2.22e-106 |
| ACO94468.1 | polyketide\_synthase\_type\_I | BGC0000029 | Polyketide:Modular type I polyketide | 31.0 | 57.4 | 380.0 | 2.25e-106 |
| AEC13071.1 | fosE | BGC0000060 | Polyketide | 30.0 | 59.0 | 380.0 | 2.34e-106 |
| ABC84469.1 | NigAIX | BGC0000114 | Polyketide:Modular type I polyketide | 30.0 | 58.3 | 379.0 | 2.35e-106 |
| UHH90012.1 | VicP4 | BGC0002634 | Polyketide+NRP+Other | 30.0 | 57.2 | 380.0 | 2.51e-106 |
| AGC09499.1 | LobS4 | BGC0001183 | Polyketide | 30.0 | 62.8 | 380.0 | 2.59e-106 |
| APZ78742.1 | polyketide\_synthase | BGC0001422 | NRP:Cyclic depsipeptide+Polyketide:Iterative type I polyketide | 30.0 | 57.4 | 379.0 | 2.68e-106 |
| APZ78754.1 | polyketide\_synthase | BGC0001423 | NRP:Cyclic depsipeptide+Polyketide:Iterative type I polyketide | 30.0 | 57.0 | 379.0 | 2.68e-106 |
| ABV97154.1 | Beta-ketoacyl\_synthase | BGC0000137 | Polyketide | 30.0 | 57.7 | 377.0 | 3.16e-106 |
| QEA08906.1 | JenA11 | BGC0002559 | Polyketide | 30.0 | 61.8 | 379.0 | 3.63e-106 |
| CAQ18832.1 | polyketide\_synthase | BGC0000954 | NRP+Polyketide:Modular type I polyketide | 29.0 | 58.3 | 379.0 | 3.68e-106 |
| TXD00025.1 | SDR\_family\_NAD(P)-dependent\_oxidoreductase | BGC0001877 | Polyketide | 31.0 | 56.2 | 380.0 | 3.88e-106 |
| AQM37582.1 | polyketide\_synthase | BGC0001424 | NRP:Cyclic depsipeptide+Polyketide:Iterative type I polyketide | 30.0 | 57.2 | 378.0 | 4.7e-106 |
| CQR60494.1 | Polyketide\_synthase,\_type\_I,\_module\_8 | BGC0001287 | Polyketide | 29.0 | 56.7 | 377.0 | 5.19e-106 |
| AAU93807.2 | polyketide\_synthase\_modules\_1\_and\_2 | BGC0000054 | Polyketide | 32.0 | 53.0 | 379.0 | 5.41e-106 |
| ARV85765.1 | PieA6\_type\_I\_PKS | BGC0001742 | Polyketide | 30.0 | 54.4 | 378.0 | 5.97e-106 |
| APD26279.1 | PtmA | BGC0001726 | NRP+Polyketide | 30.0 | 61.4 | 379.0 | 6.42e-106 |
| WP\_033261454.1 | type\_I\_polyketide\_synthase | BGC0002009 | Polyketide | 30.0 | 57.3 | 379.0 | 6.67e-106 |
| QKG20159.1 | type\_I\_polyketide\_synthase | BGC0002124 | Polyketide | 30.0 | 54.1 | 375.0 | 6.93e-106 |
| BAW35654.1 | modular\_polyketide\_synthase | BGC0002355 | Polyketide+Other | 30.0 | 54.4 | 375.0 | 6.98e-106 |
| CAJ88184.1 | Type\_I\_modular\_polyketide\_synthase | BGC0000151 | Polyketide:Modular type I polyketide+Saccharide:Hybrid/tailoring saccharide | 31.0 | 53.7 | 379.0 | 7.26e-106 |
| AAC01713.1 | RifD | BGC0000136 | Polyketide | 30.0 | 57.2 | 376.0 | 7.32e-106 |
| CAQ64689.1 | lasalocid\_modular\_polyketide\_synthase | BGC0000087 | Polyketide | 31.0 | 53.1 | 375.0 | 7.96e-106 |
| AAM81584.2 | putative\_type\_I\_polyketide\_synthase | BGC0000047 | Polyketide | 30.0 | 61.5 | 379.0 | 8.23e-106 |
| CAC22144.1 | CpkC;\_Polyketide\_synthase\_module\_5 | BGC0000038 | Polyketide:Modular type I polyketide | 29.0 | 57.6 | 377.0 | 8.26e-106 |
| AFD30954.1 | CrmA | BGC0000966 | NRP+Polyketide | 33.0 | 53.3 | 378.0 | 8.45e-106 |
| AKA59092.1 | type-I\_PKS | BGC0001619 | Polyketide | 33.0 | 52.4 | 379.0 | 8.59e-106 |
| ABG02264.1 | SalB | BGC0000143 | Polyketide | 31.0 | 53.6 | 378.0 | 8.66e-106 |
| WP\_015031691.1 | type\_I\_polyketide\_synthase | BGC0001819 | Polyketide | 30.0 | 55.1 | 371.0 | 8.89e-106 |
| TMU97098.1 | acyltransferase\_domain-containing\_protein | BGC0002038 | Polyketide | 31.0 | 54.3 | 375.0 | 9.42e-106 |
| QBL56184.1 | PKS | BGC0002376 | Polyketide | 31.0 | 57.2 | 378.0 | 9.47e-106 |
| WP\_051137606.1 | type\_I\_polyketide\_synthase | BGC0002011 | Polyketide | 30.0 | 59.3 | 378.0 | 9.64e-106 |
| CAF05651.1 | TubF\_protein | BGC0001053 | NRP+Polyketide | 32.0 | 53.5 | 378.0 | 1.02e-105 |
| AAC46028.1 | polyketide\_synthase\_module\_7 | BGC0000113 | Polyketide | 32.0 | 54.1 | 376.0 | 1.06e-105 |
| AP234\_RS37235 | type\_I\_polyketide\_synthase | BGC0001653 | Polyketide | 33.0 | 47.5 | 360.0 | 1.1e-105 |
| ANH11414.1 | SceS | BGC0001770 | Polyketide | 30.0 | 57.3 | 378.0 | 1.18e-105 |
| ONK09689.1 | Beta-ketoacyl-acyl-carrier-protein\_synthase\_I | BGC0001647 | Polyketide | 32.0 | 59.3 | 377.0 | 1.18e-105 |
| AEP40935.1 | polyketide\_synthase\_type\_I | BGC0000021 | Polyketide | 32.0 | 47.1 | 378.0 | 1.19e-105 |
| AEC13072.1 | fosF | BGC0000060 | Polyketide | 30.0 | 55.3 | 376.0 | 1.25e-105 |
| ADH04660.1 | TugD | BGC0001342 | NRP+Polyketide | 32.0 | 53.9 | 378.0 | 1.29e-105 |
| BAV56011.1 | PKS\_(KS-AT-DH-ER-KR-ACP-KS-AT-DH-ER-KR-ACP) | BGC0001597 | Polyketide | 30.0 | 58.3 | 378.0 | 1.42e-105 |
| ACR50773.1 | polyketide\_synthase | BGC0000163 | Polyketide | 30.0 | 57.4 | 378.0 | 1.55e-105 |
| QGJ79676.1 | Polyketide\_synthase | BGC0002552 | Polyketide | 30.0 | 59.3 | 377.0 | 1.69e-105 |
| ARS01476.1 | NcmAIV | BGC0001702 | NRP+Polyketide | 31.0 | 53.0 | 375.0 | 1.73e-105 |
| WP\_018540593.1 | type\_I\_polyketide\_synthase | BGC0001332 | NRP+Polyketide | 30.0 | 62.3 | 377.0 | 1.79e-105 |
| ARW71486.1 | type\_I\_PKS\_module\_6 | BGC0001812 | Polyketide | 32.0 | 53.5 | 373.0 | 1.98e-105 |
| AAQ84157.1 | Plm2-3 | BGC0000123 | Polyketide | 32.0 | 54.8 | 377.0 | 2.1e-105 |
| ABP55221.1 | acyl\_transferase\_domain\_protein | BGC0000142 | Polyketide | 31.0 | 53.6 | 373.0 | 2.15e-105 |
| ctg1\_orf522 |  | BGC0001199 | Polyketide | 29.0 | 59.9 | 377.0 | 2.22e-105 |
| WP\_102918844.1 | type\_I\_polyketide\_synthase | BGC0002104 | NRP+Polyketide | 33.0 | 48.5 | 377.0 | 2.27e-105 |
| SAI82908.1 | HrnD;\_Macrolactam\_polyketidesynthase\_type\_I;\_modules\_3-4 | BGC0002101 | Polyketide | 30.0 | 57.6 | 377.0 | 2.79e-105 |
| EHK80166.1 | beta-ketoacyl\_synthase | BGC0001447 | Polyketide | 33.0 | 47.6 | 377.0 | 3e-105 |
| CCP20050.1 | divL3\_protein | BGC0001119 | Polyketide:Modular type I polyketide | 31.0 | 59.2 | 375.0 | 3.56e-105 |
| ABV83223.1 | CppK | BGC0000116 | Polyketide | 32.0 | 52.8 | 375.0 | 3.67e-105 |
| AHB82057.1 | polyketide\_synthase | BGC0001019 | NRP+Polyketide:Modular type I polyketide | 32.0 | 54.0 | 372.0 | 3.84e-105 |
| AAU93806.2 | polyketide\_synthase\_modules\_3\_and\_4 | BGC0000054 | Polyketide | 30.0 | 60.8 | 376.0 | 3.86e-105 |
| AXI91545.1 | FunP8 | BGC0001944 | Polyketide | 31.0 | 56.8 | 375.0 | 4.33e-105 |
| CCM44338.1 | Polyketide\_synthase | BGC0001056 | NRP+Polyketide:Modular type I polyketide+Polyketide:PUFA synthase or related polyketide | 31.0 | 53.7 | 372.0 | 4.45e-105 |
| AKA59089.1 | type-I\_PKS | BGC0001619 | Polyketide | 29.0 | 61.1 | 376.0 | 4.7e-105 |
| AJD47482.1 | erythronolide\_synthase | BGC0002418 | NRP+Polyketide | 31.0 | 53.3 | 375.0 | 4.78e-105 |
| QUQ72348.1 | type\_I\_polyketide\_synthase | BGC0002349 | Polyketide+Saccharide | 33.0 | 49.0 | 375.0 | 5.91e-105 |
| BAV56006.1 | PKS\_(ACP-KS-AT-DH-ER-KR-ACP-KS-AT-KR-ACP) | BGC0001597 | Polyketide | 29.0 | 62.3 | 375.0 | 7.2e-105 |
| ADC79618.1 | BafAIII | BGC0000028 | Polyketide:Modular type I polyketide | 31.0 | 58.1 | 375.0 | 7.26e-105 |
| ctg1\_orf521 |  | BGC0001199 | Polyketide | 30.0 | 60.1 | 375.0 | 8.14e-105 |
| AHH99925.1 | PKS\_I | BGC0000002 | Polyketide | 30.0 | 52.9 | 375.0 | 8.19e-105 |
| BAJ16469.1 | polyketide\_synthase | BGC0000058 | Polyketide | 30.0 | 57.9 | 374.0 | 8.49e-105 |
| SAI82911.1 | HrnF;\_Macrolactam\_polyketide\_synthase\_type\_I;\_modules\_6-7 | BGC0002101 | Polyketide | 29.0 | 58.6 | 375.0 | 8.55e-105 |
| AAY28225.1 | HbmAI | BGC0000074 | Polyketide | 29.0 | 59.2 | 375.0 | 9.17e-105 |
| AGZ15473.1 | putative\_type\_I\_polyketide\_synthase | BGC0001036 | NRP+Polyketide | 30.0 | 58.0 | 373.0 | 1e-104 |
| AAZ77673.1 | ChlB1 | BGC0000036 | Polyketide:Modular type I polyketide+Polyketide:Iterative type I polyketide+Saccharide:Oligosaccharide | 31.0 | 57.3 | 372.0 | 1.11e-104 |
| AGZ15472.1 | putative\_modular\_polyketide\_synthase | BGC0001036 | NRP+Polyketide | 32.0 | 53.5 | 362.0 | 1.17e-104 |
| CAM00064.1 | EryAII\_Erythromycin\_polyketide\_synthase\_modules\_3\_and\_4 | BGC0000055 | Polyketide:Modular type I polyketide+Saccharide:Hybrid/tailoring saccharide | 30.0 | 59.9 | 375.0 | 1.18e-104 |
| AEC13080.1 | fosB | BGC0000060 | Polyketide | 29.0 | 63.6 | 373.0 | 1.36e-104 |
| CAO85893.1 | modular\_polyketide\_synthase\_NorA | BGC0000110 | Polyketide:Modular type I polyketide | 31.0 | 51.8 | 373.0 | 1.44e-104 |
| QRI43531.1 | type\_I\_polyketide\_synthase | BGC0002454 | Polyketide | 29.0 | 55.6 | 374.0 | 1.59e-104 |
| AXI91548.1 | FunP5 | BGC0001944 | Polyketide | 31.0 | 57.1 | 375.0 | 1.61e-104 |
| BCK51637.1 | modular\_modular\_polyketide\_synthase | BGC0002520 | Polyketide | 30.0 | 52.9 | 372.0 | 1.66e-104 |
| QOD94998.1 | PldAIII | BGC0002102 | Polyketide | 31.0 | 53.7 | 374.0 | 1.82e-104 |
| BAB69195.1 | modular\_polyketide\_synthase | BGC0000117 | Polyketide | 31.0 | 53.4 | 374.0 | 1.84e-104 |
| ACO94499.1 | polyketide\_synthase\_type\_I | BGC0000097 | Polyketide:Modular type I polyketide | 30.0 | 58.2 | 374.0 | 1.97e-104 |
| EPH46605.1 | putative\_Oleandomycin\_polyketide\_synthase,\_modules\_5\_and\_6 | BGC0001519 | NRP+Polyketide | 32.0 | 53.1 | 361.0 | 2.27e-104 |
| ATX68114.1 | malonyl\_CoA-acyl\_carrier\_protein\_transacylase | BGC0001772 | Polyketide | 33.0 | 40.0 | 362.0 | 2.39e-104 |
| AFV52200.1 | polyketide\_synthase\_module | BGC0000081 | NRP+Polyketide:Iterative type I polyketide+Polyketide:Enediyne type I polyketide | 31.0 | 56.3 | 372.0 | 2.4e-104 |
| ctg1\_orf255 |  | BGC0001200 | Polyketide | 30.0 | 57.6 | 373.0 | 2.46e-104 |
| AJO72735.1 | Type\_I\_modular\_polyketide\_synthase | BGC0001381 | Polyketide | 32.0 | 56.5 | 374.0 | 2.48e-104 |
| AEZ53951.1 | polyketide\_synthase | BGC0000144 | Polyketide:Modular type I polyketide | 30.0 | 53.3 | 370.0 | 2.48e-104 |
| QLD23837.1 | SDR\_family\_NAD(P)-dependent\_oxidoreductase | BGC0002086 | Polyketide | 32.0 | 53.5 | 370.0 | 2.7e-104 |
| ALA09355.1 | type\_I\_modular\_PKS | BGC0001303 | Polyketide | 33.0 | 50.9 | 373.0 | 3.1e-104 |
| WP\_039806854.1 | type\_I\_polyketide\_synthase | BGC0002001 | NRP+Polyketide | 31.0 | 55.3 | 369.0 | 3.1e-104 |
| QGJ79675.1 | Polyketide\_synthase | BGC0002552 | Polyketide | 28.0 | 59.9 | 374.0 | 3.11e-104 |
| CAM00065.1 | EryAIII\_Erythromycin\_polyketide\_synthase\_modules\_5\_and\_6 | BGC0000055 | Polyketide:Modular type I polyketide+Saccharide:Hybrid/tailoring saccharide | 31.0 | 53.1 | 373.0 | 3.26e-104 |
| BBM96637.1 | modular\_polyketide\_synthase | BGC0002452 | Polyketide | 31.0 | 52.9 | 371.0 | 3.86e-104 |
| ABF87031.1 | non-ribosomal\_peptide\_synthetase/polyketide\_synthase | BGC0000393 | NRP+Polyketide:Modular type I polyketide | 31.0 | 55.4 | 374.0 | 4.26e-104 |
| BAW35657.1 | modular\_polyketide\_synthase | BGC0002355 | Polyketide+Other | 30.0 | 53.9 | 373.0 | 4.38e-104 |
| APZ78858.1 | polyketide\_synthase | BGC0001432 | NRP:Cyclic depsipeptide+Polyketide:Iterative type I polyketide | 31.0 | 51.2 | 372.0 | 5.15e-104 |
| BCB17033.1 | modular\_polyketide\_synthase | BGC0002523 | NRP | 30.0 | 58.2 | 373.0 | 5.34e-104 |
| AHB82052.1 | polyketide\_synthase | BGC0001019 | NRP+Polyketide:Modular type I polyketide | 35.0 | 38.9 | 355.0 | 5.68e-104 |
| EPH46607.1 | putative\_Phenolphthiocerol\_synthesis\_polyketide\_synthase\_type\_I\_Pks15/1 | BGC0001519 | NRP+Polyketide | 32.0 | 58.0 | 372.0 | 5.83e-104 |
| ibo19 |  | BGC0001619 | Polyketide | 31.0 | 56.4 | 372.0 | 6.35e-104 |
| BCK51647.1 | modular\_polyketide\_synthase | BGC0002520 | Polyketide | 30.0 | 54.0 | 369.0 | 6.66e-104 |
| ALA09357.1 | type\_I\_modular\_PKS | BGC0001303 | Polyketide | 31.0 | 52.7 | 372.0 | 7.08e-104 |
| QRI43528.1 | type\_I\_polyketide\_synthase | BGC0002454 | Polyketide | 30.0 | 57.5 | 371.0 | 7.57e-104 |
| QBG82529.1 | Polyketide\_synthase | BGC0002587 | Polyketide | 29.0 | 62.2 | 372.0 | 7.65e-104 |
| ARV85761.1 | PieA2\_type\_I\_PKS | BGC0001742 | Polyketide | 29.0 | 60.4 | 372.0 | 8.1e-104 |
| AWW87425.1 | polyketide\_synthase | BGC0001755 | Polyketide | 30.0 | 58.1 | 372.0 | 8.68e-104 |
| AHA38202.1 | GphI | BGC0000069 | Polyketide | 31.0 | 59.7 | 371.0 | 1.04e-103 |
| BAT51067.1 | type\_I\_polyketide\_synthase | BGC0001296 | Polyketide | 29.0 | 55.1 | 371.0 | 1.05e-103 |
| OAP25819.1 | Phenolphthiocerol\_synthesis\_polyketide\_synthase\_type\_I\_Pks15/1 | BGC0001658 | Polyketide | 29.0 | 56.4 | 369.0 | 1.06e-103 |
| AAY28226.1 | HbmAII | BGC0000074 | Polyketide | 31.0 | 54.2 | 372.0 | 1.07e-103 |
| AAC46027.1 | polyketide\_synthase\_module\_6 | BGC0000113 | Polyketide | 31.0 | 53.5 | 368.0 | 1.1e-103 |
| AFU82614.1 | mixed\_NRPS\_PKS | BGC0000998 | NRP+Polyketide | 29.0 | 57.7 | 371.0 | 1.27e-103 |
| AMB20393.1 | polyketide\_synthase | BGC0002072 | Polyketide:Modular type I polyketide | 30.0 | 58.5 | 372.0 | 1.4e-103 |
| CAA60462.1 | polyketide\_synthase | BGC0001040 | NRP+Polyketide | 30.0 | 61.3 | 372.0 | 1.45e-103 |
| BBM96639.1 | modular\_polyketide\_synthase | BGC0002452 | Polyketide | 30.0 | 61.9 | 371.0 | 1.58e-103 |
| APZ78767.1 | polyketide\_synthase | BGC0001425 | NRP:Cyclic depsipeptide+Polyketide:Iterative type I polyketide | 29.0 | 56.5 | 370.0 | 1.69e-103 |
| AHB82063.1 | polyketide\_synthase | BGC0001231 | NRP+Polyketide:Modular type I polyketide | 34.0 | 39.4 | 354.0 | 1.69e-103 |
| CAJ88185.2 | Type\_I\_modular\_polyketide\_synthase | BGC0000151 | Polyketide:Modular type I polyketide+Saccharide:Hybrid/tailoring saccharide | 30.0 | 53.3 | 371.0 | 1.73e-103 |
| QYA95663.1 | acyltransferase\_domain-containing\_protein | BGC0002676 | NRP | 31.0 | 54.2 | 365.0 | 2e-103 |
| UMP03509.1 | NmvAII | BGC0002649 | NRP+Polyketide | 32.0 | 48.6 | 368.0 | 2.05e-103 |
| AVV61982.1 | type\_I\_modular\_PKS | BGC0001477 | NRP+Polyketide:Modular type I polyketide | 28.0 | 63.2 | 369.0 | 2.71e-103 |
| ctg1\_11 |  | BGC0001931 | Polyketide | 32.0 | 57.1 | 369.0 | 3.14e-103 |
| UMP03507.1 | NmvAIV | BGC0002649 | NRP+Polyketide | 31.0 | 60.6 | 368.0 | 3.45e-103 |
| ACB37743.1 | putative\_type\_I\_polyketide\_synthase | BGC0000162 | Polyketide | 30.0 | 56.4 | 366.0 | 3.65e-103 |
| ctg1\_orf523 |  | BGC0001199 | Polyketide | 31.0 | 55.1 | 370.0 | 3.67e-103 |
| QLD23491.1 | Polyketide\_synthase | BGC0002085 | Saccharide:Oligosaccharide | 31.0 | 56.7 | 362.0 | 3.84e-103 |
| AWR88404.1 | putative\_beta-ketoacyl\_synthase | BGC0001522 | Polyketide | 30.0 | 58.2 | 370.0 | 4.2e-103 |
| BBM96638.1 | modular\_polyketide\_synthase | BGC0002452 | Polyketide | 31.0 | 53.3 | 366.0 | 4.21e-103 |
| QKW94294.1 | short-chain\_dehydrogenase/reductase\_SDR | BGC0002342 | NRP+Polyketide | 31.0 | 48.5 | 354.0 | 4.25e-103 |
| QNN81299.1 | IonAIII | BGC0002446 | Polyketide | 30.0 | 58.7 | 368.0 | 4.4e-103 |
| QUQ72347.1 | 3-ketoacyl-CoA\_thiolase | BGC0002349 | Polyketide+Saccharide | 33.0 | 47.4 | 370.0 | 6.15e-103 |
| ADC79616.1 | BafAI | BGC0000028 | Polyketide:Modular type I polyketide | 31.0 | 55.9 | 369.0 | 7.02e-103 |
| ADU86003.1 | putative\_modular\_polyketide\_synthase | BGC0000165 | Polyketide:Modular type I polyketide | 30.0 | 57.1 | 369.0 | 7.08e-103 |
| QQZ01587.1 | PKS | BGC0002498 | Other | 31.0 | 53.5 | 369.0 | 7.08e-103 |
| AGZ15474.1 | putative\_type\_I\_polyketide\_synthase | BGC0001036 | NRP+Polyketide | 31.0 | 57.4 | 368.0 | 7.22e-103 |
| ctg1\_14 |  | BGC0001931 | Polyketide | 30.0 | 61.1 | 368.0 | 7.22e-103 |
| AJO72737.1 | Type\_I\_modular\_polyketide\_synthase | BGC0001381 | Polyketide | 32.0 | 54.9 | 369.0 | 7.89e-103 |
| ctg1\_13 |  | BGC0001931 | Polyketide | 29.0 | 62.3 | 369.0 | 8.07e-103 |
| AWC08658.1 | polyketide\_synthase\_type\_I | BGC0001662 | Polyketide | 30.0 | 53.3 | 366.0 | 8.14e-103 |
| AAS79462.1 | polyketide\_synthase\_subunit | BGC0000035 | Polyketide | 30.0 | 53.9 | 365.0 | 8.46e-103 |
| OJF16269.1 | AceP2 | BGC0001491 | Polyketide | 29.0 | 57.8 | 369.0 | 9.37e-103 |
| AFL48533.1 | laidlomycin\_polyketide\_synthase\_(module\_10) | BGC0000084 | Polyketide | 30.0 | 53.6 | 365.0 | 9.4e-103 |
| CAC20919.1 | PimS4\_protein | BGC0000125 | Polyketide | 34.0 | 47.5 | 367.0 | 9.92e-103 |
| AQT01395.1 | SgnS4 | BGC0001690 | Polyketide | 34.0 | 47.5 | 367.0 | 9.92e-103 |
| AAQ84144.1 | Plm4 | BGC0000123 | Polyketide | 32.0 | 53.2 | 365.0 | 1.03e-102 |
| EFL02193.1 | amino\_acid\_adenylation\_domain-containing\_protein | BGC0000996 | NRP+Polyketide:Iterative type I polyketide | 29.0 | 64.6 | 368.0 | 1.43e-102 |
| BAB69199.1 | modular\_polyketide\_synthase | BGC0000117 | Polyketide | 30.0 | 62.0 | 368.0 | 1.45e-102 |
| BAQ25511.1 | type\_I\_polyketide\_synthase | BGC0001288 | Polyketide | 29.0 | 63.9 | 369.0 | 1.51e-102 |
| ADC45515.1 | modular\_polyketide\_synthase | BGC0000093 | Polyketide | 31.0 | 56.3 | 367.0 | 1.51e-102 |
| AXI91549.1 | FunP4 | BGC0001944 | Polyketide | 31.0 | 57.0 | 368.0 | 1.62e-102 |
| AWC08661.1 | polyketide\_synthase\_type\_I | BGC0001662 | Polyketide | 30.0 | 53.3 | 368.0 | 1.64e-102 |
| ALA09356.1 | type\_I\_modular\_PKS | BGC0001303 | Polyketide | 32.0 | 53.0 | 364.0 | 1.68e-102 |
| ABY66019.1 | 6-methylsalicylic\_acid\_synthase | BGC0001008 | Polyketide:Iterative type I polyketide+Polyketide:Enediyne type I polyketide | 31.0 | 54.5 | 365.0 | 1.79e-102 |
| AWR88405.1 | putative\_phosphopantetheine-binding\_domain-\_containing\_prot\_ein | BGC0001522 | Polyketide | 29.0 | 57.5 | 368.0 | 1.86e-102 |
| BAW35638.1 | modular\_polyketide\_synthase | BGC0002356 | Polyketide+Other | 31.0 | 53.6 | 365.0 | 1.92e-102 |
| AEZ64505.1 | Herb | BGC0001065 | Polyketide | 32.0 | 54.1 | 368.0 | 2.37e-102 |
| AKL64833.1 | polyketide\_synthase | BGC0002072 | Polyketide:Modular type I polyketide | 32.0 | 51.8 | 368.0 | 2.4e-102 |
| BAW35611.1 | modular\_polyketide\_synthase | BGC0002357 | Polyketide+Other | 31.0 | 53.4 | 364.0 | 2.6e-102 |
| AXI91547.1 | FunP6 | BGC0001944 | Polyketide | 30.0 | 53.2 | 367.0 | 2.89e-102 |
| MBC5793765.1 | polyketide\_synthase | BGC0002480 | Polyketide+NRP | 33.0 | 39.9 | 360.0 | 2.91e-102 |
| ADC79638.1 | TamAII | BGC0001052 | NRP+Polyketide:Modular type I polyketide | 28.0 | 63.9 | 367.0 | 2.97e-102 |
| AGM05532.1 | beta-ketoacyl\_synthase | BGC0002098 | Polyketide | 29.0 | 65.0 | 367.0 | 3.01e-102 |
| AAS79461.1 | polyketide\_synthase\_subunit | BGC0000035 | Polyketide | 31.0 | 54.6 | 367.0 | 3.24e-102 |
| CAD70195.1 | non-ribosomal\_peptide\_synthetase | BGC0001047 | NRP+Polyketide | 28.0 | 62.3 | 367.0 | 3.45e-102 |
| AAS79459.1 | polyketide\_synthase\_subunit | BGC0000035 | Polyketide | 31.0 | 53.8 | 367.0 | 3.56e-102 |
| BCB17029.1 | modular\_polyketide\_synthase | BGC0002523 | NRP | 30.0 | 56.0 | 367.0 | 3.7e-102 |
| WP\_081238289.1 | type\_I\_polyketide\_synthase | BGC0002105 | Polyketide | 31.0 | 51.8 | 365.0 | 3.82e-102 |
| CCA29203.1 | non-ribosomal\_peptide\_synthetase/polyketide\_synthase | BGC0000955 | NRP+Polyketide:Modular type I polyketide | 31.0 | 53.6 | 367.0 | 3.9e-102 |
| sipP2 | Type\_I\_Modular\_PKS | BGC0001452 | Polyketide | 30.0 | 57.9 | 367.0 | 3.93e-102 |
| QRI43527.1 | type\_I\_polyketide\_synthase | BGC0002454 | Polyketide | 29.0 | 60.2 | 367.0 | 4.15e-102 |
| AHH99920.1 | PKS\_I | BGC0000002 | Polyketide | 32.0 | 49.2 | 366.0 | 4.61e-102 |
| BAW35635.1 | modular\_polyketide\_synthase | BGC0002356 | Polyketide+Other | 31.0 | 53.6 | 366.0 | 4.96e-102 |
| AAC01711.1 | RifB | BGC0000136 | Polyketide | 30.0 | 57.1 | 367.0 | 5.01e-102 |
| ABB86409.1 | GelB | BGC0000067 | Polyketide | 31.0 | 53.7 | 366.0 | 5.27e-102 |
| ACO94470.1 | polyketide\_synthase\_type\_I | BGC0000029 | Polyketide:Modular type I polyketide | 32.0 | 49.2 | 365.0 | 6.57e-102 |
| AHH99921.1 | PKS\_I | BGC0000002 | Polyketide | 32.0 | 47.7 | 366.0 | 7.61e-102 |
| BAQ25507.1 | type\_I\_polyketide\_synthase | BGC0001288 | Polyketide | 30.0 | 57.4 | 363.0 | 9.21e-102 |
| ctg1\_12 |  | BGC0001931 | Polyketide | 31.0 | 53.3 | 362.0 | 1.22e-101 |
| CAJ88186.1 | Type\_I\_modular\_polyketide\_synthase | BGC0000151 | Polyketide:Modular type I polyketide+Saccharide:Hybrid/tailoring saccharide | 31.0 | 53.5 | 365.0 | 1.25e-101 |
| AGY62753.1 | EbeA | BGC0000051 | Polyketide | 31.0 | 50.9 | 352.0 | 1.41e-101 |
| WP\_157358234.1 | SDR\_family\_NAD(P)-dependent\_oxidoreductase | BGC0002011 | Polyketide | 28.0 | 59.9 | 365.0 | 1.41e-101 |
| WP\_081238284.1 | type\_I\_polyketide\_synthase | BGC0002105 | Polyketide | 30.0 | 55.4 | 365.0 | 1.44e-101 |
| AKA59090.1 | type-I\_PKS | BGC0001619 | Polyketide | 32.0 | 56.0 | 365.0 | 1.49e-101 |
| ACN64831.1 | PokM1 | BGC0001061 | Polyketide:Iterative type I polyketide+Polyketide:Type II polyketide+Saccharide:Hybrid/tailoring saccharide | 31.0 | 54.1 | 362.0 | 1.68e-101 |
| ABY21540.1 | AngAIII | BGC0000018 | Polyketide | 31.0 | 58.3 | 365.0 | 1.74e-101 |
| QUQ72353.1 | type\_I\_polyketide\_synthase | BGC0002349 | Polyketide+Saccharide | 31.0 | 52.7 | 365.0 | 1.78e-101 |
| CAI94713.1 | putative\_polyketide\_synthase | BGC0000141 | Polyketide | 31.0 | 53.3 | 365.0 | 2.01e-101 |
| ACN69992.1 | polyketide\_synthase | BGC0000079 | Polyketide | 30.0 | 55.4 | 364.0 | 2.03e-101 |
| QWF78547.1 | 3-ketoacyl-CoA\_thiolase | BGC0002142 | Polyketide | 29.0 | 57.4 | 365.0 | 2.06e-101 |
| AAQ84147.1 | Plm7 | BGC0000123 | Polyketide | 32.0 | 49.4 | 362.0 | 2.86e-101 |
| WP\_159041997.1 | SDR\_family\_NAD(P)-dependent\_oxidoreductase | BGC0002033 | Polyketide | 31.0 | 54.7 | 364.0 | 2.97e-101 |
| AAX98190.1 | polyketide\_synthase\_type\_I | BGC0000052 | Polyketide | 30.0 | 55.6 | 363.0 | 3.51e-101 |
| OSS48297.1 | hypothetical\_protein | BGC0002194 | Polyketide | 29.0 | 66.5 | 363.0 | 3.61e-101 |
| ADX66459.1 | ScnS4 | BGC0000108 | Polyketide | 33.0 | 47.4 | 362.0 | 3.75e-101 |
| AWR88393.1 | putative\_beta-ketoacyl\_synthase | BGC0001522 | Polyketide | 30.0 | 57.7 | 363.0 | 3.99e-101 |
| CAJ46689.1 | polyketide\_synthase | BGC0000969 | NRP:Cyclic depsipeptide+Polyketide:Modular type I polyketide | 31.0 | 53.7 | 363.0 | 4.21e-101 |
| AEU17899.1 | putative\_type\_I\_PKS | BGC0001072 | Saccharide+Polyketide:Modular type I polyketide+Polyketide:Type II polyketide+Other:Aminocoumarin | 30.0 | 57.9 | 363.0 | 4.32e-101 |
| BAV32159.1 | polyketide\_synthase | BGC0001373 | Polyketide | 32.0 | 50.1 | 363.0 | 4.44e-101 |
| QSE03603.1 | LcmE | BGC0002333 | Polyketide | 32.0 | 49.8 | 362.0 | 4.7e-101 |
| QWF78550.1 | 3-ketoacyl-CoA\_thiolase | BGC0002142 | Polyketide | 30.0 | 53.1 | 363.0 | 4.98e-101 |
| BAD08358.1 | polyketide\_synthase\_modules\_4 | BGC0000167 | Polyketide | 29.0 | 59.2 | 362.0 | 5.03e-101 |
| ACB37742.1 | putative\_type\_I\_polyketide\_synthase | BGC0000162 | Polyketide | 30.0 | 52.8 | 361.0 | 5.32e-101 |
| BAQ25512.1 | type\_I\_polyketide\_synthase | BGC0001288 | Polyketide | 28.0 | 61.5 | 363.0 | 6.94e-101 |
| ACO94498.1 | polyketide\_synthase\_type\_I | BGC0000097 | Polyketide:Modular type I polyketide | 32.0 | 47.7 | 361.0 | 8.25e-101 |
| AAC01714.1 | RifE | BGC0000136 | Polyketide | 31.0 | 51.4 | 362.0 | 8.46e-101 |
| sipP1 | Type\_I\_Modular\_PKS | BGC0001452 | Polyketide | 29.0 | 58.2 | 362.0 | 9.17e-101 |
| AAF71774.1 | nysA | BGC0000115 | Polyketide:Modular type I polyketide+Saccharide:Hybrid/tailoring saccharide | 33.0 | 47.4 | 356.0 | 1.06e-100 |
| AXI91546.1 | FunP7 | BGC0001944 | Polyketide | 30.0 | 57.7 | 362.0 | 1.2e-100 |
| WP\_019032754.1 | type\_I\_polyketide\_synthase | BGC0001331 | NRP:Cyclic depsipeptide+Polyketide:Modular type I polyketide | 31.0 | 56.3 | 360.0 | 1.24e-100 |
| AAZ94389.1 | modular\_polyketide\_synthase | BGC0000040 | Polyketide | 28.0 | 64.9 | 362.0 | 1.24e-100 |
| BAE93728.1 | type\_I\_polyketide\_synthase | BGC0000164 | Polyketide | 31.0 | 53.7 | 359.0 | 1.45e-100 |
| AAG13917.1 | megalomicin\_6-deoxyerythronolide\_B\_synthase\_1 | BGC0000092 | Polyketide | 32.0 | 53.6 | 362.0 | 1.52e-100 |
| sipP5 | Type\_I\_Modular\_PKS | BGC0001452 | Polyketide | 30.0 | 60.2 | 362.0 | 1.6e-100 |
| AAO06917.1 | GdmAII | BGC0000066 | Polyketide | 30.0 | 53.7 | 361.0 | 1.96e-100 |
| ctg1\_orf9 |  | BGC0000053 | Polyketide | 30.0 | 52.2 | 358.0 | 2.35e-100 |
| SAI82910.1 | HrnG;\_Macrolactam\_polyketide\_synthase\_type\_I;\_module\_8 | BGC0002101 | Polyketide | 31.0 | 50.1 | 360.0 | 2.57e-100 |
| AEZ53953.1 | polyketide\_synthase | BGC0000144 | Polyketide:Modular type I polyketide | 29.0 | 57.9 | 360.0 | 2.8e-100 |
| WP\_106731933.1 | type\_I\_polyketide\_synthase | BGC0001332 | NRP+Polyketide | 31.0 | 54.7 | 359.0 | 3.79e-100 |
| AAK57190.1 | MxaF | BGC0001022 | NRP+Polyketide | 30.0 | 51.1 | 360.0 | 3.86e-100 |
| ATV95616.1 | 6-methylsalicylic\_acid\_synthase | BGC0001503 | Polyketide | 30.0 | 53.3 | 358.0 | 5.05e-100 |
| AUA09465.1 | Erythronolide\_synthase,\_modules\_5\_and\_6 | BGC0002291 | Polyketide | 30.0 | 51.9 | 357.0 | 5.75e-100 |
| EHK80165.1 | beta-ketoacyl\_synthase | BGC0001447 | Polyketide | 30.0 | 51.8 | 358.0 | 5.9e-100 |
| BBM95964.1 | modular\_polyketide\_synthase | BGC0002558 | Polyketide | 31.0 | 53.1 | 357.0 | 5.96e-100 |
| AAG13918.1 | megalomicin\_6-deoxyerythronolide\_B\_synthase\_2 | BGC0000092 | Polyketide | 30.0 | 57.8 | 360.0 | 6.11e-100 |
| QQZ01584.1 | PKS | BGC0002498 | Other | 30.0 | 53.6 | 357.0 | 6.24e-100 |
| QIE07131.1 | OvmM | BGC0001719 | Polyketide | 30.0 | 53.2 | 359.0 | 6.32e-100 |
| AAD03048.1 | type\_I\_polyketide\_synthase | BGC0000041 | Polyketide | 31.0 | 53.1 | 358.0 | 6.56e-100 |
| ARE67851.1 | AbsB3 | BGC0001492 | Polyketide | 30.0 | 53.5 | 348.0 | 8.38e-100 |
| QLJ99331.2 | acyltransferase\_domain-containing\_protein | BGC0002088 | Polyketide+Saccharide:Oligosaccharide | 30.0 | 57.1 | 352.0 | 9.52e-100 |
| WP\_019634550.1 | type\_I\_polyketide\_synthase | BGC0001443 | NRP+Polyketide | 32.0 | 52.8 | 355.0 | 9.58e-100 |
| BCK51633.1 | modular\_polyketide\_synthase | BGC0002520 | Polyketide | 31.0 | 53.2 | 359.0 | 9.74e-100 |
| QIE07129.1 | OvmL3 | BGC0001719 | Polyketide | 30.0 | 61.2 | 357.0 | 9.75e-100 |
| BCB17032.1 | modular\_polyketide\_synthase | BGC0002523 | NRP | 30.0 | 55.5 | 359.0 | 1.01e-99 |
| CAO98852.1 | polyketide\_synthase\_AufI | BGC0000023 | Polyketide:Modular type I polyketide | 31.0 | 52.4 | 358.0 | 1.08e-99 |
| CBZ41586.1 | Type\_I\_modular\_polyketide\_synthase | BGC0000151 | Polyketide:Modular type I polyketide+Saccharide:Hybrid/tailoring saccharide | 31.0 | 53.0 | 355.0 | 1.11e-99 |
| AEH42474.1 | polyketide\_synthase | BGC0000032 | Polyketide | 30.0 | 53.1 | 357.0 | 1.51e-99 |
| AKG06378.1 | polyketide\_synthase\_type\_1 | BGC0001830 | Polyketide | 31.0 | 52.7 | 355.0 | 2.03e-99 |
| AEZ53945.1 | polyketide\_synthase | BGC0000144 | Polyketide:Modular type I polyketide | 30.0 | 56.9 | 358.0 | 2.23e-99 |
| ALA09358.1 | type\_I\_modular\_PKS | BGC0001303 | Polyketide | 31.0 | 51.2 | 354.0 | 2.23e-99 |
| BAW35614.1 | modular\_polyketide\_synthase | BGC0002357 | Polyketide+Other | 31.0 | 54.3 | 358.0 | 2.25e-99 |
| ADH04680.1 | hybrid\_polyketide\_synthase/non-ribosomal\_peptide\_synthetase | BGC0001344 | NRP+Polyketide | 31.0 | 54.1 | 358.0 | 2.42e-99 |
| WP\_053138522.1 | type\_I\_polyketide\_synthase | BGC0002033 | Polyketide | 31.0 | 57.3 | 356.0 | 2.51e-99 |
| CCP20051.1 | divM\_protein | BGC0001119 | Polyketide:Modular type I polyketide | 30.0 | 53.2 | 357.0 | 3.35e-99 |
| QWF78548.1 | 3-ketoacyl-CoA\_thiolase | BGC0002142 | Polyketide | 30.0 | 54.0 | 357.0 | 4.94e-99 |
| BAD08373.1 | polyketide\_synthase\_modules\_1-3 | BGC0000167 | Polyketide | 29.0 | 58.9 | 357.0 | 5.47e-99 |
| ABV83228.1 | CppA | BGC0000116 | Polyketide | 33.0 | 47.4 | 346.0 | 6.21e-99 |
| ABJ97439.1 | MerC | BGC0001012 | NRP+Polyketide | 30.0 | 60.1 | 357.0 | 6.42e-99 |
| BBM95963.1 | modular\_polyketide\_synthase | BGC0002558 | Polyketide | 30.0 | 57.7 | 356.0 | 6.43e-99 |
| ACY13414.1 | amino\_acid\_adenylation\_domain\_protein | BGC0001367 | NRP+Polyketide | 30.0 | 57.5 | 356.0 | 8.23e-99 |
| ABV83222.1 | CppJ | BGC0000116 | Polyketide | 34.0 | 45.1 | 356.0 | 9.28e-99 |
| QIQ28639.1 | Nbc43 | BGC0002541 | Other | 29.0 | 62.1 | 356.0 | 1.02e-98 |
| ctg1\_orf31 |  | BGC0000096 | Polyketide | 31.0 | 54.4 | 354.0 | 1.03e-98 |
| ATG32077.1 | polyketide\_synthase | BGC0001750 | NRP+Polyketide | 30.0 | 60.4 | 354.0 | 1.16e-98 |
| QWF78545.1 | 3-ketoacyl-CoA\_thiolase | BGC0002142 | Polyketide | 30.0 | 53.0 | 356.0 | 1.24e-98 |
| ADC79637.1 | TamAI | BGC0001052 | NRP+Polyketide:Modular type I polyketide | 30.0 | 52.8 | 356.0 | 1.26e-98 |
| BAC76492.1 | lankamycin\_synthase\_LkmAII | BGC0000085 | Polyketide | 30.0 | 58.7 | 355.0 | 1.32e-98 |
| QIE07124.1 | OvmK2 | BGC0001719 | Polyketide | 31.0 | 55.9 | 353.0 | 1.47e-98 |
| ARS01473.1 | NcmAI | BGC0001702 | NRP+Polyketide | 31.0 | 56.1 | 355.0 | 1.55e-98 |
| AAQ84146.1 | Plm6 | BGC0000123 | Polyketide | 31.0 | 53.6 | 352.0 | 1.58e-98 |
| CAJ88177.1 | Type\_I\_modular\_polyketide\_synthase | BGC0000151 | Polyketide:Modular type I polyketide+Saccharide:Hybrid/tailoring saccharide | 32.0 | 51.2 | 355.0 | 1.65e-98 |
| CAJ88176.1 | Type\_I\_modular\_polyketide\_synthase | BGC0000151 | Polyketide:Modular type I polyketide+Saccharide:Hybrid/tailoring saccharide | 32.0 | 51.7 | 355.0 | 1.74e-98 |
| ARS01474.1 | NcmAII | BGC0001702 | NRP+Polyketide | 30.0 | 48.5 | 353.0 | 1.77e-98 |
| ADC45516.1 | modular\_polyketide\_synthase | BGC0000093 | Polyketide | 30.0 | 52.8 | 354.0 | 1.78e-98 |
| ANY10599.1 | polyketide\_synthase | BGC0001773 | Polyketide | 32.0 | 51.4 | 354.0 | 2.05e-98 |
| CBA11582.1 | polyketide\_synthase\_type\_I | BGC0001046 | NRP+Polyketide:Modular type I polyketide+Saccharide:Hybrid/tailoring saccharide | 30.0 | 57.6 | 355.0 | 2.2e-98 |
| ACB46486.1 | polyketide\_synthase | BGC0000082 | Polyketide | 30.0 | 57.6 | 352.0 | 3.17e-98 |
| AAB66507.1 | tylactone\_synthase\_module\_6 | BGC0000166 | Polyketide | 31.0 | 54.6 | 351.0 | 3.34e-98 |
| QNN81298.1 | IonAII | BGC0002446 | Polyketide | 29.0 | 53.6 | 352.0 | 3.37e-98 |
| BBA20952.1 | type\_I\_polyketide\_synthase | BGC0001763 | NRP+Polyketide | 29.0 | 63.1 | 354.0 | 3.53e-98 |
| CCP20047.1 | divK\_protein | BGC0001119 | Polyketide:Modular type I polyketide | 30.0 | 56.0 | 354.0 | 3.65e-98 |
| QVV57684.1 | beta-ketoacyl\_synthase | BGC0002338 | Polyketide | 33.0 | 49.0 | 353.0 | 4.01e-98 |
| QFU80899.1 | PKS | BGC0002550 | Polyketide | 30.0 | 52.5 | 352.0 | 5.67e-98 |
| KFG78606.1 | polyketide\_synthase | BGC0002240 | Polyketide | 29.0 | 69.0 | 353.0 | 6.03e-98 |
| AAR87760.2 | ZmaK | BGC0001059 | NRP+Polyketide | 31.0 | 48.0 | 353.0 | 6.47e-98 |
| ACY06290.1 | type\_I\_polyketide\_synthase | BGC0001042 | NRP+Polyketide | 31.0 | 54.0 | 353.0 | 6.6e-98 |
| CAQ52624.1 | type\_I\_polyketide\_synthase,\_modules\_7-8 | BGC0001066 | Polyketide:Modular type I polyketide | 30.0 | 60.9 | 353.0 | 6.66e-98 |
| ABY21542.1 | AngAV | BGC0000018 | Polyketide | 31.0 | 58.1 | 352.0 | 6.83e-98 |
| BAP34763.1 | type\_I\_polyketide\_synthase | BGC0000078 | Polyketide | 28.0 | 63.4 | 353.0 | 7.25e-98 |
| UHH90010.1 | VicP2 | BGC0002634 | Polyketide+NRP+Other | 28.0 | 59.9 | 352.0 | 9.21e-98 |
| AQZ37113.1 | polyketide\_synthase | BGC0001511 | Polyketide | 32.0 | 53.7 | 353.0 | 1.04e-97 |
| QTT72099.1 | type\_I\_polyketide\_synthase | BGC0002350 | NRP+Polyketide+Saccharide | 32.0 | 52.9 | 351.0 | 1.12e-97 |
| ABB86408.1 | GelA | BGC0000067 | Polyketide | 30.0 | 54.4 | 353.0 | 1.21e-97 |
| BAQ25482.1 | type\_I\_polyketide\_synthase | BGC0001288 | Polyketide | 29.0 | 60.5 | 352.0 | 1.27e-97 |
| AEH42473.1 | polyketide\_synthase | BGC0000032 | Polyketide | 31.0 | 54.1 | 352.0 | 1.33e-97 |
| AJO72736.1 | Type\_I\_modular\_polyketide\_synthase | BGC0001381 | Polyketide | 30.0 | 57.8 | 352.0 | 2.9e-97 |
| QGJ79644.1 | Polyketide\_synthase | BGC0002552 | Polyketide | 32.0 | 44.9 | 351.0 | 3.28e-97 |
| CAJ76298.1 | putative\_hybrid\_polyketide-non-ribosomal\_peptide\_synthetase | BGC0000972 | NRP+Polyketide:Modular type I polyketide+Polyketide:Trans-AT type I polyketide | 31.0 | 53.1 | 351.0 | 3.31e-97 |
| BAF02921.1 | type\_I\_polyketide\_synthase | BGC0000073 | Polyketide | 29.0 | 56.7 | 350.0 | 5.25e-97 |
| AGI99495.1 | Type\_I\_polyketide\_synthase | BGC0001004 | Polyketide:Modular type I polyketide | 30.0 | 52.5 | 348.0 | 5.35e-97 |
| AQW44871.1 | polyketide\_synthase | BGC0001761 | Polyketide | 35.0 | 39.2 | 350.0 | 5.58e-97 |
| AAK89721.2 | polyketide\_synthetase,\_siderophore\_biosynthesis\_protein | BGC0002107 | NRP+Polyketide | 31.0 | 53.3 | 346.0 | 5.58e-97 |
| UHH90025.1 | VicP1 | BGC0002634 | Polyketide+NRP+Other | 29.0 | 59.6 | 350.0 | 6.09e-97 |
| AXI91550.1 | FunP3 | BGC0001944 | Polyketide | 30.0 | 53.7 | 350.0 | 7.25e-97 |
| AGC65513.1 | TtcA | BGC0001876 | NRP | 29.0 | 53.5 | 349.0 | 7.42e-97 |
| FAA01291.1 | polyketide\_synthase-nonribosomal\_peptide\_synthetase\_PyvA | BGC0002210 | Polyketide+NRP | 29.0 | 62.7 | 350.0 | 8.81e-97 |
| QSE03602.1 | LcmA | BGC0002333 | Polyketide | 31.0 | 53.0 | 349.0 | 9.54e-97 |
| QBQ83704.1 | polyketide\_synthase-nonribosomal\_peptide\_synthetase | BGC0002093 | Polyketide+NRP | 27.0 | 64.0 | 349.0 | 1.18e-96 |
| EJK79843.1 | amino\_acid\_adenylation\_enzyme/thioester\_reductase\_family\_protein | BGC0000436 | NRP | 29.0 | 53.5 | 349.0 | 1.2e-96 |
| AJO72734.1 | Type\_I\_modular\_polyketide\_synthase | BGC0001381 | Polyketide | 30.0 | 53.1 | 349.0 | 1.21e-96 |
| QBG82526.1 | Polyketide\_synthase | BGC0002587 | Polyketide | 30.0 | 56.9 | 349.0 | 1.32e-96 |
| UMP03506.1 | NmvAV | BGC0002649 | NRP+Polyketide | 31.0 | 48.6 | 348.0 | 1.82e-96 |
| QWF78552.1 | 3-ketoacyl-CoA\_thiolase | BGC0002142 | Polyketide | 29.0 | 53.1 | 349.0 | 2.09e-96 |
| CAQ52623.1 | type\_I\_polyketide\_synthase,\_module\_6 | BGC0001066 | Polyketide:Modular type I polyketide | 29.0 | 59.9 | 347.0 | 2.36e-96 |
| AAB66504.1 | tylactone\_synthase\_starter\_module\_and\_modules\_1\_&\_2 | BGC0000166 | Polyketide | 30.0 | 59.9 | 348.0 | 2.86e-96 |
| CAC20920.1 | PimS3\_protein | BGC0000125 | Polyketide | 32.0 | 47.5 | 346.0 | 2.95e-96 |
| AQT01394.1 | SgnS3 | BGC0001690 | Polyketide | 32.0 | 47.5 | 346.0 | 2.95e-96 |
| BAC76491.1 | lankamycin\_synthase\_LkmAIII | BGC0000085 | Polyketide | 30.0 | 53.2 | 347.0 | 7.1e-96 |
| AAG02357.1 | polyketide\_synthase | BGC0000963 | NRP:Glycopeptide+Polyketide:Modular type I polyketide+Saccharide:Hybrid/tailoring saccharide | 31.0 | 52.9 | 345.0 | 7.46e-96 |
| ACF35447.1 | mbcAIII | BGC0000090 | Polyketide | 31.0 | 58.2 | 346.0 | 1.05e-95 |
| CCE88377.1 | non-ribosomal\_peptide\_synthetase/polyketide\_synthase | BGC0001034 | NRP+Polyketide:Modular type I polyketide | 31.0 | 52.1 | 346.0 | 1.07e-95 |
| AAK89729.1 | polyketide\_synthase,\_siderophore\_biosynthesis\_protein | BGC0002107 | NRP+Polyketide | 30.0 | 53.3 | 342.0 | 1.34e-95 |
| AWX24483.1 | type\_I\_polyketide\_synthase | BGC0001695 | NRP | 31.0 | 53.2 | 341.0 | 1.66e-95 |
| KKP04599.1 | Non-ribosomal\_peptide\_synthetase\_-\_Polyketide\_synthase | BGC0002066 | NRP+Polyketide:Iterative type I polyketide | 29.0 | 59.0 | 345.0 | 2.44e-95 |
| AGU50952.1 | putative\_polyketide\_synthase | BGC0002417 | NRP+Polyketide | 30.0 | 54.2 | 341.0 | 3.14e-95 |
| ACS20361.1 | KR\_domain\_protein | BGC0002420 | NRP+Polyketide | 30.0 | 53.7 | 341.0 | 3.14e-95 |
| ADX66460.1 | ScnS3 | BGC0000108 | Polyketide | 32.0 | 47.2 | 343.0 | 3.71e-95 |
| CAQ52622.1 | type\_I\_polyketide\_synthase,\_modules\_4-5 | BGC0001066 | Polyketide:Modular type I polyketide | 29.0 | 60.2 | 344.0 | 5.59e-95 |
| AAQ84156.1 | Plm1 | BGC0000123 | Polyketide | 30.0 | 51.3 | 343.0 | 6.12e-95 |
| BAE93730.1 | type\_I\_polyketide\_synthase | BGC0000164 | Polyketide | 29.0 | 58.2 | 344.0 | 6.57e-95 |
| AAF86396.1 | FkbA | BGC0000994 | NRP+Polyketide | 34.0 | 40.3 | 344.0 | 6.84e-95 |
| AWH12938.1 | StmC | BGC0001784 | Polyketide | 29.0 | 60.9 | 343.0 | 7.01e-95 |
| CCC55921.1 | non-ribosomal\_peptide\_synthetase/polyketide\_synthase\_hybrid\_protein | BGC0000973 | NRP+Polyketide:Modular type I polyketide | 30.0 | 53.1 | 343.0 | 8.62e-95 |
| ABP55222.1 | beta-ketoacyl\_synthase | BGC0000142 | Polyketide | 31.0 | 52.7 | 343.0 | 1.05e-94 |
| QUQ72349.1 | type\_I\_polyketide\_synthase | BGC0002349 | Polyketide+Saccharide | 32.0 | 47.3 | 342.0 | 1.23e-94 |
| EYT83439.1 | beta-ketoacyl\_synthase | BGC0001213 | Polyketide | 32.0 | 50.3 | 340.0 | 1.66e-94 |
| AFA26384.1 | polyketide\_synthase\_A | BGC0001874 | NRP+Polyketide | 31.0 | 50.0 | 342.0 | 1.67e-94 |
| AKG06375.1 | polyketide\_synthase\_type\_1 | BGC0001830 | Polyketide | 30.0 | 54.4 | 342.0 | 1.79e-94 |
| AGC09486.1 | LobS3 | BGC0001183 | Polyketide | 30.0 | 54.6 | 340.0 | 1.99e-94 |
| ANY10589.1 | polyketide\_synthase | BGC0001773 | Polyketide | 32.0 | 47.5 | 342.0 | 2.11e-94 |
| ABP55223.1 | beta-ketoacyl\_synthase | BGC0000142 | Polyketide | 30.0 | 53.1 | 342.0 | 2.68e-94 |
| AJO72743.1 | Type\_I\_modular\_polyketide\_synthase | BGC0001381 | Polyketide | 30.0 | 55.0 | 341.0 | 3.05e-94 |
| ABB88519.1 | polyketide\_synthase\_type\_I | BGC0000050 | Polyketide | 31.0 | 53.4 | 342.0 | 3.06e-94 |
| AAG13919.1 | megalomicin\_6-deoxyerythronolide\_B\_synthase\_3 | BGC0000092 | Polyketide | 30.0 | 58.7 | 341.0 | 3.32e-94 |
| ACM79805.1 | ZmaA | BGC0001059 | NRP+Polyketide | 29.0 | 53.2 | 341.0 | 3.43e-94 |
| QSV12659.1 | AvmB | BGC0002456 | Polyketide+NRP | 29.0 | 58.3 | 341.0 | 3.98e-94 |
| EHA19289.1 | hypothetical\_protein | BGC0001124 | Polyketide | 28.0 | 66.6 | 340.0 | 4e-94 |
| BCA42568.1 | polyketide\_synthase\_GrgA | BGC0002185 | Polyketide | 30.0 | 58.7 | 340.0 | 4.33e-94 |
| ADU85988.1 | putative\_iterative\_type\_I\_polyketide\_synthase | BGC0000165 | Polyketide:Modular type I polyketide | 29.0 | 58.0 | 339.0 | 4.41e-94 |
| AHH99924.1 | PKS\_I | BGC0000002 | Polyketide | 33.0 | 46.0 | 338.0 | 6.78e-94 |
| ctg1\_orf253 |  | BGC0001200 | Polyketide | 29.0 | 53.3 | 340.0 | 7.44e-94 |
| ARO38317.1 | nonribosomal\_peptide\_synthetase | BGC0001560 | NRP+Polyketide | 32.0 | 53.3 | 340.0 | 1.08e-93 |
| ASK38717.1 | polyketide\_synthase | BGC0001436 | Polyketide:Iterative type I polyketide | 29.0 | 60.0 | 339.0 | 1.29e-93 |
| AGM05536.1 | type\_I\_polyketide\_synthase | BGC0002098 | Polyketide | 31.0 | 47.8 | 338.0 | 1.74e-93 |
| OAP25815.1 | Phenolphthiocerol\_synthesis\_polyketide\_synthase\_type\_I\_Pks15/1 | BGC0001658 | Polyketide | 29.0 | 62.3 | 338.0 | 2.67e-93 |
| AMJ52080.1 | lijA | BGC0002255 | Polyketide | 30.0 | 59.5 | 338.0 | 3.22e-93 |
| ABB88520.1 | polyketide\_synthase\_type\_I | BGC0000050 | Polyketide | 31.0 | 50.9 | 338.0 | 3.27e-93 |
| MBA0053740.1 | acyltransferase\_domain-containing\_protein | BGC0002096 | Polyketide | 29.0 | 59.9 | 337.0 | 5e-93 |
| WP\_079030450.1 | type\_I\_polyketide\_synthase | BGC0002033 | Polyketide | 29.0 | 61.1 | 336.0 | 5.94e-93 |
| CAO91861.1 | PKS-NRPS\_hybrid | BGC0000968 | NRP+Polyketide:Iterative type I polyketide | 29.0 | 59.8 | 337.0 | 6.12e-93 |
| ABY21538.1 | AngAI | BGC0000018 | Polyketide | 33.0 | 43.2 | 337.0 | 6.45e-93 |
| EHA28244.1 | hypothetical\_protein | BGC0001143 | Polyketide | 28.0 | 62.9 | 337.0 | 7.09e-93 |
| BAK26562.1 | PKS-NRPS\_hybrid | BGC0000977 | NRP+Polyketide | 31.0 | 50.2 | 336.0 | 1.38e-92 |
| CAQ52626.1 | type\_I\_polyketide\_synthase,\_loading\_module\_and\_modules\_1-3 | BGC0001066 | Polyketide:Modular type I polyketide | 30.0 | 52.5 | 335.0 | 2.9e-92 |
| ALJ49910.1 | TlmH | BGC0001237 | Polyketide | 29.0 | 63.7 | 334.0 | 3.27e-92 |
| AHD05619.1 | putative\_polyketide\_synthase\_subunit | BGC0001033 | NRP+Polyketide | 28.0 | 53.7 | 332.0 | 4.01e-92 |
| ABP73645.1 | SalA | BGC0000145 | NRP+Polyketide | 32.0 | 50.9 | 333.0 | 5.85e-92 |
| AQZ37114.1 | polyketide\_synthase | BGC0001511 | Polyketide | 31.0 | 53.3 | 333.0 | 1.07e-91 |
| BAQ21947.1 | putative\_type\_I\_polyketide\_synthase | BGC0001204 | Polyketide | 27.0 | 61.7 | 333.0 | 1.19e-91 |
| CBX99534.1 | similar\_to\_polyketide\_synthase | BGC0001899 | Polyketide | 27.0 | 64.5 | 332.0 | 1.5e-91 |
| BAQ25481.1 | type\_I\_polyketide\_synthase | BGC0001288 | Polyketide | 28.0 | 65.4 | 333.0 | 1.58e-91 |
| BAF92601.1 | iterative\_type\_I\_PKS | BGC0000118 | Polyketide | 30.0 | 55.8 | 331.0 | 1.75e-91 |
| ACJ24875.1 | 6-methylsalicylic\_acid\_synthase | BGC0000119 | Polyketide:Iterative type I polyketide+Saccharide:Hybrid/tailoring saccharide | 30.0 | 55.8 | 331.0 | 1.75e-91 |
| KKP00963.1 | fatty\_acid\_synthase\_S-acetyltransferase | BGC0001854 | Polyketide:Iterative type I polyketide | 29.0 | 61.1 | 332.0 | 1.78e-91 |
| BAP34733.1 | type\_I\_polyketide\_synthase | BGC0000078 | Polyketide | 31.0 | 47.4 | 332.0 | 2.07e-91 |
| WP\_053138519.1 | type\_I\_polyketide\_synthase | BGC0002033 | Polyketide | 30.0 | 53.1 | 330.0 | 2.18e-91 |
| CAL69597.1 | PKS-NRPS | BGC0001049 | NRP+Polyketide:Iterative type I polyketide | 29.0 | 61.1 | 332.0 | 2.26e-91 |
| ABP53498.1 | PKS\_(ACP-AT-AT-KS-ACP-C) | BGC0001041 | NRP+Polyketide | 31.0 | 50.9 | 331.0 | 2.33e-91 |
| ANZ22988.1 | ZinE | BGC0001828 | Polyketide | 30.0 | 54.8 | 330.0 | 2.58e-91 |
| BBQ09587.1 | PKS-NRPS\_hybrid | BGC0002261 | Polyketide | 29.0 | 62.1 | 332.0 | 3.75e-91 |
| QUQ72344.1 | 3-ketoacyl-CoA\_thiolase | BGC0002349 | Polyketide+Saccharide | 32.0 | 45.8 | 331.0 | 4.23e-91 |
| CDG12864.1 | non-ribosomal\_peptide\_synthetase | BGC0001415 | NRP+Polyketide | 30.0 | 53.3 | 332.0 | 4.33e-91 |
| ABP55220.1 | beta-ketoacyl\_synthase | BGC0000142 | Polyketide | 30.0 | 53.1 | 331.0 | 6.29e-91 |
| BAV56012.1 | PKS\_(KS-AT-DH-ER-KR-ACP-TE) | BGC0001597 | Polyketide | 28.0 | 61.0 | 330.0 | 9.86e-91 |
| OJF16272.1 | AceP1 | BGC0001491 | Polyketide | 33.0 | 40.1 | 328.0 | 1.04e-90 |
| CAO98848.1 | polyketide\_synthase\_AufE | BGC0000023 | Polyketide:Modular type I polyketide | 33.0 | 41.3 | 328.0 | 1.15e-90 |
| AKL78824.1 | GLPKS3 | BGC0001187 | NRP:Lipopeptide+Polyketide:Iterative type I polyketide | 28.0 | 58.5 | 329.0 | 1.61e-90 |
| IH19\_RS1000000149540 | SDR\_family\_NAD(P)-dependent\_oxidoreductase | BGC0002106 | Polyketide | 33.0 | 41.1 | 311.0 | 2.09e-90 |
| AAO06916.1 | GdmAI | BGC0000066 | Polyketide | 29.0 | 54.4 | 329.0 | 2.43e-90 |
| AEP40932.1 | polyketide\_synthase\_type\_I | BGC0000021 | Polyketide | 31.0 | 49.0 | 327.0 | 2.88e-90 |
| BBG28498.1 | putative\_polyketide\_synthase | BGC0001913 | Polyketide | 29.0 | 56.2 | 328.0 | 3.11e-90 |
| AAV66110.2 | fusaridione\_A\_synthetase | BGC0000992 | NRP+Polyketide | 28.0 | 64.5 | 328.0 | 3.42e-90 |
| EPS29069.1 | hypothetical\_protein | BGC0001724 | NRP+Polyketide | 28.0 | 73.0 | 328.0 | 3.46e-90 |
| BBM95965.1 | modular\_polyketide\_synthase | BGC0002558 | Polyketide | 29.0 | 54.7 | 327.0 | 4.85e-90 |
| FGK60\_03740 | SDR\_family\_NAD(P)-dependent\_oxidoreductase | BGC0002038 | Polyketide | 33.0 | 42.1 | 324.0 | 1.01e-89 |
| BAE93731.1 | type\_I\_polyketide\_synthase | BGC0000164 | Polyketide | 29.0 | 58.3 | 327.0 | 1.21e-89 |
| AQH32482.1 | type\_1\_polyketide\_synthase | BGC0001667 | NRP+Polyketide | 31.0 | 39.5 | 326.0 | 2.3e-89 |
| AKD43765.1 | HerE | BGC0001349 | NRP+Polyketide | 34.0 | 40.7 | 323.0 | 2.59e-89 |
| ADN43685.1 | PKS-NRPS | BGC0001136 | NRP+Polyketide:Iterative type I polyketide | 28.0 | 61.0 | 324.0 | 7.18e-89 |
| CAQ34917.1 | polyketide\_synthase | BGC0000986 | NRP+Polyketide | 34.0 | 39.4 | 320.0 | 9.94e-89 |
| CAJ76291.1 | putative\_polyketide\_synthase | BGC0000972 | NRP+Polyketide:Modular type I polyketide+Polyketide:Trans-AT type I polyketide | 29.0 | 54.4 | 314.0 | 1.05e-88 |
| WP\_051137607.1 | type\_I\_polyketide\_synthase | BGC0002011 | Polyketide | 29.0 | 51.3 | 323.0 | 1.06e-88 |
| AAF86393.1 | FkbB | BGC0000994 | NRP+Polyketide | 34.0 | 39.9 | 324.0 | 1.16e-88 |
| EWG54266.1 | hypothetical\_protein | BGC0001190 | Polyketide | 26.0 | 66.1 | 322.0 | 1.77e-88 |
| AAM54078.1 | polyketide\_synthase | BGC0000020 | Polyketide | 29.0 | 56.0 | 322.0 | 2.46e-88 |
| AAC68815.1 | FK506\_polyketide\_synthase | BGC0000353 | NRP | 33.0 | 40.8 | 322.0 | 4.58e-88 |
| BAD97694.1 | Aft9-1 | BGC0000003 | Polyketide | 31.0 | 49.5 | 321.0 | 5.16e-88 |
| BCK51642.1 | modular\_polyketide\_synthase | BGC0002520 | Polyketide | 34.0 | 40.5 | 321.0 | 5.62e-88 |
| EHA52508.1 | mycocerosic\_acid\_synthase | BGC0001749 | Polyketide | 31.0 | 50.1 | 321.0 | 5.78e-88 |
| QSE03604.1 | LcmD | BGC0002333 | Polyketide | 34.0 | 41.0 | 320.0 | 6.17e-88 |
| QBK15049.1 | PKS-NRPS\_hybrid\_TraA | BGC0002197 | Polyketide+NRP | 28.0 | 63.2 | 321.0 | 8.12e-88 |
| ACC40923.1 | polyketide\_synthase\_Pks9 | BGC0001665 | Polyketide | 28.0 | 54.6 | 311.0 | 8.31e-88 |
| CBF80487.1 | hybrid\_PKS-NRPS\_(Eurofung) | BGC0000959 | NRP+Polyketide:Iterative type I polyketide | 29.0 | 59.8 | 320.0 | 1.42e-87 |
| QOJ72663.1 | XenE | BGC0002505 | Polyketide+NRP | 30.0 | 60.7 | 320.0 | 1.43e-87 |
| BBG28484.1 | polyketide\_synthase\_CdmE | BGC0001926 | Polyketide | 28.0 | 54.1 | 319.0 | 1.75e-87 |
| AZZ09613.1 | PvhA | BGC0002304 | Polyketide+NRP | 31.0 | 49.6 | 320.0 | 1.89e-87 |
| CCT72377.1 | probable\_polyketide\_synthase | BGC0001305 | Polyketide | 28.0 | 60.2 | 319.0 | 2.19e-87 |
| AQZ37095.1 | polyketide\_synthase | BGC0001511 | Polyketide | 29.0 | 56.7 | 319.0 | 2.21e-87 |
| AAM54075.1 | polyketide\_synthase | BGC0000020 | Polyketide | 29.0 | 57.6 | 319.0 | 2.68e-87 |
| BAG23201.1 | putative\_type-I\_PKS | BGC0002673 | Polyketide+Alkaloid | 33.0 | 39.8 | 319.0 | 2.71e-87 |
| AHE80996.1 | PieA6 | BGC0001169 | Polyketide:Modular type I polyketide | 28.0 | 55.1 | 318.0 | 2.74e-87 |
| OJJ98486.1 | hypothetical\_protein | BGC0002169 | Polyketide+NRP | 28.0 | 61.5 | 318.0 | 2.79e-87 |
| XP\_659388.1 | hypothetical\_protein | BGC0001998 | Polyketide | 29.0 | 60.8 | 318.0 | 3.84e-87 |
| KGO40478.1 | Acyl\_transferase/acyl\_hydrolase/lysophospholipase | BGC0001205 | Polyketide | 28.0 | 62.7 | 318.0 | 4.65e-87 |
| EAU38971.1 | PKS-NRPS\_hybrid | BGC0001122 | NRP+Polyketide:Iterative type I polyketide | 28.0 | 62.1 | 318.0 | 7.31e-87 |
| QNH68024.1 | PfpA | BGC0002268 | Polyketide+NRP | 28.0 | 58.6 | 318.0 | 7.45e-87 |
| QXF14600.1 | PydA | BGC0002239 | Polyketide+NRP | 28.0 | 63.9 | 317.0 | 1.73e-86 |
| AGO86662.1 | equisetin\_synthetase | BGC0001255 | NRP+Polyketide | 28.0 | 59.8 | 316.0 | 2.2e-86 |
| ATZ45182.1 | Bcboa6 | BGC0001892 | Polyketide | 30.0 | 48.6 | 315.0 | 3.36e-86 |
| QOG08944.1 | FfsA | BGC0002204 | Polyketide+NRP | 29.0 | 60.1 | 315.0 | 5.07e-86 |
| EED49862.1 | hybrid\_PKS/NRPS\_enzyme,\_putative | BGC0001445 | NRP+Polyketide:Iterative type I polyketide | 29.0 | 58.2 | 315.0 | 6.59e-86 |
| ABA02240.1 | polyketide\_synthase | BGC0000098 | Polyketide | 32.0 | 45.4 | 313.0 | 1.05e-85 |
| AAM77986.1 | iterative\_type\_I\_polyketide\_synthase | BGC0000112 | Polyketide:Iterative type I polyketide+Polyketide:Enediyne type I polyketide | 29.0 | 56.5 | 312.0 | 1.1e-85 |
| KFL51883.1 | amino\_acid\_adenylation\_protein | BGC0001711 | NRP+Polyketide | 39.0 | 27.5 | 314.0 | 1.21e-85 |
| AHE80991.1 | PieA1 | BGC0001169 | Polyketide:Modular type I polyketide | 28.0 | 54.5 | 313.0 | 1.31e-85 |
| EHA22196.1 | polyketide\_synthase | BGC0000170 | Polyketide | 28.0 | 52.9 | 311.0 | 2.66e-85 |
| ACS68554.1 | hybrid\_PKS-NRPS\_protein | BGC0001026 | NRP+Polyketide | 28.0 | 63.3 | 312.0 | 3.4e-85 |
| ADF88262.1 | mixed\_nonribosomal\_peptide\_synthetase/\_polyketide\_synthase | BGC0000979 | NRP+Polyketide | 29.0 | 43.6 | 308.0 | 5.58e-85 |
| ADF88265.1 | mixed\_nonribosomal\_peptide\_synthetase/\_polyketide\_synthase | BGC0000980 | NRP+Polyketide | 29.0 | 43.6 | 308.0 | 5.58e-85 |
| ACZ57548.1 | polyketide\_synthase | BGC0000046 | Polyketide:Iterative type I polyketide | 28.0 | 59.3 | 311.0 | 6.56e-85 |
| EPS34234.1 | nonribosomal\_peptide\_synthatase-polyketide\_synthase | BGC0002067 | NRP+Polyketide:Iterative type I polyketide | 29.0 | 59.3 | 311.0 | 7.76e-85 |
| ABV83230.1 | CppC | BGC0000116 | Polyketide | 34.0 | 39.0 | 311.0 | 7.9e-85 |
| EAQ84779.1 | hypothetical\_protein | BGC0001219 | Polyketide | 43.0 | 22.7 | 310.0 | 8.64e-85 |
| ABL74938.1 | PKS | BGC0001048 | NRP:Glycopeptide+Polyketide:Modular type I polyketide+Saccharide:Hybrid/tailoring saccharide | 29.0 | 51.9 | 308.0 | 8.91e-85 |
| ARP51711.1 | PKS-NRPS\_hybrid\_protein | BGC0001741 | NRP+Polyketide | 29.0 | 63.0 | 311.0 | 1.03e-84 |
| CBF82304.1 | polyketide\_synthase,\_putative\_(JCVI) | BGC0002180 | Polyketide | 29.0 | 55.9 | 310.0 | 1.19e-84 |
| BAC20566.1 | polyketide\_synthase | BGC0000039 | Polyketide | 28.0 | 64.6 | 310.0 | 1.25e-84 |
| ARE67852.1 | AbsB2 | BGC0001492 | Polyketide | 28.0 | 56.5 | 310.0 | 1.28e-84 |
| OPB37944.1 | putative\_polyketide\_synthase | BGC0002206 | Polyketide | 31.0 | 48.2 | 310.0 | 1.3e-84 |
| QCQ67874.1 | type\_I\_polyketide\_synthase | BGC0002297 | NRP+Polyketide | 31.0 | 39.8 | 310.0 | 1.33e-84 |
| IF55\_RS32375 | beta-ketoacyl\_synthase | BGC0001348 | Polyketide:Modular type I polyketide | 35.0 | 34.0 | 310.0 | 1.33e-84 |
| BAJ14522.1 | polyketide\_synthase | BGC0001254 | Polyketide | 28.0 | 59.3 | 310.0 | 1.56e-84 |
| AMY15057.1 | tetraketide\_synthase\_MF-SQTKS | BGC0001339 | Polyketide:Iterative type I polyketide | 30.0 | 47.3 | 310.0 | 1.67e-84 |
| AEK75502.1 | type\_1\_polyketide\_synthase | BGC0000001 | Polyketide:Modular type I polyketide | 33.0 | 39.7 | 310.0 | 2.7e-84 |
| AGY62756.1 | EbeD | BGC0000051 | Polyketide | 34.0 | 34.4 | 293.0 | 4.07e-84 |
| QQW45467.1 | polyketide\_synthase\_CalA' | BGC0002168 | Polyketide | 30.0 | 52.3 | 308.0 | 4.23e-84 |
| BBM05082.1 | polyketide\_synthase | BGC0002170 | Polyketide | 30.0 | 52.3 | 308.0 | 4.23e-84 |
| ARV85762.1 | PieA3\_type\_I\_PKS | BGC0001742 | Polyketide | 33.0 | 39.9 | 307.0 | 4.46e-84 |
| AQM58285.1 | polyketide\_synthase | BGC0001816 | NRP+Polyketide | 27.0 | 66.2 | 308.0 | 4.82e-84 |
| QFU19831.1 | PKS | BGC0002431 | Polyketide+Saccharide | 34.0 | 39.4 | 295.0 | 7.02e-84 |
| QCL09089.1 | dmxL2 | BGC0002063 | Polyketide:Iterative type I polyketide | 29.0 | 59.5 | 307.0 | 1.03e-83 |
| BAQ25466.1 | polyketide\_synthase | BGC0001264 | Polyketide | 28.0 | 59.0 | 306.0 | 1.66e-83 |
| BBU42026.1 | putative\_polyketide\_synthase | BGC0002222 | Polyketide | 28.0 | 55.9 | 306.0 | 1.98e-83 |
| QTE75992.1 | ZopPKS | BGC0002224 | Polyketide | 28.0 | 55.9 | 306.0 | 1.98e-83 |
| QKV49768.1 | PKS | BGC0002526 | Polyketide | 35.0 | 33.9 | 289.0 | 2.26e-83 |
| EHK18438.1 | putative\_polyketide\_synthase | BGC0002233 | Polyketide | 28.0 | 59.6 | 306.0 | 2.32e-83 |
| AHE80992.1 | PieA2 | BGC0001169 | Polyketide:Modular type I polyketide | 28.0 | 54.5 | 306.0 | 3.23e-83 |
| AFP73394.1 | FusA | BGC0001268 | NRP+Polyketide | 27.0 | 61.7 | 306.0 | 3.44e-83 |
| AKD43764.1 | HerF | BGC0001349 | NRP+Polyketide | 33.0 | 38.9 | 305.0 | 4.26e-83 |
| AAS98200.1 | MSAS-type\_polyketide\_synthase | BGC0001273 | Polyketide | 27.0 | 53.5 | 303.0 | 6.47e-83 |
| CEN60541.1 | hypothetical\_protein | BGC0002266 | Terpene+Polyketide | 29.0 | 58.7 | 304.0 | 9.01e-83 |
| CAO85898.1 | modular\_polyketide\_synthase\_NorC | BGC0000110 | Polyketide:Modular type I polyketide | 29.0 | 54.1 | 303.0 | 1.47e-82 |
| EKJ70677.1 | PKS6 | BGC0002188 | NRP+Polyketide | 26.0 | 65.2 | 303.0 | 1.72e-82 |
| AYM48705.1 | type\_I\_polyketide\_synthetase | BGC0002364 | Polyketide | 33.0 | 34.4 | 289.0 | 3.18e-82 |
| QIZ24099.1 | type\_I\_polyketide\_synthase | BGC0002540 | Polyketide | 34.0 | 39.4 | 299.0 | 1.41e-81 |
| CBF87072.1 | polyketide\_synthase,\_putative\_(Eurofung) | BGC0001290 | NRP | 28.0 | 62.9 | 300.0 | 1.52e-81 |
| AKA59437.1 | polyketide\_synthase | BGC0001202 | NRP+Polyketide | 30.0 | 48.0 | 287.0 | 1.78e-81 |
| ESU15174.1 | hypothetical\_protein | BGC0002186 | NRP+Polyketide | 30.0 | 59.0 | 300.0 | 2.01e-81 |
| QSJ20136.1 | polyketide\_synthase | BGC0002572 | NRP+Polyketide | 38.0 | 24.7 | 277.0 | 2.3e-81 |
| CBJ89766.1 | Polyketide\_synthase\_involved\_in\_xenocoumacin\_synthesis | BGC0001054 | NRP+Polyketide:Modular type I polyketide | 28.0 | 52.6 | 300.0 | 2.58e-81 |
| OQD69647.1 | hypothetical\_protein | BGC0002745 | Polyketide | 30.0 | 45.6 | 299.0 | 3.49e-81 |
| QPC57090.1 | polyketide\_synthase-nonribosomal\_peptide\_synthetase | BGC0002230 | Polyketide+NRP | 31.0 | 45.1 | 299.0 | 3.72e-81 |
| AHA38199.1 | GphF | BGC0000069 | Polyketide | 37.0 | 27.3 | 299.0 | 4.05e-81 |
| AAT28740.1 | FUSS | BGC0000064 | NRP+Polyketide | 28.0 | 58.5 | 299.0 | 4.68e-81 |
| EHA55875.1 | polyketide\_synthase | BGC0002235 | Polyketide+NRP | 30.0 | 49.7 | 299.0 | 4.93e-81 |
| AOE23578.1 | FoxBII | BGC0001598 | NRP+Polyketide | 40.0 | 24.9 | 298.0 | 1.19e-80 |
| QHD43130.1 | NRPS/PKS\_hybrid\_protein | BGC0002546 | NRP+Polyketide | 32.0 | 49.4 | 297.0 | 1.45e-80 |
| ACD39758.1 | reducing\_polyketide\_synthase | BGC0000076 | Polyketide | 29.0 | 59.0 | 296.0 | 2.13e-80 |
| ACD39767.1 | reducing\_polyketide\_synthase | BGC0000077 | Polyketide | 29.0 | 59.0 | 296.0 | 2.13e-80 |
| ACD39774.1 | reducing\_polyketide\_synthase | BGC0000134 | Polyketide | 28.0 | 58.7 | 296.0 | 2.85e-80 |
| mycI | polyketide\_synthase | BGC0002055 | NRP+Polyketide:Trans-AT type I polyketide | 33.0 | 34.0 | 295.0 | 6.52e-80 |
| EAL85113.2 | hybrid\_PKS-NRPS\_enzyme | BGC0001037 | NRP+Polyketide:Iterative type I polyketide | 28.0 | 60.7 | 295.0 | 9.75e-80 |
| ADA82585.1 | hybrid\_trans-AT\_polyketide\_synthase\_-\_nonribosomal\_peptide\_synthetase | BGC0001110 | NRP+Polyketide:Trans-AT type I polyketide | 32.0 | 39.1 | 295.0 | 9.93e-80 |
| CUX96955.1 | TmcH | BGC0001829 | NRP+Polyketide | 29.0 | 53.5 | 293.0 | 1.55e-79 |
| AEZ54376.1 | PieA3 | BGC0000124 | Polyketide | 33.0 | 39.9 | 291.0 | 2.79e-79 |
| QVV57688.1 | KR\_domain-containing\_protein | BGC0002338 | Polyketide | 32.0 | 39.2 | 292.0 | 3.23e-79 |
| KZL86691.1 | polyketide\_synthase | BGC0002228 | NRP | 27.0 | 63.2 | 292.0 | 3.4e-79 |
| OAQ63050.2 | polyketide\_synthase | BGC0002187 | Polyketide | 27.0 | 58.6 | 292.0 | 4.39e-79 |
| ALD83686.1 | tAT\_polyketide\_synthase | BGC0001300 | Polyketide | 38.0 | 25.2 | 293.0 | 4.51e-79 |
| EAW09117.1 | hybrid\_NRPS/PKS\_enzyme,\_putative | BGC0000983 | NRP+Polyketide:Iterative type I polyketide | 29.0 | 49.6 | 292.0 | 5.02e-79 |
| CEF75886.1 |  | BGC0001600 | Polyketide | 28.0 | 60.0 | 291.0 | 6.46e-79 |
| ALD82522.1 | polyketide\_synthase | BGC0001212 | NRP+Polyketide | 40.0 | 24.7 | 292.0 | 6.49e-79 |
| AVX51106.1 | nysA | BGC0001709 | Polyketide | 32.0 | 40.9 | 288.0 | 8.85e-79 |
| AHB38509.1 | polyketide\_synthase | BGC0000345 | NRP+Polyketide:Modular type I polyketide | 30.0 | 50.8 | 290.0 | 1.02e-78 |
| CTQ34881.1 | AtcD;\_polyketide\_synthase,\_modules\_1-4 | BGC0001301 | Polyketide | 38.0 | 26.2 | 291.0 | 1.03e-78 |
| EYE95336.1 | polyketide\_synthase | BGC0002234 | Polyketide | 29.0 | 59.1 | 290.0 | 1.32e-78 |
| EHA55860.1 | polyketide\_synthase/peptide\_synthetase | BGC0002235 | Polyketide+NRP | 29.0 | 51.0 | 291.0 | 1.49e-78 |
| BBI47418.1 | polyketide\_synthase | BGC0002258 | Polyketide | 28.0 | 51.4 | 290.0 | 1.85e-78 |
| ADU86004.1 | putative\_modular\_polyketide\_synthase | BGC0000165 | Polyketide:Modular type I polyketide | 34.0 | 32.5 | 289.0 | 4.08e-78 |
| KYC42746.1 | beta-ketoacyl\_synthase | BGC0002484 | NRP+Polyketide | 37.0 | 24.7 | 267.0 | 6.56e-78 |
| EHK80168.1 | acyl\_transferase | BGC0001447 | Polyketide | 32.0 | 36.6 | 271.0 | 1.64e-77 |
| KFL51881.1 | beta-ketoacyl\_synthase | BGC0001711 | NRP+Polyketide | 36.0 | 27.5 | 285.0 | 1.68e-77 |
| AAO56104.1 | yersiniabactin\_polyketide/non-ribosomal\_peptide\_synthetase | BGC0002570 | NRP+Polyketide | 28.0 | 53.0 | 287.0 | 1.98e-77 |
| CAN89632.1 | putative\_polyketide\_synthase | BGC0001070 | NRP+Polyketide:Modular type I polyketide+Polyketide:Trans-AT type I polyketide | 38.0 | 25.7 | 286.0 | 4.05e-77 |
| ACR12418.1 | modular\_polyketide\_synthase,\_type\_I\_PKS | BGC0000185 | Polyketide | 35.0 | 24.9 | 286.0 | 4.25e-77 |
| ATZ45185.1 | Bcboa9 | BGC0001892 | Polyketide | 28.0 | 51.0 | 285.0 | 5.81e-77 |
| AHE80993.1 | PieA3 | BGC0001169 | Polyketide:Modular type I polyketide | 32.0 | 39.5 | 284.0 | 7.89e-77 |
| BAN19720.1 | polyketide\_synthase | BGC0001252 | Polyketide | 26.0 | 59.0 | 285.0 | 8.32e-77 |
| QBC19710.1 | TwmB | BGC0001954 | NRP+Polyketide | 32.0 | 44.9 | 285.0 | 9.68e-77 |
| AHB38498.1 | polyketide\_synthase | BGC0000346 | NRP+Polyketide:Modular type I polyketide | 30.0 | 45.4 | 283.0 | 1.38e-76 |
| AMY15068.1 | hexaketide\_synthase\_MF-SQHKS | BGC0001339 | Polyketide:Iterative type I polyketide | 28.0 | 53.6 | 284.0 | 1.46e-76 |
| OAP25811.1 | Phenolphthiocerol\_synthesis\_polyketide\_synthase\_type\_I\_Pks15/1 | BGC0001658 | Polyketide | 34.0 | 32.6 | 269.0 | 2.29e-76 |
| WP\_012753526.1 | polyketide\_synthase | BGC0001991 | Polyketide | 36.0 | 26.7 | 276.0 | 2.59e-76 |
| AP234\_RS38640 | polyketide\_synthase | BGC0001653 | Polyketide | 37.0 | 24.6 | 264.0 | 2.65e-76 |
| AKQ22699.1 | malonyl\_CoA-acyl\_carrier\_protein\_transacylase | BGC0001186 | Polyketide | 31.0 | 39.6 | 283.0 | 3.02e-76 |
| ADI59533.1 | CorK | BGC0001091 | NRP+Polyketide | 34.0 | 31.3 | 283.0 | 4.54e-76 |
| QGY73445.1 | Itm13 | BGC0002451 | Polyketide | 38.0 | 27.5 | 282.0 | 6.82e-76 |
| ADU85981.1 | putative\_modular\_polyketide\_synthase | BGC0000165 | Polyketide:Modular type I polyketide | 33.0 | 33.4 | 280.0 | 7.04e-76 |
| QGW49095.1 | putative\_polyketide\_synthase | BGC0002731 | Polyketide | 28.0 | 63.7 | 280.0 | 2.07e-75 |
| AIA58899.1 | HRPKS | BGC0001141 | Polyketide:Iterative type I polyketide | 29.0 | 55.6 | 280.0 | 2.7e-75 |
| ABI91469.1 | beta-ketoacyl\_synthase | BGC0001094 | NRP+Polyketide | 36.0 | 26.7 | 279.0 | 3.24e-75 |
| AFB35635.1 | KS-AT-ACP | BGC0000935 | Polyketide+Other:Aminocoumarin | 32.0 | 39.6 | 271.0 | 3.31e-75 |
| AAF86392.1 | FkbC | BGC0000994 | NRP+Polyketide | 34.0 | 34.5 | 278.0 | 1.11e-74 |
| ADI59531.1 | CorI | BGC0001091 | NRP+Polyketide | 36.0 | 27.7 | 278.0 | 1.16e-74 |
| QZA73305.1 | type\_I\_polyketide\_synthase | BGC0002385 | Polyketide | 36.0 | 27.0 | 278.0 | 1.44e-74 |
| QSE03601.1 | LcmB | BGC0002333 | Polyketide | 34.0 | 33.7 | 278.0 | 1.81e-74 |
| ACY01400.1 | AT-less\_polyketide\_synthase | BGC0000083 | Polyketide:Modular type I polyketide+Polyketide:Trans-AT type I polyketide | 37.0 | 25.1 | 277.0 | 1.87e-74 |
| ASA76643.1 | polyketide\_synthase | BGC0001751 | NRP+Polyketide | 35.0 | 33.7 | 276.0 | 2.11e-74 |
| SAI82912.1 | HrnE;\_Macrolactam\_polyketide\_synthase\_type\_I;\_module\_5 | BGC0002101 | Polyketide | 34.0 | 33.0 | 275.0 | 3.58e-74 |
| ADI59534.1 | CorL | BGC0001091 | NRP+Polyketide | 36.0 | 26.9 | 276.0 | 3.79e-74 |
| ATX68124.1 | malonyl\_CoA-acyl\_carrier\_protein\_transacylase | BGC0001795 | Polyketide | 31.0 | 39.8 | 276.0 | 5.28e-74 |
| OAG05545.1 | PKSKA1\_protein | BGC0002211 | Polyketide | 28.0 | 55.6 | 275.0 | 5.52e-74 |
| EWM62997.1 | non-ribosomal\_peptide\_synthetase | BGC0001328 | NRP:Cyclic depsipeptide+Polyketide:Modular type I polyketide | 32.0 | 35.9 | 273.0 | 5.92e-74 |
| ACO94500.1 | polyketide\_synthase\_type\_I | BGC0000097 | Polyketide:Modular type I polyketide | 34.0 | 33.0 | 274.0 | 6.2e-74 |
| AIW00670.1 | mellein\_synthase | BGC0001244 | Polyketide | 28.0 | 44.4 | 274.0 | 7.82e-74 |
| WP\_234353271.1 | SDR\_family\_NAD(P)-dependent\_oxidoreductase | BGC0001537 | Polyketide | 35.0 | 34.3 | 275.0 | 7.93e-74 |
| AFN27483.1 | pks\_BonD | BGC0000173 | Polyketide:Modular type I polyketide | 38.0 | 26.3 | 275.0 | 1.04e-73 |
| BBA21072.1 | putative\_modular\_polyketide\_synthase | BGC0001740 | NRP+Polyketide | 34.0 | 31.9 | 275.0 | 1.12e-73 |
| ACY01390.1 | AT-less\_polyketide\_synthase | BGC0000177 | Polyketide:Modular type I polyketide+Polyketide:Trans-AT type I polyketide | 37.0 | 25.1 | 275.0 | 1.2e-73 |
| AZF85947.1 | type\_I\_polyketide\_synthase | BGC0001963 | NRP+Polyketide | 37.0 | 26.1 | 275.0 | 1.21e-73 |
| FS847\_01975 | type\_I\_polyketide\_synthase | BGC0001877 | Polyketide | 37.0 | 25.2 | 256.0 | 1.45e-73 |
| OJF16268.1 | AceP6 | BGC0001491 | Polyketide | 35.0 | 33.3 | 274.0 | 1.95e-73 |
| QIE07126.1 | OvmK4 | BGC0001719 | Polyketide | 29.0 | 42.2 | 273.0 | 2.31e-73 |
| ARR97039.1 | SphF | BGC0001780 | NRP | 34.0 | 30.4 | 273.0 | 2.31e-73 |
| QGY73449.1 | Itm17 | BGC0002451 | Polyketide | 37.0 | 25.8 | 274.0 | 2.52e-73 |
| OJF16270.1 | AceP3 | BGC0001491 | Polyketide | 34.0 | 33.3 | 274.0 | 2.94e-73 |
| ctg3\_19 |  | BGC0001853 | NRP+Polyketide:Modular type I polyketide | 36.0 | 27.7 | 273.0 | 4.83e-73 |
| AAC38075.1 | polyketide\_synthase\_type\_I | BGC0000127 | Polyketide | 34.0 | 30.6 | 272.0 | 4.97e-73 |
| CAN93347.1 | Polyketide\_synthase | BGC0000179 | Polyketide:Trans-AT type I polyketide | 36.0 | 27.7 | 273.0 | 5.78e-73 |
| AAY89049.1 | polyketide\_synthase | BGC0001069 | NRP+Polyketide:Trans-AT type I polyketide | 36.0 | 27.6 | 273.0 | 6e-73 |
| AHH99922.1 | PKS\_I | BGC0000002 | Polyketide | 32.0 | 34.8 | 272.0 | 7.19e-73 |
| MCC5026025.1 | SDR\_family\_NAD(P)-dependent\_oxidoreductase | BGC0002118 | NRP+Polyketide | 37.0 | 25.9 | 272.0 | 1.09e-72 |
| MCC5025981.1 | SDR\_family\_NAD(P)-dependent\_oxidoreductase | BGC0002119 | NRP+Polyketide | 37.0 | 25.9 | 272.0 | 1.09e-72 |
| QUQ72345.1 | 3-ketoacyl-CoA\_thiolase | BGC0002349 | Polyketide+Saccharide | 33.0 | 33.7 | 272.0 | 1.09e-72 |
| BAD38874.1 | polyketide\_synthase | BGC0000111 | Polyketide | 37.0 | 27.3 | 271.0 | 1.35e-72 |
| CAA60459.1 | polyketide\_synthase | BGC0001040 | NRP+Polyketide | 34.0 | 34.8 | 271.0 | 1.51e-72 |
| AGN74892.1 | nonribosomal\_peptide\_synthetase/polyketide\_synthase\_hybrid\_protein | BGC0000459 | NRP:Cyclic depsipeptide+Polyketide:Trans-AT type I polyketide | 37.0 | 27.8 | 271.0 | 1.64e-72 |
| CBJ89764.1 | Polyketide\_synthase\_involved\_in\_xenocoumacin\_synthesis | BGC0001054 | NRP+Polyketide:Modular type I polyketide | 27.0 | 45.7 | 270.0 | 1.85e-72 |
| AUS29500.1 | polyketide\_synthase | BGC0002607 | NRP+Polyketide | 28.0 | 55.0 | 270.0 | 2e-72 |
| QGZ36672.1 | amino\_acid\_adenylation\_domain-containing\_protein | BGC0002082 | NRP+Polyketide | 35.0 | 27.8 | 271.0 | 2.42e-72 |
| gene4 |  | BGC0001907 | Polyketide | 27.0 | 53.2 | 270.0 | 2.75e-72 |
| ADD82941.1 | Bat3 | BGC0001099 | NRP+Polyketide:Modular type I polyketide+Polyketide:Trans-AT type I polyketide | 36.0 | 25.1 | 270.0 | 2.95e-72 |
| ABI91465.1 | beta-ketoacyl\_synthase | BGC0001094 | NRP+Polyketide | 37.0 | 24.9 | 270.0 | 2.98e-72 |
| QDG75033.1 | mixed\_type\_I\_polyketide\_synthase\_-\_peptide\_synthetase | BGC0002068 | NRP+Polyketide | 34.0 | 30.9 | 270.0 | 3.07e-72 |
| AGC09485.1 | LobS2 | BGC0001183 | Polyketide | 35.0 | 30.9 | 270.0 | 4.24e-72 |
| ANR02549.1 | LodH | BGC0001648 | Polyketide | 36.0 | 25.5 | 269.0 | 4.25e-72 |
| ABY21539.1 | AngAII | BGC0000018 | Polyketide | 31.0 | 37.8 | 269.0 | 4.74e-72 |
| AAQ90173.1 | polyketide\_synthase\_type\_I | BGC0000128 | Polyketide | 36.0 | 25.7 | 269.0 | 5.66e-72 |
| ACO94472.1 | polyketide\_synthase\_type\_I | BGC0000029 | Polyketide:Modular type I polyketide | 33.0 | 33.0 | 268.0 | 6.55e-72 |
| CAL69890.1 | RhiC\_protein | BGC0001112 | NRP+Polyketide:Trans-AT type I polyketide | 36.0 | 27.5 | 269.0 | 6.68e-72 |
| ABI91464.1 | beta-ketoacyl\_synthase | BGC0001094 | NRP+Polyketide | 31.0 | 39.6 | 268.0 | 7.56e-72 |
| ABI91467.1 | beta-ketoacyl\_synthase | BGC0001094 | NRP+Polyketide | 37.0 | 26.5 | 268.0 | 7.81e-72 |
| AWH12667.1 | RmpD1 | BGC0001759 | Polyketide | 35.0 | 27.2 | 251.0 | 1.19e-71 |
| AFN27480.1 | pks\_BonA | BGC0000173 | Polyketide:Modular type I polyketide | 36.0 | 27.6 | 268.0 | 1.27e-71 |
| QTX15955.1 | polyketide\_synthase | BGC0002598 | Polyketide | 28.0 | 55.0 | 267.0 | 2.32e-71 |
| OJF16267.1 | AceP5 | BGC0001491 | Polyketide | 34.0 | 32.7 | 266.0 | 3.59e-71 |
| EAL85129.1 | polyketide\_synthase | BGC0001067 | Terpene+Polyketide:Iterative type I polyketide | 26.0 | 67.3 | 266.0 | 3.87e-71 |
| CAN93348.1 | polyketide\_synthase | BGC0000179 | Polyketide:Trans-AT type I polyketide | 37.0 | 25.0 | 266.0 | 3.96e-71 |
| DAC80076.1 | PKS | BGC0001835 | Polyketide | 36.0 | 26.8 | 266.0 | 4.15e-71 |
| AEP40938.1 | polyketide\_synthase\_type\_I | BGC0000021 | Polyketide | 36.0 | 24.6 | 247.0 | 4.25e-71 |
| ATG32074.1 | putative\_nonfunctional\_polyketide\_synthase\_module | BGC0001750 | NRP+Polyketide | 33.0 | 33.1 | 255.0 | 4.34e-71 |
| CAA60460.1 | polyketide\_synthase | BGC0001040 | NRP+Polyketide | 33.0 | 34.9 | 266.0 | 4.98e-71 |
| SKB24633.1 |  | BGC0002455 | Polyketide | 36.0 | 27.6 | 266.0 | 5.92e-71 |
| ALD83702.1 | tAT\_polyketide\_synthase | BGC0001299 | Polyketide | 35.0 | 27.5 | 266.0 | 6.14e-71 |
| API82671.1 | putative\_polyketide\_synthase | BGC0002616 | Polyketide | 25.0 | 60.4 | 265.0 | 6.23e-71 |
| ADI59532.1 | CorJ | BGC0001091 | NRP+Polyketide | 36.0 | 27.1 | 266.0 | 6.81e-71 |
| CBK62733.1 |  | BGC0001115 | NRP+Polyketide | 34.0 | 27.7 | 266.0 | 8.17e-71 |
| ABS90475.1 | PKS | BGC0001106 | NRP+Polyketide | 36.0 | 25.9 | 265.0 | 9.89e-71 |
| AAY32964.1 | DszA | BGC0001093 | NRP+Polyketide | 36.0 | 26.8 | 265.0 | 1.04e-70 |
| ATY69589.1 | type\_I\_polyketide\_synthase | BGC0001823 | NRP+Polyketide | 37.0 | 27.0 | 265.0 | 1.09e-70 |
| QDG75035.1 | mixed\_type\_I\_polyketide\_synthase\_-\_peptide\_synthetase | BGC0002068 | NRP+Polyketide | 34.0 | 26.7 | 265.0 | 1.11e-70 |
| DAC76730.1 | type\_I\_polyketide\_synthase | BGC0001885 | NRP+Polyketide | 36.0 | 27.2 | 265.0 | 1.12e-70 |
| ATX68112.1 | malonyl\_CoA-acyl\_carrier\_protein\_transacylase | BGC0001772 | Polyketide | 35.0 | 24.9 | 265.0 | 1.29e-70 |
| CAG23966.1 | polyketide\_synthase\_type\_I | BGC0000181 | Polyketide | 36.0 | 25.0 | 265.0 | 1.32e-70 |
| ADN68479.1 | SorD | BGC0000184 | Polyketide:Trans-AT type I polyketide | 35.0 | 27.7 | 265.0 | 1.37e-70 |
| AQW44870.1 | polyketide\_synthase | BGC0001761 | Polyketide | 36.0 | 25.3 | 265.0 | 1.42e-70 |
| AVR48533.1 | CusA | BGC0001564 | NRP+Polyketide | 33.0 | 31.2 | 265.0 | 1.71e-70 |
| API82664.1 | putative\_polyketide\_synthase | BGC0001677 | Polyketide | 25.0 | 60.4 | 264.0 | 1.85e-70 |
| ACY01401.1 | AT-less\_polyketide\_synthase | BGC0000083 | Polyketide:Modular type I polyketide+Polyketide:Trans-AT type I polyketide | 36.0 | 27.7 | 265.0 | 1.92e-70 |
| BAP34739.1 | type\_I\_polyketide\_synthase | BGC0000078 | Polyketide | 37.0 | 25.8 | 264.0 | 2.03e-70 |
| ADN68477.1 | SorB | BGC0000184 | Polyketide:Trans-AT type I polyketide | 32.0 | 32.8 | 264.0 | 2.23e-70 |
| ADH01489.1 | type\_I\_polyketide\_synthase | BGC0000995 | NRP+Polyketide | 36.0 | 25.5 | 263.0 | 2.78e-70 |
| ABF89568.1 | polyketide\_synthase | BGC0001025 | NRP+Polyketide:Trans-AT type I polyketide | 37.0 | 27.1 | 263.0 | 3.71e-70 |
| AIC32694.1 | FR9GH | BGC0001113 | NRP+Polyketide | 36.0 | 25.5 | 263.0 | 3.82e-70 |
| OEI73461.1 | hypothetical\_protein | BGC0001520 | Polyketide | 36.0 | 24.9 | 256.0 | 4.9e-70 |
| AFX60334.1 | polyketide\_synthase | BGC0001032 | NRP+Polyketide | 37.0 | 25.0 | 262.0 | 5.73e-70 |
| QLG04866.1 | PulE | BGC0002374 | Polyketide | 36.0 | 26.8 | 263.0 | 5.77e-70 |
| EAU29808.1 | hypothetical\_protein | BGC0001400 | Polyketide | 29.0 | 53.8 | 262.0 | 5.81e-70 |
| AJY78093.1 | polyketide\_synthase | BGC0001902 | NRP+Polyketide | 30.0 | 40.3 | 261.0 | 6.07e-70 |
| ATQ39432.1 | PKS | BGC0001565 | NRP | 27.0 | 61.0 | 262.0 | 6.09e-70 |
| AJO72742.1 | Type\_I\_modular\_polyketide\_synthase | BGC0001381 | Polyketide | 36.0 | 27.4 | 262.0 | 7.64e-70 |
| ALD83703.1 | tAT\_polyketide\_synthase | BGC0001299 | Polyketide | 36.0 | 27.6 | 262.0 | 9.6e-70 |
| EAT91803.2 | hypothetical\_protein | BGC0002205 | Polyketide+NRP | 29.0 | 49.2 | 262.0 | 1.05e-69 |
| AIJ04683.1 | polyketide\_synthase | BGC0001383 | Polyketide | 36.0 | 25.0 | 261.0 | 1.16e-69 |
| ERM18799.1 | polyketide\_synthase | BGC0000172 | Polyketide | 35.0 | 27.2 | 261.0 | 1.58e-69 |
| AXA20092.1 | trans-AT\_PKS\_LgaC | BGC0001646 | NRP+Polyketide | 33.0 | 30.5 | 261.0 | 1.61e-69 |
| AFX60311.1 | polyketide\_synthase | BGC0001031 | NRP+Polyketide | 38.0 | 24.9 | 261.0 | 1.68e-69 |
| QDG75024.1 | trans-\_AT\_polyketide\_synthase\_type\_I | BGC0002068 | NRP+Polyketide | 35.0 | 27.0 | 261.0 | 2.48e-69 |
| CAJ57409.1 | polyketide\_synthase\_type\_I | BGC0000176 | Polyketide | 35.0 | 26.9 | 261.0 | 2.59e-69 |
| bin5\_1\_edit\_las\_13 |  | BGC0002153 | Polyketide | 37.0 | 26.2 | 261.0 | 2.83e-69 |
| QZA73307.1 | type\_I\_polyketide\_synthase | BGC0002385 | Polyketide | 34.0 | 28.1 | 260.0 | 3.35e-69 |
| CCA89328.1 | mixed\_trans-AT\_type\_I\_polyketide\_synthase/nonribosomal\_peptide\_synthetase | BGC0001111 | NRP+Polyketide:Trans-AT type I polyketide | 35.0 | 27.4 | 260.0 | 3.73e-69 |
| RLV64601.1 | polyketide\_synthase\_of\_type\_I | BGC0001845 | Polyketide+NRP+Other:Aminocoumarin | 37.0 | 25.7 | 260.0 | 3.87e-69 |
| CAG23977.1 | polyketide\_synthase\_type\_I | BGC0000176 | Polyketide | 34.0 | 27.5 | 260.0 | 4.14e-69 |
| ACR13997.1 | modular\_polyketide\_synthase,\_type\_I\_PKS | BGC0000185 | Polyketide | 35.0 | 26.8 | 260.0 | 4.27e-69 |
| BAP05595.1 | calG | BGC0000967 | NRP+Polyketide:Trans-AT type I polyketide | 33.0 | 27.6 | 260.0 | 4.59e-69 |
| AGN74893.1 | nonribosomal\_peptide\_synthetase/polyketide\_synthase\_hybrid\_protein | BGC0000459 | NRP:Cyclic depsipeptide+Polyketide:Trans-AT type I polyketide | 37.0 | 28.3 | 259.0 | 4.7e-69 |
| DAD54487.1 | trans-acyltransferase\_polyketide\_synthase | BGC0002059 | NRP+Polyketide:Trans-AT type I polyketide | 35.0 | 24.9 | 259.0 | 5.72e-69 |
| ADA69237.1 | trans-AT\_polyketide\_synthase | BGC0001071 | NRP+Polyketide:Modular type I polyketide+Polyketide:Trans-AT type I polyketide | 34.0 | 25.4 | 259.0 | 7.71e-69 |
| EHK80169.1 | acyl\_transferase | BGC0001447 | Polyketide | 37.0 | 24.8 | 259.0 | 8.07e-69 |
| ADN68476.1 | sorA | BGC0000184 | Polyketide:Trans-AT type I polyketide | 35.0 | 27.3 | 259.0 | 8.46e-69 |
| ACR50796.1 | putative\_polyketide\_synthase | BGC0000163 | Polyketide | 34.0 | 27.4 | 258.0 | 8.93e-69 |
| CAG23958.2 | polyketide\_synthase\_of\_type\_I | BGC0001089 | Polyketide+NRP | 35.0 | 25.2 | 259.0 | 9.57e-69 |
| AJY78091.1 | polyketide\_synthase | BGC0001902 | NRP+Polyketide | 38.0 | 25.4 | 258.0 | 1.27e-68 |
| CAN93351.1 | polyketide\_synthase | BGC0000179 | Polyketide:Trans-AT type I polyketide | 32.0 | 32.6 | 258.0 | 1.5e-68 |
| AMH40423.1 | PKS | BGC0001350 | Polyketide | 35.0 | 26.4 | 258.0 | 1.63e-68 |
| CAG23960.2 | hybrid\_NRPS/PKS\_protein | BGC0001089 | Polyketide+NRP | 34.0 | 25.0 | 258.0 | 1.75e-68 |
| AUS29490.1 | polyketide\_synthase | BGC0002606 | NRP+Polyketide | 27.0 | 51.2 | 257.0 | 2.05e-68 |
| DAC80097.1 | PKS\_(DH-FkbM-FkbH-ACP-KS-ECH-ECH-ACP-ACP-KS-KR-ACP-KS) | BGC0001837 | Polyketide | 32.0 | 32.5 | 258.0 | 2.24e-68 |
| AGN74894.1 | nonribosomal\_peptide\_synthetase/polyketide\_synthase\_hybrid\_protein | BGC0000459 | NRP:Cyclic depsipeptide+Polyketide:Trans-AT type I polyketide | 35.0 | 25.7 | 257.0 | 2.31e-68 |
| AJQ95705.1 | polyketide\_synthase\_modules-related\_protein | BGC0001644 | Polyketide | 34.0 | 30.9 | 258.0 | 2.37e-68 |
| ALD83704.1 | tAT\_polyketide\_synthase | BGC0001299 | Polyketide | 36.0 | 26.2 | 257.0 | 2.75e-68 |
| AIU36103.1 | LglD | BGC0000180 | Polyketide:Trans-AT type I polyketide | 31.0 | 30.4 | 257.0 | 2.9e-68 |
| AAS47564.1 | mixed\_type\_I\_polyketide\_synthase/nonribosomal\_peptide\_synthetase | BGC0001108 | NRP+Polyketide:Trans-AT type I polyketide | 33.0 | 32.4 | 257.0 | 3.31e-68 |
| ctg1\_orf6 |  | BGC0001109 | NRP+Polyketide | 33.0 | 32.4 | 257.0 | 3.31e-68 |
| AMH40422.1 | PKS | BGC0001350 | Polyketide | 31.0 | 32.2 | 257.0 | 3.86e-68 |
| RAT98528.1 | trans-acyltransferase\_polyketide\_synthase | BGC0001470 | Polyketide:Trans-AT type I polyketide | 35.0 | 27.4 | 256.0 | 4.8e-68 |
| QZA73308.1 | type\_I\_polyketide\_synthase | BGC0002385 | Polyketide | 34.0 | 30.5 | 256.0 | 5.26e-68 |
| ABM63527.1 | BryB | BGC0000174 | Polyketide | 32.0 | 30.7 | 256.0 | 6.8e-68 |
| ABF92489.1 | mixed\_type\_I\_polyketide\_synthase\_-\_peptide\_synthetase | BGC0001025 | NRP+Polyketide:Trans-AT type I polyketide | 35.0 | 25.0 | 256.0 | 8.74e-68 |
| CCG06113.1 | type\_I\_polyketide\_synthase | BGC0001543 | Polyketide | 35.0 | 24.8 | 254.0 | 9.98e-68 |
| CAG23964.1 | polyketide\_synthase\_type\_I | BGC0000181 | Polyketide | 34.0 | 25.2 | 255.0 | 1.06e-67 |
| ASX95227.1 | IlaE | BGC0001620 | NRP+Polyketide | 30.0 | 41.0 | 255.0 | 1.12e-67 |
| CBK62724.1 |  | BGC0001115 | NRP+Polyketide | 34.0 | 25.4 | 255.0 | 1.19e-67 |
| mycH | polyketide\_synthase | BGC0002055 | NRP+Polyketide:Trans-AT type I polyketide | 33.0 | 27.6 | 255.0 | 1.23e-67 |
| WP\_003598535.1 | SDR\_family\_NAD(P)-dependent\_oxidoreductase | BGC0001991 | Polyketide | 37.0 | 26.0 | 254.0 | 1.25e-67 |
| BAP05593.1 | calE | BGC0000967 | NRP+Polyketide:Trans-AT type I polyketide | 34.0 | 25.5 | 255.0 | 1.26e-67 |
| AIJ04681.1 | polyketide\_synthase | BGC0001383 | Polyketide | 34.0 | 25.2 | 255.0 | 1.39e-67 |
| ACY01391.1 | AT-less\_polyketide\_synthase | BGC0000177 | Polyketide:Modular type I polyketide+Polyketide:Trans-AT type I polyketide | 36.0 | 25.7 | 255.0 | 1.66e-67 |
| AAY89052.1 | polyketide\_synthase | BGC0001069 | NRP+Polyketide:Trans-AT type I polyketide | 34.0 | 31.4 | 254.0 | 1.93e-67 |
| AFX60309.1 | polyketide\_synthase | BGC0001031 | NRP+Polyketide | 34.0 | 25.7 | 254.0 | 2.27e-67 |
| ADA69239.2 | trans-AT\_hybrid\_polyketide\_synthase-NRPS | BGC0001071 | NRP+Polyketide:Modular type I polyketide+Polyketide:Trans-AT type I polyketide | 33.0 | 30.0 | 254.0 | 2.86e-67 |
| ATX68125.1 | malonyl\_CoA-acyl\_carrier\_protein\_transacylase | BGC0001795 | Polyketide | 33.0 | 29.8 | 254.0 | 2.91e-67 |
| ERM18798.1 | polyketide\_synthase | BGC0000172 | Polyketide | 35.0 | 26.7 | 253.0 | 3.17e-67 |
| CAG23978.1 | polyketide\_synthase\_type\_I | BGC0000176 | Polyketide | 33.0 | 25.2 | 253.0 | 3.37e-67 |
| ANY10600.1 | polyketide\_synthase | BGC0001773 | Polyketide | 33.0 | 33.0 | 254.0 | 3.83e-67 |
| BAD38875.1 | polyketide\_synthase | BGC0000111 | Polyketide | 35.0 | 27.7 | 245.0 | 3.95e-67 |
| AEC04356.1 | polyketide\_synthase | BGC0000178 | Polyketide:Trans-AT type I polyketide | 35.0 | 24.5 | 253.0 | 4.32e-67 |
| BBA84070.1 | type\_I\_polyketide\_synthase | BGC0001649 | Polyketide | 32.0 | 33.2 | 253.0 | 4.55e-67 |
| SKB24638.1 |  | BGC0002455 | Polyketide | 33.0 | 30.6 | 253.0 | 4.6e-67 |
| AGY62758.1 | EbeF | BGC0000051 | Polyketide | 32.0 | 34.5 | 253.0 | 5.24e-67 |
| AXM42948.1 | type\_1\_polyketide\_synthase | BGC0001941 | NRP+Polyketide | 36.0 | 25.4 | 253.0 | 6.2e-67 |
| AFO59866.1 | ChxE | BGC0000175 | Polyketide:Trans-AT type I polyketide | 34.0 | 30.2 | 253.0 | 6.24e-67 |
| ATY69569.1 | type\_I\_polyketide\_synthase | BGC0001611 | NRP+Polyketide | 36.0 | 27.3 | 253.0 | 6.33e-67 |
| CTQ34882.1 | AtcE;\_polyketide\_synthase,\_modules\_5-7 | BGC0001301 | Polyketide | 35.0 | 27.5 | 253.0 | 7.29e-67 |
| ATX68111.1 | malonyl\_CoA-acyl\_carrier\_protein\_transacylase | BGC0001772 | Polyketide | 35.0 | 25.2 | 253.0 | 7.58e-67 |
| AAV97870.1 | OnnB | BGC0001105 | NRP+Polyketide:Trans-AT type I polyketide | 34.0 | 26.1 | 252.0 | 9.48e-67 |
| QIE07365.1 | polyketide\_synthase\_NecF | BGC0002050 | NRP+Polyketide:Trans-AT type I polyketide | 35.0 | 25.9 | 252.0 | 1.05e-66 |
| AAM12913.2 | MmpD | BGC0000182 | Polyketide:Iterative type I polyketide+Polyketide:Trans-AT type I polyketide | 35.0 | 27.2 | 252.0 | 1.06e-66 |
| DAC80098.1 | PKS\_(MT-ACP-KS-KR-ACP-KS-KR-MT-ACP-KS-KR-ACP-KS-ACP-ACP-KS-MT-\_KR-ACP) | BGC0001837 | Polyketide | 32.0 | 31.2 | 252.0 | 1.08e-66 |
| AAY39343.1 | Beta-ketoacyl\_synthase:Beta-ketoacyl\_synthase:Phosphopantetheine-binding\_protein | BGC0002060 | Polyketide:Trans-AT type I polyketide | 34.0 | 27.7 | 251.0 | 1.51e-66 |
| DAC80101.1 | PKS\_(KS-ACP-KS-KR-ACP-KS-DH-PS-KR-ACP-KS-ACP) | BGC0001837 | Polyketide | 35.0 | 24.8 | 251.0 | 1.64e-66 |
| bin5\_1\_edit\_las\_12 |  | BGC0002153 | Polyketide | 36.0 | 24.9 | 251.0 | 1.71e-66 |
| bin5\_1\_edit\_las\_14 |  | BGC0002153 | Polyketide | 32.0 | 30.2 | 249.0 | 1.78e-66 |
| CCC21123.1 | type-I\_polyketide\_synthases | BGC0000171 | Polyketide:Modular type I polyketide | 34.0 | 27.3 | 251.0 | 1.81e-66 |
| ADA82581.1 | trans-AT\_polyketide\_synthase | BGC0001110 | NRP+Polyketide:Trans-AT type I polyketide | 36.0 | 25.5 | 251.0 | 1.89e-66 |
| AXA20096.1 | trans-AT\_PKS\_LgaG | BGC0001646 | NRP+Polyketide | 36.0 | 26.9 | 251.0 | 1.92e-66 |
| DAC80061.1 | PKS | BGC0001836 | Polyketide:Trans-AT type I polyketide | 33.0 | 26.1 | 251.0 | 2.14e-66 |
| AJQ95678.1 | polyketide\_synthase\_modules-related\_protein | BGC0002046 | NRP+Polyketide:Trans-AT type I polyketide | 33.0 | 25.7 | 251.0 | 2.2e-66 |
| AFX60318.1 | polyketide\_synthase | BGC0001031 | NRP+Polyketide | 35.0 | 25.5 | 251.0 | 2.27e-66 |
| AFX60332.1 | polyketide\_synthase | BGC0001032 | NRP+Polyketide | 32.0 | 30.6 | 251.0 | 2.61e-66 |
| ASA76642.1 | polyketide\_synthase | BGC0001751 | NRP+Polyketide | 33.0 | 30.7 | 251.0 | 2.72e-66 |
| AGN11881.1 | tstDEF | BGC0001114 | NRP+Polyketide | 32.0 | 31.2 | 251.0 | 3.25e-66 |
| CAG23959.2 | polyketide\_synthase\_of\_type\_I | BGC0001089 | Polyketide+NRP | 33.0 | 27.9 | 250.0 | 3.35e-66 |
| AUS29495.1 | polyketide\_synthase | BGC0001030 | NRP+Polyketide | 26.0 | 63.9 | 249.0 | 4.7e-66 |
| CAL69891.1 | RhiD\_protein | BGC0001112 | NRP+Polyketide:Trans-AT type I polyketide | 35.0 | 25.0 | 250.0 | 4.73e-66 |
| AMH40421.1 | PKS | BGC0001350 | Polyketide | 34.0 | 25.5 | 250.0 | 5.48e-66 |
| mycF | polyketide\_synthase | BGC0002055 | NRP+Polyketide:Trans-AT type I polyketide | 35.0 | 26.4 | 250.0 | 5.71e-66 |
| SKB24635.1 |  | BGC0002455 | Polyketide | 35.0 | 25.8 | 249.0 | 7.66e-66 |
| RAT98525.1 | trans-acyltransferase\_polyketide\_synthase | BGC0001470 | Polyketide:Trans-AT type I polyketide | 35.0 | 25.7 | 249.0 | 8.33e-66 |
| ADN68480.1 | SorE | BGC0000184 | Polyketide:Trans-AT type I polyketide | 35.0 | 26.4 | 249.0 | 8.73e-66 |
| ACY01402.1 | AT-less\_polyketide\_synthase | BGC0000083 | Polyketide:Modular type I polyketide+Polyketide:Trans-AT type I polyketide | 33.0 | 28.0 | 248.0 | 8.82e-66 |
| AAS47562.1 | mixed\_type\_I\_polyketide\_synthase\_-\_peptide\_synthetase | BGC0001108 | NRP+Polyketide:Trans-AT type I polyketide | 33.0 | 25.0 | 249.0 | 9.15e-66 |
| ctg1\_orf8 |  | BGC0001109 | NRP+Polyketide | 33.0 | 25.0 | 249.0 | 9.15e-66 |
| BAF50727.1 | hybrid\_polyketide\_synthase-non\_ribosomal\_peptide\_synthetase | BGC0001116 | NRP+Polyketide | 36.0 | 26.2 | 249.0 | 9.35e-66 |
| ADD82940.1 | Bat2 | BGC0001099 | NRP+Polyketide:Modular type I polyketide+Polyketide:Trans-AT type I polyketide | 32.0 | 30.5 | 249.0 | 9.59e-66 |
| OEI73466.1 | hypothetical\_protein | BGC0001520 | Polyketide | 32.0 | 32.7 | 248.0 | 1.1e-65 |
| ABM63528.1 | BryC | BGC0000174 | Polyketide | 32.0 | 31.2 | 249.0 | 1.16e-65 |
| AEC04357.1 | polyketide\_synthase | BGC0000178 | Polyketide:Trans-AT type I polyketide | 31.0 | 33.0 | 249.0 | 1.18e-65 |
| QRN75753.1 | Polyketide\_synthase | BGC0002114 | NRP+Polyketide | 39.0 | 21.7 | 248.0 | 1.31e-65 |
| ALD83687.1 | tAT\_polyketide\_synthase | BGC0001300 | Polyketide | 35.0 | 27.5 | 248.0 | 1.43e-65 |
| AEC04361.1 | polyketide\_synthase | BGC0000178 | Polyketide:Trans-AT type I polyketide | 34.0 | 27.1 | 247.0 | 1.89e-65 |
| BAP05591.1 | calC | BGC0000967 | NRP+Polyketide:Trans-AT type I polyketide | 35.0 | 24.9 | 248.0 | 1.9e-65 |
| AGN11882.1 | tstGH | BGC0001114 | NRP+Polyketide | 36.0 | 25.3 | 248.0 | 1.93e-65 |
| CAE52339.1 | Polyketide\_non-ribosomal\_peptide\_synthase | BGC0001088 | NRP+Polyketide | 35.0 | 26.8 | 248.0 | 2.1e-65 |
| AAN85523.1 | polyketide\_synthase | BGC0001101 | NRP+Polyketide:Modular type I polyketide+Polyketide:Trans-AT type I polyketide | 36.0 | 25.3 | 248.0 | 2.13e-65 |
| AVR48535.1 | CusC | BGC0001564 | NRP+Polyketide | 35.0 | 24.5 | 248.0 | 2.22e-65 |
| AIU36104.1 | LglE | BGC0000180 | Polyketide:Trans-AT type I polyketide | 34.0 | 25.0 | 245.0 | 2.82e-65 |
| ALD83688.1 | tAT\_polyketide\_synthase | BGC0001300 | Polyketide | 35.0 | 27.8 | 247.0 | 3.99e-65 |
| ACG60781.1 | PKS(KS/AT/MT/KR/ACP) | BGC0001058 | NRP:Glycopeptide+Polyketide:Modular type I polyketide+Saccharide:Hybrid/tailoring saccharide | 32.0 | 39.5 | 246.0 | 4.84e-65 |
| ATX68127.1 | malonyl\_CoA-acyl\_carrier\_protein\_transacylase | BGC0001795 | Polyketide | 34.0 | 25.5 | 247.0 | 4.86e-65 |
| DAD54486.1 | trans-acyltransferase\_polyketide\_synthase | BGC0002059 | NRP+Polyketide:Trans-AT type I polyketide | 33.0 | 26.9 | 247.0 | 4.88e-65 |
| DAC80102.1 | PKS\_(ACP-KS-KR-ACP-KS-ACP-ACP-ACP-KS-ACP-C) | BGC0001837 | Polyketide | 33.0 | 28.3 | 246.0 | 5.47e-65 |
| ABC34675.1 | polyketide\_synthase,\_putative | BGC0000186 | NRP+Polyketide:Modular type I polyketide | 34.0 | 27.4 | 246.0 | 5.91e-65 |
| ADH01490.1 | type\_I\_polyketide\_synthase | BGC0000995 | NRP+Polyketide | 35.0 | 27.5 | 246.0 | 6.13e-65 |
| AIC32695.1 | FR9I | BGC0001113 | NRP+Polyketide | 35.0 | 27.5 | 246.0 | 6.13e-65 |
| bin5\_1\_edit\_las\_08 |  | BGC0002153 | Polyketide | 34.0 | 25.6 | 246.0 | 6.26e-65 |
| CAG23957.2 | hybrid\_NRPS/PKS\_protein | BGC0001089 | Polyketide+NRP | 33.0 | 25.3 | 246.0 | 7.51e-65 |
| ADH01487.1 | polyketide\_synthase | BGC0000995 | NRP+Polyketide | 36.0 | 26.1 | 246.0 | 9.8e-65 |
| AIC32693.1 | FR9DEF | BGC0001113 | NRP+Polyketide | 36.0 | 26.1 | 246.0 | 1.09e-64 |
| ADD82939.1 | Bat1 | BGC0001099 | NRP+Polyketide:Modular type I polyketide+Polyketide:Trans-AT type I polyketide | 35.0 | 25.2 | 245.0 | 1.09e-64 |
| CTQ34883.1 | AtcF;\_polyketide\_synthase,\_modules\_8-10 | BGC0001301 | Polyketide | 35.0 | 26.6 | 245.0 | 1.18e-64 |
| BBA21074.1 | putative\_modular\_polyketide\_synthase | BGC0001740 | NRP+Polyketide | 33.0 | 27.2 | 245.0 | 1.33e-64 |
| ABI91468.1 | beta-ketoacyl\_synthase | BGC0001094 | NRP+Polyketide | 36.0 | 25.7 | 244.0 | 1.35e-64 |
| AGN11883.1 | tstI | BGC0001114 | NRP+Polyketide | 35.0 | 27.6 | 245.0 | 1.38e-64 |
| RAT98526.1 | trans-acyltransferase\_polyketide\_synthase | BGC0001470 | Polyketide:Trans-AT type I polyketide | 33.0 | 25.4 | 244.0 | 1.48e-64 |
| BAP05594.1 | calF | BGC0000967 | NRP+Polyketide:Trans-AT type I polyketide | 34.0 | 28.1 | 245.0 | 1.76e-64 |
| ABF85931.1 | non-ribosomal\_peptide\_synthase/polyketide\_synthase\_Ta1 | BGC0001025 | NRP+Polyketide:Trans-AT type I polyketide | 35.0 | 25.3 | 244.0 | 2.5e-64 |
| bin5\_1\_edit\_las\_15 |  | BGC0002153 | Polyketide | 34.0 | 25.8 | 244.0 | 2.55e-64 |
| DAC76734.1 | type\_I\_polyketide\_synthase/non-ribosomal\_peptide\_synthetase | BGC0001885 | NRP+Polyketide | 33.0 | 25.5 | 244.0 | 3.24e-64 |
| OEI73463.1 | hypothetical\_protein | BGC0001520 | Polyketide | 33.0 | 25.5 | 243.0 | 5.01e-64 |
| BAC76474.1 | type\_I\_polyketide\_synthase\_LkcC | BGC0001100 | NRP+Polyketide | 33.0 | 26.7 | 242.0 | 7.69e-64 |
| QLG04867.1 | PulF | BGC0002374 | Polyketide | 33.0 | 28.5 | 243.0 | 8.35e-64 |
| RAT98529.1 | trans-acyltransferase\_polyketide\_synthase | BGC0001470 | Polyketide:Trans-AT type I polyketide | 35.0 | 25.3 | 242.0 | 9.52e-64 |
| QIE07364.1 | polyketide\_synthase\_NecE | BGC0002050 | NRP+Polyketide:Trans-AT type I polyketide | 34.0 | 25.7 | 242.0 | 1.15e-63 |
| QGZ36670.1 | SDR\_family\_NAD(P)-dependent\_oxidoreductase | BGC0002082 | NRP+Polyketide | 34.0 | 25.3 | 242.0 | 1.21e-63 |
| QCC63000.1 | BII-rafflesfungin\_polyketide\_synthase | BGC0001966 | NRP+Polyketide | 35.0 | 25.4 | 241.0 | 1.44e-63 |
| AFX60341.1 | polyketide\_synthase | BGC0001032 | NRP+Polyketide | 34.0 | 25.5 | 242.0 | 1.5e-63 |
| pelC | polyketide\_synthase | BGC0002056 | NRP+Polyketide:Trans-AT type I polyketide | 34.0 | 24.9 | 242.0 | 1.63e-63 |
| AKQ22682.1 | malonyl\_CoA-acyl\_carrier\_protein\_transacylase | BGC0001656 | Polyketide | 34.0 | 25.2 | 241.0 | 2e-63 |
| BAP05589.1 | calA | BGC0000967 | NRP+Polyketide:Trans-AT type I polyketide | 32.0 | 25.7 | 241.0 | 2.3e-63 |
| AEC04362.1 | polyketide\_synthase | BGC0000178 | Polyketide:Trans-AT type I polyketide | 30.0 | 30.1 | 241.0 | 2.47e-63 |
| AAY32965.1 | DszB | BGC0001093 | NRP+Polyketide | 34.0 | 28.4 | 241.0 | 2.67e-63 |
| CCP45170.1 | Polyketide\_synthetase\_MbtC\_(polyketide\_synthase) | BGC0001021 | NRP+Polyketide | 33.0 | 25.7 | 224.0 | 2.87e-63 |
| AJQ95707.1 | polyketide\_synthase\_modules-related\_protein | BGC0001644 | Polyketide | 32.0 | 27.8 | 241.0 | 3.13e-63 |
| AKQ22681.1 | malonyl\_CoA-acyl\_carrier\_protein\_transacylase | BGC0001656 | Polyketide | 34.0 | 25.0 | 240.0 | 4.35e-63 |
| AKQ22669.1 | malonyl\_CoA-acyl\_carrier\_protein\_transacylase | BGC0001656 | Polyketide | 33.0 | 25.6 | 240.0 | 4.5e-63 |
| UPA71921.1 | phthiocerol\_synthesis\_polyketide\_synthase\_type\_I\_PpsE | BGC0002636 | Polyketide | 30.0 | 39.2 | 234.0 | 4.57e-63 |
| DAC80077.1 | PKS | BGC0001835 | Polyketide | 33.0 | 27.6 | 240.0 | 4.61e-63 |
| ABS90470.1 | NRPS/PKS | BGC0001106 | NRP+Polyketide | 33.0 | 27.0 | 240.0 | 4.78e-63 |
| ATX68109.1 | malonyl\_CoA-acyl\_carrier\_protein\_transacylase | BGC0001772 | Polyketide | 34.0 | 25.3 | 240.0 | 4.8e-63 |
| BAE93740.1 | type\_I\_polyketide\_synthase-related\_protein | BGC0000164 | Polyketide | 33.0 | 27.5 | 239.0 | 5.35e-63 |
| QZA73306.1 | type\_I\_polyketide\_synthase | BGC0002385 | Polyketide | 34.0 | 27.5 | 240.0 | 5.54e-63 |
| BBA21068.1 | putative\_non-ribosomal\_peptide\_synthetase | BGC0001740 | NRP+Polyketide | 34.0 | 26.0 | 240.0 | 5.95e-63 |
| CAN89634.1 | putative\_polyketide\_synthase | BGC0001070 | NRP+Polyketide:Modular type I polyketide+Polyketide:Trans-AT type I polyketide | 35.0 | 25.4 | 240.0 | 6.05e-63 |
| pelD | polyketide\_synthase | BGC0002056 | NRP+Polyketide:Trans-AT type I polyketide | 34.0 | 25.0 | 240.0 | 6.34e-63 |
| CAJ57411.1 | polyketide\_synthase\_type\_I | BGC0000176 | Polyketide | 32.0 | 31.0 | 239.0 | 7.67e-63 |
| AMH40443.1 | PKS | BGC0001350 | Polyketide | 33.0 | 27.5 | 239.0 | 1.04e-62 |
| AJQ95708.1 | polyketide\_synthase\_modules-related\_protein | BGC0001644 | Polyketide | 34.0 | 25.3 | 239.0 | 1.51e-62 |
| AJQ95706.1 | polyketide\_synthase\_modules-related\_protein | BGC0001644 | Polyketide | 35.0 | 24.8 | 238.0 | 1.87e-62 |
| OEI73462.1 | hypothetical\_protein | BGC0001520 | Polyketide | 35.0 | 25.0 | 238.0 | 2.19e-62 |
| RAT98517.1 | trans-acyltransferase\_polyketide\_synthase | BGC0001470 | Polyketide:Trans-AT type I polyketide | 32.0 | 27.7 | 238.0 | 2.85e-62 |
| AKQ22696.1 | malonyl\_CoA-acyl\_carrier\_protein\_transacylase | BGC0001186 | Polyketide | 33.0 | 25.6 | 238.0 | 3.19e-62 |
| AJQ95675.1 | polyketide\_synthase\_modules-related\_protein | BGC0002046 | NRP+Polyketide:Trans-AT type I polyketide | 34.0 | 25.2 | 237.0 | 4.44e-62 |
| ABC34176.1 | polyketide\_synthase,\_putative | BGC0000186 | NRP+Polyketide:Modular type I polyketide | 35.0 | 24.6 | 236.0 | 4.69e-62 |
| ABC35027.1 | JamP | BGC0000961 | NRP+Polyketide | 33.0 | 28.0 | 235.0 | 5.29e-62 |
| RAT98527.1 | trans-acyltransferase\_polyketide\_synthase | BGC0001470 | Polyketide:Trans-AT type I polyketide | 35.0 | 24.2 | 236.0 | 6.58e-62 |
| AAY89053.1 | polyketide\_synthase | BGC0001069 | NRP+Polyketide:Trans-AT type I polyketide | 33.0 | 27.3 | 236.0 | 8.85e-62 |
| bin5\_1\_edit\_las\_10 |  | BGC0002153 | Polyketide | 35.0 | 25.1 | 233.0 | 9.18e-62 |
| CCG06109.1 | type\_I\_polyketide\_synthase | BGC0001543 | Polyketide | 34.0 | 25.0 | 235.0 | 1.01e-61 |
| ASA76633.1 | polyketide\_synthase | BGC0001751 | NRP+Polyketide | 34.0 | 28.2 | 233.0 | 1.23e-61 |
| ctg3\_18 |  | BGC0001853 | NRP+Polyketide:Modular type I polyketide | 33.0 | 27.6 | 235.0 | 1.39e-61 |
| BAP05590.1 | calB | BGC0000967 | NRP+Polyketide:Trans-AT type I polyketide | 33.0 | 27.3 | 235.0 | 1.56e-61 |
| AKQ22698.1 | malonyl\_CoA-acyl\_carrier\_protein\_transacylase | BGC0001186 | Polyketide | 34.0 | 25.1 | 235.0 | 1.64e-61 |
| ERM18797.1 | polyketide\_synthase | BGC0000172 | Polyketide | 30.0 | 27.8 | 235.0 | 1.68e-61 |
| DAC80063.1 | PKS | BGC0001836 | Polyketide:Trans-AT type I polyketide | 33.0 | 27.7 | 235.0 | 1.86e-61 |
| BAF50722.1 | polyketide\_synthase | BGC0001116 | NRP+Polyketide | 36.0 | 23.6 | 226.0 | 1.99e-61 |
| AXA20091.1 | hybrid\_trans-AT\_PKS/NRPS\_LgaB | BGC0001646 | NRP+Polyketide | 34.0 | 27.3 | 235.0 | 2.03e-61 |
| AAM12909.2 | MmpA | BGC0000182 | Polyketide:Iterative type I polyketide+Polyketide:Trans-AT type I polyketide | 32.0 | 30.9 | 234.0 | 2.61e-61 |
| CAL69893.1 | RhiE\_protein | BGC0001112 | NRP+Polyketide:Trans-AT type I polyketide | 34.0 | 24.6 | 234.0 | 3.09e-61 |
| AFX60336.1 | polyketide\_synthase | BGC0001032 | NRP+Polyketide | 33.0 | 26.7 | 234.0 | 3.5e-61 |
| ATX68110.1 | malonyl\_CoA-acyl\_carrier\_protein\_transacylase | BGC0001772 | Polyketide | 34.0 | 24.6 | 234.0 | 4.91e-61 |
| AJQ95704.1 | polyketide\_synthase\_modules-related\_protein | BGC0001644 | Polyketide | 33.0 | 25.3 | 233.0 | 5.88e-61 |
| AAY89051.1 | hybrid\_nonribosomal\_peptide\_synthetase/polyketide\_synthase | BGC0001069 | NRP+Polyketide:Trans-AT type I polyketide | 33.0 | 25.8 | 233.0 | 7.24e-61 |
| ACR13065.1 | modular\_polyketide\_synthase,\_type\_I\_PKS | BGC0000185 | Polyketide | 34.0 | 25.5 | 233.0 | 7.25e-61 |
| WP\_018960020.1 | type\_I\_polyketide\_synthase | BGC0002010 | NRP+Polyketide | 31.0 | 39.2 | 228.0 | 7.49e-61 |
| CAN93352.1 | polyketide\_synthase | BGC0000179 | Polyketide:Trans-AT type I polyketide | 34.0 | 26.8 | 233.0 | 7.72e-61 |
| DAC80074.1 | PKS | BGC0001835 | Polyketide | 33.0 | 25.4 | 232.0 | 1.3e-60 |
| AIJ04680.1 | polyketide\_synthase | BGC0001383 | Polyketide | 31.0 | 27.7 | 230.0 | 2.1e-60 |
| CCA89329.1 | trans-AT\_type\_I\_polyketide\_synthase | BGC0001111 | NRP+Polyketide:Trans-AT type I polyketide | 34.0 | 25.1 | 231.0 | 2.65e-60 |
| AHD05679.1 | putative\_non-ribosomal\_peptide\_ligase/\_polyketide\_synthase\_hybrid | BGC0000402 | NRP | 33.0 | 25.4 | 230.0 | 4.57e-60 |
| CAG23965.1 | polyketide\_synthase\_type\_I | BGC0000181 | Polyketide | 31.0 | 27.7 | 229.0 | 4.75e-60 |
| XP\_001220460.1 | uncharacterized\_protein | BGC0001182 | NRP+Polyketide:Iterative type I polyketide | 31.0 | 41.2 | 230.0 | 4.82e-60 |
| AJQ95676.1 | polyketide\_synthase\_modules-related\_protein | BGC0002046 | NRP+Polyketide:Trans-AT type I polyketide | 33.0 | 25.1 | 230.0 | 6.6e-60 |
| ATX68126.1 | malonyl\_CoA-acyl\_carrier\_protein\_transacylase | BGC0001795 | Polyketide | 34.0 | 24.6 | 229.0 | 8.14e-60 |
| BAP05596.1 | calH | BGC0000967 | NRP+Polyketide:Trans-AT type I polyketide | 31.0 | 26.1 | 229.0 | 8.32e-60 |
| MCC5026026.1 | polyketide\_synthase\_dehydratase\_domain-containing\_protein | BGC0002118 | NRP+Polyketide | 34.0 | 24.9 | 229.0 | 8.44e-60 |
| MCC5025982.1 | polyketide\_synthase\_dehydratase\_domain-containing\_protein | BGC0002119 | NRP+Polyketide | 34.0 | 24.9 | 229.0 | 8.44e-60 |
| ABC34832.1 | polyketide\_synthase | BGC0000186 | NRP+Polyketide:Modular type I polyketide | 33.0 | 25.3 | 229.0 | 1.13e-59 |
| CAN93349.1 | polyketide\_synthase | BGC0000179 | Polyketide:Trans-AT type I polyketide | 31.0 | 27.5 | 229.0 | 1.3e-59 |
| RAT98518.1 | trans-acyltransferase\_polyketide\_synthase | BGC0001470 | Polyketide:Trans-AT type I polyketide | 32.0 | 26.8 | 228.0 | 2.19e-59 |
| ATY69600.1 | type\_I\_polyketide\_synthase | BGC0001823 | NRP+Polyketide | 33.0 | 25.9 | 227.0 | 2.77e-59 |
| AAF08795.1 | MycA | BGC0001103 | NRP+Polyketide | 31.0 | 30.8 | 228.0 | 3.03e-59 |
| SKB24637.1 |  | BGC0002455 | Polyketide | 32.0 | 27.8 | 226.0 | 1.13e-58 |
| MCC5026027.1 | amino\_acid\_adenylation\_domain-containing\_protein | BGC0002118 | NRP+Polyketide | 32.0 | 27.2 | 225.0 | 1.18e-58 |
| AEC04363.1 | polyketide\_synthase | BGC0000178 | Polyketide:Trans-AT type I polyketide | 31.0 | 25.0 | 226.0 | 1.21e-58 |
| KZM69122.1 | beta-ketoacyl\_synthase | BGC0002352 | Other | 33.0 | 25.7 | 210.0 | 1.4e-58 |
| MCC5025983.1 | amino\_acid\_adenylation\_domain-containing\_protein | BGC0002119 | NRP+Polyketide | 32.0 | 27.2 | 225.0 | 1.58e-58 |
| WP\_013310341.1 | aminotransferase\_class\_III-fold\_pyridoxal\_phosphate-dependent\_enzyme | BGC0001728 | NRP+Polyketide | 30.0 | 32.8 | 224.0 | 1.75e-58 |
| AEW98133.1 | putative\_type-I\_PKS | BGC0002642 | Alkaloid | 33.0 | 27.4 | 216.0 | 2.45e-58 |
| AIJ04685.1 | polyketide\_synthase | BGC0001383 | Polyketide | 33.0 | 25.0 | 223.0 | 6.1e-58 |
| ALJ49922.1 | TtmG | BGC0001236 | Polyketide | 32.0 | 30.7 | 214.0 | 8.05e-58 |
| SKB24636.1 |  | BGC0002455 | Polyketide | 33.0 | 26.8 | 223.0 | 8.29e-58 |
| ABS74181.1 | bacillomycin\_D\_synthetase\_A\_ | BGC0001090 | Polyketide+NRP:Lipopeptide | 31.0 | 25.7 | 223.0 | 1.02e-57 |
| BAB69698.1 | iturin\_A\_synthetase\_A | BGC0001098 | NRP+Polyketide | 31.0 | 25.7 | 223.0 | 1.02e-57 |
| CAG23969.1 | polyketide\_synthase\_type\_I | BGC0000181 | Polyketide | 33.0 | 25.0 | 222.0 | 1.38e-57 |
| ARR97037.1 | SphD | BGC0001780 | NRP | 32.0 | 27.1 | 221.0 | 1.58e-57 |
| AKQ22670.1 | malonyl\_CoA-acyl\_carrier\_protein\_transacylase | BGC0001656 | Polyketide | 32.0 | 24.8 | 220.0 | 5.34e-57 |
| ABM63537.1 | BryA | BGC0000174 | Polyketide | 32.0 | 26.8 | 220.0 | 5.46e-57 |
| AAN85522.1 | hybrid\_nonribosomal\_peptide\_synthetase\_/\_polyketide\_synthase | BGC0001101 | NRP+Polyketide:Modular type I polyketide+Polyketide:Trans-AT type I polyketide | 33.0 | 27.1 | 219.0 | 9.13e-57 |
| BAD55609.1 | putative\_polyketide\_synthase | BGC0001027 | NRP+Polyketide | 33.0 | 25.5 | 204.0 | 9.67e-57 |
| ACY06292.1 | modular\_polyketide\_synthase | BGC0001042 | NRP+Polyketide | 27.0 | 46.3 | 218.0 | 1.56e-56 |
| BAL90255.1 | putative\_beta-ketoacyl\_synthase | BGC0002021 | Polyketide | 34.0 | 25.5 | 208.0 | 2.08e-56 |
| AAY39344.1 | Beta-ketoacyl\_synthase:Beta-ketoacyl\_synthase:Phosphopantetheine-binding\_protein | BGC0002060 | Polyketide:Trans-AT type I polyketide | 33.0 | 25.0 | 218.0 | 2.4e-56 |
| QIE07367.1 | polyketide\_synthase\_NecH | BGC0002050 | NRP+Polyketide:Trans-AT type I polyketide | 32.0 | 25.5 | 218.0 | 2.58e-56 |
| AKQ22697.1 | malonyl\_CoA-acyl\_carrier\_protein\_transacylase | BGC0001186 | Polyketide | 30.0 | 27.6 | 217.0 | 4.63e-56 |
| ELY54\_RS14405 | SDR\_family\_NAD(P)-dependent\_oxidoreductase | BGC0002087 | NRP+Polyketide | 31.0 | 27.2 | 216.0 | 1.16e-55 |
| AAY89050.1 | polyketide\_synthase | BGC0001069 | NRP+Polyketide:Trans-AT type I polyketide | 34.0 | 24.9 | 216.0 | 1.18e-55 |
| DAC80073.1 | PKS | BGC0001835 | Polyketide | 31.0 | 27.5 | 215.0 | 1.96e-55 |
| KAF1058474.1 | Polyketide\_synthase\_PksN | BGC0002083 | Polyketide:Trans-AT type I polyketide | 31.0 | 27.2 | 215.0 | 2.07e-55 |
| ARR97036.1 | SphC | BGC0001780 | NRP | 32.0 | 24.8 | 214.0 | 3.22e-55 |
| ABM63530.1 | BryD | BGC0000174 | Polyketide | 32.0 | 27.0 | 214.0 | 3.69e-55 |
| AJQ95674.1 | polyketide\_synthase\_modules-related\_protein | BGC0002046 | NRP+Polyketide:Trans-AT type I polyketide | 32.0 | 24.9 | 212.0 | 4.31e-55 |
| AAY32966.1 | DszC | BGC0001093 | NRP+Polyketide | 32.0 | 27.4 | 212.0 | 1.93e-54 |
| QLG04868.1 | PulG | BGC0002374 | Polyketide | 32.0 | 24.9 | 212.0 | 2.16e-54 |
| AAM12925.2 | MmpE | BGC0000182 | Polyketide:Iterative type I polyketide+Polyketide:Trans-AT type I polyketide | 33.0 | 25.8 | 209.0 | 3.4e-54 |
| ABS90472.1 | PKS | BGC0001106 | NRP+Polyketide | 30.0 | 32.6 | 209.0 | 3.52e-54 |
| QLG04869.1 | PulH | BGC0002374 | Polyketide | 31.0 | 32.6 | 211.0 | 3.99e-54 |
| ABC34599.1 | polyketide\_synthase,\_putative | BGC0000186 | NRP+Polyketide:Modular type I polyketide | 33.0 | 27.1 | 209.0 | 7.03e-54 |
| AFX60313.1 | polyketide\_synthase | BGC0001031 | NRP+Polyketide | 30.0 | 28.0 | 209.0 | 1.11e-53 |
| QRN75756.1 | Short-chain\_dehydrogenase/reductase\_SDR | BGC0002114 | NRP+Polyketide | 31.0 | 27.7 | 206.0 | 3.98e-53 |
| CCG06108.1 | type\_I\_polyketide\_synthase | BGC0001543 | Polyketide | 31.0 | 25.2 | 204.0 | 7.77e-53 |
| XP\_011392698.1 | uncharacterized\_protein | BGC0001281 | Polyketide | 28.0 | 31.9 | 205.0 | 1.4e-52 |
| CAG23961.2 | polyketide\_synthase\_of\_type\_I | BGC0001089 | Polyketide+NRP | 29.0 | 24.9 | 205.0 | 2.05e-52 |
| ABS75102.2 | MBL\_fold\_metallo-hydrolase | BGC0002641 | NRP | 30.0 | 25.4 | 200.0 | 5.12e-52 |
| KAF1058475.1 | Polyketide\_synthase\_PksL | BGC0002083 | Polyketide:Trans-AT type I polyketide | 32.0 | 25.5 | 203.0 | 6.91e-52 |
| WP\_126241644.1 | SDR\_family\_oxidoreductase | BGC0002087 | NRP+Polyketide | 32.0 | 25.5 | 203.0 | 6.91e-52 |
| CCA89326.1 | mixed\_trans-AT\_type\_I\_polyketide\_synthase/nonribosomal\_peptide\_synthetase | BGC0001111 | NRP+Polyketide:Trans-AT type I polyketide | 31.0 | 27.0 | 202.0 | 1.54e-51 |
| ABC38737.1 | polyketide\_synthase | BGC0000964 | NRP:Cyclic depsipeptide+Polyketide:Trans-AT type I polyketide | 30.0 | 37.7 | 201.0 | 2e-51 |
| AHD05620.1 | putative\_polyketide\_synthase\_subunit | BGC0001033 | NRP+Polyketide | 25.0 | 39.6 | 197.0 | 2.83e-50 |
| CAL69889.1 | RhiB\_protein | BGC0001112 | NRP+Polyketide:Trans-AT type I polyketide | 32.0 | 24.9 | 197.0 | 4.76e-50 |
| OEI73460.1 | hypothetical\_protein | BGC0001520 | Polyketide | 33.0 | 20.3 | 194.0 | 6.48e-49 |
| AKQ22680.1 | malonyl\_CoA-acyl\_carrier\_protein\_transacylase | BGC0001656 | Polyketide | 30.0 | 24.8 | 193.0 | 8.7e-49 |
| BAC76470.1 | type\_I\_polyketide\_synthase\_LkcG | BGC0001100 | NRP+Polyketide | 31.0 | 24.4 | 190.0 | 8.76e-49 |
| CBK62729.1 |  | BGC0001115 | NRP+Polyketide | 30.0 | 27.5 | 192.0 | 9.35e-49 |
| ABP57746.1 | DepB | BGC0000993 | NRP:Cyclic depsipeptide+Polyketide:Modular type I polyketide | 31.0 | 25.5 | 192.0 | 1.36e-48 |
| QIE07362.1 | polyketide\_synthase\_NecC | BGC0002050 | NRP+Polyketide:Trans-AT type I polyketide | 32.0 | 25.5 | 191.0 | 2.16e-48 |
| AFX60317.1 | polyketide\_synthase | BGC0001031 | NRP+Polyketide | 31.0 | 25.5 | 190.0 | 7.39e-48 |
| AFX60340.1 | polyketide\_synthase | BGC0001032 | NRP+Polyketide | 31.0 | 24.6 | 187.0 | 4.91e-47 |
| AXA20090.1 | hybrid\_trans-AT\_PKS/NRPS\_LgaA | BGC0001646 | NRP+Polyketide | 30.0 | 28.0 | 183.0 | 8.88e-46 |
| AFR69332.1 | polyketide\_synthase\_SpiB | BGC0001045 | NRP:Cyclic depsipeptide+Polyketide:Modular type I polyketide | 31.0 | 25.2 | 179.0 | 8.15e-45 |
| CAJ76285.1 | putative\_polyketide\_synthase | BGC0000972 | NRP+Polyketide:Modular type I polyketide+Polyketide:Trans-AT type I polyketide | 33.0 | 22.5 | 174.0 | 8.05e-44 |
| ADN68483.1 | sorH | BGC0000184 | Polyketide:Trans-AT type I polyketide | 30.0 | 25.5 | 172.0 | 1.73e-42 |
| AGC65514.1 | TtcB | BGC0001876 | NRP | 29.0 | 26.9 | 172.0 | 1.84e-42 |
| QUS58936.1 | non-ribosomal\_peptide\_synthetase | BGC0002123 | NRP+Polyketide | 28.0 | 26.0 | 170.0 | 8.07e-42 |
| AGC65516.1 | NRPS/PKS\_hybrid | BGC0001050 | NRP:Lipopeptide+Polyketide:Trans-AT type I polyketide | 30.0 | 27.6 | 170.0 | 1.22e-41 |
| ATY69551.1 | hybrid\_nonribosomal\_peptide\_synthetase/type\_I\_polyketide\_synthase | BGC0001611 | NRP+Polyketide | 31.0 | 25.0 | 169.0 | 1.25e-41 |
| DAB41484.1 | nonribosomal\_peptide\_synthetase/polyketide\_synthase\_type\_I | BGC0001230 | NRP:Cyclic depsipeptide+Polyketide:Modular type I polyketide | 28.0 | 34.7 | 166.0 | 1.94e-40 |
| AAO39107.1 | AdmM | BGC0000956 | NRP:Beta-lactam+Polyketide:Type II polyketide | 28.0 | 25.0 | 155.0 | 1.05e-37 |
| AAQ17110.2 | enediyne\_polyketide\_synthase | BGC0001008 | Polyketide:Iterative type I polyketide+Polyketide:Enediyne type I polyketide | 24.0 | 48.1 | 156.0 | 1.11e-37 |
| ADN68484.1 | sorI | BGC0000184 | Polyketide:Trans-AT type I polyketide | 26.0 | 30.0 | 150.0 | 7.24e-36 |
| QQZ01636.1 | PKS | BGC0002497 | Other | 27.0 | 27.3 | 148.0 | 3.62e-35 |
| ACI12950.1 | PfaC | BGC0000861 | Other | 27.0 | 27.1 | 138.0 | 4.79e-32 |
| MCI2961728.1 | 3-hydroxyacyl-[acyl-carrier-protein]\_dehydratase\_FabA | BGC0002648 | Polyketide | 27.0 | 27.0 | 137.0 | 6.23e-32 |
| ABC87512.1 | polyketide\_synthase | BGC0001011 | NRP+Polyketide | 26.0 | 30.4 | 121.0 | 6.08e-27 |
| CAM00062.1 | EryAI\_Erythromycin\_polyketide\_synthase\_modules\_1\_and\_2 | BGC0000055 | Polyketide:Modular type I polyketide+Saccharide:Hybrid/tailoring saccharide | 27.0 | 22.2 | 117.0 | 1.55e-25 |
