# Supplementary material for: Uncovering the transcriptional landscape of Fomes fomentarius during fungal-based material production through gene co-expression network analysis
Source: Fungal Biol Biotechnol. 2025 Feb 13;12:1. doi: 10.1186/s40694-024-00192-3 (PMC11827164; doi:10.1186/s40694-024-00192-3)
Supplement: Supplementary file 1 — Supplementary Material 1 [file 40694_2024_192_MOESM1_ESM.zip › knownclusterblast/region1/jgi.p_Fomfom1_1371341_mibig_hits.html]

| MIBiG Protein | Description | MIBiG Cluster | MiBiG Product | % ID | % Coverage | BLAST Score | E-value |
| --- | --- | --- | --- | --- | --- | --- | --- |
| EHK18435.1 | hypothetical\_protein | BGC0002233 | Polyketide | 35.0 | 97.5 | 139.0 | 1.97e-39 |
| EHA52498.1 | hypothetical\_protein | BGC0001749 | Polyketide | 33.0 | 98.6 | 131.0 | 3.19e-36 |
| AGZ20196.1 | short\_chain\_dehydrogenase | BGC0002618 | Terpene | 37.0 | 84.7 | 126.0 | 1.36e-34 |
| EAA36368.1 | short-chain\_dehydrogenase/reductase | BGC0002729 | Polyketide | 33.0 | 95.4 | 127.0 | 1.5e-34 |
| AMJ52082.1 | lijC | BGC0002255 | Polyketide | 33.0 | 95.4 | 123.0 | 2.61e-33 |
| KAF7526513.1 | hypothetical\_protein | BGC0002244 | Polyketide | 31.0 | 91.1 | 121.0 | 1.4e-32 |
| QGA70091.1 | putative\_oxidoreductase | BGC0002517 | Polyketide | 36.0 | 89.0 | 116.0 | 1.22e-30 |
| ABI75134.1 | short-chain\_alcohol\_dehydrogenase | BGC0000887 | Alkaloid | 38.0 | 65.1 | 100.0 | 4.82e-25 |
| KDN80075.1 | 3-ketoacyl-ACP\_reductase | BGC0001074 | Saccharide+Polyketide | 35.0 | 65.5 | 99.0 | 4.25e-24 |
| AAP11945.1 | Short\_chain\_dehydrogenase | BGC0000612 | RiPP:Thiopeptide | 31.0 | 80.4 | 99.0 | 4.38e-24 |
| EDY47129.1 | clavaldehyde\_dehydrogenase | BGC0000845 | Other:Non-NRP beta-lactam | 32.0 | 71.5 | 97.0 | 1.3e-23 |
| ALJ49935.1 | TtmK | BGC0001236 | Polyketide | 34.0 | 65.8 | 97.0 | 3.05e-23 |
| ABI75108.1 | short-chain\_alcohol\_dehydrogenase | BGC0000188 | Alkaloid | 33.0 | 82.6 | 91.0 | 2.56e-21 |
| AWH12904.1 | KR\_domain-containing\_protein | BGC0001784 | Polyketide | 31.0 | 90.7 | 91.0 | 5.35e-21 |
| AMK92578.1 | oxidoreductase | BGC0001377 | Polyketide | 33.0 | 69.0 | 90.0 | 8.77e-21 |
| CAL80830.1 | dehydrogenase-related\_protein | BGC0000997 | NRP+Polyketide | 27.0 | 90.7 | 90.0 | 1.12e-20 |
| AXA20103.1 | ketoreductase\_LgaK | BGC0001646 | NRP+Polyketide | 33.0 | 70.5 | 87.0 | 7.24e-20 |
| OOH83075.1 | 3-oxoacyl-ACP\_reductase | BGC0001987 | Polyketide | 34.0 | 68.0 | 86.0 | 1.67e-19 |
| QTA30590.1 | short\_chain\_dehydrogenase | BGC0002143 | Polyketide | 31.0 | 93.2 | 86.0 | 2.97e-19 |
| AME18007.1 | oxidoreductase | BGC0001378 | Polyketide:Enediyne type I polyketide | 32.0 | 68.7 | 86.0 | 3.98e-19 |
| ABL09970.1 | oxidoreductase | BGC0000197 | Polyketide:Type II polyketide+Saccharide:Hybrid/tailoring saccharide | 30.0 | 69.0 | 85.0 | 6.86e-19 |
| WP\_051892705.1 | 7-ketoreductase | BGC0001851 | Polyketide:Type II polyketide+Saccharide:Hybrid/tailoring saccharide | 34.0 | 67.3 | 83.0 | 2.98e-18 |
| AXL88813.1 | ketoacyl\_reductase | BGC0001895 | Polyketide | 33.0 | 69.0 | 82.0 | 4.05e-18 |
| AJI44188.1 | hypothetical\_protein | BGC0001193 | NRP | 37.0 | 67.3 | 82.0 | 4.84e-18 |
| AAF73457.1 | putative\_aklaviketone\_reductase | BGC0000193 | Polyketide | 30.0 | 72.6 | 82.0 | 6.02e-18 |
| ARF06204.1 | short-chain\_dehydrogenase/reductase\_SDR | BGC0001593 | NRP | 30.0 | 64.8 | 81.0 | 9.11e-18 |
| AJS09392.1 | Ketoacyl-(acyl-carrier-protein)\_reductase | BGC0001150 | Polyketide:Type II polyketide+Polyketide:Type III polyketide | 32.0 | 66.9 | 82.0 | 9.3e-18 |
| WP\_016640238.1 | 3-oxoacyl-ACP\_reductase\_FabG | BGC0002000 | Polyketide | 30.0 | 77.6 | 81.0 | 1.44e-17 |
| QNL10616.1 | Ketoacyl\_reductase | BGC0002514 | Polyketide | 32.0 | 77.6 | 81.0 | 1.99e-17 |
| ATJ00769.1 | C-7\_ketoreductase | BGC0001568 | Polyketide | 34.0 | 70.8 | 80.0 | 2.25e-17 |
| AFN69430.1 | ElxO | BGC0000509 | RiPP:Lanthipeptide | 32.0 | 66.2 | 79.0 | 4.27e-17 |
| WP\_051808697.1 | 7-ketoreductase | BGC0001852 | Polyketide:Type II polyketide+Saccharide:Hybrid/tailoring saccharide | 33.0 | 62.3 | 79.0 | 7.35e-17 |
| ABO15851.1 | oxidoreductase | BGC0000130 | Polyketide | 29.0 | 90.0 | 79.0 | 1.35e-16 |
| BAB69696.1 |  | BGC0001098 | NRP+Polyketide | 29.0 | 77.2 | 78.0 | 1.35e-16 |
| ATJ00771.1 | ketoacyl\_reductase | BGC0001568 | Polyketide | 32.0 | 70.1 | 78.0 | 1.35e-16 |
| WP\_018891734.1 | SDR\_family\_oxidoreductase | BGC0001558 | Polyketide | 34.0 | 70.1 | 78.0 | 1.43e-16 |
| AHA81975.1 | Ketoreductase | BGC0000199 | Polyketide:Type II polyketide+Saccharide:Hybrid/tailoring saccharide | 30.0 | 69.0 | 78.0 | 1.71e-16 |
| ACX83621.1 | keto\_reductase | BGC0000221 | Polyketide | 29.0 | 76.5 | 78.0 | 1.86e-16 |
| KDN80050.1 | ketoacyl\_reductase | BGC0001074 | Saccharide+Polyketide | 32.0 | 69.8 | 78.0 | 1.86e-16 |
| WP\_008704360.1 | SDR\_family\_oxidoreductase | BGC0001575 | NRP | 33.0 | 60.1 | 77.0 | 2.22e-16 |
| EDY42546.1 | ketoreductase | BGC0000212 | Polyketide:Type II polyketide | 32.0 | 70.1 | 78.0 | 2.27e-16 |
| WP\_020275098.1 | SDR\_family\_NAD(P)-dependent\_oxidoreductase | BGC0002012 | Polyketide | 31.0 | 71.2 | 77.0 | 2.56e-16 |
| QOP59273.1 | keto-reductase | BGC0002504 | Polyketide | 33.0 | 67.3 | 77.0 | 2.56e-16 |
| ABS74183.1 | YxjF | BGC0001090 | Polyketide+NRP:Lipopeptide | 28.0 | 76.9 | 77.0 | 3.52e-16 |
| AHN91930.1 | short-chain\_dehydrogenase/reductase\_SDR | BGC0000340 | NRP | 30.0 | 64.4 | 77.0 | 3.72e-16 |
| QBA57755.1 | 3-oxoacyl-reductase | BGC0002377 | NRP | 32.0 | 69.4 | 77.0 | 4.02e-16 |
| ADZ24992.1 | dehydrogenase | BGC0000380 | NRP+Polyketide:Modular type I polyketide | 30.0 | 69.8 | 77.0 | 4.32e-16 |
| CCH32748.1 | Ketoreductase | BGC0002070 | Polyketide | 32.0 | 69.0 | 77.0 | 4.7e-16 |
| BAI63274.1 | putative\_oxidoreductase | BGC0000434 | NRP | 31.0 | 62.6 | 76.0 | 5.77e-16 |
| CBA63665.1 | 2,3-dihydro-2,3-dihydroxybenzoate\_dehydrogenase | BGC0000368 | NRP | 34.0 | 70.1 | 77.0 | 5.88e-16 |
| ACR12253.1 | 2,3-dihydro-2,3-dihydroxybenzoate\_dehydrogenase | BGC0000451 | NRP | 28.0 | 69.0 | 76.0 | 6.11e-16 |
| CAG44632.1 | SriL03.9 | BGC0000712 | Saccharide | 30.0 | 68.0 | 77.0 | 6.55e-16 |
| AAG06718.1 | probable\_short\_chain\_dehydrogenase | BGC0002037 | NRP | 27.0 | 90.4 | 77.0 | 8.05e-16 |
| EDY42534.1 | monensin\_polyketide\_synthase\_ketoacyl\_reductase | BGC0000212 | Polyketide:Type II polyketide | 32.0 | 74.7 | 76.0 | 9.12e-16 |
| BCN28648.1 | 3-oxoacyl-ACP\_reductase | BGC0002441 | Polyketide+NRP | 29.0 | 70.5 | 76.0 | 1.12e-15 |
| AQW35064.1 | Polyketide\_C-9\_ketoreductase | BGC0001675 | Polyketide | 31.0 | 67.6 | 76.0 | 1.27e-15 |
| BAB72043.1 | AknA | BGC0000191 | Polyketide | 30.0 | 73.0 | 75.0 | 1.72e-15 |
| AAL24452.1 | RdmJ | BGC0000265 | Polyketide | 30.0 | 73.0 | 75.0 | 1.79e-15 |
| QFS19054.1 | ketoreductase | BGC0002506 | Polyketide | 30.0 | 71.2 | 75.0 | 1.81e-15 |
| KDN80052.1 | ketoreductase | BGC0001074 | Saccharide+Polyketide | 33.0 | 70.8 | 75.0 | 1.84e-15 |
| CAJ42322.1 | ketoreductase | BGC0000273 | Polyketide:Type II polyketide+Saccharide:Hybrid/tailoring saccharide | 29.0 | 70.1 | 75.0 | 2.03e-15 |
| CAH10175.1 | ChaZ\_protein | BGC0000207 | Polyketide | 31.0 | 71.2 | 75.0 | 2.36e-15 |
| BAC79042.1 | keto\_reductase\_(KR) | BGC0000245 | Polyketide | 31.0 | 73.3 | 75.0 | 2.36e-15 |
| QLQ40537.1 | SDR\_family\_oxidoreductase | BGC0002097 | NRP+Polyketide:Type II polyketide+Saccharide:Hybrid/tailoring saccharide | 32.0 | 75.8 | 74.0 | 2.52e-15 |
| QCX41951.1 | Amc14 | BGC0001957 | Polyketide | 30.0 | 64.8 | 74.0 | 2.56e-15 |
| QHW08548.1 | SDR\_family\_oxidoreductase | BGC0002054 | Polyketide+NRP+Saccharide | 30.0 | 64.8 | 74.0 | 2.56e-15 |
| ADE34504.1 | ssfK | BGC0000269 | Polyketide:Type II polyketide+Saccharide:Hybrid/tailoring saccharide | 32.0 | 68.3 | 74.0 | 2.64e-15 |
| ADE34491.1 | ssfU | BGC0000269 | Polyketide:Type II polyketide+Saccharide:Hybrid/tailoring saccharide | 30.0 | 70.1 | 74.0 | 3.24e-15 |
| ALG65299.1 | Cal37 | BGC0001297 | NRP | 31.0 | 70.8 | 74.0 | 3.73e-15 |
| SCN11974.1 | short-chain\_dehydrogenase/reductase\_SDR | BGC0001580 | Polyketide | 30.0 | 68.0 | 74.0 | 3.89e-15 |
| WP\_040253449.1 | SDR\_family\_NAD(P)-dependent\_oxidoreductase | BGC0001596 | Polyketide | 31.0 | 67.6 | 74.0 | 4.61e-15 |
| CAK50783.1 | ketoreductase | BGC0000247 | Polyketide:Type II polyketide+Saccharide:Oligosaccharide | 35.0 | 63.0 | 74.0 | 5.05e-15 |
| ABY83179.1 | Azi41 | BGC0000960 | NRP+Polyketide | 29.0 | 82.2 | 74.0 | 5.19e-15 |
| AJW65399.1 | oxidoreductase | BGC0001195 | NRP+Polyketide | 29.0 | 67.3 | 74.0 | 5.19e-15 |
| AQW35069.1 | ketoreductase | BGC0001675 | Polyketide | 32.0 | 65.1 | 74.0 | 5.7e-15 |
| PPQ57492.1 | ketoacyl\_reductase | BGC0002016 | Polyketide | 29.0 | 75.4 | 74.0 | 6.09e-15 |
| TRO56980.1 | SDR\_family\_NAD(P)-dependent\_oxidoreductase | BGC0002361 | Polyketide+Saccharide | 31.0 | 67.6 | 74.0 | 6.48e-15 |
| CAA09652.1 | polyketide\_ketoreductase | BGC0000227 | Polyketide:Type II polyketide | 30.0 | 76.5 | 74.0 | 6.97e-15 |
| AHL24460.1 | short-chain\_dehydrogenase/reductase\_SDR | BGC0000806 | Saccharide | 31.0 | 75.4 | 73.0 | 7.42e-15 |
| ADB02846.1 | AzicD | BGC0000202 | Polyketide | 32.0 | 68.7 | 73.0 | 7.93e-15 |
| CAH10114.1 | putative\_ketoreducatse | BGC0000268 | Polyketide | 31.0 | 67.6 | 73.0 | 8.88e-15 |
| QLK01228.1 | SDR\_family\_oxidoreductase | BGC0002353 | RiPP | 30.0 | 69.4 | 73.0 | 1.1e-14 |
| AFJ52671.1 | ketoreductase | BGC0001073 | NRP+Polyketide | 29.0 | 76.5 | 73.0 | 1.11e-14 |
| AAO65349.1 | putative\_ketoreductase | BGC0000236 | Polyketide | 31.0 | 69.8 | 72.0 | 1.57e-14 |
| AVO00812.1 | May13 | BGC0001661 | Polyketide | 31.0 | 69.8 | 72.0 | 1.59e-14 |
| CAJ34364.1 | NAD\_or\_NADP\_oxidoreductase | BGC0000445 | NRP:Cyclic depsipeptide | 31.0 | 59.4 | 72.0 | 1.62e-14 |
| OKJ61997.1 | short-chain\_dehydrogenase | BGC0002147 | NRP | 32.0 | 60.9 | 72.0 | 1.81e-14 |
| AAA65202.1 | daunorubicin-doxorubicin\_polyketide\_synthase | BGC0000218 | Polyketide | 33.0 | 69.4 | 72.0 | 1.89e-14 |
| QCX41935.1 | AmcA | BGC0001957 | Polyketide | 29.0 | 66.5 | 72.0 | 2.12e-14 |
| QHW08564.1 | SDR\_family\_oxidoreductase | BGC0002054 | Polyketide+NRP+Saccharide | 29.0 | 66.5 | 72.0 | 2.12e-14 |
| MCG7203803.1 | 3-oxoacyl-ACP\_reductase | BGC0000248 | Polyketide | 30.0 | 76.5 | 72.0 | 2.14e-14 |
| C5F59\_12930 | dehydrogenase | BGC0002016 | Polyketide | 29.0 | 78.3 | 72.0 | 2.49e-14 |
| AAZ55906.1 | 2,3-dihydro-2,3-dihydroxybenzoate\_dehydrogenase;\_RBL00455 | BGC0000359 | NRP | 32.0 | 64.8 | 72.0 | 2.9e-14 |
| AEI98650.1 | CtcG | BGC0000209 | Polyketide | 29.0 | 70.8 | 72.0 | 2.97e-14 |
| ADC45536.1 | reductase | BGC0000093 | Polyketide | 31.0 | 71.9 | 72.0 | 3.12e-14 |
| WP\_031147019.1 | 9-ketoreductase | BGC0001851 | Polyketide:Type II polyketide+Saccharide:Hybrid/tailoring saccharide | 30.0 | 69.8 | 71.0 | 4.02e-14 |
| BAA84591.1 | reductase | BGC0000025 | Polyketide | 33.0 | 53.4 | 71.0 | 4.02e-14 |
| AHD25940.1 | putative\_ketoreductase | BGC0000208 | Polyketide | 29.0 | 76.2 | 71.0 | 4.07e-14 |
| AAZ78333.1 | OxyJ | BGC0000254 | Polyketide | 29.0 | 69.4 | 71.0 | 4.12e-14 |
| BCD52387.1 | short-chain\_dehydrogenase/reductase\_SptI | BGC0002537 | Polyketide+Terpene | 34.0 | 63.7 | 71.0 | 5.5e-14 |
| WP\_030957358.1 | 9-ketoreductase | BGC0001852 | Polyketide:Type II polyketide+Saccharide:Hybrid/tailoring saccharide | 31.0 | 69.8 | 71.0 | 5.5e-14 |
| BAI70380.1 | short\_chain\_dehydrogenase | BGC0000896 | Other | 29.0 | 78.6 | 71.0 | 5.9e-14 |
| ANY57970.1 | Short\_chain\_dehydrogenase | BGC0001369 | Polyketide | 30.0 | 75.1 | 71.0 | 6.32e-14 |
| CAI94720.1 | putative\_oxyacyl-(acyl\_carrier\_protein)\_reductase | BGC0000141 | Polyketide | 29.0 | 78.3 | 71.0 | 6.48e-14 |
| QVQ68802.1 | mmyTIII | BGC0002129 | Polyketide | 30.0 | 70.8 | 71.0 | 6.57e-14 |
| TMU80021.1 | SDR\_family\_oxidoreductase | BGC0002069 | Other:Aminocoumarin | 30.0 | 60.1 | 71.0 | 7.17e-14 |
| POM23765.1 | putative\_ketoacyl\_reductase | BGC0002369 | Polyketide | 31.0 | 70.5 | 71.0 | 7.43e-14 |
| NHN68323.1 | SDR\_family\_oxidoreductase | BGC0002719 | NRP | 30.0 | 70.8 | 71.0 | 7.43e-14 |
| BBA97253.1 | putative\_ketoreductase | BGC0002383 | Polyketide | 31.0 | 67.6 | 71.0 | 7.52e-14 |
| AKT74300.1 | TxnC4 | BGC0002141 | Polyketide | 31.0 | 72.2 | 70.0 | 7.88e-14 |
| QDG00823.1 | polyketide\_ketoreductase | BGC0002028 | Polyketide | 32.0 | 67.3 | 70.0 | 1.02e-13 |
| ARD70870.1 | Polyketide\_synthesis,\_ketoreductase | BGC0001693 | Polyketide | 28.0 | 67.6 | 70.0 | 1.03e-13 |
| ANY58988.1 | short-chain\_dehydrogenase | BGC0001615 | NRP | 27.0 | 81.5 | 71.0 | 1.05e-13 |
| ADE22326.1 | dehydrogenase | BGC0000065 | Polyketide:Iterative type I polyketide | 31.0 | 66.5 | 70.0 | 1.11e-13 |
| MCG7203804.1 | SDR\_family\_oxidoreductase | BGC0000248 | Polyketide | 33.0 | 59.8 | 70.0 | 1.15e-13 |
| BAF85845.1 | putative\_oxidoreductase | BGC0000109 | Polyketide | 31.0 | 67.3 | 70.0 | 1.29e-13 |
| AEW95637.1 | hypothetical\_protein | BGC0002697 | NRP+Polyketide | 28.0 | 90.4 | 70.0 | 1.37e-13 |
| AAK57528.1 | PgaD | BGC0000262 | Polyketide:Type II polyketide+Saccharide:Hybrid/tailoring saccharide | 31.0 | 77.2 | 70.0 | 1.41e-13 |
| CAB15190.2 | 2,3-dihydro-2,3-dihydroxybenzoate\_dehydrogenase | BGC0000309 | NRP | 28.0 | 66.5 | 70.0 | 1.41e-13 |
| MUL41457.1 | SDR\_family\_oxidoreductase | BGC0002045 | Polyketide:Type II polyketide | 30.0 | 74.4 | 69.0 | 1.5e-13 |
| QED90618.1 | ketoacyl\_reductase | BGC0002081 | Polyketide | 29.0 | 68.7 | 69.0 | 2.05e-13 |
| AAL15603.1 | SimJ1 | BGC0000270 | Polyketide | 32.0 | 69.4 | 69.0 | 2.19e-13 |
| AAK06807.1 | putative\_3-keto-acyl-reductase\_SimD2 | BGC0001072 | Saccharide+Polyketide:Modular type I polyketide+Polyketide:Type II polyketide+Other:Aminocoumarin | 32.0 | 69.4 | 69.0 | 2.19e-13 |
| BAW27709.1 | dehydrogenase | BGC0001764 | NRP | 28.0 | 64.8 | 69.0 | 2.51e-13 |
| BBC20651.1 | enoyl-(acyl\_carrier\_protein)\_reductase | BGC0001917 | Polyketide | 32.0 | 67.6 | 69.0 | 3.07e-13 |
| AAF70104.1 | AknA | BGC0000192 | Polyketide | 29.0 | 73.0 | 69.0 | 3.58e-13 |
| ABL09955.1 | ketoreductase | BGC0000197 | Polyketide:Type II polyketide+Saccharide:Hybrid/tailoring saccharide | 27.0 | 70.1 | 69.0 | 3.58e-13 |
| CBH32818.1 | putative\_ketoreductase | BGC0000263 | Polyketide | 28.0 | 80.1 | 69.0 | 3.58e-13 |
| AHL46698.1 | ketoreductase | BGC0001177 | Polyketide:Type II polyketide | 30.0 | 69.8 | 69.0 | 3.58e-13 |
| AHL46733.1 | ketoreductase | BGC0001179 | Polyketide:Type II polyketide | 30.0 | 67.6 | 69.0 | 3.99e-13 |
| APR73624.1 | dehydrogenase | BGC0001625 | Polyketide | 31.0 | 67.3 | 68.0 | 4.51e-13 |
| CAG14968.1 | ketoreductase | BGC0000253 | Polyketide:Type II polyketide | 30.0 | 67.6 | 68.0 | 4.84e-13 |
| ARK36158.1 | ketoacyl\_reductase | BGC0001723 | Polyketide | 30.0 | 67.6 | 68.0 | 4.84e-13 |
| BAV17002.1 | putative\_ketoreductase | BGC0001384 | Polyketide | 30.0 | 67.6 | 68.0 | 4.89e-13 |
| AYU66237.1 | TjhC3 | BGC0002461 | Polyketide | 31.0 | 69.8 | 68.0 | 4.89e-13 |
| OKI81342.1 | ketoacyl\_reductase | BGC0002478 | Polyketide | 30.0 | 68.0 | 68.0 | 4.89e-13 |
| QTA30612.1 | sorbitol\_utilization\_protein\_SOU2 | BGC0002143 | Polyketide | 35.0 | 60.5 | 68.0 | 5.47e-13 |
| ACX35428.1 | BacC | BGC0000888 | Other | 26.0 | 66.9 | 68.0 | 6.09e-13 |
| ACP19356.1 | SaqD | BGC0000267 | Polyketide:Type II polyketide+Saccharide:Oligosaccharide | 29.0 | 70.5 | 68.0 | 6.17e-13 |
| BCP96887.1 | short-chain\_dehydrogenase | BGC0002614 | NRP+Polyketide | 30.0 | 68.7 | 68.0 | 6.24e-13 |
| ABP54645.1 | short-chain\_dehydrogenase/reductase\_SDR | BGC0000241 | Polyketide:Type II polyketide+Saccharide:Hybrid/tailoring saccharide | 29.0 | 70.8 | 68.0 | 6.67e-13 |
| QHZ32176.1 | putative\_ketoacyl\_reductase | BGC0002047 | Polyketide | 30.0 | 69.8 | 68.0 | 6.67e-13 |
| ACI88864.1 | AlnP\_ketoreductase | BGC0000195 | Polyketide:Type II polyketide | 30.0 | 69.4 | 68.0 | 6.82e-13 |
| AAD13539.1 | reductase\_homolog | BGC0000239 | Polyketide:Type II polyketide+Saccharide:Hybrid/tailoring saccharide | 29.0 | 70.1 | 67.0 | 9.1e-13 |
| ABS75820.1 | BacC | BGC0001184 | Other | 27.0 | 66.9 | 67.0 | 1.14e-12 |
| EHM27508.1 | short-chain\_dehydrogenase/reductase\_SDR | BGC0000235 | Polyketide | 30.0 | 67.6 | 67.0 | 1.18e-12 |
| OKI59859.1 | ketoacyl\_reductase | BGC0002477 | Polyketide | 30.0 | 67.6 | 67.0 | 1.24e-12 |
| ARD70866.1 | Short-chain\_dehydrogenase/reductase | BGC0001693 | Polyketide | 28.0 | 71.9 | 67.0 | 1.36e-12 |
| CBH32080.1 | putative\_polyketide\_ketoreductase | BGC0000211 | Polyketide | 29.0 | 69.8 | 67.0 | 1.69e-12 |
| QTA30588.1 | SDR\_family\_NAD(P)-dependent\_oxidoreductase | BGC0002143 | Polyketide | 33.0 | 69.0 | 67.0 | 1.69e-12 |
| ALG65315.1 | Cal21 | BGC0001297 | NRP | 32.0 | 47.7 | 67.0 | 1.95e-12 |
| AAS79466.1 | putative\_post-PKS\_ketoreductase | BGC0000035 | Polyketide | 31.0 | 67.3 | 66.0 | 2e-12 |
| CCM44333.1 | 3-oxoacyl-ACP\_reductase | BGC0001056 | NRP+Polyketide:Modular type I polyketide+Polyketide:PUFA synthase or related polyketide | 32.0 | 68.0 | 66.0 | 2.16e-12 |
| PHM26609.1 | 3-ketoacyl-ACP-reductase\_CylG | BGC0001130 | NRP+Polyketide | 29.0 | 76.5 | 66.0 | 2.16e-12 |
| EHM27499.1 | putative\_short\_chain\_oxidoreductase | BGC0000235 | Polyketide | 35.0 | 44.1 | 66.0 | 2.33e-12 |
| QFS19040.1 | OxyM\_family\_protein | BGC0002506 | Polyketide | 28.0 | 71.2 | 66.0 | 2.95e-12 |
| AQZ26590.1 | 2,3-dihydro-2,3-dihydroxybenzoate\_dehydrogenase | BGC0001437 | NRP | 27.0 | 68.3 | 66.0 | 2.98e-12 |
| BBG28485.1 | short-chain\_dehydrogenase/reductase\_CdmF | BGC0001926 | Polyketide | 30.0 | 67.3 | 66.0 | 2.98e-12 |
| AEE65465.1 | ketoreductase | BGC0000223 | Polyketide:Type II polyketide | 33.0 | 67.3 | 66.0 | 3.11e-12 |
| CAC44199.1 | ketoacyl\_reductase | BGC0000194 | Polyketide:Type II polyketide | 29.0 | 70.5 | 66.0 | 3.14e-12 |
| AGO50613.1 | ketoreductase | BGC0000229 | Polyketide:Type II polyketide+Saccharide:Hybrid/tailoring saccharide | 28.0 | 69.8 | 66.0 | 3.14e-12 |
| BAJ07854.1 | putative\_ketoreductase | BGC0000232 | Polyketide | 28.0 | 69.8 | 66.0 | 3.14e-12 |
| ARO44671.1 | ketoreductase | BGC0001769 | Polyketide | 29.0 | 69.8 | 66.0 | 3.14e-12 |
| MBW8699686.1 | putative\_ketoacyl\_reductase | BGC0002140 | Polyketide | 29.0 | 69.8 | 66.0 | 3.14e-12 |
| ABB52548.1 | 3-oxoacyl-(acyl-carrier-protein)-reductase | BGC0000047 | Polyketide | 29.0 | 68.7 | 66.0 | 3.72e-12 |
| AHY06369.1 | 3-oxoacyl-ACP\_reductase | BGC0002496 | NRP | 31.0 | 65.5 | 66.0 | 3.72e-12 |
| UPN68084.1 | reductase | BGC0002672 | Polyketide | 31.0 | 68.7 | 66.0 | 4.24e-12 |
| CAA60458.1 | ketoreductase/dehydrogenase | BGC0001040 | NRP+Polyketide | 29.0 | 72.6 | 66.0 | 4.41e-12 |
| ABY83162.1 | Azi24 | BGC0000960 | NRP+Polyketide | 35.0 | 41.3 | 66.0 | 4.76e-12 |
| SEG87456.1 | ketoreductase | BGC0002712 | Polyketide | 31.0 | 70.1 | 66.0 | 4.98e-12 |
| ABO15849.1 | oxidoreductase | BGC0000130 | Polyketide | 27.0 | 72.2 | 65.0 | 5.77e-12 |
| WP\_037817190.1 | 3-oxoacyl-ACP\_reductase\_FabG | BGC0002137 | Polyketide | 29.0 | 67.3 | 65.0 | 5.83e-12 |
| WP\_010369414.1 | SDR\_family\_oxidoreductase | BGC0000314 | Polyketide+NRP:Cyclic depsipeptide+Other:Aminocoumarin | 29.0 | 57.7 | 65.0 | 6.53e-12 |
| QBA57739.1 | NAD(P)-dependent\_oxidoreductase | BGC0002377 | NRP | 31.0 | 46.6 | 65.0 | 6.6e-12 |
| ACN38371.1 | short-chain\_dehydrogenase/reductase | BGC0000714 | Saccharide | 29.0 | 69.0 | 65.0 | 6.68e-12 |
| CAA74346.1 | oxidoreductase | BGC0000507 | RiPP:Lanthipeptide | 28.0 | 65.5 | 65.0 | 6.84e-12 |
| BAU98027.1 | dehydrogenase | BGC0001386 | Polyketide | 30.0 | 66.9 | 65.0 | 6.84e-12 |
| AAL15583.1 | Sim5 | BGC0000270 | Polyketide | 30.0 | 68.7 | 65.0 | 7.93e-12 |
| AAK06787.1 | putative\_ketoreductase\_SimA6 | BGC0001072 | Saccharide+Polyketide:Modular type I polyketide+Polyketide:Type II polyketide+Other:Aminocoumarin | 30.0 | 68.7 | 65.0 | 7.93e-12 |
| OKI81335.1 | short-chain\_dehydrogenase | BGC0002478 | Polyketide | 33.0 | 43.8 | 64.0 | 8.1e-12 |
| AAB36565.1 | ketoreductase | BGC0000234 | Polyketide | 27.0 | 67.6 | 65.0 | 8.17e-12 |
| ABB69757.1 | PlaU | BGC0000654 | Terpene+Saccharide:Hybrid/tailoring saccharide | 29.0 | 69.0 | 64.0 | 9.01e-12 |
| ADG86311.1 | 3-ketoacyl\_ACP-reductase | BGC0000190 | Polyketide | 28.0 | 82.9 | 64.0 | 9.21e-12 |
| KGA48900.1 | 2,3-dihydro-2,3-dihydroxybenzoate\_dehydrogenase | BGC0002413 | NRP | 25.0 | 64.4 | 64.0 | 9.73e-12 |
| ADE22339.1 | iterative\_type\_I\_PKS\_(highly\_reducing\_type) | BGC0000065 | Polyketide:Iterative type I polyketide | 31.0 | 50.2 | 66.0 | 1.16e-11 |
| BBE36453.1 | dehydrogenase | BGC0001922 | Polyketide | 28.0 | 73.7 | 64.0 | 1.45e-11 |
| BAL90288.1 | putative\_short-chain\_dehydrogenase | BGC0002021 | Polyketide | 30.0 | 68.0 | 64.0 | 1.45e-11 |
| BAL90266.1 | putative\_short-chain\_dehydrogenase | BGC0002021 | Polyketide | 31.0 | 66.2 | 64.0 | 1.71e-11 |
| AFO85456.1 | reductase/oxidase | BGC0000391 | NRP | 27.0 | 68.3 | 64.0 | 1.79e-11 |
| ALJ99868.1 | FlsQ2 | BGC0001904 | Polyketide | 30.0 | 62.6 | 64.0 | 1.8e-11 |
| AAC18111.1 | ketoreductase | BGC0000225 | Polyketide | 31.0 | 66.5 | 64.0 | 2.19e-11 |
| CAH10174.1 | ChaL\_protein | BGC0000207 | Polyketide | 29.0 | 67.6 | 63.0 | 2.33e-11 |
| QNH67547.1 | Cip19 | BGC0002108 | NRP | 31.0 | 67.3 | 63.0 | 2.38e-11 |
| CBJ82074.1 | 3-oxoacyl-[acyl-carrier-protein]\_reductase\_(3-ketoacyl-acyl\_carrier\_protein\_reductase) | BGC0001872 | Polyketide | 28.0 | 71.2 | 63.0 | 2.56e-11 |
| AHL46696.1 | ketoreductase | BGC0001177 | Polyketide:Type II polyketide | 30.0 | 66.2 | 63.0 | 2.69e-11 |
| QBK46640.1 | HrsK3 | BGC0001960 | Polyketide | 28.0 | 69.8 | 63.0 | 2.71e-11 |
| QDQ37882.1 | ketoreductase | BGC0001979 | Polyketide | 29.0 | 76.5 | 63.0 | 2.71e-11 |
| AAF23366.1 | PhaB | BGC0000866 | Other | 27.0 | 77.9 | 63.0 | 3.17e-11 |
| ANC94959.1 | AlmK | BGC0001396 | Polyketide | 29.0 | 68.7 | 63.0 | 3.24e-11 |
| CAA09651.1 |  | BGC0000227 | Polyketide:Type II polyketide | 33.0 | 59.1 | 63.0 | 3.27e-11 |
| ALJ99852.1 | FlsE | BGC0001904 | Polyketide | 29.0 | 67.6 | 63.0 | 3.65e-11 |
| QLQ36629.1 | SDR\_family\_NAD(P)-dependent\_oxidoreductase | BGC0002097 | NRP+Polyketide:Type II polyketide+Saccharide:Hybrid/tailoring saccharide | 29.0 | 67.3 | 63.0 | 3.68e-11 |
| AXM42926.1 | ketoacyl\_reductase | BGC0001940 | Polyketide | 29.0 | 68.7 | 63.0 | 3.82e-11 |
| ADI71446.1 | putative\_ketoreductase | BGC0000203 | Polyketide | 28.0 | 79.0 | 62.0 | 5e-11 |
| AHZ61856.1 | short-chain\_dehydrogenase/reductase | BGC0000240 | Polyketide:Type II polyketide+Saccharide:Hybrid/tailoring saccharide | 28.0 | 70.8 | 62.0 | 5e-11 |
| AGK13423.1 | 2,3-dihydro-2,3-dihydroxybenzoate\_dehydrogenase,\_short-chain\_dehydrogenase/reductase | BGC0002528 | NRP | 28.0 | 74.7 | 62.0 | 5.19e-11 |
| OKI59868.1 | short-chain\_dehydrogenase | BGC0002477 | Polyketide | 34.0 | 44.1 | 62.0 | 5.2e-11 |
| AAQ08912.1 | putative\_3-ketoacyl-ACP\_reductase | BGC0000224 | Polyketide:Type II polyketide | 29.0 | 68.0 | 62.0 | 5.24e-11 |
| QMX85612.1 | SapE | BGC0002510 | Polyketide | 24.0 | 68.7 | 62.0 | 5.87e-11 |
| ADE22303.1 | putative\_short-chain\_dehydrogenase/reductase\_SDR | BGC0000279 | Polyketide | 28.0 | 77.6 | 62.0 | 6.53e-11 |
| PYC67523.1 | NAD(P)-dependent\_oxidoreductase | BGC0001473 | RiPP:Thiopeptide | 30.0 | 67.6 | 62.0 | 7.41e-11 |
| AIL50168.1 | putative\_ketoreductase | BGC0000213 | Polyketide:Type II polyketide | 27.0 | 68.0 | 62.0 | 7.98e-11 |
| QDQ37876.1 | ketoreductase | BGC0001979 | Polyketide | 30.0 | 61.6 | 61.0 | 1.39e-10 |
| CAM34348.1 | putative\_short-chain\_dehydrogenase/reductase\_SDR | BGC0000242 | Polyketide | 29.0 | 68.0 | 61.0 | 1.63e-10 |
| AAF67503.1 | b-ketotyrosyl-reductase | BGC0000834 | Saccharide:Hybrid/tailoring saccharide+Other:Aminocoumarin | 30.0 | 69.4 | 61.0 | 1.71e-10 |
| ABP54641.1 | short-chain\_dehydrogenase/reductase\_SDR | BGC0000241 | Polyketide:Type II polyketide+Saccharide:Hybrid/tailoring saccharide | 28.0 | 72.6 | 61.0 | 1.73e-10 |
| CBG67532.1 | putative\_oxidoreductase | BGC0002367 | NRP | 30.0 | 46.6 | 61.0 | 1.89e-10 |
| AFJ52673.1 | C-9\_ketoreductase | BGC0001073 | NRP+Polyketide | 29.0 | 67.3 | 61.0 | 2.3e-10 |
| CBK62720.1 |  | BGC0001115 | NRP+Polyketide | 26.0 | 79.7 | 60.0 | 2.59e-10 |
| QED93096.1 | short-chain\_dehydrogenase/reductase\_SDR | BGC0002556 | Alkaloid | 28.0 | 66.2 | 60.0 | 2.69e-10 |
| CCA54213.1 | CmlJ | BGC0000893 | NRP | 25.0 | 65.8 | 60.0 | 3.19e-10 |
| AFV52172.1 | ketoreductase | BGC0000081 | NRP+Polyketide:Iterative type I polyketide+Polyketide:Enediyne type I polyketide | 28.0 | 69.8 | 60.0 | 3.69e-10 |
| AAG23281.1 | probable\_keto\_acyl\_reductase | BGC0000148 | Polyketide | 28.0 | 78.3 | 59.0 | 5.44e-10 |
| AAF81727.1 | putative\_ketoreductase\_EncD | BGC0000220 | Polyketide:Type II polyketide | 32.0 | 61.9 | 59.0 | 5.96e-10 |
| QIC03941.1 | EncD | BGC0002366 | Polyketide | 32.0 | 61.9 | 59.0 | 5.96e-10 |
| AAM12932.1 | MupS | BGC0000182 | Polyketide:Iterative type I polyketide+Polyketide:Trans-AT type I polyketide | 29.0 | 67.3 | 59.0 | 7.5e-10 |
| AKT74293.1 | TxnC2 | BGC0002141 | Polyketide | 27.0 | 69.8 | 59.0 | 7.76e-10 |
| AAO39097.1 | AdmC | BGC0000956 | NRP:Beta-lactam+Polyketide:Type II polyketide | 30.0 | 68.7 | 59.0 | 9.48e-10 |
| AAG03071.1 | putative\_reductase | BGC0000266 | Polyketide | 30.0 | 66.9 | 58.0 | 1.28e-09 |
| AUI41032.1 | 3-oxoacyl-(acyl-carrier\_protein)\_reductase | BGC0001512 | Polyketide | 30.0 | 72.2 | 58.0 | 1.36e-09 |
| QED88053.1 | 3-oxoacyl-ACP\_reductase | BGC0001967 | NRP+Polyketide | 29.0 | 67.6 | 58.0 | 1.42e-09 |
| AAN65226.1 | b-ketotyrosyl-reductase | BGC0000832 | Saccharide:Hybrid/tailoring saccharide+Other:Aminocoumarin | 31.0 | 68.7 | 58.0 | 1.88e-09 |
| QQO98484.1 | FrzI | BGC0002146 | NRP | 28.0 | 57.7 | 57.0 | 2.54e-09 |
| AGJ76606.1 | SDR | BGC0000869 | Other | 25.0 | 66.2 | 57.0 | 2.72e-09 |
| CAG23976.1 | 3-oxoacyl-(acyl\_carrier\_protein)\_reductase | BGC0000176 | Polyketide | 30.0 | 68.3 | 57.0 | 3.08e-09 |
| AAN85505.1 | short-chain\_dehydrogenase/reductase | BGC0001101 | NRP+Polyketide:Modular type I polyketide+Polyketide:Trans-AT type I polyketide | 29.0 | 71.2 | 57.0 | 3.9e-09 |
| AAM33668.1 | putative\_3-oxoacyl-ACP\_reductase | BGC0000230 | Polyketide:Type II polyketide | 30.0 | 67.6 | 57.0 | 4.32e-09 |
| CAM34340.1 | putative\_3-oxoacyl-ACP\_reductase | BGC0000242 | Polyketide | 27.0 | 66.2 | 56.0 | 5.89e-09 |
| OWA25242.1 | ketoacyl\_reductase | BGC0001438 | Polyketide+Saccharide:Hybrid/tailoring saccharide | 27.0 | 67.6 | 56.0 | 6.37e-09 |
| QGY73451.1 | Itm19 | BGC0002451 | Polyketide | 29.0 | 59.8 | 55.0 | 1.41e-08 |
| ACN80658.1 | SioU | BGC0000611 | RiPP:Thiopeptide | 26.0 | 69.0 | 55.0 | 1.51e-08 |
| ACN52304.1 | TsrN | BGC0000614 | RiPP:Thiopeptide | 28.0 | 49.5 | 55.0 | 1.7e-08 |
| AMX23331.1 | putative\_acyl\_carrier\_protein | BGC0001500 | Polyketide | 31.0 | 65.8 | 55.0 | 1.89e-08 |
| QDJ74266.1 | 3-oxoacyl-ACP\_reductase | BGC0002109 | NRP | 28.0 | 67.6 | 55.0 | 1.94e-08 |
| BAJ52686.1 | putative\_3-oxoacyl-ACP\_reductase | BGC0000222 | Polyketide | 27.0 | 66.9 | 55.0 | 1.95e-08 |
| AAQ08926.1 | putative\_3-ketoacyl-ACP\_reductase | BGC0000224 | Polyketide:Type II polyketide | 28.0 | 56.9 | 55.0 | 1.95e-08 |
| ACO31265.1 | Ptm01 | BGC0001140 | Terpene | 27.0 | 70.1 | 55.0 | 2.03e-08 |
| CZT62787.1 | 3-oxoacyl-(Acyl-carrier-protein)\_reductase,\_involved\_in\_Hassallidin\_biosynthesis | BGC0001614 | NRP | 30.0 | 67.3 | 55.0 | 2.03e-08 |
| AIE54248.1 | PauY28 | BGC0001732 | Other | 30.0 | 41.6 | 55.0 | 2.04e-08 |
| CAP12610.1 | dehydrogenase | BGC0000219 | Polyketide:Type II polyketide+Saccharide:Hybrid/tailoring saccharide | 31.0 | 45.9 | 55.0 | 2.12e-08 |
| OBR09785.1 | Short\_chain\_dehydrogenase | BGC0002429 | Terpene+Polyketide | 28.0 | 59.1 | 55.0 | 2.23e-08 |
| ADD83002.1 | PtnO1 | BGC0001156 | Terpene | 27.0 | 70.1 | 54.0 | 2.74e-08 |
| CAE15172.1 |  | BGC0001736 | NRP | 27.0 | 67.6 | 54.0 | 2.74e-08 |
| QXJ26486.1 | mycofactocin-coupled\_SDR\_family\_oxidoreductase | BGC0002370 | NRP | 30.0 | 47.0 | 54.0 | 3.12e-08 |
| ABW96534.1 | putative\_dehydrogenase | BGC0000159 | Polyketide:Modular type I polyketide | 28.0 | 67.6 | 54.0 | 3.23e-08 |
| AGN71606.1 | ketoreductase\_ | BGC0000027 | Polyketide:Iterative type I polyketide | 25.0 | 61.9 | 54.0 | 3.62e-08 |
| AHX24706.1 | 3-oxoacyl-ACP\_reductase | BGC0000200 | Polyketide:Type II polyketide+Saccharide:Hybrid/tailoring saccharide | 27.0 | 61.2 | 54.0 | 3.64e-08 |
| ARS01461.1 | short-chain\_dehydrogenase | BGC0001702 | NRP+Polyketide | 29.0 | 64.1 | 54.0 | 5.57e-08 |
| CBZ42141.1 | Acyl-Co\_Adehydrogenase | BGC0001117 | NRP | 31.0 | 47.7 | 54.0 | 5.73e-08 |
| AEE65483.1 | short-chain\_dehydrogenase/reductase\_SDR | BGC0000223 | Polyketide:Type II polyketide | 27.0 | 74.4 | 53.0 | 6.1e-08 |
| ABW11827.1 | short-chain\_dehydrogenase/reductase\_SDR | BGC0001197 | Polyketide | 28.0 | 67.3 | 53.0 | 6.29e-08 |
| CDM31314.1 | Short-chain\_dehydrogenase/reductase\_SDR | BGC0000667 | Terpene | 29.0 | 73.7 | 54.0 | 7.4e-08 |
| AAG30255.1 | NADPH-dependent\_acetoacetyl\_CoA\_reductase | BGC0000867 | Other | 30.0 | 44.1 | 52.0 | 8.18e-08 |
| BAR73004.1 | putative\_short-chain\_dehydrogenase | BGC0001194 | Polyketide | 29.0 | 49.5 | 53.0 | 8.28e-08 |
| AIE54195.1 | Pau28 | BGC0001731 | Other | 30.0 | 41.6 | 53.0 | 9.13e-08 |
| MBE3200470.1 | SDR\_family\_oxidoreductase | BGC0002409 | NRP | 32.0 | 39.5 | 52.0 | 1.15e-07 |
| ABD65959.1 | acyl-CoA\_dehydrogenase/reductase | BGC0000341 | NRP | 25.0 | 72.2 | 52.0 | 1.38e-07 |
| QBG38776.1 | Atr15 | BGC0001975 | NRP | 28.0 | 66.9 | 52.0 | 1.58e-07 |
| ANY57964.1 | MtcE\_3-ketoacyl-ACP\_reductase | BGC0001369 | Polyketide | 28.0 | 63.0 | 52.0 | 2.17e-07 |
| AHZ20771.1 | 3-oxoacyl-acyl-carrier-protein\_reductase | BGC0000369 | NRP+Saccharide:Hybrid/tailoring saccharide | 27.0 | 80.4 | 52.0 | 2.22e-07 |
| ABX71123.1 | Lct40 | BGC0000238 | Polyketide | 32.0 | 67.6 | 51.0 | 2.95e-07 |
| BCB17007.1 | putative\_3-hydroxybutyrate\_dehydrogenase | BGC0002523 | NRP | 30.0 | 38.8 | 51.0 | 3.18e-07 |
| ADD83005.1 | PtnO7 | BGC0001156 | Terpene | 25.0 | 82.2 | 51.0 | 3.78e-07 |
| AHX24712.1 | 3-oxoacyl-ACP\_reductase | BGC0000200 | Polyketide:Type II polyketide+Saccharide:Hybrid/tailoring saccharide | 31.0 | 67.3 | 51.0 | 3.79e-07 |
| XP\_028481816.1 | short\_chain\_dehydrogenase/reductase | BGC0001866 | Polyketide | 36.0 | 30.6 | 51.0 | 5.15e-07 |
| QXL90830.1 | 3-oxoacyl-(acyl-carrier\_protein)\_reductase | BGC0002426 | NRP | 32.0 | 55.2 | 50.0 | 8.44e-07 |
| antaM | 3-oxoacyl-ACP\_reductase | BGC0001455 | NRP+Polyketide | 33.0 | 41.6 | 50.0 | 1.03e-06 |
| AGH68923.1 | oxidoreductase | BGC0001083 | Terpene+Polyketide:Type III polyketide | 29.0 | 40.6 | 49.0 | 1.32e-06 |
| AAP11948.1 | Short\_chain\_dehydrogenase | BGC0000612 | RiPP:Thiopeptide | 26.0 | 77.9 | 49.0 | 1.33e-06 |
| QKG86292.1 | enoyl\_reductase | BGC0002253 | Polyketide | 25.0 | 78.3 | 50.0 | 1.34e-06 |
| ACO31293.1 | PtmO7 | BGC0001140 | Terpene | 24.0 | 83.6 | 49.0 | 2.2e-06 |
| ADC45556.1 | 2-hydroxycyclohexanecarboxyl-CoA\_dehyrogenase | BGC0000093 | Polyketide | 27.0 | 71.9 | 49.0 | 2.91e-06 |
| ADG86325.1 | ketoreductase | BGC0000190 | Polyketide | 28.0 | 61.2 | 48.0 | 3.11e-06 |
| ADB23395.1 | dehydrogenase | BGC0001062 | Polyketide | 29.0 | 56.6 | 48.0 | 4.18e-06 |
| ESK96611.1 | carbonyl\_reductase | BGC0002212 | Polyketide | 32.0 | 54.1 | 47.0 | 6.82e-06 |
