# Supplementary material for: Uncovering the transcriptional landscape of Fomes fomentarius during fungal-based material production through gene co-expression network analysis
Source: Fungal Biol Biotechnol. 2025 Feb 13;12:1. doi: 10.1186/s40694-024-00192-3 (PMC11827164; doi:10.1186/s40694-024-00192-3)
Supplement: Supplementary file 1 — Supplementary Material 1 [file 40694_2024_192_MOESM1_ESM.zip › knownclusterblast/region1/jgi.p_Fomfom1_1371342_mibig_hits.html]

| MIBiG Protein | Description | MIBiG Cluster | MiBiG Product | % ID | % Coverage | BLAST Score | E-value |
| --- | --- | --- | --- | --- | --- | --- | --- |
| EHK18384.1 | hypothetical\_protein | BGC0002216 | Terpene | 38.0 | 104.7 | 316.0 | 7.34e-100 |
| ACA34720.1 | CtnD | BGC0000894 | Other | 35.0 | 104.2 | 308.0 | 1e-96 |
| ALI92648.1 | CitC\_oxidoreductase | BGC0001338 | Polyketide:Iterative type I polyketide | 35.0 | 104.2 | 308.0 | 1e-96 |
| BAQ25461.1 | putative\_dehydrogenase | BGC0001264 | Polyketide | 35.0 | 102.9 | 308.0 | 1.09e-96 |
| BBD84647.1 | putative\_GMC\_oxidoreductase | BGC0001775 | Terpene | 34.0 | 101.3 | 286.0 | 2.36e-88 |
| BAE62226.1 |  | BGC0002237 | Polyketide | 32.0 | 99.2 | 234.0 | 8.79e-70 |
| AIG62134.1 | patulin\_synthase | BGC0000120 | Polyketide:Iterative type I polyketide | 29.0 | 100.5 | 231.0 | 2.07e-67 |
| EIN09539.1 | pyranose\_dehydrogenase | BGC0002213 | Polyketide | 30.0 | 101.5 | 230.0 | 3e-67 |
| ctg1\_orf10 |  | BGC0000846 | Other | 30.0 | 97.8 | 232.0 | 3.63e-67 |
| CBF83141.1 | conserved\_hypothetical\_protein | BGC0001722 | Polyketide | 30.0 | 101.3 | 229.0 | 5.54e-67 |
| ACH72898.1 | AflK | BGC0000011 | Polyketide | 30.0 | 102.9 | 221.0 | 1.74e-63 |
| AAS90019.1 | VBS | BGC0000007 | Polyketide | 30.0 | 102.4 | 220.0 | 2.69e-63 |
| AAS90088.1 | VBS | BGC0000010 | Polyketide | 30.0 | 101.3 | 220.0 | 3.73e-63 |
| AAS90042.1 | VBS | BGC0000008 | Polyketide | 30.0 | 101.3 | 219.0 | 5.18e-63 |
| BAE71331.1 | versicolorin\_B\_synthase | BGC0000004 | Polyketide | 30.0 | 101.3 | 219.0 | 9.95e-63 |
| AAS90066.1 | VBS | BGC0000009 | Polyketide | 30.0 | 101.3 | 218.0 | 1.84e-62 |
| AAS90106.1 | VBS | BGC0000006 | Polyketide | 30.0 | 101.7 | 217.0 | 3.67e-62 |
| ATV82114.1 | GMC\_oxidoreductase/oxidase/dehydrogenase | BGC0001909 | Polyketide | 30.0 | 103.0 | 207.0 | 7.71e-59 |
| FAA01296.1 | glucose-methanol-choline\_family\_oxidoreductase\_PyvF | BGC0002210 | Polyketide+NRP | 28.0 | 100.7 | 206.0 | 4.96e-58 |
| KNA98285.1 | hypothetical\_protein | BGC0002670 | Other | 29.0 | 98.7 | 197.0 | 5.84e-55 |
| EAU32818.1 | predicted\_protein | BGC0000160 | Polyketide | 30.0 | 99.2 | 192.0 | 1.69e-53 |
| AEF33092.1 | choline\_dehydrogenase | BGC0001039 | NRP+Polyketide | 30.0 | 98.0 | 184.0 | 4.74e-51 |
| AVT42378.1 | glucose-methanol-choline\_oxidoreductase | BGC0001476 | Other:Nucleoside | 27.0 | 97.5 | 170.0 | 7.24e-46 |
| EEP98515.1 | Glucose-methanol-choline\_oxidoreductase | BGC0002091 | NRP | 26.0 | 100.3 | 155.0 | 1.58e-40 |
| KDM89831.1 | glucose-methanol-choline\_oxidoreductase | BGC0002412 | NRP | 28.0 | 102.2 | 155.0 | 1.88e-40 |
| KAF7526514.1 | hypothetical\_protein | BGC0002244 | Polyketide | 28.0 | 99.0 | 147.0 | 1.2e-37 |
| AJI44177.1 | glucose-methanol-choline\_oxidoreductase | BGC0001193 | NRP | 27.0 | 99.8 | 137.0 | 1.82e-34 |
| CAD62204.1 | Ata10\_protein | BGC0000873 | Other | 32.0 | 53.1 | 135.0 | 8.91e-34 |
| MCB8905710.1 | GMC\_family\_oxidoreductase\_N-terminal\_domain-containing\_protein | BGC0002340 | NRP+Other | 26.0 | 107.6 | 135.0 | 1.15e-33 |
| CBK62747.1 |  | BGC0001115 | NRP+Polyketide | 24.0 | 102.0 | 126.0 | 1.46e-30 |
| CAM56763.1 | hypothetical\_protein | BGC0000354 | NRP | 31.0 | 54.5 | 123.0 | 1.07e-29 |
| EIN09902.1 | FAD/NAD(P)-binding\_domain-containing\_protein | BGC0002220 | Terpene | 28.0 | 49.2 | 105.0 | 1.72e-24 |
