# Supplementary material for: Uncovering the transcriptional landscape of Fomes fomentarius during fungal-based material production through gene co-expression network analysis
Source: Fungal Biol Biotechnol. 2025 Feb 13;12:1. doi: 10.1186/s40694-024-00192-3 (PMC11827164; doi:10.1186/s40694-024-00192-3)
Supplement: Supplementary file 1 — Supplementary Material 1 [file 40694_2024_192_MOESM1_ESM.zip › knownclusterblast/region1/jgi.p_Fomfom1_1289136_mibig_hits.html]

| MIBiG Protein | Description | MIBiG Cluster | MiBiG Product | % ID | % Coverage | BLAST Score | E-value |
| --- | --- | --- | --- | --- | --- | --- | --- |
| ESK96610.1 | hypothetical\_protein | BGC0002212 | Polyketide | 25.0 | 97.0 | 282.0 | 3.18e-79 |
| EAU35432.1 | predicted\_protein | BGC0002734 | Polyketide | 26.0 | 97.7 | 268.0 | 2.69e-74 |
| KIA75587.1 | NRPS-like\_enzyme | BGC0002209 | Polyketide | 26.0 | 98.7 | 259.0 | 4.01e-71 |
| ASK38699.1 | putative\_nonribosomal\_peptide\_synthetase-like\_protein | BGC0001436 | Polyketide:Iterative type I polyketide | 27.0 | 94.1 | 256.0 | 1.71e-70 |
| KFA69336.1 | hypothetical\_protein | BGC0001626 | Polyketide | 29.0 | 74.2 | 256.0 | 1.71e-70 |
| BBF25314.1 | NRPS-like\_oxidoreductase | BGC0001923 | Terpene+Polyketide | 26.0 | 98.8 | 255.0 | 6.36e-70 |
| BAV19380.1 | NRPS-like\_enzyme | BGC0001390 | NRP+Polyketide | 27.0 | 82.7 | 244.0 | 1.89e-66 |
| EWG54274.1 | hypothetical\_protein | BGC0001190 | Polyketide | 26.0 | 92.1 | 229.0 | 1.26e-61 |
| CEF75881.1 |  | BGC0001600 | Polyketide | 28.0 | 84.8 | 227.0 | 1.16e-60 |
| AMJ52084.1 | lijE | BGC0002255 | Polyketide | 33.0 | 27.1 | 135.0 | 1.9e-31 |
| CAP95404.1 |  | BGC0001404 | Polyketide | 32.0 | 30.4 | 133.0 | 7.38e-31 |
| QCO93110.1 | polyketide\_synthase | BGC0001976 | Terpene | 29.0 | 38.9 | 132.0 | 9.7e-31 |
| AWM95789.1 | non-reduciing\_polyketide\_synthase\_methylorcinaldehyde\_synthase | BGC0001827 | Polyketide | 30.0 | 36.0 | 131.0 | 2.2e-30 |
| CCE67070.1 | polyketide\_synthase | BGC0001242 | Polyketide | 27.0 | 47.5 | 130.0 | 3.6e-30 |
| KFH44362.1 | Conidial\_yellow\_pigment\_biosynthesis\_polyketide\_synthase-like\_protein | BGC0002190 | Polyketide | 29.0 | 31.4 | 128.0 | 2.52e-29 |
| AGC45618.1 | non-ribosomal\_peptide\_synthetase | BGC0001394 | NRP+Polyketide | 29.0 | 23.6 | 82.0 | 2.93e-15 |
| AAX31557.1 | peptide\_synthetase\_1 | BGC0000336 | NRP | 25.0 | 31.0 | 51.0 | 8.36e-06 |
