# Supplementary material for: Uncovering the transcriptional landscape of Fomes fomentarius during fungal-based material production through gene co-expression network analysis
Source: Fungal Biol Biotechnol. 2025 Feb 13;12:1. doi: 10.1186/s40694-024-00192-3 (PMC11827164; doi:10.1186/s40694-024-00192-3)
Supplement: Supplementary file 1 — Supplementary Material 1 [file 40694_2024_192_MOESM1_ESM.zip › knownclusterblast/region1/jgi.p_Fomfom1_138762_mibig_hits.html]

| MIBiG Protein | Description | MIBiG Cluster | MiBiG Product | % ID | % Coverage | BLAST Score | E-value |
| --- | --- | --- | --- | --- | --- | --- | --- |
| ACR78134.1 | predicted\_Rab7-like\_GTPase | BGC0000312 | NRP | 40.0 | 41.6 | 119.0 | 2.41e-31 |
| XP\_011392744.1 | uncharacterized\_protein | BGC0001281 | Polyketide | 33.0 | 32.1 | 79.0 | 2.41e-15 |
