# Supplementary material for: Uncovering the transcriptional landscape of Fomes fomentarius during fungal-based material production through gene co-expression network analysis
Source: Fungal Biol Biotechnol. 2025 Feb 13;12:1. doi: 10.1186/s40694-024-00192-3 (PMC11827164; doi:10.1186/s40694-024-00192-3)
Supplement: Supplementary file 1 — Supplementary Material 1 [file 40694_2024_192_MOESM1_ESM.zip › knownclusterblast/region1/jgi.p_Fomfom1_1371970_mibig_hits.html]

| MIBiG Protein | Description | MIBiG Cluster | MiBiG Product | % ID | % Coverage | BLAST Score | E-value |
| --- | --- | --- | --- | --- | --- | --- | --- |
| XP\_007301853.1 | MFS\_general\_substrate\_transporter | BGC0001617 | Terpene | 24.0 | 76.8 | 134.0 | 5.78e-33 |
| ABB05093.1 | LipEx1 | BGC0001003 | NRP:Lipopeptide+Polyketide:Modular type I polyketide+Saccharide:Hybrid/tailoring saccharide | 30.0 | 79.0 | 130.0 | 2.32e-31 |
| ABC87516.1 | drug\_efflux\_transporter | BGC0001011 | NRP+Polyketide | 26.0 | 76.9 | 116.0 | 2.19e-27 |
| AQW35077.1 | MFS\_transporter | BGC0001675 | Polyketide | 25.0 | 71.7 | 115.0 | 4.27e-27 |
| ACN38360.1 | putative\_sisomicin\_exporter | BGC0000714 | Saccharide | 24.0 | 88.4 | 114.0 | 4.18e-26 |
| QXL90840.1 | MFS\_transporter | BGC0002426 | NRP | 27.0 | 82.5 | 112.0 | 5.47e-26 |
| QFS19051.1 | Alp1R\_family\_transporter | BGC0002506 | Polyketide | 27.0 | 62.1 | 108.0 | 9.6e-25 |
| AYV61423.1 | MFS\_transporter | BGC0001965 | Other | 27.0 | 86.7 | 104.0 | 2.58e-23 |
| ctg3\_1 |  | BGC0001853 | NRP+Polyketide:Modular type I polyketide | 25.0 | 77.6 | 103.0 | 3.81e-23 |
| AEW95643.1 | transmembrane\_efflux\_protein | BGC0002697 | NRP+Polyketide | 25.0 | 85.5 | 102.0 | 7.97e-23 |
| POM23771.1 | putative\_MFS-type\_transporter\_EfpA | BGC0002369 | Polyketide | 25.0 | 59.6 | 100.0 | 8.33e-22 |
| AAF81738.1 | putative\_efflux\_protein\_EncT | BGC0000220 | Polyketide:Type II polyketide | 30.0 | 42.1 | 97.0 | 7.78e-21 |
| ADC79648.1 | TamJ | BGC0001052 | NRP+Polyketide:Modular type I polyketide | 26.0 | 72.6 | 94.0 | 6.36e-20 |
| CBA11577.1 | putative\_transport\_integral\_membrain\_protein | BGC0001046 | NRP+Polyketide:Modular type I polyketide+Saccharide:Hybrid/tailoring saccharide | 31.0 | 45.5 | 94.0 | 6.9e-20 |
| QYA95649.1 | MFS\_transporter | BGC0002676 | NRP | 24.0 | 74.7 | 92.0 | 2.48e-19 |
| BAP16698.1 | transmembrane\_transporter | BGC0000376 | NRP | 24.0 | 75.9 | 91.0 | 4.46e-19 |
| AAP69589.1 | putative\_transmembrane\_efflux\_protein | BGC0000226 | Polyketide | 30.0 | 64.5 | 91.0 | 7.72e-19 |
| ARG41907.1 | IstD | BGC0001622 | Polyketide | 28.0 | 45.5 | 90.0 | 1.07e-18 |
| CAC36763.1 | methylenomycin\_A\_resistance\_protein,\_Mmr | BGC0000914 | Other | 25.0 | 75.9 | 90.0 | 1.32e-18 |
| ATY46599.1 | drug\_resistance\_transporter | BGC0001666 | Polyketide | 28.0 | 40.4 | 87.0 | 1.39e-17 |
| OWA01612.1 | hypothetical\_protein | BGC0001439 | Polyketide+Saccharide:Hybrid/tailoring saccharide | 24.0 | 63.6 | 87.0 | 1.76e-17 |
| ctg1\_orf530 |  | BGC0001199 | Polyketide | 28.0 | 42.1 | 85.0 | 4.49e-17 |
| CAH60162.2 | putative\_transmembrane\_efflux\_protein | BGC0000700 | Saccharide | 29.0 | 43.4 | 82.0 | 4.1e-16 |
| QKW60385.1 | MFS\_transporter | BGC0002288 | NRP | 27.0 | 43.9 | 78.0 | 9.41e-15 |
| ARE67836.1 | AbsD | BGC0001492 | Polyketide | 29.0 | 38.2 | 76.0 | 3.71e-14 |
| OKI59854.1 | arabinose\_transporter\_permease | BGC0002477 | Polyketide | 26.0 | 42.8 | 74.0 | 8.65e-14 |
| AFU65888.1 | DacR2 | BGC0000216 | Polyketide | 25.0 | 54.4 | 74.0 | 1.32e-13 |
| ADE34501.1 | ssfR | BGC0000269 | Polyketide:Type II polyketide+Saccharide:Hybrid/tailoring saccharide | 25.0 | 40.7 | 74.0 | 1.38e-13 |
| ACZ57546.1 | predicted\_MFS\_transporter | BGC0000046 | Polyketide:Iterative type I polyketide | 25.0 | 58.9 | 65.0 | 1.53e-10 |
| PPQ57485.1 | MFS\_transporter | BGC0002016 | Polyketide | 24.0 | 46.0 | 62.0 | 7.21e-10 |
