# Supplementary material for: Uncovering the transcriptional landscape of Fomes fomentarius during fungal-based material production through gene co-expression network analysis
Source: Fungal Biol Biotechnol. 2025 Feb 13;12:1. doi: 10.1186/s40694-024-00192-3 (PMC11827164; doi:10.1186/s40694-024-00192-3)
Supplement: Supplementary file 1 — Supplementary Material 1 [file 40694_2024_192_MOESM1_ESM.zip › knownclusterblast/region1/jgi.p_Fomfom1_1319276_mibig_hits.html]

| MIBiG Protein | Description | MIBiG Cluster | MiBiG Product | % ID | % Coverage | BLAST Score | E-value |
| --- | --- | --- | --- | --- | --- | --- | --- |
| AEO57486.1 | hypothetical\_protein | BGC0001449 | NRP+Alkaloid+Polyketide:Iterative type I polyketide | 36.0 | 94.0 | 233.0 | 1.57e-72 |
| CAE02620.1 | Yx01\_protein | BGC0000433 | NRP:Lipopeptide | 26.0 | 99.1 | 121.0 | 2.51e-30 |
| QWT72263.1 | Zn-dependent\_alcohol\_dehydrogenase | BGC0002430 | NRP+Saccharide | 28.0 | 97.4 | 101.0 | 4.53e-23 |
| BBA21077.1 | putative\_inosine-5'-monophosphate\_dehydrogenase | BGC0001740 | NRP+Polyketide | 38.0 | 42.8 | 98.0 | 2.96e-22 |
| QCF28943.1 | aldehyde\_dehydrogenase | BGC0002308 | Alkaloid+Polyketide | 31.0 | 50.8 | 87.0 | 3.72e-18 |
| AGI87371.1 | Zinc-binding\_dehydrogenase | BGC0002358 | Polyketide | 36.0 | 28.0 | 78.0 | 2.11e-15 |
