# Supplementary material for: Uncovering the transcriptional landscape of Fomes fomentarius during fungal-based material production through gene co-expression network analysis
Source: Fungal Biol Biotechnol. 2025 Feb 13;12:1. doi: 10.1186/s40694-024-00192-3 (PMC11827164; doi:10.1186/s40694-024-00192-3)
Supplement: Supplementary file 1 — Supplementary Material 1 [file 40694_2024_192_MOESM1_ESM.zip › knownclusterblast/region1/jgi.p_Fomfom1_1319667_mibig_hits.html]

| MIBiG Protein | Description | MIBiG Cluster | MiBiG Product | % ID | % Coverage | BLAST Score | E-value |
| --- | --- | --- | --- | --- | --- | --- | --- |
| ESK96610.1 | hypothetical\_protein | BGC0002212 | Polyketide | 28.0 | 100.9 | 349.0 | 7.79e-103 |
| ASK38699.1 | putative\_nonribosomal\_peptide\_synthetase-like\_protein | BGC0001436 | Polyketide:Iterative type I polyketide | 31.0 | 81.8 | 315.0 | 1.18e-90 |
| KFA69336.1 | hypothetical\_protein | BGC0001626 | Polyketide | 27.0 | 90.1 | 282.0 | 4.19e-79 |
| EAU35432.1 | predicted\_protein | BGC0002734 | Polyketide | 26.0 | 102.6 | 280.0 | 1.76e-78 |
| BAV19380.1 | NRPS-like\_enzyme | BGC0001390 | NRP+Polyketide | 25.0 | 99.5 | 269.0 | 7.59e-75 |
| EWG54274.1 | hypothetical\_protein | BGC0001190 | Polyketide | 26.0 | 92.7 | 268.0 | 1.4e-74 |
| AWM95789.1 | non-reduciing\_polyketide\_synthase\_methylorcinaldehyde\_synthase | BGC0001827 | Polyketide | 30.0 | 36.6 | 141.0 | 1.9e-33 |
| CAP95404.1 |  | BGC0001404 | Polyketide | 27.0 | 41.3 | 132.0 | 9.8e-31 |
| AUW31047.1 | PKS-like\_protein | BGC0002483 | Polyketide | 27.0 | 31.6 | 115.0 | 1.19e-27 |
| ATY72525.1 | non-ribosomal\_peptide\_synthetase | BGC0001574 | NRP | 25.0 | 57.2 | 117.0 | 5.3e-26 |
| AEA29644.1 | putative\_nonribosomal\_peptide\_synthetase\_and\_kinurenine\_monooxygenase | BGC0000409 | NRP | 26.0 | 58.5 | 115.0 | 2.36e-25 |
| AGO86662.1 | equisetin\_synthetase | BGC0001255 | NRP+Polyketide | 28.0 | 22.1 | 59.0 | 4.93e-08 |
| XP\_001220460.1 | uncharacterized\_protein | BGC0001182 | NRP+Polyketide:Iterative type I polyketide | 24.0 | 21.8 | 58.0 | 6.52e-08 |
| QHD43130.1 | NRPS/PKS\_hybrid\_protein | BGC0002546 | NRP+Polyketide | 22.0 | 36.3 | 56.0 | 3.26e-07 |
| ATY37592.1 | BogE | BGC0001532 | NRP | 22.0 | 27.9 | 54.0 | 1.21e-06 |
