# Supplementary material for: Uncovering the transcriptional landscape of Fomes fomentarius during fungal-based material production through gene co-expression network analysis
Source: Fungal Biol Biotechnol. 2025 Feb 13;12:1. doi: 10.1186/s40694-024-00192-3 (PMC11827164; doi:10.1186/s40694-024-00192-3)
Supplement: Supplementary file 1 — Supplementary Material 1 [file 40694_2024_192_MOESM1_ESM.zip › knownclusterblast/region1/jgi.p_Fomfom1_1360149_mibig_hits.html]

| MIBiG Protein | Description | MIBiG Cluster | MiBiG Product | % ID | % Coverage | BLAST Score | E-value |
| --- | --- | --- | --- | --- | --- | --- | --- |
| XP\_007301850.1 | cytochrome\_P450 | BGC0001617 | Terpene | 55.0 | 93.5 | 577.0 | 1.27e-203 |
| QJQ03972.1 | CYP-Arm3 | BGC0002445 | Terpene | 46.0 | 90.7 | 482.0 | 3.27e-166 |
| XP\_007301602.1 | cytochrome\_P450 | BGC0001617 | Terpene | 46.0 | 90.3 | 456.0 | 5.85e-156 |
| KAA1470686.1 | cytochrome\_P450 | BGC0002218 | Terpene | 44.0 | 90.9 | 447.0 | 1.4e-152 |
| XP\_007301852.1 | cytochrome\_P450 | BGC0001617 | Terpene | 45.0 | 90.3 | 442.0 | 1.02e-150 |
| QJQ03971.1 | CYP-Arm2 | BGC0002445 | Terpene | 43.0 | 94.0 | 429.0 | 1.7e-145 |
| XP\_007301851.1 | cytochrome\_P450 | BGC0001617 | Terpene | 41.0 | 88.6 | 375.0 | 2.95e-124 |
| EHA22193.1 | hypothetical\_protein | BGC0000170 | Polyketide | 34.0 | 94.0 | 308.0 | 2e-98 |
| EYE95339.1 | cytochrome\_P450 | BGC0002234 | Polyketide | 33.0 | 96.8 | 299.0 | 1.06e-94 |
| KJA16708.1 | hypothetical\_protein | BGC0002246 | Terpene | 34.0 | 91.0 | 298.0 | 1.78e-94 |
| QBC75451.1 | MacC | BGC0002615 | Terpene | 32.0 | 95.7 | 297.0 | 5.55e-94 |
| QJQ03974.1 | CYP-Arm4 | BGC0002445 | Terpene | 32.0 | 96.1 | 281.0 | 2.25e-88 |
| DAB41655.1 | cytochrome\_P450\_monooxygenase | BGC0001585 | Alkaloid | 33.0 | 93.1 | 279.0 | 5.57e-87 |
| BCI98769.1 | putative\_cytochrome\_P450 | BGC0002181 | Terpene | 35.0 | 91.4 | 278.0 | 7.04e-87 |
| QGW49097.1 | putative\_cytochrome\_P450 | BGC0002731 | Polyketide | 34.0 | 95.0 | 278.0 | 1.07e-86 |
| OJJ99915.1 | hypothetical\_protein | BGC0002225 | Terpene | 36.0 | 94.0 | 275.0 | 8.43e-86 |
| AQZ42158.1 | putative\_cytochrome\_P450 | BGC0001820 | NRP | 33.0 | 91.2 | 275.0 | 2.6e-85 |
| EHK18434.1 | hypothetical\_protein | BGC0002233 | Polyketide | 33.0 | 91.6 | 275.0 | 3.11e-85 |
| CEN60544.1 | hypothetical\_protein | BGC0002266 | Terpene+Polyketide | 35.0 | 93.1 | 270.0 | 9.11e-84 |
| FAC38\_04 |  | BGC0002198 | NRP | 34.0 | 95.0 | 270.0 | 1.09e-83 |
| PPQ83215.1 | Dimethyltryptamine\_4-hydroxylase\_(PsiH) | BGC0002207 | Other | 35.0 | 85.1 | 265.0 | 3.16e-82 |
| KIJ60843.1 | hypothetical\_protein | BGC0002214 | Polyketide | 33.0 | 87.1 | 261.0 | 2.92e-81 |
| KIJ60846.1 | hypothetical\_protein | BGC0002214 | Polyketide | 33.0 | 93.8 | 259.0 | 9.63e-80 |
| OQD69071.1 | hypothetical\_protein | BGC0002745 | Polyketide | 31.0 | 93.7 | 259.0 | 1.72e-79 |
| QJQ03970.1 | CYP-Arm1 | BGC0002445 | Terpene | 32.0 | 95.1 | 258.0 | 3.39e-79 |
| KIJ60837.1 | hypothetical\_protein | BGC0002214 | Polyketide | 34.0 | 84.3 | 257.0 | 6.64e-79 |
| EIN09540.1 | cytochrome\_P450 | BGC0002213 | Polyketide | 33.0 | 96.1 | 256.0 | 1.31e-78 |
| PPQ83216.1 | Dimethyrltryptamine\_4-hydroxylase\_(PsiH) | BGC0002207 | Other | 34.0 | 85.8 | 253.0 | 9.03e-78 |
| AIG62144.1 | M-cresol\_hydroxylase | BGC0000120 | Polyketide:Iterative type I polyketide | 30.0 | 91.2 | 253.0 | 2.72e-77 |
| BBB04330.1 | cytochrome\_P450 | BGC0001717 | NRP | 32.0 | 97.4 | 246.0 | 9.53e-75 |
| AIG62145.1 | M-hydroxybenzyl\_alcohol\_hydroxylase | BGC0000120 | Polyketide:Iterative type I polyketide | 29.0 | 94.6 | 245.0 | 2.43e-74 |
| OJJ97582.1 | hypothetical\_protein | BGC0002229 | Polyketide | 30.0 | 92.5 | 241.0 | 1.11e-72 |
| QQO98481.1 | FrzL | BGC0002146 | NRP | 32.0 | 91.6 | 240.0 | 1.94e-72 |
| EIM84826.1 | cytochrome\_P450 | BGC0002219 | Terpene | 35.0 | 89.7 | 238.0 | 1.57e-71 |
| KIJ60841.1 | hypothetical\_protein | BGC0002214 | Polyketide | 32.0 | 84.1 | 234.0 | 1.86e-70 |
| BAH23999.1 | cytochrome\_P450 | BGC0000356 | NRP+Alkaloid | 31.0 | 95.0 | 224.0 | 3.53e-66 |
| BAE60008.1 |  | BGC0001518 | Terpene | 29.0 | 98.3 | 220.0 | 8.9e-65 |
| BAE71330.1 | oxidoreductase\_A;oxidoreductase/cytochrome\_P450\_monooxygenase | BGC0000004 | Polyketide | 29.0 | 98.1 | 214.0 | 1.53e-62 |
| AAS90081.1 | OrdA | BGC0000010 | Polyketide | 29.0 | 98.1 | 214.0 | 1.53e-62 |
| AAS90013.1 | OrdA | BGC0000007 | Polyketide | 29.0 | 98.5 | 214.0 | 2.14e-62 |
| AAS90035.1 | OrdA | BGC0000008 | Polyketide | 29.0 | 98.1 | 212.0 | 8.07e-62 |
| AAS90105.1 | OrdA | BGC0000006 | Polyketide | 29.0 | 98.1 | 212.0 | 1.12e-61 |
| AAS90061.1 | OrdA | BGC0000009 | Polyketide | 30.0 | 91.0 | 209.0 | 1.6e-60 |
| BCI98774.1 | putative\_cytochrome\_P450 | BGC0002181 | Terpene | 27.0 | 91.4 | 203.0 | 1.54e-58 |
| EAL85111.2 | cytochrome\_P450\_oxidoreductase | BGC0001037 | NRP+Polyketide:Iterative type I polyketide | 29.0 | 93.5 | 199.0 | 7.02e-57 |
| QJQ82463.1 | BisI | BGC0002290 | Other | 28.0 | 92.9 | 181.0 | 1.82e-50 |
| QMS79071.1 | fumitremorgin\_C\_synthase | BGC0002198 | NRP | 28.0 | 93.1 | 177.0 | 1.09e-48 |
| NP\_001130688.1 | uncharacterized\_protein\_LOC100191791 | BGC0002391 | Terpene | 26.0 | 90.5 | 154.0 | 1.91e-40 |
| BAF09102.1 |  | BGC0000672 | Terpene | 28.0 | 97.8 | 151.0 | 1.31e-39 |
| AGK82817.1 | cytochrome\_P450-1 | BGC0001324 | Terpene | 26.0 | 93.5 | 148.0 | 1.52e-38 |
| ALS30800.1 | putative\_cytochrome\_P450\_monooxygenase | BGC0001286 | Other | 25.0 | 92.9 | 148.0 | 2.94e-38 |
| KGN46390.1 | hypothetical\_protein | BGC0001315 | Terpene | 24.0 | 90.1 | 147.0 | 3.58e-38 |
| AGK82815.1 | cytochrome\_P450-2 | BGC0001324 | Terpene | 25.0 | 89.4 | 146.0 | 6.62e-38 |
| KGN46388.1 | hypothetical\_protein | BGC0001315 | Terpene | 24.0 | 93.3 | 146.0 | 6.8e-38 |
| AGK82807.1 | cytochrome\_P450-1 | BGC0001322 | Terpene | 25.0 | 89.6 | 145.0 | 2.32e-37 |
| AGK82824.1 | cytochrome\_P450-2 | BGC0001323 | Terpene | 25.0 | 89.2 | 144.0 | 4.34e-37 |
| AGK82831.1 | cytochrome\_P450-2 | BGC0001321 | Terpene | 25.0 | 88.2 | 144.0 | 4.52e-37 |
| AFW60201.1 | benzoxazinone\_synthesis4 | BGC0000810 | Alkaloid | 25.0 | 87.5 | 143.0 | 1.1e-36 |
| BAF09101.1 |  | BGC0000672 | Terpene | 25.0 | 89.9 | 140.0 | 5.76e-36 |
| EEF48745.1 | cytochrome\_P450,\_putative | BGC0002393 | Terpene | 26.0 | 88.2 | 138.0 | 6.34e-35 |
| pseudo106205\_112773 |  | BGC0001322 | Terpene | 25.0 | 88.2 | 137.0 | 8.73e-35 |
| AFW60200.1 | benzoxazinone\_synthesis3 | BGC0000810 | Alkaloid | 26.0 | 86.0 | 138.0 | 8.91e-35 |
| NP\_001142188.1 | putative\_cytochrome\_P450\_superfamily\_protein | BGC0002390 | Terpene | 24.0 | 94.0 | 135.0 | 7.77e-34 |
| XP\_020393230.1 | dolabradiene\_monooxygenase | BGC0002390 | Terpene | 25.0 | 91.8 | 134.0 | 1.39e-33 |
| XP\_008665446.1 | cytochrome\_P450\_81Q32 | BGC0002391 | Terpene | 26.0 | 88.6 | 132.0 | 5.42e-33 |
| chr3.CM0292.110.r2.m |  | BGC0001317 | Terpene | 27.0 | 84.7 | 132.0 | 5.84e-33 |
| NP\_001141366.1 | zealexin\_A1\_synthase | BGC0002390 | Terpene | 24.0 | 92.5 | 132.0 | 6.4e-33 |
| EEF48736.1 | cytochrome\_P450,\_putative | BGC0002393 | Terpene | 24.0 | 86.9 | 129.0 | 9.65e-32 |
| BAT00637.1 |  | BGC0002392 | Terpene | 24.0 | 91.8 | 128.0 | 2.13e-31 |
| XP\_044984106.1 | indole-2-monooxygenase-like | BGC0002721 | Saccharide | 24.0 | 88.1 | 125.0 | 1.23e-30 |
| AQU14205.1 | cytochrome\_p450 | BGC0001490 | Other:PBDE | 23.0 | 95.1 | 125.0 | 1.35e-30 |
| EEF48750.1 | (S)-N-methylcoclaurine\_3'-hydroxylase\_isozyme,\_putative | BGC0002393 | Terpene | 25.0 | 87.7 | 124.0 | 3.54e-30 |
| XP\_044963914.1 | cytochrome\_P450\_99A2-like | BGC0002395 | Terpene | 24.0 | 91.4 | 123.0 | 6.88e-30 |
| AAD34565.1 | cytochrome\_P450\_monooxygenase | BGC0000088 | Polyketide | 24.0 | 96.6 | 120.0 | 8.69e-29 |
| BAF14086.1 |  | BGC0000671 | Terpene | 25.0 | 88.1 | 119.0 | 1.4e-28 |
| AFW60212.1 | benzoxazinone\_synthesis2 | BGC0000810 | Alkaloid | 25.0 | 86.0 | 120.0 | 1.43e-28 |
| XP\_044963915.1 | cytochrome\_P450\_99A2-like | BGC0002395 | Terpene | 23.0 | 85.3 | 119.0 | 1.86e-28 |
| EER93095.1 | hypothetical\_protein | BGC0000798 | Saccharide | 24.0 | 83.4 | 119.0 | 3.93e-28 |
| EER93097.1 | hypothetical\_protein | BGC0000798 | Saccharide | 22.0 | 88.6 | 118.0 | 5.23e-28 |
| XP\_037497843.1 | premnaspirodiene\_oxygenase | BGC0002724 | Terpene | 24.0 | 87.5 | 117.0 | 1e-27 |
| XP\_044969119.1 | cytochrome\_P450\_99A2-like | BGC0002395 | Terpene | 24.0 | 85.8 | 116.0 | 1.54e-27 |
| BAF14091.1 |  | BGC0000671 | Terpene | 22.0 | 89.2 | 115.0 | 5.3e-27 |
| NP\_199610.1 | cytochrome\_P450,\_family\_705,\_subfamily\_A,\_polypeptide\_5 | BGC0000670 | Terpene | 22.0 | 90.7 | 115.0 | 5.46e-27 |
| XP\_044969120.1 | cytochrome\_P450\_99A2-like | BGC0002395 | Terpene | 23.0 | 85.8 | 114.0 | 1.24e-26 |
| XP\_044969126.1 | cytochrome\_P450\_99A2-like | BGC0002395 | Terpene | 22.0 | 91.4 | 110.0 | 1.31e-25 |
| chr3.CM0241.700.r2.m |  | BGC0001316 | Other | 23.0 | 91.0 | 110.0 | 2.22e-25 |
| XP\_044969124.1 | cytochrome\_P450\_99A2-like | BGC0002395 | Terpene | 22.0 | 91.4 | 110.0 | 2.37e-25 |
| XP\_044963918.1 | cytochrome\_P450\_99A2-like | BGC0002395 | Terpene | 22.0 | 91.4 | 110.0 | 2.37e-25 |
| NP\_199072.1 | cytochrome\_P450,\_family\_705,\_subfamily\_A,\_polypeptide\_12 | BGC0000669 | Terpene | 23.0 | 83.4 | 108.0 | 5.71e-25 |
| NP\_193268.3 | cytochrome\_P450,\_family\_705,\_subfamily\_A,\_polypeptide\_1 | BGC0001313 | Terpene | 23.0 | 85.6 | 107.0 | 2.72e-24 |
| NP\_193273.1 | cytochrome\_P450,\_family\_705,\_subfamily\_A,\_polypeptide\_4 | BGC0001313 | Terpene | 22.0 | 85.3 | 107.0 | 2.79e-24 |
| CYP82X2 |  | BGC0001325 | Alkaloid | 23.0 | 96.3 | 106.0 | 6.09e-24 |
| KGN46389.1 | hypothetical\_protein | BGC0001315 | Terpene | 23.0 | 93.7 | 105.0 | 6.83e-24 |
| XP\_044984006.1 | 4-hydroxyphenylacetaldehyde\_oxime\_monooxygenase-like | BGC0002721 | Saccharide | 23.0 | 87.1 | 105.0 | 9.45e-24 |
| XP\_044984175.1 | tyrosine\_N-monooxygenase-like | BGC0002721 | Saccharide | 21.0 | 92.0 | 105.0 | 1.23e-23 |
| chr3.CM0241.310.r2.m |  | BGC0001316 | Other | 23.0 | 91.0 | 103.0 | 3.18e-23 |
| NP\_193270.1 | cytochrome\_P450,\_family\_705,\_subfamily\_A,\_polypeptide\_2 | BGC0001313 | Terpene | 22.0 | 85.3 | 100.0 | 2.83e-22 |
| XP\_044963916.1 | cytochrome\_P450\_89A2-like | BGC0002395 | Terpene | 22.0 | 93.3 | 96.0 | 1.64e-20 |
| XP\_044969122.1 | cytochrome\_P450\_89A2-like | BGC0002395 | Terpene | 22.0 | 93.3 | 96.0 | 1.64e-20 |
| XP\_044969123.1 | cytochrome\_P450\_89A2-like | BGC0002395 | Terpene | 22.0 | 93.3 | 96.0 | 1.64e-20 |
| XP\_037497855.1 | LOW\_QUALITY\_PROTEIN:\_premnaspirodiene\_oxygenase-like | BGC0002724 | Terpene | 22.0 | 94.6 | 93.0 | 9.95e-20 |
| AAO27756.1 | monooxygenase | BGC0001278 | Terpene | 23.0 | 86.8 | 92.0 | 1.4e-19 |
| NP\_193271.5 | cytochrome\_P450,\_family\_705,\_subfamily\_A,\_polypeptide\_3 | BGC0001313 | Terpene | 21.0 | 85.4 | 90.0 | 9.66e-19 |
| Manes.12G133500 |  | BGC0001318 | Other | 29.0 | 31.7 | 89.0 | 2.38e-18 |
| NP\_001329686.1 | cytochrome\_P450,\_family\_705,\_subfamily\_A,\_polypeptide\_1 | BGC0001313 | Terpene | 32.0 | 33.8 | 89.0 | 2.57e-18 |
| TXD00003.1 | cytochrome\_P450 | BGC0001877 | Polyketide | 25.0 | 81.9 | 86.0 | 1.73e-17 |
| AFW60213.1 | benzoxazinone\_synthesis2 | BGC0000810 | Alkaloid | 26.0 | 67.2 | 85.0 | 1.98e-17 |
| CYP719A21 |  | BGC0001325 | Alkaloid | 23.0 | 98.3 | 85.0 | 3.39e-17 |
| ADO85579.1 | PntI | BGC0000653 | Terpene | 24.0 | 84.1 | 84.0 | 5.28e-17 |
| AAO34681.1 | monooxygenase | BGC0001277 | Terpene | 23.0 | 86.9 | 84.0 | 5.83e-17 |
| EIN09535.1 | cytochrome\_P450 | BGC0002213 | Polyketide | 31.0 | 35.4 | 84.0 | 1.29e-16 |
| BAC70710.1 | pentalenene\_C13\_hydroxylase;\_cytochrome\_P450 | BGC0000678 | Terpene | 22.0 | 81.2 | 82.0 | 2.84e-16 |
| QLH55579.1 | cytochrome\_P450\_family\_protein | BGC0002043 | RiPP | 24.0 | 85.1 | 82.0 | 3.8e-16 |
| BAV69303.1 | PrhB | BGC0001729 | Polyketide+Terpene | 29.0 | 37.7 | 81.0 | 5.93e-16 |
| BBA21073.1 | putative\_non-ribosomal\_peptide\_synthetase | BGC0001740 | NRP+Polyketide | 23.0 | 90.1 | 82.0 | 6.45e-16 |
| ATZ56106.1 | Bcbot1 | BGC0000631 | Terpene | 28.0 | 35.8 | 79.0 | 4.62e-15 |
| EMD93703.1 | hypothetical\_protein | BGC0002245 | Terpene | 21.0 | 90.1 | 78.0 | 6.06e-15 |
| AEW95633.1 | cytochrome\_P450\_protein | BGC0002697 | NRP+Polyketide | 24.0 | 88.2 | 76.0 | 3.76e-14 |
| KJA16715.1 | hypothetical\_protein | BGC0002246 | Terpene | 24.0 | 90.9 | 75.0 | 6.37e-14 |
| BAD29972.1 | P450\_monooxygenase\_1 | BGC0000676 | Terpene | 27.0 | 42.4 | 69.0 | 3.8e-12 |
| KJA16707.1 | hypothetical\_protein | BGC0002246 | Terpene | 27.0 | 45.3 | 69.0 | 4.27e-12 |
| BAJ52677.1 | putative\_cytochrome\_P450 | BGC0000222 | Polyketide | 29.0 | 40.7 | 66.0 | 3.76e-11 |
| QVK45106.1 | cytochrome\_P450 | BGC0002438 | Alkaloid | 28.0 | 29.5 | 58.0 | 1.34e-08 |
| CEO59279.1 | Putative\_Benzoate\_4-monooxygenase\_cytochrome\_P450 | BGC0002278 | Alkaloid+NRP | 29.0 | 32.1 | 58.0 | 1.82e-08 |
| ARE67844.1 | AbsV | BGC0001492 | Polyketide | 30.0 | 28.9 | 52.0 | 9.62e-07 |
| KZL86693.1 | cytochrome\_p450\_family\_protein | BGC0002228 | NRP | 28.0 | 32.1 | 52.0 | 1.53e-06 |
| ABF86324.1 | cytochrome\_P450 | BGC0001025 | NRP+Polyketide:Trans-AT type I polyketide | 28.0 | 29.9 | 51.0 | 1.97e-06 |
| AET79191.1 | elymoclavine\_monooxygenase | BGC0001241 | Terpene | 26.0 | 34.3 | 51.0 | 2.67e-06 |
| CCE30234.1 | related\_to\_trichodiene\_oxygenase\_cytochrome\_P450 | BGC0002232 | Alkaloid | 26.0 | 34.3 | 51.0 | 2.67e-06 |
| BAQ25476.1 | cytochrome\_P450 | BGC0001288 | Polyketide | 29.0 | 30.4 | 50.0 | 4e-06 |
| g115.t1 |  | BGC0001997 | Terpene | 27.0 | 20.1 | 47.0 | 4.2e-06 |
| BBA66514.1 | cytochrome\_P450 | BGC0001495 | Polyketide | 26.0 | 38.1 | 49.0 | 8.99e-06 |
