# Supplementary material for: Uncovering the transcriptional landscape of Fomes fomentarius during fungal-based material production through gene co-expression network analysis
Source: Fungal Biol Biotechnol. 2025 Feb 13;12:1. doi: 10.1186/s40694-024-00192-3 (PMC11827164; doi:10.1186/s40694-024-00192-3)
Supplement: Supplementary file 1 — Supplementary Material 1 [file 40694_2024_192_MOESM1_ESM.zip › knownclusterblast/region1/jgi.p_Fomfom1_1210368_mibig_hits.html]

| MIBiG Protein | Description | MIBiG Cluster | MiBiG Product | % ID | % Coverage | BLAST Score | E-value |
| --- | --- | --- | --- | --- | --- | --- | --- |
| THU83563.1 | arylalcohol\_dehydrogenase | BGC0002746 | RiPP | 51.0 | 97.4 | 373.0 | 1.17e-127 |
| AAS90040.1 | NorA | BGC0000008 | Polyketide | 50.0 | 91.6 | 330.0 | 6.24e-111 |
| AAS90007.1 | NorA | BGC0000007 | Polyketide | 49.0 | 91.1 | 328.0 | 3.55e-110 |
| AAS90094.1 | NorA | BGC0000006 | Polyketide | 50.0 | 91.6 | 327.0 | 1.01e-109 |
| AAS90055.1 | NorA | BGC0000009 | Polyketide | 49.0 | 96.6 | 327.0 | 1.01e-109 |
| AAS90074.1 | NorA | BGC0000010 | Polyketide | 50.0 | 91.6 | 327.0 | 1.01e-109 |
| ACI31321.1 | AflE | BGC0000011 | Polyketide | 51.0 | 90.1 | 319.0 | 1.02e-106 |
| AAC49206.1 | putative\_dehydrogenase | BGC0000152 | Polyketide | 50.0 | 90.6 | 319.0 | 1.44e-106 |
| AAS90044.1 | NorB | BGC0000009 | Polyketide | 47.0 | 88.7 | 295.0 | 3.86e-97 |
| AAS89996.1 | NorB | BGC0000007 | Polyketide | 47.0 | 89.0 | 292.0 | 4.37e-96 |
| BAE71322.1 | reductase/dehydrogenase | BGC0000004 | Polyketide | 50.0 | 81.9 | 289.0 | 5.9e-96 |
| KAF7526526.1 | hypothetical\_protein | BGC0002244 | Polyketide | 42.0 | 93.2 | 271.0 | 7.77e-88 |
| UPA71923.1 | aldo-keto\_reductase\_IolS | BGC0002636 | Polyketide | 34.0 | 89.3 | 172.0 | 3.15e-50 |
| ACI88870.1 | alnumycin\_B\_aldoreductase | BGC0000195 | Polyketide:Type II polyketide | 36.0 | 87.4 | 169.0 | 7.09e-49 |
| OWA25480.1 | aldo/keto\_reductase | BGC0001438 | Polyketide+Saccharide:Hybrid/tailoring saccharide | 36.0 | 87.4 | 167.0 | 3.39e-48 |
| AAZ23049.1 | possible\_oxidoreductase | BGC0000291 | NRP | 31.0 | 91.6 | 151.0 | 3.47e-42 |
| QLJ99351.1 | aldo/keto\_reductase | BGC0002088 | Polyketide+Saccharide:Oligosaccharide | 32.0 | 85.3 | 131.0 | 1.53e-34 |
| UHH90016.1 | VicE | BGC0002634 | Polyketide+NRP+Other | 29.0 | 86.9 | 127.0 | 1.76e-33 |
| QLH55584.1 | oxidoreductase\_aldo/keto\_reductase\_family | BGC0002043 | RiPP | 31.0 | 88.7 | 125.0 | 8.23e-33 |
| ctg1\_orf20 |  | BGC0000053 | Polyketide | 28.0 | 85.3 | 124.0 | 2.43e-32 |
| AUA09454.1 | L-glyceraldehyde\_3-phosphate\_reductase | BGC0002291 | Polyketide | 28.0 | 85.3 | 124.0 | 3.37e-32 |
| BAD08364.1 | dTDP-4-keto-6-deoxyhexose\_2,3-reductase | BGC0000167 | Polyketide | 28.0 | 86.9 | 123.0 | 9.01e-32 |
| ABB05109.1 | LipDig3 | BGC0001003 | NRP:Lipopeptide+Polyketide:Modular type I polyketide+Saccharide:Hybrid/tailoring saccharide | 29.0 | 84.6 | 118.0 | 6.12e-30 |
| WP\_004562643.1 | aldo/keto\_reductase | BGC0002009 | Polyketide | 29.0 | 88.7 | 117.0 | 1.86e-29 |
| BBE36476.1 | dTDP-2,3-ketoreductase | BGC0001922 | Polyketide | 28.0 | 88.7 | 114.0 | 1.28e-28 |
| AJO72719.1 | dTDP-4-keto-6-deoxy-hexose\_2,3-reductase | BGC0001381 | Polyketide | 27.0 | 88.2 | 112.0 | 5.81e-28 |
| BAQ21942.1 | putative\_TDP-sugar\_2,3-reductase | BGC0001204 | Polyketide | 27.0 | 85.3 | 110.0 | 3.06e-27 |
| ctg1\_37 |  | BGC0001931 | Polyketide | 27.0 | 88.2 | 108.0 | 3.09e-26 |
| BAA84599.1 | dTDP-4-keto-6-deoxy-L-hexose\_2,3-reductase | BGC0000025 | Polyketide | 25.0 | 85.3 | 103.0 | 1.68e-24 |
| AFP87517.1 | oxidoreductase | BGC0001159 | NRP+Polyketide:Modular type I polyketide | 26.0 | 86.9 | 91.0 | 3.34e-20 |
| CAE17537.1 | side-chain\_ketoreductase | BGC0000210 | Polyketide:Type II polyketide+Saccharide:Oligosaccharide | 29.0 | 73.6 | 86.0 | 2.97e-18 |
| CAK50787.1 | putative\_side\_chain\_reductase | BGC0000247 | Polyketide:Type II polyketide+Saccharide:Oligosaccharide | 26.0 | 87.7 | 84.0 | 9.99e-18 |
| AAK83185.1 | putative\_oxidoreductase | BGC0000026 | Saccharide:Oligosaccharide | 25.0 | 87.7 | 84.0 | 1.04e-17 |
| QVQ68806.1 | mmyW | BGC0002129 | Polyketide | 28.0 | 68.3 | 79.0 | 4.53e-16 |
| CAD19081.1 | potassium\_channel\_beta\_chain | BGC0000153 | NRP+Polyketide:Modular type I polyketide | 27.0 | 87.7 | 76.0 | 5.9e-15 |
| AFH74311.1 | Cvm1-like\_aldo/keto\_reductase | BGC0001151 | Other:Non-NRP beta-lactam | 25.0 | 86.4 | 76.0 | 8.7e-15 |
| KDN80036.1 | oxidoreductase | BGC0001074 | Saccharide+Polyketide | 25.0 | 73.3 | 74.0 | 1.24e-14 |
| ATJ00785.1 | aldo/keto\_reductase | BGC0001568 | Polyketide | 27.0 | 70.2 | 72.0 | 1.03e-13 |
| QLJ99328.1 | aldo/keto\_reductase | BGC0002088 | Polyketide+Saccharide:Oligosaccharide | 24.0 | 85.6 | 65.0 | 3.38e-11 |
| AAS79441.1 | conserved\_hypothetical\_protein | BGC0000035 | Polyketide | 25.0 | 74.6 | 64.0 | 4.38e-11 |
| BAG22769.1 | putative\_oxidoreductase | BGC0000724 | Saccharide | 27.0 | 45.8 | 54.0 | 1.34e-07 |
