# Supplementary material for: Uncovering the transcriptional landscape of Fomes fomentarius during fungal-based material production through gene co-expression network analysis
Source: Fungal Biol Biotechnol. 2025 Feb 13;12:1. doi: 10.1186/s40694-024-00192-3 (PMC11827164; doi:10.1186/s40694-024-00192-3)
Supplement: Supplementary file 1 — Supplementary Material 1 [file 40694_2024_192_MOESM1_ESM.zip › knownclusterblast/region1/jgi.p_Fomfom1_1373023_mibig_hits.html]

| MIBiG Protein | Description | MIBiG Cluster | MiBiG Product | % ID | % Coverage | BLAST Score | E-value |
| --- | --- | --- | --- | --- | --- | --- | --- |
| XP\_007301850.1 | cytochrome\_P450 | BGC0001617 | Terpene | 60.0 | 89.7 | 627.0 | 4.84e-223 |
| XP\_007301602.1 | cytochrome\_P450 | BGC0001617 | Terpene | 51.0 | 91.0 | 527.0 | 9.12e-184 |
| QJQ03972.1 | CYP-Arm3 | BGC0002445 | Terpene | 48.0 | 94.2 | 492.0 | 3.83e-170 |
| KAA1470686.1 | cytochrome\_P450 | BGC0002218 | Terpene | 47.0 | 90.1 | 485.0 | 1.58e-167 |
| QJQ03971.1 | CYP-Arm2 | BGC0002445 | Terpene | 48.0 | 94.6 | 471.0 | 6.03e-162 |
| XP\_007301852.1 | cytochrome\_P450 | BGC0001617 | Terpene | 44.0 | 92.2 | 461.0 | 2.9e-158 |
| XP\_007301851.1 | cytochrome\_P450 | BGC0001617 | Terpene | 39.0 | 89.0 | 378.0 | 2.63e-125 |
| EHK18434.1 | hypothetical\_protein | BGC0002233 | Polyketide | 33.0 | 94.0 | 312.0 | 9.64e-100 |
| QJQ03974.1 | CYP-Arm4 | BGC0002445 | Terpene | 36.0 | 89.0 | 303.0 | 1.1e-96 |
| AQZ42158.1 | putative\_cytochrome\_P450 | BGC0001820 | NRP | 33.0 | 93.8 | 301.0 | 2.27e-95 |
| FAC38\_04 |  | BGC0002198 | NRP | 36.0 | 90.5 | 298.0 | 8.61e-95 |
| DAB41655.1 | cytochrome\_P450\_monooxygenase | BGC0001585 | Alkaloid | 33.0 | 96.1 | 298.0 | 2.22e-94 |
| EHA22193.1 | hypothetical\_protein | BGC0000170 | Polyketide | 33.0 | 93.1 | 296.0 | 1.59e-93 |
| EYE95339.1 | cytochrome\_P450 | BGC0002234 | Polyketide | 31.0 | 93.1 | 291.0 | 1.38e-91 |
| KJA16708.1 | hypothetical\_protein | BGC0002246 | Terpene | 34.0 | 89.2 | 288.0 | 1.27e-90 |
| QBC75451.1 | MacC | BGC0002615 | Terpene | 31.0 | 97.4 | 288.0 | 1.99e-90 |
| QGW49097.1 | putative\_cytochrome\_P450 | BGC0002731 | Polyketide | 35.0 | 95.7 | 286.0 | 4.24e-90 |
| OJJ99915.1 | hypothetical\_protein | BGC0002225 | Terpene | 34.0 | 96.1 | 279.0 | 1.99e-87 |
| CEN60544.1 | hypothetical\_protein | BGC0002266 | Terpene+Polyketide | 36.0 | 87.1 | 269.0 | 2.52e-83 |
| BCI98769.1 | putative\_cytochrome\_P450 | BGC0002181 | Terpene | 33.0 | 92.5 | 266.0 | 2.65e-82 |
| BAE60012.1 |  | BGC0001518 | Terpene | 31.0 | 89.7 | 263.0 | 3e-81 |
| PPQ83215.1 | Dimethyltryptamine\_4-hydroxylase\_(PsiH) | BGC0002207 | Other | 32.0 | 88.4 | 262.0 | 4.79e-81 |
| KIJ60843.1 | hypothetical\_protein | BGC0002214 | Polyketide | 33.0 | 87.3 | 260.0 | 8.09e-81 |
| EIN09540.1 | cytochrome\_P450 | BGC0002213 | Polyketide | 33.0 | 95.3 | 261.0 | 1.14e-80 |
| QQO98481.1 | FrzL | BGC0002146 | NRP | 32.0 | 91.8 | 261.0 | 1.64e-80 |
| QJQ03970.1 | CYP-Arm1 | BGC0002445 | Terpene | 31.0 | 98.9 | 261.0 | 2.26e-80 |
| CBF82795.1 | cytochrome\_P450,\_putative\_(Eurofung) | BGC0001668 | NRP | 33.0 | 92.2 | 261.0 | 2.56e-80 |
| OJJ97582.1 | hypothetical\_protein | BGC0002229 | Polyketide | 30.0 | 95.7 | 258.0 | 3.99e-79 |
| OJJ97580.1 | hypothetical\_protein | BGC0002229 | Polyketide | 31.0 | 93.3 | 255.0 | 4.05e-78 |
| BCI98773.1 | putative\_cytochrome\_P450 | BGC0002181 | Terpene | 32.0 | 90.3 | 252.0 | 5.21e-77 |
| KIJ60846.1 | hypothetical\_protein | BGC0002214 | Polyketide | 31.0 | 94.4 | 249.0 | 4.54e-76 |
| PPQ83216.1 | Dimethyrltryptamine\_4-hydroxylase\_(PsiH) | BGC0002207 | Other | 31.0 | 86.4 | 246.0 | 3.99e-75 |
| KIJ60837.1 | hypothetical\_protein | BGC0002214 | Polyketide | 31.0 | 91.6 | 245.0 | 2.36e-74 |
| EAL85116.1 | cytochrome\_P450\_oxidoreductase | BGC0001067 | Terpene+Polyketide:Iterative type I polyketide | 31.0 | 91.8 | 243.0 | 2.33e-73 |
| OQD69071.1 | hypothetical\_protein | BGC0002745 | Polyketide | 29.0 | 95.9 | 238.0 | 1.38e-71 |
| EIM84826.1 | cytochrome\_P450 | BGC0002219 | Terpene | 34.0 | 86.9 | 238.0 | 1.57e-71 |
| KIJ60841.1 | hypothetical\_protein | BGC0002214 | Polyketide | 30.0 | 88.2 | 227.0 | 1.53e-67 |
| CBF82292.1 | cytochrome\_P450,\_putative\_(Eurofung) | BGC0002180 | Polyketide | 31.0 | 91.6 | 224.0 | 2.83e-66 |
| AAS90013.1 | OrdA | BGC0000007 | Polyketide | 28.0 | 93.3 | 222.0 | 1.96e-65 |
| AAS90081.1 | OrdA | BGC0000010 | Polyketide | 28.0 | 93.3 | 221.0 | 2.74e-65 |
| BAE60008.1 |  | BGC0001518 | Terpene | 32.0 | 94.4 | 221.0 | 4.57e-65 |
| AAS90035.1 | OrdA | BGC0000008 | Polyketide | 28.0 | 93.3 | 220.0 | 7.45e-65 |
| AAS90105.1 | OrdA | BGC0000006 | Polyketide | 28.0 | 93.3 | 218.0 | 3.94e-64 |
| AAS90061.1 | OrdA | BGC0000009 | Polyketide | 28.0 | 93.3 | 218.0 | 7.68e-64 |
| XP\_020057670.1 | uncharacterized\_protein | BGC0001718 | NRP | 28.0 | 95.9 | 216.0 | 2.91e-63 |
| BAE71330.1 | oxidoreductase\_A;oxidoreductase/cytochrome\_P450\_monooxygenase | BGC0000004 | Polyketide | 28.0 | 93.3 | 215.0 | 7.88e-63 |
| QJQ82463.1 | BisI | BGC0002290 | Other | 30.0 | 94.2 | 212.0 | 6.65e-62 |
| BAH23999.1 | cytochrome\_P450 | BGC0000356 | NRP+Alkaloid | 30.0 | 96.3 | 210.0 | 4.06e-61 |
| BBB04330.1 | cytochrome\_P450 | BGC0001717 | NRP | 29.0 | 97.2 | 207.0 | 8.54e-60 |
| QMS79071.1 | fumitremorgin\_C\_synthase | BGC0002198 | NRP | 30.0 | 93.5 | 207.0 | 1e-59 |
| BCI98774.1 | putative\_cytochrome\_P450 | BGC0002181 | Terpene | 28.0 | 94.8 | 199.0 | 8.09e-57 |
| EAL85111.2 | cytochrome\_P450\_oxidoreductase | BGC0001037 | NRP+Polyketide:Iterative type I polyketide | 28.0 | 86.4 | 197.0 | 3.65e-56 |
| AGK82817.1 | cytochrome\_P450-1 | BGC0001324 | Terpene | 26.0 | 89.9 | 171.0 | 7.48e-47 |
| AGK82807.1 | cytochrome\_P450-1 | BGC0001322 | Terpene | 26.0 | 89.9 | 169.0 | 6.34e-46 |
| AGK82824.1 | cytochrome\_P450-2 | BGC0001323 | Terpene | 27.0 | 86.8 | 168.0 | 8.76e-46 |
| ALS30800.1 | putative\_cytochrome\_P450\_monooxygenase | BGC0001286 | Other | 29.0 | 95.9 | 168.0 | 1.52e-45 |
| AGK82815.1 | cytochrome\_P450-2 | BGC0001324 | Terpene | 26.0 | 88.1 | 163.0 | 7.87e-44 |
| KGN46390.1 | hypothetical\_protein | BGC0001315 | Terpene | 26.0 | 87.7 | 162.0 | 1.52e-43 |
| AGK82831.1 | cytochrome\_P450-2 | BGC0001321 | Terpene | 27.0 | 85.3 | 160.0 | 7.75e-43 |
| pseudo106205\_112773 |  | BGC0001322 | Terpene | 25.0 | 86.8 | 153.0 | 2.3e-40 |
| KGN46388.1 | hypothetical\_protein | BGC0001315 | Terpene | 26.0 | 89.4 | 153.0 | 3.21e-40 |
| BAF09102.1 |  | BGC0000672 | Terpene | 27.0 | 90.1 | 150.0 | 2.46e-39 |
| NP\_001141366.1 | zealexin\_A1\_synthase | BGC0002390 | Terpene | 27.0 | 88.1 | 144.0 | 5.86e-37 |
| chr3.CM0241.850.r2.m |  | BGC0001316 | Other | 27.0 | 80.6 | 144.0 | 6.17e-37 |
| NP\_001130688.1 | uncharacterized\_protein\_LOC100191791 | BGC0002391 | Terpene | 25.0 | 86.8 | 141.0 | 4.38e-36 |
| EEF48750.1 | (S)-N-methylcoclaurine\_3'-hydroxylase\_isozyme,\_putative | BGC0002393 | Terpene | 26.0 | 86.0 | 140.0 | 6.32e-36 |
| NP\_001142188.1 | putative\_cytochrome\_P450\_superfamily\_protein | BGC0002390 | Terpene | 26.0 | 86.4 | 139.0 | 2.59e-35 |
| XP\_008665446.1 | cytochrome\_P450\_81Q32 | BGC0002391 | Terpene | 25.0 | 86.0 | 134.0 | 2.16e-33 |
| EEF48745.1 | cytochrome\_P450,\_putative | BGC0002393 | Terpene | 27.0 | 86.8 | 131.0 | 1.58e-32 |
| XP\_044963915.1 | cytochrome\_P450\_99A2-like | BGC0002395 | Terpene | 25.0 | 91.4 | 130.0 | 2.06e-32 |
| NP\_199073.1 | cytochrome\_P450\_71A16 | BGC0000669 | Terpene | 26.0 | 86.9 | 128.0 | 1.7e-31 |
| BAT00637.1 |  | BGC0002392 | Terpene | 25.0 | 86.4 | 128.0 | 2.13e-31 |
| BAT00632.1 |  | BGC0002392 | Terpene | 26.0 | 89.6 | 127.0 | 2.83e-31 |
| EEF48736.1 | cytochrome\_P450,\_putative | BGC0002393 | Terpene | 25.0 | 84.3 | 126.0 | 8.15e-31 |
| NP\_199610.1 | cytochrome\_P450,\_family\_705,\_subfamily\_A,\_polypeptide\_5 | BGC0000670 | Terpene | 24.0 | 81.7 | 125.0 | 2.24e-30 |
| BAF14091.1 |  | BGC0000671 | Terpene | 24.0 | 89.4 | 124.0 | 3.94e-30 |
| EEF48740.1 | cytochrome\_P450,\_putative | BGC0002393 | Terpene | 24.0 | 91.6 | 120.0 | 6.83e-29 |
| NP\_199072.1 | cytochrome\_P450,\_family\_705,\_subfamily\_A,\_polypeptide\_12 | BGC0000669 | Terpene | 25.0 | 79.9 | 120.0 | 1.01e-28 |
| AQU14205.1 | cytochrome\_p450 | BGC0001490 | Other:PBDE | 25.0 | 95.9 | 119.0 | 1.27e-28 |
| AHG26152.1 | putative\_cytochrome\_P450 | BGC0000812 | Alkaloid | 24.0 | 91.2 | 115.0 | 3.99e-27 |
| NP\_193270.1 | cytochrome\_P450,\_family\_705,\_subfamily\_A,\_polypeptide\_2 | BGC0001313 | Terpene | 25.0 | 78.4 | 115.0 | 5.38e-27 |
| NP\_193273.1 | cytochrome\_P450,\_family\_705,\_subfamily\_A,\_polypeptide\_4 | BGC0001313 | Terpene | 22.0 | 80.0 | 113.0 | 1.87e-26 |
| chr3.CM0241.700.r2.m |  | BGC0001316 | Other | 23.0 | 93.8 | 110.0 | 1.66e-25 |
| NP\_193271.5 | cytochrome\_P450,\_family\_705,\_subfamily\_A,\_polypeptide\_3 | BGC0001313 | Terpene | 23.0 | 81.7 | 110.0 | 2.12e-25 |
| NP\_193268.3 | cytochrome\_P450,\_family\_705,\_subfamily\_A,\_polypeptide\_1 | BGC0001313 | Terpene | 24.0 | 80.0 | 108.0 | 8.42e-25 |
| chr3.CM0241.310.r2.m |  | BGC0001316 | Other | 23.0 | 89.7 | 106.0 | 5.56e-24 |
| XP\_037497855.1 | LOW\_QUALITY\_PROTEIN:\_premnaspirodiene\_oxygenase-like | BGC0002724 | Terpene | 23.0 | 95.0 | 100.0 | 3.16e-22 |
| NP\_001329686.1 | cytochrome\_P450,\_family\_705,\_subfamily\_A,\_polypeptide\_1 | BGC0001313 | Terpene | 31.0 | 33.6 | 94.0 | 4.57e-20 |
| KGN46389.1 | hypothetical\_protein | BGC0001315 | Terpene | 23.0 | 90.7 | 91.0 | 5.32e-19 |
| Manes.12G133500 |  | BGC0001318 | Other | 32.0 | 32.5 | 88.0 | 4.2e-18 |
| ATX68108.1 | cytochrome\_P450 | BGC0001772 | Polyketide | 21.0 | 89.0 | 87.0 | 7.1e-18 |
| XP\_044963916.1 | cytochrome\_P450\_89A2-like | BGC0002395 | Terpene | 23.0 | 97.2 | 87.0 | 1.13e-17 |
| XP\_044969122.1 | cytochrome\_P450\_89A2-like | BGC0002395 | Terpene | 23.0 | 97.2 | 87.0 | 1.13e-17 |
| XP\_044969123.1 | cytochrome\_P450\_89A2-like | BGC0002395 | Terpene | 23.0 | 97.2 | 87.0 | 1.13e-17 |
| KON97023.1 | NADPH--cytochrome\_P450\_reductase | BGC0002122 | NRP | 23.0 | 78.7 | 85.0 | 7.04e-17 |
| TXD00003.1 | cytochrome\_P450 | BGC0001877 | Polyketide | 23.0 | 85.1 | 81.0 | 9.53e-16 |
| ATN39911.1 | MstQ | BGC0001664 | Terpene | 24.0 | 88.1 | 77.0 | 8.66e-15 |
| BBF25317.1 | bifunctional\_P-450:NADPH-P450\_reductase | BGC0001923 | Terpene+Polyketide | 23.0 | 84.7 | 78.0 | 1.07e-14 |
| XP\_001826052.1 |  | BGC0001995 | Terpene | 32.0 | 30.6 | 76.0 | 3.44e-14 |
| ABM21573.1 | crpE | BGC0000975 | NRP+Polyketide | 31.0 | 33.0 | 72.0 | 6.27e-13 |
| EAT85331.1 | hypothetical\_protein | BGC0002165 | Polyketide | 29.0 | 36.9 | 71.0 | 1.74e-12 |
| BAV69303.1 | PrhB | BGC0001729 | Polyketide+Terpene | 24.0 | 38.1 | 69.0 | 3.83e-12 |
| ABF86324.1 | cytochrome\_P450 | BGC0001025 | NRP+Polyketide:Trans-AT type I polyketide | 33.0 | 28.7 | 68.0 | 1.18e-11 |
| CEO59279.1 | Putative\_Benzoate\_4-monooxygenase\_cytochrome\_P450 | BGC0002278 | Alkaloid+NRP | 30.0 | 32.5 | 64.0 | 1.57e-10 |
| BAD29972.1 | P450\_monooxygenase\_1 | BGC0000676 | Terpene | 26.0 | 33.6 | 63.0 | 3.41e-10 |
| KJA16707.1 | hypothetical\_protein | BGC0002246 | Terpene | 28.0 | 31.2 | 62.0 | 8.68e-10 |
| BAQ25465.1 | cytochrome\_P450 | BGC0001264 | Polyketide | 25.0 | 34.9 | 62.0 | 1.11e-09 |
| ARE67844.1 | AbsV | BGC0001492 | Polyketide | 32.0 | 31.9 | 59.0 | 4.53e-09 |
| AEC13073.1 | fosG | BGC0000060 | Polyketide | 22.0 | 46.6 | 56.0 | 4.49e-08 |
| CEF75883.1 |  | BGC0001600 | Polyketide | 25.0 | 37.3 | 54.0 | 2.32e-07 |
| BAJ09784.1 | P450 | BGC0000146 | Polyketide | 27.0 | 29.5 | 54.0 | 2.7e-07 |
| QSV12651.1 | AvmP | BGC0002456 | Polyketide+NRP | 33.0 | 20.9 | 51.0 | 1.71e-06 |
| ctg1\_orf257 |  | BGC0001200 | Polyketide | 28.0 | 27.6 | 51.0 | 2.09e-06 |
| NP\_001318756.1 | cytochrome\_P450,\_family\_708,\_subfamily\_A,\_polypeptide\_2 | BGC0000670 | Terpene | 21.0 | 89.9 | 51.0 | 2.57e-06 |
| NP\_851152.1 | cytochrome\_P450,\_family\_708,\_subfamily\_A,\_polypeptide\_2 | BGC0000670 | Terpene | 21.0 | 89.9 | 51.0 | 2.71e-06 |
