# Supplementary material for: Uncovering the transcriptional landscape of Fomes fomentarius during fungal-based material production through gene co-expression network analysis
Source: Fungal Biol Biotechnol. 2025 Feb 13;12:1. doi: 10.1186/s40694-024-00192-3 (PMC11827164; doi:10.1186/s40694-024-00192-3)
Supplement: Supplementary file 1 — Supplementary Material 1 [file 40694_2024_192_MOESM1_ESM.zip › knownclusterblast/region1/jgi.p_Fomfom1_1373025_mibig_hits.html]

| MIBiG Protein | Description | MIBiG Cluster | MiBiG Product | % ID | % Coverage | BLAST Score | E-value |
| --- | --- | --- | --- | --- | --- | --- | --- |
| KIJ60843.1 | hypothetical\_protein | BGC0002214 | Polyketide | 40.0 | 90.7 | 338.0 | 3.46e-111 |
| QJQ03974.1 | CYP-Arm4 | BGC0002445 | Terpene | 34.0 | 98.8 | 332.0 | 3.79e-108 |
| BCI98769.1 | putative\_cytochrome\_P450 | BGC0002181 | Terpene | 36.0 | 100.6 | 328.0 | 1.27e-106 |
| XP\_007301851.1 | cytochrome\_P450 | BGC0001617 | Terpene | 36.0 | 98.8 | 325.0 | 5.88e-105 |
| PPQ83215.1 | Dimethyltryptamine\_4-hydroxylase\_(PsiH) | BGC0002207 | Other | 36.0 | 92.9 | 318.0 | 2.71e-103 |
| KIJ60846.1 | hypothetical\_protein | BGC0002214 | Polyketide | 36.0 | 97.4 | 317.0 | 1.53e-102 |
| QJQ03971.1 | CYP-Arm2 | BGC0002445 | Terpene | 36.0 | 99.4 | 316.0 | 5.7e-102 |
| QJQ03972.1 | CYP-Arm3 | BGC0002445 | Terpene | 36.0 | 100.6 | 311.0 | 4.41e-100 |
| PPQ83216.1 | Dimethyrltryptamine\_4-hydroxylase\_(PsiH) | BGC0002207 | Other | 36.0 | 92.7 | 308.0 | 3.18e-99 |
| KIJ60837.1 | hypothetical\_protein | BGC0002214 | Polyketide | 36.0 | 90.1 | 307.0 | 1.45e-98 |
| EIM84826.1 | cytochrome\_P450 | BGC0002219 | Terpene | 36.0 | 89.9 | 306.0 | 2.29e-98 |
| KAA1470686.1 | cytochrome\_P450 | BGC0002218 | Terpene | 35.0 | 99.6 | 298.0 | 7.81e-95 |
| XP\_007301850.1 | cytochrome\_P450 | BGC0001617 | Terpene | 34.0 | 100.8 | 292.0 | 1.11e-92 |
| KIJ60841.1 | hypothetical\_protein | BGC0002214 | Polyketide | 35.0 | 90.9 | 290.0 | 4.47e-92 |
| EIN09540.1 | cytochrome\_P450 | BGC0002213 | Polyketide | 33.0 | 98.4 | 290.0 | 7.22e-92 |
| XP\_007301602.1 | cytochrome\_P450 | BGC0001617 | Terpene | 34.0 | 95.4 | 288.0 | 6.38e-91 |
| KJA16708.1 | hypothetical\_protein | BGC0002246 | Terpene | 33.0 | 97.8 | 271.0 | 1.64e-84 |
| EAL85116.1 | cytochrome\_P450\_oxidoreductase | BGC0001067 | Terpene+Polyketide:Iterative type I polyketide | 32.0 | 101.2 | 271.0 | 1.82e-84 |
| CBF82795.1 | cytochrome\_P450,\_putative\_(Eurofung) | BGC0001668 | NRP | 34.0 | 97.0 | 269.0 | 1.19e-83 |
| FAC38\_04 |  | BGC0002198 | NRP | 32.0 | 96.4 | 255.0 | 1.81e-78 |
| XP\_007301852.1 | cytochrome\_P450 | BGC0001617 | Terpene | 31.0 | 99.6 | 246.0 | 4.27e-75 |
| AAS90061.1 | OrdA | BGC0000009 | Polyketide | 29.0 | 100.0 | 243.0 | 5.94e-74 |
| AAS90013.1 | OrdA | BGC0000007 | Polyketide | 29.0 | 100.0 | 243.0 | 8.33e-74 |
| AAS90105.1 | OrdA | BGC0000006 | Polyketide | 28.0 | 100.0 | 237.0 | 1.31e-71 |
| AAS90081.1 | OrdA | BGC0000010 | Polyketide | 28.0 | 100.0 | 237.0 | 1.31e-71 |
| AAS90035.1 | OrdA | BGC0000008 | Polyketide | 28.0 | 100.0 | 236.0 | 5.04e-71 |
| BAE71330.1 | oxidoreductase\_A;oxidoreductase/cytochrome\_P450\_monooxygenase | BGC0000004 | Polyketide | 28.0 | 100.0 | 234.0 | 1.94e-70 |
| OJJ97580.1 | hypothetical\_protein | BGC0002229 | Polyketide | 32.0 | 92.1 | 234.0 | 1.98e-70 |
| OJJ99915.1 | hypothetical\_protein | BGC0002225 | Terpene | 30.0 | 96.0 | 232.0 | 7.5e-70 |
| QBC75451.1 | MacC | BGC0002615 | Terpene | 31.0 | 103.6 | 229.0 | 1.31e-68 |
| EHA22193.1 | hypothetical\_protein | BGC0000170 | Polyketide | 31.0 | 104.2 | 227.0 | 1.41e-67 |
| QGW49097.1 | putative\_cytochrome\_P450 | BGC0002731 | Polyketide | 31.0 | 96.4 | 223.0 | 3.14e-66 |
| CEN60544.1 | hypothetical\_protein | BGC0002266 | Terpene+Polyketide | 30.0 | 97.8 | 217.0 | 6.34e-64 |
| AIG62145.1 | M-hydroxybenzyl\_alcohol\_hydroxylase | BGC0000120 | Polyketide:Iterative type I polyketide | 30.0 | 90.1 | 216.0 | 9.25e-64 |
| AQZ42158.1 | putative\_cytochrome\_P450 | BGC0001820 | NRP | 28.0 | 99.8 | 211.0 | 1.44e-61 |
| QDO73503.1 | PeniB | BGC0002557 | Terpene | 30.0 | 97.0 | 211.0 | 1.53e-61 |
| DAB41655.1 | cytochrome\_P450\_monooxygenase | BGC0001585 | Alkaloid | 28.0 | 98.6 | 209.0 | 5.01e-61 |
| BCI98773.1 | putative\_cytochrome\_P450 | BGC0002181 | Terpene | 27.0 | 101.8 | 209.0 | 6.83e-61 |
| BAE60012.1 |  | BGC0001518 | Terpene | 31.0 | 94.6 | 206.0 | 5.55e-60 |
| QQO98481.1 | FrzL | BGC0002146 | NRP | 32.0 | 97.6 | 206.0 | 7.74e-60 |
| AIG62144.1 | M-cresol\_hydroxylase | BGC0000120 | Polyketide:Iterative type I polyketide | 29.0 | 88.3 | 202.0 | 1.42e-58 |
| OQD69071.1 | hypothetical\_protein | BGC0002745 | Polyketide | 29.0 | 100.2 | 201.0 | 5.08e-58 |
| BBB04330.1 | cytochrome\_P450 | BGC0001717 | NRP | 27.0 | 94.0 | 201.0 | 8.23e-58 |
| XP\_020057670.1 | uncharacterized\_protein | BGC0001718 | NRP | 29.0 | 91.5 | 199.0 | 4.22e-57 |
| BCI98774.1 | putative\_cytochrome\_P450 | BGC0002181 | Terpene | 29.0 | 102.8 | 198.0 | 5.53e-57 |
| OJJ97582.1 | hypothetical\_protein | BGC0002229 | Polyketide | 29.0 | 90.5 | 197.0 | 1.68e-56 |
| EAU32821.1 | predicted\_protein | BGC0000160 | Polyketide | 29.0 | 84.1 | 181.0 | 3.48e-51 |
| CBF82292.1 | cytochrome\_P450,\_putative\_(Eurofung) | BGC0002180 | Polyketide | 28.0 | 98.6 | 179.0 | 7.82e-50 |
| EAL85111.2 | cytochrome\_P450\_oxidoreductase | BGC0001037 | NRP+Polyketide:Iterative type I polyketide | 28.0 | 96.4 | 179.0 | 9.22e-50 |
| ALS30800.1 | putative\_cytochrome\_P450\_monooxygenase | BGC0001286 | Other | 27.0 | 99.6 | 167.0 | 2.22e-45 |
| CCT72382.1 | related\_to\_O-methylsterigmatocystin\_oxidoreductase | BGC0001305 | Polyketide | 28.0 | 94.6 | 155.0 | 5.65e-41 |
| AGK82817.1 | cytochrome\_P450-1 | BGC0001324 | Terpene | 25.0 | 94.4 | 150.0 | 1.39e-39 |
| AGK82831.1 | cytochrome\_P450-2 | BGC0001321 | Terpene | 26.0 | 95.4 | 150.0 | 1.81e-39 |
| BCA42574.1 | cytochrome\_P450\_monooxygenase\_GrgG | BGC0002185 | Polyketide | 27.0 | 99.0 | 149.0 | 8.73e-39 |
| AGK82807.1 | cytochrome\_P450-1 | BGC0001322 | Terpene | 26.0 | 92.5 | 147.0 | 2.19e-38 |
| QMS79071.1 | fumitremorgin\_C\_synthase | BGC0002198 | NRP | 27.0 | 105.6 | 148.0 | 2.55e-38 |
| AGK82815.1 | cytochrome\_P450-2 | BGC0001324 | Terpene | 25.0 | 94.2 | 144.0 | 1.99e-37 |
| AHG26152.1 | putative\_cytochrome\_P450 | BGC0000812 | Alkaloid | 27.0 | 101.4 | 145.0 | 2.55e-37 |
| pseudo106205\_112773 |  | BGC0001322 | Terpene | 25.0 | 94.4 | 144.0 | 2.77e-37 |
| AGK82824.1 | cytochrome\_P450-2 | BGC0001323 | Terpene | 25.0 | 91.1 | 141.0 | 3.38e-36 |
| BAF09102.1 |  | BGC0000672 | Terpene | 27.0 | 94.6 | 138.0 | 3.49e-35 |
| XP\_008665446.1 | cytochrome\_P450\_81Q32 | BGC0002391 | Terpene | 24.0 | 94.0 | 137.0 | 1.21e-34 |
| NP\_001130688.1 | uncharacterized\_protein\_LOC100191791 | BGC0002391 | Terpene | 25.0 | 90.9 | 132.0 | 4.87e-33 |
| BAF09101.1 |  | BGC0000672 | Terpene | 26.0 | 89.9 | 128.0 | 1.08e-31 |
| BAF09098.1 |  | BGC0000672 | Terpene | 27.0 | 93.7 | 125.0 | 1.04e-30 |
| EER93095.1 | hypothetical\_protein | BGC0000798 | Saccharide | 26.0 | 95.2 | 125.0 | 3.3e-30 |
| BAF09099.1 |  | BGC0000672 | Terpene | 27.0 | 90.9 | 122.0 | 8.77e-30 |
| BAF14086.1 |  | BGC0000671 | Terpene | 26.0 | 86.5 | 117.0 | 8.48e-28 |
| EEF48734.1 | cytochrome\_P450,\_putative | BGC0002393 | Terpene | 26.0 | 93.7 | 117.0 | 8.76e-28 |
| XP\_044984106.1 | indole-2-monooxygenase-like | BGC0002721 | Saccharide | 27.0 | 84.1 | 117.0 | 9.19e-28 |
| EEF48738.1 | cytochrome\_P450,\_putative | BGC0002393 | Terpene | 26.0 | 88.7 | 113.0 | 2.12e-26 |
| chr3.CM0292.110.r2.m |  | BGC0001317 | Terpene | 25.0 | 89.7 | 111.0 | 5.43e-26 |
| BAT00632.1 |  | BGC0002392 | Terpene | 24.0 | 95.2 | 111.0 | 6.26e-26 |
| AFW60200.1 | benzoxazinone\_synthesis3 | BGC0000810 | Alkaloid | 23.0 | 92.1 | 109.0 | 3.08e-25 |
| EEF48740.1 | cytochrome\_P450,\_putative | BGC0002393 | Terpene | 25.0 | 92.3 | 109.0 | 3.84e-25 |
| XP\_037497843.1 | premnaspirodiene\_oxygenase | BGC0002724 | Terpene | 25.0 | 86.7 | 108.0 | 5.17e-25 |
| AFW60202.1 | benzoxazinone\_synthesis5 | BGC0000810 | Alkaloid | 25.0 | 91.9 | 108.0 | 5.5e-25 |
| EEF48750.1 | (S)-N-methylcoclaurine\_3'-hydroxylase\_isozyme,\_putative | BGC0002393 | Terpene | 24.0 | 93.7 | 108.0 | 7.86e-25 |
| XP\_044984006.1 | 4-hydroxyphenylacetaldehyde\_oxime\_monooxygenase-like | BGC0002721 | Saccharide | 24.0 | 93.5 | 106.0 | 3.08e-24 |
| EEF48742.1 | cytochrome\_P450,\_putative | BGC0002393 | Terpene | 24.0 | 91.1 | 103.0 | 2.04e-23 |
| AQU14205.1 | cytochrome\_p450 | BGC0001490 | Other:PBDE | 24.0 | 91.1 | 102.0 | 6.38e-23 |
| QNL15172.1 | AetB | BGC0002643 | Alkaloid | 22.0 | 93.3 | 99.0 | 8.62e-22 |
| Manes.12G132800 |  | BGC0001318 | Other | 35.0 | 32.7 | 95.0 | 1.84e-20 |
| EEF48736.1 | cytochrome\_P450,\_putative | BGC0002393 | Terpene | 24.0 | 87.3 | 94.0 | 3.07e-20 |
| KGN46389.1 | hypothetical\_protein | BGC0001315 | Terpene | 24.0 | 101.6 | 93.0 | 7.91e-20 |
| Manes.12G132900 |  | BGC0001318 | Other | 33.0 | 33.1 | 93.0 | 1.02e-19 |
| XP\_037497855.1 | LOW\_QUALITY\_PROTEIN:\_premnaspirodiene\_oxygenase-like | BGC0002724 | Terpene | 24.0 | 93.7 | 93.0 | 1.1e-19 |
| EEF48748.1 | cytochrome\_P450,\_putative | BGC0002393 | Terpene | 23.0 | 77.4 | 92.0 | 1.14e-19 |
| Manes.12G133500 |  | BGC0001318 | Other | 35.0 | 29.6 | 87.0 | 1.11e-17 |
| XP\_044984175.1 | tyrosine\_N-monooxygenase-like | BGC0002721 | Saccharide | 22.0 | 104.2 | 82.0 | 3.2e-16 |
| CYP82Y1 |  | BGC0001325 | Alkaloid | 24.0 | 95.2 | 80.0 | 1.91e-15 |
| OJJ99917.1 | hypothetical\_protein | BGC0002225 | Terpene | 33.0 | 31.7 | 76.0 | 2.8e-14 |
| EAL89317.1 | cytochrome\_P450\_monooxygenase,\_putative | BGC0000686 | Terpene | 31.0 | 39.1 | 74.0 | 1.37e-13 |
| NP\_198460.1 | cytochrome\_P450,\_family\_716,\_subfamily\_A,\_polypeptide\_1 | BGC0001314 | Terpene | 24.0 | 92.5 | 73.0 | 2.57e-13 |
| AAK53577.1 | trichodiene\_oxygenase | BGC0000930 | Terpene | 24.0 | 71.2 | 73.0 | 2.86e-13 |
| EAL89316.1 | cytochrome\_P450\_monooxygenase,\_putative | BGC0000686 | Terpene | 31.0 | 32.3 | 72.0 | 3.69e-13 |
| GAP90685.1 | putative\_benzoate\_4-monooxygenase\_cytochrome | BGC0002651 | Terpene | 23.0 | 76.6 | 72.0 | 3.83e-13 |
| BAT32890.1 | stellatic\_acid\_synthase | BGC0002610 | Terpene | 30.0 | 39.5 | 72.0 | 5.05e-13 |
| AAK33073.1 | cytochrome\_P450 | BGC0001278 | Terpene | 24.0 | 95.6 | 72.0 | 6.67e-13 |
| AAK33083.1 | putative\_cytochrome\_P450 | BGC0001277 | Terpene | 25.0 | 97.2 | 71.0 | 8.85e-13 |
| NP\_198462.1 | Cytochrome\_P450\_superfamily\_protein | BGC0001314 | Terpene | 36.0 | 20.6 | 65.0 | 2.54e-12 |
| AQA28566.1 | cytochrome\_P450 | BGC0001663 | Polyketide | 22.0 | 90.5 | 69.0 | 5.53e-12 |
| BAX01960.1 | trichodiene\_oxygenase | BGC0001811 | Terpene | 24.0 | 71.2 | 66.0 | 6.02e-11 |
| AHA38204.1 | GphK | BGC0000069 | Polyketide | 32.0 | 31.7 | 64.0 | 1.19e-10 |
| QPP46751.1 | cytochrome\_P450 | BGC0002500 | Polyketide | 29.0 | 35.1 | 64.0 | 1.29e-10 |
| CAL58689.1 | cytochrome\_P450\_dependent\_monooxygenase | BGC0000149 | Polyketide:Modular type I polyketide | 23.0 | 89.5 | 62.0 | 6.65e-10 |
| EAL89312.1 | cytochrome\_P450\_monooxygenase,\_putative | BGC0000686 | Terpene | 29.0 | 28.0 | 62.0 | 9.68e-10 |
| EHK21999.1 | hypothetical\_protein | BGC0001609 | NRP | 31.0 | 31.9 | 59.0 | 6.87e-09 |
| EAU36841.1 | predicted\_protein | BGC0002736 | Terpene | 25.0 | 34.9 | 56.0 | 5.37e-08 |
| AQM58280.1 | cytochrome\_P450 | BGC0001816 | NRP+Polyketide | 25.0 | 37.5 | 56.0 | 6.66e-08 |
| ADD83003.1 | PtnO2 | BGC0001156 | Terpene | 31.0 | 21.6 | 49.0 | 8.97e-06 |
