# Supplementary material for: Uncovering the transcriptional landscape of Fomes fomentarius during fungal-based material production through gene co-expression network analysis
Source: Fungal Biol Biotechnol. 2025 Feb 13;12:1. doi: 10.1186/s40694-024-00192-3 (PMC11827164; doi:10.1186/s40694-024-00192-3)
Supplement: Supplementary file 1 — Supplementary Material 1 [file 40694_2024_192_MOESM1_ESM.zip › knownclusterblast/region1/jgi.p_Fomfom1_314476_mibig_hits.html]

| MIBiG Protein | Description | MIBiG Cluster | MiBiG Product | % ID | % Coverage | BLAST Score | E-value |
| --- | --- | --- | --- | --- | --- | --- | --- |
| AKC91846.1 | amino\_acid\_permease | BGC0001414 | NRP | 43.0 | 96.4 | 331.0 | 7.48e-109 |
| RSO11789.1 | amino\_acid\_permease | BGC0002637 | NRP | 40.0 | 98.0 | 326.0 | 4.75e-107 |
| ACN29726.1 | amino\_acid/metabolite\_permease | BGC0000814 | Alkaloid | 40.0 | 103.1 | 299.0 | 2.22e-96 |
| AQV04225.1 | putative\_amino\_acid\_transporter | BGC0001794 | NRP+Polyketide | 32.0 | 100.0 | 226.0 | 1.37e-68 |
