# Supplementary material for: Uncovering the transcriptional landscape of Fomes fomentarius during fungal-based material production through gene co-expression network analysis
Source: Fungal Biol Biotechnol. 2025 Feb 13;12:1. doi: 10.1186/s40694-024-00192-3 (PMC11827164; doi:10.1186/s40694-024-00192-3)
Supplement: Supplementary file 1 — Supplementary Material 1 [file 40694_2024_192_MOESM1_ESM.zip › knownclusterblast/region1/jgi.p_Fomfom1_314666_mibig_hits.html]

| MIBiG Protein | Description | MIBiG Cluster | MiBiG Product | % ID | % Coverage | BLAST Score | E-value |
| --- | --- | --- | --- | --- | --- | --- | --- |
| EIW83693.1 | terpenoid\_synthase | BGC0002707 | Terpene | 33.0 | 106.6 | 175.0 | 3.24e-52 |
| EIW83595.1 | terpene\_synthase | BGC0002708 | Terpene | 34.0 | 104.7 | 162.0 | 4.26e-47 |
| ATZ56107.1 | Bcbot2 | BGC0000631 | Terpene | 26.0 | 68.8 | 91.0 | 2.51e-20 |
| CCT72694.1 | related\_to\_pentalenene\_synthase | BGC0001642 | Terpene | 26.0 | 65.4 | 79.0 | 2.82e-16 |
| QDO73502.1 | PeniA | BGC0002557 | Terpene | 22.0 | 110.0 | 79.0 | 4e-16 |
