# Supplementary material for: Uncovering the transcriptional landscape of Fomes fomentarius during fungal-based material production through gene co-expression network analysis
Source: Fungal Biol Biotechnol. 2025 Feb 13;12:1. doi: 10.1186/s40694-024-00192-3 (PMC11827164; doi:10.1186/s40694-024-00192-3)
Supplement: Supplementary file 1 — Supplementary Material 1 [file 40694_2024_192_MOESM1_ESM.zip › knownclusterblast/region1/jgi.p_Fomfom1_1222227_mibig_hits.html]

| MIBiG Protein | Description | MIBiG Cluster | MiBiG Product | % ID | % Coverage | BLAST Score | E-value |
| --- | --- | --- | --- | --- | --- | --- | --- |
| KON97023.1 | NADPH--cytochrome\_P450\_reductase | BGC0002122 | NRP | 24.0 | 100.2 | 151.0 | 7.73e-38 |
| TXD00031.1 | cytochrome\_P450 | BGC0001877 | Polyketide | 26.0 | 101.1 | 143.0 | 3.37e-35 |
| BBM05078.1 | putative\_bifunctional\_cytochrome\_P450/NADPH-P450\_reductase | BGC0002170 | Polyketide | 24.0 | 99.0 | 128.0 | 1.82e-30 |
| QQW45471.1 | bifunctional\_cytochrome\_P450/NADPH-P450\_reductase\_CalG' | BGC0002168 | Polyketide | 24.0 | 99.0 | 128.0 | 2.42e-30 |
| BBF25317.1 | bifunctional\_P-450:NADPH-P450\_reductase | BGC0001923 | Terpene+Polyketide | 24.0 | 97.1 | 115.0 | 3.69e-26 |
| AAM54108.1 | cytochrome\_P450 | BGC0000020 | Polyketide | 27.0 | 60.7 | 108.0 | 3.2e-24 |
