# Supplementary material for: Uncovering the transcriptional landscape of Fomes fomentarius during fungal-based material production through gene co-expression network analysis
Source: Fungal Biol Biotechnol. 2025 Feb 13;12:1. doi: 10.1186/s40694-024-00192-3 (PMC11827164; doi:10.1186/s40694-024-00192-3)
Supplement: Supplementary file 1 — Supplementary Material 1 [file 40694_2024_192_MOESM1_ESM.zip › knownclusterblast/region1/jgi.p_Fomfom1_377229_mibig_hits.html]

| MIBiG Protein | Description | MIBiG Cluster | MiBiG Product | % ID | % Coverage | BLAST Score | E-value |
| --- | --- | --- | --- | --- | --- | --- | --- |
| QJS40199.1 | 5-enolpyruvyl-shikimate-3-phosphate\_synthase | BGC0002439 | Other | 38.0 | 27.9 | 201.0 | 7.83e-56 |
| WP\_016325509.1 | 3-phosphoshikimate\_1-carboxyvinyltransferase | BGC0001283 | Polyketide | 32.0 | 27.4 | 197.0 | 2.25e-54 |
| ACF35443.1 | mbcO | BGC0000090 | Polyketide | 33.0 | 22.9 | 162.0 | 4.76e-43 |
| WP\_020275084.1 | 3-phosphoshikimate\_1-carboxyvinyltransferase | BGC0002012 | Polyketide | 32.0 | 28.2 | 150.0 | 4.01e-38 |
| ADI58632.1 | 5-Enolpyruvylshikimate-3-phosphate\_synthase/CHC-CoA\_ligase | BGC0000187 | Polyketide:Type II polyketide | 29.0 | 30.2 | 130.0 | 4.01e-30 |
| CAH60159.1 | 2-deoxy-scyllo-inosose\_synthase | BGC0000700 | Saccharide | 29.0 | 26.8 | 122.0 | 3.58e-29 |
| ADZ13553.1 | YtkP | BGC0000466 | NRP | 29.0 | 20.3 | 108.0 | 1.2e-24 |
| AHH25585.1 | AMP-dependent\_synthetase\_and\_ligase | BGC0000957 | NRP+Polyketide | 27.0 | 23.1 | 84.0 | 1.07e-15 |
