# Supplementary material for: Uncovering the transcriptional landscape of Fomes fomentarius during fungal-based material production through gene co-expression network analysis
Source: Fungal Biol Biotechnol. 2025 Feb 13;12:1. doi: 10.1186/s40694-024-00192-3 (PMC11827164; doi:10.1186/s40694-024-00192-3)
Supplement: Supplementary file 1 — Supplementary Material 1 [file 40694_2024_192_MOESM1_ESM.zip › knownclusterblast/region2/jgi.p_Fomfom1_1228059_mibig_hits.html]

| MIBiG Protein | Description | MIBiG Cluster | MiBiG Product | % ID | % Coverage | BLAST Score | E-value |
| --- | --- | --- | --- | --- | --- | --- | --- |
| ADA00385.1 | signal\_recognition\_particle\_protein\_SRP | BGC0000472 | RiPP:Cyanobactin | 33.0 | 76.4 | 206.0 | 1.56e-59 |
| BAE46928.1 | goadsporin\_biosynthetic\_protein | BGC0000565 | RiPP:LAP | 32.0 | 78.3 | 195.0 | 4.66e-55 |
