# Supplementary material for: Uncovering the transcriptional landscape of Fomes fomentarius during fungal-based material production through gene co-expression network analysis
Source: Fungal Biol Biotechnol. 2025 Feb 13;12:1. doi: 10.1186/s40694-024-00192-3 (PMC11827164; doi:10.1186/s40694-024-00192-3)
Supplement: Supplementary file 1 — Supplementary Material 1 [file 40694_2024_192_MOESM1_ESM.zip › knownclusterblast/region2/jgi.p_Fomfom1_1363054_mibig_hits.html]

| MIBiG Protein | Description | MIBiG Cluster | MiBiG Product | % ID | % Coverage | BLAST Score | E-value |
| --- | --- | --- | --- | --- | --- | --- | --- |
| AAS92542.1 | SirJ | BGC0001044 | NRP | 44.0 | 79.6 | 287.0 | 4.41e-93 |
| BAE56605.1 |  | BGC0001123 | NRP | 44.0 | 81.8 | 288.0 | 5.15e-93 |
| QVK45105.1 | membrane\_dipeptidase | BGC0002438 | Alkaloid | 41.0 | 87.9 | 281.0 | 1.68e-89 |
| CCE28982.1 | related\_to\_microsomal\_dipeptidase\_precursor | BGC0001365 | NRP | 39.0 | 83.4 | 246.0 | 1.03e-76 |
| AQZ42165.1 | putative\_membrane\_dipeptidase | BGC0001820 | NRP | 46.0 | 64.3 | 237.0 | 1.62e-74 |
| EAU36747.1 | hypothetical\_protein | BGC0000292 | NRP | 38.0 | 83.6 | 239.0 | 3.21e-74 |
| QPP19386.1 | PenJ | BGC0002501 | Alkaloid | 51.0 | 61.3 | 240.0 | 5.44e-74 |
| ctg1\_orf8 |  | BGC0000321 | NRP | 39.0 | 30.8 | 96.0 | 4.84e-23 |
