# Supplementary material for: Uncovering the transcriptional landscape of Fomes fomentarius during fungal-based material production through gene co-expression network analysis
Source: Fungal Biol Biotechnol. 2025 Feb 13;12:1. doi: 10.1186/s40694-024-00192-3 (PMC11827164; doi:10.1186/s40694-024-00192-3)
Supplement: Supplementary file 1 — Supplementary Material 1 [file 40694_2024_192_MOESM1_ESM.zip › knownclusterblast/region2/jgi.p_Fomfom1_1187483_mibig_hits.html]

| MIBiG Protein | Description | MIBiG Cluster | MiBiG Product | % ID | % Coverage | BLAST Score | E-value |
| --- | --- | --- | --- | --- | --- | --- | --- |
| KYC42615.1 | hypothetical\_protein | BGC0002484 | NRP+Polyketide | 26.0 | 63.2 | 67.0 | 6.82e-12 |
| QSJ20132.1 | cupin-like\_domain-containing\_protein | BGC0002572 | NRP+Polyketide | 28.0 | 63.6 | 66.0 | 1.78e-11 |
| KYC42616.1 | hypothetical\_protein | BGC0002484 | NRP+Polyketide | 27.0 | 63.2 | 65.0 | 2.39e-11 |
