# Supplementary material for: Uncovering the transcriptional landscape of Fomes fomentarius during fungal-based material production through gene co-expression network analysis
Source: Fungal Biol Biotechnol. 2025 Feb 13;12:1. doi: 10.1186/s40694-024-00192-3 (PMC11827164; doi:10.1186/s40694-024-00192-3)
Supplement: Supplementary file 1 — Supplementary Material 1 [file 40694_2024_192_MOESM1_ESM.zip › knownclusterblast/region2/jgi.p_Fomfom1_73955_mibig_hits.html]

| MIBiG Protein | Description | MIBiG Cluster | MiBiG Product | % ID | % Coverage | BLAST Score | E-value |
| --- | --- | --- | --- | --- | --- | --- | --- |
| CAJ96465.1 | ornithine\_N-monooxygenase | BGC0000330 | NRP:NRP siderophore | 35.0 | 96.5 | 259.0 | 4.98e-80 |
| ADJ63845.1 | L-ornithine\_N5-monooxygenase\_protein | BGC0000424 | NRP:NRP siderophore | 35.0 | 95.6 | 258.0 | 7.34e-80 |
| NAO96324.1 | SidA/IucD/PvdA\_family\_monooxygenase | BGC0002117 | NRP | 35.0 | 97.3 | 254.0 | 1.12e-78 |
| ACO78740.1 | L-ornithine\_N5-oxygenase-PvdA-like\_protein | BGC0002433 | NRP | 37.0 | 75.3 | 251.0 | 3.11e-77 |
| ABM34270.1 | L-lysine\_6-monooxygenase\_(NADPH) | BGC0002419 | NRP+Polyketide | 35.0 | 96.3 | 249.0 | 1.18e-76 |
| WP\_039806874.1 | lysine\_N(6)-hydroxylase/L-ornithine\_N(5)-oxygenase\_family\_protein | BGC0002001 | NRP+Polyketide | 33.0 | 97.6 | 249.0 | 1.38e-76 |
| ACS20356.1 | L-lysine\_6-monooxygenase\_(NADPH) | BGC0002420 | NRP+Polyketide | 33.0 | 99.3 | 248.0 | 3.21e-76 |
| ABC39452.1 | l-ornithine\_5-monooxygenase | BGC0000386 | NRP:NRP siderophore | 34.0 | 99.6 | 248.0 | 2.48e-75 |
| CAQ71838.1 | L-ornithine\_5-monooxygenase(L-ornithine\_N5-oxygenase)\_L-lysine\_6-monooxygenase\_(Lysine\_N(6)-hydroxylase);\_Siderophore\_biosynthesis\_protein | BGC0001189 | NRP | 34.0 | 97.4 | 243.0 | 3.73e-74 |
| CAL17537.1 | L-ornithine\_N5-oxygenase | BGC0002465 | NRP | 40.0 | 73.5 | 239.0 | 6.1e-73 |
| ALG65346.1 | Var11 | BGC0002416 | NRP+Polyketide | 34.0 | 97.8 | 239.0 | 7.77e-73 |
| QBQ12451.1 | ornithine\_monooxygenase | BGC0002693 | NRP | 37.0 | 74.4 | 239.0 | 9.11e-73 |
| AGU50947.1 | putative\_monooxygenase | BGC0002417 | NRP+Polyketide | 32.0 | 98.4 | 239.0 | 1.46e-72 |
| ABE35420.1 | l-ornithine\_5-monooxygenase | BGC0002421 | NRP | 34.0 | 97.8 | 239.0 | 1.71e-72 |
| AAY93340.1 | L-ornithine\_5-monooxygenase\_PvdA | BGC0000413 | NRP | 36.0 | 75.0 | 236.0 | 1.36e-71 |
| CAR51997.1 | putative\_L-ornithine\_5-monooxygenase | BGC0002569 | NRP | 35.0 | 99.6 | 235.0 | 3.75e-71 |
| ABX37378.1 | L-lysine\_6-monooxygenase\_(NADPH) | BGC0000984 | NRP+Polyketide | 37.0 | 72.9 | 234.0 | 5.22e-71 |
| NKI69297.1 | SidA/IucD/PvdA\_family\_monooxygenase | BGC0002408 | NRP | 33.0 | 99.5 | 231.0 | 1.07e-69 |
| AJD47500.1 | L-lysine\_6-monooxygenase | BGC0002418 | NRP+Polyketide | 32.0 | 97.8 | 228.0 | 1.28e-68 |
| WP\_039794392.1 | lysine\_N(6)-hydroxylase/L-ornithine\_N(5)-oxygenase\_family\_protein | BGC0001211 | NRP | 37.0 | 63.3 | 208.0 | 6.38e-61 |
| QYI86760.1 | putative\_L-ornithine\_N5-oxygenase | BGC0002424 | NRP | 34.0 | 72.2 | 207.0 | 1.54e-60 |
| WP\_028847741.1 | lysine\_N(6)-hydroxylase/L-ornithine\_N(5)-oxygenase\_family\_protein | BGC0002467 | NRP | 37.0 | 60.0 | 200.0 | 4.44e-58 |
| CAB53328.1 | putative\_peptide\_monooxygenase | BGC0000325 | NRP | 37.0 | 71.5 | 199.0 | 1.48e-57 |
| ATW47209.1 | L-lysine\_6-monooxygenase | BGC0002466 | NRP | 35.0 | 74.0 | 199.0 | 2.07e-57 |
| WP\_141576255.1 | lysine\_N(6)-hydroxylase/L-ornithine\_N(5)-oxygenase\_family\_protein | BGC0002686 | NRP | 34.0 | 72.8 | 197.0 | 3.51e-57 |
| EFL06875.1 | peptide\_monooxygenase | BGC0000300 | NRP | 35.0 | 71.8 | 196.0 | 1.18e-56 |
| MBD2892720.1 | L-ornithine\_N(5)-monooxygenase | BGC0002718 | NRP | 36.0 | 66.9 | 195.0 | 3.76e-56 |
| AAZ55902.1 | putative\_peptide\_monooxygenase | BGC0000359 | NRP | 34.0 | 72.4 | 192.0 | 4.3e-55 |
| ctg1\_orf18 |  | BGC0001767 | NRP | 35.0 | 71.8 | 192.0 | 7.18e-55 |
| CAM02311.1 | putative\_peptide\_monooxygenase | BGC0000349 | NRP | 34.0 | 71.7 | 190.0 | 2.41e-54 |
| QIE08741.1 | ornithine\_N-monooxygenase | BGC0002544 | NRP | 36.0 | 58.1 | 189.0 | 6.2e-54 |
| WP\_019634560.1 | lysine\_N(6)-hydroxylase/L-ornithine\_N(5)-oxygenase\_family\_protein | BGC0001443 | NRP+Polyketide | 38.0 | 59.2 | 188.0 | 1.42e-53 |
| CBG75497.1 | putative\_peptide\_N-oxygenase | BGC0000423 | NRP | 37.0 | 59.6 | 189.0 | 1.63e-53 |
| MBE3202949.1 | lysine\_N(6)-hydroxylase/L-ornithine\_N(5)-oxygenase\_family\_protein | BGC0002410 | NRP | 36.0 | 60.3 | 185.0 | 3.93e-52 |
| ctg1\_orf1261 |  | BGC0001752 | NRP | 34.0 | 72.2 | 184.0 | 1.27e-51 |
| ACY06283.1 | L-lysine\_6-monooxygenase | BGC0001042 | NRP+Polyketide | 33.0 | 72.4 | 183.0 | 1.41e-51 |
| CBZ42147.1 | peptide\_monooxygenase | BGC0001117 | NRP | 33.0 | 73.5 | 182.0 | 2.78e-51 |
| AMK48246.1 | L-ornithine-5-monooxygenase | BGC0001351 | NRP | 36.0 | 60.3 | 181.0 | 4.71e-51 |
| AJV88381.1 | MfnI | BGC0001214 | NRP | 34.0 | 72.0 | 179.0 | 2.72e-50 |
| AFJ20768.1 | ornithine\_monooxygenase | BGC0002300 | NRP | 35.0 | 59.2 | 178.0 | 7.71e-50 |
| AGE11900.1 | L-ornithine\_5-monooxygenase | BGC0000366 | NRP | 32.0 | 71.8 | 177.0 | 2.52e-49 |
| AUD11991.1 | PvdA | BGC0001721 | NRP | 32.0 | 85.9 | 167.0 | 2.83e-46 |
| ABV56589.1 | KtzI | BGC0000378 | NRP | 30.0 | 71.8 | 160.0 | 2.03e-43 |
| BAC87904.1 | probable\_acinetobactin\_biosynthesis\_protein | BGC0000294 | NRP | 28.0 | 74.2 | 137.0 | 3.71e-35 |
| ABS50185.1 | lysine\_6-monooxygenase-like\_protein | BGC0000410 | NRP | 28.0 | 66.4 | 130.0 | 2.1e-32 |
| CDL83401.1 | L-lysine\_6-monooxygenase | BGC0001498 | Other | 29.0 | 58.3 | 129.0 | 4.67e-32 |
| CAM86884.1 | putative\_lysine/ornithine\_N-monooxygenase | BGC0002471 | Other | 29.0 | 58.5 | 122.0 | 1.98e-29 |
| RUP78900.1 | L-lysine\_6-monooxygenase | BGC0002073 | NRP | 27.0 | 72.8 | 121.0 | 2.54e-29 |
| BAE16005.1 | lysine\_N6-monooxygenase | BGC0000939 | Other | 27.0 | 72.2 | 120.0 | 3.05e-29 |
| BAM09327.1 | putative\_acinetoferrin\_biosynthesis\_protein | BGC0000295 | NRP | 28.0 | 58.0 | 118.0 | 3.5e-28 |
