# Supplementary material for: Uncovering the transcriptional landscape of Fomes fomentarius during fungal-based material production through gene co-expression network analysis
Source: Fungal Biol Biotechnol. 2025 Feb 13;12:1. doi: 10.1186/s40694-024-00192-3 (PMC11827164; doi:10.1186/s40694-024-00192-3)
Supplement: Supplementary file 1 — Supplementary Material 1 [file 40694_2024_192_MOESM1_ESM.zip › knownclusterblast/region2/jgi.p_Fomfom1_1195869_mibig_hits.html]

| MIBiG Protein | Description | MIBiG Cluster | MiBiG Product | % ID | % Coverage | BLAST Score | E-value |
| --- | --- | --- | --- | --- | --- | --- | --- |
| EIW83693.1 | terpenoid\_synthase | BGC0002707 | Terpene | 49.0 | 94.5 | 328.0 | 1.46e-111 |
| EIW83595.1 | terpene\_synthase | BGC0002708 | Terpene | 46.0 | 97.1 | 315.0 | 3.75e-106 |
| QDO73502.1 | PeniA | BGC0002557 | Terpene | 29.0 | 91.6 | 148.0 | 3.15e-41 |
| CCT72694.1 | related\_to\_pentalenene\_synthase | BGC0001642 | Terpene | 28.0 | 87.5 | 136.0 | 1.16e-36 |
