# Supplementary material for: Uncovering the transcriptional landscape of Fomes fomentarius during fungal-based material production through gene co-expression network analysis
Source: Fungal Biol Biotechnol. 2025 Feb 13;12:1. doi: 10.1186/s40694-024-00192-3 (PMC11827164; doi:10.1186/s40694-024-00192-3)
Supplement: Supplementary file 1 — Supplementary Material 1 [file 40694_2024_192_MOESM1_ESM.zip › knownclusterblast/region2/jgi.p_Fomfom1_629213_mibig_hits.html]

| MIBiG Protein | Description | MIBiG Cluster | MiBiG Product | % ID | % Coverage | BLAST Score | E-value |
| --- | --- | --- | --- | --- | --- | --- | --- |
| EIW83595.1 | terpene\_synthase | BGC0002708 | Terpene | 61.0 | 96.8 | 442.0 | 3.61e-156 |
| EIW83693.1 | terpenoid\_synthase | BGC0002707 | Terpene | 53.0 | 95.4 | 362.0 | 1.04e-124 |
| CCT72694.1 | related\_to\_pentalenene\_synthase | BGC0001642 | Terpene | 28.0 | 90.2 | 135.0 | 3.28e-36 |
