# Supplementary material for: Uncovering the transcriptional landscape of Fomes fomentarius during fungal-based material production through gene co-expression network analysis
Source: Fungal Biol Biotechnol. 2025 Feb 13;12:1. doi: 10.1186/s40694-024-00192-3 (PMC11827164; doi:10.1186/s40694-024-00192-3)
Supplement: Supplementary file 1 — Supplementary Material 1 [file 40694_2024_192_MOESM1_ESM.zip › knownclusterblast/region2/jgi.p_Fomfom1_629377_mibig_hits.html]

| MIBiG Protein | Description | MIBiG Cluster | MiBiG Product | % ID | % Coverage | BLAST Score | E-value |
| --- | --- | --- | --- | --- | --- | --- | --- |
| EIW83694.1 | aldo-keto\_reductase | BGC0002707 | Terpene | 62.0 | 99.3 | 367.0 | 1.59e-128 |
| AHI59116.1 | putative\_2,5-diketo-D-gluconic\_acid\_reductase | BGC0001005 | NRP+Polyketide | 41.0 | 92.5 | 189.0 | 6.68e-59 |
| ASK38702.1 | putative\_aldo/keto\_reductase | BGC0001436 | Polyketide:Iterative type I polyketide | 30.0 | 100.7 | 127.0 | 1.41e-34 |
