# Supplementary material for: Uncovering the transcriptional landscape of Fomes fomentarius during fungal-based material production through gene co-expression network analysis
Source: Fungal Biol Biotechnol. 2025 Feb 13;12:1. doi: 10.1186/s40694-024-00192-3 (PMC11827164; doi:10.1186/s40694-024-00192-3)
Supplement: Supplementary file 1 — Supplementary Material 1 [file 40694_2024_192_MOESM1_ESM.zip › knownclusterblast/region2/jgi.p_Fomfom1_888032_mibig_hits.html]

| MIBiG Protein | Description | MIBiG Cluster | MiBiG Product | % ID | % Coverage | BLAST Score | E-value |
| --- | --- | --- | --- | --- | --- | --- | --- |
| QBE85645.1 | BuaE | BGC0001857 | Alkaloid+NRP+Polyketide:Iterative type I polyketide | 39.0 | 97.0 | 204.0 | 3.49e-63 |
| QIW91874.1 | OXY2 | BGC0002543 | NRP | 37.0 | 98.8 | 192.0 | 1.35e-58 |
| AMM63174.1 | AniF | BGC0001371 | NRP | 35.0 | 98.2 | 190.0 | 7.68e-58 |
| EPE34347.1 | nonheme\_mononuclear\_iron\_oxygenase | BGC0001035 | Polyketide+NRP | 34.0 | 99.1 | 187.0 | 8.23e-57 |
| XP\_020058093.1 | uncharacterized\_protein | BGC0001220 | NRP | 35.0 | 99.1 | 187.0 | 1.19e-56 |
| AMM63173.1 | AniK | BGC0001371 | NRP | 34.0 | 99.4 | 185.0 | 1.01e-55 |
| XP\_002379987.1 | gibberellin\_2-oxidase,\_putative | BGC0001621 | NRP | 34.0 | 104.8 | 184.0 | 3.25e-55 |
| AKJ70940.1 | non-heme\_iron\_a-ketoglutarate\_dependent\_dioxygenase | BGC0002611 | NRP | 34.0 | 98.8 | 180.0 | 7.91e-54 |
| QIW91883.1 | OXY4 | BGC0002543 | NRP | 33.0 | 99.4 | 170.0 | 4.31e-50 |
| AKJ70947.1 | non-heme\_iron\_a-ketoglutarate\_dependent\_dioxygenase | BGC0002611 | NRP | 31.0 | 106.8 | 169.0 | 2.22e-49 |
| AEO57483.1 | 2OG-Fe(II)\_oxygenase-like\_protein | BGC0001449 | NRP+Alkaloid+Polyketide:Iterative type I polyketide | 35.0 | 100.0 | 166.0 | 1.39e-48 |
| EPE34350.1 | nonheme\_mononuclear\_iron\_oxygenase | BGC0001035 | Polyketide+NRP | 33.0 | 98.5 | 165.0 | 3.58e-48 |
| XP\_020058105.1 | uncharacterized\_protein | BGC0001220 | NRP | 33.0 | 100.6 | 164.0 | 1.5e-47 |
| AFU82618.1 | 2-oxoglutarate\_dependent\_oxygenase | BGC0000998 | NRP+Polyketide | 34.0 | 64.6 | 130.0 | 1.08e-35 |
| KIA75589.1 | oxidoreductase | BGC0002209 | Polyketide | 32.0 | 104.8 | 124.0 | 1.28e-32 |
| ALI92653.1 | CitB\_Fe(II)-dependent\_oxygenase | BGC0001338 | Polyketide:Iterative type I polyketide | 30.0 | 102.1 | 123.0 | 3.61e-32 |
| AYV61422.1 | isopenicillin\_N\_synthase\_family\_oxygenase | BGC0001965 | Other | 31.0 | 89.6 | 122.0 | 8.76e-32 |
| AYV61421.1 | isopenicillin\_N\_synthase\_family\_oxygenase | BGC0001965 | Other | 26.0 | 99.4 | 110.0 | 1.37e-27 |
| XP\_002379992.1 | oligopeptide\_transporter,\_putative | BGC0001621 | NRP | 35.0 | 57.4 | 110.0 | 1.01e-26 |
| QBK15042.1 | clavatol\_oxidase\_ClaD | BGC0002196 | Polyketide | 30.0 | 105.1 | 103.0 | 9.67e-25 |
| XP\_006352055.1 | 1-aminocyclopropane-1-carboxylate\_oxidase\_homolog | BGC0002722 | Alkaloid+Terpene+Saccharide | 26.0 | 90.8 | 92.0 | 1.74e-20 |
| QBK15056.1 | FeII/2-oxoglutarate-dependent\_oxygenase\_TraH | BGC0002197 | Polyketide+NRP | 26.0 | 96.4 | 89.0 | 1.05e-19 |
| XP\_006351987.1 | 1-aminocyclopropane-1-carboxylate\_oxidase\_homolog | BGC0002722 | Alkaloid+Terpene+Saccharide | 26.0 | 89.9 | 88.0 | 2.89e-19 |
