# Supplementary material for: Uncovering the transcriptional landscape of Fomes fomentarius during fungal-based material production through gene co-expression network analysis
Source: Fungal Biol Biotechnol. 2025 Feb 13;12:1. doi: 10.1186/s40694-024-00192-3 (PMC11827164; doi:10.1186/s40694-024-00192-3)
Supplement: Supplementary file 1 — Supplementary Material 1 [file 40694_2024_192_MOESM1_ESM.zip › knownclusterblast/region2/jgi.p_Fomfom1_1457457_mibig_hits.html]

| MIBiG Protein | Description | MIBiG Cluster | MiBiG Product | % ID | % Coverage | BLAST Score | E-value |
| --- | --- | --- | --- | --- | --- | --- | --- |
| ACR48329.1 | Nos3 | BGC0000610 | RiPP:Thiopeptide | 61.0 | 66.1 | 142.0 | 5.9e-44 |
| QVN25642.1 | ribosomal\_protein | BGC0002363 | RiPP | 61.0 | 66.1 | 142.0 | 5.9e-44 |
| ADO67783.1 | tpa8 | BGC0000615 | RiPP:Thiopeptide | 59.0 | 66.1 | 135.0 | 2.23e-41 |
