# Supplementary material for: Uncovering the transcriptional landscape of Fomes fomentarius during fungal-based material production through gene co-expression network analysis
Source: Fungal Biol Biotechnol. 2025 Feb 13;12:1. doi: 10.1186/s40694-024-00192-3 (PMC11827164; doi:10.1186/s40694-024-00192-3)
Supplement: Supplementary file 1 — Supplementary Material 1 [file 40694_2024_192_MOESM1_ESM.zip › knownclusterblast/region2/jgi.p_Fomfom1_1287664_mibig_hits.html]

| MIBiG Protein | Description | MIBiG Cluster | MiBiG Product | % ID | % Coverage | BLAST Score | E-value |
| --- | --- | --- | --- | --- | --- | --- | --- |
| EFY99277.1 | long-chain-fatty-acid--CoA\_ligase | BGC0002710 | NRP | 39.0 | 96.1 | 400.0 | 5.24e-132 |
| AAF08801.1 | YngI | BGC0001103 | NRP+Polyketide | 40.0 | 83.5 | 383.0 | 8.69e-126 |
| AEW31026.1 | short-chain\_acyl-CoA\_synthetase | BGC0000407 | NRP | 39.0 | 83.7 | 380.0 | 9.65e-125 |
| ABS74201.1 | YngI | BGC0001095 | NRP | 39.0 | 83.1 | 372.0 | 1.7e-121 |
| KGO40476.1 | AMP-dependent\_synthetase/ligase | BGC0001205 | Polyketide | 43.0 | 55.7 | 286.0 | 4.38e-89 |
| CAH10130.1 | feruloyl-CoA\_synthetase | BGC0000268 | Polyketide | 31.0 | 80.7 | 202.0 | 2.73e-57 |
| WP\_010639243.1 | AMP-binding\_protein | BGC0000958 | NRP:Cyclic depsipeptide+Polyketide:Modular type I polyketide | 31.0 | 82.6 | 189.0 | 1.07e-52 |
| AGM05529.1 | long-chain\_acyl-CoA\_synthetase | BGC0002098 | Polyketide | 31.0 | 86.3 | 186.0 | 1.5e-51 |
| ADI58647.1 | 5-Aminolevulinate\_CoA\_ligase | BGC0000187 | Polyketide:Type II polyketide | 31.0 | 80.6 | 182.0 | 3.02e-50 |
| AKA54630.1 | AMP\_dependent\_CoA-ligase | BGC0001216 | NRP+Polyketide | 35.0 | 58.9 | 180.0 | 4.67e-50 |
| AFV52199.1 | acyl-ACP\_synthetase | BGC0000081 | NRP+Polyketide:Iterative type I polyketide+Polyketide:Enediyne type I polyketide | 34.0 | 56.4 | 181.0 | 9.24e-49 |
| QQZ01621.1 | long-chain\_fatty\_acid--CoA\_ligase | BGC0002497 | Other | 29.0 | 81.1 | 173.0 | 8.1e-47 |
| AIL50189.1 | putative\_5-aminolevulinate\_CoA-ligase | BGC0000213 | Polyketide:Type II polyketide | 29.0 | 82.8 | 172.0 | 2.88e-46 |
| ADC79613.1 | BafX | BGC0000028 | Polyketide:Modular type I polyketide | 34.0 | 57.8 | 169.0 | 2.46e-45 |
| ABU70356.1 | hypothetical\_protein | BGC0001890 | NRP | 28.0 | 81.2 | 169.0 | 5.26e-45 |
| AYU66239.1 | TjhA5 | BGC0002461 | Polyketide | 29.0 | 82.9 | 168.0 | 8.27e-45 |
| BBA21084.1 | putative\_5-aminolevulinate\_CoA-ligase | BGC0001740 | NRP+Polyketide | 28.0 | 82.6 | 166.0 | 2.24e-44 |
| ABB86408.1 | GelA | BGC0000067 | Polyketide | 29.0 | 89.4 | 167.0 | 1.22e-42 |
| AAO06916.1 | GdmAI | BGC0000066 | Polyketide | 29.0 | 89.4 | 166.0 | 1.63e-42 |
| AGY30675.1 | Ann3 | BGC0001298 | Polyketide | 30.0 | 81.4 | 160.0 | 4.93e-42 |
| AAY28225.1 | HbmAI | BGC0000074 | Polyketide | 29.0 | 89.4 | 165.0 | 5.2e-42 |
| AWH12671.1 | RmpA1 | BGC0001759 | Polyketide | 30.0 | 85.9 | 163.0 | 1.5e-41 |
| AAC01710.1 | RifA | BGC0000136 | Polyketide | 29.0 | 89.8 | 163.0 | 1.6e-41 |
| AAX98210.1 | acyl\_CoA\_ligase | BGC0000052 | Polyketide | 29.0 | 81.4 | 158.0 | 2.1e-41 |
| QKW94281.1 | long-chain-fatty-acid--CoA\_ligase | BGC0002342 | NRP+Polyketide | 27.0 | 82.6 | 158.0 | 2.85e-41 |
| CQR60497.1 | Polyketide\_synthase,\_type\_I,\_modules:\_loading,\_1,\_2\_and\_3 | BGC0001287 | Polyketide | 30.0 | 89.0 | 162.0 | 5.22e-41 |
| ABC34346.1 | acyl-CoA\_ligase | BGC0001102 | NRP+Polyketide:Modular type I polyketide+Polyketide:Trans-AT type I polyketide | 28.0 | 82.9 | 157.0 | 1.73e-40 |
| AHH25585.1 | AMP-dependent\_synthetase\_and\_ligase | BGC0000957 | NRP+Polyketide | 28.0 | 82.5 | 159.0 | 2.01e-40 |
| ABV97151.1 | AMP-dependent\_synthetase\_and\_ligase | BGC0000137 | Polyketide | 30.0 | 82.9 | 159.0 | 3.94e-40 |
| ACR50792.1 | putative\_acyl-CoA\_synthase | BGC0000163 | Polyketide | 28.0 | 82.6 | 154.0 | 4.24e-40 |
| CAO85890.1 | aryl-coA\_ligase | BGC0000110 | Polyketide:Modular type I polyketide | 32.0 | 56.2 | 155.0 | 7.45e-40 |
| WP\_003060229.1 | type\_I\_polyketide\_synthase | BGC0002009 | Polyketide | 29.0 | 84.9 | 158.0 | 9.31e-40 |
| ATY46587.1 | polyketide\_synthase | BGC0001666 | Polyketide | 30.0 | 85.6 | 157.0 | 1.67e-39 |
| AAF81723.1 | putative\_acyl-CoA\_ligase\_EncH | BGC0000220 | Polyketide:Type II polyketide | 27.0 | 89.6 | 151.0 | 8.36e-39 |
| ABF88003.1 | putative\_long-chain-fatty-acid\_CoA\_ligase | BGC0000871 | Other:Nucleoside | 26.0 | 89.8 | 154.0 | 9.19e-39 |
| ADI58632.1 | 5-Enolpyruvylshikimate-3-phosphate\_synthase/CHC-CoA\_ligase | BGC0000187 | Polyketide:Type II polyketide | 30.0 | 84.6 | 152.0 | 5.12e-38 |
| ABP55493.1 | thioester\_reductase\_domain | BGC0001006 | NRP+Polyketide | 30.0 | 86.3 | 151.0 | 1.48e-37 |
| ACF35445.1 | mbcAI | BGC0000090 | Polyketide | 29.0 | 85.7 | 151.0 | 1.74e-37 |
| BCJ07564.1 | AMP-dependent\_synthetase | BGC0002379 | NRP | 28.0 | 86.6 | 147.0 | 1.82e-37 |
| CAK50779.1 | acyl\_CoA\_ligase | BGC0000247 | Polyketide:Type II polyketide+Saccharide:Oligosaccharide | 28.0 | 73.9 | 147.0 | 1.84e-37 |
| QIC03945.1 | EncH | BGC0002366 | Polyketide | 26.0 | 89.6 | 147.0 | 2.42e-37 |
| QGJ79675.1 | Polyketide\_synthase | BGC0002552 | Polyketide | 29.0 | 84.8 | 149.0 | 5.27e-37 |
| AAQ84158.1 | PlmJK | BGC0000123 | Polyketide | 29.0 | 80.6 | 149.0 | 5.27e-37 |
| CAE17553.1 | acyl\_CoA\_ligase | BGC0000210 | Polyketide:Type II polyketide+Saccharide:Oligosaccharide | 26.0 | 85.7 | 145.0 | 5.53e-37 |
| QES95474.1 | type\_I\_polyketide\_synthase | BGC0002453 | Polyketide | 29.0 | 85.6 | 149.0 | 7.2e-37 |
| ADM46356.1 | polyketide\_synthase | BGC0000106 | Polyketide | 29.0 | 82.6 | 149.0 | 7.24e-37 |
| WP\_157358234.1 | SDR\_family\_NAD(P)-dependent\_oxidoreductase | BGC0002011 | Polyketide | 30.0 | 84.8 | 149.0 | 9.36e-37 |
| CAI94705.1 | putative\_acid\_AMP\_ligase | BGC0000141 | Polyketide | 28.0 | 82.5 | 144.0 | 1.94e-36 |
| QBF51769.1 | type\_I\_polyketide\_synthase | BGC0001856 | Polyketide:Modular type I polyketide | 30.0 | 82.3 | 147.0 | 2.64e-36 |
| CCA65703.1 | anthranilate-CoA\_ligase | BGC0001343 | Polyketide | 25.0 | 83.5 | 142.0 | 5.44e-36 |
| CAJ88630.1 | putative\_acyl-CoA\_synthetase | BGC0000327 | NRP | 32.0 | 56.1 | 141.0 | 1.64e-35 |
| CAI94682.1 | putative\_polyketide\_synthase | BGC0000141 | Polyketide | 29.0 | 83.4 | 145.0 | 1.69e-35 |
| AFV30247.1 | polyketide\_synthase | BGC0000075 | Polyketide | 29.0 | 86.2 | 144.0 | 2.26e-35 |
| TXD00261.1 | AMP-binding\_protein | BGC0001877 | Polyketide | 29.0 | 82.5 | 144.0 | 3.49e-35 |
| CAA60460.1 | polyketide\_synthase | BGC0001040 | NRP+Polyketide | 29.0 | 85.2 | 143.0 | 5.53e-35 |
| ABV99085.1 | thioester\_reductase\_domain | BGC0001007 | Polyketide+NRP | 29.0 | 84.8 | 143.0 | 6.19e-35 |
| QTT72113.1 | type\_I\_polyketide\_synthase | BGC0002350 | NRP+Polyketide+Saccharide | 29.0 | 83.4 | 142.0 | 1.13e-34 |
| AFU65902.1 | DacH | BGC0000216 | Polyketide | 31.0 | 57.6 | 139.0 | 1.28e-34 |
| AAF86393.1 | FkbB | BGC0000994 | NRP+Polyketide | 29.0 | 84.6 | 141.0 | 3.05e-34 |
| AAC68815.1 | FK506\_polyketide\_synthase | BGC0000353 | NRP | 30.0 | 85.2 | 141.0 | 3.05e-34 |
| ACB12556.1 | Fum10 | BGC0000063 | Polyketide | 28.0 | 88.2 | 137.0 | 8.23e-34 |
| QTC09983.1 | benzoate-CoA\_ligase | BGC0002372 | Polyketide+Terpene+Alkaloid | 29.0 | 58.5 | 136.0 | 1.28e-33 |
| ATY12791.1 | long-chain\_fatty\_acid--CoA\_ligase | BGC0001504 | Polyketide | 31.0 | 61.0 | 135.0 | 1.35e-33 |
| AXM42922.1 | AMP-dependent\_synthetase\_and\_ligase/heterocyclase | BGC0001940 | Polyketide | 25.0 | 92.2 | 136.0 | 2.75e-33 |
| AAN74813.2 | Fum10p | BGC0000062 | Polyketide | 28.0 | 86.0 | 135.0 | 3.36e-33 |
| CAQ52626.1 | type\_I\_polyketide\_synthase,\_loading\_module\_and\_modules\_1-3 | BGC0001066 | Polyketide:Modular type I polyketide | 28.0 | 84.2 | 137.0 | 3.92e-33 |
| AEA35021.1 | hypothetical\_protein | BGC0002502 | Polyketide | 28.0 | 86.6 | 134.0 | 4.92e-33 |
| AHD25943.1 | putative\_acyl-CoA\_ligase/oxygenase\_fusion\_protein | BGC0000208 | Polyketide | 27.0 | 86.2 | 135.0 | 5.04e-33 |
| WP\_015031692.1 | type\_I\_polyketide\_synthase | BGC0001819 | Polyketide | 29.0 | 84.3 | 136.0 | 7.83e-33 |
| CAJ34366.1 | putative\_3-hydroxy-quinaldate-AMP-Ligase | BGC0000445 | NRP:Cyclic depsipeptide | 28.0 | 82.9 | 133.0 | 1.11e-32 |
| DAC74137.1 | AMP-dependent\_synthetase | BGC0002019 | Terpene | 30.0 | 57.0 | 132.0 | 1.55e-32 |
| BAO66547.1 | cyclohexanecarboxylate-CoA\_ligase | BGC0000042 | Polyketide | 31.0 | 56.5 | 132.0 | 1.63e-32 |
| AFV52184.1 | acyl-CoA\_synthetase/P450\_monooxygenase | BGC0000081 | NRP+Polyketide:Iterative type I polyketide+Polyketide:Enediyne type I polyketide | 32.0 | 57.8 | 134.0 | 2.22e-32 |
| ACN64850.1 | PokL | BGC0001061 | Polyketide:Iterative type I polyketide+Polyketide:Type II polyketide+Saccharide:Hybrid/tailoring saccharide | 28.0 | 63.8 | 131.0 | 2.69e-32 |
| APZ78718.1 | benzoate-CoA\_ligase\_family\_protein | BGC0001420 | NRP:Cyclic depsipeptide+Polyketide:Iterative type I polyketide | 25.0 | 83.7 | 130.0 | 6.2e-32 |
| CAC17498.1 | putative\_AMP-binding\_ligase | BGC0000324 | NRP | 28.0 | 84.9 | 130.0 | 8.9e-32 |
| ASZ00147.1 | polyketide\_synthase | BGC0001785 | Polyketide | 32.0 | 59.0 | 133.0 | 1.18e-31 |
| AEI98652.1 | CtcI | BGC0000209 | Polyketide | 31.0 | 57.9 | 129.0 | 2.07e-31 |
| AKD43499.1 | Acyl-CoA\_ligase | BGC0001409 | Polyketide | 29.0 | 62.4 | 127.0 | 7.25e-31 |
| ACS20362.1 | amino\_acid\_adenylation\_domain\_protein | BGC0002420 | NRP+Polyketide | 27.0 | 88.2 | 130.0 | 9.14e-31 |
| QIE07123.1 | OvmK1 | BGC0001719 | Polyketide | 26.0 | 86.0 | 128.0 | 1.81e-30 |
| OWA01623.1 | acyl--CoA\_ligase | BGC0001439 | Polyketide+Saccharide:Hybrid/tailoring saccharide | 30.0 | 61.0 | 125.0 | 6.66e-30 |
| AGN74880.1 | 3-hydroxypicolinic\_acid:AMP\_ligase | BGC0000459 | NRP:Cyclic depsipeptide+Polyketide:Trans-AT type I polyketide | 27.0 | 84.2 | 124.0 | 8.93e-30 |
| ABL70475.1 | 2,3-dihydroxybenzoate-AMP\_ligase | BGC0002493 | NRP | 31.0 | 56.7 | 124.0 | 1.2e-29 |
| CCP20047.1 | divK\_protein | BGC0001119 | Polyketide:Modular type I polyketide | 26.0 | 86.0 | 126.0 | 1.44e-29 |
| BAI63284.1 | putative\_peptide\_arylation\_enzyme | BGC0000434 | NRP | 28.0 | 82.8 | 123.0 | 2.61e-29 |
| WP\_069848012.1 | salicylate\_synthase | BGC0002472 | NRP | 25.0 | 83.4 | 124.0 | 4.57e-29 |
| ABY66018.1 | CoA\_ligase | BGC0001008 | Polyketide:Iterative type I polyketide+Polyketide:Enediyne type I polyketide | 28.0 | 81.7 | 122.0 | 5.54e-29 |
| UHY14129.1 | PKS\_I | BGC0002671 | Polyketide | 27.0 | 64.0 | 124.0 | 5.82e-29 |
| ALE27504.1 | 3-HPA:AMP\_ligase | BGC0001292 | Other | 27.0 | 89.1 | 122.0 | 6.01e-29 |
| QQZ01622.1 | amide\_synthase | BGC0002497 | Other | 31.0 | 55.9 | 121.0 | 7.42e-29 |
| ABX37385.1 | amino\_acid\_adenylation\_domain\_protein | BGC0000984 | NRP+Polyketide | 26.0 | 88.4 | 124.0 | 8.46e-29 |
| BAR73008.1 | putative\_ATP-dependent\_b-aminoacyl-ACP\_synthetase | BGC0001194 | Polyketide | 29.0 | 57.3 | 121.0 | 1.18e-28 |
| AIS24844.1 | dst22 | BGC0001147 | NRP | 30.0 | 56.2 | 120.0 | 1.46e-28 |
| ARO38317.1 | nonribosomal\_peptide\_synthetase | BGC0001560 | NRP+Polyketide | 27.0 | 58.9 | 122.0 | 4.32e-28 |
| AFP87519.1 | proline\_adenyltransferase | BGC0001159 | NRP+Polyketide:Modular type I polyketide | 27.0 | 85.2 | 119.0 | 5.52e-28 |
| ACG60755.1 | acyl-CoA\_synthetase | BGC0001058 | NRP:Glycopeptide+Polyketide:Modular type I polyketide+Saccharide:Hybrid/tailoring saccharide | 30.0 | 58.1 | 119.0 | 6.79e-28 |
| BAC16758.1 | probable\_2,3-dihydroxybenzoate-AMP\_ligase\_protein | BGC0002474 | NRP | 24.0 | 82.8 | 119.0 | 7.27e-28 |
| ADJ63842.1 | Serobactin\_synthetase | BGC0000424 | NRP:NRP siderophore | 28.0 | 62.0 | 120.0 | 1.03e-27 |
| AGU50953.1 | putative\_non-ribosomal\_peptide\_synthetase | BGC0002417 | NRP+Polyketide | 27.0 | 88.2 | 119.0 | 1.87e-27 |
| PKY07881.1 | hypothetical\_protein | BGC0001544 | NRP+Polyketide | 27.0 | 84.3 | 119.0 | 2.1e-27 |
| QJD55556.1 | acetyl-CoA\_ligase | BGC0001909 | Polyketide | 25.0 | 91.5 | 117.0 | 2.84e-27 |
| ABM34280.1 | amino\_acid\_adenylation\_domain\_protein | BGC0002419 | NRP+Polyketide | 26.0 | 89.1 | 117.0 | 7.63e-27 |
| ALK21567.1 | 2,3-dihydroxybenzoate-AMP\_ligase | BGC0002678 | NRP | 27.0 | 82.3 | 115.0 | 7.93e-27 |
| BAB69698.1 | iturin\_A\_synthetase\_A | BGC0001098 | NRP+Polyketide | 27.0 | 62.1 | 116.0 | 2.1e-26 |
| AAY93445.1 | non-ribosomal\_peptide\_synthetase\_PvdL | BGC0000413 | NRP | 27.0 | 86.8 | 116.0 | 2.12e-26 |
| WP\_051729283.1 | AMP-binding\_protein | BGC0002137 | Polyketide | 30.0 | 49.1 | 114.0 | 3.57e-26 |
| ABS74181.1 | bacillomycin\_D\_synthetase\_A\_ | BGC0001090 | Polyketide+NRP:Lipopeptide | 26.0 | 62.0 | 115.0 | 3.67e-26 |
| ATG32071.1 | proline\_specific\_adenylation\_domain-containing\_protein | BGC0001750 | NRP+Polyketide | 26.0 | 84.2 | 113.0 | 4.29e-26 |
| AFY58523.1 | amino\_acid\_adenylation\_enzyme/thioester\_reductase\_family\_protein | BGC0002411 | NRP+Polyketide | 26.0 | 60.6 | 114.0 | 1.12e-25 |
| ALA09365.1 | AMP-dependent\_synthetase\_and\_ligase | BGC0001303 | Polyketide | 27.0 | 57.0 | 111.0 | 1.55e-25 |
| ADZ24989.1 | prolin\_adenylation\_protein | BGC0000380 | NRP+Polyketide:Modular type I polyketide | 23.0 | 83.1 | 110.0 | 5.05e-25 |
| UKO95761.1 | amino\_acid\_adenylation\_domain-containing\_protein | BGC0002632 | NRP | 23.0 | 83.1 | 110.0 | 5.71e-25 |
| BAC87906.1 | probable\_acinetobactin\_biosynthesis\_protein | BGC0000294 | NRP | 26.0 | 83.4 | 110.0 | 5.74e-25 |
| AAO07763.1 | 2,3-dihydroxybenzoate-AMP\_ligase | BGC0000460 | NRP | 25.0 | 83.5 | 110.0 | 5.74e-25 |
| ESP90847.1 | amino\_acid\_adenylation\_domain\_protein | BGC0000891 | Other:Aminocoumarin | 23.0 | 84.5 | 108.0 | 1.17e-24 |
| ADZ13540.1 | YtkG | BGC0000466 | NRP | 26.0 | 85.9 | 108.0 | 1.37e-24 |
| AAG04385.1 | probable\_coenzyme\_A\_ligase | BGC0000922 | Other | 26.0 | 68.3 | 108.0 | 1.55e-24 |
| QRN75754.1 | Polyketide\_synthase | BGC0002114 | NRP+Polyketide | 27.0 | 58.2 | 110.0 | 1.8e-24 |
| WP\_039806856.1 | non-ribosomal\_peptide\_synthetase | BGC0002001 | NRP+Polyketide | 28.0 | 62.1 | 109.0 | 2.71e-24 |
| QXJ21811.1 | amino\_acid\_adenylation\_domain-containing\_protein | BGC0002370 | NRP | 28.0 | 58.7 | 107.0 | 2.78e-24 |
| EGJ35087.1 | Fatty-acyl-ACP\_ligase | BGC0001163 | Polyketide:Modular type I polyketide | 25.0 | 94.7 | 108.0 | 2.99e-24 |
| ACU71632.1 | amide\_synthetase | BGC0001154 | Other | 28.0 | 56.8 | 107.0 | 3.27e-24 |
| NAO96316.1 | AMP-binding\_protein | BGC0002117 | NRP | 27.0 | 67.7 | 108.0 | 4.52e-24 |
| MBN3579117.1 | AMP-binding\_protein | BGC0002613 | NRP+Polyketide | 28.0 | 56.1 | 106.0 | 7.34e-24 |
| AAQ59159.1 | 2,3-dihydroxybenzoate-AMP\_ligase | BGC0002679 | NRP | 27.0 | 83.9 | 106.0 | 7.34e-24 |
| DAB41914.1 | ArzL\_-\_PKS\_(FAAL,\_ACP) | BGC0001884 | NRP+Polyketide | 27.0 | 65.2 | 107.0 | 8.59e-24 |
| AAF67505.1 | amide\_synthetase | BGC0000834 | Saccharide:Hybrid/tailoring saccharide+Other:Aminocoumarin | 25.0 | 83.5 | 106.0 | 9.33e-24 |
| ACN69986.1 | proline\_adenyltransferase | BGC0000079 | Polyketide | 27.0 | 84.0 | 105.0 | 1.01e-23 |
| CAG23957.2 | hybrid\_NRPS/PKS\_protein | BGC0001089 | Polyketide+NRP | 25.0 | 84.6 | 107.0 | 1.32e-23 |
| AEH42484.1 | adenylation\_for\_L-proline | BGC0000032 | Polyketide | 27.0 | 84.0 | 105.0 | 1.78e-23 |
| AHH34188.1 | fatty\_acyl\_ACP\_ligase | BGC0001162 | Polyketide:Modular type I polyketide | 26.0 | 67.5 | 106.0 | 2.14e-23 |
| RAT94090.1 | NRPS | BGC0001469 | NRP | 27.0 | 58.9 | 107.0 | 2.33e-23 |
| AMB48444.1 | long-chain-fatty-acid--CoA\_ligase | BGC0001357 | Polyketide | 23.0 | 95.8 | 105.0 | 2.38e-23 |
| ABP55217.1 | AMP-dependent\_synthetase\_and\_ligase | BGC0000142 | Polyketide | 24.0 | 83.4 | 105.0 | 2.44e-23 |
| AEF33098.1 | 3-hydroxypicolinic\_acid:AMP\_ligase | BGC0001039 | NRP+Polyketide | 26.0 | 82.8 | 104.0 | 3.22e-23 |
| ADN13832.1 | Polyketide\_Synthase | BGC0001164 | Polyketide:Modular type I polyketide | 26.0 | 66.0 | 106.0 | 3.71e-23 |
| AZZ09613.1 | PvhA | BGC0002304 | Polyketide+NRP | 25.0 | 89.1 | 106.0 | 3.91e-23 |
| AHF22853.1 | MarM | BGC0000091 | Polyketide | 25.0 | 85.4 | 104.0 | 4.02e-23 |
| QST87270.1 | amino\_acid\_adenylation\_domain-containing\_protein | BGC0002572 | NRP+Polyketide | 26.0 | 58.5 | 106.0 | 4.13e-23 |
| ABS75100.1 | AMP-binding\_protein | BGC0002641 | NRP | 27.0 | 66.1 | 104.0 | 4.42e-23 |
| AFY58527.1 | acyl-CoA\_synthetase\_(AMP-forming)/AMP-acid\_ligase\_II | BGC0002411 | NRP+Polyketide | 27.0 | 61.8 | 104.0 | 5.8e-23 |
| ABI22131.1 | putative\_non-ribosomal\_peptide\_synthetase | BGC0000422 | NRP | 27.0 | 63.2 | 105.0 | 5.88e-23 |
| AAO23333.1 | NcpA | BGC0000397 | NRP | 25.0 | 58.9 | 104.0 | 1.55e-22 |
| AEU11006.1 | NpnB | BGC0001029 | NRP+Polyketide | 25.0 | 57.0 | 104.0 | 1.59e-22 |
| ACO94489.1 | putative\_AMP-dependent\_acyl-CoA\_synthetase/ligase | BGC0000097 | Polyketide:Modular type I polyketide | 26.0 | 85.2 | 102.0 | 1.69e-22 |
| ACY02013.1 | enterobactin\_synthase\_subunit\_E | BGC0000343 | NRP | 26.0 | 83.1 | 102.0 | 1.77e-22 |
| AGS77309.1 | NRPS\_modules\_4-6 | BGC0001178 | NRP:Glycopeptide | 27.0 | 84.0 | 103.0 | 2.07e-22 |
| AIU36103.1 | LglD | BGC0000180 | Polyketide:Trans-AT type I polyketide | 26.0 | 70.7 | 103.0 | 2.11e-22 |
| extra\_gene | NRPS/PKS | BGC0002095 | NRP | 28.0 | 60.4 | 103.0 | 2.53e-22 |
| AEC14346.1 | nonribosomal\_peptide\_synthetase | BGC0000377 | NRP | 24.0 | 59.2 | 103.0 | 2.56e-22 |
| ATY37609.1 | BreD | BGC0001536 | NRP | 27.0 | 60.9 | 103.0 | 2.65e-22 |
| ACR11042.1 | Enterobactin\_synthetase\_component\_E/2,3-dihydroxybenzoate-AMP\_ligase | BGC0000451 | NRP | 24.0 | 69.3 | 101.0 | 3.09e-22 |
| AXM43064.1 | AMP-dependent\_synthetase\_and\_ligase | BGC0001945 | NRP | 26.0 | 81.7 | 102.0 | 3.13e-22 |
| ATY37589.1 | BogB | BGC0001532 | NRP | 26.0 | 61.8 | 103.0 | 3.36e-22 |
| BBA21073.1 | putative\_non-ribosomal\_peptide\_synthetase | BGC0001740 | NRP+Polyketide | 32.0 | 46.1 | 103.0 | 3.55e-22 |
| AHA34037.1 | Bmp4,\_prolyl\_adenylation\_domain | BGC0000890 | Other:Aminocoumarin | 23.0 | 83.5 | 101.0 | 3.64e-22 |
| ACA99172.1 | polyketide\_synthase | BGC0001160 | Polyketide:Modular type I polyketide | 26.0 | 66.0 | 102.0 | 4.49e-22 |
| AHD05621.1 | non-ribosomal\_peptide\_ligase\_domain\_protein | BGC0001033 | NRP+Polyketide | 21.0 | 83.9 | 102.0 | 4.5e-22 |
| AAN65228.1 | amide\_synthetase | BGC0000832 | Saccharide:Hybrid/tailoring saccharide+Other:Aminocoumarin | 25.0 | 84.5 | 100.0 | 5.18e-22 |
| WP\_026723805.1 | AMP-dependent\_synthetase | BGC0001467 | NRP:Cyclic depsipeptide+Polyketide:Modular type I polyketide | 26.0 | 62.6 | 101.0 | 5.24e-22 |
| AFV96135.1 | AMP-dependent\_synthetase/ligase | BGC0001064 | Polyketide:Modular type I polyketide+Polyketide:Type III polyketide | 23.0 | 95.7 | 101.0 | 5.4e-22 |
| ARU81115.1 | CylA | BGC0001566 | Polyketide | 23.0 | 95.7 | 101.0 | 5.4e-22 |
| AAN65233.1 | acyl-CoA\_synthetase | BGC0000832 | Saccharide:Hybrid/tailoring saccharide+Other:Aminocoumarin | 25.0 | 84.9 | 100.0 | 7.91e-22 |
| AEA30274.1 | peptide\_synthetase | BGC0000429 | Polyketide+NRP:Cyclic depsipeptide | 29.0 | 56.1 | 102.0 | 8.24e-22 |
| AGN74885.1 | nonribosomal\_peptide\_synthetase | BGC0000459 | NRP:Cyclic depsipeptide+Polyketide:Trans-AT type I polyketide | 25.0 | 96.6 | 102.0 | 8.54e-22 |
| ALV86864.1 | Tlo18 | BGC0001406 | NRP | 26.0 | 62.7 | 100.0 | 8.78e-22 |
| QDJ74293.1 | peptide\_synthetase | BGC0002109 | NRP | 28.0 | 60.4 | 101.0 | 9.02e-22 |
| BCK51628.1 | non-ribosomal\_peptide\_synthetase | BGC0002520 | Polyketide | 27.0 | 56.1 | 101.0 | 9.02e-22 |
| AAG29784.2 | amide\_synthetase | BGC0000833 | Saccharide:Hybrid/tailoring saccharide+Other:Aminocoumarin | 27.0 | 60.9 | 100.0 | 9.27e-22 |
| ALG65336.1 | Var3 | BGC0002416 | NRP+Polyketide | 27.0 | 61.5 | 101.0 | 9.32e-22 |
| SDF67357.1 | amino\_acid\_adenylation\_domain-containing\_protein | BGC0002422 | NRP | 22.0 | 83.5 | 101.0 | 9.7e-22 |
| WP\_013310341.1 | aminotransferase\_class\_III-fold\_pyridoxal\_phosphate-dependent\_enzyme | BGC0001728 | NRP+Polyketide | 26.0 | 63.8 | 101.0 | 9.72e-22 |
| KGA48878.1 | (2,3-dihydroxybenzoyl)adenylate\_synthase | BGC0002413 | NRP | 27.0 | 85.6 | 100.0 | 9.97e-22 |
| AAX31555.1 | acyl-CoA\_ligase | BGC0000336 | NRP | 27.0 | 61.8 | 100.0 | 1.23e-21 |
| EEP98516.1 | Peptide\_synthetase | BGC0002091 | NRP | 27.0 | 59.9 | 100.0 | 1.36e-21 |
| APZ78768.1 | nonribosomal\_peptide\_synthetase | BGC0001425 | NRP:Cyclic depsipeptide+Polyketide:Iterative type I polyketide | 27.0 | 58.4 | 101.0 | 1.39e-21 |
| AAG29789.1 | acyl-CoA\_synthetase | BGC0000833 | Saccharide:Hybrid/tailoring saccharide+Other:Aminocoumarin | 25.0 | 84.8 | 99.0 | 1.41e-21 |
| QYA95680.1 | amino\_acid\_adenylation\_domain-containing\_protein | BGC0002676 | NRP | 29.0 | 56.1 | 101.0 | 1.43e-21 |
| ABA23460.1 | Amino\_acid\_adenylation | BGC0000427 | NRP | 26.0 | 58.4 | 100.0 | 1.53e-21 |
| BAE93741.1 | putative\_acyltransferase\_family\_protein | BGC0000164 | Polyketide | 26.0 | 87.0 | 100.0 | 1.56e-21 |
| ABB69752.1 | PlaP4 | BGC0000654 | Terpene+Saccharide:Hybrid/tailoring saccharide | 26.0 | 84.3 | 100.0 | 1.61e-21 |
| AXG47007.1 | non-ribosomal\_peptide\_synthetase | BGC0000383 | NRP+Polyketide:Modular type I polyketide | 24.0 | 60.9 | 100.0 | 1.74e-21 |
| CAJ34370.1 | NRPS\_protein | BGC0000445 | NRP:Cyclic depsipeptide | 28.0 | 61.6 | 100.0 | 1.77e-21 |
| AHD05679.1 | putative\_non-ribosomal\_peptide\_ligase/\_polyketide\_synthase\_hybrid | BGC0000402 | NRP | 26.0 | 62.0 | 100.0 | 1.89e-21 |
| APZ78794.1 | nonribosomal\_peptide\_synthetase | BGC0001427 | NRP:Cyclic depsipeptide+Polyketide:Iterative type I polyketide | 27.0 | 57.0 | 100.0 | 2.41e-21 |
| AXA20090.1 | hybrid\_trans-AT\_PKS/NRPS\_LgaA | BGC0001646 | NRP+Polyketide | 26.0 | 84.5 | 100.0 | 2.46e-21 |
| AQZ26587.1 | obafluorin\_dimodular\_nonribosomal\_peptide\_synthetase | BGC0001437 | NRP | 25.0 | 61.3 | 100.0 | 2.9e-21 |
| QKW94305.1 | AMP-dependent\_synthetase/ligase | BGC0002342 | NRP+Polyketide | 29.0 | 64.1 | 100.0 | 3.26e-21 |
| CCA29203.1 | non-ribosomal\_peptide\_synthetase/polyketide\_synthase | BGC0000955 | NRP+Polyketide:Modular type I polyketide | 26.0 | 59.3 | 100.0 | 3.39e-21 |
| ACU71638.1 | peptide\_synthetase-like\_protein | BGC0001154 | Other | 24.0 | 85.4 | 99.0 | 3.73e-21 |
| MBE8994627.1 | fatty\_acyl-AMP\_ligase | BGC0002623 | NRP+Polyketide | 25.0 | 64.3 | 98.0 | 4.77e-21 |
| SAI82901.1 | HrnJ;\_Putative\_AMP-dependent\_acyl-CoA\_synthetase/ligase;\_AMP-binding\_enzyme;\_Pfam00501 | BGC0002101 | Polyketide | 25.0 | 85.4 | 98.0 | 5.21e-21 |
| CCA53797.1 | Long-chain-fatty-acid--CoA\_ligase | BGC0001801 | NRP | 26.0 | 82.9 | 98.0 | 5.23e-21 |
| AQX77690.1 | NocL | BGC0001704 | Other | 23.0 | 91.9 | 98.0 | 5.67e-21 |
| ACO78745.1 | Non-ribosomal\_peptide\_synthase:Amino\_acid\_adenylation | BGC0002433 | NRP | 25.0 | 95.7 | 99.0 | 5.78e-21 |
| QHZ99336.1 | proline\_adenyltransferase | BGC0001875 | Polyketide | 28.0 | 57.1 | 97.0 | 5.79e-21 |
| EDT06082.1 | AMP-dependent\_synthetase\_and\_ligase | BGC0001897 | Polyketide | 29.0 | 65.7 | 98.0 | 6.71e-21 |
| BAO66533.1 | nonribosomal\_peptide\_synthase | BGC0000042 | Polyketide | 26.0 | 59.5 | 97.0 | 6.76e-21 |
| BAP27942.1 | nonribosomal\_peptide\_synthetase | BGC0001085 | NRP+Terpene | 26.0 | 85.4 | 99.0 | 7.06e-21 |
| AAZ03550.1 | McnA | BGC0000332 | NRP | 25.0 | 57.1 | 99.0 | 7.11e-21 |
| AAF08795.1 | MycA | BGC0001103 | NRP+Polyketide | 25.0 | 67.7 | 99.0 | 7.55e-21 |
| AIG26883.1 | NRPS\_domain-containing\_protein | BGC0002432 | NRP | 26.0 | 60.6 | 99.0 | 7.59e-21 |
| AAQ90177.1 | putative\_acyl-CoA\_synthetase | BGC0000128 | Polyketide | 24.0 | 82.8 | 97.0 | 7.67e-21 |
| AAO62588.1 | peptide\_sythetase | BGC0001016 | NRP+Polyketide | 25.0 | 58.7 | 99.0 | 7.68e-21 |
| CAD17792.1 | probable\_non\_ribosomal\_peptide\_synthetase\_protein | BGC0001363 | NRP+Polyketide | 27.0 | 62.4 | 99.0 | 7.9e-21 |
| CCC55921.1 | non-ribosomal\_peptide\_synthetase/polyketide\_synthase\_hybrid\_protein | BGC0000973 | NRP+Polyketide:Modular type I polyketide | 25.0 | 58.5 | 99.0 | 9.35e-21 |
| AQH32483.1 | hybrid\_peptide\_synthetase/polyketide\_synthase | BGC0001667 | NRP+Polyketide | 27.0 | 86.0 | 99.0 | 9.4e-21 |
| AHA12086.1 | amino\_acid\_adenyltransferase | BGC0001172 | NRP+Polyketide:Modular type I polyketide | 28.0 | 57.1 | 97.0 | 9.62e-21 |
| AKL71645.1 | NocL | BGC0001703 | Other | 26.0 | 66.5 | 97.0 | 9.83e-21 |
| AHH34187.1 | fatty\_acyl\_ACP\_ligase | BGC0001161 | Polyketide:Modular type I polyketide | 26.0 | 69.1 | 98.0 | 1.01e-20 |
| QIE08737.1 | non-ribosomal\_peptide\_synthetase | BGC0002544 | NRP | 27.0 | 58.4 | 98.0 | 1.22e-20 |
| AQA28568.1 | acyl-CoA\_synthase | BGC0001663 | Polyketide | 26.0 | 62.4 | 97.0 | 1.23e-20 |
| QOV09198.1 | ClyA/NocL | BGC0002597 | NRP+Polyketide | 24.0 | 93.0 | 97.0 | 1.33e-20 |
| AWX24482.1 | non-ribosomal\_peptide\_synthetase | BGC0001695 | NRP | 28.0 | 63.0 | 98.0 | 1.33e-20 |
| WP\_018960015.1 | fatty\_acyl-AMP\_ligase | BGC0002010 | NRP+Polyketide | 27.0 | 63.8 | 97.0 | 1.57e-20 |
| AGC09519.1 | AMP-dependent\_synthetase/ligase | BGC0001183 | Polyketide | 26.0 | 57.6 | 97.0 | 1.62e-20 |
| AKJ15827.1 | peptide\_synthetase | BGC0002735 | Polyketide+NRP | 26.0 | 59.2 | 98.0 | 1.63e-20 |
| EOY45602.1 | Adenylation\_and\_reductase\_domains\_containing\_protein | BGC0001168 | NRP | 27.0 | 46.0 | 97.0 | 2.03e-20 |
| BAW32334.1 | hybrid\_cis-AT\_polyketide\_synthase\_-\_nonribosomal\_peptide\_synthetase | BGC0001631 | NRP+Polyketide | 28.0 | 57.6 | 97.0 | 2.29e-20 |
| ABY83163.1 | Azi25 | BGC0000960 | NRP+Polyketide | 27.0 | 61.5 | 97.0 | 2.49e-20 |
| AAZ23074.1 | acyl-CoA\_ligase | BGC0000291 | NRP | 26.0 | 62.9 | 96.0 | 2.75e-20 |
| ALK21569.1 | non-ribosomal\_peptide\_synthetase | BGC0002678 | NRP | 30.0 | 47.4 | 97.0 | 3.24e-20 |
| ABI22132.1 | putative\_non-ribosomal\_peptide\_synthetase | BGC0000422 | NRP | 28.0 | 59.9 | 96.0 | 3.73e-20 |
| MBC5793764.1 | fatty\_acyl-AMP\_ligase | BGC0002480 | Polyketide+NRP | 27.0 | 63.2 | 96.0 | 3.93e-20 |
| CAC48361.1 | peptide\_synthetase | BGC0000311 | NRP | 27.0 | 83.5 | 97.0 | 3.97e-20 |
| SCO70321.1 | AMP-dependent\_synthetase/ligase | BGC0001433 | Polyketide:Modular type I polyketide | 26.0 | 65.5 | 95.0 | 4.26e-20 |
| QCF28941.1 | fatty\_acyl-AMP\_ligase | BGC0002308 | Alkaloid+Polyketide | 26.0 | 64.8 | 95.0 | 4.78e-20 |
| AZF85942.1 | long-chain-fatty-acid-CoA\_ligase | BGC0001963 | NRP+Polyketide | 25.0 | 63.8 | 95.0 | 4.79e-20 |
| AXG22420.1 | proline\_adenyltransferase | BGC0002024 | Polyketide | 26.0 | 83.7 | 95.0 | 4.81e-20 |
| CAA11794.1 | PCZA363.3 | BGC0000322 | NRP | 25.0 | 83.9 | 96.0 | 5.07e-20 |
| WP\_051803379.1 | D-alanine--poly(phosphoribitol)\_ligase | BGC0002381 | Alkaloid+Polyketide | 25.0 | 84.5 | 95.0 | 5.2e-20 |
| ATL73045.1 | amino\_acid\_adenylation\_domain\_protein | BGC0001807 | NRP+Polyketide | 26.0 | 57.6 | 96.0 | 6.48e-20 |
| CZT62794.1 | Non-ribosomal\_peptide\_synthase\_involved\_in\_Hassallidin\_biosynthesis | BGC0001614 | NRP | 27.0 | 59.8 | 96.0 | 6.58e-20 |
| AVI26393.1 | nonribosomal\_peptide\_synthase | BGC0001800 | NRP+Polyketide | 25.0 | 58.2 | 96.0 | 6.89e-20 |
| ADD82940.1 | Bat2 | BGC0001099 | NRP+Polyketide:Modular type I polyketide+Polyketide:Trans-AT type I polyketide | 24.0 | 82.9 | 96.0 | 7.24e-20 |
| sipL1 | AMP-dependent\_synthetase\_and\_ligase | BGC0001452 | Polyketide | 26.0 | 57.3 | 94.0 | 7.5e-20 |
| AXN93598.1 | PuwC | BGC0001952 | NRP | 26.0 | 61.6 | 94.0 | 8.27e-20 |
| AGC24265.1 | prlK | BGC0001038 | NRP+Polyketide:Modular type I polyketide | 28.0 | 58.7 | 94.0 | 8.72e-20 |
| APZ78808.1 | nonribosomal\_peptide\_synthetase | BGC0001428 | NRP:Cyclic depsipeptide+Polyketide:Iterative type I polyketide | 26.0 | 57.0 | 96.0 | 8.77e-20 |
| AEA60663.1 | pyoverdine\_synthetase | BGC0000892 | Other | 27.0 | 62.9 | 94.0 | 8.78e-20 |
| OKA09423.1 | non-ribosomal\_peptide\_synthetase | BGC0001459 | NRP:Glycopeptide | 24.0 | 85.4 | 96.0 | 8.81e-20 |
| ABF87031.1 | non-ribosomal\_peptide\_synthetase/polyketide\_synthase | BGC0000393 | NRP+Polyketide:Modular type I polyketide | 27.0 | 64.3 | 96.0 | 9.75e-20 |
| QYA95657.1 | amino\_acid\_adenylation\_domain-containing\_protein | BGC0002676 | NRP | 27.0 | 56.5 | 94.0 | 1.11e-19 |
| AIS24862.1 | dst18 | BGC0001147 | NRP | 26.0 | 84.5 | 95.0 | 1.12e-19 |
| QCT05736.1 | Tri3 | BGC0001983 | Other | 29.0 | 54.2 | 94.0 | 1.14e-19 |
| AJK49758.1 | non-ribosomal\_peptide\_synthase | BGC0002565 | NRP | 27.0 | 62.1 | 95.0 | 1.14e-19 |
| OLZ50885.1 | non-ribosomal\_peptide\_synthetase | BGC0001461 | NRP:Glycopeptide | 26.0 | 83.1 | 95.0 | 1.2e-19 |
| AWI62626.1 | nonribosomal\_peptide\_synthetase | BGC0001822 | NRP | 23.0 | 91.5 | 95.0 | 1.22e-19 |
| AXA20091.1 | hybrid\_trans-AT\_PKS/NRPS\_LgaB | BGC0001646 | NRP+Polyketide | 25.0 | 84.5 | 95.0 | 1.24e-19 |
| AWR88409.1 | putative\_AMP-dependent\_synthetase\_and\_ligase | BGC0001522 | Polyketide | 26.0 | 57.3 | 94.0 | 1.32e-19 |
| BBG06561.1 | AMP\_binding\_enzyme | BGC0001925 | Alkaloid | 28.0 | 60.7 | 94.0 | 1.32e-19 |
| UPA71926.1 | long-chain-fatty-acid--AMP\_ligase\_FadD26 | BGC0002636 | Polyketide | 27.0 | 64.3 | 94.0 | 1.43e-19 |
| CCC55922.1 | putative\_non-ribosomal\_peptide\_synthetase | BGC0000973 | NRP+Polyketide:Modular type I polyketide | 28.0 | 60.7 | 94.0 | 1.5e-19 |
| WP\_018540607.1 | non-ribosomal\_peptide\_synthetase | BGC0001332 | NRP+Polyketide | 26.0 | 56.7 | 95.0 | 1.53e-19 |
| MCF2150416.1 | Non-ribosomal\_peptide\_synthetase | BGC0002625 | NRP+Polyketide | 23.0 | 62.0 | 95.0 | 1.63e-19 |
| AXN93611.1 | PuwC | BGC0001953 | NRP | 26.0 | 62.9 | 93.0 | 1.74e-19 |
| AJV88375.1 | MfnC | BGC0001214 | NRP | 27.0 | 59.9 | 94.0 | 2.05e-19 |
| AEH41789.1 | HrmK | BGC0000374 | NRP:Cyclic depsipeptide | 28.0 | 56.8 | 93.0 | 2.05e-19 |
| AYA22334.1 | KerC | BGC0001955 | NRP | 26.0 | 83.1 | 94.0 | 2.08e-19 |
| CAD91212.1 | putative\_non-ribosomal\_peptide\_synthetase,\_modules\_4-6 | BGC0000289 | NRP:Glycopeptide+Saccharide:Hybrid/tailoring saccharide | 25.0 | 84.5 | 94.0 | 2.08e-19 |
| APO47822.1 | non-ribosomal\_peptide\_synthetase | BGC0002653 | NRP | 25.0 | 58.7 | 94.0 | 2.15e-19 |
| AAD24881.1 | putative\_acyl-CoA\_synthetase | BGC0000127 | Polyketide | 26.0 | 58.1 | 92.0 | 2.38e-19 |
| QCQ67877.1 | hybrid\_peptide\_synthetase/polyketide\_synthase | BGC0002297 | NRP+Polyketide | 25.0 | 83.1 | 94.0 | 2.59e-19 |
| AQZ69228.1 | hypothetical\_protein | BGC0001635 | NRP+Polyketide | 26.0 | 89.6 | 94.0 | 2.7e-19 |
| ABD65958.1 | nonribosomal\_peptide\_synthetase | BGC0000341 | NRP | 25.0 | 85.7 | 94.0 | 2.89e-19 |
| AIW58892.1 | non-ribosomal\_peptide\_synthetase | BGC0001582 | NRP | 28.0 | 58.2 | 94.0 | 2.94e-19 |
| MBA0053730.1 | D-alanine--poly(phosphoribitol)\_ligase | BGC0002096 | Polyketide | 26.0 | 84.6 | 92.0 | 3.04e-19 |
| AFD30954.1 | CrmA | BGC0000966 | NRP+Polyketide | 25.0 | 57.5 | 94.0 | 3.37e-19 |
| ABP57748.1 | DepD | BGC0000993 | NRP:Cyclic depsipeptide+Polyketide:Modular type I polyketide | 26.0 | 57.5 | 94.0 | 3.48e-19 |
| AXG49819.1 | hybrid\_non-ribosomal\_peptide\_synthetase/type\_I\_polyketide\_synthase | BGC0000383 | NRP+Polyketide:Modular type I polyketide | 24.0 | 85.4 | 94.0 | 3.61e-19 |
| QED90620.1 | AMP-dependent\_ligase | BGC0002081 | Polyketide | 27.0 | 55.9 | 92.0 | 3.62e-19 |
| AEF33078.1 | dimodular\_nonribosomal\_peptide\_synthetase | BGC0001039 | NRP+Polyketide | 28.0 | 59.8 | 93.0 | 4.32e-19 |
| AGZ15459.1 | putative\_non-ribosomal\_peptide\_synthetase | BGC0001036 | NRP+Polyketide | 26.0 | 58.9 | 93.0 | 4.53e-19 |
| APZ78781.1 | nonribosomal\_peptide\_synthetase | BGC0001426 | NRP:Cyclic depsipeptide+Polyketide:Iterative type I polyketide | 26.0 | 58.4 | 93.0 | 4.59e-19 |
| AJQ95677.1 | polyketide\_synthase\_modules-related\_protein | BGC0002046 | NRP+Polyketide:Trans-AT type I polyketide | 25.0 | 56.5 | 93.0 | 4.68e-19 |
| ACC80702.1 | AMP-dependent\_synthetase\_and\_ligase | BGC0002677 | Other | 26.0 | 61.6 | 93.0 | 4.89e-19 |
| QCQ67881.1 | non-ribosomal\_peptide\_synthetase | BGC0002297 | NRP+Polyketide | 26.0 | 47.8 | 93.0 | 4.91e-19 |
| ALV82356.1 | CDA\_peptide\_synthetase\_I | BGC0001370 | NRP | 28.0 | 57.9 | 93.0 | 4.96e-19 |
| UPA71912.1 | tyrocidine\_synthase\_3 | BGC0002636 | Polyketide | 26.0 | 58.1 | 92.0 | 4.99e-19 |
| antaC | NRPS | BGC0001455 | NRP+Polyketide | 28.0 | 60.2 | 93.0 | 6.02e-19 |
| AAZ55900.1 | non-ribosomal\_peptide\_synthase:Amino\_acid\_adenylation | BGC0000359 | NRP | 26.0 | 58.1 | 93.0 | 6.18e-19 |
| AZM57024.1 | non-ribosomal\_peptide\_synthetase | BGC0002314 | NRP | 29.0 | 60.1 | 93.0 | 6.33e-19 |
| QNL14923.1 | AptC | BGC0002512 | NRP | 25.0 | 56.4 | 92.0 | 7.76e-19 |
| QYC40289.1 | A50926\_NRPS,\_modules\_4-5-6 | BGC0002344 | NRP | 25.0 | 83.2 | 92.0 | 8.24e-19 |
| ACA97580.1 | PmxE | BGC0000408 | NRP | 24.0 | 58.5 | 92.0 | 8.52e-19 |
| CAB38518.1 | CDA\_peptide\_synthetase\_I\_(CdaPs1) | BGC0000315 | NRP:Lipopeptide:Ca+-dependent lipopeptide | 26.0 | 91.8 | 92.0 | 8.61e-19 |
| WP\_019032757.1 | type\_I\_polyketide\_synthase | BGC0001331 | NRP:Cyclic depsipeptide+Polyketide:Modular type I polyketide | 27.0 | 62.1 | 92.0 | 9.97e-19 |
| CAC48360.1 | peptide\_synthetase | BGC0000311 | NRP | 24.0 | 83.9 | 92.0 | 1.05e-18 |
| AWS21283.1 | acyl-CoA\_synthetase | BGC0001934 | Polyketide | 28.0 | 65.4 | 91.0 | 1.07e-18 |
| AZY91992.1 | putative\_fatty\_acyl-AMP\_ligase | BGC0002022 | Polyketide | 28.0 | 65.4 | 91.0 | 1.07e-18 |
| AJM89738.1 | PmxE | BGC0001192 | NRP | 24.0 | 58.2 | 92.0 | 1.12e-18 |
| AVR48535.1 | CusC | BGC0001564 | NRP+Polyketide | 24.0 | 58.9 | 92.0 | 1.15e-18 |
| CAL17540.1 | peptide\_synthetase,\_putative | BGC0002465 | NRP | 25.0 | 58.4 | 92.0 | 1.25e-18 |
| AME30287.1 | L-proline\_adenyl\_transferase | BGC0001463 | Other | 23.0 | 83.5 | 90.0 | 1.31e-18 |
| WP\_053065267.1 | type\_I\_polyketide\_synthase | BGC0001330 | NRP:Cyclic depsipeptide+Polyketide:Modular type I polyketide | 29.0 | 50.8 | 92.0 | 1.31e-18 |
| AKA54626.1 | NRPS | BGC0001216 | NRP+Polyketide | 25.0 | 93.3 | 92.0 | 1.37e-18 |
| ctg1\_orf1265 |  | BGC0001752 | NRP | 26.0 | 59.6 | 92.0 | 1.39e-18 |
| AZM57022.1 | non-ribosomal\_peptide\_synthetase | BGC0002314 | NRP | 25.0 | 84.8 | 92.0 | 1.42e-18 |
| OKA09424.1 | non-ribosomal\_peptide\_synthetase | BGC0001459 | NRP:Glycopeptide | 26.0 | 82.1 | 92.0 | 1.43e-18 |
| KYC42747.1 | hypothetical\_protein | BGC0002484 | NRP+Polyketide | 26.0 | 59.5 | 92.0 | 1.48e-18 |
| AEH59100.1 | amino\_acid\_adenylation\_domain-containing\_protein/NRPS | BGC0000385 | NRP | 25.0 | 58.1 | 92.0 | 1.5e-18 |
| AHB38515.1 | non-ribosomal\_peptide\_synthetase | BGC0000345 | NRP+Polyketide:Modular type I polyketide | 27.0 | 57.9 | 91.0 | 1.71e-18 |
| CBA63680.1 | nonribosomal\_peptide\_synthetase\_NRPS | BGC0000368 | NRP | 25.0 | 62.3 | 91.0 | 1.76e-18 |
| QBC75017.1 | acyl-CoA\_synthase | BGC0001968 | NRP | 27.0 | 64.6 | 90.0 | 1.8e-18 |
| WP\_036342114.1 | type\_I\_polyketide\_synthase | BGC0001327 | NRP:Cyclic depsipeptide+Polyketide:Modular type I polyketide | 27.0 | 63.2 | 91.0 | 1.81e-18 |
| AEI58865.1 | peptide\_synthetase | BGC0000455 | NRP | 24.0 | 86.0 | 91.0 | 1.83e-18 |
| OLZ52457.1 | non-ribosomal\_peptide\_synthetase | BGC0001462 | NRP:Glycopeptide | 26.0 | 83.5 | 91.0 | 1.88e-18 |
| AEZ51520.1 | pmxE | BGC0001153 | NRP:Lipopeptide | 24.0 | 58.5 | 91.0 | 2.56e-18 |
| QNL14925.1 | AptD | BGC0002512 | NRP | 25.0 | 57.3 | 91.0 | 2.65e-18 |
| AQI70\_32580 |  | BGC0001561 | NRP | 25.0 | 82.8 | 91.0 | 2.72e-18 |
| AAM80539.1 | StaA | BGC0000290 | NRP:Glycopeptide | 26.0 | 87.4 | 91.0 | 2.95e-18 |
| KYC42745.1 | AMP-dependent\_synthetase | BGC0002484 | NRP+Polyketide | 23.0 | 89.4 | 90.0 | 3.09e-18 |
| AIG79241.1 | Hypothetical\_protein | BGC0000419 | Saccharide+NRP:Glycopeptide | 26.0 | 85.4 | 91.0 | 3.25e-18 |
| BBD17759.1 | non-ribosomal\_peptide\_synthetase | BGC0001919 | NRP+Polyketide | 26.0 | 58.9 | 91.0 | 3.3e-18 |
| AAS47562.1 | mixed\_type\_I\_polyketide\_synthase\_-\_peptide\_synthetase | BGC0001108 | NRP+Polyketide:Trans-AT type I polyketide | 25.0 | 56.4 | 91.0 | 3.37e-18 |
| ctg1\_orf8 |  | BGC0001109 | NRP+Polyketide | 25.0 | 56.4 | 91.0 | 3.37e-18 |
| ATY72525.1 | non-ribosomal\_peptide\_synthetase | BGC0001574 | NRP | 27.0 | 57.3 | 90.0 | 3.52e-18 |
| ACO94461.1 | putative\_AMP-dependent\_acyl-CoA\_synthetase/ligase | BGC0000029 | Polyketide:Modular type I polyketide | 26.0 | 58.5 | 89.0 | 3.56e-18 |
| AKD43754.1 | HerJ | BGC0001349 | NRP+Polyketide | 25.0 | 84.2 | 89.0 | 3.56e-18 |
| WA1\_15570 | hypothetical\_protein | BGC0002484 | NRP+Polyketide | 24.0 | 57.5 | 90.0 | 4.22e-18 |
| CAL17541.1 | peptide\_synthetase,\_putative | BGC0002465 | NRP | 26.0 | 57.8 | 90.0 | 4.24e-18 |
| QBG38782.1 | Atr21 | BGC0001975 | NRP | 25.0 | 82.5 | 90.0 | 4.28e-18 |
| AIE77059.1 | peptide\_synthetase | BGC0000418 | NRP | 26.0 | 85.9 | 90.0 | 4.28e-18 |
| ASX95241.1 | IlaS | BGC0001620 | NRP+Polyketide | 27.0 | 56.4 | 90.0 | 4.5e-18 |
| BAW32322.1 | nonribosomal\_peptide\_synthetase | BGC0001630 | NRP+Polyketide | 26.0 | 58.4 | 90.0 | 4.59e-18 |
| AWS21276.1 | amino\_acid\_adenyltransferase | BGC0001934 | Polyketide | 25.0 | 89.4 | 88.0 | 5.34e-18 |
| AZY92000.1 | proline\_adenylation\_protein | BGC0002022 | Polyketide | 25.0 | 89.4 | 88.0 | 5.34e-18 |
| AHZ20774.1 | non-ribosomal\_peptide\_synthase | BGC0000369 | NRP+Saccharide:Hybrid/tailoring saccharide | 24.0 | 61.3 | 90.0 | 5.59e-18 |
| AAM80537.1 | StaC | BGC0000290 | NRP:Glycopeptide | 25.0 | 84.9 | 90.0 | 5.65e-18 |
| ACZ65474.1 | palmitoyl-CoA\_synthetase | BGC0000140 | Polyketide | 27.0 | 63.5 | 89.0 | 5.75e-18 |
| AGE11891.1 | nonribosomal\_peptide\_synthetase | BGC0000366 | NRP | 27.0 | 58.1 | 89.0 | 5.79e-18 |
| AHH53506.1 | non-ribosomal\_peptide\_synthetase | BGC0000439 | NRP:Lipopeptide:Ca+-dependent lipopeptide | 27.0 | 61.5 | 90.0 | 5.81e-18 |
| ABW00331.1 | amino\_acid\_adenylation\_domain | BGC0000333 | NRP | 27.0 | 57.6 | 90.0 | 5.91e-18 |
| QGU18619.1 | polyketide\_synthase/non-ribosomal\_peptide\_synthetase | BGC0002365 | Other+Polyketide | 28.0 | 56.8 | 89.0 | 7.22e-18 |
| ABP55169.1 | amino\_acid\_adenylation\_domain | BGC0000150 | NRP+Polyketide:Enediyne type I polyketide | 26.0 | 57.1 | 88.0 | 7.43e-18 |
| AEI58866.1 | peptide\_synthetase | BGC0000455 | NRP | 26.0 | 82.3 | 89.0 | 7.43e-18 |
| QDQ83032.1 | amino\_acid\_adenylation\_domain-containing\_protein | BGC0002564 | NRP | 27.0 | 58.4 | 89.0 | 7.61e-18 |
| WP\_068925909.1 | non-ribosomal\_peptide\_synthetase | BGC0002688 | NRP | 27.0 | 48.8 | 89.0 | 9.12e-18 |
| AJD47485.1 | PpsD | BGC0002418 | NRP+Polyketide | 26.0 | 58.5 | 89.0 | 9.25e-18 |
| CCJ67647.1 | JagC | BGC0001127 | NRP | 27.0 | 58.1 | 89.0 | 9.68e-18 |
| AAT09804.1 | NocA | BGC0000395 | NRP | 29.0 | 58.4 | 89.0 | 9.69e-18 |
| ABW70809.1 | PchE | BGC0002475 | NRP | 24.0 | 61.2 | 89.0 | 9.79e-18 |
| CAA11795.1 | PCZA363.4 | BGC0000322 | NRP | 26.0 | 84.2 | 89.0 | 9.79e-18 |
| QBG38784.1 | Atr23 | BGC0001975 | NRP | 25.0 | 72.2 | 89.0 | 9.94e-18 |
| CRI73800.1 | loading\_module\_of\_NRPS-PKS | BGC0001215 | NRP | 26.0 | 86.5 | 89.0 | 9.98e-18 |
| CAJ88192.1 | Putative\_acyl-CoA\_synthetase | BGC0000151 | Polyketide:Modular type I polyketide+Saccharide:Hybrid/tailoring saccharide | 26.0 | 64.4 | 88.0 | 1e-17 |
| CAN89633.1 | putative\_hybrid\_non-ribosomal\_peptide\_synthetase/polyketide\_synthase | BGC0001070 | NRP+Polyketide:Modular type I polyketide+Polyketide:Trans-AT type I polyketide | 27.0 | 80.7 | 89.0 | 1.09e-17 |
| AAN32979.1 | BarE | BGC0000962 | NRP+Polyketide:Modular type I polyketide | 27.0 | 64.1 | 89.0 | 1.12e-17 |
| AAF00957.1 | mcyG | BGC0001017 | NRP+Polyketide:Modular type I polyketide | 26.0 | 61.6 | 89.0 | 1.22e-17 |
| BAF50711.1 | non\_ribosomal\_peptide\_synthetase\_for\_virginiamycin\_S | BGC0001116 | NRP+Polyketide | 27.0 | 56.8 | 89.0 | 1.22e-17 |
| AOZ21320.1 | SulM | BGC0001790 | NRP | 26.0 | 45.0 | 89.0 | 1.24e-17 |
| KMO93435.1 | NRPS/PKS | BGC0002095 | NRP | 28.0 | 58.2 | 88.0 | 1.24e-17 |
| E0F75\_025360 | amino\_acid\_adenylation\_domain-containing\_protein | BGC0002340 | NRP+Other | 26.0 | 61.3 | 88.0 | 1.26e-17 |
| EPS29069.1 | hypothetical\_protein | BGC0001724 | NRP+Polyketide | 24.0 | 89.0 | 89.0 | 1.29e-17 |
| QVQ62868.1 | nonribosomal\_peptide\_synthase | BGC0002373 | NRP | 27.0 | 60.1 | 89.0 | 1.31e-17 |
| AAC06346.1 | bacitracin\_synthetase\_1 | BGC0000310 | NRP | 26.0 | 59.9 | 89.0 | 1.32e-17 |
| ARR97039.1 | SphF | BGC0001780 | NRP | 25.0 | 58.4 | 88.0 | 1.54e-17 |
| APZ78722.1 | acyl-CoA\_synthetase | BGC0001420 | NRP:Cyclic depsipeptide+Polyketide:Iterative type I polyketide | 25.0 | 62.3 | 87.0 | 1.63e-17 |
| QYI86762.1 | non-ribosomal\_peptide\_synthetase | BGC0002424 | NRP | 26.0 | 60.4 | 88.0 | 1.71e-17 |
| CCP42824.1 | Possible\_fatty-acid-CoA\_ligase\_FadD10\_(fatty-acid-CoA\_synthetase)\_(fatty-acid-CoA\_synthase) | BGC0001627 | NRP | 25.0 | 85.2 | 87.0 | 1.99e-17 |
| AQV04230.1 | SwnK | BGC0001794 | NRP+Polyketide | 26.0 | 59.0 | 88.0 | 2.1e-17 |
| WP\_010639240.1 | non-ribosomal\_peptide\_synthetase | BGC0000958 | NRP:Cyclic depsipeptide+Polyketide:Modular type I polyketide | 24.0 | 88.0 | 88.0 | 2.15e-17 |
| APZ78679.1 | nonribosomal\_peptide\_synthetase | BGC0001417 | NRP:Cyclic depsipeptide+Polyketide:Iterative type I polyketide | 25.0 | 50.2 | 88.0 | 2.16e-17 |
| EME52990.1 | amino\_acid\_adenylation\_protein | BGC0001460 | NRP:Glycopeptide | 24.0 | 86.3 | 88.0 | 2.17e-17 |
| WP\_006051170.1 | non-ribosomal\_peptide\_synthetase | BGC0001999 | NRP | 26.0 | 73.1 | 88.0 | 2.29e-17 |
| BCK51629.1 | non-ribosomal\_peptide\_synthetase | BGC0002520 | Polyketide | 27.0 | 57.9 | 87.0 | 2.3e-17 |
| CUX96954.1 | TmcG | BGC0001829 | NRP+Polyketide | 27.0 | 58.5 | 87.0 | 2.7e-17 |
| RSO11553.1 | non-ribosomal\_peptide\_synthetase | BGC0002637 | NRP | 25.0 | 84.0 | 87.0 | 2.7e-17 |
| ANS62968.1 | actinomycin\_synthetase\_II | BGC0001567 | NRP | 25.0 | 83.5 | 87.0 | 2.77e-17 |
| AHB82058.1 | non\_ribosomal\_peptide\_synthetase | BGC0001019 | NRP+Polyketide:Modular type I polyketide | 29.0 | 54.3 | 87.0 | 2.94e-17 |
| AXN93578.1 | PuwC | BGC0001950 | NRP | 26.0 | 62.4 | 86.0 | 3.04e-17 |
| AXN93587.1 | PuwC | BGC0001951 | NRP | 26.0 | 62.4 | 86.0 | 3.04e-17 |
| CBZ42145.1 | non-ribosomal\_peptide\_synthetase | BGC0001117 | NRP | 28.0 | 62.1 | 87.0 | 3.06e-17 |
| BCD58482.1 | gamma-poly-L-2,4-diaminobutyric\_acid\_synthetase | BGC0002535 | NRP | 27.0 | 57.5 | 87.0 | 3.14e-17 |
| EWM62997.1 | non-ribosomal\_peptide\_synthetase | BGC0001328 | NRP:Cyclic depsipeptide+Polyketide:Modular type I polyketide | 26.0 | 62.7 | 87.0 | 3.14e-17 |
| ACM68682.1 | AerA | BGC0000298 | NRP | 24.0 | 64.1 | 87.0 | 3.19e-17 |
| AJV88376.1 | MfnD | BGC0001214 | NRP | 28.0 | 56.5 | 87.0 | 3.27e-17 |
| AGZ15460.1 | putative\_non-ribosomal\_peptide\_synthetase | BGC0001036 | NRP+Polyketide | 27.0 | 66.6 | 87.0 | 3.34e-17 |
| BAP34707.1 | AMP-dependent\_synthetase\_and\_ligase | BGC0000078 | Polyketide | 24.0 | 59.0 | 86.0 | 3.41e-17 |
| AAP92491.1 | nonribosomal\_peptide\_synthetase | BGC0000458 | NRP | 24.0 | 60.2 | 87.0 | 3.53e-17 |
| AAL33756.1 | putative\_non-ribosomal\_peptide\_synthetase | BGC0000421 | NRP | 28.0 | 59.2 | 87.0 | 3.64e-17 |
| AKA59436.1 | non-ribosomal\_peptide\_synthetase | BGC0001202 | NRP+Polyketide | 29.0 | 60.9 | 87.0 | 3.65e-17 |
| AAC44129.1 | saframycin\_Mx1\_synthetase\_A | BGC0002706 | NRP | 26.0 | 58.4 | 87.0 | 3.66e-17 |
| QNS30807.1 | hybrid\_non-ribosomal\_peptide\_synthetase/type\_I\_polyketide\_syn-thase | BGC0002509 | NRP | 26.0 | 57.9 | 87.0 | 3.76e-17 |
| CBK62746.1 |  | BGC0001115 | NRP+Polyketide | 25.0 | 59.2 | 87.0 | 3.84e-17 |
| CBG75492.1 | putative\_NRPS/siderophore\_biosynthesis\_protein | BGC0000423 | NRP | 25.0 | 58.1 | 87.0 | 4e-17 |
| BBA20967.1 | nonribosomal\_peptide\_synthetase | BGC0001763 | NRP+Polyketide | 27.0 | 56.4 | 87.0 | 4.04e-17 |
| WP\_063738219.1 | amino\_acid\_adenylation\_domain-containing\_protein | BGC0002010 | NRP+Polyketide | 26.0 | 57.3 | 86.0 | 4.71e-17 |
| AAC06347.1 | bacitracin\_synthetase\_2 | BGC0000310 | NRP | 24.0 | 62.1 | 87.0 | 4.82e-17 |
| AJI44176.1 | nonribosomal\_peptide\_synthetase | BGC0001193 | NRP | 25.0 | 63.5 | 86.0 | 4.9e-17 |
| EME52989.1 | amino\_acid\_adenylation\_protein | BGC0001460 | NRP:Glycopeptide | 27.0 | 57.5 | 87.0 | 5.09e-17 |
| CAM02313.1 | putative\_non-ribosomal\_peptide\_synthetase | BGC0000349 | NRP | 28.0 | 58.2 | 87.0 | 5.21e-17 |
| AKJ75110.1 | Bmp4 | BGC0001464 | Other | 23.0 | 82.9 | 85.0 | 5.29e-17 |
| APZ78728.1 | nonribosomal\_peptide\_synthetase | BGC0001421 | NRP:Cyclic depsipeptide+Polyketide:Iterative type I polyketide | 24.0 | 53.1 | 86.0 | 6.49e-17 |
| AJD77023.1 | IkaA | BGC0001435 | NRP+Polyketide:Iterative type I polyketide | 27.0 | 56.5 | 86.0 | 6.51e-17 |
| QBG38783.1 | Atr22 | BGC0001975 | NRP | 26.0 | 85.7 | 86.0 | 6.63e-17 |
| AGI89790.1 | ATP-dependent\_valine\_adenylase | BGC0001792 | NRP | 27.0 | 59.8 | 86.0 | 6.72e-17 |
| ABO15875.1 | amino\_acid\_adenyltransferase | BGC0000131 | Polyketide | 24.0 | 86.0 | 85.0 | 6.76e-17 |
| AEU11003.1 | NpnC | BGC0001029 | NRP+Polyketide | 25.0 | 58.2 | 86.0 | 6.82e-17 |
| KYC42612.1 | hypothetical\_protein | BGC0002484 | NRP+Polyketide | 23.0 | 59.0 | 86.0 | 6.88e-17 |
| CBG70278.1 | thaxtomin\_synthetase\_B | BGC0002089 | NRP | 26.0 | 79.0 | 86.0 | 7.4e-17 |
| CAC17499.1 | putative\_non-ribosomal\_peptide\_synthase | BGC0000324 | NRP | 27.0 | 57.5 | 86.0 | 8.14e-17 |
| AQV04224.1 | SwnK | BGC0001793 | NRP+Polyketide | 24.0 | 84.2 | 86.0 | 8.3e-17 |
| ABM21569.1 | crpA | BGC0000975 | NRP+Polyketide | 25.0 | 62.1 | 86.0 | 8.5e-17 |
| AAR12528.1 | non-ribosomal\_peptide\_synthetase | BGC0002468 | NRP | 27.0 | 56.7 | 86.0 | 8.52e-17 |
| KYC41483.1 | hypothetical\_protein | BGC0002484 | NRP+Polyketide | 23.0 | 57.6 | 86.0 | 8.82e-17 |
| ABV56588.1 | KtzH | BGC0000378 | NRP | 27.0 | 58.1 | 86.0 | 8.99e-17 |
| QWM97862.1 | hybrid\_non-ribosomal\_peptide\_synthetase/type\_I\_polyketide\_synthase | BGC0002434 | Polyketide+NRP | 28.0 | 58.4 | 86.0 | 1.13e-16 |
| AZM51139.1 | non-ribosomal\_peptide\_synthetase | BGC0002702 | NRP | 23.0 | 86.5 | 86.0 | 1.13e-16 |
| AUD08663.1 | iPKS-NRPS | BGC0001553 | NRP+Polyketide | 29.0 | 57.6 | 86.0 | 1.13e-16 |
| QXJ21808.1 | amino\_acid\_adenylation\_domain-containing\_protein | BGC0002370 | NRP | 24.0 | 86.0 | 86.0 | 1.14e-16 |
| ATP76246.1 | SpuB | BGC0001748 | NRP+Polyketide | 23.0 | 64.3 | 86.0 | 1.16e-16 |
| CAD70195.1 | non-ribosomal\_peptide\_synthetase | BGC0001047 | NRP+Polyketide | 26.0 | 60.4 | 86.0 | 1.16e-16 |
| BAH43766.1 | tyrocidine\_synthetase\_III | BGC0000452 | NRP | 27.0 | 56.8 | 86.0 | 1.2e-16 |
| WP\_084702182.1 | non-ribosomal\_peptide\_synthetase | BGC0001211 | NRP | 28.0 | 57.8 | 86.0 | 1.21e-16 |
| CBW54660.1 | Acyl-CoA\_ligase | BGC0000971 | NRP+Polyketide:Modular type I polyketide | 27.0 | 65.7 | 84.0 | 1.23e-16 |
| APZ78845.1 | nonribosomal\_peptide\_synthetase | BGC0001431 | NRP:Cyclic depsipeptide+Polyketide:Iterative type I polyketide | 26.0 | 52.3 | 85.0 | 1.48e-16 |
| QIE08736.1 | non-ribosomal\_peptide\_synthetase | BGC0002544 | NRP | 26.0 | 59.6 | 85.0 | 1.52e-16 |
| ctg1\_orf1264 |  | BGC0001752 | NRP | 25.0 | 62.1 | 85.0 | 1.53e-16 |
| AAC83656.1 | dihydroaeruginoic\_acid\_synthetase | BGC0000412 | NRP | 29.0 | 44.6 | 85.0 | 1.67e-16 |
| CAM56771.1 |  | BGC0000354 | NRP | 27.0 | 57.8 | 85.0 | 1.88e-16 |
| AAT12283.1 | LtxA | BGC0000384 | NRP | 26.0 | 46.4 | 85.0 | 1.89e-16 |
| ADG27358.1 | peptide\_synthetase | BGC0000296 | NRP | 26.0 | 57.9 | 85.0 | 1.9e-16 |
| AQM37583.1 | nonribosomal\_peptide\_synthetase | BGC0001424 | NRP:Cyclic depsipeptide+Polyketide:Iterative type I polyketide | 24.0 | 59.8 | 85.0 | 1.94e-16 |
| APZ78691.1 | nonribosomal\_peptide\_synthetase | BGC0001418 | NRP:Cyclic depsipeptide+Polyketide:Iterative type I polyketide | 25.0 | 49.2 | 85.0 | 1.95e-16 |
| APZ78703.1 | nonribosomal\_peptide\_synthetase | BGC0001419 | NRP:Cyclic depsipeptide+Polyketide:Iterative type I polyketide | 24.0 | 57.0 | 85.0 | 1.95e-16 |
| CAG29031.1 | nonribosomal\_peptide\_synthetase\_(modules\_1\_and\_2) | BGC0001023 | NRP+Polyketide:Modular type I polyketide | 26.0 | 50.6 | 85.0 | 1.95e-16 |
| APZ78821.1 | nonribosomal\_peptide\_synthetase | BGC0001429 | NRP:Cyclic depsipeptide+Polyketide:Iterative type I polyketide | 26.0 | 50.6 | 85.0 | 1.95e-16 |
| BAG17643.1 | putative\_NRPS-type-I\_PKS\_fusion\_protein | BGC0001043 | NRP+Polyketide | 27.0 | 56.8 | 85.0 | 1.95e-16 |
| AHB82071.1 | non\_ribosomal\_peptide\_synthetase | BGC0001231 | NRP+Polyketide:Modular type I polyketide | 25.0 | 75.2 | 85.0 | 2.01e-16 |
| AGI89788.1 | Nonribosomal\_peptide\_synthetase | BGC0001792 | NRP | 25.0 | 58.2 | 85.0 | 2.01e-16 |
| EJK79843.1 | amino\_acid\_adenylation\_enzyme/thioester\_reductase\_family\_protein | BGC0000436 | NRP | 23.0 | 72.8 | 85.0 | 2.01e-16 |
| APZ78834.1 | nonribosomal\_peptide\_synthetase | BGC0001430 | NRP:Cyclic depsipeptide+Polyketide:Iterative type I polyketide | 26.0 | 58.2 | 85.0 | 2.02e-16 |
| BAH22764.1 | nonribosomal\_peptide\_synthetase | BGC0001018 | NRP | 26.0 | 58.4 | 85.0 | 2.03e-16 |
| QRN75755.1 | Amino\_acid\_adenylation\_domain\_protein | BGC0002114 | NRP+Polyketide | 24.0 | 89.6 | 84.0 | 2.42e-16 |
| CAA16183.1 | polyketide\_synthase | BGC0001063 | NRP+Polyketide | 24.0 | 91.6 | 84.0 | 2.46e-16 |
| CAD29795.1 | peptide\_synthetase | BGC0001015 | NRP+Polyketide | 24.0 | 87.0 | 84.0 | 2.51e-16 |
| CAJ45639.1 | vanchrobactin\_non\_ribosomal\_peptide\_synthetase | BGC0000454 | NRP | 24.0 | 58.5 | 84.0 | 2.54e-16 |
| AAQ59905.1 | synthetase\_CbsF | BGC0002680 | NRP | 22.0 | 91.6 | 84.0 | 2.54e-16 |
| WP\_069848010.1 | non-ribosomal\_peptide\_synthetase | BGC0002472 | NRP | 27.0 | 55.7 | 84.0 | 2.56e-16 |
| APZ78755.1 | nonribosomal\_peptide\_synthetase | BGC0001423 | NRP:Cyclic depsipeptide+Polyketide:Iterative type I polyketide | 25.0 | 47.4 | 84.0 | 2.56e-16 |
| APZ78715.1 | nonribosomal\_peptide\_synthetase | BGC0001420 | NRP:Cyclic depsipeptide+Polyketide:Iterative type I polyketide | 25.0 | 49.2 | 84.0 | 2.56e-16 |
| CEK23364.1 | putative\_Phenylalanine\_racemase\_(ATP-hydrolyzing) | BGC0001716 | NRP | 25.0 | 58.1 | 84.0 | 2.61e-16 |
| AAZ03552.1 | McnC | BGC0000332 | NRP | 26.0 | 58.4 | 84.0 | 2.67e-16 |
| BAP82667.1 | non-ribosomal\_peptide\_synthetase\_A-domain\_containing\_protein | BGC0001148 | NRP+RiPP | 24.0 | 87.0 | 83.0 | 3.19e-16 |
| ALJ49909.1 | TlmI | BGC0001237 | Polyketide | 26.0 | 57.0 | 84.0 | 3.2e-16 |
| AGE11898.1 | nonribosomal\_peptide\_synthetase | BGC0000366 | NRP | 26.0 | 56.4 | 84.0 | 3.29e-16 |
| APZ78855.1 | nonribosomal\_peptide\_synthetase | BGC0001432 | NRP:Cyclic depsipeptide+Polyketide:Iterative type I polyketide | 25.0 | 50.2 | 84.0 | 3.37e-16 |
| APZ78833.1 | nonribosomal\_peptide\_synthetase | BGC0001430 | NRP:Cyclic depsipeptide+Polyketide:Iterative type I polyketide | 25.0 | 58.2 | 84.0 | 3.37e-16 |
| AGP37410.1 | peptide\_synthetase | BGC0002386 | NRP+Polyketide | 24.0 | 58.1 | 84.0 | 3.44e-16 |
| CAL80821.1 | sylD-like\_NRPS/PKS | BGC0000997 | NRP+Polyketide | 26.0 | 60.6 | 84.0 | 3.48e-16 |
| KYC42613.1 | non-ribosomal\_peptide\_synthetase | BGC0002484 | NRP+Polyketide | 24.0 | 66.0 | 84.0 | 3.53e-16 |
| AAC06348.1 | bacitracin\_synthetase\_3 | BGC0000310 | NRP | 26.0 | 59.6 | 84.0 | 3.58e-16 |
| AQW35047.1 | Acyl-CoA\_synthetase | BGC0001675 | Polyketide | 26.0 | 65.7 | 83.0 | 3.66e-16 |
| QGA70148.1 | nonribosomal\_peptide\_synthetase | BGC0002293 | NRP | 26.0 | 57.8 | 84.0 | 3.69e-16 |
| AIW82280.1 | PuwC | BGC0001125 | NRP+Polyketide | 26.0 | 62.4 | 83.0 | 3.83e-16 |
| EFL06865.1 | hypothetical\_protein | BGC0000300 | NRP | 26.0 | 57.6 | 84.0 | 4.27e-16 |
| CAC11137.1 | NikP1\_protein | BGC0000876 | Other | 27.0 | 57.5 | 83.0 | 4.39e-16 |
| BAH04161.1 | putative\_non-ribosomal\_peptide\_synthetase | BGC0000450 | NRP | 27.0 | 49.7 | 84.0 | 4.45e-16 |
| AXF16146.1 | non-ribosomal\_peptide\_synthetase | BGC0002563 | NRP | 25.0 | 72.8 | 84.0 | 4.68e-16 |
| AOE23577.1 | FoxBI | BGC0001598 | NRP+Polyketide | 24.0 | 62.6 | 82.0 | 4.91e-16 |
| DAB41484.1 | nonribosomal\_peptide\_synthetase/polyketide\_synthase\_type\_I | BGC0001230 | NRP:Cyclic depsipeptide+Polyketide:Modular type I polyketide | 25.0 | 84.3 | 83.0 | 5.69e-16 |
| WP\_010369430.1 | non-ribosomal\_peptide\_synthetase | BGC0000314 | Polyketide+NRP:Cyclic depsipeptide+Other:Aminocoumarin | 24.0 | 59.3 | 83.0 | 5.88e-16 |
| BCD33690.1 | non-ribosomal\_peptide\_synthetase | BGC0002448 | NRP | 28.0 | 59.9 | 83.0 | 6.19e-16 |
| mycF | polyketide\_synthase | BGC0002055 | NRP+Polyketide:Trans-AT type I polyketide | 27.0 | 49.5 | 83.0 | 6.31e-16 |
| CAI94718.1 | putative\_CoA\_ligase | BGC0000141 | Polyketide | 25.0 | 85.4 | 83.0 | 6.44e-16 |
| AXG47410.1 | non-ribosomal\_peptide\_synthetase | BGC0002715 | NRP+Polyketide | 29.0 | 43.0 | 83.0 | 7.21e-16 |
| AAG06715.1 | probable\_non-ribosomal\_peptide\_synthetase | BGC0002037 | NRP | 23.0 | 85.6 | 83.0 | 7.39e-16 |
| APZ78756.1 | nonribosomal\_peptide\_synthetase | BGC0001423 | NRP:Cyclic depsipeptide+Polyketide:Iterative type I polyketide | 23.0 | 85.9 | 83.0 | 7.77e-16 |
| WP\_003981346.1 | non-ribosomal\_peptide\_synthetase | BGC0001813 | NRP | 27.0 | 56.5 | 83.0 | 7.8e-16 |
| AJV88377.1 | MfnE | BGC0001214 | NRP | 26.0 | 72.2 | 83.0 | 7.82e-16 |
| AAX31558.1 | peptide\_synthetase\_2 | BGC0000336 | NRP | 24.0 | 85.9 | 83.0 | 8.21e-16 |
| CAD15513.1 | non-ribosomal\_peptide\_synthetase | BGC0001014 | NRP:NRP siderophore+Polyketide:Modular type I polyketide+Polyketide:Iterative type I polyketide | 26.0 | 50.6 | 82.0 | 9.45e-16 |
| QYC40287.1 | A50926\_NRPS,\_modules\_1-2 | BGC0002344 | NRP | 26.0 | 82.8 | 82.0 | 9.53e-16 |
| CAD91220.1 | putative\_non-ribosomal\_peptide\_synthetase,\_modules\_1-2 | BGC0000289 | NRP:Glycopeptide+Saccharide:Hybrid/tailoring saccharide | 26.0 | 82.6 | 82.0 | 9.53e-16 |
| ABD65956.1 | nonribosomal\_peptide\_synthetase | BGC0000341 | NRP | 29.0 | 57.0 | 82.0 | 9.54e-16 |
| PVC99845.1 | Adenylation-domain-containg\_protein | BGC0002100 | NRP+Other | 28.0 | 59.9 | 81.0 | 9.58e-16 |
| QBC75023.1 | non-ribosomal\_peptide\_synthetase | BGC0001968 | NRP | 28.0 | 45.3 | 82.0 | 9.97e-16 |
| AQX14441.1 | EM5400\_NRPS\_scaffold | BGC0001671 | NRP | 26.0 | 59.2 | 82.0 | 1e-15 |
| AXN93595.1 | PuwI | BGC0001952 | NRP | 25.0 | 62.9 | 82.0 | 1.05e-15 |
| BAF50727.1 | hybrid\_polyketide\_synthase-non\_ribosomal\_peptide\_synthetase | BGC0001116 | NRP+Polyketide | 25.0 | 56.7 | 82.0 | 1.08e-15 |
| ABI26079.1 | OciC | BGC0000331 | NRP | 25.0 | 59.0 | 82.0 | 1.14e-15 |
| QED55419.1 | fatty-acyl\_AMP\_ligase | BGC0001984 | NRP | 26.0 | 51.2 | 81.0 | 1.16e-15 |
| EFY95969.1 | polyketide\_synthase | BGC0002270 | NRP+Polyketide | 26.0 | 59.3 | 82.0 | 1.29e-15 |
| QBA57735.1 | NRPS | BGC0002377 | NRP | 26.0 | 71.9 | 82.0 | 1.3e-15 |
| ABD14712.1 | cesB | BGC0000320 | NRP:Cyclic depsipeptide | 26.0 | 60.9 | 82.0 | 1.31e-15 |
| ctg1\_orf20 |  | BGC0001767 | NRP | 27.0 | 58.5 | 82.0 | 1.39e-15 |
| TRX17524.1 | amino\_acid\_adenylation\_domain-containing\_protein | BGC0002329 | NRP | 24.0 | 85.4 | 82.0 | 1.4e-15 |
| BAK64635.1 | putative\_CoA\_ligase | BGC0000135 | Polyketide | 26.0 | 64.6 | 81.0 | 1.49e-15 |
| ABC34305.1 | peptide\_synthetase,\_putative | BGC0000961 | NRP+Polyketide | 24.0 | 56.7 | 82.0 | 1.51e-15 |
| BAG84247.1 | putative\_L-prolyl-AMP\_ligase | BGC0000257 | Polyketide | 24.0 | 74.8 | 81.0 | 1.63e-15 |
| EHK22005.1 | putative\_non-ribosomal\_peptide\_synthetase\_GliP | BGC0001609 | NRP | 26.0 | 72.7 | 82.0 | 1.64e-15 |
| APZ78743.1 | nonribosomal\_peptide\_synthetase | BGC0001422 | NRP:Cyclic depsipeptide+Polyketide:Iterative type I polyketide | 23.0 | 60.6 | 82.0 | 1.75e-15 |
| EWS95124.1 | hypothetical\_protein | BGC0000306 | NRP:Lipopeptide | 27.0 | 58.7 | 82.0 | 1.81e-15 |
| AXN93608.1 | PuwI | BGC0001953 | NRP | 24.0 | 62.6 | 81.0 | 1.85e-15 |
| AAL15600.1 | SimH | BGC0000270 | Polyketide | 25.0 | 84.8 | 81.0 | 2.28e-15 |
| AAK06804.1 | Tyroxyl-AMP-forming\_enzyme | BGC0001072 | Saccharide+Polyketide:Modular type I polyketide+Polyketide:Type II polyketide+Other:Aminocoumarin | 25.0 | 84.8 | 81.0 | 2.3e-15 |
| AEH59099.1 | amino\_acid\_adenylation\_domain-containing\_protein/NRPS | BGC0000385 | NRP | 23.0 | 84.6 | 81.0 | 2.35e-15 |
| ABC35522.1 | thiotemplate\_mechanism\_natural\_product\_synthetase | BGC0000186 | NRP+Polyketide:Modular type I polyketide | 26.0 | 59.0 | 81.0 | 2.36e-15 |
| APZ78729.1 | nonribosomal\_peptide\_synthetase | BGC0001421 | NRP:Cyclic depsipeptide+Polyketide:Iterative type I polyketide | 24.0 | 91.6 | 81.0 | 2.42e-15 |
| ALV86867.1 | Tlo21 | BGC0001406 | NRP | 28.0 | 56.1 | 81.0 | 2.43e-15 |
| MCF2151708.1 | Non-ribosomal\_peptide\_synthetase | BGC0002625 | NRP+Polyketide | 26.0 | 57.5 | 81.0 | 2.44e-15 |
| AZM57023.1 | non-ribosomal\_peptide\_synthetase | BGC0002314 | NRP | 25.0 | 83.9 | 81.0 | 2.67e-15 |
| WP\_020993844.1 | non-ribosomal\_peptide\_synthetase | BGC0001575 | NRP | 24.0 | 61.2 | 81.0 | 3.01e-15 |
| WP\_050383082.1 | non-ribosomal\_peptide\_synthetase | BGC0001451 | NRP | 25.0 | 84.5 | 81.0 | 3.03e-15 |
| AFD30953.1 | CrmB | BGC0000966 | NRP+Polyketide | 24.0 | 84.0 | 81.0 | 3.08e-15 |
| BAV57443.1 | NRPS\_(C-A-PCP-TE) | BGC0001818 | NRP | 25.0 | 58.7 | 81.0 | 3.41e-15 |
| AWI62627.1 | nonribosomal\_peptide\_synthetase | BGC0001822 | NRP | 24.0 | 57.8 | 81.0 | 3.78e-15 |
| APD26279.1 | PtmA | BGC0001726 | NRP+Polyketide | 26.0 | 58.1 | 81.0 | 3.98e-15 |
| WP\_012408785.1 | non-ribosomal\_peptide\_synthetase | BGC0002061 | NRP:Cyclic depsipeptide+Polyketide:Modular type I polyketide | 27.0 | 49.1 | 81.0 | 4.07e-15 |
| CAA16182.1 | putative\_peptide\_synthase | BGC0001063 | NRP+Polyketide | 26.0 | 78.7 | 79.0 | 4.08e-15 |
| AID65222.1 | putative\_aspartate\_racemase | BGC0000335 | NRP+Polyketide | 25.0 | 70.2 | 81.0 | 4.1e-15 |
| SDF67296.1 | Acyl-CoA\_synthetase\_(AMP-forming)/AMP-acid\_ligase\_II | BGC0002422 | NRP | 27.0 | 66.1 | 80.0 | 4.2e-15 |
| ACO78736.1 | Non-ribosomal\_peptide\_synthase,\_PvdD-like\_protein | BGC0002433 | NRP | 23.0 | 84.3 | 80.0 | 5.19e-15 |
| WP\_153044786.1 | non-ribosomal\_peptide\_synthetase | BGC0001826 | NRP | 26.0 | 61.2 | 80.0 | 5.72e-15 |
| AFR69331.1 | nonribosomal\_peptide\_synthetase\_SpiA | BGC0001045 | NRP:Cyclic depsipeptide+Polyketide:Modular type I polyketide | 26.0 | 58.2 | 80.0 | 6.21e-15 |
| CAG25758.1 | putative\_type\_I\_polyketide\_synthase | BGC0000311 | NRP | 29.0 | 52.3 | 78.0 | 6.4e-15 |
| CZT62784.1 | Non-ribosomal\_peptide\_synthase,\_involved\_in\_Hassallidin\_biosynthesis | BGC0001614 | NRP | 24.0 | 60.6 | 80.0 | 7.01e-15 |
| APZ78809.1 | nonribosomal\_peptide\_synthetase | BGC0001428 | NRP:Cyclic depsipeptide+Polyketide:Iterative type I polyketide | 24.0 | 87.4 | 80.0 | 7.12e-15 |
| CEK23605.1 | Non-ribosomal\_peptide\_synthase\_involved\_in\_xenematides\_synthesis | BGC0001825 | NRP | 24.0 | 59.8 | 80.0 | 7.18e-15 |
| ACO78737.1 | Non-ribosomal\_peptide\_synthase,\_PvdD/PvdJ-like\_protein | BGC0002433 | NRP | 24.0 | 58.7 | 80.0 | 7.2e-15 |
| AAF19815.1 | mtaG | BGC0001024 | NRP+Polyketide:Modular type I polyketide | 29.0 | 42.9 | 79.0 | 8.26e-15 |
| ALK21570.1 | non-ribosomal\_peptide\_synthetase | BGC0002678 | NRP | 26.0 | 63.5 | 79.0 | 8.33e-15 |
| CCA53804.1 | pyochelin\_synthetase\_F | BGC0001801 | NRP | 24.0 | 71.6 | 79.0 | 8.33e-15 |
| AAK89720.1 | non-ribosomal\_peptide\_synthetase,\_siderophore\_biosynthesis\_protein | BGC0002107 | NRP+Polyketide | 23.0 | 58.7 | 79.0 | 8.75e-15 |
| ALK27916.1 | non-ribosomal\_peptide\_synthase | BGC0001233 | NRP | 25.0 | 59.8 | 79.0 | 9.14e-15 |
| CAE53352.1 | non-ribosomal\_peptide\_synthetase | BGC0000440 | NRP:Glycopeptide | 28.0 | 56.5 | 79.0 | 9.3e-15 |
| CAG15011.1 | peptide\_synthetase,\_module\_4-6 | BGC0000441 | NRP | 28.0 | 56.5 | 79.0 | 9.3e-15 |
| ABI26078.1 | OciB | BGC0000331 | NRP | 25.0 | 60.9 | 79.0 | 9.41e-15 |
| AEC14347.1 | nonribosomal\_peptide\_synthetase | BGC0000377 | NRP | 24.0 | 60.6 | 79.0 | 1.06e-14 |
| CCA29202.1 | non-ribosomal\_peptide\_synthetase | BGC0000955 | NRP+Polyketide:Modular type I polyketide | 25.0 | 54.5 | 79.0 | 1.15e-14 |
| ALG65319.1 | Cal17 | BGC0001297 | NRP | 26.0 | 82.1 | 79.0 | 1.16e-14 |
| DAB41477.1 | nonribosomal\_peptide\_synthetase | BGC0001230 | NRP:Cyclic depsipeptide+Polyketide:Modular type I polyketide | 26.0 | 64.9 | 79.0 | 1.22e-14 |
| QXF14600.1 | PydA | BGC0002239 | Polyketide+NRP | 26.0 | 62.7 | 79.0 | 1.23e-14 |
| AEH41793.1 | HrmO | BGC0000374 | NRP:Cyclic depsipeptide | 25.0 | 55.6 | 79.0 | 1.25e-14 |
| QEO75075.1 | condensation\_domain-containing\_protein | BGC0002079 | NRP:Cyclic depsipeptide | 27.0 | 57.5 | 79.0 | 1.25e-14 |
| CBL93730.1 | NRPS | BGC0000360 | NRP | 28.0 | 46.0 | 79.0 | 1.28e-14 |
| KFL51881.1 | beta-ketoacyl\_synthase | BGC0001711 | NRP+Polyketide | 23.0 | 89.3 | 79.0 | 1.37e-14 |
| AAW49319.1 | thaxtomin\_synthetase\_B | BGC0000444 | NRP | 25.0 | 77.6 | 79.0 | 1.38e-14 |
| ABL86391.1 | hybrid\_polyketide\_synthase\_and\_nonribosomal\_peptide\_synthetase | BGC0000999 | NRP+Polyketide | 24.0 | 85.9 | 79.0 | 1.57e-14 |
| CAN89638.1 | putative\_non-ribosomal\_peptide\_synthetase | BGC0001070 | NRP+Polyketide:Modular type I polyketide+Polyketide:Trans-AT type I polyketide | 27.0 | 58.9 | 78.0 | 1.6e-14 |
| AHJ31215.1 | Long-chain-fatty-acid--CoA\_ligase | BGC0000430 | NRP+Polyketide:Modular type I polyketide | 25.0 | 59.9 | 79.0 | 1.61e-14 |
| WP\_245566645.1 | amino\_acid\_adenylation\_domain-containing\_protein | BGC0002467 | NRP | 26.0 | 58.5 | 79.0 | 1.64e-14 |
| EFE73312.1 | nonribosomal\_peptide\_synthetase | BGC0000431 | NRP:Cyclic depsipeptide | 25.0 | 59.9 | 79.0 | 1.65e-14 |
| WP\_078586793.1 | non-ribosomal\_peptide\_synthetase | BGC0001760 | NRP | 25.0 | 82.8 | 78.0 | 1.78e-14 |
| AAF00961.1 | mcyB | BGC0001017 | NRP+Polyketide:Modular type I polyketide | 25.0 | 57.8 | 78.0 | 1.95e-14 |
| AJW76711.1 | DsaI | BGC0001196 | NRP | 25.0 | 57.3 | 78.0 | 1.99e-14 |
| AHH53508.1 | non-ribosomal\_peptide\_synthetase | BGC0000439 | NRP:Lipopeptide:Ca+-dependent lipopeptide | 25.0 | 88.7 | 78.0 | 1.99e-14 |
| AWO77084.1 | hybrid\_non-ribosomal\_peptide\_synthetase/type\_I\_polyketide\_synthase | BGC0001556 | NRP+Polyketide | 26.0 | 57.0 | 78.0 | 2.07e-14 |
| BAZ95823.1 | PKS-NRPS\_hybrid\_cpaA | BGC0001563 | NRP+Polyketide | 23.0 | 77.8 | 78.0 | 2.1e-14 |
| AZM50111.1 | non-ribosomal\_peptide\_synthetase | BGC0002702 | NRP | 25.0 | 88.4 | 78.0 | 2.13e-14 |
| SJZ83675.1 | non-ribosomal\_peptide\_synthase\_domain\_TIGR01720/amino\_acid\_adenylation\_domain-containing\_protein/thioester\_reductase\_domain-containing\_protein | BGC0002660 | NRP | 22.0 | 93.3 | 78.0 | 2.17e-14 |
| WP\_050383094.1 | non-ribosomal\_peptide\_synthetase | BGC0001451 | NRP | 25.0 | 84.5 | 78.0 | 2.26e-14 |
| QEO74979.1 | omn4 | BGC0002078 | NRP:Cyclic depsipeptide | 26.0 | 50.5 | 77.0 | 2.3e-14 |
| CAC17500.1 | putative\_non-ribosomal\_peptide\_synthase | BGC0000324 | NRP | 28.0 | 53.6 | 78.0 | 2.5e-14 |
| WP\_051206792.1 | fatty\_acyl-AMP\_ligase | BGC0002624 | NRP+Polyketide | 26.0 | 63.8 | 77.0 | 2.58e-14 |
| AGZ15458.1 | putative\_non-ribosomal\_peptide\_synthetase | BGC0001036 | NRP+Polyketide | 26.0 | 59.6 | 78.0 | 2.65e-14 |
| EPH46596.1 | putative\_Linear\_gramicidin\_synthase\_subunit\_C | BGC0001519 | NRP+Polyketide | 24.0 | 52.8 | 78.0 | 2.68e-14 |
| CBJ79915.1 | putative\_Phenylalanine\_racemase\_(ATP-hydrolyzing) | BGC0001133 | NRP | 24.0 | 50.0 | 78.0 | 2.77e-14 |
| APZ78769.1 | nonribosomal\_peptide\_synthetase | BGC0001425 | NRP:Cyclic depsipeptide+Polyketide:Iterative type I polyketide | 23.0 | 92.1 | 78.0 | 2.8e-14 |
| APZ78782.1 | nonribosomal\_peptide\_synthetase | BGC0001426 | NRP:Cyclic depsipeptide+Polyketide:Iterative type I polyketide | 23.0 | 93.0 | 78.0 | 2.8e-14 |
| CBJ79916.1 | putative\_Ornithine\_racemase | BGC0001133 | NRP | 24.0 | 50.0 | 78.0 | 2.81e-14 |
| KDM89832.1 | peptide\_synthetase | BGC0002412 | NRP | 24.0 | 61.2 | 77.0 | 2.87e-14 |
| AHF22854.1 | MarL | BGC0000091 | Polyketide | 25.0 | 64.3 | 77.0 | 3.41e-14 |
| AAY37650.1 | Amino\_acid\_adenylation | BGC0000437 | NRP | 28.0 | 57.0 | 77.0 | 3.43e-14 |
| EPH46597.1 | putative\_Linear\_gramicidin\_synthase\_subunit\_C | BGC0001519 | NRP+Polyketide | 24.0 | 63.5 | 77.0 | 3.48e-14 |
| AJK49757.1 | non-ribosomal\_peptide\_synthase | BGC0002565 | NRP | 25.0 | 62.1 | 77.0 | 3.49e-14 |
| AAT01806.1 | non-ribosomal\_peptide\_synthetase | BGC0000365 | NRP | 24.0 | 57.8 | 77.0 | 3.6e-14 |
| AJK49766.1 | non-ribosomal\_peptide\_synthase | BGC0002565 | NRP | 24.0 | 91.8 | 77.0 | 3.62e-14 |
| WP\_050383088.1 | non-ribosomal\_peptide\_synthetase | BGC0001451 | NRP | 27.0 | 58.2 | 77.0 | 3.64e-14 |
| EET76303.1 | AMP-binding\_enzyme | BGC0002685 | NRP | 25.0 | 99.4 | 77.0 | 4.09e-14 |
| CAJ76286.1 | putative\_non-ribosomal\_peptide\_synthetase | BGC0000972 | NRP+Polyketide:Modular type I polyketide+Polyketide:Trans-AT type I polyketide | 22.0 | 81.7 | 77.0 | 4.1e-14 |
| CAJ34367.1 | NRPS\_protein | BGC0000445 | NRP:Cyclic depsipeptide | 24.0 | 84.5 | 76.0 | 4.39e-14 |
| AET98916.1 | putative\_non-ribosomal\_peptide\_synthetase | BGC0000415 | NRP | 26.0 | 58.7 | 76.0 | 4.5e-14 |
| WP\_082191961.1 | non-ribosomal\_peptide\_synthetase | BGC0001451 | NRP | 24.0 | 84.2 | 77.0 | 4.66e-14 |
| UMM61371.1 | Tsk10 | BGC0002661 | NRP | 24.0 | 87.6 | 77.0 | 4.75e-14 |
| APZ78680.1 | nonribosomal\_peptide\_synthetase | BGC0001417 | NRP:Cyclic depsipeptide+Polyketide:Iterative type I polyketide | 23.0 | 88.8 | 77.0 | 4.78e-14 |
| CAJ87590.1 | putative\_peptide\_synthase | BGC0001055 | NRP+Polyketide | 25.0 | 52.8 | 77.0 | 5.8e-14 |
| MBN3579112.1 | amino\_acid\_adenylation\_domain-containing\_protein | BGC0002613 | NRP+Polyketide | 28.0 | 43.3 | 77.0 | 5.8e-14 |
| ALG65318.1 | Cal18 | BGC0001297 | NRP | 27.0 | 57.3 | 77.0 | 6.01e-14 |
| AHB82051.1 | polyketide\_synthase | BGC0001019 | NRP+Polyketide:Modular type I polyketide | 27.0 | 51.7 | 77.0 | 6.19e-14 |
| AAY91420.2 | non-ribosomal\_peptide\_synthetase\_OfaB | BGC0000399 | NRP:Cyclic depsipeptide | 23.0 | 84.0 | 77.0 | 6.35e-14 |
| MCC5036785.1 | amino\_acid\_adenylation\_domain-containing\_protein | BGC0002638 | NRP | 24.0 | 84.2 | 77.0 | 6.37e-14 |
| AAW49318.1 | thaxtomin\_synthetase\_A | BGC0000444 | NRP | 24.0 | 75.2 | 76.0 | 7.09e-14 |
| QEO75071.1 | AMP-dependent\_synthetase\_and\_ligase | BGC0002079 | NRP:Cyclic depsipeptide | 26.0 | 50.5 | 76.0 | 7.16e-14 |
| AGS77307.1 | NRPS\_modules\_1-2 | BGC0001178 | NRP:Glycopeptide | 26.0 | 81.7 | 76.0 | 7.66e-14 |
| AAY93356.2 | non-ribosomal\_peptide\_synthetase\_PvdI | BGC0000413 | NRP | 23.0 | 57.6 | 76.0 | 8.23e-14 |
| ATJ34005.1 | adenylation\_domain-containing\_protein | BGC0001442 | NRP | 27.0 | 56.8 | 75.0 | 8.26e-14 |
| AAY93354.1 | non-ribosomal\_peptide\_synthetase\_PvdD | BGC0000413 | NRP | 23.0 | 57.6 | 76.0 | 8.35e-14 |
| KPN90369.1 | NunE | BGC0001416 | NRP | 24.0 | 57.3 | 76.0 | 8.36e-14 |
| QNH67551.1 | Cip23 | BGC0002108 | NRP | 26.0 | 55.9 | 76.0 | 8.4e-14 |
| QED55423.1 | nonribosomal\_peptide\_synthetase | BGC0001984 | NRP | 26.0 | 59.9 | 76.0 | 8.57e-14 |
| BAY02129.1 | barbamide\_biosynthesis\_protein\_BarG | BGC0002532 | NRP+Polyketide | 24.0 | 58.5 | 76.0 | 8.7e-14 |
| CAJ34381.1 | NRPS\_protein | BGC0000445 | NRP:Cyclic depsipeptide | 26.0 | 56.7 | 76.0 | 9.8e-14 |
| BAH04173.1 | putative\_non-ribosomal\_peptide\_synthetase | BGC0000450 | NRP | 23.0 | 82.6 | 75.0 | 1.01e-13 |
| ADG27359.1 | peptide\_synthetase | BGC0000296 | NRP | 28.0 | 58.7 | 76.0 | 1.09e-13 |
| QRG35014.1 | NRPS | BGC0002378 | NRP | 25.0 | 56.8 | 76.0 | 1.11e-13 |
| WP\_100939443.1 | non-ribosomal\_peptide\_synthetase | BGC0002071 | NRP:Lipopeptide | 26.0 | 57.9 | 76.0 | 1.12e-13 |
| ABU70377.1 | hypothetical\_protein | BGC0001890 | NRP | 23.0 | 73.0 | 76.0 | 1.19e-13 |
| CCA53799.1 | iron\_aquisition\_yersiniabactin\_synthesis\_enzyme | BGC0001801 | NRP | 25.0 | 60.6 | 76.0 | 1.24e-13 |
| QNL14922.1 | AptA | BGC0002512 | NRP | 22.0 | 61.8 | 76.0 | 1.34e-13 |
| CDG17982.1 | Non-ribosomal\_peptide\_synthetase | BGC0000464 | NRP:Cyclic depsipeptide | 21.0 | 86.3 | 76.0 | 1.45e-13 |
| ABX71111.1 | Lct28 | BGC0000238 | Polyketide | 26.0 | 59.8 | 74.0 | 1.47e-13 |
| QEO74982.1 | omn7 | BGC0002078 | NRP:Cyclic depsipeptide | 27.0 | 56.4 | 76.0 | 1.48e-13 |
| AAL33758.1 | putative\_non-ribosomal\_peptide\_synthetase | BGC0000421 | NRP | 25.0 | 57.1 | 75.0 | 1.61e-13 |
| DAB41478.1 | nonribosomal\_peptide\_synthetase | BGC0001230 | NRP:Cyclic depsipeptide+Polyketide:Modular type I polyketide | 26.0 | 58.4 | 75.0 | 1.64e-13 |
| ABO15844.1 | amino\_acid\_adenyltransferase | BGC0000130 | Polyketide | 23.0 | 82.8 | 74.0 | 1.73e-13 |
| WP\_010369428.1 | non-ribosomal\_peptide\_synthetase | BGC0000314 | Polyketide+NRP:Cyclic depsipeptide+Other:Aminocoumarin | 24.0 | 59.8 | 75.0 | 1.8e-13 |
| ACG60776.1 | NRPS(AL/ACP/C/A/PCP/C/A) | BGC0001058 | NRP:Glycopeptide+Polyketide:Modular type I polyketide+Saccharide:Hybrid/tailoring saccharide | 25.0 | 56.2 | 75.0 | 1.81e-13 |
| AXB34356.1 | non-ribosomal\_peptide\_synthetase | BGC0002415 | NRP | 23.0 | 61.5 | 75.0 | 1.82e-13 |
| APZ78795.1 | nonribosomal\_peptide\_synthetase | BGC0001427 | NRP:Cyclic depsipeptide+Polyketide:Iterative type I polyketide | 22.0 | 87.9 | 75.0 | 1.9e-13 |
| APZ78846.1 | nonribosomal\_peptide\_synthetase | BGC0001431 | NRP:Cyclic depsipeptide+Polyketide:Iterative type I polyketide | 25.0 | 59.8 | 75.0 | 1.9e-13 |
| ATU31794.1 | NRPS | BGC0001814 | NRP | 25.0 | 56.8 | 75.0 | 1.9e-13 |
| AEA30273.1 | peptide\_synthetase | BGC0000429 | Polyketide+NRP:Cyclic depsipeptide | 25.0 | 84.0 | 75.0 | 1.9e-13 |
| ATW47208.1 | non-ribosomal\_peptide\_synthetase | BGC0002466 | NRP | 25.0 | 59.3 | 75.0 | 1.92e-13 |
| ABL74939.1 | NRPS | BGC0001048 | NRP:Glycopeptide+Polyketide:Modular type I polyketide+Saccharide:Hybrid/tailoring saccharide | 26.0 | 59.5 | 75.0 | 1.97e-13 |
| FIS9431\_RS32925 | non-ribosomal\_peptide\_synthetase | BGC0001467 | NRP:Cyclic depsipeptide+Polyketide:Modular type I polyketide | 24.0 | 55.3 | 75.0 | 2.14e-13 |
| ABC39418.1 | dihydroaeruginoic\_acid\_synthetase | BGC0000964 | NRP:Cyclic depsipeptide+Polyketide:Trans-AT type I polyketide | 29.0 | 40.7 | 75.0 | 2.2e-13 |
| QPI18727.1 | nonribosomal\_peptide\_synthetase | BGC0002125 | NRP:Cyclic depsipeptide | 25.0 | 90.7 | 75.0 | 2.41e-13 |
| CAO98843.1 | hypothetical\_protein | BGC0000023 | Polyketide:Modular type I polyketide | 25.0 | 64.6 | 74.0 | 2.44e-13 |
| AGI87382.1 | Peptide\_synthase | BGC0002358 | Polyketide | 25.0 | 83.2 | 75.0 | 2.44e-13 |
| RSO11554.1 | non-ribosomal\_peptide\_synthetase | BGC0002637 | NRP | 25.0 | 73.3 | 75.0 | 2.51e-13 |
| AIE77058.1 | peptide\_synthetase\_module\_3 | BGC0000418 | NRP | 23.0 | 85.7 | 74.0 | 2.52e-13 |
| CDG17980.1 | Putative\_Ornithine\_racemase\_(fragment) | BGC0000464 | NRP:Cyclic depsipeptide | 22.0 | 84.3 | 75.0 | 2.57e-13 |
| CAJ77716.1 | Mps2\_protein | BGC0000364 | NRP | 25.0 | 57.6 | 74.0 | 3.11e-13 |
| AAT28740.1 | FUSS | BGC0000064 | NRP+Polyketide | 26.0 | 58.9 | 74.0 | 3.23e-13 |
| AGO86662.1 | equisetin\_synthetase | BGC0001255 | NRP+Polyketide | 22.0 | 86.2 | 74.0 | 3.25e-13 |
| QYA95681.1 | amino\_acid\_adenylation\_domain-containing\_protein | BGC0002676 | NRP | 25.0 | 84.0 | 74.0 | 3.29e-13 |
| BAW32332.1 | nonribosomal\_peptide\_synthetase | BGC0001631 | NRP+Polyketide | 24.0 | 71.6 | 74.0 | 3.6e-13 |
| BAH22765.1 | nonribosomal\_peptide\_synthetase | BGC0001018 | NRP | 23.0 | 61.8 | 74.0 | 3.63e-13 |
| CAG15025.1 | Acyl-CoA\_synthase | BGC0000441 | NRP | 26.0 | 50.0 | 73.0 | 4.06e-13 |
| CAE53367.1 | acyl-CoA\_ligase | BGC0000440 | NRP:Glycopeptide | 26.0 | 50.0 | 73.0 | 4.08e-13 |
| ADH04679.1 | non-ribosomal\_peptide\_synthetase | BGC0001344 | NRP+Polyketide | 24.0 | 75.3 | 74.0 | 4.09e-13 |
| WP\_028678148.1 | non-ribosomal\_peptide\_synthetase | BGC0001228 | NRP:Cyclic depsipeptide | 26.0 | 52.3 | 74.0 | 4.18e-13 |
| AGN74892.1 | nonribosomal\_peptide\_synthetase/polyketide\_synthase\_hybrid\_protein | BGC0000459 | NRP:Cyclic depsipeptide+Polyketide:Trans-AT type I polyketide | 25.0 | 57.1 | 74.0 | 4.31e-13 |
| AKA59447.1 | non-ribosomal\_peptide\_synthetase | BGC0001203 | NRP+Polyketide | 26.0 | 49.1 | 74.0 | 4.32e-13 |
| AGI89789.1 | Nonribosomal\_peptide\_synthetase | BGC0001792 | NRP | 26.0 | 59.5 | 74.0 | 4.43e-13 |
| ABL74936.1 | NRPS | BGC0001048 | NRP:Glycopeptide+Polyketide:Modular type I polyketide+Saccharide:Hybrid/tailoring saccharide | 27.0 | 60.7 | 74.0 | 5.22e-13 |
| BAX64247.1 | NRPS | BGC0001623 | NRP+Polyketide | 24.0 | 84.5 | 74.0 | 5.33e-13 |
| UHJ79953.1 | non-ribosomal\_peptide\_synthetase | BGC0002654 | NRP | 25.0 | 58.2 | 74.0 | 5.49e-13 |
| AGM16414.1 | paenibacterin\_synthetase\_C | BGC0000400 | NRP | 23.0 | 57.9 | 74.0 | 5.5e-13 |
| AHB82062.1 | polyketide\_synthase | BGC0001231 | NRP+Polyketide:Modular type I polyketide | 25.0 | 63.0 | 74.0 | 5.55e-13 |
| AGA37269.1 | NRPS | BGC0000819 | NRP+Alkaloid | 26.0 | 44.3 | 73.0 | 7.01e-13 |
| AFK57215.1 | DidD | BGC0000985 | Polyketide+NRP:Cyclic depsipeptide | 28.0 | 55.7 | 73.0 | 7.35e-13 |
| AEF16021.1 | acyl-CoA\_synthase | BGC0000379 | NRP | 25.0 | 63.4 | 72.0 | 9.53e-13 |
| AAF17280.1 | nosC | BGC0001028 | Polyketide+NRP:Cyclic depsipeptide | 22.0 | 60.2 | 73.0 | 9.53e-13 |
| AFP73394.1 | FusA | BGC0001268 | NRP+Polyketide | 25.0 | 60.1 | 73.0 | 9.64e-13 |
| AGM16413.1 | paenibacterin\_synthetase\_B | BGC0000400 | NRP | 23.0 | 57.9 | 73.0 | 9.97e-13 |
| CAJ77696.1 | MPS2\_protein | BGC0000363 | NRP | 25.0 | 58.4 | 72.0 | 1.22e-12 |
| BAE98155.1 | putative\_non-ribosomal\_peptide\_synthetase | BGC0000339 | NRP | 26.0 | 47.8 | 72.0 | 1.22e-12 |
| AHH53507.1 | non-ribosomal\_peptide\_synthetase | BGC0000439 | NRP:Lipopeptide:Ca+-dependent lipopeptide | 27.0 | 57.5 | 72.0 | 1.32e-12 |
| WP\_141576286.1 | non-ribosomal\_peptide\_synthetase | BGC0002686 | NRP | 27.0 | 57.8 | 72.0 | 1.34e-12 |
| AOA33121.1 | Nonribosomal\_peptide\_synthetase | BGC0001346 | NRP:Cyclic depsipeptide | 28.0 | 56.8 | 72.0 | 1.56e-12 |
| EAU38971.1 | PKS-NRPS\_hybrid | BGC0001122 | NRP+Polyketide:Iterative type I polyketide | 26.0 | 44.1 | 72.0 | 1.67e-12 |
| CAG29032.1 | nonribosomal\_peptide\_synthetase\_(modules\_3\_to\_6) | BGC0001023 | NRP+Polyketide:Modular type I polyketide | 23.0 | 85.1 | 72.0 | 1.69e-12 |
| APZ78822.1 | nonribosomal\_peptide\_synthetase | BGC0001429 | NRP:Cyclic depsipeptide+Polyketide:Iterative type I polyketide | 23.0 | 85.1 | 72.0 | 1.69e-12 |
| NAO96320.1 | amino\_acid\_adenylation\_domain-containing\_protein | BGC0002117 | NRP | 24.0 | 56.1 | 72.0 | 1.69e-12 |
| CAH55654.1 | putative\_L-prolyl-AMP\_ligase | BGC0000259 | Polyketide | 26.0 | 58.2 | 71.0 | 1.74e-12 |
| CCA89326.1 | mixed\_trans-AT\_type\_I\_polyketide\_synthase/nonribosomal\_peptide\_synthetase | BGC0001111 | NRP+Polyketide:Trans-AT type I polyketide | 27.0 | 51.4 | 72.0 | 1.76e-12 |
| QCE43603.1 | nonribosomal\_peptide\_synthetase\_(NRPS),\_subunit\_2 | BGC0001834 | NRP | 24.0 | 89.8 | 72.0 | 2.25e-12 |
| EFG10344.1 | Non-ribosomal\_peptide\_synthetase | BGC0000373 | NRP | 24.0 | 60.6 | 71.0 | 2.31e-12 |
| QBA57736.1 | NRPS | BGC0002377 | NRP | 26.0 | 57.8 | 71.0 | 2.77e-12 |
| WP\_024483797.1 | non-ribosomal\_peptide\_synthetase | BGC0002002 | NRP | 24.0 | 88.2 | 71.0 | 2.82e-12 |
| WP\_012408783.1 | non-ribosomal\_peptide\_synthetase | BGC0002061 | NRP:Cyclic depsipeptide+Polyketide:Modular type I polyketide | 23.0 | 61.6 | 71.0 | 2.91e-12 |
| AAZ03554.1 | McnE | BGC0000332 | NRP | 22.0 | 62.0 | 71.0 | 3.27e-12 |
| AQZ71347.1 | hypothetical\_protein | BGC0001635 | NRP+Polyketide | 28.0 | 57.9 | 71.0 | 3.32e-12 |
| ARU08069.1 | mlcG | BGC0001448 | NRP:Lipopeptide:Ca+-dependent lipopeptide | 24.0 | 64.4 | 70.0 | 3.71e-12 |
| CAJ34375.1 | NRPS | BGC0000445 | NRP:Cyclic depsipeptide | 25.0 | 66.1 | 71.0 | 3.72e-12 |
| AAF15891.2 | nosA | BGC0001028 | Polyketide+NRP:Cyclic depsipeptide | 22.0 | 61.5 | 71.0 | 3.82e-12 |
| ARR97036.1 | SphC | BGC0001780 | NRP | 25.0 | 56.4 | 71.0 | 3.87e-12 |
| BBC83957.1 | nonribosomal\_peptide\_synthetase | BGC0001636 | NRP | 24.0 | 51.9 | 71.0 | 3.99e-12 |
| MBD2892727.1 | Phenyloxazoline\_synthase\_MbtB | BGC0002718 | NRP | 27.0 | 57.5 | 71.0 | 4.03e-12 |
| ARR97038.1 | SphE | BGC0001780 | NRP | 25.0 | 56.4 | 71.0 | 4.8e-12 |
| AHZ20784.1 | non-ribosomal\_peptide\_synthase | BGC0000369 | NRP+Saccharide:Hybrid/tailoring saccharide | 23.0 | 49.7 | 71.0 | 4.84e-12 |
| OKA09664.1 | non-ribosomal\_peptide\_synthetase | BGC0001459 | NRP:Glycopeptide | 28.0 | 47.0 | 70.0 | 4.86e-12 |
| ABC36450.1 | peptide\_synthetase-like\_protein | BGC0000386 | NRP:NRP siderophore | 26.0 | 59.8 | 71.0 | 4.91e-12 |
| MBD2892722.1 | D-alanine--D-alanyl\_carrier\_protein\_ligase | BGC0002718 | NRP | 29.0 | 56.2 | 71.0 | 4.99e-12 |
| QED88054.1 | nonribosomal\_peptide\_synthetase | BGC0001967 | NRP+Polyketide | 24.0 | 56.8 | 71.0 | 5.04e-12 |
| AAC82550.1 | FxbC | BGC0000351 | NRP | 26.0 | 58.5 | 71.0 | 5.06e-12 |
| ABX37382.1 | amino\_acid\_adenylation\_domain\_protein | BGC0000984 | NRP+Polyketide | 25.0 | 57.6 | 71.0 | 5.12e-12 |
| QNH67550.1 | Cip22 | BGC0002108 | NRP | 25.0 | 55.9 | 71.0 | 5.15e-12 |
| QSJ20135.1 | non-ribosomal\_peptide\_synthase/polyketide\_synthase | BGC0002572 | NRP+Polyketide | 24.0 | 60.7 | 71.0 | 5.24e-12 |
| AAC83657.1 | pyochelin\_synthetase | BGC0000412 | NRP | 22.0 | 65.1 | 70.0 | 5.96e-12 |
| QDQ83033.1 | amino\_acid\_adenylation\_domain-containing\_protein | BGC0002564 | NRP | 23.0 | 57.8 | 70.0 | 5.98e-12 |
| QEO75077.1 | condensation\_domain-containing\_protein | BGC0002079 | NRP:Cyclic depsipeptide | 27.0 | 57.3 | 70.0 | 6.19e-12 |
| BAI63283.1 | putative\_non-ribosomal\_peptide\_synthetase | BGC0000434 | NRP | 25.0 | 64.8 | 69.0 | 6.42e-12 |
| QWT72279.1 | non-ribosomal\_peptide\_synthetase | BGC0002430 | NRP+Saccharide | 26.0 | 55.6 | 70.0 | 6.81e-12 |
| ABV56587.1 | KtzG | BGC0000378 | NRP | 25.0 | 61.0 | 70.0 | 7.24e-12 |
| AFO85453.1 | non-ribosomal\_peptide\_synthetase | BGC0000391 | NRP | 27.0 | 48.1 | 70.0 | 8.36e-12 |
| SDF67417.1 | amino\_acid\_adenylation\_domain-containing\_protein | BGC0002422 | NRP | 24.0 | 83.2 | 70.0 | 8.4e-12 |
| ABW17377.1 | PsoC | BGC0000411 | NRP | 25.0 | 57.0 | 70.0 | 8.57e-12 |
| BAX90000.1 | Non-ribosomal\_peptide\_synthetase | BGC0001628 | NRP | 22.0 | 85.9 | 70.0 | 8.58e-12 |
| AAX31557.1 | peptide\_synthetase\_1 | BGC0000336 | NRP | 26.0 | 59.6 | 70.0 | 8.81e-12 |
| QRD93053.1 | putative\_nonribosomal\_peptide\_synthase | BGC0002160 | NRP | 27.0 | 63.4 | 70.0 | 8.92e-12 |
| PHM26613.1 | pyoverdine\_synthetase\_D | BGC0001130 | NRP+Polyketide | 25.0 | 60.6 | 69.0 | 1.07e-11 |
| CBL93718.1 | NRPS\_didomain\_PCP-C | BGC0000360 | NRP | 25.0 | 57.0 | 69.0 | 1.41e-11 |
| WP\_069848004.1 | non-ribosomal\_peptide\_synthetase | BGC0002472 | NRP | 24.0 | 45.7 | 69.0 | 1.74e-11 |
| AAO56106.1 | yersiniabactin\_non-ribosomal\_peptide\_synthetase | BGC0002570 | NRP+Polyketide | 26.0 | 49.7 | 69.0 | 1.82e-11 |
| PLB34720.1 | polyketide\_synthase | BGC0002749 | NRP+Polyketide | 24.0 | 59.3 | 69.0 | 1.87e-11 |
| AAU34203.1 | mannopeptimycin\_peptide\_synthetase\_MppB | BGC0000388 | NRP | 26.0 | 56.7 | 69.0 | 1.94e-11 |
| WP\_054234643.1 | non-ribosomal\_peptide\_synthetase | BGC0002014 | NRP+Polyketide | 23.0 | 84.6 | 69.0 | 1.94e-11 |
| QKW60392.1 | amino\_acid\_adenylation\_domain-containing\_protein | BGC0002288 | NRP | 25.0 | 56.5 | 68.0 | 2.07e-11 |
| ABD65965.1 | acyl-CoA\_ligase/dehydrogenase\_fusion\_protein | BGC0000341 | NRP | 27.0 | 46.6 | 68.0 | 2.12e-11 |
| CBG70279.1 | thaxtomin\_synthetase\_A | BGC0002089 | NRP | 24.0 | 75.9 | 68.0 | 2.24e-11 |
| AAX31559.1 | peptide\_synthetase\_3 | BGC0000336 | NRP | 23.0 | 83.1 | 68.0 | 2.44e-11 |
| CAJ77695.1 | MPS1\_protein | BGC0000363 | NRP | 24.0 | 83.9 | 68.0 | 2.53e-11 |
| QGZ36672.1 | amino\_acid\_adenylation\_domain-containing\_protein | BGC0002082 | NRP+Polyketide | 23.0 | 83.5 | 68.0 | 2.64e-11 |
| AHN85651.1 | Phn2 | BGC0000122 | Polyketide:Modular type I polyketide | 25.0 | 62.1 | 68.0 | 2.65e-11 |
| CAE16560.1 |  | BGC0000196 | Polyketide:Type II polyketide | 21.0 | 57.1 | 67.0 | 3.03e-11 |
| OLZ52442.1 | non-ribosomal\_peptide\_synthetase | BGC0001462 | NRP:Glycopeptide | 27.0 | 59.2 | 67.0 | 3.38e-11 |
| ARF06222.1 | non-ribosomal\_peptide\_synthetase | BGC0001593 | NRP | 23.0 | 87.9 | 67.0 | 3.83e-11 |
| ALV82388.1 | CDA\_peptide\_synthetase\_III | BGC0001370 | NRP | 24.0 | 55.9 | 67.0 | 4.21e-11 |
| AEI58879.1 | peptide\_synthetase | BGC0000455 | NRP | 26.0 | 59.6 | 67.0 | 4.34e-11 |
| CBG67537.1 | putative\_NRPS-associated\_AMP-binding\_protein | BGC0002367 | NRP | 25.0 | 65.4 | 67.0 | 4.37e-11 |
| QUF98525.1 | non-ribosomal\_peptide\_synthetase | BGC0002582 | NRP | 26.0 | 58.1 | 67.0 | 4.69e-11 |
| BAX89998.1 | Non-ribosomal\_peptide\_synthetase | BGC0001628 | NRP | 24.0 | 59.3 | 67.0 | 5.58e-11 |
| BAI63289.1 | putative\_non-ribosomal\_peptide\_synthetase | BGC0000434 | NRP | 25.0 | 50.9 | 67.0 | 5.7e-11 |
| QEO74905.1 | condensation\_domain-containing\_protein | BGC0002588 | Other | 24.0 | 72.7 | 67.0 | 5.76e-11 |
| QBK15049.1 | PKS-NRPS\_hybrid\_TraA | BGC0002197 | Polyketide+NRP | 23.0 | 71.1 | 67.0 | 5.79e-11 |
| QEO74981.1 | omn6 | BGC0002078 | NRP:Cyclic depsipeptide | 26.0 | 57.8 | 67.0 | 5.85e-11 |
| ANZ15840.1 | non-ribosomal\_peptide\_synthase/amino\_acid\_adenylation\_enzyme | BGC0001569 | NRP | 24.0 | 85.1 | 67.0 | 5.87e-11 |
| EKJ70673.1 | hypothetical\_protein | BGC0002188 | NRP+Polyketide | 23.0 | 60.9 | 67.0 | 6.04e-11 |
| CAF32362.1 | putative\_non-ribosomal\_peptide\_synthetase | BGC0000712 | Saccharide | 25.0 | 58.1 | 67.0 | 6.23e-11 |
| AAK89731.2 | siderophore\_biosynthesis\_protein | BGC0002107 | NRP+Polyketide | 24.0 | 57.5 | 67.0 | 6.27e-11 |
| AAT01807.1 | non-ribosomal\_peptide\_synthetase | BGC0000365 | NRP | 23.0 | 83.5 | 67.0 | 7.33e-11 |
| AZC86156.1 | peramine\_synthetase | BGC0002166 | NRP | 25.0 | 57.0 | 67.0 | 7.4e-11 |
| QPI18726.1 | nonribosomal\_peptide\_synthetase | BGC0002125 | NRP:Cyclic depsipeptide | 24.0 | 58.5 | 66.0 | 8e-11 |
| DAC80524.1 | peptide\_synthetase | BGC0001841 | NRP+Polyketide | 27.0 | 46.0 | 66.0 | 8.5e-11 |
| QPI18723.1 | nonribosomal\_peptide\_synthetase | BGC0002125 | NRP:Cyclic depsipeptide | 24.0 | 58.5 | 66.0 | 8.52e-11 |
| OJJ98497.1 | hypothetical\_protein | BGC0002169 | Polyketide+NRP | 24.0 | 90.2 | 66.0 | 8.92e-11 |
| AAM80536.1 | StaD | BGC0000290 | NRP:Glycopeptide | 25.0 | 87.6 | 66.0 | 9.21e-11 |
| AKQ52531.1 | nonribosomal\_peptide\_synthetase | BGC0002533 | NRP+Polyketide | 25.0 | 43.8 | 66.0 | 9.28e-11 |
| ATY69568.1 | adenylation\_protein | BGC0001611 | NRP+Polyketide | 24.0 | 60.9 | 66.0 | 9.7e-11 |
| QPI18729.1 | nonribosomal\_peptide\_synthetase | BGC0002125 | NRP:Cyclic depsipeptide | 24.0 | 58.5 | 66.0 | 9.83e-11 |
| KJY94240.1 | peptide\_synthetase | BGC0002691 | NRP | 21.0 | 59.5 | 66.0 | 9.95e-11 |
| AFJ23825.1 | WLIP\_synthetase\_B | BGC0001838 | NRP | 23.0 | 90.7 | 66.0 | 1.01e-10 |
| ALK27914.1 | non-ribosomal\_peptide\_synthase | BGC0001233 | NRP | 24.0 | 59.3 | 66.0 | 1.02e-10 |
| AAW03330.1 | CtaG | BGC0000982 | NRP+Polyketide | 27.0 | 42.9 | 66.0 | 1.2e-10 |
| KGA48739.1 | amino\_acid\_adenylation\_domain\_protein | BGC0002413 | NRP | 24.0 | 46.4 | 66.0 | 1.26e-10 |
| QOJ72663.1 | XenE | BGC0002505 | Polyketide+NRP | 25.0 | 58.9 | 66.0 | 1.32e-10 |
| CBZ42146.1 | putative\_non-ribosomal\_peptide\_synthetase | BGC0001117 | NRP | 25.0 | 48.4 | 66.0 | 1.34e-10 |
| CRG85572.1 | nonribosomal\_peptide\_synthase,\_putative | BGC0001402 | NRP | 24.0 | 59.6 | 66.0 | 1.34e-10 |
| QDJ74273.1 | non-ribosomal\_peptide\_synthetase | BGC0002109 | NRP | 28.0 | 49.8 | 66.0 | 1.34e-10 |
| BBQ09587.1 | PKS-NRPS\_hybrid | BGC0002261 | Polyketide | 25.0 | 54.0 | 66.0 | 1.73e-10 |
| EME52974.1 | non-ribosomal\_peptide\_synthetase | BGC0001460 | NRP:Glycopeptide | 27.0 | 47.8 | 65.0 | 1.78e-10 |
| CAC48369.1 | peptide\_synthetase | BGC0000311 | NRP | 26.0 | 56.7 | 65.0 | 1.79e-10 |
| CAD89778.1 | MelG\_protein | BGC0001010 | NRP+Polyketide:Modular type I polyketide | 27.0 | 42.9 | 65.0 | 2.07e-10 |
| CAD55498.1 | CDA\_peptide\_synthetase\_III\_(CdaPs3) | BGC0000315 | NRP:Lipopeptide:Ca+-dependent lipopeptide | 25.0 | 60.1 | 65.0 | 2.17e-10 |
| ACS68554.1 | hybrid\_PKS-NRPS\_protein | BGC0001026 | NRP+Polyketide | 24.0 | 58.7 | 65.0 | 2.27e-10 |
| QTT72098.1 | non-ribosomal\_peptide\_synthetase | BGC0002350 | NRP+Polyketide+Saccharide | 24.0 | 57.9 | 65.0 | 2.51e-10 |
| ACO94492.1 | NRPS\_adenylation\_domain\_protein | BGC0000097 | Polyketide:Modular type I polyketide | 25.0 | 49.8 | 64.0 | 2.76e-10 |
| ACY06285.1 | non-ribosomal\_peptide\_synthetase | BGC0001042 | NRP+Polyketide | 23.0 | 56.4 | 65.0 | 2.96e-10 |
| AIG79224.1 | Non-ribosomal\_peptide\_synthetase/andenylation\_domain | BGC0000419 | Saccharide+NRP:Glycopeptide | 26.0 | 59.2 | 64.0 | 3.1e-10 |
| AAF63833.1 | PstD | BGC0000362 | NRP | 26.0 | 47.0 | 64.0 | 3.43e-10 |
| KJY85279.1 | long-chain\_fatty\_acid--CoA\_ligase | BGC0002491 | NRP | 25.0 | 57.1 | 64.0 | 3.45e-10 |
| BAP16697.1 | nonribosomal\_peptide\_synthetase | BGC0000376 | NRP | 25.0 | 59.2 | 64.0 | 3.46e-10 |
| TRX17523.1 | non-ribosomal\_peptide\_synthetase | BGC0002329 | NRP | 25.0 | 48.1 | 64.0 | 3.63e-10 |
| QXJ21807.1 | amino\_acid\_adenylation\_domain-containing\_protein | BGC0002370 | NRP | 25.0 | 46.6 | 64.0 | 3.73e-10 |
| BBC43184.1 | PKS-NRPS\_hybrid | BGC0001738 | NRP+Polyketide | 25.0 | 41.8 | 64.0 | 3.91e-10 |
| ESU15173.1 | hypothetical\_protein | BGC0002186 | NRP+Polyketide | 24.0 | 49.5 | 64.0 | 5.18e-10 |
| ACZ66258.1 | APS1 | BGC0000304 | NRP | 22.0 | 91.1 | 64.0 | 5.22e-10 |
| AAO39110.1 | AdmP | BGC0000956 | NRP:Beta-lactam+Polyketide:Type II polyketide | 23.0 | 57.3 | 63.0 | 5.38e-10 |
| ACO94464.1 | NRPS\_adenylation\_domain\_protein | BGC0000029 | Polyketide:Modular type I polyketide | 26.0 | 52.0 | 63.0 | 6.37e-10 |
| QJY30853.1 | PKS-NRPS\_hybrid\_protein | BGC0002539 | Alkaloid | 27.0 | 46.9 | 64.0 | 6.71e-10 |
| ABD65957.1 | nonribosomal\_peptide\_synthetase | BGC0000341 | NRP | 25.0 | 58.7 | 64.0 | 6.94e-10 |
| OLZ50899.1 | non-ribosomal\_peptide\_synthetase | BGC0001461 | NRP:Glycopeptide | 26.0 | 59.2 | 63.0 | 7.13e-10 |
| AYA22318.1 | KerE | BGC0001955 | NRP | 26.0 | 59.2 | 63.0 | 7.13e-10 |
| AAG02358.1 | peptide\_synthetase\_NRPS6 | BGC0000963 | NRP:Glycopeptide+Polyketide:Modular type I polyketide+Saccharide:Hybrid/tailoring saccharide | 25.0 | 58.2 | 63.0 | 7.53e-10 |
| APZ78744.1 | nonribosomal\_peptide\_synthetase | BGC0001422 | NRP:Cyclic depsipeptide+Polyketide:Iterative type I polyketide | 25.0 | 59.3 | 63.0 | 8.93e-10 |
| AMM63162.1 | AniA | BGC0001371 | NRP | 23.0 | 63.2 | 63.0 | 9.13e-10 |
| ATQ39428.1 | cyclosporin\_C\_synthetase | BGC0001565 | NRP | 24.0 | 48.1 | 63.0 | 9.3e-10 |
| AIE77076.1 | peptide\_synthetase | BGC0000418 | NRP | 26.0 | 49.1 | 62.0 | 9.41e-10 |
| AFK57213.1 | DidB | BGC0000985 | Polyketide+NRP:Cyclic depsipeptide | 25.0 | 64.0 | 63.0 | 1.07e-09 |
| QBL56187.1 | long-chain-fatty-acid-CoA\_ligase | BGC0002376 | Polyketide | 25.0 | 60.9 | 62.0 | 1.13e-09 |
| AGI89791.1 | Nonribosomal\_peptide\_synthetase | BGC0001792 | NRP | 24.0 | 57.5 | 63.0 | 1.19e-09 |
| ABK39646.1 | nonribosomal\_peptide\_synthetase | BGC0001502 | NRP | 24.0 | 81.2 | 62.0 | 1.24e-09 |
| QEO75074.1 | condensation\_domain-containing\_protein | BGC0002079 | NRP:Cyclic depsipeptide | 27.0 | 58.1 | 62.0 | 1.32e-09 |
| BAE06845.2 | peramine\_synthetase | BGC0002164 | NRP | 26.0 | 46.6 | 62.0 | 1.49e-09 |
| CAJ77715.1 | Mps1\_protein | BGC0000364 | NRP | 25.0 | 57.8 | 62.0 | 1.51e-09 |
| AHB38497.1 | non-ribosomal\_peptide\_synthetase | BGC0000346 | NRP+Polyketide:Modular type I polyketide | 25.0 | 64.9 | 62.0 | 1.55e-09 |
| AGM16412.1 | paenibacterin\_synthetase\_A | BGC0000400 | NRP | 24.0 | 59.0 | 62.0 | 1.57e-09 |
| AKJ70942.1 | non-ribosomal\_peptide\_synthetase | BGC0002611 | NRP | 22.0 | 89.4 | 62.0 | 2.07e-09 |
| AXF14775.1 | non-ribosomal\_peptide\_synthetase | BGC0002563 | NRP | 24.0 | 72.7 | 62.0 | 2.45e-09 |
| QIW91877.1 | NRPS | BGC0002543 | NRP | 24.0 | 66.8 | 62.0 | 2.71e-09 |
| QCC62999.1 | BII-rafflesfungin\_nonribosomal\_protein\_synthetase | BGC0001966 | NRP+Polyketide | 24.0 | 74.4 | 62.0 | 2.74e-09 |
| AQZ42163.1 | putative\_nonribosomal\_peptide\_synthase | BGC0001820 | NRP | 25.0 | 59.0 | 61.0 | 3.24e-09 |
| AQM58286.1 | non-ribosomal\_peptide\_synthase | BGC0001816 | NRP+Polyketide | 25.0 | 39.9 | 61.0 | 3.35e-09 |
| CDN62030.1 | Peptide\_synthetase | BGC0001599 | NRP | 25.0 | 59.0 | 61.0 | 3.87e-09 |
| NKI69295.1 | amino\_acid\_adenylation\_domain-containing\_protein | BGC0002408 | NRP | 22.0 | 57.9 | 61.0 | 4.48e-09 |
| BCJ07529.1 | hypothetical\_protein | BGC0002379 | NRP | 25.0 | 59.2 | 61.0 | 4.5e-09 |
| AJD47484.1 | protein\_PvdD | BGC0002418 | NRP+Polyketide | 25.0 | 57.0 | 61.0 | 4.54e-09 |
| BAO66530.1 | nonribosomal\_peptide\_synthase | BGC0000042 | Polyketide | 25.0 | 56.7 | 60.0 | 4.56e-09 |
| AEP18655.1 | WAPS2 | BGC0000461 | NRP | 25.0 | 59.3 | 61.0 | 4.68e-09 |
| CAH55637.1 | putative\_L-prolyl-AMP\_ligase | BGC0000258 | Polyketide | 28.0 | 46.4 | 60.0 | 5.76e-09 |
| XP\_003044554.1 | uncharacterized\_protein | BGC0001768 | NRP | 26.0 | 64.1 | 61.0 | 6.09e-09 |
| EFL06867.1 | predicted\_protein | BGC0000300 | NRP | 24.0 | 60.4 | 60.0 | 7.49e-09 |
| AWI62628.1 | nonribosomal\_peptide\_synthetase | BGC0001822 | NRP | 24.0 | 58.4 | 60.0 | 7.74e-09 |
| CBF76038.1 | nonribosomal\_peptide\_synthase,\_putative\_(Eurofung) | BGC0001399 | NRP | 28.0 | 48.8 | 60.0 | 9.21e-09 |
| AAD44234.1 | PstB | BGC0000362 | NRP | 24.0 | 57.6 | 60.0 | 9.93e-09 |
| AVI26390.1 | polyketide\_synthase\_/\_nonribosomal\_peptide\_synthase\_hybrid | BGC0001800 | NRP+Polyketide | 22.0 | 58.4 | 60.0 | 1.04e-08 |
| CCE28989.1 | non-ribosomal\_peptide\_synthetase | BGC0001365 | NRP | 26.0 | 59.9 | 59.0 | 1.27e-08 |
| CBF87069.1 | nonribosomal\_peptide\_synthase,\_putative\_(Eurofung) | BGC0001290 | NRP | 26.0 | 38.4 | 59.0 | 1.39e-08 |
| SDF67478.1 | Phosphopantetheine\_attachment\_site | BGC0002422 | NRP | 24.0 | 57.6 | 59.0 | 1.49e-08 |
| SAI82904.1 | HrnL;\_NRPS\_adenylation\_domain;\_AMP-binding\_enzyme;\_Long-chain\_fatty\_acid\_CoA\_ligase;\_Pfam00501 | BGC0002101 | Polyketide | 25.0 | 49.8 | 58.0 | 1.79e-08 |
| ABP55216.1 | AMP-dependent\_synthetase\_and\_ligase | BGC0000142 | Polyketide | 26.0 | 53.7 | 58.0 | 1.82e-08 |
| CCB53264.1 | non-ribosomal\_peptide\_synthetase | BGC0001393 | NRP | 21.0 | 60.7 | 58.0 | 2.93e-08 |
| MAA\_10043 | non-ribosomal\_peptide\_synthetase | BGC0000337 | NRP | 26.0 | 64.3 | 58.0 | 3.15e-08 |
| XP\_020058100.1 | uncharacterized\_protein | BGC0001220 | NRP | 28.0 | 34.5 | 58.0 | 4.12e-08 |
| BAR73011.1 | putative\_ATP-dependent\_aminoacyl-ACP\_synthetase | BGC0001194 | Polyketide | 25.0 | 52.5 | 57.0 | 4.2e-08 |
| AAO39104.1 | AdmJ | BGC0000956 | NRP:Beta-lactam+Polyketide:Type II polyketide | 25.0 | 44.9 | 57.0 | 4.33e-08 |
| EHA55860.1 | polyketide\_synthase/peptide\_synthetase | BGC0002235 | Polyketide+NRP | 25.0 | 42.7 | 57.0 | 5.27e-08 |
| AAZ23075.1 | peptide\_synthetase | BGC0000291 | NRP | 26.0 | 59.0 | 57.0 | 5.38e-08 |
| BBU42014.1 | e-poly-L-lysine\_synthetase | BGC0002174 | NRP | 24.0 | 55.9 | 57.0 | 6.08e-08 |
| AHD05615.1 | putative\_non-ribosomal\_peptide\_ligase/\_polyketide\_synthase\_hybrid | BGC0001033 | NRP+Polyketide | 24.0 | 59.6 | 57.0 | 6.78e-08 |
| QEO74983.1 | omn8 | BGC0002078 | NRP:Cyclic depsipeptide | 26.0 | 57.6 | 57.0 | 8.69e-08 |
| AHZ34242.1 | CipE | BGC0001389 | NRP | 22.0 | 58.1 | 56.0 | 1.17e-07 |
| QYA95682.1 | amino\_acid\_adenylation\_domain-containing\_protein | BGC0002676 | NRP | 23.0 | 58.9 | 56.0 | 1.2e-07 |
| ALA09366.1 | AMP-dependent\_synthetase\_and\_ligase | BGC0001303 | Polyketide | 25.0 | 53.0 | 56.0 | 1.27e-07 |
| BBB04327.1 | nonribosomal\_peptide\_synthetase | BGC0001717 | NRP | 25.0 | 40.8 | 56.0 | 1.47e-07 |
| BGRAMDRAFT\_RS22640 | amino\_acid\_adenylation\_domain-containing\_protein | BGC0001999 | NRP | 24.0 | 47.2 | 56.0 | 1.8e-07 |
| AET98905.1 | putative\_non-ribosomal\_peptide\_synthetase | BGC0000415 | NRP | 26.0 | 65.5 | 56.0 | 2.02e-07 |
| EAT91803.2 | hypothetical\_protein | BGC0002205 | Polyketide+NRP | 21.0 | 41.3 | 56.0 | 2.05e-07 |
| AEA30272.1 | peptide\_synthetase | BGC0000429 | Polyketide+NRP:Cyclic depsipeptide | 23.0 | 58.9 | 56.0 | 2.07e-07 |
| AKD43757.1 | HerL | BGC0001349 | NRP+Polyketide | 24.0 | 52.0 | 54.0 | 2.88e-07 |
| CCP42826.1 | Probable\_peptide\_synthetase\_Nrp\_(peptide\_synthase) | BGC0001627 | NRP | 23.0 | 56.1 | 55.0 | 3.42e-07 |
| AHZ20773.1 | non-ribosomal\_peptide\_synthase | BGC0000369 | NRP+Saccharide:Hybrid/tailoring saccharide | 23.0 | 37.6 | 55.0 | 3.47e-07 |
| ctg1\_orf00001 |  | BGC0000901 | Other | 21.0 | 73.9 | 55.0 | 3.49e-07 |
| ORC16618.1 | hypothetical\_protein | BGC0001341 | NRP | 24.0 | 47.7 | 55.0 | 3.5e-07 |
| EGX96627.1 | non-ribosomal\_peptide\_synthase,\_putative | BGC0002259 | Polyketide+NRP | 24.0 | 63.4 | 55.0 | 3.57e-07 |
| EPE34341.1 | non-ribosomal\_peptide\_synthetase | BGC0001035 | Polyketide+NRP | 25.0 | 48.0 | 55.0 | 3.62e-07 |
| AHB82069.1 | non\_ribosomal\_peptide\_synthetase | BGC0001231 | NRP+Polyketide:Modular type I polyketide | 23.0 | 58.5 | 54.0 | 4.43e-07 |
| ALK27915.1 | non-ribosomal\_peptide\_synthase | BGC0001233 | NRP | 27.0 | 34.9 | 54.0 | 4.77e-07 |
| ACJ04424.1 | aureobasidin\_A1\_biosynthesis\_complex | BGC0000307 | NRP | 24.0 | 53.0 | 53.0 | 1.09e-06 |
| QGA70084.1 | ATP-dependent\_ligase | BGC0002517 | Polyketide | 25.0 | 46.0 | 52.0 | 1.17e-06 |
| UHH90022.1 | VicM | BGC0002634 | Polyketide+NRP+Other | 25.0 | 49.5 | 52.0 | 1.55e-06 |
| ctg1\_orf000000 |  | BGC0000901 | Other | 22.0 | 65.2 | 52.0 | 1.78e-06 |
| KFA69336.1 | hypothetical\_protein | BGC0001626 | Polyketide | 29.0 | 30.0 | 52.0 | 2.04e-06 |
| CZT62785.1 | Non-ribosomal\_peptide\_synthase,\_involved\_in\_Hassallidin\_biosynthesis | BGC0001614 | NRP | 21.0 | 59.3 | 52.0 | 2.33e-06 |
| BAE98156.1 | putative\_non-ribosomal\_peptide\_synthetase | BGC0000339 | NRP | 24.0 | 63.4 | 52.0 | 2.34e-06 |
| ctg3\_34 |  | BGC0001853 | NRP+Polyketide:Modular type I polyketide | 22.0 | 65.1 | 51.0 | 2.61e-06 |
| BAV56002.1 | ATP-dependent\_ligase | BGC0001597 | Polyketide | 24.0 | 58.2 | 51.0 | 2.64e-06 |
| CAD91221.1 | putative\_non-ribosomal\_peptide\_synthetase,\_module\_3 | BGC0000289 | NRP:Glycopeptide+Saccharide:Hybrid/tailoring saccharide | 25.0 | 30.3 | 52.0 | 2.67e-06 |
| CBF76036.1 | putative\_nonribosomal\_peptide\_synthetase\_(Eurofung) | BGC0001399 | NRP | 22.0 | 34.0 | 52.0 | 3e-06 |
| BAD08370.1 | non-ribosomal\_peptide\_synthetase | BGC0000167 | Polyketide | 23.0 | 58.4 | 50.0 | 4.71e-06 |
| AET13879.1 | epichloenin\_A\_synthetase | BGC0001251 | NRP | 25.0 | 65.5 | 51.0 | 5.4e-06 |
| AAY37647.1 | Amino\_acid\_adenylation | BGC0000437 | NRP | 23.0 | 57.8 | 51.0 | 5.51e-06 |
| MCC5026027.1 | amino\_acid\_adenylation\_domain-containing\_protein | BGC0002118 | NRP+Polyketide | 26.0 | 34.8 | 50.0 | 8.68e-06 |
| AJK49765.1 | non-ribosomal\_peptide\_synthase | BGC0002565 | NRP | 25.0 | 59.8 | 50.0 | 8.73e-06 |
| MCC5025983.1 | amino\_acid\_adenylation\_domain-containing\_protein | BGC0002119 | NRP+Polyketide | 26.0 | 34.8 | 50.0 | 8.94e-06 |
