# Supplementary material for: Uncovering the transcriptional landscape of Fomes fomentarius during fungal-based material production through gene co-expression network analysis
Source: Fungal Biol Biotechnol. 2025 Feb 13;12:1. doi: 10.1186/s40694-024-00192-3 (PMC11827164; doi:10.1186/s40694-024-00192-3)
Supplement: Supplementary file 1 — Supplementary Material 1 [file 40694_2024_192_MOESM1_ESM.zip › knownclusterblast/region2/jgi.p_Fomfom1_1287699_mibig_hits.html]

| MIBiG Protein | Description | MIBiG Cluster | MiBiG Product | % ID | % Coverage | BLAST Score | E-value |
| --- | --- | --- | --- | --- | --- | --- | --- |
| APZ78746.1 | ATP-dependent\_RNA\_helicase\_RhlE | BGC0001422 | NRP:Cyclic depsipeptide+Polyketide:Iterative type I polyketide | 32.0 | 45.9 | 128.0 | 8.83e-31 |
| AQM37586.1 | ATP-dependent\_RNA\_helicase\_RhlE | BGC0001424 | NRP:Cyclic depsipeptide+Polyketide:Iterative type I polyketide | 32.0 | 44.7 | 127.0 | 1.42e-30 |
| APZ78797.1 | ATP-dependent\_RNA\_helicase\_RhlE | BGC0001427 | NRP:Cyclic depsipeptide+Polyketide:Iterative type I polyketide | 32.0 | 45.9 | 126.0 | 3.69e-30 |
| APZ78811.1 | ATP-dependent\_RNA\_helicase\_RhlE | BGC0001428 | NRP:Cyclic depsipeptide+Polyketide:Iterative type I polyketide | 32.0 | 45.9 | 125.0 | 4.07e-30 |
| APZ78771.1 | ATP-dependent\_RNA\_helicase\_RhlE | BGC0001425 | NRP:Cyclic depsipeptide+Polyketide:Iterative type I polyketide | 32.0 | 45.9 | 125.0 | 4.35e-30 |
| APZ78784.1 | ATP-dependent\_RNA\_helicase\_RhlE | BGC0001426 | NRP:Cyclic depsipeptide+Polyketide:Iterative type I polyketide | 32.0 | 45.9 | 125.0 | 4.35e-30 |
| APZ78731.1 | ATP-dependent\_RNA\_helicase\_RhlE | BGC0001421 | NRP:Cyclic depsipeptide+Polyketide:Iterative type I polyketide | 31.0 | 45.9 | 125.0 | 4.64e-30 |
| APZ78758.1 | ATP-dependent\_RNA\_helicase\_RhlE | BGC0001423 | NRP:Cyclic depsipeptide+Polyketide:Iterative type I polyketide | 31.0 | 45.9 | 124.0 | 1.07e-29 |
| AAY39327.1 | Helicase,\_C-terminal:DEAD/DEAH\_box\_helicase,\_N-terminal | BGC0002060 | Polyketide:Trans-AT type I polyketide | 30.0 | 45.0 | 117.0 | 1.87e-27 |
| ACG60739.1 | probable\_DEAD-box\_RNA\_helicase | BGC0001058 | NRP:Glycopeptide+Polyketide:Modular type I polyketide+Saccharide:Hybrid/tailoring saccharide | 28.0 | 54.7 | 106.0 | 8.61e-24 |
| AAK53492.1 | putative\_ATP-dependent\_RNA\_helicase | BGC0000774 | Saccharide:Lipopolysaccharide | 30.0 | 31.2 | 81.0 | 4.29e-17 |
