# Supplementary material for: Uncovering the transcriptional landscape of Fomes fomentarius during fungal-based material production through gene co-expression network analysis
Source: Fungal Biol Biotechnol. 2025 Feb 13;12:1. doi: 10.1186/s40694-024-00192-3 (PMC11827164; doi:10.1186/s40694-024-00192-3)
Supplement: Supplementary file 1 — Supplementary Material 1 [file 40694_2024_192_MOESM1_ESM.zip › knownclusterblast/region2/jgi.p_Fomfom1_1205933_mibig_hits.html]

| MIBiG Protein | Description | MIBiG Cluster | MiBiG Product | % ID | % Coverage | BLAST Score | E-value |
| --- | --- | --- | --- | --- | --- | --- | --- |
| AAK57184.1 | MxaA | BGC0001022 | NRP+Polyketide | 29.0 | 100.7 | 479.0 | 4.23e-144 |
| BAY02139.1 | nonribosomal\_protein\_synthetase | BGC0002532 | NRP+Polyketide | 29.0 | 100.1 | 460.0 | 3.8e-137 |
| AGC45618.1 | non-ribosomal\_peptide\_synthetase | BGC0001394 | NRP+Polyketide | 29.0 | 99.8 | 445.0 | 5.45e-132 |
| AHJ31215.1 | Long-chain-fatty-acid--CoA\_ligase | BGC0000430 | NRP+Polyketide:Modular type I polyketide | 28.0 | 101.6 | 449.0 | 1.44e-130 |
| AQW44894.1 | non-ribosomal\_peptide\_synthetase | BGC0001737 | NRP+Polyketide | 29.0 | 95.2 | 437.0 | 3.23e-129 |
| AAT12283.1 | LtxA | BGC0000384 | NRP | 29.0 | 100.3 | 438.0 | 6.5e-127 |
| ATD51278.1 | nonribosomal\_peptide\_synthase | BGC0001650 | NRP | 29.0 | 95.5 | 400.0 | 2.31e-114 |
| BAO84866.1 | putative\_non-ribosomal\_peptide\_synthetase | BGC0000414 | NRP | 27.0 | 101.6 | 374.0 | 2.38e-107 |
| ATD51280.1 | nonribosomal\_peptide\_synthase | BGC0001650 | NRP | 28.0 | 98.3 | 374.0 | 4.38e-107 |
| AGD80618.1 | non-ribosomal\_peptide\_synthetase | BGC0000394 | NRP | 30.0 | 77.4 | 372.0 | 1.44e-106 |
| BAP27942.1 | nonribosomal\_peptide\_synthetase | BGC0001085 | NRP+Terpene | 30.0 | 77.6 | 369.0 | 2.74e-104 |
| EOY45602.1 | Adenylation\_and\_reductase\_domains\_containing\_protein | BGC0001168 | NRP | 29.0 | 77.8 | 350.0 | 5.74e-102 |
| AOC89001.1 | putative\_nonribosomal\_peptide\_synthetase | BGC0001652 | NRP | 28.0 | 98.0 | 357.0 | 2.14e-101 |
| QUF98525.1 | non-ribosomal\_peptide\_synthetase | BGC0002582 | NRP | 30.0 | 80.8 | 343.0 | 6.96e-99 |
| AAK89727.1 | peptide\_synthetase,\_siderophore\_biosynthesis\_protein | BGC0002107 | NRP+Polyketide | 28.0 | 95.0 | 349.0 | 1.21e-98 |
| CAF32362.1 | putative\_non-ribosomal\_peptide\_synthetase | BGC0000712 | Saccharide | 30.0 | 81.7 | 341.0 | 7.79e-98 |
| CDN62030.1 | Peptide\_synthetase | BGC0001599 | NRP | 29.0 | 79.7 | 342.0 | 1.3e-97 |
| ABI26078.1 | OciB | BGC0000331 | NRP | 29.0 | 68.7 | 345.0 | 1.59e-96 |
| ATJ04411.1 | NRPS,\_TomB\_binding | BGC0001637 | NRP | 27.0 | 73.6 | 337.0 | 7.37e-95 |
| AFO85453.1 | non-ribosomal\_peptide\_synthetase | BGC0000391 | NRP | 31.0 | 68.8 | 334.0 | 6.23e-93 |
| ABI22133.1 | putative\_non-ribosomal\_peptide\_synthetase | BGC0000422 | NRP | 29.0 | 79.2 | 331.0 | 9.82e-93 |
| BAH22764.1 | nonribosomal\_peptide\_synthetase | BGC0001018 | NRP | 29.0 | 68.3 | 331.0 | 5.91e-92 |
| CDG76959.1 | non-ribosomal\_peptide\_synthetase,\_terminal\_component | BGC0000446 | NRP:Pyrrolobenzodiazepine | 28.0 | 73.5 | 328.0 | 6.35e-92 |
| CBG67541.1 | putative\_non-ribosomal\_peptide\_synthetase | BGC0002367 | NRP | 30.0 | 72.3 | 325.0 | 5.77e-90 |
| ABY83163.1 | Azi25 | BGC0000960 | NRP+Polyketide | 28.0 | 89.2 | 321.0 | 1.88e-89 |
| CAJ77696.1 | MPS2\_protein | BGC0000363 | NRP | 27.0 | 84.8 | 322.0 | 3.72e-89 |
| AAZ03552.1 | McnC | BGC0000332 | NRP | 29.0 | 68.5 | 322.0 | 3.97e-89 |
| AAO23334.1 | NcpB | BGC0000397 | NRP | 29.0 | 70.0 | 322.0 | 4e-89 |
| CAJ77716.1 | Mps2\_protein | BGC0000364 | NRP | 30.0 | 67.4 | 320.0 | 2.03e-88 |
| CAB15186.3 | siderophore\_2,3-dihydroxybenzoate-glycine-threonine\_trimeric\_ester\_bacillibactin\_synthetase | BGC0000309 | NRP | 29.0 | 70.1 | 318.0 | 5.8e-88 |
| AAO23333.1 | NcpA | BGC0000397 | NRP | 28.0 | 69.5 | 318.0 | 6.07e-88 |
| AAF17280.1 | nosC | BGC0001028 | Polyketide+NRP:Cyclic depsipeptide | 29.0 | 70.9 | 318.0 | 7.55e-88 |
| BAY02137.1 | amino\_acid\_adenylation\_domain-containing\_protein | BGC0002532 | NRP+Polyketide | 30.0 | 64.8 | 316.0 | 2.66e-87 |
| WP\_012408786.1 | non-ribosomal\_peptide\_synthetase | BGC0002061 | NRP:Cyclic depsipeptide+Polyketide:Modular type I polyketide | 29.0 | 69.5 | 315.0 | 7.83e-87 |
| AFY58523.1 | amino\_acid\_adenylation\_enzyme/thioester\_reductase\_family\_protein | BGC0002411 | NRP+Polyketide | 29.0 | 69.3 | 312.0 | 5.71e-86 |
| WP\_068925909.1 | non-ribosomal\_peptide\_synthetase | BGC0002688 | NRP | 30.0 | 68.8 | 311.0 | 9.29e-86 |
| ABS75232.1 | DhbF | BGC0001185 | NRP:NRP siderophore | 29.0 | 68.7 | 310.0 | 1.63e-85 |
| AAF17281.1 | nosD | BGC0001028 | Polyketide+NRP:Cyclic depsipeptide | 29.0 | 69.0 | 310.0 | 2.95e-85 |
| AKC91849.1 | nonribosomal\_peptide\_synthetase | BGC0001414 | NRP | 29.0 | 68.8 | 308.0 | 1.15e-84 |
| ATL73036.1 | amino\_acid\_adenylation\_protein | BGC0001807 | NRP+Polyketide | 29.0 | 68.7 | 308.0 | 1.45e-84 |
| EAA62478.1 | hypothetical\_protein | BGC0002276 | NRP | 29.0 | 68.4 | 303.0 | 3.92e-84 |
| BAH22765.1 | nonribosomal\_peptide\_synthetase | BGC0001018 | NRP | 28.0 | 68.3 | 303.0 | 8.81e-84 |
| MBV7329455.1 | amino\_acid\_adenylation\_domain-containing\_protein | BGC0002131 | Polyketide+NRP:Glycopeptide+Saccharide:Hybrid/tailoring saccharide | 29.0 | 69.7 | 305.0 | 9.08e-84 |
| AKC91856.1 | nonribosomal\_peptide\_synthetase | BGC0001414 | NRP | 30.0 | 68.4 | 304.0 | 2.12e-83 |
| AAZ03554.1 | McnE | BGC0000332 | NRP | 28.0 | 68.2 | 301.0 | 2.49e-83 |
| MBE8994631.1 | amino\_acid\_adenylation\_domain-containing\_protein | BGC0002623 | NRP+Polyketide | 28.0 | 68.2 | 297.0 | 7.3e-83 |
| ACN39015.1 | putative\_nonribosomal\_peptide\_synthetase\_TomB | BGC0000448 | NRP | 30.0 | 73.2 | 300.0 | 9.71e-83 |
| AEI70245.1 | nonribosomal\_peptide\_synthetase\_NRPS | BGC0000401 | NRP | 30.0 | 69.7 | 301.0 | 1.04e-82 |
| AAD44234.1 | PstB | BGC0000362 | NRP | 28.0 | 83.0 | 301.0 | 1.12e-82 |
| AEU11006.1 | NpnB | BGC0001029 | NRP+Polyketide | 28.0 | 63.6 | 302.0 | 1.2e-82 |
| MCF2151708.1 | Non-ribosomal\_peptide\_synthetase | BGC0002625 | NRP+Polyketide | 28.0 | 68.2 | 302.0 | 1.31e-82 |
| WP\_012408785.1 | non-ribosomal\_peptide\_synthetase | BGC0002061 | NRP:Cyclic depsipeptide+Polyketide:Modular type I polyketide | 29.0 | 69.9 | 301.0 | 1.46e-82 |
| BAH22763.1 | nonribosomal\_peptide\_synthetase | BGC0001018 | NRP | 27.0 | 64.8 | 295.0 | 2.05e-82 |
| KYC42613.1 | non-ribosomal\_peptide\_synthetase | BGC0002484 | NRP+Polyketide | 29.0 | 64.2 | 300.0 | 3.74e-82 |
| OAQ83772.1 | nonribosomal\_peptide\_synthase | BGC0001358 | NRP+Polyketide | 29.0 | 69.3 | 300.0 | 4.4e-82 |
| ABI26079.1 | OciC | BGC0000331 | NRP | 27.0 | 68.5 | 297.0 | 5.8e-82 |
| WP\_041754829.1 | non-ribosomal\_peptide\_synthetase | BGC0001844 | NRP:Lipopeptide | 28.0 | 68.0 | 300.0 | 7.01e-82 |
| AAF15891.2 | nosA | BGC0001028 | Polyketide+NRP:Cyclic depsipeptide | 28.0 | 69.5 | 299.0 | 8.23e-82 |
| QBG38783.1 | Atr22 | BGC0001975 | NRP | 29.0 | 68.6 | 299.0 | 1.01e-81 |
| CAB38518.1 | CDA\_peptide\_synthetase\_I\_(CdaPs1) | BGC0000315 | NRP:Lipopeptide:Ca+-dependent lipopeptide | 30.0 | 70.9 | 299.0 | 1.26e-81 |
| WP\_012408783.1 | non-ribosomal\_peptide\_synthetase | BGC0002061 | NRP:Cyclic depsipeptide+Polyketide:Modular type I polyketide | 28.0 | 69.0 | 298.0 | 1.44e-81 |
| MBE8994632.1 | amino\_acid\_adenylation\_domain-containing\_protein | BGC0002623 | NRP+Polyketide | 28.0 | 69.3 | 298.0 | 1.8e-81 |
| ABI26077.1 | OciA | BGC0000331 | NRP | 27.0 | 69.1 | 297.0 | 3.01e-81 |
| CBW75453.1 | Non-ribosomal\_peptide\_synthetase\_modules\_(EC\_6.3.2.-) | BGC0002048 | NRP:Cyclic depsipeptide | 28.0 | 68.8 | 296.0 | 6.18e-81 |
| AKJ15828.1 | peptide\_synthetase | BGC0002735 | Polyketide+NRP | 31.0 | 68.6 | 292.0 | 1.2e-80 |
| QSJ20135.1 | non-ribosomal\_peptide\_synthase/polyketide\_synthase | BGC0002572 | NRP+Polyketide | 26.0 | 64.3 | 295.0 | 2.22e-80 |
| QNH67551.1 | Cip23 | BGC0002108 | NRP | 30.0 | 69.5 | 295.0 | 2.43e-80 |
| QNH85840.1 | BolH | BGC0002327 | NRP | 27.0 | 69.0 | 294.0 | 3.16e-80 |
| ADG27358.1 | peptide\_synthetase | BGC0000296 | NRP | 31.0 | 63.5 | 293.0 | 4.07e-80 |
| AAZ03551.1 | McnB | BGC0000332 | NRP | 27.0 | 64.8 | 288.0 | 5.55e-80 |
| AGI87384.1 | Peptide\_synthase | BGC0002358 | Polyketide | 28.0 | 63.2 | 293.0 | 7.05e-80 |
| AIW82277.1 | PuwA | BGC0001125 | NRP+Polyketide | 28.0 | 68.2 | 293.0 | 7.65e-80 |
| AXN93575.1 | PuwA | BGC0001950 | NRP | 28.0 | 68.0 | 293.0 | 7.65e-80 |
| AXN93584.1 | PuwA | BGC0001951 | NRP | 28.0 | 68.0 | 293.0 | 7.65e-80 |
| AJM89738.1 | PmxE | BGC0001192 | NRP | 27.0 | 70.6 | 293.0 | 8.02e-80 |
| BAW32323.1 | hybrid\_cis-AT\_polyketide\_synthase\_-\_nonribosomal\_peptide\_synthetase | BGC0001630 | NRP+Polyketide | 27.0 | 64.6 | 293.0 | 8.86e-80 |
| QBG38784.1 | Atr23 | BGC0001975 | NRP | 31.0 | 65.8 | 292.0 | 1.3e-79 |
| CZT62794.1 | Non-ribosomal\_peptide\_synthase\_involved\_in\_Hassallidin\_biosynthesis | BGC0001614 | NRP | 28.0 | 69.5 | 291.0 | 1.75e-79 |
| BCD33690.1 | non-ribosomal\_peptide\_synthetase | BGC0002448 | NRP | 29.0 | 69.3 | 292.0 | 1.84e-79 |
| QNN94286.1 | EmeB | BGC0002555 | NRP+Polyketide | 29.0 | 67.6 | 292.0 | 2.14e-79 |
| AXN93592.1 | PuwA | BGC0001952 | NRP | 28.0 | 68.6 | 291.0 | 2.33e-79 |
| ACA97580.1 | PmxE | BGC0000408 | NRP | 27.0 | 70.1 | 291.0 | 3.24e-79 |
| CBW75452.1 | Non-ribosomal\_peptide\_synthetase\_modules\_(EC\_6.3.2.-) | BGC0002048 | NRP:Cyclic depsipeptide | 28.0 | 67.7 | 290.0 | 3.43e-79 |
| AWI62628.1 | nonribosomal\_peptide\_synthetase | BGC0001822 | NRP | 31.0 | 63.7 | 290.0 | 4.47e-79 |
| NPC94426.1 | amino\_acid\_adenylation\_domain-containing\_protein | BGC0002695 | NRP | 28.0 | 68.8 | 290.0 | 6.1e-79 |
| WP\_126241403.1 | non-ribosomal\_peptide\_synthetase | BGC0002336 | NRP | 27.0 | 68.3 | 289.0 | 1.19e-78 |
| AYJ71720.1 | non-ribosomal\_peptide\_synthetase | BGC0001942 | NRP+Polyketide | 29.0 | 69.7 | 288.0 | 1.25e-78 |
| QDA77045.1 | polyketide\_synthase/nonribosomal\_peptide\_synthetase | BGC0002025 | NRP+Polyketide | 27.0 | 68.0 | 289.0 | 1.34e-78 |
| AXN93603.1 | PuwH | BGC0001952 | NRP | 28.0 | 69.7 | 284.0 | 1.65e-78 |
| AXN93616.1 | PuwH | BGC0001953 | NRP | 28.0 | 69.6 | 284.0 | 1.65e-78 |
| BAY02138.1 | peptide\_synthetase | BGC0002532 | NRP+Polyketide | 28.0 | 64.0 | 283.0 | 1.72e-78 |
| AEZ51520.1 | pmxE | BGC0001153 | NRP:Lipopeptide | 27.0 | 69.3 | 289.0 | 1.72e-78 |
| ABF87031.1 | non-ribosomal\_peptide\_synthetase/polyketide\_synthase | BGC0000393 | NRP+Polyketide:Modular type I polyketide | 28.0 | 68.6 | 289.0 | 1.94e-78 |
| AAL33758.1 | putative\_non-ribosomal\_peptide\_synthetase | BGC0000421 | NRP | 26.0 | 78.2 | 286.0 | 2.39e-78 |
| AQH32486.1 | peptide\_synthetase | BGC0001667 | NRP+Polyketide | 26.0 | 67.6 | 285.0 | 3.51e-78 |
| CAJ14039.1 | peptide\_synthetase | BGC0000406 | NRP | 30.0 | 70.0 | 282.0 | 3.69e-78 |
| QED88055.1 | nonribosomal\_peptide\_synthetase | BGC0001967 | NRP+Polyketide | 29.0 | 69.5 | 287.0 | 4.3e-78 |
| WP\_003981346.1 | non-ribosomal\_peptide\_synthetase | BGC0001813 | NRP | 28.0 | 68.8 | 286.0 | 9.93e-78 |
| CAD29799.1 | microcystin\_synthetase | BGC0001015 | NRP+Polyketide | 26.0 | 68.1 | 283.0 | 1.21e-77 |
| QGA70148.1 | nonribosomal\_peptide\_synthetase | BGC0002293 | NRP | 29.0 | 68.3 | 286.0 | 1.79e-77 |
| AEH59099.1 | amino\_acid\_adenylation\_domain-containing\_protein/NRPS | BGC0000385 | NRP | 28.0 | 67.9 | 285.0 | 2.37e-77 |
| EPH46598.1 | putative\_Dimodular\_nonribosomal\_peptide\_synthase | BGC0001519 | NRP+Polyketide | 28.0 | 68.6 | 282.0 | 2.47e-77 |
| AXN93605.1 | PuwA | BGC0001953 | NRP | 28.0 | 68.4 | 285.0 | 2.66e-77 |
| AXN93602.1 | PuwF-G | BGC0001952 | NRP | 28.0 | 64.2 | 285.0 | 2.73e-77 |
| QDA77059.1 | polyketide\_synthase/nonribosomal\_peptide\_synthetase | BGC0002026 | NRP+Polyketide | 29.0 | 69.3 | 285.0 | 3e-77 |
| QYA95681.1 | amino\_acid\_adenylation\_domain-containing\_protein | BGC0002676 | NRP | 29.0 | 68.1 | 285.0 | 3.39e-77 |
| AGI87382.1 | Peptide\_synthase | BGC0002358 | Polyketide | 28.0 | 67.2 | 284.0 | 5.16e-77 |
| WA1\_15570 | hypothetical\_protein | BGC0002484 | NRP+Polyketide | 27.0 | 65.5 | 283.0 | 6.86e-77 |
| AEH41794.1 | HrmP | BGC0000374 | NRP:Cyclic depsipeptide | 29.0 | 70.2 | 283.0 | 6.88e-77 |
| AGP37410.1 | peptide\_synthetase | BGC0002386 | NRP+Polyketide | 29.0 | 70.6 | 283.0 | 7.11e-77 |
| AHD05679.1 | putative\_non-ribosomal\_peptide\_ligase/\_polyketide\_synthase\_hybrid | BGC0000402 | NRP | 29.0 | 63.2 | 283.0 | 7.33e-77 |
| KYC42612.1 | hypothetical\_protein | BGC0002484 | NRP+Polyketide | 27.0 | 69.7 | 283.0 | 8.27e-77 |
| AIW82282.1 | PuwE | BGC0001125 | NRP+Polyketide | 28.0 | 64.1 | 282.0 | 1.48e-76 |
| ABQ96384.2 | fusaricidin\_synthetase | BGC0001152 | Polyketide+NRP:Lipopeptide | 28.0 | 68.5 | 283.0 | 1.54e-76 |
| QSJ20140.1 | amino\_acid\_adenylation\_domain-containing\_protein | BGC0002572 | NRP+Polyketide | 29.0 | 63.3 | 280.0 | 2.6e-76 |
| AIW82283.1 | PuwF | BGC0001125 | NRP+Polyketide | 29.0 | 68.9 | 281.0 | 2.79e-76 |
| BBA21073.1 | putative\_non-ribosomal\_peptide\_synthetase | BGC0001740 | NRP+Polyketide | 29.0 | 63.6 | 281.0 | 2.79e-76 |
| AAU34202.1 | mannopeptimycin\_peptide\_synthetase\_MppA | BGC0000388 | NRP | 28.0 | 67.2 | 281.0 | 3.16e-76 |
| AXN93582.1 | PuwH | BGC0001950 | NRP | 29.0 | 65.3 | 276.0 | 3.78e-76 |
| AXN93591.1 | PuwH | BGC0001951 | NRP | 29.0 | 65.3 | 276.0 | 4.68e-76 |
| AEA30273.1 | peptide\_synthetase | BGC0000429 | Polyketide+NRP:Cyclic depsipeptide | 29.0 | 68.3 | 281.0 | 5.46e-76 |
| AFH75330.1 | nonribosomal\_peptide\_synthetase | BGC0000398 | NRP:Cyclic depsipeptide | 28.0 | 67.9 | 281.0 | 5.52e-76 |
| XP\_002373813.1 | NRPS-like\_enzyme,\_putative | BGC0001516 | NRP | 27.0 | 73.9 | 275.0 | 6.24e-76 |
| AEH59100.1 | amino\_acid\_adenylation\_domain-containing\_protein/NRPS | BGC0000385 | NRP | 28.0 | 68.3 | 281.0 | 6.26e-76 |
| BAH43870.1 | putative\_linear\_pentadecapeptide\_gramicidin\_synthetase\_LgrB | BGC0000367 | NRP | 28.0 | 64.4 | 280.0 | 8.13e-76 |
| AEU11003.1 | NpnC | BGC0001029 | NRP+Polyketide | 27.0 | 71.5 | 280.0 | 9.72e-76 |
| AZL87944.1 | aspergillic\_acid\_non-ribosomal\_peptide\_sythetase-like\_(NRPS-like)\_(AsaC) | BGC0002602 | Alkaloid | 27.0 | 73.9 | 274.0 | 1.12e-75 |
| ctg1\_orf19 |  | BGC0001013 | NRP+Polyketide | 27.0 | 67.2 | 278.0 | 1.26e-75 |
| ABC87508.1 | NRPS\_for\_pipecolate\_incorporation | BGC0001011 | NRP+Polyketide | 27.0 | 67.2 | 278.0 | 1.3e-75 |
| MCF2150416.1 | Non-ribosomal\_peptide\_synthetase | BGC0002625 | NRP+Polyketide | 28.0 | 68.1 | 280.0 | 1.36e-75 |
| ABM21572.1 | crpD | BGC0000975 | NRP+Polyketide | 27.0 | 67.4 | 279.0 | 1.89e-75 |
| WP\_039806856.1 | non-ribosomal\_peptide\_synthetase | BGC0002001 | NRP+Polyketide | 28.0 | 67.9 | 278.0 | 2.28e-75 |
| CBF73453.1 | nonribosomal\_peptide\_synthase,\_putative\_(JCVI) | BGC0001515 | NRP | 27.0 | 75.6 | 279.0 | 2.43e-75 |
| ACM68690.1 | AerG1 | BGC0000298 | NRP | 27.0 | 63.9 | 274.0 | 2.43e-75 |
| AAO56329.1 | non-ribosomal\_peptide\_synthetase\_SyfB | BGC0000435 | NRP | 28.0 | 67.6 | 278.0 | 4.06e-75 |
| ATY37609.1 | BreD | BGC0001536 | NRP | 27.0 | 68.6 | 278.0 | 4.23e-75 |
| CDG17980.1 | Putative\_Ornithine\_racemase\_(fragment) | BGC0000464 | NRP:Cyclic depsipeptide | 27.0 | 69.7 | 278.0 | 4.28e-75 |
| extra\_gene | NRPS/PKS | BGC0002095 | NRP | 28.0 | 70.0 | 277.0 | 4.6e-75 |
| AIW82285.1 | PuwH | BGC0001125 | NRP+Polyketide | 28.0 | 65.1 | 273.0 | 4.8e-75 |
| WP\_053065269.1 | non-ribosomal\_peptide\_synthetase | BGC0001330 | NRP:Cyclic depsipeptide+Polyketide:Modular type I polyketide | 30.0 | 68.0 | 276.0 | 7.03e-75 |
| QDF82259.1 | non-ribosomal\_peptide\_synthetase | BGC0001980 | NRP | 28.0 | 68.3 | 277.0 | 9.33e-75 |
| ACN39727.1 | SibD | BGC0000428 | NRP | 28.0 | 82.7 | 275.0 | 1.05e-74 |
| AJK49758.1 | non-ribosomal\_peptide\_synthase | BGC0002565 | NRP | 29.0 | 68.6 | 276.0 | 1.19e-74 |
| AHB82059.1 | non\_ribosomal\_peptide\_synthetase/polyketide\_synthase | BGC0001019 | NRP+Polyketide:Modular type I polyketide | 29.0 | 67.6 | 276.0 | 1.21e-74 |
| QRG35014.1 | NRPS | BGC0002378 | NRP | 29.0 | 68.6 | 276.0 | 1.55e-74 |
| AFP87549.1 | NrpS | BGC0001135 | NRP | 27.0 | 68.9 | 276.0 | 1.6e-74 |
| ALK27916.1 | non-ribosomal\_peptide\_synthase | BGC0001233 | NRP | 29.0 | 69.2 | 276.0 | 1.76e-74 |
| ABB90279.1 | non-ribosomal\_peptide\_synthetase | BGC0001057 | NRP+Polyketide | 26.0 | 72.4 | 275.0 | 1.77e-74 |
| QBQ12463.1 | amino\_acid\_adenylation\_domain-containing\_protein | BGC0002693 | NRP | 28.0 | 67.3 | 276.0 | 1.91e-74 |
| AXN93590.1 | PuwF-G | BGC0001951 | NRP | 28.0 | 68.2 | 276.0 | 2.13e-74 |
| QED55421.1 | nonribosomal\_peptide\_synthetase | BGC0001984 | NRP | 27.0 | 68.7 | 275.0 | 2.46e-74 |
| QST87270.1 | amino\_acid\_adenylation\_domain-containing\_protein | BGC0002572 | NRP+Polyketide | 27.0 | 64.2 | 275.0 | 2.99e-74 |
| AGN74885.1 | nonribosomal\_peptide\_synthetase | BGC0000459 | NRP:Cyclic depsipeptide+Polyketide:Trans-AT type I polyketide | 28.0 | 68.8 | 275.0 | 4.87e-74 |
| AIW82284.1 | PuwG | BGC0001125 | NRP+Polyketide | 28.0 | 67.8 | 274.0 | 7.11e-74 |
| QNL14925.1 | AptD | BGC0002512 | NRP | 26.0 | 69.5 | 272.0 | 7.23e-74 |
| WP\_039806852.1 | non-ribosomal\_peptide\_synthetase | BGC0002001 | NRP+Polyketide | 28.0 | 63.0 | 269.0 | 9.22e-74 |
| CAL80824.1 | NRPS\_module\_protein | BGC0000997 | NRP+Polyketide | 32.0 | 50.9 | 269.0 | 9.74e-74 |
| AKJ15826.1 | peptide\_synthetase | BGC0002735 | Polyketide+NRP | 31.0 | 53.1 | 273.0 | 1.03e-73 |
| ALV86866.1 | Tlo20 | BGC0001406 | NRP | 29.0 | 69.1 | 273.0 | 1.4e-73 |
| QBG38782.1 | Atr21 | BGC0001975 | NRP | 30.0 | 62.7 | 273.0 | 1.72e-73 |
| CUX79060.1 | Octapeptin\_synthase\_subunit\_A | BGC0001715 | NRP | 26.0 | 69.0 | 273.0 | 1.75e-73 |
| QPB41097.1 | non-ribosomal\_peptide\_synthetase | BGC0002503 | NRP+Polyketide | 29.0 | 68.4 | 273.0 | 1.82e-73 |
| AFH75322.1 | nonribosomal\_peptide\_synthetase | BGC0000425 | NRP:Cyclic depsipeptide | 28.0 | 67.8 | 273.0 | 2.03e-73 |
| ACC81024.1 | non-ribosomal\_peptide\_synthetase | BGC0001479 | NRP | 26.0 | 69.0 | 270.0 | 2.3e-73 |
| QBC75022.1 | non-ribosomal\_peptide\_synthetase | BGC0001968 | NRP | 29.0 | 69.3 | 272.0 | 2.56e-73 |
| AXN93581.1 | PuwF-G | BGC0001950 | NRP | 28.0 | 68.2 | 272.0 | 2.58e-73 |
| AEC14349.1 | nonribosomal\_peptide\_synthetase | BGC0000377 | NRP | 27.0 | 59.7 | 266.0 | 2.6e-73 |
| MBE3200466.1 | non-ribosomal\_peptide\_synthetase | BGC0002409 | NRP | 28.0 | 64.4 | 272.0 | 3.49e-73 |
| KON97028.1 | phenylalanine\_racemase | BGC0002122 | NRP | 26.0 | 68.0 | 271.0 | 4.13e-73 |
| QRG35013.1 | NRPS | BGC0002378 | NRP | 32.0 | 49.9 | 271.0 | 4.61e-73 |
| ACZ55945.1 | non-ribosomal\_peptide\_synthetase | BGC0000302 | NRP | 31.0 | 46.2 | 271.0 | 4.63e-73 |
| AXN93580.1 | PuwE | BGC0001950 | NRP | 28.0 | 64.1 | 271.0 | 4.65e-73 |
| AXN93589.1 | PuwE | BGC0001951 | NRP | 28.0 | 64.1 | 271.0 | 4.65e-73 |
| QNH67550.1 | Cip22 | BGC0002108 | NRP | 32.0 | 52.6 | 271.0 | 4.68e-73 |
| ANY58984.1 | non-ribosomal\_synthetase | BGC0001615 | NRP | 27.0 | 64.4 | 269.0 | 5.24e-73 |
| ADG27359.1 | peptide\_synthetase | BGC0000296 | NRP | 30.0 | 68.7 | 271.0 | 5.36e-73 |
| AKJ15827.1 | peptide\_synthetase | BGC0002735 | Polyketide+NRP | 28.0 | 63.7 | 271.0 | 5.4e-73 |
| ADN26248.1 | peptide\_synthetase | BGC0000951 | NRP | 27.0 | 67.8 | 263.0 | 5.54e-73 |
| WP\_051700111.1 | non-ribosomal\_peptide\_synthetase | BGC0001368 | NRP | 28.0 | 71.6 | 269.0 | 6.06e-73 |
| ANS62968.1 | actinomycin\_synthetase\_II | BGC0001567 | NRP | 29.0 | 62.9 | 270.0 | 9.28e-73 |
| AGI89790.1 | ATP-dependent\_valine\_adenylase | BGC0001792 | NRP | 29.0 | 71.3 | 270.0 | 9.33e-73 |
| APO47825.1 | non-ribosomal\_peptide\_synthetase | BGC0002653 | NRP | 28.0 | 63.2 | 266.0 | 1.08e-72 |
| AFJ14794.1 | PlpE | BGC0000403 | NRP | 27.0 | 68.7 | 270.0 | 1.45e-72 |
| ABJ97436.1 | MerP | BGC0001012 | NRP+Polyketide | 27.0 | 68.7 | 268.0 | 1.47e-72 |
| APZ78756.1 | nonribosomal\_peptide\_synthetase | BGC0001423 | NRP:Cyclic depsipeptide+Polyketide:Iterative type I polyketide | 30.0 | 63.8 | 270.0 | 1.48e-72 |
| AEW31022.1 | plipastatin\_synthetase | BGC0000407 | NRP | 26.0 | 68.5 | 270.0 | 1.52e-72 |
| AGZ15458.1 | putative\_non-ribosomal\_peptide\_synthetase | BGC0001036 | NRP+Polyketide | 29.0 | 63.2 | 269.0 | 1.63e-72 |
| APZ78834.1 | nonribosomal\_peptide\_synthetase | BGC0001430 | NRP:Cyclic depsipeptide+Polyketide:Iterative type I polyketide | 28.0 | 69.1 | 270.0 | 1.65e-72 |
| WP\_019032755.1 | non-ribosomal\_peptide\_synthetase | BGC0001331 | NRP:Cyclic depsipeptide+Polyketide:Modular type I polyketide | 29.0 | 68.8 | 268.0 | 2.38e-72 |
| AGM16413.1 | paenibacterin\_synthetase\_B | BGC0000400 | NRP | 27.0 | 63.0 | 269.0 | 2.4e-72 |
| AAS98786.1 | nonribosomal\_peptide\_synthetase | BGC0001001 | NRP+Polyketide | 27.0 | 67.9 | 265.0 | 2.49e-72 |
| QBQ12464.1 | amino\_acid\_adenylation\_domain-containing\_protein | BGC0002693 | NRP | 28.0 | 64.7 | 269.0 | 2.66e-72 |
| DAB41484.1 | nonribosomal\_peptide\_synthetase/polyketide\_synthase\_type\_I | BGC0001230 | NRP:Cyclic depsipeptide+Polyketide:Modular type I polyketide | 29.0 | 69.5 | 268.0 | 2.81e-72 |
| ACC81022.1 | non-ribosomal\_peptide\_synthetase | BGC0001479 | NRP | 27.0 | 63.7 | 264.0 | 2.95e-72 |
| CCJ67640.1 | TaaE | BGC0000447 | NRP:Lipopeptide | 27.0 | 68.0 | 269.0 | 3.23e-72 |
| AKC91857.1 | nonribosomal\_peptide\_synthetase | BGC0001414 | NRP | 28.0 | 64.2 | 266.0 | 3.72e-72 |
| AXN93615.1 | PuwG | BGC0001953 | NRP | 30.0 | 46.7 | 268.0 | 4.13e-72 |
| WP\_080679150.1 | non-ribosomal\_peptide\_synthetase | BGC0001228 | NRP:Cyclic depsipeptide | 29.0 | 53.7 | 268.0 | 4.92e-72 |
| ADA69239.2 | trans-AT\_hybrid\_polyketide\_synthase-NRPS | BGC0001071 | NRP+Polyketide:Modular type I polyketide+Polyketide:Trans-AT type I polyketide | 27.0 | 68.9 | 268.0 | 5.76e-72 |
| ACZ55944.1 | non-ribosomal\_peptide\_synthetase | BGC0000302 | NRP | 27.0 | 68.4 | 267.0 | 6.47e-72 |
| AEH41793.1 | HrmO | BGC0000374 | NRP:Cyclic depsipeptide | 29.0 | 69.9 | 268.0 | 6.9e-72 |
| CAD29798.1 | peptide\_synthetase | BGC0001015 | NRP+Polyketide | 26.0 | 64.4 | 267.0 | 7.2e-72 |
| CAJ14037.1 | peptide\_synthetase | BGC0000406 | NRP | 29.0 | 68.3 | 264.0 | 7.35e-72 |
| ACC81021.1 | non-ribosomal\_peptide\_synthetase | BGC0001479 | NRP | 29.0 | 47.9 | 266.0 | 9.68e-72 |
| CCJ67636.1 | TaaA | BGC0000447 | NRP:Lipopeptide | 28.0 | 63.6 | 267.0 | 9.9e-72 |
| AKJ15829.1 | peptide\_synthetase | BGC0002735 | Polyketide+NRP | 27.0 | 68.6 | 264.0 | 1.14e-71 |
| AAU39361.1 | lichenysin\_synthase\_LchAC | BGC0000381 | NRP | 27.0 | 65.2 | 264.0 | 1.26e-71 |
| EWM63002.1 | non-ribosomal\_peptide\_synthetase | BGC0001328 | NRP:Cyclic depsipeptide+Polyketide:Modular type I polyketide | 29.0 | 68.1 | 263.0 | 1.41e-71 |
| WP\_043882190.1 | non-ribosomal\_peptide\_synthetase | BGC0001728 | NRP+Polyketide | 27.0 | 71.0 | 266.0 | 1.69e-71 |
| KPN90376.1 | NupC | BGC0001416 | NRP | 33.0 | 50.5 | 266.0 | 1.79e-71 |
| UKO95756.1 | amino\_acid\_adenylation\_domain-containing\_protein | BGC0002632 | NRP | 30.0 | 47.5 | 265.0 | 1.82e-71 |
| MBD2892722.1 | D-alanine--D-alanyl\_carrier\_protein\_ligase | BGC0002718 | NRP | 28.0 | 68.3 | 266.0 | 1.91e-71 |
| ACC81023.1 | non-ribosomal\_peptide\_synthetase | BGC0001479 | NRP | 25.0 | 67.9 | 265.0 | 2.59e-71 |
| QNH67552.1 | Cip24 | BGC0002108 | NRP | 32.0 | 52.5 | 263.0 | 2.66e-71 |
| CAQ34921.1 | nonribosomal\_peptide\_synthetase | BGC0000986 | NRP+Polyketide | 28.0 | 64.4 | 264.0 | 2.99e-71 |
| CDG17986.1 | Non-ribosomal\_peptide\_synthetase | BGC0000464 | NRP:Cyclic depsipeptide | 28.0 | 69.3 | 265.0 | 3.01e-71 |
| BCD33691.1 | non-ribosomal\_peptide\_synthetase | BGC0002448 | NRP | 31.0 | 48.1 | 263.0 | 3.37e-71 |
| QNL34618.1 | SteC | BGC0002092 | NRP:Cyclic depsipeptide | 27.0 | 68.0 | 265.0 | 3.73e-71 |
| ANZ15839.1 | peptide\_synthetase\_ScpsB | BGC0001569 | NRP | 28.0 | 70.4 | 265.0 | 4.57e-71 |
| ATY37590.1 | BogC | BGC0001532 | NRP | 28.0 | 53.9 | 265.0 | 6.03e-71 |
| QSJ20139.1 | non-ribosomal\_peptide\_synthase/polyketide\_synthase | BGC0002572 | NRP+Polyketide | 27.0 | 64.2 | 265.0 | 6.2e-71 |
| QDJ74273.1 | non-ribosomal\_peptide\_synthetase | BGC0002109 | NRP | 28.0 | 63.0 | 265.0 | 6.4e-71 |
| AGM16412.1 | paenibacterin\_synthetase\_A | BGC0000400 | NRP | 29.0 | 53.1 | 265.0 | 6.62e-71 |
| ACZ55942.1 | non-ribosomal\_peptide\_synthetase | BGC0000302 | NRP | 27.0 | 69.0 | 264.0 | 6.68e-71 |
| AGI89789.1 | Nonribosomal\_peptide\_synthetase | BGC0001792 | NRP | 27.0 | 69.7 | 265.0 | 6.82e-71 |
| AYA44686.1 | icosalide\_NRPS | BGC0001833 | NRP:Lipopeptide | 27.0 | 67.9 | 264.0 | 8.16e-71 |
| ACZ55946.1 | non-ribosomal\_peptide\_synthetase | BGC0000302 | NRP | 26.0 | 68.3 | 262.0 | 8.19e-71 |
| WP\_018540607.1 | non-ribosomal\_peptide\_synthetase | BGC0001332 | NRP+Polyketide | 28.0 | 68.8 | 264.0 | 8.84e-71 |
| ESU17760.1 | hypothetical\_protein | BGC0002172 | NRP | 29.0 | 68.3 | 264.0 | 9.46e-71 |
| APU91750.1 | Non-Ribosomal\_Peptide\_Synthetase | BGC0001806 | NRP | 30.0 | 52.2 | 264.0 | 9.6e-71 |
| APO47822.1 | non-ribosomal\_peptide\_synthetase | BGC0002653 | NRP | 27.0 | 70.2 | 264.0 | 1.14e-70 |
| WP\_013428324.1 | non-ribosomal\_peptide\_synthetase | BGC0001758 | NRP | 31.0 | 47.9 | 264.0 | 1.18e-70 |
| AXM43052.1 | non-ribosomal\_peptide\_synthetase | BGC0001945 | NRP | 28.0 | 68.8 | 263.0 | 1.62e-70 |
| QED55422.1 | nonribosomal\_peptide\_synthetase | BGC0001984 | NRP | 28.0 | 69.3 | 263.0 | 1.76e-70 |
| UKO95748.1 | amino\_acid\_adenylation\_domain-containing\_protein | BGC0002632 | NRP | 27.0 | 69.0 | 263.0 | 1.92e-70 |
| CDG17985.1 | Putative\_Ornithine\_racemase\_(fragment) | BGC0000464 | NRP:Cyclic depsipeptide | 27.0 | 67.7 | 263.0 | 2.03e-70 |
| UEF20591.1 | nonribosomal\_peptide\_synthetase | BGC0002360 | NRP | 28.0 | 67.9 | 261.0 | 2.12e-70 |
| ATP76244.1 | NdaB | BGC0001705 | NRP+Polyketide | 26.0 | 68.3 | 260.0 | 2.14e-70 |
| AAY91420.2 | non-ribosomal\_peptide\_synthetase\_OfaB | BGC0000399 | NRP:Cyclic depsipeptide | 32.0 | 48.1 | 263.0 | 2.35e-70 |
| QLY89263.1 | pseudodesmin\_synthetase | BGC0002522 | NRP | 32.0 | 46.5 | 263.0 | 2.35e-70 |
| AEG64697.1 | LpmC | BGC0000379 | NRP | 27.0 | 68.8 | 263.0 | 2.64e-70 |
| AGU50953.1 | putative\_non-ribosomal\_peptide\_synthetase | BGC0002417 | NRP+Polyketide | 27.0 | 64.3 | 261.0 | 2.66e-70 |
| WP\_144411596.1 | non-ribosomal\_peptide\_synthetase | BGC0002001 | NRP+Polyketide | 28.0 | 67.3 | 260.0 | 2.84e-70 |
| MBE8994630.1 | amino\_acid\_adenylation\_domain-containing\_protein | BGC0002623 | NRP+Polyketide | 27.0 | 63.7 | 262.0 | 3.24e-70 |
| WP\_100939443.1 | non-ribosomal\_peptide\_synthetase | BGC0002071 | NRP:Lipopeptide | 30.0 | 45.9 | 262.0 | 3.38e-70 |
| KYC42747.1 | hypothetical\_protein | BGC0002484 | NRP+Polyketide | 27.0 | 69.2 | 262.0 | 3.47e-70 |
| ABX37383.1 | amino\_acid\_adenylation\_domain\_protein | BGC0000984 | NRP+Polyketide | 29.0 | 69.0 | 262.0 | 3.64e-70 |
| AAY91421.3 | non-ribosomal\_peptide\_synthetase\_OfaC | BGC0000399 | NRP:Cyclic depsipeptide | 32.0 | 45.8 | 262.0 | 4.23e-70 |
| CUX79062.1 | Octapeptin\_synthase\_subunit\_C | BGC0001715 | NRP | 27.0 | 62.5 | 258.0 | 4.25e-70 |
| ACY06285.1 | non-ribosomal\_peptide\_synthetase | BGC0001042 | NRP+Polyketide | 28.0 | 69.0 | 261.0 | 4.99e-70 |
| ASA76632.1 | polyketide\_synthase\_non-ribosomal\_peptide\_synthetase\_hybrid | BGC0001751 | NRP+Polyketide | 28.0 | 68.3 | 261.0 | 5.1e-70 |
| AHZ20784.1 | non-ribosomal\_peptide\_synthase | BGC0000369 | NRP+Saccharide:Hybrid/tailoring saccharide | 28.0 | 68.0 | 261.0 | 5.78e-70 |
| QNL14922.1 | AptA | BGC0002512 | NRP | 29.0 | 45.9 | 261.0 | 6.17e-70 |
| ABS74206.1 | fengycin\_synthetase\_D | BGC0001095 | NRP | 26.0 | 68.2 | 261.0 | 6.55e-70 |
| WP\_054234617.1 | non-ribosomal\_peptide\_synthetase | BGC0002014 | NRP+Polyketide | 29.0 | 67.5 | 261.0 | 6.96e-70 |
| ATU31795.1 | NRPS | BGC0001814 | NRP | 27.0 | 69.9 | 261.0 | 7.28e-70 |
| AAC44128.1 | saframycin\_Mx1\_synthetase\_B | BGC0002706 | NRP | 29.0 | 63.7 | 260.0 | 8.04e-70 |
| AFY58522.1 | non-ribosomal\_peptide\_synthase/amino\_acid\_adenylation\_enzyme | BGC0002411 | NRP+Polyketide | 27.0 | 63.1 | 259.0 | 1.01e-69 |
| WA1\_15565 | non-ribosomal\_peptide\_synthetase | BGC0002484 | NRP+Polyketide | 27.0 | 64.2 | 260.0 | 1.16e-69 |
| AIW58892.1 | non-ribosomal\_peptide\_synthetase | BGC0001582 | NRP | 29.0 | 68.5 | 261.0 | 1.16e-69 |
| QLY89264.1 | pseudodesmin\_synthetase | BGC0002522 | NRP | 28.0 | 68.2 | 260.0 | 1.17e-69 |
| ABV79987.1 | ApnC | BGC0000301 | NRP | 27.0 | 68.5 | 260.0 | 1.23e-69 |
| AGC09528.1 | NRPS | BGC0001183 | Polyketide | 28.0 | 63.2 | 259.0 | 1.85e-69 |
| AJM89734.1 | PmxB | BGC0001192 | NRP | 27.0 | 63.5 | 255.0 | 2.79e-69 |
| CAG29032.1 | nonribosomal\_peptide\_synthetase\_(modules\_3\_to\_6) | BGC0001023 | NRP+Polyketide:Modular type I polyketide | 28.0 | 63.4 | 259.0 | 2.83e-69 |
| APZ78822.1 | nonribosomal\_peptide\_synthetase | BGC0001429 | NRP:Cyclic depsipeptide+Polyketide:Iterative type I polyketide | 28.0 | 63.4 | 259.0 | 2.83e-69 |
| ABS74180.1 | bacillomycin\_D\_synthetase\_B | BGC0001090 | Polyketide+NRP:Lipopeptide | 26.0 | 70.6 | 259.0 | 2.98e-69 |
| ALV82356.1 | CDA\_peptide\_synthetase\_I | BGC0001370 | NRP | 32.0 | 45.7 | 259.0 | 3.19e-69 |
| WP\_030498974.1 | tyrocidine\_synthase\_3 | BGC0001327 | NRP:Cyclic depsipeptide+Polyketide:Modular type I polyketide | 28.0 | 68.1 | 255.0 | 3.28e-69 |
| KYC41483.1 | hypothetical\_protein | BGC0002484 | NRP+Polyketide | 28.0 | 64.9 | 255.0 | 3.41e-69 |
| QBQ12465.1 | amino\_acid\_adenylation\_domain-containing\_protein | BGC0002693 | NRP | 27.0 | 68.1 | 259.0 | 3.62e-69 |
| QWP75305.1 | non-ribosomal\_peptide\_synthase | BGC0002126 | NRP:Cyclic depsipeptide | 30.0 | 48.8 | 259.0 | 4.11e-69 |
| UHJ79953.1 | non-ribosomal\_peptide\_synthetase | BGC0002654 | NRP | 27.0 | 68.8 | 258.0 | 4.23e-69 |
| ATJ34002.1 | non-ribosomal\_peptide\_synthetase | BGC0001442 | NRP | 28.0 | 69.7 | 257.0 | 4.5e-69 |
| AJV88375.1 | MfnC | BGC0001214 | NRP | 29.0 | 63.4 | 258.0 | 4.55e-69 |
| KFL51887.1 | amino\_acid\_adenylation\_protein | BGC0001711 | NRP+Polyketide | 27.0 | 69.5 | 258.0 | 5.58e-69 |
| PHM26613.1 | pyoverdine\_synthetase\_D | BGC0001130 | NRP+Polyketide | 28.0 | 70.0 | 258.0 | 5.79e-69 |
| BAX64247.1 | NRPS | BGC0001623 | NRP+Polyketide | 33.0 | 45.6 | 257.0 | 8.15e-69 |
| AEO14744.1 | NdaB | BGC0000396 | NRP | 26.0 | 68.1 | 255.0 | 8.17e-69 |
| CAY48788.1 | putative\_non-ribosomal\_peptide\_synthetase | BGC0001312 | NRP | 31.0 | 48.4 | 258.0 | 8.39e-69 |
| QWT72292.1 | putative\_non-ribosomal\_peptide\_synthetase | BGC0002430 | NRP+Saccharide | 27.0 | 69.3 | 258.0 | 9.15e-69 |
| CAL17541.1 | peptide\_synthetase,\_putative | BGC0002465 | NRP | 28.0 | 68.5 | 257.0 | 1.03e-68 |
| ACS20362.1 | amino\_acid\_adenylation\_domain\_protein | BGC0002420 | NRP+Polyketide | 28.0 | 67.9 | 256.0 | 1.28e-68 |
| QNL34617.1 | SteB | BGC0002092 | NRP:Cyclic depsipeptide | 31.0 | 48.1 | 257.0 | 1.46e-68 |
| QBA57735.1 | NRPS | BGC0002377 | NRP | 34.0 | 46.1 | 256.0 | 1.47e-68 |
| NAO96320.1 | amino\_acid\_adenylation\_domain-containing\_protein | BGC0002117 | NRP | 27.0 | 68.3 | 257.0 | 1.48e-68 |
| UEF20592.1 | nonribosomal\_peptide\_synthetase | BGC0002360 | NRP | 29.0 | 52.3 | 254.0 | 1.5e-68 |
| ACZ55943.1 | non-ribosomal\_peptide\_synthetase | BGC0000302 | NRP | 27.0 | 63.7 | 253.0 | 1.58e-68 |
| QEO74905.1 | condensation\_domain-containing\_protein | BGC0002588 | Other | 28.0 | 63.7 | 256.0 | 1.78e-68 |
| AJW76711.1 | DsaI | BGC0001196 | NRP | 28.0 | 68.8 | 256.0 | 1.82e-68 |
| BBD17759.1 | non-ribosomal\_peptide\_synthetase | BGC0001919 | NRP+Polyketide | 30.0 | 63.4 | 256.0 | 1.96e-68 |
| ACA97577.1 | PmxB | BGC0000408 | NRP | 27.0 | 63.6 | 253.0 | 2.07e-68 |
| AAY93355.1 | non-ribosomal\_peptide\_synthetase\_PvdJ | BGC0000413 | NRP | 26.0 | 68.4 | 256.0 | 2.22e-68 |
| QCP68976.1 | VatQ | BGC0002296 | NRP+Polyketide | 27.0 | 67.1 | 255.0 | 2.35e-68 |
| QPB41096.1 | non-ribosomal\_peptide\_synthetase | BGC0002503 | NRP+Polyketide | 31.0 | 45.1 | 256.0 | 2.68e-68 |
| AXN93601.1 | PuwE | BGC0001952 | NRP | 26.0 | 63.7 | 256.0 | 2.88e-68 |
| ADZ24999.1 | non-ribosomal\_peptide\_synthase | BGC0000380 | NRP+Polyketide:Modular type I polyketide | 28.0 | 69.7 | 254.0 | 2.98e-68 |
| ABA23460.1 | Amino\_acid\_adenylation | BGC0000427 | NRP | 29.0 | 46.9 | 249.0 | 3.02e-68 |
| QKF54435.2 | nonribosomal\_peptide\_synthetase | BGC0002581 | NRP | 29.0 | 52.3 | 255.0 | 3.39e-68 |
| AEO14743.1 | NdaA | BGC0000396 | NRP | 28.0 | 68.6 | 255.0 | 3.43e-68 |
| AWI62626.1 | nonribosomal\_peptide\_synthetase | BGC0001822 | NRP | 28.0 | 68.4 | 256.0 | 3.46e-68 |
| QEO74904.1 | AMP-dependent\_synthetase\_and\_ligase | BGC0002588 | Other | 32.0 | 46.1 | 255.0 | 3.79e-68 |
| AWN90\_15505 | non-ribosomal\_peptide\_synthetase | BGC0002352 | Other | 28.0 | 63.8 | 254.0 | 4.16e-68 |
| APZ78809.1 | nonribosomal\_peptide\_synthetase | BGC0001428 | NRP:Cyclic depsipeptide+Polyketide:Iterative type I polyketide | 28.0 | 68.1 | 255.0 | 4.43e-68 |
| CUX79061.1 | Octapeptin\_synthase\_subunit\_B | BGC0001715 | NRP | 27.0 | 62.5 | 255.0 | 4.55e-68 |
| QIH29228.1 | endopyrrole\_NRPS\_A | BGC0002326 | NRP | 30.0 | 48.0 | 255.0 | 4.63e-68 |
| QMS47798.1 | JesB | BGC0001629 | NRP:Lipopeptide | 31.0 | 50.5 | 255.0 | 4.69e-68 |
| AXN93614.1 | PuwF | BGC0001953 | NRP | 30.0 | 46.7 | 255.0 | 4.78e-68 |
| EFE73312.1 | nonribosomal\_peptide\_synthetase | BGC0000431 | NRP:Cyclic depsipeptide | 32.0 | 45.8 | 255.0 | 4.81e-68 |
| QDQ83031.1 | amino\_acid\_adenylation\_domain-containing\_protein | BGC0002564 | NRP | 29.0 | 52.1 | 254.0 | 5e-68 |
| BAH22762.1 | nonribosomal\_peptide\_synthetase | BGC0001018 | NRP | 27.0 | 69.2 | 251.0 | 5.27e-68 |
| ALG65319.1 | Cal17 | BGC0001297 | NRP | 32.0 | 51.4 | 254.0 | 5.82e-68 |
| QED55423.1 | nonribosomal\_peptide\_synthetase | BGC0001984 | NRP | 30.0 | 69.3 | 255.0 | 6.51e-68 |
| AXN93613.1 | PuwE | BGC0001953 | NRP | 26.0 | 63.7 | 254.0 | 6.57e-68 |
| AJV88376.1 | MfnD | BGC0001214 | NRP | 29.0 | 63.9 | 253.0 | 6.65e-68 |
| AIG26883.1 | NRPS\_domain-containing\_protein | BGC0002432 | NRP | 25.0 | 68.8 | 254.0 | 7.51e-68 |
| APZ78795.1 | nonribosomal\_peptide\_synthetase | BGC0001427 | NRP:Cyclic depsipeptide+Polyketide:Iterative type I polyketide | 28.0 | 68.1 | 254.0 | 7.67e-68 |
| KPN93065.1 | NunD | BGC0001416 | NRP | 28.0 | 53.2 | 254.0 | 7.91e-68 |
| QBA57737.1 | NRPS | BGC0002377 | NRP | 29.0 | 70.9 | 254.0 | 8.42e-68 |
| AHI59108.1 | locillomycin\_synthase\_A | BGC0001005 | NRP+Polyketide | 27.0 | 68.7 | 254.0 | 9.3e-68 |
| ABS74181.1 | bacillomycin\_D\_synthetase\_A\_ | BGC0001090 | Polyketide+NRP:Lipopeptide | 27.0 | 68.8 | 254.0 | 9.7e-68 |
| BAB69698.1 | iturin\_A\_synthetase\_A | BGC0001098 | NRP+Polyketide | 27.0 | 68.6 | 254.0 | 9.7e-68 |
| BAC67535.1 | arthrofactin\_synthetase\_B | BGC0000305 | NRP:Lipopeptide | 31.0 | 47.9 | 254.0 | 1e-67 |
| APZ78782.1 | nonribosomal\_peptide\_synthetase | BGC0001426 | NRP:Cyclic depsipeptide+Polyketide:Iterative type I polyketide | 28.0 | 68.1 | 254.0 | 1.01e-67 |
| KUM80514.1 | hypothetical\_protein | BGC0001562 | NRP | 31.0 | 45.6 | 249.0 | 1.05e-67 |
| APU91751.1 | Non-Ribosomal\_Peptide\_Synthetase | BGC0001806 | NRP | 27.0 | 67.2 | 254.0 | 1.11e-67 |
| BAV57443.1 | NRPS\_(C-A-PCP-TE) | BGC0001818 | NRP | 29.0 | 68.3 | 252.0 | 1.12e-67 |
| CCJ67639.1 | TaaD | BGC0000447 | NRP:Lipopeptide | 31.0 | 47.9 | 254.0 | 1.18e-67 |
| ABX37385.1 | amino\_acid\_adenylation\_domain\_protein | BGC0000984 | NRP+Polyketide | 27.0 | 69.3 | 253.0 | 1.18e-67 |
| QWP75304.1 | non-ribosomal\_peptide\_synthase | BGC0002126 | NRP:Cyclic depsipeptide | 27.0 | 67.9 | 253.0 | 1.28e-67 |
| CAM59606.1 | non-ribosomal\_peptide\_synthetase | BGC0000297 | NRP:Glycopeptide+Polyketide:Other polyketide+Saccharide:Hybrid/tailoring saccharide | 26.0 | 63.9 | 253.0 | 1.31e-67 |
| WP\_064118560.1 | non-ribosomal\_peptide\_synthetase | BGC0001509 | NRP | 32.0 | 46.3 | 254.0 | 1.32e-67 |
| AJK49757.1 | non-ribosomal\_peptide\_synthase | BGC0002565 | NRP | 29.0 | 64.5 | 253.0 | 1.37e-67 |
| CAE02630.1 | surfactin\_synthetase\_A | BGC0000433 | NRP:Lipopeptide | 26.0 | 68.1 | 253.0 | 1.61e-67 |
| APZ78744.1 | nonribosomal\_peptide\_synthetase | BGC0001422 | NRP:Cyclic depsipeptide+Polyketide:Iterative type I polyketide | 28.0 | 68.3 | 253.0 | 1.75e-67 |
| ABA73956.1 | putative\_non-ribosomal\_peptide\_synthetase | BGC0001842 | NRP:Lipopeptide | 27.0 | 67.6 | 253.0 | 1.8e-67 |
| CAE15497.1 |  | BGC0002286 | NRP | 30.0 | 47.5 | 253.0 | 1.85e-67 |
| EPH46596.1 | putative\_Linear\_gramicidin\_synthase\_subunit\_C | BGC0001519 | NRP+Polyketide | 27.0 | 70.2 | 253.0 | 1.89e-67 |
| QDF82255.1 | non-ribosomal\_peptide\_synthetase | BGC0001980 | NRP | 31.0 | 47.9 | 253.0 | 2.28e-67 |
| ALG65318.1 | Cal18 | BGC0001297 | NRP | 30.0 | 52.8 | 253.0 | 2.31e-67 |
| BAF50711.1 | non\_ribosomal\_peptide\_synthetase\_for\_virginiamycin\_S | BGC0001116 | NRP+Polyketide | 28.0 | 63.5 | 253.0 | 2.37e-67 |
| ACM79812.1 | ZmaQ | BGC0001059 | NRP+Polyketide | 26.0 | 68.8 | 252.0 | 2.63e-67 |
| QED88054.1 | nonribosomal\_peptide\_synthetase | BGC0001967 | NRP+Polyketide | 32.0 | 45.6 | 253.0 | 3.07e-67 |
| AAO62588.1 | peptide\_sythetase | BGC0001016 | NRP+Polyketide | 26.0 | 69.0 | 250.0 | 3.2e-67 |
| KPN93063.1 | NupA | BGC0001416 | NRP | 31.0 | 50.5 | 253.0 | 3.56e-67 |
| QNL14921.1 | AptB | BGC0002512 | NRP | 29.0 | 46.9 | 248.0 | 3.72e-67 |
| CAJ96468.1 | non-ribosomal\_peptide\_synthetase | BGC0000330 | NRP:NRP siderophore | 32.0 | 46.4 | 248.0 | 3.9e-67 |
| AFH75321.1 | nonribosomal\_peptide\_synthetase | BGC0000425 | NRP:Cyclic depsipeptide | 31.0 | 46.8 | 252.0 | 4.39e-67 |
| CEK23366.1 | putative\_Ornithine\_racemase | BGC0001716 | NRP | 27.0 | 66.7 | 252.0 | 4.63e-67 |
| AEZ51517.1 | pmxB | BGC0001153 | NRP:Lipopeptide | 27.0 | 63.5 | 248.0 | 4.8e-67 |
| BAW32324.1 | nonribosomal\_peptide\_synthetase | BGC0001630 | NRP+Polyketide | 27.0 | 62.8 | 252.0 | 4.82e-67 |
| QVQ62868.1 | nonribosomal\_peptide\_synthase | BGC0002373 | NRP | 28.0 | 68.8 | 252.0 | 5.33e-67 |
| AEF33078.1 | dimodular\_nonribosomal\_peptide\_synthetase | BGC0001039 | NRP+Polyketide | 33.0 | 45.8 | 251.0 | 5.92e-67 |
| AAZ03550.1 | McnA | BGC0000332 | NRP | 30.0 | 45.6 | 248.0 | 6.2e-67 |
| CBA63680.1 | nonribosomal\_peptide\_synthetase\_NRPS | BGC0000368 | NRP | 28.0 | 69.9 | 251.0 | 6.71e-67 |
| ACO78737.1 | Non-ribosomal\_peptide\_synthase,\_PvdD/PvdJ-like\_protein | BGC0002433 | NRP | 29.0 | 52.3 | 251.0 | 7.22e-67 |
| ATY37608.1 | BreC | BGC0001536 | NRP | 25.0 | 63.0 | 251.0 | 8.32e-67 |
| ATU31794.1 | NRPS | BGC0001814 | NRP | 26.0 | 68.0 | 251.0 | 9.24e-67 |
| ABE35421.1 | Non-ribosomal\_peptide\_synthetase | BGC0002421 | NRP | 28.0 | 64.3 | 250.0 | 9.63e-67 |
| AEA30272.1 | peptide\_synthetase | BGC0000429 | Polyketide+NRP:Cyclic depsipeptide | 29.0 | 64.2 | 251.0 | 1.22e-66 |
| AVI26390.1 | polyketide\_synthase\_/\_nonribosomal\_peptide\_synthase\_hybrid | BGC0001800 | NRP+Polyketide | 27.0 | 67.8 | 251.0 | 1.22e-66 |
| QMS47800.1 | JesC | BGC0001629 | NRP:Lipopeptide | 30.0 | 48.2 | 251.0 | 1.41e-66 |
| EFE73313.1 | nonribosomal\_peptide\_synthetase | BGC0000431 | NRP:Cyclic depsipeptide | 28.0 | 63.7 | 250.0 | 1.5e-66 |
| ABF87167.1 | non-ribosomal\_peptide\_synthase\_MxcG | BGC0002492 | NRP | 32.0 | 47.9 | 249.0 | 1.59e-66 |
| BAV56270.1 |  | BGC0001657 | NRP | 28.0 | 68.7 | 250.0 | 2.41e-66 |
| CDG17981.1 | Non-ribosomal\_peptide\_synthetase | BGC0000464 | NRP:Cyclic depsipeptide | 26.0 | 69.0 | 249.0 | 2.45e-66 |
| BAH43765.1 | tyrocidine\_synthetase\_II | BGC0000452 | NRP | 27.0 | 68.3 | 249.0 | 2.51e-66 |
| CAD70195.1 | non-ribosomal\_peptide\_synthetase | BGC0001047 | NRP+Polyketide | 27.0 | 63.1 | 249.0 | 2.67e-66 |
| APZ78856.1 | nonribosomal\_peptide\_synthetase | BGC0001432 | NRP:Cyclic depsipeptide+Polyketide:Iterative type I polyketide | 28.0 | 67.5 | 249.0 | 2.72e-66 |
| AWX24482.1 | non-ribosomal\_peptide\_synthetase | BGC0001695 | NRP | 31.0 | 63.8 | 249.0 | 2.77e-66 |
| AAC06348.1 | bacitracin\_synthetase\_3 | BGC0000310 | NRP | 25.0 | 67.9 | 249.0 | 2.98e-66 |
| QCQ67881.1 | non-ribosomal\_peptide\_synthetase | BGC0002297 | NRP+Polyketide | 26.0 | 68.6 | 247.0 | 3.14e-66 |
| QVQ62855.1 | nonribosomal\_peptide\_synthase | BGC0002373 | NRP | 31.0 | 48.5 | 249.0 | 3.29e-66 |
| AAO72425.1 | syringopeptin\_synthetase\_C | BGC0000438 | NRP | 31.0 | 45.9 | 249.0 | 3.31e-66 |
| AEG64698.1 | LpmD | BGC0000379 | NRP | 28.0 | 64.7 | 249.0 | 3.42e-66 |
| KPN93064.1 | NupB | BGC0001416 | NRP | 30.0 | 46.0 | 249.0 | 3.56e-66 |
| EPH46597.1 | putative\_Linear\_gramicidin\_synthase\_subunit\_C | BGC0001519 | NRP+Polyketide | 27.0 | 63.3 | 249.0 | 3.61e-66 |
| ARU08074.1 | mlcL | BGC0001448 | NRP:Lipopeptide:Ca+-dependent lipopeptide | 28.0 | 69.2 | 249.0 | 3.82e-66 |
| CCJ67638.1 | TaaC | BGC0000447 | NRP:Lipopeptide | 33.0 | 46.0 | 249.0 | 4.19e-66 |
| APZ78769.1 | nonribosomal\_peptide\_synthetase | BGC0001425 | NRP:Cyclic depsipeptide+Polyketide:Iterative type I polyketide | 27.0 | 68.1 | 249.0 | 4.71e-66 |
| BAE98155.1 | putative\_non-ribosomal\_peptide\_synthetase | BGC0000339 | NRP | 34.0 | 36.4 | 248.0 | 4.79e-66 |
| QYA95682.1 | amino\_acid\_adenylation\_domain-containing\_protein | BGC0002676 | NRP | 29.0 | 64.6 | 249.0 | 4.8e-66 |
| AGC65516.1 | NRPS/PKS\_hybrid | BGC0001050 | NRP:Lipopeptide+Polyketide:Trans-AT type I polyketide | 28.0 | 69.3 | 249.0 | 4.97e-66 |
| ANS62966.1 | non-ribosomal\_peptide\_synthetase | BGC0001567 | NRP | 28.0 | 64.8 | 247.0 | 5.06e-66 |
| WP\_010639240.1 | non-ribosomal\_peptide\_synthetase | BGC0000958 | NRP:Cyclic depsipeptide+Polyketide:Modular type I polyketide | 29.0 | 63.3 | 248.0 | 5.13e-66 |
| QGY73453.1 | Itm21 | BGC0002451 | Polyketide | 27.0 | 68.9 | 248.0 | 5.24e-66 |
| AAF00961.1 | mcyB | BGC0001017 | NRP+Polyketide:Modular type I polyketide | 26.0 | 68.1 | 248.0 | 5.3e-66 |
| QIE08737.1 | non-ribosomal\_peptide\_synthetase | BGC0002544 | NRP | 28.0 | 67.6 | 248.0 | 5.9e-66 |
| AFH75329.1 | nonribosomal\_peptide\_synthetase | BGC0000398 | NRP:Cyclic depsipeptide | 31.0 | 47.6 | 248.0 | 6.15e-66 |
| AAG31130.1 | MxcG | BGC0001345 | NRP | 30.0 | 48.4 | 247.0 | 6.38e-66 |
| AET98906.1 | putative\_non-ribosomal\_peptide\_synthetase | BGC0000415 | NRP | 31.0 | 51.4 | 248.0 | 6.4e-66 |
| AAC06346.1 | bacitracin\_synthetase\_1 | BGC0000310 | NRP | 26.0 | 67.1 | 248.0 | 6.5e-66 |
| QMN69932.1 | PsoA | BGC0002521 | NRP | 31.0 | 47.5 | 248.0 | 6.94e-66 |
| AED90003.1 | non-ribosomal\_peptide\_synthetase\_ThaB | BGC0000443 | NRP:Beta-lactam | 28.0 | 67.9 | 248.0 | 8.19e-66 |
| NHN68324.1 | amino\_acid\_adenylation\_domain-containing\_protein | BGC0002719 | NRP | 31.0 | 48.1 | 248.0 | 9.35e-66 |
| ACO78736.1 | Non-ribosomal\_peptide\_synthase,\_PvdD-like\_protein | BGC0002433 | NRP | 28.0 | 68.4 | 247.0 | 1.16e-65 |
| FIS9431\_RS32925 | non-ribosomal\_peptide\_synthetase | BGC0001467 | NRP:Cyclic depsipeptide+Polyketide:Modular type I polyketide | 27.0 | 57.4 | 246.0 | 1.19e-65 |
| AGU50951.1 | putative\_non-ribosomal\_peptide\_synthetase | BGC0002417 | NRP+Polyketide | 32.0 | 45.4 | 244.0 | 1.19e-65 |
| AQH32485.1 | peptide\_synthetase | BGC0001667 | NRP+Polyketide | 26.0 | 63.8 | 247.0 | 1.21e-65 |
| AVR48535.1 | CusC | BGC0001564 | NRP+Polyketide | 26.0 | 69.3 | 248.0 | 1.26e-65 |
| AAY37655.1 | Amino\_acid\_adenylation | BGC0000437 | NRP | 31.0 | 45.9 | 248.0 | 1.3e-65 |
| EFL06865.1 | hypothetical\_protein | BGC0000300 | NRP | 27.0 | 69.0 | 247.0 | 1.33e-65 |
| ATY72525.1 | non-ribosomal\_peptide\_synthetase | BGC0001574 | NRP | 28.0 | 57.7 | 246.0 | 1.38e-65 |
| BAH04162.1 | trsJ | BGC0000450 | NRP | 31.0 | 51.5 | 246.0 | 1.93e-65 |
| CAD17792.1 | probable\_non\_ribosomal\_peptide\_synthetase\_protein | BGC0001363 | NRP+Polyketide | 28.0 | 68.6 | 247.0 | 2.06e-65 |
| CBW75451.1 | Non-ribosomal\_peptide\_synthetase\_modules | BGC0002048 | NRP:Cyclic depsipeptide | 27.0 | 55.7 | 246.0 | 2.24e-65 |
| QIH29229.1 | endopyrrole\_NRPS\_B | BGC0002326 | NRP | 27.0 | 55.7 | 246.0 | 2.24e-65 |
| AGM16414.1 | paenibacterin\_synthetase\_C | BGC0000400 | NRP | 27.0 | 63.7 | 246.0 | 2.8e-65 |
| ABV79986.1 | ApnB | BGC0000301 | NRP | 29.0 | 46.0 | 243.0 | 2.9e-65 |
| ACA97576.1 | PmxA | BGC0000408 | NRP | 27.0 | 62.8 | 246.0 | 3.31e-65 |
| TRX17524.1 | amino\_acid\_adenylation\_domain-containing\_protein | BGC0002329 | NRP | 32.0 | 46.6 | 246.0 | 3.38e-65 |
| AHD05678.1 | nonribosomal\_peptide\_ligase\_subunit | BGC0000402 | NRP | 32.0 | 45.6 | 246.0 | 3.38e-65 |
| AAO72424.1 | syringopeptin\_synthetase\_B | BGC0000438 | NRP | 30.0 | 50.5 | 246.0 | 3.4e-65 |
| WP\_064118559.1 | non-ribosomal\_peptide\_synthase/polyketide\_synthase | BGC0001509 | NRP | 26.0 | 68.5 | 246.0 | 3.46e-65 |
| CAY48789.1 | putative\_non-ribosomal\_peptide\_synthetase | BGC0001312 | NRP | 30.0 | 46.1 | 246.0 | 3.98e-65 |
| ARU08075.1 | mlcM | BGC0001448 | NRP:Lipopeptide:Ca+-dependent lipopeptide | 30.0 | 48.4 | 245.0 | 5.1e-65 |
| AFY58519.1 | amino\_acid\_adenylation\_enzyme/thioester\_reductase\_family\_protein,thioester\_reductase-like\_protein | BGC0002411 | NRP+Polyketide | 27.0 | 49.2 | 244.0 | 5.61e-65 |
| AFJ14795.1 | PlpF | BGC0000403 | NRP | 29.0 | 46.4 | 242.0 | 6.2e-65 |
| ABV79985.1 | ApnA | BGC0000301 | NRP | 28.0 | 48.8 | 244.0 | 6.41e-65 |
| CCJ67637.1 | TaaB | BGC0000447 | NRP:Lipopeptide | 30.0 | 46.0 | 245.0 | 6.48e-65 |
| ABD14711.1 | cesA | BGC0000320 | NRP:Cyclic depsipeptide | 27.0 | 68.1 | 245.0 | 6.58e-65 |
| CAJ21198.2 | non-ribosomal\_peptide\_synthetase | BGC0000297 | NRP:Glycopeptide+Polyketide:Other polyketide+Saccharide:Hybrid/tailoring saccharide | 30.0 | 45.9 | 244.0 | 7.28e-65 |
| BBD17741.1 | non-ribosomal\_peptide\_synthetase | BGC0001918 | NRP+Polyketide | 29.0 | 63.4 | 245.0 | 7.37e-65 |
| AKA54626.1 | NRPS | BGC0001216 | NRP+Polyketide | 29.0 | 63.4 | 244.0 | 7.98e-65 |
| BAD55611.1 | putative\_non-ribosomal\_peptide\_synthetase | BGC0001027 | NRP+Polyketide | 27.0 | 62.5 | 244.0 | 8.16e-65 |
| QCQ67880.1 | non-ribosomal\_peptide\_synthetase | BGC0002297 | NRP+Polyketide | 26.0 | 63.6 | 244.0 | 8.29e-65 |
| ABG94125.1 | non-ribosomal\_peptide\_synthetase | BGC0000417 | NRP | 27.0 | 65.3 | 244.0 | 9.12e-65 |
| CAG15012.1 | peptide\_synthetase,\_module\_7 | BGC0000441 | NRP | 29.0 | 52.3 | 244.0 | 9.33e-65 |
| CDG17982.1 | Non-ribosomal\_peptide\_synthetase | BGC0000464 | NRP:Cyclic depsipeptide | 26.0 | 70.4 | 244.0 | 9.9e-65 |
| WP\_018540603.1 | non-ribosomal\_peptide\_synthetase | BGC0001332 | NRP+Polyketide | 27.0 | 67.4 | 241.0 | 1.11e-64 |
| CAM56771.1 |  | BGC0000354 | NRP | 28.0 | 63.4 | 244.0 | 1.2e-64 |
| ATP76246.1 | SpuB | BGC0001748 | NRP+Polyketide | 32.0 | 36.3 | 244.0 | 1.23e-64 |
| CAE53353.1 | non-ribosomal\_peptide\_synthetase | BGC0000440 | NRP:Glycopeptide | 29.0 | 52.3 | 243.0 | 1.23e-64 |
| ATY37591.1 | BogD | BGC0001532 | NRP | 29.0 | 46.1 | 244.0 | 1.28e-64 |
| ABS90473.1 | NRPS | BGC0001106 | NRP+Polyketide | 30.0 | 52.9 | 243.0 | 1.34e-64 |
| CAJ46691.1 | non-ribosomal\_peptide\_synthase | BGC0000969 | NRP:Cyclic depsipeptide+Polyketide:Modular type I polyketide | 28.0 | 65.2 | 241.0 | 1.58e-64 |
| AKJ29411.1 | peptide\_synthetase | BGC0001608 | NRP | 27.0 | 70.0 | 244.0 | 1.65e-64 |
| CCP42826.1 | Probable\_peptide\_synthetase\_Nrp\_(peptide\_synthase) | BGC0001627 | NRP | 28.0 | 58.6 | 243.0 | 2.18e-64 |
| AJM89735.1 | PmxA | BGC0001192 | NRP | 27.0 | 63.8 | 243.0 | 2.26e-64 |
| QDQ83032.1 | amino\_acid\_adenylation\_domain-containing\_protein | BGC0002564 | NRP | 30.0 | 64.3 | 243.0 | 2.3e-64 |
| KAF7597140.1 | hypothetical\_protein | BGC0002264 | NRP | 26.0 | 78.0 | 239.0 | 2.39e-64 |
| CAM56770.1 |  | BGC0000354 | NRP | 27.0 | 69.0 | 243.0 | 2.41e-64 |
| AHZ20774.1 | non-ribosomal\_peptide\_synthase | BGC0000369 | NRP+Saccharide:Hybrid/tailoring saccharide | 27.0 | 62.9 | 243.0 | 2.67e-64 |
| ABC36785.1 | peptide\_synthetase,\_putative | BGC0000964 | NRP:Cyclic depsipeptide+Polyketide:Trans-AT type I polyketide | 31.0 | 46.3 | 243.0 | 2.67e-64 |
| BAH43766.1 | tyrocidine\_synthetase\_III | BGC0000452 | NRP | 26.0 | 68.3 | 243.0 | 3.15e-64 |
| UEF20578.1 | nonribosomal\_peptide\_synthetase | BGC0002360 | NRP | 29.0 | 52.7 | 243.0 | 3.31e-64 |
| AIG26884.1 | NRPS\_domain-containing\_protein | BGC0002432 | NRP | 27.0 | 53.8 | 243.0 | 3.44e-64 |
| AGS77310.1 | NRPS\_module\_7 | BGC0001178 | NRP:Glycopeptide | 30.0 | 52.3 | 242.0 | 3.72e-64 |
| AFJ23825.1 | WLIP\_synthetase\_B | BGC0001838 | NRP | 29.0 | 46.0 | 243.0 | 3.73e-64 |
| AAC44129.1 | saframycin\_Mx1\_synthetase\_A | BGC0002706 | NRP | 27.0 | 69.7 | 242.0 | 3.87e-64 |
| ATP76243.1 | NdaA | BGC0001705 | NRP+Polyketide | 27.0 | 68.1 | 242.0 | 3.89e-64 |
| CAJ96470.1 | non-ribosomal\_peptide\_synthetase | BGC0000330 | NRP:NRP siderophore | 30.0 | 64.7 | 242.0 | 4.05e-64 |
| AHB82072.1 | non\_ribosomal\_peptide\_synthetase/polyketide\_synthase | BGC0001231 | NRP+Polyketide:Modular type I polyketide | 30.0 | 48.1 | 242.0 | 4.18e-64 |
| AJW76710.1 | DsaH | BGC0001196 | NRP | 31.0 | 45.9 | 241.0 | 5.98e-64 |
| AZH29360.1 | amino\_acid\_adenylation\_domain-containing\_protein | BGC0001843 | NRP | 27.0 | 63.9 | 242.0 | 6.03e-64 |
| OLZ50885.1 | non-ribosomal\_peptide\_synthetase | BGC0001461 | NRP:Glycopeptide | 28.0 | 63.5 | 242.0 | 6.33e-64 |
| CAK15814.1 | putative\_non-ribosomal\_peptide\_synthetase,\_terminal\_component | BGC0000344 | NRP | 27.0 | 68.1 | 242.0 | 6.69e-64 |
| AGU50950.1 | putative\_non-ribosomal\_peptide\_synthetase | BGC0002417 | NRP+Polyketide | 27.0 | 67.2 | 241.0 | 6.72e-64 |
| CAD17793.1 | probable\_non\_ribosomal\_peptide\_synthetase\_protein | BGC0001363 | NRP+Polyketide | 29.0 | 46.8 | 242.0 | 7.04e-64 |
| QWT72279.1 | non-ribosomal\_peptide\_synthetase | BGC0002430 | NRP+Saccharide | 29.0 | 65.2 | 242.0 | 7.49e-64 |
| AAS47564.1 | mixed\_type\_I\_polyketide\_synthase/nonribosomal\_peptide\_synthetase | BGC0001108 | NRP+Polyketide:Trans-AT type I polyketide | 27.0 | 63.7 | 242.0 | 7.51e-64 |
| ctg1\_orf6 |  | BGC0001109 | NRP+Polyketide | 27.0 | 63.7 | 242.0 | 7.51e-64 |
| AHF21228.1 | TriD | BGC0000449 | NRP | 30.0 | 46.1 | 242.0 | 7.92e-64 |
| AGE11899.1 | nonribosomal\_peptide\_synthetase | BGC0000366 | NRP | 31.0 | 45.6 | 241.0 | 8.14e-64 |
| QYC40290.1 | A50926\_NRPS,\_module\_7 | BGC0002344 | NRP | 30.0 | 52.1 | 241.0 | 8.48e-64 |
| ABS74207.1 | fengycin\_synthetase\_C | BGC0001095 | NRP | 26.0 | 66.9 | 241.0 | 8.67e-64 |
| ACO78738.1 | Non-ribosomal\_peptide\_synthase,\_PvdJ(2)-like\_protein | BGC0002433 | NRP | 29.0 | 53.0 | 241.0 | 8.75e-64 |
| AGZ15459.1 | putative\_non-ribosomal\_peptide\_synthetase | BGC0001036 | NRP+Polyketide | 26.0 | 63.2 | 241.0 | 9.23e-64 |
| AHZ20773.1 | non-ribosomal\_peptide\_synthase | BGC0000369 | NRP+Saccharide:Hybrid/tailoring saccharide | 27.0 | 68.6 | 241.0 | 9.68e-64 |
| QKF54436.1 | nonribosomal\_peptide\_synthetase | BGC0002581 | NRP | 29.0 | 52.7 | 241.0 | 9.91e-64 |
| QUJ09166.1 | Lon19 | BGC0002440 | NRP | 30.0 | 46.1 | 241.0 | 9.91e-64 |
| DAD54574.1 | NRPS-like\_oxidoreductase | BGC0002256 | NRP+Other | 27.0 | 68.4 | 237.0 | 1.03e-63 |
| AZM57022.1 | non-ribosomal\_peptide\_synthetase | BGC0002314 | NRP | 29.0 | 51.4 | 241.0 | 1.06e-63 |
| ACS20360.1 | amino\_acid\_adenylation\_domain\_protein | BGC0002420 | NRP+Polyketide | 31.0 | 45.4 | 238.0 | 1.09e-63 |
| CAA11796.1 | PCZA363.5 | BGC0000322 | NRP | 30.0 | 52.6 | 240.0 | 1.11e-63 |
| AAY93354.1 | non-ribosomal\_peptide\_synthetase\_PvdD | BGC0000413 | NRP | 26.0 | 68.8 | 241.0 | 1.13e-63 |
| AHD05677.1 | nonribosomal\_peptide\_ligase\_subunit | BGC0000402 | NRP | 28.0 | 53.2 | 241.0 | 1.16e-63 |
| AOA33123.1 | Nonribosomal\_peptide\_synthetase | BGC0001346 | NRP:Cyclic depsipeptide | 30.0 | 46.9 | 241.0 | 1.2e-63 |
| AAO62587.1 | peptide\_sythetase | BGC0001016 | NRP+Polyketide | 27.0 | 68.6 | 240.0 | 1.29e-63 |
| QKF54438.1 | nonribosomal\_peptide\_synthetase | BGC0002581 | NRP | 28.0 | 63.5 | 241.0 | 1.43e-63 |
| ATY37592.1 | BogE | BGC0001532 | NRP | 29.0 | 45.6 | 240.0 | 1.47e-63 |
| BAP05596.1 | calH | BGC0000967 | NRP+Polyketide:Trans-AT type I polyketide | 26.0 | 69.3 | 241.0 | 1.52e-63 |
| CDG17987.1 | Putative\_Ornithine\_racemase\_(fragment) | BGC0000464 | NRP:Cyclic depsipeptide | 29.0 | 51.7 | 241.0 | 1.53e-63 |
| ABP55169.1 | amino\_acid\_adenylation\_domain | BGC0000150 | NRP+Polyketide:Enediyne type I polyketide | 30.0 | 48.4 | 228.0 | 1.67e-63 |
| CAK15815.1 | putative\_non\_ribosomal\_peptide\_synthetase | BGC0000344 | NRP | 29.0 | 51.6 | 241.0 | 1.7e-63 |
| AHZ34232.1 | CifA | BGC0000323 | NRP:Lipopeptide | 29.0 | 45.8 | 240.0 | 1.7e-63 |
| AFJ14793.1 | PlpD | BGC0000403 | NRP | 27.0 | 63.7 | 239.0 | 1.76e-63 |
| UEF20580.1 | nonribosomal\_peptide\_synthetase | BGC0002360 | NRP | 28.0 | 63.5 | 240.0 | 1.87e-63 |
| CAD91211.1 | putative\_non-ribosomal\_peptide\_synthetase,\_module\_7 | BGC0000289 | NRP:Glycopeptide+Saccharide:Hybrid/tailoring saccharide | 30.0 | 52.3 | 239.0 | 1.92e-63 |
| AEW31019.1 | plipastatin\_synthetase | BGC0000407 | NRP | 27.0 | 63.5 | 240.0 | 1.98e-63 |
| SDF67417.1 | amino\_acid\_adenylation\_domain-containing\_protein | BGC0002422 | NRP | 30.0 | 45.7 | 240.0 | 2.19e-63 |
| PVC99865.1 | non-ribosomal\_peptide\_synthetase | BGC0002100 | NRP+Other | 31.0 | 45.6 | 240.0 | 2.41e-63 |
| AHZ34238.1 | CipA | BGC0001389 | NRP | 30.0 | 45.4 | 240.0 | 2.64e-63 |
| AWI62627.1 | nonribosomal\_peptide\_synthetase | BGC0001822 | NRP | 31.0 | 50.0 | 239.0 | 2.98e-63 |
| ABR67744.1 | CmnA | BGC0000316 | NRP | 30.0 | 46.6 | 239.0 | 3.26e-63 |
| CBL93718.1 | NRPS\_didomain\_PCP-C | BGC0000360 | NRP | 27.0 | 69.3 | 239.0 | 3.27e-63 |
| CDF96614.1 | NRPS | BGC0001149 | NRP:Lipopeptide+Saccharide:Hybrid/tailoring saccharide | 28.0 | 53.0 | 239.0 | 3.29e-63 |
| QGQ63519.1 | nonribosomal\_peptide\_synthetase\_modules\_B | BGC0002548 | NRP | 32.0 | 45.7 | 239.0 | 3.34e-63 |
| QDJ74275.1 | non-ribosomal\_peptide\_synthetase | BGC0002109 | NRP | 32.0 | 36.2 | 239.0 | 3.49e-63 |
| ABU70377.1 | hypothetical\_protein | BGC0001890 | NRP | 30.0 | 45.4 | 238.0 | 3.58e-63 |
| ABC94347.1 | vicibactin\_biosynthesis\_non-ribosomal\_peptide\_synthase\_protein | BGC0000457 | NRP | 27.0 | 63.7 | 238.0 | 3.71e-63 |
| AFH75328.1 | nonribosomal\_peptide\_synthetase | BGC0000398 | NRP:Cyclic depsipeptide | 27.0 | 63.9 | 239.0 | 3.86e-63 |
| AAY37647.1 | Amino\_acid\_adenylation | BGC0000437 | NRP | 30.0 | 48.6 | 239.0 | 3.92e-63 |
| AHB82056.1 | non\_ribosomal\_peptide\_synthetase | BGC0001019 | NRP+Polyketide:Modular type I polyketide | 28.0 | 68.0 | 239.0 | 4.11e-63 |
| CAQ71829.1 | non\_ribosomal\_peptide\_synthase,\_antibiotic\_synthesis;\_contains\_4\_condensation\_domains,\_3\_AMP-acid\_ligases\_II\_domains,\_3\_PP-binding,\_Phosphopantetheine\_attachment\_site\_and\_a\_putative\_thioesterase\_domain | BGC0001189 | NRP | 30.0 | 51.7 | 239.0 | 4.26e-63 |
| BAB69700.1 | iturin\_A\_synthetase\_C | BGC0001098 | NRP+Polyketide | 25.0 | 63.6 | 239.0 | 4.57e-63 |
| ACS20359.1 | amino\_acid\_adenylation\_domain\_protein | BGC0002420 | NRP+Polyketide | 28.0 | 63.0 | 239.0 | 4.58e-63 |
| DAB41476.1 | nonribosomal\_peptide\_synthetase | BGC0001230 | NRP:Cyclic depsipeptide+Polyketide:Modular type I polyketide | 29.0 | 64.5 | 237.0 | 4.91e-63 |
| APZ78833.1 | nonribosomal\_peptide\_synthetase | BGC0001430 | NRP:Cyclic depsipeptide+Polyketide:Iterative type I polyketide | 27.0 | 62.8 | 239.0 | 5.01e-63 |
| AHZ34242.1 | CipE | BGC0001389 | NRP | 29.0 | 50.6 | 239.0 | 5.02e-63 |
| ALG65317.1 | Cal19 | BGC0001297 | NRP | 27.0 | 70.1 | 238.0 | 5.89e-63 |
| RAT94090.1 | NRPS | BGC0001469 | NRP | 27.0 | 54.4 | 239.0 | 6.27e-63 |
| CCP45168.1 | Peptide\_synthetase\_MbtE\_(peptide\_synthase) | BGC0001021 | NRP+Polyketide | 27.0 | 63.4 | 238.0 | 6.56e-63 |
| AHZ34239.1 | CipB | BGC0001389 | NRP | 30.0 | 45.4 | 238.0 | 6.71e-63 |
| ATD51279.1 | nonribosomal\_peptide\_synthase | BGC0001650 | NRP | 27.0 | 63.9 | 238.0 | 6.8e-63 |
| CEK23364.1 | putative\_Phenylalanine\_racemase\_(ATP-hydrolyzing) | BGC0001716 | NRP | 25.0 | 64.1 | 238.0 | 7.13e-63 |
| CZT62784.1 | Non-ribosomal\_peptide\_synthase,\_involved\_in\_Hassallidin\_biosynthesis | BGC0001614 | NRP | 27.0 | 65.2 | 238.0 | 7.16e-63 |
| AAY93356.2 | non-ribosomal\_peptide\_synthetase\_PvdI | BGC0000413 | NRP | 27.0 | 68.5 | 238.0 | 7.19e-63 |
| AZM57023.1 | non-ribosomal\_peptide\_synthetase | BGC0002314 | NRP | 27.0 | 53.6 | 237.0 | 7.31e-63 |
| AJD47484.1 | protein\_PvdD | BGC0002418 | NRP+Polyketide | 29.0 | 52.1 | 238.0 | 7.38e-63 |
| QPI18726.1 | nonribosomal\_peptide\_synthetase | BGC0002125 | NRP:Cyclic depsipeptide | 27.0 | 67.4 | 234.0 | 7.79e-63 |
| QGY73448.1 | Itm16 | BGC0002451 | Polyketide | 30.0 | 50.7 | 236.0 | 7.81e-63 |
| ATO51563.1 | non-ribosomal\_peptide\_synthetase | BGC0001796 | NRP | 29.0 | 46.4 | 238.0 | 8.37e-63 |
| NAO96319.1 | amino\_acid\_adenylation\_domain-containing\_protein | BGC0002117 | NRP | 29.0 | 46.4 | 238.0 | 9.04e-63 |
| AOA33122.1 | Nonribosomal\_peptide\_synthetase | BGC0001346 | NRP:Cyclic depsipeptide | 30.0 | 50.9 | 238.0 | 9.98e-63 |
| AUD11993.1 | OrbJ | BGC0001721 | NRP | 29.0 | 64.0 | 237.0 | 1.14e-62 |
| AQI70\_32580 |  | BGC0001561 | NRP | 32.0 | 45.2 | 236.0 | 1.22e-62 |
| QEO75077.1 | condensation\_domain-containing\_protein | BGC0002079 | NRP:Cyclic depsipeptide | 30.0 | 52.8 | 237.0 | 1.24e-62 |
| ALG65341.1 | Var6 | BGC0002416 | NRP+Polyketide | 27.0 | 67.4 | 237.0 | 1.35e-62 |
| QRK05501.1 | myxochelin\_non-ribosomal\_peptide\_synthetase\_MxcG | BGC0002324 | NRP+Polyketide | 31.0 | 48.8 | 236.0 | 1.43e-62 |
| QCQ67875.1 | hybrid\_peptide\_synthetase/polyketide\_synthase | BGC0002297 | NRP+Polyketide | 26.0 | 68.0 | 237.0 | 1.59e-62 |
| KGA48739.1 | amino\_acid\_adenylation\_domain\_protein | BGC0002413 | NRP | 29.0 | 52.1 | 237.0 | 1.69e-62 |
| AEW31021.1 | plipastatin\_synthetase | BGC0000407 | NRP | 28.0 | 53.0 | 237.0 | 1.77e-62 |
| QKM21620.1 | non-ribosomal\_peptide\_synthetase | BGC0002351 | NRP | 27.0 | 69.2 | 237.0 | 1.87e-62 |
| CCJ67648.1 | JagD | BGC0001127 | NRP | 27.0 | 64.1 | 237.0 | 1.92e-62 |
| NKI69296.1 | amino\_acid\_adenylation\_domain-containing\_protein | BGC0002408 | NRP | 28.0 | 65.5 | 236.0 | 1.99e-62 |
| WP\_064118561.1 | non-ribosomal\_peptide\_synthetase | BGC0001509 | NRP | 31.0 | 45.4 | 236.0 | 2.01e-62 |
| ABM34280.1 | amino\_acid\_adenylation\_domain\_protein | BGC0002419 | NRP+Polyketide | 27.0 | 63.9 | 236.0 | 2.14e-62 |
| AAF08795.1 | MycA | BGC0001103 | NRP+Polyketide | 26.0 | 68.5 | 237.0 | 2.2e-62 |
| EME52988.1 | amino\_acid\_adenylation\_protein | BGC0001460 | NRP:Glycopeptide | 30.0 | 52.3 | 236.0 | 2.23e-62 |
| OKA09425.1 | non-ribosomal\_peptide\_synthetase | BGC0001459 | NRP:Glycopeptide | 30.0 | 52.4 | 236.0 | 2.27e-62 |
| QPI18728.1 | nonribosomal\_peptide\_synthetase | BGC0002125 | NRP:Cyclic depsipeptide | 29.0 | 53.0 | 236.0 | 2.3e-62 |
| CAE15637.1 |  | BGC0001128 | NRP | 31.0 | 47.6 | 237.0 | 2.39e-62 |
| AXG48275.1 | non-ribosomal\_peptide\_synthetase | BGC0002716 | NRP | 31.0 | 47.6 | 237.0 | 2.39e-62 |
| AAY37654.1 | Amino\_acid\_adenylation | BGC0000437 | NRP | 30.0 | 46.3 | 237.0 | 2.42e-62 |
| BAC67536.1 | arthrofactin\_synthetase\_C | BGC0000305 | NRP:Lipopeptide | 26.0 | 68.8 | 237.0 | 2.46e-62 |
| CBF76038.1 | nonribosomal\_peptide\_synthase,\_putative\_(Eurofung) | BGC0001399 | NRP | 30.0 | 57.5 | 235.0 | 2.67e-62 |
| AVI26392.1 | putative\_nonribosomal\_peptide\_synthase | BGC0001800 | NRP+Polyketide | 27.0 | 63.4 | 236.0 | 2.87e-62 |
| AIE77060.1 | peptide\_synthetase\_module\_7 | BGC0000418 | NRP | 30.0 | 52.2 | 236.0 | 2.98e-62 |
| AAF08797.1 | MycC | BGC0001103 | NRP+Polyketide | 26.0 | 63.6 | 236.0 | 3.1e-62 |
| AMK48226.1 | nonribosomal\_peptide\_synthetase | BGC0001351 | NRP | 32.0 | 45.1 | 236.0 | 3.11e-62 |
| QMS47799.1 | JesA | BGC0001629 | NRP:Lipopeptide | 30.0 | 45.8 | 236.0 | 3.16e-62 |
| APZ78755.1 | nonribosomal\_peptide\_synthetase | BGC0001423 | NRP:Cyclic depsipeptide+Polyketide:Iterative type I polyketide | 26.0 | 69.2 | 236.0 | 3.39e-62 |
| QUS58939.1 | amino\_acid\_adenylation\_domain-containing\_protein | BGC0002123 | NRP+Polyketide | 25.0 | 68.1 | 236.0 | 4.23e-62 |
| WP\_051700105.1 | non-ribosomal\_peptide\_synthetase | BGC0001368 | NRP | 30.0 | 46.7 | 234.0 | 4.3e-62 |
| CAR51995.1 | ornibactin\_biosynthesis\_non-ribosomal\_peptide\_synthase | BGC0002569 | NRP | 29.0 | 64.2 | 235.0 | 4.44e-62 |
| AHZ20781.1 | non-ribosomal\_peptide\_synthase | BGC0000369 | NRP+Saccharide:Hybrid/tailoring saccharide | 30.0 | 49.5 | 235.0 | 4.61e-62 |
| AFJ23826.1 | WLIP\_synthetase\_C | BGC0001838 | NRP | 27.0 | 67.8 | 236.0 | 4.92e-62 |
| AEI58867.1 | peptide\_synthetase | BGC0000455 | NRP | 30.0 | 52.6 | 235.0 | 5.17e-62 |
| KPN90369.1 | NunE | BGC0001416 | NRP | 27.0 | 70.1 | 236.0 | 5.21e-62 |
| APO47826.1 | hypothetical\_protein | BGC0002653 | NRP | 27.0 | 62.8 | 236.0 | 5.36e-62 |
| AKJ29412.1 | peptide\_synthetase | BGC0001608 | NRP | 28.0 | 69.5 | 236.0 | 5.59e-62 |
| QLY89262.1 | pseudodesmin\_synthetase | BGC0002522 | NRP | 31.0 | 45.6 | 235.0 | 6.04e-62 |
| AJD47481.1 | amino\_acid\_adenylation\_domain-containing\_protein | BGC0002418 | NRP+Polyketide | 27.0 | 69.7 | 232.0 | 6.37e-62 |
| OLZ52442.1 | non-ribosomal\_peptide\_synthetase | BGC0001462 | NRP:Glycopeptide | 30.0 | 47.9 | 223.0 | 6.8e-62 |
| AHD05621.1 | non-ribosomal\_peptide\_ligase\_domain\_protein | BGC0001033 | NRP+Polyketide | 26.0 | 69.7 | 235.0 | 7.27e-62 |
| AAM80536.1 | StaD | BGC0000290 | NRP:Glycopeptide | 30.0 | 51.5 | 234.0 | 8.94e-62 |
| AHZ34243.1 | CipF | BGC0001389 | NRP | 31.0 | 45.4 | 235.0 | 9.98e-62 |
| AXA94654.1 | hypothetical\_protein | BGC0002044 | NRP | 30.0 | 45.8 | 232.0 | 1.08e-61 |
| OLZ52457.1 | non-ribosomal\_peptide\_synthetase | BGC0001462 | NRP:Glycopeptide | 29.0 | 54.8 | 234.0 | 1.14e-61 |
| OLZ50899.1 | non-ribosomal\_peptide\_synthetase | BGC0001461 | NRP:Glycopeptide | 30.0 | 47.7 | 223.0 | 1.22e-61 |
| AYA22318.1 | KerE | BGC0001955 | NRP | 30.0 | 47.7 | 223.0 | 1.22e-61 |
| BAB69699.1 | iturin\_A\_synthetase\_B | BGC0001098 | NRP+Polyketide | 27.0 | 63.6 | 234.0 | 1.24e-61 |
| AHH53508.1 | non-ribosomal\_peptide\_synthetase | BGC0000439 | NRP:Lipopeptide:Ca+-dependent lipopeptide | 28.0 | 64.3 | 234.0 | 1.5e-61 |
| UMM61372.1 | Tsk11 | BGC0002661 | NRP | 27.0 | 70.6 | 233.0 | 1.53e-61 |
| AIG79240.1 | Hypothetical\_protein | BGC0000419 | Saccharide+NRP:Glycopeptide | 30.0 | 52.1 | 233.0 | 1.55e-61 |
| CZT62785.1 | Non-ribosomal\_peptide\_synthase,\_involved\_in\_Hassallidin\_biosynthesis | BGC0001614 | NRP | 27.0 | 64.4 | 234.0 | 1.75e-61 |
| BAI63283.1 | putative\_non-ribosomal\_peptide\_synthetase | BGC0000434 | NRP | 31.0 | 46.2 | 222.0 | 1.83e-61 |
| ATY37588.1 | BogA | BGC0001532 | NRP | 28.0 | 45.6 | 224.0 | 1.88e-61 |
| AKP45399.1 | CysK | BGC0001413 | NRP | 27.0 | 64.2 | 234.0 | 2.05e-61 |
| AED90002.1 | non-ribosomal\_peptide\_synthetase\_ThaA | BGC0000443 | NRP:Beta-lactam | 26.0 | 67.6 | 234.0 | 2.14e-61 |
| QGQ63520.1 | nonribosomal\_peptide\_synthetase\_modules\_C | BGC0002548 | NRP | 30.0 | 46.2 | 233.0 | 2.18e-61 |
| MBX9445647.1 | amino\_acid\_adenylation\_domain-containing\_protein | BGC0002414 | NRP | 28.0 | 52.2 | 233.0 | 2.22e-61 |
| AIG79224.1 | Non-ribosomal\_peptide\_synthetase/andenylation\_domain | BGC0000419 | Saccharide+NRP:Glycopeptide | 29.0 | 47.7 | 222.0 | 2.22e-61 |
| WP\_012988806.1 | non-ribosomal\_peptide\_synthetase | BGC0002135 | NRP:Lipopeptide | 28.0 | 51.2 | 233.0 | 2.54e-61 |
| CAC48362.1 | peptide\_synthetase | BGC0000311 | NRP | 29.0 | 52.6 | 233.0 | 2.68e-61 |
| AEZ51516.1 | pmxA | BGC0001153 | NRP:Lipopeptide | 26.0 | 62.8 | 233.0 | 2.75e-61 |
| AIE77076.1 | peptide\_synthetase | BGC0000418 | NRP | 30.0 | 47.7 | 221.0 | 3.07e-61 |
| AXG47007.1 | non-ribosomal\_peptide\_synthetase | BGC0000383 | NRP+Polyketide:Modular type I polyketide | 26.0 | 64.9 | 230.0 | 3.14e-61 |
| ABW17377.1 | PsoC | BGC0000411 | NRP | 26.0 | 70.1 | 233.0 | 3.32e-61 |
| BAP05590.1 | calB | BGC0000967 | NRP+Polyketide:Trans-AT type I polyketide | 26.0 | 69.3 | 233.0 | 3.86e-61 |
| AHH53507.1 | non-ribosomal\_peptide\_synthetase | BGC0000439 | NRP:Lipopeptide:Ca+-dependent lipopeptide | 30.0 | 52.2 | 233.0 | 3.92e-61 |
| DAB41479.1 | nonribosomal\_peptide\_synthetase | BGC0001230 | NRP:Cyclic depsipeptide+Polyketide:Modular type I polyketide | 29.0 | 49.7 | 231.0 | 4.02e-61 |
| AEG64696.1 | LpmB | BGC0000379 | NRP | 29.0 | 63.6 | 233.0 | 4.06e-61 |
| AAU39359.1 | lichenysin\_synthase\_LchAA | BGC0000381 | NRP | 28.0 | 46.1 | 233.0 | 4.28e-61 |
| AZF85940.1 | non-ribosomal\_peptide\_synthase | BGC0001963 | NRP+Polyketide | 26.0 | 71.6 | 233.0 | 4.32e-61 |
| QYI86762.1 | non-ribosomal\_peptide\_synthetase | BGC0002424 | NRP | 28.0 | 64.8 | 233.0 | 4.61e-61 |
| AZH23822.1 | MgiJ | BGC0001971 | NRP+Polyketide | 27.0 | 57.9 | 231.0 | 4.83e-61 |
| NHN68325.1 | amino\_acid\_adenylation\_domain-containing\_protein | BGC0002719 | NRP | 31.0 | 47.1 | 233.0 | 4.96e-61 |
| AHD05618.1 | putative\_non-ribosomal\_peptide\_ligase\_domain\_protein | BGC0001033 | NRP+Polyketide | 30.0 | 46.4 | 229.0 | 5.15e-61 |
| CEK23605.1 | Non-ribosomal\_peptide\_synthase\_involved\_in\_xenematides\_synthesis | BGC0001825 | NRP | 27.0 | 63.9 | 232.0 | 6.24e-61 |
| BCJ07533.1 | hypothetical\_protein | BGC0002379 | NRP | 30.0 | 45.3 | 232.0 | 6.24e-61 |
| BAI63288.1 | putative\_non-ribosomal\_peptide\_synthetase | BGC0000434 | NRP | 31.0 | 51.4 | 232.0 | 6.25e-61 |
| UHJ79948.1 | non-ribosomal\_peptide\_synthetase | BGC0002654 | NRP | 27.0 | 68.5 | 231.0 | 6.54e-61 |
| WP\_100939442.1 | non-ribosomal\_peptide\_synthetase | BGC0002071 | NRP:Lipopeptide | 30.0 | 48.3 | 232.0 | 7e-61 |
| QGQ63518.1 | nonribosomal\_peptide\_synthetase\_modules\_A | BGC0002548 | NRP | 29.0 | 45.6 | 231.0 | 7.08e-61 |
| AXF16146.1 | non-ribosomal\_peptide\_synthetase | BGC0002563 | NRP | 31.0 | 47.2 | 232.0 | 8.46e-61 |
| BCJ07532.1 | hypothetical\_protein | BGC0002379 | NRP | 30.0 | 45.6 | 232.0 | 8.54e-61 |
| QMN69933.1 | PsoB | BGC0002521 | NRP | 28.0 | 46.1 | 232.0 | 8.92e-61 |
| CCA89328.1 | mixed\_trans-AT\_type\_I\_polyketide\_synthase/nonribosomal\_peptide\_synthetase | BGC0001111 | NRP+Polyketide:Trans-AT type I polyketide | 30.0 | 46.3 | 232.0 | 8.99e-61 |
| AKJ29410.1 | peptide\_synthetase | BGC0001608 | NRP | 30.0 | 51.3 | 231.0 | 9.28e-61 |
| QNL34616.1 | SteA | BGC0002092 | NRP:Cyclic depsipeptide | 30.0 | 45.2 | 231.0 | 9.31e-61 |
| WP\_153044786.1 | non-ribosomal\_peptide\_synthetase | BGC0001826 | NRP | 27.0 | 53.7 | 229.0 | 1e-60 |
| CCA29203.1 | non-ribosomal\_peptide\_synthetase/polyketide\_synthase | BGC0000955 | NRP+Polyketide:Modular type I polyketide | 29.0 | 53.7 | 231.0 | 1.1e-60 |
| WP\_019634550.1 | type\_I\_polyketide\_synthase | BGC0001443 | NRP+Polyketide | 32.0 | 40.9 | 230.0 | 1.39e-60 |
| RSO11554.1 | non-ribosomal\_peptide\_synthetase | BGC0002637 | NRP | 30.0 | 45.6 | 231.0 | 1.42e-60 |
| ABV56585.1 | KtzE | BGC0000378 | NRP | 31.0 | 47.6 | 230.0 | 1.54e-60 |
| QIE07359.1 | dimodular\_nonribosomal\_peptide\_synthase\_NecA | BGC0002050 | NRP+Polyketide:Trans-AT type I polyketide | 26.0 | 62.8 | 229.0 | 1.72e-60 |
| AEA30274.1 | peptide\_synthetase | BGC0000429 | Polyketide+NRP:Cyclic depsipeptide | 32.0 | 36.3 | 231.0 | 1.73e-60 |
| DAC80528.1 | peptide\_synthetase | BGC0001878 | NRP+Polyketide | 27.0 | 67.9 | 229.0 | 1.95e-60 |
| CAC48360.1 | peptide\_synthetase | BGC0000311 | NRP | 30.0 | 53.4 | 230.0 | 2.09e-60 |
| RSO11553.1 | non-ribosomal\_peptide\_synthetase | BGC0002637 | NRP | 31.0 | 45.4 | 230.0 | 2.16e-60 |
| ABM34277.1 | amino\_acid\_adenylation\_domain\_protein | BGC0002419 | NRP+Polyketide | 28.0 | 69.4 | 230.0 | 2.49e-60 |
| WP\_006051170.1 | non-ribosomal\_peptide\_synthetase | BGC0001999 | NRP | 33.0 | 45.4 | 230.0 | 3.3e-60 |
| CBZ42143.1 | putative\_non-ribosomal\_peptide\_synthetase | BGC0001117 | NRP | 32.0 | 45.7 | 229.0 | 3.53e-60 |
| AGD80623.1 | non-ribosomal\_peptide\_synthetase | BGC0000394 | NRP | 27.0 | 68.4 | 229.0 | 3.57e-60 |
| OKA09423.1 | non-ribosomal\_peptide\_synthetase | BGC0001459 | NRP:Glycopeptide | 30.0 | 53.0 | 229.0 | 3.6e-60 |
| CZT62792.1 | non-ribosomal\_peptide\_synthase\_involved\_in\_Hassallidin\_biosynthesis | BGC0001614 | NRP | 30.0 | 45.9 | 229.0 | 3.68e-60 |
| AZH29361.1 | amino\_acid\_adenylation\_domain-containing\_protein | BGC0001843 | NRP | 26.0 | 66.2 | 229.0 | 3.87e-60 |
| CAD91212.1 | putative\_non-ribosomal\_peptide\_synthetase,\_modules\_4-6 | BGC0000289 | NRP:Glycopeptide+Saccharide:Hybrid/tailoring saccharide | 30.0 | 51.0 | 229.0 | 3.99e-60 |
| KUM80513.1 | hypothetical\_protein | BGC0001562 | NRP | 27.0 | 70.6 | 229.0 | 4.28e-60 |
| ATV95610.1 | NRPS\_A-PCP\_didomain\_protein | BGC0001503 | Polyketide | 30.0 | 40.0 | 218.0 | 4.41e-60 |
| EJK79843.1 | amino\_acid\_adenylation\_enzyme/thioester\_reductase\_family\_protein | BGC0000436 | NRP | 27.0 | 70.0 | 229.0 | 5.27e-60 |
| QUJ09168.1 | Lon21 | BGC0002440 | NRP | 27.0 | 68.8 | 228.0 | 5.37e-60 |
| AAF99707.2 | syringopeptin\_synthetase | BGC0000438 | NRP | 29.0 | 51.2 | 229.0 | 5.65e-60 |
| AHH53506.1 | non-ribosomal\_peptide\_synthetase | BGC0000439 | NRP:Lipopeptide:Ca+-dependent lipopeptide | 29.0 | 53.0 | 229.0 | 5.74e-60 |
| EME52974.1 | non-ribosomal\_peptide\_synthetase | BGC0001460 | NRP:Glycopeptide | 30.0 | 47.9 | 218.0 | 6.21e-60 |
| ctg1\_orf17 |  | BGC0001457 | NRP | 29.0 | 52.9 | 226.0 | 6.95e-60 |
| SDF67386.1 | non-ribosomal\_peptide\_synthase\_domain\_TIGR01720/amino\_acid\_adenylation\_domain-containing\_protein | BGC0002422 | NRP | 29.0 | 45.7 | 229.0 | 7.15e-60 |
| AAY37653.1 | Amino\_acid\_adenylation | BGC0000437 | NRP | 28.0 | 50.9 | 229.0 | 7.42e-60 |
| AAN32981.1 | BarG | BGC0000962 | NRP+Polyketide:Modular type I polyketide | 31.0 | 45.9 | 228.0 | 7.8e-60 |
| ALK27915.1 | non-ribosomal\_peptide\_synthase | BGC0001233 | NRP | 27.0 | 53.2 | 229.0 | 7.97e-60 |
| QMN69934.1 | PsoC | BGC0002521 | NRP | 26.0 | 67.3 | 228.0 | 8.79e-60 |
| CAJ46692.1 | non-ribosomal\_peptide\_synthase | BGC0000969 | NRP:Cyclic depsipeptide+Polyketide:Modular type I polyketide | 28.0 | 58.3 | 228.0 | 8.91e-60 |
| AEI58879.1 | peptide\_synthetase | BGC0000455 | NRP | 30.0 | 46.4 | 216.0 | 1.05e-59 |
| QUJ09167.1 | Lon20 | BGC0002440 | NRP | 30.0 | 46.4 | 228.0 | 1.2e-59 |
| KFL51883.1 | amino\_acid\_adenylation\_protein | BGC0001711 | NRP+Polyketide | 25.0 | 68.8 | 228.0 | 1.22e-59 |
| AAZ23075.1 | peptide\_synthetase | BGC0000291 | NRP | 27.0 | 63.7 | 228.0 | 1.32e-59 |
| ABW17376.1 | PsoB | BGC0000411 | NRP | 28.0 | 45.8 | 228.0 | 1.36e-59 |
| WP\_102918835.1 | amino\_acid\_adenylation\_domain-containing\_protein | BGC0002104 | NRP+Polyketide | 30.0 | 47.9 | 217.0 | 1.5e-59 |
| ABA70582.1 | alpha-aminoadypil-cysteinyl-valine\_synthetase | BGC0000404 | NRP | 27.0 | 53.8 | 228.0 | 1.52e-59 |
| ABR12615.1 | ACV\_synthetase | BGC0000405 | NRP:Beta-lactam | 27.0 | 53.8 | 228.0 | 1.52e-59 |
| QYA95680.1 | amino\_acid\_adenylation\_domain-containing\_protein | BGC0002676 | NRP | 31.0 | 36.3 | 228.0 | 1.54e-59 |
| AAO56328.1 | non-ribosomal\_peptide\_synthetase\_SyfA | BGC0000435 | NRP | 31.0 | 46.5 | 227.0 | 1.85e-59 |
| AAZ23078.1 | peptide\_synthetase | BGC0000291 | NRP | 31.0 | 45.8 | 227.0 | 2.06e-59 |
| BAP05597.1 | calI | BGC0000967 | NRP+Polyketide:Trans-AT type I polyketide | 29.0 | 45.5 | 227.0 | 2.23e-59 |
| QBC75023.1 | non-ribosomal\_peptide\_synthetase | BGC0001968 | NRP | 29.0 | 52.6 | 227.0 | 2.28e-59 |
| CAB53322.1 | putative\_peptide\_synthetase | BGC0000325 | NRP | 30.0 | 50.2 | 227.0 | 2.58e-59 |
| BAH04173.1 | putative\_non-ribosomal\_peptide\_synthetase | BGC0000450 | NRP | 30.0 | 47.4 | 216.0 | 2.74e-59 |
| BAP05591.1 | calC | BGC0000967 | NRP+Polyketide:Trans-AT type I polyketide | 27.0 | 46.0 | 227.0 | 2.79e-59 |
| AAY89051.1 | hybrid\_nonribosomal\_peptide\_synthetase/polyketide\_synthase | BGC0001069 | NRP+Polyketide:Trans-AT type I polyketide | 29.0 | 46.7 | 227.0 | 2.8e-59 |
| ATY37589.1 | BogB | BGC0001532 | NRP | 29.0 | 46.3 | 226.0 | 2.83e-59 |
| RSO11556.1 | non-ribosomal\_peptide\_synthetase | BGC0002637 | NRP | 30.0 | 50.4 | 226.0 | 3.18e-59 |
| QNS30807.1 | hybrid\_non-ribosomal\_peptide\_synthetase/type\_I\_polyketide\_syn-thase | BGC0002509 | NRP | 27.0 | 71.0 | 226.0 | 3.2e-59 |
| NAO96318.1 | amino\_acid\_adenylation\_domain-containing\_protein | BGC0002117 | NRP | 28.0 | 48.6 | 226.0 | 3.31e-59 |
| CBJ90082.1 | Non\_Ribosomal\_peptide\_synthetase\_(-succinylbenzoate--CoA\_ligase) | BGC0001132 | NRP | 30.0 | 45.8 | 226.0 | 3.55e-59 |
| ALG65342.1 | Var7 | BGC0002416 | NRP+Polyketide | 30.0 | 45.7 | 226.0 | 3.62e-59 |
| AXA91302.1 | non-ribosomal\_peptide\_synthetase | BGC0002044 | NRP | 28.0 | 46.1 | 226.0 | 3.8e-59 |
| AET98916.1 | putative\_non-ribosomal\_peptide\_synthetase | BGC0000415 | NRP | 30.0 | 47.4 | 216.0 | 3.91e-59 |
| PHM49485.1 | Amino\_acid\_adenylation | BGC0001131 | NRP | 30.0 | 46.5 | 226.0 | 3.98e-59 |
| CAE02633.1 | surfactin\_synthetase\_C\_ | BGC0000433 | NRP:Lipopeptide | 25.0 | 62.6 | 224.0 | 4.04e-59 |
| AHH25592.1 | NRPS | BGC0000957 | NRP+Polyketide | 29.0 | 45.2 | 221.0 | 4.29e-59 |
| AAU34203.1 | mannopeptimycin\_peptide\_synthetase\_MppB | BGC0000388 | NRP | 30.0 | 47.7 | 226.0 | 4.47e-59 |
| AJW65406.1 | nonribosomal\_peptide\_synthetase | BGC0001195 | NRP+Polyketide | 27.0 | 68.1 | 225.0 | 4.92e-59 |
| QBA57736.1 | NRPS | BGC0002377 | NRP | 33.0 | 35.5 | 226.0 | 4.95e-59 |
| ABA73955.1 | putative\_non-ribosomal\_peptide\_synthetase | BGC0001842 | NRP:Lipopeptide | 30.0 | 45.4 | 226.0 | 5.01e-59 |
| ABM21571.1 | crpC | BGC0000975 | NRP+Polyketide | 27.0 | 57.4 | 225.0 | 5.15e-59 |
| WP\_050383088.1 | non-ribosomal\_peptide\_synthetase | BGC0001451 | NRP | 29.0 | 51.5 | 223.0 | 5.26e-59 |
| AEP18656.1 | WAPS1 | BGC0000461 | NRP | 31.0 | 45.1 | 226.0 | 5.49e-59 |
| NKI69295.1 | amino\_acid\_adenylation\_domain-containing\_protein | BGC0002408 | NRP | 27.0 | 52.8 | 226.0 | 5.57e-59 |
| ATW47208.1 | non-ribosomal\_peptide\_synthetase | BGC0002466 | NRP | 27.0 | 64.3 | 226.0 | 6.51e-59 |
| QVQ62856.1 | nonribosomal\_peptide\_synthase | BGC0002373 | NRP | 29.0 | 45.8 | 215.0 | 6.71e-59 |
| AFH75320.1 | nonribosomal\_peptide\_synthetase | BGC0000425 | NRP:Cyclic depsipeptide | 28.0 | 51.2 | 226.0 | 6.81e-59 |
| QDF82254.1 | non-ribosomal\_peptide\_synthetase | BGC0001980 | NRP | 29.0 | 45.4 | 225.0 | 7.39e-59 |
| CAJ34382.1 | NRPS\_protein | BGC0000445 | NRP:Cyclic depsipeptide | 27.0 | 67.8 | 224.0 | 8.23e-59 |
| CAJ34374.1 | NRPS\_protein | BGC0000445 | NRP:Cyclic depsipeptide | 32.0 | 35.6 | 225.0 | 8.56e-59 |
| WP\_013310342.1 | non-ribosomal\_peptide\_synthetase | BGC0001728 | NRP+Polyketide | 25.0 | 69.2 | 225.0 | 9.22e-59 |
| DMA15\_34345 | non-ribosomal\_peptide\_synthetase | BGC0002314 | NRP | 29.0 | 52.5 | 223.0 | 1.28e-58 |
| BAC67534.2 | arthrofactin\_synthetase\_A | BGC0000305 | NRP:Lipopeptide | 29.0 | 48.1 | 224.0 | 1.28e-58 |
| WP\_054234643.1 | non-ribosomal\_peptide\_synthetase | BGC0002014 | NRP+Polyketide | 33.0 | 37.2 | 224.0 | 1.35e-58 |
| AGA37267.1 | NRPS | BGC0000816 | NRP+Alkaloid | 27.0 | 59.3 | 224.0 | 1.38e-58 |
| ALV86867.1 | Tlo21 | BGC0001406 | NRP | 31.0 | 45.7 | 224.0 | 1.51e-58 |
| AJF34464.1 | Txo2 | BGC0001207 | NRP | 29.0 | 49.1 | 224.0 | 1.54e-58 |
| AHY86403.1 | non\_ribosomal\_peptide\_synthetase | BGC0000329 | NRP | 32.0 | 35.2 | 224.0 | 1.7e-58 |
| CAQ71828.1 | non\_ribosomal\_peptide\_synthase,\_antibiotic\_synthesis;\_contains\_3\_condensation\_domains,\_2\_AMP-acid\_ligases\_II\_domains,\_2\_PP-binding,\_Phosphopantetheine\_attachment\_site | BGC0001189 | NRP | 32.0 | 45.8 | 224.0 | 1.94e-58 |
| QBE85649.1 | BuaA | BGC0001857 | Alkaloid+NRP+Polyketide:Iterative type I polyketide | 26.0 | 78.5 | 224.0 | 2.36e-58 |
| AJQ95678.1 | polyketide\_synthase\_modules-related\_protein | BGC0002046 | NRP+Polyketide:Trans-AT type I polyketide | 29.0 | 45.9 | 224.0 | 2.5e-58 |
| OLZ52456.1 | non-ribosomal\_peptide\_synthetase | BGC0001462 | NRP:Glycopeptide | 29.0 | 52.3 | 223.0 | 2.54e-58 |
| CAA60461.1 | pipecolate\_incorporating\_enzyme | BGC0001040 | NRP+Polyketide | 29.0 | 46.5 | 223.0 | 2.55e-58 |
| AAF08796.1 | MycB | BGC0001103 | NRP+Polyketide | 24.0 | 68.3 | 224.0 | 2.56e-58 |
| ALK27914.1 | non-ribosomal\_peptide\_synthase | BGC0001233 | NRP | 29.0 | 52.8 | 224.0 | 2.57e-58 |
| ADY76664.1 | non-ribosomal\_peptide\_synthetase | BGC0000950 | NRP:Uridylpeptide+Other:Nucleoside | 33.0 | 35.0 | 218.0 | 2.59e-58 |
| ACM79806.1 | ZmaB | BGC0001059 | NRP+Polyketide | 28.0 | 45.9 | 223.0 | 2.61e-58 |
| ABL74936.1 | NRPS | BGC0001048 | NRP:Glycopeptide+Polyketide:Modular type I polyketide+Saccharide:Hybrid/tailoring saccharide | 27.0 | 69.6 | 223.0 | 2.9e-58 |
| CAE53352.1 | non-ribosomal\_peptide\_synthetase | BGC0000440 | NRP:Glycopeptide | 26.0 | 64.3 | 223.0 | 3.12e-58 |
| AXA91301.1 | non-ribosomal\_peptide\_synthetase | BGC0002044 | NRP | 29.0 | 48.1 | 223.0 | 3.47e-58 |
| AHB82058.1 | non\_ribosomal\_peptide\_synthetase | BGC0001019 | NRP+Polyketide:Modular type I polyketide | 30.0 | 45.3 | 223.0 | 4.13e-58 |
| MQQ32958.1 | amino\_acid\_adenylation\_domain-containing\_protein | BGC0002518 | NRP | 28.0 | 48.3 | 223.0 | 4.89e-58 |
| QQO98485.1 | FrzA | BGC0002146 | NRP | 32.0 | 40.5 | 219.0 | 5.05e-58 |
| CAD29794.1 | peptide\_synthetase | BGC0001015 | NRP+Polyketide | 25.0 | 68.5 | 223.0 | 5.1e-58 |
| CEO59272.1 | hypothetical\_protein | BGC0002278 | Alkaloid+NRP | 31.0 | 43.4 | 219.0 | 5.32e-58 |
| BAH43871.1 | truncated\_linear\_pentadecapeptide\_gramicidin\_synthetase\_LgrC | BGC0000367 | NRP | 33.0 | 35.8 | 223.0 | 6.02e-58 |
| AGN11881.1 | tstDEF | BGC0001114 | NRP+Polyketide | 30.0 | 47.1 | 223.0 | 6.24e-58 |
| AJD77023.1 | IkaA | BGC0001435 | NRP+Polyketide:Iterative type I polyketide | 25.0 | 68.6 | 222.0 | 6.39e-58 |
| ABE35422.1 | Non-ribosomal\_peptide\_synthase | BGC0002421 | NRP | 28.0 | 50.9 | 222.0 | 6.44e-58 |
| KFL51885.1 | amino\_acid\_adenylation\_protein | BGC0001711 | NRP+Polyketide | 28.0 | 63.1 | 221.0 | 6.86e-58 |
| ABS74209.1 | fengycin\_synthetase\_A | BGC0001095 | NRP | 25.0 | 64.4 | 222.0 | 7.52e-58 |
| CAQ43084.1 | non\_ribosomal\_polypeptide\_synthetase | BGC0000970 | NRP+Polyketide:Modular type I polyketide | 29.0 | 46.5 | 221.0 | 8.29e-58 |
| QCP68969.1 | VatN | BGC0002296 | NRP+Polyketide | 27.0 | 57.5 | 221.0 | 8.73e-58 |
| KJY94240.1 | peptide\_synthetase | BGC0002691 | NRP | 26.0 | 65.6 | 221.0 | 1.17e-57 |
| CAB38517.1 | CDA\_peptide\_synthetase\_II\_(CdaPs2) | BGC0000315 | NRP:Lipopeptide:Ca+-dependent lipopeptide | 33.0 | 35.7 | 221.0 | 1.18e-57 |
| AHZ34240.1 | CipC | BGC0001389 | NRP | 29.0 | 45.8 | 220.0 | 1.32e-57 |
| CAQ71827.1 | non\_ribosomal\_peptide\_synthase,\_antibiotic\_synthesis;\_contains\_1\_condensation\_domain,\_1\_AMP-acid\_ligases\_II\_domain | BGC0001189 | NRP | 29.0 | 51.9 | 218.0 | 1.46e-57 |
| QUS58938.1 | amino\_acid\_adenylation\_domain-containing\_protein | BGC0002123 | NRP+Polyketide | 26.0 | 69.4 | 221.0 | 1.51e-57 |
| AAG29780.1 | peptide\_synthetase-like\_protein | BGC0000833 | Saccharide:Hybrid/tailoring saccharide+Other:Aminocoumarin | 29.0 | 45.6 | 211.0 | 1.55e-57 |
| AHD05614.1 | putative\_non-ribosomal\_peptide\_ligase/\_polyketide\_synthase\_hybrid | BGC0001033 | NRP+Polyketide | 27.0 | 45.3 | 221.0 | 1.72e-57 |
| QWM97319.1 | non-ribosomal\_peptide\_synthetase | BGC0002384 | NRP | 29.0 | 47.8 | 221.0 | 1.89e-57 |
| ABC36450.1 | peptide\_synthetase-like\_protein | BGC0000386 | NRP:NRP siderophore | 29.0 | 51.4 | 221.0 | 1.95e-57 |
| ABA73954.1 | putative\_non-ribosomal\_peptide\_synthetase | BGC0001842 | NRP:Lipopeptide | 28.0 | 50.7 | 220.0 | 1.97e-57 |
| ADH04680.1 | hybrid\_polyketide\_synthase/non-ribosomal\_peptide\_synthetase | BGC0001344 | NRP+Polyketide | 29.0 | 49.8 | 221.0 | 2e-57 |
| OKA09664.1 | non-ribosomal\_peptide\_synthetase | BGC0001459 | NRP:Glycopeptide | 29.0 | 47.9 | 210.0 | 2.02e-57 |
| ABM34278.1 | amino\_acid\_adenylation\_domain\_protein | BGC0002419 | NRP+Polyketide | 31.0 | 46.4 | 218.0 | 2.08e-57 |
| OLZ50886.1 | non-ribosomal\_peptide\_synthetase | BGC0001461 | NRP:Glycopeptide | 29.0 | 52.3 | 220.0 | 2.27e-57 |
| AYA22333.1 | KerD | BGC0001955 | NRP | 29.0 | 52.3 | 220.0 | 2.27e-57 |
| ABS74179.1 | bacillomycin\_D\_synthetase\_C | BGC0001090 | Polyketide+NRP:Lipopeptide | 28.0 | 46.6 | 220.0 | 2.28e-57 |
| AAT01807.1 | non-ribosomal\_peptide\_synthetase | BGC0000365 | NRP | 33.0 | 37.7 | 220.0 | 2.95e-57 |
| ARO38317.1 | nonribosomal\_peptide\_synthetase | BGC0001560 | NRP+Polyketide | 28.0 | 50.4 | 220.0 | 3.08e-57 |
| BAY02129.1 | barbamide\_biosynthesis\_protein\_BarG | BGC0002532 | NRP+Polyketide | 27.0 | 48.4 | 218.0 | 3.1e-57 |
| AGZ03651.1 | sevB | BGC0000426 | NRP | 28.0 | 47.3 | 220.0 | 3.1e-57 |
| ACU71638.1 | peptide\_synthetase-like\_protein | BGC0001154 | Other | 29.0 | 46.4 | 210.0 | 3.26e-57 |
| DAC76736.1 | non-ribosomal\_peptide\_synthetase | BGC0001885 | NRP+Polyketide | 27.0 | 59.6 | 219.0 | 3.27e-57 |
| AOA33121.1 | Nonribosomal\_peptide\_synthetase | BGC0001346 | NRP:Cyclic depsipeptide | 29.0 | 45.9 | 219.0 | 3.4e-57 |
| ATP76239.1 | NdaF | BGC0001705 | NRP+Polyketide | 25.0 | 69.3 | 220.0 | 3.43e-57 |
| BAE98162.1 | putative\_non-ribosomal\_peptide\_synthetase | BGC0000339 | NRP | 30.0 | 47.1 | 210.0 | 3.73e-57 |
| ANI24100.1 | nonribosomal\_peptide\_synthetase | BGC0001235 | NRP+Polyketide | 29.0 | 57.0 | 220.0 | 3.74e-57 |
| BCJ07529.1 | hypothetical\_protein | BGC0002379 | NRP | 32.0 | 43.1 | 219.0 | 4.48e-57 |
| AAO62582.1 | polyketide\_synthase\_peptide\_sythetase\_fusion\_protein | BGC0001016 | NRP+Polyketide | 25.0 | 69.0 | 219.0 | 4.51e-57 |
| ABS90476.1 | NRPS | BGC0001106 | NRP+Polyketide | 28.0 | 50.8 | 218.0 | 4.68e-57 |
| WP\_050383084.1 | non-ribosomal\_peptide\_synthetase | BGC0001451 | NRP | 29.0 | 52.6 | 219.0 | 5.04e-57 |
| mycF | polyketide\_synthase | BGC0002055 | NRP+Polyketide:Trans-AT type I polyketide | 24.0 | 69.4 | 219.0 | 7.35e-57 |
| AHF21229.1 | TriE | BGC0000449 | NRP | 26.0 | 66.5 | 219.0 | 7.98e-57 |
| AAF86395.1 | FkbP | BGC0000994 | NRP+Polyketide | 30.0 | 45.8 | 218.0 | 8.58e-57 |
| AAF00958.1 | mcyE | BGC0001017 | NRP+Polyketide:Modular type I polyketide | 26.0 | 68.3 | 218.0 | 1.02e-56 |
| KYQ85937.1 | hypothetical\_protein | BGC0002437 | NRP | 30.0 | 44.9 | 218.0 | 1.03e-56 |
| QOJ72663.1 | XenE | BGC0002505 | Polyketide+NRP | 26.0 | 71.6 | 218.0 | 1.07e-56 |
| AEE88284.1 | CurF | BGC0000976 | NRP+Polyketide:Modular type I polyketide | 28.0 | 47.0 | 218.0 | 1.29e-56 |
| AAT70101.1 | CurF | BGC0001165 | NRP+Polyketide:Modular type I polyketide | 28.0 | 47.0 | 218.0 | 1.29e-56 |
| MCC5036785.1 | amino\_acid\_adenylation\_domain-containing\_protein | BGC0002638 | NRP | 31.0 | 45.8 | 218.0 | 1.47e-56 |
| WP\_039806850.1 | non-ribosomal\_peptide\_synthetase | BGC0002001 | NRP+Polyketide | 26.0 | 64.0 | 218.0 | 1.53e-56 |
| QKM21619.1 | non-ribosomal\_peptide\_synthetase | BGC0002351 | NRP | 30.0 | 46.3 | 218.0 | 1.7e-56 |
| AAY93445.1 | non-ribosomal\_peptide\_synthetase\_PvdL | BGC0000413 | NRP | 26.0 | 67.8 | 218.0 | 1.89e-56 |
| BBA21071.1 | putative\_non-ribosomal\_peptide\_synthetase | BGC0001740 | NRP+Polyketide | 29.0 | 51.1 | 215.0 | 2.14e-56 |
| ABD65958.1 | nonribosomal\_peptide\_synthetase | BGC0000341 | NRP | 28.0 | 46.1 | 218.0 | 2.17e-56 |
| AAF67501.2 | peptide\_synthetase-like\_protein | BGC0000834 | Saccharide:Hybrid/tailoring saccharide+Other:Aminocoumarin | 30.0 | 45.1 | 207.0 | 2.33e-56 |
| QEO75076.1 | AMP-dependent\_synthetase\_and\_ligase | BGC0002079 | NRP:Cyclic depsipeptide | 32.0 | 45.9 | 214.0 | 2.57e-56 |
| AGN74876.1 | nonribosomal\_peptide\_synthetase | BGC0000459 | NRP:Cyclic depsipeptide+Polyketide:Trans-AT type I polyketide | 31.0 | 45.0 | 217.0 | 2.66e-56 |
| AAQ59905.1 | synthetase\_CbsF | BGC0002680 | NRP | 30.0 | 46.5 | 217.0 | 2.78e-56 |
| CBD77746.1 | non-ribosomal\_peptide\_synthetase/polyketide\_synthase | BGC0000974 | NRP+Polyketide | 28.0 | 64.6 | 217.0 | 2.83e-56 |
| AKP45395.1 | CysG | BGC0001413 | NRP | 25.0 | 68.8 | 216.0 | 2.84e-56 |
| ABC35522.1 | thiotemplate\_mechanism\_natural\_product\_synthetase | BGC0000186 | NRP+Polyketide:Modular type I polyketide | 26.0 | 68.7 | 217.0 | 3.16e-56 |
| CAL17540.1 | peptide\_synthetase,\_putative | BGC0002465 | NRP | 30.0 | 44.6 | 216.0 | 3.41e-56 |
| WP\_013310343.1 | non-ribosomal\_peptide\_synthetase | BGC0001728 | NRP+Polyketide | 27.0 | 66.2 | 216.0 | 3.57e-56 |
| QYC40289.1 | A50926\_NRPS,\_modules\_4-5-6 | BGC0002344 | NRP | 28.0 | 53.3 | 216.0 | 4.2e-56 |
| AQZ69229.1 | hypothetical\_protein | BGC0001635 | NRP+Polyketide | 27.0 | 53.0 | 216.0 | 4.32e-56 |
| QDG75035.1 | mixed\_type\_I\_polyketide\_synthase\_-\_peptide\_synthetase | BGC0002068 | NRP+Polyketide | 26.0 | 68.1 | 216.0 | 4.85e-56 |
| ADJ63842.1 | Serobactin\_synthetase | BGC0000424 | NRP:NRP siderophore | 29.0 | 53.0 | 216.0 | 4.93e-56 |
| EME52990.1 | amino\_acid\_adenylation\_protein | BGC0001460 | NRP:Glycopeptide | 29.0 | 53.5 | 216.0 | 5.01e-56 |
| WP\_044618979.1 | non-ribosomal\_peptide\_synthetase | BGC0001791 | NRP | 29.0 | 44.4 | 216.0 | 5.43e-56 |
| AAZ23077.1 | peptide\_synthetase | BGC0000291 | NRP | 26.0 | 63.9 | 216.0 | 5.9e-56 |
| AUD11994.1 | OrbI | BGC0001721 | NRP | 29.0 | 46.1 | 216.0 | 6.58e-56 |
| RAT94091.1 | NRPS | BGC0001469 | NRP | 28.0 | 47.1 | 213.0 | 6.81e-56 |
| CAL80821.1 | sylD-like\_NRPS/PKS | BGC0000997 | NRP+Polyketide | 26.0 | 65.6 | 216.0 | 7.3e-56 |
| ADH01485.1 | putative\_mixed\_polyketide\_synthase/non-ribosomal\_peptide\_synthetase | BGC0000995 | NRP+Polyketide | 30.0 | 44.6 | 215.0 | 7.48e-56 |
| AAV97877.1 | OnnI | BGC0001105 | NRP+Polyketide:Trans-AT type I polyketide | 27.0 | 62.2 | 216.0 | 7.67e-56 |
| ABV79988.1 | ApnD | BGC0000301 | NRP | 27.0 | 46.2 | 214.0 | 9.19e-56 |
| AEI58866.1 | peptide\_synthetase | BGC0000455 | NRP | 29.0 | 50.5 | 215.0 | 9.49e-56 |
| EME52989.1 | amino\_acid\_adenylation\_protein | BGC0001460 | NRP:Glycopeptide | 29.0 | 50.7 | 215.0 | 9.49e-56 |
| CAA11795.1 | PCZA363.4 | BGC0000322 | NRP | 28.0 | 51.4 | 215.0 | 9.51e-56 |
| ACO78745.1 | Non-ribosomal\_peptide\_synthase:Amino\_acid\_adenylation | BGC0002433 | NRP | 27.0 | 64.4 | 215.0 | 9.67e-56 |
| CAQ46279.1 | putative\_enterobactin\_synthetase\_component\_F | BGC0002689 | NRP | 30.0 | 47.0 | 214.0 | 9.74e-56 |
| ctg1\_orf20 |  | BGC0001767 | NRP | 27.0 | 64.8 | 215.0 | 1.01e-55 |
| BAV56271.1 |  | BGC0001657 | NRP | 31.0 | 45.3 | 215.0 | 1.06e-55 |
| ABM34276.1 | amino\_acid\_adenylation\_domain\_protein | BGC0002419 | NRP+Polyketide | 31.0 | 46.1 | 214.0 | 1.12e-55 |
| APD26279.1 | PtmA | BGC0001726 | NRP+Polyketide | 25.0 | 68.5 | 215.0 | 1.13e-55 |
| CAR51994.1 | ornibactin\_biosynthesis\_non-ribosomal\_peptide\_synthase | BGC0002569 | NRP | 30.0 | 46.2 | 215.0 | 1.15e-55 |
| simA |  | BGC0000334 | NRP | 25.0 | 64.6 | 215.0 | 1.17e-55 |
| AAZ55900.1 | non-ribosomal\_peptide\_synthase:Amino\_acid\_adenylation | BGC0000359 | NRP | 34.0 | 35.1 | 215.0 | 1.2e-55 |
| AHB82069.1 | non\_ribosomal\_peptide\_synthetase | BGC0001231 | NRP+Polyketide:Modular type I polyketide | 30.0 | 45.6 | 214.0 | 1.2e-55 |
| AIZ66877.1 | nonribosomal\_peptide\_synthetase | BGC0002666 | NRP+Alkaloid | 30.0 | 48.3 | 205.0 | 1.27e-55 |
| UMM61373.1 | Tsk12 | BGC0002661 | NRP | 30.0 | 45.7 | 213.0 | 1.29e-55 |
| AMK48228.1 | nonribosomal\_peptide\_synthetase | BGC0001351 | NRP | 27.0 | 63.7 | 212.0 | 1.31e-55 |
| BAX64244.1 | NRPS | BGC0001623 | NRP+Polyketide | 29.0 | 50.4 | 214.0 | 1.33e-55 |
| AMK48227.1 | nonribosomal\_peptide\_synthetas | BGC0001351 | NRP | 28.0 | 52.0 | 214.0 | 1.35e-55 |
| AIC32693.1 | FR9DEF | BGC0001113 | NRP+Polyketide | 30.0 | 44.6 | 215.0 | 1.43e-55 |
| ADQ55475.1 | NRPS | BGC0000350 | NRP:Beta-lactam | 26.0 | 48.6 | 213.0 | 1.65e-55 |
| AAF00962.1 | mcyC | BGC0001017 | NRP+Polyketide:Modular type I polyketide | 29.0 | 41.0 | 213.0 | 1.67e-55 |
| ADF88262.1 | mixed\_nonribosomal\_peptide\_synthetase/\_polyketide\_synthase | BGC0000979 | NRP+Polyketide | 28.0 | 48.8 | 213.0 | 2.02e-55 |
| E0F75\_025360 | amino\_acid\_adenylation\_domain-containing\_protein | BGC0002340 | NRP+Other | 30.0 | 49.3 | 212.0 | 2.05e-55 |
| CAG15011.1 | peptide\_synthetase,\_module\_4-6 | BGC0000441 | NRP | 28.0 | 51.7 | 214.0 | 2.15e-55 |
| AAC06347.1 | bacitracin\_synthetase\_2 | BGC0000310 | NRP | 28.0 | 44.8 | 214.0 | 2.34e-55 |
| SJZ83675.1 | non-ribosomal\_peptide\_synthase\_domain\_TIGR01720/amino\_acid\_adenylation\_domain-containing\_protein/thioester\_reductase\_domain-containing\_protein | BGC0002660 | NRP | 28.0 | 46.0 | 214.0 | 2.37e-55 |
| CAJ45639.1 | vanchrobactin\_non\_ribosomal\_peptide\_synthetase | BGC0000454 | NRP | 29.0 | 45.9 | 214.0 | 2.44e-55 |
| ABX60161.1 | mixed\_NRPS/PKS | BGC0000978 | NRP+Alkaloid+Polyketide:Modular type I polyketide | 28.0 | 48.8 | 214.0 | 2.48e-55 |
| ALG65313.1 | Cal23 | BGC0001297 | NRP | 30.0 | 44.2 | 204.0 | 2.54e-55 |
| AAP92491.1 | nonribosomal\_peptide\_synthetase | BGC0000458 | NRP | 30.0 | 46.1 | 213.0 | 2.67e-55 |
| AGS77309.1 | NRPS\_modules\_4-6 | BGC0001178 | NRP:Glycopeptide | 30.0 | 45.6 | 214.0 | 2.82e-55 |
| ABC37099.1 | non-ribosomal\_peptide\_synthetase,\_putative | BGC0000386 | NRP:NRP siderophore | 30.0 | 46.1 | 213.0 | 2.94e-55 |
| ACM68684.1 | AerB | BGC0000298 | NRP | 25.0 | 47.9 | 213.0 | 3.37e-55 |
| ADF88265.1 | mixed\_nonribosomal\_peptide\_synthetase/\_polyketide\_synthase | BGC0000980 | NRP+Polyketide | 28.0 | 48.8 | 212.0 | 3.5e-55 |
| ALV82384.1 | CDA\_peptide\_synthetase\_II | BGC0001370 | NRP | 33.0 | 35.0 | 213.0 | 3.57e-55 |
| AQX14497.1 | monobactam\_NRPS\_scaffold\_4 | BGC0001672 | NRP | 25.0 | 64.6 | 213.0 | 3.89e-55 |
| AHZ34233.1 | CifB | BGC0000323 | NRP:Lipopeptide | 30.0 | 41.7 | 213.0 | 4.04e-55 |
| CEK23367.1 | putative\_Ornithine\_racemase | BGC0001716 | NRP | 27.0 | 46.2 | 213.0 | 4.17e-55 |
| DAD54486.1 | trans-acyltransferase\_polyketide\_synthase | BGC0002059 | NRP+Polyketide:Trans-AT type I polyketide | 30.0 | 46.3 | 213.0 | 4.27e-55 |
| ADF88279.1 | mixed\_NRPS/PKS | BGC0000981 | NRP+Polyketide | 28.0 | 48.8 | 213.0 | 4.27e-55 |
| AEW98135.1 | amino\_acid\_adenylation\_domain-containing\_protein | BGC0002642 | Alkaloid | 29.0 | 47.9 | 209.0 | 4.43e-55 |
| AAZ55898.1 | amino\_acid\_adenylation | BGC0000359 | NRP | 28.0 | 64.3 | 211.0 | 5.59e-55 |
| AZH23792.1 | MgcJ | BGC0001970 | NRP+Polyketide | 27.0 | 57.9 | 212.0 | 5.78e-55 |
| AAK89719.2 | peptide\_synthetase,\_siderophore\_biosynthesis\_protein | BGC0002107 | NRP+Polyketide | 32.0 | 35.8 | 212.0 | 6.88e-55 |
| ASX95241.1 | IlaS | BGC0001620 | NRP+Polyketide | 31.0 | 37.0 | 213.0 | 7.33e-55 |
| ALD82526.1 | non-ribosomal\_peptide\_synthase | BGC0001212 | NRP+Polyketide | 26.0 | 64.1 | 212.0 | 7.92e-55 |
| CBA11570.1 | non-ribosomal\_peptide\_synthetase | BGC0001046 | NRP+Polyketide:Modular type I polyketide+Saccharide:Hybrid/tailoring saccharide | 30.0 | 41.6 | 202.0 | 8.16e-55 |
| QDJ74274.1 | non-ribosomal\_peptide\_synthetase | BGC0002109 | NRP | 29.0 | 47.2 | 209.0 | 8.54e-55 |
| BBA20967.1 | nonribosomal\_peptide\_synthetase | BGC0001763 | NRP+Polyketide | 31.0 | 37.0 | 212.0 | 9.61e-55 |
| AAF63833.1 | PstD | BGC0000362 | NRP | 28.0 | 70.1 | 211.0 | 1.06e-54 |
| MCC5036783.1 | amino\_acid\_adenylation\_domain-containing\_protein | BGC0002638 | NRP | 30.0 | 45.3 | 209.0 | 1.06e-54 |
| OKA09424.1 | non-ribosomal\_peptide\_synthetase | BGC0001459 | NRP:Glycopeptide | 27.0 | 63.5 | 212.0 | 1.1e-54 |
| AAN65224.1 | peptide\_synthetase-like\_protein | BGC0000832 | Saccharide:Hybrid/tailoring saccharide+Other:Aminocoumarin | 29.0 | 45.6 | 202.0 | 1.13e-54 |
| CAF05648.1 | TubC\_protein | BGC0001053 | NRP+Polyketide | 31.0 | 35.2 | 211.0 | 1.2e-54 |
| AQM37584.1 | nonribosomal\_peptide\_synthetase | BGC0001424 | NRP:Cyclic depsipeptide+Polyketide:Iterative type I polyketide | 33.0 | 42.5 | 211.0 | 1.36e-54 |
| MCC5036786.1 | amino\_acid\_adenylation\_domain-containing\_protein | BGC0002638 | NRP | 27.0 | 52.1 | 211.0 | 1.42e-54 |
| AJF34463.1 | Txo1 | BGC0001207 | NRP | 25.0 | 68.3 | 211.0 | 1.6e-54 |
| CCM44337.1 | Nonribosomal\_peptide\_synthetase | BGC0001056 | NRP+Polyketide:Modular type I polyketide+Polyketide:PUFA synthase or related polyketide | 29.0 | 57.5 | 211.0 | 1.84e-54 |
| AAC68816.1 | FK506\_peptide\_synthetase | BGC0000353 | NRP | 30.0 | 45.7 | 210.0 | 2.09e-54 |
| ABD65957.1 | nonribosomal\_peptide\_synthetase | BGC0000341 | NRP | 26.0 | 62.9 | 211.0 | 2.13e-54 |
| MCC5036784.1 | amino\_acid\_adenylation\_domain-containing\_protein | BGC0002638 | NRP | 28.0 | 50.9 | 210.0 | 2.18e-54 |
| AAL15600.1 | SimH | BGC0000270 | Polyketide | 33.0 | 35.2 | 207.0 | 2.73e-54 |
| AAK06804.1 | Tyroxyl-AMP-forming\_enzyme | BGC0001072 | Saccharide+Polyketide:Modular type I polyketide+Polyketide:Type II polyketide+Other:Aminocoumarin | 33.0 | 35.2 | 207.0 | 2.95e-54 |
| QCE43602.1 | nonribosomal\_peptide\_synthetase\_(NRPS),\_subunit\_1 | BGC0001834 | NRP | 29.0 | 47.1 | 210.0 | 3.11e-54 |
| AAY32966.1 | DszC | BGC0001093 | NRP+Polyketide | 28.0 | 51.4 | 210.0 | 3.19e-54 |
| BAV57451.1 | NRPS\_(A-PCP) | BGC0001818 | NRP | 28.0 | 47.5 | 201.0 | 3.37e-54 |
| WP\_084702182.1 | non-ribosomal\_peptide\_synthetase | BGC0001211 | NRP | 29.0 | 51.0 | 210.0 | 3.7e-54 |
| DAB41478.1 | nonribosomal\_peptide\_synthetase | BGC0001230 | NRP:Cyclic depsipeptide+Polyketide:Modular type I polyketide | 26.0 | 68.5 | 209.0 | 3.81e-54 |
| AAX31558.1 | peptide\_synthetase\_2 | BGC0000336 | NRP | 30.0 | 46.4 | 210.0 | 4.85e-54 |
| QUS58937.1 | non-ribosomal\_peptide\_synthetase | BGC0002123 | NRP+Polyketide | 27.0 | 57.8 | 208.0 | 5.28e-54 |
| BCJ07531.1 | hypothetical\_protein | BGC0002379 | NRP | 31.0 | 39.6 | 207.0 | 6e-54 |
| BAP16689.1 | nonribosomal\_peptide\_synthetase | BGC0000376 | NRP | 28.0 | 46.6 | 208.0 | 6.21e-54 |
| CAJ96472.1 | non-ribosomal\_peptide\_synthetase | BGC0000330 | NRP:NRP siderophore | 28.0 | 50.1 | 209.0 | 6.67e-54 |
| WP\_050383082.1 | non-ribosomal\_peptide\_synthetase | BGC0001451 | NRP | 28.0 | 52.4 | 209.0 | 6.71e-54 |
| CAE02631.1 | surfactin\_synthetase\_B\_ | BGC0000433 | NRP:Lipopeptide | 28.0 | 40.3 | 209.0 | 7.07e-54 |
| AXG47411.1 | hybrid\_non-ribosomal\_peptide\_synthetase/type\_I\_polyketide\_synthase | BGC0002715 | NRP+Polyketide | 29.0 | 45.9 | 209.0 | 7.27e-54 |
| WP\_013184322.1 | non-ribosomal\_peptide\_synthetase | BGC0001692 | NRP | 24.0 | 65.5 | 208.0 | 7.28e-54 |
| EWS95124.1 | hypothetical\_protein | BGC0000306 | NRP:Lipopeptide | 33.0 | 34.8 | 209.0 | 7.48e-54 |
| AZM57024.1 | non-ribosomal\_peptide\_synthetase | BGC0002314 | NRP | 27.0 | 45.9 | 209.0 | 7.66e-54 |
| KJY94239.1 | thioester\_reductase | BGC0002691 | NRP | 25.0 | 65.1 | 208.0 | 7.96e-54 |
| XP\_003044554.1 | uncharacterized\_protein | BGC0001768 | NRP | 27.0 | 64.4 | 209.0 | 8.05e-54 |
| AEW31020.1 | plipastatin\_synthetase | BGC0000407 | NRP | 31.0 | 36.0 | 208.0 | 1.05e-53 |
| ACM79810.1 | ZmaO | BGC0001059 | NRP+Polyketide | 28.0 | 44.0 | 207.0 | 1.08e-53 |
| CAQ34918.1 | nonribosomal\_peptide\_synthetase/\_polyketide\_synthase | BGC0000986 | NRP+Polyketide | 28.0 | 47.8 | 208.0 | 1.13e-53 |
| AOZ21320.1 | SulM | BGC0001790 | NRP | 25.0 | 69.4 | 208.0 | 1.13e-53 |
| CAJ34367.1 | NRPS\_protein | BGC0000445 | NRP:Cyclic depsipeptide | 29.0 | 45.6 | 199.0 | 1.14e-53 |
| AIS24862.1 | dst18 | BGC0001147 | NRP | 31.0 | 46.0 | 206.0 | 1.2e-53 |
| CBJ79916.1 | putative\_Ornithine\_racemase | BGC0001133 | NRP | 28.0 | 47.7 | 208.0 | 1.32e-53 |
| QBM78313.1 | non-ribosomal\_peptide\_synthatase | BGC0002542 | Polyketide+NRP | 26.0 | 46.8 | 206.0 | 1.38e-53 |
| antaC | NRPS | BGC0001455 | NRP+Polyketide | 32.0 | 36.3 | 208.0 | 1.48e-53 |
| QCP68975.1 | VatS | BGC0002296 | NRP+Polyketide | 27.0 | 57.8 | 207.0 | 1.54e-53 |
| AHI59110.1 | locillomycin\_synthase\_C | BGC0001005 | NRP+Polyketide | 23.0 | 68.1 | 207.0 | 1.59e-53 |
| BAX90000.1 | Non-ribosomal\_peptide\_synthetase | BGC0001628 | NRP | 34.0 | 36.1 | 208.0 | 1.63e-53 |
| AJI44167.1 | long-chain-fatty-acid-CoA\_ligase | BGC0001193 | NRP | 29.0 | 47.2 | 207.0 | 1.67e-53 |
| WP\_050383094.1 | non-ribosomal\_peptide\_synthetase | BGC0001451 | NRP | 28.0 | 51.1 | 206.0 | 1.69e-53 |
| CAG23960.2 | hybrid\_NRPS/PKS\_protein | BGC0001089 | Polyketide+NRP | 23.0 | 69.5 | 208.0 | 1.79e-53 |
| QWM97862.1 | hybrid\_non-ribosomal\_peptide\_synthetase/type\_I\_polyketide\_synthase | BGC0002434 | Polyketide+NRP | 27.0 | 68.8 | 207.0 | 2e-53 |
| ctg3\_15 |  | BGC0001853 | NRP+Polyketide:Modular type I polyketide | 28.0 | 45.8 | 199.0 | 2.19e-53 |
| RSO11555.1 | non-ribosomal\_peptide\_synthetase | BGC0002637 | NRP | 30.0 | 46.6 | 206.0 | 2.56e-53 |
| EDY47118.1 | N-(5-amino-5-carboxypentanoyl)-L-cysteinyl-D-\_valine\_synthase | BGC0000319 | NRP:Beta-lactam | 29.0 | 45.9 | 207.0 | 2.79e-53 |
| AKC54422.1 | fumosorinone\_biosynthesis\_polyketide\_synthase | BGC0001218 | NRP+Polyketide | 27.0 | 69.9 | 207.0 | 2.88e-53 |
| AAX31557.1 | peptide\_synthetase\_1 | BGC0000336 | NRP | 30.0 | 46.0 | 207.0 | 3.12e-53 |
| AIG79243.1 | Non-ribosomal\_peptide\_synthetase | BGC0000419 | Saccharide+NRP:Glycopeptide | 29.0 | 47.5 | 206.0 | 3.52e-53 |
| AAY91419.3 | non-ribosomal\_peptide\_synthetase\_OfaA | BGC0000399 | NRP:Cyclic depsipeptide | 32.0 | 36.0 | 206.0 | 3.6e-53 |
| AAG02356.1 | peptide\_synthetase\_NRPS7 | BGC0000963 | NRP:Glycopeptide+Polyketide:Modular type I polyketide+Saccharide:Hybrid/tailoring saccharide | 27.0 | 63.5 | 204.0 | 3.72e-53 |
| ABW00331.1 | amino\_acid\_adenylation\_domain | BGC0000333 | NRP | 33.0 | 35.9 | 207.0 | 4.29e-53 |
| ATQ39428.1 | cyclosporin\_C\_synthetase | BGC0001565 | NRP | 26.0 | 64.4 | 207.0 | 4.57e-53 |
| QBC75021.1 | non-ribosomal\_peptide\_synthetase | BGC0001968 | NRP | 27.0 | 62.8 | 206.0 | 5.57e-53 |
| ADZ24995.1 | non-ribosomal\_peptide\_synthase/polyketide\_synthase | BGC0000380 | NRP+Polyketide:Modular type I polyketide | 29.0 | 45.7 | 206.0 | 5.8e-53 |
| AZM58102.1 | non-ribosomal\_peptide\_synthetase | BGC0002314 | NRP | 30.0 | 40.1 | 206.0 | 6.41e-53 |
| ANG60379.1 | nonribosomal\_peptide\_synthetase\_BudA | BGC0001434 | NRP | 29.0 | 41.0 | 204.0 | 7.13e-53 |
| CAJ76290.1 | putative\_non-ribosomal\_peptide\_synthase | BGC0000972 | NRP+Polyketide:Modular type I polyketide+Polyketide:Trans-AT type I polyketide | 29.0 | 46.2 | 205.0 | 8.29e-53 |
| AIG79241.1 | Hypothetical\_protein | BGC0000419 | Saccharide+NRP:Glycopeptide | 27.0 | 52.2 | 206.0 | 8.44e-53 |
| BCJ07599.1 | hypothetical\_protein | BGC0002379 | NRP | 31.0 | 40.6 | 203.0 | 8.56e-53 |
| AAX31559.1 | peptide\_synthetase\_3 | BGC0000336 | NRP | 30.0 | 46.9 | 205.0 | 8.83e-53 |
| ANS62967.1 | non-ribosomal\_peptide\_synthase/amino\_acid\_adenylation\_enzyme | BGC0001567 | NRP | 33.0 | 35.0 | 205.0 | 9.26e-53 |
| CAL69889.1 | RhiB\_protein | BGC0001112 | NRP+Polyketide:Trans-AT type I polyketide | 28.0 | 42.8 | 206.0 | 9.47e-53 |
| ADN43685.1 | PKS-NRPS | BGC0001136 | NRP+Polyketide:Iterative type I polyketide | 26.0 | 64.8 | 205.0 | 1.12e-52 |
| QTT72100.1 | non-ribosomal\_peptide\_synthetase | BGC0002350 | NRP+Polyketide+Saccharide | 27.0 | 63.5 | 203.0 | 1.14e-52 |
| QEO74981.1 | omn6 | BGC0002078 | NRP:Cyclic depsipeptide | 33.0 | 37.2 | 205.0 | 1.14e-52 |
| mycH | polyketide\_synthase | BGC0002055 | NRP+Polyketide:Trans-AT type I polyketide | 24.0 | 68.5 | 205.0 | 1.25e-52 |
| EWS95122.1 | hypothetical\_protein | BGC0000306 | NRP:Lipopeptide | 29.0 | 52.2 | 205.0 | 1.39e-52 |
| BAH43869.1 | linear\_pentadecapeptide\_gramicidin\_synthetase\_LgrA | BGC0000367 | NRP | 27.0 | 45.6 | 204.0 | 1.48e-52 |
| CCA29202.1 | non-ribosomal\_peptide\_synthetase | BGC0000955 | NRP+Polyketide:Modular type I polyketide | 28.0 | 45.7 | 204.0 | 1.53e-52 |
| ACG60772.1 | NRPS(C/A/PCP/Cy/A/PCP/Cy) | BGC0001058 | NRP:Glycopeptide+Polyketide:Modular type I polyketide+Saccharide:Hybrid/tailoring saccharide | 29.0 | 46.1 | 204.0 | 1.62e-52 |
| WP\_019032753.1 | non-ribosomal\_peptide\_synthetase | BGC0001331 | NRP:Cyclic depsipeptide+Polyketide:Modular type I polyketide | 28.0 | 58.1 | 204.0 | 1.68e-52 |
| QCP68971.1 | VatR | BGC0002296 | NRP+Polyketide | 25.0 | 52.4 | 204.0 | 1.68e-52 |
| BCJ07600.1 | hypothetical\_protein | BGC0002379 | NRP | 32.0 | 36.3 | 198.0 | 2.02e-52 |
| UHJ79951.1 | non-ribosomal\_peptide\_synthetase | BGC0002654 | NRP | 28.0 | 45.6 | 204.0 | 2.06e-52 |
| EHA27898.1 | hypothetical\_protein | BGC0002171 | NRP+Polyketide | 29.0 | 45.7 | 204.0 | 2.16e-52 |
| AHD05617.1 | putative\_non-ribosomal\_peptide\_ligase\_domain\_protein | BGC0001033 | NRP+Polyketide | 29.0 | 46.5 | 203.0 | 2.33e-52 |
| WP\_051700112.1 | non-ribosomal\_peptide\_synthetase | BGC0001368 | NRP | 33.0 | 38.5 | 202.0 | 2.43e-52 |
| AID65222.1 | putative\_aspartate\_racemase | BGC0000335 | NRP+Polyketide | 26.0 | 69.1 | 204.0 | 2.54e-52 |
| PYH50506.1 | hypothetical\_protein | BGC0002275 | NRP+Polyketide | 29.0 | 45.7 | 204.0 | 2.78e-52 |
| AIE77059.1 | peptide\_synthetase | BGC0000418 | NRP | 27.0 | 53.1 | 204.0 | 3.28e-52 |
| APZ78846.1 | nonribosomal\_peptide\_synthetase | BGC0001431 | NRP:Cyclic depsipeptide+Polyketide:Iterative type I polyketide | 32.0 | 35.1 | 204.0 | 3.39e-52 |
| AKA59436.1 | non-ribosomal\_peptide\_synthetase | BGC0001202 | NRP+Polyketide | 32.0 | 38.8 | 203.0 | 3.59e-52 |
| AAG02364.1 | peptide\_synthetase\_NRPS2-1 | BGC0000963 | NRP:Glycopeptide+Polyketide:Modular type I polyketide+Saccharide:Hybrid/tailoring saccharide | 29.0 | 46.0 | 203.0 | 3.65e-52 |
| QTT72092.1 | amino\_acid\_adenylation\_domain-containing\_protein | BGC0002350 | NRP+Polyketide+Saccharide | 29.0 | 46.0 | 203.0 | 4.79e-52 |
| CAD29797.1 | peptide\_synthetase | BGC0001015 | NRP+Polyketide | 28.0 | 39.5 | 203.0 | 4.96e-52 |
| AQH32481.1 | hybrid\_polyketide\_synthase/peptide\_synthetase | BGC0001667 | NRP+Polyketide | 26.0 | 56.5 | 203.0 | 5.42e-52 |
| CAD32904.2 | non-ribosomal\_peptide\_synthetase\_A | BGC0000354 | NRP | 27.0 | 46.0 | 201.0 | 5.98e-52 |
| WP\_011146892.1 | non-ribosomal\_peptide\_synthetase | BGC0001641 | NRP | 32.0 | 41.4 | 202.0 | 9.06e-52 |
| ABO15860.1 | polyketide\_synthase | BGC0000130 | Polyketide | 31.0 | 35.6 | 202.0 | 9.81e-52 |
| WP\_078586793.1 | non-ribosomal\_peptide\_synthetase | BGC0001760 | NRP | 31.0 | 40.2 | 201.0 | 1e-51 |
| CCC55921.1 | non-ribosomal\_peptide\_synthetase/polyketide\_synthase\_hybrid\_protein | BGC0000973 | NRP+Polyketide:Modular type I polyketide | 29.0 | 46.9 | 202.0 | 1.07e-51 |
| AAF00960.1 | mcyA | BGC0001017 | NRP+Polyketide:Modular type I polyketide | 30.0 | 39.4 | 202.0 | 1.12e-51 |
| ANZ15840.1 | non-ribosomal\_peptide\_synthase/amino\_acid\_adenylation\_enzyme | BGC0001569 | NRP | 30.0 | 46.5 | 202.0 | 1.33e-51 |
| QEO75075.1 | condensation\_domain-containing\_protein | BGC0002079 | NRP:Cyclic depsipeptide | 32.0 | 38.2 | 202.0 | 1.38e-51 |
| ABL74945.1 | NRPS | BGC0001048 | NRP:Glycopeptide+Polyketide:Modular type I polyketide+Saccharide:Hybrid/tailoring saccharide | 29.0 | 46.8 | 201.0 | 1.42e-51 |
| WP\_053065270.1 | non-ribosomal\_peptide\_synthetase | BGC0001330 | NRP:Cyclic depsipeptide+Polyketide:Modular type I polyketide | 28.0 | 56.4 | 201.0 | 1.48e-51 |
| AHB38515.1 | non-ribosomal\_peptide\_synthetase | BGC0000345 | NRP+Polyketide:Modular type I polyketide | 31.0 | 41.9 | 201.0 | 1.64e-51 |
| AGZ15460.1 | putative\_non-ribosomal\_peptide\_synthetase | BGC0001036 | NRP+Polyketide | 27.0 | 57.4 | 201.0 | 1.71e-51 |
| ALV86868.1 | Tlo22 | BGC0001406 | NRP | 29.0 | 40.7 | 201.0 | 1.77e-51 |
| AGZ03650.1 | sevA | BGC0000426 | NRP | 27.0 | 45.4 | 199.0 | 1.96e-51 |
| ABL86391.1 | hybrid\_polyketide\_synthase\_and\_nonribosomal\_peptide\_synthetase | BGC0000999 | NRP+Polyketide | 25.0 | 69.2 | 201.0 | 2.02e-51 |
| EFE73308.1 | non-ribosomal\_peptide\_synthetase | BGC0000431 | NRP:Cyclic depsipeptide | 29.0 | 45.2 | 199.0 | 2.06e-51 |
| AZZ09613.1 | PvhA | BGC0002304 | Polyketide+NRP | 25.0 | 62.6 | 201.0 | 2.2e-51 |
| KFL51886.1 | amino\_acid\_adenylation\_protein | BGC0001711 | NRP+Polyketide | 27.0 | 45.3 | 201.0 | 2.61e-51 |
| QBA57740.1 | NRPS | BGC0002377 | NRP | 31.0 | 45.9 | 199.0 | 2.63e-51 |
| ALV82388.1 | CDA\_peptide\_synthetase\_III | BGC0001370 | NRP | 29.0 | 53.4 | 200.0 | 3.06e-51 |
| AAR87760.2 | ZmaK | BGC0001059 | NRP+Polyketide | 28.0 | 45.4 | 200.0 | 3.17e-51 |
| AAC82549.1 | FxbB | BGC0000351 | NRP | 30.0 | 47.1 | 200.0 | 3.17e-51 |
| QEO74982.1 | omn7 | BGC0002078 | NRP:Cyclic depsipeptide | 31.0 | 35.3 | 201.0 | 3.21e-51 |
| AYA22334.1 | KerC | BGC0001955 | NRP | 28.0 | 51.4 | 200.0 | 3.8e-51 |
| ctg1\_orf1265 |  | BGC0001752 | NRP | 29.0 | 46.2 | 200.0 | 4.63e-51 |
| CAF05649.1 | TubD\_protein | BGC0001053 | NRP+Polyketide | 28.0 | 46.1 | 200.0 | 4.76e-51 |
| AHI59109.1 | locillomycin\_synthase\_B | BGC0001005 | NRP+Polyketide | 29.0 | 36.5 | 200.0 | 4.91e-51 |
| AEG64689.1 | LpmA | BGC0000379 | NRP | 29.0 | 44.4 | 197.0 | 5.3e-51 |
| CAJ88610.1 | putative\_non-ribosomal\_peptide\_synthetase | BGC0000327 | NRP | 29.0 | 48.1 | 197.0 | 5.69e-51 |
| AIE77057.1 | peptide\_synthetase | BGC0000418 | NRP | 30.0 | 45.2 | 199.0 | 6.23e-51 |
| AGJ76605.1 | HglB | BGC0000869 | Other | 33.0 | 27.0 | 189.0 | 6.25e-51 |
| WP\_099111429.1 | non-ribosomal\_peptide\_synthetase | BGC0001826 | NRP | 27.0 | 48.1 | 198.0 | 6.77e-51 |
| CDG12864.1 | non-ribosomal\_peptide\_synthetase | BGC0001415 | NRP+Polyketide | 27.0 | 50.2 | 199.0 | 6.98e-51 |
| DMH02\_027320 | amino\_acid\_adenylation\_domain-containing\_protein | BGC0002638 | NRP | 29.0 | 44.4 | 196.0 | 7.48e-51 |
| ARU08073.1 | mlcK | BGC0001448 | NRP:Lipopeptide:Ca+-dependent lipopeptide | 27.0 | 64.1 | 199.0 | 7.79e-51 |
| AGN74886.1 | nonribosomal\_peptide\_synthetase | BGC0000459 | NRP:Cyclic depsipeptide+Polyketide:Trans-AT type I polyketide | 29.0 | 51.5 | 198.0 | 8.17e-51 |
| ABO15888.1 | polyketide\_synthase | BGC0000132 | Polyketide | 35.0 | 26.4 | 199.0 | 8.2e-51 |
| CAC48369.1 | peptide\_synthetase | BGC0000311 | NRP | 31.0 | 36.3 | 191.0 | 8.29e-51 |
| WP\_141576257.1 | non-ribosomal\_peptide\_synthetase | BGC0002686 | NRP | 33.0 | 36.4 | 199.0 | 8.54e-51 |
| QCQ67879.1 | non-ribosomal\_peptide\_synthetase | BGC0002297 | NRP+Polyketide | 28.0 | 39.8 | 199.0 | 9.79e-51 |
| AJK49765.1 | non-ribosomal\_peptide\_synthase | BGC0002565 | NRP | 28.0 | 65.3 | 198.0 | 1.01e-50 |
| CAQ18831.1 | nonribosomal\_polypetide\_synthetase | BGC0000954 | NRP+Polyketide:Modular type I polyketide | 31.0 | 37.0 | 197.0 | 1.2e-50 |
| AAN85512.1 | nonribosomal\_peptide\_synthetase | BGC0001101 | NRP+Polyketide:Modular type I polyketide+Polyketide:Trans-AT type I polyketide | 27.0 | 52.5 | 198.0 | 1.22e-50 |
| RLV64601.1 | polyketide\_synthase\_of\_type\_I | BGC0001845 | Polyketide+NRP+Other:Aminocoumarin | 26.0 | 64.3 | 198.0 | 1.42e-50 |
| AAV66110.2 | fusaridione\_A\_synthetase | BGC0000992 | NRP+Polyketide | 24.0 | 84.1 | 198.0 | 1.47e-50 |
| QEO74983.1 | omn8 | BGC0002078 | NRP:Cyclic depsipeptide | 31.0 | 36.5 | 198.0 | 1.52e-50 |
| CAD55498.1 | CDA\_peptide\_synthetase\_III\_(CdaPs3) | BGC0000315 | NRP:Lipopeptide:Ca+-dependent lipopeptide | 29.0 | 52.6 | 198.0 | 1.57e-50 |
| QYA95662.1 | amino\_acid\_adenylation\_domain-containing\_protein | BGC0002676 | NRP | 27.0 | 56.4 | 196.0 | 1.73e-50 |
| AWO77084.1 | hybrid\_non-ribosomal\_peptide\_synthetase/type\_I\_polyketide\_synthase | BGC0001556 | NRP+Polyketide | 25.0 | 70.9 | 197.0 | 2.38e-50 |
| APZ78680.1 | nonribosomal\_peptide\_synthetase | BGC0001417 | NRP:Cyclic depsipeptide+Polyketide:Iterative type I polyketide | 31.0 | 36.8 | 197.0 | 2.5e-50 |
| EAL85113.2 | hybrid\_PKS-NRPS\_enzyme | BGC0001037 | NRP+Polyketide:Iterative type I polyketide | 24.0 | 88.3 | 197.0 | 2.53e-50 |
| AXG49819.1 | hybrid\_non-ribosomal\_peptide\_synthetase/type\_I\_polyketide\_synthase | BGC0000383 | NRP+Polyketide:Modular type I polyketide | 25.0 | 69.2 | 197.0 | 2.55e-50 |
| CAC11137.1 | NikP1\_protein | BGC0000876 | Other | 29.0 | 48.7 | 191.0 | 2.84e-50 |
| AAZ23076.1 | peptide\_synthetase | BGC0000291 | NRP | 29.0 | 45.8 | 197.0 | 3.28e-50 |
| AAG06715.1 | probable\_non-ribosomal\_peptide\_synthetase | BGC0002037 | NRP | 28.0 | 48.4 | 197.0 | 3.48e-50 |
| QPI18729.1 | nonribosomal\_peptide\_synthetase | BGC0002125 | NRP:Cyclic depsipeptide | 31.0 | 35.9 | 197.0 | 4.01e-50 |
| AYJ71721.1 | non-ribosomal\_peptide\_synthetase | BGC0001942 | NRP+Polyketide | 25.0 | 64.3 | 196.0 | 4.59e-50 |
| AFD30954.1 | CrmA | BGC0000966 | NRP+Polyketide | 29.0 | 45.7 | 196.0 | 4.72e-50 |
| AEP18655.1 | WAPS2 | BGC0000461 | NRP | 26.0 | 64.9 | 197.0 | 4.91e-50 |
| SCO70310.1 | Type\_I\_polyketide\_synthase | BGC0001433 | Polyketide:Modular type I polyketide | 34.0 | 36.5 | 196.0 | 5.76e-50 |
| BAH43764.1 | tyrocidine\_synthetase\_I | BGC0000452 | NRP | 29.0 | 40.6 | 194.0 | 5.77e-50 |
| QPB41098.1 | non-ribosomal\_peptide\_synthetase | BGC0002503 | NRP+Polyketide | 32.0 | 36.2 | 195.0 | 6.03e-50 |
| CBW54672.1 | non\_ribosomal\_peptide\_synthetase | BGC0000971 | NRP+Polyketide:Modular type I polyketide | 32.0 | 35.7 | 189.0 | 6.21e-50 |
| WP\_051462298.1 | non-ribosomal\_peptide\_synthetase | BGC0001873 | NRP:Lipopeptide | 29.0 | 45.4 | 196.0 | 8.12e-50 |
| EET76303.1 | AMP-binding\_enzyme | BGC0002685 | NRP | 28.0 | 48.6 | 195.0 | 8.54e-50 |
| AAC82550.1 | FxbC | BGC0000351 | NRP | 29.0 | 53.6 | 196.0 | 1.03e-49 |
| DAC80541.1 | NRPS/PKS | BGC0001840 | NRP+Polyketide | 32.0 | 35.3 | 194.0 | 1.04e-49 |
| APZ78729.1 | nonribosomal\_peptide\_synthetase | BGC0001421 | NRP:Cyclic depsipeptide+Polyketide:Iterative type I polyketide | 31.0 | 35.8 | 196.0 | 1.06e-49 |
| AEC14346.1 | nonribosomal\_peptide\_synthetase | BGC0000377 | NRP | 24.0 | 63.3 | 195.0 | 1.09e-49 |
| BAE06845.2 | peramine\_synthetase | BGC0002164 | NRP | 31.0 | 38.4 | 195.0 | 1.13e-49 |
| BAG17643.1 | putative\_NRPS-type-I\_PKS\_fusion\_protein | BGC0001043 | NRP+Polyketide | 28.0 | 46.3 | 195.0 | 1.19e-49 |
| QTT72101.1 | non-ribosomal\_peptide\_synthetase | BGC0002350 | NRP+Polyketide+Saccharide | 30.0 | 37.7 | 195.0 | 1.28e-49 |
| AAG02355.1 | peptide\_synthetase\_NRPS9-8 | BGC0000963 | NRP:Glycopeptide+Polyketide:Modular type I polyketide+Saccharide:Hybrid/tailoring saccharide | 30.0 | 37.7 | 195.0 | 1.29e-49 |
| CBG75492.1 | putative\_NRPS/siderophore\_biosynthesis\_protein | BGC0000423 | NRP | 29.0 | 51.3 | 195.0 | 1.42e-49 |
| QWM97320.1 | non-ribosomal\_peptide\_synthetase | BGC0002384 | NRP | 31.0 | 35.6 | 195.0 | 1.42e-49 |
| CBL93720.1 | NRPS\_didomain\_PCP-A | BGC0000360 | NRP | 29.0 | 46.5 | 191.0 | 1.47e-49 |
| DAC80524.1 | peptide\_synthetase | BGC0001841 | NRP+Polyketide | 31.0 | 39.4 | 194.0 | 1.51e-49 |
| ABD65960.1 | nonribosomal\_peptide\_synthetase | BGC0000341 | NRP | 29.0 | 48.0 | 191.0 | 1.56e-49 |
| AZM51140.1 | non-ribosomal\_peptide\_synthetase | BGC0002702 | NRP | 31.0 | 41.0 | 194.0 | 1.58e-49 |
| DAB41477.1 | nonribosomal\_peptide\_synthetase | BGC0001230 | NRP:Cyclic depsipeptide+Polyketide:Modular type I polyketide | 32.0 | 35.7 | 195.0 | 1.69e-49 |
| ARR97038.1 | SphE | BGC0001780 | NRP | 30.0 | 46.0 | 194.0 | 1.91e-49 |
| DAC76734.1 | type\_I\_polyketide\_synthase/non-ribosomal\_peptide\_synthetase | BGC0001885 | NRP+Polyketide | 26.0 | 68.5 | 195.0 | 1.93e-49 |
| QWT72291.1 | amino\_acid\_adenylation\_domain-containing\_protein | BGC0002430 | NRP+Saccharide | 28.0 | 46.0 | 192.0 | 2.63e-49 |
| AAF19811.1 | mtaC | BGC0001024 | NRP+Polyketide:Modular type I polyketide | 28.0 | 44.7 | 193.0 | 2.75e-49 |
| ARR97036.1 | SphC | BGC0001780 | NRP | 30.0 | 46.1 | 194.0 | 3.12e-49 |
| AAO62586.1 | peptide\_sythetase | BGC0001016 | NRP+Polyketide | 27.0 | 39.6 | 194.0 | 3.35e-49 |
| QMW33900.1 | hypothetical\_protein | BGC0002167 | NRP | 29.0 | 48.8 | 192.0 | 3.37e-49 |
| AQZ69228.1 | hypothetical\_protein | BGC0001635 | NRP+Polyketide | 29.0 | 45.6 | 194.0 | 3.69e-49 |
| ACS20358.1 | amino\_acid\_adenylation\_domain\_protein | BGC0002420 | NRP+Polyketide | 31.0 | 35.8 | 192.0 | 3.98e-49 |
| CBZ42146.1 | putative\_non-ribosomal\_peptide\_synthetase | BGC0001117 | NRP | 31.0 | 34.9 | 194.0 | 4.03e-49 |
| AQZ26587.1 | obafluorin\_dimodular\_nonribosomal\_peptide\_synthetase | BGC0001437 | NRP | 28.0 | 46.9 | 192.0 | 6e-49 |
| AAT09805.1 | NocB | BGC0000395 | NRP | 29.0 | 45.7 | 192.0 | 7.92e-49 |
| AKL78825.1 | GLNRPS7 | BGC0001187 | NRP:Lipopeptide+Polyketide:Iterative type I polyketide | 26.0 | 58.1 | 189.0 | 7.97e-49 |
| WP\_010369425.1 | non-ribosomal\_peptide\_synthetase | BGC0000314 | Polyketide+NRP:Cyclic depsipeptide+Other:Aminocoumarin | 26.0 | 46.6 | 192.0 | 8.14e-49 |
| AJV88377.1 | MfnE | BGC0001214 | NRP | 28.0 | 58.4 | 192.0 | 8.35e-49 |
| CAM02313.1 | putative\_non-ribosomal\_peptide\_synthetase | BGC0000349 | NRP | 28.0 | 50.7 | 192.0 | 9.21e-49 |
| APZ78692.1 | nonribosomal\_peptide\_synthetase | BGC0001418 | NRP:Cyclic depsipeptide+Polyketide:Iterative type I polyketide | 32.0 | 35.1 | 192.0 | 1.16e-48 |
| APZ78704.1 | nonribosomal\_peptide\_synthetase | BGC0001419 | NRP:Cyclic depsipeptide+Polyketide:Iterative type I polyketide | 32.0 | 35.1 | 192.0 | 1.16e-48 |
| APZ78716.1 | nonribosomal\_peptide\_synthetase | BGC0001420 | NRP:Cyclic depsipeptide+Polyketide:Iterative type I polyketide | 32.0 | 35.1 | 192.0 | 1.16e-48 |
| AOZ21316.1 | SulI | BGC0001790 | NRP | 28.0 | 45.7 | 190.0 | 1.21e-48 |
| ALG65314.1 | Cal22 | BGC0001297 | NRP | 30.0 | 47.1 | 189.0 | 1.36e-48 |
| AEW31015.1 | plipastatin\_synthetase | BGC0000407 | NRP | 29.0 | 36.5 | 191.0 | 1.4e-48 |
| AFY58521.1 | amino\_acid\_adenylation\_enzyme/thioester\_reductase\_family\_protein | BGC0002411 | NRP+Polyketide | 28.0 | 35.9 | 191.0 | 1.43e-48 |
| QBK15049.1 | PKS-NRPS\_hybrid\_TraA | BGC0002197 | Polyketide+NRP | 25.0 | 79.0 | 192.0 | 1.46e-48 |
| XP\_001217690.1 | hypothetical\_protein | BGC0001517 | NRP | 26.0 | 62.1 | 191.0 | 1.55e-48 |
| WP\_030498980.1 | hypothetical\_protein | BGC0001327 | NRP:Cyclic depsipeptide+Polyketide:Modular type I polyketide | 31.0 | 39.1 | 189.0 | 1.61e-48 |
| CUX96954.1 | TmcG | BGC0001829 | NRP+Polyketide | 31.0 | 42.0 | 191.0 | 2.56e-48 |
| QXJ21808.1 | amino\_acid\_adenylation\_domain-containing\_protein | BGC0002370 | NRP | 26.0 | 69.0 | 191.0 | 3.22e-48 |
| UMM61371.1 | Tsk10 | BGC0002661 | NRP | 31.0 | 36.8 | 191.0 | 3.23e-48 |
| WP\_245566645.1 | amino\_acid\_adenylation\_domain-containing\_protein | BGC0002467 | NRP | 26.0 | 64.2 | 190.0 | 4.68e-48 |
| AKQ52532.1 | nonribosomal\_peptide\_synthetase | BGC0002533 | NRP+Polyketide | 30.0 | 37.0 | 190.0 | 5.72e-48 |
| AHZ34241.1 | CipD | BGC0001389 | NRP | 31.0 | 35.1 | 190.0 | 5.84e-48 |
| ABL74937.1 | NRPS | BGC0001048 | NRP:Glycopeptide+Polyketide:Modular type I polyketide+Saccharide:Hybrid/tailoring saccharide | 26.0 | 63.7 | 188.0 | 6.36e-48 |
| AHB82071.1 | non\_ribosomal\_peptide\_synthetase | BGC0001231 | NRP+Polyketide:Modular type I polyketide | 26.0 | 58.6 | 189.0 | 7.56e-48 |
| EEP98516.1 | Peptide\_synthetase | BGC0002091 | NRP | 31.0 | 34.1 | 187.0 | 9.25e-48 |
| CBD77749.1 | non-ribosomal\_peptide\_synthetase | BGC0000974 | NRP+Polyketide | 30.0 | 41.4 | 188.0 | 9.65e-48 |
| AAN85522.1 | hybrid\_nonribosomal\_peptide\_synthetase\_/\_polyketide\_synthase | BGC0001101 | NRP+Polyketide:Modular type I polyketide+Polyketide:Trans-AT type I polyketide | 26.0 | 50.5 | 189.0 | 1.01e-47 |
| AKQ22680.1 | malonyl\_CoA-acyl\_carrier\_protein\_transacylase | BGC0001656 | Polyketide | 27.0 | 47.5 | 189.0 | 1.03e-47 |
| AQH32484.1 | peptide\_synthetase | BGC0001667 | NRP+Polyketide | 27.0 | 39.6 | 189.0 | 1.14e-47 |
| AHD05627.1 | putative\_non-ribosomal\_peptide\_ligase\_domain\_protein | BGC0001033 | NRP+Polyketide | 26.0 | 49.2 | 188.0 | 1.23e-47 |
| AXG46163.1 | non-ribosomal\_peptide\_synthetase | BGC0002713 | NRP | 30.0 | 35.5 | 187.0 | 1.36e-47 |
| AQX14499.1 | monobactam\_NRPS\_scaffold\_1 | BGC0001672 | NRP | 28.0 | 34.7 | 188.0 | 1.36e-47 |
| AAS47562.1 | mixed\_type\_I\_polyketide\_synthase\_-\_peptide\_synthetase | BGC0001108 | NRP+Polyketide:Trans-AT type I polyketide | 25.0 | 68.7 | 189.0 | 1.42e-47 |
| ctg1\_orf8 |  | BGC0001109 | NRP+Polyketide | 25.0 | 68.7 | 189.0 | 1.42e-47 |
| ARF06222.1 | non-ribosomal\_peptide\_synthetase | BGC0001593 | NRP | 30.0 | 39.4 | 187.0 | 1.44e-47 |
| ctg4\_5 |  | BGC0002017 | NRP | 30.0 | 39.3 | 188.0 | 1.51e-47 |
| WP\_082191961.1 | non-ribosomal\_peptide\_synthetase | BGC0001451 | NRP | 28.0 | 42.3 | 188.0 | 1.54e-47 |
| AJI44176.1 | nonribosomal\_peptide\_synthetase | BGC0001193 | NRP | 27.0 | 48.4 | 186.0 | 1.67e-47 |
| AHB38497.1 | non-ribosomal\_peptide\_synthetase | BGC0000346 | NRP+Polyketide:Modular type I polyketide | 27.0 | 46.5 | 188.0 | 1.79e-47 |
| AGU50949.1 | putative\_non-ribosomal\_peptide\_synthetase | BGC0002417 | NRP+Polyketide | 31.0 | 36.0 | 187.0 | 1.84e-47 |
| EFY95969.1 | polyketide\_synthase | BGC0002270 | NRP+Polyketide | 26.0 | 47.5 | 188.0 | 1.86e-47 |
| AJW76709.1 | DsaG | BGC0001196 | NRP | 31.0 | 39.3 | 188.0 | 1.91e-47 |
| MBE3202942.1 | non-ribosomal\_peptide\_synthetase | BGC0002410 | NRP | 30.0 | 46.0 | 187.0 | 1.94e-47 |
| AXB34356.1 | non-ribosomal\_peptide\_synthetase | BGC0002415 | NRP | 27.0 | 45.6 | 188.0 | 1.97e-47 |
| ABW17375.1 | PsoA | BGC0000411 | NRP | 31.0 | 36.1 | 187.0 | 2.22e-47 |
| EFL06864.1 | non-ribosomal\_peptide\_synthetase | BGC0000300 | NRP | 31.0 | 33.7 | 186.0 | 2.75e-47 |
| AAM80539.1 | StaA | BGC0000290 | NRP:Glycopeptide | 30.0 | 43.5 | 187.0 | 2.87e-47 |
| AZM50111.1 | non-ribosomal\_peptide\_synthetase | BGC0002702 | NRP | 30.0 | 40.6 | 187.0 | 3.01e-47 |
| EFL06867.1 | predicted\_protein | BGC0000300 | NRP | 32.0 | 35.6 | 187.0 | 3.09e-47 |
| WP\_004571779.1 | non-ribosomal\_peptide\_synthetase | BGC0001760 | NRP | 29.0 | 37.9 | 185.0 | 3.33e-47 |
| AAG02349.1 | peptide\_synthetase\_NRPS11-10 | BGC0000963 | NRP:Glycopeptide+Polyketide:Modular type I polyketide+Saccharide:Hybrid/tailoring saccharide | 28.0 | 52.1 | 187.0 | 3.4e-47 |
| EWM63010.1 | non-ribosomal\_peptide\_synthetase | BGC0001328 | NRP:Cyclic depsipeptide+Polyketide:Modular type I polyketide | 31.0 | 39.1 | 185.0 | 3.52e-47 |
| AID65224.1 | nonribosomal\_peptide\_synthetase | BGC0000335 | NRP+Polyketide | 30.0 | 35.8 | 187.0 | 4.25e-47 |
| AJD47485.1 | PpsD | BGC0002418 | NRP+Polyketide | 26.0 | 55.6 | 187.0 | 4.28e-47 |
| MAA\_10043 | non-ribosomal\_peptide\_synthetase | BGC0000337 | NRP | 28.0 | 44.4 | 187.0 | 4.32e-47 |
| QTT72106.1 | amino\_acid\_adenylation\_domain-containing\_protein | BGC0002350 | NRP+Polyketide+Saccharide | 28.0 | 52.1 | 187.0 | 4.45e-47 |
| QGU18619.1 | polyketide\_synthase/non-ribosomal\_peptide\_synthetase | BGC0002365 | Other+Polyketide | 25.0 | 69.2 | 187.0 | 4.64e-47 |
| ABS90470.1 | NRPS/PKS | BGC0001106 | NRP+Polyketide | 26.0 | 69.2 | 187.0 | 5.65e-47 |
| AGZ15476.1 | putative\_non\_ribosomal\_peptide\_synthetase | BGC0001036 | NRP+Polyketide | 27.0 | 55.6 | 185.0 | 5.7e-47 |
| AIZ66879.1 | nonribosomal\_peptide\_synthetase | BGC0002666 | NRP+Alkaloid | 28.0 | 45.9 | 186.0 | 6.06e-47 |
| QPI18723.1 | nonribosomal\_peptide\_synthetase | BGC0002125 | NRP:Cyclic depsipeptide | 30.0 | 35.8 | 185.0 | 6.46e-47 |
| AZM51141.1 | non-ribosomal\_peptide\_synthetase | BGC0002702 | NRP | 30.0 | 38.3 | 186.0 | 6.7e-47 |
| ACJ04424.1 | aureobasidin\_A1\_biosynthesis\_complex | BGC0000307 | NRP | 29.0 | 46.1 | 186.0 | 7.7e-47 |
| QPI18727.1 | nonribosomal\_peptide\_synthetase | BGC0002125 | NRP:Cyclic depsipeptide | 29.0 | 41.4 | 186.0 | 7.86e-47 |
| CAE52334.1 | non-ribosomal\_peptide\_synthase | BGC0001088 | NRP+Polyketide | 26.0 | 46.3 | 186.0 | 8.19e-47 |
| PHM26612.1 | pvdj | BGC0001130 | NRP+Polyketide | 28.0 | 51.9 | 186.0 | 8.79e-47 |
| BAT51067.1 | type\_I\_polyketide\_synthase | BGC0001296 | Polyketide | 32.0 | 35.2 | 186.0 | 8.92e-47 |
| AGE11891.1 | nonribosomal\_peptide\_synthetase | BGC0000366 | NRP | 32.0 | 35.5 | 184.0 | 1.06e-46 |
| AAU39360.1 | lichenysin\_synthase\_LchAB | BGC0000381 | NRP | 28.0 | 35.3 | 186.0 | 1.09e-46 |
| ABV99085.1 | thioester\_reductase\_domain | BGC0001007 | Polyketide+NRP | 31.0 | 37.3 | 185.0 | 1.21e-46 |
| WP\_069848010.1 | non-ribosomal\_peptide\_synthetase | BGC0002472 | NRP | 29.0 | 36.3 | 185.0 | 1.35e-46 |
| APZ78715.1 | nonribosomal\_peptide\_synthetase | BGC0001420 | NRP:Cyclic depsipeptide+Polyketide:Iterative type I polyketide | 33.0 | 34.7 | 185.0 | 1.36e-46 |
| CAC22144.1 | CpkC;\_Polyketide\_synthase\_module\_5 | BGC0000038 | Polyketide:Modular type I polyketide | 32.0 | 34.9 | 185.0 | 1.5e-46 |
| ANG60380.1 | nonribosomal\_peptide\_synthetase\_BudB | BGC0001434 | NRP | 25.0 | 53.0 | 184.0 | 1.51e-46 |
| WP\_010369428.1 | non-ribosomal\_peptide\_synthetase | BGC0000314 | Polyketide+NRP:Cyclic depsipeptide+Other:Aminocoumarin | 24.0 | 54.6 | 185.0 | 1.67e-46 |
| EKJ70673.1 | hypothetical\_protein | BGC0002188 | NRP+Polyketide | 28.0 | 46.8 | 185.0 | 1.69e-46 |
| APZ78728.1 | nonribosomal\_peptide\_synthetase | BGC0001421 | NRP:Cyclic depsipeptide+Polyketide:Iterative type I polyketide | 33.0 | 34.7 | 185.0 | 1.78e-46 |
| CAJ18237.2 | non-ribosomal\_peptide\_synthetase\_B | BGC0000354 | NRP | 31.0 | 36.5 | 185.0 | 1.8e-46 |
| ABP57749.1 | DepE | BGC0000993 | NRP:Cyclic depsipeptide+Polyketide:Modular type I polyketide | 29.0 | 48.3 | 184.0 | 1.8e-46 |
| ALJ49920.1 | TtmI | BGC0001236 | Polyketide | 27.0 | 47.4 | 177.0 | 1.9e-46 |
| ABD65956.1 | nonribosomal\_peptide\_synthetase | BGC0000341 | NRP | 32.0 | 35.4 | 184.0 | 1.94e-46 |
| AAM80537.1 | StaC | BGC0000290 | NRP:Glycopeptide | 29.0 | 40.6 | 185.0 | 1.95e-46 |
| ABL74939.1 | NRPS | BGC0001048 | NRP:Glycopeptide+Polyketide:Modular type I polyketide+Saccharide:Hybrid/tailoring saccharide | 29.0 | 46.1 | 183.0 | 2.01e-46 |
| BAX89999.1 | Non-ribosomal\_peptide\_synthetase | BGC0001628 | NRP | 32.0 | 35.0 | 184.0 | 2.16e-46 |
| CBJ79915.1 | putative\_Phenylalanine\_racemase\_(ATP-hydrolyzing) | BGC0001133 | NRP | 29.0 | 36.2 | 184.0 | 2.53e-46 |
| AEF16059.1 | non-ribosomal\_peptide\_synthetase | BGC0000953 | Saccharide:Aminoglycoside | 26.0 | 45.6 | 181.0 | 2.54e-46 |
| MBD2892727.1 | Phenyloxazoline\_synthase\_MbtB | BGC0002718 | NRP | 30.0 | 36.1 | 183.0 | 2.55e-46 |
| KZM69124.1 | non-ribosomal\_peptide\_synthetase | BGC0002352 | Other | 29.0 | 48.4 | 184.0 | 2.6e-46 |
| ATY69584.1 | hybrid\_nonribosomal\_peptide\_synthetase/type\_I\_polyketide\_synthase | BGC0001823 | NRP+Polyketide | 29.0 | 45.4 | 184.0 | 2.65e-46 |
| AAK81826.1 | peptide\_synthetase | BGC0000326 | NRP | 27.0 | 52.4 | 184.0 | 2.66e-46 |
| ATX68112.1 | malonyl\_CoA-acyl\_carrier\_protein\_transacylase | BGC0001772 | Polyketide | 27.0 | 46.1 | 184.0 | 2.67e-46 |
| QWT72293.1 | non-ribosomal\_peptide\_synthetase | BGC0002430 | NRP+Saccharide | 31.0 | 35.3 | 184.0 | 2.76e-46 |
| AYJ71712.1 | non-ribosomal\_peptide\_synthetase | BGC0001942 | NRP+Polyketide | 27.0 | 48.5 | 177.0 | 2.95e-46 |
| AWS21279.1 | type\_I\_polyketide\_synthase | BGC0001934 | Polyketide | 33.0 | 26.5 | 183.0 | 3.12e-46 |
| AZY91989.1 | polyketide\_synthase | BGC0002022 | Polyketide | 33.0 | 26.5 | 183.0 | 3.12e-46 |
| ABP73646.1 | SalB | BGC0000145 | NRP+Polyketide | 30.0 | 35.6 | 178.0 | 3.27e-46 |
| ABP53497.1 | NRPS\_(ACP-P) | BGC0001041 | NRP+Polyketide | 30.0 | 35.6 | 178.0 | 3.27e-46 |
| EAT91803.2 | hypothetical\_protein | BGC0002205 | Polyketide+NRP | 23.0 | 97.4 | 184.0 | 3.34e-46 |
| ABS74208.1 | fengycin\_synthetase\_B | BGC0001095 | NRP | 28.0 | 35.7 | 184.0 | 3.74e-46 |
| KMO93435.1 | NRPS/PKS | BGC0002095 | NRP | 32.0 | 33.1 | 182.0 | 5.06e-46 |
| APZ78808.1 | nonribosomal\_peptide\_synthetase | BGC0001428 | NRP:Cyclic depsipeptide+Polyketide:Iterative type I polyketide | 32.0 | 36.2 | 183.0 | 5.27e-46 |
| BAI63289.1 | putative\_non-ribosomal\_peptide\_synthetase | BGC0000434 | NRP | 27.0 | 58.1 | 183.0 | 5.33e-46 |
| BBA84067.1 | type\_I\_polyketide\_synthase | BGC0001649 | Polyketide | 31.0 | 36.1 | 183.0 | 5.35e-46 |
| AJK49766.1 | non-ribosomal\_peptide\_synthase | BGC0002565 | NRP | 30.0 | 45.6 | 183.0 | 5.62e-46 |
| FAA01291.1 | polyketide\_synthase-nonribosomal\_peptide\_synthetase\_PyvA | BGC0002210 | Polyketide+NRP | 23.0 | 89.4 | 183.0 | 5.71e-46 |
| CAC17499.1 | putative\_non-ribosomal\_peptide\_synthase | BGC0000324 | NRP | 31.0 | 38.2 | 183.0 | 5.97e-46 |
| AGN74895.1 | nonribosomal\_peptide\_synthetase/polyketide\_synthase\_hybrid\_protein | BGC0000459 | NRP:Cyclic depsipeptide+Polyketide:Trans-AT type I polyketide | 26.0 | 46.0 | 183.0 | 6.48e-46 |
| APZ78691.1 | nonribosomal\_peptide\_synthetase | BGC0001418 | NRP:Cyclic depsipeptide+Polyketide:Iterative type I polyketide | 33.0 | 34.7 | 183.0 | 6.91e-46 |
| CCC55922.1 | putative\_non-ribosomal\_peptide\_synthetase | BGC0000973 | NRP+Polyketide:Modular type I polyketide | 29.0 | 44.9 | 181.0 | 7.08e-46 |
| AQZ69227.1 | hypothetical\_protein | BGC0001635 | NRP+Polyketide | 29.0 | 46.0 | 182.0 | 7.18e-46 |
| AGS77307.1 | NRPS\_modules\_1-2 | BGC0001178 | NRP:Glycopeptide | 32.0 | 36.7 | 182.0 | 7.52e-46 |
| CCJ67645.1 | JagA | BGC0001127 | NRP | 29.0 | 47.2 | 182.0 | 8.05e-46 |
| AED90004.1 | non-ribosomal\_peptide\_synthetase\_ThaC1 | BGC0000443 | NRP:Beta-lactam | 26.0 | 48.8 | 176.0 | 8.18e-46 |
| QIE07362.1 | polyketide\_synthase\_NecC | BGC0002050 | NRP+Polyketide:Trans-AT type I polyketide | 27.0 | 47.8 | 182.0 | 9.09e-46 |
| CAJ34375.1 | NRPS | BGC0000445 | NRP:Cyclic depsipeptide | 26.0 | 58.4 | 182.0 | 9.16e-46 |
| AUD08663.1 | iPKS-NRPS | BGC0001553 | NRP+Polyketide | 24.0 | 68.3 | 182.0 | 9.3e-46 |
| QBA57741.1 | NRPS | BGC0002377 | NRP | 32.0 | 37.0 | 176.0 | 9.63e-46 |
| ABS74205.1 | fengycin\_synthetase\_E | BGC0001095 | NRP | 27.0 | 36.4 | 181.0 | 9.95e-46 |
| ALP32042.1 | CycB | BGC0001293 | Polyketide | 30.0 | 36.1 | 182.0 | 1e-45 |
| BBG06551.1 | AMP\_binding\_enzyme | BGC0001925 | Alkaloid | 28.0 | 40.6 | 174.0 | 1.72e-45 |
| ARU08063.1 | mlcA | BGC0001448 | NRP:Lipopeptide:Ca+-dependent lipopeptide | 26.0 | 46.9 | 180.0 | 1.87e-45 |
| APZ78794.1 | nonribosomal\_peptide\_synthetase | BGC0001427 | NRP:Cyclic depsipeptide+Polyketide:Iterative type I polyketide | 32.0 | 35.2 | 181.0 | 2.04e-45 |
| MBN3579113.1 | amino\_acid\_adenylation\_domain-containing\_protein | BGC0002613 | NRP+Polyketide | 29.0 | 38.8 | 181.0 | 2.22e-45 |
| AJD47483.1 | amino\_acid\_adenylation\_domain-containing\_protein | BGC0002418 | NRP+Polyketide | 27.0 | 51.7 | 179.0 | 2.43e-45 |
| ADD82940.1 | Bat2 | BGC0001099 | NRP+Polyketide:Modular type I polyketide+Polyketide:Trans-AT type I polyketide | 24.0 | 67.2 | 181.0 | 2.5e-45 |
| AGS77308.1 | NRPS\_module\_3 | BGC0001178 | NRP:Glycopeptide | 27.0 | 51.4 | 180.0 | 2.56e-45 |
| CCJ67646.1 | JagB | BGC0001127 | NRP | 30.0 | 45.1 | 181.0 | 2.64e-45 |
| APZ78781.1 | nonribosomal\_peptide\_synthetase | BGC0001426 | NRP:Cyclic depsipeptide+Polyketide:Iterative type I polyketide | 33.0 | 35.6 | 181.0 | 2.68e-45 |
| APZ78703.1 | nonribosomal\_peptide\_synthetase | BGC0001419 | NRP:Cyclic depsipeptide+Polyketide:Iterative type I polyketide | 28.0 | 58.4 | 181.0 | 2.68e-45 |
| ATL73045.1 | amino\_acid\_adenylation\_domain\_protein | BGC0001807 | NRP+Polyketide | 28.0 | 45.8 | 179.0 | 2.71e-45 |
| AAT09804.1 | NocA | BGC0000395 | NRP | 26.0 | 64.0 | 181.0 | 2.85e-45 |
| CAC48361.1 | peptide\_synthetase | BGC0000311 | NRP | 29.0 | 37.2 | 181.0 | 2.92e-45 |
| AEC14347.1 | nonribosomal\_peptide\_synthetase | BGC0000377 | NRP | 23.0 | 67.5 | 180.0 | 3.04e-45 |
| KDM89832.1 | peptide\_synthetase | BGC0002412 | NRP | 26.0 | 46.7 | 179.0 | 3.08e-45 |
| ADH04641.1 | TgaC | BGC0001051 | NRP+Polyketide:Modular type I polyketide | 32.0 | 34.4 | 181.0 | 3.21e-45 |
| QMS79067.1 | nonribosomal\_peptide\_synthetase\_12 | BGC0002198 | NRP | 26.0 | 46.0 | 181.0 | 3.24e-45 |
| BAH04161.1 | putative\_non-ribosomal\_peptide\_synthetase | BGC0000450 | NRP | 26.0 | 58.2 | 181.0 | 3.55e-45 |
| ABP55493.1 | thioester\_reductase\_domain | BGC0001006 | NRP+Polyketide | 30.0 | 36.3 | 180.0 | 4.13e-45 |
| CAN89638.1 | putative\_non-ribosomal\_peptide\_synthetase | BGC0001070 | NRP+Polyketide:Modular type I polyketide+Polyketide:Trans-AT type I polyketide | 27.0 | 63.5 | 179.0 | 4.16e-45 |
| ABK36076.1 | nonribosomal\_peptide\_synthetase | BGC0001502 | NRP | 25.0 | 68.2 | 180.0 | 5e-45 |
| QEO75073.1 | condensation\_domain-containing\_protein | BGC0002079 | NRP:Cyclic depsipeptide | 31.0 | 35.4 | 180.0 | 6.16e-45 |
| BAP16699.1 | nonribosomal\_peptide\_synthetase | BGC0000376 | NRP | 29.0 | 46.7 | 179.0 | 8.03e-45 |
| AVI26393.1 | nonribosomal\_peptide\_synthase | BGC0001800 | NRP+Polyketide | 31.0 | 35.9 | 179.0 | 8.61e-45 |
| AHD05615.1 | putative\_non-ribosomal\_peptide\_ligase/\_polyketide\_synthase\_hybrid | BGC0001033 | NRP+Polyketide | 29.0 | 36.3 | 179.0 | 1.03e-44 |
| APZ78679.1 | nonribosomal\_peptide\_synthetase | BGC0001417 | NRP:Cyclic depsipeptide+Polyketide:Iterative type I polyketide | 33.0 | 34.8 | 179.0 | 1.04e-44 |
| APZ78768.1 | nonribosomal\_peptide\_synthetase | BGC0001425 | NRP:Cyclic depsipeptide+Polyketide:Iterative type I polyketide | 32.0 | 35.6 | 179.0 | 1.04e-44 |
| ACR33075.1 | Proline\_adenylation\_protein | BGC0000017 | Alkaloid+Polyketide:Modular type I polyketide | 27.0 | 39.8 | 171.0 | 1.14e-44 |
| QXJ21807.1 | amino\_acid\_adenylation\_domain-containing\_protein | BGC0002370 | NRP | 26.0 | 63.6 | 179.0 | 1.21e-44 |
| TXC99989.1 | non-ribosomal\_peptide\_synthetase | BGC0001877 | Polyketide | 27.0 | 45.6 | 177.0 | 1.28e-44 |
| CAD89778.1 | MelG\_protein | BGC0001010 | NRP+Polyketide:Modular type I polyketide | 32.0 | 29.8 | 178.0 | 1.31e-44 |
| AET98905.1 | putative\_non-ribosomal\_peptide\_synthetase | BGC0000415 | NRP | 26.0 | 57.3 | 179.0 | 1.38e-44 |
| WP\_010369430.1 | non-ribosomal\_peptide\_synthetase | BGC0000314 | Polyketide+NRP:Cyclic depsipeptide+Other:Aminocoumarin | 24.0 | 52.9 | 178.0 | 1.83e-44 |
| ACS68554.1 | hybrid\_PKS-NRPS\_protein | BGC0001026 | NRP+Polyketide | 24.0 | 73.2 | 178.0 | 1.93e-44 |
| AXG46164.1 | non-ribosomal\_peptide\_synthetase | BGC0002713 | NRP | 26.0 | 53.5 | 177.0 | 2.05e-44 |
| QIE08738.1 | non-ribosomal\_peptide\_synthetase | BGC0002544 | NRP | 28.0 | 38.4 | 177.0 | 2.11e-44 |
| QGZ36672.1 | amino\_acid\_adenylation\_domain-containing\_protein | BGC0002082 | NRP+Polyketide | 26.0 | 64.9 | 178.0 | 2.13e-44 |
| ABV56604.1 | adenylation\_domain\_protein | BGC0000378 | NRP | 27.0 | 43.9 | 172.0 | 2.18e-44 |
| QRN75754.1 | Polyketide\_synthase | BGC0002114 | NRP+Polyketide | 29.0 | 36.5 | 178.0 | 2.46e-44 |
| OAL11475.1 | non-ribosomal\_peptide\_synthetase\_module | BGC0001570 | NRP | 28.0 | 45.9 | 172.0 | 2.46e-44 |
| BAD55612.1 | non-ribosomal\_peptide\_synthetase | BGC0001027 | NRP+Polyketide | 29.0 | 45.9 | 177.0 | 2.6e-44 |
| ALJ49909.1 | TlmI | BGC0001237 | Polyketide | 27.0 | 50.7 | 176.0 | 2.67e-44 |
| BAO84868.1 | putative\_non-ribosomal\_peptide\_synthetase | BGC0000414 | NRP | 30.0 | 41.7 | 175.0 | 3.04e-44 |
| WP\_028678148.1 | non-ribosomal\_peptide\_synthetase | BGC0001228 | NRP:Cyclic depsipeptide | 28.0 | 53.1 | 177.0 | 3.1e-44 |
| AZM51139.1 | non-ribosomal\_peptide\_synthetase | BGC0002702 | NRP | 30.0 | 36.5 | 177.0 | 3.13e-44 |
| DAB41917.1 | ArzO\_-\_NRPS\_(Cy,\_A,\_Ox,\_PCP) | BGC0001884 | NRP+Polyketide | 29.0 | 35.1 | 177.0 | 3.15e-44 |
| AGC09519.1 | AMP-dependent\_synthetase/ligase | BGC0001183 | Polyketide | 28.0 | 45.8 | 176.0 | 3.28e-44 |
| AGI89788.1 | Nonribosomal\_peptide\_synthetase | BGC0001792 | NRP | 31.0 | 38.9 | 177.0 | 3.35e-44 |
| CAG23957.2 | hybrid\_NRPS/PKS\_protein | BGC0001089 | Polyketide+NRP | 26.0 | 36.0 | 177.0 | 4.58e-44 |
| AXF14775.1 | non-ribosomal\_peptide\_synthetase | BGC0002563 | NRP | 28.0 | 46.2 | 176.0 | 5.47e-44 |
| QYC40287.1 | A50926\_NRPS,\_modules\_1-2 | BGC0002344 | NRP | 31.0 | 36.3 | 176.0 | 5.81e-44 |
| ctg1\_orf1264 |  | BGC0001752 | NRP | 27.0 | 54.3 | 177.0 | 5.82e-44 |
| KON97029.1 | phenylalanine\_racemase | BGC0002122 | NRP | 27.0 | 34.5 | 175.0 | 5.96e-44 |
| AAN85493.1 | nonribosomal\_peptide\_synthetase | BGC0001101 | NRP+Polyketide:Modular type I polyketide+Polyketide:Trans-AT type I polyketide | 29.0 | 42.1 | 175.0 | 6.05e-44 |
| ABX37382.1 | amino\_acid\_adenylation\_domain\_protein | BGC0000984 | NRP+Polyketide | 28.0 | 45.6 | 177.0 | 6.22e-44 |
| ADH04660.1 | TugD | BGC0001342 | NRP+Polyketide | 31.0 | 35.3 | 177.0 | 6.28e-44 |
| APZ78743.1 | nonribosomal\_peptide\_synthetase | BGC0001422 | NRP:Cyclic depsipeptide+Polyketide:Iterative type I polyketide | 31.0 | 36.1 | 176.0 | 6.92e-44 |
| EFG10344.1 | Non-ribosomal\_peptide\_synthetase | BGC0000373 | NRP | 28.0 | 44.8 | 175.0 | 7.82e-44 |
| QCF28926.1 | type\_I\_polyketide\_synthase | BGC0002308 | Alkaloid+Polyketide | 32.0 | 36.3 | 176.0 | 7.83e-44 |
| ATY69551.1 | hybrid\_nonribosomal\_peptide\_synthetase/type\_I\_polyketide\_synthase | BGC0001611 | NRP+Polyketide | 26.0 | 50.8 | 176.0 | 8.11e-44 |
| APZ78855.1 | nonribosomal\_peptide\_synthetase | BGC0001432 | NRP:Cyclic depsipeptide+Polyketide:Iterative type I polyketide | 31.0 | 35.2 | 176.0 | 9.08e-44 |
| BAP05589.1 | calA | BGC0000967 | NRP+Polyketide:Trans-AT type I polyketide | 26.0 | 37.5 | 176.0 | 9.69e-44 |
| CAE53350.1 | non-ribosomal\_peptide\_synthetase | BGC0000440 | NRP:Glycopeptide | 31.0 | 36.1 | 176.0 | 9.95e-44 |
| CAG15009.1 | peptide\_synthetase,\_module\_1-2 | BGC0000441 | NRP | 31.0 | 36.1 | 176.0 | 9.95e-44 |
| AAK81825.1 | peptide\_synthetase | BGC0000326 | NRP | 30.0 | 37.4 | 175.0 | 1.02e-43 |
| AFK57219.1 | DidH | BGC0000985 | Polyketide+NRP:Cyclic depsipeptide | 31.0 | 35.3 | 175.0 | 1.07e-43 |
| WP\_141576286.1 | non-ribosomal\_peptide\_synthetase | BGC0002686 | NRP | 30.0 | 35.3 | 174.0 | 1.08e-43 |
| MBE3200467.1 | amino\_acid\_adenylation\_domain-containing\_protein | BGC0002409 | NRP | 29.0 | 42.9 | 169.0 | 1.12e-43 |
| AZC86156.1 | peramine\_synthetase | BGC0002166 | NRP | 29.0 | 37.7 | 175.0 | 1.51e-43 |
| APZ78845.1 | nonribosomal\_peptide\_synthetase | BGC0001431 | NRP:Cyclic depsipeptide+Polyketide:Iterative type I polyketide | 32.0 | 34.7 | 175.0 | 1.56e-43 |
| BBC43184.1 | PKS-NRPS\_hybrid | BGC0001738 | NRP+Polyketide | 26.0 | 59.8 | 175.0 | 1.68e-43 |
| BGRAMDRAFT\_RS22640 | amino\_acid\_adenylation\_domain-containing\_protein | BGC0001999 | NRP | 26.0 | 66.0 | 174.0 | 1.81e-43 |
| AQV04224.1 | SwnK | BGC0001793 | NRP+Polyketide | 28.0 | 37.0 | 175.0 | 1.89e-43 |
| ABY83146.1 | Azi7 | BGC0000960 | NRP+Polyketide | 26.0 | 47.4 | 169.0 | 1.94e-43 |
| TRX17523.1 | non-ribosomal\_peptide\_synthetase | BGC0002329 | NRP | 25.0 | 69.5 | 174.0 | 2.12e-43 |
| ABC35796.1 | NRPS-PKS\_hybrid | BGC0001102 | NRP+Polyketide:Modular type I polyketide+Polyketide:Trans-AT type I polyketide | 33.0 | 28.4 | 175.0 | 2.22e-43 |
| CCM44336.1 | Nonribosomal\_peptide\_synthetase | BGC0001056 | NRP+Polyketide:Modular type I polyketide+Polyketide:PUFA synthase or related polyketide | 29.0 | 37.0 | 175.0 | 2.23e-43 |
| QBF51786.1 | Nrps | BGC0001856 | Polyketide:Modular type I polyketide | 27.0 | 44.0 | 173.0 | 2.51e-43 |
| CAD89774.1 | MelC\_protein | BGC0001010 | NRP+Polyketide:Modular type I polyketide | 27.0 | 44.1 | 174.0 | 2.54e-43 |
| EFL06866.1 | predicted\_protein | BGC0000300 | NRP | 27.0 | 49.1 | 174.0 | 2.74e-43 |
| AAD44233.1 | PstA | BGC0000362 | NRP | 30.0 | 36.9 | 174.0 | 2.78e-43 |
| BCK51628.1 | non-ribosomal\_peptide\_synthetase | BGC0002520 | Polyketide | 31.0 | 35.2 | 172.0 | 3.06e-43 |
| AGI89791.1 | Nonribosomal\_peptide\_synthetase | BGC0001792 | NRP | 29.0 | 35.4 | 174.0 | 3.11e-43 |
| OKJ61999.1 | peptide\_synthetase | BGC0002147 | NRP | 29.0 | 45.8 | 174.0 | 3.19e-43 |
| EPE34341.1 | non-ribosomal\_peptide\_synthetase | BGC0001035 | Polyketide+NRP | 27.0 | 45.8 | 174.0 | 3.21e-43 |
| BAX89998.1 | Non-ribosomal\_peptide\_synthetase | BGC0001628 | NRP | 27.0 | 47.9 | 174.0 | 3.29e-43 |
| iliA |  | BGC0002035 | NRP+Polyketide | 24.0 | 79.9 | 174.0 | 3.81e-43 |
| QIW91877.1 | NRPS | BGC0002543 | NRP | 25.0 | 64.6 | 174.0 | 5.53e-43 |
| AID65225.1 | nonribosomal\_peptide\_synthetase | BGC0000335 | NRP+Polyketide | 26.0 | 56.4 | 173.0 | 5.92e-43 |
| KKP04599.1 | Non-ribosomal\_peptide\_synthetase\_-\_Polyketide\_synthase | BGC0002066 | NRP+Polyketide:Iterative type I polyketide | 24.0 | 62.5 | 173.0 | 6.51e-43 |
| CAD91220.1 | putative\_non-ribosomal\_peptide\_synthetase,\_modules\_1-2 | BGC0000289 | NRP:Glycopeptide+Saccharide:Hybrid/tailoring saccharide | 30.0 | 36.1 | 173.0 | 6.69e-43 |
| AAW03326.1 | CtaC | BGC0000982 | NRP+Polyketide | 28.0 | 39.9 | 172.0 | 7.47e-43 |
| AAF19815.1 | mtaG | BGC0001024 | NRP+Polyketide:Modular type I polyketide | 32.0 | 30.5 | 172.0 | 7.76e-43 |
| AQM37583.1 | nonribosomal\_peptide\_synthetase | BGC0001424 | NRP:Cyclic depsipeptide+Polyketide:Iterative type I polyketide | 32.0 | 35.4 | 173.0 | 7.92e-43 |
| CAG29031.1 | nonribosomal\_peptide\_synthetase\_(modules\_1\_and\_2) | BGC0001023 | NRP+Polyketide:Modular type I polyketide | 31.0 | 35.4 | 173.0 | 7.93e-43 |
| APZ78821.1 | nonribosomal\_peptide\_synthetase | BGC0001429 | NRP:Cyclic depsipeptide+Polyketide:Iterative type I polyketide | 31.0 | 35.4 | 173.0 | 7.93e-43 |
| BAG23199.1 | putative\_type-I\_PKS | BGC0002673 | Polyketide+Alkaloid | 32.0 | 34.5 | 172.0 | 8.79e-43 |
| CAJ77695.1 | MPS1\_protein | BGC0000363 | NRP | 30.0 | 36.3 | 172.0 | 1.08e-42 |
| EXU96269.1 | nonribosomal\_peptide\_synthetase,\_serinocyclin\_synthetase\_NPS1 | BGC0001240 | NRP | 27.0 | 46.1 | 172.0 | 1.28e-42 |
| AWI62629.1 | nonribosomal\_peptide\_synthetase | BGC0001822 | NRP | 31.0 | 34.9 | 172.0 | 1.36e-42 |
| BCK51629.1 | non-ribosomal\_peptide\_synthetase | BGC0002520 | Polyketide | 32.0 | 35.6 | 171.0 | 1.53e-42 |
| BCD58482.1 | gamma-poly-L-2,4-diaminobutyric\_acid\_synthetase | BGC0002535 | NRP | 30.0 | 37.1 | 171.0 | 1.77e-42 |
| AFP87523.1 | type\_I\_polyketide\_synthase | BGC0001159 | NRP+Polyketide:Modular type I polyketide | 28.0 | 35.7 | 171.0 | 2.06e-42 |
| XP\_020058100.1 | uncharacterized\_protein | BGC0001220 | NRP | 25.0 | 51.0 | 172.0 | 2.14e-42 |
| CAQ18839.1 | hybrid\_polyketide\_synthase/nonribosomal\_polypetide\_synthetase | BGC0000954 | NRP+Polyketide:Modular type I polyketide | 29.0 | 33.8 | 171.0 | 2.36e-42 |
| QDQ83033.1 | amino\_acid\_adenylation\_domain-containing\_protein | BGC0002564 | NRP | 29.0 | 46.0 | 171.0 | 3.14e-42 |
| ADY16697.1 | TqaA | BGC0001142 | NRP | 27.0 | 46.2 | 171.0 | 3.33e-42 |
| CAL69597.1 | PKS-NRPS | BGC0001049 | NRP+Polyketide:Iterative type I polyketide | 24.0 | 66.4 | 171.0 | 3.36e-42 |
| AFK57212.1 | DidA | BGC0000985 | Polyketide+NRP:Cyclic depsipeptide | 29.0 | 37.5 | 171.0 | 3.43e-42 |
| AGN74898.1 | nonribosomal\_peptide\_synthetase | BGC0000459 | NRP:Cyclic depsipeptide+Polyketide:Trans-AT type I polyketide | 28.0 | 41.6 | 168.0 | 3.64e-42 |
| CBJ90359.1 | putative\_Peptide\_synthetase | BGC0000465 | NRP | 24.0 | 56.5 | 170.0 | 4e-42 |
| AAY42397.1 | Nonribosomal\_peptide\_synthetase | BGC0001000 | NRP:Lipopeptide+Polyketide:Modular type I polyketide | 28.0 | 34.6 | 171.0 | 4.14e-42 |
| AZF85929.1 | non-ribosomal\_peptide\_synthase | BGC0001963 | NRP+Polyketide | 27.0 | 47.5 | 169.0 | 4.17e-42 |
| XP\_002379984.1 | nonribosomal\_peptide\_synthase,\_putative | BGC0001621 | NRP | 25.0 | 64.8 | 170.0 | 4.44e-42 |
| AAK81827.1 | peptide\_synthetase | BGC0000326 | NRP | 30.0 | 36.4 | 170.0 | 4.55e-42 |
| ALG65336.1 | Var3 | BGC0002416 | NRP+Polyketide | 30.0 | 40.7 | 170.0 | 5.18e-42 |
| WP\_035121683.1 | non-ribosomal\_peptide\_synthetase | BGC0002624 | NRP+Polyketide | 28.0 | 34.5 | 169.0 | 5.58e-42 |
| AFD30953.1 | CrmB | BGC0000966 | NRP+Polyketide | 28.0 | 47.2 | 169.0 | 5.78e-42 |
| AQZ71347.1 | hypothetical\_protein | BGC0001635 | NRP+Polyketide | 30.0 | 33.8 | 169.0 | 5.94e-42 |
| BAW27693.1 | NRPS(C-A-T-TE) | BGC0001764 | NRP | 29.0 | 36.7 | 169.0 | 6.92e-42 |
| AAT01806.1 | non-ribosomal\_peptide\_synthetase | BGC0000365 | NRP | 30.0 | 37.0 | 170.0 | 7.16e-42 |
| BS330\_28385 | non-ribosomal\_peptide\_synthetase | BGC0001461 | NRP:Glycopeptide | 30.0 | 38.0 | 169.0 | 7.66e-42 |
| CBF76036.1 | putative\_nonribosomal\_peptide\_synthetase\_(Eurofung) | BGC0001399 | NRP | 27.0 | 46.3 | 169.0 | 8.12e-42 |
| QCC62999.1 | BII-rafflesfungin\_nonribosomal\_protein\_synthetase | BGC0001966 | NRP+Polyketide | 27.0 | 49.1 | 170.0 | 8.55e-42 |
| AYA22336.1 | KerA | BGC0001955 | NRP | 30.0 | 38.0 | 169.0 | 1e-41 |
| ABI22132.1 | putative\_non-ribosomal\_peptide\_synthetase | BGC0000422 | NRP | 31.0 | 36.0 | 168.0 | 1.08e-41 |
| ABM91454.1 | lysergyl\_peptide\_synthetase\_LpsB | BGC0000348 | NRP | 26.0 | 47.8 | 168.0 | 1.2e-41 |
| CAC17500.1 | putative\_non-ribosomal\_peptide\_synthase | BGC0000324 | NRP | 28.0 | 33.0 | 169.0 | 1.22e-41 |
| ABV56588.1 | KtzH | BGC0000378 | NRP | 29.0 | 35.6 | 169.0 | 1.35e-41 |
| QRN75755.1 | Amino\_acid\_adenylation\_domain\_protein | BGC0002114 | NRP+Polyketide | 30.0 | 36.1 | 168.0 | 1.75e-41 |
| PLB34720.1 | polyketide\_synthase | BGC0002749 | NRP+Polyketide | 28.0 | 36.5 | 168.0 | 1.89e-41 |
| AMK48225.1 | nonribosomal\_peptide\_synthetase | BGC0001351 | NRP | 30.0 | 35.8 | 167.0 | 2.15e-41 |
| AZM50110.1 | non-ribosomal\_peptide\_synthetase | BGC0002702 | NRP | 31.0 | 35.2 | 167.0 | 2.39e-41 |
| ctg3\_18 |  | BGC0001853 | NRP+Polyketide:Modular type I polyketide | 28.0 | 40.3 | 168.0 | 2.93e-41 |
| BAD55613.1 | putative\_non-ribosomal\_peptide\_synthetase | BGC0001027 | NRP+Polyketide | 28.0 | 47.7 | 166.0 | 3.77e-41 |
| CAJ76289.1 | putative\_hybrid\_non-ribosomal\_peptide-polyketide\_synthetase | BGC0000972 | NRP+Polyketide:Modular type I polyketide+Polyketide:Trans-AT type I polyketide | 30.0 | 34.0 | 167.0 | 3.97e-41 |
| CAG15010.1 | peptide\_synthetase,\_module\_3 | BGC0000441 | NRP | 29.0 | 34.1 | 166.0 | 4.08e-41 |
| AEW95634.1 | non-ribosomal\_peptide\_synthetase | BGC0002697 | NRP+Polyketide | 28.0 | 46.1 | 167.0 | 4.18e-41 |
| AAW03330.1 | CtaG | BGC0000982 | NRP+Polyketide | 35.0 | 25.3 | 167.0 | 4.56e-41 |
| ABX71145.1 | Lcz28 | BGC0000237 | Polyketide | 28.0 | 39.2 | 160.0 | 4.98e-41 |
| AAW49319.1 | thaxtomin\_synthetase\_B | BGC0000444 | NRP | 32.0 | 33.8 | 166.0 | 6.92e-41 |
| CAA79245.2 | enniatin\_synthetase | BGC0000342 | NRP | 24.0 | 46.3 | 166.0 | 7.96e-41 |
| AAF19812.1 | MtaD | BGC0001024 | NRP+Polyketide:Modular type I polyketide | 30.0 | 34.4 | 166.0 | 8.08e-41 |
| AAL33756.1 | putative\_non-ribosomal\_peptide\_synthetase | BGC0000421 | NRP | 28.0 | 36.9 | 165.0 | 8.13e-41 |
| AAY42398.1 | Nonribosomal\_peptide\_synthetase | BGC0001000 | NRP:Lipopeptide+Polyketide:Modular type I polyketide | 28.0 | 38.8 | 166.0 | 8.48e-41 |
| AJO72717.1 | adenylation\_domain-containing\_protein | BGC0001381 | Polyketide | 26.0 | 46.5 | 165.0 | 8.81e-41 |
| CAE53351.1 | non-ribosomal\_peptide\_synthetase | BGC0000440 | NRP:Glycopeptide | 29.0 | 33.8 | 165.0 | 9.33e-41 |
| ACG60773.1 | NRPS(C/A/PCP) | BGC0001058 | NRP:Glycopeptide+Polyketide:Modular type I polyketide+Saccharide:Hybrid/tailoring saccharide | 26.0 | 52.8 | 165.0 | 1.02e-40 |
| AFU82614.1 | mixed\_NRPS\_PKS | BGC0000998 | NRP+Polyketide | 28.0 | 44.7 | 166.0 | 1.25e-40 |
| AGE11898.1 | nonribosomal\_peptide\_synthetase | BGC0000366 | NRP | 29.0 | 46.0 | 166.0 | 1.28e-40 |
| AFR69334.1 | nonribosomal\_peptide\_synthetase\_SpiDE1 | BGC0001045 | NRP:Cyclic depsipeptide+Polyketide:Modular type I polyketide | 30.0 | 36.1 | 166.0 | 1.47e-40 |
| AAK81824.1 | peptide\_synthetase | BGC0000326 | NRP | 31.0 | 35.5 | 165.0 | 1.53e-40 |
| CBG70278.1 | thaxtomin\_synthetase\_B | BGC0002089 | NRP | 32.0 | 33.9 | 165.0 | 1.55e-40 |
| CDG12865.1 | non-ribosomal\_peptide\_synthetase | BGC0001415 | NRP+Polyketide | 25.0 | 46.5 | 165.0 | 1.62e-40 |
| AAK89720.1 | non-ribosomal\_peptide\_synthetase,\_siderophore\_biosynthesis\_protein | BGC0002107 | NRP+Polyketide | 30.0 | 36.6 | 165.0 | 1.62e-40 |
| ctg1\_orf18 |  | BGC0001329 | Polyketide+NRP:Cyclic depsipeptide | 31.0 | 30.0 | 155.0 | 1.72e-40 |
| ACN39728.1 | SibE | BGC0000428 | NRP | 26.0 | 45.8 | 160.0 | 1.94e-40 |
| QYA95659.1 | amino\_acid\_adenylation\_domain-containing\_protein | BGC0002676 | NRP | 27.0 | 46.2 | 164.0 | 1.99e-40 |
| CAJ96471.1 | non-ribosomal\_peptide\_synthetase | BGC0000330 | NRP:NRP siderophore | 29.0 | 36.5 | 164.0 | 1.99e-40 |
| AQV04230.1 | SwnK | BGC0001794 | NRP+Polyketide | 26.0 | 37.5 | 165.0 | 2.17e-40 |
| AZH23788.1 | MgcR | BGC0001970 | NRP+Polyketide | 27.0 | 36.7 | 165.0 | 2.19e-40 |
| AJY78094.1 | nonribosomal\_peptide\_synthetase | BGC0001902 | NRP+Polyketide | 27.0 | 41.0 | 164.0 | 2.4e-40 |
| AMM63162.1 | AniA | BGC0001371 | NRP | 27.0 | 46.1 | 165.0 | 2.77e-40 |
| ABI22131.1 | putative\_non-ribosomal\_peptide\_synthetase | BGC0000422 | NRP | 30.0 | 35.8 | 164.0 | 3.16e-40 |
| WP\_081656241.1 | non-ribosomal\_peptide\_synthetase | BGC0001467 | NRP:Cyclic depsipeptide+Polyketide:Modular type I polyketide | 24.0 | 62.1 | 164.0 | 3.62e-40 |
| AQX14495.1 | monobactam\_NRPS\_scaffold\_3 | BGC0001672 | NRP | 24.0 | 45.4 | 164.0 | 3.64e-40 |
| OTA20325.1 | peptide\_synthase | BGC0001824 | NRP | 25.0 | 45.5 | 164.0 | 4.27e-40 |
| ADI59531.1 | CorI | BGC0001091 | NRP+Polyketide | 25.0 | 46.6 | 164.0 | 4.28e-40 |
| ACB46193.1 | nonribosomal\_peptide\_synthetase | BGC0000989 | NRP+Polyketide | 28.0 | 34.8 | 163.0 | 4.35e-40 |
| CBJ90358.1 | putative\_Peptide\_synthetase | BGC0000465 | NRP | 25.0 | 57.1 | 164.0 | 4.38e-40 |
| AEI58865.1 | peptide\_synthetase | BGC0000455 | NRP | 29.0 | 36.1 | 164.0 | 5.3e-40 |
| AAK89731.2 | siderophore\_biosynthesis\_protein | BGC0002107 | NRP+Polyketide | 29.0 | 36.1 | 162.0 | 5.49e-40 |
| EYT83459.1 | hypothetical\_protein | BGC0001213 | Polyketide | 32.0 | 26.5 | 154.0 | 5.64e-40 |
| ADB12489.1 | EpoB | BGC0000990 | NRP+Polyketide | 29.0 | 34.9 | 163.0 | 5.71e-40 |
| CBG70279.1 | thaxtomin\_synthetase\_A | BGC0002089 | NRP | 30.0 | 36.8 | 163.0 | 5.92e-40 |
| WP\_020993844.1 | non-ribosomal\_peptide\_synthetase | BGC0001575 | NRP | 25.0 | 45.6 | 163.0 | 6.84e-40 |
| ATJ34005.1 | adenylation\_domain-containing\_protein | BGC0001442 | NRP | 28.0 | 39.8 | 156.0 | 6.97e-40 |
| SDF67478.1 | Phosphopantetheine\_attachment\_site | BGC0002422 | NRP | 25.0 | 54.8 | 162.0 | 7e-40 |
| CAJ77715.1 | Mps1\_protein | BGC0000364 | NRP | 29.0 | 35.4 | 163.0 | 7.14e-40 |
| AAS98783.1 | polyketide\_synthase/nonribosomal\_peptide\_synthase\_hybrid | BGC0001001 | NRP+Polyketide | 32.0 | 23.6 | 163.0 | 7.37e-40 |
| AEA29644.1 | putative\_nonribosomal\_peptide\_synthetase\_and\_kinurenine\_monooxygenase | BGC0000409 | NRP | 27.0 | 54.8 | 163.0 | 7.39e-40 |
| AAF26925.1 | nonribosomal\_peptide\_synthetase | BGC0000988 | NRP+Polyketide | 28.0 | 34.8 | 162.0 | 7.5e-40 |
| QCE43603.1 | nonribosomal\_peptide\_synthetase\_(NRPS),\_subunit\_2 | BGC0001834 | NRP | 25.0 | 57.9 | 163.0 | 7.94e-40 |
| QCX41915.1 | Mhr9 | BGC0001956 | Polyketide | 30.0 | 34.0 | 160.0 | 1.06e-39 |
| QHD26315.1 | non-ribosomal\_peptide\_synthetase | BGC0002479 | Polyketide+NRP+Saccharide | 30.0 | 34.0 | 160.0 | 1.06e-39 |
| AAM80538.1 | StaB | BGC0000290 | NRP:Glycopeptide | 28.0 | 35.6 | 162.0 | 1.37e-39 |
| BAU50944.1 | nonribosomal\_peptide\_synthetase | BGC0001379 | NRP | 27.0 | 48.4 | 157.0 | 1.42e-39 |
| ACK77757.1 | putative\_indigoidine\_synthase\_IndC | BGC0000727 | Saccharide | 27.0 | 33.2 | 161.0 | 1.52e-39 |
| CAA11794.1 | PCZA363.3 | BGC0000322 | NRP | 30.0 | 34.6 | 162.0 | 1.57e-39 |
| QRI43520.1 | NRPS/PKS\_hybrid | BGC0002454 | Polyketide | 30.0 | 35.6 | 162.0 | 1.62e-39 |
| QIE08736.1 | non-ribosomal\_peptide\_synthetase | BGC0002544 | NRP | 31.0 | 37.3 | 162.0 | 1.65e-39 |
| BBC83957.1 | nonribosomal\_peptide\_synthetase | BGC0001636 | NRP | 29.0 | 35.5 | 162.0 | 1.92e-39 |
| CAD91221.1 | putative\_non-ribosomal\_peptide\_synthetase,\_module\_3 | BGC0000289 | NRP:Glycopeptide+Saccharide:Hybrid/tailoring saccharide | 29.0 | 40.0 | 160.0 | 1.93e-39 |
| BAE98156.1 | putative\_non-ribosomal\_peptide\_synthetase | BGC0000339 | NRP | 26.0 | 52.7 | 162.0 | 2.05e-39 |
| CAJ34381.1 | NRPS\_protein | BGC0000445 | NRP:Cyclic depsipeptide | 28.0 | 36.2 | 161.0 | 2.09e-39 |
| NSC23530.1 | amino\_acid\_adenylation\_domain-containing\_protein | BGC0002359 | NRP | 29.0 | 46.4 | 161.0 | 2.16e-39 |
| AMQ36132.1 | PsyA | BGC0002617 | NRP | 25.0 | 46.7 | 161.0 | 2.33e-39 |
| ADC79642.1 | TamD | BGC0001052 | NRP+Polyketide:Modular type I polyketide | 30.0 | 35.8 | 160.0 | 2.48e-39 |
| BAW27670.1 | NRPS(A-T) | BGC0001764 | NRP | 28.0 | 36.4 | 156.0 | 2.5e-39 |
| QXJ26485.1 | amino\_acid\_adenylation\_domain-containing\_protein | BGC0002370 | NRP | 27.0 | 45.6 | 160.0 | 2.63e-39 |
| BAJ19066.1 | L-lysine\_activating\_non-ribosomal\_peptide\_synthetase | BGC0000288 | NRP | 26.0 | 54.4 | 160.0 | 2.73e-39 |
| MBA0053739.1 | acyltransferase\_domain-containing\_protein | BGC0002096 | Polyketide | 29.0 | 38.0 | 160.0 | 3.14e-39 |
| ABF85931.1 | non-ribosomal\_peptide\_synthase/polyketide\_synthase\_Ta1 | BGC0001025 | NRP+Polyketide:Trans-AT type I polyketide | 28.0 | 33.7 | 161.0 | 3.21e-39 |
| DAC80525.1 | malonyl\_CoA-acyl\_carrier\_protein\_transacylase | BGC0001841 | NRP+Polyketide | 28.0 | 39.0 | 161.0 | 3.39e-39 |
| ABS75103.1 | non-ribosomal\_peptide\_synthetase | BGC0002641 | NRP | 26.0 | 36.4 | 161.0 | 3.6e-39 |
| AAF62881.1 | EpoB | BGC0000991 | NRP+Polyketide | 29.0 | 34.7 | 160.0 | 3.84e-39 |
| AZF85912.1 | non-ribosomal\_peptide\_synthase | BGC0001963 | NRP+Polyketide | 29.0 | 36.5 | 159.0 | 4.11e-39 |
| EFL02193.1 | amino\_acid\_adenylation\_domain-containing\_protein | BGC0000996 | NRP+Polyketide:Iterative type I polyketide | 25.0 | 62.5 | 160.0 | 4.46e-39 |
| AXM42952.1 | non-ribosomal\_peptide\_synthetase | BGC0001941 | NRP+Polyketide | 33.0 | 29.1 | 160.0 | 4.61e-39 |
| BAP82667.1 | non-ribosomal\_peptide\_synthetase\_A-domain\_containing\_protein | BGC0001148 | NRP+RiPP | 28.0 | 40.9 | 156.0 | 5e-39 |
| KJY85279.1 | long-chain\_fatty\_acid--CoA\_ligase | BGC0002491 | NRP | 25.0 | 59.0 | 160.0 | 5.17e-39 |
| NAO96317.1 | amino\_acid\_adenylation\_domain-containing\_protein | BGC0002117 | NRP | 26.0 | 39.3 | 159.0 | 5.77e-39 |
| CAI94718.1 | putative\_CoA\_ligase | BGC0000141 | Polyketide | 28.0 | 37.1 | 159.0 | 6.15e-39 |
| KPN90374.1 | NunB1 | BGC0001416 | NRP | 25.0 | 46.2 | 155.0 | 6.4e-39 |
| AAN85501.1 | nonribosomal\_peptide\_synthetase | BGC0001101 | NRP+Polyketide:Modular type I polyketide+Polyketide:Trans-AT type I polyketide | 25.0 | 58.4 | 159.0 | 6.53e-39 |
| CBZ42140.1 | non-ribosomal\_peptide\_synthetase | BGC0001117 | NRP | 28.0 | 46.0 | 159.0 | 6.58e-39 |
| AZH23819.1 | MgiR | BGC0001971 | NRP+Polyketide | 28.0 | 36.8 | 160.0 | 6.6e-39 |
| BAF50727.1 | hybrid\_polyketide\_synthase-non\_ribosomal\_peptide\_synthetase | BGC0001116 | NRP+Polyketide | 30.0 | 35.2 | 160.0 | 7.03e-39 |
| ADA82585.1 | hybrid\_trans-AT\_polyketide\_synthase\_-\_nonribosomal\_peptide\_synthetase | BGC0001110 | NRP+Polyketide:Trans-AT type I polyketide | 27.0 | 39.2 | 160.0 | 7.4e-39 |
| AHD05616.1 | putative\_non-ribosomal\_peptide\_ligase\_domain\_protein | BGC0001033 | NRP+Polyketide | 27.0 | 35.7 | 159.0 | 8.02e-39 |
| CBK62746.1 |  | BGC0001115 | NRP+Polyketide | 25.0 | 44.4 | 159.0 | 8.44e-39 |
| AAW49318.1 | thaxtomin\_synthetase\_A | BGC0000444 | NRP | 30.0 | 37.9 | 159.0 | 8.96e-39 |
| CDE97356.1 | plipastatin\_synthase\_subunit\_C | BGC0001686 | NRP | 27.0 | 36.4 | 159.0 | 9.55e-39 |
| QIQ51365.1 | hypothetical\_protein | BGC0002199 | Alkaloid | 27.0 | 46.1 | 159.0 | 9.58e-39 |
| ctg1\_orf000000 |  | BGC0000901 | Other | 26.0 | 46.2 | 133.0 | 1.3e-30 |
| EAL89049.1 | nonribosomal\_peptide\_synthetase | BGC0000355 | NRP | 27.0 | 44.0 | 159.0 | 1.1e-38 |
| SDF67357.1 | amino\_acid\_adenylation\_domain-containing\_protein | BGC0002422 | NRP | 29.0 | 37.5 | 158.0 | 1.21e-38 |
| WP\_051872436.1 | non-ribosomal\_peptide\_synthetase | BGC0001771 | NRP | 27.0 | 46.3 | 158.0 | 1.28e-38 |
| ACU36654.1 | amino\_acid\_adenylation\_domain\_protein | BGC0000392 | NRP | 29.0 | 35.1 | 159.0 | 1.39e-38 |
| AKJ70942.1 | non-ribosomal\_peptide\_synthetase | BGC0002611 | NRP | 28.0 | 37.6 | 159.0 | 1.59e-38 |
| AQX14441.1 | EM5400\_NRPS\_scaffold | BGC0001671 | NRP | 27.0 | 45.7 | 159.0 | 1.73e-38 |
| ACG60778.1 | NRPS(A/PCP) | BGC0001058 | NRP:Glycopeptide+Polyketide:Modular type I polyketide+Saccharide:Hybrid/tailoring saccharide | 27.0 | 41.6 | 154.0 | 1.96e-38 |
| CBF87069.1 | nonribosomal\_peptide\_synthase,\_putative\_(Eurofung) | BGC0001290 | NRP | 27.0 | 46.3 | 159.0 | 2.07e-38 |
| AEC14348.1 | nonribosomal\_peptide\_synthetase | BGC0000377 | NRP | 27.0 | 37.2 | 158.0 | 2.87e-38 |
| AKA59447.1 | non-ribosomal\_peptide\_synthetase | BGC0001203 | NRP+Polyketide | 29.0 | 39.2 | 158.0 | 3.36e-38 |
| AGC83576.1 | NRPS | BGC0000818 | NRP | 27.0 | 44.6 | 157.0 | 3.54e-38 |
| ABL74940.1 | NRPS | BGC0001048 | NRP:Glycopeptide+Polyketide:Modular type I polyketide+Saccharide:Hybrid/tailoring saccharide | 27.0 | 39.3 | 157.0 | 3.85e-38 |
| CCC55927.1 | hypothetical\_protein | BGC0000973 | NRP+Polyketide:Modular type I polyketide | 29.0 | 34.5 | 157.0 | 4.3e-38 |
| ANG60381.1 | nonribosomal\_peptide\_synthetase\_BudC | BGC0001434 | NRP | 23.0 | 57.4 | 157.0 | 4.4e-38 |
| ACZ66258.1 | APS1 | BGC0000304 | NRP | 27.0 | 45.4 | 157.0 | 4.47e-38 |
| ACG60782.1 | NRPS(C/A/PCP/C/A/PCP) | BGC0001058 | NRP:Glycopeptide+Polyketide:Modular type I polyketide+Saccharide:Hybrid/tailoring saccharide | 28.0 | 41.6 | 157.0 | 4.54e-38 |
| AUS29484.1 | non-ribosomal\_peptide\_synthetase | BGC0002605 | NRP+Polyketide | 27.0 | 45.4 | 157.0 | 5.56e-38 |
| ABW70809.1 | PchE | BGC0002475 | NRP | 28.0 | 35.2 | 156.0 | 6.02e-38 |
| QYC40288.1 | A50926\_NRPS,\_module\_3 | BGC0002344 | NRP | 29.0 | 37.5 | 155.0 | 6.87e-38 |
| DAC80540.1 | nrps | BGC0001840 | NRP+Polyketide | 28.0 | 45.6 | 157.0 | 6.9e-38 |
| WP\_030185025.1 | thioester\_reductase\_domain-containing\_protein | BGC0001813 | NRP | 31.0 | 33.9 | 155.0 | 7.79e-38 |
| QKG86306.1 | non-ribosomal\_peptide\_synthetase | BGC0002254 | Polyketide | 23.0 | 63.7 | 156.0 | 8.01e-38 |
| ABP57748.1 | DepD | BGC0000993 | NRP:Cyclic depsipeptide+Polyketide:Modular type I polyketide | 28.0 | 40.9 | 156.0 | 8.97e-38 |
| AAG02343.1 | peptide\_synthetase\_NRPS12 | BGC0000963 | NRP:Glycopeptide+Polyketide:Modular type I polyketide+Saccharide:Hybrid/tailoring saccharide | 29.0 | 35.5 | 151.0 | 1.28e-37 |
| QTT72111.1 | non-ribosomal\_peptide\_synthetase | BGC0002350 | NRP+Polyketide+Saccharide | 29.0 | 35.5 | 151.0 | 1.28e-37 |
| QOE83925.1 | linear\_gramicidin\_synthase\_subunit\_B | BGC0002051 | NRP | 28.0 | 36.2 | 152.0 | 1.45e-37 |
| AAR87759.1 | ZmaJ | BGC0001059 | NRP+Polyketide | 27.0 | 34.8 | 150.0 | 1.49e-37 |
| AET79179.1 | lysergyl\_peptide\_synthetase\_subunit\_2 | BGC0001241 | Terpene | 26.0 | 45.8 | 155.0 | 1.6e-37 |
| CCE30235.1 | related\_to\_non-ribosomal\_peptide\_synthetase | BGC0002232 | Alkaloid | 26.0 | 45.8 | 155.0 | 1.6e-37 |
| QPB41099.1 | non-ribosomal\_peptide\_synthetase | BGC0002503 | NRP+Polyketide | 26.0 | 47.6 | 155.0 | 2.03e-37 |
| AAL33757.1 | putative\_non-ribosomal\_peptide\_synthetase | BGC0000421 | NRP | 29.0 | 35.6 | 154.0 | 2.06e-37 |
| AAW03327.1 | CtaD | BGC0000982 | NRP+Polyketide | 30.0 | 35.1 | 155.0 | 2.06e-37 |
| ABW70808.1 | PchF | BGC0002475 | NRP | 28.0 | 33.5 | 155.0 | 2.11e-37 |
| AKA59440.1 | non-ribosomal\_peptide\_synthetase | BGC0001202 | NRP+Polyketide | 29.0 | 34.3 | 150.0 | 2.16e-37 |
| QDX19369.1 | NRPS(A-Ox-T-TE-Tau) | BGC0002295 | NRP+Saccharide | 26.0 | 35.7 | 154.0 | 2.23e-37 |
| BAW32334.1 | hybrid\_cis-AT\_polyketide\_synthase\_-\_nonribosomal\_peptide\_synthetase | BGC0001631 | NRP+Polyketide | 26.0 | 35.7 | 155.0 | 2.84e-37 |
| ARR97039.1 | SphF | BGC0001780 | NRP | 27.0 | 36.5 | 154.0 | 3.9e-37 |
| ABW71853.1 | nonribosomal\_peptide\_synthetase | BGC0000303 | NRP | 25.0 | 52.1 | 154.0 | 4.03e-37 |
| EAU36744.1 | predicted\_protein | BGC0000292 | NRP | 27.0 | 35.6 | 153.0 | 5.48e-37 |
| CCJ67647.1 | JagC | BGC0001127 | NRP | 27.0 | 47.7 | 154.0 | 6.23e-37 |
| AFV27434.1 | indigoidine\_synthase | BGC0000375 | NRP | 28.0 | 33.9 | 153.0 | 6.62e-37 |
| ABX71111.1 | Lct28 | BGC0000238 | Polyketide | 26.0 | 39.1 | 147.0 | 7.01e-37 |
| WP\_069848004.1 | non-ribosomal\_peptide\_synthetase | BGC0002472 | NRP | 32.0 | 23.9 | 153.0 | 7.54e-37 |
| CAD89775.1 | MelD\_protein | BGC0001010 | NRP+Polyketide:Modular type I polyketide | 29.0 | 35.0 | 153.0 | 7.96e-37 |
| AGQ43600.1 | HC-toxin\_synthetase | BGC0001166 | NRP | 25.0 | 46.5 | 153.0 | 8.73e-37 |
| BAH33409.1 | putative\_non-ribosomal\_peptide\_synthetase | BGC0000371 | NRP | 28.0 | 45.1 | 153.0 | 1.1e-36 |
| EGX96627.1 | non-ribosomal\_peptide\_synthase,\_putative | BGC0002259 | Polyketide+NRP | 26.0 | 41.2 | 153.0 | 1.13e-36 |
| ATG32076.1 | nonribosomal\_peptide\_synthetase | BGC0001750 | NRP+Polyketide | 29.0 | 34.3 | 152.0 | 1.16e-36 |
| QGY73445.1 | Itm13 | BGC0002451 | Polyketide | 30.0 | 36.2 | 153.0 | 1.17e-36 |
| ACI30655.1 | BEAS\_beauvericin\_nonribosomal\_cyclodepsipeptide\_synthetase | BGC0000313 | NRP | 25.0 | 51.5 | 152.0 | 1.36e-36 |
| AFK57214.1 | DidC | BGC0000985 | Polyketide+NRP:Cyclic depsipeptide | 30.0 | 37.6 | 152.0 | 1.45e-36 |
| ALK21570.1 | non-ribosomal\_peptide\_synthetase | BGC0002678 | NRP | 28.0 | 36.6 | 152.0 | 1.86e-36 |
| AIG79242.1 | Non-ribosomal\_peptide\_synthetase | BGC0000419 | Saccharide+NRP:Glycopeptide | 29.0 | 35.6 | 150.0 | 2.38e-36 |
| AGN74892.1 | nonribosomal\_peptide\_synthetase/polyketide\_synthase\_hybrid\_protein | BGC0000459 | NRP:Cyclic depsipeptide+Polyketide:Trans-AT type I polyketide | 29.0 | 39.3 | 152.0 | 2.52e-36 |
| BAW32333.1 | nonribosomal\_peptide\_synthetase | BGC0001631 | NRP+Polyketide | 27.0 | 38.1 | 151.0 | 2.64e-36 |
| ESU05146.1 | hypothetical\_protein | BGC0002178 | NRP | 28.0 | 38.9 | 150.0 | 2.97e-36 |
| AHB38511.1 | adenylation-thiolation\_bidomain | BGC0000345 | NRP+Polyketide:Modular type I polyketide | 27.0 | 36.5 | 148.0 | 3.09e-36 |
| QEO75074.1 | condensation\_domain-containing\_protein | BGC0002079 | NRP:Cyclic depsipeptide | 30.0 | 35.9 | 150.0 | 3.17e-36 |
| ARS01470.1 | NcmB | BGC0001702 | NRP+Polyketide | 24.0 | 67.7 | 150.0 | 3.4e-36 |
| CEK23365.1 | conserved\_hypothetical\_protein | BGC0001716 | NRP | 29.0 | 35.8 | 150.0 | 3.56e-36 |
| EAW16180.1 | nonribosomal\_peptide\_synthase,\_putative | BGC0000293 | NRP | 28.0 | 34.9 | 151.0 | 3.62e-36 |
| BAX64246.1 | NRPS | BGC0001623 | NRP+Polyketide | 28.0 | 45.5 | 151.0 | 3.81e-36 |
| BS329\_14150 | non-ribosomal\_peptide\_synthetase | BGC0001462 | NRP:Glycopeptide | 29.0 | 37.0 | 150.0 | 4.49e-36 |
| BBE36469.1 | amino\_acid\_adenylation\_domain-containing\_protein | BGC0001922 | Polyketide | 28.0 | 37.0 | 145.0 | 5.54e-36 |
| AMQ36134.1 | PsyC | BGC0002617 | NRP | 26.0 | 49.1 | 149.0 | 6.42e-36 |
| ABP57745.1 | DepA | BGC0000993 | NRP:Cyclic depsipeptide+Polyketide:Modular type I polyketide | 28.0 | 41.1 | 150.0 | 6.89e-36 |
| QXF14600.1 | PydA | BGC0002239 | Polyketide+NRP | 23.0 | 71.9 | 150.0 | 7.3e-36 |
| AGC65514.1 | TtcB | BGC0001876 | NRP | 25.0 | 49.0 | 150.0 | 7.57e-36 |
| QSV12656.1 | AvmN | BGC0002456 | Polyketide+NRP | 24.0 | 57.9 | 149.0 | 7.87e-36 |
| AGZ20183.1 | non-ribosomal\_peptide\_synthetase | BGC0002618 | Terpene | 25.0 | 46.3 | 150.0 | 9.24e-36 |
| CAP93139.1 | cyclic\_hydrophobic\_tetrapeptide | BGC0000357 | NRP:Cyclic depsipeptide | 27.0 | 45.8 | 150.0 | 1.01e-35 |
| BAH23995.1 | nonribosomal\_peptide\_synthetase | BGC0000356 | NRP+Alkaloid | 26.0 | 46.5 | 149.0 | 1.04e-35 |
| QRD93053.1 | putative\_nonribosomal\_peptide\_synthase | BGC0002160 | NRP | 27.0 | 44.2 | 150.0 | 1.04e-35 |
| AQM58286.1 | non-ribosomal\_peptide\_synthase | BGC0001816 | NRP+Polyketide | 25.0 | 39.8 | 149.0 | 1.11e-35 |
| CBL93723.1 | NRPS\_didomain\_A-PCP | BGC0000360 | NRP | 27.0 | 36.1 | 145.0 | 1.16e-35 |
| WP\_004571777.1 | non-ribosomal\_peptide\_synthetase | BGC0001760 | NRP | 28.0 | 39.1 | 149.0 | 1.17e-35 |
| QDG75033.1 | mixed\_type\_I\_polyketide\_synthase\_-\_peptide\_synthetase | BGC0002068 | NRP+Polyketide | 26.0 | 45.2 | 149.0 | 1.32e-35 |
| ABV56582.1 | KtzB | BGC0000378 | NRP | 29.0 | 30.7 | 144.0 | 1.35e-35 |
| ADQ55476.1 | NRPS | BGC0000350 | NRP:Beta-lactam | 23.0 | 46.1 | 149.0 | 1.61e-35 |
| CCE88377.1 | non-ribosomal\_peptide\_synthetase/polyketide\_synthase | BGC0001034 | NRP+Polyketide:Modular type I polyketide | 30.0 | 34.0 | 149.0 | 1.63e-35 |
| AGC65513.1 | TtcA | BGC0001876 | NRP | 26.0 | 46.5 | 149.0 | 1.76e-35 |
| ADY76684.1 | non-ribosomal\_peptide\_synthetase | BGC0000950 | NRP:Uridylpeptide+Other:Nucleoside | 26.0 | 41.5 | 143.0 | 2.14e-35 |
| RGP42808.1 | non-ribosomal\_peptide\_synthetase | BGC0002696 | NRP | 27.0 | 52.1 | 149.0 | 2.15e-35 |
| CBJ89761.1 | Non-ribosomal\_peptide\_synthase\_involved\_in\_xenocoumacin\_synthesis | BGC0001054 | NRP+Polyketide:Modular type I polyketide | 25.0 | 46.9 | 147.0 | 2.33e-35 |
| AAS92545.1 | SirP | BGC0001044 | NRP | 25.0 | 48.1 | 148.0 | 3.05e-35 |
| EFY99276.1 | non-ribosomal\_peptide\_synthetase | BGC0002710 | NRP | 27.0 | 46.3 | 147.0 | 3.63e-35 |
| ABY83142.1 | Azi3 | BGC0000960 | NRP+Polyketide | 29.0 | 37.2 | 147.0 | 3.98e-35 |
| WP\_052165466.1 | non-ribosomal\_peptide\_synthetase | BGC0001327 | NRP:Cyclic depsipeptide+Polyketide:Modular type I polyketide | 31.0 | 29.0 | 147.0 | 4.26e-35 |
| AIE77058.1 | peptide\_synthetase\_module\_3 | BGC0000418 | NRP | 28.0 | 35.6 | 146.0 | 4.85e-35 |
| QXJ21811.1 | amino\_acid\_adenylation\_domain-containing\_protein | BGC0002370 | NRP | 29.0 | 40.2 | 142.0 | 4.89e-35 |
| CRG85572.1 | nonribosomal\_peptide\_synthase,\_putative | BGC0001402 | NRP | 22.0 | 61.8 | 147.0 | 6.64e-35 |
| AVV61987.1 | putative\_non-ribosomal\_peptide\_synthetase | BGC0001477 | NRP+Polyketide:Modular type I polyketide | 27.0 | 36.9 | 145.0 | 8.14e-35 |
| CBG67531.1 | NRPS\_multienzyme\_(AMP-binding\_and\_pcp) | BGC0002367 | NRP | 31.0 | 39.1 | 144.0 | 8.56e-35 |
| CCA89326.1 | mixed\_trans-AT\_type\_I\_polyketide\_synthase/nonribosomal\_peptide\_synthetase | BGC0001111 | NRP+Polyketide:Trans-AT type I polyketide | 27.0 | 35.5 | 147.0 | 9.31e-35 |
| ABD14712.1 | cesB | BGC0000320 | NRP:Cyclic depsipeptide | 26.0 | 35.6 | 146.0 | 1.28e-34 |
| ACG60776.1 | NRPS(AL/ACP/C/A/PCP/C/A) | BGC0001058 | NRP:Glycopeptide+Polyketide:Modular type I polyketide+Saccharide:Hybrid/tailoring saccharide | 29.0 | 34.9 | 146.0 | 1.28e-34 |
| ORC16618.1 | hypothetical\_protein | BGC0001341 | NRP | 27.0 | 36.1 | 146.0 | 1.36e-34 |
| EWM63005.1 | linear\_gramicidin\_synthetase\_LgrC | BGC0001328 | NRP:Cyclic depsipeptide+Polyketide:Modular type I polyketide | 31.0 | 29.0 | 145.0 | 1.38e-34 |
| EJK79842.1 | amino\_acid\_adenylation\_enzyme/thioester\_reductase\_family\_protein | BGC0000436 | NRP | 27.0 | 34.7 | 145.0 | 1.59e-34 |
| AFK57220.1 | DidI | BGC0000985 | Polyketide+NRP:Cyclic depsipeptide | 29.0 | 36.2 | 144.0 | 1.71e-34 |
| CBF87869.1 | nonribosomal\_peptide\_synthase,\_putative\_(Eurofung) | BGC0001699 | NRP | 25.0 | 46.5 | 145.0 | 1.97e-34 |
| AQM58287.1 | non-ribosomal\_peptide\_synthase | BGC0001816 | NRP+Polyketide | 26.0 | 46.8 | 145.0 | 2.11e-34 |
| CAD15508.1 | polyketide\_synthase/non-ribosomal\_peptide\_synthetase | BGC0001014 | NRP:NRP siderophore+Polyketide:Modular type I polyketide+Polyketide:Iterative type I polyketide | 29.0 | 35.3 | 145.0 | 2.45e-34 |
| CAD70194.1 | non-ribosomal\_peptide\_synthetase | BGC0001047 | NRP+Polyketide | 26.0 | 37.5 | 144.0 | 3.34e-34 |
| QCS37521.1 | pyiS | BGC0001881 | NRP+Polyketide:Iterative type I polyketide | 24.0 | 72.7 | 144.0 | 4.17e-34 |
| AFN69082.1 | nonribosomal\_peptide\_synthetase\_6 | BGC0001249 | NRP | 26.0 | 46.1 | 144.0 | 5.43e-34 |
| BAO84861.1 | putative\_non-ribosomal\_peptide\_synthetase | BGC0000414 | NRP | 26.0 | 40.7 | 143.0 | 5.97e-34 |
| ADM34138.1 | non-ribosomal\_peptide\_synthetase | BGC0001084 | NRP+Terpene+Alkaloid | 24.0 | 62.8 | 144.0 | 6.06e-34 |
| AXM42949.1 | hybrid\_type\_1\_PKS/NRPS | BGC0001941 | NRP+Polyketide | 27.0 | 34.6 | 144.0 | 6.8e-34 |
| ESU15173.1 | hypothetical\_protein | BGC0002186 | NRP+Polyketide | 25.0 | 45.7 | 143.0 | 9.51e-34 |
| KMO93436.1 | NRPS/PKS | BGC0002095 | NRP | 27.0 | 39.5 | 138.0 | 1.14e-33 |
| CAA46313.1 | peptide\_synthetase | BGC0000406 | NRP | 26.0 | 49.1 | 139.0 | 1.24e-33 |
| ctg1\_orf4 |  | BGC0001329 | Polyketide+NRP:Cyclic depsipeptide | 34.0 | 22.7 | 142.0 | 1.24e-33 |
| AUS29489.1 | non-ribosomal\_peptide\_synthetase | BGC0002606 | NRP+Polyketide | 27.0 | 46.2 | 142.0 | 1.26e-33 |
| AKP45396.1 | CysH | BGC0001413 | NRP | 28.0 | 32.9 | 141.0 | 1.51e-33 |
| CCA53799.1 | iron\_aquisition\_yersiniabactin\_synthesis\_enzyme | BGC0001801 | NRP | 33.0 | 27.3 | 141.0 | 2.5e-33 |
| DAC80529.1 | malonyl\_CoA-acyl\_carrier\_protein\_transacylase | BGC0001878 | NRP+Polyketide | 27.0 | 50.2 | 142.0 | 2.59e-33 |
| ABC39418.1 | dihydroaeruginoic\_acid\_synthetase | BGC0000964 | NRP:Cyclic depsipeptide+Polyketide:Trans-AT type I polyketide | 28.0 | 40.9 | 141.0 | 2.69e-33 |
| QYA95657.1 | amino\_acid\_adenylation\_domain-containing\_protein | BGC0002676 | NRP | 28.0 | 40.3 | 137.0 | 2.94e-33 |
| BBQ09587.1 | PKS-NRPS\_hybrid | BGC0002261 | Polyketide | 23.0 | 66.3 | 141.0 | 3.59e-33 |
| AGD80616.1 | amino\_acid\_adenylation\_domain-containing\_protein | BGC0000394 | NRP | 26.0 | 42.1 | 140.0 | 3.66e-33 |
| EJP62835.1 | nonribosomal\_peptide\_synthase,\_putative | BGC0002203 | NRP+Polyketide+Other | 26.0 | 37.9 | 141.0 | 3.72e-33 |
| WP\_030498977.1 | hypothetical\_protein | BGC0001327 | NRP:Cyclic depsipeptide+Polyketide:Modular type I polyketide | 24.0 | 61.5 | 140.0 | 4.4e-33 |
| ADZ45339.1 | non-ribosomal\_peptide\_synthetase | BGC0001020 | NRP+Polyketide | 28.0 | 29.3 | 136.0 | 5.27e-33 |
| CAJ76292.1 | putative\_non-ribosomal\_peptide\_synthase | BGC0000972 | NRP+Polyketide:Modular type I polyketide+Polyketide:Trans-AT type I polyketide | 25.0 | 39.6 | 140.0 | 5.84e-33 |
| ctg1\_orf00001 |  | BGC0000901 | Other | 26.0 | 44.8 | 140.0 | 5.89e-33 |
| EPH46610.1 | putative\_Dimodular\_nonribosomal\_peptide\_synthase | BGC0001519 | NRP+Polyketide | 28.0 | 31.5 | 139.0 | 6.81e-33 |
| ATY69590.1 | adenylation\_protein | BGC0001823 | NRP+Polyketide | 27.0 | 39.7 | 135.0 | 7.96e-33 |
| ESU05145.1 | hypothetical\_protein | BGC0002178 | NRP | 27.0 | 33.8 | 140.0 | 8.26e-33 |
| QCX41944.1 | Amc7 | BGC0001957 | Polyketide | 28.0 | 39.7 | 138.0 | 1.08e-32 |
| QHW08555.1 | non-ribosomal\_peptide\_synthetase | BGC0002054 | Polyketide+NRP+Saccharide | 28.0 | 39.7 | 138.0 | 1.08e-32 |
| CAN89633.1 | putative\_hybrid\_non-ribosomal\_peptide\_synthetase/polyketide\_synthase | BGC0001070 | NRP+Polyketide:Modular type I polyketide+Polyketide:Trans-AT type I polyketide | 27.0 | 36.4 | 139.0 | 1.29e-32 |
| AGZ15455.1 | non-ribosomal\_peptide\_synthetase | BGC0001036 | NRP+Polyketide | 27.0 | 34.7 | 135.0 | 1.46e-32 |
| BAE60013.1 |  | BGC0001518 | Terpene | 25.0 | 45.1 | 139.0 | 1.52e-32 |
| AYA22335.1 | KerB | BGC0001955 | NRP | 30.0 | 35.8 | 138.0 | 1.53e-32 |
| QNL14923.1 | AptC | BGC0002512 | NRP | 24.0 | 53.7 | 139.0 | 1.64e-32 |
| AAF01762.1 | AM-toxin\_synthetase | BGC0001261 | NRP | 27.0 | 43.0 | 139.0 | 1.85e-32 |
| CUX96957.1 | TmcJ | BGC0001829 | NRP+Polyketide | 27.0 | 36.8 | 136.0 | 2.17e-32 |
| ADY76667.1 | non-ribosomal\_peptide\_synthetase | BGC0000950 | NRP:Uridylpeptide+Other:Nucleoside | 26.0 | 36.1 | 137.0 | 2.36e-32 |
| QKW60392.1 | amino\_acid\_adenylation\_domain-containing\_protein | BGC0002288 | NRP | 26.0 | 44.8 | 137.0 | 2.77e-32 |
| ESU17758.1 | hypothetical\_protein | BGC0002172 | NRP | 28.0 | 37.0 | 137.0 | 2.8e-32 |
| AGC65517.1 | NRPS | BGC0001050 | NRP:Lipopeptide+Polyketide:Trans-AT type I polyketide | 28.0 | 37.3 | 137.0 | 3.06e-32 |
| AAD24881.1 | putative\_acyl-CoA\_synthetase | BGC0000127 | Polyketide | 25.0 | 38.3 | 133.0 | 3.2e-32 |
| UMP03490.1 | NmvB | BGC0002649 | NRP+Polyketide | 27.0 | 38.4 | 137.0 | 3.27e-32 |
| UOH28374.1 | AceN | BGC0002149 | NRP+Terpene | 25.0 | 46.8 | 137.0 | 3.32e-32 |
| AAR12528.1 | non-ribosomal\_peptide\_synthetase | BGC0002468 | NRP | 27.0 | 35.9 | 137.0 | 3.45e-32 |
| RLV71192.1 | non-ribosomal\_peptide\_synthetase | BGC0001846 | NRP+Saccharide:Hybrid/tailoring saccharide | 23.0 | 68.9 | 138.0 | 3.71e-32 |
| AAG02359.1 | peptide\_synthetase\_NRPS5-4-3 | BGC0000963 | NRP:Glycopeptide+Polyketide:Modular type I polyketide+Saccharide:Hybrid/tailoring saccharide | 30.0 | 29.7 | 138.0 | 3.72e-32 |
| QTT72097.1 | amino\_acid\_adenylation\_domain-containing\_protein | BGC0002350 | NRP+Polyketide+Saccharide | 30.0 | 29.7 | 138.0 | 3.73e-32 |
| CAF05647.1 | TubB\_protein | BGC0001053 | NRP+Polyketide | 28.0 | 34.1 | 137.0 | 3.81e-32 |
| WP\_024483797.1 | non-ribosomal\_peptide\_synthetase | BGC0002002 | NRP | 27.0 | 37.7 | 134.0 | 3.82e-32 |
| BAL15726.1 | NRPS | BGC0000432 | NRP | 26.0 | 47.7 | 135.0 | 3.94e-32 |
| EHA55860.1 | polyketide\_synthase/peptide\_synthetase | BGC0002235 | Polyketide+NRP | 24.0 | 73.4 | 138.0 | 4.1e-32 |
| ADN26251.1 | ATP-dependent\_adenylase | BGC0000951 | NRP | 26.0 | 39.3 | 133.0 | 4.65e-32 |
| TXD00259.1 | amino\_acid\_adenylation\_domain-containing\_protein | BGC0001877 | Polyketide | 26.0 | 33.3 | 137.0 | 5.65e-32 |
| AGK15447.1 | Non-ribosomal\_peptide\_synthetase,\_with\_condensation,\_AMP\_binding\_and\_thioesterase\_modules | BGC0002529 | NRP | 24.0 | 37.0 | 137.0 | 6.08e-32 |
| AFR69331.1 | nonribosomal\_peptide\_synthetase\_SpiA | BGC0001045 | NRP:Cyclic depsipeptide+Polyketide:Modular type I polyketide | 27.0 | 40.3 | 137.0 | 6.9e-32 |
| ABC34305.1 | peptide\_synthetase,\_putative | BGC0000961 | NRP+Polyketide | 26.0 | 45.0 | 136.0 | 1.08e-31 |
| QJX57338.1 | ChaA | BGC0002538 | Polyketide | 24.0 | 65.3 | 136.0 | 1.2e-31 |
| AAC83657.1 | pyochelin\_synthetase | BGC0000412 | NRP | 31.0 | 24.8 | 136.0 | 1.24e-31 |
| EAL92291.2 | nonribosomal\_peptide\_synthtease | BGC0000372 | NRP | 25.0 | 42.2 | 136.0 | 1.35e-31 |
| BAE56606.1 |  | BGC0001123 | NRP | 25.0 | 46.3 | 135.0 | 1.53e-31 |
| EAU29302.1 | hypothetical\_protein | BGC0002272 | NRP | 25.0 | 34.5 | 135.0 | 1.8e-31 |
| ACY13414.1 | amino\_acid\_adenylation\_domain\_protein | BGC0001367 | NRP+Polyketide | 29.0 | 34.5 | 135.0 | 2.08e-31 |
| WP\_051803379.1 | D-alanine--poly(phosphoribitol)\_ligase | BGC0002381 | Alkaloid+Polyketide | 26.0 | 40.0 | 131.0 | 2.27e-31 |
| ARB50206.1 | lysergyl\_peptide\_synthetase\_2 | BGC0001573 | Alkaloid | 25.0 | 49.7 | 135.0 | 2.3e-31 |
| QBL56187.1 | long-chain-fatty-acid-CoA\_ligase | BGC0002376 | Polyketide | 27.0 | 39.1 | 131.0 | 2.34e-31 |
| EED49862.1 | hybrid\_PKS/NRPS\_enzyme,\_putative | BGC0001445 | NRP+Polyketide:Iterative type I polyketide | 24.0 | 65.5 | 135.0 | 2.7e-31 |
| CAJ34370.1 | NRPS\_protein | BGC0000445 | NRP:Cyclic depsipeptide | 28.0 | 29.8 | 133.0 | 2.84e-31 |
| CBL93716.1 | NRPS\_A-domain | BGC0000360 | NRP | 26.0 | 29.2 | 130.0 | 3.17e-31 |
| BBA21068.1 | putative\_non-ribosomal\_peptide\_synthetase | BGC0001740 | NRP+Polyketide | 27.0 | 45.3 | 134.0 | 4.91e-31 |
| AKJ15896.1 | non\_ribosomal\_peptide\_synthetase | BGC0002735 | Polyketide+NRP | 31.0 | 21.4 | 133.0 | 5.28e-31 |
| CCM44334.1 | Thioester\_reductase | BGC0001056 | NRP+Polyketide:Modular type I polyketide+Polyketide:PUFA synthase or related polyketide | 32.0 | 21.0 | 127.0 | 7.04e-31 |
| UKO95761.1 | amino\_acid\_adenylation\_domain-containing\_protein | BGC0002632 | NRP | 24.0 | 41.7 | 129.0 | 1e-30 |
| AEH42484.1 | adenylation\_for\_L-proline | BGC0000032 | Polyketide | 24.0 | 41.4 | 129.0 | 1.03e-30 |
| AET79177.1 | lysergyl\_peptide\_synthetase\_subunit\_3 | BGC0001241 | Terpene | 33.0 | 25.3 | 133.0 | 1.07e-30 |
| CCE30237.1 | related\_to\_AM-toxin\_synthetase\_(AMT) | BGC0002232 | Alkaloid | 33.0 | 25.3 | 133.0 | 1.07e-30 |
| KAF7597139.1 | hypothetical\_protein | BGC0002264 | NRP | 26.0 | 47.0 | 132.0 | 1.15e-30 |
| WP\_013663194.1 | amino\_acid\_adenylation\_domain-containing\_protein | BGC0001465 | Other | 24.0 | 39.0 | 129.0 | 1.24e-30 |
| EAW09117.1 | hybrid\_NRPS/PKS\_enzyme,\_putative | BGC0000983 | NRP+Polyketide:Iterative type I polyketide | 23.0 | 65.8 | 133.0 | 1.37e-30 |
| AGK13426.1 | enterochelin\_sythetase\_component\_F\_(Serine-activating\_enzyme)\_(Seryl-AMP\_ligase) | BGC0002528 | NRP | 28.0 | 33.3 | 132.0 | 1.53e-30 |
| ADL64235.1 | aureusimine\_non-ribosomal\_peptide\_synthetase | BGC0000308 | NRP | 23.0 | 58.3 | 132.0 | 1.58e-30 |
| EAL89046.1 | nonribosomal\_peptide\_synthetase | BGC0000355 | NRP | 27.0 | 38.9 | 131.0 | 2.34e-30 |
| ALK21569.1 | non-ribosomal\_peptide\_synthetase | BGC0002678 | NRP | 26.0 | 33.6 | 131.0 | 2.84e-30 |
| CAE52342.1 | non-ribosomal\_peptide\_synthase | BGC0001088 | NRP+Polyketide | 31.0 | 24.6 | 130.0 | 3.09e-30 |
| QXJ21809.1 | amino\_acid\_adenylation\_domain-containing\_protein | BGC0002370 | NRP | 32.0 | 25.9 | 131.0 | 4.84e-30 |
| EPH46593.1 | putative\_Bacitracin\_synthase\_3 | BGC0001519 | NRP+Polyketide | 27.0 | 34.6 | 127.0 | 5.07e-30 |
| OAL11435.1 | non-ribosomal\_peptide\_synthetase | BGC0001570 | NRP | 27.0 | 40.1 | 130.0 | 6.7e-30 |
| BAW27672.1 | NRPS(A-T-C) | BGC0001764 | NRP | 25.0 | 46.8 | 129.0 | 7.99e-30 |
| QRD90553.1 | non-ribosomal\_peptide\_synthetase\_module | BGC0002157 | NRP+Alkaloid | 26.0 | 46.1 | 130.0 | 1.08e-29 |
| QTX15956.1 | nonribosomal\_peptide\_synthase | BGC0002598 | Polyketide | 24.0 | 42.3 | 129.0 | 1.26e-29 |
| AUS29499.1 | non-ribosomal\_peptide\_synthetase | BGC0002607 | NRP+Polyketide | 24.0 | 42.3 | 129.0 | 1.26e-29 |
| XP\_020057667.1 | uncharacterized\_protein | BGC0001718 | NRP | 27.0 | 36.2 | 129.0 | 1.29e-29 |
| QUS58936.1 | non-ribosomal\_peptide\_synthetase | BGC0002123 | NRP+Polyketide | 25.0 | 45.4 | 129.0 | 1.46e-29 |
| XP\_011325838.1 | hypothetical\_protein | BGC0001545 | NRP | 24.0 | 52.1 | 129.0 | 1.8e-29 |
| ADH04679.1 | non-ribosomal\_peptide\_synthetase | BGC0001344 | NRP+Polyketide | 28.0 | 35.1 | 129.0 | 1.85e-29 |
| ACR78148.1 | BSLS | BGC0000312 | NRP | 26.0 | 34.5 | 129.0 | 2.54e-29 |
| AKD43754.1 | HerJ | BGC0001349 | NRP+Polyketide | 25.0 | 41.2 | 125.0 | 2.95e-29 |
| MBN3579112.1 | amino\_acid\_adenylation\_domain-containing\_protein | BGC0002613 | NRP+Polyketide | 27.0 | 33.4 | 128.0 | 3.84e-29 |
| AXG22420.1 | proline\_adenyltransferase | BGC0002024 | Polyketide | 28.0 | 32.0 | 124.0 | 4.63e-29 |
| ATG32071.1 | proline\_specific\_adenylation\_domain-containing\_protein | BGC0001750 | NRP+Polyketide | 25.0 | 39.2 | 124.0 | 5.11e-29 |
| BBU42014.1 | e-poly-L-lysine\_synthetase | BGC0002174 | NRP | 27.0 | 37.5 | 127.0 | 5.14e-29 |
| ABA59547.1 | NRPS | BGC0000453 | NRP:Cyclic depsipeptide | 27.0 | 33.9 | 127.0 | 5.81e-29 |
| WP\_078857609.1 | non-ribosomal\_peptide\_synthetase | BGC0001368 | NRP | 26.0 | 41.5 | 125.0 | 5.86e-29 |
| AQM58288.1 | non-ribosomal\_peptide\_synthase | BGC0001816 | NRP+Polyketide | 25.0 | 46.7 | 127.0 | 5.88e-29 |
| ADN26252.1 | peptide\_synthetase | BGC0000951 | NRP | 25.0 | 37.5 | 126.0 | 6.97e-29 |
| MCC5026027.1 | amino\_acid\_adenylation\_domain-containing\_protein | BGC0002118 | NRP+Polyketide | 31.0 | 26.6 | 127.0 | 8.23e-29 |
| MCC5025983.1 | amino\_acid\_adenylation\_domain-containing\_protein | BGC0002119 | NRP+Polyketide | 31.0 | 26.6 | 127.0 | 9.2e-29 |
| ADB02873.1 | AzicV | BGC0000202 | Polyketide | 26.0 | 31.3 | 122.0 | 1.84e-28 |
| ADH04678.1 | non-ribosomal\_peptide\_synthetase | BGC0001344 | NRP+Polyketide | 27.0 | 34.1 | 124.0 | 3.82e-28 |
| AGC65515.1 | TtcC | BGC0001876 | NRP | 26.0 | 36.6 | 124.0 | 5.47e-28 |
| QPP19367.1 | PenP | BGC0002501 | Alkaloid | 27.0 | 35.6 | 124.0 | 6.71e-28 |
| AAN85530.1 | nonribosomal\_peptide\_synthetase,\_discrete\_adenylation\_domain | BGC0001101 | NRP+Polyketide:Modular type I polyketide+Polyketide:Trans-AT type I polyketide | 28.0 | 32.1 | 120.0 | 8.05e-28 |
| AYJ71713.1 | non-ribosomal\_peptide\_synthetase | BGC0001942 | NRP+Polyketide | 24.0 | 46.8 | 122.0 | 1.47e-27 |
| ARB50207.1 | lysergyl\_peptide\_synthetase\_21 | BGC0001573 | Alkaloid | 25.0 | 46.8 | 122.0 | 1.95e-27 |
| ALA09365.1 | AMP-dependent\_synthetase\_and\_ligase | BGC0001303 | Polyketide | 24.0 | 41.2 | 119.0 | 1.98e-27 |
| ABP55217.1 | AMP-dependent\_synthetase\_and\_ligase | BGC0000142 | Polyketide | 28.0 | 30.5 | 119.0 | 2.53e-27 |
| ctg1\_orf13 |  | BGC0001329 | Polyketide+NRP:Cyclic depsipeptide | 31.0 | 23.1 | 121.0 | 2.87e-27 |
| RIH60556.1 | hypothetical\_protein | BGC0002652 | Other+Saccharide | 30.0 | 20.6 | 114.0 | 3.22e-27 |
| CBF77087.1 | conserved\_hypothetical\_protein | BGC0001679 | NRP | 27.0 | 34.5 | 121.0 | 3.54e-27 |
| ABC34483.1 | nonribosomal\_peptide\_synthetase,\_putative | BGC0000961 | NRP+Polyketide | 23.0 | 61.6 | 121.0 | 3.6e-27 |
| ORC16617.1 | hypothetical\_protein | BGC0001341 | NRP | 27.0 | 35.9 | 121.0 | 4.12e-27 |
| QCY50741.1 | non-ribosomal\_peptide\_synthetase | BGC0002287 | NRP | 23.0 | 34.2 | 120.0 | 4.24e-27 |
| QKF54440.2 | P450+A+T | BGC0002581 | NRP | 25.0 | 39.1 | 120.0 | 4.69e-27 |
| AKQ52531.1 | nonribosomal\_peptide\_synthetase | BGC0002533 | NRP+Polyketide | 25.0 | 34.4 | 121.0 | 4.9e-27 |
| UEF20583.1 | nonribosomal\_peptide\_synthetase | BGC0002360 | NRP | 25.0 | 39.1 | 120.0 | 6.16e-27 |
| SAI82901.1 | HrnJ;\_Putative\_AMP-dependent\_acyl-CoA\_synthetase/ligase;\_AMP-binding\_enzyme;\_Pfam00501 | BGC0002101 | Polyketide | 24.0 | 41.2 | 117.0 | 7.1e-27 |
| AET79184.1 | lysergyl\_peptide\_synthetase\_subunit\_1 | BGC0001241 | Terpene | 24.0 | 44.6 | 120.0 | 7.53e-27 |
| CCE30225.1 | non-ribosomal\_peptide\_synthetase | BGC0002232 | Alkaloid | 24.0 | 44.6 | 120.0 | 7.53e-27 |
| AAQ90177.1 | putative\_acyl-CoA\_synthetase | BGC0000128 | Polyketide | 26.0 | 29.2 | 117.0 | 8.95e-27 |
| AKJ75110.1 | Bmp4 | BGC0001464 | Other | 24.0 | 38.9 | 117.0 | 9.04e-27 |
| AFJ20782.1 | nonribosomal\_peptide\_synthetase | BGC0002300 | NRP | 28.0 | 34.7 | 120.0 | 1.03e-26 |
| BAP34707.1 | AMP-dependent\_synthetase\_and\_ligase | BGC0000078 | Polyketide | 27.0 | 29.4 | 117.0 | 1.26e-26 |
| ABB69081.1 | putative\_L-prolyl-AMP\_ligase | BGC0000260 | Polyketide | 25.0 | 38.4 | 117.0 | 1.37e-26 |
| CAJ87590.1 | putative\_peptide\_synthase | BGC0001055 | NRP+Polyketide | 29.0 | 30.4 | 119.0 | 1.47e-26 |
| CCE28989.1 | non-ribosomal\_peptide\_synthetase | BGC0001365 | NRP | 25.0 | 35.9 | 119.0 | 1.47e-26 |
| QHZ99336.1 | proline\_adenyltransferase | BGC0001875 | Polyketide | 27.0 | 30.3 | 116.0 | 1.61e-26 |
| BAP16684.1 | nonribosomal\_peptide\_synthetase | BGC0000376 | NRP | 29.0 | 35.8 | 117.0 | 1.96e-26 |
| WP\_002308266.1 | NRPS,\_A-domain\_Leucine | BGC0002058 | Polyketide+NRP | 23.0 | 34.2 | 118.0 | 2.18e-26 |
| AET79183.1 | lysergyl\_peptide\_synthetase\_subunit\_1 | BGC0001241 | Terpene | 25.0 | 44.4 | 119.0 | 2.21e-26 |
| CCE30226.1 | non-ribosomal\_peptide\_synthetase | BGC0002232 | Alkaloid | 25.0 | 44.4 | 119.0 | 2.21e-26 |
| CDG76957.1 | non-ribosomal\_peptide\_synthetase | BGC0000446 | NRP:Pyrrolobenzodiazepine | 24.0 | 41.7 | 115.0 | 2.34e-26 |
| AHF22853.1 | MarM | BGC0000091 | Polyketide | 25.0 | 40.8 | 115.0 | 2.96e-26 |
| BBB04327.1 | nonribosomal\_peptide\_synthetase | BGC0001717 | NRP | 26.0 | 36.3 | 118.0 | 3.27e-26 |
| RLV71193.1 | non-ribosomal\_peptide\_synthetase | BGC0001846 | NRP+Saccharide:Hybrid/tailoring saccharide | 27.0 | 35.6 | 118.0 | 3.74e-26 |
| ACO94489.1 | putative\_AMP-dependent\_acyl-CoA\_synthetase/ligase | BGC0000097 | Polyketide:Modular type I polyketide | 25.0 | 41.2 | 115.0 | 3.99e-26 |
| AUS29494.1 | non-ribosomal\_peptide\_synthetase | BGC0001030 | NRP+Polyketide | 23.0 | 46.5 | 117.0 | 7.23e-26 |
| CBL93730.1 | NRPS | BGC0000360 | NRP | 24.0 | 45.2 | 117.0 | 7.41e-26 |
| KIA75688.1 | nonribosomal\_peptide\_synthase | BGC0002242 | NRP | 24.0 | 38.9 | 117.0 | 8.65e-26 |
| ATJ04409.1 | NRPS | BGC0001637 | NRP | 25.0 | 41.6 | 114.0 | 9.92e-26 |
| WP\_064118616.1 | non-ribosomal\_peptide\_synthetase | BGC0002075 | Alkaloid+NRP:Lipopeptide | 26.0 | 35.2 | 116.0 | 1.35e-25 |
| CAH55654.1 | putative\_L-prolyl-AMP\_ligase | BGC0000259 | Polyketide | 26.0 | 28.9 | 113.0 | 1.51e-25 |
| ANY57892.1 | PenN | BGC0001372 | Terpene | 26.0 | 33.9 | 116.0 | 1.83e-25 |
| AAQ93484.1 | CmaA | BGC0000328 | NRP | 24.0 | 43.9 | 114.0 | 1.98e-25 |
| ACO94461.1 | putative\_AMP-dependent\_acyl-CoA\_synthetase/ligase | BGC0000029 | Polyketide:Modular type I polyketide | 24.0 | 41.3 | 113.0 | 2.23e-25 |
| BAG84247.1 | putative\_L-prolyl-AMP\_ligase | BGC0000257 | Polyketide | 25.0 | 34.5 | 114.0 | 2.35e-25 |
| ADN26257.1 | ATP-dependent\_adenylase | BGC0000951 | NRP | 27.0 | 33.8 | 112.0 | 3.17e-25 |
| UHJ79942.1 | non-ribosomal\_peptide\_synthetase | BGC0002654 | NRP | 30.0 | 29.4 | 114.0 | 3.57e-25 |
| AAC83656.1 | dihydroaeruginoic\_acid\_synthetase | BGC0000412 | NRP | 26.0 | 33.5 | 114.0 | 4.26e-25 |
| CCB53264.1 | non-ribosomal\_peptide\_synthetase | BGC0001393 | NRP | 26.0 | 28.4 | 114.0 | 5.16e-25 |
| AFL68053.1 | amino\_acid\_adenylation\_enzyme/thioester\_reductase\_family\_protein | BGC0001524 | NRP+Polyketide | 24.0 | 36.9 | 114.0 | 8.67e-25 |
| AGA37269.1 | NRPS | BGC0000819 | NRP+Alkaloid | 24.0 | 44.0 | 114.0 | 8.93e-25 |
| ACN69986.1 | proline\_adenyltransferase | BGC0000079 | Polyketide | 27.0 | 31.9 | 110.0 | 1.21e-24 |
| BAO84864.1 | putative\_non-ribosomal\_peptide\_synthetase | BGC0000414 | NRP | 31.0 | 22.6 | 112.0 | 1.41e-24 |
| ESP90847.1 | amino\_acid\_adenylation\_domain\_protein | BGC0000891 | Other:Aminocoumarin | 23.0 | 40.7 | 110.0 | 1.47e-24 |
| CAH55637.1 | putative\_L-prolyl-AMP\_ligase | BGC0000258 | Polyketide | 25.0 | 28.9 | 110.0 | 1.54e-24 |
| ATY69568.1 | adenylation\_protein | BGC0001611 | NRP+Polyketide | 26.0 | 37.5 | 110.0 | 1.78e-24 |
| AAO56106.1 | yersiniabactin\_non-ribosomal\_peptide\_synthetase | BGC0002570 | NRP+Polyketide | 28.0 | 27.0 | 112.0 | 1.91e-24 |
| QOE83923.1 | tyrocidine\_synthase\_3 | BGC0002051 | NRP | 27.0 | 39.3 | 112.0 | 2.32e-24 |
| EAU36105.1 | predicted\_protein | BGC0002273 | NRP | 24.0 | 46.9 | 112.0 | 2.67e-24 |
| sipL1 | AMP-dependent\_synthetase\_and\_ligase | BGC0001452 | Polyketide | 26.0 | 28.9 | 110.0 | 2.68e-24 |
| CAE52339.1 | Polyketide\_non-ribosomal\_peptide\_synthase | BGC0001088 | NRP+Polyketide | 25.0 | 49.0 | 112.0 | 3.06e-24 |
| CCP45167.1 | Peptide\_synthetase\_MbtF\_(peptide\_synthase) | BGC0001021 | NRP+Polyketide | 27.0 | 35.9 | 111.0 | 3.75e-24 |
| AWR88409.1 | putative\_AMP-dependent\_synthetase\_and\_ligase | BGC0001522 | Polyketide | 26.0 | 28.9 | 109.0 | 4.69e-24 |
| KIA75458.1 | hypothetical\_protein | BGC0002208 | NRP | 25.0 | 36.1 | 111.0 | 6.46e-24 |
| CAQ34914.1 | putative\_L-prolyl\_AMP-ligase | BGC0000986 | NRP+Polyketide | 24.0 | 39.5 | 108.0 | 7.51e-24 |
| CBW75455.1 | D-alanine-activating\_enzyme\_(EC\_6.3.2.-) | BGC0002048 | NRP:Cyclic depsipeptide | 26.0 | 22.8 | 108.0 | 9.31e-24 |
| QIH29226.1 | adenylation\_domain\_protein | BGC0002326 | NRP | 26.0 | 22.8 | 108.0 | 9.31e-24 |
| BAL15718.1 | NRPS | BGC0000432 | NRP | 27.0 | 29.1 | 107.0 | 1.31e-23 |
| AME30287.1 | L-proline\_adenyl\_transferase | BGC0001463 | Other | 24.0 | 33.1 | 106.0 | 2.15e-23 |
| DAD54576.1 | NRPS-like\_tryptophan\_epimerase | BGC0002256 | NRP+Other | 25.0 | 45.4 | 108.0 | 4.28e-23 |
| AXG47410.1 | non-ribosomal\_peptide\_synthetase | BGC0002715 | NRP+Polyketide | 26.0 | 34.2 | 108.0 | 4.89e-23 |
| ABA59548.1 | NRPS | BGC0000453 | NRP:Cyclic depsipeptide | 23.0 | 68.0 | 107.0 | 1.17e-22 |
| ABK36691.1 | dimodular\_nonribosomal\_peptide\_synthetase | BGC0001502 | NRP | 29.0 | 29.2 | 103.0 | 1.88e-22 |
| AET13879.1 | epichloenin\_A\_synthetase | BGC0001251 | NRP | 24.0 | 47.4 | 106.0 | 2.2e-22 |
| AXG46165.1 | non-ribosomal\_peptide\_synthetase | BGC0002713 | NRP | 21.0 | 45.8 | 105.0 | 2.46e-22 |
| QPB84034.1 | AMP-binding\_protein | BGC0002675 | Polyketide | 22.0 | 28.9 | 102.0 | 5.13e-22 |
| AEO57481.1 | PKS-NRPSs | BGC0001449 | NRP+Alkaloid+Polyketide:Iterative type I polyketide | 24.0 | 44.9 | 104.0 | 8.28e-22 |
| AET13875.1 | epichloenin\_A\_synthetase | BGC0001250 | NRP | 24.0 | 47.4 | 104.0 | 8.45e-22 |
| AHA34037.1 | Bmp4,\_prolyl\_adenylation\_domain | BGC0000890 | Other:Aminocoumarin | 22.0 | 32.6 | 101.0 | 1.03e-21 |
| AFK57215.1 | DidD | BGC0000985 | Polyketide+NRP:Cyclic depsipeptide | 29.0 | 24.5 | 103.0 | 1.08e-21 |
| CBJ90287.1 | peptide\_synthetase | BGC0000416 | NRP | 21.0 | 49.1 | 103.0 | 1.49e-21 |
| WP\_013184317.1 | non-ribosomal\_peptide\_synthetase | BGC0001692 | NRP | 21.0 | 49.1 | 103.0 | 1.49e-21 |
| PVC99845.1 | Adenylation-domain-containg\_protein | BGC0002100 | NRP+Other | 27.0 | 31.0 | 100.0 | 2.47e-21 |
| CBJ90289.1 | peptide\_synthetase | BGC0000416 | NRP | 22.0 | 45.7 | 102.0 | 2.79e-21 |
| WP\_013184319.1 | non-ribosomal\_peptide\_synthetase | BGC0001692 | NRP | 22.0 | 45.7 | 102.0 | 2.79e-21 |
| BAL15732.1 | NRPS | BGC0000432 | NRP | 28.0 | 25.4 | 100.0 | 3.14e-21 |
| AFP87519.1 | proline\_adenyltransferase | BGC0001159 | NRP+Polyketide:Modular type I polyketide | 23.0 | 41.3 | 100.0 | 3.82e-21 |
| BAV56002.1 | ATP-dependent\_ligase | BGC0001597 | Polyketide | 24.0 | 33.2 | 100.0 | 3.93e-21 |
| AGC24265.1 | prlK | BGC0001038 | NRP+Polyketide:Modular type I polyketide | 25.0 | 29.0 | 99.0 | 5.47e-21 |
| CBJ90288.1 | Peptide\_synthetase | BGC0000416 | NRP | 25.0 | 24.1 | 100.0 | 7.56e-21 |
| WP\_013184318.1 | non-ribosomal\_peptide\_synthetase | BGC0001692 | NRP | 25.0 | 24.1 | 100.0 | 7.56e-21 |
| CAP20364.1 | equibactin\_equibactin\_nonribosomal\_peptide\_synthase\_protein | BGC0000347 | NRP | 24.0 | 34.2 | 100.0 | 1.08e-20 |
| AAN65233.1 | acyl-CoA\_synthetase | BGC0000832 | Saccharide:Hybrid/tailoring saccharide+Other:Aminocoumarin | 24.0 | 30.1 | 98.0 | 1.61e-20 |
| AAG05690.1 | AmbE | BGC0000287 | NRP | 27.0 | 28.0 | 100.0 | 1.88e-20 |
| MBA0053730.1 | D-alanine--poly(phosphoribitol)\_ligase | BGC0002096 | Polyketide | 27.0 | 23.2 | 93.0 | 6.01e-19 |
| CCB53269.1 | AMP-binding\_enzyme | BGC0001393 | NRP | 22.0 | 31.9 | 93.0 | 7.03e-19 |
| AHA12086.1 | amino\_acid\_adenyltransferase | BGC0001172 | NRP+Polyketide:Modular type I polyketide | 24.0 | 30.1 | 92.0 | 7.83e-19 |
| AWS21276.1 | amino\_acid\_adenyltransferase | BGC0001934 | Polyketide | 27.0 | 23.3 | 92.0 | 1.08e-18 |
| AZY92000.1 | proline\_adenylation\_protein | BGC0002022 | Polyketide | 27.0 | 23.3 | 92.0 | 1.08e-18 |
| ABO15875.1 | amino\_acid\_adenyltransferase | BGC0000131 | Polyketide | 26.0 | 23.2 | 92.0 | 1.4e-18 |
| AAG29789.1 | acyl-CoA\_synthetase | BGC0000833 | Saccharide:Hybrid/tailoring saccharide+Other:Aminocoumarin | 24.0 | 30.3 | 91.0 | 2.65e-18 |
| ABO15844.1 | amino\_acid\_adenyltransferase | BGC0000130 | Polyketide | 25.0 | 23.3 | 91.0 | 2.9e-18 |
| ALD82525.1 | non-ribosomal\_peptide\_synthase | BGC0001212 | NRP+Polyketide | 28.0 | 24.0 | 92.0 | 3.8e-18 |
| ABK39646.1 | nonribosomal\_peptide\_synthetase | BGC0001502 | NRP | 25.0 | 34.7 | 92.0 | 4.13e-18 |
| OJJ96433.1 | hypothetical\_protein | BGC0002226 | NRP | 25.0 | 35.6 | 92.0 | 5.59e-18 |
| ABC34137.1 | peptide\_synthetase,\_putative | BGC0000961 | NRP+Polyketide | 26.0 | 32.0 | 86.0 | 1.66e-16 |
| QCQ67877.1 | hybrid\_peptide\_synthetase/polyketide\_synthase | BGC0002297 | NRP+Polyketide | 24.0 | 38.1 | 82.0 | 3.67e-15 |
| AAX98208.1 | amide\_synthetase | BGC0000052 | Polyketide | 25.0 | 29.8 | 79.0 | 1.3e-14 |
| AAF00957.1 | mcyG | BGC0001017 | NRP+Polyketide:Modular type I polyketide | 22.0 | 40.0 | 80.0 | 2.42e-14 |
| WP\_019634554.1 | AMP-binding\_protein | BGC0001443 | NRP+Polyketide | 24.0 | 29.4 | 78.0 | 3e-14 |
| AJV88383.1 | MfnK | BGC0001214 | NRP | 23.0 | 39.3 | 78.0 | 3.12e-14 |
| MAA\_10036 | nonribosomal\_peptide\_synthase\_GliP-like,\_putative | BGC0000337 | NRP | 26.0 | 22.2 | 79.0 | 4.02e-14 |
| AQH32483.1 | hybrid\_peptide\_synthetase/polyketide\_synthase | BGC0001667 | NRP+Polyketide | 23.0 | 37.6 | 79.0 | 5.44e-14 |
| KKZ73241.1 | fatty\_acid--CoA\_ligase | BGC0001778 | Other | 24.0 | 24.2 | 77.0 | 6.09e-14 |
| OAL11476.1 | non-ribosomal\_peptide\_synthetase/polyketide\_synthase | BGC0001570 | NRP | 29.0 | 27.2 | 76.0 | 2.76e-13 |
| AAO62585.1 | peptide\_sythetase\_polyketide\_synthase\_fusion\_protein | BGC0001016 | NRP+Polyketide | 22.0 | 39.7 | 75.0 | 6.15e-13 |
| AYM48693.1 | T12 | BGC0002364 | Polyketide | 26.0 | 25.5 | 73.0 | 1.01e-12 |
| WP\_240809958.1 | long-chain\_fatty\_acid--CoA\_ligase | BGC0002686 | NRP | 23.0 | 42.3 | 72.0 | 2.04e-12 |
| ATY72527.1 | AMP-ligase | BGC0001574 | NRP | 23.0 | 29.9 | 71.0 | 4.53e-12 |
| MBD2892711.1 | 3-methylmercaptopropionyl-CoA\_ligase | BGC0002718 | NRP | 23.0 | 36.0 | 71.0 | 4.72e-12 |
| ATP76242.1 | NdaC | BGC0001705 | NRP+Polyketide | 23.0 | 39.3 | 72.0 | 6.96e-12 |
| AZF85933.1 | long-chain-fatty-acid-CoA\_ligase | BGC0001963 | NRP+Polyketide | 23.0 | 39.8 | 70.0 | 1.01e-11 |
| CAE14766.1 |  | BGC0002020 | Saccharide+NRP | 26.0 | 22.8 | 69.0 | 1.97e-11 |
| CAD29795.1 | peptide\_synthetase | BGC0001015 | NRP+Polyketide | 21.0 | 39.0 | 69.0 | 3.51e-11 |
| ACC80687.1 | AMP-dependent\_synthetase\_and\_ligase | BGC0002711 | Polyketide | 21.0 | 33.1 | 66.0 | 2.75e-10 |
| AKD43499.1 | Acyl-CoA\_ligase | BGC0001409 | Polyketide | 24.0 | 30.1 | 65.0 | 3.49e-10 |
| AFY58527.1 | acyl-CoA\_synthetase\_(AMP-forming)/AMP-acid\_ligase\_II | BGC0002411 | NRP+Polyketide | 23.0 | 34.7 | 65.0 | 4.81e-10 |
| AQA28568.1 | acyl-CoA\_synthase | BGC0001663 | Polyketide | 23.0 | 33.5 | 65.0 | 5.61e-10 |
| BBA21082.1 | putative\_CoA\_ligase | BGC0001740 | NRP+Polyketide | 25.0 | 25.5 | 64.0 | 1.12e-09 |
| ctg3\_28 |  | BGC0001853 | NRP+Polyketide:Modular type I polyketide | 23.0 | 33.2 | 63.0 | 1.8e-09 |
| ATY12791.1 | long-chain\_fatty\_acid--CoA\_ligase | BGC0001504 | Polyketide | 22.0 | 42.2 | 63.0 | 1.93e-09 |
| AAO06916.1 | GdmAI | BGC0000066 | Polyketide | 23.0 | 37.7 | 62.0 | 4.77e-09 |
| ABB86408.1 | GelA | BGC0000067 | Polyketide | 23.0 | 37.7 | 62.0 | 4.77e-09 |
| AYU66239.1 | TjhA5 | BGC0002461 | Polyketide | 26.0 | 23.3 | 61.0 | 1.01e-08 |
| QIC03949.1 | CyaA | BGC0002347 | Alkaloid | 24.0 | 33.9 | 60.0 | 1.32e-08 |
| AAY28225.1 | HbmAI | BGC0000074 | Polyketide | 23.0 | 37.7 | 61.0 | 1.83e-08 |
| QOE83926.1 | linear\_gramicidin\_synthase\_subunit\_B | BGC0002051 | NRP | 26.0 | 26.7 | 59.0 | 3.18e-08 |
| QJD55556.1 | acetyl-CoA\_ligase | BGC0001909 | Polyketide | 23.0 | 27.5 | 59.0 | 3.46e-08 |
| QBL56161.1 | long-chain-fatty-acid--CoA\_ligase | BGC0002376 | Polyketide | 24.0 | 24.8 | 59.0 | 4.16e-08 |
| AXM42922.1 | AMP-dependent\_synthetase\_and\_ligase/heterocyclase | BGC0001940 | Polyketide | 21.0 | 33.5 | 56.0 | 4.26e-07 |
| AGM05527.1 | AMP-dependent\_synthetase\_and\_ligase | BGC0002098 | Polyketide | 24.0 | 25.8 | 55.0 | 4.73e-07 |
