# Supplementary material for: Uncovering the transcriptional landscape of Fomes fomentarius during fungal-based material production through gene co-expression network analysis
Source: Fungal Biol Biotechnol. 2025 Feb 13;12:1. doi: 10.1186/s40694-024-00192-3 (PMC11827164; doi:10.1186/s40694-024-00192-3)
Supplement: Supplementary file 1 — Supplementary Material 1 [file 40694_2024_192_MOESM1_ESM.zip › knownclusterblast/region2/jgi.p_Fomfom1_1220113_mibig_hits.html]

| MIBiG Protein | Description | MIBiG Cluster | MiBiG Product | % ID | % Coverage | BLAST Score | E-value |
| --- | --- | --- | --- | --- | --- | --- | --- |
| EIM84829.1 | fatty\_acid-2\_hydroxylase | BGC0002219 | Terpene | 64.0 | 100.5 | 489.0 | 6.5e-174 |
| AAO27755.1 | reductase | BGC0001278 | Terpene | 34.0 | 20.4 | 61.0 | 6.1e-10 |
