# Supplementary material for: Uncovering the transcriptional landscape of Fomes fomentarius during fungal-based material production through gene co-expression network analysis
Source: Fungal Biol Biotechnol. 2025 Feb 13;12:1. doi: 10.1186/s40694-024-00192-3 (PMC11827164; doi:10.1186/s40694-024-00192-3)
Supplement: Supplementary file 1 — Supplementary Material 1 [file 40694_2024_192_MOESM1_ESM.zip › knownclusterblast/region2/jgi.p_Fomfom1_1372685_mibig_hits.html]

| MIBiG Protein | Description | MIBiG Cluster | MiBiG Product | % ID | % Coverage | BLAST Score | E-value |
| --- | --- | --- | --- | --- | --- | --- | --- |
| BAF98642.1 | putative\_glutamyl-tRNA\_amidotransferase\_subunit\_A | BGC0000651 | Terpene | 37.0 | 101.2 | 266.0 | 1.65e-82 |
| ADC45523.1 | secreted\_amidase | BGC0000093 | Polyketide | 36.0 | 99.6 | 246.0 | 1.53e-74 |
| ADQ55485.1 | aspartyl\_glutamyl-tRNA\_Asn\_Gln\_amidotransferase\_subunit\_A | BGC0000350 | NRP:Beta-lactam | 35.0 | 38.4 | 110.0 | 5.34e-26 |
| ATJ34008.1 | amidase | BGC0001442 | NRP | 28.0 | 94.8 | 108.0 | 5.12e-25 |
| ACN29700.1 | putative\_amidase | BGC0000814 | Alkaloid | 39.0 | 29.2 | 76.0 | 2.33e-14 |
| BAV32171.1 | hypothetical\_protein | BGC0001373 | Polyketide | 26.0 | 50.7 | 64.0 | 2.87e-10 |
