# Supplementary material for: Uncovering the transcriptional landscape of Fomes fomentarius during fungal-based material production through gene co-expression network analysis
Source: Fungal Biol Biotechnol. 2025 Feb 13;12:1. doi: 10.1186/s40694-024-00192-3 (PMC11827164; doi:10.1186/s40694-024-00192-3)
Supplement: Supplementary file 1 — Supplementary Material 1 [file 40694_2024_192_MOESM1_ESM.zip › knownclusterblast/region2/jgi.p_Fomfom1_1299880_mibig_hits.html]

| MIBiG Protein | Description | MIBiG Cluster | MiBiG Product | % ID | % Coverage | BLAST Score | E-value |
| --- | --- | --- | --- | --- | --- | --- | --- |
| ESK96610.1 | hypothetical\_protein | BGC0002212 | Polyketide | 27.0 | 101.4 | 343.0 | 1.31e-100 |
| ASK38699.1 | putative\_nonribosomal\_peptide\_synthetase-like\_protein | BGC0001436 | Polyketide:Iterative type I polyketide | 30.0 | 82.0 | 309.0 | 1.3e-88 |
| KFA69336.1 | hypothetical\_protein | BGC0001626 | Polyketide | 27.0 | 83.7 | 281.0 | 5.74e-79 |
| EAU35432.1 | predicted\_protein | BGC0002734 | Polyketide | 26.0 | 101.2 | 281.0 | 7.14e-79 |
| EWG54274.1 | hypothetical\_protein | BGC0001190 | Polyketide | 27.0 | 83.7 | 276.0 | 3.32e-77 |
| BAV19380.1 | NRPS-like\_enzyme | BGC0001390 | NRP+Polyketide | 26.0 | 94.3 | 274.0 | 2.03e-76 |
| AWM95789.1 | non-reduciing\_polyketide\_synthase\_methylorcinaldehyde\_synthase | BGC0001827 | Polyketide | 30.0 | 36.2 | 144.0 | 2.83e-34 |
| CAP95404.1 |  | BGC0001404 | Polyketide | 28.0 | 38.8 | 127.0 | 4.4e-29 |
| AUW31047.1 | PKS-like\_protein | BGC0002483 | Polyketide | 29.0 | 32.0 | 109.0 | 1.99e-25 |
| ATY72525.1 | non-ribosomal\_peptide\_synthetase | BGC0001574 | NRP | 26.0 | 56.1 | 109.0 | 1.23e-23 |
| AEA29644.1 | putative\_nonribosomal\_peptide\_synthetase\_and\_kinurenine\_monooxygenase | BGC0000409 | NRP | 28.0 | 41.7 | 104.0 | 4.72e-22 |
| ATY37592.1 | BogE | BGC0001532 | NRP | 24.0 | 40.3 | 61.0 | 1.23e-08 |
| QHD43130.1 | NRPS/PKS\_hybrid\_protein | BGC0002546 | NRP+Polyketide | 25.0 | 22.7 | 60.0 | 2.2e-08 |
| XP\_001220460.1 | uncharacterized\_protein | BGC0001182 | NRP+Polyketide:Iterative type I polyketide | 25.0 | 23.7 | 57.0 | 1.46e-07 |
| BBC43184.1 | PKS-NRPS\_hybrid | BGC0001738 | NRP+Polyketide | 27.0 | 20.7 | 56.0 | 2.49e-07 |
