# Supplementary material for: Uncovering the transcriptional landscape of Fomes fomentarius during fungal-based material production through gene co-expression network analysis
Source: Fungal Biol Biotechnol. 2025 Feb 13;12:1. doi: 10.1186/s40694-024-00192-3 (PMC11827164; doi:10.1186/s40694-024-00192-3)
Supplement: Supplementary file 1 — Supplementary Material 1 [file 40694_2024_192_MOESM1_ESM.zip › knownclusterblast/region2/jgi.p_Fomfom1_1299910_mibig_hits.html]

| MIBiG Protein | Description | MIBiG Cluster | MiBiG Product | % ID | % Coverage | BLAST Score | E-value |
| --- | --- | --- | --- | --- | --- | --- | --- |
| ESK96610.1 | hypothetical\_protein | BGC0002212 | Polyketide | 27.0 | 105.8 | 165.0 | 1.34e-43 |
| CEF75881.1 |  | BGC0001600 | Polyketide | 27.0 | 114.9 | 147.0 | 1.74e-37 |
| EWG54274.1 | hypothetical\_protein | BGC0001190 | Polyketide | 28.0 | 103.4 | 135.0 | 1.27e-33 |
| AUW31047.1 | PKS-like\_protein | BGC0002483 | Polyketide | 29.0 | 67.0 | 101.0 | 1.52e-23 |
| AEA29644.1 | putative\_nonribosomal\_peptide\_synthetase\_and\_kinurenine\_monooxygenase | BGC0000409 | NRP | 29.0 | 68.3 | 84.0 | 1.21e-16 |
| EOY45602.1 | Adenylation\_and\_reductase\_domains\_containing\_protein | BGC0001168 | NRP | 28.0 | 67.0 | 80.0 | 2.23e-15 |
