# Supplementary material for: Uncovering the transcriptional landscape of Fomes fomentarius during fungal-based material production through gene co-expression network analysis
Source: Fungal Biol Biotechnol. 2025 Feb 13;12:1. doi: 10.1186/s40694-024-00192-3 (PMC11827164; doi:10.1186/s40694-024-00192-3)
Supplement: Supplementary file 1 — Supplementary Material 1 [file 40694_2024_192_MOESM1_ESM.zip › knownclusterblast/region2/jgi.p_Fomfom1_1319683_mibig_hits.html]

| MIBiG Protein | Description | MIBiG Cluster | MiBiG Product | % ID | % Coverage | BLAST Score | E-value |
| --- | --- | --- | --- | --- | --- | --- | --- |
| ESK96610.1 | hypothetical\_protein | BGC0002212 | Polyketide | 28.0 | 101.6 | 359.0 | 2.4e-106 |
| ASK38699.1 | putative\_nonribosomal\_peptide\_synthetase-like\_protein | BGC0001436 | Polyketide:Iterative type I polyketide | 31.0 | 82.3 | 310.0 | 8.89e-89 |
| EAU35432.1 | predicted\_protein | BGC0002734 | Polyketide | 26.0 | 100.7 | 284.0 | 5.86e-80 |
| KFA69336.1 | hypothetical\_protein | BGC0001626 | Polyketide | 27.0 | 89.5 | 284.0 | 6.41e-80 |
| BAV19380.1 | NRPS-like\_enzyme | BGC0001390 | NRP+Polyketide | 25.0 | 99.5 | 273.0 | 3.54e-76 |
| EWG54274.1 | hypothetical\_protein | BGC0001190 | Polyketide | 26.0 | 90.8 | 269.0 | 9.97e-75 |
| AWM95789.1 | non-reduciing\_polyketide\_synthase\_methylorcinaldehyde\_synthase | BGC0001827 | Polyketide | 30.0 | 36.4 | 142.0 | 1.43e-33 |
| CAP95404.1 |  | BGC0001404 | Polyketide | 28.0 | 40.0 | 138.0 | 1.65e-32 |
| ATY72525.1 | non-ribosomal\_peptide\_synthetase | BGC0001574 | NRP | 25.0 | 57.1 | 119.0 | 1.35e-26 |
| AUW31047.1 | PKS-like\_protein | BGC0002483 | Polyketide | 31.0 | 22.2 | 108.0 | 2.68e-25 |
| AEA29644.1 | putative\_nonribosomal\_peptide\_synthetase\_and\_kinurenine\_monooxygenase | BGC0000409 | NRP | 28.0 | 44.1 | 113.0 | 9.12e-25 |
| XP\_001220460.1 | uncharacterized\_protein | BGC0001182 | NRP+Polyketide:Iterative type I polyketide | 25.0 | 24.0 | 61.0 | 9.82e-09 |
| QHD43130.1 | NRPS/PKS\_hybrid\_protein | BGC0002546 | NRP+Polyketide | 22.0 | 36.7 | 59.0 | 4.91e-08 |
| AGO86662.1 | equisetin\_synthetase | BGC0001255 | NRP+Polyketide | 28.0 | 22.2 | 55.0 | 5.55e-07 |
