# Supplementary material for: Uncovering the transcriptional landscape of Fomes fomentarius during fungal-based material production through gene co-expression network analysis
Source: Fungal Biol Biotechnol. 2025 Feb 13;12:1. doi: 10.1186/s40694-024-00192-3 (PMC11827164; doi:10.1186/s40694-024-00192-3)
Supplement: Supplementary file 1 — Supplementary Material 1 [file 40694_2024_192_MOESM1_ESM.zip › knownclusterblast/region2/jgi.p_Fomfom1_1304648_mibig_hits.html]

| MIBiG Protein | Description | MIBiG Cluster | MiBiG Product | % ID | % Coverage | BLAST Score | E-value |
| --- | --- | --- | --- | --- | --- | --- | --- |
| KJA16714.1 | hypothetical\_protein | BGC0002246 | Terpene | 46.0 | 101.9 | 233.0 | 3.61e-76 |
| EIW83690.1 | NAD(P)-binding\_protein | BGC0002707 | Terpene | 45.0 | 99.6 | 219.0 | 8.16e-71 |
| CAL80830.1 | dehydrogenase-related\_protein | BGC0000997 | NRP+Polyketide | 32.0 | 97.4 | 136.0 | 2.34e-38 |
| WP\_018891734.1 | SDR\_family\_oxidoreductase | BGC0001558 | Polyketide | 34.0 | 64.9 | 71.0 | 4.99e-14 |
| ATJ00769.1 | C-7\_ketoreductase | BGC0001568 | Polyketide | 34.0 | 64.9 | 71.0 | 5.33e-14 |
| QCT05738.1 | Tri5 | BGC0001983 | Other | 30.0 | 76.1 | 69.0 | 1.14e-13 |
| BAF14088.1 |  | BGC0000671 | Terpene | 31.0 | 66.0 | 70.0 | 1.35e-13 |
| KDN80052.1 | ketoreductase | BGC0001074 | Saccharide+Polyketide | 34.0 | 64.9 | 68.0 | 3.32e-13 |
| BAF14087.1 |  | BGC0000671 | Terpene | 30.0 | 66.0 | 64.0 | 1.04e-11 |
| QVQ68802.1 | mmyTIII | BGC0002129 | Polyketide | 32.0 | 63.4 | 64.0 | 1.53e-11 |
| AHN91930.1 | short-chain\_dehydrogenase/reductase\_SDR | BGC0000340 | NRP | 31.0 | 67.2 | 63.0 | 2.64e-11 |
| ABL09955.1 | ketoreductase | BGC0000197 | Polyketide:Type II polyketide+Saccharide:Hybrid/tailoring saccharide | 30.0 | 67.9 | 63.0 | 3.16e-11 |
| ABS75820.1 | BacC | BGC0001184 | Other | 30.0 | 53.0 | 62.0 | 5.43e-11 |
| EEF48747.1 | short\_chain\_alcohol\_dehydrogenase,\_putative | BGC0002393 | Terpene | 29.0 | 63.4 | 62.0 | 9.49e-11 |
| ACX35428.1 | BacC | BGC0000888 | Other | 30.0 | 53.0 | 61.0 | 1.37e-10 |
| AHX24712.1 | 3-oxoacyl-ACP\_reductase | BGC0000200 | Polyketide:Type II polyketide+Saccharide:Hybrid/tailoring saccharide | 33.0 | 62.3 | 59.0 | 4.33e-10 |
| ABY83162.1 | Azi24 | BGC0000960 | NRP+Polyketide | 27.0 | 65.7 | 57.0 | 3.33e-09 |
| ACA34721.1 | CtnE | BGC0000894 | Other | 27.0 | 66.0 | 48.0 | 4.31e-06 |
| ALI92649.1 | CitE\_dehydrogenase | BGC0001338 | Polyketide:Iterative type I polyketide | 27.0 | 66.0 | 48.0 | 4.6e-06 |
