# Supplementary material for: Uncovering the transcriptional landscape of Fomes fomentarius during fungal-based material production through gene co-expression network analysis
Source: Fungal Biol Biotechnol. 2025 Feb 13;12:1. doi: 10.1186/s40694-024-00192-3 (PMC11827164; doi:10.1186/s40694-024-00192-3)
Supplement: Supplementary file 1 — Supplementary Material 1 [file 40694_2024_192_MOESM1_ESM.zip › knownclusterblast/region2/jgi.p_Fomfom1_1391491_mibig_hits.html]

| MIBiG Protein | Description | MIBiG Cluster | MiBiG Product | % ID | % Coverage | BLAST Score | E-value |
| --- | --- | --- | --- | --- | --- | --- | --- |
| KJA16714.1 | hypothetical\_protein | BGC0002246 | Terpene | 44.0 | 97.6 | 243.0 | 1.03e-79 |
| EIW83690.1 | NAD(P)-binding\_protein | BGC0002707 | Terpene | 42.0 | 98.6 | 214.0 | 1.74e-68 |
| QQW45476.1 | short-chain\_dehydrogenase/reductase\_CalM' | BGC0002168 | Polyketide | 34.0 | 99.3 | 153.0 | 1.96e-44 |
| BBM05072.1 | putative\_oxidoreductase | BGC0002170 | Polyketide | 34.0 | 99.3 | 153.0 | 1.96e-44 |
| ATZ56112.1 | Bcbot7 | BGC0000631 | Terpene | 42.0 | 75.4 | 145.0 | 3.47e-41 |
| QTA30590.1 | short\_chain\_dehydrogenase | BGC0002143 | Polyketide | 34.0 | 94.5 | 139.0 | 2.37e-39 |
| ALJ49935.1 | TtmK | BGC0001236 | Polyketide | 31.0 | 94.5 | 129.0 | 2.76e-35 |
| AGY62761.1 | reductase | BGC0000051 | Polyketide | 33.0 | 97.3 | 128.0 | 7.65e-35 |
| SCN11955.1 | short-chain\_alcohol\_dehydrogenase | BGC0001580 | Polyketide | 33.0 | 97.3 | 128.0 | 7.65e-35 |
| KDN80075.1 | 3-ketoacyl-ACP\_reductase | BGC0001074 | Saccharide+Polyketide | 32.0 | 94.2 | 125.0 | 4.11e-34 |
| QGA70091.1 | putative\_oxidoreductase | BGC0002517 | Polyketide | 32.0 | 90.8 | 114.0 | 1.69e-29 |
| BAB72043.1 | AknA | BGC0000191 | Polyketide | 32.0 | 78.5 | 82.0 | 4.87e-18 |
| QTA30588.1 | SDR\_family\_NAD(P)-dependent\_oxidoreductase | BGC0002143 | Polyketide | 31.0 | 77.1 | 81.0 | 1.75e-17 |
| AGO50613.1 | ketoreductase | BGC0000229 | Polyketide:Type II polyketide+Saccharide:Hybrid/tailoring saccharide | 30.0 | 76.1 | 79.0 | 6.27e-17 |
| AAF70104.1 | AknA | BGC0000192 | Polyketide | 32.0 | 78.5 | 77.0 | 5.78e-16 |
| BAV17002.1 | putative\_ketoreductase | BGC0001384 | Polyketide | 29.0 | 76.1 | 76.0 | 1.49e-15 |
| ARO44671.1 | ketoreductase | BGC0001769 | Polyketide | 29.0 | 76.1 | 75.0 | 2.04e-15 |
| MBW8699686.1 | putative\_ketoacyl\_reductase | BGC0002140 | Polyketide | 29.0 | 76.1 | 75.0 | 2.04e-15 |
| WP\_018891732.1 | 3-oxoacyl-ACP\_reductase\_FabG | BGC0001558 | Polyketide | 31.0 | 77.1 | 75.0 | 2.8e-15 |
| ATJ00771.1 | ketoacyl\_reductase | BGC0001568 | Polyketide | 31.0 | 77.1 | 74.0 | 5.25e-15 |
| CAH10114.1 | putative\_ketoreducatse | BGC0000268 | Polyketide | 29.0 | 76.1 | 74.0 | 7.66e-15 |
| TRO56980.1 | SDR\_family\_NAD(P)-dependent\_oxidoreductase | BGC0002361 | Polyketide+Saccharide | 29.0 | 76.1 | 74.0 | 7.66e-15 |
| XP\_004252844.1 | uncharacterized\_protein\_LOC101268370 | BGC0002404 | Other | 32.0 | 68.6 | 73.0 | 1.5e-14 |
| ATJ00769.1 | C-7\_ketoreductase | BGC0001568 | Polyketide | 30.0 | 67.9 | 72.0 | 1.55e-14 |
| PPQ57492.1 | ketoacyl\_reductase | BGC0002016 | Polyketide | 29.0 | 76.5 | 72.0 | 3.44e-14 |
| WP\_018891734.1 | SDR\_family\_oxidoreductase | BGC0001558 | Polyketide | 30.0 | 66.9 | 71.0 | 3.73e-14 |
| WP\_020275098.1 | SDR\_family\_NAD(P)-dependent\_oxidoreductase | BGC0002012 | Polyketide | 31.0 | 66.9 | 71.0 | 4.71e-14 |
| ACX83621.1 | keto\_reductase | BGC0000221 | Polyketide | 31.0 | 66.2 | 71.0 | 6.43e-14 |
| KDN80050.1 | ketoacyl\_reductase | BGC0001074 | Saccharide+Polyketide | 31.0 | 77.5 | 71.0 | 6.43e-14 |
| AAA65204.1 | daunorubicin-doxorubicin\_polyketide\_synthase | BGC0000218 | Polyketide | 31.0 | 66.9 | 70.0 | 1.2e-13 |
| CCA54213.1 | CmlJ | BGC0000893 | NRP | 25.0 | 60.8 | 69.0 | 2.11e-13 |
| BAF14088.1 |  | BGC0000671 | Terpene | 31.0 | 68.3 | 69.0 | 3.51e-13 |
| ABL09955.1 | ketoreductase | BGC0000197 | Polyketide:Type II polyketide+Saccharide:Hybrid/tailoring saccharide | 29.0 | 66.9 | 69.0 | 4.15e-13 |
| QNL10616.1 | Ketoacyl\_reductase | BGC0002514 | Polyketide | 28.0 | 66.9 | 68.0 | 7.72e-13 |
| AAG03071.1 | putative\_reductase | BGC0000266 | Polyketide | 29.0 | 74.1 | 67.0 | 9.16e-13 |
| KDN80052.1 | ketoreductase | BGC0001074 | Saccharide+Polyketide | 32.0 | 71.7 | 67.0 | 1.58e-12 |
| QLQ36629.1 | SDR\_family\_NAD(P)-dependent\_oxidoreductase | BGC0002097 | NRP+Polyketide:Type II polyketide+Saccharide:Hybrid/tailoring saccharide | 30.0 | 78.2 | 67.0 | 1.95e-12 |
| BAF14087.1 |  | BGC0000671 | Terpene | 30.0 | 75.8 | 67.0 | 2.22e-12 |
| ADI71446.1 | putative\_ketoreductase | BGC0000203 | Polyketide | 29.0 | 66.6 | 66.0 | 2.65e-12 |
| EEF48747.1 | short\_chain\_alcohol\_dehydrogenase,\_putative | BGC0002393 | Terpene | 29.0 | 59.7 | 66.0 | 4.45e-12 |
| AAL24452.1 | RdmJ | BGC0000265 | Polyketide | 32.0 | 59.7 | 66.0 | 5.06e-12 |
| AHN91930.1 | short-chain\_dehydrogenase/reductase\_SDR | BGC0000340 | NRP | 27.0 | 64.5 | 65.0 | 7.45e-12 |
| CAC44199.1 | ketoacyl\_reductase | BGC0000194 | Polyketide:Type II polyketide | 30.0 | 70.6 | 64.0 | 1.23e-11 |
| AHL46698.1 | ketoreductase | BGC0001177 | Polyketide:Type II polyketide | 27.0 | 66.6 | 63.0 | 3.08e-11 |
| AAN05759.1 | dehydrogenase\_DhgA | BGC0000769 | Saccharide | 26.0 | 84.6 | 62.0 | 1.02e-10 |
| BAL90288.1 | putative\_short-chain\_dehydrogenase | BGC0002021 | Polyketide | 29.0 | 69.3 | 61.0 | 1.4e-10 |
| AHD25940.1 | putative\_ketoreductase | BGC0000208 | Polyketide | 27.0 | 67.2 | 61.0 | 1.43e-10 |
| ABY83162.1 | Azi24 | BGC0000960 | NRP+Polyketide | 26.0 | 62.1 | 57.0 | 4.24e-09 |
| EEF48735.1 | short\_chain\_alcohol\_dehydrogenase,\_putative | BGC0002393 | Terpene | 26.0 | 65.2 | 56.0 | 1.12e-08 |
| QVQ68802.1 | mmyTIII | BGC0002129 | Polyketide | 27.0 | 67.2 | 56.0 | 1.19e-08 |
| QCX41935.1 | AmcA | BGC0001957 | Polyketide | 27.0 | 40.3 | 53.0 | 1.04e-07 |
| QHW08564.1 | SDR\_family\_oxidoreductase | BGC0002054 | Polyketide+NRP+Saccharide | 27.0 | 40.3 | 53.0 | 1.04e-07 |
| BBC20651.1 | enoyl-(acyl\_carrier\_protein)\_reductase | BGC0001917 | Polyketide | 26.0 | 76.1 | 49.0 | 1.86e-06 |
