# Supplementary material for: Uncovering the transcriptional landscape of Fomes fomentarius during fungal-based material production through gene co-expression network analysis
Source: Fungal Biol Biotechnol. 2025 Feb 13;12:1. doi: 10.1186/s40694-024-00192-3 (PMC11827164; doi:10.1186/s40694-024-00192-3)
Supplement: Supplementary file 1 — Supplementary Material 1 [file 40694_2024_192_MOESM1_ESM.zip › knownclusterblast/region2/jgi.p_Fomfom1_1210441_mibig_hits.html]

| MIBiG Protein | Description | MIBiG Cluster | MiBiG Product | % ID | % Coverage | BLAST Score | E-value |
| --- | --- | --- | --- | --- | --- | --- | --- |
| ABB90286.1 | protein\_kinase\_Eg2-like\_protein | BGC0001057 | NRP+Polyketide | 29.0 | 24.0 | 126.0 | 4e-31 |
| XP\_011392657.1 | serine/threonine\_protein\_kinase\_ATG1 | BGC0001281 | Polyketide | 25.0 | 22.6 | 93.0 | 1.42e-18 |
| APZ78839.1 | protein\_kinase | BGC0001430 | NRP:Cyclic depsipeptide+Polyketide:Iterative type I polyketide | 26.0 | 21.1 | 56.0 | 1.85e-07 |
