# Supplementary material for: Uncovering the transcriptional landscape of Fomes fomentarius during fungal-based material production through gene co-expression network analysis
Source: Fungal Biol Biotechnol. 2025 Feb 13;12:1. doi: 10.1186/s40694-024-00192-3 (PMC11827164; doi:10.1186/s40694-024-00192-3)
Supplement: Supplementary file 1 — Supplementary Material 1 [file 40694_2024_192_MOESM1_ESM.zip › knownclusterblast/region2/jgi.p_Fomfom1_1373402_mibig_hits.html]

| MIBiG Protein | Description | MIBiG Cluster | MiBiG Product | % ID | % Coverage | BLAST Score | E-value |
| --- | --- | --- | --- | --- | --- | --- | --- |
| KJA16714.1 | hypothetical\_protein | BGC0002246 | Terpene | 55.0 | 78.9 | 71.0 | 5.43e-16 |
| ATZ45194.1 | Bcboa17 | BGC0001892 | Polyketide | 46.0 | 77.9 | 59.0 | 1.2e-11 |
| QTA30590.1 | short\_chain\_dehydrogenase | BGC0002143 | Polyketide | 53.0 | 62.1 | 56.0 | 1.62e-10 |
| ALJ49935.1 | TtmK | BGC0001236 | Polyketide | 53.0 | 57.9 | 56.0 | 2.19e-10 |
| KDN80075.1 | 3-ketoacyl-ACP\_reductase | BGC0001074 | Saccharide+Polyketide | 53.0 | 57.9 | 54.0 | 7.85e-10 |
