# Supplementary material for: Uncovering the transcriptional landscape of Fomes fomentarius during fungal-based material production through gene co-expression network analysis
Source: Fungal Biol Biotechnol. 2025 Feb 13;12:1. doi: 10.1186/s40694-024-00192-3 (PMC11827164; doi:10.1186/s40694-024-00192-3)
Supplement: Supplementary file 1 — Supplementary Material 1 [file 40694_2024_192_MOESM1_ESM.zip › knownclusterblast/region2/jgi.p_Fomfom1_1373404_mibig_hits.html]

| MIBiG Protein | Description | MIBiG Cluster | MiBiG Product | % ID | % Coverage | BLAST Score | E-value |
| --- | --- | --- | --- | --- | --- | --- | --- |
| KJA16714.1 | hypothetical\_protein | BGC0002246 | Terpene | 47.0 | 98.6 | 261.0 | 1.74e-86 |
| EIW83690.1 | NAD(P)-binding\_protein | BGC0002707 | Terpene | 47.0 | 98.3 | 242.0 | 3.66e-79 |
| WP\_099049898.1 | SDR\_family\_NAD(P)-dependent\_oxidoreductase | BGC0002002 | NRP | 34.0 | 94.6 | 149.0 | 3.09e-43 |
| QQW45476.1 | short-chain\_dehydrogenase/reductase\_CalM' | BGC0002168 | Polyketide | 34.0 | 100.0 | 139.0 | 4.25e-39 |
| BBM05072.1 | putative\_oxidoreductase | BGC0002170 | Polyketide | 34.0 | 100.0 | 139.0 | 4.25e-39 |
| EDY42546.1 | ketoreductase | BGC0000212 | Polyketide:Type II polyketide | 32.0 | 60.0 | 77.0 | 3.86e-16 |
| QTA30588.1 | SDR\_family\_NAD(P)-dependent\_oxidoreductase | BGC0002143 | Polyketide | 27.0 | 91.9 | 76.0 | 1.12e-15 |
| CAE45661.1 | putative\_ketoreductase | BGC0000031 | Polyketide:Modular type I polyketide | 27.0 | 77.3 | 76.0 | 1.18e-15 |
| ALV82322.1 | ketoreductase | BGC0001533 | Polyketide | 27.0 | 77.3 | 76.0 | 1.18e-15 |
| TRO56980.1 | SDR\_family\_NAD(P)-dependent\_oxidoreductase | BGC0002361 | Polyketide+Saccharide | 27.0 | 93.6 | 75.0 | 2.24e-15 |
| WP\_016640238.1 | 3-oxoacyl-ACP\_reductase\_FabG | BGC0002000 | Polyketide | 27.0 | 92.2 | 74.0 | 3.94e-15 |
| CAH10114.1 | putative\_ketoreducatse | BGC0000268 | Polyketide | 27.0 | 92.2 | 74.0 | 4.2e-15 |
| AHX24706.1 | 3-oxoacyl-ACP\_reductase | BGC0000200 | Polyketide:Type II polyketide+Saccharide:Hybrid/tailoring saccharide | 33.0 | 65.1 | 74.0 | 4.78e-15 |
| CAA54861.1 | ketoreductase | BGC0000231 | Polyketide | 27.0 | 91.5 | 73.0 | 1.01e-14 |
| PPQ57492.1 | ketoacyl\_reductase | BGC0002016 | Polyketide | 26.0 | 91.2 | 73.0 | 1.38e-14 |
| AGO50613.1 | ketoreductase | BGC0000229 | Polyketide:Type II polyketide+Saccharide:Hybrid/tailoring saccharide | 27.0 | 90.8 | 72.0 | 1.89e-14 |
| EEF48747.1 | short\_chain\_alcohol\_dehydrogenase,\_putative | BGC0002393 | Terpene | 27.0 | 84.7 | 72.0 | 4.53e-14 |
| EEF48735.1 | short\_chain\_alcohol\_dehydrogenase,\_putative | BGC0002393 | Terpene | 28.0 | 64.1 | 72.0 | 4.58e-14 |
| AAX57194.1 | auricin\_polyketide\_ketoreductase | BGC0000201 | Polyketide+Saccharide:Hybrid/tailoring saccharide | 27.0 | 91.5 | 71.0 | 4.83e-14 |
| AAO65349.1 | putative\_ketoreductase | BGC0000236 | Polyketide | 25.0 | 91.2 | 69.0 | 2.29e-13 |
| ARO44671.1 | ketoreductase | BGC0001769 | Polyketide | 26.0 | 90.8 | 69.0 | 3.12e-13 |
| MBW8699686.1 | putative\_ketoacyl\_reductase | BGC0002140 | Polyketide | 26.0 | 90.8 | 69.0 | 3.12e-13 |
| AXL88813.1 | ketoacyl\_reductase | BGC0001895 | Polyketide | 25.0 | 92.2 | 69.0 | 4.3e-13 |
| WP\_020275098.1 | SDR\_family\_NAD(P)-dependent\_oxidoreductase | BGC0002012 | Polyketide | 27.0 | 91.9 | 67.0 | 1.08e-12 |
| BAF14087.1 |  | BGC0000671 | Terpene | 32.0 | 65.1 | 67.0 | 1.23e-12 |
| BAV17002.1 | putative\_ketoreductase | BGC0001384 | Polyketide | 25.0 | 91.5 | 67.0 | 1.46e-12 |
| AAB36565.1 | ketoreductase | BGC0000234 | Polyketide | 25.0 | 92.2 | 67.0 | 1.51e-12 |
| CAG14968.1 | ketoreductase | BGC0000253 | Polyketide:Type II polyketide | 25.0 | 91.5 | 67.0 | 1.97e-12 |
| ARK36158.1 | ketoacyl\_reductase | BGC0001723 | Polyketide | 25.0 | 91.5 | 67.0 | 1.97e-12 |
| QDG00823.1 | polyketide\_ketoreductase | BGC0002028 | Polyketide | 26.0 | 92.9 | 67.0 | 1.97e-12 |
| AVO00812.1 | May13 | BGC0001661 | Polyketide | 26.0 | 90.2 | 67.0 | 2.01e-12 |
| BAF14088.1 |  | BGC0000671 | Terpene | 31.0 | 64.4 | 66.0 | 5.69e-12 |
| AFN69430.1 | ElxO | BGC0000509 | RiPP:Lanthipeptide | 29.0 | 64.4 | 65.0 | 5.92e-12 |
| ABS74183.1 | YxjF | BGC0001090 | Polyketide+NRP:Lipopeptide | 25.0 | 56.3 | 65.0 | 6.81e-12 |
| BAB69696.1 |  | BGC0001098 | NRP+Polyketide | 25.0 | 56.3 | 65.0 | 6.81e-12 |
| QNL10616.1 | Ketoacyl\_reductase | BGC0002514 | Polyketide | 26.0 | 92.2 | 63.0 | 3.15e-11 |
| BCJ07579.1 | 3-oxoacyl-ACP\_reductase | BGC0002379 | NRP | 30.0 | 68.1 | 62.0 | 5.9e-11 |
| ADI71446.1 | putative\_ketoreductase | BGC0000203 | Polyketide | 25.0 | 92.2 | 62.0 | 7.85e-11 |
| ABS75820.1 | BacC | BGC0001184 | Other | 25.0 | 62.4 | 58.0 | 2.04e-09 |
| ACX35428.1 | BacC | BGC0000888 | Other | 25.0 | 62.4 | 56.0 | 9.2e-09 |
| AHX24712.1 | 3-oxoacyl-ACP\_reductase | BGC0000200 | Polyketide:Type II polyketide+Saccharide:Hybrid/tailoring saccharide | 31.0 | 47.5 | 53.0 | 9.47e-08 |
