# Supplementary material for: Uncovering the transcriptional landscape of Fomes fomentarius during fungal-based material production through gene co-expression network analysis
Source: Fungal Biol Biotechnol. 2025 Feb 13;12:1. doi: 10.1186/s40694-024-00192-3 (PMC11827164; doi:10.1186/s40694-024-00192-3)
Supplement: Supplementary file 1 — Supplementary Material 1 [file 40694_2024_192_MOESM1_ESM.zip › knownclusterblast/region2/jgi.p_Fomfom1_1210450_mibig_hits.html]

| MIBiG Protein | Description | MIBiG Cluster | MiBiG Product | % ID | % Coverage | BLAST Score | E-value |
| --- | --- | --- | --- | --- | --- | --- | --- |
| BBQ09589.1 | MFS\_transporter | BGC0002261 | Polyketide | 29.0 | 101.0 | 193.0 | 5.69e-55 |
| ATY69599.1 | antibiotic\_efflux\_protein | BGC0001823 | NRP+Polyketide | 31.0 | 100.6 | 190.0 | 3.57e-54 |
| BAV16993.1 | putative\_transporter | BGC0001384 | Polyketide | 31.0 | 101.8 | 186.0 | 1.06e-52 |
| QEU90619.1 | MFS\_transporter | BGC0000703 | Saccharide | 31.0 | 100.0 | 188.0 | 1.12e-51 |
| CAF60521.1 | putative\_efflux\_protein | BGC0000704 | Saccharide | 31.0 | 100.0 | 188.0 | 1.12e-51 |
| CAF31575.1 | putative\_kanamycin\_efflux\_protein | BGC0000705 | Saccharide | 31.0 | 100.0 | 188.0 | 1.12e-51 |
| AAQ08935.1 | putative\_membrane\_transporter | BGC0000224 | Polyketide:Type II polyketide | 32.0 | 99.2 | 181.0 | 8.23e-51 |
| AEI98661.1 | CtcR | BGC0000209 | Polyketide | 30.0 | 101.2 | 177.0 | 1.49e-49 |
| AHW57792.1 | PgaJ3 | BGC0000262 | Polyketide:Type II polyketide+Saccharide:Hybrid/tailoring saccharide | 30.0 | 99.2 | 177.0 | 3.87e-49 |
| ADG86335.1 | transporter | BGC0000190 | Polyketide | 32.0 | 100.2 | 176.0 | 3.98e-49 |
| ARO44650.1 | transporter | BGC0001769 | Polyketide | 29.0 | 101.2 | 174.0 | 2.69e-48 |
| MBW8699677.1 | Multidrug\_resistance\_protein\_3 | BGC0002140 | Polyketide | 29.0 | 101.2 | 174.0 | 3.73e-48 |
| AAD13557.1 | LanJ | BGC0000239 | Polyketide:Type II polyketide+Saccharide:Hybrid/tailoring saccharide | 29.0 | 101.2 | 173.0 | 8.49e-48 |
| ATY69558.1 | antibiotic\_efflux\_protein | BGC0001611 | NRP+Polyketide | 28.0 | 100.2 | 170.0 | 1.17e-46 |
| AGO50605.1 | transporter | BGC0000229 | Polyketide:Type II polyketide+Saccharide:Hybrid/tailoring saccharide | 30.0 | 95.5 | 169.0 | 1.79e-46 |
| ACS68556.1 | major\_facilitator\_superfamily\_protein | BGC0001026 | NRP+Polyketide | 27.0 | 97.5 | 170.0 | 1.91e-46 |
| TRO57000.1 | DHA2\_family\_efflux\_MFS\_transporter\_permease\_subunit | BGC0002361 | Polyketide+Saccharide | 28.0 | 100.4 | 167.0 | 7.92e-46 |
| CAH10123.1 | putative\_transporter | BGC0000268 | Polyketide | 28.0 | 100.8 | 167.0 | 1.3e-45 |
| ABV91295.1 | putative\_multidrug\_transporter | BGC0000158 | Polyketide:Modular type I polyketide | 29.0 | 100.4 | 166.0 | 4.09e-45 |
| QLJ99336.2 | MFS\_transporter | BGC0002088 | Polyketide+Saccharide:Oligosaccharide | 30.0 | 101.6 | 165.0 | 5e-45 |
| ACP19369.1 | SaqJ1 | BGC0000267 | Polyketide:Type II polyketide+Saccharide:Oligosaccharide | 29.0 | 102.1 | 165.0 | 5.69e-45 |
| QOG08945.1 | FfsH | BGC0002204 | Polyketide+NRP | 30.0 | 93.4 | 163.0 | 7.74e-44 |
| CAP93755.1 |  | BGC0001882 | Polyketide | 26.0 | 101.4 | 162.0 | 1.62e-43 |
| AAF00219.1 | transporter | BGC0000277 | Polyketide | 27.0 | 100.2 | 160.0 | 5.77e-43 |
| QCS37513.1 | pyiT | BGC0001881 | NRP+Polyketide:Iterative type I polyketide | 26.0 | 104.9 | 155.0 | 9.52e-41 |
| OWA25250.1 | MFS\_transporter | BGC0001438 | Polyketide+Saccharide:Hybrid/tailoring saccharide | 29.0 | 101.4 | 153.0 | 1.95e-40 |
| OJJ98488.1 | hypothetical\_protein | BGC0002169 | Polyketide+NRP | 27.0 | 102.1 | 151.0 | 9.45e-40 |
| AAM94765.1 | CalT1 | BGC0000033 | Polyketide | 28.0 | 102.3 | 150.0 | 1.3e-39 |
| OSS48493.1 | hypothetical\_protein | BGC0002194 | Polyketide | 26.0 | 101.2 | 150.0 | 2.31e-39 |
| EHA55869.1 | hypothetical\_protein | BGC0002235 | Polyketide+NRP | 27.0 | 100.8 | 150.0 | 2.31e-39 |
| AFU65888.1 | DacR2 | BGC0000216 | Polyketide | 27.0 | 98.6 | 147.0 | 1.53e-38 |
| AAL15595.1 | Sim17 | BGC0000270 | Polyketide | 28.0 | 102.3 | 140.0 | 6.81e-36 |
| AFB35617.1 | Exporter | BGC0000935 | Polyketide+Other:Aminocoumarin | 27.0 | 100.8 | 139.0 | 1.26e-35 |
| OWA01612.1 | hypothetical\_protein | BGC0001439 | Polyketide+Saccharide:Hybrid/tailoring saccharide | 27.0 | 100.2 | 140.0 | 3.15e-35 |
| AAK06799.1 | simocyclinone-specific\_efflux\_pump | BGC0001072 | Saccharide+Polyketide:Modular type I polyketide+Polyketide:Type II polyketide+Other:Aminocoumarin | 27.0 | 102.3 | 138.0 | 4.42e-35 |
| CAA09636.1 | putative\_transmembrane\_protein | BGC0000227 | Polyketide:Type II polyketide | 25.0 | 104.9 | 137.0 | 7.61e-35 |
| ABC87523.1 | putative\_drug\_efflux\_transporter | BGC0001011 | NRP+Polyketide | 32.0 | 85.6 | 135.0 | 1.89e-34 |
| KIS69144.1 | Major\_Facilitator\_invovled\_in\_MEL\_transport | BGC0001888 | Other | 26.0 | 100.8 | 136.0 | 4.25e-34 |
| AMY15059.1 | MFS\_transporter | BGC0001339 | Polyketide:Iterative type I polyketide | 27.0 | 94.9 | 135.0 | 6.97e-34 |
| AFW04557.1 | drug\_resistance\_transporter | BGC0001783 | Other | 29.0 | 97.1 | 134.0 | 1.01e-33 |
| QCL09095.1 | DmxR4 | BGC0002063 | Polyketide:Iterative type I polyketide | 25.0 | 98.2 | 133.0 | 3.27e-33 |
| AAD34558.1 | unknown | BGC0000088 | Polyketide | 28.0 | 102.3 | 131.0 | 1.25e-32 |
| ABA02247.1 | efflux\_pump | BGC0000098 | Polyketide | 26.0 | 99.6 | 124.0 | 3.09e-30 |
| QDK64761.1 | AshT | BGC0002301 | Polyketide | 23.0 | 100.4 | 124.0 | 4.35e-30 |
| AFW04570.1 | drug\_resistance\_transporter | BGC0001783 | Other | 29.0 | 97.7 | 122.0 | 2.18e-29 |
| ADI24949.1 | GsfJ | BGC0000070 | Polyketide:Iterative type I polyketide | 25.0 | 101.8 | 121.0 | 3.76e-29 |
| BAZ95819.1 | cpaN1\_MFS\_transporter | BGC0001563 | NRP+Polyketide | 28.0 | 66.1 | 114.0 | 7.08e-27 |
| QWM97859.1 | MFS\_transporter | BGC0002434 | Polyketide+NRP | 32.0 | 61.6 | 112.0 | 2.05e-26 |
| EYT83437.1 | multidrug\_MFS\_transporter | BGC0001213 | Polyketide | 31.0 | 101.2 | 112.0 | 2.88e-26 |
| AEK75506.1 | EmrB/QacA\_drug\_resistance\_transporter | BGC0000001 | Polyketide:Modular type I polyketide | 26.0 | 85.4 | 105.0 | 4.68e-24 |
| AAO65328.1 | putative\_transmembrane\_efflux\_protein | BGC0000236 | Polyketide | 25.0 | 83.2 | 100.0 | 2.1e-22 |
| WP\_063778692.1 | MFS\_transporter | BGC0001596 | Polyketide | 29.0 | 48.3 | 87.0 | 8.87e-18 |
| CAJ34383.1 | putative\_permease\_for\_glucides | BGC0000445 | NRP:Cyclic depsipeptide | 26.0 | 83.6 | 67.0 | 1.37e-11 |
| CAE52325.1 | putative\_albicidin\_efflux\_pump | BGC0001088 | NRP+Polyketide | 22.0 | 88.3 | 58.0 | 1.48e-08 |
| XP\_001827195.1 |  | BGC0001996 | Other | 29.0 | 30.6 | 50.0 | 5.29e-06 |
