# Supplementary material for: Uncovering the transcriptional landscape of Fomes fomentarius during fungal-based material production through gene co-expression network analysis
Source: Fungal Biol Biotechnol. 2025 Feb 13;12:1. doi: 10.1186/s40694-024-00192-3 (PMC11827164; doi:10.1186/s40694-024-00192-3)
Supplement: Supplementary file 1 — Supplementary Material 1 [file 40694_2024_192_MOESM1_ESM.zip › knownclusterblast/region3/jgi.p_Fomfom1_78215_mibig_hits.html]

| MIBiG Protein | Description | MIBiG Cluster | MiBiG Product | % ID | % Coverage | BLAST Score | E-value |
| --- | --- | --- | --- | --- | --- | --- | --- |
| XP\_011392665.1 | uncharacterized\_protein | BGC0001281 | Polyketide | 42.0 | 103.5 | 280.0 | 4.38e-92 |
| KIA75588.1 | NAD\_dependent\_dehydratase | BGC0002209 | Polyketide | 34.0 | 99.7 | 186.0 | 3.89e-56 |
| EHA28232.1 | hypothetical\_protein | BGC0001143 | Polyketide | 35.0 | 99.4 | 180.0 | 1.23e-53 |
| ART41210.1 | AdrE | BGC0001508 | Polyketide | 31.0 | 100.0 | 128.0 | 4.72e-34 |
| gene3 |  | BGC0001907 | Polyketide | 33.0 | 68.2 | 121.0 | 7.42e-32 |
| CBK62736.1 |  | BGC0001115 | NRP+Polyketide | 27.0 | 80.8 | 80.0 | 2.38e-16 |
