# Supplementary material for: Uncovering the transcriptional landscape of Fomes fomentarius during fungal-based material production through gene co-expression network analysis
Source: Fungal Biol Biotechnol. 2025 Feb 13;12:1. doi: 10.1186/s40694-024-00192-3 (PMC11827164; doi:10.1186/s40694-024-00192-3)
Supplement: Supplementary file 1 — Supplementary Material 1 [file 40694_2024_192_MOESM1_ESM.zip › knownclusterblast/region3/jgi.p_Fomfom1_1313385_mibig_hits.html]

| MIBiG Protein | Description | MIBiG Cluster | MiBiG Product | % ID | % Coverage | BLAST Score | E-value |
| --- | --- | --- | --- | --- | --- | --- | --- |
| QQO98481.1 | FrzL | BGC0002146 | NRP | 31.0 | 115.6 | 170.0 | 1.87e-47 |
| CBF82795.1 | cytochrome\_P450,\_putative\_(Eurofung) | BGC0001668 | NRP | 29.0 | 119.9 | 153.0 | 6.65e-41 |
| EAL85116.1 | cytochrome\_P450\_oxidoreductase | BGC0001067 | Terpene+Polyketide:Iterative type I polyketide | 25.0 | 122.7 | 142.0 | 4.66e-37 |
| XP\_007301850.1 | cytochrome\_P450 | BGC0001617 | Terpene | 30.0 | 68.8 | 132.0 | 2.18e-33 |
| FAC38\_04 |  | BGC0002198 | NRP | 28.0 | 118.9 | 131.0 | 3.62e-33 |
| QJQ03972.1 | CYP-Arm3 | BGC0002445 | Terpene | 32.0 | 70.2 | 131.0 | 3.73e-33 |
| KIJ60843.1 | hypothetical\_protein | BGC0002214 | Polyketide | 31.0 | 68.6 | 129.0 | 9.05e-33 |
| QJQ03971.1 | CYP-Arm2 | BGC0002445 | Terpene | 36.0 | 54.1 | 124.0 | 1.3e-30 |
| KIJ60837.1 | hypothetical\_protein | BGC0002214 | Polyketide | 34.0 | 61.2 | 122.0 | 3.24e-30 |
| KIJ60846.1 | hypothetical\_protein | BGC0002214 | Polyketide | 31.0 | 71.9 | 122.0 | 4.56e-30 |
| PPQ83216.1 | Dimethyrltryptamine\_4-hydroxylase\_(PsiH) | BGC0002207 | Other | 34.0 | 58.4 | 122.0 | 5.16e-30 |
| KAA1470686.1 | cytochrome\_P450 | BGC0002218 | Terpene | 37.0 | 48.7 | 120.0 | 1.69e-29 |
| KIJ60841.1 | hypothetical\_protein | BGC0002214 | Polyketide | 42.0 | 34.8 | 105.0 | 2.14e-24 |
| XP\_007301851.1 | cytochrome\_P450 | BGC0001617 | Terpene | 26.0 | 94.8 | 91.0 | 1.92e-19 |
| KJA16708.1 | hypothetical\_protein | BGC0002246 | Terpene | 23.0 | 81.8 | 76.0 | 1.44e-14 |
| CYP82Y1 |  | BGC0001325 | Alkaloid | 37.0 | 20.3 | 64.0 | 1.78e-10 |
| XP\_020057670.1 | uncharacterized\_protein | BGC0001718 | NRP | 25.0 | 104.7 | 60.0 | 2.18e-09 |
| BAF09099.1 |  | BGC0000672 | Terpene | 33.0 | 23.2 | 54.0 | 2.51e-07 |
| BAF09098.1 |  | BGC0000672 | Terpene | 31.0 | 23.2 | 52.0 | 7.73e-07 |
| chr3.CM0241.540.r2.d |  | BGC0001316 | Other | 32.0 | 23.6 | 50.0 | 1.17e-06 |
| chr3.CM0241.880.r2.m |  | BGC0001316 | Other | 33.0 | 23.2 | 48.0 | 1.26e-06 |
