# Supplementary material for: Uncovering the transcriptional landscape of Fomes fomentarius during fungal-based material production through gene co-expression network analysis
Source: Fungal Biol Biotechnol. 2025 Feb 13;12:1. doi: 10.1186/s40694-024-00192-3 (PMC11827164; doi:10.1186/s40694-024-00192-3)
Supplement: Supplementary file 1 — Supplementary Material 1 [file 40694_2024_192_MOESM1_ESM.zip › knownclusterblast/region3/jgi.p_Fomfom1_1193074_mibig_hits.html]

| MIBiG Protein | Description | MIBiG Cluster | MiBiG Product | % ID | % Coverage | BLAST Score | E-value |
| --- | --- | --- | --- | --- | --- | --- | --- |
| ACF70484.1 | oxidosqualene\_clavarinone\_cyclase | BGC0001248 | Terpene | 66.0 | 98.1 | 1024.0 | 0.0 |
| EAL89318.1 | squalene-hopene-cyclase,\_putative | BGC0000686 | Terpene | 44.0 | 96.0 | 624.0 | 8.41e-216 |
| KNA14396.1 | hypothetical\_protein | BGC0002402 | Terpene | 41.0 | 90.0 | 497.0 | 5.69e-166 |
| chr3.CM0292.40.r2.m |  | BGC0001317 | Terpene | 40.0 | 90.4 | 490.0 | 1.93e-163 |
| NP\_567462.1 | pentacyclic\_triterpene\_synthase\_1 | BGC0001313 | Terpene | 38.0 | 93.1 | 461.0 | 3.41e-152 |
| XP\_015166195.1 | LOW\_QUALITY\_PROTEIN:\_beta-amyrin\_synthase-like | BGC0002722 | Alkaloid+Terpene+Saccharide | 40.0 | 82.4 | 452.0 | 2.29e-149 |
| NP\_001329547.1 | baruol\_synthase\_1 | BGC0001313 | Terpene | 37.0 | 92.6 | 450.0 | 5.37e-148 |
| NP\_001318680.1 | putative\_pentacyclic\_triterpene\_synthase\_3 | BGC0001314 | Terpene | 37.0 | 91.2 | 450.0 | 6.02e-148 |
| NP\_193272.1 | baruol\_synthase\_1 | BGC0001313 | Terpene | 37.0 | 92.8 | 449.0 | 2.31e-147 |
| NP\_001078733.1 | thalianol\_synthase\_1 | BGC0000670 | Terpene | 37.0 | 91.7 | 448.0 | 5.59e-147 |
| NP\_199612.3 | thalianol\_synthase\_1 | BGC0000670 | Terpene | 37.0 | 91.7 | 435.0 | 3.62e-142 |
| NP\_199074.1 | marneral\_synthase | BGC0000669 | Terpene | 36.0 | 90.9 | 415.0 | 2.9e-134 |
| QIJ55911.1 | squalene--hopene\_cyclase | BGC0002449 | Other | 33.0 | 90.1 | 375.0 | 1.91e-115 |
| NP\_001328610.1 | pentacyclic\_triterpene\_synthase\_1 | BGC0001313 | Terpene | 37.0 | 66.9 | 315.0 | 2.66e-98 |
| AXA20105.1 | squalene--hopene\_cyclase | BGC0001646 | NRP+Polyketide | 22.0 | 94.5 | 129.0 | 1.33e-30 |
| EDP50814.1 | squalene-hopene-cyclase,\_putative | BGC0002173 | Terpene+Saccharide | 23.0 | 86.2 | 119.0 | 1.93e-27 |
