# Supplementary material for: Uncovering the transcriptional landscape of Fomes fomentarius during fungal-based material production through gene co-expression network analysis
Source: Fungal Biol Biotechnol. 2025 Feb 13;12:1. doi: 10.1186/s40694-024-00192-3 (PMC11827164; doi:10.1186/s40694-024-00192-3)
Supplement: Supplementary file 1 — Supplementary Material 1 [file 40694_2024_192_MOESM1_ESM.zip › knownclusterblast/region3/jgi.p_Fomfom1_1366674_mibig_hits.html]

| MIBiG Protein | Description | MIBiG Cluster | MiBiG Product | % ID | % Coverage | BLAST Score | E-value |
| --- | --- | --- | --- | --- | --- | --- | --- |
| AAM54105.1 | glucose\_1-dehydrogenase | BGC0000020 | Polyketide | 28.0 | 65.5 | 69.0 | 9.63e-13 |
| AQZ37116.1 | 3-oxoacyl-[acyl-carrier\_protein]\_reductase | BGC0001511 | Polyketide | 28.0 | 65.5 | 67.0 | 3.15e-12 |
| AKT74271.1 | TxnP4 | BGC0002141 | Polyketide | 32.0 | 62.8 | 56.0 | 9.77e-09 |
| BAF85845.1 | putative\_oxidoreductase | BGC0000109 | Polyketide | 27.0 | 72.3 | 51.0 | 5.84e-07 |
