# Supplementary material for: Uncovering the transcriptional landscape of Fomes fomentarius during fungal-based material production through gene co-expression network analysis
Source: Fungal Biol Biotechnol. 2025 Feb 13;12:1. doi: 10.1186/s40694-024-00192-3 (PMC11827164; doi:10.1186/s40694-024-00192-3)
Supplement: Supplementary file 1 — Supplementary Material 1 [file 40694_2024_192_MOESM1_ESM.zip › knownclusterblast/region3/jgi.p_Fomfom1_1196561_mibig_hits.html]

| MIBiG Protein | Description | MIBiG Cluster | MiBiG Product | % ID | % Coverage | BLAST Score | E-value |
| --- | --- | --- | --- | --- | --- | --- | --- |
| EWG54260.1 | homoserine\_O-acetyltransferase | BGC0001190 | Polyketide | 48.0 | 90.8 | 365.0 | 1.13e-122 |
| BAI70379.1 | homoserine\_O-acetyltransferase | BGC0000896 | Other | 28.0 | 90.1 | 172.0 | 6.21e-49 |
