# Supplementary material for: Uncovering the transcriptional landscape of Fomes fomentarius during fungal-based material production through gene co-expression network analysis
Source: Fungal Biol Biotechnol. 2025 Feb 13;12:1. doi: 10.1186/s40694-024-00192-3 (PMC11827164; doi:10.1186/s40694-024-00192-3)
Supplement: Supplementary file 1 — Supplementary Material 1 [file 40694_2024_192_MOESM1_ESM.zip › knownclusterblast/region3/jgi.p_Fomfom1_1196827_mibig_hits.html]

| MIBiG Protein | Description | MIBiG Cluster | MiBiG Product | % ID | % Coverage | BLAST Score | E-value |
| --- | --- | --- | --- | --- | --- | --- | --- |
| EIW83595.1 | terpene\_synthase | BGC0002708 | Terpene | 38.0 | 96.8 | 226.0 | 1.53e-71 |
| EIW83693.1 | terpenoid\_synthase | BGC0002707 | Terpene | 37.0 | 96.2 | 220.0 | 4.27e-69 |
| QDO73502.1 | PeniA | BGC0002557 | Terpene | 26.0 | 92.7 | 123.0 | 6.58e-32 |
