# Supplementary material for: Uncovering the transcriptional landscape of Fomes fomentarius during fungal-based material production through gene co-expression network analysis
Source: Fungal Biol Biotechnol. 2025 Feb 13;12:1. doi: 10.1186/s40694-024-00192-3 (PMC11827164; doi:10.1186/s40694-024-00192-3)
Supplement: Supplementary file 1 — Supplementary Material 1 [file 40694_2024_192_MOESM1_ESM.zip › knownclusterblast/region3/jgi.p_Fomfom1_639831_mibig_hits.html]

| MIBiG Protein | Description | MIBiG Cluster | MiBiG Product | % ID | % Coverage | BLAST Score | E-value |
| --- | --- | --- | --- | --- | --- | --- | --- |
| EIW83693.1 | terpenoid\_synthase | BGC0002707 | Terpene | 36.0 | 93.7 | 206.0 | 8.73e-64 |
| QJQ03973.1 | Pro1 | BGC0002445 | Terpene | 31.0 | 97.4 | 182.0 | 1.65e-54 |
| QDO73502.1 | PeniA | BGC0002557 | Terpene | 27.0 | 95.4 | 129.0 | 3.96e-34 |
| EDY49122.1 | terpene\_synthase | BGC0000674 | Terpene | 26.0 | 90.8 | 100.0 | 1.83e-23 |
