# Supplementary material for: Uncovering the transcriptional landscape of Fomes fomentarius during fungal-based material production through gene co-expression network analysis
Source: Fungal Biol Biotechnol. 2025 Feb 13;12:1. doi: 10.1186/s40694-024-00192-3 (PMC11827164; doi:10.1186/s40694-024-00192-3)
Supplement: Supplementary file 1 — Supplementary Material 1 [file 40694_2024_192_MOESM1_ESM.zip › knownclusterblast/region3/jgi.p_Fomfom1_1423974_mibig_hits.html]

| MIBiG Protein | Description | MIBiG Cluster | MiBiG Product | % ID | % Coverage | BLAST Score | E-value |
| --- | --- | --- | --- | --- | --- | --- | --- |
| EAU38965.1 | predicted\_protein | BGC0001122 | NRP+Polyketide:Iterative type I polyketide | 30.0 | 91.0 | 339.0 | 7.73e-103 |
| KIF57409.1 | DNA\_mismatch\_repair\_protein\_MutS | BGC0001879 | Other | 24.0 | 62.0 | 129.0 | 2.66e-30 |
| XP\_011392759.1 | uncharacterized\_protein | BGC0001281 | Polyketide | 22.0 | 91.1 | 122.0 | 6.73e-28 |
