# Supplementary material for: Uncovering the transcriptional landscape of Fomes fomentarius during fungal-based material production through gene co-expression network analysis
Source: Fungal Biol Biotechnol. 2025 Feb 13;12:1. doi: 10.1186/s40694-024-00192-3 (PMC11827164; doi:10.1186/s40694-024-00192-3)
Supplement: Supplementary file 1 — Supplementary Material 1 [file 40694_2024_192_MOESM1_ESM.zip › knownclusterblast/region3/jgi.p_Fomfom1_1275688_mibig_hits.html]

| MIBiG Protein | Description | MIBiG Cluster | MiBiG Product | % ID | % Coverage | BLAST Score | E-value |
| --- | --- | --- | --- | --- | --- | --- | --- |
| NP\_001314933.1 | uncharacterized\_protein\_LOC100382880 | BGC0002390 | Terpene | 41.0 | 56.3 | 197.0 | 2.08e-55 |
| ACR33036.1 | ATPase\_AAA | BGC0000495 | RiPP:Lanthipeptide | 28.0 | 49.3 | 77.0 | 1.32e-14 |
