# Supplementary material for: Uncovering the transcriptional landscape of Fomes fomentarius during fungal-based material production through gene co-expression network analysis
Source: Fungal Biol Biotechnol. 2025 Feb 13;12:1. doi: 10.1186/s40694-024-00192-3 (PMC11827164; doi:10.1186/s40694-024-00192-3)
Supplement: Supplementary file 1 — Supplementary Material 1 [file 40694_2024_192_MOESM1_ESM.zip › knownclusterblast/region3/jgi.p_Fomfom1_1319469_mibig_hits.html]

| MIBiG Protein | Description | MIBiG Cluster | MiBiG Product | % ID | % Coverage | BLAST Score | E-value |
| --- | --- | --- | --- | --- | --- | --- | --- |
| EWG54271.1 | hypothetical\_protein | BGC0001190 | Polyketide | 48.0 | 97.7 | 236.0 | 4.03e-77 |
| AFV52183.1 | aldolase | BGC0000081 | NRP+Polyketide:Iterative type I polyketide+Polyketide:Enediyne type I polyketide | 52.0 | 78.5 | 214.0 | 2.28e-69 |
| CEG06582.1 | Ribulose\_5-phosphate\_4\_epimerase | BGC0001928 | Other | 47.0 | 81.5 | 188.0 | 1.12e-58 |
| ADY16691.1 | TqaM | BGC0001142 | NRP | 41.0 | 79.2 | 164.0 | 5.1e-49 |
