# Supplementary material for: Uncovering the transcriptional landscape of Fomes fomentarius during fungal-based material production through gene co-expression network analysis
Source: Fungal Biol Biotechnol. 2025 Feb 13;12:1. doi: 10.1186/s40694-024-00192-3 (PMC11827164; doi:10.1186/s40694-024-00192-3)
Supplement: Supplementary file 1 — Supplementary Material 1 [file 40694_2024_192_MOESM1_ESM.zip › knownclusterblast/region3/jgi.p_Fomfom1_1295759_mibig_hits.html]

| MIBiG Protein | Description | MIBiG Cluster | MiBiG Product | % ID | % Coverage | BLAST Score | E-value |
| --- | --- | --- | --- | --- | --- | --- | --- |
| ADG29297.1 | putative\_dTDP-4-dehydrorhamnose\_reductase | BGC0000796 | Saccharide | 34.0 | 82.0 | 115.0 | 3.69e-30 |
| EDN75898.1 | dTDP-4-dehydrorhamnose\_reductase | BGC0002302 | Saccharide | 35.0 | 71.3 | 104.0 | 5.09e-26 |
| AAN65241.1 | dTDP-4-keto-6-deoxyhexose\_reductase | BGC0000832 | Saccharide:Hybrid/tailoring saccharide+Other:Aminocoumarin | 32.0 | 85.0 | 101.0 | 7.88e-25 |
| AAF67512.1 | dTDP-4-keto-6-deoxyhexose\_reductase | BGC0000834 | Saccharide:Hybrid/tailoring saccharide+Other:Aminocoumarin | 30.0 | 99.0 | 98.0 | 2.07e-23 |
| ABM21445.1 | sugar\_biosynthesis\_protein | BGC0000767 | Saccharide | 33.0 | 73.7 | 97.0 | 7.34e-23 |
| ABM21422.1 | sugar\_biosynthesis\_protein | BGC0000766 | Saccharide | 33.0 | 73.7 | 96.0 | 2.66e-22 |
| CAB65207.1 | RmlD\_protein | BGC0000775 | Saccharide | 29.0 | 101.0 | 92.0 | 2.95e-21 |
| AAR99613.1 | dTDP-4-dehydrorhamnose\_reductase | BGC0000794 | Saccharide | 31.0 | 71.7 | 88.0 | 6.13e-20 |
| AAC35923.1 | putative\_dTDP-4-keto-L-rhamnose\_reductase | BGC0000792 | Saccharide | 28.0 | 94.3 | 79.0 | 1.14e-16 |
| AXH03896.1 | NAD(P)-dependent\_oxidoreductase | BGC0001894 | Saccharide | 28.0 | 94.0 | 73.0 | 1.35e-14 |
| CBH32105.1 | putative\_NDP-hexose\_4-ketoreductase | BGC0000211 | Polyketide | 29.0 | 54.7 | 73.0 | 1.69e-14 |
| AXH03906.1 | dTDP-4-dehydrorhamnose\_reductase | BGC0001894 | Saccharide | 32.0 | 68.3 | 72.0 | 5.07e-14 |
| BAV57070.1 | NAD-dependent\_epimerase/dehydratase | BGC0001514 | Other | 28.0 | 54.0 | 55.0 | 2.45e-08 |
