# Supplementary material for: Uncovering the transcriptional landscape of Fomes fomentarius during fungal-based material production through gene co-expression network analysis
Source: Fungal Biol Biotechnol. 2025 Feb 13;12:1. doi: 10.1186/s40694-024-00192-3 (PMC11827164; doi:10.1186/s40694-024-00192-3)
Supplement: Supplementary file 1 — Supplementary Material 1 [file 40694_2024_192_MOESM1_ESM.zip › knownclusterblast/region3/jgi.p_Fomfom1_1208090_mibig_hits.html]

| MIBiG Protein | Description | MIBiG Cluster | MiBiG Product | % ID | % Coverage | BLAST Score | E-value |
| --- | --- | --- | --- | --- | --- | --- | --- |
| ABX02675.1 | serine/threonine\_protein\_kinase | BGC0001521 | NRP | 26.0 | 28.9 | 77.0 | 6.02e-14 |
| CAF05640.1 | hypothetical\_protein | BGC0001053 | NRP+Polyketide | 29.0 | 27.9 | 52.0 | 3.11e-06 |
