# Supplementary material for: Uncovering the transcriptional landscape of Fomes fomentarius during fungal-based material production through gene co-expression network analysis
Source: Fungal Biol Biotechnol. 2025 Feb 13;12:1. doi: 10.1186/s40694-024-00192-3 (PMC11827164; doi:10.1186/s40694-024-00192-3)
Supplement: Supplementary file 1 — Supplementary Material 1 [file 40694_2024_192_MOESM1_ESM.zip › knownclusterblast/region3/jgi.p_Fomfom1_1388504_mibig_hits.html]

| MIBiG Protein | Description | MIBiG Cluster | MiBiG Product | % ID | % Coverage | BLAST Score | E-value |
| --- | --- | --- | --- | --- | --- | --- | --- |
| ESK96610.1 | hypothetical\_protein | BGC0002212 | Polyketide | 28.0 | 101.4 | 348.0 | 1.45e-102 |
| ASK38699.1 | putative\_nonribosomal\_peptide\_synthetase-like\_protein | BGC0001436 | Polyketide:Iterative type I polyketide | 31.0 | 76.1 | 305.0 | 3.89e-87 |
| EAU35432.1 | predicted\_protein | BGC0002734 | Polyketide | 26.0 | 103.0 | 280.0 | 1.29e-78 |
| KFA69336.1 | hypothetical\_protein | BGC0001626 | Polyketide | 27.0 | 90.0 | 271.0 | 2.03e-75 |
| EWG54274.1 | hypothetical\_protein | BGC0001190 | Polyketide | 27.0 | 83.3 | 269.0 | 1.03e-74 |
| BAV19380.1 | NRPS-like\_enzyme | BGC0001390 | NRP+Polyketide | 25.0 | 99.3 | 261.0 | 3.09e-72 |
| AWM95789.1 | non-reduciing\_polyketide\_synthase\_methylorcinaldehyde\_synthase | BGC0001827 | Polyketide | 30.0 | 36.3 | 139.0 | 7.39e-33 |
| CAP95404.1 |  | BGC0001404 | Polyketide | 29.0 | 36.2 | 132.0 | 1.68e-30 |
| ATY72525.1 | non-ribosomal\_peptide\_synthetase | BGC0001574 | NRP | 26.0 | 57.2 | 122.0 | 8.9e-28 |
| AUW31047.1 | PKS-like\_protein | BGC0002483 | Polyketide | 28.0 | 31.8 | 112.0 | 1.79e-26 |
| AEA29644.1 | putative\_nonribosomal\_peptide\_synthetase\_and\_kinurenine\_monooxygenase | BGC0000409 | NRP | 28.0 | 43.9 | 112.0 | 1.2e-24 |
| QHD43130.1 | NRPS/PKS\_hybrid\_protein | BGC0002546 | NRP+Polyketide | 23.0 | 30.2 | 60.0 | 1.68e-08 |
| XP\_001220460.1 | uncharacterized\_protein | BGC0001182 | NRP+Polyketide:Iterative type I polyketide | 25.0 | 24.0 | 60.0 | 1.69e-08 |
| AGO86662.1 | equisetin\_synthetase | BGC0001255 | NRP+Polyketide | 28.0 | 22.2 | 52.0 | 4.82e-06 |
