# Supplementary material for: Uncovering the transcriptional landscape of Fomes fomentarius during fungal-based material production through gene co-expression network analysis
Source: Fungal Biol Biotechnol. 2025 Feb 13;12:1. doi: 10.1186/s40694-024-00192-3 (PMC11827164; doi:10.1186/s40694-024-00192-3)
Supplement: Supplementary file 1 — Supplementary Material 1 [file 40694_2024_192_MOESM1_ESM.zip › knownclusterblast/region3/jgi.p_Fomfom1_1373552_mibig_hits.html]

| MIBiG Protein | Description | MIBiG Cluster | MiBiG Product | % ID | % Coverage | BLAST Score | E-value |
| --- | --- | --- | --- | --- | --- | --- | --- |
| ESK96610.1 | hypothetical\_protein | BGC0002212 | Polyketide | 27.0 | 103.3 | 307.0 | 4.46e-88 |
| ASK38699.1 | putative\_nonribosomal\_peptide\_synthetase-like\_protein | BGC0001436 | Polyketide:Iterative type I polyketide | 27.0 | 102.3 | 301.0 | 3.16e-86 |
| EAU35432.1 | predicted\_protein | BGC0002734 | Polyketide | 25.0 | 94.6 | 284.0 | 3.27e-80 |
| KIA75587.1 | NRPS-like\_enzyme | BGC0002209 | Polyketide | 25.0 | 108.0 | 261.0 | 5.78e-72 |
| EWG54274.1 | hypothetical\_protein | BGC0001190 | Polyketide | 26.0 | 100.7 | 253.0 | 1.89e-69 |
| CEF75881.1 |  | BGC0001600 | Polyketide | 25.0 | 106.0 | 248.0 | 6.55e-68 |
| AMJ52084.1 | lijE | BGC0002255 | Polyketide | 30.0 | 41.7 | 158.0 | 1.33e-38 |
| QBK15044.1 | clavatol\_synthase\_ClaF | BGC0002196 | Polyketide | 30.0 | 35.5 | 149.0 | 7.07e-36 |
| EAU31923.1 | hypothetical\_protein | BGC0002267 | Polyketide | 29.0 | 35.7 | 138.0 | 1.96e-32 |
| EAA65602.1 | hypothetical\_protein | BGC0000022 | Polyketide | 27.0 | 34.4 | 120.0 | 6.98e-27 |
| AEA29644.1 | putative\_nonribosomal\_peptide\_synthetase\_and\_kinurenine\_monooxygenase | BGC0000409 | NRP | 24.0 | 41.8 | 79.0 | 1.95e-14 |
| ABW71853.1 | nonribosomal\_peptide\_synthetase | BGC0000303 | NRP | 24.0 | 36.6 | 69.0 | 2.09e-11 |
