# Supplementary material for: Uncovering the transcriptional landscape of Fomes fomentarius during fungal-based material production through gene co-expression network analysis
Source: Fungal Biol Biotechnol. 2025 Feb 13;12:1. doi: 10.1186/s40694-024-00192-3 (PMC11827164; doi:10.1186/s40694-024-00192-3)
Supplement: Supplementary file 1 — Supplementary Material 1 [file 40694_2024_192_MOESM1_ESM.zip › knownclusterblast/region3/jgi.p_Fomfom1_324375_mibig_hits.html]

| MIBiG Protein | Description | MIBiG Cluster | MiBiG Product | % ID | % Coverage | BLAST Score | E-value |
| --- | --- | --- | --- | --- | --- | --- | --- |
| ASK38699.1 | putative\_nonribosomal\_peptide\_synthetase-like\_protein | BGC0001436 | Polyketide:Iterative type I polyketide | 27.0 | 100.3 | 307.0 | 5.89e-88 |
| EAU35432.1 | predicted\_protein | BGC0002734 | Polyketide | 26.0 | 103.1 | 295.0 | 1.11e-83 |
| ESK96610.1 | hypothetical\_protein | BGC0002212 | Polyketide | 28.0 | 100.8 | 293.0 | 6.39e-83 |
| KIA75587.1 | NRPS-like\_enzyme | BGC0002209 | Polyketide | 25.0 | 106.4 | 244.0 | 1.86e-66 |
| EWG54274.1 | hypothetical\_protein | BGC0001190 | Polyketide | 26.0 | 88.9 | 225.0 | 3.62e-60 |
| AUW31047.1 | PKS-like\_protein | BGC0002483 | Polyketide | 30.0 | 31.0 | 119.0 | 5.62e-29 |
