# Supplementary material for: Uncovering the transcriptional landscape of Fomes fomentarius during fungal-based material production through gene co-expression network analysis
Source: Fungal Biol Biotechnol. 2025 Feb 13;12:1. doi: 10.1186/s40694-024-00192-3 (PMC11827164; doi:10.1186/s40694-024-00192-3)
Supplement: Supplementary file 1 — Supplementary Material 1 [file 40694_2024_192_MOESM1_ESM.zip › knownclusterblast/region3/jgi.p_Fomfom1_324542_mibig_hits.html]

| MIBiG Protein | Description | MIBiG Cluster | MiBiG Product | % ID | % Coverage | BLAST Score | E-value |
| --- | --- | --- | --- | --- | --- | --- | --- |
| ESK96610.1 | hypothetical\_protein | BGC0002212 | Polyketide | 28.0 | 103.0 | 339.0 | 1.98e-99 |
| ASK38699.1 | putative\_nonribosomal\_peptide\_synthetase-like\_protein | BGC0001436 | Polyketide:Iterative type I polyketide | 27.0 | 99.6 | 323.0 | 7.76e-94 |
| EAU35432.1 | predicted\_protein | BGC0002734 | Polyketide | 27.0 | 102.3 | 303.0 | 1.17e-86 |
| KFA69336.1 | hypothetical\_protein | BGC0001626 | Polyketide | 29.0 | 92.2 | 296.0 | 2.54e-84 |
| KIA75587.1 | NRPS-like\_enzyme | BGC0002209 | Polyketide | 26.0 | 106.2 | 268.0 | 1.88e-74 |
| BAV19380.1 | NRPS-like\_enzyme | BGC0001390 | NRP+Polyketide | 26.0 | 101.1 | 267.0 | 3.54e-74 |
| CEF75881.1 |  | BGC0001600 | Polyketide | 26.0 | 103.9 | 263.0 | 1.36e-72 |
| EWG54274.1 | hypothetical\_protein | BGC0001190 | Polyketide | 25.0 | 94.4 | 259.0 | 1.99e-71 |
| AUW31047.1 | PKS-like\_protein | BGC0002483 | Polyketide | 31.0 | 31.1 | 134.0 | 5.58e-34 |
