# Supplementary material for: Uncovering the transcriptional landscape of Fomes fomentarius during fungal-based material production through gene co-expression network analysis
Source: Fungal Biol Biotechnol. 2025 Feb 13;12:1. doi: 10.1186/s40694-024-00192-3 (PMC11827164; doi:10.1186/s40694-024-00192-3)
Supplement: Supplementary file 1 — Supplementary Material 1 [file 40694_2024_192_MOESM1_ESM.zip › knownclusterblast/region4/jgi.p_Fomfom1_1322654_mibig_hits.html]

| MIBiG Protein | Description | MIBiG Cluster | MiBiG Product | % ID | % Coverage | BLAST Score | E-value |
| --- | --- | --- | --- | --- | --- | --- | --- |
| QPP19364.1 | Pen5 | BGC0002501 | Alkaloid | 73.0 | 93.7 | 697.0 | 8.73e-251 |
| EAU29807.1 | ATP\_synthase\_beta\_chain,\_mitochondrial\_precursor | BGC0001400 | Polyketide | 78.0 | 75.0 | 590.0 | 3.28e-210 |
