# Supplementary material for: Uncovering the transcriptional landscape of Fomes fomentarius during fungal-based material production through gene co-expression network analysis
Source: Fungal Biol Biotechnol. 2025 Feb 13;12:1. doi: 10.1186/s40694-024-00192-3 (PMC11827164; doi:10.1186/s40694-024-00192-3)
Supplement: Supplementary file 1 — Supplementary Material 1 [file 40694_2024_192_MOESM1_ESM.zip › knownclusterblast/region4/jgi.p_Fomfom1_1190301_mibig_hits.html]

| MIBiG Protein | Description | MIBiG Cluster | MiBiG Product | % ID | % Coverage | BLAST Score | E-value |
| --- | --- | --- | --- | --- | --- | --- | --- |
| EIW83693.1 | terpenoid\_synthase | BGC0002707 | Terpene | 49.0 | 90.1 | 317.0 | 4.1e-106 |
| EIW83595.1 | terpene\_synthase | BGC0002708 | Terpene | 47.0 | 90.4 | 314.0 | 6.07e-105 |
