# Supplementary material for: Uncovering the transcriptional landscape of Fomes fomentarius during fungal-based material production through gene co-expression network analysis
Source: Fungal Biol Biotechnol. 2025 Feb 13;12:1. doi: 10.1186/s40694-024-00192-3 (PMC11827164; doi:10.1186/s40694-024-00192-3)
Supplement: Supplementary file 1 — Supplementary Material 1 [file 40694_2024_192_MOESM1_ESM.zip › knownclusterblast/region4/jgi.p_Fomfom1_1216621_mibig_hits.html]

| MIBiG Protein | Description | MIBiG Cluster | MiBiG Product | % ID | % Coverage | BLAST Score | E-value |
| --- | --- | --- | --- | --- | --- | --- | --- |
| AZL87943.1 | GA4\_family\_aspergillic\_acid\_hydroxylase\_(AsaB) | BGC0002602 | Alkaloid | 35.0 | 102.8 | 166.0 | 1.22e-49 |
| PKX88479.1 | methyltransferase | BGC0001708 | Polyketide+Terpene | 36.0 | 89.8 | 139.0 | 1.8e-39 |
| QVK45115.1 | hypothetical\_protein | BGC0002438 | Alkaloid | 37.0 | 86.3 | 132.0 | 1.76e-36 |
| EDY47110.1 | 7-alpha-cephem-methoxylase\_P8\_chain | BGC0000319 | NRP:Beta-lactam | 32.0 | 101.8 | 124.0 | 2.7e-33 |
| BAE56599.1 |  | BGC0001123 | NRP | 44.0 | 49.1 | 114.0 | 3.4e-30 |
| ANV81295.1 | GA4\_desaturase | BGC0001604 | Terpene | 33.0 | 48.4 | 55.0 | 2.4e-08 |
| XP\_002373812.1 | GA4\_desaturase\_family\_protein | BGC0001516 | NRP | 36.0 | 26.3 | 50.0 | 8.34e-07 |
