# Supplementary material for: Uncovering the transcriptional landscape of Fomes fomentarius during fungal-based material production through gene co-expression network analysis
Source: Fungal Biol Biotechnol. 2025 Feb 13;12:1. doi: 10.1186/s40694-024-00192-3 (PMC11827164; doi:10.1186/s40694-024-00192-3)
Supplement: Supplementary file 1 — Supplementary Material 1 [file 40694_2024_192_MOESM1_ESM.zip › knownclusterblast/region4/jgi.p_Fomfom1_1315830_mibig_hits.html]

| MIBiG Protein | Description | MIBiG Cluster | MiBiG Product | % ID | % Coverage | BLAST Score | E-value |
| --- | --- | --- | --- | --- | --- | --- | --- |
| XP\_011392748.1 | hexadecenal\_dehydrogenase | BGC0001281 | Polyketide | 41.0 | 91.1 | 390.0 | 8.7e-131 |
| AXM43059.1 | aldehyde\_dehydrogenase | BGC0001945 | NRP | 36.0 | 83.7 | 302.0 | 5.08e-97 |
| AAL15593.1 | Sim15 | BGC0000270 | Polyketide | 37.0 | 84.7 | 280.0 | 5.91e-89 |
| AAK06797.1 | putative\_alcohol\_dehydrogenase\_SimC5 | BGC0001072 | Saccharide+Polyketide:Modular type I polyketide+Polyketide:Type II polyketide+Other:Aminocoumarin | 37.0 | 84.7 | 280.0 | 1.17e-88 |
| WP\_033283297.1 | aldehyde\_dehydrogenase | BGC0001739 | Other | 32.0 | 66.0 | 127.0 | 1.6e-31 |
| AEF33093.1 | putative\_aldehyde\_dehydrogenase | BGC0001039 | NRP+Polyketide | 25.0 | 84.7 | 126.0 | 6.23e-31 |
| BAK64640.1 | putative\_aldehyde\_dehydrogenase | BGC0000135 | Polyketide | 31.0 | 64.1 | 119.0 | 1.76e-28 |
| QEG98940.1 | ant3 | BGC0002042 | NRP | 26.0 | 75.5 | 117.0 | 4.65e-28 |
| WP\_030845402.1 | aldehyde\_dehydrogenase\_family\_protein | BGC0001368 | NRP | 28.0 | 84.7 | 110.0 | 1.53e-25 |
| BAV57467.1 | aldehyde\_dehydrogenase | BGC0001818 | NRP | 25.0 | 91.3 | 108.0 | 5.9e-25 |
| ACI30658.1 | methylmalonate\_semialdehyde\_dehydrogenase | BGC0000313 | NRP | 25.0 | 77.9 | 107.0 | 1.82e-24 |
| BAP16694.1 | aldehyde\_dehydrogenase | BGC0000376 | NRP | 24.0 | 77.7 | 102.0 | 9.21e-23 |
| CAJ14050.1 | putative\_aldehyde\_dehydrogenase | BGC0000406 | NRP | 28.0 | 63.1 | 97.0 | 2.65e-21 |
| ADC45553.1 | aldehyde\_dehydrogenase | BGC0000093 | Polyketide | 28.0 | 51.7 | 57.0 | 3.82e-08 |
