# Supplementary figures and images for: Uncovering the transcriptional landscape of Fomes fomentarius during fungal-based material production through gene co-expression network analysis
Source: Fungal Biol Biotechnol. 2025 Feb 13;12:1. doi: 10.1186/s40694-024-00192-3 (PMC11827164; doi:10.1186/s40694-024-00192-3)

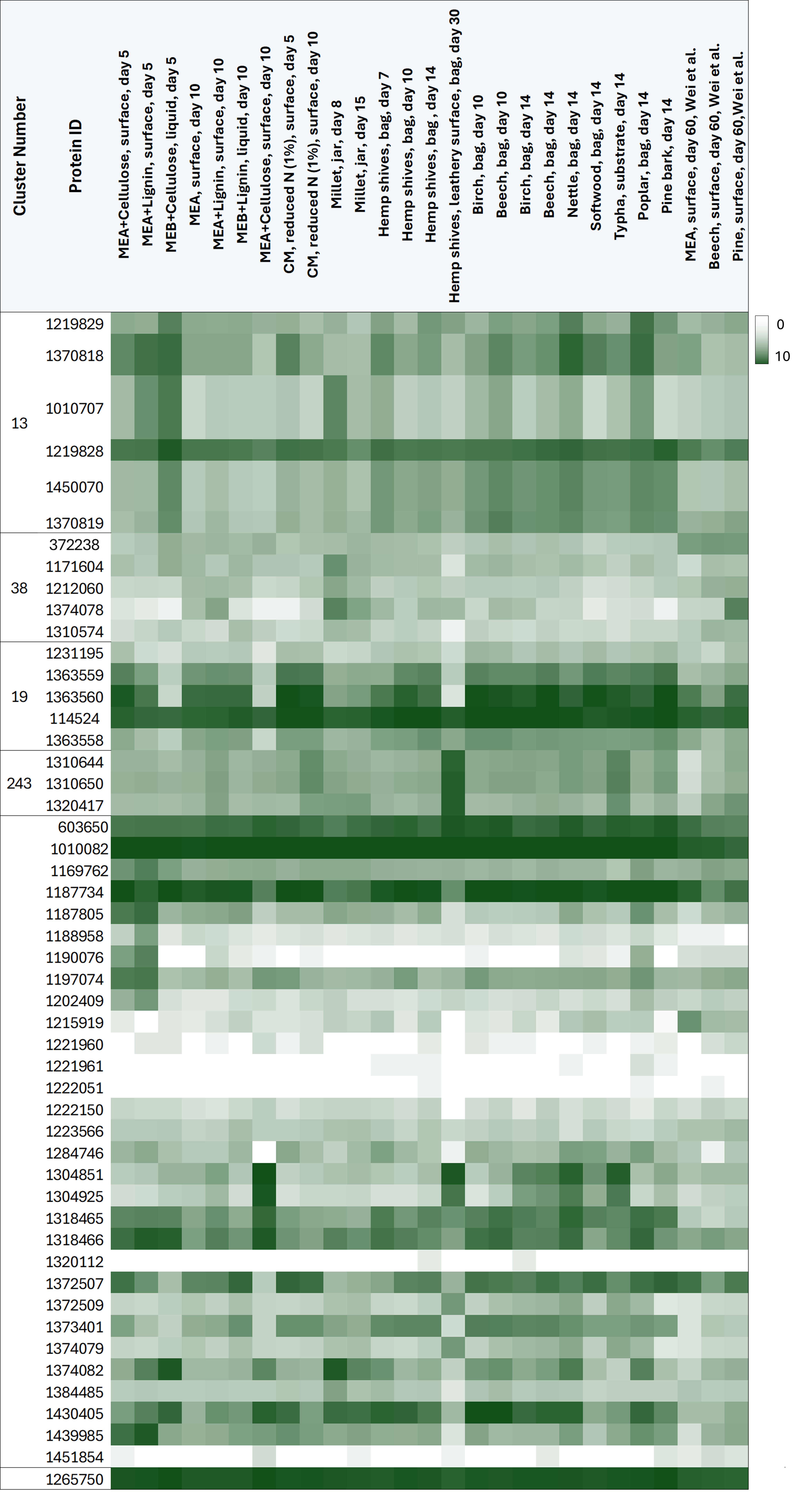

Supplement: Supplementary file 8 — Supplementary Table 1 [file 40694_2024_192_MOESM8_ESM.png]
