# Supplementary material for: Uncovering the transcriptional landscape of Fomes fomentarius during fungal-based material production through gene co-expression network analysis
Source: Fungal Biol Biotechnol. 2025 Feb 13;12:1. doi: 10.1186/s40694-024-00192-3 (PMC11827164; doi:10.1186/s40694-024-00192-3)
Supplement: Supplementary file 1 — Supplementary Material 1 [file 40694_2024_192_MOESM1_ESM.zip › knownclusterblast/region4/jgi.p_Fomfom1_1216625_mibig_hits.html]

| MIBiG Protein | Description | MIBiG Cluster | MiBiG Product | % ID | % Coverage | BLAST Score | E-value |
| --- | --- | --- | --- | --- | --- | --- | --- |
| XP\_011392748.1 | hexadecenal\_dehydrogenase | BGC0001281 | Polyketide | 34.0 | 94.5 | 281.0 | 2.28e-88 |
| AAL15593.1 | Sim15 | BGC0000270 | Polyketide | 34.0 | 86.1 | 222.0 | 2.14e-66 |
| AXM43059.1 | aldehyde\_dehydrogenase | BGC0001945 | NRP | 31.0 | 87.2 | 222.0 | 2.65e-66 |
| AAK06797.1 | putative\_alcohol\_dehydrogenase\_SimC5 | BGC0001072 | Saccharide+Polyketide:Modular type I polyketide+Polyketide:Type II polyketide+Other:Aminocoumarin | 34.0 | 86.1 | 221.0 | 4.2e-66 |
| SKB24611.1 |  | BGC0002455 | Polyketide | 22.0 | 83.2 | 50.0 | 4.52e-06 |
