# Supplementary material for: Uncovering the transcriptional landscape of Fomes fomentarius during fungal-based material production through gene co-expression network analysis
Source: Fungal Biol Biotechnol. 2025 Feb 13;12:1. doi: 10.1186/s40694-024-00192-3 (PMC11827164; doi:10.1186/s40694-024-00192-3)
Supplement: Supplementary file 1 — Supplementary Material 1 [file 40694_2024_192_MOESM1_ESM.zip › knownclusterblast/region4/jgi.p_Fomfom1_1256164_mibig_hits.html]

| MIBiG Protein | Description | MIBiG Cluster | MiBiG Product | % ID | % Coverage | BLAST Score | E-value |
| --- | --- | --- | --- | --- | --- | --- | --- |
| BAQ25461.1 | putative\_dehydrogenase | BGC0001264 | Polyketide | 34.0 | 102.8 | 290.0 | 1.41e-89 |
| ACA34720.1 | CtnD | BGC0000894 | Other | 32.0 | 104.1 | 283.0 | 5.54e-87 |
| ALI92648.1 | CitC\_oxidoreductase | BGC0001338 | Polyketide:Iterative type I polyketide | 32.0 | 104.1 | 283.0 | 5.54e-87 |
| EHK18384.1 | hypothetical\_protein | BGC0002216 | Terpene | 31.0 | 100.7 | 254.0 | 3.3e-76 |
| BBD84647.1 | putative\_GMC\_oxidoreductase | BGC0001775 | Terpene | 31.0 | 99.8 | 243.0 | 3.79e-72 |
| BAE62226.1 |  | BGC0002237 | Polyketide | 31.0 | 98.7 | 234.0 | 1.29e-69 |
| CBF83141.1 | conserved\_hypothetical\_protein | BGC0001722 | Polyketide | 29.0 | 100.2 | 221.0 | 1.54e-63 |
| EIN09539.1 | pyranose\_dehydrogenase | BGC0002213 | Polyketide | 29.0 | 102.3 | 203.0 | 3.84e-57 |
| ATV82114.1 | GMC\_oxidoreductase/oxidase/dehydrogenase | BGC0001909 | Polyketide | 28.0 | 104.3 | 196.0 | 1.26e-54 |
| FAA01296.1 | glucose-methanol-choline\_family\_oxidoreductase\_PyvF | BGC0002210 | Polyketide+NRP | 25.0 | 99.0 | 184.0 | 3.07e-50 |
| KAF7526514.1 | hypothetical\_protein | BGC0002244 | Polyketide | 28.0 | 99.7 | 163.0 | 4.61e-43 |
| ctg1\_orf10 |  | BGC0000846 | Other | 33.0 | 72.6 | 152.0 | 1.09e-38 |
| EEP98515.1 | Glucose-methanol-choline\_oxidoreductase | BGC0002091 | NRP | 24.0 | 97.9 | 130.0 | 7.99e-32 |
| KDM89831.1 | glucose-methanol-choline\_oxidoreductase | BGC0002412 | NRP | 26.0 | 100.2 | 125.0 | 4.28e-30 |
| CBK62747.1 |  | BGC0001115 | NRP+Polyketide | 30.0 | 55.9 | 113.0 | 3.59e-26 |
| CAM56763.1 | hypothetical\_protein | BGC0000354 | NRP | 29.0 | 52.3 | 109.0 | 3.86e-25 |
| EIN09902.1 | FAD/NAD(P)-binding\_domain-containing\_protein | BGC0002220 | Terpene | 33.0 | 29.2 | 89.0 | 5.3e-19 |
| AJI44177.1 | glucose-methanol-choline\_oxidoreductase | BGC0001193 | NRP | 29.0 | 54.9 | 87.0 | 1.64e-17 |
