# Supplementary material for: Uncovering the transcriptional landscape of Fomes fomentarius during fungal-based material production through gene co-expression network analysis
Source: Fungal Biol Biotechnol. 2025 Feb 13;12:1. doi: 10.1186/s40694-024-00192-3 (PMC11827164; doi:10.1186/s40694-024-00192-3)
Supplement: Supplementary file 1 — Supplementary Material 1 [file 40694_2024_192_MOESM1_ESM.zip › knownclusterblast/region4/jgi.p_Fomfom1_1256193_mibig_hits.html]

| MIBiG Protein | Description | MIBiG Cluster | MiBiG Product | % ID | % Coverage | BLAST Score | E-value |
| --- | --- | --- | --- | --- | --- | --- | --- |
| QJD55556.1 | acetyl-CoA\_ligase | BGC0001909 | Polyketide | 40.0 | 103.5 | 404.0 | 8.52e-134 |
| EGX96626.1 | AMP\_dependent\_CoA\_ligase,\_putative | BGC0002259 | Polyketide+NRP | 33.0 | 102.3 | 286.0 | 4.02e-88 |
| CBF87076.1 | conserved\_hypothetical\_protein | BGC0001290 | NRP | 31.0 | 99.0 | 261.0 | 2.34e-79 |
| EJP62834.1 | 4-coumarate-CoA\_ligase | BGC0002203 | NRP+Polyketide+Other | 33.0 | 88.7 | 250.0 | 7.43e-76 |
| QIW91880.1 | Ligase | BGC0002543 | NRP | 30.0 | 100.2 | 249.0 | 7.04e-75 |
| XP\_020058103.1 | uncharacterized\_protein | BGC0001220 | NRP | 30.0 | 98.1 | 235.0 | 8.22e-70 |
| OAQ83766.1 | phenylacetyl-\_ligase\_protein | BGC0001358 | NRP+Polyketide | 39.0 | 63.8 | 229.0 | 2.29e-69 |
| AMM63166.1 | AniI | BGC0001371 | NRP | 30.0 | 98.6 | 231.0 | 2.3e-68 |
| AKJ70945.1 | AMP-dependent\_CoA\_ligase | BGC0002611 | NRP | 31.0 | 98.6 | 230.0 | 6.66e-68 |
| EPE34349.1 | acyl-AMP\_ligase | BGC0001035 | Polyketide+NRP | 29.0 | 99.1 | 230.0 | 1.06e-67 |
| QCC62990.1 | AMP\_dependent\_ligase | BGC0001966 | NRP+Polyketide | 31.0 | 95.5 | 212.0 | 6.54e-61 |
| EHA28233.1 | hypothetical\_protein | BGC0001143 | Polyketide | 30.0 | 98.8 | 210.0 | 1.04e-60 |
| CAH10130.1 | feruloyl-CoA\_synthetase | BGC0000268 | Polyketide | 30.0 | 84.3 | 208.0 | 5.25e-60 |
| EKJ70674.1 | hypothetical\_protein | BGC0002188 | NRP+Polyketide | 29.0 | 95.3 | 207.0 | 2.71e-59 |
| ACC80687.1 | AMP-dependent\_synthetase\_and\_ligase | BGC0002711 | Polyketide | 28.0 | 89.5 | 195.0 | 2.66e-55 |
| ABU70356.1 | hypothetical\_protein | BGC0001890 | NRP | 28.0 | 92.0 | 189.0 | 7.66e-53 |
| AYU66239.1 | TjhA5 | BGC0002461 | Polyketide | 30.0 | 84.5 | 182.0 | 2.14e-50 |
| QKW94281.1 | long-chain-fatty-acid--CoA\_ligase | BGC0002342 | NRP+Polyketide | 32.0 | 63.8 | 176.0 | 3.8e-48 |
| ABS74201.1 | YngI | BGC0001095 | NRP | 28.0 | 88.2 | 166.0 | 1.54e-44 |
| AGY30675.1 | Ann3 | BGC0001298 | Polyketide | 29.0 | 85.4 | 164.0 | 6.19e-44 |
| AXM42922.1 | AMP-dependent\_synthetase\_and\_ligase/heterocyclase | BGC0001940 | Polyketide | 27.0 | 86.1 | 165.0 | 2.2e-43 |
| BAE61264.1 |  | BGC0002238 | Polyketide | 29.0 | 89.2 | 163.0 | 2.39e-43 |
| OWA01623.1 | acyl--CoA\_ligase | BGC0001439 | Polyketide+Saccharide:Hybrid/tailoring saccharide | 29.0 | 82.6 | 163.0 | 2.43e-43 |
| AEI98652.1 | CtcI | BGC0000209 | Polyketide | 28.0 | 88.3 | 161.0 | 8.36e-43 |
| AFU65902.1 | DacH | BGC0000216 | Polyketide | 30.0 | 84.5 | 160.0 | 1.34e-42 |
| ADI58647.1 | 5-Aminolevulinate\_CoA\_ligase | BGC0000187 | Polyketide:Type II polyketide | 28.0 | 86.1 | 155.0 | 3.19e-41 |
| AAF08801.1 | YngI | BGC0001103 | NRP+Polyketide | 26.0 | 91.5 | 155.0 | 2.02e-40 |
| CAE17553.1 | acyl\_CoA\_ligase | BGC0000210 | Polyketide:Type II polyketide+Saccharide:Oligosaccharide | 30.0 | 65.0 | 154.0 | 3.9e-40 |
| AEW31026.1 | short-chain\_acyl-CoA\_synthetase | BGC0000407 | NRP | 26.0 | 92.9 | 153.0 | 7.05e-40 |
| BBA21084.1 | putative\_5-aminolevulinate\_CoA-ligase | BGC0001740 | NRP+Polyketide | 27.0 | 86.4 | 152.0 | 7.62e-40 |
| AGM05529.1 | long-chain\_acyl-CoA\_synthetase | BGC0002098 | Polyketide | 28.0 | 85.9 | 151.0 | 2.01e-39 |
| CAE02600.1 | p-nitrobenzoate\_CoA\_ligase | BGC0000024 | Polyketide:Modular type I polyketide | 27.0 | 93.7 | 149.0 | 1.21e-38 |
| AAX98210.1 | acyl\_CoA\_ligase | BGC0000052 | Polyketide | 28.0 | 86.4 | 149.0 | 1.67e-38 |
| QED90620.1 | AMP-dependent\_ligase | BGC0002081 | Polyketide | 27.0 | 91.5 | 150.0 | 1.72e-38 |
| CAK50779.1 | acyl\_CoA\_ligase | BGC0000247 | Polyketide:Type II polyketide+Saccharide:Oligosaccharide | 29.0 | 84.3 | 146.0 | 1.21e-37 |
| ACR50792.1 | putative\_acyl-CoA\_synthase | BGC0000163 | Polyketide | 27.0 | 96.9 | 146.0 | 1.79e-37 |
| DAC74137.1 | AMP-dependent\_synthetase | BGC0002019 | Terpene | 32.0 | 64.5 | 143.0 | 1.19e-36 |
| EFY99277.1 | long-chain-fatty-acid--CoA\_ligase | BGC0002710 | NRP | 26.0 | 92.5 | 144.0 | 1.58e-36 |
| ADC79613.1 | BafX | BGC0000028 | Polyketide:Modular type I polyketide | 27.0 | 85.7 | 142.0 | 5.9e-36 |
| QQZ01621.1 | long-chain\_fatty\_acid--CoA\_ligase | BGC0002497 | Other | 26.0 | 85.9 | 139.0 | 3.12e-35 |
| APZ78718.1 | benzoate-CoA\_ligase\_family\_protein | BGC0001420 | NRP:Cyclic depsipeptide+Polyketide:Iterative type I polyketide | 26.0 | 82.8 | 135.0 | 6.92e-34 |
| AQZ26587.1 | obafluorin\_dimodular\_nonribosomal\_peptide\_synthetase | BGC0001437 | NRP | 27.0 | 87.1 | 137.0 | 2.59e-33 |
| ABC34346.1 | acyl-CoA\_ligase | BGC0001102 | NRP+Polyketide:Modular type I polyketide+Polyketide:Trans-AT type I polyketide | 25.0 | 88.5 | 135.0 | 3.21e-33 |
| AIL50189.1 | putative\_5-aminolevulinate\_CoA-ligase | BGC0000213 | Polyketide:Type II polyketide | 27.0 | 85.5 | 133.0 | 5.66e-33 |
| NP\_626687.1 | acyl-CoA\_synthetase | BGC0000595 | RiPP | 30.0 | 65.2 | 127.0 | 3.94e-31 |
| WP\_003976362.1 | acyl-CoA\_synthetase | BGC0000596 | RiPP | 29.0 | 65.2 | 127.0 | 3.94e-31 |
| MBD2892711.1 | 3-methylmercaptopropionyl-CoA\_ligase | BGC0002718 | NRP | 28.0 | 88.0 | 127.0 | 7.3e-31 |
| ARM20260.1 | SauE | BGC0002591 | Polyketide+Terpene | 27.0 | 86.6 | 126.0 | 1.37e-30 |
| ADM46356.1 | polyketide\_synthase | BGC0000106 | Polyketide | 26.0 | 89.5 | 128.0 | 1.76e-30 |
| WP\_028847739.1 | AMP-binding\_protein | BGC0002467 | NRP | 24.0 | 97.0 | 125.0 | 3.19e-30 |
| QIC03945.1 | EncH | BGC0002366 | Polyketide | 25.0 | 96.7 | 124.0 | 9.61e-30 |
| AGL76720.1 | fatty\_acid\_CoA\_ligase | BGC0001137 | Alkaloid | 28.0 | 63.9 | 123.0 | 1.21e-29 |
| SSTG\_01180 | acyl-CoA\_synthetase | BGC0000597 | RiPP:Proteusin | 28.0 | 66.6 | 120.0 | 4.13e-29 |
| AEA35021.1 | hypothetical\_protein | BGC0002502 | Polyketide | 26.0 | 92.2 | 122.0 | 5.15e-29 |
| AAF81723.1 | putative\_acyl-CoA\_ligase\_EncH | BGC0000220 | Polyketide:Type II polyketide | 26.0 | 86.4 | 120.0 | 1.87e-28 |
| QBC75017.1 | acyl-CoA\_synthase | BGC0001968 | NRP | 29.0 | 73.9 | 120.0 | 1.91e-28 |
| BCK51658.1 | putative\_acyl-CoA\_synthetase | BGC0002520 | Polyketide | 30.0 | 63.1 | 119.0 | 2.54e-28 |
| AQZ37113.1 | polyketide\_synthase | BGC0001511 | Polyketide | 29.0 | 63.8 | 119.0 | 2.2e-27 |
| CAP20363.1 | equibactin\_siderophore\_biosynthetic\_protein | BGC0000347 | NRP | 25.0 | 86.8 | 116.0 | 3.38e-27 |
| AAM54075.1 | polyketide\_synthase | BGC0000020 | Polyketide | 27.0 | 84.8 | 117.0 | 5.19e-27 |
| AQM58276.1 | hypothetical\_protein | BGC0001816 | NRP+Polyketide | 27.0 | 71.4 | 117.0 | 5.54e-27 |
| EAU36837.1 | predicted\_protein | BGC0002348 | NRP | 27.0 | 71.4 | 115.0 | 1.2e-26 |
| AEI58865.1 | peptide\_synthetase | BGC0000455 | NRP | 26.0 | 90.6 | 114.0 | 6.37e-26 |
| ACC80702.1 | AMP-dependent\_synthetase\_and\_ligase | BGC0002677 | Other | 29.0 | 72.0 | 114.0 | 6.63e-26 |
| AAL15600.1 | SimH | BGC0000270 | Polyketide | 26.0 | 93.2 | 113.0 | 7.62e-26 |
| AAK06804.1 | Tyroxyl-AMP-forming\_enzyme | BGC0001072 | Saccharide+Polyketide:Modular type I polyketide+Polyketide:Type II polyketide+Other:Aminocoumarin | 26.0 | 93.2 | 113.0 | 7.69e-26 |
| ACB12556.1 | Fum10 | BGC0000063 | Polyketide | 26.0 | 67.9 | 112.0 | 1.04e-25 |
| ACA99172.1 | polyketide\_synthase | BGC0001160 | Polyketide:Modular type I polyketide | 29.0 | 72.1 | 112.0 | 1.93e-25 |
| QDJ74273.1 | non-ribosomal\_peptide\_synthetase | BGC0002109 | NRP | 28.0 | 81.9 | 108.0 | 3.56e-24 |
| BAQ25518.1 | acyl-CoA\_synthetase | BGC0001288 | Polyketide | 28.0 | 64.8 | 106.0 | 4.28e-24 |
| BAF50711.1 | non\_ribosomal\_peptide\_synthetase\_for\_virginiamycin\_S | BGC0001116 | NRP+Polyketide | 26.0 | 82.2 | 108.0 | 4.32e-24 |
| EME52990.1 | amino\_acid\_adenylation\_protein | BGC0001460 | NRP:Glycopeptide | 26.0 | 92.5 | 108.0 | 4.44e-24 |
| MBE8994627.1 | fatty\_acyl-AMP\_ligase | BGC0002623 | NRP+Polyketide | 29.0 | 72.5 | 107.0 | 4.87e-24 |
| BAE93741.1 | putative\_acyltransferase\_family\_protein | BGC0000164 | Polyketide | 26.0 | 71.1 | 107.0 | 4.95e-24 |
| AXN93595.1 | PuwI | BGC0001952 | NRP | 29.0 | 72.0 | 107.0 | 5.15e-24 |
| QGA70148.1 | nonribosomal\_peptide\_synthetase | BGC0002293 | NRP | 29.0 | 74.6 | 108.0 | 6.54e-24 |
| WP\_082191961.1 | non-ribosomal\_peptide\_synthetase | BGC0001451 | NRP | 25.0 | 87.1 | 107.0 | 7.75e-24 |
| AKP45400.1 | CysL | BGC0001413 | NRP | 27.0 | 64.1 | 107.0 | 9.63e-24 |
| UPA71912.1 | tyrocidine\_synthase\_3 | BGC0002636 | Polyketide | 27.0 | 67.2 | 105.0 | 1.26e-23 |
| AEC14348.1 | nonribosomal\_peptide\_synthetase | BGC0000377 | NRP | 25.0 | 91.5 | 107.0 | 1.33e-23 |
| AAN74813.2 | Fum10p | BGC0000062 | Polyketide | 25.0 | 67.9 | 105.0 | 1.36e-23 |
| EFE73313.1 | nonribosomal\_peptide\_synthetase | BGC0000431 | NRP:Cyclic depsipeptide | 24.0 | 86.6 | 106.0 | 1.87e-23 |
| QNH67551.1 | Cip23 | BGC0002108 | NRP | 27.0 | 98.8 | 106.0 | 1.91e-23 |
| AEI70245.1 | nonribosomal\_peptide\_synthetase\_NRPS | BGC0000401 | NRP | 26.0 | 83.1 | 105.0 | 3.04e-23 |
| UMM61371.1 | Tsk10 | BGC0002661 | NRP | 26.0 | 81.5 | 105.0 | 3.25e-23 |
| ADG27359.1 | peptide\_synthetase | BGC0000296 | NRP | 29.0 | 83.6 | 105.0 | 3.31e-23 |
| CAE16560.1 |  | BGC0000196 | Polyketide:Type II polyketide | 26.0 | 64.5 | 103.0 | 3.57e-23 |
| AFY58527.1 | acyl-CoA\_synthetase\_(AMP-forming)/AMP-acid\_ligase\_II | BGC0002411 | NRP+Polyketide | 28.0 | 72.8 | 104.0 | 3.97e-23 |
| BAH43766.1 | tyrocidine\_synthetase\_III | BGC0000452 | NRP | 24.0 | 92.5 | 105.0 | 4.54e-23 |
| AEA60663.1 | pyoverdine\_synthetase | BGC0000892 | Other | 27.0 | 81.5 | 103.0 | 5.36e-23 |
| ACO78745.1 | Non-ribosomal\_peptide\_synthase:Amino\_acid\_adenylation | BGC0002433 | NRP | 28.0 | 80.3 | 105.0 | 5.82e-23 |
| AIL50191.1 | putative\_amide\_synthetase | BGC0000213 | Polyketide:Type II polyketide | 25.0 | 82.1 | 103.0 | 6.44e-23 |
| ABI22131.1 | putative\_non-ribosomal\_peptide\_synthetase | BGC0000422 | NRP | 28.0 | 71.6 | 104.0 | 6.67e-23 |
| CBA63680.1 | nonribosomal\_peptide\_synthetase\_NRPS | BGC0000368 | NRP | 24.0 | 99.1 | 104.0 | 7.13e-23 |
| AAR87760.2 | ZmaK | BGC0001059 | NRP+Polyketide | 26.0 | 77.7 | 104.0 | 7.18e-23 |
| QNH67552.1 | Cip24 | BGC0002108 | NRP | 28.0 | 84.5 | 104.0 | 7.92e-23 |
| WP\_013310341.1 | aminotransferase\_class\_III-fold\_pyridoxal\_phosphate-dependent\_enzyme | BGC0001728 | NRP+Polyketide | 26.0 | 73.5 | 104.0 | 9.07e-23 |
| CBZ42146.1 | putative\_non-ribosomal\_peptide\_synthetase | BGC0001117 | NRP | 24.0 | 90.8 | 104.0 | 1.04e-22 |
| ABF88003.1 | putative\_long-chain-fatty-acid\_CoA\_ligase | BGC0000871 | Other:Nucleoside | 23.0 | 95.3 | 103.0 | 1.09e-22 |
| AXN93598.1 | PuwC | BGC0001952 | NRP | 27.0 | 73.0 | 102.0 | 1.18e-22 |
| QEO74905.1 | condensation\_domain-containing\_protein | BGC0002588 | Other | 24.0 | 91.6 | 103.0 | 1.32e-22 |
| AAU34203.1 | mannopeptimycin\_peptide\_synthetase\_MppB | BGC0000388 | NRP | 27.0 | 76.1 | 103.0 | 1.33e-22 |
| ADB02855.1 | AzicM | BGC0000202 | Polyketide | 25.0 | 80.3 | 102.0 | 1.34e-22 |
| MCF2151708.1 | Non-ribosomal\_peptide\_synthetase | BGC0002625 | NRP+Polyketide | 23.0 | 92.0 | 103.0 | 1.4e-22 |
| BAI63283.1 | putative\_non-ribosomal\_peptide\_synthetase | BGC0000434 | NRP | 27.0 | 75.1 | 102.0 | 1.53e-22 |
| QUS58939.1 | amino\_acid\_adenylation\_domain-containing\_protein | BGC0002123 | NRP+Polyketide | 25.0 | 96.5 | 103.0 | 1.69e-22 |
| ACU71632.1 | amide\_synthetase | BGC0001154 | Other | 29.0 | 66.7 | 102.0 | 1.75e-22 |
| WP\_026723805.1 | AMP-dependent\_synthetase | BGC0001467 | NRP:Cyclic depsipeptide+Polyketide:Modular type I polyketide | 28.0 | 72.8 | 102.0 | 2.09e-22 |
| CAL48957.1 | anthranilate-CoA-ACP\_transferase | BGC0001343 | Polyketide | 27.0 | 76.1 | 101.0 | 2.44e-22 |
| ABM34280.1 | amino\_acid\_adenylation\_domain\_protein | BGC0002419 | NRP+Polyketide | 31.0 | 55.1 | 102.0 | 2.7e-22 |
| QUJ09167.1 | Lon20 | BGC0002440 | NRP | 25.0 | 88.2 | 102.0 | 3.13e-22 |
| AXN93611.1 | PuwC | BGC0001953 | NRP | 27.0 | 73.0 | 100.0 | 3.15e-22 |
| AAT12283.1 | LtxA | BGC0000384 | NRP | 24.0 | 90.6 | 102.0 | 3.85e-22 |
| AHD05679.1 | putative\_non-ribosomal\_peptide\_ligase/\_polyketide\_synthase\_hybrid | BGC0000402 | NRP | 25.0 | 92.0 | 102.0 | 4.12e-22 |
| QWP75305.1 | non-ribosomal\_peptide\_synthase | BGC0002126 | NRP:Cyclic depsipeptide | 23.0 | 92.2 | 102.0 | 4.29e-22 |
| CAG25758.1 | putative\_type\_I\_polyketide\_synthase | BGC0000311 | NRP | 26.0 | 72.3 | 100.0 | 4.72e-22 |
| AXN93608.1 | PuwI | BGC0001953 | NRP | 29.0 | 72.0 | 101.0 | 4.93e-22 |
| CAJ88192.1 | Putative\_acyl-CoA\_synthetase | BGC0000151 | Polyketide:Modular type I polyketide+Saccharide:Hybrid/tailoring saccharide | 29.0 | 73.5 | 100.0 | 5.02e-22 |
| CCP42824.1 | Possible\_fatty-acid-CoA\_ligase\_FadD10\_(fatty-acid-CoA\_synthetase)\_(fatty-acid-CoA\_synthase) | BGC0001627 | NRP | 29.0 | 65.9 | 100.0 | 5.43e-22 |
| ABS74180.1 | bacillomycin\_D\_synthetase\_B | BGC0001090 | Polyketide+NRP:Lipopeptide | 25.0 | 85.2 | 102.0 | 5.6e-22 |
| AJK49758.1 | non-ribosomal\_peptide\_synthase | BGC0002565 | NRP | 27.0 | 78.7 | 101.0 | 6.91e-22 |
| BAB69699.1 | iturin\_A\_synthetase\_B | BGC0001098 | NRP+Polyketide | 25.0 | 85.2 | 101.0 | 7.41e-22 |
| AAX31557.1 | peptide\_synthetase\_1 | BGC0000336 | NRP | 27.0 | 90.4 | 101.0 | 7.46e-22 |
| ABD65956.1 | nonribosomal\_peptide\_synthetase | BGC0000341 | NRP | 24.0 | 95.5 | 101.0 | 8.65e-22 |
| ABS74181.1 | bacillomycin\_D\_synthetase\_A\_ | BGC0001090 | Polyketide+NRP:Lipopeptide | 25.0 | 73.7 | 101.0 | 9.55e-22 |
| AAN32979.1 | BarE | BGC0000962 | NRP+Polyketide:Modular type I polyketide | 26.0 | 72.6 | 100.0 | 1.09e-21 |
| ACU71638.1 | peptide\_synthetase-like\_protein | BGC0001154 | Other | 24.0 | 84.7 | 100.0 | 1.16e-21 |
| AEA30274.1 | peptide\_synthetase | BGC0000429 | Polyketide+NRP:Cyclic depsipeptide | 26.0 | 91.5 | 100.0 | 1.26e-21 |
| QBG38783.1 | Atr22 | BGC0001975 | NRP | 26.0 | 93.7 | 100.0 | 2.19e-21 |
| AED90002.1 | non-ribosomal\_peptide\_synthetase\_ThaA | BGC0000443 | NRP:Beta-lactam | 25.0 | 75.4 | 100.0 | 2.27e-21 |
| APZ78722.1 | acyl-CoA\_synthetase | BGC0001420 | NRP:Cyclic depsipeptide+Polyketide:Iterative type I polyketide | 27.0 | 72.1 | 99.0 | 2.52e-21 |
| AKJ15827.1 | peptide\_synthetase | BGC0002735 | Polyketide+NRP | 25.0 | 94.1 | 100.0 | 2.76e-21 |
| ADH04679.1 | non-ribosomal\_peptide\_synthetase | BGC0001344 | NRP+Polyketide | 30.0 | 54.0 | 100.0 | 2.77e-21 |
| AEF33078.1 | dimodular\_nonribosomal\_peptide\_synthetase | BGC0001039 | NRP+Polyketide | 24.0 | 91.5 | 99.0 | 3.53e-21 |
| KYC42745.1 | AMP-dependent\_synthetase | BGC0002484 | NRP+Polyketide | 27.0 | 73.0 | 99.0 | 3.57e-21 |
| AGE11898.1 | nonribosomal\_peptide\_synthetase | BGC0000366 | NRP | 26.0 | 87.5 | 99.0 | 3.65e-21 |
| WP\_028678148.1 | non-ribosomal\_peptide\_synthetase | BGC0001228 | NRP:Cyclic depsipeptide | 27.0 | 67.2 | 99.0 | 3.76e-21 |
| WP\_105852536.1 | acyl-CoA\_ligase\_(AMP-forming),\_exosortase\_A\_system-associated | BGC0002336 | NRP | 23.0 | 85.7 | 98.0 | 3.82e-21 |
| CUX79060.1 | Octapeptin\_synthase\_subunit\_A | BGC0001715 | NRP | 23.0 | 99.0 | 99.0 | 3.88e-21 |
| AKJ15828.1 | peptide\_synthetase | BGC0002735 | Polyketide+NRP | 24.0 | 92.7 | 99.0 | 4.02e-21 |
| AQH32483.1 | hybrid\_peptide\_synthetase/polyketide\_synthase | BGC0001667 | NRP+Polyketide | 29.0 | 67.9 | 99.0 | 4.85e-21 |
| BAB69698.1 | iturin\_A\_synthetase\_A | BGC0001098 | NRP+Polyketide | 25.0 | 73.7 | 99.0 | 5.12e-21 |
| NAO96320.1 | amino\_acid\_adenylation\_domain-containing\_protein | BGC0002117 | NRP | 25.0 | 88.9 | 99.0 | 5.19e-21 |
| ALV82356.1 | CDA\_peptide\_synthetase\_I | BGC0001370 | NRP | 25.0 | 93.0 | 99.0 | 5.36e-21 |
| EME52974.1 | non-ribosomal\_peptide\_synthetase | BGC0001460 | NRP:Glycopeptide | 25.0 | 74.9 | 97.0 | 6.17e-21 |
| NKI69295.1 | amino\_acid\_adenylation\_domain-containing\_protein | BGC0002408 | NRP | 26.0 | 70.6 | 99.0 | 6.61e-21 |
| QNH85847.1 | BolO | BGC0002327 | NRP | 23.0 | 85.9 | 97.0 | 6.77e-21 |
| ACZ65474.1 | palmitoyl-CoA\_synthetase | BGC0000140 | Polyketide | 29.0 | 73.3 | 97.0 | 8.7e-21 |
| OKA09423.1 | non-ribosomal\_peptide\_synthetase | BGC0001459 | NRP:Glycopeptide | 24.0 | 91.1 | 98.0 | 8.71e-21 |
| WP\_019032757.1 | type\_I\_polyketide\_synthase | BGC0001331 | NRP:Cyclic depsipeptide+Polyketide:Modular type I polyketide | 28.0 | 71.1 | 98.0 | 1.09e-20 |
| WP\_018960015.1 | fatty\_acyl-AMP\_ligase | BGC0002010 | NRP+Polyketide | 28.0 | 74.6 | 97.0 | 1.16e-20 |
| AHD05678.1 | nonribosomal\_peptide\_ligase\_subunit | BGC0000402 | NRP | 23.0 | 88.2 | 98.0 | 1.22e-20 |
| AHN85651.1 | Phn2 | BGC0000122 | Polyketide:Modular type I polyketide | 25.0 | 100.9 | 98.0 | 1.24e-20 |
| BAH43870.1 | putative\_linear\_pentadecapeptide\_gramicidin\_synthetase\_LgrB | BGC0000367 | NRP | 21.0 | 92.7 | 98.0 | 1.24e-20 |
| AAZ23074.1 | acyl-CoA\_ligase | BGC0000291 | NRP | 29.0 | 72.1 | 97.0 | 1.5e-20 |
| QDA77058.1 | polyketide\_synthase | BGC0002026 | NRP+Polyketide | 28.0 | 71.6 | 97.0 | 1.5e-20 |
| QBA57737.1 | NRPS | BGC0002377 | NRP | 25.0 | 96.3 | 97.0 | 1.51e-20 |
| QBG38784.1 | Atr23 | BGC0001975 | NRP | 26.0 | 77.4 | 97.0 | 1.59e-20 |
| EFE73312.1 | nonribosomal\_peptide\_synthetase | BGC0000431 | NRP:Cyclic depsipeptide | 22.0 | 99.3 | 97.0 | 1.62e-20 |
| AGM16414.1 | paenibacterin\_synthetase\_C | BGC0000400 | NRP | 24.0 | 85.5 | 97.0 | 2.02e-20 |
| EDT06082.1 | AMP-dependent\_synthetase\_and\_ligase | BGC0001897 | Polyketide | 30.0 | 73.2 | 96.0 | 2.04e-20 |
| AAX98208.1 | amide\_synthetase | BGC0000052 | Polyketide | 25.0 | 98.4 | 96.0 | 2.07e-20 |
| BBD17759.1 | non-ribosomal\_peptide\_synthetase | BGC0001919 | NRP+Polyketide | 28.0 | 68.1 | 97.0 | 2.1e-20 |
| CAI94718.1 | putative\_CoA\_ligase | BGC0000141 | Polyketide | 26.0 | 66.2 | 97.0 | 2.17e-20 |
| ABD65958.1 | nonribosomal\_peptide\_synthetase | BGC0000341 | NRP | 25.0 | 91.5 | 97.0 | 2.19e-20 |
| AHH34188.1 | fatty\_acyl\_ACP\_ligase | BGC0001162 | Polyketide:Modular type I polyketide | 26.0 | 74.4 | 96.0 | 2.2e-20 |
| BAK64635.1 | putative\_CoA\_ligase | BGC0000135 | Polyketide | 28.0 | 73.5 | 96.0 | 2.57e-20 |
| CAA11794.1 | PCZA363.3 | BGC0000322 | NRP | 25.0 | 90.9 | 97.0 | 2.66e-20 |
| KON97028.1 | phenylalanine\_racemase | BGC0002122 | NRP | 22.0 | 91.5 | 97.0 | 2.77e-20 |
| BAW32322.1 | nonribosomal\_peptide\_synthetase | BGC0001630 | NRP+Polyketide | 29.0 | 68.5 | 96.0 | 2.93e-20 |
| AQX77690.1 | NocL | BGC0001704 | Other | 27.0 | 74.4 | 96.0 | 2.98e-20 |
| QEO75077.1 | condensation\_domain-containing\_protein | BGC0002079 | NRP:Cyclic depsipeptide | 25.0 | 98.8 | 96.0 | 3.35e-20 |
| BAX64247.1 | NRPS | BGC0001623 | NRP+Polyketide | 26.0 | 86.4 | 96.0 | 3.39e-20 |
| AXN93578.1 | PuwC | BGC0001950 | NRP | 27.0 | 71.8 | 95.0 | 3.52e-20 |
| AXN93587.1 | PuwC | BGC0001951 | NRP | 27.0 | 71.8 | 95.0 | 3.52e-20 |
| CAR51994.1 | ornibactin\_biosynthesis\_non-ribosomal\_peptide\_synthase | BGC0002569 | NRP | 28.0 | 68.8 | 96.0 | 3.53e-20 |
| QYA95680.1 | amino\_acid\_adenylation\_domain-containing\_protein | BGC0002676 | NRP | 26.0 | 90.9 | 96.0 | 3.61e-20 |
| QEO75073.1 | condensation\_domain-containing\_protein | BGC0002079 | NRP:Cyclic depsipeptide | 24.0 | 91.1 | 96.0 | 4.67e-20 |
| ALV86866.1 | Tlo20 | BGC0001406 | NRP | 23.0 | 94.1 | 96.0 | 4.86e-20 |
| AXM43064.1 | AMP-dependent\_synthetase\_and\_ligase | BGC0001945 | NRP | 25.0 | 90.8 | 95.0 | 4.94e-20 |
| QST87270.1 | amino\_acid\_adenylation\_domain-containing\_protein | BGC0002572 | NRP+Polyketide | 21.0 | 94.8 | 96.0 | 5.02e-20 |
| AEI58879.1 | peptide\_synthetase | BGC0000455 | NRP | 25.0 | 74.0 | 94.0 | 5.67e-20 |
| CAF05648.1 | TubC\_protein | BGC0001053 | NRP+Polyketide | 24.0 | 90.9 | 96.0 | 5.99e-20 |
| AGN74876.1 | nonribosomal\_peptide\_synthetase | BGC0000459 | NRP:Cyclic depsipeptide+Polyketide:Trans-AT type I polyketide | 27.0 | 83.3 | 96.0 | 5.99e-20 |
| SDF67478.1 | Phosphopantetheine\_attachment\_site | BGC0002422 | NRP | 24.0 | 86.9 | 95.0 | 6.18e-20 |
| AIW82280.1 | PuwC | BGC0001125 | NRP+Polyketide | 26.0 | 71.8 | 94.0 | 6.32e-20 |
| AIG26884.1 | NRPS\_domain-containing\_protein | BGC0002432 | NRP | 23.0 | 99.8 | 96.0 | 6.75e-20 |
| AFV27434.1 | indigoidine\_synthase | BGC0000375 | NRP | 24.0 | 80.3 | 95.0 | 6.76e-20 |
| WP\_013310343.1 | non-ribosomal\_peptide\_synthetase | BGC0001728 | NRP+Polyketide | 24.0 | 87.3 | 95.0 | 7.02e-20 |
| CZT62785.1 | Non-ribosomal\_peptide\_synthase,\_involved\_in\_Hassallidin\_biosynthesis | BGC0001614 | NRP | 23.0 | 90.8 | 95.0 | 8.08e-20 |
| QEO74904.1 | AMP-dependent\_synthetase\_and\_ligase | BGC0002588 | Other | 25.0 | 87.1 | 95.0 | 8.11e-20 |
| WP\_063738219.1 | amino\_acid\_adenylation\_domain-containing\_protein | BGC0002010 | NRP+Polyketide | 27.0 | 92.7 | 94.0 | 9.51e-20 |
| AKJ29412.1 | peptide\_synthetase | BGC0001608 | NRP | 23.0 | 94.8 | 95.0 | 1.14e-19 |
| ATY37588.1 | BogA | BGC0001532 | NRP | 21.0 | 91.6 | 94.0 | 1.24e-19 |
| AKL71645.1 | NocL | BGC0001703 | Other | 26.0 | 73.5 | 93.0 | 1.61e-19 |
| BAH43869.1 | linear\_pentadecapeptide\_gramicidin\_synthetase\_LgrA | BGC0000367 | NRP | 23.0 | 90.2 | 94.0 | 1.78e-19 |
| CBD77732.1 | polyketide\_synthase | BGC0000974 | NRP+Polyketide | 27.0 | 74.2 | 94.0 | 1.82e-19 |
| AEH41794.1 | HrmP | BGC0000374 | NRP:Cyclic depsipeptide | 24.0 | 92.9 | 94.0 | 1.9e-19 |
| AHB82062.1 | polyketide\_synthase | BGC0001231 | NRP+Polyketide:Modular type I polyketide | 25.0 | 93.6 | 94.0 | 1.9e-19 |
| QRG35014.1 | NRPS | BGC0002378 | NRP | 25.0 | 83.6 | 94.0 | 1.96e-19 |
| AEH41793.1 | HrmO | BGC0000374 | NRP:Cyclic depsipeptide | 26.0 | 82.1 | 94.0 | 1.97e-19 |
| ABD65957.1 | nonribosomal\_peptide\_synthetase | BGC0000341 | NRP | 26.0 | 69.3 | 94.0 | 2.01e-19 |
| EGJ35087.1 | Fatty-acyl-ACP\_ligase | BGC0001163 | Polyketide:Modular type I polyketide | 24.0 | 95.1 | 93.0 | 2.07e-19 |
| ABP55169.1 | amino\_acid\_adenylation\_domain | BGC0000150 | NRP+Polyketide:Enediyne type I polyketide | 24.0 | 86.9 | 92.0 | 2.54e-19 |
| SJZ83675.1 | non-ribosomal\_peptide\_synthase\_domain\_TIGR01720/amino\_acid\_adenylation\_domain-containing\_protein/thioester\_reductase\_domain-containing\_protein | BGC0002660 | NRP | 23.0 | 85.2 | 94.0 | 2.63e-19 |
| AGM16412.1 | paenibacterin\_synthetase\_A | BGC0000400 | NRP | 23.0 | 92.7 | 94.0 | 2.64e-19 |
| KYC42747.1 | hypothetical\_protein | BGC0002484 | NRP+Polyketide | 22.0 | 95.8 | 94.0 | 2.65e-19 |
| AAC44128.1 | saframycin\_Mx1\_synthetase\_B | BGC0002706 | NRP | 31.0 | 58.5 | 93.0 | 2.95e-19 |
| AAQ59905.1 | synthetase\_CbsF | BGC0002680 | NRP | 25.0 | 96.3 | 93.0 | 3.23e-19 |
| ATG32071.1 | proline\_specific\_adenylation\_domain-containing\_protein | BGC0001750 | NRP+Polyketide | 25.0 | 65.9 | 92.0 | 3.34e-19 |
| AEA30272.1 | peptide\_synthetase | BGC0000429 | Polyketide+NRP:Cyclic depsipeptide | 25.0 | 97.9 | 93.0 | 3.41e-19 |
| MBE3200466.1 | non-ribosomal\_peptide\_synthetase | BGC0002409 | NRP | 23.0 | 84.8 | 93.0 | 3.49e-19 |
| AWI62628.1 | nonribosomal\_peptide\_synthetase | BGC0001822 | NRP | 24.0 | 98.1 | 93.0 | 4.35e-19 |
| CAE02630.1 | surfactin\_synthetase\_A | BGC0000433 | NRP:Lipopeptide | 26.0 | 78.4 | 93.0 | 4.39e-19 |
| AEU11006.1 | NpnB | BGC0001029 | NRP+Polyketide | 23.0 | 89.4 | 93.0 | 4.51e-19 |
| AQA28568.1 | acyl-CoA\_synthase | BGC0001663 | Polyketide | 27.0 | 73.0 | 92.0 | 4.74e-19 |
| QED88054.1 | nonribosomal\_peptide\_synthetase | BGC0001967 | NRP+Polyketide | 25.0 | 89.9 | 92.0 | 5.94e-19 |
| AEA30273.1 | peptide\_synthetase | BGC0000429 | Polyketide+NRP:Cyclic depsipeptide | 24.0 | 92.2 | 92.0 | 5.95e-19 |
| QWT72275.1 | fatty\_acyl-AMP\_ligase | BGC0002430 | NRP+Saccharide | 28.0 | 73.0 | 91.0 | 6.18e-19 |
| BAH22765.1 | nonribosomal\_peptide\_synthetase | BGC0001018 | NRP | 21.0 | 101.7 | 92.0 | 6.32e-19 |
| AHH34187.1 | fatty\_acyl\_ACP\_ligase | BGC0001161 | Polyketide:Modular type I polyketide | 26.0 | 75.8 | 92.0 | 6.37e-19 |
| extra\_gene | NRPS/PKS | BGC0002095 | NRP | 28.0 | 66.4 | 92.0 | 7.26e-19 |
| BBA21073.1 | putative\_non-ribosomal\_peptide\_synthetase | BGC0001740 | NRP+Polyketide | 27.0 | 61.8 | 92.0 | 7.64e-19 |
| CAC48369.1 | peptide\_synthetase | BGC0000311 | NRP | 25.0 | 81.4 | 91.0 | 7.74e-19 |
| CAE15637.1 |  | BGC0001128 | NRP | 25.0 | 92.3 | 92.0 | 7.92e-19 |
| AXG48275.1 | non-ribosomal\_peptide\_synthetase | BGC0002716 | NRP | 25.0 | 92.3 | 92.0 | 7.92e-19 |
| MBC5793764.1 | fatty\_acyl-AMP\_ligase | BGC0002480 | Polyketide+NRP | 25.0 | 91.3 | 91.0 | 8.6e-19 |
| AGZ15460.1 | putative\_non-ribosomal\_peptide\_synthetase | BGC0001036 | NRP+Polyketide | 24.0 | 89.9 | 92.0 | 8.83e-19 |
| APZ78728.1 | nonribosomal\_peptide\_synthetase | BGC0001421 | NRP:Cyclic depsipeptide+Polyketide:Iterative type I polyketide | 25.0 | 81.7 | 92.0 | 9.91e-19 |
| WA1\_15570 | hypothetical\_protein | BGC0002484 | NRP+Polyketide | 23.0 | 87.5 | 92.0 | 1.01e-18 |
| AHB82071.1 | non\_ribosomal\_peptide\_synthetase | BGC0001231 | NRP+Polyketide:Modular type I polyketide | 24.0 | 91.5 | 92.0 | 1.02e-18 |
| CCM44336.1 | Nonribosomal\_peptide\_synthetase | BGC0001056 | NRP+Polyketide:Modular type I polyketide+Polyketide:PUFA synthase or related polyketide | 23.0 | 81.9 | 92.0 | 1.03e-18 |
| AHJ31215.1 | Long-chain-fatty-acid--CoA\_ligase | BGC0000430 | NRP+Polyketide:Modular type I polyketide | 24.0 | 85.0 | 92.0 | 1.03e-18 |
| ACY13414.1 | amino\_acid\_adenylation\_domain\_protein | BGC0001367 | NRP+Polyketide | 27.0 | 77.0 | 92.0 | 1.03e-18 |
| OKA09664.1 | non-ribosomal\_peptide\_synthetase | BGC0001459 | NRP:Glycopeptide | 26.0 | 83.1 | 91.0 | 1.03e-18 |
| ATY37608.1 | BreC | BGC0001536 | NRP | 23.0 | 100.5 | 92.0 | 1.09e-18 |
| ABV79985.1 | ApnA | BGC0000301 | NRP | 23.0 | 92.5 | 91.0 | 1.24e-18 |
| WP\_053065267.1 | type\_I\_polyketide\_synthase | BGC0001330 | NRP:Cyclic depsipeptide+Polyketide:Modular type I polyketide | 27.0 | 70.7 | 91.0 | 1.25e-18 |
| WP\_068925909.1 | non-ribosomal\_peptide\_synthetase | BGC0002688 | NRP | 24.0 | 91.8 | 91.0 | 1.26e-18 |
| AQV04230.1 | SwnK | BGC0001794 | NRP+Polyketide | 23.0 | 80.7 | 91.0 | 1.27e-18 |
| CBD77754.1 | hypothetical\_protein | BGC0000974 | NRP+Polyketide | 24.0 | 104.0 | 91.0 | 1.28e-18 |
| KUM80513.1 | hypothetical\_protein | BGC0001562 | NRP | 25.0 | 82.9 | 91.0 | 1.28e-18 |
| AAU34202.1 | mannopeptimycin\_peptide\_synthetase\_MppA | BGC0000388 | NRP | 23.0 | 94.1 | 91.0 | 1.29e-18 |
| ADN13832.1 | Polyketide\_Synthase | BGC0001164 | Polyketide:Modular type I polyketide | 28.0 | 72.5 | 91.0 | 1.29e-18 |
| EWM62997.1 | non-ribosomal\_peptide\_synthetase | BGC0001328 | NRP:Cyclic depsipeptide+Polyketide:Modular type I polyketide | 28.0 | 73.5 | 91.0 | 1.45e-18 |
| ACN39727.1 | SibD | BGC0000428 | NRP | 26.0 | 79.4 | 91.0 | 1.5e-18 |
| AAX31559.1 | peptide\_synthetase\_3 | BGC0000336 | NRP | 24.0 | 97.2 | 91.0 | 1.66e-18 |
| ABC36450.1 | peptide\_synthetase-like\_protein | BGC0000386 | NRP:NRP siderophore | 27.0 | 69.2 | 91.0 | 1.74e-18 |
| AQW35047.1 | Acyl-CoA\_synthetase | BGC0001675 | Polyketide | 27.0 | 74.2 | 90.0 | 1.78e-18 |
| QYA95681.1 | amino\_acid\_adenylation\_domain-containing\_protein | BGC0002676 | NRP | 24.0 | 92.2 | 91.0 | 1.81e-18 |
| AFJ14793.1 | PlpD | BGC0000403 | NRP | 24.0 | 79.1 | 91.0 | 1.98e-18 |
| ALG65317.1 | Cal19 | BGC0001297 | NRP | 24.0 | 95.1 | 91.0 | 2.1e-18 |
| AUD11994.1 | OrbI | BGC0001721 | NRP | 28.0 | 69.2 | 91.0 | 2.29e-18 |
| QYA95682.1 | amino\_acid\_adenylation\_domain-containing\_protein | BGC0002676 | NRP | 25.0 | 97.0 | 91.0 | 2.39e-18 |
| QKW94305.1 | AMP-dependent\_synthetase/ligase | BGC0002342 | NRP+Polyketide | 27.0 | 71.3 | 90.0 | 2.43e-18 |
| CAG15025.1 | Acyl-CoA\_synthase | BGC0000441 | NRP | 27.0 | 73.2 | 89.0 | 2.48e-18 |
| CAE53367.1 | acyl-CoA\_ligase | BGC0000440 | NRP:Glycopeptide | 27.0 | 73.2 | 89.0 | 2.5e-18 |
| AQZ71347.1 | hypothetical\_protein | BGC0001635 | NRP+Polyketide | 29.0 | 66.7 | 90.0 | 2.61e-18 |
| AQV04224.1 | SwnK | BGC0001793 | NRP+Polyketide | 24.0 | 78.9 | 90.0 | 2.93e-18 |
| BAP27942.1 | nonribosomal\_peptide\_synthetase | BGC0001085 | NRP+Terpene | 27.0 | 58.5 | 90.0 | 2.93e-18 |
| AEW31020.1 | plipastatin\_synthetase | BGC0000407 | NRP | 21.0 | 95.8 | 90.0 | 2.94e-18 |
| ARU08069.1 | mlcG | BGC0001448 | NRP:Lipopeptide:Ca+-dependent lipopeptide | 27.0 | 72.1 | 89.0 | 3.23e-18 |
| ACM79806.1 | ZmaB | BGC0001059 | NRP+Polyketide | 23.0 | 72.8 | 90.0 | 3.67e-18 |
| ACS20359.1 | amino\_acid\_adenylation\_domain\_protein | BGC0002420 | NRP+Polyketide | 24.0 | 86.1 | 90.0 | 3.89e-18 |
| WP\_036342114.1 | type\_I\_polyketide\_synthase | BGC0001327 | NRP:Cyclic depsipeptide+Polyketide:Modular type I polyketide | 28.0 | 73.5 | 90.0 | 3.95e-18 |
| APD26279.1 | PtmA | BGC0001726 | NRP+Polyketide | 25.0 | 90.8 | 90.0 | 3.99e-18 |
| WP\_012408783.1 | non-ribosomal\_peptide\_synthetase | BGC0002061 | NRP:Cyclic depsipeptide+Polyketide:Modular type I polyketide | 21.0 | 94.1 | 90.0 | 4.13e-18 |
| OLZ50899.1 | non-ribosomal\_peptide\_synthetase | BGC0001461 | NRP:Glycopeptide | 27.0 | 73.5 | 89.0 | 4.18e-18 |
| QEO75075.1 | condensation\_domain-containing\_protein | BGC0002079 | NRP:Cyclic depsipeptide | 24.0 | 74.6 | 90.0 | 4.2e-18 |
| ACA97580.1 | PmxE | BGC0000408 | NRP | 24.0 | 92.5 | 90.0 | 4.24e-18 |
| CAD17792.1 | probable\_non\_ribosomal\_peptide\_synthetase\_protein | BGC0001363 | NRP+Polyketide | 24.0 | 79.1 | 90.0 | 4.26e-18 |
| CAJ76286.1 | putative\_non-ribosomal\_peptide\_synthetase | BGC0000972 | NRP+Polyketide:Modular type I polyketide+Polyketide:Trans-AT type I polyketide | 28.0 | 55.9 | 89.0 | 4.51e-18 |
| AKD43754.1 | HerJ | BGC0001349 | NRP+Polyketide | 27.0 | 66.4 | 88.0 | 4.86e-18 |
| EOY45602.1 | Adenylation\_and\_reductase\_domains\_containing\_protein | BGC0001168 | NRP | 24.0 | 84.3 | 89.0 | 5.09e-18 |
| ABS74206.1 | fengycin\_synthetase\_D | BGC0001095 | NRP | 22.0 | 86.6 | 89.0 | 5.35e-18 |
| AIE77076.1 | peptide\_synthetase | BGC0000418 | NRP | 27.0 | 73.7 | 88.0 | 5.55e-18 |
| AIZ66877.1 | nonribosomal\_peptide\_synthetase | BGC0002666 | NRP+Alkaloid | 25.0 | 74.4 | 88.0 | 5.57e-18 |
| ALV86864.1 | Tlo18 | BGC0001406 | NRP | 29.0 | 57.3 | 88.0 | 5.61e-18 |
| ABY83163.1 | Azi25 | BGC0000960 | NRP+Polyketide | 26.0 | 68.3 | 89.0 | 6.06e-18 |
| QOV09198.1 | ClyA/NocL | BGC0002597 | NRP+Polyketide | 27.0 | 74.9 | 88.0 | 6.36e-18 |
| AAL15617.1 | acetyl-coenzyme\_A\_synthetase | BGC0000270 | Polyketide | 26.0 | 50.2 | 86.0 | 6.43e-18 |
| CAD29795.1 | peptide\_synthetase | BGC0001015 | NRP+Polyketide | 27.0 | 68.5 | 89.0 | 6.79e-18 |
| AGZ03651.1 | sevB | BGC0000426 | NRP | 21.0 | 82.1 | 89.0 | 6.85e-18 |
| CAJ96472.1 | non-ribosomal\_peptide\_synthetase | BGC0000330 | NRP:NRP siderophore | 30.0 | 57.1 | 89.0 | 6.94e-18 |
| AIG79224.1 | Non-ribosomal\_peptide\_synthetase/andenylation\_domain | BGC0000419 | Saccharide+NRP:Glycopeptide | 26.0 | 81.2 | 88.0 | 7.35e-18 |
| AYA22318.1 | KerE | BGC0001955 | NRP | 27.0 | 73.5 | 88.0 | 7.35e-18 |
| CDG17985.1 | Putative\_Ornithine\_racemase\_(fragment) | BGC0000464 | NRP:Cyclic depsipeptide | 23.0 | 97.4 | 89.0 | 7.44e-18 |
| QUS58937.1 | non-ribosomal\_peptide\_synthetase | BGC0002123 | NRP+Polyketide | 23.0 | 89.7 | 89.0 | 7.72e-18 |
| AMB48444.1 | long-chain-fatty-acid--CoA\_ligase | BGC0001357 | Polyketide | 24.0 | 93.6 | 88.0 | 7.82e-18 |
| AHB38515.1 | non-ribosomal\_peptide\_synthetase | BGC0000345 | NRP+Polyketide:Modular type I polyketide | 25.0 | 66.0 | 89.0 | 8.65e-18 |
| AID65224.1 | nonribosomal\_peptide\_synthetase | BGC0000335 | NRP+Polyketide | 27.0 | 66.0 | 89.0 | 8.91e-18 |
| CBW75453.1 | Non-ribosomal\_peptide\_synthetase\_modules\_(EC\_6.3.2.-) | BGC0002048 | NRP:Cyclic depsipeptide | 24.0 | 86.2 | 89.0 | 9.06e-18 |
| QIH29228.1 | endopyrrole\_NRPS\_A | BGC0002326 | NRP | 24.0 | 86.2 | 89.0 | 9.62e-18 |
| ACR50797.1 | acyltransferase | BGC0000163 | Polyketide | 25.0 | 71.3 | 87.0 | 9.89e-18 |
| AXF14775.1 | non-ribosomal\_peptide\_synthetase | BGC0002563 | NRP | 26.0 | 69.3 | 88.0 | 1.12e-17 |
| QEO74983.1 | omn8 | BGC0002078 | NRP:Cyclic depsipeptide | 25.0 | 75.1 | 88.0 | 1.16e-17 |
| AGE11899.1 | nonribosomal\_peptide\_synthetase | BGC0000366 | NRP | 25.0 | 95.5 | 88.0 | 1.16e-17 |
| CAC48360.1 | peptide\_synthetase | BGC0000311 | NRP | 24.0 | 92.0 | 88.0 | 1.21e-17 |
| BAP05589.1 | calA | BGC0000967 | NRP+Polyketide:Trans-AT type I polyketide | 24.0 | 93.0 | 88.0 | 1.24e-17 |
| QBG38782.1 | Atr21 | BGC0001975 | NRP | 25.0 | 78.6 | 88.0 | 1.24e-17 |
| APO47826.1 | hypothetical\_protein | BGC0002653 | NRP | 23.0 | 93.2 | 88.0 | 1.27e-17 |
| QNL34618.1 | SteC | BGC0002092 | NRP:Cyclic depsipeptide | 25.0 | 85.5 | 88.0 | 1.28e-17 |
| AAG31130.1 | MxcG | BGC0001345 | NRP | 25.0 | 75.1 | 88.0 | 1.37e-17 |
| WP\_054234617.1 | non-ribosomal\_peptide\_synthetase | BGC0002014 | NRP+Polyketide | 26.0 | 80.5 | 88.0 | 1.55e-17 |
| AEO14743.1 | NdaA | BGC0000396 | NRP | 23.0 | 78.6 | 88.0 | 1.56e-17 |
| AFO85453.1 | non-ribosomal\_peptide\_synthetase | BGC0000391 | NRP | 24.0 | 96.7 | 88.0 | 1.58e-17 |
| QGY73453.1 | Itm21 | BGC0002451 | Polyketide | 26.0 | 66.9 | 88.0 | 1.59e-17 |
| APZ78755.1 | nonribosomal\_peptide\_synthetase | BGC0001423 | NRP:Cyclic depsipeptide+Polyketide:Iterative type I polyketide | 25.0 | 81.7 | 88.0 | 1.59e-17 |
| RLV64601.1 | polyketide\_synthase\_of\_type\_I | BGC0001845 | Polyketide+NRP+Other:Aminocoumarin | 25.0 | 90.6 | 88.0 | 1.62e-17 |
| AAZ03550.1 | McnA | BGC0000332 | NRP | 22.0 | 98.3 | 87.0 | 1.63e-17 |
| AAQ90177.1 | putative\_acyl-CoA\_synthetase | BGC0000128 | Polyketide | 23.0 | 90.2 | 86.0 | 1.77e-17 |
| AAS98774.1 | Acyl-ACP\_Synthetase | BGC0001001 | NRP+Polyketide | 27.0 | 72.3 | 87.0 | 1.77e-17 |
| QCP68979.1 | VatA | BGC0002296 | NRP+Polyketide | 25.0 | 93.6 | 87.0 | 1.8e-17 |
| AQX14441.1 | EM5400\_NRPS\_scaffold | BGC0001671 | NRP | 24.0 | 89.4 | 87.0 | 2.08e-17 |
| SDF67357.1 | amino\_acid\_adenylation\_domain-containing\_protein | BGC0002422 | NRP | 24.0 | 83.4 | 87.0 | 2.08e-17 |
| ABS75100.1 | AMP-binding\_protein | BGC0002641 | NRP | 22.0 | 82.9 | 86.0 | 2.11e-17 |
| AAF17280.1 | nosC | BGC0001028 | Polyketide+NRP:Cyclic depsipeptide | 22.0 | 102.3 | 87.0 | 2.12e-17 |
| BAI23334.1 | putative\_non-ribosomal\_peptide\_synthetase | BGC0000949 | NRP | 24.0 | 91.3 | 87.0 | 2.15e-17 |
| AKP45399.1 | CysK | BGC0001413 | NRP | 23.0 | 92.2 | 87.0 | 2.19e-17 |
| QVQ62868.1 | nonribosomal\_peptide\_synthase | BGC0002373 | NRP | 25.0 | 93.2 | 87.0 | 2.19e-17 |
| BAF50727.1 | hybrid\_polyketide\_synthase-non\_ribosomal\_peptide\_synthetase | BGC0001116 | NRP+Polyketide | 32.0 | 53.1 | 87.0 | 2.25e-17 |
| AAZ03554.1 | McnE | BGC0000332 | NRP | 21.0 | 101.2 | 87.0 | 2.37e-17 |
| QCP68969.1 | VatN | BGC0002296 | NRP+Polyketide | 23.0 | 78.9 | 87.0 | 2.63e-17 |
| QWT72293.1 | non-ribosomal\_peptide\_synthetase | BGC0002430 | NRP+Saccharide | 25.0 | 96.2 | 87.0 | 2.68e-17 |
| QEO74979.1 | omn4 | BGC0002078 | NRP:Cyclic depsipeptide | 25.0 | 71.3 | 86.0 | 2.82e-17 |
| AZF85940.1 | non-ribosomal\_peptide\_synthase | BGC0001963 | NRP+Polyketide | 24.0 | 81.9 | 87.0 | 2.83e-17 |
| AJV88375.1 | MfnC | BGC0001214 | NRP | 25.0 | 80.8 | 87.0 | 2.83e-17 |
| CAF32362.1 | putative\_non-ribosomal\_peptide\_synthetase | BGC0000712 | Saccharide | 27.0 | 60.3 | 87.0 | 2.86e-17 |
| PHM49485.1 | Amino\_acid\_adenylation | BGC0001131 | NRP | 24.0 | 71.4 | 87.0 | 2.96e-17 |
| QWT72279.1 | non-ribosomal\_peptide\_synthetase | BGC0002430 | NRP+Saccharide | 27.0 | 89.9 | 87.0 | 2.99e-17 |
| QWM97319.1 | non-ribosomal\_peptide\_synthetase | BGC0002384 | NRP | 25.0 | 79.8 | 87.0 | 3e-17 |
| QPI18723.1 | nonribosomal\_peptide\_synthetase | BGC0002125 | NRP:Cyclic depsipeptide | 24.0 | 88.2 | 87.0 | 3.02e-17 |
| ABV79987.1 | ApnC | BGC0000301 | NRP | 24.0 | 81.5 | 87.0 | 3.57e-17 |
| APZ78703.1 | nonribosomal\_peptide\_synthetase | BGC0001419 | NRP:Cyclic depsipeptide+Polyketide:Iterative type I polyketide | 24.0 | 83.3 | 87.0 | 3.65e-17 |
| QPI18729.1 | nonribosomal\_peptide\_synthetase | BGC0002125 | NRP:Cyclic depsipeptide | 24.0 | 88.2 | 87.0 | 3.67e-17 |
| WP\_050383082.1 | non-ribosomal\_peptide\_synthetase | BGC0001451 | NRP | 24.0 | 76.3 | 87.0 | 3.67e-17 |
| QSJ20139.1 | non-ribosomal\_peptide\_synthase/polyketide\_synthase | BGC0002572 | NRP+Polyketide | 21.0 | 97.0 | 87.0 | 3.83e-17 |
| UHJ79951.1 | non-ribosomal\_peptide\_synthetase | BGC0002654 | NRP | 26.0 | 69.7 | 87.0 | 3.85e-17 |
| KFL51881.1 | beta-ketoacyl\_synthase | BGC0001711 | NRP+Polyketide | 28.0 | 56.1 | 86.0 | 4.18e-17 |
| AGC45618.1 | non-ribosomal\_peptide\_synthetase | BGC0001394 | NRP+Polyketide | 24.0 | 94.6 | 86.0 | 4.21e-17 |
| QBC75023.1 | non-ribosomal\_peptide\_synthetase | BGC0001968 | NRP | 24.0 | 96.3 | 86.0 | 4.76e-17 |
| APZ78715.1 | nonribosomal\_peptide\_synthetase | BGC0001420 | NRP:Cyclic depsipeptide+Polyketide:Iterative type I polyketide | 24.0 | 83.3 | 86.0 | 4.82e-17 |
| APZ78808.1 | nonribosomal\_peptide\_synthetase | BGC0001428 | NRP:Cyclic depsipeptide+Polyketide:Iterative type I polyketide | 25.0 | 83.3 | 86.0 | 4.82e-17 |
| APZ78794.1 | nonribosomal\_peptide\_synthetase | BGC0001427 | NRP:Cyclic depsipeptide+Polyketide:Iterative type I polyketide | 25.0 | 83.3 | 86.0 | 4.82e-17 |
| AEH59099.1 | amino\_acid\_adenylation\_domain-containing\_protein/NRPS | BGC0000385 | NRP | 23.0 | 82.2 | 86.0 | 4.94e-17 |
| AJM89735.1 | PmxA | BGC0001192 | NRP | 23.0 | 92.2 | 86.0 | 5.05e-17 |
| APO47822.1 | non-ribosomal\_peptide\_synthetase | BGC0002653 | NRP | 24.0 | 88.3 | 86.0 | 5.13e-17 |
| MCF2150416.1 | Non-ribosomal\_peptide\_synthetase | BGC0002625 | NRP+Polyketide | 22.0 | 89.2 | 86.0 | 5.13e-17 |
| CAD32910.2 | acyl-CoA\_synthase | BGC0000354 | NRP | 26.0 | 74.4 | 85.0 | 5.42e-17 |
| QCF28941.1 | fatty\_acyl-AMP\_ligase | BGC0002308 | Alkaloid+Polyketide | 28.0 | 73.7 | 85.0 | 5.49e-17 |
| AJV88376.1 | MfnD | BGC0001214 | NRP | 23.0 | 91.1 | 86.0 | 5.59e-17 |
| CAJ77696.1 | MPS2\_protein | BGC0000363 | NRP | 24.0 | 91.3 | 86.0 | 6.21e-17 |
| APZ78691.1 | nonribosomal\_peptide\_synthetase | BGC0001418 | NRP:Cyclic depsipeptide+Polyketide:Iterative type I polyketide | 24.0 | 83.3 | 86.0 | 6.36e-17 |
| AAY93445.1 | non-ribosomal\_peptide\_synthetase\_PvdL | BGC0000413 | NRP | 24.0 | 66.6 | 86.0 | 6.59e-17 |
| QEO75071.1 | AMP-dependent\_synthetase\_and\_ligase | BGC0002079 | NRP:Cyclic depsipeptide | 24.0 | 73.0 | 85.0 | 6.73e-17 |
| AFV96135.1 | AMP-dependent\_synthetase/ligase | BGC0001064 | Polyketide:Modular type I polyketide+Polyketide:Type III polyketide | 27.0 | 72.5 | 85.0 | 7.45e-17 |
| ARU81115.1 | CylA | BGC0001566 | Polyketide | 27.0 | 72.5 | 85.0 | 7.45e-17 |
| ACG60782.1 | NRPS(C/A/PCP/C/A/PCP) | BGC0001058 | NRP:Glycopeptide+Polyketide:Modular type I polyketide+Saccharide:Hybrid/tailoring saccharide | 26.0 | 65.5 | 86.0 | 7.94e-17 |
| APZ78679.1 | nonribosomal\_peptide\_synthetase | BGC0001417 | NRP:Cyclic depsipeptide+Polyketide:Iterative type I polyketide | 24.0 | 81.0 | 86.0 | 8.38e-17 |
| APZ78768.1 | nonribosomal\_peptide\_synthetase | BGC0001425 | NRP:Cyclic depsipeptide+Polyketide:Iterative type I polyketide | 25.0 | 83.3 | 86.0 | 8.38e-17 |
| QPI18726.1 | nonribosomal\_peptide\_synthetase | BGC0002125 | NRP:Cyclic depsipeptide | 23.0 | 88.2 | 85.0 | 8.4e-17 |
| ALK27916.1 | non-ribosomal\_peptide\_synthase | BGC0001233 | NRP | 24.0 | 87.1 | 86.0 | 8.49e-17 |
| ABI22132.1 | putative\_non-ribosomal\_peptide\_synthetase | BGC0000422 | NRP | 33.0 | 50.7 | 85.0 | 8.61e-17 |
| QEO74981.1 | omn6 | BGC0002078 | NRP:Cyclic depsipeptide | 23.0 | 98.1 | 86.0 | 8.71e-17 |
| EAL85117.1 | acetate-CoA\_ligase,\_putative | BGC0001067 | Terpene+Polyketide:Iterative type I polyketide | 32.0 | 35.2 | 85.0 | 8.88e-17 |
| AJM89738.1 | PmxE | BGC0001192 | NRP | 23.0 | 92.3 | 86.0 | 8.91e-17 |
| AJF34464.1 | Txo2 | BGC0001207 | NRP | 23.0 | 92.9 | 86.0 | 8.92e-17 |
| QEO74982.1 | omn7 | BGC0002078 | NRP:Cyclic depsipeptide | 24.0 | 77.9 | 86.0 | 8.93e-17 |
| AGN11881.1 | tstDEF | BGC0001114 | NRP+Polyketide | 25.0 | 66.9 | 86.0 | 9.02e-17 |
| AIW58892.1 | non-ribosomal\_peptide\_synthetase | BGC0001582 | NRP | 24.0 | 86.8 | 86.0 | 9.2e-17 |
| BAV57443.1 | NRPS\_(C-A-PCP-TE) | BGC0001818 | NRP | 24.0 | 99.8 | 85.0 | 9.45e-17 |
| AAK57184.1 | MxaA | BGC0001022 | NRP+Polyketide | 22.0 | 76.1 | 85.0 | 9.69e-17 |
| CAQ34914.1 | putative\_L-prolyl\_AMP-ligase | BGC0000986 | NRP+Polyketide | 24.0 | 85.0 | 84.0 | 1.01e-16 |
| AHF22854.1 | MarL | BGC0000091 | Polyketide | 27.0 | 76.8 | 85.0 | 1.06e-16 |
| AKA59436.1 | non-ribosomal\_peptide\_synthetase | BGC0001202 | NRP+Polyketide | 26.0 | 66.6 | 85.0 | 1.08e-16 |
| QBA57736.1 | NRPS | BGC0002377 | NRP | 25.0 | 94.9 | 85.0 | 1.08e-16 |
| AAF00957.1 | mcyG | BGC0001017 | NRP+Polyketide:Modular type I polyketide | 26.0 | 69.3 | 85.0 | 1.08e-16 |
| UHJ79953.1 | non-ribosomal\_peptide\_synthetase | BGC0002654 | NRP | 26.0 | 68.1 | 85.0 | 1.11e-16 |
| AEW31022.1 | plipastatin\_synthetase | BGC0000407 | NRP | 24.0 | 76.8 | 85.0 | 1.13e-16 |
| CAL80824.1 | NRPS\_module\_protein | BGC0000997 | NRP+Polyketide | 24.0 | 88.2 | 85.0 | 1.14e-16 |
| AWX24482.1 | non-ribosomal\_peptide\_synthetase | BGC0001695 | NRP | 24.0 | 83.4 | 85.0 | 1.15e-16 |
| ALV86867.1 | Tlo21 | BGC0001406 | NRP | 25.0 | 87.8 | 85.0 | 1.17e-16 |
| CUX96954.1 | TmcG | BGC0001829 | NRP+Polyketide | 26.0 | 52.6 | 85.0 | 1.39e-16 |
| QNL14923.1 | AptC | BGC0002512 | NRP | 24.0 | 71.1 | 85.0 | 1.43e-16 |
| AAG02359.1 | peptide\_synthetase\_NRPS5-4-3 | BGC0000963 | NRP:Glycopeptide+Polyketide:Modular type I polyketide+Saccharide:Hybrid/tailoring saccharide | 27.0 | 72.8 | 85.0 | 1.43e-16 |
| AHZ20784.1 | non-ribosomal\_peptide\_synthase | BGC0000369 | NRP+Saccharide:Hybrid/tailoring saccharide | 24.0 | 77.2 | 85.0 | 1.45e-16 |
| SAI82901.1 | HrnJ;\_Putative\_AMP-dependent\_acyl-CoA\_synthetase/ligase;\_AMP-binding\_enzyme;\_Pfam00501 | BGC0002101 | Polyketide | 26.0 | 78.4 | 84.0 | 1.45e-16 |
| APZ78781.1 | nonribosomal\_peptide\_synthetase | BGC0001426 | NRP:Cyclic depsipeptide+Polyketide:Iterative type I polyketide | 24.0 | 83.3 | 85.0 | 1.46e-16 |
| AAF08795.1 | MycA | BGC0001103 | NRP+Polyketide | 24.0 | 74.4 | 85.0 | 1.5e-16 |
| EWS95124.1 | hypothetical\_protein | BGC0000306 | NRP:Lipopeptide | 24.0 | 88.0 | 85.0 | 1.51e-16 |
| AAZ03552.1 | McnC | BGC0000332 | NRP | 22.0 | 96.3 | 85.0 | 1.52e-16 |
| ACA97576.1 | PmxA | BGC0000408 | NRP | 24.0 | 92.9 | 85.0 | 1.53e-16 |
| AJF34463.1 | Txo1 | BGC0001207 | NRP | 23.0 | 92.9 | 85.0 | 1.55e-16 |
| CDN62030.1 | Peptide\_synthetase | BGC0001599 | NRP | 29.0 | 51.6 | 84.0 | 1.56e-16 |
| ABF87031.1 | non-ribosomal\_peptide\_synthetase/polyketide\_synthase | BGC0000393 | NRP+Polyketide:Modular type I polyketide | 23.0 | 98.3 | 85.0 | 1.6e-16 |
| EAU31031.1 | predicted\_protein | BGC0002193 | Other | 27.0 | 53.7 | 84.0 | 1.86e-16 |
| AAM12931.1 | MupQ | BGC0000182 | Polyketide:Iterative type I polyketide+Polyketide:Trans-AT type I polyketide | 26.0 | 54.7 | 83.0 | 1.87e-16 |
| ALG65319.1 | Cal17 | BGC0001297 | NRP | 25.0 | 78.0 | 84.0 | 1.88e-16 |
| QGU18619.1 | polyketide\_synthase/non-ribosomal\_peptide\_synthetase | BGC0002365 | Other+Polyketide | 24.0 | 94.9 | 84.0 | 1.93e-16 |
| CDG17987.1 | Putative\_Ornithine\_racemase\_(fragment) | BGC0000464 | NRP:Cyclic depsipeptide | 22.0 | 86.1 | 84.0 | 2.01e-16 |
| AEH59100.1 | amino\_acid\_adenylation\_domain-containing\_protein/NRPS | BGC0000385 | NRP | 24.0 | 93.9 | 84.0 | 2.07e-16 |
| ACK77757.1 | putative\_indigoidine\_synthase\_IndC | BGC0000727 | Saccharide | 23.0 | 86.8 | 84.0 | 2.12e-16 |
| ALV82388.1 | CDA\_peptide\_synthetase\_III | BGC0001370 | NRP | 25.0 | 93.0 | 84.0 | 2.45e-16 |
| EFY95969.1 | polyketide\_synthase | BGC0002270 | NRP+Polyketide | 24.0 | 74.6 | 84.0 | 2.47e-16 |
| APZ78743.1 | nonribosomal\_peptide\_synthetase | BGC0001422 | NRP:Cyclic depsipeptide+Polyketide:Iterative type I polyketide | 24.0 | 81.4 | 84.0 | 2.54e-16 |
| AAF15891.2 | nosA | BGC0001028 | Polyketide+NRP:Cyclic depsipeptide | 21.0 | 89.4 | 84.0 | 2.63e-16 |
| AHB38497.1 | non-ribosomal\_peptide\_synthetase | BGC0000346 | NRP+Polyketide:Modular type I polyketide | 27.0 | 69.7 | 84.0 | 2.66e-16 |
| QWT72292.1 | putative\_non-ribosomal\_peptide\_synthetase | BGC0002430 | NRP+Saccharide | 25.0 | 94.9 | 84.0 | 2.68e-16 |
| AXN93602.1 | PuwF-G | BGC0001952 | NRP | 23.0 | 85.2 | 84.0 | 2.68e-16 |
| CAB38518.1 | CDA\_peptide\_synthetase\_I\_(CdaPs1) | BGC0000315 | NRP:Lipopeptide:Ca+-dependent lipopeptide | 24.0 | 83.6 | 84.0 | 2.71e-16 |
| AZH29360.1 | amino\_acid\_adenylation\_domain-containing\_protein | BGC0001843 | NRP | 23.0 | 82.8 | 84.0 | 2.77e-16 |
| QBA57735.1 | NRPS | BGC0002377 | NRP | 25.0 | 88.3 | 84.0 | 3.27e-16 |
| WP\_050383088.1 | non-ribosomal\_peptide\_synthetase | BGC0001451 | NRP | 24.0 | 76.3 | 83.0 | 3.37e-16 |
| AGP37410.1 | peptide\_synthetase | BGC0002386 | NRP+Polyketide | 24.0 | 94.9 | 84.0 | 3.41e-16 |
| AHB82058.1 | non\_ribosomal\_peptide\_synthetase | BGC0001019 | NRP+Polyketide:Modular type I polyketide | 26.0 | 94.1 | 84.0 | 3.45e-16 |
| CAD17793.1 | probable\_non\_ribosomal\_peptide\_synthetase\_protein | BGC0001363 | NRP+Polyketide | 23.0 | 84.1 | 84.0 | 3.54e-16 |
| ATO51563.1 | non-ribosomal\_peptide\_synthetase | BGC0001796 | NRP | 22.0 | 82.2 | 84.0 | 3.55e-16 |
| BBA20967.1 | nonribosomal\_peptide\_synthetase | BGC0001763 | NRP+Polyketide | 30.0 | 57.1 | 84.0 | 3.59e-16 |
| ASX95241.1 | IlaS | BGC0001620 | NRP+Polyketide | 30.0 | 57.1 | 84.0 | 3.59e-16 |
| CCA89326.1 | mixed\_trans-AT\_type\_I\_polyketide\_synthase/nonribosomal\_peptide\_synthetase | BGC0001111 | NRP+Polyketide:Trans-AT type I polyketide | 27.0 | 52.1 | 84.0 | 3.64e-16 |
| AWS21283.1 | acyl-CoA\_synthetase | BGC0001934 | Polyketide | 25.0 | 75.4 | 82.0 | 3.97e-16 |
| AZY91992.1 | putative\_fatty\_acyl-AMP\_ligase | BGC0002022 | Polyketide | 25.0 | 75.4 | 82.0 | 3.97e-16 |
| AAM80539.1 | StaA | BGC0000290 | NRP:Glycopeptide | 25.0 | 91.8 | 83.0 | 4.16e-16 |
| QRG35013.1 | NRPS | BGC0002378 | NRP | 24.0 | 91.5 | 83.0 | 4.41e-16 |
| AIW82284.1 | PuwG | BGC0001125 | NRP+Polyketide | 23.0 | 78.4 | 83.0 | 4.47e-16 |
| CAD91212.1 | putative\_non-ribosomal\_peptide\_synthetase,\_modules\_4-6 | BGC0000289 | NRP:Glycopeptide+Saccharide:Hybrid/tailoring saccharide | 25.0 | 84.8 | 83.0 | 4.54e-16 |
| ACO78737.1 | Non-ribosomal\_peptide\_synthase,\_PvdD/PvdJ-like\_protein | BGC0002433 | NRP | 26.0 | 67.9 | 83.0 | 4.62e-16 |
| AGN74885.1 | nonribosomal\_peptide\_synthetase | BGC0000459 | NRP:Cyclic depsipeptide+Polyketide:Trans-AT type I polyketide | 25.0 | 87.3 | 83.0 | 4.65e-16 |
| AEZ51520.1 | pmxE | BGC0001153 | NRP:Lipopeptide | 23.0 | 92.3 | 83.0 | 4.68e-16 |
| AAX31558.1 | peptide\_synthetase\_2 | BGC0000336 | NRP | 24.0 | 94.9 | 83.0 | 4.71e-16 |
| ABQ96384.2 | fusaricidin\_synthetase | BGC0001152 | Polyketide+NRP:Lipopeptide | 23.0 | 79.3 | 83.0 | 4.73e-16 |
| DAD54486.1 | trans-acyltransferase\_polyketide\_synthase | BGC0002059 | NRP+Polyketide:Trans-AT type I polyketide | 26.0 | 67.6 | 83.0 | 4.74e-16 |
| QSJ20135.1 | non-ribosomal\_peptide\_synthase/polyketide\_synthase | BGC0002572 | NRP+Polyketide | 21.0 | 97.0 | 83.0 | 4.8e-16 |
| UPA71926.1 | long-chain-fatty-acid--AMP\_ligase\_FadD26 | BGC0002636 | Polyketide | 27.0 | 73.9 | 82.0 | 5.09e-16 |
| CAE53350.1 | non-ribosomal\_peptide\_synthetase | BGC0000440 | NRP:Glycopeptide | 29.0 | 59.8 | 83.0 | 5.49e-16 |
| CAG15009.1 | peptide\_synthetase,\_module\_1-2 | BGC0000441 | NRP | 29.0 | 59.8 | 83.0 | 5.49e-16 |
| QTT72097.1 | amino\_acid\_adenylation\_domain-containing\_protein | BGC0002350 | NRP+Polyketide+Saccharide | 27.0 | 71.3 | 83.0 | 5.71e-16 |
| AXN93614.1 | PuwF | BGC0001953 | NRP | 22.0 | 86.4 | 83.0 | 5.77e-16 |
| AQZ69228.1 | hypothetical\_protein | BGC0001635 | NRP+Polyketide | 24.0 | 91.1 | 83.0 | 5.92e-16 |
| CAB38517.1 | CDA\_peptide\_synthetase\_II\_(CdaPs2) | BGC0000315 | NRP:Lipopeptide:Ca+-dependent lipopeptide | 24.0 | 80.3 | 83.0 | 5.92e-16 |
| AIE77059.1 | peptide\_synthetase | BGC0000418 | NRP | 25.0 | 85.4 | 83.0 | 5.97e-16 |
| BAR73008.1 | putative\_ATP-dependent\_b-aminoacyl-ACP\_synthetase | BGC0001194 | Polyketide | 25.0 | 66.9 | 82.0 | 5.98e-16 |
| ABY83142.1 | Azi3 | BGC0000960 | NRP+Polyketide | 28.0 | 70.9 | 82.0 | 6.07e-16 |
| AXN93581.1 | PuwF-G | BGC0001950 | NRP | 23.0 | 77.4 | 83.0 | 6.14e-16 |
| QBC75021.1 | non-ribosomal\_peptide\_synthetase | BGC0001968 | NRP | 23.0 | 89.4 | 83.0 | 6.21e-16 |
| WP\_050383094.1 | non-ribosomal\_peptide\_synthetase | BGC0001451 | NRP | 24.0 | 76.3 | 82.0 | 6.37e-16 |
| ABS74205.1 | fengycin\_synthetase\_E | BGC0001095 | NRP | 25.0 | 56.1 | 82.0 | 6.39e-16 |
| QCQ67881.1 | non-ribosomal\_peptide\_synthetase | BGC0002297 | NRP+Polyketide | 22.0 | 79.6 | 82.0 | 6.44e-16 |
| CAN89633.1 | putative\_hybrid\_non-ribosomal\_peptide\_synthetase/polyketide\_synthase | BGC0001070 | NRP+Polyketide:Modular type I polyketide+Polyketide:Trans-AT type I polyketide | 26.0 | 75.4 | 82.0 | 6.78e-16 |
| AAX31555.1 | acyl-CoA\_ligase | BGC0000336 | NRP | 28.0 | 58.4 | 82.0 | 6.93e-16 |
| APZ78855.1 | nonribosomal\_peptide\_synthetase | BGC0001432 | NRP:Cyclic depsipeptide+Polyketide:Iterative type I polyketide | 24.0 | 84.0 | 82.0 | 7.66e-16 |
| AAY93356.2 | non-ribosomal\_peptide\_synthetase\_PvdI | BGC0000413 | NRP | 21.0 | 93.9 | 82.0 | 7.82e-16 |
| WP\_054234643.1 | non-ribosomal\_peptide\_synthetase | BGC0002014 | NRP+Polyketide | 24.0 | 80.0 | 82.0 | 7.84e-16 |
| AFP87519.1 | proline\_adenyltransferase | BGC0001159 | NRP+Polyketide:Modular type I polyketide | 24.0 | 64.3 | 81.0 | 8.14e-16 |
| KJY85279.1 | long-chain\_fatty\_acid--CoA\_ligase | BGC0002491 | NRP | 24.0 | 66.9 | 82.0 | 8.78e-16 |
| AGU50950.1 | putative\_non-ribosomal\_peptide\_synthetase | BGC0002417 | NRP+Polyketide | 23.0 | 88.3 | 82.0 | 9.9e-16 |
| ATU31795.1 | NRPS | BGC0001814 | NRP | 25.0 | 96.3 | 82.0 | 9.92e-16 |
| AQM37583.1 | nonribosomal\_peptide\_synthetase | BGC0001424 | NRP:Cyclic depsipeptide+Polyketide:Iterative type I polyketide | 24.0 | 81.4 | 82.0 | 1.01e-15 |
| BAE98156.1 | putative\_non-ribosomal\_peptide\_synthetase | BGC0000339 | NRP | 27.0 | 65.2 | 82.0 | 1.01e-15 |
| AAT09804.1 | NocA | BGC0000395 | NRP | 31.0 | 53.1 | 82.0 | 1.03e-15 |
| QUF98525.1 | non-ribosomal\_peptide\_synthetase | BGC0002582 | NRP | 26.0 | 60.3 | 82.0 | 1.04e-15 |
| AHF21228.1 | TriD | BGC0000449 | NRP | 22.0 | 82.4 | 82.0 | 1.1e-15 |
| OLZ52442.1 | non-ribosomal\_peptide\_synthetase | BGC0001462 | NRP:Glycopeptide | 25.0 | 74.0 | 81.0 | 1.17e-15 |
| AEF16021.1 | acyl-CoA\_synthase | BGC0000379 | NRP | 26.0 | 73.7 | 81.0 | 1.24e-15 |
| AIW82283.1 | PuwF | BGC0001125 | NRP+Polyketide | 23.0 | 78.4 | 82.0 | 1.29e-15 |
| ABD14712.1 | cesB | BGC0000320 | NRP:Cyclic depsipeptide | 23.0 | 90.4 | 82.0 | 1.31e-15 |
| APZ78845.1 | nonribosomal\_peptide\_synthetase | BGC0001431 | NRP:Cyclic depsipeptide+Polyketide:Iterative type I polyketide | 23.0 | 82.8 | 82.0 | 1.33e-15 |
| CRG85572.1 | nonribosomal\_peptide\_synthase,\_putative | BGC0001402 | NRP | 27.0 | 87.3 | 82.0 | 1.4e-15 |
| AAC06348.1 | bacitracin\_synthetase\_3 | BGC0000310 | NRP | 23.0 | 87.3 | 82.0 | 1.41e-15 |
| UKO95761.1 | amino\_acid\_adenylation\_domain-containing\_protein | BGC0002632 | NRP | 24.0 | 88.2 | 81.0 | 1.42e-15 |
| CBJ90359.1 | putative\_Peptide\_synthetase | BGC0000465 | NRP | 24.0 | 77.7 | 81.0 | 1.5e-15 |
| CAB15186.3 | siderophore\_2,3-dihydroxybenzoate-glycine-threonine\_trimeric\_ester\_bacillibactin\_synthetase | BGC0000309 | NRP | 22.0 | 80.1 | 81.0 | 1.7e-15 |
| QCQ67877.1 | hybrid\_peptide\_synthetase/polyketide\_synthase | BGC0002297 | NRP+Polyketide | 25.0 | 68.8 | 81.0 | 1.72e-15 |
| CAJ34375.1 | NRPS | BGC0000445 | NRP:Cyclic depsipeptide | 27.0 | 58.7 | 81.0 | 1.76e-15 |
| AET98905.1 | putative\_non-ribosomal\_peptide\_synthetase | BGC0000415 | NRP | 26.0 | 57.1 | 81.0 | 1.76e-15 |
| AAU39359.1 | lichenysin\_synthase\_LchAA | BGC0000381 | NRP | 25.0 | 77.5 | 81.0 | 1.78e-15 |
| CCJ67647.1 | JagC | BGC0001127 | NRP | 22.0 | 73.9 | 81.0 | 1.79e-15 |
| QBC75022.1 | non-ribosomal\_peptide\_synthetase | BGC0001968 | NRP | 24.0 | 90.1 | 81.0 | 1.85e-15 |
| CBL93730.1 | NRPS | BGC0000360 | NRP | 25.0 | 52.6 | 81.0 | 1.9e-15 |
| AAG02358.1 | peptide\_synthetase\_NRPS6 | BGC0000963 | NRP:Glycopeptide+Polyketide:Modular type I polyketide+Saccharide:Hybrid/tailoring saccharide | 28.0 | 65.3 | 81.0 | 1.91e-15 |
| NPC94426.1 | amino\_acid\_adenylation\_domain-containing\_protein | BGC0002695 | NRP | 23.0 | 86.8 | 81.0 | 2.23e-15 |
| AAC44129.1 | saframycin\_Mx1\_synthetase\_A | BGC0002706 | NRP | 23.0 | 94.8 | 81.0 | 2.26e-15 |
| ATJ04409.1 | NRPS | BGC0001637 | NRP | 28.0 | 55.6 | 80.0 | 2.27e-15 |
| AJW76709.1 | DsaG | BGC0001196 | NRP | 23.0 | 92.7 | 81.0 | 2.27e-15 |
| EPH46596.1 | putative\_Linear\_gramicidin\_synthase\_subunit\_C | BGC0001519 | NRP+Polyketide | 25.0 | 67.1 | 81.0 | 2.29e-15 |
| CEK23366.1 | putative\_Ornithine\_racemase | BGC0001716 | NRP | 23.0 | 80.3 | 81.0 | 2.33e-15 |
| DAD54574.1 | NRPS-like\_oxidoreductase | BGC0002256 | NRP+Other | 24.0 | 67.6 | 81.0 | 2.35e-15 |
| AGO86662.1 | equisetin\_synthetase | BGC0001255 | NRP+Polyketide | 24.0 | 86.9 | 81.0 | 2.37e-15 |
| PHM26612.1 | pvdj | BGC0001130 | NRP+Polyketide | 23.0 | 92.5 | 81.0 | 2.39e-15 |
| AEU11003.1 | NpnC | BGC0001029 | NRP+Polyketide | 21.0 | 83.8 | 81.0 | 2.42e-15 |
| ABL74939.1 | NRPS | BGC0001048 | NRP:Glycopeptide+Polyketide:Modular type I polyketide+Saccharide:Hybrid/tailoring saccharide | 25.0 | 66.6 | 81.0 | 2.46e-15 |
| BAP05597.1 | calI | BGC0000967 | NRP+Polyketide:Trans-AT type I polyketide | 25.0 | 79.3 | 81.0 | 2.99e-15 |
| BAH04161.1 | putative\_non-ribosomal\_peptide\_synthetase | BGC0000450 | NRP | 26.0 | 57.1 | 81.0 | 3.06e-15 |
| DAB41477.1 | nonribosomal\_peptide\_synthetase | BGC0001230 | NRP:Cyclic depsipeptide+Polyketide:Modular type I polyketide | 26.0 | 74.4 | 81.0 | 3.13e-15 |
| AFR69334.1 | nonribosomal\_peptide\_synthetase\_SpiDE1 | BGC0001045 | NRP:Cyclic depsipeptide+Polyketide:Modular type I polyketide | 25.0 | 61.7 | 81.0 | 3.13e-15 |
| AID65222.1 | putative\_aspartate\_racemase | BGC0000335 | NRP+Polyketide | 24.0 | 85.7 | 81.0 | 3.14e-15 |
| ATW47208.1 | non-ribosomal\_peptide\_synthetase | BGC0002466 | NRP | 24.0 | 92.0 | 81.0 | 3.19e-15 |
| BAV56270.1 |  | BGC0001657 | NRP | 25.0 | 84.3 | 81.0 | 3.28e-15 |
| QMS47800.1 | JesC | BGC0001629 | NRP:Lipopeptide | 24.0 | 86.2 | 81.0 | 3.29e-15 |
| AAG02343.1 | peptide\_synthetase\_NRPS12 | BGC0000963 | NRP:Glycopeptide+Polyketide:Modular type I polyketide+Saccharide:Hybrid/tailoring saccharide | 26.0 | 65.9 | 79.0 | 3.6e-15 |
| QTT72111.1 | non-ribosomal\_peptide\_synthetase | BGC0002350 | NRP+Polyketide+Saccharide | 26.0 | 65.9 | 79.0 | 3.6e-15 |
| AFK57213.1 | DidB | BGC0000985 | Polyketide+NRP:Cyclic depsipeptide | 26.0 | 72.8 | 80.0 | 3.69e-15 |
| AAY37650.1 | Amino\_acid\_adenylation | BGC0000437 | NRP | 22.0 | 86.9 | 79.0 | 3.84e-15 |
| ATY37589.1 | BogB | BGC0001532 | NRP | 21.0 | 93.6 | 80.0 | 3.92e-15 |
| ACO78736.1 | Non-ribosomal\_peptide\_synthase,\_PvdD-like\_protein | BGC0002433 | NRP | 24.0 | 69.3 | 80.0 | 3.98e-15 |
| AHD05615.1 | putative\_non-ribosomal\_peptide\_ligase/\_polyketide\_synthase\_hybrid | BGC0001033 | NRP+Polyketide | 21.0 | 91.3 | 80.0 | 4e-15 |
| WP\_069848010.1 | non-ribosomal\_peptide\_synthetase | BGC0002472 | NRP | 24.0 | 62.7 | 80.0 | 4.01e-15 |
| KYQ85937.1 | hypothetical\_protein | BGC0002437 | NRP | 21.0 | 94.3 | 80.0 | 4.07e-15 |
| BAX90000.1 | Non-ribosomal\_peptide\_synthetase | BGC0001628 | NRP | 24.0 | 84.0 | 80.0 | 4.1e-15 |
| AIG79241.1 | Hypothetical\_protein | BGC0000419 | Saccharide+NRP:Glycopeptide | 25.0 | 85.7 | 80.0 | 4.12e-15 |
| AXN93590.1 | PuwF-G | BGC0001951 | NRP | 23.0 | 77.4 | 80.0 | 4.23e-15 |
| CBG75492.1 | putative\_NRPS/siderophore\_biosynthesis\_protein | BGC0000423 | NRP | 23.0 | 91.1 | 80.0 | 4.25e-15 |
| ABW00331.1 | amino\_acid\_adenylation\_domain | BGC0000333 | NRP | 26.0 | 67.4 | 80.0 | 4.29e-15 |
| ABI22133.1 | putative\_non-ribosomal\_peptide\_synthetase | BGC0000422 | NRP | 24.0 | 86.4 | 80.0 | 4.65e-15 |
| AQW44894.1 | non-ribosomal\_peptide\_synthetase | BGC0001737 | NRP+Polyketide | 24.0 | 93.7 | 80.0 | 4.68e-15 |
| ADH01485.1 | putative\_mixed\_polyketide\_synthase/non-ribosomal\_peptide\_synthetase | BGC0000995 | NRP+Polyketide | 23.0 | 69.3 | 80.0 | 4.85e-15 |
| WP\_102918835.1 | amino\_acid\_adenylation\_domain-containing\_protein | BGC0002104 | NRP+Polyketide | 24.0 | 74.4 | 79.0 | 5.02e-15 |
| AET98906.1 | putative\_non-ribosomal\_peptide\_synthetase | BGC0000415 | NRP | 24.0 | 97.0 | 80.0 | 5.2e-15 |
| AAU39360.1 | lichenysin\_synthase\_LchAB | BGC0000381 | NRP | 24.0 | 79.4 | 80.0 | 5.37e-15 |
| WA1\_15565 | non-ribosomal\_peptide\_synthetase | BGC0002484 | NRP+Polyketide | 21.0 | 94.4 | 80.0 | 5.4e-15 |
| AIC32693.1 | FR9DEF | BGC0001113 | NRP+Polyketide | 23.0 | 69.3 | 80.0 | 5.65e-15 |
| ACO94461.1 | putative\_AMP-dependent\_acyl-CoA\_synthetase/ligase | BGC0000029 | Polyketide:Modular type I polyketide | 25.0 | 78.7 | 79.0 | 5.69e-15 |
| WP\_078586793.1 | non-ribosomal\_peptide\_synthetase | BGC0001760 | NRP | 26.0 | 67.1 | 79.0 | 6.03e-15 |
| ADZ45333.1 | NRPS/PKS\_hybrid | BGC0001020 | NRP+Polyketide | 24.0 | 93.4 | 79.0 | 6.32e-15 |
| CBW54660.1 | Acyl-CoA\_ligase | BGC0000971 | NRP+Polyketide:Modular type I polyketide | 27.0 | 73.2 | 79.0 | 6.45e-15 |
| QUJ09166.1 | Lon19 | BGC0002440 | NRP | 24.0 | 100.0 | 79.0 | 6.63e-15 |
| AGZ15458.1 | putative\_non-ribosomal\_peptide\_synthetase | BGC0001036 | NRP+Polyketide | 25.0 | 65.9 | 79.0 | 6.82e-15 |
| AHD05621.1 | non-ribosomal\_peptide\_ligase\_domain\_protein | BGC0001033 | NRP+Polyketide | 23.0 | 86.8 | 79.0 | 6.87e-15 |
| CDG76957.1 | non-ribosomal\_peptide\_synthetase | BGC0000446 | NRP:Pyrrolobenzodiazepine | 29.0 | 55.7 | 78.0 | 7.02e-15 |
| QUS58938.1 | amino\_acid\_adenylation\_domain-containing\_protein | BGC0002123 | NRP+Polyketide | 26.0 | 78.0 | 79.0 | 7.06e-15 |
| CAG23957.2 | hybrid\_NRPS/PKS\_protein | BGC0001089 | Polyketide+NRP | 23.0 | 68.5 | 79.0 | 7.27e-15 |
| AAF08796.1 | MycB | BGC0001103 | NRP+Polyketide | 23.0 | 73.3 | 79.0 | 7.3e-15 |
| DAC80524.1 | peptide\_synthetase | BGC0001841 | NRP+Polyketide | 24.0 | 77.4 | 79.0 | 7.71e-15 |
| ACZ55946.1 | non-ribosomal\_peptide\_synthetase | BGC0000302 | NRP | 23.0 | 72.0 | 79.0 | 7.96e-15 |
| EAU38971.1 | PKS-NRPS\_hybrid | BGC0001122 | NRP+Polyketide:Iterative type I polyketide | 22.0 | 84.1 | 79.0 | 9.39e-15 |
| WP\_126241403.1 | non-ribosomal\_peptide\_synthetase | BGC0002336 | NRP | 22.0 | 93.4 | 79.0 | 9.51e-15 |
| AXG47007.1 | non-ribosomal\_peptide\_synthetase | BGC0000383 | NRP+Polyketide:Modular type I polyketide | 21.0 | 88.2 | 79.0 | 9.56e-15 |
| WP\_245566645.1 | amino\_acid\_adenylation\_domain-containing\_protein | BGC0002467 | NRP | 23.0 | 91.6 | 79.0 | 9.63e-15 |
| CBF87869.1 | nonribosomal\_peptide\_synthase,\_putative\_(Eurofung) | BGC0001699 | NRP | 25.0 | 56.6 | 79.0 | 9.68e-15 |
| AAS47562.1 | mixed\_type\_I\_polyketide\_synthase\_-\_peptide\_synthetase | BGC0001108 | NRP+Polyketide:Trans-AT type I polyketide | 24.0 | 68.1 | 79.0 | 9.7e-15 |
| ctg1\_orf8 |  | BGC0001109 | NRP+Polyketide | 24.0 | 68.1 | 79.0 | 9.7e-15 |
| ABU70377.1 | hypothetical\_protein | BGC0001890 | NRP | 25.0 | 81.9 | 79.0 | 1.03e-14 |
| DAB41914.1 | ArzL\_-\_PKS\_(FAAL,\_ACP) | BGC0001884 | NRP+Polyketide | 27.0 | 72.6 | 78.0 | 1.06e-14 |
| CAD55498.1 | CDA\_peptide\_synthetase\_III\_(CdaPs3) | BGC0000315 | NRP:Lipopeptide:Ca+-dependent lipopeptide | 24.0 | 92.0 | 79.0 | 1.17e-14 |
| WP\_080679150.1 | non-ribosomal\_peptide\_synthetase | BGC0001228 | NRP:Cyclic depsipeptide | 27.0 | 71.1 | 79.0 | 1.18e-14 |
| AWS21276.1 | amino\_acid\_adenyltransferase | BGC0001934 | Polyketide | 24.0 | 65.3 | 77.0 | 1.19e-14 |
| AZY92000.1 | proline\_adenylation\_protein | BGC0002022 | Polyketide | 24.0 | 65.3 | 77.0 | 1.19e-14 |
| SDF67417.1 | amino\_acid\_adenylation\_domain-containing\_protein | BGC0002422 | NRP | 25.0 | 50.3 | 79.0 | 1.21e-14 |
| BAI63289.1 | putative\_non-ribosomal\_peptide\_synthetase | BGC0000434 | NRP | 26.0 | 66.6 | 79.0 | 1.21e-14 |
| CAL80821.1 | sylD-like\_NRPS/PKS | BGC0000997 | NRP+Polyketide | 23.0 | 76.5 | 79.0 | 1.24e-14 |
| BAH22764.1 | nonribosomal\_peptide\_synthetase | BGC0001018 | NRP | 22.0 | 96.5 | 79.0 | 1.26e-14 |
| WP\_019634550.1 | type\_I\_polyketide\_synthase | BGC0001443 | NRP+Polyketide | 26.0 | 70.7 | 78.0 | 1.42e-14 |
| CAE52334.1 | non-ribosomal\_peptide\_synthase | BGC0001088 | NRP+Polyketide | 25.0 | 55.6 | 78.0 | 1.49e-14 |
| AAP92491.1 | nonribosomal\_peptide\_synthetase | BGC0000458 | NRP | 24.0 | 102.4 | 78.0 | 1.51e-14 |
| AAO62586.1 | peptide\_sythetase | BGC0001016 | NRP+Polyketide | 22.0 | 63.9 | 78.0 | 1.57e-14 |
| AEH42484.1 | adenylation\_for\_L-proline | BGC0000032 | Polyketide | 23.0 | 93.0 | 77.0 | 1.58e-14 |
| QNS30807.1 | hybrid\_non-ribosomal\_peptide\_synthetase/type\_I\_polyketide\_syn-thase | BGC0002509 | NRP | 25.0 | 66.0 | 78.0 | 1.6e-14 |
| AEG64696.1 | LpmB | BGC0000379 | NRP | 23.0 | 85.0 | 78.0 | 1.6e-14 |
| ABX37383.1 | amino\_acid\_adenylation\_domain\_protein | BGC0000984 | NRP+Polyketide | 26.0 | 67.9 | 78.0 | 1.61e-14 |
| AWO77084.1 | hybrid\_non-ribosomal\_peptide\_synthetase/type\_I\_polyketide\_synthase | BGC0001556 | NRP+Polyketide | 27.0 | 66.0 | 78.0 | 1.61e-14 |
| CAE02631.1 | surfactin\_synthetase\_B\_ | BGC0000433 | NRP:Lipopeptide | 25.0 | 66.7 | 78.0 | 1.62e-14 |
| CAL17541.1 | peptide\_synthetase,\_putative | BGC0002465 | NRP | 21.0 | 91.3 | 78.0 | 1.62e-14 |
| AZM57022.1 | non-ribosomal\_peptide\_synthetase | BGC0002314 | NRP | 25.0 | 77.2 | 78.0 | 1.62e-14 |
| CBJ79915.1 | putative\_Phenylalanine\_racemase\_(ATP-hydrolyzing) | BGC0001133 | NRP | 24.0 | 67.4 | 78.0 | 1.63e-14 |
| CBJ79916.1 | putative\_Ornithine\_racemase | BGC0001133 | NRP | 24.0 | 67.4 | 78.0 | 1.65e-14 |
| CUX79061.1 | Octapeptin\_synthase\_subunit\_B | BGC0001715 | NRP | 23.0 | 74.6 | 78.0 | 1.66e-14 |
| AAY91421.3 | non-ribosomal\_peptide\_synthetase\_OfaC | BGC0000399 | NRP:Cyclic depsipeptide | 25.0 | 89.7 | 78.0 | 1.66e-14 |
| KPN93065.1 | NunD | BGC0001416 | NRP | 25.0 | 73.2 | 78.0 | 1.66e-14 |
| mycF | polyketide\_synthase | BGC0002055 | NRP+Polyketide:Trans-AT type I polyketide | 25.0 | 91.1 | 78.0 | 1.71e-14 |
| CAJ34381.1 | NRPS\_protein | BGC0000445 | NRP:Cyclic depsipeptide | 23.0 | 94.9 | 78.0 | 1.94e-14 |
| QIE08737.1 | non-ribosomal\_peptide\_synthetase | BGC0002544 | NRP | 24.0 | 88.7 | 78.0 | 2.03e-14 |
| ABC36203.1 | Gonyol-Synthetase\_(NRPS-PKS\_hybrid) | BGC0001102 | NRP+Polyketide:Modular type I polyketide+Polyketide:Trans-AT type I polyketide | 23.0 | 83.6 | 78.0 | 2.07e-14 |
| AJD77023.1 | IkaA | BGC0001435 | NRP+Polyketide:Iterative type I polyketide | 23.0 | 95.6 | 78.0 | 2.1e-14 |
| AZM51139.1 | non-ribosomal\_peptide\_synthetase | BGC0002702 | NRP | 25.0 | 65.5 | 78.0 | 2.11e-14 |
| AKJ75110.1 | Bmp4 | BGC0001464 | Other | 23.0 | 64.3 | 77.0 | 2.12e-14 |
| AXA20090.1 | hybrid\_trans-AT\_PKS/NRPS\_LgaA | BGC0001646 | NRP+Polyketide | 25.0 | 84.8 | 78.0 | 2.13e-14 |
| AJQ95677.1 | polyketide\_synthase\_modules-related\_protein | BGC0002046 | NRP+Polyketide:Trans-AT type I polyketide | 24.0 | 78.4 | 78.0 | 2.13e-14 |
| QED55421.1 | nonribosomal\_peptide\_synthetase | BGC0001984 | NRP | 26.0 | 81.2 | 78.0 | 2.15e-14 |
| AJD47484.1 | protein\_PvdD | BGC0002418 | NRP+Polyketide | 27.0 | 57.3 | 78.0 | 2.15e-14 |
| QNH85840.1 | BolH | BGC0002327 | NRP | 22.0 | 95.6 | 78.0 | 2.17e-14 |
| ABI26078.1 | OciB | BGC0000331 | NRP | 22.0 | 96.5 | 78.0 | 2.18e-14 |
| CBG67541.1 | putative\_non-ribosomal\_peptide\_synthetase | BGC0002367 | NRP | 23.0 | 89.4 | 78.0 | 2.2e-14 |
| ESP90847.1 | amino\_acid\_adenylation\_domain\_protein | BGC0000891 | Other:Aminocoumarin | 23.0 | 51.4 | 77.0 | 2.24e-14 |
| QTT72098.1 | non-ribosomal\_peptide\_synthetase | BGC0002350 | NRP+Polyketide+Saccharide | 27.0 | 65.3 | 77.0 | 2.3e-14 |
| FIS9431\_RS32925 | non-ribosomal\_peptide\_synthetase | BGC0001467 | NRP:Cyclic depsipeptide+Polyketide:Modular type I polyketide | 22.0 | 84.1 | 77.0 | 2.44e-14 |
| sipL1 | AMP-dependent\_synthetase\_and\_ligase | BGC0001452 | Polyketide | 26.0 | 69.0 | 77.0 | 2.47e-14 |
| AEZ53969.1 | putative\_AMP-binding\_domain-containing\_protein | BGC0000144 | Polyketide:Modular type I polyketide | 29.0 | 57.7 | 77.0 | 2.65e-14 |
| AHD05614.1 | putative\_non-ribosomal\_peptide\_ligase/\_polyketide\_synthase\_hybrid | BGC0001033 | NRP+Polyketide | 23.0 | 82.9 | 77.0 | 2.71e-14 |
| AAF08797.1 | MycC | BGC0001103 | NRP+Polyketide | 22.0 | 87.1 | 77.0 | 2.71e-14 |
| BCK51628.1 | non-ribosomal\_peptide\_synthetase | BGC0002520 | Polyketide | 25.0 | 55.6 | 77.0 | 2.71e-14 |
| MBX9445647.1 | amino\_acid\_adenylation\_domain-containing\_protein | BGC0002414 | NRP | 24.0 | 73.7 | 77.0 | 2.74e-14 |
| AHZ20773.1 | non-ribosomal\_peptide\_synthase | BGC0000369 | NRP+Saccharide:Hybrid/tailoring saccharide | 22.0 | 87.3 | 77.0 | 2.76e-14 |
| APZ78833.1 | nonribosomal\_peptide\_synthetase | BGC0001430 | NRP:Cyclic depsipeptide+Polyketide:Iterative type I polyketide | 23.0 | 81.9 | 77.0 | 2.76e-14 |
| BAG17643.1 | putative\_NRPS-type-I\_PKS\_fusion\_protein | BGC0001043 | NRP+Polyketide | 24.0 | 95.1 | 77.0 | 2.77e-14 |
| AKP45396.1 | CysH | BGC0001413 | NRP | 27.0 | 61.8 | 77.0 | 2.79e-14 |
| AJV88377.1 | MfnE | BGC0001214 | NRP | 24.0 | 79.8 | 77.0 | 2.81e-14 |
| ABC36785.1 | peptide\_synthetase,\_putative | BGC0000964 | NRP:Cyclic depsipeptide+Polyketide:Trans-AT type I polyketide | 25.0 | 66.4 | 77.0 | 2.81e-14 |
| AWI62626.1 | nonribosomal\_peptide\_synthetase | BGC0001822 | NRP | 26.0 | 74.4 | 77.0 | 2.88e-14 |
| QDJ74275.1 | non-ribosomal\_peptide\_synthetase | BGC0002109 | NRP | 26.0 | 92.2 | 77.0 | 2.88e-14 |
| ctg1\_orf20 |  | BGC0001767 | NRP | 23.0 | 74.4 | 77.0 | 2.89e-14 |
| AIS24862.1 | dst18 | BGC0001147 | NRP | 26.0 | 79.8 | 77.0 | 2.89e-14 |
| WP\_100939443.1 | non-ribosomal\_peptide\_synthetase | BGC0002071 | NRP:Lipopeptide | 24.0 | 92.2 | 77.0 | 2.91e-14 |
| QEO75074.1 | condensation\_domain-containing\_protein | BGC0002079 | NRP:Cyclic depsipeptide | 26.0 | 75.8 | 77.0 | 3.09e-14 |
| ALG65318.1 | Cal18 | BGC0001297 | NRP | 27.0 | 57.0 | 77.0 | 3.56e-14 |
| QDJ74293.1 | peptide\_synthetase | BGC0002109 | NRP | 26.0 | 69.9 | 77.0 | 3.58e-14 |
| AAD24881.1 | putative\_acyl-CoA\_synthetase | BGC0000127 | Polyketide | 21.0 | 86.8 | 76.0 | 3.73e-14 |
| QYC40289.1 | A50926\_NRPS,\_modules\_4-5-6 | BGC0002344 | NRP | 25.0 | 85.9 | 77.0 | 3.74e-14 |
| AZM57024.1 | non-ribosomal\_peptide\_synthetase | BGC0002314 | NRP | 26.0 | 67.6 | 77.0 | 3.78e-14 |
| CBK62721.1 |  | BGC0001115 | NRP+Polyketide | 25.0 | 61.0 | 76.0 | 4.1e-14 |
| AAF86395.1 | FkbP | BGC0000994 | NRP+Polyketide | 23.0 | 102.4 | 77.0 | 4.25e-14 |
| AHZ34240.1 | CipC | BGC0001389 | NRP | 25.0 | 72.5 | 77.0 | 4.28e-14 |
| ABR67744.1 | CmnA | BGC0000316 | NRP | 24.0 | 78.9 | 77.0 | 4.65e-14 |
| ANZ15839.1 | peptide\_synthetase\_ScpsB | BGC0001569 | NRP | 25.0 | 75.6 | 77.0 | 4.71e-14 |
| ADZ24995.1 | non-ribosomal\_peptide\_synthase/polyketide\_synthase | BGC0000380 | NRP+Polyketide:Modular type I polyketide | 22.0 | 84.7 | 77.0 | 4.78e-14 |
| PVC99865.1 | non-ribosomal\_peptide\_synthetase | BGC0002100 | NRP+Other | 25.0 | 91.3 | 77.0 | 4.88e-14 |
| OKA09424.1 | non-ribosomal\_peptide\_synthetase | BGC0001459 | NRP:Glycopeptide | 25.0 | 84.8 | 77.0 | 4.92e-14 |
| AEC14347.1 | nonribosomal\_peptide\_synthetase | BGC0000377 | NRP | 21.0 | 79.4 | 76.0 | 5.66e-14 |
| CAL17540.1 | peptide\_synthetase,\_putative | BGC0002465 | NRP | 21.0 | 91.3 | 76.0 | 5.85e-14 |
| EFL06865.1 | hypothetical\_protein | BGC0000300 | NRP | 23.0 | 91.3 | 76.0 | 6.11e-14 |
| BAB69700.1 | iturin\_A\_synthetase\_C | BGC0001098 | NRP+Polyketide | 23.0 | 79.6 | 76.0 | 6.19e-14 |
| CAJ77695.1 | MPS1\_protein | BGC0000363 | NRP | 26.0 | 84.8 | 76.0 | 6.39e-14 |
| AHF21229.1 | TriE | BGC0000449 | NRP | 22.0 | 85.4 | 76.0 | 6.43e-14 |
| KYC42613.1 | non-ribosomal\_peptide\_synthetase | BGC0002484 | NRP+Polyketide | 22.0 | 96.9 | 76.0 | 6.58e-14 |
| CAG23960.2 | hybrid\_NRPS/PKS\_protein | BGC0001089 | Polyketide+NRP | 22.0 | 85.5 | 76.0 | 6.6e-14 |
| AEG64697.1 | LpmC | BGC0000379 | NRP | 26.0 | 80.1 | 76.0 | 6.68e-14 |
| ATY37609.1 | BreD | BGC0001536 | NRP | 21.0 | 75.8 | 76.0 | 8.34e-14 |
| AFJ23826.1 | WLIP\_synthetase\_C | BGC0001838 | NRP | 27.0 | 57.8 | 76.0 | 8.48e-14 |
| AIG26883.1 | NRPS\_domain-containing\_protein | BGC0002432 | NRP | 21.0 | 94.1 | 76.0 | 8.55e-14 |
| QTT72100.1 | non-ribosomal\_peptide\_synthetase | BGC0002350 | NRP+Polyketide+Saccharide | 25.0 | 84.3 | 76.0 | 8.76e-14 |
| QBA57740.1 | NRPS | BGC0002377 | NRP | 24.0 | 84.1 | 76.0 | 8.81e-14 |
| CBL93716.1 | NRPS\_A-domain | BGC0000360 | NRP | 21.0 | 96.7 | 75.0 | 9.13e-14 |
| TXD00259.1 | amino\_acid\_adenylation\_domain-containing\_protein | BGC0001877 | Polyketide | 22.0 | 80.5 | 76.0 | 9.3e-14 |
| BAP16697.1 | nonribosomal\_peptide\_synthetase | BGC0000376 | NRP | 25.0 | 69.0 | 76.0 | 9.66e-14 |
| ADN26248.1 | peptide\_synthetase | BGC0000951 | NRP | 26.0 | 65.7 | 75.0 | 1.04e-13 |
| ABD65960.1 | nonribosomal\_peptide\_synthetase | BGC0000341 | NRP | 24.0 | 74.4 | 75.0 | 1.05e-13 |
| AAG06715.1 | probable\_non-ribosomal\_peptide\_synthetase | BGC0002037 | NRP | 27.0 | 69.9 | 76.0 | 1.06e-13 |
| ALV86868.1 | Tlo22 | BGC0001406 | NRP | 27.0 | 56.1 | 76.0 | 1.06e-13 |
| EDY47118.1 | N-(5-amino-5-carboxypentanoyl)-L-cysteinyl-D-\_valine\_synthase | BGC0000319 | NRP:Beta-lactam | 23.0 | 92.5 | 76.0 | 1.12e-13 |
| ATP76246.1 | SpuB | BGC0001748 | NRP+Polyketide | 24.0 | 81.7 | 76.0 | 1.12e-13 |
| QBQ12465.1 | amino\_acid\_adenylation\_domain-containing\_protein | BGC0002693 | NRP | 22.0 | 83.8 | 76.0 | 1.12e-13 |
| AKA59447.1 | non-ribosomal\_peptide\_synthetase | BGC0001203 | NRP+Polyketide | 26.0 | 53.1 | 76.0 | 1.13e-13 |
| ANH11404.1 | SceI | BGC0001770 | Polyketide | 25.0 | 59.4 | 74.0 | 1.18e-13 |
| QGA70084.1 | ATP-dependent\_ligase | BGC0002517 | Polyketide | 25.0 | 68.8 | 74.0 | 1.22e-13 |
| BGRAMDRAFT\_RS22640 | amino\_acid\_adenylation\_domain-containing\_protein | BGC0001999 | NRP | 26.0 | 61.5 | 75.0 | 1.22e-13 |
| AAZ23076.1 | peptide\_synthetase | BGC0000291 | NRP | 27.0 | 66.6 | 75.0 | 1.37e-13 |
| NAO96318.1 | amino\_acid\_adenylation\_domain-containing\_protein | BGC0002117 | NRP | 23.0 | 90.8 | 75.0 | 1.38e-13 |
| BAE98155.1 | putative\_non-ribosomal\_peptide\_synthetase | BGC0000339 | NRP | 24.0 | 75.3 | 75.0 | 1.41e-13 |
| CAG29031.1 | nonribosomal\_peptide\_synthetase\_(modules\_1\_and\_2) | BGC0001023 | NRP+Polyketide:Modular type I polyketide | 23.0 | 83.4 | 75.0 | 1.44e-13 |
| APZ78821.1 | nonribosomal\_peptide\_synthetase | BGC0001429 | NRP:Cyclic depsipeptide+Polyketide:Iterative type I polyketide | 23.0 | 83.4 | 75.0 | 1.44e-13 |
| BBC43184.1 | PKS-NRPS\_hybrid | BGC0001738 | NRP+Polyketide | 28.0 | 53.7 | 75.0 | 1.47e-13 |
| ctg1\_orf1264 |  | BGC0001752 | NRP | 24.0 | 67.6 | 75.0 | 1.48e-13 |
| RAT94090.1 | NRPS | BGC0001469 | NRP | 24.0 | 79.1 | 75.0 | 1.51e-13 |
| ADJ63842.1 | Serobactin\_synthetase | BGC0000424 | NRP:NRP siderophore | 24.0 | 78.0 | 75.0 | 1.54e-13 |
| TRX17523.1 | non-ribosomal\_peptide\_synthetase | BGC0002329 | NRP | 27.0 | 58.9 | 75.0 | 1.77e-13 |
| AYJ71721.1 | non-ribosomal\_peptide\_synthetase | BGC0001942 | NRP+Polyketide | 26.0 | 53.0 | 75.0 | 1.84e-13 |
| CAJ77716.1 | Mps2\_protein | BGC0000364 | NRP | 25.0 | 66.2 | 75.0 | 1.86e-13 |
| BAH04162.1 | trsJ | BGC0000450 | NRP | 24.0 | 74.0 | 75.0 | 1.87e-13 |
| AUD08663.1 | iPKS-NRPS | BGC0001553 | NRP+Polyketide | 25.0 | 88.9 | 75.0 | 1.91e-13 |
| AXG49819.1 | hybrid\_non-ribosomal\_peptide\_synthetase/type\_I\_polyketide\_synthase | BGC0000383 | NRP+Polyketide:Modular type I polyketide | 23.0 | 66.7 | 75.0 | 1.95e-13 |
| AAL33756.1 | putative\_non-ribosomal\_peptide\_synthetase | BGC0000421 | NRP | 23.0 | 94.3 | 74.0 | 1.96e-13 |
| ABA73956.1 | putative\_non-ribosomal\_peptide\_synthetase | BGC0001842 | NRP:Lipopeptide | 23.0 | 69.2 | 75.0 | 1.97e-13 |
| TRX17524.1 | amino\_acid\_adenylation\_domain-containing\_protein | BGC0002329 | NRP | 27.0 | 58.9 | 75.0 | 1.98e-13 |
| AFH75320.1 | nonribosomal\_peptide\_synthetase | BGC0000425 | NRP:Cyclic depsipeptide | 25.0 | 73.9 | 75.0 | 2e-13 |
| AAG02356.1 | peptide\_synthetase\_NRPS7 | BGC0000963 | NRP:Glycopeptide+Polyketide:Modular type I polyketide+Saccharide:Hybrid/tailoring saccharide | 25.0 | 84.1 | 74.0 | 2.01e-13 |
| MBD2892727.1 | Phenyloxazoline\_synthase\_MbtB | BGC0002718 | NRP | 27.0 | 67.1 | 74.0 | 2.05e-13 |
| ABX71111.1 | Lct28 | BGC0000238 | Polyketide | 22.0 | 88.0 | 74.0 | 2.09e-13 |
| ABL74936.1 | NRPS | BGC0001048 | NRP:Glycopeptide+Polyketide:Modular type I polyketide+Saccharide:Hybrid/tailoring saccharide | 25.0 | 68.5 | 74.0 | 2.38e-13 |
| WP\_020993844.1 | non-ribosomal\_peptide\_synthetase | BGC0001575 | NRP | 23.0 | 82.9 | 74.0 | 2.49e-13 |
| AEW31015.1 | plipastatin\_synthetase | BGC0000407 | NRP | 24.0 | 51.9 | 74.0 | 2.81e-13 |
| AAO62588.1 | peptide\_sythetase | BGC0001016 | NRP+Polyketide | 21.0 | 94.4 | 74.0 | 2.81e-13 |
| ACO94489.1 | putative\_AMP-dependent\_acyl-CoA\_synthetase/ligase | BGC0000097 | Polyketide:Modular type I polyketide | 24.0 | 96.0 | 73.0 | 2.91e-13 |
| AAF17281.1 | nosD | BGC0001028 | Polyketide+NRP:Cyclic depsipeptide | 21.0 | 89.4 | 74.0 | 3.2e-13 |
| BAX89998.1 | Non-ribosomal\_peptide\_synthetase | BGC0001628 | NRP | 24.0 | 92.3 | 74.0 | 3.22e-13 |
| QXJ21809.1 | amino\_acid\_adenylation\_domain-containing\_protein | BGC0002370 | NRP | 27.0 | 49.3 | 74.0 | 3.24e-13 |
| WP\_013310342.1 | non-ribosomal\_peptide\_synthetase | BGC0001728 | NRP+Polyketide | 21.0 | 90.6 | 74.0 | 3.27e-13 |
| CAJ77715.1 | Mps1\_protein | BGC0000364 | NRP | 25.0 | 71.8 | 74.0 | 3.32e-13 |
| AJK49766.1 | non-ribosomal\_peptide\_synthase | BGC0002565 | NRP | 27.0 | 53.1 | 74.0 | 3.35e-13 |
| ABV56588.1 | KtzH | BGC0000378 | NRP | 27.0 | 65.3 | 74.0 | 3.43e-13 |
| ALK27914.1 | non-ribosomal\_peptide\_synthase | BGC0001233 | NRP | 24.0 | 89.0 | 74.0 | 3.44e-13 |
| AHA12086.1 | amino\_acid\_adenyltransferase | BGC0001172 | NRP+Polyketide:Modular type I polyketide | 25.0 | 65.3 | 73.0 | 3.44e-13 |
| ABK39646.1 | nonribosomal\_peptide\_synthetase | BGC0001502 | NRP | 28.0 | 57.7 | 74.0 | 3.45e-13 |
| ESU17760.1 | hypothetical\_protein | BGC0002172 | NRP | 23.0 | 92.5 | 74.0 | 3.53e-13 |
| AXN93616.1 | PuwH | BGC0001953 | NRP | 22.0 | 89.0 | 74.0 | 3.55e-13 |
| QNL14925.1 | AptD | BGC0002512 | NRP | 21.0 | 78.4 | 74.0 | 3.79e-13 |
| AMK48227.1 | nonribosomal\_peptide\_synthetas | BGC0001351 | NRP | 23.0 | 83.6 | 74.0 | 3.86e-13 |
| AMQ36132.1 | PsyA | BGC0002617 | NRP | 21.0 | 88.3 | 74.0 | 4.14e-13 |
| AAK89720.1 | non-ribosomal\_peptide\_synthetase,\_siderophore\_biosynthesis\_protein | BGC0002107 | NRP+Polyketide | 25.0 | 57.7 | 74.0 | 4.2e-13 |
| AAD44234.1 | PstB | BGC0000362 | NRP | 27.0 | 66.0 | 74.0 | 4.24e-13 |
| BAI63288.1 | putative\_non-ribosomal\_peptide\_synthetase | BGC0000434 | NRP | 24.0 | 65.3 | 74.0 | 4.24e-13 |
| AXB34356.1 | non-ribosomal\_peptide\_synthetase | BGC0002415 | NRP | 23.0 | 76.8 | 74.0 | 4.29e-13 |
| WP\_003981346.1 | non-ribosomal\_peptide\_synthetase | BGC0001813 | NRP | 25.0 | 84.5 | 74.0 | 4.39e-13 |
| QXJ26485.1 | amino\_acid\_adenylation\_domain-containing\_protein | BGC0002370 | NRP | 27.0 | 56.8 | 73.0 | 4.41e-13 |
| CAA11795.1 | PCZA363.4 | BGC0000322 | NRP | 24.0 | 84.8 | 74.0 | 4.44e-13 |
| QED55422.1 | nonribosomal\_peptide\_synthetase | BGC0001984 | NRP | 23.0 | 95.6 | 74.0 | 4.45e-13 |
| AGI89791.1 | Nonribosomal\_peptide\_synthetase | BGC0001792 | NRP | 24.0 | 55.7 | 74.0 | 4.53e-13 |
| ARO38317.1 | nonribosomal\_peptide\_synthetase | BGC0001560 | NRP+Polyketide | 24.0 | 70.0 | 74.0 | 4.56e-13 |
| AGI89789.1 | Nonribosomal\_peptide\_synthetase | BGC0001792 | NRP | 24.0 | 68.1 | 74.0 | 4.59e-13 |
| AFJ14795.1 | PlpF | BGC0000403 | NRP | 23.0 | 91.6 | 73.0 | 4.66e-13 |
| ACN69986.1 | proline\_adenyltransferase | BGC0000079 | Polyketide | 23.0 | 70.4 | 72.0 | 4.68e-13 |
| AXN93603.1 | PuwH | BGC0001952 | NRP | 21.0 | 89.0 | 73.0 | 4.68e-13 |
| QBL56187.1 | long-chain-fatty-acid-CoA\_ligase | BGC0002376 | Polyketide | 23.0 | 68.5 | 72.0 | 4.92e-13 |
| ACS20358.1 | amino\_acid\_adenylation\_domain\_protein | BGC0002420 | NRP+Polyketide | 28.0 | 66.4 | 73.0 | 4.96e-13 |
| ABM34276.1 | amino\_acid\_adenylation\_domain\_protein | BGC0002419 | NRP+Polyketide | 26.0 | 76.5 | 73.0 | 4.97e-13 |
| AWR88409.1 | putative\_AMP-dependent\_synthetase\_and\_ligase | BGC0001522 | Polyketide | 25.0 | 68.8 | 72.0 | 5.36e-13 |
| AAF00960.1 | mcyA | BGC0001017 | NRP+Polyketide:Modular type I polyketide | 21.0 | 83.3 | 73.0 | 5.64e-13 |
| AAN32981.1 | BarG | BGC0000962 | NRP+Polyketide:Modular type I polyketide | 21.0 | 75.8 | 73.0 | 5.66e-13 |
| AGC24265.1 | prlK | BGC0001038 | NRP+Polyketide:Modular type I polyketide | 24.0 | 64.5 | 72.0 | 5.79e-13 |
| OLZ52457.1 | non-ribosomal\_peptide\_synthetase | BGC0001462 | NRP:Glycopeptide | 24.0 | 84.0 | 73.0 | 5.84e-13 |
| mycH | polyketide\_synthase | BGC0002055 | NRP+Polyketide:Trans-AT type I polyketide | 23.0 | 80.5 | 73.0 | 6.02e-13 |
| AEP18655.1 | WAPS2 | BGC0000461 | NRP | 23.0 | 91.5 | 73.0 | 6.04e-13 |
| KMO93435.1 | NRPS/PKS | BGC0002095 | NRP | 23.0 | 62.0 | 73.0 | 6.06e-13 |
| ACZ55945.1 | non-ribosomal\_peptide\_synthetase | BGC0000302 | NRP | 23.0 | 86.6 | 73.0 | 7.19e-13 |
| MBE8994632.1 | amino\_acid\_adenylation\_domain-containing\_protein | BGC0002623 | NRP+Polyketide | 21.0 | 99.3 | 73.0 | 7.32e-13 |
| ABB69752.1 | PlaP4 | BGC0000654 | Terpene+Saccharide:Hybrid/tailoring saccharide | 22.0 | 76.5 | 72.0 | 7.53e-13 |
| QMN69933.1 | PsoB | BGC0002521 | NRP | 26.0 | 71.4 | 73.0 | 7.94e-13 |
| AFJ14794.1 | PlpE | BGC0000403 | NRP | 22.0 | 85.0 | 73.0 | 7.98e-13 |
| ABF85931.1 | non-ribosomal\_peptide\_synthase/polyketide\_synthase\_Ta1 | BGC0001025 | NRP+Polyketide:Trans-AT type I polyketide | 23.0 | 65.7 | 73.0 | 8e-13 |
| QKW60393.1 | amino\_acid\_adenylation\_domain-containing\_protein | BGC0002288 | NRP | 26.0 | 77.0 | 72.0 | 8.56e-13 |
| ABC34137.1 | peptide\_synthetase,\_putative | BGC0000961 | NRP+Polyketide | 24.0 | 64.8 | 72.0 | 9.22e-13 |
| CAM56771.1 |  | BGC0000354 | NRP | 25.0 | 52.6 | 72.0 | 9.57e-13 |
| CZT62794.1 | Non-ribosomal\_peptide\_synthase\_involved\_in\_Hassallidin\_biosynthesis | BGC0001614 | NRP | 22.0 | 65.2 | 72.0 | 9.78e-13 |
| QOJ72663.1 | XenE | BGC0002505 | Polyketide+NRP | 21.0 | 80.0 | 72.0 | 1.01e-12 |
| CAC48361.1 | peptide\_synthetase | BGC0000311 | NRP | 26.0 | 85.0 | 72.0 | 1.01e-12 |
| QXF14600.1 | PydA | BGC0002239 | Polyketide+NRP | 23.0 | 68.6 | 72.0 | 1.01e-12 |
| RSO11554.1 | non-ribosomal\_peptide\_synthetase | BGC0002637 | NRP | 23.0 | 77.0 | 72.0 | 1.02e-12 |
| BCD33690.1 | non-ribosomal\_peptide\_synthetase | BGC0002448 | NRP | 27.0 | 61.3 | 72.0 | 1.04e-12 |
| CAM56770.1 |  | BGC0000354 | NRP | 24.0 | 90.4 | 72.0 | 1.04e-12 |
| AIG79242.1 | Non-ribosomal\_peptide\_synthetase | BGC0000419 | Saccharide+NRP:Glycopeptide | 24.0 | 83.4 | 72.0 | 1.05e-12 |
| ABW70809.1 | PchE | BGC0002475 | NRP | 26.0 | 66.6 | 72.0 | 1.08e-12 |
| AGS77307.1 | NRPS\_modules\_1-2 | BGC0001178 | NRP:Glycopeptide | 28.0 | 54.4 | 72.0 | 1.24e-12 |
| AFL68053.1 | amino\_acid\_adenylation\_enzyme/thioester\_reductase\_family\_protein | BGC0001524 | NRP+Polyketide | 24.0 | 55.6 | 72.0 | 1.25e-12 |
| CAJ34374.1 | NRPS\_protein | BGC0000445 | NRP:Cyclic depsipeptide | 28.0 | 66.2 | 72.0 | 1.27e-12 |
| ABS74179.1 | bacillomycin\_D\_synthetase\_C | BGC0001090 | Polyketide+NRP:Lipopeptide | 23.0 | 92.7 | 72.0 | 1.28e-12 |
| AAO23333.1 | NcpA | BGC0000397 | NRP | 22.0 | 88.3 | 72.0 | 1.32e-12 |
| EME52989.1 | amino\_acid\_adenylation\_protein | BGC0001460 | NRP:Glycopeptide | 25.0 | 84.8 | 72.0 | 1.33e-12 |
| AYA22334.1 | KerC | BGC0001955 | NRP | 25.0 | 85.2 | 72.0 | 1.33e-12 |
| OLZ50885.1 | non-ribosomal\_peptide\_synthetase | BGC0001461 | NRP:Glycopeptide | 25.0 | 85.2 | 72.0 | 1.33e-12 |
| AGS77309.1 | NRPS\_modules\_4-6 | BGC0001178 | NRP:Glycopeptide | 24.0 | 89.2 | 72.0 | 1.33e-12 |
| AAZ23077.1 | peptide\_synthetase | BGC0000291 | NRP | 25.0 | 64.8 | 72.0 | 1.35e-12 |
| ABX37382.1 | amino\_acid\_adenylation\_domain\_protein | BGC0000984 | NRP+Polyketide | 25.0 | 86.1 | 72.0 | 1.36e-12 |
| QDA77059.1 | polyketide\_synthase/nonribosomal\_peptide\_synthetase | BGC0002026 | NRP+Polyketide | 25.0 | 63.6 | 72.0 | 1.39e-12 |
| CUX79062.1 | Octapeptin\_synthase\_subunit\_C | BGC0001715 | NRP | 22.0 | 102.8 | 72.0 | 1.39e-12 |
| CCC55922.1 | putative\_non-ribosomal\_peptide\_synthetase | BGC0000973 | NRP+Polyketide:Modular type I polyketide | 25.0 | 68.8 | 72.0 | 1.4e-12 |
| QNL14922.1 | AptA | BGC0002512 | NRP | 24.0 | 68.6 | 72.0 | 1.64e-12 |
| QWM97862.1 | hybrid\_non-ribosomal\_peptide\_synthetase/type\_I\_polyketide\_synthase | BGC0002434 | Polyketide+NRP | 27.0 | 55.7 | 72.0 | 1.71e-12 |
| AKJ29410.1 | peptide\_synthetase | BGC0001608 | NRP | 22.0 | 80.1 | 72.0 | 1.72e-12 |
| CCJ67639.1 | TaaD | BGC0000447 | NRP:Lipopeptide | 23.0 | 99.1 | 72.0 | 1.72e-12 |
| AAZ23075.1 | peptide\_synthetase | BGC0000291 | NRP | 23.0 | 97.4 | 72.0 | 1.8e-12 |
| CAJ34370.1 | NRPS\_protein | BGC0000445 | NRP:Cyclic depsipeptide | 24.0 | 68.3 | 71.0 | 2.08e-12 |
| ACG60776.1 | NRPS(AL/ACP/C/A/PCP/C/A) | BGC0001058 | NRP:Glycopeptide+Polyketide:Modular type I polyketide+Saccharide:Hybrid/tailoring saccharide | 27.0 | 67.1 | 71.0 | 2.22e-12 |
| QJY30853.1 | PKS-NRPS\_hybrid\_protein | BGC0002539 | Alkaloid | 22.0 | 78.7 | 71.0 | 2.29e-12 |
| BBQ09587.1 | PKS-NRPS\_hybrid | BGC0002261 | Polyketide | 26.0 | 56.1 | 71.0 | 2.3e-12 |
| QED55423.1 | nonribosomal\_peptide\_synthetase | BGC0001984 | NRP | 26.0 | 58.9 | 71.0 | 2.37e-12 |
| AVR48535.1 | CusC | BGC0001564 | NRP+Polyketide | 22.0 | 84.7 | 71.0 | 2.4e-12 |
| AAO72425.1 | syringopeptin\_synthetase\_C | BGC0000438 | NRP | 25.0 | 56.6 | 71.0 | 2.42e-12 |
| AAY37655.1 | Amino\_acid\_adenylation | BGC0000437 | NRP | 25.0 | 56.6 | 71.0 | 2.42e-12 |
| KDM89832.1 | peptide\_synthetase | BGC0002412 | NRP | 23.0 | 75.4 | 71.0 | 2.46e-12 |
| BCD58482.1 | gamma-poly-L-2,4-diaminobutyric\_acid\_synthetase | BGC0002535 | NRP | 21.0 | 83.3 | 71.0 | 2.59e-12 |
| NKI69296.1 | amino\_acid\_adenylation\_domain-containing\_protein | BGC0002408 | NRP | 23.0 | 70.2 | 71.0 | 2.72e-12 |
| CAC17500.1 | putative\_non-ribosomal\_peptide\_synthase | BGC0000324 | NRP | 29.0 | 56.3 | 71.0 | 2.76e-12 |
| WP\_012408786.1 | non-ribosomal\_peptide\_synthetase | BGC0002061 | NRP:Cyclic depsipeptide+Polyketide:Modular type I polyketide | 21.0 | 93.4 | 71.0 | 2.9e-12 |
| CAJ45639.1 | vanchrobactin\_non\_ribosomal\_peptide\_synthetase | BGC0000454 | NRP | 23.0 | 71.4 | 71.0 | 2.93e-12 |
| ABW17377.1 | PsoC | BGC0000411 | NRP | 25.0 | 53.5 | 71.0 | 3.01e-12 |
| AAC06346.1 | bacitracin\_synthetase\_1 | BGC0000310 | NRP | 22.0 | 75.1 | 71.0 | 3.08e-12 |
| CAN89638.1 | putative\_non-ribosomal\_peptide\_synthetase | BGC0001070 | NRP+Polyketide:Modular type I polyketide+Polyketide:Trans-AT type I polyketide | 23.0 | 74.4 | 71.0 | 3.14e-12 |
| BAW32332.1 | nonribosomal\_peptide\_synthetase | BGC0001631 | NRP+Polyketide | 22.0 | 91.3 | 71.0 | 3.41e-12 |
| AHD05677.1 | nonribosomal\_peptide\_ligase\_subunit | BGC0000402 | NRP | 23.0 | 88.2 | 71.0 | 3.82e-12 |
| AHZ34242.1 | CipE | BGC0001389 | NRP | 27.0 | 66.7 | 71.0 | 3.89e-12 |
| AEI58866.1 | peptide\_synthetase | BGC0000455 | NRP | 25.0 | 85.4 | 71.0 | 3.99e-12 |
| AXF16146.1 | non-ribosomal\_peptide\_synthetase | BGC0002563 | NRP | 26.0 | 65.9 | 71.0 | 4.07e-12 |
| AXA20091.1 | hybrid\_trans-AT\_PKS/NRPS\_LgaB | BGC0001646 | NRP+Polyketide | 24.0 | 84.8 | 71.0 | 4.09e-12 |
| AHD05618.1 | putative\_non-ribosomal\_peptide\_ligase\_domain\_protein | BGC0001033 | NRP+Polyketide | 22.0 | 93.6 | 70.0 | 4.23e-12 |
| OAL11436.1 | hypothetical\_protein | BGC0001570 | NRP | 25.0 | 67.2 | 70.0 | 4.28e-12 |
| AAN65233.1 | acyl-CoA\_synthetase | BGC0000832 | Saccharide:Hybrid/tailoring saccharide+Other:Aminocoumarin | 22.0 | 88.7 | 69.0 | 4.49e-12 |
| AAG29789.1 | acyl-CoA\_synthetase | BGC0000833 | Saccharide:Hybrid/tailoring saccharide+Other:Aminocoumarin | 22.0 | 84.8 | 69.0 | 4.49e-12 |
| BAD08370.1 | non-ribosomal\_peptide\_synthetase | BGC0000167 | Polyketide | 23.0 | 66.6 | 69.0 | 4.72e-12 |
| BAE06845.2 | peramine\_synthetase | BGC0002164 | NRP | 29.0 | 58.5 | 70.0 | 5.07e-12 |
| CAB53322.1 | putative\_peptide\_synthetase | BGC0000325 | NRP | 24.0 | 69.0 | 70.0 | 5.2e-12 |
| ABC35522.1 | thiotemplate\_mechanism\_natural\_product\_synthetase | BGC0000186 | NRP+Polyketide:Modular type I polyketide | 28.0 | 54.0 | 70.0 | 5.23e-12 |
| CAE53352.1 | non-ribosomal\_peptide\_synthetase | BGC0000440 | NRP:Glycopeptide | 30.0 | 43.2 | 70.0 | 5.25e-12 |
| CAG15011.1 | peptide\_synthetase,\_module\_4-6 | BGC0000441 | NRP | 30.0 | 43.2 | 70.0 | 5.25e-12 |
| AKJ29411.1 | peptide\_synthetase | BGC0001608 | NRP | 23.0 | 88.0 | 70.0 | 5.27e-12 |
| EJP62835.1 | nonribosomal\_peptide\_synthase,\_putative | BGC0002203 | NRP+Polyketide+Other | 23.0 | 70.7 | 70.0 | 5.31e-12 |
| AFH75321.1 | nonribosomal\_peptide\_synthetase | BGC0000425 | NRP:Cyclic depsipeptide | 24.0 | 85.7 | 70.0 | 5.39e-12 |
| AME30287.1 | L-proline\_adenyl\_transferase | BGC0001463 | Other | 24.0 | 51.4 | 69.0 | 5.87e-12 |
| AJY78094.1 | nonribosomal\_peptide\_synthetase | BGC0001902 | NRP+Polyketide | 23.0 | 67.8 | 70.0 | 5.91e-12 |
| QRN75755.1 | Amino\_acid\_adenylation\_domain\_protein | BGC0002114 | NRP+Polyketide | 21.0 | 90.9 | 70.0 | 6.45e-12 |
| QCQ67879.1 | non-ribosomal\_peptide\_synthetase | BGC0002297 | NRP+Polyketide | 24.0 | 54.2 | 70.0 | 6.67e-12 |
| AXM43052.1 | non-ribosomal\_peptide\_synthetase | BGC0001945 | NRP | 23.0 | 76.0 | 70.0 | 6.81e-12 |
| QCE43603.1 | nonribosomal\_peptide\_synthetase\_(NRPS),\_subunit\_2 | BGC0001834 | NRP | 26.0 | 72.8 | 70.0 | 7.06e-12 |
| AJK49765.1 | non-ribosomal\_peptide\_synthase | BGC0002565 | NRP | 27.0 | 51.6 | 69.0 | 8.35e-12 |
| AWR88407.1 | putative\_AMP-dependent\_synthetase\_and\_ligase | BGC0001522 | Polyketide | 25.0 | 53.5 | 69.0 | 8.6e-12 |
| CBG67537.1 | putative\_NRPS-associated\_AMP-binding\_protein | BGC0002367 | NRP | 25.0 | 57.0 | 69.0 | 8.87e-12 |
| AGI89790.1 | ATP-dependent\_valine\_adenylase | BGC0001792 | NRP | 23.0 | 68.1 | 69.0 | 9.11e-12 |
| AGN74892.1 | nonribosomal\_peptide\_synthetase/polyketide\_synthase\_hybrid\_protein | BGC0000459 | NRP:Cyclic depsipeptide+Polyketide:Trans-AT type I polyketide | 27.0 | 68.3 | 69.0 | 9.15e-12 |
| CBZ42140.1 | non-ribosomal\_peptide\_synthetase | BGC0001117 | NRP | 28.0 | 54.5 | 69.0 | 9.34e-12 |
| AIE77058.1 | peptide\_synthetase\_module\_3 | BGC0000418 | NRP | 24.0 | 71.1 | 69.0 | 9.51e-12 |
| APO47825.1 | non-ribosomal\_peptide\_synthetase | BGC0002653 | NRP | 22.0 | 91.1 | 69.0 | 9.64e-12 |
| NSC23530.1 | amino\_acid\_adenylation\_domain-containing\_protein | BGC0002359 | NRP | 29.0 | 52.8 | 69.0 | 1.03e-11 |
| EHK22005.1 | putative\_non-ribosomal\_peptide\_synthetase\_GliP | BGC0001609 | NRP | 23.0 | 76.0 | 69.0 | 1.11e-11 |
| BCJ07529.1 | hypothetical\_protein | BGC0002379 | NRP | 24.0 | 70.7 | 69.0 | 1.18e-11 |
| BAW32334.1 | hybrid\_cis-AT\_polyketide\_synthase\_-\_nonribosomal\_peptide\_synthetase | BGC0001631 | NRP+Polyketide | 22.0 | 66.0 | 69.0 | 1.19e-11 |
| EGX96627.1 | non-ribosomal\_peptide\_synthase,\_putative | BGC0002259 | Polyketide+NRP | 23.0 | 54.4 | 69.0 | 1.21e-11 |
| QGZ36672.1 | amino\_acid\_adenylation\_domain-containing\_protein | BGC0002082 | NRP+Polyketide | 25.0 | 67.1 | 69.0 | 1.23e-11 |
| AAW03326.1 | CtaC | BGC0000982 | NRP+Polyketide | 23.0 | 67.4 | 69.0 | 1.34e-11 |
| AGU50949.1 | putative\_non-ribosomal\_peptide\_synthetase | BGC0002417 | NRP+Polyketide | 27.0 | 67.2 | 69.0 | 1.35e-11 |
| AHH53508.1 | non-ribosomal\_peptide\_synthetase | BGC0000439 | NRP:Lipopeptide:Ca+-dependent lipopeptide | 24.0 | 87.3 | 69.0 | 1.49e-11 |
| KIA75458.1 | hypothetical\_protein | BGC0002208 | NRP | 25.0 | 89.0 | 69.0 | 1.57e-11 |
| AKA59440.1 | non-ribosomal\_peptide\_synthetase | BGC0001202 | NRP+Polyketide | 27.0 | 53.1 | 68.0 | 1.57e-11 |
| AVI26393.1 | nonribosomal\_peptide\_synthase | BGC0001800 | NRP+Polyketide | 23.0 | 98.8 | 69.0 | 1.57e-11 |
| AGI89788.1 | Nonribosomal\_peptide\_synthetase | BGC0001792 | NRP | 25.0 | 56.8 | 69.0 | 1.57e-11 |
| AAO23334.1 | NcpB | BGC0000397 | NRP | 23.0 | 78.2 | 69.0 | 1.59e-11 |
| AAO56329.1 | non-ribosomal\_peptide\_synthetase\_SyfB | BGC0000435 | NRP | 24.0 | 66.4 | 69.0 | 1.61e-11 |
| BAP05590.1 | calB | BGC0000967 | NRP+Polyketide:Trans-AT type I polyketide | 23.0 | 65.5 | 69.0 | 1.61e-11 |
| CAO98843.1 | hypothetical\_protein | BGC0000023 | Polyketide:Modular type I polyketide | 25.0 | 72.6 | 68.0 | 1.68e-11 |
| MBE8994631.1 | amino\_acid\_adenylation\_domain-containing\_protein | BGC0002623 | NRP+Polyketide | 22.0 | 88.5 | 68.0 | 1.7e-11 |
| AGE11891.1 | nonribosomal\_peptide\_synthetase | BGC0000366 | NRP | 27.0 | 66.4 | 68.0 | 1.74e-11 |
| CAC11137.1 | NikP1\_protein | BGC0000876 | Other | 23.0 | 94.6 | 68.0 | 1.77e-11 |
| AQI70\_32580 |  | BGC0001561 | NRP | 27.0 | 56.3 | 68.0 | 1.82e-11 |
| CAJ18237.2 | non-ribosomal\_peptide\_synthetase\_B | BGC0000354 | NRP | 26.0 | 43.7 | 68.0 | 2.02e-11 |
| AGI87382.1 | Peptide\_synthase | BGC0002358 | Polyketide | 23.0 | 69.7 | 68.0 | 2.04e-11 |
| QCE43602.1 | nonribosomal\_peptide\_synthetase\_(NRPS),\_subunit\_1 | BGC0001834 | NRP | 23.0 | 58.4 | 68.0 | 2.04e-11 |
| CZT62784.1 | Non-ribosomal\_peptide\_synthase,\_involved\_in\_Hassallidin\_biosynthesis | BGC0001614 | NRP | 21.0 | 76.0 | 68.0 | 2.05e-11 |
| AJQ95678.1 | polyketide\_synthase\_modules-related\_protein | BGC0002046 | NRP+Polyketide:Trans-AT type I polyketide | 22.0 | 87.1 | 68.0 | 2.09e-11 |
| AEZ51516.1 | pmxA | BGC0001153 | NRP:Lipopeptide | 22.0 | 79.6 | 68.0 | 2.09e-11 |
| AJI44176.1 | nonribosomal\_peptide\_synthetase | BGC0001193 | NRP | 26.0 | 68.3 | 68.0 | 2.17e-11 |
| ATY72525.1 | non-ribosomal\_peptide\_synthetase | BGC0001574 | NRP | 25.0 | 67.1 | 68.0 | 2.37e-11 |
| AAO07755.1 | Non-ribosomal\_peptide\_synthetase\_module,\_siderophore\_biosynthesis\_vulnibactin-specific | BGC0000460 | NRP | 22.0 | 77.5 | 68.0 | 2.4e-11 |
| ARU08073.1 | mlcK | BGC0001448 | NRP:Lipopeptide:Ca+-dependent lipopeptide | 22.0 | 88.7 | 68.0 | 2.66e-11 |
| AAV97877.1 | OnnI | BGC0001105 | NRP+Polyketide:Trans-AT type I polyketide | 22.0 | 88.3 | 68.0 | 2.75e-11 |
| QNH67550.1 | Cip22 | BGC0002108 | NRP | 23.0 | 93.0 | 68.0 | 2.8e-11 |
| AAY37647.1 | Amino\_acid\_adenylation | BGC0000437 | NRP | 24.0 | 50.7 | 68.0 | 2.82e-11 |
| WP\_141576286.1 | non-ribosomal\_peptide\_synthetase | BGC0002686 | NRP | 24.0 | 68.6 | 67.0 | 2.93e-11 |
| ABO15875.1 | amino\_acid\_adenyltransferase | BGC0000131 | Polyketide | 23.0 | 65.7 | 67.0 | 3.11e-11 |
| QDQ83033.1 | amino\_acid\_adenylation\_domain-containing\_protein | BGC0002564 | NRP | 25.0 | 55.1 | 67.0 | 3.28e-11 |
| BBB04327.1 | nonribosomal\_peptide\_synthetase | BGC0001717 | NRP | 26.0 | 55.4 | 67.0 | 3.31e-11 |
| XP\_020057667.1 | uncharacterized\_protein | BGC0001718 | NRP | 25.0 | 67.4 | 67.0 | 3.32e-11 |
| ARR97039.1 | SphF | BGC0001780 | NRP | 23.0 | 73.0 | 67.0 | 3.33e-11 |
| KGA48739.1 | amino\_acid\_adenylation\_domain\_protein | BGC0002413 | NRP | 23.0 | 72.6 | 67.0 | 3.4e-11 |
| AGZ15459.1 | putative\_non-ribosomal\_peptide\_synthetase | BGC0001036 | NRP+Polyketide | 22.0 | 91.8 | 67.0 | 3.46e-11 |
| QDQ83032.1 | amino\_acid\_adenylation\_domain-containing\_protein | BGC0002564 | NRP | 25.0 | 55.1 | 67.0 | 3.63e-11 |
| AHH53507.1 | non-ribosomal\_peptide\_synthetase | BGC0000439 | NRP:Lipopeptide:Ca+-dependent lipopeptide | 26.0 | 66.7 | 67.0 | 3.68e-11 |
| AFY58519.1 | amino\_acid\_adenylation\_enzyme/thioester\_reductase\_family\_protein,thioester\_reductase-like\_protein | BGC0002411 | NRP+Polyketide | 23.0 | 65.9 | 67.0 | 4.34e-11 |
| UHH90022.1 | VicM | BGC0002634 | Polyketide+NRP+Other | 22.0 | 66.9 | 66.0 | 4.38e-11 |
| AWI62627.1 | nonribosomal\_peptide\_synthetase | BGC0001822 | NRP | 24.0 | 66.7 | 67.0 | 4.42e-11 |
| ABS74208.1 | fengycin\_synthetase\_B | BGC0001095 | NRP | 23.0 | 76.8 | 67.0 | 4.51e-11 |
| AJK49757.1 | non-ribosomal\_peptide\_synthase | BGC0002565 | NRP | 22.0 | 92.5 | 67.0 | 4.53e-11 |
| WP\_012408785.1 | non-ribosomal\_peptide\_synthetase | BGC0002061 | NRP:Cyclic depsipeptide+Polyketide:Modular type I polyketide | 22.0 | 95.6 | 67.0 | 4.68e-11 |
| RGP42808.1 | non-ribosomal\_peptide\_synthetase | BGC0002696 | NRP | 23.0 | 93.4 | 67.0 | 4.71e-11 |
| AAC83656.1 | dihydroaeruginoic\_acid\_synthetase | BGC0000412 | NRP | 27.0 | 62.4 | 67.0 | 5.41e-11 |
| CAD91220.1 | putative\_non-ribosomal\_peptide\_synthetase,\_modules\_1-2 | BGC0000289 | NRP:Glycopeptide+Saccharide:Hybrid/tailoring saccharide | 26.0 | 67.6 | 67.0 | 5.79e-11 |
| sipL7 | AMP-dependent\_synthetase\_and\_ligase | BGC0001452 | Polyketide | 25.0 | 53.5 | 66.0 | 6.1e-11 |
| WP\_012015338.1 | AMP-binding\_protein | BGC0001458 | Other:Aminocoumarin | 25.0 | 56.1 | 66.0 | 6.21e-11 |
| APZ78846.1 | nonribosomal\_peptide\_synthetase | BGC0001431 | NRP:Cyclic depsipeptide+Polyketide:Iterative type I polyketide | 23.0 | 86.1 | 67.0 | 6.22e-11 |
| ADD82940.1 | Bat2 | BGC0001099 | NRP+Polyketide:Modular type I polyketide+Polyketide:Trans-AT type I polyketide | 24.0 | 69.9 | 67.0 | 6.39e-11 |
| BAR73011.1 | putative\_ATP-dependent\_aminoacyl-ACP\_synthetase | BGC0001194 | Polyketide | 25.0 | 54.5 | 66.0 | 7.62e-11 |
| ACC81021.1 | non-ribosomal\_peptide\_synthetase | BGC0001479 | NRP | 23.0 | 56.4 | 66.0 | 7.65e-11 |
| AGD80623.1 | non-ribosomal\_peptide\_synthetase | BGC0000394 | NRP | 24.0 | 82.2 | 66.0 | 7.96e-11 |
| CAD70195.1 | non-ribosomal\_peptide\_synthetase | BGC0001047 | NRP+Polyketide | 24.0 | 67.8 | 66.0 | 8.14e-11 |
| AHH53506.1 | non-ribosomal\_peptide\_synthetase | BGC0000439 | NRP:Lipopeptide:Ca+-dependent lipopeptide | 23.0 | 90.1 | 66.0 | 8.29e-11 |
| E0F75\_025360 | amino\_acid\_adenylation\_domain-containing\_protein | BGC0002340 | NRP+Other | 26.0 | 68.8 | 66.0 | 8.74e-11 |
| ARF06222.1 | non-ribosomal\_peptide\_synthetase | BGC0001593 | NRP | 22.0 | 76.7 | 66.0 | 9.3e-11 |
| AJW76711.1 | DsaI | BGC0001196 | NRP | 24.0 | 90.1 | 66.0 | 1.02e-10 |
| RLV71192.1 | non-ribosomal\_peptide\_synthetase | BGC0001846 | NRP+Saccharide:Hybrid/tailoring saccharide | 25.0 | 52.4 | 66.0 | 1.03e-10 |
| ALV82384.1 | CDA\_peptide\_synthetase\_II | BGC0001370 | NRP | 21.0 | 89.2 | 66.0 | 1.06e-10 |
| AAM80537.1 | StaC | BGC0000290 | NRP:Glycopeptide | 23.0 | 86.9 | 66.0 | 1.07e-10 |
| AHZ34241.1 | CipD | BGC0001389 | NRP | 24.0 | 66.4 | 66.0 | 1.07e-10 |
| APZ78729.1 | nonribosomal\_peptide\_synthetase | BGC0001421 | NRP:Cyclic depsipeptide+Polyketide:Iterative type I polyketide | 21.0 | 96.7 | 66.0 | 1.09e-10 |
| WP\_030498980.1 | hypothetical\_protein | BGC0001327 | NRP:Cyclic depsipeptide+Polyketide:Modular type I polyketide | 26.0 | 66.2 | 66.0 | 1.1e-10 |
| CCA53799.1 | iron\_aquisition\_yersiniabactin\_synthesis\_enzyme | BGC0001801 | NRP | 26.0 | 60.8 | 66.0 | 1.25e-10 |
| BAX64244.1 | NRPS | BGC0001623 | NRP+Polyketide | 23.0 | 97.0 | 66.0 | 1.35e-10 |
| AOZ21320.1 | SulM | BGC0001790 | NRP | 23.0 | 67.2 | 66.0 | 1.37e-10 |
| ATY69568.1 | adenylation\_protein | BGC0001611 | NRP+Polyketide | 25.0 | 50.7 | 65.0 | 1.39e-10 |
| AFD30953.1 | CrmB | BGC0000966 | NRP+Polyketide | 24.0 | 72.0 | 65.0 | 1.5e-10 |
| CBK62746.1 |  | BGC0001115 | NRP+Polyketide | 26.0 | 52.8 | 65.0 | 1.53e-10 |
| EEP98516.1 | Peptide\_synthetase | BGC0002091 | NRP | 24.0 | 65.5 | 65.0 | 1.53e-10 |
| ABO15844.1 | amino\_acid\_adenyltransferase | BGC0000130 | Polyketide | 22.0 | 65.2 | 64.0 | 1.6e-10 |
| CAC17499.1 | putative\_non-ribosomal\_peptide\_synthase | BGC0000324 | NRP | 25.0 | 67.2 | 65.0 | 1.75e-10 |
| AJD47485.1 | PpsD | BGC0002418 | NRP+Polyketide | 25.0 | 67.2 | 65.0 | 1.78e-10 |
| BAP34718.1 | AMP-dependent\_synthetase\_and\_ligase | BGC0000078 | Polyketide | 24.0 | 72.6 | 64.0 | 1.78e-10 |
| ctg1\_orf1265 |  | BGC0001752 | NRP | 25.0 | 66.0 | 65.0 | 1.82e-10 |
| AZH29361.1 | amino\_acid\_adenylation\_domain-containing\_protein | BGC0001843 | NRP | 22.0 | 79.8 | 65.0 | 1.84e-10 |
| BAH33409.1 | putative\_non-ribosomal\_peptide\_synthetase | BGC0000371 | NRP | 23.0 | 93.2 | 65.0 | 1.85e-10 |
| WP\_006051170.1 | non-ribosomal\_peptide\_synthetase | BGC0001999 | NRP | 24.0 | 65.9 | 65.0 | 1.88e-10 |
| WP\_084702182.1 | non-ribosomal\_peptide\_synthetase | BGC0001211 | NRP | 23.0 | 78.6 | 65.0 | 1.9e-10 |
| EXU96269.1 | nonribosomal\_peptide\_synthetase,\_serinocyclin\_synthetase\_NPS1 | BGC0001240 | NRP | 22.0 | 78.2 | 65.0 | 1.92e-10 |
| BCK51629.1 | non-ribosomal\_peptide\_synthetase | BGC0002520 | Polyketide | 27.0 | 52.6 | 65.0 | 2.06e-10 |
| DAB41476.1 | nonribosomal\_peptide\_synthetase | BGC0001230 | NRP:Cyclic depsipeptide+Polyketide:Modular type I polyketide | 24.0 | 97.6 | 65.0 | 2.1e-10 |
| AQX14497.1 | monobactam\_NRPS\_scaffold\_4 | BGC0001672 | NRP | 23.0 | 50.9 | 65.0 | 2.32e-10 |
| ALA09365.1 | AMP-dependent\_synthetase\_and\_ligase | BGC0001303 | Polyketide | 23.0 | 102.1 | 64.0 | 2.34e-10 |
| CAE52339.1 | Polyketide\_non-ribosomal\_peptide\_synthase | BGC0001088 | NRP+Polyketide | 23.0 | 57.3 | 65.0 | 2.49e-10 |
| APU91750.1 | Non-Ribosomal\_Peptide\_Synthetase | BGC0001806 | NRP | 25.0 | 73.9 | 65.0 | 2.54e-10 |
| AHA34037.1 | Bmp4,\_prolyl\_adenylation\_domain | BGC0000890 | Other:Aminocoumarin | 21.0 | 64.1 | 64.0 | 3.07e-10 |
| ctg4\_5 |  | BGC0002017 | NRP | 24.0 | 76.1 | 64.0 | 3.1e-10 |
| AAR87759.1 | ZmaJ | BGC0001059 | NRP+Polyketide | 21.0 | 86.8 | 64.0 | 3.13e-10 |
| CBJ89761.1 | Non-ribosomal\_peptide\_synthase\_involved\_in\_xenocoumacin\_synthesis | BGC0001054 | NRP+Polyketide:Modular type I polyketide | 25.0 | 50.0 | 64.0 | 3.18e-10 |
| AXG47411.1 | hybrid\_non-ribosomal\_peptide\_synthetase/type\_I\_polyketide\_synthase | BGC0002715 | NRP+Polyketide | 25.0 | 51.4 | 64.0 | 3.19e-10 |
| CCA29203.1 | non-ribosomal\_peptide\_synthetase/polyketide\_synthase | BGC0000955 | NRP+Polyketide:Modular type I polyketide | 23.0 | 64.6 | 64.0 | 3.25e-10 |
| ABP57745.1 | DepA | BGC0000993 | NRP:Cyclic depsipeptide+Polyketide:Modular type I polyketide | 28.0 | 51.4 | 64.0 | 3.82e-10 |
| MBA0053730.1 | D-alanine--poly(phosphoribitol)\_ligase | BGC0002096 | Polyketide | 22.0 | 64.1 | 63.0 | 3.88e-10 |
| QYC40287.1 | A50926\_NRPS,\_modules\_1-2 | BGC0002344 | NRP | 24.0 | 80.5 | 64.0 | 3.94e-10 |
| QXJ21807.1 | amino\_acid\_adenylation\_domain-containing\_protein | BGC0002370 | NRP | 26.0 | 49.7 | 64.0 | 4e-10 |
| AAT01807.1 | non-ribosomal\_peptide\_synthetase | BGC0000365 | NRP | 23.0 | 91.1 | 64.0 | 4.04e-10 |
| ALA09366.1 | AMP-dependent\_synthetase\_and\_ligase | BGC0001303 | Polyketide | 25.0 | 53.3 | 63.0 | 4.06e-10 |
| ABL86391.1 | hybrid\_polyketide\_synthase\_and\_nonribosomal\_peptide\_synthetase | BGC0000999 | NRP+Polyketide | 22.0 | 88.0 | 64.0 | 4.12e-10 |
| ACU36654.1 | amino\_acid\_adenylation\_domain\_protein | BGC0000392 | NRP | 25.0 | 92.0 | 64.0 | 4.15e-10 |
| AGC83576.1 | NRPS | BGC0000818 | NRP | 24.0 | 69.5 | 64.0 | 5.23e-10 |
| KJY94240.1 | peptide\_synthetase | BGC0002691 | NRP | 22.0 | 87.6 | 64.0 | 5.48e-10 |
| QIE08736.1 | non-ribosomal\_peptide\_synthetase | BGC0002544 | NRP | 24.0 | 74.4 | 64.0 | 5.5e-10 |
| EKJ70673.1 | hypothetical\_protein | BGC0002188 | NRP+Polyketide | 23.0 | 71.3 | 64.0 | 5.71e-10 |
| QHZ99336.1 | proline\_adenyltransferase | BGC0001875 | Polyketide | 23.0 | 66.0 | 62.0 | 6.89e-10 |
| AAT01806.1 | non-ribosomal\_peptide\_synthetase | BGC0000365 | NRP | 27.0 | 50.3 | 63.0 | 7.17e-10 |
| AHZ20774.1 | non-ribosomal\_peptide\_synthase | BGC0000369 | NRP+Saccharide:Hybrid/tailoring saccharide | 25.0 | 50.3 | 63.0 | 7.2e-10 |
| AFK57215.1 | DidD | BGC0000985 | Polyketide+NRP:Cyclic depsipeptide | 26.0 | 58.2 | 63.0 | 7.23e-10 |
| CAK15814.1 | putative\_non-ribosomal\_peptide\_synthetase,\_terminal\_component | BGC0000344 | NRP | 23.0 | 87.5 | 63.0 | 7.33e-10 |
| APZ78795.1 | nonribosomal\_peptide\_synthetase | BGC0001427 | NRP:Cyclic depsipeptide+Polyketide:Iterative type I polyketide | 22.0 | 84.5 | 63.0 | 9.59e-10 |
| CAG29032.1 | nonribosomal\_peptide\_synthetase\_(modules\_3\_to\_6) | BGC0001023 | NRP+Polyketide:Modular type I polyketide | 23.0 | 73.3 | 63.0 | 9.59e-10 |
| APZ78822.1 | nonribosomal\_peptide\_synthetase | BGC0001429 | NRP:Cyclic depsipeptide+Polyketide:Iterative type I polyketide | 23.0 | 73.3 | 63.0 | 9.59e-10 |
| ANS62968.1 | actinomycin\_synthetase\_II | BGC0001567 | NRP | 24.0 | 77.4 | 62.0 | 1.21e-09 |
| QXJ21811.1 | amino\_acid\_adenylation\_domain-containing\_protein | BGC0002370 | NRP | 28.0 | 30.3 | 62.0 | 1.25e-09 |
| AFY58523.1 | amino\_acid\_adenylation\_enzyme/thioester\_reductase\_family\_protein | BGC0002411 | NRP+Polyketide | 22.0 | 85.7 | 62.0 | 1.25e-09 |
| AEH41789.1 | HrmK | BGC0000374 | NRP:Cyclic depsipeptide | 22.0 | 65.5 | 62.0 | 1.26e-09 |
| CAK15815.1 | putative\_non\_ribosomal\_peptide\_synthetase | BGC0000344 | NRP | 26.0 | 69.7 | 62.0 | 1.3e-09 |
| EWM63010.1 | non-ribosomal\_peptide\_synthetase | BGC0001328 | NRP:Cyclic depsipeptide+Polyketide:Modular type I polyketide | 25.0 | 66.2 | 62.0 | 1.31e-09 |
| QNN94286.1 | EmeB | BGC0002555 | NRP+Polyketide | 22.0 | 70.7 | 62.0 | 1.32e-09 |
| AAO39110.1 | AdmP | BGC0000956 | NRP:Beta-lactam+Polyketide:Type II polyketide | 23.0 | 65.5 | 62.0 | 1.35e-09 |
| CAA60461.1 | pipecolate\_incorporating\_enzyme | BGC0001040 | NRP+Polyketide | 23.0 | 68.1 | 62.0 | 1.48e-09 |
| ACO94492.1 | NRPS\_adenylation\_domain\_protein | BGC0000097 | Polyketide:Modular type I polyketide | 22.0 | 72.3 | 61.0 | 1.61e-09 |
| BCJ07532.1 | hypothetical\_protein | BGC0002379 | NRP | 24.0 | 70.0 | 62.0 | 1.68e-09 |
| ABW17376.1 | PsoB | BGC0000411 | NRP | 22.0 | 72.3 | 62.0 | 1.7e-09 |
| BAO84861.1 | putative\_non-ribosomal\_peptide\_synthetase | BGC0000414 | NRP | 21.0 | 73.9 | 62.0 | 1.8e-09 |
| QXJ21808.1 | amino\_acid\_adenylation\_domain-containing\_protein | BGC0002370 | NRP | 25.0 | 60.8 | 62.0 | 2.15e-09 |
| AEP18656.1 | WAPS1 | BGC0000461 | NRP | 25.0 | 78.6 | 62.0 | 2.24e-09 |
| ctg1\_orf13 |  | BGC0001329 | Polyketide+NRP:Cyclic depsipeptide | 25.0 | 55.4 | 61.0 | 2.43e-09 |
| CAD89774.1 | MelC\_protein | BGC0001010 | NRP+Polyketide:Modular type I polyketide | 24.0 | 68.1 | 61.0 | 2.48e-09 |
| AHB82069.1 | non\_ribosomal\_peptide\_synthetase | BGC0001231 | NRP+Polyketide:Modular type I polyketide | 21.0 | 88.3 | 61.0 | 2.7e-09 |
| ABA70582.1 | alpha-aminoadypil-cysteinyl-valine\_synthetase | BGC0000404 | NRP | 23.0 | 79.3 | 61.0 | 2.83e-09 |
| BAP34707.1 | AMP-dependent\_synthetase\_and\_ligase | BGC0000078 | Polyketide | 23.0 | 67.9 | 61.0 | 2.94e-09 |
| QIE07359.1 | dimodular\_nonribosomal\_peptide\_synthase\_NecA | BGC0002050 | NRP+Polyketide:Trans-AT type I polyketide | 22.0 | 85.2 | 61.0 | 3.28e-09 |
| CAP96445.1 | NRPS | BGC0000420 | NRP | 24.0 | 68.3 | 61.0 | 3.58e-09 |
| APZ78756.1 | nonribosomal\_peptide\_synthetase | BGC0001423 | NRP:Cyclic depsipeptide+Polyketide:Iterative type I polyketide | 22.0 | 92.7 | 61.0 | 3.7e-09 |
| AAZ55900.1 | non-ribosomal\_peptide\_synthase:Amino\_acid\_adenylation | BGC0000359 | NRP | 23.0 | 85.4 | 61.0 | 3.72e-09 |
| ATU31794.1 | NRPS | BGC0001814 | NRP | 25.0 | 70.7 | 61.0 | 3.77e-09 |
| AAN85506.1 | nonribosomal\_peptide\_synthetase | BGC0001101 | NRP+Polyketide:Modular type I polyketide+Polyketide:Trans-AT type I polyketide | 24.0 | 65.9 | 61.0 | 3.9e-09 |
| QMW30133.1 | hypothetical\_protein | BGC0002248 | Terpene+NRP | 23.0 | 70.0 | 61.0 | 4.09e-09 |
| WP\_024483797.1 | non-ribosomal\_peptide\_synthetase | BGC0002002 | NRP | 28.0 | 57.3 | 60.0 | 4.17e-09 |
| AAF63833.1 | PstD | BGC0000362 | NRP | 24.0 | 68.3 | 61.0 | 4.35e-09 |
| MCC5026027.1 | amino\_acid\_adenylation\_domain-containing\_protein | BGC0002118 | NRP+Polyketide | 25.0 | 61.5 | 61.0 | 4.55e-09 |
| MCC5025983.1 | amino\_acid\_adenylation\_domain-containing\_protein | BGC0002119 | NRP+Polyketide | 25.0 | 61.5 | 61.0 | 4.73e-09 |
| OTA20325.1 | peptide\_synthase | BGC0001824 | NRP | 25.0 | 53.8 | 61.0 | 4.9e-09 |
| AZZ09613.1 | PvhA | BGC0002304 | Polyketide+NRP | 21.0 | 77.2 | 61.0 | 4.92e-09 |
| EJK79843.1 | amino\_acid\_adenylation\_enzyme/thioester\_reductase\_family\_protein | BGC0000436 | NRP | 26.0 | 60.1 | 61.0 | 4.93e-09 |
| CCA53804.1 | pyochelin\_synthetase\_F | BGC0001801 | NRP | 25.0 | 67.6 | 60.0 | 5.99e-09 |
| AFD30954.1 | CrmA | BGC0000966 | NRP+Polyketide | 25.0 | 77.9 | 60.0 | 6.22e-09 |
| ABR12615.1 | ACV\_synthetase | BGC0000405 | NRP:Beta-lactam | 23.0 | 79.3 | 60.0 | 6.44e-09 |
| XP\_020058100.1 | uncharacterized\_protein | BGC0001220 | NRP | 23.0 | 64.6 | 60.0 | 6.64e-09 |
| DAC76736.1 | non-ribosomal\_peptide\_synthetase | BGC0001885 | NRP+Polyketide | 24.0 | 54.2 | 60.0 | 7.99e-09 |
| APZ78834.1 | nonribosomal\_peptide\_synthetase | BGC0001430 | NRP:Cyclic depsipeptide+Polyketide:Iterative type I polyketide | 23.0 | 81.9 | 60.0 | 8.54e-09 |
| AZC86156.1 | peramine\_synthetase | BGC0002166 | NRP | 27.0 | 58.5 | 59.0 | 1.09e-08 |
| ARB50207.1 | lysergyl\_peptide\_synthetase\_21 | BGC0001573 | Alkaloid | 23.0 | 70.7 | 59.0 | 1.11e-08 |
| EAL85113.2 | hybrid\_PKS-NRPS\_enzyme | BGC0001037 | NRP+Polyketide:Iterative type I polyketide | 22.0 | 88.3 | 59.0 | 1.12e-08 |
| BAO66533.1 | nonribosomal\_peptide\_synthase | BGC0000042 | Polyketide | 26.0 | 72.5 | 59.0 | 1.17e-08 |
| QKW60392.1 | amino\_acid\_adenylation\_domain-containing\_protein | BGC0002288 | NRP | 27.0 | 49.7 | 59.0 | 1.23e-08 |
| CAH55654.1 | putative\_L-prolyl-AMP\_ligase | BGC0000259 | Polyketide | 22.0 | 51.9 | 58.0 | 1.46e-08 |
| WP\_069848004.1 | non-ribosomal\_peptide\_synthetase | BGC0002472 | NRP | 23.0 | 69.5 | 59.0 | 1.76e-08 |
| AHB82056.1 | non\_ribosomal\_peptide\_synthetase | BGC0001019 | NRP+Polyketide:Modular type I polyketide | 22.0 | 90.4 | 59.0 | 1.84e-08 |
| BCJ07533.1 | hypothetical\_protein | BGC0002379 | NRP | 30.0 | 25.8 | 59.0 | 1.86e-08 |
| AET79183.1 | lysergyl\_peptide\_synthetase\_subunit\_1 | BGC0001241 | Terpene | 22.0 | 68.6 | 59.0 | 1.91e-08 |
| CCE30226.1 | non-ribosomal\_peptide\_synthetase | BGC0002232 | Alkaloid | 22.0 | 68.6 | 59.0 | 1.91e-08 |
| AFJ23825.1 | WLIP\_synthetase\_B | BGC0001838 | NRP | 24.0 | 67.1 | 59.0 | 1.93e-08 |
| ABP55216.1 | AMP-dependent\_synthetase\_and\_ligase | BGC0000142 | Polyketide | 23.0 | 66.6 | 58.0 | 2.01e-08 |
| ABC34305.1 | peptide\_synthetase,\_putative | BGC0000961 | NRP+Polyketide | 27.0 | 49.1 | 58.0 | 2.27e-08 |
| AFP73394.1 | FusA | BGC0001268 | NRP+Polyketide | 25.0 | 51.7 | 58.0 | 2.52e-08 |
| QMN69934.1 | PsoC | BGC0002521 | NRP | 25.0 | 54.4 | 58.0 | 2.52e-08 |
| CBF77087.1 | conserved\_hypothetical\_protein | BGC0001679 | NRP | 24.0 | 67.2 | 57.0 | 4.03e-08 |
| APZ78680.1 | nonribosomal\_peptide\_synthetase | BGC0001417 | NRP:Cyclic depsipeptide+Polyketide:Iterative type I polyketide | 22.0 | 82.8 | 57.0 | 4.36e-08 |
| AET13875.1 | epichloenin\_A\_synthetase | BGC0001250 | NRP | 24.0 | 67.1 | 57.0 | 4.41e-08 |
| AET13879.1 | epichloenin\_A\_synthetase | BGC0001251 | NRP | 24.0 | 67.1 | 57.0 | 4.41e-08 |
| AGQ43600.1 | HC-toxin\_synthetase | BGC0001166 | NRP | 22.0 | 82.8 | 57.0 | 4.43e-08 |
| SAI82904.1 | HrnL;\_NRPS\_adenylation\_domain;\_AMP-binding\_enzyme;\_Long-chain\_fatty\_acid\_CoA\_ligase;\_Pfam00501 | BGC0002101 | Polyketide | 23.0 | 70.9 | 57.0 | 4.56e-08 |
| ABY66004.1 | type\_II\_beta-Tyr\_adenylation\_domain\_protein | BGC0001008 | Polyketide:Iterative type I polyketide+Polyketide:Enediyne type I polyketide | 25.0 | 66.6 | 57.0 | 4.89e-08 |
| AFR69331.1 | nonribosomal\_peptide\_synthetase\_SpiA | BGC0001045 | NRP:Cyclic depsipeptide+Polyketide:Modular type I polyketide | 27.0 | 50.9 | 57.0 | 5.28e-08 |
| EHA55860.1 | polyketide\_synthase/peptide\_synthetase | BGC0002235 | Polyketide+NRP | 23.0 | 57.7 | 57.0 | 5.74e-08 |
| AGM16413.1 | paenibacterin\_synthetase\_B | BGC0000400 | NRP | 23.0 | 57.0 | 57.0 | 5.87e-08 |
| CBF87069.1 | nonribosomal\_peptide\_synthase,\_putative\_(Eurofung) | BGC0001290 | NRP | 23.0 | 69.0 | 57.0 | 5.88e-08 |
| AMM63162.1 | AniA | BGC0001371 | NRP | 22.0 | 83.3 | 57.0 | 5.89e-08 |
| AXG22420.1 | proline\_adenyltransferase | BGC0002024 | Polyketide | 24.0 | 67.6 | 56.0 | 6.17e-08 |
| AAS92545.1 | SirP | BGC0001044 | NRP | 25.0 | 65.9 | 57.0 | 7.18e-08 |
| ABP57748.1 | DepD | BGC0000993 | NRP:Cyclic depsipeptide+Polyketide:Modular type I polyketide | 23.0 | 50.3 | 57.0 | 7.41e-08 |
| CAE14766.1 |  | BGC0002020 | Saccharide+NRP | 25.0 | 50.9 | 56.0 | 7.43e-08 |
| ARU08074.1 | mlcL | BGC0001448 | NRP:Lipopeptide:Ca+-dependent lipopeptide | 23.0 | 79.6 | 57.0 | 7.67e-08 |
| QPB84034.1 | AMP-binding\_protein | BGC0002675 | Polyketide | 21.0 | 90.6 | 56.0 | 7.9e-08 |
| BAO66530.1 | nonribosomal\_peptide\_synthase | BGC0000042 | Polyketide | 23.0 | 54.2 | 56.0 | 8.1e-08 |
| ABP55217.1 | AMP-dependent\_synthetase\_and\_ligase | BGC0000142 | Polyketide | 23.0 | 68.1 | 56.0 | 8.4e-08 |
| UOH28374.1 | AceN | BGC0002149 | NRP+Terpene | 25.0 | 55.1 | 56.0 | 8.74e-08 |
| ATY69551.1 | hybrid\_nonribosomal\_peptide\_synthetase/type\_I\_polyketide\_synthase | BGC0001611 | NRP+Polyketide | 22.0 | 68.8 | 56.0 | 9.51e-08 |
| AAT28740.1 | FUSS | BGC0000064 | NRP+Polyketide | 23.0 | 50.9 | 56.0 | 1.3e-07 |
| AAC83657.1 | pyochelin\_synthetase | BGC0000412 | NRP | 26.0 | 54.7 | 56.0 | 1.6e-07 |
| CCA29202.1 | non-ribosomal\_peptide\_synthetase | BGC0000955 | NRP+Polyketide:Modular type I polyketide | 25.0 | 55.7 | 56.0 | 1.65e-07 |
| QIW91877.1 | NRPS | BGC0002543 | NRP | 23.0 | 78.7 | 56.0 | 1.75e-07 |
| CAA16182.1 | putative\_peptide\_synthase | BGC0001063 | NRP+Polyketide | 25.0 | 52.4 | 55.0 | 1.9e-07 |
| OJJ98497.1 | hypothetical\_protein | BGC0002169 | Polyketide+NRP | 22.0 | 81.7 | 55.0 | 2.05e-07 |
| ABC39418.1 | dihydroaeruginoic\_acid\_synthetase | BGC0000964 | NRP:Cyclic depsipeptide+Polyketide:Trans-AT type I polyketide | 28.0 | 49.1 | 55.0 | 2.08e-07 |
| ALK21570.1 | non-ribosomal\_peptide\_synthetase | BGC0002678 | NRP | 23.0 | 53.8 | 55.0 | 2.1e-07 |
| ADM34138.1 | non-ribosomal\_peptide\_synthetase | BGC0001084 | NRP+Terpene+Alkaloid | 22.0 | 72.5 | 55.0 | 2.15e-07 |
| ABA59548.1 | NRPS | BGC0000453 | NRP:Cyclic depsipeptide | 24.0 | 45.8 | 55.0 | 2.18e-07 |
| QYI86762.1 | non-ribosomal\_peptide\_synthetase | BGC0002424 | NRP | 24.0 | 55.1 | 55.0 | 2.26e-07 |
| APZ78782.1 | nonribosomal\_peptide\_synthetase | BGC0001426 | NRP:Cyclic depsipeptide+Polyketide:Iterative type I polyketide | 22.0 | 80.5 | 55.0 | 2.97e-07 |
| CAH55637.1 | putative\_L-prolyl-AMP\_ligase | BGC0000258 | Polyketide | 23.0 | 53.0 | 54.0 | 3.14e-07 |
| APZ78809.1 | nonribosomal\_peptide\_synthetase | BGC0001428 | NRP:Cyclic depsipeptide+Polyketide:Iterative type I polyketide | 22.0 | 80.8 | 54.0 | 3.9e-07 |
| ESU05145.1 | hypothetical\_protein | BGC0002178 | NRP | 21.0 | 70.2 | 54.0 | 3.9e-07 |
| APZ78744.1 | nonribosomal\_peptide\_synthetase | BGC0001422 | NRP:Cyclic depsipeptide+Polyketide:Iterative type I polyketide | 22.0 | 80.3 | 54.0 | 5.12e-07 |
| CBF76038.1 | nonribosomal\_peptide\_synthase,\_putative\_(Eurofung) | BGC0001399 | NRP | 22.0 | 98.8 | 54.0 | 6.09e-07 |
| ACJ04424.1 | aureobasidin\_A1\_biosynthesis\_complex | BGC0000307 | NRP | 26.0 | 57.5 | 54.0 | 6.93e-07 |
| ADN26257.1 | ATP-dependent\_adenylase | BGC0000951 | NRP | 24.0 | 49.8 | 53.0 | 7.39e-07 |
| EFG10344.1 | Non-ribosomal\_peptide\_synthetase | BGC0000373 | NRP | 27.0 | 54.5 | 53.0 | 7.56e-07 |
| ABL74940.1 | NRPS | BGC0001048 | NRP:Glycopeptide+Polyketide:Modular type I polyketide+Saccharide:Hybrid/tailoring saccharide | 24.0 | 67.1 | 53.0 | 8.58e-07 |
| ctg1\_orf00001 |  | BGC0000901 | Other | 23.0 | 84.0 | 53.0 | 8.67e-07 |
| QBK15049.1 | PKS-NRPS\_hybrid\_TraA | BGC0002197 | Polyketide+NRP | 22.0 | 51.7 | 53.0 | 8.77e-07 |
| AHF22853.1 | MarM | BGC0000091 | Polyketide | 22.0 | 52.3 | 52.0 | 1e-06 |
| ctg1\_orf000000 |  | BGC0000900 | Other | 24.0 | 69.0 | 53.0 | 1.08e-06 |
| BAG84247.1 | putative\_L-prolyl-AMP\_ligase | BGC0000257 | Polyketide | 26.0 | 52.4 | 52.0 | 1.09e-06 |
| ACZ66258.1 | APS1 | BGC0000304 | NRP | 23.0 | 73.5 | 53.0 | 1.17e-06 |
| AAO39104.1 | AdmJ | BGC0000956 | NRP:Beta-lactam+Polyketide:Type II polyketide | 23.0 | 51.9 | 52.0 | 1.34e-06 |
| WP\_051803379.1 | D-alanine--poly(phosphoribitol)\_ligase | BGC0002381 | Alkaloid+Polyketide | 23.0 | 53.5 | 52.0 | 1.34e-06 |
| WP\_043882190.1 | non-ribosomal\_peptide\_synthetase | BGC0001728 | NRP+Polyketide | 21.0 | 79.1 | 52.0 | 1.55e-06 |
| ALK27915.1 | non-ribosomal\_peptide\_synthase | BGC0001233 | NRP | 26.0 | 67.6 | 52.0 | 1.56e-06 |
| CAJ87590.1 | putative\_peptide\_synthase | BGC0001055 | NRP+Polyketide | 25.0 | 56.8 | 52.0 | 1.89e-06 |
| AXM42949.1 | hybrid\_type\_1\_PKS/NRPS | BGC0001941 | NRP+Polyketide | 23.0 | 64.8 | 52.0 | 1.97e-06 |
| ANZ15840.1 | non-ribosomal\_peptide\_synthase/amino\_acid\_adenylation\_enzyme | BGC0001569 | NRP | 25.0 | 66.9 | 52.0 | 2.01e-06 |
| MAA\_10043 | non-ribosomal\_peptide\_synthetase | BGC0000337 | NRP | 25.0 | 63.2 | 52.0 | 2.04e-06 |
| QCC62999.1 | BII-rafflesfungin\_nonribosomal\_protein\_synthetase | BGC0001966 | NRP+Polyketide | 24.0 | 51.2 | 52.0 | 2.06e-06 |
| AAF19812.1 | MtaD | BGC0001024 | NRP+Polyketide:Modular type I polyketide | 22.0 | 82.8 | 51.0 | 3.4e-06 |
| CCE28989.1 | non-ribosomal\_peptide\_synthetase | BGC0001365 | NRP | 28.0 | 49.1 | 51.0 | 4.3e-06 |
| AFK57221.1 | DidJ | BGC0000985 | Polyketide+NRP:Cyclic depsipeptide | 25.0 | 52.3 | 51.0 | 4.32e-06 |
| AGA37269.1 | NRPS | BGC0000819 | NRP+Alkaloid | 25.0 | 52.6 | 51.0 | 4.37e-06 |
| CCC55921.1 | non-ribosomal\_peptide\_synthetase/polyketide\_synthase\_hybrid\_protein | BGC0000973 | NRP+Polyketide:Modular type I polyketide | 26.0 | 58.7 | 51.0 | 4.39e-06 |
| QRD90553.1 | non-ribosomal\_peptide\_synthetase\_module | BGC0002157 | NRP+Alkaloid | 23.0 | 72.1 | 51.0 | 4.39e-06 |
| APZ78769.1 | nonribosomal\_peptide\_synthetase | BGC0001425 | NRP:Cyclic depsipeptide+Polyketide:Iterative type I polyketide | 23.0 | 80.3 | 51.0 | 4.54e-06 |
| AXG47410.1 | non-ribosomal\_peptide\_synthetase | BGC0002715 | NRP+Polyketide | 24.0 | 51.2 | 50.0 | 5.65e-06 |
| AAO56106.1 | yersiniabactin\_non-ribosomal\_peptide\_synthetase | BGC0002570 | NRP+Polyketide | 25.0 | 55.9 | 50.0 | 5.65e-06 |
| AAF00961.1 | mcyB | BGC0001017 | NRP+Polyketide:Modular type I polyketide | 23.0 | 39.2 | 50.0 | 5.67e-06 |
| CAD89775.1 | MelD\_protein | BGC0001010 | NRP+Polyketide:Modular type I polyketide | 23.0 | 55.9 | 50.0 | 5.86e-06 |
