# Supplementary material for: Uncovering the transcriptional landscape of Fomes fomentarius during fungal-based material production through gene co-expression network analysis
Source: Fungal Biol Biotechnol. 2025 Feb 13;12:1. doi: 10.1186/s40694-024-00192-3 (PMC11827164; doi:10.1186/s40694-024-00192-3)
Supplement: Supplementary file 1 — Supplementary Material 1 [file 40694_2024_192_MOESM1_ESM.zip › knownclusterblast/region4/jgi.p_Fomfom1_1202404_mibig_hits.html]

| MIBiG Protein | Description | MIBiG Cluster | MiBiG Product | % ID | % Coverage | BLAST Score | E-value |
| --- | --- | --- | --- | --- | --- | --- | --- |
| ESU17105.1 | hypothetical\_protein | BGC0002176 | Terpene | 23.0 | 72.8 | 62.0 | 2.5e-10 |
