# Supplementary material for: Uncovering the transcriptional landscape of Fomes fomentarius during fungal-based material production through gene co-expression network analysis
Source: Fungal Biol Biotechnol. 2025 Feb 13;12:1. doi: 10.1186/s40694-024-00192-3 (PMC11827164; doi:10.1186/s40694-024-00192-3)
Supplement: Supplementary file 1 — Supplementary Material 1 [file 40694_2024_192_MOESM1_ESM.zip › knownclusterblast/region4/jgi.p_Fomfom1_906865_mibig_hits.html]

| MIBiG Protein | Description | MIBiG Cluster | MiBiG Product | % ID | % Coverage | BLAST Score | E-value |
| --- | --- | --- | --- | --- | --- | --- | --- |
| BCI98769.1 | putative\_cytochrome\_P450 | BGC0002181 | Terpene | 39.0 | 97.6 | 322.0 | 1.94e-104 |
| KIJ60843.1 | hypothetical\_protein | BGC0002214 | Polyketide | 39.0 | 91.8 | 317.0 | 2.67e-103 |
| KIJ60837.1 | hypothetical\_protein | BGC0002214 | Polyketide | 39.0 | 91.6 | 306.0 | 2.55e-98 |
| QJQ03974.1 | CYP-Arm4 | BGC0002445 | Terpene | 36.0 | 96.4 | 301.0 | 1.48e-96 |
| KIJ60846.1 | hypothetical\_protein | BGC0002214 | Polyketide | 38.0 | 96.8 | 299.0 | 1.37e-95 |
| PPQ83215.1 | Dimethyltryptamine\_4-hydroxylase\_(PsiH) | BGC0002207 | Other | 35.0 | 93.8 | 293.0 | 2.37e-93 |
| AAS90061.1 | OrdA | BGC0000009 | Polyketide | 34.0 | 98.4 | 288.0 | 5.63e-91 |
| XP\_007301850.1 | cytochrome\_P450 | BGC0001617 | Terpene | 36.0 | 100.2 | 284.0 | 1.29e-89 |
| AAS90105.1 | OrdA | BGC0000006 | Polyketide | 34.0 | 97.8 | 283.0 | 3.39e-89 |
| AAS90081.1 | OrdA | BGC0000010 | Polyketide | 34.0 | 98.4 | 282.0 | 9.45e-89 |
| BAE71330.1 | oxidoreductase\_A;oxidoreductase/cytochrome\_P450\_monooxygenase | BGC0000004 | Polyketide | 34.0 | 98.4 | 280.0 | 5.21e-88 |
| AAS90035.1 | OrdA | BGC0000008 | Polyketide | 34.0 | 98.4 | 280.0 | 5.21e-88 |
| QJQ03972.1 | CYP-Arm3 | BGC0002445 | Terpene | 35.0 | 99.6 | 275.0 | 3.64e-86 |
| KIJ60841.1 | hypothetical\_protein | BGC0002214 | Polyketide | 36.0 | 93.6 | 274.0 | 6.8e-86 |
| QJQ03971.1 | CYP-Arm2 | BGC0002445 | Terpene | 34.0 | 98.0 | 270.0 | 3.49e-84 |
| EIN09540.1 | cytochrome\_P450 | BGC0002213 | Polyketide | 35.0 | 97.2 | 265.0 | 2.77e-82 |
| EIM84826.1 | cytochrome\_P450 | BGC0002219 | Terpene | 34.0 | 91.8 | 261.0 | 6.55e-81 |
| XP\_007301851.1 | cytochrome\_P450 | BGC0001617 | Terpene | 35.0 | 97.0 | 261.0 | 2.17e-80 |
| KAA1470686.1 | cytochrome\_P450 | BGC0002218 | Terpene | 34.0 | 98.0 | 259.0 | 6.33e-80 |
| CBF82795.1 | cytochrome\_P450,\_putative\_(Eurofung) | BGC0001668 | NRP | 35.0 | 98.6 | 254.0 | 4.23e-78 |
| XP\_007301602.1 | cytochrome\_P450 | BGC0001617 | Terpene | 33.0 | 101.2 | 253.0 | 1.11e-77 |
| QBC75451.1 | MacC | BGC0002615 | Terpene | 31.0 | 103.4 | 232.0 | 1.13e-69 |
| FAC38\_04 |  | BGC0002198 | NRP | 32.0 | 99.8 | 227.0 | 8.24e-68 |
| EYE95339.1 | cytochrome\_P450 | BGC0002234 | Polyketide | 32.0 | 91.4 | 220.0 | 3.85e-65 |
| EHA22193.1 | hypothetical\_protein | BGC0000170 | Polyketide | 30.0 | 100.2 | 219.0 | 1.03e-64 |
| QGW49097.1 | putative\_cytochrome\_P450 | BGC0002731 | Polyketide | 34.0 | 91.4 | 218.0 | 3.07e-64 |
| XP\_020057670.1 | uncharacterized\_protein | BGC0001718 | NRP | 31.0 | 91.2 | 211.0 | 6.7e-62 |
| EHK18434.1 | hypothetical\_protein | BGC0002233 | Polyketide | 31.0 | 100.0 | 208.0 | 2.15e-60 |
| OJJ97580.1 | hypothetical\_protein | BGC0002229 | Polyketide | 30.0 | 98.6 | 204.0 | 3.8e-59 |
| QQO98481.1 | FrzL | BGC0002146 | NRP | 31.0 | 97.0 | 203.0 | 7.22e-59 |
| CEN60544.1 | hypothetical\_protein | BGC0002266 | Terpene+Polyketide | 34.0 | 88.2 | 203.0 | 9.27e-59 |
| BBB04330.1 | cytochrome\_P450 | BGC0001717 | NRP | 28.0 | 98.6 | 196.0 | 2.85e-56 |
| BAH23999.1 | cytochrome\_P450 | BGC0000356 | NRP+Alkaloid | 32.0 | 101.6 | 195.0 | 1.01e-55 |
| AQZ42158.1 | putative\_cytochrome\_P450 | BGC0001820 | NRP | 30.0 | 102.0 | 191.0 | 3.82e-54 |
| BCI98773.1 | putative\_cytochrome\_P450 | BGC0002181 | Terpene | 27.0 | 101.6 | 187.0 | 4.97e-53 |
| CBF82292.1 | cytochrome\_P450,\_putative\_(Eurofung) | BGC0002180 | Polyketide | 29.0 | 101.0 | 188.0 | 5.46e-53 |
| DAB41655.1 | cytochrome\_P450\_monooxygenase | BGC0001585 | Alkaloid | 29.0 | 103.2 | 186.0 | 3.5e-52 |
| AIG62144.1 | M-cresol\_hydroxylase | BGC0000120 | Polyketide:Iterative type I polyketide | 31.0 | 93.2 | 184.0 | 1.35e-51 |
| QJQ03970.1 | CYP-Arm1 | BGC0002445 | Terpene | 29.0 | 104.0 | 182.0 | 8.88e-51 |
| OJJ97582.1 | hypothetical\_protein | BGC0002229 | Polyketide | 29.0 | 95.8 | 174.0 | 3.83e-48 |
| AGK82815.1 | cytochrome\_P450-2 | BGC0001324 | Terpene | 28.0 | 91.8 | 165.0 | 8.3e-45 |
| AGK82817.1 | cytochrome\_P450-1 | BGC0001324 | Terpene | 29.0 | 92.0 | 164.0 | 1.77e-44 |
| AGK82831.1 | cytochrome\_P450-2 | BGC0001321 | Terpene | 29.0 | 92.4 | 163.0 | 4.38e-44 |
| AGK82807.1 | cytochrome\_P450-1 | BGC0001322 | Terpene | 28.0 | 92.0 | 161.0 | 1.51e-43 |
| pseudo106205\_112773 |  | BGC0001322 | Terpene | 28.0 | 92.4 | 159.0 | 1.46e-42 |
| AGK82824.1 | cytochrome\_P450-2 | BGC0001323 | Terpene | 28.0 | 92.6 | 157.0 | 3.76e-42 |
| EER93095.1 | hypothetical\_protein | BGC0000798 | Saccharide | 28.0 | 93.2 | 154.0 | 1.91e-40 |
| chr3.CM0241.850.r2.m |  | BGC0001316 | Other | 27.0 | 91.6 | 142.0 | 9.41e-37 |
| EEF48745.1 | cytochrome\_P450,\_putative | BGC0002393 | Terpene | 27.0 | 89.0 | 138.0 | 3.89e-35 |
| BAF09097.1 |  | BGC0000672 | Terpene | 27.0 | 97.8 | 132.0 | 3.92e-33 |
| KGN46390.1 | hypothetical\_protein | BGC0001315 | Terpene | 26.0 | 90.8 | 132.0 | 4.19e-33 |
| EEF48738.1 | cytochrome\_P450,\_putative | BGC0002393 | Terpene | 26.0 | 88.2 | 130.0 | 1.89e-32 |
| EEF48750.1 | (S)-N-methylcoclaurine\_3'-hydroxylase\_isozyme,\_putative | BGC0002393 | Terpene | 26.0 | 92.6 | 129.0 | 6e-32 |
| EEF48740.1 | cytochrome\_P450,\_putative | BGC0002393 | Terpene | 25.0 | 91.4 | 121.0 | 3.49e-29 |
| AQU14205.1 | cytochrome\_p450 | BGC0001490 | Other:PBDE | 26.0 | 98.4 | 119.0 | 1.63e-28 |
| EEF48742.1 | cytochrome\_P450,\_putative | BGC0002393 | Terpene | 25.0 | 88.4 | 117.0 | 5.77e-28 |
| ALS30800.1 | putative\_cytochrome\_P450\_monooxygenase | BGC0001286 | Other | 24.0 | 98.8 | 116.0 | 1.96e-27 |
| chr3.CM0241.940.r2.a |  | BGC0001316 | Other | 39.0 | 34.6 | 99.0 | 3.37e-22 |
| BAI44339.1 | P450 | BGC0000677 | Terpene | 27.0 | 70.4 | 100.0 | 3.94e-22 |
| AAM54108.1 | cytochrome\_P450 | BGC0000020 | Polyketide | 25.0 | 102.0 | 100.0 | 1.16e-21 |
| KGN46389.1 | hypothetical\_protein | BGC0001315 | Terpene | 24.0 | 89.6 | 97.0 | 5.72e-21 |
| BAD86796.1 | putative\_P450 | BGC0000286 | Polyketide | 31.0 | 36.8 | 87.0 | 8.1e-18 |
| BAR97462.1 | cytochrome\_P450 | BGC0001911 | Terpene | 23.0 | 91.2 | 81.0 | 7.72e-16 |
| Manes.12G132800 |  | BGC0001318 | Other | 29.0 | 45.0 | 79.0 | 2.26e-15 |
| Manes.12G132900 |  | BGC0001318 | Other | 28.0 | 45.0 | 78.0 | 5.23e-15 |
| EAU36102.1 | predicted\_protein | BGC0002273 | NRP | 25.0 | 71.0 | 72.0 | 2.38e-13 |
| OJJ99917.1 | hypothetical\_protein | BGC0002225 | Terpene | 27.0 | 36.0 | 71.0 | 8.22e-13 |
| BAE78976.1 | P450 | BGC0001078 | Terpene+Polyketide | 28.0 | 40.0 | 67.0 | 1.56e-11 |
| APY21860.1 | FmsC | BGC0001659 | Terpene | 29.0 | 34.4 | 64.0 | 1.41e-10 |
| QGW49100.1 | putative\_cytochrome\_P450 | BGC0002731 | Polyketide | 33.0 | 31.4 | 62.0 | 4.99e-10 |
| ATZ56106.1 | Bcbot1 | BGC0000631 | Terpene | 32.0 | 28.4 | 62.0 | 7.28e-10 |
| GAP90685.1 | putative\_benzoate\_4-monooxygenase\_cytochrome | BGC0002651 | Terpene | 25.0 | 40.4 | 62.0 | 9.85e-10 |
| CAQ18837.1 | putative\_cytochrome\_P450 | BGC0000954 | NRP+Polyketide:Modular type I polyketide | 29.0 | 35.2 | 61.0 | 1.23e-09 |
| BAV32152.1 | cytochrome\_P450\_monooxygenase | BGC0001373 | Polyketide | 27.0 | 49.0 | 61.0 | 1.77e-09 |
| NP\_198460.1 | cytochrome\_P450,\_family\_716,\_subfamily\_A,\_polypeptide\_1 | BGC0001314 | Terpene | 21.0 | 95.8 | 59.0 | 6.48e-09 |
| ART41207.1 | AdrA | BGC0001508 | Polyketide | 25.0 | 41.4 | 58.0 | 1.58e-08 |
| QCL09096.1 | dmxR5 | BGC0002063 | Polyketide:Iterative type I polyketide | 31.0 | 31.8 | 58.0 | 1.58e-08 |
| BAT32890.1 | stellatic\_acid\_synthase | BGC0002610 | Terpene | 25.0 | 44.2 | 58.0 | 1.61e-08 |
| EAU29532.1 | conserved\_hypothetical\_protein | BGC0000682 | Terpene | 29.0 | 27.2 | 57.0 | 3.55e-08 |
| QPP46751.1 | cytochrome\_P450 | BGC0002500 | Polyketide | 25.0 | 30.4 | 56.0 | 4.62e-08 |
| EAU36094.1 | predicted\_protein | BGC0002273 | NRP | 24.0 | 56.6 | 53.0 | 5.11e-07 |
| ALJ49930.1 | TtmP | BGC0001236 | Polyketide | 26.0 | 48.0 | 52.0 | 9.29e-07 |
| CAP93752.1 |  | BGC0001882 | Polyketide | 27.0 | 43.6 | 51.0 | 1.82e-06 |
| AAF86397.1 | FkbD | BGC0000994 | NRP+Polyketide | 29.0 | 29.4 | 50.0 | 2.61e-06 |
