# Supplementary material for: Uncovering the transcriptional landscape of Fomes fomentarius during fungal-based material production through gene co-expression network analysis
Source: Fungal Biol Biotechnol. 2025 Feb 13;12:1. doi: 10.1186/s40694-024-00192-3 (PMC11827164; doi:10.1186/s40694-024-00192-3)
Supplement: Supplementary file 1 — Supplementary Material 1 [file 40694_2024_192_MOESM1_ESM.zip › knownclusterblast/region4/jgi.p_Fomfom1_906870_mibig_hits.html]

| MIBiG Protein | Description | MIBiG Cluster | MiBiG Product | % ID | % Coverage | BLAST Score | E-value |
| --- | --- | --- | --- | --- | --- | --- | --- |
| BCI98769.1 | putative\_cytochrome\_P450 | BGC0002181 | Terpene | 38.0 | 95.5 | 295.0 | 1.64e-94 |
| AAS90081.1 | OrdA | BGC0000010 | Polyketide | 36.0 | 96.8 | 267.0 | 1.84e-83 |
| AAS90035.1 | OrdA | BGC0000008 | Polyketide | 36.0 | 96.8 | 266.0 | 3.64e-83 |
| AAS90013.1 | OrdA | BGC0000007 | Polyketide | 35.0 | 96.8 | 265.0 | 1.42e-82 |
| PPQ83215.1 | Dimethyltryptamine\_4-hydroxylase\_(PsiH) | BGC0002207 | Other | 36.0 | 90.9 | 263.0 | 2.12e-82 |
| BAE71330.1 | oxidoreductase\_A;oxidoreductase/cytochrome\_P450\_monooxygenase | BGC0000004 | Polyketide | 35.0 | 96.8 | 264.0 | 2.81e-82 |
| AAS90061.1 | OrdA | BGC0000009 | Polyketide | 35.0 | 96.8 | 264.0 | 2.81e-82 |
| KIJ60843.1 | hypothetical\_protein | BGC0002214 | Polyketide | 36.0 | 98.3 | 261.0 | 3.6e-82 |
| AAS90105.1 | OrdA | BGC0000006 | Polyketide | 35.0 | 96.8 | 263.0 | 5.55e-82 |
| KIJ60837.1 | hypothetical\_protein | BGC0002214 | Polyketide | 36.0 | 90.7 | 254.0 | 6.46e-79 |
| PPQ83216.1 | Dimethyrltryptamine\_4-hydroxylase\_(PsiH) | BGC0002207 | Other | 36.0 | 90.7 | 249.0 | 4.91e-77 |
| XP\_007301850.1 | cytochrome\_P450 | BGC0001617 | Terpene | 36.0 | 96.1 | 245.0 | 5.03e-75 |
| KIJ60846.1 | hypothetical\_protein | BGC0002214 | Polyketide | 34.0 | 96.3 | 243.0 | 2.67e-74 |
| QJQ03972.1 | CYP-Arm3 | BGC0002445 | Terpene | 34.0 | 97.0 | 236.0 | 6.97e-72 |
| EIM84826.1 | cytochrome\_P450 | BGC0002219 | Terpene | 35.0 | 89.4 | 235.0 | 1.67e-71 |
| CBF82795.1 | cytochrome\_P450,\_putative\_(Eurofung) | BGC0001668 | NRP | 34.0 | 95.2 | 229.0 | 7.04e-69 |
| KIJ60841.1 | hypothetical\_protein | BGC0002214 | Polyketide | 34.0 | 91.6 | 221.0 | 5.01e-66 |
| EAL85116.1 | cytochrome\_P450\_oxidoreductase | BGC0001067 | Terpene+Polyketide:Iterative type I polyketide | 30.0 | 95.5 | 218.0 | 1.44e-64 |
| QJQ03971.1 | CYP-Arm2 | BGC0002445 | Terpene | 32.0 | 96.1 | 215.0 | 9.1e-64 |
| XP\_007301851.1 | cytochrome\_P450 | BGC0001617 | Terpene | 33.0 | 88.3 | 199.0 | 1.2e-57 |
| QBC75451.1 | MacC | BGC0002615 | Terpene | 29.0 | 98.3 | 190.0 | 3.68e-54 |
| XP\_007301852.1 | cytochrome\_P450 | BGC0001617 | Terpene | 32.0 | 91.1 | 188.0 | 9.64e-54 |
| EHA22193.1 | hypothetical\_protein | BGC0000170 | Polyketide | 29.0 | 97.4 | 189.0 | 1.01e-53 |
| EHK18434.1 | hypothetical\_protein | BGC0002233 | Polyketide | 32.0 | 99.6 | 184.0 | 4.83e-52 |
| FAC38\_04 |  | BGC0002198 | NRP | 30.0 | 96.1 | 183.0 | 7.18e-52 |
| CBF82292.1 | cytochrome\_P450,\_putative\_(Eurofung) | BGC0002180 | Polyketide | 29.0 | 97.8 | 178.0 | 8.38e-50 |
| QGW49097.1 | putative\_cytochrome\_P450 | BGC0002731 | Polyketide | 31.0 | 86.4 | 177.0 | 2.21e-49 |
| OQD69071.1 | hypothetical\_protein | BGC0002745 | Polyketide | 28.0 | 97.4 | 176.0 | 3.76e-49 |
| QQO98481.1 | FrzL | BGC0002146 | NRP | 28.0 | 98.1 | 174.0 | 1.71e-48 |
| DAB41655.1 | cytochrome\_P450\_monooxygenase | BGC0001585 | Alkaloid | 30.0 | 101.1 | 169.0 | 1.37e-46 |
| QDO73503.1 | PeniB | BGC0002557 | Terpene | 29.0 | 96.1 | 166.0 | 2.2e-45 |
| BAE60012.1 |  | BGC0001518 | Terpene | 29.0 | 89.6 | 147.0 | 8.44e-39 |
| QMS79071.1 | fumitremorgin\_C\_synthase | BGC0002198 | NRP | 26.0 | 100.4 | 140.0 | 4.78e-36 |
| OJJ97582.1 | hypothetical\_protein | BGC0002229 | Polyketide | 26.0 | 90.0 | 132.0 | 4.22e-33 |
| NP\_001130688.1 | uncharacterized\_protein\_LOC100191791 | BGC0002391 | Terpene | 25.0 | 88.7 | 120.0 | 2.8e-29 |
| AGK82831.1 | cytochrome\_P450-2 | BGC0001321 | Terpene | 25.0 | 90.0 | 119.0 | 8.95e-29 |
| AGK82817.1 | cytochrome\_P450-1 | BGC0001324 | Terpene | 25.0 | 89.4 | 115.0 | 1.43e-27 |
| AGK82824.1 | cytochrome\_P450-2 | BGC0001323 | Terpene | 24.0 | 89.6 | 113.0 | 8.32e-27 |
| AGK82815.1 | cytochrome\_P450-2 | BGC0001324 | Terpene | 24.0 | 89.4 | 113.0 | 1.13e-26 |
| pseudo106205\_112773 |  | BGC0001322 | Terpene | 24.0 | 90.0 | 113.0 | 1.13e-26 |
| AGK82807.1 | cytochrome\_P450-1 | BGC0001322 | Terpene | 24.0 | 89.6 | 111.0 | 3.76e-26 |
| KGN46388.1 | hypothetical\_protein | BGC0001315 | Terpene | 22.0 | 96.8 | 109.0 | 1.71e-25 |
| EEF48734.1 | cytochrome\_P450,\_putative | BGC0002393 | Terpene | 25.0 | 85.5 | 109.0 | 2.28e-25 |
| NP\_199610.1 | cytochrome\_P450,\_family\_705,\_subfamily\_A,\_polypeptide\_5 | BGC0000670 | Terpene | 26.0 | 97.2 | 108.0 | 5.77e-25 |
| XP\_044984106.1 | indole-2-monooxygenase-like | BGC0002721 | Saccharide | 24.0 | 90.0 | 107.0 | 7.83e-25 |
| chr3.CM0241.850.r2.m |  | BGC0001316 | Other | 26.0 | 88.3 | 106.0 | 2.54e-24 |
| NP\_001329686.1 | cytochrome\_P450,\_family\_705,\_subfamily\_A,\_polypeptide\_1 | BGC0001313 | Terpene | 30.0 | 47.2 | 104.0 | 7.2e-24 |
| NP\_193268.3 | cytochrome\_P450,\_family\_705,\_subfamily\_A,\_polypeptide\_1 | BGC0001313 | Terpene | 30.0 | 47.2 | 104.0 | 8.51e-24 |
| NP\_193270.1 | cytochrome\_P450,\_family\_705,\_subfamily\_A,\_polypeptide\_2 | BGC0001313 | Terpene | 31.0 | 46.1 | 104.0 | 1.12e-23 |
| Manes.12G132800 |  | BGC0001318 | Other | 33.0 | 37.0 | 103.0 | 2.1e-23 |
| AFW60212.1 | benzoxazinone\_synthesis2 | BGC0000810 | Alkaloid | 26.0 | 90.9 | 103.0 | 2.37e-23 |
| Manes.12G132900 |  | BGC0001318 | Other | 30.0 | 41.1 | 102.0 | 3.7e-23 |
| XP\_037497843.1 | premnaspirodiene\_oxygenase | BGC0002724 | Terpene | 23.0 | 86.8 | 101.0 | 9.51e-23 |
| chr3.CM0241.940.r2.a |  | BGC0001316 | Other | 36.0 | 41.6 | 100.0 | 1.38e-22 |
| BAT00637.1 |  | BGC0002392 | Terpene | 23.0 | 88.1 | 97.0 | 2.36e-21 |
| NP\_193271.5 | cytochrome\_P450,\_family\_705,\_subfamily\_A,\_polypeptide\_3 | BGC0001313 | Terpene | 35.0 | 35.1 | 97.0 | 4.36e-21 |
| EEF48745.1 | cytochrome\_P450,\_putative | BGC0002393 | Terpene | 24.0 | 87.9 | 97.0 | 4.46e-21 |
| XP\_044984175.1 | tyrosine\_N-monooxygenase-like | BGC0002721 | Saccharide | 33.0 | 39.0 | 96.0 | 7.65e-21 |
| ESP90850.1 | cytochrome\_P450 | BGC0000891 | Other:Aminocoumarin | 33.0 | 32.9 | 93.0 | 6.97e-20 |
| CYP82X2 |  | BGC0001325 | Alkaloid | 35.0 | 36.8 | 92.0 | 1.16e-19 |
| AFW60213.1 | benzoxazinone\_synthesis2 | BGC0000810 | Alkaloid | 32.0 | 40.3 | 91.0 | 1.21e-19 |
| AAD34565.1 | cytochrome\_P450\_monooxygenase | BGC0000088 | Polyketide | 26.0 | 55.8 | 90.0 | 5.86e-19 |
| XP\_001213595.1 | hypothetical\_protein | BGC0001475 | Terpene | 25.0 | 90.7 | 89.0 | 1.84e-18 |
| NP\_193273.1 | cytochrome\_P450,\_family\_705,\_subfamily\_A,\_polypeptide\_4 | BGC0001313 | Terpene | 28.0 | 46.5 | 88.0 | 2.49e-18 |
| BAI44339.1 | P450 | BGC0000677 | Terpene | 22.0 | 77.3 | 85.0 | 2.03e-17 |
| EAL89316.1 | cytochrome\_P450\_monooxygenase,\_putative | BGC0000686 | Terpene | 30.0 | 39.2 | 84.0 | 7.66e-17 |
| CYP719A21 |  | BGC0001325 | Alkaloid | 24.0 | 88.3 | 83.0 | 9.55e-17 |
| QMW30128.1 | hypothetical\_protein | BGC0002248 | Terpene+NRP | 25.0 | 76.6 | 84.0 | 9.92e-17 |
| EIN09535.1 | cytochrome\_P450 | BGC0002213 | Polyketide | 26.0 | 81.2 | 82.0 | 2e-16 |
| KAF5858312.1 | Cytochrome\_P450 | BGC0002139 | Polyketide | 25.0 | 55.4 | 80.0 | 1.24e-15 |
| EAU36102.1 | predicted\_protein | BGC0002273 | NRP | 26.0 | 77.1 | 79.0 | 1.95e-15 |
| ABV57820.1 | cytochrome\_P450\_monooxygenase | BGC0001267 | Terpene | 27.0 | 61.0 | 79.0 | 2.41e-15 |
| AHA38204.1 | GphK | BGC0000069 | Polyketide | 24.0 | 82.7 | 77.0 | 8.15e-15 |
| AAK33073.1 | cytochrome\_P450 | BGC0001278 | Terpene | 24.0 | 89.8 | 74.0 | 9.95e-14 |
| AET79191.1 | elymoclavine\_monooxygenase | BGC0001241 | Terpene | 28.0 | 59.7 | 73.0 | 1.71e-13 |
| CCE30234.1 | related\_to\_trichodiene\_oxygenase\_cytochrome\_P450 | BGC0002232 | Alkaloid | 28.0 | 59.7 | 73.0 | 1.71e-13 |
| BAR97452.1 | cytochrome\_P450 | BGC0001910 | Terpene | 23.0 | 87.9 | 72.0 | 2.62e-13 |
| QBK15048.1 | cytochrome\_P450\_ClaJ | BGC0002196 | Polyketide | 24.0 | 39.6 | 71.0 | 5.86e-13 |
| KIA75687.1 | hypothetical\_protein | BGC0002242 | NRP | 29.0 | 36.1 | 71.0 | 9.66e-13 |
| OJJ99917.1 | hypothetical\_protein | BGC0002225 | Terpene | 24.0 | 57.6 | 70.0 | 1.61e-12 |
| GAP90685.1 | putative\_benzoate\_4-monooxygenase\_cytochrome | BGC0002651 | Terpene | 23.0 | 75.8 | 69.0 | 3.99e-12 |
| CAQ18836.1 | putative\_cytochrome\_P450 | BGC0000954 | NRP+Polyketide:Modular type I polyketide | 25.0 | 61.9 | 67.0 | 1.43e-11 |
| AAM97314.1 | cytochrome\_P450\_hydroxylase | BGC0000093 | Polyketide | 26.0 | 43.5 | 67.0 | 1.43e-11 |
| DAC76739.1 | cytochrome\_P450 | BGC0001885 | NRP+Polyketide | 27.0 | 46.1 | 67.0 | 1.43e-11 |
| QPP19376.1 | Pen14 | BGC0002501 | Alkaloid | 25.0 | 52.2 | 66.0 | 4.95e-11 |
| BAJ09784.1 | P450 | BGC0000146 | Polyketide | 26.0 | 51.3 | 65.0 | 5.93e-11 |
| AKG47164.1 | HysX1 | BGC0001223 | Other | 27.0 | 53.7 | 64.0 | 9.26e-11 |
| AKG47165.1 | HysX2 | BGC0001223 | Other | 26.0 | 53.7 | 64.0 | 9.26e-11 |
| BAR97462.1 | cytochrome\_P450 | BGC0001911 | Terpene | 24.0 | 63.2 | 64.0 | 1.02e-10 |
| EPS29076.1 | hypothetical\_protein | BGC0001724 | NRP+Polyketide | 23.0 | 76.8 | 64.0 | 1.53e-10 |
| AGA37280.1 | P450\_monooxygenase | BGC0000819 | NRP+Alkaloid | 31.0 | 37.2 | 64.0 | 1.59e-10 |
| DAC76738.1 | cytochrome\_P450 | BGC0001885 | NRP+Polyketide | 25.0 | 74.9 | 62.0 | 4.28e-10 |
| CEO59279.1 | Putative\_Benzoate\_4-monooxygenase\_cytochrome\_P450 | BGC0002278 | Alkaloid+NRP | 24.0 | 78.4 | 62.0 | 8.42e-10 |
| AAY42399.1 | Cytochrome\_P450\_monooxygenase | BGC0001000 | NRP:Lipopeptide+Polyketide:Modular type I polyketide | 23.0 | 73.8 | 61.0 | 1.71e-09 |
| AGA37282.1 | P450\_monooxygenase | BGC0000819 | NRP+Alkaloid | 29.0 | 44.8 | 60.0 | 2.21e-09 |
| ARB50204.1 | clavine\_oxidase | BGC0001573 | Alkaloid | 34.0 | 31.8 | 60.0 | 3.36e-09 |
| ART41207.1 | AdrA | BGC0001508 | Polyketide | 26.0 | 34.8 | 58.0 | 1.03e-08 |
| DAD54578.1 | putative\_tryptophan\_6-hydroxylase | BGC0002256 | NRP+Other | 29.0 | 25.3 | 58.0 | 1.04e-08 |
| QVK45111.1 | cytochrome\_P450 | BGC0002438 | Alkaloid | 23.0 | 46.5 | 58.0 | 1.38e-08 |
| AZZ09610.1 | PvhE | BGC0002304 | Polyketide+NRP | 28.0 | 35.7 | 55.0 | 1.34e-07 |
| QKG86309.1 | NotH | BGC0002254 | Polyketide | 24.0 | 36.4 | 54.0 | 2.2e-07 |
| KJA16715.1 | hypothetical\_protein | BGC0002246 | Terpene | 26.0 | 44.6 | 54.0 | 2.35e-07 |
| ADD83003.1 | PtnO2 | BGC0001156 | Terpene | 24.0 | 43.7 | 54.0 | 2.69e-07 |
| CAQ18837.1 | putative\_cytochrome\_P450 | BGC0000954 | NRP+Polyketide:Modular type I polyketide | 28.0 | 44.6 | 53.0 | 3.85e-07 |
| KAF7597145.1 | hypothetical\_protein | BGC0002264 | NRP | 25.0 | 33.3 | 52.0 | 6.93e-07 |
| BAE78976.1 | P450 | BGC0001078 | Terpene+Polyketide | 26.0 | 48.1 | 52.0 | 8.19e-07 |
| CAL58689.1 | cytochrome\_P450\_dependent\_monooxygenase | BGC0000149 | Polyketide:Modular type I polyketide | 23.0 | 59.1 | 52.0 | 8.5e-07 |
| AAM18769.1 | AflL | BGC0000011 | Polyketide | 23.0 | 76.0 | 52.0 | 1.19e-06 |
| AAS90059.1 | VerB | BGC0000009 | Polyketide | 24.0 | 42.0 | 51.0 | 1.59e-06 |
| QJY30857.1 | cytochrome\_P450 | BGC0002539 | Alkaloid | 26.0 | 39.8 | 50.0 | 3.63e-06 |
| QIW91879.1 | P450-2 | BGC0002543 | NRP | 22.0 | 63.4 | 49.0 | 6.61e-06 |
| AAC49200.1 | putative\_p450\_monooxygenase | BGC0000152 | Polyketide | 23.0 | 76.8 | 49.0 | 8.44e-06 |
| UOH28367.1 | AceR | BGC0002149 | NRP+Terpene | 26.0 | 39.2 | 49.0 | 8.68e-06 |
