# Supplementary material for: Uncovering the transcriptional landscape of Fomes fomentarius during fungal-based material production through gene co-expression network analysis
Source: Fungal Biol Biotechnol. 2025 Feb 13;12:1. doi: 10.1186/s40694-024-00192-3 (PMC11827164; doi:10.1186/s40694-024-00192-3)
Supplement: Supplementary file 1 — Supplementary Material 1 [file 40694_2024_192_MOESM1_ESM.zip › knownclusterblast/region4/jgi.p_Fomfom1_1210865_mibig_hits.html]

| MIBiG Protein | Description | MIBiG Cluster | MiBiG Product | % ID | % Coverage | BLAST Score | E-value |
| --- | --- | --- | --- | --- | --- | --- | --- |
| ESK96610.1 | hypothetical\_protein | BGC0002212 | Polyketide | 28.0 | 103.5 | 328.0 | 1.45e-95 |
| ASK38699.1 | putative\_nonribosomal\_peptide\_synthetase-like\_protein | BGC0001436 | Polyketide:Iterative type I polyketide | 28.0 | 99.9 | 327.0 | 4.5e-95 |
| EAU35432.1 | predicted\_protein | BGC0002734 | Polyketide | 25.0 | 102.8 | 299.0 | 2.62e-85 |
| KFA69336.1 | hypothetical\_protein | BGC0001626 | Polyketide | 28.0 | 92.5 | 298.0 | 5.4e-85 |
| KIA75587.1 | NRPS-like\_enzyme | BGC0002209 | Polyketide | 24.0 | 106.3 | 263.0 | 9.23e-73 |
| EWG54274.1 | hypothetical\_protein | BGC0001190 | Polyketide | 26.0 | 98.4 | 261.0 | 2.44e-72 |
| CEF75881.1 |  | BGC0001600 | Polyketide | 26.0 | 99.6 | 262.0 | 2.48e-72 |
| BAV19380.1 | NRPS-like\_enzyme | BGC0001390 | NRP+Polyketide | 24.0 | 101.4 | 260.0 | 8.03e-72 |
| AUW31047.1 | PKS-like\_protein | BGC0002483 | Polyketide | 29.0 | 31.2 | 126.0 | 3.27e-31 |
| AEA29644.1 | putative\_nonribosomal\_peptide\_synthetase\_and\_kinurenine\_monooxygenase | BGC0000409 | NRP | 26.0 | 40.7 | 85.0 | 4.55e-16 |
| ABW71853.1 | nonribosomal\_peptide\_synthetase | BGC0000303 | NRP | 26.0 | 37.6 | 78.0 | 4.23e-14 |
