# Supplementary material for: Uncovering the transcriptional landscape of Fomes fomentarius during fungal-based material production through gene co-expression network analysis
Source: Fungal Biol Biotechnol. 2025 Feb 13;12:1. doi: 10.1186/s40694-024-00192-3 (PMC11827164; doi:10.1186/s40694-024-00192-3)
Supplement: Supplementary file 1 — Supplementary Material 1 [file 40694_2024_192_MOESM1_ESM.zip › knownclusterblast/region5/jgi.p_Fomfom1_1188506_mibig_hits.html]

| MIBiG Protein | Description | MIBiG Cluster | MiBiG Product | % ID | % Coverage | BLAST Score | E-value |
| --- | --- | --- | --- | --- | --- | --- | --- |
| ESK96610.1 | hypothetical\_protein | BGC0002212 | Polyketide | 28.0 | 105.5 | 333.0 | 1.84e-97 |
| ASK38699.1 | putative\_nonribosomal\_peptide\_synthetase-like\_protein | BGC0001436 | Polyketide:Iterative type I polyketide | 27.0 | 105.1 | 314.0 | 1.23e-90 |
| KFA69336.1 | hypothetical\_protein | BGC0001626 | Polyketide | 26.0 | 106.9 | 294.0 | 1.42e-83 |
| EAU35432.1 | predicted\_protein | BGC0002734 | Polyketide | 24.0 | 105.2 | 277.0 | 1.01e-77 |
| BBF25314.1 | NRPS-like\_oxidoreductase | BGC0001923 | Terpene+Polyketide | 25.0 | 88.0 | 275.0 | 4.36e-77 |
| BAV19380.1 | NRPS-like\_enzyme | BGC0001390 | NRP+Polyketide | 25.0 | 102.6 | 271.0 | 6.42e-76 |
| KIA75587.1 | NRPS-like\_enzyme | BGC0002209 | Polyketide | 26.0 | 90.0 | 265.0 | 2.65e-73 |
| EWG54274.1 | hypothetical\_protein | BGC0001190 | Polyketide | 25.0 | 105.6 | 244.0 | 9.12e-67 |
| ALI92655.1 | CitS\_citrinin\_polyketide\_synthase | BGC0001338 | Polyketide:Iterative type I polyketide | 30.0 | 36.5 | 172.0 | 4.96e-43 |
| EED18001.1 | NR-PKS | BGC0000154 | Polyketide:Iterative type I polyketide | 28.0 | 40.0 | 164.0 | 2.12e-40 |
| QBK15044.1 | clavatol\_synthase\_ClaF | BGC0002196 | Polyketide | 30.0 | 36.5 | 163.0 | 2.76e-40 |
| AGD80618.1 | non-ribosomal\_peptide\_synthetase | BGC0000394 | NRP | 21.0 | 97.3 | 61.0 | 1.06e-08 |
| QTT72092.1 | amino\_acid\_adenylation\_domain-containing\_protein | BGC0002350 | NRP+Polyketide+Saccharide | 25.0 | 33.1 | 56.0 | 2.94e-07 |
| AAG02364.1 | peptide\_synthetase\_NRPS2-1 | BGC0000963 | NRP:Glycopeptide+Polyketide:Modular type I polyketide+Saccharide:Hybrid/tailoring saccharide | 24.0 | 33.1 | 55.0 | 6.61e-07 |
| QCP68976.1 | VatQ | BGC0002296 | NRP+Polyketide | 22.0 | 44.4 | 53.0 | 1.84e-06 |
