# Supplementary material for: Uncovering the transcriptional landscape of Fomes fomentarius during fungal-based material production through gene co-expression network analysis
Source: Fungal Biol Biotechnol. 2025 Feb 13;12:1. doi: 10.1186/s40694-024-00192-3 (PMC11827164; doi:10.1186/s40694-024-00192-3)
Supplement: Supplementary file 1 — Supplementary Material 1 [file 40694_2024_192_MOESM1_ESM.zip › knownclusterblast/region5/jgi.p_Fomfom1_1189961_mibig_hits.html]

| MIBiG Protein | Description | MIBiG Cluster | MiBiG Product | % ID | % Coverage | BLAST Score | E-value |
| --- | --- | --- | --- | --- | --- | --- | --- |
| AAW03334.1 | hypothetical\_protein | BGC0000982 | NRP+Polyketide | 44.0 | 93.5 | 357.0 | 9.59e-119 |
| QCF28930.1 | aldehyde\_dehydrogenase | BGC0002308 | Alkaloid+Polyketide | 33.0 | 95.9 | 212.0 | 1.81e-62 |
| CAJ88625.1 | putative\_aldehyde\_dehydrogenase | BGC0000327 | NRP | 33.0 | 93.5 | 198.0 | 1.59e-57 |
| AJQ95720.1 | NAD-dependent\_aldehyde\_dehydrogenase | BGC0001644 | Polyketide | 34.0 | 87.9 | 197.0 | 3.37e-57 |
| WP\_033283297.1 | aldehyde\_dehydrogenase | BGC0001739 | Other | 30.0 | 94.5 | 196.0 | 1.25e-56 |
| BAV57467.1 | aldehyde\_dehydrogenase | BGC0001818 | NRP | 32.0 | 91.8 | 196.0 | 1.74e-56 |
| CAE45692.1 | hypothetical\_protein | BGC0000031 | Polyketide:Modular type I polyketide | 32.0 | 90.4 | 191.0 | 4.58e-55 |
| ABB90277.1 | aldehyde\_dehydrogenase | BGC0001057 | NRP+Polyketide | 32.0 | 99.2 | 192.0 | 6.19e-55 |
| BAK64640.1 | putative\_aldehyde\_dehydrogenase | BGC0000135 | Polyketide | 33.0 | 93.9 | 191.0 | 6.45e-55 |
| BCJ07576.1 | NAD-dependent\_phenylacetaldehyde\_dehydrogenase | BGC0002379 | NRP | 32.0 | 91.6 | 191.0 | 1.6e-54 |
| WP\_063744463.1 | aldehyde\_dehydrogenase\_family\_protein | BGC0001739 | Other | 32.0 | 82.8 | 190.0 | 1.97e-54 |
| QEG98940.1 | ant3 | BGC0002042 | NRP | 33.0 | 91.4 | 189.0 | 2.83e-54 |
| CAQ52616.1 | benzaldehyde\_degydrogenase | BGC0001066 | Polyketide:Modular type I polyketide | 34.0 | 90.8 | 187.0 | 2e-53 |
| AIS24863.1 | Dst3 | BGC0001147 | NRP | 34.0 | 81.6 | 180.0 | 1.11e-50 |
| WP\_015031703.1 | aldehyde\_dehydrogenase\_family\_protein | BGC0001819 | Polyketide | 34.0 | 82.4 | 172.0 | 9.77e-48 |
| KAF1058469.1 | putative\_aldehyde\_dehydrogenase\_AldA | BGC0002083 | Polyketide:Trans-AT type I polyketide | 30.0 | 98.2 | 169.0 | 1.35e-46 |
| WP\_105851111.1 | aldehyde\_dehydrogenase\_family\_protein | BGC0002087 | NRP+Polyketide | 30.0 | 98.2 | 168.0 | 3.57e-46 |
| OJJ98489.1 | hypothetical\_protein | BGC0002169 | Polyketide+NRP | 29.0 | 90.6 | 161.0 | 8.33e-44 |
| AMJ52086.1 | lijG | BGC0002255 | Polyketide | 29.0 | 73.2 | 144.0 | 1.21e-37 |
| AAK06797.1 | putative\_alcohol\_dehydrogenase\_SimC5 | BGC0001072 | Saccharide+Polyketide:Modular type I polyketide+Polyketide:Type II polyketide+Other:Aminocoumarin | 32.0 | 69.3 | 121.0 | 1.42e-29 |
| AAL15593.1 | Sim15 | BGC0000270 | Polyketide | 32.0 | 69.3 | 119.0 | 6.64e-29 |
| AXM43059.1 | aldehyde\_dehydrogenase | BGC0001945 | NRP | 29.0 | 73.0 | 114.0 | 3.29e-27 |
| WP\_052143141.1 | aldehyde\_dehydrogenase\_family\_protein | BGC0002087 | NRP+Polyketide | 34.0 | 40.5 | 91.0 | 8.48e-21 |
