# Supplementary material for: Uncovering the transcriptional landscape of Fomes fomentarius during fungal-based material production through gene co-expression network analysis
Source: Fungal Biol Biotechnol. 2025 Feb 13;12:1. doi: 10.1186/s40694-024-00192-3 (PMC11827164; doi:10.1186/s40694-024-00192-3)
Supplement: Supplementary file 1 — Supplementary Material 1 [file 40694_2024_192_MOESM1_ESM.zip › knownclusterblast/region5/jgi.p_Fomfom1_110009_mibig_hits.html]

| MIBiG Protein | Description | MIBiG Cluster | MiBiG Product | % ID | % Coverage | BLAST Score | E-value |
| --- | --- | --- | --- | --- | --- | --- | --- |
| CAP93753.1 |  | BGC0001882 | Polyketide | 46.0 | 101.1 | 218.0 | 1.45e-70 |
| EHK18428.1 | hypothetical\_protein | BGC0002233 | Polyketide | 42.0 | 102.2 | 192.0 | 2.11e-60 |
| QJQ82460.1 | BisF | BGC0002290 | Other | 39.0 | 102.6 | 176.0 | 6.22e-54 |
| APC57595.1 | SDR | BGC0001624 | Other | 36.0 | 102.6 | 161.0 | 4.25e-48 |
| AVY05522.1 | short-chain\_dehydrogenase/reductase | BGC0001571 | Terpene | 37.0 | 79.0 | 99.0 | 2.81e-24 |
| FAC38\_10 |  | BGC0002198 | NRP | 32.0 | 98.1 | 99.0 | 3.01e-24 |
| QDQ37876.1 | ketoreductase | BGC0001979 | Polyketide | 34.0 | 73.8 | 97.0 | 8.17e-24 |
| CAG44632.1 | SriL03.9 | BGC0000712 | Saccharide | 31.0 | 76.0 | 66.0 | 2.41e-12 |
| QCO93107.1 | short-chain\_dehydrogenase/reductase | BGC0001976 | Terpene | 26.0 | 84.3 | 51.0 | 4.07e-07 |
| AGZ20196.1 | short\_chain\_dehydrogenase | BGC0002618 | Terpene | 26.0 | 73.8 | 49.0 | 1.73e-06 |
