# Supplementary material for: Uncovering the transcriptional landscape of Fomes fomentarius during fungal-based material production through gene co-expression network analysis
Source: Fungal Biol Biotechnol. 2025 Feb 13;12:1. doi: 10.1186/s40694-024-00192-3 (PMC11827164; doi:10.1186/s40694-024-00192-3)
Supplement: Supplementary file 1 — Supplementary Material 1 [file 40694_2024_192_MOESM1_ESM.zip › knownclusterblast/region6/jgi.p_Fomfom1_130999_mibig_hits.html]

| MIBiG Protein | Description | MIBiG Cluster | MiBiG Product | % ID | % Coverage | BLAST Score | E-value |
| --- | --- | --- | --- | --- | --- | --- | --- |
| EIW83693.1 | terpenoid\_synthase | BGC0002707 | Terpene | 34.0 | 99.1 | 181.0 | 2.53e-54 |
| QDO73502.1 | PeniA | BGC0002557 | Terpene | 27.0 | 100.0 | 110.0 | 4.58e-27 |
